# Global, regional, and national burden of stroke and its risk factors, 1990–2021: a systematic analysis for the Global Burden of Disease Study 2021

**DOI:** 10.1016/S1474-4422(24)00369-7

**Published:** 2024-10

**Authors:** GBD 2021 Stroke Risk Factor Collaborators, Valery L Feigin, Melsew Dagne Abate, Yohannes Habtegiorgis Abate, Samar Abd ElHafeez, Foad Abd-Allah, Ahmed Abdelalim, Atef Abdelkader, Michael Abdelmasseh, Sherief Abd-Elsalam, Parsa Abdi, Arash Abdollahi, Meriem Abdoun, Rami Abd-Rabu, Deldar Morad Abdulah, Auwal Abdullahi, Mesfin Abebe, Roberto Ariel Abeldaño Zuñiga, E S Abhilash, Olugbenga Olusola Abiodun, Olumide Abiodun, Rahim Abo Kasem, Richard Gyan Aboagye, Mohamed Abouzid, Lucas Guimarães Abreu, Woldu Aberhe Abrha, Dariush Abtahi, Samir Abu Rumeileh, Ahmed Abualhasan, Hasan Abualruz, Eman Abu-Gharbieh, Hana J Abukhadijah, Niveen ME Abu-Rmeileh, Salahdein Aburuz, Ahmed Abu-Zaid, Juan Manuel Acuna, Denberu Eshetie Adane, Mesafint Molla Adane, Isaac Yeboah Addo, Rufus Adesoji Adedoyin, Oyelola A Adegboye, Victor Adekanmbi, Kishor Adhikari, Qorinah Estiningtyas Sakilah Adnani, Saryia Adra, Leticia Akua Adzigbli, Abdelrahman Yousry Afify, Aanuoluwapo Adeyimika Afolabi, Fatemeh Afrashteh, Muhammad Sohail Afzal, Saira Afzal, Shahin Aghamiri, Williams Agyemang-Duah, Bright Opoku Ahinkorah, Aqeel Ahmad, Muayyad M Ahmad, Sajjad Ahmad, Shahzaib Ahmad, Tauseef Ahmad, Amir Mahmoud Ahmadzade, Ali Ahmed, Ayman Ahmed, Haroon Ahmed, Syed Anees Ahmed, Marjan Ajami, Budi Aji, Essona Matatom Akara, Rufus Olusola Akinyemi, Mohammed Ahmed Akkaif, Ashley E Akrami, Salah Al Awaidy, Hanadi Al Hamad, Syed Mahfuz Al Hasan, Mohammad Al Qadire, Omar Al Ta'ani, Yazan Al-Ajlouni, Samer O Alalalmeh, Tariq A Alalwan, Ziyad Al-Aly, Rasmieh Mustafa Al-amer, Wafa A Aldhaleei, Mohammed S Aldossary, Seyedeh Yasaman Alemohammad, Bassam Al-Fatly, Adel Ali Saeed Al-Gheethi, Fadwa Naji Alhalaiqa, Maryam Alharrasi, Abid Ali, Mohammed Usman Ali, Rafat Ali, Syed Shujait Ali, Waad Ali, Akram Al-Ibraheem, Sheikh Mohammad Alif, Syed Mohamed Aljunid, Wael Almahmeed, Sabah Al-Marwani, Mahmoud A Alomari, Jordi Alonso, Jaber S Alqahtani, Rajaa M Mohammad Al-Raddadi, Ahmad Alrawashdeh, Mohammed A Alsabri, Najim Z Alshahrani, Zaid Altaany, Awais Altaf, Alaa B Al-Tammemi, Diala Altwalbeh, Nelson Alvis-Guzman, Hassan Alwafi, Mohammad Al-Wardat, Yaser Mohammed Al-Worafi, Hany Aly, Safwat Aly, Mohammad Sharif Ibrahim Alyahya, Karem H Alzoubi, Walid Adnan Al-Zyoud, Reza Amani, Prince M Amegbor, Tewodros Getnet Amera, Tarek Tawfik Amin, Alireza Amindarolzarbi, Sohrab Amiri, Hubert Amu, Dickson A Amugsi, Ganiyu Adeniyi Amusa, Robert Ancuceanu, Deanna Anderlini, Dhanalakshmi Angappan, Abhishek Anil, Mohammed Tahir Tahir Ansari, Alireza Ansari-Moghaddam, Rockson Ansong, Saeid Anvari, Saleha Anwar, Sumadi Lukman Anwar, Ekenedilichukwu Emmanuel Anyabolo, Anayochukwu Edward Anyasodor, Geminn Louis Carace Apostol, Francis Appiah, Muhammad Aqeel, Jalal Arabloo, Razman Arabzadeh Bahri, Mosab Arafat, Aleksandr Y Aravkin, Ali Ardekani, Demelash Areda, Brhane Berhe Aregawi, Getnet Mesfin Aregu, Olatunde Aremu, Hidayat Arifin, Johan Ärnlöv, Anton A Artamonov, Judie Arulappan, Umesh Raj Aryal, Zahra Aryan, Akram M Asbeutah, Mulusew A Asemahagn, Mulu Tiruneh Asemu, Mohammad Asghari-Jafarabadi, Mubarek Yesse Ashemo, Tahira Ashraf, Armin Aslani, Haftu Asmerom Asmerom, Thomas Astell-Burt, Seyyed Shamsadin Athari, Prince Atorkey, Maha Moh'd Wahbi Atout, Alok Atreya, Avinash Aujayeb, Marcel Ausloos, Abolfazl Avan, Hamzeh Awad, Adedapo Wasiu Awotidebe, Lemessa Assefa A Ayana, Setognal Birara Aychiluhm, Amdehiwot A Aynalem, Zewdu Bishaw Aynalem, Sina Azadnajafabad, Hiva Azami, Shahkaar Aziz, Ahmed Y Azzam, Abraham Samuel Babu, Giridhara Rathnaiah Babu, Muhammad Badar, Ashish D Badiye, Pegah Bahrami Taghanaki, Saeed Bahramian, Ruhai Bai, Atif Amin Baig, Shankar M Bakkannavar, Abdulaziz T Bako, Ovidiu Constantin Baltatu, Kiran Bam, Maciej Banach, Morteza Banakar, Soham Bandyopadhyay, Palash Chandra Banik, Kannu Bansal, Yanping Bao, Miguel A Barboza, Mainak Bardhan, Noel C Barengo, Suzanne Lyn Barker-Collo, Till Winfried Bärnighausen, Hiba Jawdat Barqawi, Amadou Barrow, Lingkan Barua, Azadeh Bashiri, Hameed Akande Bashiru, Afisu Basiru, Mohammad-Mahdi Bastan, Sanjay Basu, Saurav Basu, Kavita Batra, Ahmet Begde, Babak Behnam, Amir Hossein Behnoush, Melesse B Y Belayneh, Michael Belingheri, Umar Muhammad Bello, Derrick A Bennett, Isabela M Bensenor, Fentaw Tadese Berhe, Amiel Nazer C Bermudez, Habtamu B B Beyene, Kebede A Beyene, Devidas S Bhagat, Akshaya Srikanth Bhagavathula, Neeraj Bhala, Ashish Bhalla, Nikha Bhardwaj, Pankaj Bhardwaj, Sonu Bhaskar, Ajay Nagesh Bhat, Vivek Bhat, Gurjit Kaur Bhatti, Jasvinder Singh Singh Bhatti, Mohiuddin Ahmed Bhuiyan, Subarna Bhusal, Boris Bikbov, Cem Bilgin, Antonio Biondi, Keralem Anteneh Bishaw, Atanu Biswas, Bijit Biswas, Trupti Bodhare, Eyob Ketema Bogale, Archith Boloor, Milad Bonakdar Hashemi, Aime Bonny, Berrak Bora Basara, Hamed Borhany, Samuel Adolf Bosoka, Souad Bouaoud, Abdelhakim Bouyahya, Edward J Boyko, Marija M Bozic, Dejana Braithwaite, Susanne Breitner, Hermann Brenner, Gabrielle Britton, Andre R Brunoni, Dana Bryazka, Raffaele Bugiardini, Lemma N Bulto, Katrin Burkart, Yasser Bustanji, Zahid A Butt, Florentino Luciano Caetano dos Santos, Luis Alberto Cámera, Luciana Aparecida Campos, Ismael R Campos-Nonato, Fan Cao, Angelo Capodici, Rosario Cárdenas, Sinclair Carr, Giulia Carreras, Andre F Carvalho, Felix Carvalho, Joao Mauricio Castaldelli-Maia, Carlos A Castañeda-Orjuela, Giulio Castelpietra, Alberico L Catapano, Maria Sofia Cattaruzza, Luca Cegolon, Francieli Cembranel, Edina Cenko, Ester Cerin, Joshua Chadwick, Chiranjib Chakraborty, Sandip Chakraborty, Jeffrey Shi Kai Chan, Rama Mohan Chandika, Eeshwar K Chandrasekar, Gashaw Sisay Chanie, Vijay Kumar Chattu, Anis Ahmad Chaudhary, Akhilanand Chaurasia, Haowei Chen, Mingling Chen, Simiao Chen, Gerald Chi, Fatemeh Chichagi, Ritesh Chimoriya, Patrick R Ching, Abdulaal Chitheer, So Mi Jemma Cho, Dong-Woo Choi, Bryan Chong, Chean Lin Chong, Hitesh Chopra, Sonali Gajanan Choudhari, Rahul Choudhary, Dinh-Toi Chu, Isaac Sunday Chukwu, Sheng-Chia Chung, Zinhle Cindi, Iolanda Cioffi, Rebecca M Cogen, Alyssa Columbus, Simona Costanzo, Rosa A S Couto, Michael H Criqui, Natalia Cruz-Martins, Silvia Magali Cuadra-Hernández, Alanna Gomes da Silva, Sriharsha Dadana, Omid Dadras, Xiaochen Dai, Koustuv Dalal, Lachlan L Dalli, Giovanni Damiani, Emanuele D'Amico, Lalit Dandona, Rakhi Dandona, Amira Hamed Darwish, Saswati Das, Mohsen Dashti, Mohadese Dashtkoohi, Mohammad Dashtkoohi, Maedeh Dastmardi, Kairat Davletov, Vanessa De la Cruz-Góngora, Sean DeAngelo, Aklilu Tamire Debele, Shayom Debopadhaya, Ivan Delgado-Enciso, Berecha Hundessa Demessa, Andreas K Demetriades, Edgar Denova-Gutiérrez, Emina Dervišević, Hardik Dineshbhai Desai, Aragaw Tesfaw Desale, Fikreab Desta, Vinoth Gnana Chellaiyan Devanbu, Devananda Devegowda, Syed Masudur Rahman Dewan, Amol S Dhane, Meghnath Dhimal, Vishal R Dhulipala, Michael J Diaz, Mengistie Diress, Milad Dodangeh, Phidelia Theresa Doegah, Sushil Dohare, Mohamed Fahmy Doheim, Klara Georgieva Dokova, Deepa Dongarwar, Mario D'Oria, Ojas Prakashbhai Doshi, Rajkumar Prakashbhai Doshi, Abdel Douiri, Robert Kokou Dowou, Ashel Chelsea Dsouza, Haneil Larson Dsouza, Viola Savy Dsouza, Bruce B Duncan, Andre Rodrigues Duraes, Arkadiusz Marian Dziedzic, Michael Ekholuenetale, Ibrahim Farahat El Bayoumy, Maysaa El Sayed Zaki, Iffat Elbarazi, Faris El-Dahiyat, Islam Y Elgendy, Muhammed Elhadi, Waseem El-Huneidi, Mohamed A Elmonem, Adel B Elmoselhi, Chadi Eltaha, Theophilus I Emeto, Christopher Imokhuede Esezobor, Negin Esfandiari, Zahra Esmaeili, Francesco Esposito, Mohammad Etoom, Natalia Fabin, Ibtihal Fadhil, Adeniyi Francis Fagbamigbe, Omotayo Francis Fagbule, Shahriar Faghani, Ayesha Fahim, Ildar Ravisovich Fakhradiyev, Luca Falzone, Mohammad Fareed, Jawad Fares, Carla Sofia e Sá Farinha, MoezAlIslam Ezzat Mahmoud Faris, Pawan Sirwan Faris, Mohsen Farjoud Kouhanjani, Andre Faro, Hossein Farrokhpour, Abidemi Omolara Fasanmi, Nelsensius Klau Fauk, Patrick Fazeli, Timur Fazylov, Alireza Feizkhah, Ginenus Fekadu, Xiaoqi Feng, Seyed-Mohammad Fereshtehnejad, Pietro Ferrara, Nuno Ferreira, Getahun Fetensa, Bikila Regassa Feyisa, Florian Fischer, Luisa S Flor, Kristen Marie Foley, Ana Catarina Fonseca, Roham Foroumadi, Behzad Foroutan, Daniela Fortuna, Matteo Foschi, Richard Charles Franklin, Ni Kadek Yuni Fridayani, Sridevi G, Peter Andras Gaal, Abhay Motiramji Gaidhane, Abduzhappar Gaipov, Yaseen Galali, Silvano Gallus, Aravind P Gandhi, Balasankar Ganesan, Danijela Gasevic, Prem Gautam, Rupesh K Gautam, Miglas Welay Gebregergis, Mesfin Gebrehiwot, Kebre Gebrekirstos Gebrekidan, Lemma Getacher, Genanew K Getahun, Molla Getie, Delaram J Ghadimi, Fataneh Ghadirian, Amir Ghaffari Jolfayi, Mansour Ghafourifard, Mohammad-Reza Ghasemi, Afsaneh Ghasemzadeh, Ramy Mohamed Ghazy, Ehsan Gholami, Zainab Gholami, Sherief Ghozy, Stefano Giannoni Luza, Jaleed Ahmed Gilani, Tiffany K Gill, Richard F Gillum, Ebisa Zerihun Gindaba, Elena V Gnedovskaya, Amit Goel, Mohamad Goldust, Mahaveer Golechha, Pouya Goleij, Davide Golinelli, Philimon N Gona, Giuseppe Gorini, Alessandra C Goulart, Barbara Niegia Garcia Goulart, Mahdi Gouravani, Michal Grivna, Giuseppe Grosso, Ashna Grover, Shi-Yang Guan, Giovanni Guarducci, Avirup Guha, Stefano Guicciardi, Snigdha Gulati, Damitha Asanga Gunawardane, Cui Guo, Zhifeng Guo, Anish Kumar Gupta, Bhawna Gupta, Mohak Gupta, Rahul Gupta, Rajat Das Gupta, Rajeev Gupta, Sapna Gupta, Farrokh Habibzadeh, Najah R Hadi, Mohammad Haghani Dogahe, Hamed Haghi-Aminjan, Dariush Haghmorad, Arvin Haj-Mirzaian, Aram Halimi, Nadia M Hamdy, Samer Hamidi, Erin B Hamilton, Asif Hanif, Nasrin Hanifi, Graeme J Hankey, Md Abdul Hannan, Zaim Anan Haq, Arief Hargono, Netanja I Harlianto, Josep Maria Haro, Eka Mishbahatul Marah Has, Ahmed I Hasaballah, Ikramul Hasan, Md Saquib Hasnain, Ikrama Hassan, Mahgol Sadat Hassan Zadeh Tabatabaei, Johannes Haubold, Rasmus J Havmoeller, Simon I Hay, Youssef Hbid, Jeffrey J Hebert, Omar E Hegazi, Mohammad Heidari, Mehdi Hemmati, Demisu Zenbaba Heyi, Kamal Hezam, Yuta Hiraike, Nguyen Quoc Hoan, Ramesh Holla, Nobuyuki Horita, Md Mahbub Hossain, Hassan Hosseinzadeh, Mehdi Hosseinzadeh, Ahmad Hosseinzadeh Adli, Mihaela Hostiuc, Sorin Hostiuc, Ben Hu, Chengxi Hu, Junjie Huang, Ayesha Humayun, Salman Hussain, Le Duc Huy, Hong-Han Huynh, Bing-Fang Hwang, Segun Emmanuel Ibitoye, Nayu Ikeda, Adalia Ikiroma, Olayinka Stephen Ilesanmi, Irena M Ilic, Milena D Ilic, Mohammad Tarique Imam, Mustapha Immurana, Leeberk Raja Inbaraj, Muhammad Iqhrammullah, Arnaud Iradukunda, Lalu Muhammad Irham, Md Rabiul Islam, Faisal Ismail, Nahlah Elkudssiah Ismail, Hiroyasu Iso, Gaetano Isola, Ramaiah Itumalla, Masao Iwagami, Chidozie Declan CD Iwu, Vinothini J, Jalil Jaafari, Louis Jacob, Abdollah Jafarzadeh, Haitham Jahrami, Akhil Jain, Nityanand Jain, Ammar Abdulrahman Jairoun, Abhishek Jaiswal, Mihajlo Jakovljevic, Reza Jalilzadeh Yengejeh, Balamurugan Janakiraman, Abubakar Ibrahim Jatau, Sathish Kumar Jayapal, Shubha Jayaram, Sun Ha Jee, Jayakumar Jeganathan, Mihretu Jegnie, Alelign Tasew Jema, Bijay Mukesh Jeswani, Angeline Jeyakumar, Anil K Jha, Ravi Prakash Jha, Zixiang Ji, Heng Jiang, Shuai Jin, Yingzhao Jin, Mohammad Jokar, Jost B Jonas, Tamas Joo, Jobinse Jose, Nitin Joseph, Charity Ehimwenma Joshua, Farahnaz Joukar, Jacek Jerzy Jozwiak, Mikk Jürisson, Ali Kabir, Md Awal Kabir, Zubair Kabir, Vidya Kadashetti, Rizwan Kalani, Sanjay Kalra, Vineet Kumar Kamal, Arun Kamireddy, Haidong Kan, Mona Kanaan, Himal Kandel, Kehinde Kazeem Kanmodi, Rami S Kantar, Neeti Kapoor, Paschalis Karakasis, Ibraheem M Karaye, André Karch, Hanie Karimi, Salah Eddin Karimi, Yeganeh Karimi, Arman Karimi Behnagh, Prabin Karki, Hengameh Kasraei, Joonas H Kauppila, Harkiran Kaur, Neda Kaydi, Gbenga A Kayode, Foad Kazemi, Sina Kazemian, Emmanuelle Kesse-Guyot, Yousef Saleh Khader, Morteza Abdullatif Khafaie, Inn Kynn Khaing, Himanshu Khajuria, Amirmohammad Khalaji, Nauman Khalid, Anees Ahmed Khalil, Asaduzzaman Khan, Fayaz Khan, M Nuruzzaman Khan, Maseer Khan, Mohammad Jobair Khan, Moien AB Khan, Yusra H Khan, Shaghayegh Khanmohammadi, Khaled Khatab, Haitham Khatatbeh, Moawiah Mohammad Khatatbeh, Sorour Khateri, Mahalaqua Nazli Khatib, Maryam Khayamzadeh, Hamid Reza Khayat Kashani, Feriha Fatima Khidri, Manoj Khokhar, Atulya Aman Khosla, Majid Khosravi, Jagdish Khubchandani, Saeid Kian, Kwanghyun Kim, Min Seo Kim, Yun Jin Kim, Ruth W Kimokoti, Adnan Kisa, Sezer Kisa, Ali-Asghar Kolahi, Kamrun Nahar Koly, Farzad Kompani, Shivakumar KM Marulasiddaiah Kondlahalli, Miikka Korja, Vladimir Andreevich Korshunov, Oleksii Korzh, Soewarta Kosen, Karel Kostev, Nikhil Kothari, Ashwin Laxmikant Kotnis, Sindhura Lakshmi Koulmane Laxminarayana, Kewal Krishan, Varun Krishna, Vijay Krishnamoorthy, Bindu Krishnan, Jera Kruja, Barthelemy Kuate Defo, Burcu Kucuk Bicer, Md Abdul Kuddus, Mohammed Kuddus, Nuworza Kugbey, Mukhtar Kulimbet, Vishnutheertha Kulkarni, Akshay Kumar, Ashish Kumar, Dewesh Kumar, G Anil Kumar, Nithin Kumar, Rakesh Kumar, Senthil Kumaran D, Amartya Kundu, Satyajit Kundu, Setor K Kunutsor, Om P Kurmi, Dian Kusuma, L V Simhachalam Kutikuppala, Ambily Kuttikkattu, Ville Kytö, Carlo La Vecchia, Ben Lacey, Chandrakant Lahariya, Dharmesh Kumar Lal, Tea Lallukka, Judit Lám, Iván Landires, Anders O Larsson, Savita Lasrado, Kaveh Latifinaibin, Paolo Lauriola, Pablo M Lavados, Basira Kankia Lawal, Long Khanh Dao Le, Nhi Huu Hanh Le, Thao Thi Thu Le, Trang Diep Thanh Le, Paul H Lee, Seung Won Lee, Wei-Chen Lee, Yo Han Lee, Ming-Chieh Li, Wei Li, Xiaopan Li, Yichong Li, Lee-Ling Lim, Stephen S Lim, John C Lin, Daniel Lindholm, Shai Linn, Gang Liu, Runben Liu, Shuke Liu, Xiaofeng Liu, Xuefeng Liu, Erand Llanaj, Chun-Han Lo, Warren David Lo, Valerie Lohner, José Francisco López-Gil, László Lorenzovici, Stefan Lorkowski, Paulo A Lotufo, Giancarlo Lucchetti, Lisha Luo, Jay B Lusk, Zheng Feei Ma, Monika Machoy, Farzan Madadizadeh, Ralph Maddison, Elham Mahmoudi, Golnaz Mahmoudvand, Omar M Makram, Elaheh Malakan Rad, Kashish Malhotra, Ahmad Azam Malik, Iram Malik, Tauqeer Hussain Mallhi, Deborah Carvalho Malta, Abdullah A Mamun, Yosef Manla, Mohammad Hadi Mansouri, Pejman Mansouri, Vahid Mansouri, Mohammad Ali Mansournia, Lorenzo Giovanni Mantovani, Emmanuel Manu, Hamid Reza Marateb, Abdoljalal Marjani, Daniela Martini, Santi Martini, Miquel Martorell, Sharmeen Maryam, Roy Rillera Marzo, Awoke Masrie, Yasith Mathangasinghe, Pallab K Maulik, Mahsa Mayeli, Mohsen Mazidi, Martin McKee, Steven M McPhail, Enkeleint A Mechili, Asim Mehmood, Kamran Mehrabani-Zeinabad, Tesfahun Mekene Meto, Hadush Negash Meles, Walter Mendoza, Ritesh G Menezes, George A Mensah, Sultan Ayoub Meo, Atte Meretoja, Tuomo J Meretoja, Tomislav Mestrovic, Chamila Dinushi Kukulege Mettananda, Tomasz Miazgowski, Irmina Maria Michalek, Ana Carolina Micheletti Gomide Nogueira de Sá, Giuseppe Minervini, Le Huu Nhat Minh, GK Mini, Mojgan Mirghafourvand, Andreea Mirica, Erkin M Mirrakhimov, Mohammad Mirza-Aghazadeh-Attari, Manish Mishra, Sanjeev Misra, Prasanna Mithra, Ahmed Ismail Mohamed, Jama Mohamed, Nouh Saad Mohamed, Ameen Mosa Mohammad, Esmaeil Mohammadi, Saeed Mohammadi, Soheil Mohammadi, Abdollah Mohammadian-Hafshejani, Ibrahim Mohammadzadeh, Hussen Mohammed, Mustapha Mohammed, Salahuddin Mohammed, Shafiu Mohammed, Ali H Mokdad, Hossein Molavi Vardanjani, Mariam Molokhia, Shaher Momani, Lorenzo Monasta, Mohammad Ali Moni, Fateme Montazeri, AmirAli Moodi Ghalibaf, Mahmood Moosazadeh, Maryam Moradi, Yousef Moradi, Paula Moraga, Lidia Morawska, Rafael Silveira Moreira, Shane Douglas Morrison, Reza Mosaddeghi Heris, Elias Mossialos, Parsa Mousavi, Ahmed Msherghi, Sumaira Mubarik, Lorenzo Muccioli, Admir Mulita, Malaisamy Muniyandi, Kavita Munjal, Efren Murillo-Zamora, Sathish Muthu, Woojae Myung, Amin Nabavi, Ashraf Fawzy Nabhan, Ayoub Nafei, Ahamarshan Jayaraman Nagarajan, Pirouz Naghavi, Ganesh R Naik, Gurudatta Naik, Mukhammad David Naimzada, Sanjeev Nair, Tapas Sadasivan Nair, Soroush Najdaghi, Hastyar Hama Rashid Najmuldeen, Noureddin Nakhostin Ansari, Vinay Nangia, Sreenivas Narasimha Swamy, Shumaila Nargus, Delaram Narimani Davani, Bruno Ramos Nascimento, Gustavo G Nascimento, Ali Nasrollahizadeh, Amir Nasrollahizadeh, Zuhair S Natto, Javaid Nauman, Samidi Nirasha Kumari Navaratna, Biswa Prakash Nayak, Vinod C Nayak, Athare Nazri-Panjaki, Rawlance Ndejjo, Ionut Negoi, Ruxandra Irina Negoi, Seyed Aria Nejadghaderi, Chakib Nejjari, Mohammad Hadi Nematollahi, Samata Nepal, Charles Richard James Newton, Dang H Nguyen, Duc Hoang Nguyen, Hau Thi Hien Nguyen, Hien Quang Nguyen, Nhien Ngoc Y Nguyen, Phat Tuan Nguyen, Van Thanh Nguyen, Robina Khan Niazi, Yeshambel T Nigatu, Nasrin Nikravangolsefid, Dina Nur Anggraini Ningrum, Chukwudi A Nnaji, Lawrence Achilles Nnyanzi, Shuhei Nomura, Syed Toukir Ahmed Noor, Bo Norrving, Nawsherwan Nawsherwan, Jean Jacques Noubiap, Chisom Adaobi Nri-Ezedi, George Ntaios, Mpiko Ntsekhe, Fred Nugen, Mario Cesare Nurchis, Dieta Nurrika, Chimezie Igwegbe Nzoputam, Ogochukwu Janet Nzoputam, Bogdan Oancea, Kehinde O Obamiro, Ismail A Odetokun, Martin James O'Donnell, James Odhiambo Oguta, In-Hwan Oh, Tolulope R Ojo-Akosile, Hassan Okati-Aliabad, Sylvester Reuben Okeke, Akinkunmi Paul Okekunle, Lawrence Okidi, Osaretin Christabel Okonji, Morteza Oladnabi, Andrew T Olagunju, Muideen Tunbosun Olaiya, Oladotun Victor Olalusi, Tosin Abiola Olasehinde, Omotola O Olasupo, Matthew Idowu Olatubi, Arão Belitardo Oliveira, Gláucia Maria Moraes Oliveira, Abdulhakeem Abayomi Olorukooba, Isaac Iyinoluwa Olufadewa, Yinka Doris Doris Oluwafemi, Gideon Olamilekan Oluwatunase, Hany A Omar, Ahmed Omar Bali, Adrienne E O'Neil, Sok King Ong, Obinna E Onwujekwe, Abdulahi Opejin Opejin, Michal Ordak, Raffaele Ornello, Doris V Ortega-Altamirano, Alberto Ortiz, Esteban Ortiz-Prado, Wael M S Osman, Uchechukwu Levi Osuagwu, Stanislav S Otstavnov, Mayowa O Owolabi, Ifeoluwa Temitayo Oyeyemi, Ahmad Ozair, Mahesh Padukudru P A, Kevin Pacheco-Barrios, Alicia Padron-Monedero, Jagadish Rao Padubidri, Tamás Palicz, Raul Felipe Palma-Alvarez, Feng Pan, Songhomitra Panda-Jonas, Deepshikha Pande Katare, Anamika Pandey, Ashok Pandey, Seithikurippu R Pandi-Perumal, Leonidas D Panos, Ioannis Pantazopoulos, Paraskevi Papadopoulou, Shahina Pardhan, Pragyan Paramita Parija, Romil R Parikh, Nicholas Parsons, Roberto Passera, Dimitrios Patoulias, Uttam Paudel, Shrikant Pawar, Amy E Peden, Paolo Pedersini, Prince Peprah, Maria Odete Pereira, Mario F P Peres, Arokiasamy Perianayagam, Norberto Perico, Simone Perna, Richard G Pestell, Ionela-Roxana Petcu, Fanny Emily Petermann-Rocha, Hoang Nhat Pham, Hoang Tran Pham, Michael R Phillips, Thomas Pilgrim, Michael A Piradov, Saeed Pirouzpanah, Evgenii Plotnikov, Dimitri Poddighe, Ramesh Poluru, Djordje S Popovic, Maarten J Postma, Akram Pourshams, Naeimeh Pourtaheri, Jalandhar Pradhan, Pranil Man Singh Pradhan, V Prakash, Manya Prasad, Elton Junio Sady Prates, Dimas Ria Angga Pribadi, Jagadeesh Puvvula, Ibrahim Qattea, Gangzhen Qian, Yanan Qiao, Alberto Raggi, Pankaja Raghav Raghav, Pracheth Raghuveer, Fakher Rahim, Md Jillur Rahim, Mahban Rahimifard, Vafa Rahimi-Movaghar, Md Mosfequr Rahman, Mohammad Hifz Ur Rahman, Mosiur Rahman, Muhammad Aziz Rahman, Amir Masoud Rahmani, Mohammad Rahmanian, Nazanin Rahmanian, Vahid Rahmanian, Rahem Rahmati, Setyaningrum Rahmawaty, Gerard Marshall Raj, Sathish Rajaa, Vinoth Rajendran, Pushp Lata Rajpoot, Prashant Rajput, Pradhum Ram, Mahmoud Mohammed Ramadan, Majed Ramadan, Venkitachalam Ramanarayanan, Shakthi Kumaran Ramasamy, Sheena Ramazanu, Juwel Rana, Kritika Rana, Rishabh Kumar Rana, Chhabi Lal Ranabhat, Nemanja Rancic, Amey Rane, Annemarei Ranta, Mithun Rao, Sowmya J Rao, Sina Rashedi, Mohammad-Mahdi Rashidi, Ashkan Rasouli-Saravani, Devarajan Rathish, Santosh Kumar Rauniyar, Salman Rawaf, Christian Razo, Murali Mohan Rama Krishna Reddy, Elrashdy Moustafa Mohamed Redwan, Inayat Ur Rehman, Giuseppe Remuzzi, Nazila Rezaei, Mohsen Rezaeian, Hossein Rezazadeh, Taeho Gregory Rhee, Mavra A Riaz, Antonio Luiz P Ribeiro, Monica Rodrigues, Thales Philipe R Rodrigues da Silva, Jefferson Antonio Buendia Rodriguez, Leonardo Roever, Debby Syahru Romadlon, Allen Guy Ross, Himanshu Sekhar Rout, Bedanta Roy, Priyanka Roy, Simanta Roy, Guilherme de Andrade Ruela, Michele Russo, Godfrey M Rwegerera, Chandan S N, Aly M A Saad, Korosh Saber, Maha Mohamed Saber-Ayad, Cameron John Sabet, Siamak Sabour, Simona Sacco, Basema Ahmad Saddik, Erfan Sadeghi, Mohammad Reza Saeb, Umar Saeed, Sher Zaman Zaman Safi, Rajesh Sagar, Alireza Saghafi, Dominic Sagoe, Fatemeh Saheb Sharif-Askari, Amirhossein Sahebkar, Pragyan Monalisa Sahoo, Soumya Swaroop Sahoo, Mirza Rizwan Sajid, Afeez Abolarinwa Salami, Luciane B Salaroli, Mohamed A Saleh, Mohammed Z Y Salem, Giovanni A Salum, Sara Samadzadeh, Saad Samargandy, Yoseph Leonardo Samodra, Vijaya Paul Samuel, Abdallah M Samy, Juan Sanabria, Itamar S Santos, Milena M Santric-Milicevic, Made Ary Sarasmita, Aswini Saravanan, Yaser Sarikhani, Gargi Sachin Sarode, Sachin C Sarode, Maheswar Satpathy, Zafer Sattouf, Ganesh Kumar Saya, Md Abu Sayeed, Mehdi Sayyah, Nikolaos Scarmeas, Benedikt Michael Schaarschmidt, Markus P Schlaich, Maria Inês Schmidt, Ione Jayce Ceola Schneider, Art Schuermans, Austin E Schumacher, Aletta Elisabeth Schutte, David C Schwebel, Siddharthan Selvaraj, Parijat Sen, Sabyasachi Senapati, Subramanian Senthilkumaran, Mihretu Tagesse Sergindo, Yashendra Sethi, Allen Seylani, Mahan Shafie, Pritik A Shah, Saeed Shahabi, Ataollah Shahbandi, Samiah Shahid, Hamid R Shahsavari, Moyad Jamal Shahwan, Masood Ali Shaikh, Ali S Shalash, Muhammad Aaqib Shamim, Mehran Shams-Beyranvand, Anas Shamsi, Alfiya Shamsutdinova, Mohd Shanawaz, Mohammed Shannawaz, Medha Sharath, Amin Sharifan, Azam Sharifi, Javad Sharifi-Rad, Anupam Sharma, Manoj Sharma, Sourabh Sharma, Ujjawal Sharma, Vishal Sharma, Rahim Ali Sheikhi, Adithi Shetty, Mahabalesh Shetty, Premalatha K Shetty, Desalegn Shiferaw, Mika Shigematsu, Tariku Shimels, Min-Jeong Shin, Rahman Shiri, Aminu Shittu, Abdul-karim Olayinka Shitu, Ivy Shiue, Seyed Afshin Shorofi, Sunil Shrestha, Kerem Shuval, Yafei Si, Emmanuel Edwar Siddig, Mithun Sikdar, João Pedro Silva, Luís Manuel Lopes Rodrigues Silva, Abhinav Singh, Baljinder Singh, Garima Singh, Harmanjit Singh, Jasvinder A Singh, Kuldeep Singh, Narinder Pal Singh, Paramdeep Singh, Puneetpal Singh, Jussi O T Sipilä, Shravan Sivakumar, Valentin Yurievich Skryabin, Anna Aleksandrovna Skryabina, David A Sleet, Farrukh Sobia, Bogdan Socea, Abdullah Al Mamun Sohag, Ranjan Solanki, Shipra Solanki, Yerukneh Solomon, Yi Song, Soroush Soraneh, Reed J D Sorensen, Houman Sotoudeh, Ireneous N Soyiri, Michael Spartalis, Chandrashekhar T Sreeramareddy, Suresh Kumar Srinivasamurthy, Panagiotis Stachteas, Lauryn K Stafford, Benjamin A Stark, Antonina V Starodubova, Narayan Subedi, Vetriselvan Subramaniyan, Muhammad Suleman, Abida Sultana, Zhong Sun, Johan Sundström, Vinay Suresh, Sri Susanty, Chandan Kumar Swain, Lukasz Szarpak, Sree Sudha T Y, Payam Tabaee Damavandi, Rafael Tabarés-Seisdedos, Seyyed Mohammad Tabatabaei, Shima Tabatabai, Celine Tabche, Mohammad Tabish, Jyothi Tadakamadla, Santosh Kumar Tadakamadla, Amirmasoud Taheri, Jabeen Taiba, Iman M Talaat, Ashis Talukder, Mircea Tampa, Jacques Lukenze Tamuzi, Ker-Kan Tan, Haosu Tang, Manoj Tanwar, Ingan Ukur Tarigan, Elvis Enowbeyang Tarkang, Nathan Y Tat, Seyed Mohammad Tavangar, Arash Tehrani-Banihashemi, Mojtaba Teimoori, Mohamad-Hani Temsah, Reem Mohamad Hani Temsah, Masayuki Teramoto, Wegen Beyene Tesfamariam, Edosa Geta Tesfaye Gta, Ramna Thakur, Pugazhenthan Thangaraju, Rajshree Thapa, Rekha Thapar, Rasiah Thayakaran, Sathish Thirunavukkarasu, Joe Thomas, Nikhil Kenny Kenny Thomas, Amanda G Thrift, Jing Tian, Ales Tichopad, Jansje Henny Vera Ticoalu, Chalachew Tiruneh, Krishna Tiwari, Amir Tiyuri, Marcello Tonelli, Roman Topor-Madry, Marcos Roberto Tovani-Palone, Khaled Trabelsi, Ngoc Ha Tran, Thang Huu Tran, Nguyen Tran Minh Duc, Domenico Trico, Samuel Joseph Tromans, Thien Tan Tri Tai Truyen, Daniel Hsiang-Te Tsai, Aristidis Tsatsakis, Evangelia Eirini Tsermpini, Ermias A A Turuse, Stefanos Tyrovolas, Aniefiok John Udoakang, Arit Udoh, Atta Ullah, Sana Ullah, Muhammad Umair, Muhammad Umar, Brigid Unim, Bhaskaran Unnikrishnan, Daniele Urso, Jibrin Sammani Usman, Marco Vacante, Seyed Mohammad Vahabi, Sanaz Vahdati, Asokan Govindaraj Vaithinathan, Omid Vakili, Rohollah Valizadeh, Jef Van den Eynde, Orsolya Varga, Shoban Babu Varthya, Tommi Juhani Vasankari, Balachandar Vellingiri, Narayanaswamy Venketasubramanian, Madhur Verma, Massimiliano Veroux, Georgios-Ioannis Verras, Dominique Vervoort, Jorge Hugo Villafañe, Simona Villani, Manish Vinayak, Maria Viskadourou, Simona Ruxandra Volovat, Victor Volovici, Hatem A Wafa, Yasir Waheed, Waseem Wahood, Cong Wang, Fang Wang, Shu Wang, Song Wang, Yanzhong Wang, Yuan-Pang Wang, Mary Njeri Wanjau, Muhammad Waqas, Emebet Gashaw Wassie, Gizachew Tadesse Wassie, Zihan Wei, Robert G Weintraub, Haftom Legese Weldetinsaa, Dakshitha Praneeth Wickramasinghe, Nuwan Darshana Wickramasinghe, Tissa Wijeratne, Peter Willeit, Charles D A Wolfe, Yen Jun Wong, Utoomporn Wongsin, Chenkai Wu, Felicia Wu, YaJuan Wu, Zenghong Wu, Hong Xiao, Suowen Xu, Xiaoyue Xu, Kazumasa Yamagishi, Danting Yang, Yuichiro Yano, Amir Yarahmadi, Habib Yaribeygi, Yuichi Yasufuku, Hiroshi Yatsuya, Fereshteh Yazdanpanah, Mohammad Hosein Yazdanpanah, Pengpeng Ye, Renjulal Yesodharan, Saber Yezli, Siyan Yi, Xinglin Yi, Dehui Yin, Dong Keon Yon, Naohiro Yonemoto, Chuanhua Yu, Elaine A Yu, Ke Yun, Hadiza Yusuf, Siddhesh Zadey, Nima Zafari, Burhan Abdullah Zaman, Sojib Bin Zaman, Aurora Zanghì, Iman Zare, Fatemeh Zarimeidani, Armin Zarrintan, Michael Zastrozhin, Dawit Zemedikun, Youjie Zeng, Beijian Zhang, Haijun Zhang, Liqun Zhang, Yunquan Zhang, Zhiqiang Zhang, Hanqing Zhao, Claire Chenwen Zhong, Shang Cheng Zhou, Bin Zhu, Lei Zhu, Abzal Zhumagaliuly, Makan Ziafati, Magdalena Zielińska, Yossef Teshome Zikarg, Ghazal Zoghi, Sa'ed H Zyoud, Samer H Zyoud, Catherine O Johnson, Gregory A Roth, Balakrishnan Sukumaran Nair, Ilari Rautalin, Anjali Bhati, Catherine Bisignano, Theo Vos, Christopher J L Murray

**Affiliations:** a National Institute for Stroke and Applied Neurosciences, Auckland University of Technology, Auckland, New Zealand; b Institute for Health Metrics and Evaluation, University of Washington, Seattle, WA, USA; c Research Center of Neurology, Moscow, Russia; d Department of Nursing, Woldia University, Woldia, Ethiopia; e Department of Clinical Governance and Quality Improvement, Aleta Wondo General Hospital, Aleta Wondo, Ethiopia; f Department of Epidemiology, Alexandria University, Alexandria, Egypt; g Department of Neurology, Cairo University, Cairo, Egypt; h Department of Neurology, Cairo University, Cairo, Egypt; i Department of Mathematics and Sciences, Ajman University, Ajman, United Arab Emirates; j Department of Surgery, Marshall University, Huntington, WV, USA; k Department of Tropical Medicine and Infectious Diseases, Tanta University, Tanta, Egypt; l Department of Medicine, Memorial University, St. John's, NL, Canada; m Minimally Invasive Surgery Research Center, Iran University of Medical Sciences, Tehran, Iran; n Department of Medicine, University of Setif Algeria, Sétif, Algeria; o Department of Health, Sétif, Algeria; p Evidence-Based Practice Center (EBP), Mayo Clinic, Rochester, MN, USA; q Community and Maternity Nursing Unit, University of Duhok, Duhok, Iraq; r Department of Physiotherapy, Bayero University Kano, Kano, Nigeria; s Department of Physiotherapy, Federal University Wukari, Wukari, Nigeria; t Department of Midwifery, Dilla University, Dilla, Ethiopia; u Postgraduate Department, University of Sierra Sur, Miahuatlan de Porfirio Diaz, Mexico; v Yhteiskuntadatatieteen Keskus (Center for Social Data Science), University of Helsinki, Helsinki, Finland; w Department of Botany, Sree Narayana Guru College Chelannur, Kozhikode, India; x Department of Internal Medicine, Federal Medical Centre, Abuja, Nigeria; y Department of Community Medicine, Babcock University, Ilishan-Remo, Nigeria; z Department of Neurosurgery, Medical University of South Carolina, Charleston, SC, USA; aa Department of Surgery, Damascus University, Damascus, Syria; ab Department of Family and Community Health, University of Health and Allied Sciences, Ho, Ghana; ac Department of Physical Pharmacy and Pharmacokinetics, Poznan University of Medical Sciences, Poznan, Poland; ad Department of Pediatric Dentistry of the School of Dentistry, Federal University of Minas Gerais, Belo Horizonte, Brazil; ae Department of Adult Health Nursing, Aksum University, Aksum, Ethiopia; af Department of Anesthesiology, Shahid Beheshti University of Medical Sciences, Tehran, Iran; ag Department of Neurology, Martin Luther University Halle-Wittenberg, Halle (Saale), Germany; ah Department of Neurology, Cairo University, Cairo, Egypt; ai Department of Nursing, Al Zaytoonah University of Jordan, Amman, Jordan; aj Department of Biopharmaceutics and Clinical Pharmacy, The University of Jordan, Amman, Jordan; ak Clinical Sciences Department, University of Sharjah, Sharjah, United Arab Emirates; al Academic Health System, Hamad Medical Corporation, Doha, Qatar; am Institute of Community and Public Health, Birzeit University, Ramallah, Palestine; an Department of Therapeutics, United Arab Emirates University, Al Ain, United Arab Emirates; ao School of Pharmacy, The University of Jordan, Amman, Jordan; ap Department of Biochemistry and Molecular Medicine, Alfaisal University, Riyadh, Saudi Arabia; aq College of Graduate Health Sciences, University of Tennessee, Memphis, TN, USA; ar Department of Clinical Medicine, American University of Antigua, Coolidge, Antigua and Barbuda; as FIU Robert Stempel College of Public Health & Social Work, Florida International University, Miami, FL, USA; at Department of Anesthesia and Critical Care, Debre Tabor University, Debre Tabor, Ethiopia; au College of Medicine and Health Sciences, Bahir Dar University, Bahir Dar, Ethiopia; av School of Medicine, University of Sydney, Sydney, NSW, Australia; aw Centre for Social Research in Health, University of New South Wales, Sydney, NSW, Australia; ax Department of Medical Rehabilitation, Obafemi Awolowo University, Ile-Ife, Nigeria; ay Menzies School of Health Research, Charles Darwin University, Darwin, NT, Australia; az Department of Obstetrics and Gynecology, University of Texas Medical Branch, Galveston, TX, USA; ba Department of Community Medicine, Tribhuvan University, Bharatpur, Nepal; bb Public Health Section, Himalayan Environment and Public Health Network (HEPHN), Chitwan, Nepal; bc Department of Public Health, Universitas Padjadjaran (Padjadjaran University), Bandung, Indonesia; bd Clinical Sciences Department, University of Sharjah, Sharjah, United Arab Emirates; be Department of Epidemiology and Biostatistics, University of Health and Allied Sciences, Ho, Ghana; bf Department of Internal Medicine, Cairo University, Cairo, Egypt; bg Technical Services Directorate, MSI Nigeria Reproductive Choices, Abuja, Nigeria; bh School of Medicine, Iran University of Medical Sciences, Tehran, Iran; bi Department of Life Sciences, University of Management and Technology, Lahore, Pakistan; bj Department of Community Medicine, King Edward Memorial Hospital, Lahore, Pakistan; bk Department of Public Health, Public Health Institute, Lahore, Pakistan; bl Department of Biotechnology, Shahid Beheshti University of Medical Sciences, Tehran, Iran; bm Department of Geography and Planning, Queen's University, Kingston, ON, Canada; bn School of Public Health, University of Technology Sydney, Sydney, NSW, Australia; bo Department of Medical Biochemistry, Shaqra University, Shaqra, Saudi Arabia; bp Clinical Department, The University of Jordan, Amman, Jordan; bq Department of Health and Biological Sciences, Abasyn University, Peshawar, Pakistan; br Department of Natural Sciences, Lebanese American University, Beirut, Lebanon; bs Department of Medical Oncology, Miami Cancer Institute, Miami, FL, USA; bt Department of Community Medicine and Preventive Health, King Edward Medical University Lahore, Lahore, Pakistan; bu School of Public Health, Zhejiang University, Hangzhou, China; bv Department of Neuroscience, Mashhad University of Medical Sciences, Mashhad, Iran; bw Department of Pharmacy Practice, Riphah Institute of Pharmaceutical Sciences, Islamabad, Pakistan; bx Division of Infectious Diseases and Global Public Health (IDGPH), University of California San Diego, San Diego, CA, USA; by Institute of Endemic Diseases, University of Khartoum, Khartoum, Sudan; bz Swiss Tropical and Public Health Institute, University of Basel, Basel, Switzerland; ca Department of Biosciences, COMSATS Institute of Information Technology, Islamabad, Pakistan; cb Brody School of Medicine, East Carolina University, Greenville, NC, USA; cc National Nutrition and Food Technology Research Institute, Shahid Beheshti University of Medical Sciences, Tehran, Iran; cd Faculty of Medicine and Public Health, Jenderal Soedirman University, Purwokerto, Indonesia; ce Moyen Mono Health District, Ministry of Health, Tohoun, Togo; cf Institute for Advanced Medical Research and Training, University of Ibadan, Ibadan, Nigeria; cg Institute of Neuroscience, Newcastle University, Newcastle upon Tyne, UK; ch Department of Cardiology, Fudan University, Shanghai, China; ci Chicago College of Osteopathic Medicine, Midwestern University, Downers Grove, IL, USA; cj Feinberg School of Medicine, Northwestern University, Chicago, IL, USA; ck Department of Communicable Diseases, Ministry of Health, Muscat, Oman; cl Middle East, Eurasia, and Africa Influenza Stakeholders Network, Muscat, Oman; cm Department of Geriatric and Long Term Care, Hamad Medical Corporation, Doha, Qatar; cn Rumailah Hospital, Hamad Medical Corporation, Doha, Qatar; co Department of Surgery, Washington University in St. Louis, St Louis, MO, USA; cp Al Al-Bayt University, Mafraq, Jordan; cq Department of Internal Medicine, Allegheny Health Network, Pittsburgh, PA, USA; cr School of Medicine, New York Medical College, Valhalla, NY, USA; cs Department of Epidemiology, Columbia University, New York, USA; ct Department of Clinical Sciences, Ajman University, Ajman, United Arab Emirates; cu Department of Biology, University of Bahrain, Zallaq, Bahrain; cv Department of Research and Development, Washington University in St. Louis, St Louis, MO, USA; cw Clinical Epidemiology Center, US Department of Veterans Affairs (VA), St Louis, MO, USA; cx School of Nursing, Yarmouk University, Irbid, Jordan; cy School of Nursing and Midwifery, Western Sydney University, Sydney, NSW, Australia; cz Division of Gastroenterology and Hepatology, Mayo Clinic, Jacksonville, FL, USA; da General Directorate of Research and Studies, Ministry of Health, Riyadh, Saudi Arabia; db Robert Stempel College of Public Health and Social Work, Florida International University, Miami, FL, USA; dc Department of Neurology, Charité Medical University Berlin, Berlin, Germany; dd Global Centre for Environmental Remediation, University of Newcastle, Newcastle, NSW, Australia; de Cooperative Research Centre for Contamination Assessment and Remediation of the Environment, Newcastle, NSW, Australia; df College of Nursing, Qatar University, Doha, Qatar; dg Psychological Sciences Association, Amman, Jordan; dh Adult Health and Critical Care Department, Sultan Qabos University, Alseeb, Oman; di Department of Zoology, Abdul Wali Khan University Mardan, Mardan, Pakistan; dj Department of Medical Rehabilitation (Physiotherapy), University of Maiduguri, Maiduguri, Nigeria; dk Department of Rehabilitation Sciences, Hong Kong Polytechnic University, Hong Kong, China; dl Department of Biosciences, Jamia Millia Islamia, New Delhi, India; dm Center for Biotechnology and Microbiology, University of Swat, Swat, Pakistan; dn Department of Geography, Sultan Qaboos University, Muscat, Oman; do Department of Nuclear Medicine, King Hussein Cancer Center, Amman, Jordan; dp Department of Diagnostic Radiology and Nuclear Medicine, The University of Jordan, Amman, Jordan; dq Institute of Health and Wellbeing, Federation University Australia, Melbourne, VIC, Australia; dr School of Public Health and Preventive Medicine, Monash University, Melbourne, VIC, Australia; ds Department of Public Health and Community Medicine, International Medical University, Kuala Lumpur, Malaysia; dt International Centre for Casemix and Clinical Coding, National University of Malaysia, Bandar Tun Razak, Malaysia; du Department of Cardiology, Heart, Vascular, and Thoracic Institute, Cleveland Clinic Abu Dhabi, Abu Dhabi, United Arab Emirates; dv College of Medicine and Health Sciences Academic Programs, Khalifa University, Abu Dhabi, United Arab Emirates; dw Department of Dentistry, Independent Consultant, Sana'a, Yemen; dx Independent Consultant, Irbid, Jordan; dy Department of Physical Therapy and Rehabilitation Sciences, Jordan University of Science and Technology, Irbid, Jordan; dz Department of Rehabilitation Sciences and Physical Therapy, Jordan University of Science and Technology, Irbid, Jordan; ea Research Program of Epidemiology and Public Health, Hospital del Mar Research Institute Barcelona (IMIM), Barcelona, Spain; eb Department of Experimental and Health Sciences, Biomedical Research Networking Center in Epidemiology and Public Health (CiberESP), Barcelona, Spain; ec Department of Respiratory Care, Prince Sultan Military College of Health Sciences, Dammam, Saudi Arabia; ed Department of Community Medicine, King Abdulaziz University, Jeddah, Saudi Arabia; ee Department of Allied Medical Sciences, Jordan University of Science and Technology, Irbid, Jordan; ef Department of Emergency Medicine, Sana'a University, Sanaa, Yemen; eg Pediatric Emergency Medicine Department, St. Christopher's Hospital for Children, Philadelphia, PA, USA; eh Department of Family and Community Medicine, University of Jeddah, Jeddah, Saudi Arabia; ei Department of Basic Sciences, Yarmouk University, Irbid, Jordan; ej Institute of Molecular Biology and Biotechnology, The University of Lahore, Lahore, Pakistan; ek Research, Policy, and Training Directorate, Jordan Center for Disease Control, Amman, Jordan; el Applied Science Research Center, Applied Science Private University, Amman, Jordan; em Department of Allied Medical Science, Al-Balqa Applied University, Karak, Jordan; en Department of Adult Health and Critical Care, Sultan Qaboos University, Muscat, Oman; eo Research Group in Health Economics, Universidad de Cartagena (University of Cartagena), Cartagena, Colombia; ep Research Group in Hospital Management and Health Policies, Universidad de la Costa (University of the Coast), Barranquilla, Colombia; eq Department of Clinical Pharmacology and Toxicology, Umm Al-Qura University, Makkah, Saudi Arabia; er Department of Rehabilitation Sciences, Jordan University of Science and Technology, Irbid, Jordan; es Department of Medical Sciences, Azal University for Human Development, Sana'a, Yemen; et Department of Clinical Sciences, University of Science and Technology of Fujairah, Fujairah, United Arab Emirates; eu Department of Pediatrics, Cleveland Clinic, Cleveland, OH, USA; ev Department of Pediatric Cardiology, Boston Children's Hospital, Boston, MA, USA; ew Department of Pediatrics, Harvard University, Boston, MA, USA; ex Faculty of Medicine, Jordan University of Science and Technology, Irbid, Jordan; ey Department of Pharmacy Practice and Pharmacotherapeutics, University of Sharjah, Sharjah, United Arab Emirates; ez Department of Clinical Pharmacy, Jordan University of Science and Technology, Irbid, Jordan; fa Department of Biomedical Engineering, German Jordanian University, Amman, Jordan; fb Interdisciplinary Graduate Program in Human Toxicology, University of Iowa, Iowa City, IA, USA; fc Health Policy Research Center, Shiraz University of Medical Sciences, Shiraz, Iran; fd School of Global Public Health, New York University, New York, NY, USA; fe Department of Public Health, Dire Dawa University, Dire Dawa, Ethiopia; ff Public Health and Community Medicine Department, Cairo University, Cairo, Egypt; fg Department of Radiology and Radiological Science, Johns Hopkins University, Baltimore, MD, USA; fh Quran and Hadith Research Center, Baqiyatallah University of Medical Sciences, Tehran, Iran; fi Department of Population and Behavioural Sciences, University of Health and Allied Sciences, Ho, Ghana; fj Department of Maternal and Child Wellbeing, African Population and Health Research Center, Nairobi, Kenya; fk Department of Medicine, University of Jos, Jos, Nigeria; fl Department of Internal Medicine, Jos University Teaching Hospital, Jos, Nigeria; fm Faculty of Pharmacy, Carol Davila University of Medicine and Pharmacy, Bucharest, Romania; fn Centre for Sensorimotor Performance, The University of Queensland, Brisbane, QLD, Australia; fo Neurology Department, Royal Brisbane and Women's Hospital, Brisbane, QLD, Australia; fp Department of Child Neurology, Oregon Health and Science University, Portland, OR, USA; fq Department of Pharmacology, All India Institute of Medical Sciences, Jodhpur, India; fr All India Institute of Medical Sciences, Bhubaneswar, India; fs School of Pharmacy, University of Nottingham Malaysia, Semenyih, Malaysia; ft Department of Epidemiology and Biostatistics, Zahedan University of Medical Sciences, Zahedan, Iran; fu Department of Nursing, University of Massachusetts Boston, Boston, MA, USA; fv Regenerative Medicine, Organ Procurement and Transplantation Multi-disciplinary Center, Guilan University of Medical Sciences, Rasht, Iran; fw Centre for Interdisciplinary Research in Basic Sciences (CIRBSc), Jamia Millia Islamia, New Delhi, India; fx School of Chemical and Life Sciences (SCLS), Jamia Hamdard, New Delhi, India; fy Department of Surgery, Gadjah Mada University, Yogyakarta, Indonesia; fz Department of Medical Laboratory Sciences, University of Nigeria Nsukka, Enugu, Nigeria; ga Operations Department, Breast Without Spot, Enugu, Nigeria; gb Rural Health Research Institute, Charles Sturt University, Orange, NSW, Australia; gc School of Medicine and Public Health, Ateneo De Manila University, Pasig City, Philippines; gd Inter-Agency Committee on Environmental Health, Department of Health Philippines, Manila, Philippines; ge Department of Social Sciences, Berekum College of Education, Berekum, Ghana; gf School of Public Health, Kwame Nkrumah University of Science and Technology, Kumasi, Ghana; gg Department of Psychology, Foundation University Islamabad, Rawalpandi, Pakistan; gh Department of Psychology, Foundation University Islamabad, Rawalpandi, Pakistan; gi Health Management and Economics Research Center, Iran University of Medical Sciences, Tehran, Iran; gj Urology Research Center, Tehran University of Medical Sciences, Tehran, Iran; gk College of Pharmacy, Al Ain University, Abu Dhabi, United Arab Emirates; gl Department of Applied Mathematics, University of Washington, Seattle, WA, USA; gm Institute for Health Metrics and Evaluation, University of Washington, Seattle, WA, USA; gn Department of Health Metrics Sciences, School of Medicine, University of Washington, Seattle, WA, USA; go Health Policy Research Center, Shiraz University of Medical Sciences, Shiraz, Iran; gp College of Art and Science, Ottawa University, Surprise, AZ, USA; gq School of Life Sciences, Arizona State University, Tempe, AZ, USA; gr College of Medicine and Health Sciences, Adigrat University, Adigrat, Ethiopia; gs Department of Medical Laboratory Science, Bahir Dar University, Bahir Dar, Ethiopia; gt Department of Public Health, Birmingham City University, Birmingham, UK; gu Faculty of Nursing, Universitas Airlangga (Airlangga University), Surabaya, Indonesia; gv Department of Neurobiology, Care Sciences and Society, Karolinska Institute, Stockholm, Sweden; gw School of Health and Social Studies, Dalarna University, Falun, Sweden; gx Institute for Biomedical Problems, Russian Academy of Sciences, Moscow, Russia; gy Department of Maternal and Child Health, Sultan Qaboos University, Muscat, Oman; gz Department of Research, Nepal Health Research Council, Kathmandu, Nepal; ha Brigham and Women's Hospital, Harvard University, Boston, MA, USA; hb Non-communicable Diseases Research Center, Tehran University of Medical Sciences, Tehran, Iran; hc Department of Radiologic Sciences, Kuwait University, Sulaibikhat, Kuwait; hd Department of Medical Radiation Sciences, Monash University, Melbourne, VIC, Australia; he School of Public Health, Bahir Dar University, Bahir Dar, Ethiopia; hf Department of Public Health, Debre Tabor University, Debre Tabor, Ethiopia; hg Cabrini Research, Cabrini Health, Malvern, VIC, Australia; hh School of Public Health and Preventative Medicine, Monash University, Melbourne, VIC, Australia; hi Department of Public Health, Jimma University, Jimma, Ethiopia; hj Department of Public Health, Wachemo University, Hossana, Ethiopia; hk University Institute of Radiological Sciences and Medical Imaging Technology, The University of Lahore, Lahore, Pakistan; hl Department of Aging Research Institute, Tabriz University of Medical Sciences, Tabriz, Iran; hm School of Medical Laboratory Sciences, Haramaya University, Harar, Ethiopia; hn School of Architecture, Design, and Planning, University of Sydney, Sydney, NSW, Australia; ho Department of Immunology, Zanjan University of Medical Sciences, Zanjan, Iran; hp School of Medicine and Public Health, University of Newcastle, Newcastle, NSW, Australia; hq Hunter New England Population Health, Wallsend, NSW, Australia; hr Faculty of Nursing, Philadelphia University, Amman, Jordan; hs Department of Forensic Medicine, Lumbini Medical College, Palpa, Nepal; ht Northumbria HealthCare NHS Foundation Trust, Newcastle upon Tyne, UK; hu School of Business, University of Leicester, Leicester, UK; hv Department of Statistics and Econometrics, Bucharest University of Economic Studies, Bucharest, Romania; hw Robarts Research Institute, The University of Western Ontario, London, ON, Canada; hx Department of Health Sciences, Higher College of Technology, Abu Dhabi, United Arab Emirates; hy Department of Physiotherapy, Bayero University Kano, Kano, Nigeria; hz School of Nursing and Public Health, University of KwaZulu-Natal, Durban, South Africa; ia Department of Public Health, Wollega University, Nekemte, Ethiopia; ib Department of Health Behavior and Society, Jimma University, Jimma, Ethiopia; ic Institute of Public Health, Gondar University, Gondar, Ethiopia; id Rural Health Research Institute, Charles Sturt University, Orange, NSW, Australia; ie School of Nursing, Hawassa University, Hawassa, Ethiopia; if Department of Nursing, Injibara University, Injibara, Ethiopia; ig Leeds Institute of Rheumatic and Musculoskeletal Medicine, University of Leeds, Leeds, UK; ih Department of Medical-Surgical Nursing, Hamadan University of Medical Sciences, Hamadan, Iran; ii Institute of Biotechnology and Genetic Engineering, The University of Agriculture, Peshawar, Pakistan; ij Montefiore-Einstein Cerebrovascular Research Lab, Albert Einstein College of Medicine, Bronx, NY, USA; ik Faculty of Medicine, October 6 University, 6th of October City, Egypt; il Department of Physiotherapy, Manipal Academy of Higher Education, Manipal, India; im Department of Population Medicine, Qatar University, Doha, Qatar; in Gomal Center of Biochemistry and Biotechnology, Gomal University, Dera Ismail Khan, Pakistan; io Department of Forensic Science, Government Institute of Forensic Science Nagpur, Nagpur, India; ip Rashtrasant Tukadoji Maharaj Nagpur University, Nagpur, India; iq Department of Biostatistics, Mashhad University of Medical Sciences, Mashhad, Iran; ir School of Medicine, Isfahan University of Medical Sciences, Isfahan, Iran; is School of Public Affairs, Nanjing University of Information Science and Technology, Nanjing, China; it International Medical School, Management and Science University, Alam, Malaysia; iu Department of Forensic Medicine and Toxicology, Manipal Academy of Higher Education, Manipal, India; iv Department of Neurosurgery, Houston Methodist Hospital, Houston, TX, USA; iw College of Medicine, Alfaisal University, Riyadh, Saudi Arabia; ix Center of Innovation, Technology and Education (CITE), Anhembi Morumbi University, São José dos Campos, Brazil; iy Department of Medicine, Monash University, Clayton, VIC, Australia; iz Department of Hypertension, Medical University of Lodz, Lodz, Poland; ja Polish Mothers' Memorial Hospital Research Institute, Lodz, Poland; jb Dental Research Center, Tehran University of Medical Sciences, Tehran, Iran; jc Health Policy Research Center, Shiraz University of Medical Sciences, Shiraz, Iran; jd Nuffield Department of Surgical Sciences, University of Oxford, Oxford, NA, UK; je Department of Neurosurgery, University of Southampton, Southampton, NA, UK; jf Department of Non-communicable Diseases, Bangladesh University of Health Sciences, Dhaka, Bangladesh; jg Department of Medicine, University of Massachusetts Medical School, Worcester, MA, USA; jh Department of Medicine, Saint Vincent Hospital, Worcester, MA, USA; ji National Institute on Drug Dependence, Peking University, Beijing, China; jj Department of Epidemiology and Health Statistics, Peking University, Beijing, China; jk Department of Neurosciences, Costa Rican Department of Social Security, San Jose, Costa Rica; jl School of Medicine, University of Costa Rica, San Pedro, Costa Rica; jm Miller School of Medicine, University of Miami, Miami, FL, USA; jn Department of Translational Medicine, Florida International University, Miami, FL, USA; jo School of Psychology, University of Auckland, Auckland, New Zealand; jp Heidelberg Institute of Global Health (HIGH), Heidelberg University, Heidelberg, Germany; jq T.H. Chan School of Public Health, Harvard University, Boston, MA, USA; jr Clinical Sciences Department, University of Sharjah, Sharjah, United Arab Emirates; js Department of Public and Environmental Health, University of The Gambia, Banjul, The Gambia; jt Department of Epidemiology, University of Florida, Gainesville, FL, USA; ju Department of Non-communicable Diseases, Bangladesh University of Health Sciences, Dhaka, Bangladesh; jv Health Information Management, Shiraz University of Medical Sciences, Shiraz, Iran; jw Department of Animal Sciences, Obafemi Awolowo University, Ile-Ife, Nigeria; jx Department of Veterinary Physiology and Biochemistry, University of Ilorin, Ilorin, Nigeria; jy Non-communicable Diseases Research Center, Tehran University of Medical Sciences, Tehran, Iran; jz School of Medicine, Iran University of Medical Sciences, Tehran, Iran; ka Center for Primary Care, Harvard University, Boston, MA, USA; kb School of Public Health, Imperial College London, London, UK; kc Department of Academics, Indian Institute of Public Health, Gurgaon, India; kd Department of Medical Education, University of Nevada Las Vegas, Las Vegas, NV, USA; ke School of Sport, Exercise and Health Sciences, Loughborough University, Loughborough, UK; kf School of Public Health, Imperial College London, London, UK; kg Avicenna Biotech Research, Germantown, MD, USA; kh Department of Regulatory Affairs, Amarex Clinical Research, Germantown, MD, USA; ki School of Medicine, Tehran University of Medical Sciences, Tehran, Iran; kj Non-Communicable Diseases Research Center (NCDRC), Tehran, Iran; kk Department of Public Health, Bahir Dar University, Bahir Dar, Ethiopia; kl Department of Public Health, University of South Africa, Pretoria, South Africa; km Department of Medicine and Surgery, University of Milano - Bicocca, Milano, Italy; kn Direzione Sanitaria (Department of Health Management), Fondazione IRCCS San Gerardo dei Tintori, Monza, Italy; ko Department of Physiotherapy and Paramedicine, Glasgow Caledonian University, Glasgow, UK; kp Nuffield Department of Population Health, University of Oxford, Oxford, UK; kq Department of Internal Medicine, University of São Paulo, São Paulo, Brazil; kr Department of Epidemiology and Biostatistics, Wollo University, Dessie, Ethiopia; ks School of Medicine and Dentistry, Griffith University, Gold Coast, QLD, Australia; kt Department of Epidemiology and Biostatistics, University of the Philippines Manila, Manila, Philippines; ku Department of Epidemiology, Brown University, Providence, RI, USA; kv Metabolomics Laboratory, Baker Heart and Diabetes Institute, Melbourne, VIC, Australia; kw Department of Microbiology, Addis Ababa University, Addis Ababa, Ethiopia; kx Department of Pharmaceutical and Administrative Sciences, University of Health Sciences and Pharmacy in St. Louis, St Louis, MO, USA; ky School of Pharmacy, University of Auckland, Auckland, New Zealand; kz Department of Forensic Chemistry, Government Institute of Forensic Science, Aurangabad, Aurangabad, India; la Department of Public Health, North Dakota State University, Fargo, ND, USA; lb Institute of Applied Health Research, University of Nottingham, Nottingham, UK; lc Institute of Applied Health Research, University of Birmingham, Birmingham, UK; ld Department of Internal Medicine, Post Graduate Institute of Medical Education and Research, Chandigarh, India; le Department of Anatomy, All India Institute of Medical Sciences, Jodhpur, India; lf Department of Community Medicine and Family Medicine, All India Institute of Medical Sciences, Jodhpur, India; lg School of Public Health, All India Institute of Medical Sciences, Jodhpur, India; lh Global Health Neurology Lab, NSW Brain Clot Bank, Sydney, NSW, Australia; li Division of Cerebrovascular Medicine and Neurology, National Cerebral and Cardiovascular Center, Suita, Japan; lj Department of General Medicine, Manipal Academy of Higher Education, Mangalore, India; lk Department of Internal Medicine, St. John's National Academy of Health Sciences, Bangalore, India; ll Department of Medical Lab Technology, Chandigarh University, Mohali, India; lm Department of Human Genetics and Molecular Medicine, Central University of Punjab, Bathinda, India; ln Department of Pharmacy, University of Asia Pacific, Dhaka, Bangladesh; lo Department of Surgery, University of Maryland School of Medicine, Baltimore, MD, USA; lp Scientific-Tools.Org, Bergamo, Italy; lq Department of Radiology, Mayo Clinic College of Medicine, Rochester, MN, USA; lr Neurovascular Research Laboratory, Mayo Clinic College of Medicine, Rochester, MN, USA; ls Department of General Surgery and Medical-Surgical Specialties, University of Catania, Catania, Italy; lt Department of Midwifery, Debre Markos University, Debre Markos, Ethiopia; lu Department of Neurology, Institute of Post-Graduate Medical Education and Research and Seth Sukhlal Karnani Memorial Hospital, Kolkata, India; lv Department of Community Medicine and Family Medicine, All India Institute of Medical Sciences, Deoghar, India; lw Department of Community & Family Medicine, All India Institute of Medical Sciences, Ramanathapuram, Tamil Nadu, India; lx Department of Health Promotion and Behavioural Science, Bahir Dar University, Bahir Dar, Ethiopia; ly Department of Internal Medicine, Manipal Academy of Higher Education, Mangalore, India; lz Department of Urology, Shahid Beheshti University of Medical Sciences, Tehran, Iran; ma Faculty of Medicine and Pharmaceutical Sciences, University of Douala, Douala, Cameroon; mb Department of Cardiology, Centre Hospitalier Montfermeil (Montfermeil Hospital Center), Montfermeil, France; mc General Directorate of Health Information Systems, Ministry of Health, Ankara, Turkiye; md Internal Medicine Department, Shahid Beheshti University of Medical Sciences, Tehran, Iran; me Disease Surveillance Department, Ghana Health Service, Ho, Ghana; mf Department of Epidemiology and Biostatistics, University of Health and Allied Sciences, Ho, Ghana; mg Department of Medicine, University Ferhat Abbas of Setif, Setif, Algeria; mh Department of Epidemiology and Preventive Medicine, University Hospital Saadna Abdenour, Setif, Algeria; mi Department of Biology, Mohammed V University in Rabat, Rabat, Morocco; mj School of Medical and Life Sciences, Sunway University, Sunway, Malaysia; mk School of Medicine, University of Washington, Seattle, WA, USA; ml General Medicine Service, Department of Veterans Affairs, Seattle, WA, USA; mm Faculty of Medicine, University of Belgrade, Belgrade, Serbia; mn University Eye Hospital, Clinical Center of Serbia, Belgrade, Serbia; mo Department of Epidemiology, University of Florida, Gainesville, FL, USA; mp Cancer Population Sciences Program, University of Florida Health Cancer Center, Gainesville, FL, USA; mq Institute for Medical Information Processing, Biometry, and Epidemiology, LMU Munich, Neuherberg, Germany; mr Institute of Epidemiology, Helmholtz Zentrum München (German Research Center for Environmental Health), Neuherberg, Germany; ms Division of Clinical Epidemiology and Aging Research, German Cancer Research Center, Heidelberg, Germany; mt Center for Neuroscience, Institute for Scientific Research and High Technology Services, Panama City, Panama; mu Gorgas Memorial Institute for Health Studies, Panama City, Panama; mv Department of Internal Medicine, University of São Paulo, São Paulo, Brazil; mw Department of Psychiatry, University of São Paulo, São Paulo, Brazil; mx Institute for Health Metrics and Evaluation, University of Washington, Seattle, WA, USA; my Department of Medical and Surgical Sciences, University of Bologna, Bologna, Italy; mz College of Nursing and Health Sciences, Flinders University, Adelaide, SA, Australia; na Institute for Health Metrics and Evaluation, University of Washington, Seattle, WA, USA; nb Department of Health Metrics Sciences, School of Medicine, University of Washington, Seattle, WA, USA; nc School of Pharmacy, The University of Jordan, Amman, Jordan; nd Department of Basic Biomedical Sciences, University of Sharjah, Sharjah, United Arab Emirates; ne School of Public Health Sciences, University of Waterloo, Waterloo, ON, Canada; nf Al Shifa School of Public Health, Al Shifa Trust Eye Hospital, Rawalpindi, Pakistan; ng Harvard Business School, Harvard University, Boston, MA, USA; nh Department of Internal Medicine, Hospital Italiano de Buenos Aires (Italian Hospital of Buenos Aires), Buenos Aires, Argentina; ni Board of Directors, Argentine Society of Medicine, Buenos Aires, Argentina; nj Institute of Biomedical Engineering, Anhembi Morumbi University, Sao Jose dos Campos, Brazil; nk Department of Biomedical Engineering, Center of Innovation, Technology and Education (CITE) at São José dos Campos Technology Park, Sao Jose dos Campos, Brazil; nl Center for Nutrition and Health Research, National Institute of Public Health, Cuernavaca, Mexico; nm Department of Ophthalmology, Beijing Institute of Ophthalmology, Beijing, China; nn Department of Health Management (Direzione Sanitaria), IRCCS Istituto Ortopedico Rizzoli, Bologna, Italy; no Interdisciplinary Research Center for Health Science, Sant'Anna School of Advanced Studies, Pisa, Italy; np Department of Health Care, Metropolitan Autonomous University, Mexico City, Mexico; nq Department of Epidemiology, Harvard University, Boston, MA, USA; nr Institute for Cancer Research, Prevention and Clinical Network, Florence, Italy; ns Institute for Mental and Physical Health and Clinical Translation (IMPACT), Deakin University, Geelong, VIC, Australia; nt Research Unit on Applied Molecular Biosciences (UCIBIO), University of Porto, Porto, Portugal; nu Department of Psychiatry, University of São Paulo, São Paulo, Brazil; nv Colombian National Health Observatory, Instituto Nacional de Salud, Bogota, Colombia; nw Epidemiology and Public Health Evaluation Group, National University of Colombia, Bogota, Colombia; nx Centre Neuchatelois de Psychiatrie, Marin Epagnier, Switzerland; ny Mental Health Flagship, World Health Organization (WHO), Copenhagen, Denmark; nz Department of Pharmacological and Biomolecular Sciences, University of Milan, Milan, Italy; oa MultiMedica Sesto San Giovanni IRCCS, Sesto San Giovanni, Italy; ob Department of Public Health and Infectious Diseases, La Sapienza University, Rome, Italy; oc Department of Medical, Surgical, and Health Sciences, University of Trieste, Trieste, Italy; od Public Health Unit, University Health Agency Giuliano-Isontina (ASUGI), Trieste, Italy; oe Department of Nutrition, Federal University of Santa Catarina, Florianópolis, Brazil; of Department of Medical and Surgical Sciences, University of Bologna, Bologna, Italy; og Mary MacKillop Institute for Health Research, Australian Catholic University, Melbourne, VIC, Australia; oh School of Public Health, University of Hong Kong, Hong Kong, China; oi Non-communicable Diseases Division, National Institute of Epidemiology, Chennai, India; oj Department of Biotechnology, Adamas University, Kolkata, India; ok Institute for Skeletal Aging & Orthopedic Surgery, Hallym University, Chuncheon, South Korea; ol State Disease Investigation Laboratory, Animal Resources Development Department, Agartala, India; om Cardio-Oncology Research Unit, Cardiovascular Analytics Group, Hong Kong, China; on Department of Clinical Nutrition, Jazan University, Jazan, Saudi Arabia; oo Department of Anesthesiology and Perioperative Medicine, University of Rochester, Rochester, NY, USA; op Department of Clinical Pharmacy, University of Gondar, Gondar, Ethiopia; oq Temerty Faculty of Medicine, University of Toronto, Toronto, ON, Canada; or Department of Community Medicine, Datta Meghe Institute of Medical Sciences, Sawangi, India; os Department of Biology, Al-Imam Mohammad Ibn Saud Islamic University, Riyadh, Saudi Arabia; ot Oral Medicine and Radiology, King George's Medical University, Lucknow, India; ou Clinical Research Center, Zhujiang hospital of Southern Medical University, Guangzhou, China; ov Department of Endocrine and Metabolic Diseases, Shanghai Jiao Tong University, Shanghai, China; ow Heidelberg Institute of Global Health (HIGH), Heidelberg University, Heidelberg, Germany; ox Division of Cardiovascular Medicine, Harvard University, Boston, MA, USA; oy Department of Scientific Research, Tehran University of Medical Sciences, Tehran, Iran; oz Translational Health Research Institute, Western Sydney University, Sydney, NSW, Australia; pa Westmead Clinical School, University of Sydney, Sydney, NSW, Australia; pb Division of Infectious Diseases, Virginia Commonwealth University, Richmond, VA, USA; pc Iraq Field Epidemiology Training Program (I-FETP), Ministry of Health, Baghdad, Iraq; pd Program in Medical and Population Genetics, Broad Institute of MIT and Harvard, Cambridge, MA, USA; pe Cardiovascular Research Center, Massachusetts General Hospital, Boston, MA, USA; pf Cancer Big Data Center, National Cancer Center, Goyang, South Korea; pg Department of Medicine, National University of Singapore, Singapore, Singapore; ph RIPAS Hospital, University of Brunei Darussalam, BSB, Brunei; pi Centre for Research Impact & Outcome, Chitkara University, Rajpura, India; pj Department of Community Medicine, Jawaharlal Nehru Medical College, Wardha, India; pk Department of Cardiology, All India Institute of Medical Sciences, Jodhpur, India; pl Center for Biomedicine and Community Health, International School, Vietnam National University Hanoi (VNUIS), Hanoi, Vietnam; pm Department of Paediatric Surgery, Federal Medical Centre, Umuahia, Nigeria; pn Department of Health Informatics, University College London, London, UK; po Health Data Research UK, London, UK; pp Department of Genetics, University of Pennsylvania, Philadelphia, PA, USA; pq Department of Food, Environmental and Nutritional Sciences, University of Milan, Milan, Italy; pr Institute for Health Metrics and Evaluation, University of Washington, Seattle, WA, USA; ps Department of Biostatistics, Johns Hopkins University, Baltimore, MD, USA; pt Department of Epidemiology and Prevention, IRCCS Neuromed, Pozzilli, Italy; pu Department of Chemical Sciences, University of Porto, Porto, Portugal; pv Department of Family Medicine and Public Health, University of California San Diego, La Jolla, CA, USA; pw Department of Diagnostic and Therapeutic Technologies, Cooperativa de Ensino Superior Politécnico e Universitário (Polytechnic and University Higher Education Cooperative), Vila Nova de Famalicão, Portugal; px Institute for Research and Innovation in Health (i3S), University of Porto, Porto, Portugal; py Center for Health Systems Research, National Institute of Public Health, Cuernavaca, Mexico; pz School of Nursing, Federal University of Minas Gerais, Belo Horizonte, Brazil; qa Department of Internal Medicine, Cheyenne Regional Medical Center, Cheyenne, WY, USA; qb Department of Global Public Health and Primary Care, University of Bergen, Bergen, Norway; qc Iranian Research Center for HIV/AIDS (IRCHA), Tehran University of Medical Sciences, Tehran, Iran; qd Institute for Health Metrics and Evaluation, University of Washington, Seattle, WA, USA; qe Department of Health Metrics Sciences, School of Medicine, University of Washington, Seattle, WA, USA; qf Institute for Health Sciences, Mid Sweden University, Sundsvall, Sweden; qg Stroke and Ageing Research Group, Monash University, Clayton, VIC, Australia; qh IRCCS Istituto Ortopedico Galeazzi, University of Milan, Milan, Italy; qi Department of Dermatology, Case Western Reserve University, Cleveland, OH, USA; qj Department of Medical and Surgical Sciences and Advanced Technologies "GF Ingrassia", University of Catania, Catania, Italy; qk Public Health Foundation of India, Gurugram, India; ql Institute for Health Metrics and Evaluation, University of Washington, Seattle, WA, USA; qm Indian Council of Medical Research, New Delhi, India; qn Public Health Foundation of India, Gurugram, India; qo Institute for Health Metrics and Evaluation, University of Washington, Seattle, WA, USA; qp Department of Health Metrics Sciences, School of Medicine, University of Washington, Seattle, WA, USA; qq Department of Pediatrics, Tanta University, Tanta, Egypt; qr Department of Biochemistry, Ministry of Health and Welfare, New Delhi, India; qs Immunology Research Center, Tabriz University of Medical Sciences, Tabriz, Iran; qt Department of obstetrics and Gynecology, Tehran University of Medical Sciences, Tehran, Iran; qu Department of Gynecology and Obstetrics, Vali-E-Asr Reproductive Health Research Center, Tehran, Iran; qv Department of Neurology, Tehran University of Medical Sciences, Tehran, Iran; qw Interdisciplinary Neuroscience Research Program, Tehran University of Medical Sciences, Tehran, Iran; qx Department of Radiology, Iran University of Medical Sciences, Tehran, Iran; qy Tehran University of Medical Sciences, Tehran, Iran; qz Health Research Institute, Kazakh National Medical University, Almaty, Kazakhstan; ra Center for Evaluation and Surveys Research, National Institute of Public Health, Cuernavaca, Mexico; rb Department of Internal Medicine, John H. Stroger, Jr. Hospital of Cook County, Chicago, IL, USA; rc Department of Health Policy and Management, Haramaya University, Harar, Ethiopia; rd Medical College, Albany Medical College, Albany, NY, USA; re School of Medicine, University of Colima, Colima, Mexico; rf Department of Research, Colima State Cancer Institute, Colima, Mexico; rg USAID-JSI Digital Health Activity, Jimma University, Addis Ababa, Ethiopia; rh Department of Neurosurgery, University of Edinburgh, Edinburgh, UK; ri Department of Neurosurgery, National Health Service (NHS) Scotland, Edinburgh, UK; rj Center for Nutrition and Health Research, National Institute of Public Health, Cuernavaca, Mexico; rk Department of Forensic Medicine, University of Sarajevo, Sarajevo, Bosnia and Herzegovina; rl Department of Research, Gujarat Adani Institute of Medical Sciences and G.K. General Hospital, Bhuj, India; rm Department of Public Health, Debre Tabor University, Debre Tabor, Ethiopia; rn Department of Public Health, Madda Walabu University, Goba, Ethiopia; ro Chettinad Hospital & Research Institute, Chettinad Academy of Research and Education, Chennai, India; rp JSS Medical College Department of Biochemistry, Jagadguru Sri Shivarathreeswara University, Mysuru, India; rq Department of Pharmacy, United International University, Dhaka, Bangladesh; rr Pharmacology Division, Center for Life Sciences Research Bangladesh, Dhaka, Bangladesh; rs Research and Development Cell, Dr. D. Y. Patil University, Pune, India; rt Research Department, Nepal Health Research Council, Kathmandu, Nepal; ru Institute of Occupational, Social and Environmental Medicine, Goethe University Frankfurt, Frankfurt am Main, Germany; rv The Zena and Michael A. Wiener Cardiovascular Institute, Icahn School of Medicine at Mount Sinai, New York, NY, USA; rw College of Medicine, University of Florida, Gainesville, FL, USA; rx Department of Human Physiology, University of Gondar, Gondar, Ethiopia; ry School of Medicine, Iran University of Medical Sciences, Tehran, Iran; rz Institute of Health Research, University of Health and Allied Sciences, Ho, Ghana; sa Department of Epidemiology, Jazan University, Jazan, Saudi Arabia; sb Neurology Department, Alexandria University, Alexandria, Egypt; sc Department of Social Medicine and Health Care Organisation, Medical University of Varna, Varna, Bulgaria; sd Health Science Center, University of Texas, Houston, TX, USA; se Department of Medical, Surgical, and Health Sciences, University of Trieste, Trieste, Italy; sf Cardio-Thoraco-Vascular Department, Azienda Sanitaria Universitaria Giuliano Isontina, Trieste, Italy; sg Independent Consultant, South Plainfield, NJ, USA; sh Department of Cardiology, St. Joseph's University Medical Center, Paterson, NJ, USA; si School of Population Health and Environmental Sciences, King's College London, London, UK; sj Department of Epidemiology and Biostatistics, University of Health and Allied Sciences, Ho, Ghana; sk Department of Medicine, Bangalore Medical College and Research Institute, Bangalore, India; sl Manipal Academy of Higher Education, Manipal, India; sm Department of Forensic Medicine and Toxicology, Kasturba Medical College Mangalore, Mangalore, India; sn Prasanna School of Public Heath (PSPH), Manipal Academy of Higher Education, Manipal, India; so Postgraduate Program in Epidemiology, Federal University of Rio Grande do Sul, Porto Alegre, Brazil; sp School of Medicine, Federal University of Bahia, Salvador, Brazil; sq Department of Internal Medicine, Escola Bahiana de Medicina e Saúde Pública (Bahiana School of Medicine and Public Health), Salvador, Brazil; sr Department of Conservative Dentistry with Endodontics, Medical University of Silesia, Katowice, Poland; ss Faculty of Science and Health, University of Portsmouth, Hampshire, UK; st Department of Public Health and Community Medicine, Texila American University, Tanta city, Egypt; su School of Public health, Texila American University, Guyana, Guyana; sv Department of Clinical Pathology, Mansoura University, Mansoura, Egypt; sw Institute of Public Health, United Arab Emirates University, Al Ain, United Arab Emirates; sx Clinical Pharmacy Program, Al Ain University, Al Ain, United Arab Emirates; sy AAU Health and Biomedical Research Center, Al Ain University, Abu Dhabi, United Arab Emirates; sz Division of Cardiovascular Medicine, University of Kentucky, Lexington, KY, USA; ta Division of Cardiology, Harvard University, Boston, MA, USA; tb Faculty of Medicine, University of Tripoli, Tripoli, Libya; tc Houston Methodist Hospital, Houston, TX, USA; td Department of Basic Medical Sciences, University of Sharjah, Sharjah, United Arab Emirates; te Egypt Center for Research and Regenerative Medicine (ECRRM), Cairo, Egypt; tf Basic Medical Sciences Department, University of Sharjah, Sharjah, United Arab Emirates; tg Research Institute of Medical & Health Sciences, University of Sharjah, Sharjah, United Arab Emirates; th Department of Pediatrics, Texas A&M University, Dallas, TX, USA; ti Department of Public Health and Tropical Medicine, James Cook University, Townsville, QLD, Australia; tj Department of Paediatrics, University of Lagos, Lagos, Nigeria; tk Department of Paediatrics, Lagos University Teaching Hospital, Lagos, Nigeria; tl Division of Epidemiology, University of Tehran, Tehran, Iran; tm Department of Cardiology, Tehran University of Medical Sciences, Tehran, Iran; tn Dipartimento di Scienze Biomediche e Neuromotorie (DIBINEM), University of Bologna, BOLOGNA, Italy; to Department of Rehabilitation Sciences, Jordan University of Science and Technology, Irbid, Jordan; tp Independent Consultant, Bologna, Italy; tq Division of Non-Communicable Diseases, Ministry of Public Health and Population, Dubai, United Arab Emirates; tr Department of Epidemiology and Medical Statistics, University of Ibadan, Ibadan, Nigeria; ts Research Centre for Healthcare and Community, Coventry University, Coventry, UK; tt Department of Periodontology and Community Dentistry, University of Ibadan, Ibadan, Nigeria; tu Department of Periodontology and Community Dentistry, University College Hospital, Ibadan, Ibadan, Nigeria; tv Interdisciplinary Neuroscience Research Program, Tehran University of Medical Sciences, Tehran, Iran; tw Department of Oral Biology, The University of Lahore, Lahore, Pakistan; tx director of the scientific and technological park, Kazakh National Medical University, Almaty, Kazakhstan; ty Department of Biomedical and Biotechnological Sciences, University of Catania, Catania, Italy; tz Epidemiology and Biostatistics Unit, IRCCS Pascale, Naples, Italy; ua Center for Global Health Research, Saveetha Institute of Medical and Technical Sciences (SIMATS), Chennai, India; ub Department of Neurological Surgery, Northwestern University, Chicago, IL, USA; uc Dissemination Division, National Institute of Statistics, Lisbon, Portugal; ud Activity Planning and Control Unit, Directorate-General of Health (DGS), Lisbon, Portugal; ue Department of Clinical Nutrition and Dietetics, Applied Science Private University, Amman, Jordan; uf Department of Biology, Salahaddin University-Erbil, Erbil, Iraq; ug Department of Biology, Cihan University-Erbil, Erbil, Iraq; uh Medical School, Shiraz University of Medical Sciences, Shiraz, Iran; ui Epilepsy Research Center, Shiraz University of Medical Sciences, Shiraz, Iran; uj Department of Psychology, Federal University of Sergipe, São Cristóvão, Brazil; uk School of Medicine, Tehran University of Medical Sciences, Tehran, Iran; ul Endocrinology and Metabolism Research Institute, Non-Communicable Diseases Research Center (NCDRC), Tehran, Iran; um Satcher Health Leadership Institute, Morehouse School of Medicine, Atlanta, GA, USA; un School of Medicine, Emory University, Atlanta, GA, USA; uo Centre for Public Health, Equity and Human Flourishing, Torrens University Australia, Adelaide, SA, Australia; up Institute of Resource Governance and Social Change, Kupang, Indonesia; uq Department of Biology and Medicine, Brown University, Providence, RI, USA; ur Laboratory of Experimental Medicine, Kazakh National Medical University, Almaty, Kazakhstan; us Department of Social Medicine and Epidemiology, Guilan University of Medical Sciences, Rasht, Iran; ut Department of Public Health and Infectious Diseases, City University of Hong Kong, Hong Kong, China; uu Department of Pharmacy, Wollega University, Nekemte, Ethiopia; uv School of Population Health, University of New South Wales, Sydney, NSW, Australia; uw National Institute of Environmental Health, Chinese Center for Disease Control and Prevention, Beijing, China; ux Division of Neurology, University of Toronto, Toronto, ON, Canada; uy Department of Neurobiology, Care Sciences, and Society, Karolinska Institute, Stockholm, Sweden; uz Center for Public Health Research, University of Milan Bicocca, Monza, Italy; va Laboratory of Public Health, IRCCS Istituto Auxologico Italiano, Milan, Italy; vb Department of Social Sciences, University of Nicosia, Nicosia, Cyprus; vc Department of Nursing, Wollega University, Nekemte, Ethiopia; vd Institute of Health Sciences, Wollega University, Nekemte, Ethiopia; ve Jimma University, Jimma, Ethiopia; vf Institute of Public Health, Charité Medical University Berlin, Berlin, Germany; vg Institute for Health Metrics and Evaluation, University of Washington, Seattle, WA, USA; vh Department of Health Metrics Sciences, School of Medicine, University of Washington, Seattle, WA, USA; vi Centre for Public Health, Equity and Human Flourishing, Torrens University Australia, Adelaide, SA, Australia; vj College of Nursing and Health Sciences, Flinders University, Adelaide, SA, Australia; vk Department of Neurology, University of Lisbon, Lisbon, Portugal; vl Department of Neurology, Hospital de Santa Maria, Lisbon, Portugal; vm Endocrinology and Metabolism Research Institute, Tehran University of Medical Sciences, Tehran, Iran; vn Department of Surgery, Tehran University of Medical Sciences, Tehran, Iran; vo Department of Pharmacology, Iranshahr University of Medical Sciences, Iranshahr, Iran; vp Innovation in Healthcare and Social Services Department, Emilia-Romagna Region, Bologna, Italy; vq Department of Neuroscience, Multiple Sclerosis Research Center, Ravenna, Italy; vr Department of Biotechnological and Applied Clincial Sciences, University of L'Aquila, L'Aquila, Italy; vs College of Public Health, Medical, and Veterinary Sciences, James Cook University, Townsville, QLD, Australia; vt Graduate Institute of Injury Prevention and Control, Taipei Medical University, Taipei, Taiwan; vu Department of Community Medicine and Family Medicine, All India Institute of Medical Sciences, Gorakhpur, India; vv Health Services Management Training Centre, Semmelweis University, Budapest, Hungary; vw Department of Applied Social Sciences, Sapientia Hungarian University of Transylvania, Târgu-Mureş, Romania; vx Department of Community Medicine, Datta Meghe Institute of Medical Sciences, Wardha, India; vy Department of Medicine, Nazarbayev University, Astana, Kazakhstan; vz Department of Food Technology, Salahaddin University-Erbil, Erbil, Iraq; wa Department of Nutrition and Dietetics, Cihan University-Erbil, Erbil, Iraq; wb Department of Medical Epidemiology, Mario Negri Institute for Pharmacological Research, Milan, Italy; wc Department of Community Medicine and Family Medicine, All India Institute of Medical Sciences, Nagpur, India; wd Institute of Health and Wellbeing, Federation University Australia, Churchill, VIC, Australia; we School of Public Health and Preventive Medicine, Monash University, Melbourne, VIC, Australia; wf The Centre for Global Health, University of Edinburgh, Edinburgh, UK; wg Professional Services Division, Texas State Board of Pharmacy, Austin, TX, USA; wh Department of Pharmacology, Indore Institute of Pharmacy, Indore, India; wi Department of Midwifery, Adigrat University, Adigrat, Ethiopia; wj Department of Environmental Health, Wollo University, Dessie, Ethiopia; wk Department of Nursing, Mekelle University, Mekelle, Ethiopia; wl Department of Public Health, Debre Berhan University, Debre Berhan, Ethiopia; wm Department of Public Health, Menelik II Medical and Health Science College, Addis Ababa, Ethiopia; wn Department of Medical Laboratory Science, Addis Ababa University, Addis Ababa, Ethiopia; wo School of Medicine, Shahid Beheshti University of Medical Sciences, Tehran, Iran; wp Psychiatric Nursing and Management Department, Shahid Beheshti University of Medical Sciences, Tehran, Iran; wq Department of Cardiology, Iran University of Medical Sciences, Tehran, Iran; wr Faculty of Medicine, Shahid Beheshti University of Medical Sciences, Tehran, Iran; ws Department of Medical Surgical Nursing, Tabriz University of Medical Sciences, Tabriz, Iran; wt Department of Medical Genetics, Shahid Beheshti University of Medical Sciences, Tehran, Iran; wu Center for Comprehensive Genetic Services, Shahid Beheshti University of Medical Sciences, Tehran, Iran; wv Immunology Research Center, Tabriz University of Medical Sciences, Tabriz, Iran; ww Tropical Health Department, Alexandria University, Alexandria, Egypt; wx Family and Community Medicine Department, King Khalid University Hospital, Abha, Saudi Arabia; wy Electrical and Computer Engineering, University of California Davis, Davis, CA, USA; wz Department of Physics, University of Zanjan, Zanjan, Iran; xa Departments of Radiology and Neurosurgery, Mayo Clinic, Rochester, MN, USA; xb SYNAPSIS Mental Health and Neurology Non-Profit Organization, Lima, Peru; xc Sensory Motor Laboratoy, Fondation Asile des Aveugles, Lausanne, Switzerland; xd Department of Medicine, Aga Khan University, Karachi, Pakistan; xe Adelaide Medical School, University of Adelaide, Adelaide, SA, Australia; xf Division of General Internal Medicine, Howard University, Washington, DC, USA; xg Department of Community and Family Medicine, Howard University, Washington, DC, USA; xh Department of Nursing, Ethiopian Public Health Institute, Chiro, Ethiopia; xi Third Department of Neurology, Research Center of Neurology, Moscow, Russia; xj Department of Hepatology, Sanjay Gandhi Postgraduate Institute of Medical Sciences, Lucknow, India; xk Department of Dermatology, Yale University, New Haven, CT, USA; xl Department of Health Systems and Policy Research, Indian Institute of Public Health, Gandhinagar, India; xm Department of Genetics, Sana Institute of Higher Education, Sari, Iran; xn Universal Scientific Education and Research Network (USERN), Kermanshah University of Medical Sciences, Kermanshah, Iran; xo Department of Life Sciences, Health and Healthcare Professions, Link Campus University, Rome, Italy; xp Health Services Research, Evaluation and Policy Unit, AUSL della Romagna, Ravenna, Italy; xq Department of Urban Public Health, University of Massachusetts Boston, Boston, MA, USA; xr Oncological Network, Prevention and Research Institute, Institute for Cancer Research, Prevention and Clinical Network, Florence, Italy; xs Department of Epidemiology, University of São Paulo, São Paulo, Brazil; xt Postgraduate Program in Epidemiology, Federal University of Rio Grande do Sul, Porto Alegre, Brazil; xu School of Medicine, Tehran University of Medical Sciences, Tehran, Iran; xv Institute of Public Health, United Arab Emirates University, Al Ain, United Arab Emirates; xw Department of Public Health and Preventive Medicine, Charles University, Prague, Czech Republic; xx Department of Biomedical and Biotechnological Sciences, University of Catania, Catania, Italy; xy Department of Endocrinology, National Institute of Health, Bethesda, MD, USA; xz Department of Epidemiology and Biostatistics, Anhui Medical University, Hefei, China; ya Post Graduate School of Public Health, University of Siena, Siena, Italy; yb Harrington Heart and Vascular Institute, Case Western Reserve University, Cleveland, OH, USA; yc Division of Cardiovascular Medicine, Ohio State University, Columbus, OH, USA; yd Health Directorate, Local Health Authority of Bologna, Bologna, Italy; ye Department of Biomedical and Neuromotor Sciences, University of Bologna, Bologna, Italy; yf Department of Thoracic Surgery, Cleveland Clinic, Cleveland, OH, USA; yg Department of Community Medicine, University of Peradeniya, Kandy, Sri Lanka; yh Department of Urban Planning and Design, University of Hong Kong, Hong Kong, China; yi Group Health Department, Nanyang Central Hospital, Nanyang, China; yj Department of Nephrology, Max Super Speciality Hospital, New Delhi, India; yk Non-communicable Diseases Division (NCD), Indian Council of Medical Research, New Delhi, India; yl Department of Public Health, Torrens University Australia, Melbourne, VIC, Australia; ym Department of Internal Medicine, Cleveland Clinic, Cleveland, OH, USA; yn Department of Cardiology, Lehigh Valley Health Network, Allentown, PA, USA; yo Department of Epidemiology and Biostatistics, University of South Carolina, Columbia, SC, USA; yp Centre for Noncommunicable Diseases and Nutrition, BRAC University, Dhaka, Bangladesh; yq Department of Preventive Cardiology & Medicine, Eternal Heart Care Centre & Research Institute, Jaipur, India; yr Department of Medicine, Mahatma Gandhi University Medical Sciences, Jaipur, India; ys Department of Toxicology, Shriram Institute for Industrial Research, Delhi, India; yt Global Virus Network, Middle East Region, Shiraz, Iran; yu Department of Clinical Pharmacology and Medicine, University of Kufa, Najaf, Iraq; yv Brachial Plexus and Peripheral Nerve Injury Center, Guilan University of Medical Sciences, Rasht, Iran; yw Pharmaceutical Sciences Research Center, Ardabil University of Medical Science, Ardabil, Iran; yx Department of Immunology, Semnan University of Medical Sciences, Semnan, Iran; yy Cancer Research Center, Semnan University of Medical Sciences, Semnan, Iran; yz Department of Radiology, Massachusetts General Hospital, Boston, MA, USA; za Obesity Research Center, Shahid Beheshti University of Medical Sciences, Tehran, Iran; zb Research Center for Social Determinants of Health, Shahid Beheshti University of Medical Sciences, Tehran, Iran; zc Biochemistry Department, Ain Shams University, Cairo, Egypt; zd School of Health and Environmental Studies, Hamdan Bin Mohammed Smart University, Dubai, United Arab Emirates; ze Institute for Health Metrics and Evaluation, University of Washington, Seattle, WA, USA; zf University Institute of Public Health, The University of Lahore, Lahore, Pakistan; zg Department of Critical Care and Emergency Nursing, Zanjan University of Medical Sciences, Zanjan, Iran; zh Centre for Neuromuscular and Neurological Disorders, The University of Western Australia, Perth, WA, Australia; zi Stroke Research Centre, Perron Institute for Neurological and Translational Science, Perth, WA, Australia; zj Department of Biochemistry and Molecular Biology, Bangladesh Agricultural University, Mymensingh, Bangladesh; zk Department of Anatomy, Dongguk University, Gyeongju, South Korea; zl The Warren Alpert Medical School, Brown University, Providence, RI, USA; zm Department of Epidemiology Population Biostatistics and Health Promotion, Universitas Airlangga (Airlangga University), Surabaya, Indonesia; zn Faculty of Medicine, Utrecht University, Utrecht, Netherlands; zo Department of Radiology, University Medical Center Utrecht, Utrecht, Netherlands; zp Research Unit, Parc Sanitari Sant Joan de Deu, Barcelona, Spain; zq Mental Health, Biomedical Research Networking Center for Mental Health Network (CiberSAM), Madrid, Spain; zr Department of Advanced Nursing, Universitas Airlangga (Airlangga University), Surabaya, Indonesia; zs School of Nursing and Midwivery, La Trobe University, Bundoora, VIC, Australia; zt Department of Zoology and Entomology, Al-Azhar University, Cairo, Egypt; zu Department of Pharmaceutical Technology, University of Dhaka, Dhaka, Bangladesh; zv Department of Pharmacy, Palamau Institute of Pharmacy, Daltonganj, India; zw Public Health Department, Dalhatu Araf Specialist Hospital, Lafia, Nigeria; zx Department of Public Health, Federal University of Lafia, Lafia, Nigeria; zy Sina Trauma and Surgery Research Center, Tehran University of Medical Sciences, Tehran, Iran; zz Department of Diagnostic and Interventional Radiology and Neuroradiology, University Hospital Essen, Essen, Germany; aaa Institute of Artificial Intelligence in Medicine, University Hospital Essen, Essen, Germany; aab Skaane University Hospital, Skaane County Council, Malmö, Sweden; aac Institute for Health Metrics and Evaluation, University of Washington, Seattle, WA, USA; aad Department of Health Metrics Sciences, School of Medicine, University of Washington, Seattle, WA, USA; aae School of Life Course & Population Sciences, King’s College London, London, UK; aaf Faculty of Kinesiology, University of New Brunswick, Fredericton, NB, Canada; aag School of Allied Health, Murdoch University, Murdoch, WA, Australia; aah Department of Clinical Sciences, Ajman University, Ajman, United Arab Emirates; aai Community-Oriented Nursing Midwifery Research Center, Shahrekord University of Medical Sciences, Shahrekord, Iran; aaj Department of Medicine, MedStar Health, Washington, DC, USA; aak Department of Medicine, Georgetown University, Washington DC, DC, USA; aal Department of Public Health, Madda Walabu University, Robe, Ethiopia; aam Department of Microbiology, Taiz University, Taiz, Yemen; aan School of Medicine, Nankai University, Tianjin, China; aao Division for Health Service Promotion, University of Tokyo, Tokyo, Japan; aap School of Dentistry, Hanoi Medical University, Hanoi, Vietnam; aaq Kasturba Medical College, Mangalore, Manipal Academy of Higher Education, Manipal, India; aar Department of Pulmonology, Yokohama City University, Yokohama, Japan; aas National Human Genome Research Institute (NHGRI), National Institutes of Health, Bethesda, MD, USA; aat Department of Decision and Information Sciences, University of Houston, Houston, TX, USA; aau Public Health Research Group, Nature Study Society of Bangladesh, Khulna, Bangladesh; aav School of Health and Society, University of Wollongong, Wollongong, NSW, Australia; aaw Institute of Research and Development, Duy Tan University, Da Nang, Vietnam; aax Department of Computer Science, University of Human Development, Sulaymaniyah, Iraq; aay Department of Virology, Tehran University of Medical Sciences, Tehran, Iran; aaz Department of Virology, Tabriz University of Medical Sciences, Tabriz, Iran; bba Department of Internal Medicine, Carol Davila University of Medicine and Pharmacy, Bucharest, Romania; bbb Department of Legal Medicine and Bioethics, Carol Davila University of Medicine and Pharmacy, Bucharest, Romania; bbc Department of Clinical Legal Medicine, National Institute of Legal Medicine Mina Minovici, Bucharest, Romania; bbd Department of Cardiology, The Fifth Clinical Medical School of Anhui Medical University, Hefei, China; bbe Department of Cardiology, The Second People's Hospital of Hefei, Hefei, China; bbf Department of Psychology, Tsinghua University, Beijing, China; bbg Faculty of Medicine, The Chinese University of Hong Kong, Hong Kong, China; bbh Department of Public Health and Community Medicine, Shaikh Zayed Postgraduate Medical Institute, Lahore, Pakistan; bbi Czech National Centre for Evidence-Based Healthcare and Knowledge Translation, Masaryk University, Brno, Czech Republic; bbj Institute of Biostatistics and Analyses, Masaryk University, Brno, Czech Republic; bbk College of Health Sciences, VinUniversity, Hanoi, Vietnam; bbl School of Health Care Administration, Taipei Medical University, Taipei, Taiwan; bbm International Master Program for Translational Science, Taipei Medical University, Taipei, Taiwan; bbn Department of Occupational Safety and Health, China Medical University Taiwan, Taichung, Taiwan; bbo Department of Occupational Therapy, Asia University, Taiwan, Taichung, Taiwan; bbp Department of Health Promotion and Education, University of Ibadan, Ibadan, Nigeria; bbq International Center for Nutrition and Information, National Institutes of Biomedical Innovation, Health and Nutrition, Tokyo, Japan; bbr The National Centre for Remote and Rural Health and Care, NHS National Services Scotland, Edinburgh, Scotland; bbs West Africa RCC, Africa Centre for Disease Control and Prevention, Abuja, Nigeria; bbt Department of Community Medicine, University College Hospital, Ibadan, Ibadan, Nigeria; bbu Faculty of Medicine, University of Belgrade, Belgrade, Serbia; bbv Faculty of Medical Sciences, University of Kragujevac, Kragujevac, Serbia; bbw Department of Clinical Pharmacy, Prince Sattam bin Abdulaziz University, Al Kharj, Saudi Arabia; bbx Institute of Health Research, University of Health and Allied Sciences, Ho, Ghana; bby Department of Health Research, ICMR National Institute for Research in Tuberculosis, Chennai, India; bbz Faculty of Public Health, Universitas Muhammadiyah Aceh, Banda Aceh, Indonesia; cca Department of Global Health and Population, Harvard University, Boston, MA, USA; ccb Department of Research and Innovation, ARNECH Research and Consulting Office, Bujumbura, Burundi; ccc Faculty of Pharmacy, Universitas Ahmad Dahlan, Yogyakarta, Indonesia; ccd School of Pharmacy, BRAC University, Dhaka, Bangladesh; cce Clinical Laboratory Department, Tobruk University, Tobruk, Libya; ccf Department of Blood Transmitted Diseases, National Centre for Disease Control (NCDC), Tobruk, Libya; ccg Department of Clinical Pharmacy & Pharmacy Practice, Asian Institute of Medicine, Science and Technology, Bedong, Malaysia; cch Malaysian Academy of Pharmacy, Puchong, Malaysia; cci Public Health Department of Social Medicine, Osaka University, Suita, Japan; ccj Department of General Surgery and Medical-Surgical Specialties, University of Catania, Catania, Italy; cck School of Management, The Apollo University, Chittoor, India; ccl Department of Health Services Research, University of Tsukuba, Tsukuba, Japan; ccm Department of Non-Communicable Disease Epidemiology, London School of Hygiene & Tropical Medicine, London, UK; ccn School of Health Systems and Public Health, University of Washington, Seattle, WA, USA; cco Department of Community Medicine and Family Medicine, All India Institute of Medical Sciences, Gorakhpur, India; ccp Department of Environmental Health Engineering, Guilan University of Medical Sciences, Rasht, Iran; ccq Department of Physical Medicine, Université Paris Cité, Paris, France; ccr Research and Development Unit, Biomedical Research Networking Center for Mental Health Network (CiberSAM), Barcelona, Spain; ccs Department of Immunology, Kerman University of Medical Sciences, Kerman, Iran; cct Department of Immunology, Rafsanjan University of Medical Sciences, Rafsanjan, Iran; ccu College of Medicine and Medical Sciences, Arabian Gulf University, Manama, Bahrain; ccv Ministry of Health, Manama, Bahrain; ccw Department of Leukemia, The University of MD Anderson Cancer Center, Houston, TX, USA; ccx Statistics Unit, Riga Stradins University, Riga, Latvia; ccy Department of Health and Safety, Dubai Municipality, Dubai, United Arab Emirates; ccz Centre for Community Medicine, All India Institute of Medical Sciences, New Delhi, India; dda The World Academy of Sciences UNESCO, Trieste, Italy; ddb Shaanxi University of Technology, Hanzhong, China; ddc Department of Environmental Engineering, Islamic Azad University, Ahvaz, Iran; ddd Faculty of Medicine and Health Sciences, SRM Institute of Science and Technology (SRMIST), Chennai, India; dde School of Pharmacy and Pharmacology, University of Tasmania, Hobart, TAS, Australia; ddf Centre of Studies and Research, Ministry of Health, Muscat, Oman; ddg Department of Biochemistry, Government Medical College, Mysuru, India; ddh Department of Epidemiology and Health Promotion, Yonsei University, Seoul, South Korea; ddi Department of General Medicine, Manipal Academy of Higher Education, Mangalore, India; ddj Department of Biomedical Sciences, Debre Tabor University, Debre Tabor, Ethiopia; ddk Department of Public Health, Madda Walabu University, Goba, Ethiopia; ddl Department of Internal Medicine, GCS Medical College, Hospital & Research Centre, Ahmedabad, India; ddm Department of Nutrition, University of Nevada Reno, Reno, NV, USA; ddn University of Johannesburg, Johannesburg, South Africa; ddo Department of Cardiovascular Medicine, Saint Vincent Hospital, Worcester, MA, USA; ddp Department of Community Medicine, Dr. Baba Saheb Ambedkar Medical College & Hospital, Delhi, India; ddq Department of Community Medicine, Banaras Hindu University, Varanasi, India; ddr Department of Public Health, Tongji University, Shanghai, China; dds Department of Public Health, La Trobe University, Melbourne, VIC, Australia; ddt Melbourne School of Population and Global Health, University of Melbourne, Melbourne, VIC, Australia; ddu School of Public Health, Sichuan University, Chengdu, China; ddv Department of Medicine & Therapeutics, The Chinese University of Hong Kong, Hong Kong, China; ddw Zoonoses Research Center, Islamic Azad University, Karaj, Iran; ddx Rothschild Foundation Hospital, Institute of Molecular and Clinical Ophthalmology Basel, Paris, France; ddy Singapore Eye Research Institute, Singapore, Singapore; ddz Health Services Management Training Centre, Semmelweis University, Budapest, Hungary; eea Hungarian Health Management Association, Budapest, Hungary; eeb Department of Community Medicine, Manipal Academy of Higher Education, Mangalore, India; eec Department of Community Medicine, Manipal Academy of Higher Education, Mangalore, India; eed Department of Economics, National Open University, Benin City, Nigeria; eee Gastrointestinal and Liver Diseases Research Center, Guilan University of Medical Sciences, Rasht, Iran; eef Caspian Digestive Disease Research Center, Guilan University of Medical Sciences, Rasht, Iran; eeg Department of Family Medicine and Public Health, University of Opole, Opole, Poland; eeh Institute of Family Medicine and Public Health, University of Tartu, Tartu, Estonia; eei Minimally Invasive Surgery Research Center, Iran University of Medical Sciences, Tehran, Iran; eej Department of Social Work, Pabna University of Science and Technology, Pabna, Bangladesh; eek School of Public Health, University College Cork, Cork, Ireland; eel Department of Oral and Maxillofacial Pathology, Krishna Vishwa Vidyapeeth (Deemed to be University), Karad, India; eem Department of Neurology, University of Washington, Seattle, WA, USA; een Department of Endocrinology, Bharti Hospital Karnal, Karnal, India; eeo University Centre for Research and Development, Chandigarh University, Mohali, India; eep Division of Epidemiology and Biostatistics, National Institute of Epidemiology, Chennai, India; eeq Department of Biostatistics, Indian Council of Medical Research, New Delhi, India; eer Russell H. Morgan Department of Radiology and Radiological Science, Johns Hopkins University, Baltimore, MD, USA; ees School of Public Health, Fudan University, Shanghai, China; eet Health Sciences, University of York, York, UK; eeu Save Sight Institute, University of Sydney, Sydney, NSW, Australia; eev Sydney Eye Hospital, South Eastern Sydney Local Health District, Sydney, NSW, Australia; eew Faculty of Dentistry, University of Puthisastra, Phnom Penh, Cambodia; eex Office of the Executive Director, Cephas Health Research Initiative Inc, Ibadan, Nigeria; eey The Hansjörg Wyss Department of Plastic and Reconstructive Surgery, Nab’a Al-Hayat Foundation for Medical Sciences and Health Care, New York, NY, USA; eez Cleft Lip and Palate Surgery Division, Global Smile Foundation, Norwood, MA, USA; ffa Department of Forensic Science, Government Institute of Forensic Science Nagpur, Nagpur, India; ffb 2nd Cardiology Department, Aristotle University of Thessaloniki, Thessaloniki, Greece; ffc School of Health Professions and Human Services, Hofstra University, Hempstead, NY, USA; ffd Department of Anesthesiology, Montefiore Medical Center, Bronx, NY, USA; ffe Institute for Epidemiology and Social Medicine, University of Münster, Münster, Germany; fff School of Medicine, Tehran University of Medical Sciences, Tehran, Iran; ffg Social Determinants of Health Research Center, Tabriz University of Medical Sciences, Tabriz, Iran; ffh Cardiovascular Diseases Research Institute, Tehran University of Medical Sciences, Tehran, Iran; ffi Endocrine Research Center, Iran University of Medical Sciences, Tehran, Iran; ffj Department of Echocardiography, Iran University of Medical Sciences, Tehran, Iran; ffk Central Department of Public Health, Institute of Medicine, Kathmandu, Nepal; ffl Eye Research Center, Iran University of Medical Sciences, Tehran, Iran; ffm Health Policy Research Center, Shiraz University of Medical Sciences, Shiraz, Iran; ffn Surgery Research Unit, University of Oulu, Oulu, Finland; ffo Department of Molecular Medicine and Surgery, Karolinska Institute, Stockholm, Sweden; ffp Public Health Foundation of India, New Delhi, India; ffq Medical Basic Sciences Research Institute, Ahvaz Jundishapur University of Medical Sciences, Ahvaz, Iran; ffr Student Research Committee, Ahvaz Jundishapur University of Medical Sciences, Ahvaz, Iran; ffs International Research Center of Excellence, Institute of Human Virology Nigeria, Abuja, Nigeria; fft Julius Centre for Health Sciences and Primary Care, Utrecht University, Utrecht, Netherlands; ffu Department of Neurosurgery, Johns Hopkins University, Baltimore, MD, USA; ffv Cardiac Primary Prevention Research Center, Tehran University of Medical Sciences, Tehran, Iran; ffw Department of Cardiac Electrophysiology, Tehran University of Medical Sciences, Tehran, Iran; ffx Department of Human Nutrition, National Research Institute for Agriculture, Food and Environment, Jouy-en-Josas, France; ffy Sorbonne Paris Nord University, Bobigny, France; ffz Department of Public Health, Jordan University of Science and Technology, Irbid, Jordan; gga Department of Public Health, Ahvaz Jundishapur University of Medical Sciences, Ahvaz, Iran; ggb Environmental Technologies Research Center, Medical Basic Sciences Research Institute, Ahvaz Jundishapur University of Medical Sciences, Ahvaz, Iran; ggc Department of Public Health and Health Policy, Hiroshima University, Hiroshima, Japan; ggd Amity Institute of Forensic Sciences, Amity University, Noida, India; gge School of Medicine, Tehran University of Medical Sciences, Tehran, Iran; ggf Endocrinology and Metabolism Research Institute, Non-Communicable Diseases Research Center (NCDRC), Tehran, Iran; ggg College of Health Sciences, Abu Dhabi University, Adu Dhabi, United Arab Emirates; ggh University Institute of Diet and Nutritional Sciences, The University of Lahore, Lahore, Pakistan; ggi School of Health and Rehabilitation Sciences, The University of Queensland, Brisbane, QLD, Australia; ggj Department of Physical Therapy, King Abdulaziz University, Jeddah, Saudi Arabia; ggk Population Science Department, Jatiya Kabi Kazi Nazrul Islam University, Mymensingh, Bangladesh; ggl Department of Public Health, University of Sydney, Sydney, NSW, Australia; ggm Department of Epidemiology, Jazan University, Jazan, Saudi Arabia; ggn Department of Rehabilitation Sciences, Hong Kong Polytechnic University, Hong Kong, China; ggo Family Medicine Department, United Arab Emirates University, Al Ain, United Arab Emirates; ggp Primary Care Department, NHS North West London, London, UK; ggq Department of Clinical Pharmacy, Jouf University, Sakaka, Saudi Arabia; ggr Department of Epidemiology, Non-Communicable Diseases Research Center (NCDRC), Tehran, Iran; ggs School of Medicine, Tehran University of Medical Sciences, Tehran, Iran; ggt College of Health, Wellbeing and Life Sciences, Sheffield Hallam University, Sheffield, UK; ggu College of Arts and Sciences, Ohio University, Zanesville, OH, USA; ggv Faculty of Nursing, Jerash University, Jerash, Jordan; ggw Department of Basic Medical Sciences, Yarmouk University, Irbid, Jordan; ggx School of Medicine, Kurdistan University of Medical Sciences, Sanandaj, Iran; ggy Global Consortium for Public Health Research, Datta Meghe Institute of Higher Education and Research, Wardha, India; ggz Shahid Beheshti University of Medical Sciences, Tehran, Iran; hha Academy of Medical Science, Tehran, Iran; hhb Department of Neurosurgery, Shahid Beheshti University of Medical Sciences, Tehran, Iran; hhc Department of Biochemistry, Liaquat University Of Medical and Health Sciences, Jamshoro, Pakistan; hhd Department of Biochemistry, All India Institute of Medical Sciences, Jodhpur, India; hhe Department of Internal Medicine, Corewell Health East William Beaumont University Hospital, Royal Oak, MI, USA; hhf Department of Medical Oncology, Miami Cancer Institute, Miami, FL, USA; hhg Department of Health Management and Economics, Qom University of Medical Sciences, Qom, Iran; hhh Department of Health Economics, Iran University of Medical Sciences, Tehran, Iran; hhi Department of Public Health, New Mexico State University, Las Cruces, NM, USA; hhj Department of Cognition and Neuroscience, University of Texas, Dallas, TX, USA; hhk Graduate School of Public Health, Yonsei University, Busan, South Korea; hhl Broad Institute of MIT and Harvard, Cambridge, MA, USA; hhm Massachusetts General Hospital, Boston, MA, USA; hhn School of Traditional Chinese Medicine, Xiamen University Malaysia, Sepang, Malaysia; hho Millennium Prevention, Inc., Westwood, MA, USA; hhp School of Health Sciences, Kristiania University College, Oslo, Norway; hhq Department of International Health and Sustainable Development, Tulane University, New Orleans, LA, USA; hhr Department of Nursing and Health Promotion, Oslo Metropolitan University, Oslo, Norway; hhs Social Determinants of Health Research Center, Shahid Beheshti University of Medical Sciences, Tehran, Iran; hht Health System and Population Studies Divisions, International Centre for Diarrhoeal Disease Research, Bangladesh, Dhaka, Bangladesh; hhu Center for Global Mental Health, London School of Hygiene & Tropical Medicine, London, UK; hhv Children's Medical Center, Tehran University of Medical Sciences, Tehran, Iran; hhw Department of Public Health Dentistry, Krishna Vishwa Vidyapeeth (Deemed to be University), Karad, India; hhx Department of Neurosurgery, Helsinki University Hospital, Helsinki, Finland; hhy Department of Epidemiology and Evidence-Based Medicine, I.M. Sechenov First Moscow State Medical University, Moscow, Russia; hhz Department of General Practice and Family Medicine, Kharkiv National Medical University, Kharkiv, Ukraine; iia Independent Consultant, Jakarta, Indonesia; iib Department of Epidemiology, IQVIA, Frankfurt am Main, Germany; iic Department of Gynecology, Philipps-Universität Marburg, Marburg, Germany; iid Department of Anaesthesiology and Critical Care, All India Institute of Medical Sciences, Jodhpur, India; iie Department of Biochemistry, All India Institute of Medical Sciences, Bhopal, India; iif Kasturba Medical College, Manipal, Manipal Academy of Higher Education, Udupi, India; iig Department of Anthropology, Panjab University, Chandigarh, India; iih Department of Forensic Medicine and Toxicology, Pondicherry University, Puducherry, India; iii Department of Anesthesiology, Duke University, Durham, NC, USA; iij Department of Anesthesiology & Pain Medicine, University of Washington, Seattle, WA, USA; iik Department of Physiology, Pravara Institute of Medical Sciences (Deemed to be University), Loni, India; iil Department of Neuroscience, University of Medicine, Tirana, Albania; iim Department of Neuroscience, Medical Sciences University Hospital, Tirana, Albania; iin Department of Demography, University of Montreal, Montreal, QC, Canada; iio Department of Social and Preventive Medicine, University of Montreal, Montreal, QC, Canada; iip Faculty of Medicine, Gazi University, Ankara, Turkiye; iiq Department of Mathematics, University of Rajshahi, Rajshahi, Bangladesh; iir Department of Biochemistry, University of Hail, Hail, Saudi Arabia; iis University of Environment and Sustainable Development, Somanya, Ghana; iit Atchabarov Scientific-Research Institute of Fundamental and Applied Medicine, Kazakh National Medical University, Almaty, Kazakhstan; iiu Center of Medicine and Public Health, Asfendiyarov Kazakh National Medical University, Almaty, Kazakhstan; iiv Department of Medicine, Queensland Health, Brisbane, QLD, Australia; iiw Cardiothoracic Surgery Department, NYU Langone Health, New York City, NY, USA; iix Cardiothoracic Surgery Department, Medanta Hospital, Gurugram, India; iiy Department of Cardiovascular Medicine, Cabrini Institute, Rochester, MN, USA; iiz Department of Community Medicine, Rajendra Institute of Medical Sciences, Ranchi, India; jja Public Health Foundation of India, Gurugram, India; jjb Department of Community Medicine, Manipal Academy of Higher Education, Mangalore, India; jjc College of Public Health & Health Informatics, University of Hail, Hail, Saudi Arabia; jjd Department of Physiotherapy, Manipal Academy of Higher Education, Manipal, India; jje Division of Cardiovascular Medicine, University of Kentucky, Lexington, KY, USA; jjf School of Medicine and Dentistry, Griffith University, Gold Coast, QLD, Australia; jjg Department of Nutrition and Food Science, Patuakhali Science and Technology University, Patuakhali, Bangladesh; jjh Section of Cardiology, University of Manitoba, Winnipeg, MB, Canada; jji Department of Translational Health Sciences, University of Bristol, Bristol, UK; jjj Faculty of Health and Life Sciences, Coventry University, Coventry, UK; jjk Department of Medicine, McMaster University, Hamilton, ON, Canada; jjl Department of Health Services Research and Management, City University of London, London, UK; jjm Faculty of Public Health, University of Indonesia, Depok, Indonesia; jjn Department of General Surgery, Dr NTR University of Health Sciences, Vijayawada, India; jjo Department of Nephrology, Pushpagiri Institute of Medical Sciences and Research Centre, Thiruvalla, India; jjp Clinical Research Center, Turku University Hospital, Turku, Finland; jjq Heart Center, University of Turku, Turku, Finland; jjr Department of Clinical Sciences and Community Health, University of Milan, Milan, Italy; jjs Nuffield Department of Population Health, University of Oxford, Oxford, UK; jjt National Institute for Health Research (NIHR) Oxford Biomedical Research Centre, Oxford, UK; jju Integrated Department of Epidemiology, Health Policy, Preventive Medicine and Pediatrics, Foundation for People-centric Health Systems, New Delhi, India; jjv SD Gupta School of Public Health, Indian Institute of Health Management Research University, Jaipur, India; jjw Indian Council of Medical Research, New Delhi, India; jjx Department of Public Health, University of Helsinki, Helsinki, Finland; jjy Health Services Management Training Centre, Semmelweis University, Budapest, Hungary; jjz NEVES Society for Patient Safety, Budapest, Hungary; kka Unidad de Genética y Salud Pública, Instituto de Ciencias Médicas, Las Tablas, Panama; kkb Ministry of Health, Hospital Joaquín Pablo Franco Sayas, Las Tablas, Panama; kkc Department of Medical Sciences, Uppsala University, Uppsala, Sweden; kkd Department of Clinical Chemistry and Pharmacology, Uppsala University Hospital, Uppsala, Sweden; kke Department of Otorhinolaryngology, Father Muller Medical College, Mangalore, India; kkf Department of Anesthesiology, Iran University of Medical Sciences, Tehran, Iran; kkg International Society Doctors for the Environment, Arezzo, Italy; kkh Department of Neurology and Psychiatry, German Clinic of Santiago, Santiago, Chile; kki Faculty of Medicine, University of Development, Santiago, Chile; kkj Clinical Pharmacy and Pharmacy Management, Kaduna State University, Kaduna, Nigeria; kkk Health Economics Division, Monash University, Burwood, VIC, Australia; kkl Faculty of Medicine, University of Medicine and Pharmacy at Ho Chi Minh City, Ho Chi Minh City, Vietnam; kkm Department of Cardiovascular Research, Methodist Hospital, Merrillville, IN, USA; kkn University of Medicine and Pharmacy at Ho Chi Minh City, Ho Chi Minh City, Vietnam; kko University of Medicine and Pharmacy at Ho Chi Minh City, Ho Chi Minh City, Vietnam; kkp Independent Consultant, Ho Chi Minh City, Vietnam; kkq Southampton Clinical Trials Unit, University of Southampton, Southampton, UK; kkr Department of Precision Medicine, Sungkyunkwan University, Suwon-si, South Korea; kks Department of Family Medicine, University of Texas Medical Branch, Galveston, TX, USA; kkt Department of Preventive Medicine, Korea University, Seoul, South Korea; kku Department of Health Promotion and Health Education, National Taiwan Normal University, Taipei, Taiwan; kkv Department of Psychiatry, Yale University, New Haven, CT, USA; kkw Department of Health Management Center, Fudan University, Shanghai, China; kkx National Clinical Research Center for Cardiovascular Diseases, Chinese Academy of Medical Sciences, Shenzhen, China; kky Department of Medicine, University of Malaya, Kuala Lumpur, Malaysia; kkz Department of Medicine and Therapeutics, The Chinese University of Hong Kong, Hong Kong, China; lla Institute for Health Metrics and Evaluation, University of Washington, Seattle, WA, USA; llb Department of Health Metrics Sciences, School of Medicine, University of Washington, Seattle, WA, USA; llc Department of Medicine, University of Pennsylvania, Philadelphia, PA, USA; lld Department of Medical Sciences, Uppsala University, Uppsala, Sweden; lle Department of Medicine, Norrtälje Hospital (Tiohundra), Norrtälje, Sweden; llf School of Public Health, Zefat Academic College, Haifa, Israel; llg School of Life Sciences, University of Technology Sydney, Sydney, NSW, Australia; llh Center for Evidence-Based Medicine and Clinical Research, Hubei University of Medicine, Shiyan, China; lli Department of Cardiology, Guiqian International General Hospital, Guiyang, China; llj Department of Radiology and Biomedical Imaging, Yale University, New Haven, CT, USA; llk Department of Radiology, Massachusetts General Hospital, Boston, MA, USA; lll Lerner Research Institute, Cleveland Clinic, Cleveland, OH, USA; llm Department of Quantitative Health Science, Case Western Reserve University, Cleveland, OH, USA; lln Department of Molecular Epidemiology, German Institute of Human Nutrition Potsdam-Rehbrücke, Potsdam, Germany; llo German Center for Diabetes Research (DZD), München-Neuherberg, Germany; llp Department of Internal Medicine, Kirk Kerkorian School of Medicine at UNLV, Las Vegas, NV, USA; llq Department of Pediatrics, Ohio State University, Columbus, OH, USA; llr Department of Pediatric Neurology, Nationwide Children's Hospital, Columbus, OH, USA; lls Department of Cardiology, University of Cologne, Cologne, Germany; llt One Health Research Group, Universidad de Las Américas, Quito, Ecuador; llu Department of Health Economics, Syreon Research Romania, Targu Mures, Romania; llv Department of Doctoral Studies, George Emil Palade University of Medicine, Pharmacy, Science, and Technology of Targu Mures, Targu Mures, Romania; llw Institute of Nutritional Sciences, Friedrich Schiller University Jena, Jena, Germany; llx Competence Cluster for Nutrition and Cardiovascular Health (nutriCARD), Jena, Germany; lly Department of Medicine, University of São Paulo, São Paulo, Brazil; llz School of Medicine, Federal University of Juiz de Fora, Juiz de Fora, Brazil; mma Center for Evidence-Based and Translational Medicine, Wuhan University, Wuhan, China; mmb Department of Population Health Sciences, Duke University, Durham, NC, USA; mmc Centre for Public Health and Wellbeing, University of the West of England, Bristol, UK; mmd Department of Periodontology, Pomeranian Medical University, Szczecin, Poland; mme Department of Biostatistics and Epidemiology, Yazd University of Medical Sciences, Yazd, Iran; mmf Institute for Physical Activity and Nutrition, Deakin University, Melbourne, VIC, Australia; mmg Department of Cardiology, Tehran University of Medical Sciences, Tehran, Iran; mmh USERN Office, Lorestan University of Medical Sciences, Khorramabad, Iran; mmi Department of Medicine, Medical College of Georgia at Augusta University, Augusta, GA, USA; mmj Department of Cardiology, October 6 University, 6th of October City, Egypt; mmk Department of Pediatric Cardiology, Tehran University of Medical Sciences, Tehran, Iran; mml Rama Medical College Hospital and Research Centre, Uttar Pradesh, India; mmm Institute of Applied Health Research, University of Birmingham, Birmingham, UK; mmn Rabigh Faculty of Medicine, King Abdulaziz University, Jeddah, Saudi Arabia; mmo Department of Electrical Engineering, Prince Sattam bin Abdulaziz University, Al Kharj, Saudi Arabia; mmp Department of Clinical Pharmacy, Jouf University, Sakaka, Saudi Arabia; mmq Department of Maternal-Child Nursing and Public Health, Federal University of Minas Gerais, Belo Horizonte, Brazil; mmr Poche Centre for Indigenous Health, The University of Queensland, Brisbane, QLD, Australia; mms Smidt Heart Institute, Cedars-Sinai Medical Center, Los Angeles, CA, USA; mmt Cardiology Department, Isfahan University of Medical Sciences, Isfahan, Iran; mmu Department of Cardiology, Tehran University of Medical Sciences, Tehran, Iran; mmv Digestive Diseases Research Institute, Tehran University of Medical Sciences, Tehran, Iran; mmw Department of Epidemiology and Biostatistics, Tehran University of Medical Sciences, Tehran, Iran; mmx School of Medicine and Surgery, University of Milan Bicocca, Monza, Italy; mmy Laboratory of Public Health, Instituto Auxologico Italiano IRCCS (Italian Auxological Institute), Milan, Italy; mmz Department of Population and Behavioural Sciences, University of Health and Allied Sciences, Ho, Ghana; nna Biomedical Engineering Research Center (CREB), Universitat Politècnica de Catalunya (Barcelona Tech - UPC), Barcelona, Spain; nnb Department of Biomedical Engineering, University of Isfahan, Isfahan, Iran; nnc Department of Biochemistry, Golestan University of Medical Sciences, Gorgan, Iran; nnd Department of Food, Environmental and Nutritional Sciences, University of Milan, Milan, Italy; nne Faculty of Public Health, Universitas Airlangga (University of Airlangga), Surabaya, Indonesia; nnf Indonesian Public Health Association, Surabaya, Indonesia; nng Department of Nutrition and Dietetics, University of Concepción, Concepción, Chile; nnh Centre for Healthy Living, University of Concepción, Concepción, Chile; nni Department of Pharmacy, Bahauddin Zakariya University, Multan, Pakistan; nnj Faculty of Humanities and Health Sciences, Curtin University, Sarawak, Malaysia; nnk Jeffrey Cheah School of Medicine and Health Sciences, Monash University, Subang Jaya, Malaysia; nnl School of Public Health, Haramaya University, Harar, Ethiopia; nnm Department of Anatomy and Developmental Biology, Monash University, Clayton, VIC, Australia; nnn Department of Anatomy, Genetics and Biomedical Informatics, University of Colombo, Colombo, Sri Lanka; nno Research Division, The George Institute for Global Health, New Delhi, India; nnp School of Medicine, University of New South Wales, Sydney, NSW, Australia; nnq School of Medicine, Tehran University of Medical Sciences, Tehran, Iran; nnr Department of Twin Research and Genetic Epidemiology, King's College London, London, UK; nns Department of Health Services Research and Policy, London School of Hygiene & Tropical Medicine, London, UK; nnt Australian Centre for Health Services Innovation, Queensland University of Technology, Kelvin Grove, QLD, Australia; nnu Digital Health and Informatics Directorate, Queensland Health, Brisbane, QLD, Australia; nnv Department of Healthcare, University of Vlora, Vlora City, Albania; nnw Clinic of Social and Family Medicine, University of Crete, Heraklion, Greece; nnx Department of Health Informatics, Jazan University, Jazan, Saudi Arabia; nny Department of Epidemiology and Biostatistics, Isfahan University of Medical Sciences, Isfahan, Iran; nnz Department of Public Health, Arba Minch University, Arba Minch, Ethiopia; ooa Department of Medical Laboratory Sciences, Adigrat University, Adigrat, Ethiopia; oob Universidad Nacional Mayor de San Marcos, Lima, Peru; ooc Division of Forensic Medicine, Imam Abdulrahman Bin Faisal University, Dammam, Saudi Arabia; ood Center for Translation Research and Implementation Science, National Institutes of Health, Bethesda, MD, USA; ooe Department of Medicine, University of Cape Town, Cape Town, South Africa; oof Department of Physiology, King Saud University, Riyadh, Saudi Arabia; oog General Administration, Helsinki University Hospital, Helsinki, Finland; ooh School of Health Sciences, University of Melbourne, Melbourne, VIC, Australia; ooi Comprehensive Cancer Center, Helsinki University Hospital, Helsinki, Finland; ooj University of Helsinki, Helsinki, Finland; ook University Centre Varazdin, University North, Varazdin, Croatia; ool Institute for Health Metrics and Evaluation, University of Washington, Seattle, WA, USA; oom Department of Pharmacology, University of Kelaniya, Ragama, Sri Lanka; oon Clinical Medicine Department, Colombo North Teaching Hospital, Ragama, Sri Lanka; ooo Department of Propedeutics of Internal Diseases & Arterial Hypertension, Pomeranian Medical University, Szczecin, Poland; oop National Cancer Registry, Maria Sklodowska-Curie National Research Institute of Oncology, Warsaw, Poland; ooq Pathology Department, Maria Sklodowska-Curie National Research Institute of Oncology, Warsaw, Poland; oor Department of Maternal-Child Nursing and Public Health, Federal University of Minas Gerais, Belo Horizonte, Brazil; oos Multidisciplinary Department of Medical-Surgical and Dental Specialties, Univerity of Campania "Luigi Vanvitelli", Naples, Italy; oot Saveetha Dental College and Hospitals, Saveetha University, Chennai, India; oou International Ph.D. Program in Medicine, Taipei Medical University, Taipei, Taiwan; oov Research Center for Artificial Intelligence in Medicine, Taipei Medical University, Taipei, Taiwan; oow Department of Public Health Dentistry, Saveetha Institute of Medical and Technical Sciences (SIMATS), Chennai, India; oox Global Institute of Public Health, Ananthapuri Hospitals and Research Institute, Trivandrum, India; ooy Faculty of Nursing and Midwifery, Tabriz University of Medical Sciences, Tabriz, Iran; ooz Department of Statistics and Econometrics, Bucharest University of Economic Studies, Bucharest, Romania; ppa Internal Medicine Programme, Kyrgyz State Medical Academy, Bishkek, Kyrgyzstan; ppb Department of Atherosclerosis and Coronary Heart Disease, National Center of Cardiology and Internal Disease, Bishkek, Kyrgyzstan; ppc Department of Radiology, Tabriz University of Medical Sciences, Tabriz, Iran; ppd Social Determinants of Health Center, Urmia University of Medical Sciences, Urmia, Iran; ppe Department of Biomedical Sciences, Mercer University School of Medicine, Macon, GA, USA; ppf Department of Surgical Oncology, All India Institute of Medical Sciences, Jodhpur, India; ppg Department of Community Medicine, Manipal Academy of Higher Education, Mangalore, India; pph College of Health Science, University of Hargeisa, Hargeisa, Somalia; ppi Institute of Health Science, Jimma University, Jimma, Ethiopia; ppj College of Applied and Natural Science, University of Hargeisa, Hargeisa, Somalia; ppk Molecular Biology Unit, Sirius Training and Research Centre, Khartoum, Sudan; ppl Bio-Statistical and Molecular Biology Department, Sirius Training and Research Centre, Khartoum, Sudan; ppm College of Medicine, University of Duhok, Duhok, Iraq; ppn Non-communicable Diseases Research Center, Tehran University of Medical Sciences, Tehran, Iran; ppo Faculty of Medicine, Tehran University of Medical Sciences, Tehran, Iran; ppp Natural and Medical Sciences Research Center, University of Nizwa, Nizwa, Oman; ppq Infectious Diseases Research Center, Golestan University of Medical Sciences, Gorgan, Iran; ppr School of Medicine, Tehran University of Medical Sciences, Tehran, Iran; pps Modeling in Health Research Center, Shahrekord University of Medical Sciences, Shahrekord, Iran; ppt Skull Base Research Center, Shahid Beheshti University of Medical Sciences, Tehran, Iran; ppu Department of Public Health, Dire Dawa University, Dire Dawa, Ethiopia; ppv QU Health, Qatar University, Doha, Qatar; ppw Department of Pharmaceutical Sciences, Notre Dame of Maryland University, Baltimore, MD, USA; ppx Department of Pharmacy, Mizan-Tepi University, Mizan, Ethiopia; ppy Health Systems and Policy Research Unit, Ahmadu Bello University, Zaria, Nigeria; ppz Heidelberg Institute of Global Health (HIGH), Heidelberg University, Heidelberg, Germany; qqa Institute for Health Metrics and Evaluation, University of Washington, Seattle, WA, USA; qqb Department of Health Metrics Sciences, School of Medicine, University of Washington, Seattle, WA, USA; qqc Department of Biostatistics, Shiraz University of Medical Sciences, Shiraz, Iran; qqd Faculty of Life Sciences and Medicine, King's College London, London, UK; qqe Department of Mathematics, The University of Jordan, Amman, Jordan; qqf Nonlinear Dynamics Research Center (NDRC), Ajman University, Ajman, United Arab Emirates; qqg Clinical Epidemiology and Public Health Research Unit, Burlo Garofolo Institute for Maternal and Child Health, Trieste, Italy; qqh AI & Cyber Futures Institute, Charles Sturt University, Bathurst, NSW, Australia; qqi The University of Queensland, Brisbane, QLD, Australia; qqj Department of Ophthalmology & Vision Science, University of California Davis, Sacramento, CA, USA; qqk Non-Communicable Diseases Research Center (NCDRC), Tehran, Iran; qql Faculty of Medicine, Birjand University of Medical Sciences, Birjand, Iran; qqm Health Sciences Research Center, Mazandaran University of Medical Sciences, Sari, Iran; qqn Iran University of Medical Sciences, Tehran, Iran; qqo Department of Epidemiology and Biostatistics, Kurdistan University of Medical Sciences, Sanandaj, Iran; qqp Computer, Electrical, and Mathematical Sciences and Engineering Division, King Abdullah University of Science and Technology, Thuwal, Saudi Arabia; qqq International Laboratory for Air Quality and Health, Queensland University of Technology, Brisbane, QLD, Australia; qqr Department of Public Health, Oswaldo Cruz Foundation, Recife, Brazil; qqs Department of Public Health, Federal University of Pernambuco, Recife, Brazil; qqt Division of Plastic and Reconstructive Surgery, University of Washington, Seattle, WA, USA; qqu Neurosciences Research Center (NSRC), Tabriz University of Medical Sciences, Tabriz, Iran; qqv Student Research Committee, Tabriz University of Medical Sciences, Tabriz, Iran; qqw Department of Health Policy, London School of Economics and Political Science, London, UK; qqx Department of Surgery and Cancer, Imperial College London, London, UK; qqy Non-communicable Diseases Research Center, Tehran University of Medical Sciences, Tehran, Iran; qqz Department of Radiology, University of Tripoli, Tripoli, Libya; rra Unit of Pharmacotherapy, Epidemiology and Economics, University of Groningen (Rijksuniversiteit Groningen), Groningen, Netherlands; rrb Department of Epidemiology and Biostatistics, Wuhan University, Wuhan, China; rrc Department of Biomedical and Neuromotor Sciences, University of Bologna, Bologna, Italy; rrd Department of Medicine, Democritus University of Thrace, Alexandroupolis, Greece; rre Department of Health Economics, National Institute for Research in Tuberculosis, Chennai, India; rrf Amity Institute of Pharmacy, Amity University, Noida, India; rrg Clinical Epidemiology Research Unit, Mexican Institute of Social Security, Villa de Alvarez, Mexico; rrh Postgraduate in Medical Sciences, Universidad de Colima, Colima, Mexico; rri Department of Research Methodology, Orthopaedic Research Group, Coimbatore, India; rrj Department of Biotechnology, Karpagam Academy of Higher Education (Deemed to be University), Coimbatore, India; rrk Department of Psychiatry, Seoul National University, Seoul, South Korea; rrl Department of Neuropsychiatry, Seoul National University Bundang Hospital, Seongnam, South Korea; rrm Department of Ophthalmology, Guilan University of Medical Sciences, Rasht, Iran; rrn Department of Obstetrics and Gynecology, Ain Shams University, Cairo, Egypt; rro Department of Knowledge Translation and Utilization, Egyptian Center for Evidence Based Medicine, Cairo, Egypt; rrp Elderly Health Research Center, Research and Academic Institution, Tehran, Iran; rrq Research and Analytics Department, Initiative for Financing Health and Human Development, Chennai, India; rrr Department of Research and Analytics, Bioinsilico Technologies, Chennai, India; rrs Department of Computer Science, University of Illinois Urbana-Champaign, Urbana, IL, USA; rrt College of Medicine and Public Health, Flinders University, Adelaide, SA, Australia; rru Department of Engineering, Western Sydney University, Sydney, NSW, Australia; rrv Department Health Services Research, University of Alabama at Birmingham, Birmingham, AL, USA; rrw Laboratory of Public Health Indicators Analysis and Health Digitalization, Moscow Institute of Physics and Technology, Dolgoprudny, Russia; rrx Experimental Surgery and Oncology Laboratory, Kursk State Medical University, Kursk, Russia; rry Department of Pulmonary Medicine, Government Medical College Thrissur, Thrissur, India; rrz Health Action by People, Trivandrum, India; ssa Department of Community Medicine, MOSC Medical College, Kolenchery, India; ssb Heart Failure Research Center, Isfahan University of Medical Sciences, Isfahan, Iran; ssc Neuroscience Research Center, Isfahan University of Medical Sciences, Isfahan, Iran; ssd Department of Medical Laboratory Analysis, Cihan University Sulaymaniya, Sulaymaniyah, Iraq; sse Department of Physiotherapy, Tehran University of Medical Sciences, Tehran, Iran; ssf Research Center for War-affected People, Tehran University of Medical Sciences, Tehran, Iran; ssg Suraj Eye Institute, Nagpur, India; ssh Mysore Medical College and Research Institute, Government Medical College, Mysore, India; ssi University Institute of Public Health, The University of Lahore, Lahore, Pakistan; ssj Heart Failure Research Center, Isfahan University of Medical Sciences, Isfahan, Iran; ssk Department of Clinical Medicine, Federal University of Minas Gerais, Belo Horizonte, Brazil; ssl Clinical Hospital, Federal University of Minas Gerais, Belo Horizonte, Brazil; ssm National Dental Research Institute Singapore, Duke-NUS Medical School, Singapore, Singapore; ssn Health Management and Economics Research Center, Iran University of Medical Sciences, Tehran, Iran; sso Faculty of Medicine, Isfahan University of Medical Sciences, Isfahan, Iran; ssp Tehran Heart Center, Tehran University of Medical Sciences, Tehran, Iran; ssq Faculty of Medicine, Tehran University of Medical Sciences, Tehran, Iran; ssr Department of Dental Public Health, King Abdulaziz University, Jeddah, Saudi Arabia; sss Department of Health Policy and Oral Epidemiology, Harvard University, Boston, MA, USA; sst College of Medicine and Health Sciences, United Arab Emirates University, Al Ain, United Arab Emirates; ssu Department of Circulation and Medical Imaging, Norwegian University of Science and Technology, Trondheim, Norway; ssv Department of Community Medicine, University of Peradeniya, Kandy, Sri Lanka; ssw Postgraduate Institute of Medicine, University of Colombo, Colombo, Sri Lanka; ssx Amity Institute of Forensic Sciences, Amity University, Noida, India; ssy Department of Forensic Medicine, Manipal Academy of Higher Education, Manipal, India; ssz Department of Health Promotion, Zahedan University of Medical Sciences, Zahedan, Iran; tta Department of Disease Control and Environmental Health, Makerere University, Kampala, Uganda; ttb Department of General Surgery, Carol Davila University of Medicine and Pharmacy, Bucharest, Romania; ttc Department of General Surgery, Emergency University Hospital Bucharest, Bucharest, Romania; ttd Department of Anatomy and Embryology, Carol Davila University of Medicine and Pharmacy, Bucharest, Romania; tte Department of Cardiology, Cardio-Aid, Bucharest, Romania; ttf HIV/STI Surveillance Research Center, Kerman University of Medical Sciences, Kerman, Iran; ttg Department of Epidemiology, Non-Communicable Diseases Research Center (NCDRC), Tehran, Iran; tth Faculty of Medicine, Euromed University of Fes, Fez, Morocco; tti Faculty of Medicine, University Sidi Mohammed Ben Abdellah, Fez, Morocco; ttj Applied Cellular and Molecular Research Center, Kerman University of Medical Sciences, Kerman, Iran; ttk Department of Community Medicine, Kathmandu University, Palpa, Nepal; ttl Department of Psychiatry, University of Oxford, Oxford, UK; ttm Department of Neurosciences, Kenya Medical Research Institute/Wellcome Trust Research Programme, Kilifi, Kenya; ttn Division of Cardiology, Massachusetts General Hospital, Boston, MA, USA; tto Department of Medical Engineering, University of South Florida, Tampa, FL, USA; ttp Cardiovascular laboratory, Methodist Hospital, Merrillville, Merrillville, IN, USA; ttq Department of Allergy, Immunology and Dermatology, Hanoi Medical University, Hanoi, Vietnam; ttr Faculty of Medicine, Duy Tan University, Da Nang, Vietnam; tts Institute for Research and Training in Medicine, Biology and Pharmacy, Duy Tan University, Da Nang, Vietnam; ttt Cardiovascular Research Department, Methodist Hospital, Merrillville, IL, USA; ttu Department of General Medicine, Pham Ngoc Thach University of Medicine, Ho Chi Minh City, Vietnam; ttv Faculty of Medicine, Pham Ngoc Thach University of Medicine, Ho Chi Minh City, Vietnam; ttw Department of Surgery, Danang Family Hospital, Danang, Vietnam; ttx Department of General Medicine, University of Medicine and Pharmacy at Ho Chi Minh City, Ho Chi Minh City, Vietnam; tty International Islamic University Islamabad, Islamabad, Pakistan; ttz Institute for Mental Health Policy Research, Centre for Addiction and Mental Health, Toronto, ON, Canada; uua Department of Nephrology and Hypertension, Mayo Clinic, Rochester, MN, USA; uub Public Health Department, Universitas Negeri Semarang (State University of Semarang?), Kota Semarang, Indonesia; uuc Graduate Institute of Biomedical Informatics, Taipei Medical University, Taipei, Taiwan; uud Technical Department, South African Medical Research Council, Cape Town, South Africa; uue School of Public Health and Family Medicine, University of Cape Town, Cape Town, South Africa; uuf Center for Public Health, Teesside University, Middlesbrough, UK; uug Department of Health Policy and Management, Keio University, Tokyo, Japan; uuh Department of Global Health Policy, University of Tokyo, Tokyo, Japan; uui Department of Maternal and Child health, International Centre for Diarrhoeal Disease Research, Bangladesh, Dhaka, Bangladesh; uuj Department of Statistics, Shahjalal University of Science and Technology, Sylhet, Bangladesh; uuk Department of Clinical Sciences, Lund University, Lund, Sweden; uul School of Medicine, Xiamen University, Xiamen, China; uum Centre for Heart Rhythm Disorders, University of Adelaide, Adelaide, SA, Australia; uun Department of Paediatrics, Nnamdi Azikiwe University, Awka, Nigeria; uuo Department of Internal Medicine, University of Thessaly, Larissa, Greece; uup Division of Cardiology, University of Cape Town, Cape Town, South Africa; uuq The Cardiac Clinic, Groote Schuur Hospital, Cape Town, South Africa; uur Department of Radiology, Mayo Clinic, Rochester, MN, USA; uus School of Information, University of California Berkeley, Berkeley, CA, USA; uut Department of Health Sciences and Public Health, Università Cattolica del Sacro Cuore, Rome, Italy; uuu School of Economics, Università Cattolica del Sacro Cuore, Rome, Italy; uuv Department of Public Health, Banten School of Health Science, South Tangerang, Indonesia; uuw Ministry of Research, Technology and Higher Education, Higher Education Service Institutions (LL-DIKTI) Region IV, Bandung, Indonesia; uux Center of Excellence in Reproductive Health Innovation (CERHI), University of Benin, Benin City, Nigeria; uuy Department of Physiology, University of Benin, Edo, Nigeria; uuz Department of Physiology, Benson Idahosa University, Benin City, Nigeria; vva Department of Applied Economics and Quantitative Analysis, University of Bucharest, Bucharest, Romania; vvb James Cook University, Townsville, QLD, Australia; vvc Department of Veterinary Public Health and Preventive Medicine, University of Ilorin, Ilorin, Nigeria; vvd Department of Medicine, National University of Ireland - Galway, Galway, Ireland; vve School of Health and Related Research, University of Sheffield, Sheffield, UK; vvf Department of Preventive Medicine, Kyung Hee University, Seoul, South Korea; vvg Department of Gynecology and Obstetrics, Emory University, Atlanta, GA, USA; vvh Health Promotion Research Center, Zahedan University of Medical Sciences, Zahedan, Iran; vvi Centre for Social Research in Health, University of New South Wales, Sydney, NSW, Australia; vvj University of Sydney, Sydney, NSW, Australia; vvk Department of Food and Nutrition, Seoul National University, Seoul, South Korea; vvl College of Medicine, University of Ibadan, Ibadan, Nigeria; vvm Department of Food Science and Postharvest Technology, Gulu University, Gulu, Uganda; vvn School of Pharmacy, University of the Western Cape, Cape Town, South Africa; vvo Gorgan Congenital Malformations Research Center, Golestan University of Medical Sciences, Gorgan, Iran; vvp Department of Psychiatry and Behavioural Neurosciences, McMaster University, Hamilton, ON, Canada; vvq Department of Psychiatry, University of Lagos, Lagos, Nigeria; vvr Department of Medicine, Monash University, Clayton, VIC, Australia; vvs Department of Neurology, University College Hospital, Ibadan, Ibadan, Nigeria; vvt Department of Medicine, University of Ibadan, Ibadan, Nigeria; vvu Nutriton and Toxicology, Federal institute of Industrial Research, Lagos, Nigeria; vvv Department of Biochemistry, Genetics and Microbiology, University of KwaZulu-Natal, Westville, South Africa; vvw Department of Health Research Methods, Evidence, and Impact, McMaster University, Hamilton, ON, Canada; vvx Department of Nursing Science, Bowen University, Iwo, Nigeria; vvy Center for Clinical and Epidemiological Research, University of São Paulo, São Paulo, Brazil; vvz Associação Brasileira de Cefaleia em Salvas e Enxaqueca (ABRACES), São Paulo, Brazil; wwa Cardiology Department, Federal University of Rio de Janeiro, Rio de Janeiro, Brazil; wwb Department of Community Medicine, Ahmadu Bello University, Zaria, Nigeria; wwc Slum and Rural Health Initiative Research Academy, Slum and Rural Health Initiative, Ibadan, Nigeria; wwd Faculty of Public Health, University of Ibadan, Ibadan, Nigeria; wwe Department of Microbiology, University of Medical Sciences, Ondo, Ondo, Nigeria; wwf Department of Anatomy, University of Medical Sciences, Ondo, Ondo, Nigeria; wwg Department of Anatomy, Olabisi Onabanjo University, Sagamu, Nigeria; wwh Department of Pharmacy Practice and Pharmacotherapeutics, University of Sharjah, Sharjah, United Arab Emirates; wwi Department of Pharmacology and Toxicology, Beni-Suef University, Beni-Suef, Egypt; wwj Diplomacy and Public Relations Department, University of Human Development, Sulaymaniyah, Iraq; wwk Institute for Mental and Physical Health and Clinical Translation (IMPACT), Deakin University, Geelong, VIC, Australia; wwl Department of Public Health, Ministry of Health, Bandar Seri Begawan, Brunei; wwm Institute of Health Sciences, Universiti Brunei Darussalam, Bandar Seri Begawan, Brunei; wwn Department of Pharmacology and Therapeutics, University of Nigeria Nsukka, Enugu, Nigeria; wwo Department of Geography, East Carolina University, Greenville, NC, USA; wwp Department of Pharmacotherapy and Pharmaceutical Care, Medical University of Warsaw, Warsaw, Poland; wwq Department of Biotechnological and Applied Clinical Sciences, University of L'Aquila, L'Aquila, Italy; wwr Department of Neurology, ASL Avezzano-Sulmona-L'Aquila, L'Aquila, Italy; wws Center for Health Systems Research, National Institute of Public Health, Cuernavaca, Mexico; wwt Department of Medicine, Universidad Autónoma de Madrid (Autonomous University of Madrid), Madrid, Spain; wwu Department of Nephrology and Hypertension, The Institute for Health Research Foundation Jiménez Díaz University Hospital, Madrid, Spain; wwv One Health Global Research Group, Universidad de las Americas (University of the Americas), Quito, Ecuador; www Department of Biology, Khalifa University, Abu Dhabi, United Arab Emirates; wwx School of Medicine, Western Sydney University, Bathurst, NSW, Australia; wwy Department of Optometry and Vision Science, University of KwaZulu-Natal, KwaZulu-Natal, South Africa; wwz Laboratory of Public Health Indicators Analysis and Health Digitalization, Moscow Institute of Physics and Technology, Dolgoprudny, Russia; xxa Department of Project Management, National Research University Higher School of Economics, Moscow, Russia; xxb Department of Medicine, University of Ibadan, Ibadan, Nigeria; xxc Department of Medicine, University College Hospital, Ibadan, Ibadan, Nigeria; xxd Department of Biosciences and Biotechnology, University of Medical Sciences, Ondo, Ondo, Nigeria; xxe School of Medicine, Johns Hopkins University, Baltimore, MD, USA; xxf Miami Cancer Institute, Baptist Health South Florida, Miami, FL, USA; xxg Department of Respiratory Medicine, Jagadguru Sri Shivarathreeswara University, Mysore, India; xxh Department of Physical Medicine and Rehabilitation, Harvard University, Boston, MA, USA; xxi Universidad San Ignacio de Loyola, Lima, Peru; xxj National School of Public Health, Institute of Health Carlos III, Madrid, Spain; xxk Department of Forensic Medicine and Toxicology, Manipal Academy of Higher Education, Mangalore, India; xxl Health Services Management Training Centre, Semmelweis University, Budapest, Hungary; xxm Hungarian Health Management Association, Budapest, Hungary; xxn Department of Mental Health, Hospital Universitari Vall d'Hebron (Vall d'Hebron University Hospital), Barcelona, Spain; xxo Department of Psychiatry, Mental Health and Addictions, Vall d'Hebron Institut de Recerca (Vall d'Hebron Research Institute), Barcelona, Spain; xxp Menzies Institute for Medical Research, University of Tasmania, Hobart, TAS, Australia; xxq Department of Ophthalmology, Heidelberg University, Heidelberg, Germany; xxr Amity Institute of Biotechnology, Amity University Uttar Pradesh, Noida, India; xxs Public Health Foundation of India, Gurugram, India; xxt Research Department, Nepal Health Research Council, Kathmandu, Nepal; xxu Research Department, Public Health Research Society Nepal, Kathmandu, Nepal; xxv Centre for Research and Development, Chandigarh University, Punjab, India; xxw Division of Research and Development, Lovely Professional University, Phagwara, India; xxx Department of Neurology, University of Bern, Bern, Switzerland; xxy Department of Neurology, University of Cyprus, Nicosia, Cyprus; xxz Department of Emergency Medicine, University of Thessaly, Larissa, Greece; yya Department of Emergency Medicine, University of Bern, Bern, Switzerland; yyb Department of Science and Mathematics, Deree-The American College of Greece, Athens, Greece; yyc Department of Biophysics, University of Athens, Athens, Greece; yyd Vision and Eye Research Institute, Anglia Ruskin University, Cambridge, UK; yye Department of Community Medicine, All India Institute of Medical Sciences, Jammu, India; yyf Department of Epidemiology and Community Health, University of Minnesota, Minneapolis, MN, USA; yyg School of Psychological Sciences, Monash University, Melbourne, VIC, Australia; yyh Department of Medical Sciences, University of Torino, Torino, Italy; yyi Department of Imaging, AOU Città della Salute e della Scienza di Torino, Torino, Italy; yyj Aristotle University of Thessaloniki, Greece, Thessaloniki, Greece; yyk Second Department of Cardiology, Aristotle University of Thessaloniki, Thessaloniki, Greece; yyl Research Department, Nepal Health Research Council, Kathmandu, Nepal; yym Faculty of Humanities and Social Sciences, Tribhuvan University, Kathmandu, Nepal; yyn Department of Genetics, Yale University, New Haven, CT, USA; yyo School of Public Health and Community Medicine, University of New South Wales, Sydney, NSW, Australia; yyp College of Public Health, Medical, and Veterinary Sciences, James Cook University, Townsville, QLD, Australia; yyq Clinical Research Department, IRCCS Fondazione Don Carlo Gnocchi, Milan, Italy; yyr Australian Institute of Health Innovation, Macquarie University, Sydney, NSW, Australia; yys Department of Applied Nursing, Federal University of Minas Gerais, Belo Horizonte, Brazil; yyt Department of Psychiatry, University of São Paulo, São Paulo, Brazil; yyu International Institute for Educational Planning (IIEP), Albert Einstein Hospital, São Paulo, Brazil; yyv Social and Economic Survey Research Institute, Qatar University, Doha, Qatar; yyw Mario Negri Institute for Pharmacological Research, Bergamo, Italy; yyx Department of Biology, University of Bahrain, Zallaq, Bahrain; yyy Pennsylvania Cancer and Regenerative Medicine Center, Baruch S Blumberg Institute, Doylestown, PA, USA; yyz Department of Medicine, Xavier University School of Medicine, Woodbury, NY, USA; zza Department of Statistics and Econometrics, Bucharest University of Economic Studies, Bucharest, Romania; zzb Facultad de Medicina, (Universidad Diego Portales) Diego Portales University, Santiago, Chile; zzc School of Cardiovascular and Metabolic Health, University of Glasgow, Glasgow, UK; zzd Department of Internal Medicine, University of Arizona, Tucson, AZ, USA; zze Department of Cardiovascular Medicine, Mayo Clinic, Rochester, MN, USA; zzf Department of Internal Medicine, Weiss Memorial Hospital, Chicago, IL, USA; zzg Shanghai Mental Health Center, Shanghai Jiao Tong University, Shanghai, China; zzh Departments of Psychiatry and Epidemiology, Columbia University, New York, NY, USA; zzi Department of Cardiology, University of Bern, Bern, Switzerland; zzj Research Center of Neurology, Moscow, Russia; zzk Molecular Medicine Research Center, Tabriz University of Medical Sciences, Tabriz, Iran; zzl Research School of Chemistry and Applied Biomedical Sciences, Tomsk Polytechnic University, Tomsk, Russia; zzm Siberian State Medical University, Tomsk, Russia; zzn Department of Medicine, Nazarbayev University, Astana, Kazakhstan; zzo Clinical Academic Department of Pediatrics, University Medical Center (UMC), Astana, Kazakhstan; zzp Department of Data Management and Analysis, The International Clinical Epidemiology Network (INCLEN) Trust International, New Delhi, India; zzq Department of Internal Medicine, University of Novi Sad, Novi Sad, Serbia; zzr Clinic for Endocrinology, Diabetes and Metabolic Disorders, Clinical Center of Vojvodina, Novi Sad, Serbia; zzs University Medical Center Groningen, University of Groningen, Groningen, Netherlands; zzt Center of Excellence in Higher Education for Pharmaceutical Care Innovation, Universitas Padjadjaran (Padjadjaran University), Bandung, Indonesia; zzu Digestive Diseases Research Institute, Tehran University of Medical Sciences, Tehran, Iran; zzv Non-communicable Diseases Research Center, Bam University of Medical Sciences, Bam, Iran; zzw Humanities and Social Sciences, National Institute of Technology Rourkela, Rourkela, India; zzx Department of Community Medicine, Tribhuvan University, Kathmandu, Nepal; zzy T.H. Chan School of Public Health, Harvard University, Boston, MA, USA; zzz Department of Neurophysiotherapy, MGM Institute of Physiotherapy, Chhatrapati Sambhaginagar, India; aaaa Department of Clinical Research and Epidemiology, Institute of Liver and Biliary Sciences, New Delhi, New Delhi, India; aaab Department of Maternal-Child Nursing and Public Health, Federal University of Minas Gerais, Belo Horizonte, Brazil; aaac Health Sciences Department, Muhammadiyah University of Surakarta, Sukoharjo, Indonesia; aaad Department of Biostatistics, Epidemiology, and Informatics, University of Pennsylvania, Philadelphia, PA, USA; aaae Department of Neonatology, Case Western Reserve University, Cleveland, OH, USA; aaaf Department of Cardiology, Guiqian International General Hospital, Guiyang, China; aaag Department of Epidemiology, Shandong University, Jinan, China; aaah UO Neurologia, Salute Pubblica e Disabilità, Fondazione IRCCS Istituto Neurologico Carlo Besta (The Neurology, Public Health and Disability Unit, IRCCS Foundation Carlo Besta Neurological Institute), Milan, Italy; aaai Department of Community Medicine and Family Medicine, All India Institute of Medical Sciences, Jodhpur, India; aaaj Department of Epidemiology, National Institute of Mental Health and Neurosciences, Bengaluru, India; aaak Department of Medical Laboratory Technologies, Alnoor University, Mousl, Iraq; aaal Al-Noor Center of Research and Innovation, Alnoor University, Mousl, Iraq; aaam Department of Health Policy & Organization, University of Alabama at Birmingham, Birmingham, AL, USA; aaan Department of Health Services Administration, University of Alabama at Birmingham, Birmingham, AL, USA; aaao Pharmaceutical Sciences Research Center (PSRC), Tehran University of Medical Sciences, Tehran, Iran; aaap Sina Trauma and Surgery Research Center, Tehran University of Medical Sciences, Tehran, Iran; aaaq Department of Population Science and Human Resource Development, University of Rajshahi, Rajshahi, Bangladesh; aaar Manipal TATA Medical College, Manipal Academy of Higher Education, Manipal, India; aaas Department of Population Science and Human Resource Development, University of Rajshahi, Rajshahi, Bangladesh; aaat Institute of Health and Wellbeing, Federation University Australia, Berwick, VIC, Australia; aaau School of Nursing and Midwifery, La Trobe University, Melbourne, VIC, Australia; aaav Future Technology Research Center, National Yunlin University of Science and Technology, Yunlin, Taiwan; aaaw Student Research Committee, Shahid Beheshti University of Medical Sciences, Tehran, Iran; aaax Inflammatory Lung Diseases Research Center, Guilan University of Medical Sciences, Rasht, Iran; aaay Department of Public Health, Torbat Jam Faculty of Medical Sciences, Torbat Jam, Iran; aaaz Student Research Committee, Shahrekord University of Medical Sciences, Shahrekord, Iran; bbba Department of Nutrition Science, Muhammadiyah University of Surakarta, Surakarta, Indonesia; bbbb Department of Pharmacology, All India Institute of Medical Sciences, Hyderabad, India; bbbc Department of Community Medicine, Employees' State Insurance Model Hospital, Chennai, India; bbbd Department of Community Medicine and Family Medicine, All India Institute of Medical Sciences, Gorakhpur, India; bbbe Department of Health Education and Promotion, Jazan University, Jazan, Saudi Arabia; bbbf Centre for Chronic Disease Control, New Delhi, India; bbbg Department of Cardiology, Emory University, Atlanta, GA, USA; bbbh Department of Clinical Science, University of Sharjah, Sharjah, United Arab Emirates; bbbi Department of Cardiology, Mansoura University, Mansoura, Egypt; bbbj Department of Population Health, King Saud bin Abdulaziz University for Health Sciences, Jeddah, Saudi Arabia; bbbk Department of Public Health Dentistry, Amrita Institute of Medical Sciences, Kochi, India; bbbl Department of Radiology, Stanford University, Stanford, CA, USA; bbbm School of Nursing & Health Sciences, Hong Kong Metropolitan University, Hong Kong, China; bbbn Saw Swee Hock School of Public Health, National University of Singapore, Singapore, Singapore; bbbo South Asian Institute for Social Transformation (SAIST), Dhaka, Bangladesh; bbbp Department of Epidemiology, Biostatistics and Occupational Health, McGill University, Montreal, Canada; bbbq Translational Health Research Institute, Western Sydney University, Sydney, NSW, Australia; bbbr Department of Community Medicine, Shaheed Nirmal Mahto Medical College and Hospital, Dhanbad, India; bbbs Research Department, Eastern Scientific LLC, Richmond, KY, USA; bbbt Department of Health Promotion and Administration, Eastern Kentucky University, Richmond, KY, USA; bbbu Centre for Clinical Pharmacology, University of Defence in Belgrade, Belgrade, Serbia; bbbv Centre for Clinical Pharmacology, Medical College of Georgia at Augusta University, Belgrade, Serbia; bbbw Health Economics and Outcomes Research Department, Agios Pharmaceuticals, Cambridge, MA, USA; bbbx Department of Pharmaceutical Economics and Policy, Massachusetts College of Pharmacy and Health Sciences, Boston, MA, USA; bbby Department of Medicine, University of Otago, Wellington, New Zealand; bbbz Department of Neurology, Capital & Coast District Health Board, Wellington, New Zealand; ccca Kasturba Medical College Mangalore, Manipal Academy of Higher Education, Manipal, India; cccb Department of Oral Pathology, Microbiology and Forensic Odontology, Sharavathi Dental College and Hospital, Shimogga, India; cccc Thrombosis Research Group, Brigham and Women's Hospital, Harvard Medical School, Boston, MA, USA; cccd Department of Epidemiology, Non-Communicable Diseases Research Center (NCDRC), Tehran, Iran; ccce Non-communicable Diseases Research Center, Tehran University of Medical Sciences, Tehran, Iran; cccf Social Determinants of Health Research Center, Shahid Beheshti University of Medical Sciences, Tehran, Iran; cccg Department of Immunology, Shahid Beheshti University of Medical Sciences, Tehran, Iran; ccch Department of Family Medicine, Rajarata University of Sri Lanka, Anuradhapura, Sri Lanka; ccci Department of Global Health Policy, University of Tokyo, Tokyo, Japan; cccj Department of Primary Care and Public Health, Imperial College London, London, UK; ccck Academic Public Health England, Public Health England, London, UK; cccl Institute for Health Metrics and Evaluation, University of Washington, Seattle, WA, USA; cccm Department of Internal Medicine, Manipal Academy of Higher Education, Mangalore, India; cccn Department of Biological Sciences, King Abdulaziz University, Jeddah, Egypt; ccco Department of Protein Research, Research and Academic Institution, Alexandria, Egypt; cccp Department of Pharmacy, Abdul wali Khan University Mardan, Mardan, Pakistan; cccq Mario Negri Institute for Pharmacological Research, Bergamo, Italy; cccr Non-communicable Diseases Research Center, Tehran University of Medical Sciences, Tehran, Iran; cccs Department of Epidemiology and Biostatistics, Rafsanjan University of Medical Sciences, Rafsanjan, Iran; ccct Department of Physiology, Isfahan University of Medical Sciences, Isfahan, Iran; cccu Rafsanjan University of Medical Sciences, Rafsanjan, Iran; cccv Department of Public Health Sciences, University of Connecticut, Farmington, CT, USA; cccw Department of Psychiatry, Yale University, New Haven, CT, USA; cccx The University of Lahore, Lahore, Pakistan; cccy Department of Internal Medicine, Federal University of Minas Gerais, Belo Horizonte, Brazil; cccz Centre of Telehealth, Federal University of Minas Gerais, Belo Horizonte, Brazil; ddda Department of Geography and Demography, University of Coimbra, Coimbra, Portugal; dddb Department of Nursing in Women’s Health, Federal University of São Paulo, São Paulo, Brazil; dddc Vaccination Research Observatory, Federal University of Minas Gerais, Belo Horizonte, Brazil; dddd Department of Pharmacology and Toxicology, University of Antioquia, Medellin, Colombia; ddde Warwick Medical School, University of Warwick, Coventry, UK; dddf Department of Clinical Research, Federal University of Uberlândia, Ribeirão Preto, Brazil; dddg Gilbert and Rose-Marie Chagoury School of Medicine, Lebanese American University, Beirut, Lebanon; dddh Faculty of Nursing, Chulalongkorn University, Bangkok, Thailand; dddi Rural Health research Institute, Charles Sturt University, Orange, NSW, Australia; dddj Department of Analytical and Applied Economics, Utkal University, Bhubaneswar, India; dddk RUSA Centre of Excellence in Public Policy and Governance, Utkal University, Bhubaneswar, India; dddl Faculty of Medicine, Quest International University Perak, Ipoh, Malaysia; dddm Department of Labour, Directorate of Factories Government of West Bengal, Kolkata, India; dddn Department of Epidemiology, Florida International University, Miami, FL, USA; dddo Advanced Campus Governador Valadares, Juiz de For a Federal University, Governador Valadares, Brazil; dddp Nursing Department, Universidade Presidente Antônio Carlos (President Antônio Carlos University), Governador Valadares, Brazil; dddq Department of Cardiology, S. Maria dei Battuti Hospital, Conegliano, Italy; dddr Department of Internal Medicine, University of Botswana, Gaborone, Botswana; ddds Department of Oral and Maxillofacial Surgery, Jagadguru Sri Shivarathreeswara University, Mysore, India; dddt Cardiovascular Department, Zagazig University, Zagazig, Egypt; dddu Department of Medical Physics, Isfahan University of Medical Sciences, Isfahan, Iran; dddv Clinical Sciences Department, University of Sharjah, Sharjah, United Arab Emirates; dddw Department of Medical Pharmacology, Cairo University, Giza, Egypt; dddx Department of Medicine, Georgetown University, Washington, DC, USA; dddy Department of Epidemiology, Shahid Beheshti University of Medical Sciences, Tehran, Iran; dddz Department of Neurology, University of L'Aquila, L'Aquila, Italy; eeea College of Medicine, University of Sharjah, Sharjah, United Arab Emirates; eeeb School of Population Health, University of New South Wales, Sydney, NSW, Australia; eeec Department of Biostatistics, Shiraz University of Medical Sciences, Shiraz, Iran; eeed Department of Pharmaceutical Chemistry, International Medical University, Gdańsk, Poland; eeee Clinical and Biomedical Research Center, Foundation University Islamabad, Islamabad, Pakistan; eeef International Center of Medical Sciences Research (ICMSR), Islamabad, Pakistan; eeeg Faculty of Medicine, Bioscience and Nursing, MAHSA University, Selangor, Malaysia; eeeh Interdisciplinary Research Centre in Biomedical Materials (IRCBM), COMSATS Institute of Information Technology, Lahore, Pakistan; eeei Department of Psychiatry, All India Institute of Medical Sciences, New Delhi, India; eeej Clinical Research Development Center (CRDC), Qom University of Medical Sciences, Qom, Iran; eeek Department of Psychosocial Science, University of Bergen, Bergen, Norway; eeel Sharjah Institute of Medical Sciences, University of Sharjah, Sharjah, United Arab Emirates; eeem Applied Biomedical Research Center, Mashhad University of Medical Sciences, Mashhad, Iran; eeen Biotechnology Research Center, Mashhad University of Medical Sciences, Mashhad, Iran; eeeo Department of Analytical & Applied Economics, Utkal University, Bhubaneswar, India; eeep Department of Community Medicine and Family Medicine, All India Institute of Medical Sciences, Bathinda, India; eeeq Department of Statistics, University of Gujrat, Gujrat, Pakistan; eeer Department of Oral and Maxillofacial Surgery, University College Hospital, Ibadan, Ibadan, Nigeria; eees Faculty of Dentistry, University of Puthisastra, Phnom Penh, Cambodia; eeet Department of Integrated Health Education, Federal University of Espirito Santo, Vitória, Brazil; eeeu College of Medicine, University of Sharjah, Sharjah, United Arab Emirates; eeev Faculty of Pharmacy, Mansoura University, Mansoura, Egypt; eeew Technology Management Department, University College of Applied Sciences, Gaza, Palestine; eeex School of Economics and Management, University of Kassel, Kassel, Germany; eeey Department of Global Initiatives, Child Mind Institute, New York, NY, USA; eeez Department of Psychiatry and Legal Medicine, Federal University of Rio Grande do Sul, Porto Alegre, Brazil; fffa Department of Neurology, Charité Universitätsmedizin Berlin (Charité Medical University Berlin), Berlin, Germany; fffb Department of Neurology, University of Southern Denmark, Odense, Denmark; fffc Department of Community Medicine, King Abdulaziz University, Jeddah, Saudi Arabia; fffd Institute of Epidemiology and Preventive Medicine, National Taiwan University, Taipei, Taiwan; fffe Benang Merah Research Center (BMRC), Minahasa Utara, Indonesia; ffff Department of Anatomy, Ras Al Khaimah Medical and Health Sciences University, Ras Al Khaimah, United Arab Emirates; fffg Department of Entomology, Ain Shams University, Cairo, Egypt; fffh Medical Ain Shams Research Institute (MASRI), Ain Shams University, Cairo, Egypt; fffi Department of Surgery, Marshall University, Huntington, WV, USA; fffj Department of Nutrition and Preventive Medicine, Case Western Reserve University, Cleveland, OH, USA; fffk Department of Internal Medicine, University of São Paulo, São Paulo, Brazil; fffl Center for Clinical and Epidemiological Research, University of São Paulo, São Paulo, Brazil; fffm Faculty of Medicine, University of Belgrade, Belgrade, Serbia; fffn School of Public Health and Health Management, University of Belgrade, Belgrade, Serbia; fffo Pharmacy Study Program, Udayana University, Badung, Indonesia; fffp Department of Clinical Pharmacy, Taipei Medical University, Taipei, Taiwan; fffq Department of Pharmacology and Research, All India Institute of Medical Sciences, Jodhpur, India; fffr Indira Gandhi Medical College and Research Institute, Puducherry, India; fffs Department of Public Health, Jahrom University of Medical Sciences, Jahrom, Iran; ffft Health Policy Research Center, Shiraz University of Medical Sciences, Shiraz, Iran; fffu Department of Oral Pathology and Microbiology, Dr. D. Y. Patil University, Pune, India; fffv Department of Oral Pathology and Microbiology, Dr. D. Y. Patil University, Pune, India; fffw UGC Centre of Advanced Study in Psychology, Utkal University, Bhubaneswar, India; fffx Udyam-Global Association for Sustainable Development, Bhubaneswar, India; fffy Department of Medicine, Case Western Reserve University, Cleveland, OH, USA; fffz Department of Preventive and Social Medicine, Jawaharlal Institute of Postgraduate Medical Education and Research, Puducherry, India; ggga National Centre for Epidemiology and Population Health, Australian National University, Acton, ACT, Australia; gggb Education Development Center, Ahvaz Jundishapur University of Medical Sciences, Ahvaz, Iran; gggc Department of Neurology, National and Kapodistrian University of Athens, Athens, Greece; gggd Department of Neurology, Columbia University, New York, NY, USA; ggge Department of Diagnostic and Interventional Radiology and Neuroradiology, University Hospital Essen, Essen, Germany; gggf Dobney Hypertension Centre, The University of Western Australia, Perth, WA, Australia; gggg Hypertension and Kidney Disease Laboratory, Baker Heart and Diabetes Institute, Melbourne, VIC, Australia; gggh Postgraduate Program in Epidemiology, Federal University of Rio Grande do Sul, Porto Alegre, Brazil; gggi Department of Health Sciences, Federal University of Santa Catarina, Araranguá, Brazil; gggj Cardiovascular Research Center, Massachusetts General Hospital, Boston, MA, USA; gggk Department of Cardiovascular Sciences, Katholieke Universiteit Leuven, Leuven, Belgium; gggl Institute for Health Metrics and Evaluation, University of Washington, Seattle, WA, USA; gggm School of Population Health, University of New South Wales, Sydney, NSW, Australia; gggn The George Institute for Global Health, Sydney, NSW, Australia; gggo Department of Psychology, University of Alabama at Birmingham, Birmingham, AL, USA; gggp Department of Community Oral Health and Clinical Prevention, University of Malaya, Kuala Lumpur, Malaysia; gggq Division of Pulmonary and Critical Care, University of Kentucky, Lexington, KY, USA; gggr Department of Human Genetics and Molecular Medicine, Central University of Punjab, Bathinda, India; gggs Emergency Department, Manian Medical Centre, Erode, India; gggt Public health, Wachemo University, Hossana, Ethiopia; gggu Department of Medicine and Surgery, Government Doon Medical College, Dehradun, India; gggv National Heart, Lung, and Blood Institute, National Institute of Health, Rockville, MD, USA; gggw Department of Neurology, Tehran University of Medical Sciences, Tehran, Iran; gggx Department of Microbiology, Rajiv Gandhi University of Health Sciences, Bangalore, India; gggy Health Policy Research Center, Shiraz University of Medical Sciences, Shiraz, Iran; gggz Department of Medicine, Tehran University of Medical Sciences, Tehran, Iran; hhha Institute of Molecular Biology and Biotechnology, The University of Lahore, Lahore, Pakistan; hhhb Research Centre for Health Sciences (RCHS), The University of Lahore, Lahore, Pakistan; hhhc Department of Chemistry, Institute for Advanced Studies in Basic Sciences (IASBS), Zanjan, Iran; hhhd Center for Medical and Bio-Allied Health Sciences Research, Ajman University, Ajman, United Arab Emirates; hhhe Independent Consultant, Karachi, Pakistan; hhhf Neurology Department, Ain Shams University, Cairo, Egypt; hhhg Department of Pharmacology, All India Institute of Medical Sciences, Jodhpur, India; hhhh School of Medicine, Alborz University of Medical Sciences, Karaj, Iran; hhhi Center for Medical and Bio-Allied Health Sciences Research, Ajman University, Ajman, United Arab Emirates; hhhj Centre For Interdisciplinary Research In Basic Sciences (CIRBSc), Jamia Millia Islamia, New Delhi, India; hhhk Science Department, Kazakh National Medical University, Almaty, Kazakhstan; hhhl Department of Health Education and Promotion, Jazan University, Jazan, Saudi Arabia; hhhm Amity Institute of Public Health, Amity University, Noida, India; hhhn Department of Medicine, Bangalore Medical College and Research Institute, Bangalore, India; hhho Department for Evidence-based Medicine and Evaluation, University for Continuing Education Krems, Krems, Austria; hhhp Research Center for Rational Use of Drugs, Tehran University of Medical Sciences, Tehran, Iran; hhhq Nahavand School of Allied Medical Sciences, Hamadan University of Medical Sciences, Hamadan, Nahavand, Iran; hhhr Department of Biomedical Sciences, Korea University, Seoul, South Korea; hhhs Department of Hemato-oncology, Fortis Hospital, Noida, India; hhht Department of Social and Behavioral Health, University of Nevada Las Vegas, Las Vegas, NV, USA; hhhu Department of Nephrology, Vardhman Mahavir Medical College, New Delhi, India; hhhv Department of Human Genetics and Molecular Medicine, Central University of Punjab, Bathinda, India; hhhw Institute of Forensic Science & Criminology, Panjab University, Chandigarh, India; hhhx Department of Health in Disasters and Emergencies, Shahrekord University of Medical Sciences, Shahrekord, Iran; hhhy Department of Obstetrics and Gynaecology, Manipal Academy of Higher Education, Mangalore, India; hhhz K S Hegde Medical Academy, Nitte University, Mangalore, India; iiia Manipal College of Dental Sciences, Manipal Academy of Higher Education, Mangalore, India; iiib Department of Public Health, Dambi Dollo University, Dembi Dollo, Ethiopia; iiic Department of Epidemiology, Jimma University, Jimma, Ethiopia; iiid National Institute of Infectious Diseases, Tokyo, Japan; iiie Deparment of Pharmacology, Saint Paul's Hospital Millennium Medical College, Addis Ababa, Ethiopia; iiif Korea University, Seoul, South Korea; iiig Finnish Institute of Occupational Health, Helsinki, Finland; iiih Department of Veterinary Public Health and Preventive Medicine, Usmanu Danfodiyo University, Sokoto, Sokoto, Nigeria; iiii Department of Medicine, Ladoke Akintola University, Ogbomoso, Nigeria; iiij Oulu Business School, University of Oulu, Oulu, Finland; iiik Martti Ahtisaari Institute, University of Oulu, Oulu, Finland; iiil Department of Medical-Surgical Nursing, Mazandaran University of Medical Sciences, Sari, Iran; iiim Department of Nursing and Health Sciences, Flinders University, Adelaide, SA, Australia; iiin School of Pharmacy, Monash University, Subang Jaya, Malaysia; iiio Department of Research, Kathmandu Cancer Center, Bhaktapur, Nepal; iiip The Cooper Institute, Dallas, TX, USA; iiiq School of Risk and Actuarial Studies, University of New South Wales, Sydney, NSW, Australia; iiir Unit of Basic Medical Sciences, University of Khartoum, Khartoum, Sudan; iiis Department of Medical Microbiology and Infectious Diseases, Erasmus University, Rotterdam, Netherlands; iiit Department of Culture, Anthropological Survey of India, Kolkata, India; iiiu Research Unit on Applied Molecular Biosciences (UCIBIO), University of Porto, Porto, Portugal; iiiv Sport Physical Activity and Health Research & Innovation Center (SPRINT), Polytechnic Institute of Guarda, Guarda, Portugal; iiiw CICS-UBI Health Sciences Research Center, University of Beira Interior, Covilhã, Portugal; iiix Department of Dentistry, All India Institute of Medical Sciences, Bhopal, India; iiiy Department of Biochemistry, Central University of Punjab, Bathinda, India; iiiz Department of Community Medicine, Lady Hardinge Medical College, New Delhi, India; jjja Department of Community Medicine, All India Institute of Medical Sciences, Jodhpur, India; jjjb Department of Pharmacology, Government Medical College and Hospital, Chandigarh, India; jjjc School of Medicine, Baylor College of Medicine, Houston, TX, USA; jjjd Medicine Service, US Department of Veterans Affairs (VA), Houston, TX, USA; jjje Department of Pediatrics, All India Institute of Medical Sciences Jodhpur, Jodhpur, India; jjjf Faculty of Medicine and Health Sciences, Shree Guru Gobind Singh Tricentenary University, Gurugram, India; jjjg Department of Radiodiagnosis, All India Institute of Medical Sciences, Bathinda, India; jjjh Department of Human Genetics, Punjabi University, Patiala, India; jjji Department of Neurology, North Karelia Central Hospital, Joensuu, Finland; jjjj Department of Clinical Neurosciences, University of Turku, Turku, Finland; jjjk Department of Neurology, University of Massachusetts Medical School, Worcester, MA, USA; jjjl Clinical Branch, Moscow Research and Practical Centre on Addictions, Moscow, Russia; jjjm Addiction Psychiatry Department, Russian Medical Academy of Continuous Professional Education, Moscow, Russia; jjjn Department of Infectious Diseases and Epidemiology, Pirogov Russian National Research Medical University, Moscow, Russia; jjjo Division of Injury Prevention, The Bizzell Group, Atlanta, GA, USA; jjjp Rollins School of Public Health, Emory University, Atlanta, GA, USA; jjjq Department of Health Education and Promotion, Jazan University, Jazan, Saudi Arabia; jjjr Department of General Surgery, Carol Davila University of Medicine and Pharmacy, Bucharest, Romania; jjjs Department of Surgery, "Sf. Pantelimon" Emergency Clinical Hospital Bucharest, Bucharest, Romania; jjjt School of Health and Biomedical Science, Royal Melbourne Institute of Technology (RMIT) University, Melbourne, VIC, Australia; jjju Department of Systemic Pathology, Touro College of Osteopathic Medicine, Middletown, NY, USA; jjjv Department of Pathology, American University of the Caribbean School of Medicine, Cupecoy, Saint Martin; jjjw Department of Biochemistry, American University of Integrative Sciences, Bridgetown, Barbados; jjjx Department of Biomedical Sciences, Debre Berhan University, Debre Berhan, Ethiopia; jjjy Institute of Child and Adolescent Health, Peking University, Beijing, China; jjjz Urmia University of Medical Sciences, Urmia, Iran; kkka Institute for Health Metrics and Evaluation, University of Washington, Seattle, WA, USA; kkkb Department of Global Health, University of Washington, Seattle, WA, USA; kkkc Department of Radiology, University of Alabama at Birmingham, Birmingham, AL, USA; kkkd Hull York Medical School, University of Hull, Hull City, UK; kkke 3rd Department of Cardiology, University of Athens, Athens, Greece; kkkf Division of Community Medicine and Public Health, International Medical University, Kuala Lumpur, Malaysia; kkkg Pharmacology, RAK Medical and Health Sciences University, Ras Al Khaimah, United Arab Emirates; kkkh 2nd Department of Cardiology, Aristotle University of Thessaloniki, Thessaloniki, Greece; kkki Institute for Health Metrics and Evaluation, University of Washington, Seattle, WA, USA; kkkj Institute for Health Metrics and Evaluation, University of Washington, Seattle, WA, USA; kkkk Nutrition and Dietetics Department, Federal Research Institute of Nutrition, Biotechnology and Food Safety, Moscow, Russia; kkkl Department of Internal Disease, Pirogov Russian National Research Medical University, Moscow, Russia; kkkm Central Department of Public Health, Tribhuvan University, Kathmandu, Nepal; kkkn School of Exercise and Nutrition Sciences, Deakin University, Melbourne, VIC, Australia; kkko Department of Medical Sciences, Sunway University, Subang Jaya, Malaysia; kkkp Center for Biotechnology and Microbiology, University of Swat, Swat, Pakistan; kkkq School of Life Sciences, Xiamen University, Xiamen, China; kkkr Mental Health Research Consultant, Independent Consultant, Khulna, Bangladesh; kkks Division of Global Mental Health, EviSyn Health, Khulna, Bangladesh; kkkt Department of Biomedical Sciences, Universiti Putra Malaysia, Selangor, Malaysia; kkku Department of Medical Sciences, Uppsala University, Uppsala, Sweden; kkkv The George Institute for Global Health, Sydney, NSW, Australia; kkkw Department of Neurology, King George's Medical University, Lucknow, India; kkkx Nursing Professional Education Study Program, University Halu Oleo, Kendari, Indonesia; kkky School of Nursing, Taipei Medical University, Taipei, Taiwan; kkkz Department of Analytical and Applied Economics, Utkal University, Bhubaneswar, India; llla Henry JN Taub Department of Emergency Medicine, Baylor College of Medicine, Houston, TX, USA; lllb Department of Clinical Research and Development, LUXMED Group, Warsaw, Poland; lllc Department of Pharmacology, All India Institute of Medical Sciences, Deoghar, India; llld Department of Neurology, Neurocenter of Southern Switzerland (NSI), Lugano, Switzerland; llle Department of Medicine, University of Valencia, Valencia, Spain; lllf Carlos III Health Institute, Biomedical Research Networking Center for Mental Health Network (CiberSAM), Madrid, Spain; lllg Department of Medical Informatics, Mashhad University of Medical Sciences, Mashhad, Iran; lllh Clinial Research Development Unit, Mashhad University of Medical Sciences, Mashhad, Iran; llli Department of Medical Education, Shahid Beheshti University of Medical Sciences, Tehran, Iran; lllj Department of Primary Care and Public Health, Imperial College London, London, UK; lllk Department of Pharmacology, Shaqra University, Shaqra, Saudi Arabia; llll Dentistry and Oral Health, Rural Clinical Sciences, La Trobe University, Bendigo, VIC, Australia; lllm School of Dentistry and Oral Health, Griffith University, Gold Coast, QLD, Australia; llln Department of Infectious Disease, Mazandaran University of Medical Sciences, Sari, Iran; lllo Department of Dermatology, Mazandaran University of Medical Sciences, Sari, Iran; lllp Department of Environmental, Agricultural and Occupational Health, University of Nebraska Medical Center, Omaha, NE, USA; lllq Sri Ramachandra Medical College and Research Institute, Chennai, India; lllr Clinical Sciences Department, University of Sharjah, Sharjah, United Arab Emirates; llls Department of Pathology, Alexandria University, Alexandria, Egypt; lllt National Centre for Epidemiology and Population Health, Australian National University, Acton, ACT, Australia; lllu Statistics Discipline, Khulna University, Khulna, Bangladesh; lllv Department of Dermatology, Carol Davila University of Medicine and Pharmacy, Bucharest, Romania; lllw Department of Dermato-Venereology, Dr. Victor Babes Clinical Hospital of Infectious Diseases and Tropical Diseases, Bucharest, Romania; lllx Department of Epidemiology, Stellenbosch University, Cape Town, South Africa; llly Department of Medicine, Northlands Medical Group, Omuthiya, Namibia; lllz Department of Surgery, National University of Singapore, Singapore, Singapore; mmma State Key Laboratory of Numerical Modeling for Atmospheric Sciences and Geophysical Fluid Dynamics (LASG), Chinese Academy of Sciences, Beijing, China; mmmb Department of Radiology, University of Alabama at Birmingham, Birmingham, AL, USA; mmmc National Research and Innovation Agency, Jakarta, Indonesia; mmmd Department of Population and Behavioural Sciences, University of Health and Allied Sciences, Ho, Ghana; mmme School of Nursing and Public Health, University of KwaZulu-Natal, Durban, South Africa; mmmf Department of Economics, Rice University, Houston, TX, USA; mmmg Department of Research and Innovation, Enventure Medical Innovation, Houston, TX, USA; mmmh Department of Pathology, Tehran University of Medical Sciences, Tehran, Iran; mmmi Preventive Medicine and Public Health Research Center, Iran University of Medical Sciences, Tehran, Iran; mmmj Department of Community and Family Medicine, Iran University of Medical Sciences, Tehran, Iran; mmmk Department of Urology, Sabzevar University of Medical Sciences, Sabzevar, Iran; mmml Pediatric Intensive Care Unit, King Saud University, Riyadh, Saudi Arabia; mmmm College of Pharmacy, Alfaisal University, Riyadh, Saudi Arabia; mmmn Department of Epidemiology and Biostatistics, University of California San Francisco, San Francisco, CA, USA; mmmo Department of Medical Biochemistry and Molecular Biology, Mekelle University, Mekelle, Ethiopia; mmmp Department of Public Health, Wollega University, Nekemte, Ethiopia; mmmq School of Humanities and Social Sciences, Indian Institute of Technology Mandi, Mandi, India; mmmr Department of Pharmacology, All India Institute of Medical Sciences, Raipur, India; mmms Eastern Health Clinical School, Monash University, Melbourne, VIC, Australia; mmmt Department of Community Medicine, Manipal Academy of Higher Education, Mangalore, India; mmmu Institute of Applied Health Research, University of Birmingham, Birmingham, UK; mmmv Faculty of Medicine, University of Southampton, Southampton, UK; mmmw Department of Family and Preventive Medicine, Emory University, Atlanta, GA, USA; mmmx Department of Global Health, Sustainable Policy Solutions Foundation, South Yatta, VIC, Australia; mmmy Department of Gastroenterology, St. Luke's Hospital, Patanamthitta, India; mmmz Department of Medicine, Monash University, Melbourne, VIC, Australia; nnna Menzies Institute for Medical Research, University of Tasmania, Hobart, TAS, Australia; nnnb Faculty of Biomedical Engineering, Czech Technical University, Prague, Czech Republic; nnnc Faculty of Public Health, Universitas Sam Ratulangi (Sam Ratulangi University), Manado, Indonesia; nnnd Wollo University, Dessie, Ethiopia; nnne Department of Pharmacology, All India Institute of Medical Sciences, Jodhpur, India; nnnf Department of Epidemiology and Biostatistics, Birjand University of Medical Sciences, Birjand, Iran; nnng Department of Epidemiology and Biostatistics, Iran University of Medical Sciences, Tehran, Iran; nnnh Department of Medicine, University of Calgary, Calgary, AB, Canada; nnni Institute of Public Health, Jagiellonian University Medical College, Kraków, Poland; nnnj Agency for Health Technology Assessment and Tariff System, Warsaw, Poland; nnnk Saveetha Dental College and Hospitals, Saveetha University, Chennai, India; nnnl High Institute of Sport and Physical Education of Sfax, University of Sfax, Sfax, Tunisia; nnnm School of Biomedical Engineering, University of Technology Sydney, Sydney, NSW, Australia; nnnn Department of Internal Medicine, University of Medicine and Pharmacy at Ho Chi Minh City, Ho Chi Minh City, Vietnam; nnno Department of Business Analytics, University of Massachusetts Dartmouth, Dartmouth, MA, USA; nnnp Molecular Neuroscience Research Center, Shiga University of Medical Science, Shiga, Japan; nnnq Department of Clinical and Experimental Medicine, University of Pisa, Pisa, Italy; nnnr Department of Health Sciences, University of Leicester, Leicester, UK; nnns Adult Learning Disability Service, Leicestershire Partnership National Health Service Trust, Leicester, UK; nnnt Faculty of Medicine, Nam Can Tho University, Can Tho, Vietnam; nnnu School of Pharmacy, National Cheng Kung University, Tainan, Taiwan; nnnv Centre for Neonatal and Paediatric Infection, St George's University of London, London, UK; nnnw Department of Medicine, University of Crete, Heraklion, Greece; nnnx Department of Psychiatry, Dalhousie University, Halifax, NS, Canada; nnny Department of Public Health, Wachemo University, Hossana, Ethiopia; nnnz Department of Nutrition and Food Studies, George Mason University, Fairfax, VA, USA; oooa School of Nursing, Hong Kong Polytechnic University, Hong Kong, China; ooob Department of Biosciences and Biotechnology, University of Medical Sciences, Ondo, Ondo, Nigeria; oooc Faculty of Health and Life Sciences, University of Exeter, Exeter, UK; oood Natural and Medical Sciences Research Center, University of Nizwa, Nizwa, Oman; oooe Department of Zoology, University of Education Lahore, Lahore, Pakistan; ooof Division of Science and Technology, University of Education Lahore, Lahore, Pakistan; ooog Medical Genomics Research Department, King Abdullah International Medical Research Center, Riyadh, Saudi Arabia; oooh Department of Life Sciences, University of Management and Technology, Lahore, Pakistan; oooi Lahore Business School, The University of Lahore, Lahore, Pakistan; oooj Department of Cardiovascular, Endocrine-metabolic Diseases and Aging, National Institute of Health, Rome, Italy; oook Kasturba Medical College Mangalore, Manipal Academy of Higher Education, Manipal, India; oool Center for Neurodegenerative Diseases and the Aging Brain, University of Bari, Tricase, Italy; ooom Institute of Psychiatry, Psychology & Neuroscience, King’s College London, London, UK; ooon Department of Physiotherapy, Bayero University Kano, Kano, Nigeria; oooo Department of Rehabilitation Sciences, Hong Kong Polytechnic University, Hong Kong, China; ooop Unit of Internal Medicine, ARNAS Garibaldi Hospital, Catania, Italy; oooq Faculty of Medicine, Tehran University of Medical Sciences, Tehran, Iran; ooor Department of Informatics and Radiology, Mayo Clinic, Rochester, MN, USA; ooos College of Health and Sport Sciences, University of Bahrain, Zallaq, Bahrain; ooot Department of Clinical Biochemistry, Isfahan University of Medical Sciences, Isfahan, Iran; ooou Urmia University of Medical Sciences, Urmia, Iran; ooov Department of Cardiovascular Sciences, Katholieke Universiteit Leuven, Leuven, Belgium; ooow Department of Public Health and Epidemiology, University of Debrecen, Debrecen, Hungary; ooox Department of Pharmacology, All India Institute of Medical Sciences, Jodhpur, India; oooy UKK Institute, Tampere, Finland; oooz Faculty of Medicine and Health Technology, Tampere University, Tampere, Finland; pppa Department of Zoology, Central University of Punjab, Bathinda, India; pppb Department of Human Genetics & Molecular Biology, Bharathiar University, Coimbatore, India; pppc Raffles Neuroscience Centre, Raffles Hospital, Singapore, Singapore; pppd Yong Loo Lin School of Medicine, National University of Singapore, Singapore, Singapore; pppe Department of Community Medicine and Family Medicine, All India Institute of Medical Sciences, Bathinda, India; pppf Department of Medical and Surgical Sciences and Advanced Technologies "GF Ingrassia", University of Catania, Catania, Italy; pppg Department of Surgery, General University Hospital of Patras, Patras, Greece; ppph College of Medicine and Veterinary Medicine, University of Edinburgh, Edinburgh, UK; pppi Department of Health Policy and Management, Johns Hopkins University, Baltimore, MD, USA; pppj Department of Physiotherapy, Universidad Europea de Madrid (European University of Madrid), Villaviciosa de Odón, Spain; pppk Department of Public Health, Experimental and Forensic Medicine, University of Pavia, Pavia, Italy; pppl Department of Cardiology, Icahn School of Medicine at Mount Sinai, New York, NY, USA; pppm Division of Cardiology, Johns Hopkins University, Baltimore, MD, USA; pppn Department of Medical Oncology, University of Medicine and Pharmacy "Grigore T Popa" Iasi, Iaşi, Romania; pppo Department of Medical Oncology, Regional Institute of Oncology, Iaşi, Romania; pppp Department of Neurosurgery, Erasmus University Medical Center, Rotterdam, Netherlands; pppq Center for Experimental Microsurgery, Iuliu Hațieganu University of Medicine and Pharmacy, Cluj-Napoca, Romania; pppr School of Population Health and Environmental Sciences, King's College London, London, UK; ppps School of Health Sciences, National University of Sciences and Technology (NUST), Islamabad, Pakistan; pppt Gilbert and Rose-Marie Chagoury School of Medicine, Lebanese American University, Byblos, Lebanon; pppu Department of Interventional Radiology, University of Miami, Miami, FL, USA; pppv Brigham and Women's Hospital, Boston, MA, USA; pppw Department of Surgery, Washington University in St. Louis, St. Louis, MO, USA; pppx School of Public Health, Xuzhou Medical University, Xuzhou, China; pppy Department of Neurosurgery, Capital Medical University, Beijing, China; pppz Department of Neurosurgery, Beijing Tiantan Hospital, Beijing, China; qqqa Department of Gastroenterology, Shanghai Jiao Tong University Affiliated Sixth People's Hospital, Shanghai, China; qqqb School of Life Course and Population Sciences, King's College London, London, UK; qqqc Department of Psychiatry, University of São Paulo, São Paulo, Brazil; qqqd School of Medicine and Dentistry, Griffith University, Gold Coast, QLD, Australia; qqqe School of Nursing Sciences, University of Nairobi, Nairobi, Kenya; qqqf Key Laboratory of Computer-Aided Drug Design, Guangdong Medical University, Dongguan, China; qqqg Department of Biotechnology and Genetic Engineering, Hazara University Mansehra, Mansehra, Pakistan; qqqh Department of Human Nutrition and Food Sciences, Debre Markos University, Debre Markos, Ethiopia; qqqi Department of Epidemiology and Biostatistics, Bahir Dar University, Bahir Dar, Ethiopia; qqqj Department of Neurosurgery, Xijing Hospital, Xi'an, China; qqqk Cardiology Department, Royal Children's Hospital, Melbourne, VIC, Australia; qqql Department of Critical Care and Neurosciences, Murdoch Childrens Research Institute, Parkville, VIC, Australia; qqqm Department of Medical Laboratory Sciences, Adigrat University, Adigrat, Ethiopia; qqqn Department of Surgery, University of Colombo, Colombo, Sri Lanka; qqqo Department of Community Medicine, Rajarata University of Sri Lanka, Anuradhapura, Sri Lanka; qqqp Department of Neurology, University of Melbourne, Melbourne, VIC, Australia; qqqq Department of Medicine, University of Rajarata, Saliyapura Anuradhapuraya, Sri Lanka; qqqr Institute of Clinical Epidemiology, Public Health, Health Economics, Medical Statistics and Informatics, Medical University Innsbruck, Innsbruck, Austria; qqqs Department of Public Health and Primary Care, University of Cambridge, Cambridge, UK; qqqt School of Lifecourse and population sciences, King's College London, London, UK; qqqu NIHR Biomedical Research Centre, Guy's and St. Thomas' Hospital and Kings College London, London, UK; qqqv School of Pharmacy, Monash University, Subang Jaya, Malaysia; qqqw Global Health and Health Security Department, Taipei Medical University, Taipei, Taiwan; qqqx Global Health Research Center, Duke Kunshan University, Kunshan, China; qqqy Duke Global Health Institute, Duke University, Durham, NC, USA; qqqz Department of Food Science and Human Nutrition, Michigan State University, East Lansing, MI, USA; rrra Clinical Psychology Department, City Health Center, Suzhou, China; rrrb Division of Gastroenterology, Huazhong University of Science and Technology, Wuhan, China; rrrc School of Public Health, Zhejiang University, Zhejiang, China; rrrd Department of Public Health Science, Fred Hutchinson Cancer Research Center, Seattle, WA, USA; rrre Department of Endocrinology, University of Science and Technology of China, Hefei, China; rrrf School of Medicine, University of Rochester, Rochester, NY, USA; rrrg School of Population Health, University of New South Wales, Sydney, NSW, Australia; rrrh Cardiovascular Program, The George Institute for Global Health, Sydney, NSW, Australia; rrri Department of Public Health, Juntendo University, Tokyo, Japan; rrrj Department of Public Health Medicine, University of Tsukuba, Tsukuba, Japan; rrrk Department of Epidemiology, University of Florida, Gainesville, FL, USA; rrrl Faculty of Medicine, Juntendo University, Tokyo, Japan; rrrm Department of Medicine, Shiraz University of Medical Sciences, Shiraz, Iran; rrrn Department of Medicine, Mashhad University of Medical Sciences, Mashhad, Iran; rrro Research Center of Physiology, Semnan University of Medical Sciences, Semnan, Iran; rrrp Department of Biostatistics and Data Science, Graduate School of Medicine, Osaka University, Suita, Japan; rrrq Department of Public Health and Health Systems, Nagoya University, Nagoya, Japan; rrrr Department of Public Health and Health Systems, Nagoya University, Nagoya, Japan; rrrs Department of Radiology, University of Pennsylvania, Philadelphia, PA, USA; rrrt Department of Pediatric Allergy and Immunology, Tehran University of Medical Sciences, Tehran, Iran; rrru Digestive Diseases Research Institute, Tehran University of Medical Sciences, Tehran, Iran; rrrv National Center for Chronic and Noncommunicable Disease Control and Prevention, Chinese Center for Disease Control and Prevention, Beijing, China; rrrw The George Institute for Global Health, University of New South Wales, Sydney, NSW, Australia; rrrx Manipal College of Nursing, Manipal Academy of Higher Education, Udupi, India; rrry Biostatics, Epidemiology, and Science Computing Department, King Faisal Specialist Hospital & Research Center, Riyadh, Saudi Arabia; rrrz Saw Swee Hock School of Public Health, National University of Singapore, Singapore, Singapore; sssa KHANA Center for Population Health Research, Phnom Penh, Cambodia; sssb Department of Respiratory Medicine, Military Medical University, Chongqing, China; sssc Department of Epidemiology, Xuzhou Medical University, Xuzhou, China; sssd Department of Pediatrics, Kyung Hee University, Seoul, South Korea; ssse Department of Biostatistics, University of Toyama, Toyama, Japan; sssf Department of Public Health, Juntendo University, Tokyo, Japan; sssg Department of Epidemiology and Biostatistics, Wuhan University, Wuhan, China; sssh Department of Epidemiology, Vitalant Research Institute, San Francisco, CA, USA; sssi Department of Laboratory Medicine, University of California San Francisco, San Francisco, CA, USA; sssj The First Affiliated Hospital, China Medical University, Shenyang, China; sssk Department of Clinical Pharmacy and Pharmacy Administration, University of Maiduguri, Maiduguri, Nigeria; sssl Association for Socially Applicable Research (ASAR), Pune, India; sssm Department of Emergency Medicine, Global Emergency Medicine Innovation and Implementation (GEMINI) Research Center, Durham, NC, USA; sssn Department of Medical Genetics, Mashhad University of Medical Sciences, Mashhad, Iran; ssso Basic Sciences Department, University of Duhok, Duhok, Iraq; sssp Department of Health Sciences, James Madison University, Harrisonburg, VA, USA; sssq Sant'Elia Hospital, University of Catania, Caltanissetta, Italy; sssr Research and Development Department, Sina Medical Biochemistry Technologies, Shiraz, Iran; ssss Students Research Committee, Shahrekord University of Medical Sciences, Shahrekord, Iran; ssst Department of Radiology, Mayo Clinic, Rochester, MN, USA; sssu Department of Bioengineering and Therapeutical Sciences, University of California San Francisco, San Francisco, CA, USA; sssv Department of Administration, PGxAI, San Francisco, CA, USA; sssw School of Population and Global Health, The University of Western Australia, Perth, WA, Australia; sssx Institute of Applied Health Research, University of Birmingham, Birmingham, UK; sssy Department of Anesthesiology, Third Xiangya Hospital of Central South University, Changsha, China; sssz Department of Cardiology, Fudan University, Shanghai, China; ttta School of Public Health, Peking University, Beijing, China; tttb Department of International Health, Johns Hopkins University, Baltimore, MD, USA; tttc Medical Oncology Department of Gastrointestinal Cancer, Cancer Hospital of Dalian University of Technology, Shenyang, China; tttd School of Biomedical Engineering, Dalian University of Technology, Dalian, China; ttte School of Public Health, Wuhan University of Science and Technology, Wuhan, China; tttf Hubei Province Key Laboratory of Occupational Hazard Identification and Control, Wuhan University of Science and Technology, Wuhan, China; tttg Tianjin Medical University General Hospital, Tianjin Centers for Disease Control and Prevention, Tianjin, China; ttth College of Traditional Chinese Medicine, Hebei University, Baoding, China; ttti Jockey Club School of Public Health and Primary Care, The Chinese University of Hong Kong, Hong Kong, China; tttj School of Humanities and Management, Zhejiang Chinese Medical University, Hangzhou, China; tttk School of Public Health and Emergency Management, Southern University of Science and Technology, Shenzhen, China; tttl The Institute of Cancer Research, The Third Affiliated Hospital of Kunming Medical University, Kunming, China; tttm National Research Institute of Fundamental and Applied Medicine named after B. Atchabarov, Kazakh National Medical University, Almaty, Kazakhstan; tttn Eye Research Center, Iran University of Medical Sciences, Tehran, Iran; ttto Department of Biochemistry and Pharmacogenomics, Medical University of Warsaw, Warsaw, Poland; tttp Department of Anatomy, Addis Ababa University, Addis Ababa, Ethiopia; tttq Endocrinology and Metabolism Research Center, Hormozgan University of Medical Sciences, Bandar Abbas, Iran; tttr Department of Clinical and Community Pharmacy, An-Najah National University, Nablus, Palestine; ttts Clinical Research Centre, An-Najah National University, Nablus, Palestine; tttt Center for Medical and Bio-Allied Health Sciences Research, Ajman University, Ajman, United Arab Emirates; tttu Institute for Health Metrics and Evaluation, University of Washington, Seattle, WA, USA; tttv Division of Cardiology, University of Washington, Seattle, WA, USA; tttw Institute for Health Metrics and Evaluation, University of Washington, Seattle, WA, USA; tttx Department of Health Metrics Sciences, School of Medicine, University of Washington, Seattle, WA, USA; ttty National Institute for Stroke and Applied Neurosciences, Auckland University of Technology, Auckland, New Zealand; tttz Department of Neurosurgery, Helsinki University Hospital, Helsinki, Finland; uuua National Institute for Stroke and Applied Neurosciences, Auckland University of Technology, Auckland, New Zealand; uuub National Institute for Stroke and Applied Neurosciences, Auckland University of Technology, Auckland, New Zealand; uuuc Institute for Health Metrics and Evaluation, University of Washington, Seattle, WA, USA; uuud Institute for Health Metrics and Evaluation, University of Washington, Seattle, WA, USA; uuue Department of Health Metrics Sciences, School of Medicine, University of Washington, Seattle, WA, USA; uuuf Institute for Health Metrics and Evaluation, University of Washington, Seattle, WA, USA; uuug Department of Health Metrics Sciences, School of Medicine, University of Washington, Seattle, WA, USA

## Abstract

**Background:**

Up-to-date estimates of stroke burden and attributable risks and their trends at global, regional, and national levels are essential for evidence-based health care, prevention, and resource allocation planning. We aimed to provide such estimates for the period 1990–2021.

**Methods:**

We estimated incidence, prevalence, death, and disability-adjusted life-year (DALY) counts and age-standardised rates per 100 000 people per year for overall stroke, ischaemic stroke, intracerebral haemorrhage, and subarachnoid haemorrhage, for 204 countries and territories from 1990 to 2021. We also calculated burden of stroke attributable to 23 risk factors and six risk clusters (air pollution, tobacco smoking, behavioural, dietary, environmental, and metabolic risks) at the global and regional levels (21 GBD regions and Socio-demographic Index [SDI] quintiles), using the standard GBD methodology. 95% uncertainty intervals (UIs) for each individual future estimate were derived from the 2·5th and 97·5th percentiles of distributions generated from propagating 500 draws through the multistage computational pipeline.

**Findings:**

In 2021, stroke was the third most common GBD level 3 cause of death (7·3 million [95% UI 6·6–7·8] deaths; 10·7% [9·8–11·3] of all deaths) after ischaemic heart disease and COVID-19, and the fourth most common cause of DALYs (160·5 million [147·8–171·6] DALYs; 5·6% [5·0–6·1] of all DALYs). In 2021, there were 93·8 million (89·0–99·3) prevalent and 11·9 million (10·7–13·2) incident strokes. We found disparities in stroke burden and risk factors by GBD region, country or territory, and SDI, as well as a stagnation in the reduction of incidence from 2015 onwards, and even some increases in the stroke incidence, death, prevalence, and DALY rates in southeast Asia, east Asia, and Oceania, countries with lower SDI, and people younger than 70 years. Globally, ischaemic stroke constituted 65·3% (62·4–67·7), intracerebral haemorrhage constituted 28·8% (28·3–28·8), and subarachnoid haemorrhage constituted 5·8% (5·7–6·0) of incident strokes. There were substantial increases in DALYs attributable to high BMI (88·2% [53·4–117·7]), high ambient temperature (72·4% [51·1 to 179·5]), high fasting plasma glucose (32·1% [26·7–38·1]), diet high in sugar-sweetened beverages (23·4% [12·7–35·7]), low physical activity (11·3% [1·8–34·9]), high systolic blood pressure (6·7% [2·5–11·6]), lead exposure (6·5% [4·5–11·2]), and diet low in omega-6 polyunsaturated fatty acids (5·3% [0·5–10·5]).

**Interpretation:**

Stroke burden has increased from 1990 to 2021, and the contribution of several risk factors has also increased. Effective, accessible, and affordable measures to improve stroke surveillance, prevention (with the emphasis on blood pressure, lifestyle, and environmental factors), acute care, and rehabilitation need to be urgently implemented across all countries to reduce stroke burden.

**Funding:**

Bill & Melinda Gates Foundation.

## Introduction

Evidence from the Global Burden of Disease, Injuries, and Risk Factors Study (GBD) suggests that prevalent cases of total cardiovascular disease (including stroke) nearly doubled from 271 million (95% uncertainty interval [UI] 257–285) in 1990 to 523 million (497–550) in 2019.^1^ Moreover, despite a consistent decline in age-standardised cardiovascular disease (including stroke) mortality rates globally in the second half of the 20th century,^1^ there has been a subsequent deceleration in the decline and an overall flattening of the decline in the past few years.^1^ Since 2010, age-standardised cardiovascular disease (including stroke) mortality rates have even increased in many locations (eg, Mexico, the UK, and the USA),^1^, ^2^ and the age-standardised incidence of stroke in individuals younger than 55 years has increased substantially in high-income countries.^3^, ^4^ The previous GBD study on stroke burden and risks covered the period 1990–2019, and identified stroke as the second leading cause of death in the world.^5^ The most recent GBD stroke burden project^6^ has estimated an almost doubling of disability-adjusted life-years (DALYs), deaths, and cost due to stroke from 2020 to 2050.^6^ Globally, the age-standardised prevalence of cardiovascular disease (including stroke) risk factors (including hypertension, overweight, and diabetes)^1^ are also increasing.^7^ There has been a rapid increase in the number of people who died or remained disabled from stroke over the past 30 years,^5^ with a trend towards increasing incidence rates in people younger than 55 years, and increased prevalence of major risk factors for stroke (elevated blood pressure, overweight, and diabetes) over the past 10–15 years. These findings necessitate timely updated data on the most recent changes in stroke burden and risks across the globe to inform adequate health-care planning, resource allocation, and priority setting for stroke and to assess the success or failure of measures to reduce stroke burden.

**Research in context**

**Evidence before this study**

The Global Burden of Diseases, Injuries, and Risk Factors Study (GBD) is the only global epidemiological study that produces comprehensive estimates of global, regional, and country-specific burden due to stroke. To evaluate the availability of evidence, we carried out a structured review of the published scientific literature in MEDLINE, Scopus, Google Scholar, and PubMed for relevant reports published in any language from Jan 1, 1990, to March 1, 2024, using search terms that included “stroke”, “cerebral infarction”, “isch(a)emic stroke”, “intracerebral h(a)emorrage”, “h(a)emorrhagic stroke”, or “subarachnoid h(a)emorrage”, AND “incidence”, “prevalence”, “mortality”, or “epidemiology” or “population attributable fraction (PAF)”, “risk factor(s)”, “trends”, or “disability-adjusted life-year(s) (DALYs)”. The most recent GBD report on the burden of stroke and its risk factors covered the period from 1990 to 2019 and found that the annual number of strokes and deaths due to stroke increased substantially, despite large reductions in age-standardised rates, particularly reductions among people aged 70 years or older. The highest age-standardised stroke-related mortality and DALY rates were in the World Bank low-income group, and the fastest growing risk factor for stroke between 1990 and 2019 was high BMI.

**Added value of this study**

As part of GBD 2021, this study provides the most up-to-date estimates of the burden of overall stroke, ischaemic stroke, intracerebral haemorrhage, and subarachnoid haemorrhage and its risk factors. We found that stroke burden, in terms of absolute numbers, has increased substantially from 1990 to 2021. From 1990 to 2021, there was an increase in the contribution to stroke DALYs from not only high BMI, as in the previous GBD 2019 study, but also high ambient temperature, high fasting plasma glucose, diet high in sugar-sweetened beverages, low physical activity, high systolic blood pressure, and diet low in omega-6 polyunsaturated fatty acids, emphasising the increasing role of environmental factors on the heightened burden from stroke. Stroke burden was highest in low-income and middle-income countries.

**Implications of all the available evidence**

The findings from this study can help to guide evidence-based health-care planning, prevention, and resource allocation for stroke and its pathological types, including country-specific prioritisation of these measures. Effective, accessible, and affordable measures to improve stroke surveillance, prevention (with the emphasis on elevated blood pressure, lifestyle, and environmental factors), acute care, and rehabilitation to reduce stroke burden need to be urgently implemented across all countries.

The current GBD 2021 study of stroke burden and risks covers the period from 1990 to 2021. It includes analysis of the additional data sources for 2019–21, with corresponding re-calculation of all previous stroke burden and risks estimates, including stroke incidence, prevalence, deaths, and DALYs for total stroke and its three main pathological types (ischaemic stroke, intracerebral haemorrhage, and subarachnoid haemorrhage). It also includes analysis of DALYs due to stroke and stroke pathological type attributable to 23 risk factors and six risk factor clusters at global, regional, and national (204 countries and territories) levels. This manuscript was produced as part of the GBD Collaborator Network and in accordance with the GBD Protocol.

## Methods

### Overview

Details of the GBD 2021 methods for stroke burden and risk factors estimates remained the same as for the latest GBD estimates and are described elsewhere^8^, ^9^, ^10^ (appendix pp 61–99). Stroke was defined according to the clinical WHO criteria^11^ and categorised into three pathological types (ischaemic stroke, intracerebral haemorrhage, and subarachnoid haemorrhage).^12^ To simplify the stroke modelling process and to ensure that all major pathological types were estimated correctly, vital registration and surveillance data were used to separately produce independent acute and chronic stroke models for ischaemic stroke, intracerebral haemorrhage, and subarachnoid haemorrhage type (appendix pp 75–76). As in previous GBD stroke burden estimates, we modelled first-ever-in-a-lifetime ischaemic stroke, intracerebral haemorrhage, and subarachnoid haemorrhage from the day of stroke onset to 28 days, and separately modelled survival (prevalence) beyond 28 days.^5^

Cause of Death Ensemble modelling (CODEm) was used to estimate deaths due to overall stroke and stroke pathological types. For non-fatal disease modelling (incidence and prevalence of stroke), we used the DisMod-MR 2.1 tool,^13^ a Bayesian modelling software that uses data on various disease parameters and the epidemiological relationships between these parameters.^5^ In the GBD study, the incidence rate represents new events in a given year, whereas the death rate represents those that occurred in that year regardless of when the stroke occurred.

We used data from 3736 vital registration sources, 147 verbal autopsy sources, 368 incidence sources, 346 prevalence sources, 229 excess mortality sources, 7753 risk factor exposure sources, and 2733 risk factor relative risk sources. Further details of the data sources used in this analysis are available on the GBD 2021 Sources Tool website.

Stroke incidence, mortality, prevalence, and DALY estimates are presented in absolute numbers and as age-standardised rates per 100 000 population (with 95% UIs) and are stratified by age, sex, 21 GBD regions, and seven GBD super-regions (appendix pp 202–203). Countries and territories were also grouped into quintiles of high, high-middle, middle, low-middle, and low Socio-demographic Index (SDI; a summary indicator of geometric mean of normalised values of a location's lag-distributed income per capita, the average years of schooling in the population aged 15 years or older, and the total fertility rate in females younger than 25 years),^14^ on the basis of their 2021 values. Expressed on a scale from 0 to 1, a location with an SDI of 0 would have a theoretical minimum level of development relevant to health, whereas a location with an SDI of 1 would have a theoretical maximum level.

Count data in tables are rounded to the nearest thousand or, when the count is less than 1000, to the nearest 10. Uncertainty was propagated throughout all of these calculations by creating 500 values for each incidence, prevalence, death, or DALY estimate and performing aggregations across causes and locations at the level of each of the 500 values for all intermediate steps in the calculation. The lower and upper bounds of the 95% UI are the 2·5th and 97·5th percentiles.

### Attributable burden of stroke due to risk factors

To analyse the attributable burden of stroke and its three pathological types due to 23 risk factors currently available for such analysis in GBD 2021, we calculated population attributable fractions (PAFs) of DALYs (appendix pp 31–43), using the exposure level for each risk factor and theoretical minimum risk exposure level (TMREL) that minimises risk for each individual in the population as the reference variable.^9^ We analysed data on the prevalence of exposure to a risk and derived relative risks for any risk–outcome pair for which we found sufficient evidence of a causal relationship.^15^ Adjustments for mediation were applied to account for relationships involving risk factors that act indirectly on outcomes via intermediate risks, as described elsewhere.^9^ Relative risk data were pooled using meta-regression of cohort, case–control, or intervention studies. From the prevalence and relative risk results, PAFs were estimated relative to the TMREL. The PAF represents a proportion of the stroke DALYs that would be decreased if the exposure to the risk factor in the past had been at the counterfactual level of the TMREL.

The risks included in the analysis were ambient particulate matter pollution; household air pollution from solid fuels; low ambient temperature (daily temperatures below the TMREL); high ambient temperature (daily temperatures above the TMREL); lead exposure; diet high in sodium; diet high in red meat; diet high in processed meat; diet low in fruits; diet low in vegetables; diet low in wholegrains; alcohol use (any alcohol dosage consumption); diet high in sugar-sweetened beverages; diet low in fibre; diet low in omega-6 polyunsaturated fatty acids; low physical activity (only for ischaemic stroke burden); smoking; second-hand smoke; high BMI; high fasting plasma glucose; high systolic blood pressure; high LDL cholesterol (only for ischaemic stroke burden); and kidney dysfunction, as measured by low glomerular filtration rate (not assessed for subarachnoid haemorrhage burden). We set the TMREL to zero for all harmful dietary risk factors with monotonically increasing risk functions (eg, processed meat intake), excluding sodium. For protective risks with monotonically declining risk functions with exposure (eg, fruit intake), we first determined the 85th percentile of exposure in the cohorts or trials used in the meta-regression of each outcome that was associated with the risk. Then, we determined the TMREL by weighting each risk–outcome pair by the relative global magnitude of each outcome.^5^

As with causes, GBD organises risk factors into four levels, from the broadest (level 1: environmental risks, behavioural risks, and metabolic risks) to the most specific (level 4; 23 individual risk factors). The PAFs of risk factor groups took into account mediation between risk factors included in the group, as explained elsewhere.^16^ Percentages and number of DALYs are not mutually exclusive. The crude sum of the PAF of the risk factors might exceed 100% because the effects of many of these risk factors are mediated partly or wholly through another risk factor or risk factors.^5^ Definitions of risk factors and risk groups and further details of risk factors are in the appendix (pp 31–43). Changes in the modelling of stroke for GBD 2021 are presented in the appendix (pp 44–47). Analyses were also done by cluster of risk factors. The air pollution cluster includes ambient PM_2·5_ pollution and household air pollution. The behavioural risks cluster includes smoking (including second-hand smoking), dietary risks (diet high in sodium, diet high in processed meat diet, high in red meat, diet high in sugar-sweetened beverages, diet low in omega-6 polyunsaturated fatty acids, diet low in fruits, diet low in vegetables, and diet low in wholegrains), alcohol use, and low physical activity. The dietary risks cluster includes diet high in sodium, diet high in processed meat, diet high in red meat, diet high in sugar-sweetened beverages, diet low in omega-6 polyunsaturated fatty acids, diet low in fruits, diet low in vegetables, and diet low in wholegrains. The environmental risks cluster includes the air pollution cluster, low ambient temperature, high ambient temperature, and lead exposure. The metabolic risks cluster includes high fasting plasma glucose, high LDL cholesterol, high systolic blood pressure, high BMI, and kidney dysfunction. Finally, the tobacco smoke cluster includes smoking and second-hand smoking.

### Role of the funding source

The funder of the study had no role in study design, data collection, data analysis, data interpretation, or the writing of the report.

## Results

### Stroke incidence, prevalence, death, and DALYs by geographical location

In 2021, there were 93·8 million (95% UI 89·0–99·3) stroke survivors, 11·9 million (10·7–13·2) new stroke events, 7·3 million (6·6–7·8) deaths from stroke, and 160·5 million (147·8–171·6) DALYs from stroke, comprising 10·7% (9·8–11·3) of all deaths and 5·6% (5·0–6·1) of all DALYs from all causes, the third leading cause of deaths (after ischaemic heart disease and COVID-19) and the fourth leading cause of DALYs (after COVID-19, ischaemic heart disease, and neonatal disorders; table 1; appendix p 204).

**Table 1:** Incident cases, deaths, prevalent cases, and DALYs for stroke in 2021 and percentage change in age-standardised rates for 1990–2021, by location, for both sexes

			**Incident cases**		**Deaths**		**Prevalent cases**		**DALYs**	
			Counts, 2021	Percentage change in age-standardised rates, 1990–2021	Counts, 2021	Percentage change in age-standardised rates, 1990–2021	Counts, 2021	Percentage change in age-standardised rates, 1990–2021	Counts, 2021	Percentage change in age-standardised rates, 1990–2021
**World Bank income level**										
Global			11 946 000 (10 772 000 to 13 220 000)	−21·8% (−23·7 to −19·8)	7 253 000 (6 567 000 to 7 808 000)	−39·4% (−44·0 to −34·6)	93 816 000 (89 030 000 to 99 335 000)	−8·5% (−9·7 to −7·3)	160 457 000 (147 781 000 to 171 643 000)	−38·7% (−43·4 to −34·0)
High income			1 994 000 (1 822 000 to 2 173 000)	−41·0% (−43·0 to −39·0)	930 000 (791 000 to 1 002 000)	−62·2% (−64·2 to −61·1)	21 889 000 (21 018 000 to 22 893 000)	−15·6% (−17·3 to −13·8)	16 980 000 (15 364 000 to 18 218 000)	−58·0% (−59·6 to −56·5)
Upper-middle income			5 680 000 (5 053 000 to 6 432 000)	−17·9% (−21·2 to −14·4)	3 557 000 (3 113 000 to 4 005 000)	−43·4% (−50·5 to −35·1)	38 997 000 (36 331 000 to 41 833 000)	−1·6% (−3·6 to 0·5)	73 780 000 (65 305 000 to 82 892 000)	−45·1% (−51·7 to −37·4)
Lower-middle income			3 702 000 (3 374 000 to 4 043 000)	−19·7% (−21·8 to −17·4)	2 410 000 (2 225 000 to 2 592 000)	−26·3% (−32·1 to −18·6)	28 336 000 (26 853 000 to 30 089 000)	−8·9% (−10·0 to −7·7)	60 018 000 (55 442 000 to 64 220 000)	−27·7% (−33·3 to −20·5)
Low income			561 000 (518 000 to 605 000)	−19·6% (−21·8 to −16·9)	349 000 (302 000 to 397 000)	−28·2% (−35·8 to −19·9)	4 520 000 (4 365 000 to 4 673 000)	−13·8% (−15·1 to −12·6)	9 596 000 (8 261 000 to 10 976 000)	−30·6% (−38·5 to −22·2)
**SDI level**										
High SDI			1 800 000 (1 632 000 to 1 981 000)	−37·3% (−39·0 to −35·4)	798 000 (683 000 to 860 000)	−59·4% (−61·6 to −57·7)	20 249 000 (19 375 000 to 21 279 000)	−13·4% (−15·1 to −11·5)	15 221 000 (13 730 000 to 16 390 000)	−54·5% (−56·6 to −52·5)
High-middle SDI			3 094 000 (2 748 000 to 3 480 000)	−25·6% (−27·7 to −23·3)	1 942 000 (1 726 000 to 2 138 000)	−46·9% (−52·0 to −41·5)	21 406 000 (20 065 000 to 22 783 000)	−8·4% (−10·4 to −6·3)	38 405 000 (34 662 000 to 42 300 000)	−46·7% (−51·9 to −41·3)
Middle SDI			4 215 000 (3 795 000 to 4 707 000)	−14·2% (−17·2 to −10·9)	2 681 000 (2 384 000 to 2 946 000)	−37·2% (−44·0 to −28·6)	30 207 000 (28 379 000 to 32 296 000)	−2·0% (−3·6 to −0·3)	59 875 000 (54 006 000 to 65 175 000)	−39·3% (−45·6 to −31·4)
Low-middle SDI			2 029 000 (1 855 000 to 2 209 000)	−16·8% (−18·9 to −14·4)	1 349 000 (1 240 000 to 1 454 000)	−23·6% (−29·9 to −15·2)	15 293 000 (14 510 000 to 16 186 000)	−6·5% (−7·8 to −5·3)	33 705 000 (30 995 000 to 36 498 000)	−26·4% (−32·5 to −18·7)
Low SDI			799 000 (737 000 to 866 000)	−21·8% (−23·8 to −19·6)	476 000 (425 000 to 528 000)	−26·5% (−33·6 to −18·1)	6 588 000 (6 323 000 to 6 864 000)	−13·6% (−14·9 to −12·4)	13 105 000 (11 572 000 to 14 675 000)	−29·5% (−37·0 to −21·1)
**GBD super-regions, regions, and countries and territories**										
Central Europe, eastern Europe, and central Asia			1 078 000 (968 000 to 1 198 000)	−28·4% (−30·5 to −26·3)	725 000 (669 000 to 769 000)	−45·7% (−48·2 to −43·0)	6 643 000 (6 249 000 to 7 041 000)	−13·7% (−15·6 to −11·7)	13 875 000 (12 992 000 to 14 683 000)	−43·4% (−46·1 to −40·6)
	Central Asia		166 000 (154 000 to 178 000)	−8·8% (−12·1 to −5·4)	84 000 (76 000 to 92 000)	−22·2% (−28·2 to −15·3)	1 119 000 (1 082 000 to 1 160 000)	−11·7% (−13·1 to −10·0)	1 996 000 (1 809 000 to 2 181 000)	−26·8% (−32·5 to −20·1)
		Armenia	5000 (4000 to 5000)	−40·7% (−44·2 to −36·7)	3000 (2000 to 3000)	−45·0% (−50·8 to −38·4)	41 000 (39 000 to 42 000)	−16·6% (−19·3 to −13·6)	56 000 (50 000 to 62 000)	−45·3% (−50·8 to −39·0)
		Azerbaijan	17 000 (16 000 to 19 000)	6·1% (0·6 to 12·3)	8000 (7000 to 10 000)	−20·4% (−34·1 to −2·6)	110 000 (106 000 to 115 000)	−6·3% (−9·3 to −3·4)	187 000 (153 000 to 226 000)	−27·4% (−40·7 to −10·5)
		Georgia	13 000 (12 000 to 14 000)	−16·6% (−21·5 to −11·1)	10 000 (9000 to 11 000)	−17·4% (−26·3 to −7·9)	68 000 (65 000 to 71 000)	−5·7% (−8·3 to −3·0)	184 000 (164 000 to 205 000)	−23·2% (−32·1 to −12·9)
		Kazakhstan	37 000 (34 000 to 41 000)	−18·4% (−23·9 to −12·9)	23 000 (20 000 to 26 000)	−10·0% (−21·2 to 3·0)	275 000 (265 000 to 287 000)	−20·1% (−22·9 to −17·2)	509 000 (441 000 to 576 000)	−19·3% (−29·4 to −7·5)
		Kyrgyzstan	8000 (7000 to 9000)	−38·2% (−42·1 to −33·6)	4000 (3000 to 5000)	−53·3% (−60·5 to −45·7)	56 000 (54 000 to 58 000)	−31·8% (−34·1 to −29·5)	108 000 (92 000 to 126 000)	−49·2% (−57·4 to −40·9)
		Mongolia	6000 (5000 to 6000)	0·5% (−4·1 to 5·4)	3000 (2000 to 3000)	−37·6% (−51·4 to −22·5)	36 000 (35 000 to 37 000)	−0·8% (−3·3 to 1·8)	72 000 (60 000 to 86 000)	−37·4% (−50·4 to −22·3)
		Tajikistan	12 000 (11 000 to 13 000)	11·9% (6·0 to 18·6)	6000 (4000 to 7000)	−19·0% (−36·2 to 1·9)	71 000 (68 000 to 74 000)	−8·3% (−11·0 to −5·4)	141 000 (112 000 to 170 000)	−25·8% (−41·2 to −7·3)
		Turkmenistan	9000 (9000 to 10 000)	5·9% (−0·5 to 11·6)	6000 (5000 to 7000)	21·6% (−2·7 to 50·7)	70 000 (68 000 to 73 000)	18·5% (14·9 to 22·7)	158 000 (126 000 to 193 000)	21·4% (−3·4 to 49·9)
		Uzbekistan	60 000 (55 000 to 65 000)	9·9% (3·9 to 16·5)	22 000 (19 000 to 26 000)	−20·8% (−31·3 to −7·8)	392 000 (377 000 to 407 000)	−3·2% (−6·7 to 1·0)	581 000 (505 000 to 672 000)	−28·0% (−37·7 to −16·4)
	Central Europe		302 000 (273 000 to 328 000)	−36·6% (−38·3 to −35·1)	215 000 (196 000 to 230 000)	−55·3% (−58·2 to −52·5)	1 891 000 (1 797 000 to 1 996 000)	−22·8% (−24·5 to −21·3)	3 740 000 (3 455 000 to 3 993 000)	−55·5% (−58·5 to −52·7)
		Albania	6000 (6000 to 7000)	−14·0% (−17·5 to −10·7)	6000 (5000 to 7000)	−25·1% (−40·0 to −8·3)	31 000 (30 000 to 33 000)	−15·4% (−17·6 to −12·9)	97 000 (79 000 to 116 000)	−34·6% (−47·6 to −19·8)
		Bosnia and Herzegovina	10 000 (9000 to 11 000)	−17·1% (−23·2 to −10·6)	7000 (6000 to 8000)	−32·8% (−46·2 to −18·9)	71 000 (68 000 to 74 000)	−5·2% (−8·6 to −1·4)	127 000 (103 000 to 148 000)	−36·6% (−48·8 to −24·1)
		Bulgaria	31 000 (28 000 to 34 000)	−20·9% (−25·3 to −15·9)	28 000 (25 000 to 32 000)	−37·1% (−44·2 to −29·5)	159 000 (146 000 to 173 000)	−11·3% (−16·6 to −6·4)	484 000 (424 000 to 551 000)	−37·8% (−45·4 to −29·0)
		Croatia	11 000 (10 000 to 12 000)	−43·1% (−46·0 to −40·6)	7000 (6000 to 8000)	−66·2% (−69·9 to −62·3)	72 000 (70 000 to 75 000)	−20·6% (−25·0 to −16·0)	110 000 (98 000 to 123 000)	−66·9% (−70·3 to −62·9)
		Czechia	23 000 (20 000 to 26 000)	−51·8% (−55·6 to −47·7)	10 000 (8000 to 11 000)	−79·2% (−81·4 to −76·8)	184 000 (178 000 to 192 000)	−26·1% (−29·4 to −22·4)	177 000 (156 000 to 197 000)	−76·8% (−79·3 to −74·0)
		Hungary	22 000 (20 000 to 25 000)	−51·7% (−54·7 to −48·0)	12 000 (10 000 to 13 000)	−70·7% (−74·5 to −66·9)	162 000 (155 000 to 169 000)	−39·3% (−41·4 to −36·9)	223 000 (195 000 to 251 000)	−69·0% (−72·8 to −65·2)
		Montenegro	2000 (2000 to 2000)	−6·6% (−10·4 to −2·5)	2000 (2000 to 2000)	22·6% (2·8 to 43·3)	8000 (7000 to 8000)	−10·7% (−13·1 to −7·9)	32 000 (27 000 to 37 000)	1·3% (−16·1 to 19·9)
		North Macedonia	7000 (7000 to 8000)	−8·6% (−14·5 to −2·2)	7000 (5000 to 8000)	−1·8% (−18·1 to 15·4)	40 000 (36 000 to 43 000)	−18·8% (−23·4 to −13·6)	120 000 (98 000 to 142 000)	−20·1% (−34·3 to −6·3)
		Poland	72 000 (62 000 to 83 000)	−35·1% (−37·8 to −32·0)	45 000 (40 000 to 49 000)	−65·5% (−68·2 to −62·8)	485 000 (441 000 to 535 000)	−16·8% (−20·1 to −13·6)	800 000 (726 000 to 876 000)	−63·9% (−66·6 to −60·9)
		Romania	64 000 (58 000 to 70 000)	−33·4% (−37·5 to −29·0)	53 000 (47 000 to 59 000)	−45·5% (−51·4 to −39·7)	369 000 (351 000 to 387 000)	−17·8% (−21·8 to −13·0)	903 000 (810 000 to 999 000)	−44·8% (−50·6 to −38·6)
		Serbia	32 000 (29 000 to 35 000)	−26·9% (−31·5 to −22·3)	28 000 (24 000 to 33 000)	−49·6% (−57·8 to −39·8)	152 000 (141 000 to 165 000)	−25·9% (−30·2 to −21·6)	458 000 (388 000 to 532 000)	−50·2% (−58·1 to −40·9)
		Slovakia	13 000 (11 000 to 14 000)	−37·2% (−41·9 to −32·1)	6000 (5000 to 7000)	−54·3% (−62·0 to −44·1)	106 000 (102 000 to 111 000)	−27·0% (−29·2 to −24·6)	127 000 (108 000 to 147 000)	−54·7% (−61·8 to −45·1)
		Slovenia	3000 (3000 to 4000)	−56·1% (−59·2 to −52·9)	2000 (2000 to 2000)	−69·5% (−73·2 to −66·2)	24 000 (23 000 to 25 000)	−30·8% (−34·7 to −26·5)	29 000 (25 000 to 32 000)	−71·8% (−74·8 to −68·7)
	Eastern Europe		610 000 (536 000 to 693 000)	−27·9% (−30·8 to −25·0)	426 000 (389 000 to 460 000)	−43·3% (−46·8 to −39·5)	3 633 000 (3 342 000 to 3 928 000)	−11·1% (−13·6 to −8·2)	8 139 000 (7 532 000 to 8 761 000)	−39·4% (−43·3 to −35·4)
		Belarus	27 000 (24 000 to 30 000)	−24·2% (−29·6 to −19·0)	16 000 (13 000 to 19 000)	−26·7% (−38·1 to −13·8)	177 000 (168 000 to 186 000)	−10·0% (−16·1 to −2·7)	322 000 (270 000 to 376 000)	−28·4% (−39·3 to −15·4)
		Estonia	2000 (2000 to 3000)	−58·6% (−62·1 to −54·8)	1000 (1000 to 1000)	−79·0% (−81·6 to −76·7)	18 000 (17 000 to 18 000)	−30·5% (−34·9 to −26·2)	21 000 (18 000 to 23 000)	−76·7% (−79·5 to −74·2)
		Latvia	7000 (6000 to 7000)	−38·3% (−42·3 to −34·2)	5000 (4000 to 6000)	−46·9% (−52·4 to −41·3)	39 000 (37 000 to 41 000)	−11·6% (−17·0 to −4·9)	81 000 (72 000 to 90 000)	−48·6% (−53·9 to −43·6)
		Lithuania	10 000 (9000 to 11 000)	−28·2% (−34·0 to −22·9)	5000 (4000 to 5000)	−30·2% (−38·3 to −23·3)	50 000 (44 000 to 56 000)	−6·7% (−16·7 to 3·9)	79 000 (70 000 to 87 000)	−35·9% (−43·2 to −29·5)
		Moldova	9000 (8000 to 10 000)	−31·9% (−36·5 to −27·0)	5000 (5000 to 6000)	−52·1% (−56·4 to −47·1)	53 000 (50 000 to 55 000)	−9·1% (−12·6 to −5·4)	112 000 (102 000 to 124 000)	−46·8% (−51·7 to −41·3)
		Russia	422 000 (368 000 to 481 000)	−27·0% (−30·1 to −23·8)	311 000 (285 000 to 335 000)	−43·9% (−47·5 to −40·5)	2 454 000 (2 247 000 to 2 670 000)	−9·5% (−12·5 to −6·3)	5 892 000 (5 460 000 to 6 339 000)	−40·3% (−44·0 to −36·4)
		Ukraine	134 000 (116 000 to 153 000)	−29·7% (−33·8 to −25·4)	82 000 (64 000 to 102 000)	−45·0% (−56·6 to −31·4)	844 000 (763 000 to 925 000)	−13·5% (−18·9 to −8·3)	1 632 000 (1 276 000 to 2 023 000)	−38·6% (−51·6 to −24·1)
High income			1 711 000 (1 563 000 to 1 865 000)	−41·6% (−43·7 to −39·3)	772 000 (643 000 to 838 000)	−62·3% (−64·4 to −61·1)	19 822 000 (19 019 000 to 20 724 000)	−15·9% (−17·6 to −14·0)	13 972 000 (12 517 000 to 15 091 000)	−57·7% (−59·3 to −56·2)
	Australasia		36 000 (33 000 to 40 000)	−40·7% (−43·8 to −38·0)	15 000 (12 000 to 16 000)	−63·9% (−66·4 to −61·5)	368 000 (357 000 to 380 000)	−21·5% (−23·2 to −19·8)	250 000 (221 000 to 273 000)	−60·8% (−63·1 to −58·5)
		Australia	31 000 (28 000 to 34 000)	−40·6% (−44·2 to −37·0)	12 000 (10 000 to 13 000)	−65·3% (−67·9 to −62·8)	313 000 (305 000 to 322 000)	−21·5% (−23·2 to −19·9)	205 000 (182 000 to 224 000)	−61·6% (−63·9 to −59·2)
		New Zealand	6000 (5000 to 6000)	−41·3% (−45·2 to −37·2)	3000 (2000 to 3000)	−55·5% (−58·9 to −52·4)	55 000 (50 000 to 60 000)	−21·9% (−26·6 to −17·2)	45 000 (40 000 to 49 000)	−56·6% (−59·4 to −53·8)
	High-income Asia Pacific		408 000 (372 000 to 446 000)	−46·2% (−49·0 to −43·3)	185 000 (147 000 to 207 000)	−71·2% (−73·3 to −69·6)	4 793 000 (4 539 000 to 5 071 000)	−24·1% (−26·0 to −21·6)	3 386 000 (2 955 000 to 3 726 000)	−65·5% (−67·5 to −63·6)
		Brunei	510 (460 to 560)	−45·4% (−48·5 to −41·9)	170 (150 to 200)	−51·1% (−58·5 to −40·6)	5000 (5000 to 5000)	−39·6% (−41·1 to −37·9)	5000 (4000 to 6000)	−53·0% (−60·1 to −43·3)
		Japan	305 000 (276 000 to 336 000)	−37·7% (−41·7 to −32·8)	147 000 (115 000 to 165 000)	−67·1% (−69·0 to −65·6)	3 607 000 (3 367 000 to 3 852 000)	−14·1% (−16·8 to −10·5)	2 575 000 (2 246 000 to 2 840 000)	−57·6% (−59·6 to −55·8)
		Singapore	7000 (6000 to 7000)	−63·1% (−66·0 to −60·2)	1000 (1000 to 1000)	−84·3% (−85·8 to −83·0)	83 000 (80 000 to 86 000)	−48·6% (−50·1 to −46·8)	36 000 (31 000 to 40 000)	−79·6% (−81·3 to −78·0)
		South Korea	96 000 (86 000 to 106 000)	−67·1% (−69·0 to −65·2)	37 000 (31 000 to 42 000)	−82·5% (−84·7 to −79·2)	1 097 000 (1 063 000 to 1 135 000)	−51·4% (−52·6 to −50·0)	770 000 (676 000 to 862 000)	−81·5% (−83·2 to −78·9)
	High-income North America		466 000 (410 000 to 528 000)	−33·6% (−36·7 to −30·4)	209 000 (177 000 to 225 000)	−34·1% (−37·0 to −32·0)	7 005 000 (6 589 000 to 7 467 000)	−2·6% (−6·1 to 0·9)	4 254 000 (3 847 000 to 4 597 000)	−30·1% (−32·6 to −28·2)
		Canada	54 000 (51 000 to 58 000)	−36·8% (−42·1 to −31·1)	17 000 (14 000 to 19 000)	−57·0% (−60·0 to −54·0)	705 000 (691 000 to 720 000)	−10·7% (−13·0 to −8·3)	341 000 (304 000 to 374 000)	−49·3% (−52·4 to −46·3)
		Greenland	80 (80 to 90)	−48·2% (−51·2 to −44·9)	40 (30 to 40)	−61·1% (−67·1 to −53·3)	840 (810 to 870)	−39·1% (−41·7 to −36·9)	880 (760 to 1010)	−60·7% (−66·4 to −53·7)
		USA	412 000 (358 000 to 470 000)	−33·3% (−36·4 to −30·0)	192 000 (163 000 to 207 000)	−31·3% (−34·3 to −29·1)	6 299 000 (5 889 000 to 6 761 000)	−1·7% (−5·5 to 2·2)	3 912 000 (3 534 000 to 4 228 000)	−27·9% (−30·3 to −25·9)
	Southern Latin America		82 000 (76 000 to 89 000)	−42·1% (−44·8 to −39·7)	36 000 (33 000 to 39 000)	−63·1% (−65·0 to −61·1)	799 000 (773 000 to 825 000)	−28·6% (−30·2 to −26·9)	775 000 (726 000 to 825 000)	−62·6% (−64·5 to −60·7)
		Argentina	54 000 (49 000 to 58 000)	−42·5% (−45·6 to −39·4)	23 000 (21 000 to 25 000)	−64·3% (−66·4 to −62·3)	518 000 (500 000 to 537 000)	−29·4% (−31·4 to −27·4)	510 000 (479 000 to 543 000)	−63·3% (−65·4 to −61·2)
		Chile	23 000 (21 000 to 25 000)	−39·0% (−42·6 to −35·2)	10 000 (9000 to 11 000)	−60·8% (−63·6 to −58·4)	230 000 (222 000 to 238 000)	−21·0% (−23·3 to −18·8)	202 000 (187 000 to 218 000)	−60·6% (−62·9 to −58·3)
		Uruguay	6000 (5000 to 6000)	−44·0% (−47·2 to −40·7)	3000 (3000 to 4000)	−54·7% (−57·5 to −52·1)	51 000 (49 000 to 53 000)	−36·7% (−38·4 to −34·6)	62 000 (58 000 to 66 000)	−55·8% (−58·2 to −53·3)
	Western Europe		717 000 (664 000 to 771 000)	−43·4% (−45·7 to −41·0)	326 000 (272 000 to 355 000)	−68·3% (−70·2 to −67·0)	6 858 000 (6 651 000 to 7 084 000)	−22·4% (−23·8 to −20·8)	5 307 000 (4 726 000 to 5 734 000)	−65·1% (−66·6 to −63·5)
		Andorra	90 (80 to 110)	−28·2% (−32·7 to −24·0)	40 (30 to 50)	−49·5% (−65·2 to −30·4)	930 (890 to 970)	−19·3% (−21·7 to −17·0)	640 (500 to 800)	−48·8% (−63·6 to −31·8)
		Austria	17 000 (15 000 to 19 000)	−34·2% (−39·7 to −27·3)	5000 (4000 to 5000)	−75·7% (−77·6 to −74·2)	196 000 (191 000 to 202 000)	−1·1% (−3·5 to 1·2)	94 000 (84 000 to 104 000)	−68·6% (−71·2 to −66·3)
		Belgium	17 000 (16 000 to 19 000)	−44·0% (−48·5 to −38·8)	8000 (6000 to 8000)	−67·7% (−70·1 to −65·8)	151 000 (147 000 to 155 000)	−18·1% (−21·8 to −14·3)	127 000 (112 000 to 139 000)	−63·7% (−65·7 to −61·7)
		Cyprus	1000 (1000 to 1000)	−48·3% (−52·2 to −44·1)	770 (640 to 900)	−73·1% (−78·1 to −66·9)	10 000 (9000 to 11 000)	−39·2% (−41·4 to −36·5)	12 000 (10 000 to 14 000)	−72·6% (−77·7 to −66·9)
		Denmark	8000 (7000 to 9000)	−49·1% (−52·5 to −45·2)	4000 (4000 to 5000)	−58·5% (−61·3 to −56·0)	79 000 (76 000 to 82 000)	−34·9% (−36·9 to −33·0)	70 000 (63 000 to 76 000)	−60·2% (−62·5 to −58·1)
		Finland	12 000 (11 000 to 13 000)	−40·3% (−43·5 to −36·6)	5000 (4000 to 5000)	−62·8% (−65·7 to −60·5)	127 000 (123 000 to 130 000)	−21·9% (−23·5 to −20·4)	84 000 (74 000 to 91 000)	−61·2% (−63·4 to −59·1)
		France	94 000 (88 000 to 101 000)	−23·1% (−26·7 to −18·8)	43 000 (36 000 to 47 000)	−65·7% (−68·3 to −63·5)	929 000 (907 000 to 954 000)	−3·0% (−6·0 to −0·1)	686 000 (606 000 to 750 000)	−59·8% (−62·2 to −57·1)
		Germany	185 000 (168 000 to 202 000)	−38·6% (−42·8 to −34·2)	63 000 (52 000 to 69 000)	−70·1% (−72·3 to −68·2)	1 961 000 (1 908 000 to 2 014 000)	−18·8% (−20·5 to −16·9)	1 167 000 (1 042 000 to 1 283 000)	−64·2% (−66·4 to −61·8)
		Greece	27 000 (25 000 to 29 000)	−42·9% (−46·7 to −39·0)	17 000 (15 000 to 19 000)	−66·8% (−68·7 to −65·2)	185 000 (178 000 to 193 000)	−23·2% (−25·8 to −20·4)	247 000 (221 000 to 266 000)	−63·1% (−64·8 to −61·4)
		Iceland	400 (360 to 450)	−49·2% (−53·4 to −45·2)	150 (120 to 170)	−63·0% (−66·8 to −59·0)	4000 (4000 to 4000)	−30·2% (−32·2 to −28·4)	3000 (2000 to 3000)	−62·3% (−65·4 to −59·1)
		Ireland	4000 (4000 to 5000)	−58·6% (−61·5 to −55·7)	2000 (2000 to 2000)	−73·5% (−76·2 to −71·4)	43 000 (41 000 to 45 000)	−42·3% (−44·1 to −40·1)	32 000 (28 000 to 35 000)	−72·7% (−74·5 to −70·9)
		Israel	8000 (7000 to 9000)	−50·8% (−54·1 to −47·3)	3000 (2000 to 3000)	−67·2% (−70·1 to −64·7)	92 000 (89 000 to 95 000)	−29·6% (−31·4 to −27·6)	54 000 (48 000 to 59 000)	−64·5% (−66·7 to −62·2)
		Italy	92 000 (84 000 to 102 000)	−52·5% (−56·5 to −47·8)	62 000 (50 000 to 69 000)	−65·0% (−67·4 to −63·3)	727 000 (671 000 to 790 000)	−28·6% (−30·9 to −25·8)	871 000 (744 000 to 948 000)	−64·7% (−66·5 to −63·1)
		Luxembourg	610 (560 to 650)	−57·2% (−59·7 to −54·4)	320 (280 to 360)	−79·2% (−81·2 to −77·2)	5000 (5000 to 6000)	−39·9% (−44·1 to −35·6)	5000 (5000 to 6000)	−77·9% (−79·7 to −75·9)
		Malta	560 (510 to 620)	−54·9% (−57·7 to −51·9)	270 (220 to 300)	−72·8% (−75·4 to −69·7)	5000 (5000 to 5000)	−34·6% (−37·1 to −32·2)	4000 (4000 to 5000)	−71·1% (−73·6 to −68·3)
		Monaco	80 (70 to 90)	−43·3% (−47·1 to −39·3)	50 (40 to 70)	−59·3% (−68·9 to −43·5)	700 (670 to 740)	−26·9% (−29·0 to −24·6)	800 (650 to 960)	−57·6% (−67·0 to −44·1)
		Netherlands	26 000 (23 000 to 29 000)	−46·5% (−50·0 to −42·8)	12 000 (10 000 to 14 000)	−54·1% (−57·2 to −51·3)	251 000 (242 000 to 261 000)	−36·2% (−38·0 to −34·6)	199 000 (176 000 to 217 000)	−56·5% (−59·1 to −54·2)
		Norway	9000 (8000 to 11 000)	−43·3% (−47·3 to −39·1)	3000 (2000 to 3000)	−68·6% (−70·7 to −67·0)	91 000 (84 000 to 99 000)	−28·7% (−31·7 to −25·4)	50 000 (44 000 to 55 000)	−65·9% (−68·0 to −64·2)
		Portugal	18 000 (17 000 to 20 000)	−68·7% (−70·3 to −67·0)	14 000 (12 000 to 15 000)	−80·4% (−81·9 to −79·2)	121 000 (116 000 to 127 000)	−55·3% (−57·9 to −52·4)	204 000 (181 000 to 220 000)	−79·4% (−80·6 to −78·3)
		San Marino	50 (50 to 60)	−37·8% (−41·8 to −34·2)	20 (20 to 30)	−68·7% (−77·9 to −57·7)	500 (470 to 520)	−23·5% (−25·5 to −21·5)	360 (270 to 460)	−63·0% (−72·6 to −52·2)
		Spain	71 000 (67 000 to 75 000)	−50·0% (−53·9 to −45·3)	32 000 (26 000 to 35 000)	−75·1% (−76·7 to −73·5)	694 000 (679 000 to 711 000)	−19·9% (−24·4 to −14·9)	518 000 (458 000 to 569 000)	−70·3% (−72·1 to −68·6)
		Sweden	18 000 (16 000 to 21 000)	−35·9% (−39·6 to −31·6)	7000 (6000 to 8000)	−62·3% (−65·9 to −58·8)	179 000 (165 000 to 194 000)	−16·9% (−22·3 to −11·5)	113 000 (99 000 to 127 000)	−59·5% (−62·8 to −56·3)
		Switzerland	10 000 (9 000 to 12 000)	−41·7% (−45·7 to −36·2)	4 000 (3 000 to 5 000)	−70·6% (−73·3 to −68·1)	103 000 (99 000 to 106 000)	−19·5% (−22·3 to −17·1)	67 000 (58 000 to 75 000)	−67·5% (−69·8 to −65·2)
		UK	96 000 (87 000 to 106 000)	−43·3% (−46·3 to −40·1)	41 000 (35 000 to 44 000)	−67·3% (−69·0 to −66·2)	895 000 (843 000 to 953 000)	−26·0% (−27·8 to −24·2)	690 000 (630 000 to 740 000)	−64·8% (−66·1 to −63·5)
Latin America and Caribbean			554 000 (503 000 to 611 000)	−39·6% (−41·2 to −37·9)	279 000 (254 000 to 300 000)	−53·6% (−56·6 to −50·5)	5 184 000 (4 916 000 to 5 466 000)	−26·1% (−27·3 to −24·7)	6 414 000 (5 981 000 to 6 862 000)	−53·0% (−56·1 to −49·9)
	Andean Latin America		46 000 (42 000 to 50 000)	−33·6% (−35·7 to −31·3)	22 000 (19 000 to 26 000)	−48·0% (−56·5 to −37·7)	496 000 (481 000 to 513 000)	−19·8% (−21·0 to −18·6)	544 000 (460 000 to 644 000)	−49·8% (−58·1 to −40·3)
		Bolivia	8000 (7000 to 9000)	−31·8% (−34·3 to −28·6)	5000 (4000 to 7000)	−46·1% (−57·5 to −28·9)	75 000 (72 000 to 78 000)	−25·1% (−26·8 to −23·4)	134 000 (98 000 to 180 000)	−51·0% (−61·9 to −34·1)
		Ecuador	14 000 (13 000 to 16 000)	−25·9% (−28·9 to −21·9)	6000 (5000 to 8000)	−44·7% (−54·4 to −33·6)	153 000 (148 000 to 158 000)	−18·4% (−20·3 to −16·5)	146 000 (119 000 to 178 000)	−48·8% (−57·9 to −38·5)
		Peru	24 000 (22 000 to 26 000)	−37·8% (−40·6 to −34·6)	11 000 (8000 to 13 000)	−49·6% (−62·0 to −34·1)	269 000 (260 000 to 277 000)	−19·5% (−21·3 to −17·9)	264 000 (212 000 to 327 000)	−49·3% (−60·4 to −35·2)
	Caribbean		59 000 (55 000 to 63 000)	−17·1% (−19·2 to −14·9)	39 000 (35 000 to 45 000)	−32·6% (−40·3 to −23·8)	483 000 (467 000 to 500 000)	−9·7% (−11·1 to −8·3)	893 000 (778 000 to 1 027 000)	−30·5% (−38·9 to −20·2)
		Antigua and Barbuda	110 (100 to 120)	−26·6% (−30·5 to −22·9)	70 (60 to 70)	−41·2% (−45·5 to −36·6)	920 (890 to 950)	−16·9% (−19·1 to −14·8)	1000 (1000 to 2000)	−45·2% (−49·4 to −40·4)
		The Bahamas	400 (370 to 430)	−21·2% (−25·2 to −17·2)	200 (170 to 240)	−38·0% (−49·4 to −25·3)	3700 (3600 to 3800)	−12·4% (−14·8 to −10·1)	5000 (4000 to 6000)	−40·1% (−51·2 to −26·9)
		Barbados	500 (460 to 550)	−25·2% (−29·1 to −20·7)	370 (300 to 450)	−38·4% (−50·0 to −26·0)	4200 (4000 to 4400)	−12·8% (−15·5 to −10·0)	7000 (6000 to 8000)	−38·4% (−50·4 to −25·3)
		Belize	280 (250 to 300)	−15·2% (−19·7 to −10·4)	140 (130 to 160)	−24·8% (−33·6 to −15·6)	3000 (2000 to 3000)	−8·6% (−11·0 to −6·2)	3000 (3000 to 4000)	−29·2% (−37·7 to −20·7)
		Bermuda	90 (80 to 100)	−38·6% (−42·1 to −35·2)	50 (40 to 60)	−59·8% (−65·5 to −51·7)	930 (900 to 960)	−21·3% (−23·1 to −19·3)	820 (710 to 970)	−58·9% (−64·5 to −51·6)
		Cuba	17 000 (16 000 to 19 000)	−22·6% (−26·7 to −18·2)	11 000 (10 000 to 13 000)	−30·5% (−38·6 to −22·2)	145 000 (140 000 to 151 000)	−14·3% (−16·6 to −11·8)	212 000 (187 000 to 238 000)	−33·1% (−41·1 to −24·7)
		Dominica	80 (70 to 80)	−15·5% (−20·1 to −10·8)	70 (60 to 80)	−26·1% (−35·8 to −15·0)	640 (620 to 660)	−13·1% (−15·2 to −10·8)	1000 (1000 to 2000)	−26·6% (−37·3 to −14·2)
		Dominican Republic	14 000 (12 000 to 15 000)	14·3% (9·8 to 19·5)	7000 (6000 to 9000)	−21·4% (−39·3 to 4·9)	111 000 (108 000 to 115 000)	7·7% (5·2 to 10·2)	170 000 (136 000 to 215 000)	−17·8% (−36·1 to 8·9)
		Grenada	140 (120 to 150)	−25·6% (−29·8 to −21·7)	90 (70 to 90)	−45·0% (−51·9 to −38·1)	1000 (1000 to 1000)	−19·3% (−22·5 to −15·5)	2000 (2000 to 2000)	−50·6% (−57·0 to −43·9)
		Guyana	1000 (1000 to 1000)	−37·5% (−40·2 to −34·8)	760 (600 to 940)	−49·0% (−60·0 to −37·1)	8000 (7000 to 8000)	−26·9% (−29·2 to −24·5)	18 000 (14 000 to 23 000)	−52·6% (−63·6 to −40·4)
		Haiti	13 000 (12 000 to 14 000)	−21·2% (−24·2 to −17·6)	11 000 (8 000 to 14 000)	−30·2% (−47·0 to −9·9)	89 000 (86 000 to 93 000)	−15·1% (−17·4 to −12·9)	310 000 (233 000 to 409 000)	−33·5% (−49·9 to −13·5)
		Jamaica	4000 (3000 to 4000)	−17·6% (−21·8 to −13·2)	3000 (2000 to 4000)	−27·7% (−42·4 to −9·6)	26 000 (25 000 to 27 000)	−12·5% (−15·0 to −10·2)	54 000 (43 000 to 67 000)	−31·4% (−45·3 to −13·1)
		Puerto Rico	4000 (4000 to 4000)	−32·6% (−36·1 to −29·3)	2000 (1000 to 2000)	−62·7% (−68·6 to −56·8)	44 000 (42 000 to 45 000)	−11·9% (−14·2 to −9·3)	30 000 (25 000 to 34 000)	−55·2% (−61·6 to −48·7)
		Saint Kitts and Nevis	90 (90 to 100)	−41·2% (−44·0 to −38·1)	60 (50 to 70)	−50·4% (−57·1 to −45·0)	730 (710 to 770)	−35·8% (−38·3 to −32·9)	1000 (1000 to 2000)	−53·2% (−60·3 to −46·6)
		Saint Lucia	260 (240 to 280)	−40·5% (−44·0 to −37·4)	200 (160 to 230)	−56·1% (−62·9 to −49·0)	2000 (2000 to 2000)	−25·1% (−28·0 to −21·9)	4000 (3000 to 4000)	−55·9% (−62·8 to −48·4)
		Saint Vincent and the Grenadines	160 (140 to 170)	−23·8% (−28·1 to −19·2)	110 (100 to 130)	−39·8% (−45·9 to −32·8)	1000 (1000 to 1000)	−20·9% (−23·6 to −18·2)	2000 (2000 to 3000)	−39·6% (−46·4 to −32·2)
		Suriname	840 (770 to 900)	−13·0% (−17·0 to −8·9)	550 (430 to 680)	−29·1% (−45·7 to −10·9)	6000 (6 000 to 6 000)	−12·8% (−15·0 to −10·4)	13 000 (10 000 to 16 000)	−29·0% (−44·7 to −11·3)
		Trinidad and Tobago	2000 (2000 to 2000)	−37·9% (−41·1 to −34·6)	1000 (1000 to 2000)	−49·9% (−59·8 to −38·5)	17 000 (17 000 to 18 000)	−25·7% (−27·7 to −23·5)	27 000 (21 000 to 33 000)	−47·8% (−58·2 to −34·7)
		Virgin Islands	150 (140 to 170)	−9·3% (−13·9 to −4·4)	60 (50 to 70)	−57·1% (−65·8 to −46·3)	1200 (1200 to 1300)	−7·0% (−9·4 to −4·6)	1200 (1000 to 1500)	−55·3% (−64·6 to −43·1)
	Central Latin America		204 000 (186 000 to 223 000)	−34·4% (−36·0 to −32·8)	89 000 (79 000 to 98 000)	−45·6% (−50·6 to −40·1)	2 100 000 (2 006 000 to 2 208 000)	−23·1% (−24·3 to −21·9)	2 051 000 (1 852 000 to 2 284 000)	−43·0% (−48·3 to −37·1)
		Colombia	42 000 (38 000 to 46 000)	−44·5% (−47·3 to −41·3)	17 000 (14 000 to 19 000)	−60·5% (−66·7 to −53·9)	429 000 (416 000 to 444 000)	−31·6% (−33·2 to −29·7)	369 000 (312 000 to 432 000)	−59·5% (−65·5 to −53·0)
		Costa Rica	4000 (4000 to 5000)	−29·9% (−33·4 to −26·2)	2000 (1000 to 2000)	−41·6% (−47·9 to −35·2)	44 000 (43 000 to 46 000)	−16·9% (−18·9 to −14·8)	32 000 (28 000 to 35 000)	−40·3% (−46·4 to −34·5)
		El Salvador	5000 (4000 to 5000)	−34·7% (−37·6 to −31·7)	2000 (2000 to 3000)	−46·7% (−57·4 to −34·6)	45 000 (43 000 to 47 000)	−25·2% (−26·8 to −23·3)	47 000 (39 000 to 57 000)	−50·1% (−59·8 to −39·1)
		Guatemala	10 000 (9000 to 10 000)	−22·2% (−26·0 to −18·1)	4000 (4000 to 5000)	−36·6% (−44·4 to −27·5)	89 000 (86 000 to 92 000)	−19·9% (−21·6 to −17·6)	101 000 (88 000 to 115 000)	−39·1% (−47·5 to −30·6)
		Honduras	6000 (6000 to 7000)	0·4% (−4·3 to 5·2)	6000 (5000 to 8000)	23·4% (0·2 to 54·4)	56 000 (54 000 to 58 000)	−12·6% (−14·9 to −10·5)	147 000 (120 000 to 182 000)	5·4% (−15·0 to 31·6)
		Mexico	100 000 (89 000 to 111 000)	−34·6% (−36·7 to −32·1)	38 000 (34 000 to 43 000)	−50·1% (−55·1 to −44·9)	1 100 000 (1 029 000 to 1 179 000)	−21·7% (−23·6 to −19·7)	915 000 (821 000 to 1 020 000)	−43·5% (−49·0 to −37·6)
		Nicaragua	5000 (4000 to 5000)	−33·7% (−37·5 to −30·2)	1000 (1000 to 2000)	−45·9% (−54·4 to −34·9)	43 000 (42 000 to 45 000)	−21·5% (−23·4 to −19·5)	36 000 (31 000 to 43 000)	−47·2% (−55·0 to −36·9)
		Panama	4000 (4000 to 4000)	−30·7% (−34·6 to −27·1)	2000 (2000 to 2000)	−38·6% (−51·4 to −27·6)	37 000 (36 000 to 39 000)	−17·5% (−19·4 to −15·5)	40 000 (32 000 to 48 000)	−39·0% (−51·1 to −28·1)
		Venezuela	29 000 (26 000 to 31 000)	−25·3% (−29·1 to −21·4)	16 000 (12 000 to 20 000)	−24·3% (−41·0 to −5·9)	257 000 (248 000 to 266 000)	−18·5% (−20·6 to −16·4)	363 000 (279 000 to 458 000)	−26·1% (−42·8 to −7·4)
	Tropical Latin America		245 000 (218 000 to 275 000)	−47·3% (−49·5 to −45·2)	129 000 (118 000 to 137 000)	−61·7% (−63·3 to −60·3)	2 105 000 (1 950 000 to 2 262 000)	−32·8% (−34·8 to −30·8)	2 926 000 (2 755 000 to 3 053 000)	−61·4% (−62·9 to −60·1)
		Brazil	239 000 (212 000 to 268 000)	−47·7% (−49·9 to −45·6)	126 000 (115 000 to 133 000)	−62·2% (−63·8 to −60·8)	2 053 000 (1 898 000 to 2 207 000)	−33·1% (−35·1 to −31·1)	2 843 000 (2 679 000 to 2 966 000)	−61·8% (−63·3 to −60·5)
		Paraguay	6000 (6000 to 7000)	−28·6% (−33·0 to −23·9)	4000 (3000 to 5000)	−37·3% (−51·6 to −21·8)	52 000 (50 000 to 54 000)	−19·0% (−21·5 to −16·6)	83 000 (65 000 to 103 000)	−38·1% (−52·4 to −22·0)
	North Africa and Middle East		615 000 (560 000 to 672 000)	−21·2% (−23·9 to −18·2)	372 000 (327 000 to 417 000)	−40·3% (−46·6 to −32·4)	5 573 000 (5 372 000 to 5 794 000)	−11·2% (−12·7 to −9·7)	8 891 000 (7 809 000 to 10 011 000)	−44·4% (−51·1 to −37·1)
		Afghanistan	22 000 (20 000 to 24 000)	−21·6% (−25·5 to −17·5)	15 000 (11 000 to 19 000)	−28·1% (−43·7 to −8·7)	171 000 (164 000 to 178 000)	−14·7% (−17·3 to −12·3)	459 000 (355 000 to 579 000)	−32·0% (−47·6 to −11·4)
		Algeria	52 000 (46 000 to 57 000)	−24·3% (−30·6 to −18·0)	27 000 (21 000 to 34 000)	−36·1% (−47·5 to −21·2)	458 000 (443 000 to 476 000)	−11·9% (−14·5 to −9·2)	572 000 (456 000 to 712 000)	−40·6% (−51·1 to −27·7)
		Bahrain	730 (650 to 810)	−35·5% (−39·2 to −31·4)	360 (310 to 420)	−49·1% (−57·3 to −39·0)	10 000 (10 000 to 10 000)	−21·3% (−23·5 to −19·4)	10 000 (9000 to 12 000)	−52·9% (−60·3 to −44·0)
		Egypt	105 000 (95 000 to 117 000)	3·4% (−2·4 to 11·3)	73 000 (60 000 to 89 000)	−36·1% (−47·4 to −23·7)	897 000 (859 000 to 936 000)	11·5% (6·6 to 15·8)	1 848 000 (1 512 000 to 2 235 000)	−39·8% (−50·9 to −27·5)
		Iran	76 000 (67 000 to 86 000)	−31·8% (−34·7 to −29·2)	42 000 (38 000 to 46 000)	−48·8% (−53·3 to −43·1)	787 000 (727 000 to 855 000)	−14·8% (−17·4 to −11·9)	905 000 (828 000 to 981 000)	−50·2% (−54·5 to −45·2)
		Iraq	45 000 (41 000 to 50 000)	−8·1% (−14·0 to −1·9)	31 000 (24 000 to 37 000)	−14·4% (−34·3 to 6·3)	379 000 (366 000 to 393 000)	−7·2% (−9·8 to −4·0)	764 000 (602 000 to 932 000)	−27·2% (−44·8 to −7·1)
		Jordan	12 000 (11 000 to 13 000)	−24·1% (−29·4 to −17·8)	4000 (3000 to 4000)	−53·7% (−63·8 to −40·2)	118 000 (113 000 to 122 000)	−4·6% (−7·5 to −1·1)	97 000 (81 000 to 114 000)	−55·8% (−65·0 to −43·8)
		Kuwait	3000 (3000 to 4000)	−9·2% (−15·1 to −3·7)	770 (630 to 920)	−32·7% (−43·0 to −20·4)	43 000 (41 000 to 44 000)	−8·0% (−10·6 to −5·2)	23 000 (20 000 to 27 000)	−36·9% (−45·8 to −27·0)
		Lebanon	7000 (6000 to 8000)	−24·1% (−28·5 to −19·2)	3000 (2000 to 3000)	−68·6% (−76·3 to −59·3)	65 000 (62 000 to 68 000)	−1·5% (−4·1 to 1·2)	57 000 (49 000 to 66 000)	−68·9% (−76·4 to −60·0)
		Libya	6000 (6000 to 7000)	1·7% (−4·4 to 7·9)	3000 (3000 to 5000)	−3·8% (−27·3 to 26·3)	66 000 (64 000 to 68 000)	0·8% (−1·9 to 3·5)	95 000 (71 000 to 125 000)	−6·7% (−28·8 to 20·8)
		Morocco	53 000 (48 000 to 58 000)	−5·8% (−11·2 to 0·7)	37 000 (29 000 to 46 000)	−16·6% (−33·7 to 0·8)	413 000 (395 000 to 431 000)	−3·5% (−6·5 to −0·5)	811 000 (633 000 to 1 024 000)	−25·4% (−41·0 to −9·4)
		Oman	3000 (3000 to 4000)	−13·1% (−19·4 to −6·0)	1000 (1000 to 1000)	−41·3% (−55·9 to −18·5)	40 000 (38 000 to 41 000)	−4·7% (−7·8 to −1·6)	30 000 (25 000 to 35 000)	−48·3% (−61·1 to −28·6)
		Palestine	4000 (3000 to 4000)	−13·4% (−18·3 to −8·2)	2000 (2000 to 2000)	−42·2% (−53·3 to −28·8)	29 000 (28 000 to 30 000)	−12·4% (−15·4 to −9·5)	46 000 (41 000 to 52 000)	−45·6% (−55·9 to −32·5)
		Qatar	1000 (1000 to 2000)	−36·7% (−40·8 to −33·1)	250 (190 to 320)	−65·4% (−73·3 to −56·1)	22 000 (21 000 to 23 000)	−37·1% (−38·9 to −35·4)	10 000 (8000 to 12 000)	−65·8% (−73·5 to −56·8)
		Saudi Arabia	28 000 (25 000 to 31 000)	−21·4% (−26·5 to −16·4)	13 000 (11 000 to 17 000)	−38·5% (−53·2 to −17·3)	278 000 (268 000 to 289 000)	−1·8% (−4·9 to 1·2)	439 000 (351 000 to 545 000)	−39·5% (−53·4 to −19·3)
		Sudan	34 000 (31 000 to 37 000)	−17·0% (−21·5 to −11·4)	20 000 (15 000 to 25 000)	−40·0% (−53·3 to −20·3)	293 000 (281 000 to 305 000)	−4·5% (−7·5 to −1·4)	545 000 (403 000 to 704 000)	−46·1% (−58·7 to −26·8)
		Syria	16 000 (15 000 to 18 000)	−25·7% (−29·8 to −22·0)	11 000 (8000 to 13 000)	−29·2% (−47·1 to −2·2)	148 000 (143 000 to 154 000)	−24·3% (−26·2 to −22·5)	260 000 (202 000 to 332 000)	−40·1% (−55·6 to −17·1)
		Tunisia	15 000 (13 000 to 17 000)	−11·4% (−16·4 to −6·1)	9000 (7000 to 13 000)	−33·8% (−52·2 to −11·5)	123 000 (118 000 to 128 000)	0·4% (−2·3 to 3·5)	189 000 (137 000 to 256 000)	−35·9% (−53·0 to −15·4)
		Türkiye	98 000 (87 000 to 108 000)	−37·9% (−41·8 to −33·3)	59 000 (49 000 to 71 000)	−50·2% (−59·8 to −37·5)	927 000 (895 000 to 964 000)	−30·1% (−31·9 to −28·2)	1 185 000 (996 000 to 1 388 000)	−56·9% (−65·2 to −46·7)
		United Arab Emirates	10 000 (9000 to 12 000)	−28·1% (−32·8 to −23·6)	1000 (1000 to 2000)	−34·8% (−46·1 to −21·7)	113 000 (109 000 to 118 000)	−15·8% (−18·8 to −12·9)	52 000 (43 000 to 62 000)	−43·6% (−53·2 to −32·2)
		Yemen	24 000 (22 000 to 26 000)	−15·9% (−20·7 to −10·8)	18 000 (14 000 to 25 000)	−25·2% (−44·1 to 0·4)	188 000 (180 000 to 196 000)	−8·7% (−11·5 to −5·7)	485 000 (364 000 to 635 000)	−31·6% (−48·8 to −7·1)
	South Asia		1 697 000 (1 540 000 to 1 860 000)	−22·2% (−24·7 to −19·5)	1 067 000 (976 000 to 1 173 000)	−23·1% (−32·4 to −12·4)	12 593 000 (11 789 000 to 13 537 000)	−8·2% (−10·0 to −6·5)	26 602 000 (24 487 000 to 29 128 000)	−26·4% (−35·3 to −17·2)
		Bangladesh	221 000 (204 000 to 241 000)	−15·4% (−19·3 to −10·7)	177 000 (144 000 to 215 000)	−26·7% (−42·0 to −6·7)	1 449 000 (1 395 000 to 1 511 000)	−10·5% (−12·9 to −7·9)	3 942 000 (3 209 000 to 4 819 000)	−35·8% (−49·2 to −18·1)
		Bhutan	670 (610 to 730)	−22·0% (−26·2 to −16·9)	390 (310 to 480)	−32·2% (−48·5 to −10·2)	5000 (5000 to 5000)	−10·7% (−13·2 to −7·9)	9000 (7000 to 11 000)	−37·8% (−53·2 to −18·4)
		India	1 251 000 (1 127 000 to 1 378 000)	−24·0% (−26·7 to −21·0)	773 000 (695 000 to 858 000)	−22·8% (−33·8 to −10·0)	9 338 000 (8 687 000 to 10 110 000)	−8·0% (−10·0 to −6·0)	19 436 000 (17 539 000 to 21 385 000)	−26·1% (−36·5 to −14·8)
		Nepal	26 000 (24 000 to 28 000)	−18·0% (−21·8 to −13·9)	17 000 (14 000 to 22 000)	−32·0% (−47·8 to −10·6)	181 000 (173 000 to 189 000)	−14·1% (−16·6 to −11·4)	411 000 (330 000 to 520 000)	−36·9% (−51·8 to −18·3)
		Pakistan	198 000 (180 000 to 219 000)	−15·1% (−18·7 to −11·3)	100 000 (83 000 to 124 000)	−8·1% (−25·0 to 14·7)	1 620 000 (1 500 000 to 1 746 000)	−5·6% (−8·2 to −2·8)	2 804 000 (2 318 000 to 3 459 000)	−8·5% (−25·2 to 14·2)
Southeast Asia, east Asia, and Oceania			5 425 000 (4 831 000 to 6 143 000)	−9·5% (−13·9 to −5·0)	3 554 000 (3 106 000 to 4 001 000)	−37·5% (−46·1 to −27·1)	36 232 000 (33 712 000 to 38 979 000)	6·7% (4·3 to 8·9)	77 453 000 (68 193 000 to 86 258 000)	−39·2% (−47·1 to −29·8)
	East Asia		4 220 000 (3 717 000 to 4 838 000)	−10·5% (−15·7 to −5·2)	2 664 000 (2 248 000 to 3 100 000)	−43·0% (−52·6 to −31·2)	27 268 000 (25 077 000 to 29 587 000)	10·0% (7·0 to 12·8)	54 947 000 (46 857 000 to 63 714 000)	−45·0% (−54·1 to −33·8)
		China	4 090 000 (3 594 000 to 4 700 000)	−9·8% (−15·2 to −4·2)	2 592 000 (2 179 000 to 3 033 000)	−43·0% (−52·8 to −30·9)	26 335 000 (24 155 000 to 28 626 000)	11·5% (8·3 to 14·5)	53 191 000 (45 109 000 to 61 958 000)	−45·2% (−54·4 to −33·7)
		North Korea	79 000 (72 000 to 85 000)	−8·2% (−12·8 to −2·7)	58 000 (48 000 to 71 000)	−12·8% (−30·9 to 12·2)	472 000 (455 000 to 492 000)	−6·6% (−9·4 to −3·5)	1 408 000 (1 132 000 to 1 711 000)	−9·4% (−29·7 to 16·9)
		Taiwan (province of China)	51 000 (46 000 to 56 000)	−44·6% (−48·6 to −40·8)	14 000 (12 000 to 15 000)	−76·4% (−78·3 to −74·8)	461 000 (445 000 to 477 000)	−26·1% (−28·2 to −24·2)	349 000 (312 000 to 378 000)	−70·8% (−73·0 to −68·8)
	Oceania		12 000 (11 000 to 13 000)	−16·3% (−19·0 to −12·9)	10 000 (8 000 to 12 000)	−21·8% (−35·6 to −4·8)	99 000 (96 000 to 102 000)	−11·2% (−12·7 to −9·6)	291 000 (238 000 to 348 000)	−22·4% (−37·0 to −4·9)
		American Samoa	70 (60 to 80)	−21·4% (−25·8 to −17·5)	50 (40 to 50)	−25·1% (−37·3 to −9·2)	620 (600 to 640)	−16·0% (−18·0 to −14·1)	1000 (1000 to 1000)	−24·0% (−36·3 to −8·7)
		Cook Islands	30 (30 to 40)	−21·9% (−25·7 to −17·7)	20 (10 to 20)	−52·0% (−61·6 to −40·2)	320 (310 to 330)	−4·9% (−7·2 to −2·6)	380 (310 to 440)	−49·5% (−59·5 to −37·1)
		Federated States of Micronesia	180 (160 to 190)	−13·8% (−17·4 to −10·1)	120 (90 to 150)	−25·8% (−40·6 to −5·7)	1000 (1000 to 1000)	−10·3% (−12·4 to −8·2)	4000 (3000 to 5000)	−25·9% (−41·3 to −3·5)
		Fiji	1000 (1000 to 1000)	−23·0% (−26·5 to −18·5)	770 (600 to 960)	−20·7% (−37·4 to 1·5)	12 000 (11 000 to 12 000)	−14·5% (−16·5 to −12·4)	21 000 (17 000 to 27 000)	−25·4% (−40·7 to −4·7)
		Guam	260 (240 to 280)	−21·0% (−25·3 to −16·5)	80 (70 to 90)	−60·9% (−65·9 to −54·7)	3000 (3000 to 3000)	−1·7% (−4·3 to 0·9)	3000 (2000 to 3000)	−40·5% (−47·8 to −32·3)
		Kiribati	220 (210 to 240)	−16·4% (−19·9 to −12·6)	110 (90 to 140)	−9·2% (−25·7 to 14·4)	2000 (2000 to 2000)	−13·3% (−15·4 to −11·4)	4000 (3000 to 5000)	−12·5% (−29·0 to 11·3)
		Marshall Islands	90 (80 to 90)	−9·8% (−13·2 to −6·3)	60 (40 to 70)	−19·4% (−35·2 to −0·9)	670 (650 to 690)	−5·6% (−8·0 to −3·4)	2000 (1000 to 2000)	−17·7% (−34·3 to 3·6)
		Nauru	10 (10 to 20)	−26·0% (−29·3 to −22·4)	10 (10 to 20)	−14·9% (−31·4 to 9·4)	150 (140 to 150)	−9·6% (−12·0 to −7·4)	410 (320 to 530)	−13·8% (−31·0 to 11·9)
		Niue	0 (0 to 0)	−22·5% (−26·1 to −18·1)	0 (0 to 0)	−25·4% (−38·9 to −8·7)	30 (30 to 30)	−13·0% (−15·2 to −10·7)	60 (50 to 80)	−22·8% (−37·8 to −6·0)
		Northern Mariana Islands	70 (60 to 80)	−17·2% (−21·9 to −12·0)	40 (30 to 40)	−34·4% (−47·1 to −20·9)	650 (630 to 670)	−14·0% (−16·2 to −11·9)	1000 (1000 to 1000)	−35·0% (−47·2 to −21·3)
		Palau	40 (40 to 50)	−14·7% (−18·7 to −10·5)	20 (20 to 30)	−23·7% (−41·1 to −2·0)	400 (380 to 410)	−3·9% (−6·1 to −1·5)	720 (600 to 870)	−23·8% (−40·6 to −2·2)
		Papua New Guinea	7000 (7000 to 8000)	−13·1% (−17·2 to −8·5)	7000 (5000 to 9000)	−20·4% (−40·5 to 6·0)	59 000 (57 000 to 61 000)	−7·5% (−9·8 to −5·1)	207 000 (161 000 to 258 000)	−22·4% (−42·9 to 5·0)
		Samoa	280 (260 to 310)	−16·7% (−21·1 to −12·2)	190 (160 to 230)	−22·8% (−36·0 to −4·0)	2000 (2000 to 3000)	−4·6% (−7·1 to −1·9)	5000 (4000 to 6000)	−20·7% (−34·4 to −0·9)
		Solomon Islands	1000 (1000 to 1000)	−3·8% (−8·1 to 0·9)	630 (510 to 790)	−12·5% (−30·2 to 12·5)	8000 (8 000 to 8 000)	−3·4% (−6·0 to −0·8)	19 000 (15 000 to 23 000)	−12·1% (−32·1 to 18·7)
		Tokelau	0 (0 to 0)	−29·3% (−33·1 to −25·7)	0 (0 to 0)	−39·1% (−51·3 to −23·7)	20 (20 to 20)	−10·0% (−12·3 to −7·5)	40 (40 to 50)	−35·4% (−48·5 to −18·5)
		Tongo	110 (100 to 110)	−11·7% (−16·3 to −7·0)	60 (50 to 70)	−15·3% (−34·8 to 10·5)	950 (920 to 980)	−7·2% (−9·3 to −5·0)	1000 (1000 to 2000)	−17·4% (−36·1 to 7·0)
		Tuvalu	20 (20 to 20)	−19·7% (−23·3 to −15·7)	20 (10 to 20)	−35·8% (−45·9 to −23·8)	160 (150 to 160)	−8·5% (−10·9 to −6·5)	430 (370 to 510)	−36·6% (−47·1 to −23·4)
		Vanuatu	450 (420 to 490)	−5·3% (−9·5 to −1·0)	260 (210 to 320)	−20·2% (−35·5 to −3·0)	4000 (4000 to 4000)	−1·0% (−3·6 to 1·4)	8000 (7000 to 10 000)	−18·5% (−35·3 to 1·8)
	Southeast Asia		1 193 000 (1 090 000 to 1 305 000)	−12·4% (−14·5 to −10·2)	880 000 (791 000 to 959 000)	−20·6% (−29·9 to −9·5)	8 865 000 (8 402 000 to 9 372 000)	−6·7% (−8·1 to −5·3)	22 214 000 (19 885 000 to 24 341 000)	−22·8% (−31·3 to −13·1)
		Cambodia	24 000 (22 000 to 25 000)	−11·1% (−15·5 to −6·6)	18 000 (14 000 to 22 000)	−22·1% (−39·4 to −3·3)	145 000 (140 000 to 151 000)	−6·5% (−9·1 to −3·4)	435 000 (339 000 to 535 000)	−29·8% (−45·5 to −11·9)
		Indonesia	543 000 (487 000 to 611 000)	−0·1% (−3·7 to 3·9)	405 000 (338 000 to 464 000)	5·5% (−14·2 to 26·3)	3 942 000 (3 639 000 to 4 286 000)	−6·7% (−9·0 to −4·3)	10 624 000 (8 957 000 to 12 309 000)	−5·8% (−20·9 to 12·4)
		Laos	9000 (9000 to 10 000)	−19·7% (−23·7 to −15·1)	7000 (6000 to 9000)	−38·2% (−52·9 to −19·9)	66 000 (63 000 to 68 000)	−10·8% (−13·5 to −8·2)	193 000 (154 000 to 240 000)	−43·3% (−57·6 to −26·5)
		Malaysia	45 000 (41 000 to 49 000)	−29·5% (−33·0 to −25·1)	23 000 (21 000 to 26 000)	−32·7% (−40·1 to −23·6)	401 000 (388 000 to 415 000)	−9·1% (−11·4 to −6·6)	593 000 (541 000 to 650 000)	−36·1% (−42·2 to −28·7)
		Maldives	460 (430 to 510)	−47·5% (−50·2 to −44·3)	220 (180 to 260)	−64·7% (−71·3 to −57·1)	4000 (4000 to 4000)	−40·2% (−41·8 to −38·4)	6000 (5000 to 6000)	−70·1% (−75·6 to −63·6)
		Mauritius	2000 (2000 to 2000)	−48·2% (−51·4 to −44·9)	1000 (1000 to 1000)	−62·4% (−65·0 to −60·3)	18 000 (18 000 to 19 000)	−35·2% (−37·0 to −33·3)	29 000 (27 000 to 30 000)	−60·7% (−63·4 to −58·6)
		Myanmar	88 000 (82 000 to 96 000)	−25·3% (−29·1 to −20·6)	77 000 (62 000 to 96 000)	−37·5% (−52·5 to −16·7)	597 000 (577 000 to 620 000)	−15·4% (−17·6 to −13·0)	1 961 000 (1 594 000 to 2 424 000)	−42·6% (−56·5 to −23·5)
		Philippines	133 000 (121 000 to 148 000)	27·9% (22·7 to 33·7)	84 000 (71 000 to 96 000)	−14·1% (−25·9 to −0·9)	1 052 000 (981 000 to 1 134 000)	19·6% (16·7 to 22·3)	2 364 000 (2 037 000 to 2 715 000)	−4·2% (−17·1 to 11·3)
		Sri Lanka	31 000 (28 000 to 34 000)	−24·9% (−28·5 to −20·7)	25 000 (18 000 to 33 000)	−37·4% (−56·2 to −16·5)	271 000 (260 000 to 281 000)	−15·2% (−17·3 to −13·0)	498 000 (361 000 to 642 000)	−39·1% (−57·3 to −18·5)
		Seychelles	150 (130 to 160)	−25·7% (−29·3 to −22·2)	80 (70 to 90)	−39·1% (−46·2 to −31·3)	1000 (1000 to 1000)	−16·1% (−18·5 to −13·5)	2000 (2000 to 2000)	−42·3% (−48·9 to −35·1)
		Thailand	118 000 (108 000 to 128 000)	−35·1% (−38·5 to −31·9)	70 000 (54 000 to 86 000)	−46·1% (−57·9 to −29·7)	1 070 000 (1 037 000 to 1 109 000)	−18·2% (−20·3 to −15·9)	1 676 000 (1 347 000 to 2 042 000)	−40·5% (−52·8 to −24·2)
		Timor-Leste	2000 (1000 to 2000)	2·9% (−2·5 to 8·6)	1000 (1000 to 2000)	−7·6% (−30·4 to 23·1)	10 000 (10 000 to 10 000)	1·3% (−1·4 to 4·2)	32 000 (25 000 to 41 000)	−11·9% (−34·4 to 16·1)
		Viet Nam	196 000 (183 000 to 209 000)	−9·3% (−14·8 to −4·4)	167 000 (140 000 to 193 000)	−14·3% (−33·4 to 7·4)	1 275 000 (1 238 000 to 1 318 000)	1·7% (−1·4 to 5·2)	3 769 000 (3 132 000 to 4 444 000)	−17·4% (−36·5 to 5·5)
Sub-Saharan Africa			867 000 (795 000 to 944 000)	−17·8% (−19·8 to −15·3)	484 000 (433 000 to 539 000)	−23·0% (−30·6 to −13·2)	7 769 000 (7 457 000 to 8 094 000)	−12·0% (−13·3 to −10·8)	13 251 000 (11 716 000 to 14 891 000)	−26·6% (−33·8 to −17·4)
	Central sub-Saharan Africa		103 000 (94 000 to 113 000)	−18·4% (−22·0 to −14·6)	57 000 (44 000 to 73 000)	−16·4% (−33·2 to 3·8)	837 000 (808 000 to 868 000)	−13·6% (−15·8 to −11·6)	1 587 000 (1 225 000 to 1 988 000)	−21·6% (−36·9 to −2·4)
		Angola	23 000 (21 000 to 25 000)	−23·6% (−28·2 to −18·5)	12 000 (9000 to 15 000)	−25·2% (−43·0 to −2·5)	202 000 (195 000 to 209 000)	−12·3% (−15·3 to −9·7)	340 000 (269 000 to 418 000)	−31·4% (−48·4 to −11·3)
		Central African Republic	5000 (4000 to 5000)	−14·0% (−18·7 to −9·1)	3000 (2000 to 4000)	−14·8% (−31·8 to 4·8)	35 000 (34 000 to 36 000)	−11·3% (−14·1 to −8·7)	97 000 (70 000 to 132 000)	−18·0% (−35·5 to 1·7)
		Congo (Brazzaville)	5000 (5000 to 6000)	−25·6% (−29·8 to −21·0)	3000 (2000 to 4000)	−31·4% (−45·1 to −14·2)	46 000 (44 000 to 48 000)	−18·5% (−21·0 to −15·8)	82 000 (62 000 to 103 000)	−35·1% (−49·1 to −18·5)
		Democratic Republic of the Congo	67 000 (62 000 to 74 000)	−16·2% (−20·5 to −11·7)	38 000 (28 000 to 51 000)	−10·9% (−32·9 to 16·9)	530 000 (510 000 to 550 000)	−13·9% (−16·6 to −11·2)	1 032 000 (760 000 to 1 368 000)	−16·2% (−35·8 to 9·3)
		Equatorial Guinea	860 (770 to 950)	−35·7% (−39·7 to −31·6)	410 (270 to 590)	−48·9% (−63·9 to −26·5)	8000 (8000 to 9000)	−18·0% (−20·2 to −15·7)	11 000 (8000 to 16 000)	−53·8% (−67·3 to −34·5)
		Gabon	2000 (2000 to 2000)	−20·8% (−25·7 to −15·4)	940 (710 to 1210)	−26·6% (−42·9 to −6·7)	17 000 (16 000 to 17 000)	−15·8% (−18·1 to −13·1)	24 000 (18 000 to 31 000)	−30·1% (−45·6 to −11·4)
	Eastern sub-Saharan Africa		311 000 (285 000 to 338 000)	−21·6% (−23·8 to −19·0)	169 000 (147 000 to 193 000)	−31·3% (−40·8 to −21·0)	2 612 000 (2 509 000 to 2 720 000)	−13·7% (−15·0 to −12·3)	4 698 000 (4 075 000 to 5 316 000)	−34·1% (−44·0 to −24·0)
		Burundi	9000 (9000 to 10 000)	−36·9% (−40·3 to −33·2)	6000 (4000 to 7000)	−45·9% (−59·3 to −27·1)	76 000 (74 000 to 79 000)	−29·4% (−31·4 to −27·5)	158 000 (126 000 to 194 000)	−49·4% (−62·4 to −31·9)
		Comoros	830 (750 to 910)	−26·9% (−31·7 to −22·6)	470 (360 to 580)	−38·3% (−53·0 to −17·8)	7000 (7000 to 8000)	−19·9% (−22·1 to −17·5)	12 000 (9000 to 15 000)	−41·6% (−56·0 to −21·2)
		Djibouti	1000 (1000 to 1000)	−14·6% (−19·3 to −9·4)	570 (420 to 760)	−25·0% (−43·9 to 2·2)	11 000 (11 000 to 12 000)	−9·1% (−11·7 to −6·2)	16 000 (12 000 to 22 000)	−27·5% (−46·2 to −0·8)
		Eritrea	6000 (5000 to 6000)	−28·5% (−32·1 to −24·5)	3000 (3000 to 4000)	−33·1% (−46·3 to −16·3)	47 000 (45 000 to 49 000)	−12·7% (−15·2 to −10·1)	99 000 (76 000 to 126 000)	−38·5% (−50·9 to −21·9)
		Ethiopia	59 000 (54 000 to 65 000)	−42·2% (−45·2 to −38·9)	30 000 (25 000 to 36 000)	−51·6% (−65·8 to −39·6)	500 000 (467 000 to 539 000)	−29·9% (−32·5 to −27·2)	807 000 (674 000 to 944 000)	−56·0% (−69·2 to −44·5)
		Kenya	37 000 (34 000 to 42 000)	−9·3% (−12·2 to −6·0)	18 000 (14 000 to 22 000)	−0·6% (−18·5 to 24·0)	332 000 (308 000 to 358 000)	−8·8% (−10·5 to −7·1)	468 000 (374 000 to 572 000)	−4·0% (−20·0 to 18·4)
		Madagascar	30 000 (28 000 to 32 000)	−13·4% (−17·7 to −9·0)	18 000 (14 000 to 24 000)	−18·9% (−38·3 to 3·1)	251 000 (243 000 to 259 000)	−9·9% (−12·2 to −7·5)	572 000 (431 000 to 733 000)	−21·8% (−40·2 to −0·1)
		Malawi	14 000 (12 000 to 15 000)	−15·6% (−20·3 to −10·7)	9000 (8000 to 11 000)	−6·0% (−23·0 to 14·0)	117 000 (113 000 to 122 000)	−10·8% (−13·3 to −8·0)	257 000 (215 000 to 305 000)	−9·4% (−26·0 to 11·0)
		Mozambique	29 000 (27 000 to 32 000)	3·4% (−1·9 to 9·1)	21 000 (17 000 to 26 000)	7·9% (−18·2 to 35·1)	221 000 (213 000 to 229 000)	8·8% (5·1 to 12·7)	599 000 (466 000 to 738 000)	9·7% (−15·9 to 36·0)
		Rwanda	11 000 (10 000 to 12 000)	−43·6% (−46·8 to −40·2)	6000 (5000 to 8000)	−56·6% (−68·9 to −42·9)	88 000 (85 000 to 91 000)	−34·4% (−36·4 to −32·5)	166 000 (123 000 to 212 000)	−61·3% (−72·5 to −47·8)
		Somalia	13 000 (12 000 to 15 000)	−20·5% (−24·5 to −16·6)	8000 (5000 to 10 000)	−29·3% (−45·3 to −8·6)	107 000 (103 000 to 111 000)	−14·4% (−16·6 to −12·1)	238 000 (169 000 to 320 000)	−31·0% (−47·6 to −9·3)
		South Sudan	6000 (6000 to 7000)	−19·8% (−24·2 to −16·0)	4000 (3000 to 6000)	−25·0% (−43·0 to −1·5)	55 000 (53 000 to 57 000)	−15·9% (−18·1 to −13·5)	121 000 (88 000 to 165 000)	−26·2% (−44·3 to −1·2)
		Tanzania	49 000 (45 000 to 54 000)	−3·7% (−8·5 to 1·8)	23 000 (18 000 to 29 000)	−19·1% (−35·3 to 5·2)	432 000 (417 000 to 449 000)	6·5% (3·2 to 9·6)	583 000 (461 000 to 738 000)	−25·2% (−41·6 to −2·8)
		Uganda	28 000 (25 000 to 31 000)	−15·4% (−20·4 to −9·8)	12 000 (10 000 to 16 000)	−36·0% (−51·5 to −14·4)	242 000 (233 000 to 251 000)	−12·2% (−14·5 to −9·4)	348 000 (273 000 to 441 000)	−36·7% (−52·1 to −16·3)
		Zambia	16 000 (14 000 to 17 000)	−1·2% (−6·8 to 4·9)	9000 (7000 to 12 000)	−11·6% (−32·6 to 14·3)	122 000 (118 000 to 126 000)	−2·4% (−5·2 to 0·6)	251 000 (189 000 to 328 000)	−14·8% (−37·4 to 10·7)
	Southern sub-Saharan Africa		89 000 (78 000 to 100 000)	−8·9% (−12·1 to −5·4)	54 000 (50 000 to 58 000)	14·8% (4·0 to 34·6)	789 000 (732 000 to 845 000)	−15·3% (−18·2 to −12·7)	1 325 000 (1 226 000 to 1 427 000)	4·6% (−4·2 to 18·2)
		Botswana	3000 (3000 to 3000)	−5·2% (−11·1 to 1·3)	1000 (1000 to 2000)	−43·2% (−57·1 to −22·1)	25 000 (24 000 to 26 000)	−3·7% (−7·0 to −0·3)	30 000 (24 000 to 38 000)	−43·8% (−57·9 to −25·1)
		Eswatini	1000 (1000 to 1000)	3·3% (−3·0 to 10·1)	700 (490 to 980)	−10·0% (−35·0 to 21·4)	8000 (8000 to 8000)	−0·6% (−3·6 to 2·7)	19 000 (13 000 to 27 000)	−7·7% (−33·5 to 27·6)
		Lesotho	2000 (2000 to 3000)	36·6% (28·6 to 45·7)	2000 (1000 to 2000)	43·4% (3·4 to 104·2)	14 000 (14 000 to 15 000)	19·5% (15·1 to 23·7)	48 000 (36 000 to 65 000)	50·3% (8·4 to 115·9)
		Namibia	3000 (2000 to 3000)	−16·7% (−21·7 to −11·1)	2000 (1000 to 2000)	−18·9% (−36·9 to 1·1)	19 000 (19 000 to 20 000)	−16·0% (−18·7 to −13·3)	40 000 (31 000 to 51 000)	−21·9% (−40·1 to −1·3)
		South Africa	69 000 (60 000 to 79 000)	−13·8% (−17·3 to −9·9)	39 000 (35 000 to 43 000)	16·4% (4·7 to 37·4)	621 000 (569 000 to 674 000)	−20·8% (−23·9 to −17·9)	944 000 (853 000 to 1 022 000)	0·1% (−8·3 to 11·4)
		Zimbabwe	11 000 (10 000 to 12 000)	14·6% (7·9 to 21·7)	9000 (7000 to 11 000)	39·1% (10·0 to 82·3)	102 000 (97 000 to 106 000)	10·3% (7·1 to 13·5)	244 000 (195 000 to 304 000)	50·0% (19·1 to 97·3)
	Western sub-Saharan Africa		365 000 (335 000 to 397 000)	−17·3% (−19·5 to −14·6)	204 000 (176 000 to 235 000)	−24·6% (−34·8 to −11·3)	3 531 000 (3 383 000 to 3 685 000)	−9·6% (−10·7 to −8·4)	5 641 000 (4 774 000 to 6 597 000)	−26·9% (−36·9 to −13·6)
		Benin	10 000 (9000 to 11 000)	−19·9% (−24·2 to −15·1)	6000 (5000 to 7000)	−22·7% (−35·7 to −3·8)	93 000 (90 000 to 97 000)	−14·1% (−16·3 to −11·8)	161 000 (133 000 to 194 000)	−25·8% (−39·0 to −6·9)
		Burkina Faso	14 000 (13 000 to 15 000)	−8·0% (−12·4 to −3·1)	8 000 (7 000 to 10 000)	−9·9% (−28·1 to 14·9)	131 000 (127 000 to 136 000)	−9·4% (−11·8 to −7·3)	230 000 (186 000 to 285 000)	−13·9% (−30·5 to 9·5)
		Cabo Verde	660 (610 to 720)	−3·7% (−8·6 to 1·9)	440 (360 to 530)	7·1% (−16·2 to 40·2)	7000 (7000 to 7000)	−6·8% (−9·0 to −4·6)	10 000 (8000 to 11 000)	−6·7% (−26·3 to 20·6)
		Cameroon	25 000 (23 000 to 27 000)	−6·6% (−11·0 to −1·7)	15 000 (11 000 to 20 000)	−7·1% (−28·6 to 24·2)	233 000 (226 000 to 241 000)	−4·2% (−6·7 to −1·8)	438 000 (325 000 to 575 000)	−9·3% (−30·7 to 20·6)
		Chad	13 000 (12 000 to 14 000)	−5·3% (−9·8 to −0·6)	8000 (6000 to 10 000)	4·3% (−19·3 to 34·3)	115 000 (111 000 to 119 000)	−5·8% (−8·2 to −3·5)	242 000 (188 000 to 302 000)	2·2% (−20·9 to 30·8)
		Côte d'Ivoire	23 000 (21 000 to 24 000)	−17·9% (−22·0 to −13·6)	13 000 (10 000 to 16 000)	−13·7% (−31·9 to 12·0)	224 000 (217 000 to 232 000)	−16·2% (−18·2 to −14·1)	378 000 (288 000 to 485 000)	−16·6% (−35·7 to 9·2)
		The Gambia	2000 (2000 to 2000)	−8·0% (−12·3 to −2·7)	1000 (1000 to 2000)	3·3% (−22·1 to 36·6)	19 000 (18 000 to 20 000)	−10·3% (−13·0 to −7·9)	38 000 (29 000 to 47 000)	−1·9% (−26·4 to 30·1)
		Ghana	42 000 (39 000 to 45 000)	−6·3% (−11·3 to −1·2)	25 000 (20 000 to 31 000)	−9·6% (−30·9 to 18·8)	422 000 (409 000 to 436 000)	−2·3% (−5·0 to 0·4)	694 000 (558 000 to 855 000)	−15·1% (−34·5 to 11·9)
		Guinea	12 000 (12 000 to 13 000)	−4·1% (−8·9 to 1·2)	8000 (6000 to 10 000)	−2·7% (−25·8 to 31·4)	108 000 (104 000 to 112 000)	−3·0% (−5·6 to −0·2)	215 000 (166 000 to 270 000)	−6·7% (−28·7 to 24·0)
		Guinea-Bissau	2000 (2000 to 2000)	−15·2% (−19·1 to −10·8)	1000 (1000 to 2000)	−13·8% (−33·8 to 9·0)	16 000 (16 000 to 17 000)	−12·9% (−15·1 to −10·7)	40 000 (31 000 to 50 000)	−20·1% (−39·3 to 1·1)
		Liberia	4000 (4000 to 4000)	−22·8% (−26·4 to −18·8)	3000 (2000 to 3000)	−11·3% (−31·3 to 16·9)	38 000 (37 000 to 39 000)	−18·1% (−20·0 to −16·1)	74 000 (57 000 to 96 000)	−15·9% (−35·4 to 11·5)
		Mali	14 000 (13 000 to 16 000)	−19·4% (−22·9 to −15·0)	9000 (7000 to 11 000)	−21·3% (−37·4 to −1·2)	138 000 (133 000 to 143 000)	−14·7% (−16·5 to −12·8)	253 000 (202 000 to 312 000)	−24·7% (−39·8 to −5·5)
		Mauritania	4000 (3000 to 4000)	−30·8% (−34·0 to −27·3)	2000 (2000 to 3000)	−32·4% (−47·9 to −11·3)	36 000 (35 000 to 38 000)	−24·6% (−26·4 to −22·4)	59 000 (45 000 to 77 000)	−37·3% (−51·6 to −19·1)
		Niger	16 000 (15 000 to 17 000)	−18·9% (−22·6 to −15·2)	9000 (7000 to 12 000)	−10·8% (−29·1 to 14·2)	143 000 (138 000 to 147 000)	−17·2% (−19·4 to −15·3)	259 000 (196 000 to 334 000)	−17·4% (−35·5 to 6·5)
		Nigeria	153 000 (138 000 to 170 000)	−22·9% (−25·5 to −20·0)	74 000 (61 000 to 92 000)	−39·7% (−51·0 to −22·7)	1 518 000 (1 415 000 to 1 633 000)	−10·3% (−11·9 to −8·6)	2 010 000 (1 637 000 to 2 544 000)	−41·2% (−53·2 to −23·9)
		São Tomé and Príncipe	250 (230 to 270)	−4·8% (−9·3 to 0·3)	120 (100 to 150)	−2·0% (−16·3 to 17·0)	3000 (2000 to 3000)	−3·9% (−6·4 to −1·6)	3000 (3000 to 4000)	−4·9% (−19·6 to 14·9)
		Senegal	14 000 (13 000 to 15 000)	−17·7% (−21·7 to −13·5)	10 000 (8000 to 12 000)	−13·2% (−32·2 to 8·7)	139 000 (135 000 to 144 000)	−14·9% (−17·0 to −12·8)	247 000 (199 000 to 304 000)	−20·1% (−37·6 to −0·3)
		Sierra Leone	8000 (7000 to 8000)	−12·4% (−16·6 to −7·4)	5000 (4000 to 7000)	−10·8% (−28·7 to 15·1)	77 000 (74 000 to 79 000)	−10·3% (−12·9 to −8·2)	148 000 (113 000 to 194 000)	−14·3% (−32·0 to 11·6)
		Togo	7000 (7000 to 8000)	−14·4% (−18·5 to −10·2)	5000 (4000 to 6000)	−5·5% (−26·0 to 21·4)	71 000 (69 000 to 74 000)	−13·7% (−15·7 to −11·2)	142 000 (107 000 to 178 000)	−9·6% (−30·2 to 15·9)

Data in parentheses are 95% uncertainty intervals. DALYs=disability-adjusted life-years.

**Figure 1:**
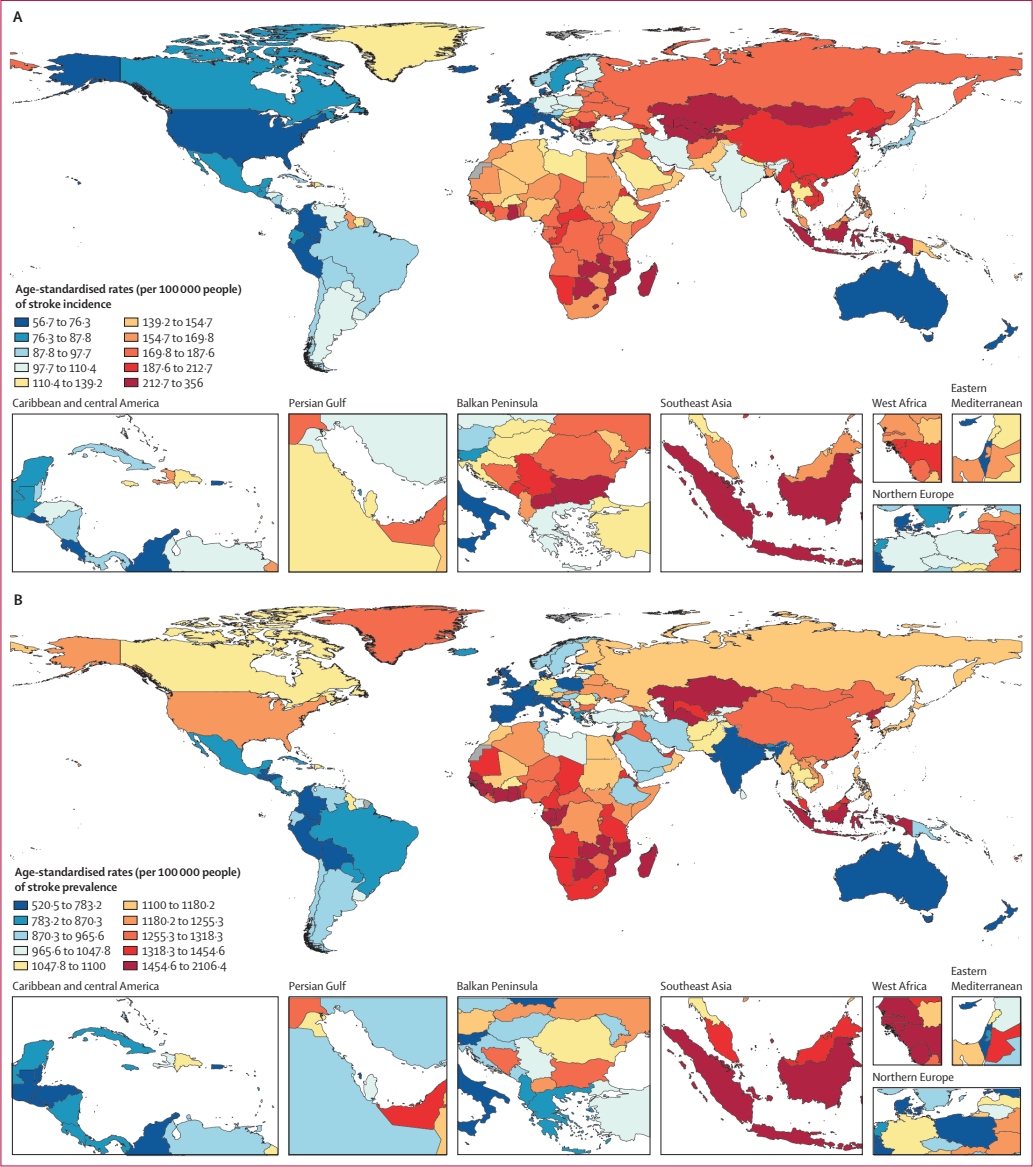

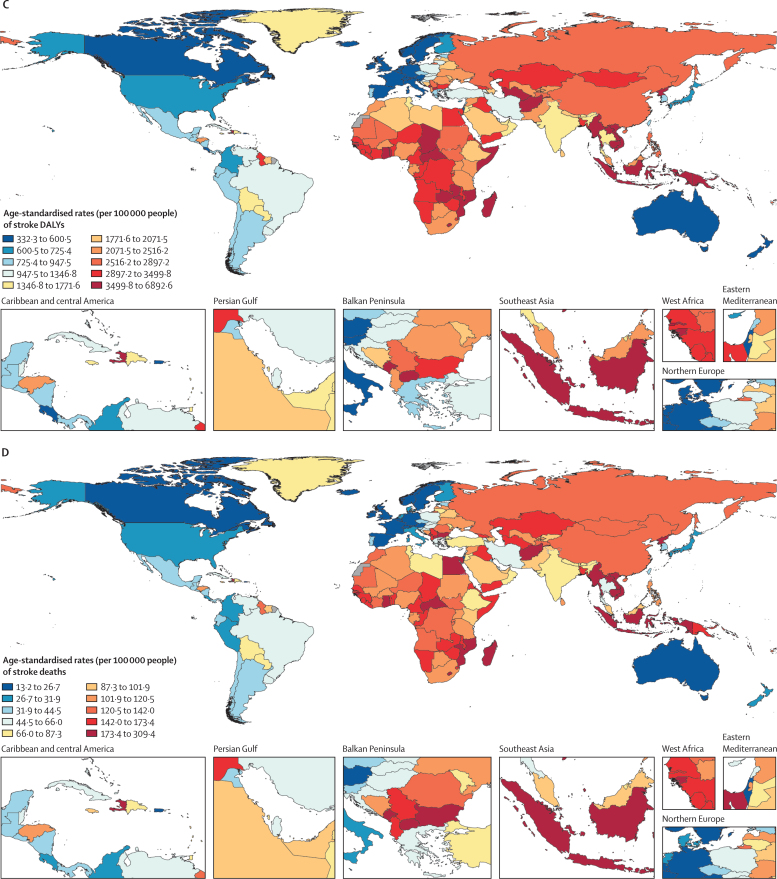
Global age-standardised rates (per 100 000 people) of stroke incidence (A), prevalence (B), DALYs (C), and deaths (D) for both sexes, 2021 DALYs=disability-adjusted life-years.

In 2021, 83·3% incident, 76·7% prevalent, and 87·2% fatal strokes, and 89·4% stroke-related DALYs occurred in all low-income and middle-income countries (LMICs) combined (appendix pp 48–49). We also observed geographical differences in age-standardised stroke incidence, with the lowest in Luxembourg (57·7 [95% UI 53·5–62·1] per 100 000) and highest in the Solomon Islands (355·0 [332·7–378·1] per 100 000); prevalence, with the highest in Ghana (2045·8 [1977·3–2120·1] per 100 000) and lowest in Cyprus (521·5 [495·7–553·5] per 100 000); deaths, with the lowest in Singapore (14·2 [12·3–15·6] per 100 000) and highest in North Macedonia (277·4 [235·5–321·2] per 100 000); and in DALY rates, with the lowest in Switzerland (333·3 [291·0–368·8] per 100 000) and highest in Nauru (6100·0 [4917·8–7576·1] per 100 000). Overall, the highest stroke burden (as measured by age-standardised incidence, prevalence, death, and DALY rates) in 2021 was observed in east Asia, central Asia, and sub-Saharan regions and lowest in high-income North America, Australasia, and Latin America regions, with the majority of the stroke burden in middle SDI, high-middle, and low-middle SDI regions (figure 1; appendix pp 50–85).

### Burden by pathological type of stroke

Ischaemic stroke constituted the largest proportion of all incident strokes (7·8 million [95% UI 6·7–8·9], or 65·3% [62·4–67·7] of all strokes), followed by intracerebral haemorrhage (3·4 million [3·1–3·8] incident events, or 28·8% [28·3–28·8] of all strokes). However, the absolute number of DALYs due to intracerebral haemorrhage (79·5 million [72·7–85·2], or 49·6% [49·3–49·8] of total DALYs due to stroke) was greater than the number of DALYs due to ischaemic stroke (70·4 million [64·1–76·0], or 43·8% [43·5–44·3]). In 2021, subarachnoid haemorrhage occurred in 0·7 million (0·6–0·8) people (5·8% [5·7–6·0] of all strokes), and there were 10·6 million (9·4–12·1) DALYs due to subarachnoid haemorrhage (6·6% of DALYs from all strokes combined). Similar to total stroke, differences were observed for age-standardised rates for the three pathological types of stroke and their trends from 1990 to 2021 globally and by SDI (appendix pp 50–85, 208): rates of incident and fatal stroke were highest for ischaemic stroke (92·4 [79·8–105·8] per 100 000 and 44·2 [39·5–47·8] per 100 000, respectively) followed by intracerebral haemorrhage (40·8 [36·2–45·2] per 100 000 and 39·1 [35·4–42·6] per 100 000, respectively) and subarachnoid haemorrhage (8·3 [7·3–9·5] per 100 000 and 4·2 [3·7–4·8] per 100 000, respectively).

In 2021, there were large variations in the proportion of ischaemic stroke and intracerebral haemorrhage between high-income countries and LMICs (appendix pp 100–101). Whereas in high-income countries ischaemic stroke constituted 74·9% (95% UI 72·3−84·1) and intracerebral haemorrhage constituted 17·8% (17·3−17·9) of all incident strokes, in all LMICs combined these stroke subtypes constituted 63·4% (53·6−73·7) and 31·1% (30·2−31·3), respectively. Among all LMICs combined, the proportion of intracerebral haemorrhage was highest in low-income countries (36·9% [36·5−37·1]). The proportion of subarachnoid haemorrhage in high-income countries (7·3% [7·2−8·3]) was higher than that in all LMICs combined (5·5% [5·4−5·7]).

### Trends in stroke burden by age, sex and geographical location

Among 11·9 million new strokes in 2021, 6·3 million (95% UI 5·6 to 7·0; or 52·6% [52·4 to 53·1]) occurred in males and 5·7 million (5·1 to 6·3; or 47·4% [47·3 to 47·6]) in females; the corresponding sex distribution of prevalent stroke was 51·0% (47·8 million [45·3 to 50·6]) for males and 49·0% (46·0 million [43·5 to 48·8]) for females; that for deaths from stroke was 52·1% (3·8 million [3·4 to 4·1]) for males and 47·9% (3·5 million [3·1 to 3·8] for females; and that for stroke-related DALYs was 55·0% (88·3 million [80·6 to 97·2]) for males and 45·0% (72·2 million [65·6 to 78·2) for females (table 1; appendix pp 138–139). From 1990 to 2021, the age-standardised incidence, prevalence, death, and DALY rates (table 1) of stroke and its pathological types were reduced virtually across all World Bank country income levels (except for ischaemic stroke incidence and prevalence in upper-middle-income countries, where the rates were increased by 1% [–4 to 5] for ischaemic stroke incidence and 11% [8 to 14] for ischaemic stroke prevalence). Although there was a trend towards lower age-standardised stroke burden rates (incidence, prevalence, deaths, and DALYs) across all quintiles of the SDI, there was a stagnation in the reduction of incidence rates from 2015 onwards, and even some increase in the prevalence rates in high-middle SDI countries from 2020 to 2021 (appendix p 209). Similar trend patterns were observed in seven GBD super-regions, with more prominent increases in age-standardised incidence and prevalence rates after 2015 in southeast Asia, east Asia, and Oceania (appendix p 206).

Although from 1990 to 2021 there was a decrease in the age-standardised incidence (–21·8% [95% UI–23·7 to –19·8]), prevalence (–8·5% [–9·7 to –7·3]), death (–39·4% [–44·0 to –34·6]), and DALY (–38·7% [–43·4 to –34·0]) stroke rates, increases were seen over that period in the numbers of people who had a new stroke (70·2% [65·9 to 74·6]), survived stroke (86·1% [83·0 to 89·4]), died from stroke (44·1% [32·3 to 56·0]), and who died or remained disabled from stroke (as measured by DALYs; 32·2% [21·7 to 42·7]; table 1; appendix pp 100–101). The percentage decline in age-standardised stroke incidence rates in the 2019–2021 period (–1·8% [–2·8 to –0·6]) was smaller than that for the overall 2010–21 period (–3·1% [–4·2 to –2·0]).

Although all-age (not age-standardised) stroke incidence, death, and DALY rates were substantially reduced in people aged 70 years or older between 1990 and 2021 (–18·2% [95% UI –21·3 to – 14·6] incidence rate, –34·2% [–39·4 to –29·3] death rate, and –35·6% [–40·2 to –30·8] DALY rate), and all-age prevalence rate in this age group did not change over this period (–1·0 [–3·1 to 1·2]), all-age incidence increased by 4·1% (0·9 to 7·6), prevalence increased in people younger than 70 years by 14·8% (13·1 to 16·8), and death and DALY rates were reduced in people younger than 70 years by 17·4% (–25·0 to –8·9) and 19·0% (–26·0 to –11·6), respectively (appendix p 140). Similar patterns were observed for all-age incidence, prevalence, death, and DALY rates of ischaemic stroke for both age groups (<70 years and ≥70 years). Of the three pathological types of stroke, only all-age subarachnoid haemorrhage prevalence rates increased in people younger than 70 years, by 3·4% (1·3 to 5·5), whereas all-age incidence, prevalence, death, and DALY rates of intracerebral haemorrhage and incidence, death, and DALY rates of subarachnoid haemorrhage were reduced in both age groups (appendix pp 88, 210–211).

### Contribution of risk factors to stroke-related DALYs

Globally, the total number of stroke-related DALYs due to risk factors increased substantially from 1990 (100·1 million [95% UI 92·7 to 107·8]) to 2021 (135·0 million [122·0 to 147·7]), but there was no substantial change in the age-standardised stroke DALYs attributable to risk factors (–0·5% [–2·4 to 1·1]). In 2021, 84·1% (77·8 to 88·8) of DALYs from stroke were attributed to the 23 risk factors analysed (table 2), with the largest proportions of attributable risks for total stroke, ischaemic stroke, intracerebral haemorrhage, and subarachnoid haemorrhage observed in eastern Europe, Asia, and sub-Saharan Africa (appendix p 212).

**Table 2:** Stroke-related DALYs associated with risk factors and their clusters by World Bank country income level, for both sexes, 2021

	**Low-income countries**		**Lower-middle-income countries**		**Upper-middle-income countries**		**High-income countries**	
	Absolute number	Percentage	Absolute number	Percentage	Absolute number	Percentage	Absolute number	Percentage
**Air pollution and environmental risks**								
Ambient particulate matter pollution	655 000 (449 000 to 922 000)	7·3% (4·9 to 9·9)	9 103 000 (5 695 000 to 12 188 000)	15·3% (9·5 to 20·2)	15 557 000 (10 431 000 to 20 052 000)	20·9% (14·8 to 25·7)	1 448 000 (1 043 000 to 1 897 000)	8·8% (6·5 to 11·5)
High ambient temperature	169 000 (78 000 to 305 000)	1·7% (0·8 to 3·1)	1 141 000 (365 000 to 2 202 000)	1·9% (0·6 to 3·7)	407 000 (−142 000 to 1 340 000)	0·6% (−0·2 to 1·8)	78 000 (−25 000 to 222 000)	0·6% (−0·1 to 1·5)
Household air pollution from solid fuels	3 492 000 (2 721 000 to 4 278 000)	38·3% (31·7 to 44·6)	11 410 000 (7 024 000 to 17 273 000)	19·1% (11·7 to 28·3)	3 244 000 (479 000 to 10 588 000)	4·3% (0·6 to 14·3)	13 000 (0 to 134 000)	0·1% (0·0 to 0·8)
Lead exposure	822 000 (−106 000 to 1 793 000)	9·3% (−1·3 to 20·9)	4 965 000 (−672 000 to 10 922 000)	8·4% (−1·1 to 18·8)	5 572 000 (−727 000 to 12 374 000)	7·4% (−1·0 to 16·5)	656 000 (−87 000 to 1 485 000)	3·7% (−0·5 to 8·5)
Low ambient temperature	299 000 (248 000 to 363 000)	3·2% (2·8 to 3·8)	1 248 000 (801 000 to 1 801 000)	2·1% (1·4 to 3·0)	4 939 000 (4 153 000 to 5 937 000)	6·7% (6·0 to 7·5)	1 119 000 (964 000 to 1 295 000)	6·3% (5·6 to 7·2)
**Dietary risks**								
Alcohol use	302 000 (61 000 to 603 000)	3·2% (0·7 to 6·3)	2 018 000 (483 000 to 3 902 000)	3·2% (0·8 to 6·1)	4 710 000 (1 122 000 to 8 961 000)	6·2% (1·5 to 11·8)	1 399 000 (251 000 to 2 856 000)	8·1% (1·6 to 16·0)
Diet high in processed meat	8 000 (2 000 to 14 000)	0·1% (0·0 to 0·2)	58 000 (13 000 to 105 000)	0·1% (0·0 to 0·2)	181 000 (42 000 to 326 000)	0·2% (0·1 to 0·4)	189 000 (46 000 to 334 000)	1·1% (0·3 to 1·9)
Diet high in red meat	−162 000 (−607 000 to 250 000)	−1·3% (−5·1 to 2·1)	−905 000 (−3 475 000 to 1 258 000)	−1·3% (−5·0 to 1·9)	−3 528 000 (−15 465 000 to 4 994 000)	−4·7% (−20·0 to 6·8)	−561 000 (−2 380 000 to 834 000)	−4·3% (−19·0 to 6·4)
Diet high in sodium	528 000 (71 000 to 1 443 000)	6·1% (0·8 to 16·0)	4 558 000 (574 000 to 11 393 000)	7·5% (0·9 to 19·0)	11 095 000 (3 669 000 to 22 286 000)	14·3% (4·8 to 27·5)	1 207 000 (159 000 to 3 109 000)	7·1% (1·0 to 18·0)
Diet high in sugar-sweetened beverages	2000 (1000 to 3000)	0·0% (0·0 to 0·0)	24 000 (12 000 to 39 000)	0·0% (0·0 to 0·1)	64 000 (31 000 to 101 000)	0·1% (0·0 to 0·1)	54 000 (26 000 to 85 000)	0·3% (0·2 to 0·5)
Diet low in fibre	240 000 (−51 000 to 502 000)	2·1% (−0·5 to 4·4)	2 357 000 (−567 000 to 4 845 000)	3·5% (−0·8 to 7·2)	1 175 000 (−243 000 to 2 521 000)	1·6% (−0·3 to 3·4)	298 000 (−59 000 to 643 000)	2·2% (−0·5 to 4·7)
Diet low in fruits	784 000 (38 000 to 1 393 000)	7·1% (0·4 to 12·7)	5 318 000 (395 000 to 9 047 000)	7·9% (0·7 to 13·8)	2 969 000 (183 000 to 5 711 000)	3·9% (0·2 to 7·2)	550 000 (49 000 to 995 000)	4·1% (0·3 to 7·2)
Diet low in omega-6 polyunsaturated fatty acids	1000 (0 to 2000)	0·0% (0·0 to 0·0)	7000 (2000 to 13 000)	0·0% (0·0 to 0·0)	8000 (2000 to 16 000)	0·0% (0·0 to 0·0)	2000 (0 to 3000)	0·0% (0·0 to 0·0)
Diet low in vegetables	689 000 (97 000 to 1 189 000)	6·3% (1·1 to 11·0)	1 477 000 (374 000 to 2 504 000)	2·3% (0·6 to 3·8)	278 000 (106 000 to 461 000)	0·4% (0·1 to 0·6)	93 000 (18 000 to 167 000)	0·6% (0·3 to 1·0)
Diet low in wholegrains	187 000 (−202 000 to 497 000)	1·8% (−1·9 to 5·1)	1 000 000 (−1 041 000 to 2 710 000)	1·5% (−1·5 to 4·4)	1 595 000 (−1 612 000 to 4 382 000)	2·1% (−2·1 to 5·8)	340 000 (−335 000 to 1 083 000)	2·3% (−2·4 to 6·7)
**Physical activity**								
Low physical activity	163 000 (68 000 to 272 000)	1·7% (0·6 to 3·0)	1 191 000 (427 000 to 2 092 000)	2·0% (0·5 to 3·7)	1 587 000 (361 000 to 3 094 000)	2·1% (0·3 to 4·3)	415 000 (−56 000 to 951 000)	2·5% (0·4 to 5·0)
**Tobacco smoking**								
Second-hand smoke	320 000 (212 000 to 434 000)	3·1% (2·1 to 4·2)	2 694 000 (1 843 000 to 3 599 000)	4·3% (3·0 to 5·7)	3 548 000 (2 410 000 to 4 722 000)	4·8% (3·3 to 6·4)	402 000 (270 000 to 549 000)	2·8% (1·9 to 3·7)
Smoking	780 000 (627 000 to 941 000)	7·3% (6·2 to 8·5)	7 248 000 (6 162 000 to 8 491 000)	11·1% (9·5 to 12·6)	12 606 000 (10 200 000 to 15 551 000)	16·4% (13·9 to 19·0)	1 870 000 (1 573 000 to 2 225 000)	13·1% (11·2 to 15·0)
**Physiological factors**								
High BMI	357 000 (31 000 to 752 000)	3·4% (0·3 to 7·0)	2 276 000 (202 000 to 4 653 000)	3·5% (0·3 to 7·0)	3 848 000 (294 000 to 8 127 000)	5·1% (0·4 to 10·5)	1 197 000 (90 000 to 2 420 000)	8·2% (0·5 to 16·4)
High fasting plasma glucose	672 000 (491 000 to 873 000)	8·4% (6·4 to 10·5)	5 510 000 (4 189 000 to 6 899 000)	10·1% (7·9 to 12·4)	7 838 000 (6 087 000 to 9 926 000)	10·6% (8·3 to 13·2)	2 433 000 (1 909 000 to 2 978 000)	13·0% (10·5 to 15·5)
High LDL cholesterol	857 000 (304 000 to 1 429 000)	9·7% (3·3 to 16·2)	6 557 000 (2 425 000 to 10 763 000)	11·4% (3·9 to 19·2)	10 521 000 (3 652 000 to 17 446 000)	14·1% (4·9 to 23·0)	3 019 000 (1 003 000 to 5 043 000)	17·3% (6·1 to 27·8)
High systolic blood pressure	5 004 000 (3 684 000 to 6 327 000)	55·5% (41·1 to 66·2)	35 018 000 (26 696 000 to 42 407 000)	59·0% (44·3 to 69·9)	42 461 000 (30 731 000 to 54 201 000)	56·7% (42·4 to 68·4)	9 286 000 (6 804 000 to 11 360 000)	53·3% (39·5 to 64·0)
Kidney dysfunction	844 000 (592 000 to 1 103 000)	9·3% (6·9 to 11·7)	6 603 000 (4 928 000 to 8 309 000)	11·1% (8·2 to 14·0)	6 119 000 (4 382 000 to 8 066 000)	8·1% (5·8 to 10·5)	1 430 000 (946 000 to 1 948 000)	7·9% (5·5 to 10·4)
**Cluster of risk factors**								
Air pollution*	4 147 000 (3 281 000 to 5 058 000)	45·5% (37·9 to 52·7)	20 516 000 (16 091 000 to 24 931 000)	34·3% (27·8 to 41·4)	18 805 000 (13 812 000 to 24 803 000)	25·2% (19·6 to 32·0)	1 461 000 (1 053 000 to 1 920 000)	8·9% (6·6 to 11·8)
Behavioural risks†	3 009 000 (1 856 000 to 4 044 000)	30·0% (18·4 to 40·5)	20 601 000 (14 385 000 to 26 447 000)	32·5% (22·6 to 42·5)	28 745 000 (20 905 000 to 38 190 000)	37·6% (27·8 to 48·4)	5 093 000 (3 497 000 to 6 929 000)	31·9% (23·1 to 41·8)
Dietary risks‡	1 887 000 (585 000 to 2 974 000)	18·7% (5·9 to 31·2)	10 873 000 (3 590 000 to 18 064 000)	17·0% (5·6 to 29·0)	12 749 000 (4 384 000 to 23 122 000)	16·5% (5·5 to 29·2)	1 846 000 (649 000 to 3 690 000)	11·1% (3·4 to 21·5)
Environmental or occupational risks§	4 828 000 (3 842 000 to 5 781 000)	53·0% (44·2 to 60·8)	24 989 000 (19 266 000 to 29 973 000)	41·9% (33·0 to 50·3)	26 451 000 (19 722 000 to 33 653 000)	35·5% (27·7 to 43·4)	3 093 000 (2 287 000 to 3 958 000)	18·1% (13·6 to 22·7)
Metabolic risks¶	5 925 000 (4 692 000 to 7 204 000)	65·9% (54·9 to 74·8)	41 558 000 (34 711 000 to 47 433 000)	70·3% (59·7 to 78·7)	51 450 000 (41 291 000 to 61 419 000)	68·9% (57·4 to 77·9)	11 980 000 (9 919 000 to 13 788 000)	68·7% (57·9 to 77·5)
Tobacco smoke‖	1 067 000 (830 000 to 1 318 000)	10·2% (8·2 to 12·2)	9 631 000 (7 866 000 to 11 484 000)	14·9% (12·3 to 17·5)	15 602 000 (12 447 000 to 19 422 000)	20·4% (16·8 to 24·0)	2 209 000 (1 825 000 to 2 669 000)	15·4% (12·8 to 17·8)
Combined risk factors**								
All risk factors	7 812 000 (6 670 000 to 8 973 000)	85·4% (79·3 to 89·2)	51 119 000 (46 489 000 to 55 450 000)	85·7% (79·7 to 89·8)	62 283 000 (53 668 000 to 70 765 000)	83·6% (76·9 to 88·7)	13 633 000 (11 938 000 to 15 151 000)	79·2% (71·6 to 85·4)

Data in parentheses are 95% uncertainty intervals. Percentages and number of DALYs are not mutually exclusive. The sum of percentages and number of DALYs in the columns exceeds the totals for all risk factors combined because the effect of many of these risk factors are mediated partly or wholly through other risk factors. 0% represents very low numbers. DALYs=disability-adjusted life-years.

* Air pollution cluster includes ambient PM_2·5_ pollution and household air pollution.

† Behavioural risks cluster includes smoking (including second-hand smoking), dietary risks (diet high in sodium, diet high in processed meat diet, high in red meat, diet high in sugar-sweetened beverages, diet low in omega-6 polyunsaturated fatty acids, diet low in fruits, diet low in vegetables, and diet low in wholegrains), alcohol use, and low physical activity.

‡ Dietary risks cluster includes diet high in sodium, diet high in processed meat diet, high in red meat, diet high in sugar-sweetened beverages, diet low in omega-6 polyunsaturated fatty acids, diet low in fruits, diet low in vegetables, and diet low in whole grains.

§ Environmental risks cluster includes air pollution cluster, low ambient temperature, high ambient temperature, and lead exposure.

¶ Metabolic risks cluster includes high fasting plasma glucose, high LDL cholesterol, high systolic blood pressure, high BMI, and kidney dysfunction.

‖ Tobacco smoke includes smoking and second-hand smoking.

** Age-standardised total percentage of DALYs due to all risk factors combined.

At level 1 of the GBD risk factors hierarchy (table 2, appendix pp 141–253), metabolic risk factors contributed most to the stroke-related DALYs (range 66–70%) across all World Bank country income levels, followed by the environmental risk cluster in low-income, lower-middle-income, and upper-middle-income countries (range 35–53%), and behavioural risks (range 30–38%) across different income level countries. Stroke burden associated with the environmental or occupational risks was lowest in high-income countries (18·1% [95% UI 13·6 to 22·7]). Similarly, regions with higher SDI (appendix pp 141–143) had a larger contribution of metabolic and behavioural risks to stroke-related DALYs, whereas environmental risks most prominently contributed to stroke-related DALYs in lower SDI quintiles. From 1990 to 2021, the age-standardised proportion of stroke DALYs attributable to risk factors increased in north Africa and the Middle East (6·8% [4·1 to 11·5]) and sub-Saharan Africa (3·3% [1·8 to 5·3]), but did not change in south Asia (0·4% [–1·0 to 1·9]) and southeast Asia, east Asia, and Oceania (–0·9% [–4·2 to 1·9]), and decreased in central Europe, eastern Europe, and central Asia (–2·0% [–3·8 to –0·7]) and Latin America and the Caribbean (–5·0% [–9·1 to –2·3]), as well as high-income GBD regions (–7·3% [–10·1 to –5·0]).

**Figure 2:**
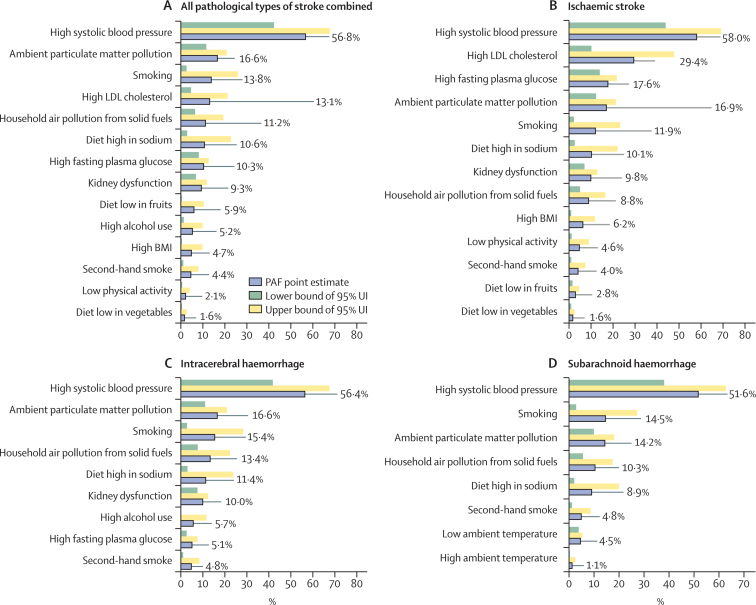
Most individually significant risk factors for total stroke (A), ischaemic stroke (B), intracerebral haemorrhage (C), and subarachnoid haemorrhage (D), as measured by the PAF of stroke DALYs attributable to the risk factors, for both sexes DALYs=disability-adjusted life-years. PAF=population attributable fraction.

Globally, of the 23 risk factors analysed, 14 individually significant risk factors for stroke were high systolic blood pressure (56·8% [95% UI 42·5–68·0] attributable DALYs), ambient particular matter (16·6% [11·5–20·9]), smoking (13·8% [2·5–26·0]), high LDL cholesterol (13·1% [4·6–21·3]), household air pollution (11·2% [6·4–19·3]), diet high in sodium (10·6% [2·8–22·8]), high fasting plasma glucose (10·3% [8·1–12·6]), kidney disfunction (9·3% [6·8–11·8]), diet low in fruits (5·9% [0·4–10·4]), high alcohol use (5·2% [1·3–9·8]), high BMI (4·7% [0·4–9·8]), second-hand smoking (4·4% [1·0–7·9]), low physical activity (2·1% [0·5–3·9]), and diet low in vegetables (1·6% [0·4–2·6]; figure 2).

**Figure 3:**
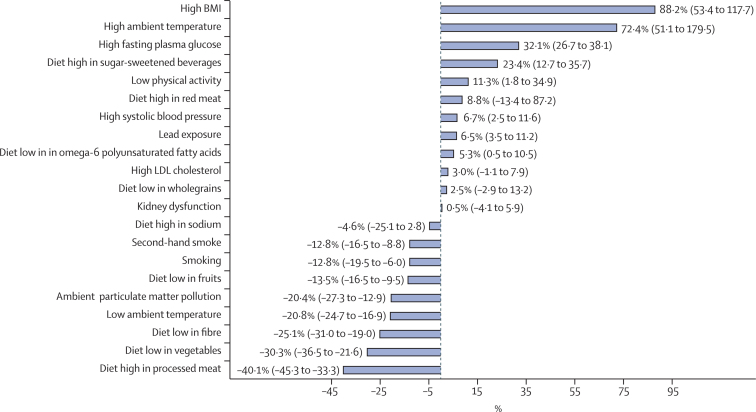
Trends in the PAF of stroke DALYs due to risk factors, for both sexes, 1990–2021 Data in parentheses are 95% uncertainty intervals. DALYs=disability-adjusted life-years. PAF=population attributable fraction.

**Figure 4:**
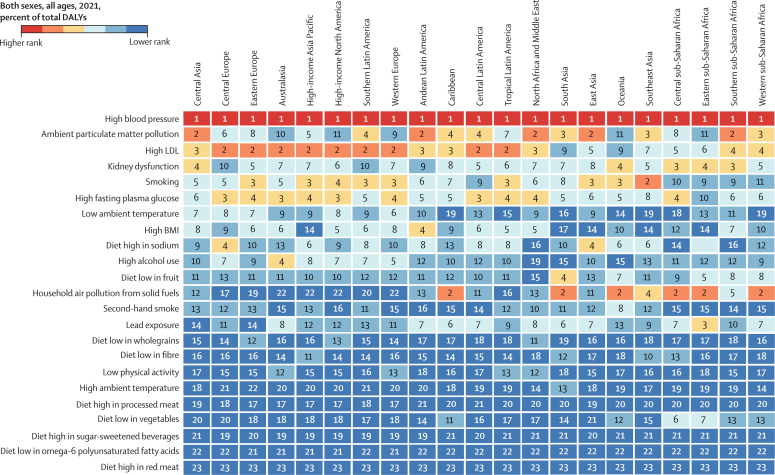
Ranking of age-standardised stroke DALYs attributable to risk factors by 21 GBD regions, for both sexes, 2021 DALYs=disability-adjusted life-years. GBD=Global Burden of Diseases, Injuries, and Risk Factors Study.

Stroke attributable to metabolic risks constituted 68·8% (95% UI 57·6 to 77·5) of all strokes, environmental risks constituted 36·7% (29·0 to 44·2), and behavioural risks constituted 35·2% (26·9 to 44·7). Although the proportion of stroke DALYs attributable to metabolic risks increased from 1990 to 2021 by 6·7% (3·8 to 10·0; mainly because of the increase in the burden attributable to high BMI, high fasting plasma glucose, and high systolic blood pressure), proportions of stroke DALYs attributable to behavioural risks decreased by 8·0% (–13·6 to –3·4) and those due to environmental risks by 14·8% (–21·6 to –8·7), mainly because of the decrease in the burden attributable to diet high in processed meat, diet low in vegetables, diet low in fibre, low ambient temperature, particulate matter pollution, diet low in fruits, and smoking (figure 3). However, from 1990 to 2021, there was a substantial increase in the stroke DALYs attributable to high ambient temperature, high fasting plasma glucose, diet high in sugar-sweetened beverages, low physical activity, diet high in red meat, lead exposure, and diet low in omega-6 polyunsaturated fatty acids. There were noticeable geographical and regional variations in the PAF of the risk factors for ischaemic stroke, intracerebral haemorrhage, subarachnoid haemorrhage, and all stroke types combined (appendix p 264), as well as in the ranking of PAFs of age-standardised stroke DALYs attributable to risk factors by 21 GBD regions (figure 4). For the PAF of risk factors by pathological type of stroke, SDI, 21 GBD regions, and 204 countries and territories were used (appendix pp 89–201). Unlike the PAF of risk factors for total stroke, high alcohol use was not associated with ischaemic stroke-related DALYs (appendix pp 92–95, 126–149, 199), and diet low in fruits and vegetables and high BMI were not associated with intracerebral haemorrhage-related DALYs (appendix pp 92–95, 150–173, 200). Unlike ischaemic stroke and intracerebral haemorrhage, non-optimal ambient temperature appeared to be associated with the subarachnoid haemorrhage-related DALYs, with the greater contribution of low ambient temperature (4·5% [3·8 to 5·3]) than high ambient temperature (1·1% [0·2 to 2·5]). Other substantial risk factors for subarachnoid haemorrhage (appendix pp 151−153) were second-hand smoking (4·7% [3·2 to 6·2]), diet high in sodium (8·9% [2·0 to 19·8]), household air pollution from solid fuels (10·3% [5·5 to 17·4]), ambient particulate matter pollution (14·2% [9·8 to 18·0]), smoking (14·5% [2·7 to 27·2]), and high systolic blood pressure (51·6% [38·0 to 62·6]).

## Discussion

In 2021, stroke remained the second most common cause (after ischaemic heart disease) of death and the third most common cause of DALYs (after ischaemic heart disease and neonatal disorders) among non-communicable disorders (NCDs) globally. However, at level 3 of the GBD all-cause hierarchy, stroke was the third most common cause of death (after ischaemic heart disease and COVID-19) and the fourth most common cause of DALYs (after COVID-19, ischaemic heart disease, and neonatal disorders), with the bulk of the stroke burden in LMICs and countries with lower SDI. Consistent with previous studies,^5^, ^17^ this study showed disparities in stroke burden (including almost two times greater proportion of intracerebral haemorrhage in LMICs than in high-income countries)^18^ and risk factors by GBD region, country, country income level, and SDI quintiles, as well as an overall trend towards decreasing age-standardised stroke incidence, prevalence, and DALY rates from 1990 to 2021. Given the leading role of arterial hypertension in the occurrence of intracerebral haemorrhage,^19^ the greater prevalence and poorer control of hypertension in LMICs^20^ than in high-income countries are the most likely causes of the differences in the proportion of intracerebral haemorrhage in those countries. Similar proportions of subarachnoid haemorrhage in high-income countries and LMICs are likely to be related to the significant reduction in tobacco smoking prevalence that we observed since 2010 in high-income countries, with almost no change in tobacco smoking prevalence in LMICs.^21^ However, the current study documented a stagnation in the reduction of age-standardised incidence rates from 2015 onwards, and even some increase in the age-standardised stroke incidence, death, prevalence, and DALY rates in southeast Asia, east Asia, and Oceania, and countries with lower SDI from 2015 onwards. Globally, there was also an increase in all-age incidence and prevalence rates in people younger than 70 years, whereas there was a reduction in all-age stroke incidence, prevalence, death, and DALY rates in people aged 70 years or older. A trend towards increasing incidence and prevalence rate of cardiovascular diseases (including stroke) in people aged 15–39 years globally^22^ and stroke incidence rates in people younger than 55 years^3^ versus older people has also been shown in previous systematic reviews and is likely to be related to the increase in prevalence of arterial hypertension^23^, ^24^ (including poorly controlled and uncontrolled hypertension),^25^ overweight or obesity,^26^, ^27^, ^28^ and type 2 diabetes^29^ in young adults, especially in LMICs.^24^, ^26^, ^27^, ^28^, ^29^ This situation is complicated by the fact that a large proportion of young adults with vascular risk factors, arterial hypertension,^30^ and dyslipidaemia^31^ remain under-treated owing at least partly to the widespread use of absolute cardiovascular disease risk^32^ treatment thresholds.^3^ The observed slowing down of the percentage of decline of age-standardised stroke incidence rates in 2019–21 is likely to be related to the decreased hospital admissions of patients with acute stroke that was observed over the COVID-19 period in many countries.^33^

Apart from population growth and ageing,^1^, ^34^ other factors responsible for the increased burden of stroke, in terms of absolute numbers in the world, are likely to be related to the insufficient effectiveness of the currently used primary stroke and cardiovascular disease prevention strategies^35^, ^36^ as well as the disparities and major gaps in stroke service provision and accessibility, and workforce of stroke care providers in many countries (especially LMICs).^17^, ^37^, ^38^ Although stroke is highly preventable, globally there were substantial increases in DALYs attributable to high BMI, high ambient temperature, high fasting plasma glucose, diet high in sugar-sweetened beverages, low physical activity, high systolic blood pressure, and diet low in polyunsaturated omega-6 fatty acids, suggesting the growing role of these environmental and behavioural risks in the stroke burden. However, from 1990 to 2021, we also observed a reduction of PAF due to diet high in processed meat, diet low in vegetables, diet low in fibre, low ambient temperature, ambient particulate matter pollution, diet low in fruits, and smoking, suggesting effectiveness of the strategies towards reduction of the exposure to these risk factors. The observed increase in the age-standardised proportion of stroke DALYs attributable to risk factors in north Africa and the Middle East and sub-Saharan Africa regions might reflect a failure in the control of stroke risk factors. However, in central Europe, eastern Europe, central Asia, Latin America and the Caribbean, and high-income GBD regions, this might reflect a success in the control of stroke risk factors.

This study is, to our knowledge, the first to show the large contribution of ambient particulate matter pollution and household air pollution from solid fuels to subarachnoid haemorrhage DALYs, with a similar PAF to that of smoking. A close relationship between ambient air pollution and subarachnoid haemorrhage mortality was found in some studies.^39^, ^40^, ^41^ Air pollution in 2021 appeared to be highly important to other types of stroke and also caused 11·9% (95% UI 10·0–13·8) of total deaths from all causes, making it the second largest cause of deaths from all causes globally (after high systolic blood pressure) and the second leading cause of DALYs (8·2% [6·9–9·6]) from all causes (after malnutrition).^42^ These findings are in line with research showing that rises in ambient temperature (including heatwaves) and climate change are associated with increased stroke morbidity and mortality.^43^, ^44^ Because ambient air pollution is reciprocally associated with the ambient temperature and climate change,^44^ all of which synergistically influence cardiovascular disease (including stroke) occurrence^44^, ^45^, ^46^ and overall health,^47^, ^48^ the importance of urgent climate actions and measures to reduce ambient air pollution cannot be overestimated.^47^, ^48^ Experts have recommended that governments increase implementation of a clean-energy economy, promote unprocessed plant-based food choices,^44^ and globally phase out industrialised animal farming.^49^

Every member state of the UN has committed to meeting the Sustainable Development Goals (SDGs), but currently few countries are on target to achieve SDG 3.4, which is to reduce by a third premature mortality from NCDs through prevention and treatment and promote mental health and wellbeing by 2030. By implementing and monitoring the World Stroke Organization–*Lancet Neurology* Commission's recommendations,^38^ the global burden of stroke would be reduced drastically this decade and beyond. Not only would this substantial reduction enable SDG 3.4, as well as other key SDGs, to be met, it would improve brain health and the overall wellbeing of millions of people across the globe. One of the most common problems in implementing stroke prevention and care recommendations is the scarcity of funding. The World Stroke Organization–*Lancet Neurology* Commission on stroke^38^ recommends introducing legislative regulations and taxation of unhealthy products by each government in the world. Such taxation would not only reduce consumption of these products and, therefore, lead to the reduction of burden from stroke and other major NCDs,^50^, ^51^, ^52^ but also generate a large revenue^50^ sufficient to fund prevention programmes and services for stroke and other major disorders, reduce poverty and inequality in health service provision, improve wellbeing of the population, and boost local economies.

The main strength of this study is the extended number of data sources included in the analysis that allowed us to generate more accurate and up-to-date stroke burden and risk factor estimates. This allows evidence-based health-care planning and resource allocation by health policy makers on the national, regional, and global levels. However, good-quality stroke epidemiological studies^7^ are still scarce in most countries, which prevented us from including in the analysis many other important risk factors, such as sickle cell disease and HIV, which are particularly important for sub-Saharan Africa. Differences in health-care systems and completeness and accuracy of stroke case ascertainment might play a part in the observed between-country differences (eg, very high stroke prevalence in Ghana compared with neighbouring countries). Although the GBD methods for estimating attributable burden of stroke due to risk factors accounts for a cumulative effect of multiple risk factors, it might not fully account for all potential confounders. Moreover, some new risk factors, such as high ambient temperature, might require further validation and examination to confirm their impact on stroke burden. Furthermore, more granular data analysis is needed. For example, stroke burden variation by race and ethnicity within countries, which can mask disparities in stroke incidence, risk factors, and outcomes among different population groups, and analysis of attributable effects of different levels of exposure to smoking, alcohol, and so on. We expect such analysis will be done in future GBD iterations.

In summary, our study findings continue to point out that currently used stroke prevention strategies are not sufficiently effective to halt, let alone reduce, the fast-growing stroke burden. Additional, more effective stroke prevention strategies (with the emphasis on population-wide measures, task shifting from doctors to nurses or health volunteers, and the wider use of evidence-based mobile and telehealth platforms) and pragmatic solutions to address the critical gaps in stroke service delivery, along with development of context-appropriate workforce capacity building and epidemiological surveillance systems,^38^ need to be urgently implemented across all countries. Without scaling up these innovative evidence-based strategies and policies that target local, national, regional, and global stroke prevention and care disparities, the burden of stroke will continue to grow, thus threatening the sustainability of health systems worldwide.

### GBD 2021 Stroke Risk Factor Collaborators

### Contributors

### Data sharing

To download GBD data used in these analyses, please visit the GBD 2021 Sources Tool website. To download forecasted estimates used in these analyses, please visit the GBD visualisation tools.

For the **GBD 2021 visualisation tools** see https://collab2021.Healthdata.Org/gbd-compare/

## Declaration of interests

A Abdelalim reports a leadership or fiduciary role in the Middle East and North Africa Stroke Organization, unpaid, as Vice President, outside the submitted work. S Afzal reports support for the present manuscript from King Edward Medical University through the provision of study material, research articles, valid data sources and authentic real time information for this manuscript; payment or honoraria for educational events and webinars with King Edward Medical University and collaborative partners including University of Johns Hopkins, University of California, University of Massachusetts, KEMCAANA, and KEMCA_UK; participation on a Data Safety Monitoring Board or Advisory Board with the National Bioethics Committee Pakistan, the King Edward Medical University Ethical Review Board, the Ethical Review Board of Fatima Jinnah Medical University and Sir Ganga Ram Hospital, and being a member of the Technical Working Group on Infectious Diseases; other financial and non-financial interests in King Edward Medical University, Annals of King Edward Medical University, Quality Enhancement Cell King Edward Medical University, Faculty of Public Health United Kingdom, Scientific Session, KEMCA-UK, International Scientific Conference, KEMCAANA, Research and Publications Higher Education Commission Pakistan, Research and Journals Committee Pakistan, Medical and Dental Council Pakistan, National Bioethics Committee Pakistan, Corona Experts Advisory Group, Technical Working Group on Infectious Diseases, Dengue Experts Advisory Group, Punjab Residency Program Research Committee, all outside the submitted work. R Akinyemi reports grants U19AG074865, U19 AG076581and R01AG072547 from the US National Institutes of Health/National Institute of Aging, GBHI ALZ UK-21- 24204 from the Alzheimer's Association and the Global Brain Health Institute, UK Royal Society/African Academy of Sciences FLAIR Grants FLR/R1/191813 and FCG/R1/201034, and GCRF Networking Grant from the UK Academy of Medical Sciences, all outside the submitted work. A Al-Ibraheem reports grants or contracts and support for attending meetings and/or travel from King Hussein Cancer Center, International Atomic Energy Agency; consulting fees from University of Jordan; leadership or fiduciary role, paid or unpaid, with Arab Society of Nuclear Medicine, and Asia Oceania Federation of Nuclear medicine and biology; all outside the submitted work. R Ancuceanu reports payment or honoraria for lectures, presentations, speakers bureaus, manuscript writing or educational events from AbbVie, Laropharm, and Reckitt, outside the submitted work. J Ärnlöv reports payment or honoraria for lectures from AstraZeneca and Novartis; participation on an Advisory Board with AstraZeneca and Astella; all outside the submitted work. M Ausloos reports grants or contracts from the project “A better understanding of socio-economic systems using quantitative methods from Physics” funded by European Union – NextgenerationEU and Romanian Government, under National Recovery and Resilience Plan for Romania, contract no.760034/ 23.05.2023, cod PNRR-C9-I8-CF 255/ 29.11.2022, through the Romanian Ministry of Research, Innovation and Digitalization, within Component 9, Investment I8, outside the submitted work. O C Baltatu reports grants or contracts from National Council for Scientific and Technological Development (CNPq, 304224/2022-7) and the Anima Institute through an AI research professor fellowship; leadership or fiduciary role, paid or unpaid, with Health and Biotechnology Advisory Board at Technology Park São José dos Campos – Center for Innovation in Health Technologies (CITS), outside the submitted work. T W Bärnighausen reports grants or contracts from National Institutes of Health, Alexander von Humboldt Foundation, German National Research Foundation (DFG), European Union, German Ministry of Education and Research, German Ministry of the Environment, Wellcome, and KfW; payment or honoraria for serving as Editor-in-Chief of PLOS Medicine; participation on a Data Safety Monitoring Board or Advisory Board, unpaid, with NIH-funded research projects in Africa on Climate Change and Health; stocks in CHEERS, an SME focusing on approaches to measure climate change and health-related variables in population cohorts; all outside the submitted work. S Bhaskar reports grants or contracts from Japan Society for the Promotion of Science (JSPS), Japanese Ministry of Education, Culture, Sports, Science and Technology (MEXT) through Grant-in-Aid for Scientific Research (KAKENHI), and from JSPS and the Australian Academy of Science through the JSPS International Fellowship; leadership or fiduciary role, paid or unpaid, with National Cerebral and Cardiovascular Center, Suita, Osaka, Japan, NSW Brain Clot Bank, Sydney, Australia, Rotary District 9675, Sydney, NSW, Australia, Global Health & Migration Hub Community, Global Health Hub Germany, Berlin, Germany, PLOS One, BMC Neurology, Frontiers in Neurology, Frontiers in Stroke, Frontiers in Aging, Frontiers in Public Health & BMC Medical Research Methodology, College of Reviewers, Canadian Institutes of Health Research (CIHR), Government of Canada, Cardiff University Biobank, Cardiff, UK, Cariplo Foundation, Milan, Italy, Pandemic Health System REsilience PROGRAM (REPROGRAM) Consortium; all outside the submitted work. B Bikbov reports grants or contracts with European Commission, Politecnico di Milano, and University of Rome; support for attending meetings and/or travel from European Renal Association; leadership or fiduciary role, unpaid, with Advocacy Group, International Society of Nephrology and Western Europe Regional Board, International Society of Nephrology; other non-financial in Scientific-Tools.org; all outside the submitted work. A Biswas reports consulting fees from Lupin Pharmaceuticals India, Intas Pharmaceuticals India, Alkem Laboratories India, and Eisai Pharmaceuticals India, all outside the submitted work. E J Boyko reports Payment or honoraria for lectures, presentations, speakers bureaus, manuscript writing or educational events from Korean Diabetes Association, Diabetes Association of the R.O.C. (Taiwan), American Diabetes Association, and the International Society for the Diabetic Foot; support for attending meetings and/or travel from Korean Diabetes Association, Diabetes Association of the R.O.C. (Taiwan), and International Society for the Diabetic Foot; all outside the submitted work. A L Catapano reports grants or contracts from Amryt Pharma, Menarini, and Ultragenyx; payment or honoraria for lectures, presentations, speakers bureaus, manuscript writing or educational events from Amarin, Amgen, Amryt Pharma, AstraZeneca, Daiichi Sankyo Esperion Ionis Pharmaceutical Medscaper, Menarini, Merck, Novartis, NovoNordisk, Peervoice Pfizer Recordati Regeneron, Sandoz, Sanofi The Corpus, Ultragenyx, and Viatris; all outside the submitted work. S Das reports a leadership or fiduciary role, unpaid, with association of Diagnostic & Laboratory Medicine India Chapter and Women in Global Health India, outside the submitted work. A Demetriades reports Leadership or fiduciary role, paid or unpaid as the Immediate Past President, European Association of Neurosurgical Societies (EANS) and Vice-President, Global Neuro Foundation, outside the submitted work. A Faro reports support for the present manuscript from National Council for Scientific and Technological Development (CNPq) through a scholarship. M Foschi reports consulting fees from Novartis and Roche; support for attending meetings and/or travel from Roche, Novartis, Biogen, Bristol-Meyer, Merck, and Sanofi; leadership or fiduciary role with MSBase Foundation as a member of the scientific leadership group; all outside the submitted work. R Franklin reports support for attending meetings and/or travel from ACTM – Tropical Medicine and Travel Medicine Conference 2022, 2023 and ISTM – Travel Medicine Conference, Basel 2023; leadership or fiduciary role, paid or unpaid, as Director of Kidsafe, Director of Auschem, Governance Committee of ISASH, Director of Farmsafe, SIG Convenor of PHAA Injury Prevention, and Vice President of ACTM; all outside the submitted work. A Guha reports grants or contracts from American Heart Association and Department of Defense; consulting fees from Pfizer and Novartis; leadership or fiduciary role, paid or unpaid, with ZERO Prostate Cancer – health equity task force; all outside the submitted work. G J Hankey reports payment or honoraria for lectures, presentations, speakers bureaus, manuscript writing or educational events from American Heart Association (for serving as Associate Editor of Circulation) Janssen (Johnson & Johnson) (for serving as Co-chair of Executive Committee, Librexia Stroke Trial, and lectures at sponsored scientific symposia), outside the submitted work. N E Ismail reports leadership or fiduciary role, unpaid, with Malaysian Academy of Pharmacy, Malaysia, and Malaysian Pharmacists Society Education Chapter, Malaysia, all outside the submitted work. T Joo reports support for the present manuscript from National Research, Development and Innovation Office in Hungary (RRF-2.3.1-21-2022-00006, Data-Driven Health Division of National Laboratory for Health Security) for funding of participation in the research project. J J Jozwiak reports Payment or honoraria for lectures, presentations, speakers bureaus, manuscript writing or educational events from Novartis, ADAMED, and Amgen; outside the submitted work. K Krishan reports non-financial support from the UGC Centre of Advanced Study, CAS II, awarded to the Department of Anthropology, Panjab University, Chandigarh, India, outside the submitted work. B Lacey reports support for the present manuscript from UK Biobank, funded largely by the UK Medical Research Council and Wellcome. P M Lavados reports grants from Boehringer Ingelheim; consulting fees from Boehringer Ingelheim for LATAM Stroke Projects Mentorship; payment or honoraria for lectures from Boehringer Ingelheim, Ferrer, Pfizer, and Novartis; support for attending Southamerican Angels meetings from Boehringer Ingelheim; participation on a Data Safety Monitoring Board or Advisory Board with Janssen, BMS, and Pfizer; leadership or fiduciary role, paid or unpaid, with Chilean Stroke Association (ACEVE) and Iberoamerican Stroke Society (SIECV-IASO); all outside the submitted work. M Li reports grants or contracts from the National Science and Technology Council, Taiwan (NSTC 112-2410-H-003-031; leadership or fiduciary role, paid or unpaid, as Technical Editor, Journal of the American Heart Association; all outside the submitted work. D Lindholm reports previous stock options and other financial or non-financial interests in AstraZeneca as a former employee, outside the submitted work. W Lo report stock or stock options in Abbott Lab, Amgen, Becton Dickson, Bristol Myers Squibb, Cardinal Health, GE Healthcare, Illumina, McKesson, Merck, Moderna, Pfizer, and Walgreens Boots, outside the submitted work. S Lorkowski reports grants or contracts from dsm-firmenich (formerly DSM Nutritional Products) as payments to their institution; consulting fees from Danone, Novartis Pharma, and Swedish Orphan Biovitrum (SOBI); payment or honoraria for lectures, presentations, speakers bureaus, manuscript writing or educational events from AMARIN Germany, Amedes Holding, Amgen, Berlin-Chemie, Boehringer Ingelheim Pharma, Daiichi Sankyo Deutschland, Danone, Hubert Burda Media Holding, Janssen-Cilag, Lilly Deutschland, Novartis Pharma, Novo Nordisk Pharma, Roche Pharma, Sanofi-Aventis, Swedish Orphan Biovitrum (SOBI), SYNLAB Holding Deutschland; support for attending meetings and/or travel from AMGEN; participation on a Data Safety Monitoring Board or Advisory Board from AMGEN, Daiichi Sankyo Deutschland, Novartis Pharma, Sanofi-Aventis; all outside the submitted work. L G Manotvani reports support for the present manuscript from the Italian Ministry of Health, Ministero della Salute, Ricerca Corrente, IRCCS Istituto Auxologico Italiano.H R Marateb reports support for their participation in present manuscript from Beatriu de Pinós post-doctoral programme from the Office of the Secretary of Universities and Research from the Ministry of Business and Knowledge of the Government of Catalonia programme: (2020 BP 00261). S Meo reports grants or contracts from Researchers Supporting Project, King Saud University, Riyadh, Saudi Arabia (RSP-2024 R47), outside the submitted work. L Monasta reports support for the present manuscript from the Italian Ministry of Health (Ricerca Corrente 34/2017), payments made to the Institute for Maternal and Child Health IRCCS Burlo Garofolo. R Moreira reports grants or contracts from CNPq (National Council for Scientific and Technological Development, scholarship registration number 316607/2021-5) outside the submitted work. S Muthu reports receiving the Luiz Vialle Award 2024 for travel support to attend Global Spine Congress 2024 from AO Spine International; leadership or fiduciary role with SICOT Awards Committee, AO Spine Associate Knowledge Forum, ICRS Next-Gen Committee; all outside the submitted work. S Nomura reports grants from Ministry of Education, Culture, Sports, Science and Technology of Japan (21H03203), and Precursory Research for Embryonic Science and Technology from the Japan Science and Technology Agency (JPMJPR22R8); outside the submitted work. B Norrving reports participation on a data safety or monitoring board or advisory board with Simbec-Orion (HOVID Trial); outside the submitted work. A Oneil reports grants or contracts from the National Health and Medical Research Council via her institution (#2009295); payment or honoraria for lectures, presentations, speakers bureaus, manuscript writing or educational events from the Exercise & Sports Science Australia conference for an invited presentation; outside the submitted work. A P Okekunle reports support for the present manuscript from National Research Foundation of Korea funded by the Ministry of Science and ICT (2020H1D3A1A04081265); support for attending meetings and/or travel from National Research Foundation of Korea funded by the Ministry of Science and ICT (2020H1D3A1A04081265); all outside the submitted work. R Ornello reports institutional grants from Novartis, consulting fees from Teva; payment or honoraria for lectures, presentations, speakers bureaus, manuscript writing or educational events from Eli Lilly, Novartis, Pfizer, Teva, AbbVie, and Lundbeck; support for attending meetings or travel from Teva; participation on a Data Safety Monitoring Board or Advisory Board with Eli Lilly; leadership or fiduciary role with The Journal of Headache and Pain Editorial Board, and Frontiers in Neurology Headache and Neurogenic Pain Section; receipt of payment of publication fees from Novartis and AbbVie; all outside the submitted work. A Ortiz reports grants to their institute from Sanofi and Catedra Mundipharma-UAM of diabetic kidney disease and the Catedra AstraZeneca-UAM of chronic kidney disease and electrolytes; consultancy or speaker fees Advicciene , Astellas, AstraZeneca, Amicus, Amgen, Boehringer-Ingelheim, Fresenius Medical Care, GSK, Bayer, Sanofi-Genzyme, Menarini, Kyowa Kirin, Alexion, Idorsia, Chiesi, Otsuka, Novo-Nordisk and Vifor Fresenius Medical Care Renal Pharma; travel support from Advicciene , Astellas, AstraZeneca, Fresenius Medical Care, Boehringer-Ingelheim Bayer, Sanofi-Genzyme, Menarini, Chiesi, Otsuka, Sysmex; leadership or fiduciary role, unpaid, with Council ERA. SOMANE; all outside the submitted work. E Ortiz-Prado reports grants or contracts from Universidad de las Americas, Quito-Ecuador outside the submitted work. R Palma-Alvarez reports payment or honoraria for lectures, presentations, speakers bureaus, manuscript writing or educational events from Angelini, Casen Recordarti, Lundbeck, Rubió, Takeda, Servier, and Neuraxpharm; support for attending meetings or travel from Angelini, Casen Recordati, Takeda, Italfarmaco, Lundbeck, and Janssen; all outside the submitted work. R Passera reports participation on Data Safety Monitoring Board dello studio “Consolidation with ADCT-402 (loncastuximab tesirine) after immunochemotherapy: a phase II study in BTKi-treated/ineligible Relapse/Refractory Mantle Cell Lymphoma (MCL) patients” - FIL, Fondazione Italiana Linfomi, Alessandria; leadership or fiduciary role, unpaid, as a Member of the EBMT Statistical Committee, European Society for Blood and Marrow Transplantation, Paris (F), and Past member 2020-2023 (biostatistician) of the IRB/IEC Comitato Etico AO SS. Antonio e Biagio Alessandria-ASL AL-VC; all outside the submitted work. A E Peden reports support for the present manuscript from [Australian] National Health and Medical Research Council (Grant Number: APP2009306). A Rane reports being a full-time employee of Agios Pharmaceuticals and owning stock and stock options, outside the submitted work. S Sacco reports grants or contracts from Novartis and Uriach; consulting fees from Novartis, Allergan-AbbVie, Teva, Lilly, Lundbeck, Pfizer, NovoNordisk, Abbott, and AstraZeneca; payment or honoraria for lectures, presentations, speakers bureaus, manuscript writing or educational events from Novartis, Allergan-Abbvie, Teva, Lilly, Lundbeck, Pfizer, NovoNordisk, Abbott, AstraZeneca; support for attending meetings and/or travel Lilly, Novartis, Teva, Lundbeck, and Pfizer; leadership or fiduciary role in other board, society, committee or advocacy group, paid or unpaid, with President European Stroke Organisation, and as Editor-in-Chief Cephalalgia; receipt of equipment, materials, drugs, medical writing, gifts or other services from Allergan-AbbVie, NovoNordisk; all outside the submitted work. Y L Samodra reports grants or contracts from FK Unpar, Indonesia; leadership or fiduciary role in other board, society, committee or advocacy group, paid or unpaid, with Benang Merah Research Center, Indonesia; all outside the submitted work. J Sanabria reports support for attending meetings and/or travel from Continuous Medical Education (CME) funds from Marshall University School of Medicine; participation on a Data Safety Monitoring Board or Advisory Board with Quality officer for the Department of Surgery; leadership or fiduciary role in other board, society, committee or advocacy group, paid or unpaid, with SSAT, ACS, IHPBA, American Board of Surgery; all outside the submitted work. N Scarmeas reports grants or contracts from Novo Nordisk through funding to their institution; participation on a Data Safety Monitoring Board or Advisory Board with the Multicultural Healthy Diet to Reduce Cognitive Decline & AD Risk NIH Funded Study at Albert Einstein College of Medicine, and with Primus AD through a Public Private funded Phase II study in Germany; all outside the submitted work. B M Schaarschmidt reports grants from Else Kröner-Fresenius Foundation, Deutsche Forschungsgemeinschaft, and PharmaCept; payment or honoraria for lectures from AstraZeneca; support for travel from Bayer AG; all outside the submitted work. A E Schutte reports grants or contracts from National Health and Medical Research Council of Australia, and the Medical Research Future Fund, Australia; consulting fees from Skylabs, Abbott, and Servier; payment or honoraria for lectures, presentations, speakers bureaus, manuscript writing or educational events from Abbott, Servier, Sanofi, Omron, Medtronic, and Aktiia; support for attending meetings or travel from Medtronic and Servier; leadership or fiduciary role, paid or unpaid, as Co-Chair National Hypertension Taskforce of Australia; all outside the submitted work. A Sharifan reports Leadership or fiduciary role, unpaid, with Cochrane Early Career Professionals Network; receipt of equipment, materials, drugs, medical writing, gifts or other services from Elsevier and Cochrane; all outside the submitted work. V Sharma reports other financial or non-financial interests in DFSS (MHA)'s research project (DFSS28(1)2019/EMR/6) at Institute of Forensic Science & Criminology, Panjab University, Chandigarh, India, outside the submitted work. S Shrestha reports other financial or non-financial interests in the School of Pharmacy, Monash University Malaysia, and the Graduate Research Merit Scholarship, outside the submitted work. J Silva reports support for the present manuscript from Portuguese Foundation for Science and Technology through salary payments (contract with reference 2021.01789.CEECIND/CP1662/CT0014). L R Silva reports grants or contracts from project code CENTRO-04-3559-FSE-000162, Fundo Social Europeu (FSE), outside the submitted work. J A Singh reports consulting fees from ROMTech, Atheneum, Clearview healthcare partners, American College of Rheumatology, Yale, Hulio, Horizon Pharmaceuticals, DINORA, Frictionless Solutions, Schipher, Crealta/Horizon, Medisys, Fidia, PK Med, Two labs, Adept Field Solutions, Clinical Care options, Putnam associates, Focus forward, Navigant consulting, Spherix, MedIQ, Jupiter Life Science, UBM, Trio Health, Medscape, WebMD, and Practice Point communications; and the National Institutes of Health; Payment or honoraria for lectures, presentations, speakers bureaus, manuscript writing or educational events on the speakers bureau of Simply Speaking; Support for attending meetings and/or travel from OMERACT as a steering committee member; Participation on a Data Safety Monitoring Board or Advisory Board with the FDA Arthritis Advisory Committee; Leadership or fiduciary role in other board, society, committee or advocacy group, paid as a past steering committee member of the OMERACT, an international organisation that develops measures for clinical trials and receives arm's length funding from 12 pharmaceutical companies, unpaid as Chair of the Veterans Affairs Rheumatology Field Advisory Committee, and unpaid as the Editor and Director of the UAB Cochrane Musculoskeletal Group Satellite Center on Network Meta-analysis; stock or stock options in Atai life sciences, Kintara therapeutics, Intelligent Biosolutions, Acumen pharmaceutical, TPT Global Tech, Vaxart pharmaceuticals, Atyu biopharma, Adaptimmune Therapeutics, GeoVax Labs, Pieris Pharmaceuticals, Enzolytics, Seres Therapeutics, Tonix Pharmaceuticals Holding Corp., Aebona Pharmaceuticals, and Charlotte's Web Holdings, Inc. and previously owned stock options in Amarin, Viking, and Moderna Pharmaceuticals; all outside the submitted work. J Sipila reports grants or contracts from Siun Sote Foundation and Eemil Aaltonen Foundation; payment or honoraria for lectures, presentations, speakers bureaus, manuscript writing or educational events from Novartis; support for attending meetings and/or travel from Lundbeck; participation on a Data Safety Monitoring Board or Advisory Board with Boehringer-Ingelheim and Sandoz; stock or stock options in Orion Corp; all outside the submitted work. J Sundstrom reports direct or indirect stock ownership in companies (Anagram kommunikation AB, Sence Research AB, Symptoms Europe AB, MinForskning AB) providing services to companies and authorities in the health sector including Amgen, AstraZeneca, Bayer, Boehringer, Eli Lilly, Gilead, GSK, Göteborg University, Itrim, Ipsen, Janssen, Karolinska Institutet, LIF, Linköping University, Novo Nordisk, Parexel, Pfizer, Region Stockholm, Region Uppsala, Sanofi, STRAMA, Takeda, TLV, Uppsala University, Vifor Pharma, WeMind, all outside the submitted work. A G Thrift reports grants or contracts from National Health & Medical Research Council (Australia), Heart Foundation (Australia), and Stroke Foundation (Australia); outside the submitted work. J H V Ticolau reports leadership or fiduciary role, paid or unpaid, with Benang Merah Research Center, Indonesia, outside the submitted work. S J Tromans reports grants or contracts from NHS Digital, via the Department of Health and Social Care, as payments to their institution; leadership or fiduciary role, unpaid, with the Academic Secretary for the Neurodevelopmental Psychiatry Special Interest Group at the Royal College of Psychiatrists, Editorial Board for BMC Psychiatry, Advances in Autism, Advances in Mental Health and Intellectual Disability, and Progress in Neurology and Psychiatry; all outside the submitted work. P Willeit reports consulting fees from Novartis Pharmaceuticals, outside the submitted work. Y Yasufuku reports grants or contracts from Shionogi & Co, through funding to their institution, outside the submitted work. M Zielinska reports other financial or non-financial interests in AstraZeneca as an employee, outside the submitted work.
