# Supplementary material for: Global, regional, and national burden of stroke and its risk factors, 1990–2021: a systematic analysis for the Global Burden of Disease Study 2021
Source: Lancet Neurol. 2024 Oct;23(10):973–1003. doi: 10.1016/S1474-4422(24)00369-7 (PMC12254192; doi:10.1016/S1474-4422(24)00369-7)
Supplement: Supplementary appendix [file mmc1.pdf]

THE LANCET
Neurology

Supplementary appendix

This appendix formed part of the original submission and has been peer reviewed. We post it as supplied by the authors.

Supplement to: GBD 2021 Stroke Risk Factor Collaborators. Global, regional, and national burden of stroke and its risk factors, 1990–2021: a systematic analysis for the Global Burden of Disease Study 2021. *Lancet Neurol* 2024; **23:** 973–1003.

Appendix

These materials are an Appendix to the manuscript "Global, regional and national stroke burden and stroke burden attributable to risk factors in 204 countries, 1990-2021: a systematic analysis for the Global Burden of Disease Study 2021" and adapted from the Appendix of methodological detail for "Global burden of 369 diseases and injuries in 204 countries and territories, 1990–2019: a systematic analysis for the Global Burden of Disease Study 2021"[1] and "Global burden of 87 risk factors in 204 countries and territories, 1990-2019: a systematic analysis for the Global Burden of Disease Study 2021"[2] as well as from Johnson et al.,[3] Roth et al.,[4] James et al.,[5] Kyu et al.,[6] and Stanaway et al.[7]

This appendix also provides further authorship detail for "Global, regional and national burden of stroke and its risk factors in 204 countries, 1990-2021: a systematic analysis for the Global Burden of Disease Study 2021"

Table of Contents

GBD 2021 STROKE RISK FACTORS COLLABORATORS 4

AFFILIATIONS 10

AUTHORS' CONTRIBUTIONS 47

PROVIDING DATA OR CRITICAL FEEDBACK ON DATA SOURCES 47
DEVELOPING METHODS OR COMPUTATIONAL MACHINERY 50
PROVIDING CRITICAL FEEDBACK ON METHODS OR RESULTS 50
DRAFTING THE WORK OR REVISING IT CRITICALLY FOR IMPORTANT INTELLECTUAL CONTENT 56
MANAGING THE ESTIMATION OR PUBLICATIONS PROCESS 61

SECTION 1. SUMMARY OF GBD METHODS FOR STROKE BURDEN ESTIMATES 61

1.1 INPUT DATA 62
1.2 SEARCH TERMS FOR SYSTEMATIC REVIEW 63
1.3 SEVERITY SPLIT INPUTS 65
1.3.1 Acute stroke severity splits 65
1.3.2 Chronic stroke severity splits 66
1.4 MODELLING STRATEGY 67
1.5 DATA SOURCES AND TYPES 69
1.6 STROKE DEFINITION 71
1.7 MODELLING STRATEGY 72
1.7.1 Ischaemic stroke 73
1.7.1.1 Input data 73
1.7.1.2 Modelling strategy 74
1.7.2 Intracerebral haemorrhage 75
1.7.2.1 Input data 75
1.7.2.2 Modelling strategy 75
1.7.3 Subarachnoid haemorrhage 75
1.7.3.1 Input data 76
1.7.3.2 Modelling strategy 76
1.8 METHODS TABLES 77
1.8.1 Table 1. ICD Codes used in fatal and non-fatal analysis. 77
1.8.2 Table 2. Selected covariates for CODEm models, overall stroke and subtypes 77
1.8.3 Table 3. Counts of data used by measure and stroke model for GBD 2021 78
1.8.4 Table 4. DisMod covariates 81

1.8.5 Table 5. Sequelae and disability weights for ischaemic stroke, intracerebral haemorrhage and subarachnoid haemorrhage *83*

SECTION 2. SUMMARY OF GBD METHODS FOR ESTIMATES OF BURDEN OF RISK FACTORS **83**

2.1 DEFINITION OF RISK FACTORS AND THEIR ESTIMATION 84
2.2 EFFECT SIZE ESTIMATION 86
2.2.1 Criteria for inclusion of risk-outcome pairs *86*
2.2.2 The World Cancer Research Fund grading system *86*
2.2.2.1 Convincing evidence 86
2.2.2.2 Probable evidence 86
2.2.2.3 Possible evidence 86
2.2.2.4 Insufficient evidence 86
2.3 DETERMINE RELATIVE RISKS 87
2.4 SEARCH TERMS AND DATA PREPARATION 88
2.5 DATA ANALYSIS 88
2.6 ESTIMATES EXPOSURE 90
2.6.1 Theoretical minimum-risk exposure level (TMREL) *91*
2.6.2 Estimate population-attributable fractions *91*
2.6.3 Estimate summary exposure values *92*
2.6.4 Mediation *92*
2.6.5 Calculating the burden of multiple risk factors. *93*
2.6.6 Adjusting for mediation *93*
2.6.7 Calculating mediation factor *94*
2.6.8 Uncertainty of aggregated and mediated PAFs *94*
2.6.9 Important assumptions in aggregating risk factors and including mediation *94*
2.6.10 Estimate attributable burden *95*
2.6.11 Decomposition analysis of deaths and DALYs *95*

SECTION 3. CHANGES IN THE MODELLING OF STROKE FOR GBD 2021 .... **96**

SECTION 5. APPENDIX TABLES **100**

APPENDIX TABLE 1. ABSOLUTE NUMBERS AND AGE-STANDARDISED RATES PER 100,000 PEOPLE PER YEAR, WITH 95% UNCERTAINTY INTERVALS (UI), OF INCIDENT AND PREVALENT STROKES, DEATHS FROM STROKE AND DISABILITY-ADJUSTED LIFE YEARS (DALYS) DUE TO STROKE BY PATHOLOGICAL TYPE OF STROKE AND WORLD BANK COUNTRY INCOME LEVEL, AND THEIR CHANGES (IN % WITH 95% UI) FROM 1990 TO 2021, FOR BOTH SEXES 100
APPENDIX TABLE 2. INCIDENT CASES, DEATHS, PREVALENT CASES, AND DALYS FOR ISCHAEMIC STROKE IN 2021 AND PERCENTAGE CHANGES OF AGE-STANDARDISED RATES FOR 1990-2021, BY LOCATION 102
APPENDIX TABLE 3. INCIDENT CASES, DEATHS, PREVALENT CASES, AND DALYS FOR INTRACEREBRAL HAEMORRHAGE IN 2021 AND PERCENTAGE CHANGES OF AGE-STANDARDISED RATES FOR 1990-2021, BY LOCATION 114
APPENDIX TABLE 4. INCIDENT CASES, DEATHS, PREVALENT CASES, AND DALYS FOR SUBARACHNOID HAEMORRHAGE IN 2021 AND PERCENTAGE CHANGES OF AGE-STANDARDISED RATES FOR 1990-2021, BY LOCATION 126
APPENDIX TABLE 5. ABSOLUTE NUMBER AND AGE-STANDARDISED RATES PER 100,000 PEOPLE PER YEAR, WITH 95% UNCERTAINTY INTERVALS (UI), OF INCIDENT AND PREVALENT STROKES, DEATHS FROM STROKE AND DALYS DUE TO STROKE IN 2021 AND PERCENTAGE CHANGE IN THE METRICS FOR 1990-2021, BY SEX AND PATHOLOGICAL TYPE OF STROKE 138
APPENDIX TABLE 6. PERCENTAGE CHANGE (%) IN STROKE INCIDENCE, PREVALENCE, DEATH, AND DALY RATES PER 100,000 BETWEEN 1990 AND 2021 BY PATHOLOGICAL TYPE OF STROKE IN PEOPLE YOUNGER AND OLDER THAT 70 YEARS (WITH 95% UIS) 140
APPENDIX TABLE 7. STROKE RELATED DISABILITY-ADJUSTED LIFE YEARS (DALYS) (ABSOLUTE NUMBERS WITH 95% UI AND AGE-STANDARDIZED PERCENTAGES WITH 95% UI) ASSOCIATED WITH RISK FACTORS AND THEIR CLUSTERS BY SOCIO-DEMOGRAPHIC INDEX (SDI) QUINTILES IN 2021, BOTH SEXES 141
APPENDIX TABLE 8. ISCHAEMIC STROKE RELATED DALYS (ABSOLUTE NUMBERS WITH 95% UI AND AGE-STANDARDIZED PERCENTAGE WITH 95% UI) ASSOCIATED WITH RISK FACTORS AND THEIR CLUSTERS IN 2021, BY SDI, ALL AGES, BOTH SEXES 144
APPENDIX TABLE 9. INTRACEREBRAL HAEMORRHAGE RELATED DALYS (ABSOLUTE NUMBERS WITH 95% UI AND AGE-STANDARDIZED PERCENTAGE WITH 95% UI) ASSOCIATED WITH RISK FACTORS AND THEIR CLUSTERS IN 2021, BY SDI, ALL AGES, BOTH SEXES 148

Appendix Table 10. Subarachnoid haemorrhage related DALYs (absolute numbers with 95% UI and age-standardized percentages with 95% UI) associated with risk factors and their clusters in 2021, by SDI, all ages, both sexes 151
Appendix Table 11. Age-standardized percent of DALYs (with 95% uncertainty intervals) due to stroke associated with individual risk factors by 7 GBD super-region, 21 GBD region and country for both sexes combined in 2021. 154
Appendix Table 12. Age-standardized percent of DALYs (with 95% uncertainty intervals) due to ischemic stroke associated with individual risk factors by 7 GBD super-region, 21 GBD region and country for both sexes combined in 2021. 178
Appendix Table 13. Age-standardized percent of DALYs (with 95% uncertainty intervals) due to intracerebral haemorrhage associated with individual risk factors by 7 GBD super-region, 21 GBD region and country for both sexes combined in 2021. 202
Appendix Table 14. Age-standardized percent of DALYs (with 95% uncertainty intervals) due to subarachnoid haemorrhage associated with individual risk factors by 7 GBD super-region, 21 GBD region and country for both sexes combined in 2021. 226
Appendix Table 15. Age-standardised rates (per 100,000 people) and percentages of DALYs (with 95% uncertainty intervals) due to stroke attributable to metabolic, behavioural and environmental/occupational risk clusters and all risk factors combined globally by SDI and sex in 2021. 250
Appendix Table 16. Age-standardised rates (per 100,000 people) and percentages of DALYs (with 95% uncertainty intervals) due to ischaemic stroke attributable to metabolic, behavioural and environmental/occupational risk clusters and all risk factors combined globally by SDI and sex in 2021. 251
Appendix Table 17. Age-standardised rates (per 100,000 people) and percentages of DALYs (with 95% uncertainty intervals) due to intracerebral haemorrhage attributable to metabolic, behavioural and environmental/occupational risk clusters and all risk factors combined globally by SDI and sex in 2021. 252
Appendix Table 18. Age-standardised rates (per 100,000 people) and percentages of DALYs (with 95% uncertainty intervals) due to subarachnoid haemorrhage attributable to metabolic, behavioural and environmental/occupational risk clusters and all risk factors combined globally by SDI and sex, 2021. 253

SECTION 6. APPENDIX FIGURES 254

Appendix Figure 1. 21 GBD regions 254
List of countries in 21 GBD regions 255
Appendix Figure 2. Global number of deaths and DALYs by causes in 2021, both sexes, all ages 256
Appendix Figure 3. Global stroke incidence, prevalence, death and DALY rates per 100,000 per year from 1990 to 2021 (shadow colours stand for 95% UIs) 257
Appendix Figure 4. Global age-standardised stroke incidence, prevalence, death and DALY rates per 100,000 per year from 1990 to 2021 in 7 GBD super regions, both sexes (shadow colours stand for 95% UIs) 258
Appendix Figure 5. Global age-standardised stroke incidence, prevalence, death and DALY rates per 100,000 per year from 1990 to 2021 by pathological type of stroke, both sexes (shadow colours stand for 95% UIs) 259
Appendix Figure 6. Global age-standardised stroke incidence, prevalence, death and DALY rates per 100,000 per year from 1990 to 2021 by SDI and pathological type of stroke, both sexes (shadow colours stand for 95% UIs) 260
Appendix Figure 7. Global trends in age-standardised stroke incidence, prevalence, death, and DALY rates per 100,000 per year for 1990-2021 by SDI, for both sexes 261
Appendix Figure 8. Annual stroke incidence, prevalence, death and DALY rates per 100,000 people younger than 70 years old (shadow areas are 95% uncertainty intervals) 262
Appendix Figure 9. Annual stroke incidence, prevalence, death and DALY rates per 100,000 people of 70+ years old (shadow areas are 95% uncertainty intervals) 263
Appendix Figure 10. Global map showing age-standardised stroke DALYs attributable to all risk factors combined by pathological type of stroke, for both sexes, 2021 264
Appendix Figure 11. Trends in the percent of stroke-related DALYs attributable to risk factors in 1990 and 2021 at level 4 of the GBD risk factors hierarchy by pathological type of stroke, both sexes globally (rank based on age-standardised DALYs). 265

REFERENCES 266

GBD 2021 Stroke Risk Factors Collaborators

Valery L Feigin, Melsew Dagnee Abate, Yohannes Habtegiorgis Abate, Samar Abd ElHafeez, Foad Abd-Allah, Ahmed Abdelalim, Atef Abdelkader, Michael Abdelmasseh, Sherief Abd-Elsalam, Parsa Abdi, Arash Abdollahi, Meriem Abdoun, Rami Abd-Rabu, Deldar Morad Abdulah, Auwal Abdullahi, Mesfin Abebe, Roberto Ariel Abeldaño Zuñiga, E S Abhilash, Olugbenga Olusola Abiodun, Olumide Abiodun, Rahim Abo Kasem, Richard Gyan Aboagye, Mohamed Abouzid, Lucas Guimarães Abreu, Woldu Aberhe Abrha, Dariush Abtahi, Samir Abu Rumeileh, Ahmed Abualhasan, Hasan Abualruz, Eman Abu-Gharbieh, Hana J Abukhadijah, Niveen ME Abu-Rmeileh, Salahdein Aburuz, Ahmed Abu-Zaid, Juan Manuel Acuna, Denberu Eshetie Adane, Mesafint Molla Adane, Isaac Yeboah Addo, Rufus Adesoji Adedoyin, Oyelola A Adegboye, Victor Adekanmbi, Kishor Adhikari, Qorinah Estiningtyas Sakilah Adnani, Saryia Adra, Leticia Akua Adzigbli, Abdelrahman Yousry Afify, Aanuoluwapo Adeyimika Afolabi, Fatemeh Afrashteh, Muhammad Sohail Afzal, Saira Afzal, Shahin Aghamiri, Williams Agyemang-Duah, Bright Opoku Ahinkorah, Aqeel Ahmad, Muayyad M Ahmad, Sajjad Ahmad, Shahzaib Ahmad, Tauseef Ahmad, Amir Mahmoud Ahmadzade, Ali Ahmed, Ayman Ahmed, Haroon Ahmed, Syed Anees Ahmed, Marjan Ajami, Budi Aji, Essona Matatom Akara, Rufus Olusola Akinyemi, Mohammed Ahmed Akkaif, Ashley E Akrami, Salah Al Awaidy, Hanadi Al Hamad, Syed Mahfuz Al Hasan, Mohammad Al Qadire, Omar Al Ta'ani, Yazan Al-Ajlouni, Samer O Alalalmeh, Tariq A Alalwan, Ziyad Al-Aly, Rasmieh Mustafa Al-amer, Wafa A Aldhaleei, Mohammed S Aldossary, Seyedeh Yasaman Alemohammad, Bassam Al-Fatly, Adel Ali Saeed Al-Gheethi, Fadwa Naji Alhalaiqa, Maryam Alharrasi, Abid Ali, Mohammed Usman Ali, Rafat Ali, Syed Shujait Ali, Waad Ali, Akram Al-Ibraheem, Sheikh Mohammad Alif, Syed Mohamed Aljunid, Wael Almahmeed, Sabah Al-Marwani, Mahmoud A Alomari, Jordi Alonso, Jaber S Alqahtani, Rajaa M Mohammad Al-Raddadi, Ahmad Alrawashdeh, Mohammed A Alsabri, Najim Z Alshahrani, Zaid Altaany, Awais Altaf, Alaa B Al-Tammemi, Diala Altwalbeh, Nelson Alvis-Guzman, Hassan Alwafi, Mohammad Al-Wardat, Yaser Mohammed Al-Worafi, Hany Aly, Safwat Aly, Mohammad Sharif Ibrahim Alyahya, Karem H Alzoubi, Walid Adnan Al-Zyoud, Reza Amani, Prince M Amegbor, Tewodros Getnet Amera, Tarek Tawfik Amin, Alireza Amindarolzarbi, Sohrab Amiri, Hubert Amu, Dickson A Amugsi, Ganiyu Adeniyi Amusa, Robert Ancuceanu, Deanna Anderlini, Dhanalakshmi Angappan, Abhishek Anil, Mohammed Tahir Tahir Ansari, Alireza Ansari-Moghaddam, Rockson Ansong, Saeid Anvari, Saleha Anwar, Sumadi Lukman Anwar, Ekenedilichukwu Emmanuel Anyabolo, Anayochukwu Edward Anyasodor, Geminn Louis Carace Apostol, Francis Appiah, Muhammad Aqeel, Jalal Arabloo, Razman Arabzadeh Bahri, Mosab Arafat, Aleksandr Y Aravkin, Ali Ardekani, Demelash Areda, Brhane Berhe Aregawi, Getnet Mesfin Aregu, Olatunde Aremu, Hidayat Arifin, Johan Ärnlöv, Anton A Artamonov, Judie Arulappan, Umesh Raj Aryal, Zahra Aryan, Akram M Asbeutah, Mulusew A Asemahagn, Mulu Tiruneh Asemu, Mohammad Asghari-Jafarabadi, Mubarek Yesse Ashemo, Tahira Ashraf, Armin Aslani, Haftu Asmerom Asmerom, Thomas Astell-Burt, Seyyed Shamsadin Athari, Prince Atorkey, Maha Moh'd Wahbi Atout, Alok Atreya, Avinash Aujayeb, Marcel Ausloos, Abolfazl Avan, Hamzeh Awad, Adedapo Wasiu Awotidebe, Lemessa Assefa A Ayana, Setognal Birara Aychiluhm, Amdehiwot A Aynalem, Zewdu Bishaw Aynalem, Sina Azadnajafabad, Hiva Azami, Shahkaar Aziz, Ahmed Y Azzam, Abraham Samuel Babu, Giridhara Rathnaiah Babu, Muhammad Badar, Ashish D Badiye, Pegah Bahrami Taghanaki,

Saeed Bahramian, Ruhai Bai, Atif Amin Baig, Shankar M Bakkannavar, Abdulaziz T Bako, Ovidiu Constantin Baltatu, Kiran Bam, Maciej Banach, Morteza Banakar, Soham Bandyopadhyay, Palash Chandra Banik, Kannu Bansal, Yanping Bao, Miguel A Barboza, Mainak Bardhan, Noel C Barengo, Suzanne Lyn Barker-Collo, Till Winfried Bärnighausen, Hiba Jawdat Barqawi, Amadou Barrow, Lingkan Barua, Azadeh Bashiri, Hameed Akande Bashiru, Afisu Basiru, Mohammad-Mahdi Bastan, Sanjay Basu, Saurav Basu, Kavita Batra, Ahmet Begde, Babak Behnam, Amir Hossein Behnoush, Melesse B Y Belayneh, Michael Belingheri, Umar Muhammad Bello, Derrick A Bennett, Isabela M Bensenor, Fentaw Tadese Berhe, Amiel Nazer C Bermudez, Habtamu B B Beyene, Kebede A Beyene, Devidas S Bhagat, Akshaya Srikanth Bhagavathula, Neeraj Bhala, Ashish Bhalla, Nikha Bhardwaj, Pankaj Bhardwaj, Sonu Bhaskar, Ajay Nagesh Bhat, Vivek Bhat, Gurjit Kaur Bhatti, Jasvinder Singh Singh Bhatti, Mohiuddin Ahmed Bhuiyan, Subarna Bhusal, Boris Bikbov, Cem Bilgin, Antonio Biondi, Keralem Anteneh Bishaw, Atanu Biswas, Bijit Biswas, Trupti Bodhare, Eyob Ketema Bogale, Archith Boloor, Milad Bonakdar Hashemi, Aime Bonny, Berrak Bora Basara, Hamed Borhany, Samuel Adolf Bosoka, Souad Bouaoud, Abdelhakim Bouyahya, Edward J Boyko, Marija M Bozic, Dejana Braithwaite, Susanne Breitner, Hermann Brenner, Gabrielle Britton, Andre R Brunoni, Dana Bryazka, Raffaele Bugiardini, Lemma N Bulto, Katrin Burkart, Yasser Bustanji, Zahid A Butt, Florentino Luciano Caetano dos Santos, Luis Alberto Cámera, Luciana Aparecida Campos, Ismael R Campos-Nonato, Fan Cao, Angelo Capodici, Rosario Cárdenas, Sinclair Carr, Giulia Carreras, Andre F Carvalho, Felix Carvalho, Joao Mauricio Castaldelli-Maia, Carlos A Castañeda-Orjuela, Giulio Castelpietra, Alberico L Catapano, Maria Sofia Cattaruzza, Luca Cegolon, Francieli Cembranel, Edina Cenko, Ester Cerin, Joshua Chadwick, Chiranjib Chakraborty, Sandip Chakraborty, Jeffrey Shi Kai Chan, Rama Mohan Chandika, Eeshwar K Chandrasekar, Gashaw Sisay Chanie, Vijay Kumar Chattu, Anis Ahmad Chaudhary, Akhilanand Chaurasia, Haowei Chen, Mingling Chen, Simiao Chen, Gerald Chi, Fatemeh Chichagi, Ritesh Chimoriya, Patrick R Ching, Abdulaal Chitheer, So Mi Jemma Cho, Dong-Woo Choi, Bryan Chong, Chean Lin Chong, Hitesh Chopra, Sonali Gajanan Choudhari, Rahul Choudhary, Dinh-Toi Chu, Isaac Sunday Chukwu, Sheng-Chia Chung, Zinhle Cindi, Iolanda Cioffi, Rebecca M Cogen, Alyssa Columbus, Simona Costanzo, Rosa A S Couto, Michael H Criqui, Natalia Cruz-Martins, Silvia Magali Cuadra-Hernández, Alanna Gomes da Silva, Sriharsha Dadana, Omid Dadras, Xiaochen Dai, Koustuv Dalal, Lachlan L Dalli, Giovanni Damiani, Emanuele D'Amico, Lalit Dandona, Rakhi Dandona, Amira Hamed Darwish, Saswati Das, Mohsen Dashti, Mohadese Dashtkoohi, Mohammad Dashtkoohi, Maedeh Dastmardi, Kairat Davletov, Vanessa De la Cruz-Góngora, Sean DeAngelo, Aklilu Tamire Debele, Shayom Debopadhaya, Ivan Delgado-Enciso, Berecha Hundessa Demessa, Andreas K Demetriades, Edgar Denova-Gutiérrez, Emina Dervišević, Hardik Dineshbhai Desai, Aragaw Tesfaw Desale, Fikreab Desta, Vinoth Gnana Chellaiyan Devanbu, Devananda Devegowda, Syed Masudur Rahman Dewan, Amol S Dhane, Meghnath Dhimal, Vishal R Dhulipala, Michael J Diaz, Mengistie Diress, Milad Dodangeh, Phidelia Theresa Doegah, Sushil Dohare, Mohamed Fahmy Doheim, Klara Georgieva Dokova, Deepa Dongarwar, Mario D'Oria, Ojas Prakashbhai Doshi, Rajkumar Prakashbhai Doshi, Abdel Douiri, Robert Kokou Dowou, Ashel Chelsea Dsouza, Haneil Larson Dsouza, Viola Savy Dsouza, Bruce B Duncan, Andre Rodrigues Duraes, Arkadiusz Marian Dziedzic, Michael Ekholuenetale, Ibrahim Farahat El Bayoumy, Maysaa El Sayed Zaki, Iffat Elbarazi, Faris El-Dahiyat, Islam Y Elgendy, Muhammed Elhadi, Waseem El-Huneidi, Mohamed A Elmonem, Adel B Elmoselhi, Chadi Eltaha, Theophilus I Emeto, Christopher Imokhuede Esezobor, Negin Esfandiari, Zahra Esmaeili, Francesco Esposito, Mohammad Etoom, Natalia

Fabin, Ibtihal Fadhil, Adeniyi Francis Fagbamigbe, Omotayo Francis Fagbule, Shahriar Faghani, Ayesha Fahim, Ildar Ravisovich Fakhradiyev, Luca Falzone, Mohammad Fareed, Jawad Fares, Carla Sofia e Sá Farinha, MoezAlIslam Ezzat Mahmoud Faris, Pawan Sirwan Faris, Mohsen Farjoud Kouhanjani, Andre Faro, Hossein Farrokhpour, Abidemi Omolara Fasanmi, Nelsensius Klau Fauk, Patrick Fazeli, Timur Fazylov, Alireza Feizkhah, Ginenus Fekadu, Xiaoqi Feng, Seyed-Mohammad Fereshtehnejad, Pietro Ferrara, Nuno Ferreira, Getahun Fetensa, Bikila Regassa Feyisa, Florian Fischer, Luisa S Flor, Kristen Marie Foley, Ana Catarina Fonseca, Roham Foroumadi, Behzad Foroutan, Daniela Fortuna, Matteo Foschi, Richard Charles Franklin, Ni Kadek Yuni Fridayani, Sridevi G, Peter Andras Gaal, Abhay Motiramji Gaidhane, Abduzhappar Gaipov, Yaseen Galali, Silvano Gallus, Aravind P Gandhi, Balasankar Ganesan, Danijela Gasevic, Prem Gautam, Rupesh K Gautam, Miglas Welay Gebregergis, Mesfin Gebrehiwot, Kebre Gebrekirstos Gebrekidan, Lemma Getacher, Genanew K Getahun, Molla Getie, Delaram J Ghadimi, Fataneh Ghadirian, Amir Ghaffari Jolfayi, Mansour Ghafourifard, Mohammad-Reza Ghasemi, Afsaneh Ghasemzadeh, Ramy Mohamed Ghazy, Ehsan Gholami, Zainab Gholami, Sherief Ghozy, Stefano Giannoni Luza, Jaleed Ahmed Gilani, Tiffany K Gill, Richard F Gillum, Ebisa Zerihun Gindaba, Elena V Gnedovskaya, Amit Goel, Mohamad Goldust, Mahaveer Golechha, Pouya Goleij, Davide Golinelli, Philimon N Gona, Giuseppe Gorini, Alessandra C Goulart, Barbara Niegia Garcia Goulart, Mahdi Gouravani, Michal Grivna, Giuseppe Grosso, Ashna Grover, Shi-Yang Guan, Giovanni Guarducci, Avirup Guha, Stefano Guicciardi, Snigdha Gulati, Damitha Asanga Gunawardane, Cui Guo, Zhifeng Guo, Anish Kumar Gupta, Bhawna Gupta, Mohak Gupta, Rahul Gupta, Rajat Das Gupta, Rajeev Gupta, Sapna Gupta, Farrokh Habibzadeh, Najah R Hadi, Mohammad Haghani Dogahe, Hamed Haghi-Aminjan, Dariush Haghmorad, Arvin Haj-Mirzaian, Aram Halimi, Nadia M Hamdy, Samer Hamidi, Erin B Hamilton, Asif Hanif, Nasrin Hanifi, Graeme J Hankey, Md Abdul Hannan, Zaim Anan Haq, Arief Hargono, Netanja I Harlianto, Josep Maria Haro, Eka Mishbahatul Marah Has, Ahmed I Hasaballah, Ikramul Hasan, Md Saquib Hasnain, Ikrama Hassan, Mahgol Sadat Hassan Zadeh Tabatabaei, Johannes Haubold, Rasmus J Havmoeller, Simon I Hay, Youssef Hbid, Jeffrey J Hebert, Omar E Hegazi, Mohammad Heidari, Mehdi Hemmati, Demisu Zenbaba Heyi, Kamal Hezam, Yuta Hiraike, Nguyen Quoc Hoan, Ramesh Holla, Nobuyuki Horita, Md Mahbub Hossain, Hassan Hosseinzadeh, Mehdi Hosseinzadeh, Ahmad Hosseinzadeh Adli, Mihaela Hostiuc, Sorin Hostiuc, Ben Hu, Chengxi Hu, Junjie Huang, Ayesha Humayun, Salman Hussain, Le Duc Huy, Hong-Han Huynh, Bing-Fang Hwang, Segun Emmanuel Ibitoye, Nayu Ikeda, Adalia Ikiroma, Olayinka Stephen Ilesanmi, Irena M Ilic, Milena D Ilic, Mohammad Tarique Imam, Mustapha Immurana, Leeberk Raja Inbaraj, Muhammad Iqhrammullah, Arnaud Iradukunda, Lalu Muhammad Irham, Md Rabiul Islam, Faisal Ismail, Nahlah Elkudssiah Ismail, Hiroyasu Iso, Gaetano Isola, Ramaiah Itumalla, Masao Iwagami, Chidozie Declan CD Iwu, Vinothini J, Jalil Jaafari, Louis Jacob, Abdollah Jafarzadeh, Haitham Jahrami, Akhil Jain, Nityanand Jain, Ammar Abdulrahman Jairoun, Abhishek Jaiswal, Mihajlo Jakovljevic, Reza Jalilzadeh Yengejeh, Balamurugan Janakiraman, Abubakar Ibrahim Jatau, Sathish Kumar Jayapal, Shubha Jayaram, Sun Ha Jee, Jayakumar Jeganathan, Mihretu Jegnie, Alelign Tasew Jema, Bijay Mukesh Jeswani, Angeline Jeyakumar, Anil K Jha, Ravi Prakash Jha, Zixiang Ji, Heng Jiang, Shuai Jin, Yingzhao Jin, Mohammad Jokar, Jost B Jonas, Tamas Joo, Jobinse Jose, Nitin Joseph, Charity Ehimwenma Joshua, Farahnaz Joukar, Jacek Jerzy Jozwiak, Mikk Jürisson, Ali Kabir, Md Awal Kabir, Zubair Kabir, Vidya Kadashetti, Rizwan Kalani, Sanjay Kalra, Vineet Kumar Kamal, Arun Kamireddy, Haidong Kan, Mona Kanaan, Himal Kandel, Kehinde Kazeem Kanmodi, Rami S Kantar, Neeti Kapoor, Paschalis Karakasis, Ibraheem M Karaye, André Karch, Hanie Karimi,

Salah Eddin Karimi, Yeganeh Karimi, Arman Karimi Behnagh, Prabin Karki, Hengameh Kasraei, Joonas H Kauppila, Harkiran Kaur, Neda Kaydi, Gbenga A Kayode, Foad Kazemi, Sina Kazemian, Emmanuelle Kesse-Guyot, Yousef Saleh Khader, Morteza Abdullatif Khafaie, Inn Kynn Khaing, Himanshu Khajuria, Amirmohammad Khalaji, Nauman Khalid, Anees Ahmed Khalil, Asaduzzaman Khan, Fayaz Khan, M Nuruzzaman Khan, Maseer Khan, Mohammad Jobair Khan, Moien AB Khan, Yusra H Khan, Shaghayegh Khanmohammadi, Khaled Khatab, Haitham Khatatbeh, Moawiah Mohammad Khatatbeh, Sorour Khateri, Mahalaqua Nazli Khatib, Maryam Khayamzadeh, Hamid Reza Khayat Kashani, Feriha Fatima Khidri, Manoj Khokhar, Atulya Aman Khosla, Majid Khosravi, Jagdish Khubchandani, Saeid Kian, Kwanghyun Kim, Min Seo Kim, Yun Jin Kim, Ruth W Kimokoti, Adnan Kisa, Sezer Kisa, Ali-Asghar Kolahi, Kamrun Nahar Koly, Farzad Kompani, Shivakumar KM Marulasiddaiah Kondlahalli, Miikka Korja, Vladimir Andreevich Korshunov, Oleksii Korzh, Soewarta Kosen, Karel Kostev, Nikhil Kothari, Ashwin Laxmikant Kotnis, Sindhura Lakshmi Koulmane Laxminarayana, Kewal Krishan, Varun Krishna, Vijay Krishnamoorthy, Bindu Krishnan, Jera Kruja, Barthelemy Kuate Defo, Burcu Kucuk Bicer, Md Abdul Kuddus, Mohammed Kuddus, Nuworza Kugbey, Mukhtar Kulimbet, Vishnutheertha Kulkarni, Akshay Kumar, Ashish Kumar, Dewesh Kumar, G Anil Kumar, Nithin Kumar, Rakesh Kumar, Senthil Kumaran D, Amartya Kundu, Satyajit Kundu, Setor K Kunutsor, Om P Kurmi, Dian Kusuma, L V Simhachalam Kutikuppala, Ambily Kuttikkattu, Ville Kytö, Carlo La Vecchia, Ben Lacey, Chandrakant Lahariya, Dharmesh Kumar Lal, Tea Lallukka, Judit Lám, Iván Landires, Anders O Larsson, Savita Lasrado, Kaveh Latifinaibin, Paolo Lauriola, Pablo M Lavados, Basira Kankia Lawal, Long Khanh Dao Le, Nhi Huu Hanh Le, Thao Thi Thu Le, Trang Diep Thanh Le, Paul H Lee, Seung Won Lee, Wei-Chen Lee, Yo Han Lee, Ming-Chieh Li, Wei Li, Xiaopan Li, Yichong Li, Lee-Ling Lim, Stephen S Lim, John C Lin, Daniel Lindholm, Shai Linn, Gang Liu, Runben Liu, Shuke Liu, Xiaofeng Liu, Xuefeng Liu, Erand Llanaj, Chun-Han Lo, Warren David Lo, Valerie Lohner, José Francisco López-Gil, László Lorenzovici, Stefan Lorkowski, Paulo A Lotufo, Giancarlo Lucchetti, Lisha Luo, Jay B Lusk, Zheng Feei Ma, Monika Machoy, Farzan Madadizadeh, Ralph Maddison, Elham Mahmoudi, Golnaz Mahmoudvand, Omar M Makram, Elaheh Malakan Rad, Kashish Malhotra, Ahmad Azam Malik, Iram Malik, Tauqeer Hussain Mallhi, Deborah Carvalho Malta, Abdullah A Mamun, Yosef Manla, Mohammad Hadi Mansouri, Pejman Mansouri, Vahid Mansouri, Mohammad Ali Mansournia, Lorenzo Giovanni Mantovani, Emmanuel Manu, Hamid Reza Marateb, Abdoljalal Marjani, Daniela Martini, Santi Martini, Miquel Martorell, Sharmeen Maryam, Roy Rillera Marzo, Awoke Masrie, Yasith Mathangasinghe, Pallab K Maulik, Mahsa Mayeli, Mohsen Mazidi, Martin McKee, Steven M McPhail, Enkeleint A Mechili, Asim Mehmood, Kamran Mehrabani-Zeinabad, Tesfahun Mekene Meto, Hadush Negash Meles, Walter Mendoza, Ritesh G Menezes, George A Mensah, Sultan Ayoub Meo, Atte Meretoja, Tuomo J Meretoja, Tomislav Mestrovic, Chamila Dinushi Kukulege Mettananda, Tomasz Miazgowski, Irmina Maria Michalek, Ana Carolina Micheletti Gomide Nogueira de Sá, Giuseppe Minervini, Le Huu Nhat Minh, GK Mini, Mojgan Mirghafourvand, Andreea Mirica, Erkin M Mirrakhimov, Mohammad Mirza-Aghazadeh-Attari, Manish Mishra, Sanjeev Misra, Prasanna Mithra, Ahmed Ismail Mohamed, Jama Mohamed, Nouh Saad Mohamed, Ameen Mosa Mohammad, Esmaeil Mohammadi, Saeed Mohammadi, Soheil Mohammadi, Abdollah Mohammadian-Hafshejani, Ibrahim Mohammadzadeh, Hussen Mohammed, Mustapha Mohammed, Salahuddin Mohammed, Shafiu Mohammed, Ali H Mokdad, Hossein Molavi Vardanjani, Mariam Molokhia, Shaher Momani, Lorenzo Monasta, Mohammad Ali Moni, Fateme Montazeri, AmirAli Moodi Ghalibaf, Mahmood Moosazadeh, Maryam Moradi, Yousef Moradi, Paula Moraga, Lidia

Morawska, Rafael Silveira Moreira, Shane Douglas Morrison, Reza Mosaddeghi Heris, Elias Mossialos, Parsa Mousavi, Ahmed Msherghi, Sumaira Mubarik, Lorenzo Muccioli, Admir Mulita, Malaisamy Muniyandi, Kavita Munjal, Efren Murillo-Zamora, Sathish Muthu, Woojae Myung, Amin Nabavi, Ashraf Fawzy Nabhan, Ayoub Nafei, Ahamarshan Jayaraman Nagarajan, Pirouz Naghavi, Ganesh R Naik, Gurudatta Naik, Mukhammad David Naimzada, Sanjeev Nair, Tapas Sadasivan Nair, Soroush Najdaghi, Hastyar Hama Rashid Najmuldeen, Noureddin Nakhostin Ansari, Vinay Nangia, Sreenivas Narasimha Swamy, Shumaila Nargus, Delaram Narimani Davani, Bruno Ramos Nascimento, Gustavo G Nascimento, Ali Nasrollahizadeh, Amir Nasrollahizadeh, Zuhair S Natto, Javaid Nauman, Samidi Nirasha Kumari Navaratna, Biswa Prakash Nayak, Vinod C Nayak, Athare Nazri-Panjaki, Rawlance Ndejjo, Ionut Negoi, Ruxandra Irina Negoi, Seyed Aria Nejadghaderi, Chakib Nejjari, Mohammad Hadi Nematollahi, Samata Nepal, Charles Richard James Newton, Dang H Nguyen, Duc Hoang Nguyen, Hau Thi Hien Nguyen, Hien Quang Nguyen, Nhien Ngoc Y Nguyen, Phat Tuan Nguyen, Van Thanh Nguyen, Robina Khan Niazi, Yeshambel T Nigatu, Nasrin Nikravangolsefid, Dina Nur Anggraini Ningrum, Chukwudi A Nnaji, Lawrence Achilles Nnyanzi, Shuhei Nomura, Syed Toukir Ahmed Noor, Bo Norrving, Nawsherwan Nawsherwan, Jean Jacques Noubiap, Chisom Adaobi Nri-Ezedi, George Ntaios, Mpiko Ntsekhe, Fred Nugen, Mario Cesare Nurchis, Dieta Nurrika, Chimezie Igwegbe Nzoputam, Ogochukwu Janet Nzoputam, Bogdan Oancea, Kehinde O Obamiro, Ismail A Odetokun, Martin James O'Donnell, James Odhiambo Oguta, In-Hwan Oh, Tolulope R Ojo-Akosile, Hassan Okati-Aliabad, Sylvester Reuben Okeke, Akinkunmi Paul Okekunle, Lawrence Okidi, Osaretin Christabel Okonji, Morteza Oladnabi, Andrew T Olagunju, Muideen Tunbosun Olaiya, Oladotun Victor Olalusi, Tosin Abiola Olasehinde, Omotola O Olasupo, Matthew Idowu Olatubi, Arão Belitardo Oliveira, Gláucia Maria Moraes Oliveira, Abdulhakeem Abayomi Olorukooba, Isaac Iyinoluwa Olufadewa, Yinka Doris Doris Oluwafemi, Gideon Olamilekan Oluwatunase, Hany A Omar, Ahmed Omar Bali, Adrienne E O'Neil, Sok King Ong, Obinna E Onwujekwe, Abdulahi Opejin Opejin, Michal Ordak, Raffaele Ornello, Doris V Ortega-Altamirano, Alberto Ortiz, Esteban Ortiz-Prado, Wael M S Osman, Uchechukwu Levi Osuagwu, Stanislav S Otstavnov, Mayowa O Owolabi, Ifeoluwa Temitayo Oyeyemi, Ahmad Ozair, Mahesh Padukudru P A, Kevin Pacheco-Barrios, Alicia Padron-Monedero, Jagadish Rao Padubidri, Tamás Palicz, Raul Felipe Palma-Alvarez, Feng Pan, Songhomitra Panda-Jonas, Deepshikha Pande Katare, Anamika Pandey, Ashok Pandey, Seithikurippu R Pandi-Perumal, Leonidas D Panos, Ioannis Pantazopoulos, Paraskevi Papadopoulou, Shahina Pardhan, Pragyan Paramita Parija, Romil R Parikh, Nicholas Parsons, Roberto Passera, Dimitrios Patoulias, Uttam Paudel, Shrikant Pawar, Amy E Peden, Paolo Pedersini, Prince Peprah, Maria Odete Pereira, Mario F P Peres, Arokiasamy Perianayagam, Norberto Perico, Simone Perna, Richard G Pestell, Ionela-Roxana Petcu, Fanny Emily Petermann-Rocha, Hoang Nhat Pham, Hoang Tran Pham, Michael R Phillips, Thomas Pilgrim, Michael A Piradov, Saeed Pirouzpanah, Evgenii Plotnikov, Dimitri Poddighe, Ramesh Poluru, Djordje S Popovic, Maarten J Postma, Akram Pourshams, Naeimeh Pourtaheri, Jalandhar Pradhan, Pranil Man Singh Pradhan, V Prakash, Manya Prasad, Elton Junio Sady Prates, Dimas Ria Angga Pribadi, Jagadeesh Puvvula, Ibrahim Qattea, Gangzhen Qian, Yanan Qiao, Alberto Raggi, Pankaja Raghav Raghav, Pracheth Raghuveer, Fakher Rahim, Md Jillur Rahim, Mahban Rahimifard, Vafa Rahimi-Movaghar, Md Mosfequr Rahman, Mohammad Hifz Ur Rahman, Mosiur Rahman, Muhammad Aziz Rahman, Amir Masoud Rahmani, Mohammad Rahmanian, Nazanin Rahmanian, Vahid Rahmanian, Rahem Rahmati, Setyaningrum Rahmawaty, Gerard Marshall Raj, Sathish Rajaa, Vinoth Rajendran, Pushp Lata Rajpoot, Prashant Rajput, Pradhum Ram, Mahmoud Mohammed

Ramadan, Majed Ramadan, Venkitachalam Ramanarayanan, Shakthi Kumaran Ramasamy, Sheena Ramazanu, Juwel Rana, Kritika Rana, Rishabh Kumar Rana, Chhabi Lal Ranabhat, Nemanja Rancic, Amey Rane, Annemarei Ranta, Mithun Rao, Sowmya J Rao, Sina Rashedi, Mohammad-Mahdi Rashidi, Ashkan Rasouli-Saravani, Devarajan Rathish, Santosh Kumar Rauniyar, Salman Rawaf, Christian Razo, Murali Mohan Rama Krishna Reddy, Elrashdy Moustafa Mohamed Redwan, Inayat Ur Rehman, Giuseppe Remuzzi, Nazila Rezaei, Mohsen Rezaeian, Hossein Rezazadeh, Taeho Gregory Rhee, Mavra A Riaz, Antonio Luiz P Ribeiro, Monica Rodrigues, Thales Philipe R Rodrigues da Silva, Jefferson Antonio Buendia Rodriguez, Leonardo Roever, Debby Syahru Romadlon, Allen Guy Ross, Himanshu Sekhar Rout, Bedanta Roy, Priyanka Roy, Simanta Roy, Guilherme de Andrade Ruela, Michele Russo, Godfrey M Rwegerera, Chandan S N, Aly M A Saad, Korosh Saber, Maha Mohamed Saber-Ayad, Cameron John Sabet, Siamak Sabour, Simona Sacco, Basema Ahmad Saddik, Erfan Sadeghi, Mohammad Reza Saeb, Umar Saeed, Sher Zaman Zaman Safi, Rajesh Sagar, Alireza Saghafi, Dominic Sagoe, Fatemeh Saheb Sharif-Askari, Amirhossein Sahebkar, Pragyan Monalisa Sahoo, Soumya Swaroop Sahoo, Mirza Rizwan Sajid, Afeez Abolarinwa Salami, Luciane B Salaroli, Mohamed A Saleh, Mohammed Z Y Salem, Giovanni A Salum, Sara Samadzadeh, Saad Samargandy, Yoseph Leonardo Samodra, Vijaya Paul Samuel, Abdallah M Samy, Juan Sanabria, Itamar S Santos, Milena M Santric-Milicevic, Made Ary Sarasmita, Aswini Saravanan, Yaser Sarikhani, Gargi Sachin Sarode, Sachin C Sarode, Maheswar Satpathy, Zafer Sattouf, Ganesh Kumar Saya, Md Abu Sayeed, Mehdi Sayyah, Nikolaos Scarmeas, Benedikt Michael Schaarschmidt, Markus P Schlaich, Maria Inês Schmidt, Ione Jayce Ceola Schneider, Art Schuermans, Austin E Schumacher, Aletta Elisabeth Schutte, David C Schwebel, Siddharthan Selvaraj, Parijat Sen, Sabyasachi Senapati, Subramanian Senthilkumaran, Mihretu Tagesse Sergindo, Yashendra Sethi, Allen Seylani, Mahan Shafie, Pritik A Shah, Saeed Shahabi, Ataollah Shahbandi, Samiah Shahid, Hamid R Shahsavari, Moyad Jamal Shahwan, Masood Ali Shaikh, Ali S Shalash, Muhammad Aaqib Shamim, Mehran Shams-Beyranvand, Anas Shamsi, Alfiya Shamsutdinova, Mohd Shanawaz, Mohammed Shannawaz, Medha Sharath, Amin Sharifan, Azam Sharifi, Javad Sharifi-Rad, Anupam Sharma, Manoj Sharma, Sourabh Sharma, Ujjawal Sharma, Vishal Sharma, Rahim Ali Sheikhi, Adithi Shetty, Mahabalesh Shetty, Premalatha K Shetty, Desalegn Shiferaw, Mika Shigematsu, Tariku Shimels, Min-Jeong Shin, Rahman Shiri, Aminu Shittu, Abdul-karim Olayinka Shitu, Ivy Shiue, Seyed Afshin Shorofi, Sunil Shrestha, Kerem Shuval, Yafei Si, Emmanuel Edwar Siddig, Mithun Sikdar, João Pedro Silva, Luís Manuel Lopes Rodrigues Silva, Abhinav Singh, Baljinder Singh, Garima Singh, Harmanjit Singh, Jasvinder A Singh, Kuldeep Singh, Narinder Pal Singh, Paramdeep Singh, Puneetpal Singh, Jussi O T Sipilä, Shravan Sivakumar, Valentin Yurievich Skryabin, Anna Aleksandrovna Skryabina, David A Sleet, Farrukh Sobia, Bogdan Socea, Abdullah Al Mamun Sohag, Ranjan Solanki, Shipra Solanki, Yerukneh Solomon, Yi Song, Soroush Soraneh, Reed J D Sorensen, Houman Sotoudeh, Ireneous N Soyiri, Michael Spartalis, Chandrashekhar T Sreeramareddy, Suresh Kumar Srinivasamurthy, Panagiotis Stachteas, Lauryn K Stafford, Benjamin A Stark, Antonina V Starodubova, Narayan Subedi, Vetriselvan Subramaniyan, Muhammad Suleman, Abida Sultana, Zhong Sun, Johan Sundström, Vinay Suresh, Sri Susanty, Chandan Kumar Swain, Lukasz Szarpak, Sree Sudha T Y, Payam Tabaee Damavandi, Rafael Tabarés-Seisdedos, Seyyed Mohammad Tabatabaei, Shima Tabatabai, Celine Tabche, Mohammad Tabish, Jyothi Tadakamadla, Santosh Kumar Tadakamadla, Amirmasoud Taheri, Jabeen Taiba, Iman M Talaat, Ashis Talukder, Mircea Tampa, Jacques Lukenze Tamuzi, Ker-Kan Tan, Haosu Tang, Manoj Tanwar, Ingan Ukur Tarigan, Elvis Enowbeyang Tarkang, Nathan Y Tat, Seyed

Mohammad Tavangar, Arash Tehrani-Banihashemi, Mojtaba Teimoori, Mohamad-Hani Temsah, Reem Mohamad Hani Temsah, Masayuki Teramoto, Wegen Beyene Tesfamariam, Edosa Geta Tesfaye Gta, Ramna Thakur, Pugazhenthan Thangaraju, Rajshree Thapa, Rekha Thapar, Rasiah Thayakaran, Sathish Thirunavukkarasu, Joe Thomas, Nikhil Kenny Kenny Thomas, Amanda G Thrift, Jing Tian, Ales Tichopad, Jansje Henny Vera Ticoalu, Chalachew Tiruneh, Krishna Tiwari, Amir Tiyuri, Marcello Tonelli, Roman Topor-Madry, Marcos Roberto Tovani-Palone, Khaled Trabelsi, Ngoc Ha Tran, Thang Huu Tran, Nguyen Tran Minh Duc, Domenico Trico, Samuel Joseph Tromans, Thien Tan Tri Tai Truyen, Daniel Hsiang-Te Tsai, Aristidis Tsatsakis, Evangelia Eirini Tsermpini, Ermias A A Turuse, Stefanos Tyrovolas, Aniefiok John Udoakang, Arit Udoh, Atta Ullah, Sana Ullah, Muhammad Umair, Muhammad Umar, Brigid Unim, Bhaskaran Unnikrishnan, Daniele Urso, Jibrin Sammani Usman, Marco Vacante, Seyed Mohammad Vahabi, Sanaz Vahdati, Asokan Govindaraj Vaithinathan, Omid Vakili, Rohollah Valizadeh, Jef Van den Eynde, Orsolya Varga, Shoban Babu Varthya, Tommi Juhani Vasankari, Balachandar Vellingiri, Narayanaswamy Venketasubramanian, Madhur Verma, Massimiliano Veroux, Georgios-Ioannis Verras, Dominique Vervoort, Jorge Hugo Villafañe, Simona Villani, Manish Vinayak, Maria Viskadourou, Simona Ruxandra Volovat, Victor Volovici, Hatem A Wafa, Yasir Waheed, Waseem Wahood, Cong Wang, Fang Wang, Shu Wang, Song Wang, Yanzhong Wang, Yuan-Pang Wang, Mary Njeri Wanjau, Muhammad Waqas, Emebet Gashaw Wassie, Gizachew Tadesse Wassie, Zihan Wei, Robert G Weintraub, Haftom Legese Weldetinsaa, Dakshitha Praneeth Wickramasinghe, Nuwan Darshana Wickramasinghe, Tissa Wijeratne, Peter Willeit, Charles D A Wolfe, Yen Jun Wong, Utoomporn Wongsin, Chenkai Wu, Felicia Wu, YaJuan Wu, Zenghong Wu, Hong Xiao, Suowen Xu, Xiaoyue Xu, Kazumasa Yamagishi, Danting Yang, Yuichiro Yano, Amir Yarahmadi, Habib Yaribeygi, Yuichi Yasufuku, Hiroshi Yatsuya, Fereshteh Yazdanpanah, Mohammad Hosein Yazdanpanah, Pengpeng Ye, Renjulal Yesodharan, Saber Yezli, Siyan Yi, Xinglin Yi, Dehui Yin, Dong Keon Yon, Naohiro Yonemoto, Chuanhua Yu, Elaine A Yu, Ke Yun, Hadiza Yusuf, Siddhesh Zadey, Nima Zafari, Burhan Abdullah Zaman, Sojib Bin Zaman, Aurora Zanghì, Iman Zare, Fatemeh Zarimeidani, Armin Zarrintan, Michael Zastrozhin, Dawit Zemedikun, Youjie Zeng, Beijian Zhang, Haijun Zhang, Liqun Zhang, Yunquan Zhang, Zhiqiang Zhang, Hanqing Zhao, Claire Chenwen Zhong, Shang Cheng Zhou, Bin Zhu, Lei Zhu, Abzal Zhumagaliuly, Makan Ziafati, Magdalena Zielińska, Yossef Teshome Zikarg, Ghazal Zoghi, Sa'ed H Zyoud, Samer H Zyoud, Catherine O Johnson, Gregory A Roth, Balakrishnan Sukumaran Nair, Ilari Rautalin, Anjali Bhati, Catherine Bisignano, Theo Vos, and Christopher J L Murray.

Affiliations

National Institute for Stroke and Applied Neurosciences (Prof V L Feigin PhD, A Bhati PhD, B S Nair MPH, I Rautalin PhD), Auckland University of Technology, Auckland, New Zealand; Institute for Health Metrics and Evaluation (Prof V L Feigin PhD, A Y Aravkin PhD, D Bryazka BA, K Burkart PhD, R M Cogen BA, X Dai PhD, Prof L Dandona MD, Prof R Dandona PhD, L S Flor MPH, E B Hamilton MPH, Prof S I Hay FMedSci, Prof S S Lim PhD, T Mestrovic PhD, Prof A H Mokdad PhD, C Razo PhD, A E Schumacher PhD, R J D Sorensen PhD, L K Stafford MS, B A Stark MA, C O Johnson PhD, G A Roth MD, C Bisignano MPH, Prof T Vos PhD, Prof C J L Murray DPhil), Department of Applied Mathematics (A Y Aravkin PhD), Department of Health Metrics Sciences, School of Medicine (A Y Aravkin PhD, K Burkart PhD, X Dai PhD, Prof R Dandona PhD,

L S Flor MPH, Prof S I Hay FMedSci, Prof S S Lim PhD, Prof A H Mokdad PhD, G A Roth MD, Prof T Vos PhD, Prof C J L Murray DPhil), School of Medicine (E J Boyko MD), School of Health Systems and Public Health (C C Iwu MPH), Department of Neurology (R Kalani MD), Department of Anesthesiology & Pain Medicine (V Krishnamoorthy MD), Division of Plastic and Reconstructive Surgery (S D Morrison MD), Department of Global Health (R J D Sorensen PhD), Division of Cardiology (G A Roth MD), University of Washington, Seattle, WA, USA; Third Department of Neurology (E V Gnedovskaya PhD), Research Center of Neurology, Moscow, Russia (Prof V L Feigin PhD, Prof M A Piradov DSc); Department of Nursing (M D Abate MSc), Woldia University, Woldia, Ethiopia; Department of Clinical Governance and Quality Improvement (Y H Abate MSc), Aleta Wondo General Hospital, Aleta Wondo, Ethiopia; Department of Epidemiology (S Abd ElHafeez DrPH), Neurology Department (M Doheim MD), Tropical Health Department (R M Ghazy PhD), Department of Pathology (Prof I M Talaat PhD), Alexandria University, Alexandria, Egypt; Department of Neurology (Prof F Abd-Allah MD, Prof A Abdelalim MD, A Abualhasan MD), Department of Internal Medicine (A Y Afify MD), Public Health and Community Medicine Department (Prof T T Amin MD), Cairo University, Cairo, Egypt; Department of Mathematics and Sciences (A Abdelkader PhD), Department of Clinical Sciences (S O Alalalmeh BPharm, O E Hegazi BPharm), Nonlinear Dynamics Research Center (NDRC) (Prof S Momani PhD), Center for Medical and Bio-Allied Health Sciences Research (Prof M J Shahwan PhD, A Shamsi PhD, S H Zyoud PhD), Ajman University, Ajman, United Arab Emirates; Department of Surgery (M Abdelmasseh MD, Prof J Sanabria MD), Marshall University, Huntington, WV, USA; Department of Tropical Medicine and Infectious Diseases (S Abd-Elsalam PhD), Department of Pediatrics (A H Darwish MD), Tanta University, Tanta, Egypt; Department of Medicine (P Abdi BEng), Memorial University, St. John's, NL, Canada; Minimally Invasive Surgery Research Center (A Abdollahi MD, A Kabir MD), School of Medicine (F Afrashteh MD, M Bastan MD, M Dodangeh MD), Health Management and Economics Research Center (J Arabloo PhD, A Nasrollahizadeh MD), Department of Radiology (M Dastmardi MD), Department of Cardiology (A Ghaffari Jolfayi MD), Endocrine Research Center (A Karimi Behnagh MD), Department of Echocardiography (A Karimi Behnagh MD), Eye Research Center (H Kasraei MD, M Ziafati MD), Department of Health Economics (M Khosravi PhD), Department of Anesthesiology (K Latifinaibin MD), Preventive Medicine and Public Health Research Center (A Tehrani-Banihashemi PhD), Department of Community and Family Medicine (A Tehrani-Banihashemi PhD), Department of Epidemiology and Biostatistics (A Tiyuri PhD), Iran University of Medical Sciences, Tehran, Iran (M Moradi MD); Department of Medicine (Prof M Abdoun PhD), University of Setif Algeria, Sétif, Algeria; Department of Health, Sétif, Algeria (Prof M Abdoun PhD); Evidence-Based Practice Center (EBP) (R Abd-Rabu MD), Departments of Radiology and Neurosurgery (S Ghozy MD), Department of Nephrology and Hypertension (N Nikravangolsefid MD), Department of Radiology (F Nugen PhD, A Zarrintan MD), Department of Cardiovascular Medicine (H Pham MD), Department of Informatics and Radiology (S Vahdati MD), Mayo Clinic, Rochester, MN, USA; Community and Maternity Nursing Unit (D M Abdulah MPH), College of Medicine (Prof A M Mohammad MD), Basic Sciences Department (B A Zaman PhD), University of Duhok, Duhok, Iraq; Department of Physiotherapy (A Abdullahi PhD, A W Awotidebe PhD, J S Usman PhD), Bayero University Kano, Kano, Nigeria; Department of Physiotherapy (A Abdullahi PhD), Federal University Wukari, Wukari, Nigeria; Department of Midwifery (M Abebe MSc), Dilla University, Dilla, Ethiopia; Postgraduate Department (Prof R Abeldaño Zuñiga PhD), University of Sierra Sur, Miahuatlan de Porfirio Diaz, Mexico; Yhteiskuntadatatieteen Keskus (Center for Social Data

Science) (Prof R Abeldaño Zuñiga PhD), Department of Public Health (Prof T Lallukka PhD), University of Helsinki, Helsinki, Finland (T J Meretoja MD); Department of Botany (E S Abhilash PhD), Sree Narayana Guru College Chelannur, Kozhikode, India; Department of Internal Medicine (O O Abiodun FWACP), Federal Medical Centre, Abuja, Nigeria; Department of Community Medicine (O Abiodun MPH), Babcock University, Ilishan-Remo, Nigeria; Department of Neurosurgery (R Abo Kasem MD), Medical University of South Carolina, Charleston, SC, USA; Department of Surgery (R Abo Kasem MD), Damascus University, Damascus, Syria; Department of Family and Community Health (R G Aboagye MPH), Department of Epidemiology and Biostatistics (L A Adzigbli BSc, S A Bosoka MPhil, R K Dowou MPhil), Department of Population and Behavioural Sciences (H Amu PhD, E Manu PhD, Prof E E Tarkang PhD), Institute of Health Research (P T Doegah PhD, M Immurana PhD), University of Health and Allied Sciences, Ho, Ghana; Department of Physical Pharmacy and Pharmacokinetics (M Abouzid PharmD), Poznan University of Medical Sciences, Poznan, Poland; Department of Pediatric Dentistry of the School of Dentistry (Prof L Abreu PhD), School of Nursing (A G da Silva PhD), Department of Maternal-Child Nursing and Public Health (Prof D C Malta PhD, Prof A C Micheletti Gomide Nogueira de Sá MSc, E J S Prates BS), Department of Clinical Medicine (Prof B R Nascimento PhD), Clinical Hospital (Prof B R Nascimento PhD), Department of Applied Nursing (Prof M O Pereira PhD), Department of Internal Medicine (Prof A P Ribeiro MD), Centre of Telehealth (Prof A P Ribeiro MD), Vaccination Research Observatory (T R Rodrigues da Silva PhD), Federal University of Minas Gerais, Belo Horizonte, Brazil; Department of Adult Health Nursing (W A Abrha MSc), Aksum University, Aksum, Ethiopia; Department of Anesthesiology (D Abtahi MD), Department of Biotechnology (S Aghamiri PhD), National Nutrition and Food Technology Research Institute (M Ajami PhD), Department of Urology (M Bonakdar Hashemi MD), Internal Medicine Department (H Borhany MD), School of Medicine (D J Ghadimi MD), Psychiatric Nursing and Management Department (F Ghadirian PhD), Faculty of Medicine (A Ghaffari Jolfayi MD), Department of Medical Genetics (M Ghasemi PhD), Center for Comprehensive Genetic Services (M Ghasemi PhD), Obesity Research Center (A Haj-Mirzaian MD), Research Center for Social Determinants of Health (A Halimi MSc), Department of Neurosurgery (H Khayat Kashani MD), Social Determinants of Health Research Center (A Kolahi MD, M Rashidi MD), Skull Base Research Center (I Mohammadzadeh MD), Student Research Committee (M Rahmanian MD), Department of Immunology (A Rasouli-Saravani PhD), Department of Epidemiology (S Sabour PhD), Department of Medical Education (S Tabatabai PhD), Shahid Beheshti University of Medical Sciences, Tehran, Iran (M Khayamzadeh MD); Department of Neurology (S Abu Rumeileh MD), Martin Luther University Halle-Wittenberg, Halle (Saale), Germany; Department of Nursing (H Abualruz PhD), Al Zaytoonah University of Jordan, Amman, Jordan; Department of Biopharmaceutics and Clinical Pharmacy (Prof E Abu-Gharbieh PhD), Department of Diagnostic Radiology and Nuclear Medicine (Prof A Al-Ibraheem MD), School of Pharmacy (Prof S Aburuz PhD, Prof Y Bustanji PhD), Clinical Department (Prof M M Ahmad PhD), Department of Mathematics (Prof S Momani PhD), The University of Jordan, Amman, Jordan; Clinical Sciences Department (Prof E Abu-Gharbieh PhD, S Adra MD, H J Barqawi MPhil, Prof M M Saber-Ayad PhD, Prof I M Talaat PhD), Department of Pharmacy Practice and Pharmacotherapeutics (Prof K H Alzoubi PhD, Prof H A Omar PhD), Department of Basic Biomedical Sciences (Prof Y Bustanji PhD), Department of Basic Medical Sciences (W El-Huneidi PhD), Basic Medical Sciences Department (A B Elmoselhi PhD), Research Institute of Medical & Health Sciences (A B Elmoselhi PhD), Department of Clinical

Science (Prof M M Ramadan PhD), College of Medicine (Prof B A Saddik PhD, Prof M A Saleh PhD), Sharjah Institute of Medical Sciences (F Saheb Sharif-Askari PhD), University of Sharjah, Sharjah, United Arab Emirates; Academic Health System (H J Abukhadijah MPH), Department of Geriatric and Long Term Care (H Al Hamad MD), Rumailah Hospital (H Al Hamad MD), Hamad Medical Corporation, Doha, Qatar; Institute of Community and Public Health (Prof N M Abu-Rmeileh PhD), Birzeit University, Ramallah, Palestine; Department of Therapeutics (Prof S Aburuz PhD), Institute of Public Health (I Elbarazi DrPH, Prof M Grivna PhD), Family Medicine Department (M A Khan MSc), College of Medicine and Health Sciences (J Nauman PhD), United Arab Emirates University, Al Ain, United Arab Emirates; Department of Biochemistry and Molecular Medicine (A Abu-Zaid PhD), College of Medicine (Prof O Baltatu PhD), College of Pharmacy (R M H Temsah PharmD), Alfaisal University, Riyadh, Saudi Arabia; College of Graduate Health Sciences (A Abu-Zaid PhD), University of Tennessee, Memphis, TN, USA; Department of Clinical Medicine (Prof J M Acuna MD), American University of Antigua, Coolidge, Antigua and Barbuda; FIU Robert Stempel College of Public Health & Social Work (Prof J M Acuna MD), Robert Stempel College of Public Health and Social Work (S Alemohammad MD), Department of Translational Medicine (N C Barengo PhD), Department of Epidemiology (S Roy MPH), Florida International University, Miami, FL, USA; Department of Anesthesia and Critical Care (D E Adane MSc), Department of Public Health (M T Asemu MSc, A T Desale MPH), Department of Biomedical Sciences (M Jegnie PhD), Debre Tabor University, Debre Tabor, Ethiopia; College of Medicine and Health Sciences (M M Adane PhD), Department of Medical Laboratory Science (G M Aregu MSc), School of Public Health (M A Asemahagn PhD), Department of Public Health (M B Y Belayneh PhD), Department of Health Promotion and Behavioural Science (E K Bogale MPH), Department of Epidemiology and Biostatistics (G T Wassie MPH), Bahir Dar University, Bahir Dar, Ethiopia; School of Medicine (I Y Addo PhD), School of Architecture, Design, and Planning (Prof T Astell-Burt PhD), Westmead Clinical School (R Chimoriya PhD), Save Sight Institute (H Kandel PhD), Department of Public Health (M Khan PhD), University of Sydney, Sydney, NSW, Australia (S R Okeke PhD); Centre for Social Research in Health (I Y Addo PhD, S R Okeke PhD), School of Population Health (X Feng PhD, Prof B A Saddik PhD, Prof A E Schutte PhD, X Xu PhD), School of Medicine (Prof P K Maulik PhD), School of Public Health and Community Medicine (A E Peden PhD), School of Risk and Actuarial Studies (Y Si PhD), The George Institute for Global Health (P Ye PhD), University of New South Wales, Sydney, NSW, Australia; Department of Medical Rehabilitation (Prof R A Adedoyin PhD), Department of Animal Sciences (H A Bashiru PhD), Obafemi Awolowo University, Ile-Ife, Nigeria; Menzies School of Health Research (Prof O A Adegboye PhD), Charles Darwin University, Darwin, NT, Australia; Department of Obstetrics and Gynecology (V Adekanmbi PhD), Department of Family Medicine (W Lee PhD), University of Texas Medical Branch, Galveston, TX, USA; Department of Community Medicine (Prof K Adhikari PhD), Tribhuvan University, Bharatpur, Nepal; Public Health Section (Prof K Adhikari PhD), Himalayan Environment and Public Health Network (HEPHN), Chitwan, Nepal; Department of Public Health (Q Adnani PhD), Center of Excellence in Higher Education for Pharmaceutical Care Innovation (Prof M J Postma PhD), Universitas Padjadjaran (Padjadjaran University), Bandung, Indonesia; Technical Services Directorate (A A Afolabi MPH), MSI Nigeria Reproductive Choices, Abuja, Nigeria; Department of Life Sciences (M S Afzal PhD, Prof M Umair PhD), University of Management and Technology, Lahore, Pakistan; Department of Community Medicine (Prof S Afzal PhD), King Edward Memorial Hospital, Lahore, Pakistan; Department of Public Health (Prof S Afzal PhD), Public Health Institute,

Lahore, Pakistan; Department of Geography and Planning (W Agyemang-Duah PhD), Queen's University, Kingston, ON, Canada; School of Public Health (B O Ahinkorah MPhil), School of Life Sciences (G Liu PhD), School of Biomedical Engineering (N Tran MD), University of Technology Sydney, Sydney, NSW, Australia; Department of Medical Biochemistry (A Ahmad PhD), Department of Pharmacology (M Tabish MPharm), Shaqra University, Shaqra, Saudi Arabia; Department of Health and Biological Sciences (S Ahmad PhD), Abasyn University, Peshawar, Pakistan; Department of Natural Sciences (S Ahmad PhD), Gilbert and Rose-Marie Chagoury School of Medicine (Prof L Roever PhD), Lebanese American University, Beirut, Lebanon; Department of Medical Oncology (S Ahmad MD, A A Khosla MD), Miami Cancer Institute, Miami, FL, USA; Department of Community Medicine and Preventive Health (S Ahmad MD), King Edward Medical University Lahore, Lahore, Pakistan; School of Public Health (T Ahmad PhD), Zhejiang University, Hangzhou, China; Department of Neuroscience (A Ahmadzade MD), Department of Biostatistics (P Bahrami Taghanaki MD), Applied Biomedical Research Center (Prof A Sahebkar PhD), Biotechnology Research Center (Prof A Sahebkar PhD), Department of Medical Informatics (S Tabatabaei PhD), Clinial Research Development Unit (S Tabatabaei PhD), Department of Medicine (A Yarahmadi PhD), Department of Medical Genetics (N Zafari MD), Mashhad University of Medical Sciences, Mashhad, Iran; Department of Pharmacy Practice (A Ahmed PhD), Riphah Institute of Pharmaceutical Sciences, Islamabad, Pakistan; Division of Infectious Diseases and Global Public Health (IDGPH) (A Ahmed PhD), University of California San Diego, San Diego, CA, USA; Institute of Endemic Diseases (A Ahmed MSc), Unit of Basic Medical Sciences (E E Siddig MD), University of Khartoum, Khartoum, Sudan; Swiss Tropical and Public Health Institute (A Ahmed MSc), University of Basel, Basel, Switzerland; Department of Biosciences (H Ahmed PhD), COMSATS Institute of Information Technology, Islamabad, Pakistan; Brody School of Medicine (S Ahmed PhD), Department of Geography (A Opejin MS), East Carolina University, Greenville, NC, USA; Faculty of Medicine and Public Health (B Aji DrPH), Jenderal Soedirman University, Purwokerto, Indonesia; Moyen Mono Health District (E Akara MD), Ministry of Health, Tohoun, Togo; Institute for Advanced Medical Research and Training (Prof R O Akinyemi PhD), Department of Epidemiology and Medical Statistics (A F Fagbamigbe PhD), Department of Periodontology and Community Dentistry (O F Fagbule FWACS), Department of Health Promotion and Education (S Ibitoye PhD), College of Medicine (A P Okekunle PhD), Department of Medicine (O V Olalusi MD, Prof M O Owolabi DrM), Faculty of Public Health (I I Olufadewa MHS), University of Ibadan, Ibadan, Nigeria; Institute of Neuroscience (Prof R O Akinyemi PhD), Newcastle University, Newcastle upon Tyne, UK; Department of Cardiology (M Akkaif PhD, B Zhang PhD), School of Public Health (Prof H Kan PhD), Department of Health Management Center (X Li PhD), Fudan University, Shanghai, China; Chicago College of Osteopathic Medicine (A E Akrami BS), Midwestern University, Downers Grove, IL, USA; Feinberg School of Medicine (A E Akrami BS), Department of Neurological Surgery (J Fares MD), Northwestern University, Chicago, IL, USA; Department of Communicable Diseases (S Al Awaidy MSc), Centre of Studies and Research (S Jayapal PhD), Ministry of Health, Muscat, Oman; Middle East, Eurasia, and Africa Influenza Stakeholders Network, Muscat, Oman (S Al Awaidy MSc); Department of Surgery (S Al Hasan PhD), Department of Research and Development (Z Al-Aly MD), Washington University in St. Louis, St Louis, MO, USA; Al Al-Bayt University, Mafraq, Jordan (Prof M Al Qadire PhD); Department of Internal Medicine (O Al Ta'ani MD), Allegheny Health Network, Pittsburgh, PA, USA; School of Medicine (Y Al-Ajlouni MD), New York Medical College, Valhalla, NY, USA; Department of Epidemiology (Y Al-Ajlouni

MD), Columbia University, New York, USA; Department of Biology (T A Alalwan PhD, Prof S Perna PhD), College of Health and Sport Sciences (A G Vaithinathan MSc), University of Bahrain, Zallaq, Bahrain; Clinical Epidemiology Center (Z Al-Aly MD), US Department of Veterans Affairs (VA), St Louis, MO, USA; School of Nursing (R M Al-amer PhD), Department of Basic Sciences (Z Altaany PhD), Department of Basic Medical Sciences (M M Khatatbeh PhD), Yarmouk University, Irbid, Jordan; School of Nursing and Midwifery (R M Al-amer PhD), Translational Health Research Institute (R Chimoriya PhD, K Rana PhD), Department of Engineering (G R Naik PhD), Western Sydney University, Sydney, NSW, Australia; Division of Gastroenterology and Hepatology (W A Aldhaleei MD), Mayo Clinic, Jacksonville, FL, USA; General Directorate of Research and Studies (M S Aldossary MClinDent), Ministry of Health, Riyadh, Saudi Arabia; Department of Neurology (B Al-Fatly MSc), Institute of Public Health (F Fischer PhD), Charité Medical University Berlin, Berlin, Germany; Global Centre for Environmental Remediation (A A S Al-Gheethi PhD), School of Medicine and Public Health (P Atorkey MPhil), University of Newcastle, Newcastle, NSW, Australia; Cooperative Research Centre for Contamination Assessment and Remediation of the Environment, Newcastle, NSW, Australia (A A S Al-Gheethi PhD); College of Nursing (Prof F N Alhalaiqa PhD), Department of Population Medicine (Prof G Babu PhD), QU Health (M Mohammed PhD), Social and Economic Survey Research Institute (Prof A Perianayagam PhD), Qatar University, Doha, Qatar; Psychological Sciences Association, Amman, Jordan (Prof F N Alhalaiqa PhD); Adult Health and Critical Care Department (M Alharrasi PhD), Sultan Qabos University, Alseeb, Oman; Department of Zoology (A Ali PhD), Abdul Wali Khan University Mardan, Mardan, Pakistan; Department of Medical Rehabilitation (Physiotherapy) (M U Ali MSc), Department of Clinical Pharmacy and Pharmacy Administration (H Yusuf PhD), University of Maiduguri, Maiduguri, Nigeria; Department of Rehabilitation Sciences (M U Ali MSc, M Khan MPH, J S Usman PhD), School of Nursing (S Tyrovolas PhD), Hong Kong Polytechnic University, Hong Kong, China; Department of Biosciences (R Ali MPhil), Centre for Interdisciplinary Research in Basic Sciences (CIRBSc) (S Anwar PhD), Centre For Interdisciplinary Research In Basic Sciences (CIRBSc) (A Shamsi PhD), Jamia Millia Islamia, New Delhi, India; Center for Biotechnology and Microbiology (S S Ali PhD, M Suleman PhD), University of Swat, Swat, Pakistan; Department of Geography (W Ali PhD), Department of Adult Health and Critical Care (D Altwalbeh PhD), Department of Maternal and Child Health (J Arulappan DSc), Sultan Qaboos University, Muscat, Oman; Department of Nuclear Medicine (Prof A Al-Ibraheem MD), King Hussein Cancer Center, Amman, Jordan; Institute of Health and Wellbeing (S M Alif PhD), Federation University Australia, Melbourne, VIC, Australia; School of Public Health and Preventive Medicine (S M Alif PhD, D Gasevic PhD), Department of Medical Radiation Sciences (Prof A M Asbeutah PhD), School of Public Health and Preventative Medicine (Prof M Asghari-Jafarabadi PhD), School of Psychological Sciences (N Parsons PhD), Eastern Health Clinical School (R Thapa PhD), Department of Medicine (Prof A G Thrift PhD), Monash University, Melbourne, VIC, Australia; Department of Public Health and Community Medicine (Prof S M Aljunid PhD), Division of Community Medicine and Public Health (Prof C T Sreeramareddy MD), International Medical University, Kuala Lumpur, Malaysia; International Centre for Casemix and Clinical Coding (Prof S M Aljunid PhD), National University of Malaysia, Bandar Tun Razak, Malaysia; Department of Cardiology, Heart, Vascular, and Thoracic Institute (Prof W Almahmeed MD), Cleveland Clinic Abu Dhabi, Abu Dhabi, United Arab Emirates; College of Medicine and Health Sciences Academic Programs (Prof W Almahmeed MD), Department of Biology (W M S Osman PhD), Khalifa University, Abu Dhabi, United Arab Emirates; Department

of Dentistry (S Al-Marwani MSc), Independent Consultant, Sana'a, Yemen; Independent Consultant, Irbid, Jordan (S Al-Marwani MSc); Department of Physical Therapy and Rehabilitation Sciences (Prof M A Alomari PhD), Department of Rehabilitation Sciences and Physical Therapy (Prof M A Alomari PhD), Department of Allied Medical Sciences (A Alrawashdeh PhD), Department of Rehabilitation Sciences (M Al-Wardat PhD, M Etoom PhD), Faculty of Medicine (Prof M S I Alyahya PhD), Department of Clinical Pharmacy (Prof K H Alzoubi PhD), Department of Public Health (Prof Y S Khader PhD), Jordan University of Science and Technology, Irbid, Jordan; Research Program of Epidemiology and Public Health (J Alonso MD), Hospital del Mar Research Institute Barcelona (IMIM), Barcelona, Spain; Department of Experimental and Health Sciences (J Alonso MD), Biomedical Research Networking Center in Epidemiology and Public Health (CiberESP), Barcelona, Spain; Department of Respiratory Care (J S Alqahtani PhD), Prince Sultan Military College of Health Sciences, Dammam, Saudi Arabia; Department of Community Medicine (R M M Al-Raddadi MD, S Samargandy PhD), Department of Physical Therapy (F Khan PhD), Rabigh Faculty of Medicine (Prof A Malik PhD), Department of Dental Public Health (Z S Natto DrPH), King Abdulaziz University, Jeddah, Saudi Arabia; Department of Emergency Medicine (M A Alsabri MD), Sana'a University, Sanaa, Yemen; Pediatric Emergency Medicine Department (M A Alsabri MD), St. Christopher's Hospital for Children, Philadelphia, PA, USA; Department of Family and Community Medicine (N Z Alshahrani MD), University of Jeddah, Jeddah, Saudi Arabia; Institute of Molecular Biology and Biotechnology (A Altaf PhD, S Shahid PhD), University Institute of Radiological Sciences and Medical Imaging Technology (T Ashraf MS), Department of Oral Biology (A Fahim PhD), University Institute of Public Health (A Hanif PhD, S Nargus PhD), University Institute of Diet and Nutritional Sciences (A Khalil PhD), Research Centre for Health Sciences (RCHS) (S Shahid PhD), Lahore Business School (M Umar MBA), The University of Lahore, Lahore, Pakistan (M A Riaz Mcom); Research, Policy, and Training Directorate (A Al-Tammemi MPH), Jordan Center for Disease Control, Amman, Jordan; Applied Science Research Center (A Al-Tammemi MPH), Department of Clinical Nutrition and Dietetics (Prof M E M Faris PhD), Applied Science Private University, Amman, Jordan; Department of Allied Medical Science (D Altwalbeh PhD), Al-Balqa Applied University, Karak, Jordan; Research Group in Health Economics (Prof N Alvis-Guzman PhD), Universidad de Cartagena (University of Cartagena), Cartagena, Colombia; Research Group in Hospital Management and Health Policies (Prof N Alvis-Guzman PhD), Universidad de la Costa (University of the Coast), Barranquilla, Colombia; Department of Clinical Pharmacology and Toxicology (H Alwafi PhD), Umm Al-Qura University, Makkah, Saudi Arabia; Department of Medical Sciences (Prof Y M Al-Worafi PhD), Azal University for Human Development, Sana'a, Yemen; Department of Clinical Sciences (Prof Y M Al-Worafi PhD), University of Science and Technology of Fujairah, Fujairah, United Arab Emirates; Department of Pediatrics (Prof H Aly MD), Department of Thoracic Surgery (S Gulati MD), Department of Internal Medicine (M Gupta MD), Lerner Research Institute (X Liu PhD), Cleveland Clinic, Cleveland, OH, USA; Department of Pediatric Cardiology (S Aly MD), Boston Children's Hospital, Boston, MA, USA; Department of Pediatrics (S Aly MD), Brigham and Women's Hospital (Z Aryan MD), T.H. Chan School of Public Health (Prof T W Bärnighausen MD, P M S Pradhan MD), Center for Primary Care (S Basu PhD), Harvard Business School (F Caetano dos Santos PhD), Department of Epidemiology (S Carr MS), Division of Cardiovascular Medicine (G Chi MD), Division of Cardiology (I Y Elgendy MD), Department of Global Health and Population (A Iradukunda MD), Department of Health Policy and Oral Epidemiology (Z S Natto DrPH), Department of Physical Medicine and Rehabilitation (K Pacheco-Barrios MD), Harvard

University, Boston, MA, USA; Department of Biomedical Engineering (W A Al-Zyoud PhD), German Jordanian University, Amman, Jordan; Interdisciplinary Graduate Program in Human Toxicology (R Amani DVM), University of Iowa, Iowa City, IA, USA; Health Policy Research Center (R Amani DVM, A Ardekani MD, M Banakar PhD, H Kasraei MD, Y Sarikhani PhD, S Shahabi PhD), Health Information Management (A Bashiri PhD), Medical School (M Farjoud Kouhanjani MD), Epilepsy Research Center (M Farjoud Kouhanjani MD), Department of Biostatistics (H Molavi Vardanjani PhD, E Sadeghi PhD), Department of Medicine (A Yarahmadi PhD), Shiraz University of Medical Sciences, Shiraz, Iran; School of Global Public Health (P M Amegbor PhD), New York University, New York, NY, USA; Department of Public Health (T G Amera MPH, H Mohammed PhD), Dire Dawa University, Dire Dawa, Ethiopia; Department of Radiology and Radiological Science (A Amindarolzarbi MD), Department of Biostatistics (A Columbus MS), Russell H. Morgan Department of Radiology and Radiological Science (A Kamireddy MD), Department of Neurosurgery (F Kazemi MD), School of Medicine (A Ozair MD), Department of Health Policy and Management (D Vervoort MD), Division of Cardiology (M Viskadourou MD), Department of International Health (H Zhang MS), Johns Hopkins University, Baltimore, MD, USA; Quran and Hadith Research Center (S Amiri PhD), Baqiyatallah University of Medical Sciences, Tehran, Iran; Department of Maternal and Child Wellbeing (D A Amugsi PhD), African Population and Health Research Center, Nairobi, Kenya; Department of Medicine (G A Amusa MD), University of Jos, Jos, Nigeria; Department of Internal Medicine (G A Amusa MD), Jos University Teaching Hospital, Jos, Nigeria; Faculty of Pharmacy (Prof R Ancuceanu PhD), Department of Internal Medicine (M Hostiuc PhD), Department of Legal Medicine and Bioethics (Prof S Hostiuc PhD), Department of General Surgery (I Negoi PhD, B Socea PhD), Department of Anatomy and Embryology (R I Negoi PhD), Department of Dermatology (M Tampa PhD), Carol Davila University of Medicine and Pharmacy, Bucharest, Romania; Centre for Sensorimotor Performance (D Anderlini MD), School of Health and Rehabilitation Sciences (A Khan PhD), Poche Centre for Indigenous Health (Prof A A Mamun PhD), The University of Queensland, Brisbane, QLD, Australia (M Moni PhD); Neurology Department (D Anderlini MD), Royal Brisbane and Women's Hospital, Brisbane, QLD, Australia; Department of Child Neurology (D Angappan MD), Oregon Health and Science University, Portland, OR, USA; Department of Pharmacology (A Anil MD, M Shamim MBBS, K Tiwari MBBS, S B Varthya MD), Department of Anatomy (N Bhardwaj MD), Department of Community Medicine and Family Medicine (Prof P Bhardwaj MD, Prof P R Raghav MD), School of Public Health (Prof P Bhardwaj MD), Department of Cardiology (R Choudhary MD), Department of Biochemistry (M Khokhar PhD), Department of Anaesthesiology and Critical Care (Prof N Kothari PhD), Department of Surgical Oncology (Prof S Misra MCh), Department of Pharmacology and Research (A Saravanan MD), Department of Community Medicine (G Singh MD), All India Institute of Medical Sciences, Jodhpur, India; All India Institute of Medical Sciences, Bhubaneswar, India (A Anil MD); School of Pharmacy (M T Ansari PhD), University of Nottingham Malaysia, Semenyih, Malaysia; Department of Epidemiology and Biostatistics (Prof A Ansari-Moghaddam PhD), Department of Health Promotion (A Nazri-Panjaki MSc), Health Promotion Research Center (H Okati-Aliabad PhD), Zahedan University of Medical Sciences, Zahedan, Iran; Department of Nursing (R Ansong MSc), Department of Urban Public Health (Prof P N Gona PhD), University of Massachusetts Boston, Boston, MA, USA; Regenerative Medicine, Organ Procurement and Transplantation Multi-disciplinary Center (S Anvari MD), Department of Social Medicine and Epidemiology (A Feizkhah MD), Brachial Plexus and Peripheral Nerve Injury Center (M Haghani Dogahe MD),

Department of Environmental Health Engineering (J Jaafari PhD), Gastrointestinal and Liver Diseases Research Center (F Joukar PhD), Caspian Digestive Disease Research Center (F Joukar PhD), Department of Ophthalmology (A Nabavi MD), Inflammatory Lung Diseases Research Center (N Rahmanian PhD), Guilan University of Medical Sciences, Rasht, Iran; School of Chemical and Life Sciences (SCLS) (S Anwar PhD), Jamia Hamdard, New Delhi, India; Department of Surgery (S Anwar PhD), Gadjah Mada University, Yogyakarta, Indonesia; Department of Medical Laboratory Sciences (E E Anyabolo BMLS), Department of Pharmacology and Therapeutics (Prof O E Onwujekwe PhD), University of Nigeria Nsukka, Enugu, Nigeria; Operations Department (E E Anyabolo BMLS), Breast Without Spot, Enugu, Nigeria; Rural Health Research Institute (A E Anyasodor PhD, S B Aychiluhm MPH), Rural Health research Institute (Prof A G Ross MD), Charles Sturt University, Orange, NSW, Australia; School of Medicine and Public Health (G C Apostol MD), Ateneo De Manila University, Pasig City, Philippines; Inter-Agency Committee on Environmental Health (G C Apostol MD), Department of Health Philippines, Manila, Philippines; Department of Social Sciences (F Appiah MPhil), Berekum College of Education, Berekum, Ghana; School of Public Health (F Appiah MPhil), Kwame Nkrumah University of Science and Technology, Kumasi, Ghana; Department of Psychology (M Aqeel PhD, M Aqeel PhD), Foundation University Islamabad, Rawalpandi, Pakistan; Urology Research Center (R Arabzadeh Bahri MD), Non-communicable Diseases Research Center (Z Aryan MD, M Bastan MD, E Mohammadi MD, P Mousavi MD, M Rashidi MD, N Rezaei MD), Dental Research Center (M Banakar PhD), School of Medicine (A Behnoush BS, H Farrokhpour MD, M Gouravani MD, H Karimi MD, A Khalaji MD, S Khanmohammadi MD, M Mayeli MD, S Mohammadi MD), Department of Scientific Research (F Chichagi MD), Iranian Research Center for HIV/AIDS (IRCHA) (O Dadras PhD), Department of obstetrics and Gynecology (M Dashtkoohi MD), Department of Neurology (M Dashtkoohi MD, M Shafie MD), Interdisciplinary Neuroscience Research Program (M Dashtkoohi MD, S Faghani MD), Department of Cardiology (Z Esmaeili MD, E Mahmoudi MD, P Mansouri MD), Endocrinology and Metabolism Research Institute (R Foroumadi MD), Department of Surgery (R Foroumadi MD), Sina Trauma and Surgery Research Center (M Hassan Zadeh Tabatabaei MD, Prof V Rahimi-Movaghar MD), Department of Virology (A Hosseinzadeh Adli PhD), Cardiovascular Diseases Research Institute (Y Karimi MD), Cardiac Primary Prevention Research Center (S Kazemian MD), Department of Cardiac Electrophysiology (S Kazemian MD), Children's Medical Center (Prof F Kompani MD), Department of Pediatric Cardiology (Prof E Malakan Rad MD), Digestive Diseases Research Institute (V Mansouri MD, Prof A Pourshams MD, M Yazdanpanah MD), Department of Epidemiology and Biostatistics (M Mansournia PhD), Faculty of Medicine (E Mohammadi MD, A Nasrollahizadeh MD, S Vahabi MD), Department of Physiotherapy (Prof N Nakhostin Ansari PhD), Research Center for War-affected People (Prof N Nakhostin Ansari PhD), Tehran Heart Center (A Nasrollahizadeh MD), Pharmaceutical Sciences Research Center (PSRC) (M Rahimifard PhD), Department of Medicine (A Shahbandi MD), Research Center for Rational Use of Drugs (A Sharifan PharmD), Department of Pathology (Prof S Tavangar MD), Department of Pediatric Allergy and Immunology (F Yazdanpanah MD), Tehran University of Medical Sciences, Tehran, Iran (M Dastmardi MD); College of Pharmacy (M Arafat PhD), AAU Health and Biomedical Research Center (Prof F El-Dahiyat PhD), Al Ain University, Abu Dhabi, United Arab Emirates; College of Art and Science (D Areda PhD), Ottawa University, Surprise, AZ, USA; School of Life Sciences (D Areda PhD), Arizona State University, Tempe, AZ, USA; College of Medicine and Health Sciences (B B Aregawi PhD), Department of Midwifery (M W Gebregergis MSc), Department

of Medical Laboratory Sciences (H N Meles MSc, H L Weldetinsaa MSc), Adigrat University, Adigrat, Ethiopia; Department of Public Health (O Aremu PhD), Birmingham City University, Birmingham, UK; Faculty of Nursing (H Arifin MKep), Department of Epidemiology Population Biostatistics and Health Promotion (A Hargono PhD), Department of Advanced Nursing (E M M Has PhD), Universitas Airlangga (Airlangga University), Surabaya, Indonesia; Department of Neurobiology, Care Sciences and Society (Prof J Ärnlöv PhD), Department of Neurobiology, Care Sciences, and Society (S Fereshtehnejad PhD), Department of Molecular Medicine and Surgery (Prof J H Kauppila MD), Karolinska Institute, Stockholm, Sweden; School of Health and Social Studies (Prof J Ärnlöv PhD), Dalarna University, Falun, Sweden; Institute for Biomedical Problems (A A Artamonov PhD), Russian Academy of Sciences, Moscow, Russia; Department of Research (U R Aryal PhD), Research Department (M Dhimal PhD, A Pandey MPH, U Paudel PhD), Nepal Health Research Council, Kathmandu, Nepal; Department of Radiologic Sciences (Prof A M Asbeutah PhD), Kuwait University, Sulaibikhat, Kuwait; Cabrini Research (Prof M Asghari-Jafarabadi PhD), Cabrini Health, Malvern, VIC, Australia; Department of Public Health (M Y Ashemo MPH), Department of Health Behavior and Society (L A A Ayana MPH), Institute of Health Science (A I Mohamed MSc), Department of Epidemiology (D Shiferaw MPH), Jimma University, Jimma, Ethiopia (B Feyisa MPH); Department of Public Health (M Y Ashemo MPH, E A A Turuse MPH), Public health (M T Sergindo MSc), Wachemo University, Hossana, Ethiopia; Department of Aging Research Institute (A Aslani MD), Immunology Research Center (M Dashti MD, A Ghasemzadeh MD), Department of Medical Surgical Nursing (M Ghafourifard PhD), Department of Virology (A Hosseinzadeh Adli PhD), Social Determinants of Health Research Center (S Karimi PhD), Faculty of Nursing and Midwifery (Prof M Mirghafourvand PhD), Department of Radiology (M Mirza-Aghazadeh-Attari MD), Neurosciences Research Center (NSRC) (R Mosaddeghi Heris MD), Student Research Committee (R Mosaddeghi Heris MD), Molecular Medicine Research Center (S Pirouzpanah PhD), Tabriz University of Medical Sciences, Tabriz, Iran; School of Medical Laboratory Sciences (H A Asmerom MSc), Department of Health Policy and Management (A T Debele MSc), School of Public Health (A Masrie MPH), Haramaya University, Harar, Ethiopia; Department of Immunology (S Athari PhD), Department of Critical Care and Emergency Nursing (N Hanifi PhD), Zanjan University of Medical Sciences, Zanjan, Iran; Hunter New England Population Health, Wallsend, NSW, Australia (P Atorkey MPhil); Faculty of Nursing (M M W Atout PhD), Philadelphia University, Amman, Jordan; Department of Forensic Medicine (A Atreya MD), Lumbini Medical College, Palpa, Nepal; Northumbria HealthCare NHS Foundation Trust, Newcastle upon Tyne, UK (A Aujayeb MBBS); School of Business (Prof M Ausloos PhD), Department of Health Sciences (S J Tromans PhD), University of Leicester, Leicester, UK; Department of Statistics and Econometrics (Prof M Ausloos PhD, A Mirica PhD, I Petcu PhD), Bucharest University of Economic Studies, Bucharest, Romania; Robarts Research Institute (A Avan MD), The University of Western Ontario, London, ON, Canada; Department of Health Sciences (H Awad PhD), Higher College of Technology, Abu Dhabi, United Arab Emirates; School of Nursing and Public Health (A W Awotidebe PhD, Prof E E Tarkang PhD), University of KwaZulu-Natal, Durban, South Africa; Department of Public Health (L A A Ayana MPH, E G Tesfaye Gta MSc), Department of Pharmacy (G Fekadu PhD), Department of Nursing (G Fetensa MSc), Institute of Health Sciences (B Feyisa MPH), Wollega University, Nekemte, Ethiopia; Institute of Public Health (S B Aychiluhm MPH), Gondar University, Gondar, Ethiopia; School of Nursing (A A Aynalem MSc), Hawassa University, Hawassa, Ethiopia; Department of Nursing (Z Aynalem MSc), Injibara University, Injibara, Ethiopia; Leeds Institute of Rheumatic and Musculoskeletal

Medicine (S Azadnajafabad MD), University of Leeds, Leeds, UK; Department of Medical-Surgical Nursing (H Azami MSc), Hamadan University of Medical Sciences, Hamadan, Iran; Institute of Biotechnology and Genetic Engineering (S Aziz MS), The University of Agriculture, Peshawar, Pakistan; Montefiore-Einstein Cerebrovascular Research Lab (A Azzam MBBCh), Albert Einstein College of Medicine, Bronx, NY, USA; Faculty of Medicine (A Azzam MBBCh), Department of Cardiology (O M Makram MD), October 6 University, 6th of October City, Egypt; Department of Physiotherapy (A S Babu PhD, S Kumaran D PhD), Department of Forensic Medicine and Toxicology (S M Bakkannavar MD), Prasanna School of Public Heath (PSPH) (V S Dsouza MSc), Kasturba Medical College, Mangalore (R Holla MD), Department of Forensic Medicine (Prof V C Nayak MD), Manipal TATA Medical College (M Rahman PhD), Kasturba Medical College Mangalore (M Rao MD, Prof B Unnikrishnan MD), Manipal Academy of Higher Education, Manipal, India (H L Dsouza MD); Gomal Center of Biochemistry and Biotechnology (M Badar PhD), Gomal University, Dera Ismail Khan, Pakistan; Department of Forensic Science (A D Badiye PhD, N Kapoor PhD), Government Institute of Forensic Science Nagpur, Nagpur, India; Rashtrasant Tukadoji Maharaj Nagpur University, Nagpur, India (A D Badiye PhD); School of Medicine (S Bahramian MD), Cardiology Department (M Mansouri MD), Department of Epidemiology and Biostatistics (K Mehrabani-Zeinabad PhD), Heart Failure Research Center (S Najdaghi MD, D Narimani Davani MD), Neuroscience Research Center (S Najdaghi MD), Faculty of Medicine (A Nasrollahizadeh MD), Department of Physiology (H Rezazadeh PhD), Department of Medical Physics (K Saber PhD), Department of Clinical Biochemistry (O Vakili PhD), Isfahan University of Medical Sciences, Isfahan, Iran; School of Public Affairs (R Bai MD), Nanjing University of Information Science and Technology, Nanjing, China; International Medical School (A A Baig PhD), Management and Science University, Alam, Malaysia; Department of Neurosurgery (A T Bako PhD), Houston Methodist Hospital, Houston, TX, USA (M Elhadi MD); Center of Innovation, Technology and Education (CITE) (Prof O Baltatu PhD), Anhembi Morumbi University, São José dos Campos, Brazil; Department of Medicine (K Bam MPH, M T Olaiya PhD), Stroke and Ageing Research Group (L L Dalli PhD), Department of Anatomy and Developmental Biology (Y Mathangasinghe PhD), Monash University, Clayton, VIC, Australia; Department of Hypertension (Prof M Banach PhD), Medical University of Lodz, Lodz, Poland; Polish Mothers' Memorial Hospital Research Institute, Lodz, Poland (Prof M Banach PhD); Nuffield Department of Surgical Sciences (S Bandyopadhyay MPH), Nuffield Department of Population Health (D A Bennett PhD, B Lacey DPhil), Department of Psychiatry (Prof C R J Newton MD), University of Oxford, Oxford, UK; Department of Neurosurgery (S Bandyopadhyay MPH), Southampton Clinical Trials Unit (P H Lee PhD), Faculty of Medicine (R Thayakaran PhD), University of Southampton, Southampton, UK; Department of Non-communicable Diseases (P C Banik MPhil, L Barua MPH), Bangladesh University of Health Sciences, Dhaka, Bangladesh; Department of Medicine (K Bansal MD), Department of Neurology (S Sivakumar MD), University of Massachusetts Medical School, Worcester, MA, USA; Department of Medicine (K Bansal MD), Department of Cardiovascular Medicine (A K Jha MD), Saint Vincent Hospital, Worcester, MA, USA; National Institute on Drug Dependence (Y Bao PhD), Department of Epidemiology and Health Statistics (Y Bao PhD), Institute of Child and Adolescent Health (Y Song PhD), School of Public Health (H Zhang MS), Peking University, Beijing, China; Department of Neurosciences (Prof M A Barboza MD), Costa Rican Department of Social Security, San Jose, Costa Rica; School of Medicine (Prof M A Barboza MD), University of Costa Rica, San Pedro, Costa Rica; Miller School of Medicine (M Bardhan MD), Department of Interventional Radiology (W Wahood MD), University of Miami,

Miami, FL, USA; School of Psychology (Prof S L Barker-Collo PhD), School of Pharmacy (K A Beyene PhD), University of Auckland, Auckland, New Zealand; Heidelberg Institute of Global Health (HIGH) (Prof T W Bärnighausen MD, S Chen DSc, Prof S Mohammed PhD), Department of Ophthalmology (S Panda-Jonas MD), Heidelberg University, Heidelberg, Germany; Department of Public and Environmental Health (A Barrow MPH), University of The Gambia, Banjul, The Gambia; Department of Epidemiology (A Barrow MPH, D Braithwaite PhD, D Yang MPH), College of Medicine (M J Diaz BS), University of Florida, Gainesville, FL, USA; Department of Veterinary Physiology and Biochemistry (A Basiru PhD), Department of Veterinary Public Health and Preventive Medicine (I A Odetokun PhD), University of Ilorin, Ilorin, Nigeria; School of Public Health (S Basu PhD, A Begde MSc), Department of Surgery and Cancer (Prof E Mossialos PhD), Department of Primary Care and Public Health (Prof S Rawaf MD, C Tabche MSc), Imperial College London, London, UK; Department of Academics (S Basu MD), Indian Institute of Public Health, Gurgaon, India; Department of Medical Education (K Batra PhD), Department of Social and Behavioral Health (Prof M Sharma PhD), University of Nevada Las Vegas, Las Vegas, NV, USA; School of Sport, Exercise and Health Sciences (A Begde MSc), Loughborough University, Loughborough, UK; Avicenna Biotech Research, Germantown, MD, USA (B Behnam MD); Department of Regulatory Affairs (B Behnam MD), Amarex Clinical Research, Germantown, MD, USA; Endocrinology and Metabolism Research Institute (H Farrokhpour MD, A Khalaji MD), Department of Epidemiology (S Khanmohammadi MD, S Nejadghaderi MD, S Rashedi MD), Non-Communicable Diseases Research Center (NCDRC), Tehran, Iran (A Behnoush BS, F Montazeri MD); Department of Public Health (M B Y Belayneh PhD), University of South Africa, Pretoria, South Africa; Department of Medicine and Surgery (M Belingheri MD), University of Milano - Bicocca, Milano, Italy; Direzione Sanitaria (Department of Health Management) (M Belingheri MD), Fondazione IRCCS San Gerardo dei Tintori, Monza, Italy; Department of Physiotherapy and Paramedicine (U M Bello PhD), Glasgow Caledonian University, Glasgow, UK; Department of Internal Medicine (I M Bensenor PhD, Prof A R Brunoni PhD, I S Santos PhD), Department of Psychiatry (Prof A R Brunoni PhD, Prof J Castaldelli-Maia PhD, Prof M F P Peres MD, Y Wang PhD), Department of Epidemiology (Prof A C Goulart PhD), Department of Medicine (Prof P A Lotufo DrPH), Center for Clinical and Epidemiological Research (A B Oliveira PhD, I S Santos PhD), University of São Paulo, São Paulo, Brazil; Department of Epidemiology and Biostatistics (F T Berhe MPH), Department of Environmental Health (M Gebrehiwot DSc), Wollo University, Dessie, Ethiopia (C Tiruneh MSc); School of Medicine and Dentistry (F T Berhe MPH, S Kundu MPH, M N Wanjau PhD), School of Dentistry and Oral Health (S K Tadakamadla PhD), Griffith University, Gold Coast, QLD, Australia; Department of Epidemiology and Biostatistics (Prof A C Bermudez MD), University of the Philippines Manila, Manila, Philippines; Department of Epidemiology (Prof A C Bermudez MD), Department of Biology and Medicine (P Fazeli MSc), The Warren Alpert Medical School (Z A Haq BA), Brown University, Providence, RI, USA; Metabolomics Laboratory (H B Beyene PhD), Hypertension and Kidney Disease Laboratory (Prof M P Schlaich MD), Baker Heart and Diabetes Institute, Melbourne, VIC, Australia; Department of Microbiology (H B Beyene PhD), Department of Medical Laboratory Science (M Getie MSc), Department of Anatomy (Y T Zikarg MSc), Addis Ababa University, Addis Ababa, Ethiopia; Department of Pharmaceutical and Administrative Sciences (K A Beyene PhD), University of Health Sciences and Pharmacy in St. Louis, St Louis, MO, USA; Department of Forensic Chemistry (D S Bhagat PhD), Government Institute of Forensic Science, Aurangabad, Aurangabad, India; Department of Public Health (A S Bhagavathula PhD), North Dakota State

University, Fargo, ND, USA; Institute of Applied Health Research (N Bhala PhD), University of Nottingham, Nottingham, UK; Institute of Applied Health Research (N Bhala PhD, K Malhotra MBBS, R Thayakaran PhD, D Zemedikun PhD), University of Birmingham, Birmingham, UK; Department of Internal Medicine (Prof A Bhalla MD), Post Graduate Institute of Medical Education and Research, Chandigarh, India; Global Health Neurology Lab (S Bhaskar PhD), NSW Brain Clot Bank, Sydney, NSW, Australia; Division of Cerebrovascular Medicine and Neurology (S Bhaskar PhD), National Cerebral and Cardiovascular Center, Suita, Japan; Department of General Medicine (A N Bhat MD, J Jeganathan MD), Department of Internal Medicine (A Boloor MD, M M R Reddy MD), Department of Community Medicine (J Jose MD, N Joseph MD, N Kumar MD, P Mithra MD, R Thapar MD), Department of Forensic Medicine and Toxicology (Prof J Padubidri MD), Department of Obstetrics and Gynaecology (A Shetty MS), Manipal College of Dental Sciences (Prof P K Shetty MDS), Manipal Academy of Higher Education, Mangalore, India; Department of Internal Medicine (V Bhat MBBS), St. John's National Academy of Health Sciences, Bangalore, India; Department of Medical Lab Technology (Prof G K Bhatti PhD), University Centre for Research and Development (S Kalra DM), Chandigarh University, Mohali, India; Department of Human Genetics and Molecular Medicine (Prof J S Bhatti PhD, S Senapati PhD, U Sharma PhD), Department of Biochemistry (B Singh PhD), Department of Zoology (B Vellingiri PhD), Central University of Punjab, Bathinda, India; Department of Pharmacy (Prof M A Bhuiyan PhD), University of Asia Pacific, Dhaka, Bangladesh; Department of Surgery (S Bhusal MBBS), University of Maryland School of Medicine, Baltimore, MD, USA; Scientific-Tools.Org, Bergamo, Italy (B Bikbov MD); Department of Radiology (C Bilgin MD), Neurovascular Research Laboratory (C Bilgin MD), Mayo Clinic College of Medicine, Rochester, MN, USA; Department of General Surgery and Medical-Surgical Specialties (Prof A Biondi PhD, Prof G Isola PhD), Department of Medical and Surgical Sciences and Advanced Technologies "GF Ingrassia" (Prof E D'Amico MD, Prof M Veroux PhD), Department of Biomedical and Biotechnological Sciences (L Falzone PhD, Prof G Grosso PhD), University of Catania, Catania, Italy; Department of Midwifery (K A Bishaw MSc), Department of Human Nutrition and Food Sciences (E G Wassie MSc), Debre Markos University, Debre Markos, Ethiopia; Department of Neurology (Prof A Biswas DM), Institute of Post-Graduate Medical Education and Research and Seth Sukhlal Karnani Memorial Hospital, Kolkata, India; Department of Community Medicine and Family Medicine (B Biswas MD), Department of Pharmacology (S T Y MD), All India Institute of Medical Sciences, Deoghar, India; Department of Community & Family Medicine (Prof T Bodhare MD), All India Institute of Medical Sciences, Ramanathapuram, Tamil Nadu, India; Faculty of Medicine and Pharmaceutical Sciences (A Bonny MD), University of Douala, Douala, Cameroon; Department of Cardiology (A Bonny MD), Centre Hospitalier Montfermeil (Montfermeil Hospital Center), Montfermeil, France; General Directorate of Health Information Systems (B Bora Basara PhD), Ministry of Health, Ankara, Turkiye; Disease Surveillance Department (S A Bosoka MPhil), Ghana Health Service, Ho, Ghana; Department of Medicine (Prof S Bouaoud DrPH), University Ferhat Abbas of Setif, Setif, Algeria; Department of Epidemiology and Preventive Medicine (Prof S Bouaoud DrPH), University Hospital Saadna Abdenour, Setif, Algeria; Department of Biology (Prof A Bouyahya PhD), Mohammed V University in Rabat, Rabat, Morocco; School of Medical and Life Sciences (Prof A Bouyahya PhD), Sunway University, Sunway, Malaysia; General Medicine Service (E J Boyko MD), Department of Veterans Affairs, Seattle, WA, USA; Faculty of Medicine (Prof M M Bozic PhD, I M Ilic PhD, Prof M M Santric-Milicevic PhD), School of Public Health and Health Management (Prof M M Santric-Milicevic PhD), University of

Belgrade, Belgrade, Serbia; University Eye Hospital (Prof M M Bozic PhD), Clinical Center of Serbia, Belgrade, Serbia; Cancer Population Sciences Program (D Braithwaite PhD), University of Florida Health Cancer Center, Gainesville, FL, USA; Institute for Medical Information Processing, Biometry, and Epidemiology (S Breitner DSc), LMU Munich, Neuherberg, Germany; Institute of Epidemiology (S Breitner DSc), Helmholtz Zentrum München (German Research Center for Environmental Health), Neuherberg, Germany; Division of Clinical Epidemiology and Aging Research (Prof H Brenner MD), German Cancer Research Center, Heidelberg, Germany; Center for Neuroscience (G Britton PhD), Institute for Scientific Research and High Technology Services, Panama City, Panama; Gorgas Memorial Institute for Health Studies, Panama City, Panama (G Britton PhD); Department of Medical and Surgical Sciences (Prof R Bugiardini MD, E Cenko MD), Department of Biomedical and Neuromotor Sciences (S Guicciardi MD, L Muccioli MD), University of Bologna, Bologna, Italy; College of Nursing and Health Sciences (L N Bulto PhD, K M Foley MPH), College of Medicine and Public Health (G R Naik PhD), Department of Nursing and Health Sciences (S Shorofi PhD), Flinders University, Adelaide, SA, Australia; School of Public Health Sciences (Z A Butt PhD), University of Waterloo, Waterloo, ON, Canada; Al Shifa School of Public Health (Z A Butt PhD), Al Shifa Trust Eye Hospital, Rawalpindi, Pakistan; Department of Internal Medicine (Prof L A Cámera MD), Hospital Italiano de Buenos Aires (Italian Hospital of Buenos Aires), Buenos Aires, Argentina; Board of Directors (Prof L A Cámera MD), Argentine Society of Medicine, Buenos Aires, Argentina; Institute of Biomedical Engineering (Prof L A Campos PhD), Anhembi Morumbi University, Sao Jose dos Campos, Brazil; Department of Biomedical Engineering (Prof L A Campos PhD), Center of Innovation, Technology and Education (CITE) at São José dos Campos Technology Park, Sao Jose dos Campos, Brazil; Center for Nutrition and Health Research (I R Campos-Nonato PhD, E Denova-Gutiérrez DSc), Center for Health Systems Research (S M Cuadra-Hernández PhD, D V Ortega-Altamirano EdD), Center for Evaluation and Surveys Research (V De la Cruz-Góngora PhD), National Institute of Public Health, Cuernavaca, Mexico; Department of Ophthalmology (F Cao MD), Beijing Institute of Ophthalmology, Beijing, China; Department of Health Management (Direzione Sanitaria) (A Capodici MD), IRCCS Istituto Ortopedico Rizzoli, Bologna, Italy; Interdisciplinary Research Center for Health Science (A Capodici MD), Sant'Anna School of Advanced Studies, Pisa, Italy; Department of Health Care (Prof R Cárdenas DSc), Metropolitan Autonomous University, Mexico City, Mexico; Oncological Network, Prevention and Research Institute (G Gorini MD), Institute for Cancer Research, Prevention and Clinical Network, Florence, Italy (G Carreras PhD); Institute for Mental and Physical Health and Clinical Translation (IMPACT) (A F Carvalho MD, Prof A E O'Neil PhD), Deakin University, Geelong, VIC, Australia; Research Unit on Applied Molecular Biosciences (UCIBIO) (Prof F Carvalho PhD, J P Silva PhD), Department of Chemical Sciences (R A S Couto MD), Institute for Research and Innovation in Health (i3S) (Prof N Cruz-Martins PhD), University of Porto, Porto, Portugal; Colombian National Health Observatory (C A Castañeda-Orjuela PhD), Instituto Nacional de Salud, Bogota, Colombia; Epidemiology and Public Health Evaluation Group (C A Castañeda-Orjuela PhD), National University of Colombia, Bogota, Colombia; Centre Neuchatelois de Psychiatrie, Marin Epagnier, Switzerland (G Castelpietra PhD); Mental Health Flagship (G Castelpietra PhD), World Health Organization (WHO), Copenhagen, Denmark; Department of Pharmacological and Biomolecular Sciences (Prof A L Catapano PhD), Department of Food, Environmental and Nutritional Sciences (I Cioffi PhD, Prof D Martini PhD), IRCCS Istituto Ortopedico Galeazzi (G Damiani MD), Department of Clinical Sciences and Community Health (Prof C La Vecchia MD), University of Milan, Milan,

Italy; MultiMedica Sesto San Giovanni IRCCS, Sesto San Giovanni, Italy (Prof A L Catapano PhD); Department of Public Health and Infectious Diseases (M S Cattaruzza PhD), La Sapienza University, Rome, Italy; Department of Medical, Surgical, and Health Sciences (L Cegolon PhD, Prof M D'Oria MD), University of Trieste, Trieste, Italy; Public Health Unit (L Cegolon PhD), University Health Agency Giuliano-Isontina (ASUGI), Trieste, Italy; Department of Nutrition (Prof F Cembranel DSc), Federal University of Santa Catarina, Florianópolis, Brazil; Mary MacKillop Institute for Health Research (Prof E Cerin PhD), Australian Catholic University, Melbourne, VIC, Australia; School of Public Health (Prof E Cerin PhD), Department of Urban Planning and Design (Prof C Guo PhD), University of Hong Kong, Hong Kong, China; Non-communicable Diseases Division (J Chadwick MD), Division of Epidemiology and Biostatistics (V K Kamal PhD), National Institute of Epidemiology, Chennai, India; Department of Biotechnology (Prof C Chakraborty PhD), Adamas University, Kolkata, India; Institute for Skeletal Aging & Orthopedic Surgery (Prof C Chakraborty PhD), Hallym University, Chuncheon, South Korea; State Disease Investigation Laboratory (S Chakraborty MVSc), Animal Resources Development Department, Agartala, India; Cardio-Oncology Research Unit (J Chan MPH), Cardiovascular Analytics Group, Hong Kong, China; Department of Clinical Nutrition (R M Chandika PhD), Department of Epidemiology (S Dohare MD, M Khan MD), Department of Health Informatics (A Mehmood PhD), Department of Health Education and Promotion (P Rajpoot PhD, M Shanawaz MD, F Sobia PhD), Jazan University, Jazan, Saudi Arabia; Department of Anesthesiology and Perioperative Medicine (E K Chandrasekar MD), School of Medicine (Prof S Xu PhD), University of Rochester, Rochester, NY, USA; Department of Clinical Pharmacy (G S Chanie MSc), Department of Human Physiology (M Diress MSc), University of Gondar, Gondar, Ethiopia; Temerty Faculty of Medicine (V Chattu MD), Division of Neurology (S Fereshtehnejad PhD), University of Toronto, Toronto, ON, Canada; Department of Community Medicine (V Chattu MD), Datta Meghe Institute of Medical Sciences, Sawangi, India; Department of Biology (A A Chaudhary PhD), Al-Imam Mohammad Ibn Saud Islamic University, Riyadh, Saudi Arabia; Oral Medicine and Radiology (A Chaurasia MD), Department of Neurology (V Suresh MBBS), King George's Medical University, Lucknow, India; Clinical Research Center (H Chen PhD), Zhujiang hospital of Southern Medical University, Guangzhou, China; Department of Endocrine and Metabolic Diseases (M Chen PhD), Shanghai Mental Health Center (Prof M R Phillips MD), Shanghai Jiao Tong University, Shanghai, China; Division of Infectious Diseases (P R Ching MD), Virginia Commonwealth University, Richmond, VA, USA; Iraq Field Epidemiology Training Program (I-FETP) (A Chitheer MD), Ministry of Health, Baghdad, Iraq; Program in Medical and Population Genetics (S J Cho PhD), Broad Institute of MIT and Harvard, Cambridge, MA, USA (M Kim MD); Cardiovascular Research Center (S J Cho PhD, A Schuermans BSc), Department of Radiology (A Haj-Mirzaian MD, X Liu PhD), Division of Cardiology (D H Nguyen BS), Massachusetts General Hospital, Boston, MA, USA (M Kim MD); Cancer Big Data Center (D Choi PhD), National Cancer Center, Goyang, South Korea; Department of Medicine (B Chong MBBS), Saw Swee Hock School of Public Health (S Ramazanu PhD, Prof S Yi PhD), Department of Surgery (K Tan PhD), Yong Loo Lin School of Medicine (Prof N Venketasubramanian MSc), National University of Singapore, Singapore, Singapore; RIPAS Hospital (C Chong MD), University of Brunei Darussalam, BSB, Brunei; Centre for Research Impact & Outcome (H Chopra PhD), Chitkara University, Rajpura, India; Department of Community Medicine (Prof S G Choudhari MD), Jawaharlal Nehru Medical College, Wardha, India; Center for Biomedicine and Community Health (D Chu PhD), International School, Vietnam National University Hanoi (VNUIS), Hanoi, Vietnam;

Department of Paediatric Surgery (I S Chukwu BMedSc), Federal Medical Centre, Umuahia, Nigeria; Department of Health Informatics (S Chung PhD), University College London, London, UK; Health Data Research UK, London, UK (S Chung PhD); Department of Genetics (Z Cindi PhD), Department of Medicine (J C Lin BS), Department of Biostatistics, Epidemiology, and Informatics (J Puvvula PhD), Department of Radiology (F Yazdanpanah MD), University of Pennsylvania, Philadelphia, PA, USA; Department of Epidemiology and Prevention (S Costanzo PhD), IRCCS Neuromed, Pozzilli, Italy; Department of Family Medicine and Public Health (Prof M H Criqui MD), University of California San Diego, La Jolla, CA, USA; Department of Diagnostic and Therapeutic Technologies (Prof N Cruz-Martins PhD), Cooperativa de Ensino Superior Politécnico e Universitário (Polytechnic and University Higher Education Cooperative), Vila Nova de Famalicão, Portugal; Department of Internal Medicine (S Dadana MD), Cheyenne Regional Medical Center, Cheyenne, WY, USA; Department of Global Public Health and Primary Care (O Dadras PhD), Department of Psychosocial Science (Prof D Sagoe PhD), University of Bergen, Bergen, Norway; Institute for Health Sciences (Prof K Dalal PhD), Mid Sweden University, Sundsvall, Sweden; Department of Dermatology (G Damiani MD), Harrington Heart and Vascular Institute (A Guha MD), Department of Quantitative Health Science (X Liu PhD), Department of Neonatology (I Qattea MD), Department of Nutrition and Preventive Medicine (Prof J Sanabria MD), Department of Medicine (Z Sattouf MD), Case Western Reserve University, Cleveland, OH, USA; Public Health Foundation of India, Gurugram, India (Prof L Dandona MD, Prof R Dandona PhD, G Kumar PhD, A Pandey PhD); Non-communicable Diseases Division (NCD) (A K Gupta PharmD), Department of Biostatistics (V K Kamal PhD), Indian Council of Medical Research, New Delhi, India (Prof L Dandona MD, D K Lal MD); Department of Biochemistry (S Das MD), Ministry of Health and Welfare, New Delhi, India; Department of Gynecology and Obstetrics (M Dashtkoohi MD), Vali-E-Asr Reproductive Health Research Center, Tehran, Iran; Health Research Institute (Prof K Davletov PhD), director of the scientific and technological park (I R Fakhradiyev PhD), Laboratory of Experimental Medicine (T Fazylov MD), Atchabarov Scientific-Research Institute of Fundamental and Applied Medicine (M Kulimbet MSc), Science Department (A Shamsutdinova MD), National Research Institute of Fundamental and Applied Medicine named after B. Atchabarov (A Zhumagaliuly MD), Kazakh National Medical University, Almaty, Kazakhstan; Department of Internal Medicine (S DeAngelo DO), John H. Stroger, Jr. Hospital of Cook County, Chicago, IL, USA; Medical College (S Debopadhaya BS), Albany Medical College, Albany, NY, USA; School of Medicine (I Delgado-Enciso DSc), University of Colima, Colima, Mexico; Department of Research (I Delgado-Enciso DSc), Colima State Cancer Institute, Colima, Mexico; USAID-JSI Digital Health Activity (B H Demessa MPH), Jimma University, Addis Ababa, Ethiopia; Department of Neurosurgery (A K Demetriades MD), The Centre for Global Health (D Gasevic PhD), College of Medicine and Veterinary Medicine (G Verras MD), University of Edinburgh, Edinburgh, UK; Department of Neurosurgery (A K Demetriades MD), National Health Service (NHS) Scotland, Edinburgh, UK; Department of Forensic Medicine (E Dervišević PhD), University of Sarajevo, Sarajevo, Bosnia and Herzegovina; Department of Research (H D Desai MD), Gujarat Adani Institute of Medical Sciences and G.K. General Hospital, Bhuj, India; Department of Public Health (F Desta MPH, A Jema MPH), Madda Walabu University, Goba, Ethiopia; Chettinad Hospital & Research Institute (Prof V Devanbu MD), Chettinad Academy of Research and Education, Chennai, India; JSS Medical College Department of Biochemistry (D Devegowda PhD), Jagadguru Sri Shivarathreeswara University, Mysuru, India; Department of Pharmacy (S Dewan PhD),

United International University, Dhaka, Bangladesh; Pharmacology Division (S Dewan PhD), Center for Life Sciences Research Bangladesh, Dhaka, Bangladesh; Research and Development Cell (A S Dhane MBA), Department of Oral Pathology and Microbiology (Prof G S Sarode PhD, Prof S C Sarode PhD), Dr. D. Y. Patil University, Pune, India; Institute of Occupational, Social and Environmental Medicine (M Dhimal PhD), Goethe University Frankfurt, Frankfurt am Main, Germany; The Zena and Michael A. Wiener Cardiovascular Institute (V R Dhulipala MD), Department of Cardiology (M Vinayak MD), Icahn School of Medicine at Mount Sinai, New York, NY, USA; Department of Social Medicine and Health Care Organisation (Prof K G Dokova PhD), Medical University of Varna, Varna, Bulgaria; Health Science Center (D Dongarwar MS), University of Texas, Houston, TX, USA; Cardio-Thoraco-Vascular Department (Prof M D'Oria MD), Azienda Sanitaria Universitaria Giuliano Isontina, Trieste, Italy; Independent Consultant, South Plainfield, NJ, USA (O P Doshi MSc); Department of Cardiology (R P Doshi MD), St. Joseph's University Medical Center, Paterson, NJ, USA; School of Population Health and Environmental Sciences (A Douiri PhD, H A Wafa MPH), School of Life Course & Population Sciences (Y Hbid PhD), Department of Twin Research and Genetic Epidemiology (M Mazidi PhD), Faculty of Life Sciences and Medicine (M Molokhia PhD), Institute of Psychiatry, Psychology & Neuroscience (D Urso MD), School of Life Course and Population Sciences (Prof Y Wang PhD), School of Lifecourse and population sciences (Prof C D A Wolfe MD), King's College London, London, UK; Department of Medicine (A C Dsouza MBBS, M Sharath MBBS), Bangalore Medical College and Research Institute, Bangalore, India; Department of Forensic Medicine and Toxicology (H L Dsouza MD), Kasturba Medical College Mangalore, Mangalore, India; Postgraduate Program in Epidemiology (Prof B B Duncan MD, Prof B N G Goulart DSc, Prof M I Schmidt MD), Department of Psychiatry and Legal Medicine (Prof G A Salum PhD), Federal University of Rio Grande do Sul, Porto Alegre, Brazil; School of Medicine (Prof A R Duraes PhD), Federal University of Bahia, Salvador, Brazil; Department of Internal Medicine (Prof A R Duraes PhD), Escola Bahiana de Medicina e Saúde Pública (Bahiana School of Medicine and Public Health), Salvador, Brazil; Department of Conservative Dentistry with Endodontics (A M Dziedzic DSc), Medical University of Silesia, Katowice, Poland; Faculty of Science and Health (M Ekholuenetale PhD), University of Portsmouth, Hampshire, UK; Department of Public Health and Community Medicine (Prof I F El Bayoumy DrPH), Texila American University, Tanta city, Egypt; School of Public health (Prof I F El Bayoumy DrPH), Texila American University, Guyana, Guyana; Department of Clinical Pathology (Prof M El Sayed Zaki PhD), Department of Cardiology (Prof M M Ramadan PhD), Faculty of Pharmacy (Prof M A Saleh PhD), Mansoura University, Mansoura, Egypt; Clinical Pharmacy Program (Prof F El-Dahiyat PhD), Al Ain University, Al Ain, United Arab Emirates; Division of Cardiovascular Medicine (I Y Elgendy MD, A Kundu MD), Division of Pulmonary and Critical Care (P Sen MD), University of Kentucky, Lexington, KY, USA; Faculty of Medicine (M Elhadi MD), Department of Radiology (A Msherghi MD), University of Tripoli, Tripoli, Libya; Egypt Center for Research and Regenerative Medicine (ECRRM), Cairo, Egypt (M A Elmonem PhD); Department of Pediatrics (C Eltaha MD), Texas A&M University, Dallas, TX, USA; Department of Public Health and Tropical Medicine (T I Emeto PhD), College of Public Health, Medical, and Veterinary Sciences (Prof R C Franklin PhD, A E Peden PhD), James Cook University, Townsville, QLD, Australia (K O Obamiro PhD); Department of Paediatrics (C I Esezobor MB), Department of Psychiatry (A T Olagunju MD), University of Lagos, Lagos, Nigeria; Department of Paediatrics (C I Esezobor MB), Lagos University Teaching Hospital, Lagos, Nigeria; Division of Epidemiology (N Esfandiari DVM), University of Tehran, Tehran, Iran; Dipartimento di Scienze

Biomediche e Neuromotorie (DIBINEM) (F Esposito MD), University of Bologna, BOLOGNA, Italy; Independent Consultant, Bologna, Italy (N Fabin MD); Division of Non-Communicable Diseases (I Fadhil PhD), Ministry of Public Health and Population, Dubai, United Arab Emirates; Research Centre for Healthcare and Community (A F Fagbamigbe PhD), Faculty of Health and Life Sciences (O P Kurmi PhD), Coventry University, Coventry, UK; Department of Periodontology and Community Dentistry (O F Fagbule FWACS), Department of Community Medicine (O S Ilesanmi PhD), Department of Neurology (O V Olalusi MD), Department of Medicine (Prof M O Owolabi DrM), Department of Oral and Maxillofacial Surgery (A A Salami BDS), University College Hospital, Ibadan, Ibadan, Nigeria; Epidemiology and Biostatistics Unit (L Falzone PhD), IRCCS Pascale, Naples, Italy; Center for Global Health Research (M Fareed PhD), Department of Public Health Dentistry (Prof G Mini PhD), Saveetha Institute of Medical and Technical Sciences (SIMATS), Chennai, India; Dissemination Division (C S e Farinha MSc), National Institute of Statistics, Lisbon, Portugal; Activity Planning and Control Unit (C S e Farinha MSc), Directorate-General of Health (DGS), Lisbon, Portugal; Department of Biology (P S Faris PhD), Department of Food Technology (Y Galali ResM), Salahaddin University-Erbil, Erbil, Iraq; Department of Biology (P S Faris PhD), Department of Nutrition and Dietetics (Y Galali ResM), Cihan University-Erbil, Erbil, Iraq; Department of Psychology (A Faro PhD), Federal University of Sergipe, São Cristóvão, Brazil; Satcher Health Leadership Institute (A O Fasanmi PhD), Morehouse School of Medicine, Atlanta, GA, USA; School of Medicine (A O Fasanmi PhD), Department of Gynecology and Obstetrics (T R Ojo-Akosile MD), Department of Cardiology (P Ram MD), Rollins School of Public Health (Prof D A Sleet PhD), Department of Family and Preventive Medicine (S Thirunavukkarasu PhD), Emory University, Atlanta, GA, USA; Centre for Public Health, Equity and Human Flourishing (N K Fauk PhD, K M Foley MPH), Torrens University Australia, Adelaide, SA, Australia; Institute of Resource Governance and Social Change, Kupang, Indonesia (N K Fauk PhD); Department of Public Health and Infectious Diseases (G Fekadu PhD), City University of Hong Kong, Hong Kong, China; National Institute of Environmental Health (X Feng PhD), National Center for Chronic and Noncommunicable Disease Control and Prevention (P Ye PhD), Chinese Center for Disease Control and Prevention, Beijing, China; Center for Public Health Research (P Ferrara PhD), School of Medicine and Surgery (Prof L G Mantovani DSc), University of Milan Bicocca, Monza, Italy; Laboratory of Public Health (P Ferrara PhD), IRCCS Istituto Auxologico Italiano, Milan, Italy; Department of Social Sciences (Prof N Ferreira PhD), University of Nicosia, Nicosia, Cyprus; Department of Neurology (Prof A Fonseca PhD), University of Lisbon, Lisbon, Portugal; Department of Neurology (Prof A Fonseca PhD), Hospital de Santa Maria, Lisbon, Portugal; Department of Pharmacology (Prof B Foroutan PhD), Iranshahr University of Medical Sciences, Iranshahr, Iran; Innovation in Healthcare and Social Services Department (D Fortuna MSc), Emilia-Romagna Region, Bologna, Italy; Department of Neuroscience (M Foschi MD), Multiple Sclerosis Research Center, Ravenna, Italy; Department of Biotechnological and Applied Clincial Sciences (M Foschi MD), University of L'Aquila, L'Aquila, Italy; Graduate Institute of Injury Prevention and Control (N Y Fridayani MS), School of Health Care Administration (L D Huy MBA), International Master Program for Translational Science (H Huynh BS), International Ph.D. Program in Medicine (L Minh MD), Research Center for Artificial Intelligence in Medicine (L Minh MD), Graduate Institute of Biomedical Informatics (D N A Ningrum PhD), Department of Clinical Pharmacy (M A Sarasmita PharmD), School of Nursing (S Susanty PhD), Global Health and Health Security Department (U Wongsin PhD), Taipei Medical University, Taipei, Taiwan; Department of Community Medicine and Family

Medicine (S G MD, V J MD, V Rajendran MD), All India Institute of Medical Sciences, Gorakhpur, India; Health Services Management Training Centre (Prof P A Gaal PhD, T Joo PhD, J Lám PhD, T Palicz MD), Semmelweis University, Budapest, Hungary; Department of Applied Social Sciences (Prof P A Gaal PhD), Sapientia Hungarian University of Transylvania, Târgu-Mureş, Romania; Department of Community Medicine (Prof A M Gaidhane MD), Datta Meghe Institute of Medical Sciences, Wardha, India; Department of Medicine (A Gaipov PhD, Prof D Poddighe PhD), Nazarbayev University, Astana, Kazakhstan; Department of Medical Epidemiology (S Gallus PhD), Mario Negri Institute for Pharmacological Research, Milan, Italy; Department of Community Medicine and Family Medicine (A P Gandhi MD), All India Institute of Medical Sciences, Nagpur, India; Institute of Health and Wellbeing (B Ganesan PhD), Federation University Australia, Churchill, VIC, Australia; Professional Services Division (P Gautam PhD), Texas State Board of Pharmacy, Austin, TX, USA; Department of Pharmacology (Prof R K Gautam PhD), Indore Institute of Pharmacy, Indore, India; Department of Public Health (L Getacher MPH), Department of Biomedical Sciences (Y Solomon MSc), Debre Berhan University, Debre Berhan, Ethiopia; Department of Public Health (G K Getahun MPH), Menelik II Medical and Health Science College, Addis Ababa, Ethiopia; Family and Community Medicine Department (R M Ghazy PhD), King Khalid University Hospital, Abha, Saudi Arabia; Electrical and Computer Engineering (E Gholami PhD), University of California Davis, Davis, CA, USA; Department of Physics (Z Gholami PhD), University of Zanjan, Zanjan, Iran; SYNAPSIS Mental Health and Neurology Non-Profit Organization, Lima, Peru (S Giannoni Luza MPH); Sensory Motor Laboratoy (S Giannoni Luza MPH), Fondation Asile des Aveugles, Lausanne, Switzerland; Department of Medicine (J A Gilani MD), Aga Khan University, Karachi, Pakistan; Adelaide Medical School (T K Gill PhD), Centre for Heart Rhythm Disorders (J Noubiap MD), University of Adelaide, Adelaide, SA, Australia; Division of General Internal Medicine (R F Gillum MD), Department of Community and Family Medicine (R F Gillum MD), Howard University, Washington, DC, USA; Department of Nursing (E Z Gindaba MSc), Ethiopian Public Health Institute, Chiro, Ethiopia; Department of Hepatology (Prof A Goel DM), Sanjay Gandhi Postgraduate Institute of Medical Sciences, Lucknow, India; Department of Dermatology (M Goldust MD), Department of Psychiatry (W Li PhD, T Rhee PhD), Department of Radiology and Biomedical Imaging (X Liu PhD), Department of Genetics (S Pawar PhD), Yale University, New Haven, CT, USA; Department of Health Systems and Policy Research (M Golechha PhD), Indian Institute of Public Health, Gandhinagar, India; Department of Genetics (P Goleij MSc), Sana Institute of Higher Education, Sari, Iran; Universal Scientific Education and Research Network (USERN) (P Goleij MSc), Kermanshah University of Medical Sciences, Kermanshah, Iran; Department of Life Sciences, Health and Healthcare Professions (Prof D Golinelli MD), Link Campus University, Rome, Italy; Health Services Research, Evaluation and Policy Unit (Prof D Golinelli MD), AUSL della Romagna, Ravenna, Italy; Department of Public Health and Preventive Medicine (Prof M Grivna PhD), Charles University, Prague, Czech Republic; Department of Endocrinology (A Grover MD), National Institute of Health, Bethesda, MD, USA; Department of Epidemiology and Biostatistics (S Guan MD), Anhui Medical University, Hefei, China; Post Graduate School of Public Health (G Guarducci MD), University of Siena, Siena, Italy; Division of Cardiovascular Medicine (A Guha MD), Department of Pediatrics (W D Lo MD), Ohio State University, Columbus, OH, USA; Health Directorate (S Guicciardi MD), Local Health Authority of Bologna, Bologna, Italy; Department of Community Medicine (D A Gunawardane MD, S N K Navaratna MD), University of Peradeniya, Kandy, Sri Lanka; Group Health Department (Z Guo MPH), Nanyang Central Hospital, Nanyang, China; Department of

Nephrology (A K Gupta PharmD), Max Super Speciality Hospital, New Delhi, India; Department of Public Health (B Gupta PhD), Torrens University Australia, Melbourne, VIC, Australia; Department of Cardiology (R Gupta MBBS), Lehigh Valley Health Network, Allentown, PA, USA; Department of Epidemiology and Biostatistics (R Gupta MPH), University of South Carolina, Columbia, SC, USA; Centre for Noncommunicable Diseases and Nutrition (R Gupta MPH), School of Pharmacy (M Islam PhD), BRAC University, Dhaka, Bangladesh; Department of Preventive Cardiology & Medicine (Prof R Gupta MD), Eternal Heart Care Centre & Research Institute, Jaipur, India; Department of Medicine (Prof R Gupta MD), Mahatma Gandhi University Medical Sciences, Jaipur, India; Department of Toxicology (S Gupta MSc), Shriram Institute for Industrial Research, Delhi, India; Global Virus Network, Middle East Region, Shiraz, Iran (F Habibzadeh MD); Department of Clinical Pharmacology and Medicine (Prof N R Hadi PhD), University of Kufa, Najaf, Iraq; Pharmaceutical Sciences Research Center (H Haghi-Aminjan PhD), Ardabil University of Medical Science, Ardabil, Iran; Department of Immunology (D Haghmorad PhD), Cancer Research Center (D Haghmorad PhD), Research Center of Physiology (H Yaribeygi PhD), Semnan University of Medical Sciences, Semnan, Iran; Biochemistry Department (Prof N M Hamdy PhD), Department of Obstetrics and Gynecology (Prof A F Nabhan PhD), Department of Entomology (A M Samy PhD), Medical Ain Shams Research Institute (MASRI) (A M Samy PhD), Neurology Department (Prof A S Shalash PhD), Ain Shams University, Cairo, Egypt; School of Health and Environmental Studies (Prof S Hamidi DrPH), Hamdan Bin Mohammed Smart University, Dubai, United Arab Emirates; Centre for Neuromuscular and Neurological Disorders (Prof G J Hankey MD), Dobney Hypertension Centre (Prof M P Schlaich MD), School of Population and Global Health (D Zemedikun PhD), The University of Western Australia, Perth, WA, Australia; Stroke Research Centre (Prof G J Hankey MD), Perron Institute for Neurological and Translational Science, Perth, WA, Australia; Department of Biochemistry and Molecular Biology (Prof M Hannan PhD), Bangladesh Agricultural University, Mymensingh, Bangladesh; Department of Anatomy (Prof M Hannan PhD), Dongguk University, Gyeongju, South Korea; Faculty of Medicine (N I Harlianto MD), Julius Centre for Health Sciences and Primary Care (G A Kayode PhD), Utrecht University, Utrecht, Netherlands; Department of Radiology (N I Harlianto MD), University Medical Center Utrecht, Utrecht, Netherlands; Research Unit (J M Haro MD), Parc Sanitari Sant Joan de Deu, Barcelona, Spain; Mental Health (J M Haro MD), Carlos III Health Institute (Prof R Tabarés-Seisdedos PhD), Biomedical Research Networking Center for Mental Health Network (CiberSAM), Madrid, Spain; School of Nursing and Midwivery (E M M Has PhD), La Trobe University, Bundoora, VIC, Australia; Department of Zoology and Entomology (A I Hasaballah PhD), Al-Azhar University, Cairo, Egypt; Department of Pharmaceutical Technology (I Hasan MPharm), University of Dhaka, Dhaka, Bangladesh; Department of Pharmacy (Prof M S Hasnain PhD), Palamau Institute of Pharmacy, Daltonganj, India; Public Health Department (I Hassan MPH), Dalhatu Araf Specialist Hospital, Lafia, Nigeria; Department of Public Health (I Hassan MPH), Federal University of Lafia, Lafia, Nigeria; Department of Diagnostic and Interventional Radiology and Neuroradiology (Prof J Haubold MD, Prof B M Schaarschmidt MD), Institute of Artificial Intelligence in Medicine (Prof J Haubold MD), University Hospital Essen, Essen, Germany; Skaane University Hospital (R J Havmoeller PhD), Skaane County Council, Malmö, Sweden; Faculty of Kinesiology (Prof J J Hebert PhD), University of New Brunswick, Fredericton, NB, Canada; School of Allied Health (Prof J J Hebert PhD), Murdoch University, Murdoch, WA, Australia; Community-Oriented Nursing Midwifery Research Center (M Heidari PhD), Modeling in Health Research Center (A Mohammadian-Hafshejani

PhD), Student Research Committee (R Rahmati MD), Department of Health in Disasters and Emergencies (R Sheikhi BHlthSci), Students Research Committee (F Zarimeidani Dipl), Shahrekord University of Medical Sciences, Shahrekord, Iran; Department of Medicine (M Hemmati MD), MedStar Health, Washington, DC, USA; Department of Medicine (M Hemmati MD), Georgetown University, Washington DC, DC, USA; Department of Public Health (D Z Heyi MPH), Madda Walabu University, Robe, Ethiopia; Department of Microbiology (K Hezam PhD), Taiz University, Taiz, Yemen; School of Medicine (K Hezam PhD), Nankai University, Tianjin, China; Division for Health Service Promotion (Y Hiraike PhD), Department of Global Health Policy (S Nomura PhD, S K Rauniyar PhD), University of Tokyo, Tokyo, Japan; School of Dentistry (N Hoan DDS), Department of Allergy, Immunology and Dermatology (D H Nguyen MD), Hanoi Medical University, Hanoi, Vietnam; Department of Pulmonology (N Horita PhD), Yokohama City University, Yokohama, Japan; National Human Genome Research Institute (NHGRI) (N Horita PhD), Center for Translation Research and Implementation Science (G A Mensah MD), National Institutes of Health, Bethesda, MD, USA; Department of Decision and Information Sciences (M Hossain DrPH), University of Houston, Houston, TX, USA; Public Health Research Group (M Hossain DrPH), Nature Study Society of Bangladesh, Khulna, Bangladesh; School of Health and Society (H Hosseinzadeh PhD), University of Wollongong, Wollongong, NSW, Australia; Institute of Research and Development (Prof M Hosseinzadeh PhD), Faculty of Medicine (H T H Nguyen MD), Institute for Research and Training in Medicine, Biology and Pharmacy (H T H Nguyen MD), Duy Tan University, Da Nang, Vietnam; Department of Computer Science (Prof M Hosseinzadeh PhD), Diplomacy and Public Relations Department (A Omar Bali PhD), University of Human Development, Sulaymaniyah, Iraq; Department of Clinical Legal Medicine (Prof S Hostiuc PhD), National Institute of Legal Medicine Mina Minovici, Bucharest, Romania; Department of Cardiology (B Hu PhD), The Fifth Clinical Medical School of Anhui Medical University, Hefei, China; Department of Cardiology (B Hu PhD), The Second People's Hospital of Hefei, Hefei, China; Department of Psychology (C Hu PhD), Tsinghua University, Beijing, China; Faculty of Medicine (J Huang MD), Department of Medicine & Therapeutics (Y Jin MD), Department of Medicine and Therapeutics (L Lim PhD), Jockey Club School of Public Health and Primary Care (C Zhong PhD), The Chinese University of Hong Kong, Hong Kong, China; Department of Public Health and Community Medicine (Prof A Humayun PhD), Shaikh Zayed Postgraduate Medical Institute, Lahore, Pakistan; Czech National Centre for Evidence-Based Healthcare and Knowledge Translation (S Hussain PhD), Institute of Biostatistics and Analyses (S Hussain PhD), Masaryk University, Brno, Czech Republic; College of Health Sciences (L D Huy MBA), VinUniversity, Hanoi, Vietnam; Department of Occupational Safety and Health (Prof B Hwang PhD), China Medical University Taiwan, Taichung, Taiwan; Department of Occupational Therapy (Prof B Hwang PhD), Asia University, Taiwan, Taichung, Taiwan; International Center for Nutrition and Information (N Ikeda PhD), National Institutes of Biomedical Innovation, Health and Nutrition, Tokyo, Japan; The National Centre for Remote and Rural Health and Care (A Ikiroma PhD), NHS National Services Scotland, Edinburgh, Scotland; West Africa RCC (O S Ilesanmi PhD), Africa Centre for Disease Control and Prevention, Abuja, Nigeria; Faculty of Medical Sciences (Prof M D Ilic PhD), University of Kragujevac, Kragujevac, Serbia; Department of Clinical Pharmacy (M Imam PhD), Department of Electrical Engineering (I Malik PhD), Prince Sattam bin Abdulaziz University, Al Kharj, Saudi Arabia; Department of Health Research (L R Inbaraj MD), ICMR National Institute for Research in Tuberculosis, Chennai, India; Faculty of Public Health (M Iqhrammullah PhD), Universitas Muhammadiyah Aceh, Banda Aceh, Indonesia; Department

of Research and Innovation (A Iradukunda MD), ARNECH Research and Consulting Office, Bujumbura, Burundi; Faculty of Pharmacy (L M Irham PhD), Universitas Ahmad Dahlan, Yogyakarta, Indonesia; Clinical Laboratory Department (F Ismail PhD), Tobruk University, Tobruk, Libya; Department of Blood Transmitted Diseases (F Ismail PhD), National Centre for Disease Control (NCDC), Tobruk, Libya; Department of Clinical Pharmacy & Pharmacy Practice (Prof N Ismail PhD), Asian Institute of Medicine, Science and Technology, Bedong, Malaysia; Malaysian Academy of Pharmacy, Puchong, Malaysia (Prof N Ismail PhD); Public Health Department of Social Medicine (Prof H Iso MD), Department of Biostatistics and Data Science, Graduate School of Medicine (Y Yasufuku MSc), Osaka University, Suita, Japan; School of Management (R Itumalla PhD), The Apollo University, Chittoor, India; Department of Health Services Research (M Iwagami PhD), Department of Public Health Medicine (Prof K Yamagishi MD), University of Tsukuba, Tsukuba, Japan; Department of Non-Communicable Disease Epidemiology (M Iwagami PhD), Center for Global Mental Health (K N Koly MSc), Department of Health Services Research and Policy (Prof M McKee DSc), London School of Hygiene & Tropical Medicine, London, UK; Department of Physical Medicine (L Jacob MD), Université Paris Cité, Paris, France; Research and Development Unit (L Jacob MD), Biomedical Research Networking Center for Mental Health Network (CiberSAM), Barcelona, Spain; Department of Immunology (Prof A Jafarzadeh PhD), HIV/STI Surveillance Research Center (S Nejadghaderi MD), Applied Cellular and Molecular Research Center (M Nematollahi PhD), Kerman University of Medical Sciences, Kerman, Iran; Department of Immunology (Prof A Jafarzadeh PhD), Department of Epidemiology and Biostatistics (Prof M Rezaeian PhD), Rafsanjan University of Medical Sciences, Rafsanjan, Iran (H Rezazadeh PhD); College of Medicine and Medical Sciences (H Jahrami PhD), Arabian Gulf University, Manama, Bahrain; Ministry of Health, Manama, Bahrain (H Jahrami PhD); Department of Leukemia (A Jain MD), The University of MD Anderson Cancer Center, Houston, TX, USA; Statistics Unit (N Jain MD), Riga Stradins University, Riga, Latvia; Department of Health and Safety (A A Jairoun PhD), Dubai Municipality, Dubai, United Arab Emirates; Centre for Community Medicine (A Jaiswal MD), Department of Psychiatry (Prof R Sagar MD), All India Institute of Medical Sciences, New Delhi, India; The World Academy of Sciences UNESCO, Trieste, Italy (Prof M Jakovljevic PhD); Shaanxi University of Technology, Hanzhong, China (Prof M Jakovljevic PhD); Department of Environmental Engineering (Prof R Jalilzadeh Yengejeh PhD), Islamic Azad University, Ahvaz, Iran; Faculty of Medicine and Health Sciences (Prof B Janakiraman MSc), SRM Institute of Science and Technology (SRMIST), Chennai, India; School of Pharmacy and Pharmacology (A Jatau PhD), Menzies Institute for Medical Research (F Pan PhD, J Tian PhD), University of Tasmania, Hobart, TAS, Australia; Department of Biochemistry (Prof S Jayaram MD), Government Medical College, Mysuru, India; Department of Epidemiology and Health Promotion (Prof S Jee PhD), Yonsei University, Seoul, South Korea; Department of Internal Medicine (B M Jeswani MBBS), GCS Medical College, Hospital & Research Centre, Ahmedabad, India; Department of Nutrition (A Jeyakumar PhD), University of Nevada Reno, Reno, NV, USA; University of Johannesburg, Johannesburg, South Africa (A Jeyakumar PhD); Department of Community Medicine (R P Jha MSc), Dr. Baba Saheb Ambedkar Medical College & Hospital, Delhi, India; Department of Community Medicine (R P Jha MSc), Banaras Hindu University, Varanasi, India; Department of Public Health (Z Ji BA), Tongji University, Shanghai, China; Department of Public Health (H Jiang PhD), School of Nursing and Midwifery (Prof M Rahman PhD), La Trobe University, Melbourne, VIC, Australia; Melbourne School of Population and Global Health (H Jiang PhD), School of Health Sciences (A Meretoja MD),

Department of Neurology (Prof T Wijeratne MD), University of Melbourne, Melbourne, VIC, Australia; School of Public Health (S Jin MD), Sichuan University, Chengdu, China; Zoonoses Research Center (M Jokar DVM), Islamic Azad University, Karaj, Iran; Rothschild Foundation Hospital (Prof J B Jonas MD), Institute of Molecular and Clinical Ophthalmology Basel, Paris, France; Singapore Eye Research Institute, Singapore, Singapore (Prof J B Jonas MD); Hungarian Health Management Association, Budapest, Hungary (T Joo PhD, T Palicz MD); Department of Economics (C E Joshua BSc), National Open University, Benin City, Nigeria; Department of Family Medicine and Public Health (J J Jozwiak PhD), University of Opole, Opole, Poland; Institute of Family Medicine and Public Health (M Jürisson PhD), University of Tartu, Tartu, Estonia; Department of Social Work (M Kabir PhD), Pabna University of Science and Technology, Pabna, Bangladesh; School of Public Health (Z Kabir PhD), University College Cork, Cork, Ireland; Department of Oral and Maxillofacial Pathology (V Kadashetti MDS), Department of Public Health Dentistry (Prof S M Kondlahalli MD), Krishna Vishwa Vidyapeeth (Deemed to be University), Karad, India; Department of Endocrinology (S Kalra DM), Bharti Hospital Karnal, Karnal, India; Health Sciences (Prof M Kanaan PhD), University of York, York, UK; Sydney Eye Hospital (H Kandel PhD), South Eastern Sydney Local Health District, Sydney, NSW, Australia; Faculty of Dentistry (K K Kanmodi MPH, A A Salami BDS), University of Puthisastra, Phnom Penh, Cambodia; Office of the Executive Director (K K Kanmodi MPH), Cephas Health Research Initiative Inc, Ibadan, Nigeria; The Hansjörg Wyss Department of Plastic and Reconstructive Surgery (R S Kantar MD), Nab'a Al-Hayat Foundation for Medical Sciences and Health Care, New York, NY, USA; Cleft Lip and Palate Surgery Division (R S Kantar MD), Global Smile Foundation, Norwood, MA, USA; 2nd Cardiology Department (P Karakasis MSc), Second Department of Cardiology (D Patoulias PhD), 2nd Department of Cardiology (P Stachteas MSc), Aristotle University of Thessaloniki, Thessaloniki, Greece; School of Health Professions and Human Services (I M Karaye MD), Hofstra University, Hempstead, NY, USA; Department of Anesthesiology (I M Karaye MD), Montefiore Medical Center, Bronx, NY, USA; Institute for Epidemiology and Social Medicine (A Karch MD), University of Münster, Münster, Germany; Central Department of Public Health (P Karki BHlthSci), Institute of Medicine, Kathmandu, Nepal; Surgery Research Unit (Prof J H Kauppila MD), Oulu Business School (I Shiue PhD), Martti Ahtisaari Institute (I Shiue PhD), University of Oulu, Oulu, Finland; Public Health Foundation of India, New Delhi, India (H Kaur MPH); Medical Basic Sciences Research Institute (N Kaydi PhD), Student Research Committee (N Kaydi PhD), Department of Public Health (M A Khafaie PhD), Environmental Technologies Research Center, Medical Basic Sciences Research Institute (M A Khafaie PhD), Education Development Center (M Sayyah MD), Ahvaz Jundishapur University of Medical Sciences, Ahvaz, Iran; International Research Center of Excellence (G A Kayode PhD), Institute of Human Virology Nigeria, Abuja, Nigeria; Department of Human Nutrition (E Kesse-Guyot PhD), National Research Institute for Agriculture, Food and Environment, Jouy-en-Josas, France; Sorbonne Paris Nord University, Bobigny, France (E Kesse-Guyot PhD); Department of Public Health and Health Policy (I Khaing MPH), Hiroshima University, Hiroshima, Japan; Amity Institute of Forensic Sciences (H Khajuria PhD, B P Nayak PhD), Amity Institute of Pharmacy (K Munjal PhD), Amity Institute of Public Health (M Shannawaz PhD), Amity University, Noida, India; College of Health Sciences (N Khalid PhD), Abu Dhabi University, Adu Dhabi, United Arab Emirates; Population Science Department (M Khan PhD), Jatiya Kabi Kazi Nazrul Islam University, Mymensingh, Bangladesh; Primary Care Department (M A Khan MSc), NHS North West London, London, UK; Department of Clinical Pharmacy (Y H Khan PhD, T Mallhi PhD), Jouf University, Sakaka, Saudi Arabia;

College of Health, Wellbeing and Life Sciences (Prof K Khatab PhD), Sheffield Hallam University, Sheffield, UK; College of Arts and Sciences (Prof K Khatab PhD), Ohio University, Zanesville, OH, USA; Faculty of Nursing (H Khatatbeh PhD), Jerash University, Jerash, Jordan; School of Medicine (S Khateri MD), Department of Epidemiology and Biostatistics (Y Moradi PhD), Kurdistan University of Medical Sciences, Sanandaj, Iran; Global Consortium for Public Health Research (Prof M Khatib PhD), Datta Meghe Institute of Higher Education and Research, Wardha, India; Academy of Medical Science, Tehran, Iran (M Khayamzadeh MD); Department of Biochemistry (F Khidri PhD), Liaquat University Of Medical and Health Sciences, Jamshoro, Pakistan; Department of Internal Medicine (A A Khosla MD), Corewell Health East William Beaumont University Hospital, Royal Oak, MI, USA; Department of Health Management and Economics (M Khosravi PhD), Clinical Research Development Center (CRDC) (A Saghafi MD), Qom University of Medical Sciences, Qom, Iran; Department of Public Health (J Khubchandani PhD), New Mexico State University, Las Cruces, NM, USA; Department of Cognition and Neuroscience (S Kian MD), University of Texas, Dallas, TX, USA; Graduate School of Public Health (K Kim PhD), Yonsei University, Busan, South Korea; School of Traditional Chinese Medicine (Y Kim PhD), Xiamen University Malaysia, Sepang, Malaysia; Millennium Prevention, Inc., Westwood, MA, USA (R W Kimokoti MD); School of Health Sciences (Prof A Kisa PhD), Kristiania University College, Oslo, Norway; Department of International Health and Sustainable Development (Prof A Kisa PhD), Tulane University, New Orleans, LA, USA; Department of Nursing and Health Promotion (S Kisa PhD), Oslo Metropolitan University, Oslo, Norway; Health System and Population Studies Divisions (K N Koly MSc), Department of Maternal and Child health (S Noor MS), International Centre for Diarrhoeal Disease Research, Bangladesh, Dhaka, Bangladesh; Department of Neurosurgery (M Korja PhD, I Rautalin PhD), General Administration (A Meretoja MD), Comprehensive Cancer Center (T J Meretoja MD), Helsinki University Hospital, Helsinki, Finland; Department of Epidemiology and Evidence-Based Medicine (V A Korshunov PhD), I.M. Sechenov First Moscow State Medical University, Moscow, Russia; Department of General Practice and Family Medicine (Prof O Korzh DSc), Kharkiv National Medical University, Kharkiv, Ukraine; Independent Consultant, Jakarta, Indonesia (S Kosen MD); Department of Epidemiology (Prof K Kostev PhD), IQVIA, Frankfurt am Main, Germany; Department of Gynecology (Prof K Kostev PhD), Philipps-Universität Marburg, Marburg, Germany; Department of Biochemistry (A L Kotnis PhD), Department of Dentistry (Prof A Singh MDS), All India Institute of Medical Sciences, Bhopal, India; Kasturba Medical College, Manipal (S Koulmane Laxminarayana MD), Manipal College of Nursing (R Yesodharan MSc), Manipal Academy of Higher Education, Udupi, India; Department of Anthropology (Prof K Krishan PhD), Institute of Forensic Science & Criminology (V Sharma PhD), Panjab University, Chandigarh, India; Department of Forensic Medicine and Toxicology (V Krishna MD), Pondicherry University, Puducherry, India; Department of Anesthesiology (V Krishnamoorthy MD), Department of Population Health Sciences (J B Lusk MD), Duke Global Health Institute (C Wu PhD), Duke University, Durham, NC, USA; Department of Physiology (B Krishnan MD), Pravara Institute of Medical Sciences (Deemed to be University), Loni, India; Department of Neuroscience (Prof J Kruja PhD), University of Medicine, Tirana, Albania; Department of Neuroscience (Prof J Kruja PhD), Medical Sciences University Hospital, Tirana, Albania; Department of Demography (Prof B Kuate Defo PhD), Department of Social and Preventive Medicine (Prof B Kuate Defo PhD), University of Montreal, Montreal, QC, Canada; Faculty of Medicine (B Kucuk Bicer PhD), Gazi University, Ankara, Turkiye; Department of Mathematics (M Kuddus PhD), Department of Population Science and Human Resource

Development (Prof M Rahman PhD, Prof M Rahman DrPH), University of Rajshahi, Rajshahi, Bangladesh; Department of Biochemistry (Prof M Kuddus PhD), College of Public Health & Health Informatics (R Kumar PhD), University of Hail, Hail, Saudi Arabia; University of Environment and Sustainable Development, Somanya, Ghana (N Kugbey PhD); Center of Medicine and Public Health (M Kulimbet MSc), Asfendiyarov Kazakh National Medical University, Almaty, Kazakhstan; Department of Medicine (V Kulkarni MS), Digital Health and Informatics Directorate (Prof S M McPhail PhD), Queensland Health, Brisbane, QLD, Australia; Cardiothoracic Surgery Department (A Kumar MD), NYU Langone Health, New York City, NY, USA; Cardiothoracic Surgery Department (A Kumar MD), Medanta Hospital, Gurugram, India; Department of Cardiovascular Medicine (A Kumar MD), Cabrini Institute, Rochester, MN, USA; Department of Community Medicine (D Kumar MD), Rajendra Institute of Medical Sciences, Ranchi, India; Department of Nutrition and Food Science (S Kundu MPH), Patuakhali Science and Technology University, Patuakhali, Bangladesh; Section of Cardiology (Prof S K Kunutsor PhD), University of Manitoba, Winnipeg, MB, Canada; Department of Translational Health Sciences (Prof S K Kunutsor PhD), University of Bristol, Bristol, UK; Department of Medicine (O P Kurmi PhD), Department of Psychiatry and Behavioural Neurosciences (A T Olagunju MD), Department of Health Research Methods, Evidence, and Impact (O O Olasupo PhD), McMaster University, Hamilton, ON, Canada; Department of Health Services Research and Management (D Kusuma DSc), City University of London, London, UK; Faculty of Public Health (D Kusuma DSc), University of Indonesia, Depok, Indonesia; Department of General Surgery (L Kutikuppala BMedSc), Dr NTR University of Health Sciences, Vijayawada, India; Department of Nephrology (A Kuttikkattu MD), Pushpagiri Institute of Medical Sciences and Research Centre, Thiruvalla, India; Clinical Research Center (V Kytö MD), Turku University Hospital, Turku, Finland; Heart Center (V Kytö MD), Department of Clinical Neurosciences (J O T Sipilä PhD), University of Turku, Turku, Finland; National Institute for Health Research (NIHR) Oxford Biomedical Research Centre, Oxford, UK (B Lacey DPhil); Integrated Department of Epidemiology, Health Policy, Preventive Medicine and Pediatrics (Prof C Lahariya MD), Foundation for People-centric Health Systems, New Delhi, India; SD Gupta School of Public Health (Prof C Lahariya MD), Indian Institute of Health Management Research University, Jaipur, India; NEVES Society for Patient Safety, Budapest, Hungary (J Lám PhD); Unidad de Genética y Salud Pública (Prof I Landires MD), Instituto de Ciencias Médicas, Las Tablas, Panama; Ministry of Health (Prof I Landires MD), Hospital Joaquín Pablo Franco Sayas, Las Tablas, Panama; Department of Medical Sciences (Prof A O Larsson PhD, D Lindholm MD, Prof J Sundström PhD), Uppsala University, Uppsala, Sweden; Department of Clinical Chemistry and Pharmacology (Prof A O Larsson PhD), Uppsala University Hospital, Uppsala, Sweden; Department of Otorhinolaryngology (S Lasrado MS), Father Muller Medical College, Mangalore, India; International Society Doctors for the Environment, Arezzo, Italy (P Lauriola MD); Department of Neurology and Psychiatry (P M Lavados MD), German Clinic of Santiago, Santiago, Chile; Faculty of Medicine (P M Lavados MD), University of Development, Santiago, Chile; Clinical Pharmacy and Pharmacy Management (B K Lawal PhD), Kaduna State University, Kaduna, Nigeria; Health Economics Division (L K D Le PhD), Monash University, Burwood, VIC, Australia; Faculty of Medicine (N Le MD), Department of General Medicine (V T Nguyen MD), Department of Internal Medicine (T H Tran MD), University of Medicine and Pharmacy at Ho Chi Minh City, Ho Chi Minh City, Vietnam (T T Le MD, T D T Le MD); Department of Cardiovascular Research (N Le MD), Methodist Hospital, Merrillville, IN, USA; Independent Consultant, Ho Chi Minh City, Vietnam (T D T Le MD); Department of Precision

Medicine (Prof S Lee MD), Sungkyunkwan University, Suwon-si, South Korea; Department of Preventive Medicine (Prof Y Lee PhD), Department of Biomedical Sciences (J Sharifi-Rad PhD), Korea University, Seoul, South Korea (Prof M Shin PhD); Department of Health Promotion and Health Education (M Li PhD), National Taiwan Normal University, Taipei, Taiwan; National Clinical Research Center for Cardiovascular Diseases (Y Li PhD), Chinese Academy of Medical Sciences, Shenzhen, China; Department of Medicine (L Lim PhD), Department of Community Oral Health and Clinical Prevention (S Selvaraj PhD), University of Malaya, Kuala Lumpur, Malaysia; Department of Medicine (D Lindholm MD), Norrtälje Hospital (Tiohundra), Norrtälje, Sweden; School of Public Health (Prof S Linn DrPH), Zefat Academic College, Haifa, Israel; Center for Evidence-Based Medicine and Clinical Research (R Liu PhD), Hubei University of Medicine, Shiyan, China; Department of Cardiology (S Liu MSc, G Qian MS), Guiqian International General Hospital, Guiyang, China; Department of Molecular Epidemiology (E Llanaj PhD), German Institute of Human Nutrition Potsdam-Rehbrücke, Potsdam, Germany; German Center for Diabetes Research (DZD), München-Neuherberg, Germany (E Llanaj PhD); Department of Internal Medicine (C Lo MD), Kirk Kerkorian School of Medicine at UNLV, Las Vegas, NV, USA; Department of Pediatric Neurology (W D Lo MD), Nationwide Children's Hospital, Columbus, OH, USA; Department of Cardiology (V Lohner PhD), University of Cologne, Cologne, Germany; One Health Research Group (J López-Gil PhD), Universidad de Las Américas, Quito, Ecuador; Department of Health Economics (L Lorenzovici MSc), Syreon Research Romania, Targu Mures, Romania; Department of Doctoral Studies (L Lorenzovici MSc), George Emil Palade University of Medicine, Pharmacy, Science, and Technology of Targu Mures, Targu Mures, Romania; Institute of Nutritional Sciences (Prof S Lorkowski PhD), Friedrich Schiller University Jena, Jena, Germany; Competence Cluster for Nutrition and Cardiovascular Health (nutriCARD), Jena, Germany (Prof S Lorkowski PhD); School of Medicine (Prof G Lucchetti PhD), Federal University of Juiz de Fora, Juiz de Fora, Brazil; Center for Evidence-Based and Translational Medicine (L Luo MPH), Department of Epidemiology and Biostatistics (Prof S Mubarik PhD, Prof C Yu PhD), Wuhan University, Wuhan, China; Centre for Public Health and Wellbeing (Z Ma PhD), University of the West of England, Bristol, UK; Department of Periodontology (Prof M Machoy PhD), Department of Propedeutics of Internal Diseases & Arterial Hypertension (Prof T Miazgowski MD), Pomeranian Medical University, Szczecin, Poland; Department of Biostatistics and Epidemiology (F Madadizadeh PhD), Yazd University of Medical Sciences, Yazd, Iran; Institute for Physical Activity and Nutrition (Prof R Maddison PhD), School of Exercise and Nutrition Sciences (N Subedi MPH), Deakin University, Melbourne, VIC, Australia; USERN Office (G Mahmoudvand MD), Lorestan University of Medical Sciences, Khorramabad, Iran; Department of Medicine (O M Makram MD), Medical College of Georgia at Augusta University, Augusta, GA, USA; Rama Medical College Hospital and Research Centre, Uttar Pradesh, India (K Malhotra MBBS); Smidt Heart Institute (Y Manla MD), Cedars-Sinai Medical Center, Los Angeles, CA, USA; Laboratory of Public Health (Prof L G Mantovani DSc), Instituto Auxologico Italiano IRCCS (Italian Auxological Institute), Milan, Italy; Biomedical Engineering Research Center (CREB) (H Marateb PhD), Universitat Politècnica de Catalunya (Barcelona Tech - UPC), Barcelona, Spain; Department of Biomedical Engineering (H Marateb PhD), University of Isfahan, Isfahan, Iran; Department of Biochemistry (A Marjani PhD), Infectious Diseases Research Center (S Mohammadi PhD), Gorgan Congenital Malformations Research Center (M Oladnabi PhD), Golestan University of Medical Sciences, Gorgan, Iran; Faculty of Public Health (Prof S Martini PhD), Universitas Airlangga (University of Airlangga), Surabaya, Indonesia; Indonesian Public Health

Association, Surabaya, Indonesia (Prof S Martini PhD); Department of Nutrition and Dietetics (M Martorell PhD), Centre for Healthy Living (M Martorell PhD), University of Concepción, Concepción, Chile; Department of Pharmacy (S Maryam PharmD), Bahauddin Zakariya University, Multan, Pakistan; Faculty of Humanities and Health Sciences (Prof R R Marzo MD), Curtin University, Sarawak, Malaysia; Jeffrey Cheah School of Medicine and Health Sciences (Prof R R Marzo MD), School of Pharmacy (S Shrestha PharmD, Y Wong PhD), Monash University, Subang Jaya, Malaysia; Department of Anatomy, Genetics and Biomedical Informatics (Y Mathangasinghe PhD), Postgraduate Institute of Medicine (S N K Navaratna MD), Department of Surgery (D P Wickramasinghe MD), University of Colombo, Colombo, Sri Lanka; Research Division (Prof P K Maulik PhD), The George Institute for Global Health, New Delhi, India; Australian Centre for Health Services Innovation (Prof S M McPhail PhD), Queensland University of Technology, Kelvin Grove, QLD, Australia; Department of Healthcare (Prof E A Mechili PhD), University of Vlora, Vlora City, Albania; Clinic of Social and Family Medicine (Prof E A Mechili PhD), Department of Medicine (Prof A Tsatsakis DSc), University of Crete, Heraklion, Greece; Department of Public Health (T Mekene Meto MPH), Arba Minch University, Arba Minch, Ethiopia; Universidad Nacional Mayor de San Marcos, Lima, Peru (W Mendoza MD); Division of Forensic Medicine (Prof R G Menezes MD), Imam Abdulrahman Bin Faisal University, Dammam, Saudi Arabia; Department of Medicine (G A Mensah MD), School of Public Health and Family Medicine (C A Nnaji PhD), Division of Cardiology (Prof M Ntsekhe PhD), University of Cape Town, Cape Town, South Africa; Department of Physiology (Prof S A Meo PhD), Pediatric Intensive Care Unit (Prof M Temsah MD), King Saud University, Riyadh, Saudi Arabia; University Centre Varazdin (T Mestrovic PhD), University North, Varazdin, Croatia; Department of Pharmacology (Prof C D K Mettananda PhD), University of Kelaniya, Ragama, Sri Lanka; Clinical Medicine Department (Prof C D K Mettananda PhD), Colombo North Teaching Hospital, Ragama, Sri Lanka; National Cancer Registry (I Michalek PhD), Pathology Department (I Michalek PhD), Maria Sklodowska-Curie National Research Institute of Oncology, Warsaw, Poland; Multidisciplinary Department of Medical-Surgical and Dental Specialties (G Minervini PhD), Univerity of Campania "Luigi Vanvitelli", Naples, Italy; Saveetha Dental College and Hospitals (G Minervini PhD, M Tovani-Palone PhD), Saveetha University, Chennai, India; Global Institute of Public Health (Prof G Mini PhD), Ananthapuri Hospitals and Research Institute, Trivandrum, India; Internal Medicine Programme (Prof E M Mirrakhimov PhD), Kyrgyz State Medical Academy, Bishkek, Kyrgyzstan; Department of Atherosclerosis and Coronary Heart Disease (Prof E M Mirrakhimov PhD), National Center of Cardiology and Internal Disease, Bishkek, Kyrgyzstan; Social Determinants of Health Center (M Mirza-Aghazadeh-Attari MD), Urmia University of Medical Sciences, Urmia, Iran (S Soraneh MD, R Valizadeh PhD); Department of Biomedical Sciences (M Mishra PhD), Mercer University School of Medicine, Macon, GA, USA; College of Health Science (A I Mohamed MSc), College of Applied and Natural Science (J Mohamed MSc), University of Hargeisa, Hargeisa, Somalia; Molecular Biology Unit (N S Mohamed MSc), Bio-Statistical and Molecular Biology Department (N S Mohamed MSc), Sirius Training and Research Centre, Khartoum, Sudan; Natural and Medical Sciences Research Center (S Mohammadi PhD, A Ullah MS), University of Nizwa, Nizwa, Oman; Department of Pharmaceutical Sciences (S Mohammed PhD), Notre Dame of Maryland University, Baltimore, MD, USA; Department of Pharmacy (S Mohammed PhD), Mizan-Tepi University, Mizan, Ethiopia; Health Systems and Policy Research Unit (Prof S Mohammed PhD), Department of Community Medicine (A A Olorukooba MD), Ahmadu Bello University, Zaria, Nigeria; Clinical Epidemiology and Public Health Research Unit (L

Monasta DSc), Burlo Garofolo Institute for Maternal and Child Health, Trieste, Italy; AI & Cyber Futures Institute (M Moni PhD), Charles Sturt University, Bathurst, NSW, Australia; Department of Ophthalmology & Vision Science (F Montazeri MD), University of California Davis, Sacramento, CA, USA; Faculty of Medicine (A Moodi Ghalibaf MD), Department of Epidemiology and Biostatistics (A Tiyuri PhD), Birjand University of Medical Sciences, Birjand, Iran; Health Sciences Research Center (M Moosazadeh PhD), Department of Medical-Surgical Nursing (S Shorofi PhD), Department of Infectious Disease (A Taheri MD), Department of Dermatology (A Taheri MD), Mazandaran University of Medical Sciences, Sari, Iran; Computer, Electrical, and Mathematical Sciences and Engineering Division (P Moraga PhD), King Abdullah University of Science and Technology, Thuwal, Saudi Arabia; International Laboratory for Air Quality and Health (Prof L Morawska PhD), Queensland University of Technology, Brisbane, QLD, Australia; Department of Public Health (Prof R S Moreira PhD), Oswaldo Cruz Foundation, Recife, Brazil; Department of Public Health (Prof R S Moreira PhD), Federal University of Pernambuco, Recife, Brazil; Department of Health Policy (Prof E Mossialos PhD), London School of Economics and Political Science, London, UK; Unit of Pharmacotherapy, Epidemiology and Economics (Prof S Mubarik PhD), University of Groningen (Rijksuniversiteit Groningen), Groningen, Netherlands; Department of Medicine (A Mulita PhD), Democritus University of Thrace, Alexandroupolis, Greece; Department of Health Economics (M Muniyandi PhD), National Institute for Research in Tuberculosis, Chennai, India; Clinical Epidemiology Research Unit (E Murillo-Zamora PhD), Mexican Institute of Social Security, Villa de Alvarez, Mexico; Postgraduate in Medical Sciences (E Murillo-Zamora PhD), Universidad de Colima, Colima, Mexico; Department of Research Methodology (S Muthu MS), Orthopaedic Research Group, Coimbatore, India; Department of Biotechnology (S Muthu MS), Karpagam Academy of Higher Education (Deemed to be University), Coimbatore, India; Department of Psychiatry (W Myung PhD), Department of Food and Nutrition (A P Okekunle PhD), Seoul National University, Seoul, South Korea; Department of Neuropsychiatry (W Myung PhD), Seoul National University Bundang Hospital, Seongnam, South Korea; Department of Knowledge Translation and Utilization (Prof A F Nabhan PhD), Egyptian Center for Evidence Based Medicine, Cairo, Egypt; Elderly Health Research Center (A Nafei PhD), Research and Academic Institution, Tehran, Iran; Research and Analytics Department (A J Nagarajan MTech), Initiative for Financing Health and Human Development, Chennai, India; Department of Research and Analytics (A J Nagarajan MTech), Bioinsilico Technologies, Chennai, India; Department of Computer Science (P Naghavi MS), University of Illinois Urbana-Champaign, Urbana, IL, USA; Department Health Services Research (G Naik MPH), Department of Health Policy & Organization (M Rahim MA), Department of Health Services Administration (M Rahim MA), Department of Psychology (D C Schwebel PhD), Department of Radiology (H Sotoudeh MD, M Tanwar MD), University of Alabama at Birmingham, Birmingham, AL, USA; Laboratory of Public Health Indicators Analysis and Health Digitalization (M Naimzada MD, S S Otstavnov PhD), Moscow Institute of Physics and Technology, Dolgoprudny, Russia; Experimental Surgery and Oncology Laboratory (M Naimzada MD), Kursk State Medical University, Kursk, Russia; Department of Pulmonary Medicine (Prof S Nair MD), Government Medical College Thrissur, Thrissur, India; Health Action by People, Trivandrum, India (Prof S Nair MD); Department of Community Medicine (T S Nair MD), MOSC Medical College, Kolenchery, India; Department of Medical Laboratory Analysis (H H Najmuldeen PhD), Cihan University Sulaymaniya, Sulaymaniyah, Iraq; Suraj Eye Institute, Nagpur, India (V Nangia MD); Mysore Medical College and Research Institute (Prof S Narasimha Swamy MD), Government Medical

College, Mysore, India; National Dental Research Institute Singapore (G G Nascimento PhD), Duke-NUS Medical School, Singapore, Singapore; Department of Circulation and Medical Imaging (J Nauman PhD), Norwegian University of Science and Technology, Trondheim, Norway; Department of Disease Control and Environmental Health (R Ndejjo MSc), Makerere University, Kampala, Uganda; Department of General Surgery (I Negoi PhD), Emergency University Hospital Bucharest, Bucharest, Romania; Department of Cardiology (R I Negoi PhD), Cardio-Aid, Bucharest, Romania; Faculty of Medicine (Prof C Nejjari PhD), Euromed University of Fes, Fez, Morocco; Faculty of Medicine (Prof C Nejjari PhD), University Sidi Mohammed Ben Abdellah, Fez, Morocco; Department of Community Medicine (S Nepal MD), Kathmandu University, Palpa, Nepal; Department of Neurosciences (Prof C R J Newton MD), Kenya Medical Research Institute/Wellcome Trust Research Programme, Kilifi, Kenya; Department of Medical Engineering (D H Nguyen BS), University of South Florida, Tampa, FL, USA; Cardiovascular laboratory (D H Nguyen MD), Methodist Hospital, Merrillville, Merrillville, IN, USA; Cardiovascular Research Department (H Q Nguyen MD), Methodist Hospital, Merrillville, IL, USA; Department of General Medicine (N N Nguyen MD), Faculty of Medicine (N N Nguyen MD), Pham Ngoc Thach University of Medicine, Ho Chi Minh City, Vietnam; Department of Surgery (P T Nguyen MD), Danang Family Hospital, Danang, Vietnam; International Islamic University Islamabad, Islamabad, Pakistan (R K Niazi PhD); Institute for Mental Health Policy Research (Y T Nigatu PhD), Centre for Addiction and Mental Health, Toronto, ON, Canada; Public Health Department (D N A Ningrum PhD), Universitas Negeri Semarang (State University of Semarang), Kota Semarang, Indonesia; Technical Department (C A Nnaji PhD), South African Medical Research Council, Cape Town, South Africa; Center for Public Health (L A Nnyanzi PhD), Teesside University, Middlesbrough, UK; Department of Health Policy and Management (S Nomura PhD), Keio University, Tokyo, Japan; Department of Statistics (S Noor MS), Shahjalal University of Science and Technology, Sylhet, Bangladesh; Department of Clinical Sciences (Prof B Norrving PhD), Lund University, Lund, Sweden; School of Medicine (Nawsherwan PhD), School of Life Sciences (M Suleman PhD), Xiamen University, Xiamen, China; Department of Paediatrics (C A Nri-Ezedi PhD), Nnamdi Azikiwe University, Awka, Nigeria; Department of Internal Medicine (Prof G Ntaios PhD), Department of Emergency Medicine (Prof I Pantazopoulos PhD), University of Thessaly, Larissa, Greece; The Cardiac Clinic (Prof M Ntsekhe PhD), Groote Schuur Hospital, Cape Town, South Africa; School of Information (F Nugen PhD), University of California Berkeley, Berkeley, CA, USA; Department of Health Sciences and Public Health (M Nurchis PhD), School of Economics (M Nurchis PhD), Università Cattolica del Sacro Cuore, Rome, Italy; Department of Public Health (D Nurrika PhD), Banten School of Health Science, South Tangerang, Indonesia; Ministry of Research, Technology and Higher Education (D Nurrika PhD), Higher Education Service Institutions (LL-DIKTI) Region IV, Bandung, Indonesia; Center of Excellence in Reproductive Health Innovation (CERHI) (C I Nzoputam MPH), University of Benin, Benin City, Nigeria; Department of Physiology (O J Nzoputam PhD), University of Benin, Edo, Nigeria; Department of Physiology (O J Nzoputam PhD), Benson Idahosa University, Benin City, Nigeria; Department of Applied Economics and Quantitative Analysis (Prof B Oancea PhD), University of Bucharest, Bucharest, Romania; Department of Medicine (M J O'Donnell PhD), National University of Ireland - Galway, Galway, Ireland; School of Health and Related Research (J O Oguta MSc), University of Sheffield, Sheffield, UK; Department of Preventive Medicine (Prof I Oh MD), Department of Pediatrics (Prof D Yon MD), Kyung Hee University, Seoul, South Korea; Department of Food Science and Postharvest Technology (L Okidi MSc), Gulu University, Gulu, Uganda; School of

Pharmacy (O C Okonji MSc), University of the Western Cape, Cape Town, South Africa; Nutriton and Toxicology (T A Olasehinde PhD), Federal institute of Industrial Research, Lagos, Nigeria; Department of Biochemistry, Genetics and Microbiology (T A Olasehinde PhD), University of KwaZulu-Natal, Westville, South Africa; Department of Nursing Science (M I Olatubi PhD), Bowen University, Iwo, Nigeria; Associação Brasileira de Cefaleia em Salvas e Enxaqueca (ABRACES), São Paulo, Brazil (A B Oliveira PhD); Cardiology Department (G M M Oliveira PhD), Federal University of Rio de Janeiro, Rio de Janeiro, Brazil; Slum and Rural Health Initiative Research Academy (I I Olufadewa MHS), Slum and Rural Health Initiative, Ibadan, Nigeria; Department of Microbiology (Y D Oluwafemi PhD), Department of Anatomy (G O Oluwatunase MSc), Department of Biosciences and Biotechnology (I T Oyeyemi PhD, A J Udoakang PhD), University of Medical Sciences, Ondo, Ondo, Nigeria; Department of Anatomy (G O Oluwatunase MSc), Olabisi Onabanjo University, Sagamu, Nigeria; Department of Pharmacology and Toxicology (Prof H A Omar PhD), Beni-Suef University, Beni-Suef, Egypt; Department of Public Health (S Ong FAMS), Ministry of Health, Bandar Seri Begawan, Brunei; Institute of Health Sciences (S Ong FAMS), Universiti Brunei Darussalam, Bandar Seri Begawan, Brunei; Department of Pharmacotherapy and Pharmaceutical Care (M Ordak PhD), Department of Biochemistry and Pharmacogenomics (M Zielińska MPharm), Medical University of Warsaw, Warsaw, Poland; Department of Biotechnological and Applied Clinical Sciences (R Ornello PhD), Department of Neurology (Prof S Sacco MD), University of L'Aquila, L'Aquila, Italy; Department of Neurology (R Ornello PhD), ASL Avezzano-Sulmona-L'Aquila, L'Aquila, Italy; Department of Medicine (Prof A Ortiz MD), Universidad Autónoma de Madrid (Autonomous University of Madrid), Madrid, Spain; Department of Nephrology and Hypertension (Prof A Ortiz MD), The Institute for Health Research Foundation Jiménez Díaz University Hospital, Madrid, Spain; One Health Global Research Group (Prof E Ortiz-Prado PhD), Universidad de las Americas (University of the Americas), Quito, Ecuador; School of Medicine (U L Osuagwu PhD), Western Sydney University, Bathurst, NSW, Australia; Department of Optometry and Vision Science (U L Osuagwu PhD), University of KwaZulu-Natal, KwaZulu-Natal, South Africa; Department of Project Management (S S Otstavnov PhD), National Research University Higher School of Economics, Moscow, Russia; Miami Cancer Institute (A Ozair MD), Baptist Health South Florida, Miami, FL, USA; Department of Respiratory Medicine (Prof M P P A DNB), Department of Oral and Maxillofacial Surgery (C S N PhD), Jagadguru Sri Shivarathreeswara University, Mysore, India; Universidad San Ignacio de Loyola, Lima, Peru (K Pacheco-Barrios MD); National School of Public Health (A Padron-Monedero PhD), Institute of Health Carlos III, Madrid, Spain; Department of Mental Health (R F Palma-Alvarez PhD), Hospital Universitari Vall d'Hebron (Vall d'Hebron University Hospital), Barcelona, Spain; Department of Psychiatry, Mental Health and Addictions (R F Palma-Alvarez PhD), Vall d'Hebron Institut de Recerca (Vall d'Hebron Research Institute), Barcelona, Spain; Amity Institute of Biotechnology (Prof D Pande Katare PhD), Amity University Uttar Pradesh, Noida, India; Research Department (A Pandey MPH), Public Health Research Society Nepal, Kathmandu, Nepal; Centre for Research and Development (S R Pandi-Perumal MSc), Chandigarh University, Punjab, India; Division of Research and Development (S R Pandi-Perumal MSc), Lovely Professional University, Phagwara, India; Department of Neurology (L D Panos MD), Department of Emergency Medicine (Prof I Pantazopoulos PhD), Department of Cardiology (T Pilgrim MD), University of Bern, Bern, Switzerland; Department of Neurology (L D Panos MD), University of Cyprus, Nicosia, Cyprus; Department of Science and Mathematics (Prof P Papadopoulou PhD), Deree-The American College of Greece, Athens,

Greece; Department of Biophysics (Prof P Papadopoulou PhD), 3rd Department of Cardiology (M Spartalis PhD), University of Athens, Athens, Greece; Vision and Eye Research Institute (Prof S Pardhan PhD), Anglia Ruskin University, Cambridge, UK; Department of Community Medicine (P P Parija MD), All India Institute of Medical Sciences, Jammu, India; Department of Epidemiology and Community Health (R R Parikh MD), University of Minnesota, Minneapolis, MN, USA; Department of Medical Sciences (R Passera PhD), University of Torino, Torino, Italy; Department of Imaging (R Passera PhD), AOU Città della Salute e della Scienza di Torino, Torino, Italy; Aristotle University of Thessaloniki, Greece, Thessaloniki, Greece (D Patoulias PhD); Faculty of Humanities and Social Sciences (U Paudel PhD), Department of Community Medicine (P M S Pradhan MD), Central Department of Public Health (N Subedi MPH), Tribhuvan University, Kathmandu, Nepal; Clinical Research Department (P Pedersini MSc), IRCCS Fondazione Don Carlo Gnocchi, Milan, Italy; Australian Institute of Health Innovation (P Peprah MSc), Macquarie University, Sydney, NSW, Australia; International Institute for Educational Planning (IIEP) (Prof M F P Peres MD), Albert Einstein Hospital, São Paulo, Brazil; Mario Negri Institute for Pharmacological Research, Bergamo, Italy (N Perico MD, Prof G Remuzzi MD); Pennsylvania Cancer and Regenerative Medicine Center (R G Pestell MD), Baruch S Blumberg Institute, Doylestown, PA, USA; Department of Medicine (R G Pestell MD), Xavier University School of Medicine, Woodbury, NY, USA; Facultad de Medicina (F E Petermann-Rocha PhD), (Universidad Diego Portales) Diego Portales University, Santiago, Chile; School of Cardiovascular and Metabolic Health (F E Petermann-Rocha PhD), University of Glasgow, Glasgow, UK; Department of Internal Medicine (H Pham MD), University of Arizona, Tucson, AZ, USA; Department of Internal Medicine (H Pham MD), Weiss Memorial Hospital, Chicago, IL, USA; Departments of Psychiatry and Epidemiology (Prof M R Phillips MD), Department of Neurology (Prof N Scarmeas PhD), Columbia University, New York, NY, USA; Research School of Chemistry and Applied Biomedical Sciences (E Plotnikov PhD), Tomsk Polytechnic University, Tomsk, Russia; Siberian State Medical University, Tomsk, Russia (E Plotnikov PhD); Clinical Academic Department of Pediatrics (Prof D Poddighe PhD), University Medical Center (UMC), Astana, Kazakhstan; Department of Data Management and Analysis (R Poluru PhD), The International Clinical Epidemiology Network (INCLEN) Trust International, New Delhi, India; Department of Internal Medicine (D S Popovic PhD), University of Novi Sad, Novi Sad, Serbia; Clinic for Endocrinology, Diabetes and Metabolic Disorders (D S Popovic PhD), Clinical Center of Vojvodina, Novi Sad, Serbia; University Medical Center Groningen (Prof M J Postma PhD), University of Groningen, Groningen, Netherlands; Non-communicable Diseases Research Center (N Pourtaheri PhD), Bam University of Medical Sciences, Bam, Iran; Humanities and Social Sciences (Prof J Pradhan PhD), National Institute of Technology Rourkela, Rourkela, India; Department of Neurophysiotherapy (V Prakash PhD), MGM Institute of Physiotherapy, Chhatrapati Sambhaginagar, India; Department of Clinical Research and Epidemiology (M Prasad MD), Institute of Liver and Biliary Sciences, New Delhi, New Delhi, India; Health Sciences Department (D R A Pribadi MSc), Muhammadiyah University of Surakarta, Sukoharjo, Indonesia; Department of Epidemiology (Y Qiao MD), Shandong University, Jinan, China; UO Neurologia, Salute Pubblica e Disabilità (A Raggi PhD), Fondazione IRCCS Istituto Neurologico Carlo Besta (The Neurology, Public Health and Disability Unit, IRCCS Foundation Carlo Besta Neurological Institute), Milan, Italy; Department of Epidemiology (P Raghuveer MD), National Institute of Mental Health and Neurosciences, Bengaluru, India; Department of Medical Laboratory Technologies (Prof F Rahim PhD), Al-Noor Center of Research and Innovation (Prof F Rahim PhD), Alnoor University, Mousl, Iraq; Institute of

Health and Wellbeing (Prof M Rahman PhD), Federation University Australia, Berwick, VIC, Australia; Future Technology Research Center (A Rahmani PhD), National Yunlin University of Science and Technology, Yunlin, Taiwan; Department of Public Health (V Rahmanian PhD), Torbat Jam Faculty of Medical Sciences, Torbat Jam, Iran; Department of Nutrition Science (S Rahmawaty PhD), Muhammadiyah University of Surakarta, Surakarta, Indonesia; Department of Pharmacology (G M Raj MD), All India Institute of Medical Sciences, Hyderabad, India; Department of Community Medicine (S Rajaa MD), Employees' State Insurance Model Hospital, Chennai, India; Centre for Chronic Disease Control, New Delhi, India (P Rajput PhD); Department of Population Health (M Ramadan DrPH), King Saud bin Abdulaziz University for Health Sciences, Jeddah, Saudi Arabia; Department of Public Health Dentistry (V Ramanarayanan MDS), Amrita Institute of Medical Sciences, Kochi, India; Department of Radiology (S Ramasamy MD), Stanford University, Stanford, CA, USA; School of Nursing & Health Sciences (S Ramazanu PhD), Hong Kong Metropolitan University, Hong Kong, China; South Asian Institute for Social Transformation (SAIST), Dhaka, Bangladesh (J Rana MPH); Department of Epidemiology, Biostatistics and Occupational Health (J Rana MPH), McGill University, Montreal, Canada; Department of Community Medicine (R K Rana MD), Shaheed Nirmal Mahto Medical College and Hospital, Dhanbad, India; Research Department (C L Ranabhat PhD), Eastern Scientific LLC, Richmond, KY, USA; Department of Health Promotion and Administration (C L Ranabhat PhD), Eastern Kentucky University, Richmond, KY, USA; Centre for Clinical Pharmacology (N Rancic PhD), University of Defence in Belgrade, Belgrade, Serbia; Centre for Clinical Pharmacology (N Rancic PhD), Medical College of Georgia at Augusta University, Belgrade, Serbia; Health Economics and Outcomes Research Department (A Rane MS), Agios Pharmaceuticals, Cambridge, MA, USA; Department of Pharmaceutical Economics and Policy (A Rane MS), Massachusetts College of Pharmacy and Health Sciences, Boston, MA, USA; Department of Medicine (Prof A Ranta PhD), University of Otago, Wellington, New Zealand; Department of Neurology (Prof A Ranta PhD), Capital & Coast District Health Board, Wellington, New Zealand; Department of Oral Pathology, Microbiology and Forensic Odontology (S Rao MDS), Sharavathi Dental College and Hospital, Shimogga, India; Thrombosis Research Group (S Rashedi MD), Brigham and Women's Hospital, Harvard Medical School, Boston, MA, USA; Department of Family Medicine (Prof D Rathish MPH), Department of Community Medicine (N D Wickramasinghe MD), Rajarata University of Sri Lanka, Anuradhapura, Sri Lanka; Academic Public Health England (Prof S Rawaf MD), Public Health England, London, UK; Department of Biological Sciences (Prof E M M Redwan PhD), King Abdulaziz University, Jeddah, Egypt; Department of Protein Research (Prof E M M Redwan PhD), Research and Academic Institution, Alexandria, Egypt; Department of Pharmacy (I Rehman PhD), Abdul wali Khan University Mardan, Mardan, Pakistan; Department of Public Health Sciences (T Rhee PhD), University of Connecticut, Farmington, CT, USA; Department of Geography and Demography (M Rodrigues PhD), University of Coimbra, Coimbra, Portugal; Department of Nursing in Women's Health (T R Rodrigues da Silva PhD), Federal University of São Paulo, São Paulo, Brazil; Department of Pharmacology and Toxicology (Prof J A B Rodriguez PhD), University of Antioquia, Medellin, Colombia; Warwick Medical School (Prof J A B Rodriguez PhD), University of Warwick, Coventry, UK; Department of Clinical Research (Prof L Roever PhD), Federal University of Uberlândia, Ribeirão Preto, Brazil; Faculty of Nursing (D S Romadlon PhD), Chulalongkorn University, Bangkok, Thailand; Department of Analytical and Applied Economics (Prof H Rout PhD, C Swain MPhil), RUSA Centre of Excellence in Public Policy and Governance (Prof H Rout PhD),

Department of Analytical & Applied Economics (P Sahoo MA), UGC Centre of Advanced Study in Psychology (M Satpathy PhD), Utkal University, Bhubaneswar, India; Faculty of Medicine (B Roy PhD), Quest International University Perak, Ipoh, Malaysia; Department of Labour (P Roy PhD), Directorate of Factories Government of West Bengal, Kolkata, India; Advanced Campus Governador Valadares (Prof G d Ruela MSc), Juiz de For a Federal University, Governador Valadares, Brazil; Nursing Department (Prof G d Ruela MSc), Universidade Presidente Antônio Carlos (President Antônio Carlos University), Governador Valadares, Brazil; Department of Cardiology (M Russo PhD), S. Maria dei Battuti Hospital, Conegliano, Italy; Department of Internal Medicine (G M Rwegerera MD), University of Botswana, Gaborone, Botswana; Cardiovascular Department (Prof A M A Saad MD), Zagazig University, Zagazig, Egypt; Department of Medical Pharmacology (Prof M M Saber-Ayad PhD), Cairo University, Giza, Egypt; Department of Medicine (C J Sabet MA), Georgetown University, Washington, DC, USA; Department of Pharmaceutical Chemistry (Prof M Saeb PhD), International Medical University, Gdańsk, Poland; Clinical and Biomedical Research Center (Prof U Saeed PhD), Foundation University Islamabad, Islamabad, Pakistan; International Center of Medical Sciences Research (ICMSR), Islamabad, Pakistan (Prof U Saeed PhD); Faculty of Medicine, Bioscience and Nursing (S Z Safi PhD), MAHSA University, Selangor, Malaysia; Interdisciplinary Research Centre in Biomedical Materials (IRCBM) (S Z Safi PhD), COMSATS Institute of Information Technology, Lahore, Pakistan; Department of Community Medicine and Family Medicine (S S Sahoo MD, M Verma MD), Department of Radiodiagnosis (P Singh MD), All India Institute of Medical Sciences, Bathinda, India; Department of Statistics (M R Sajid PhD), University of Gujrat, Gujrat, Pakistan; Department of Integrated Health Education (Prof L B Salaroli PhD), Federal University of Espirito Santo, Vitória, Brazil; Technology Management Department (Prof M Z Y Salem PhD), University College of Applied Sciences, Gaza, Palestine; School of Economics and Management (Prof M Z Y Salem PhD), University of Kassel, Kassel, Germany; Department of Global Initiatives (Prof G A Salum PhD), Child Mind Institute, New York, NY, USA; Department of Neurology (S Samadzadeh MD), Charité Universitätsmedizin Berlin (Charité Medical University Berlin), Berlin, Germany; Department of Neurology (S Samadzadeh MD), University of Southern Denmark, Odense, Denmark; Institute of Epidemiology and Preventive Medicine (Y L Samodra PhD), National Taiwan University, Taipei, Taiwan; Benang Merah Research Center (BMRC), Minahasa Utara, Indonesia (Y L Samodra PhD); Department of Anatomy (Prof V P Samuel PhD), Ras Al Khaimah Medical and Health Sciences University, Ras Al Khaimah, United Arab Emirates; Pharmacy Study Program (M A Sarasmita PharmD), Udayana University, Badung, Indonesia; Indira Gandhi Medical College and Research Institute, Puducherry, India (A Saravanan MD); Department of Public Health (Y Sarikhani PhD), Jahrom University of Medical Sciences, Jahrom, Iran; Udyam-Global Association for Sustainable Development, Bhubaneswar, India (M Satpathy PhD); Department of Preventive and Social Medicine (G Saya MD), Jawaharlal Institute of Postgraduate Medical Education and Research, Puducherry, India; National Centre for Epidemiology and Population Health (M Sayeed MS, A Talukder MSc), Australian National University, Acton, ACT, Australia; Department of Neurology (Prof N Scarmeas PhD), National and Kapodistrian University of Athens, Athens, Greece; Department of Health Sciences (I J C Schneider PhD), Federal University of Santa Catarina, Araranguá, Brazil; Department of Cardiovascular Sciences (A Schuermans BSc, J Van den Eynde BSc), Katholieke Universiteit Leuven, Leuven, Belgium; Cardiovascular Program (X Xu PhD), The George Institute for Global Health, Sydney, NSW, Australia (Prof A E Schutte PhD, Prof J Sundström PhD); Emergency Department (S

Senthilkumaran PhD), Manian Medical Centre, Erode, India; Department of Medicine and Surgery (Y Sethi MBBS), Government Doon Medical College, Dehradun, India; National Heart, Lung, and Blood Institute (A Seylani BS), National Institute of Health, Rockville, MD, USA; Department of Microbiology (P A Shah MBBS), Rajiv Gandhi University of Health Sciences, Bangalore, India; Department of Chemistry (H Shahsavari PhD), Institute for Advanced Studies in Basic Sciences (IASBS), Zanjan, Iran; Independent Consultant, Karachi, Pakistan (M A Shaikh MD); School of Medicine (M Shams-Beyranvand MSc), Alborz University of Medical Sciences, Karaj, Iran; Department for Evidence-based Medicine and Evaluation (A Sharifan PharmD), University for Continuing Education Krems, Krems, Austria; Nahavand School of Allied Medical Sciences (A Sharifi PhD), Hamadan University of Medical Sciences, Hamadan, Nahavand, Iran; Department of Hemato-oncology (A Sharma MD), Fortis Hospital, Noida, India; Department of Nephrology (S Sharma DNB), Vardhman Mahavir Medical College, New Delhi, India; K S Hegde Medical Academy (Prof M Shetty MD), Nitte University, Mangalore, India; Department of Public Health (D Shiferaw MPH), Dambi Dollo University, Dembi Dollo, Ethiopia; National Institute of Infectious Diseases, Tokyo, Japan (M Shigematsu PhD); Deparment of Pharmacology (T Shimels MSc), Saint Paul's Hospital Millennium Medical College, Addis Ababa, Ethiopia; Finnish Institute of Occupational Health, Helsinki, Finland (R Shiri PhD); Department of Veterinary Public Health and Preventive Medicine (A Shittu MSc), Usmanu Danfodiyo University, Sokoto, Sokoto, Nigeria; Department of Medicine (A O Shitu MBBS), Ladoke Akintola University, Ogbomoso, Nigeria; Department of Research (S Shrestha PharmD), Kathmandu Cancer Center, Bhaktapur, Nepal; The Cooper Institute, Dallas, TX, USA (K Shuval PhD); Department of Medical Microbiology and Infectious Diseases (E E Siddig MD), Erasmus University, Rotterdam, Netherlands; Department of Culture (M Sikdar PhD), Anthropological Survey of India, Kolkata, India; Sport Physical Activity and Health Research & Innovation Center (SPRINT) (Prof L M R Silva PhD), Polytechnic Institute of Guarda, Guarda, Portugal; CICS-UBI Health Sciences Research Center (Prof L M R Silva PhD), University of Beira Interior, Covilhã, Portugal; Department of Community Medicine (G Singh MD), Lady Hardinge Medical College, New Delhi, India; Department of Pharmacology (H Singh DM), Government Medical College and Hospital, Chandigarh, India; School of Medicine (Prof J A Singh MD), Henry JN Taub Department of Emergency Medicine (Prof L Szarpak PhD), Baylor College of Medicine, Houston, TX, USA; Medicine Service (Prof J A Singh MD), US Department of Veterans Affairs (VA), Houston, TX, USA; Department of Pediatrics (Prof K Singh DM), All India Institute of Medical Sciences Jodhpur, Jodhpur, India; Faculty of Medicine and Health Sciences (Prof N P Singh MD), Shree Guru Gobind Singh Tricentenary University, Gurugram, India; Department of Human Genetics (P Singh PhD), Punjabi University, Patiala, India; Department of Neurology (J O T Sipilä PhD), North Karelia Central Hospital, Joensuu, Finland; Clinical Branch (V Y Skryabin MD), Moscow Research and Practical Centre on Addictions, Moscow, Russia; Addiction Psychiatry Department (V Y Skryabin MD), Russian Medical Academy of Continuous Professional Education, Moscow, Russia; Department of Infectious Diseases and Epidemiology (A A Skryabina MD), Department of Internal Disease (A V Starodubova DSc), Pirogov Russian National Research Medical University, Moscow, Russia; Division of Injury Prevention (Prof D A Sleet PhD), The Bizzell Group, Atlanta, GA, USA; Department of Surgery (B Socea PhD), "Sf. Pantelimon" Emergency Clinical Hospital Bucharest, Bucharest, Romania; School of Health and Biomedical Science (A Sohag MSc), Royal Melbourne Institute of Technology (RMIT) University, Melbourne, VIC, Australia; Department of Systemic Pathology (R Solanki MD), Touro College of Osteopathic Medicine, Middletown, NY, USA; Department

of Pathology (R Solanki MD), American University of the Caribbean School of Medicine, Cupecoy, Saint Martin; Department of Biochemistry (S Solanki MD), American University of Integrative Sciences, Bridgetown, Barbados; Hull York Medical School (I N Soyiri PhD), University of Hull, Hull City, UK; Pharmacology (S Srinivasamurthy MD), RAK Medical and Health Sciences University, Ras Al Khaimah, United Arab Emirates; Nutrition and Dietetics Department (A V Starodubova DSc), Federal Research Institute of Nutrition, Biotechnology and Food Safety, Moscow, Russia; Department of Medical Sciences (Prof V Subramaniyan PhD), Sunway University, Subang Jaya, Malaysia; Mental Health Research Consultant (A Sultana MD), Independent Consultant, Khulna, Bangladesh; Division of Global Mental Health (A Sultana MD), EviSyn Health, Khulna, Bangladesh; Department of Biomedical Sciences (Z Sun PhD), Universiti Putra Malaysia, Selangor, Malaysia; Nursing Professional Education Study Program (S Susanty PhD), University Halu Oleo, Kendari, Indonesia; Department of Clinical Research and Development (Prof L Szarpak PhD), LUXMED Group, Warsaw, Poland; Department of Neurology (P Tabaee Damavandi MD), Neurocenter of Southern Switzerland (NSI), Lugano, Switzerland; Department of Medicine (Prof R Tabarés-Seisdedos PhD), University of Valencia, Valencia, Spain; Dentistry and Oral Health, Rural Clinical Sciences (J Tadakamadla PhD), La Trobe University, Bendigo, VIC, Australia; Department of Environmental, Agricultural and Occupational Health (J Taiba MPH), University of Nebraska Medical Center, Omaha, NE, USA; Sri Ramachandra Medical College and Research Institute, Chennai, India (J Taiba MPH); Statistics Discipline (A Talukder MSc), Khulna University, Khulna, Bangladesh; Department of Dermato-Venereology (M Tampa PhD), Dr. Victor Babes Clinical Hospital of Infectious Diseases and Tropical Diseases, Bucharest, Romania; Department of Epidemiology (J L Tamuzi MSc), Stellenbosch University, Cape Town, South Africa; Department of Medicine (J L Tamuzi MSc), Northlands Medical Group, Omuthiya, Namibia; State Key Laboratory of Numerical Modeling for Atmospheric Sciences and Geophysical Fluid Dynamics (LASG) (H Tang PhD), Chinese Academy of Sciences, Beijing, China; National Research and Innovation Agency, Jakarta, Indonesia (I U Tarigan PhD); Department of Economics (N Y Tat MS), Rice University, Houston, TX, USA; Department of Research and Innovation (N Y Tat MS), Enventure Medical Innovation, Houston, TX, USA; Department of Urology (M Teimoori MD), Sabzevar University of Medical Sciences, Sabzevar, Iran; Department of Epidemiology and Biostatistics (M Teramoto MD), Department of Laboratory Medicine (E A Yu PhD), Department of Bioengineering and Therapeutical Sciences (Prof M Zastrozhin PhD), University of California San Francisco, San Francisco, CA, USA; Department of Nursing (K G Gebrekidan PhD), Department of Medical Biochemistry and Molecular Biology (W B Tesfamariam MSc), Mekelle University, Mekelle, Ethiopia; School of Humanities and Social Sciences (R Thakur PhD), Indian Institute of Technology Mandi, Mandi, India; Department of Pharmacology (P Thangaraju MD), All India Institute of Medical Sciences, Raipur, India; Department of Global Health (Prof J Thomas PhD), Sustainable Policy Solutions Foundation, South Yatta, VIC, Australia; Department of Gastroenterology (N K Thomas MD), St. Luke's Hospital, Patanamthitta, India; Faculty of Biomedical Engineering (A Tichopad PhD), Czech Technical University, Prague, Czech Republic; Faculty of Public Health (J H V Ticoalu MPH), Universitas Sam Ratulangi (Sam Ratulangi University), Manado, Indonesia; Department of Medicine (Prof M Tonelli MD), University of Calgary, Calgary, AB, Canada; Institute of Public Health (R Topor-Madry PhD), Jagiellonian University Medical College, Kraków, Poland; Agency for Health Technology Assessment and Tariff System, Warsaw, Poland (R Topor-Madry PhD); High Institute of Sport and Physical Education of Sfax (K Trabelsi PhD), University of Sfax, Sfax,

Tunisia; Department of Business Analytics (T H Tran MD), University of Massachusetts Dartmouth, Dartmouth, MA, USA; Molecular Neuroscience Research Center (N Tran Minh Duc MD), Shiga University of Medical Science, Shiga, Japan; Department of Clinical and Experimental Medicine (D Trico MD), University of Pisa, Pisa, Italy; Adult Learning Disability Service (S J Tromans PhD), Leicestershire Partnership National Health Service Trust, Leicester, UK; Faculty of Medicine (T T Truyen MD), Nam Can Tho University, Can Tho, Vietnam; School of Pharmacy (D Tsai MSc), National Cheng Kung University, Tainan, Taiwan; Centre for Neonatal and Paediatric Infection (D Tsai MSc), St George's University of London, London, UK; Department of Psychiatry (E Tsermpini PhD), Dalhousie University, Halifax, NS, Canada; Department of Nutrition and Food Studies (S Tyrovolas PhD), George Mason University, Fairfax, VA, USA; Faculty of Health and Life Sciences (A Udoh PhD), University of Exeter, Exeter, UK; Department of Zoology (S Ullah PhD), Division of Science and Technology (S Ullah PhD), University of Education Lahore, Lahore, Pakistan; Medical Genomics Research Department (Prof M Umair PhD), King Abdullah International Medical Research Center, Riyadh, Saudi Arabia; Department of Cardiovascular, Endocrine-metabolic Diseases and Aging (B Unim PhD), National Institute of Health, Rome, Italy; Center for Neurodegenerative Diseases and the Aging Brain (D Urso MD), University of Bari, Tricase, Italy; Unit of Internal Medicine (M Vacante PhD), ARNAS Garibaldi Hospital, Catania, Italy; Department of Public Health and Epidemiology (O Varga PhD), University of Debrecen, Debrecen, Hungary; UKK Institute, Tampere, Finland (Prof T J Vasankari PhD); Faculty of Medicine and Health Technology (Prof T J Vasankari PhD), Tampere University, Tampere, Finland; Department of Human Genetics & Molecular Biology (B Vellingiri PhD), Bharathiar University, Coimbatore, India; Raffles Neuroscience Centre (Prof N Venketasubramanian MSc), Raffles Hospital, Singapore, Singapore; Department of Surgery (G Verras MD), General University Hospital of Patras, Patras, Greece; Department of Physiotherapy (J H Villafañe PhD), Universidad Europea de Madrid (European University of Madrid), Villaviciosa de Odón, Spain; Department of Public Health, Experimental and Forensic Medicine (Prof S Villani PhD), University of Pavia, Pavia, Italy; Department of Medical Oncology (S Volovat PhD), University of Medicine and Pharmacy "Grigore T Popa" Iasi, Iaşi, Romania; Department of Medical Oncology (S Volovat PhD), Regional Institute of Oncology, Iaşi, Romania; Department of Neurosurgery (V Volovici PhD), Erasmus University Medical Center, Rotterdam, Netherlands; Center for Experimental Microsurgery (V Volovici PhD), Iuliu Hațieganu University of Medicine and Pharmacy, Cluj-Napoca, Romania; School of Health Sciences (Prof Y Waheed PhD), National University of Sciences and Technology (NUST), Islamabad, Pakistan; Gilbert and Rose-Marie Chagoury School of Medicine (Prof Y Waheed PhD), Lebanese American University, Byblos, Lebanon; Brigham and Women's Hospital, Boston, MA, USA (C Wang PhD); Department of Surgery (C Wang PhD), Washington University in St. Louis, St. Louis, MO, USA; School of Public Health (F Wang PhD), Department of Epidemiology (D Yin DrPH), Xuzhou Medical University, Xuzhou, China; Department of Neurosurgery (S Wang MD), Capital Medical University, Beijing, China; Department of Neurosurgery (S Wang MD), Beijing Tiantan Hospital, Beijing, China; Department of Gastroenterology (S Wang PhD), Shanghai Jiao Tong University Affiliated Sixth People's Hospital, Shanghai, China; School of Nursing Sciences (M N Wanjau PhD), University of Nairobi, Nairobi, Kenya; Key Laboratory of Computer-Aided Drug Design (M Waqas PhD), Guangdong Medical University, Dongguan, China; Department of Biotechnology and Genetic Engineering (M Waqas PhD), Hazara University Mansehra, Mansehra, Pakistan; Department of Neurosurgery (Z Wei MD), Xijing Hospital, Xi'an, China; Cardiology Department (Prof R G

Weintraub MB), Royal Children's Hospital, Melbourne, VIC, Australia; Department of Critical Care and Neurosciences (Prof R G Weintraub MB), Murdoch Children's Research Institute, Parkville, VIC, Australia; Department of Medicine (Prof T Wijeratne MD), University of Rajarata, Saliyapura Anuradhapuraya, Sri Lanka; Institute of Clinical Epidemiology, Public Health, Health Economics, Medical Statistics and Informatics (Prof P Willeit PhD), Medical University Innsbruck, Innsbruck, Austria; Department of Public Health and Primary Care (Prof P Willeit PhD), University of Cambridge, Cambridge, UK; NIHR Biomedical Research Centre (Prof C D A Wolfe MD), Guy's and St. Thomas' Hospital and Kings College London, London, UK; Global Health Research Center (C Wu PhD), Duke Kunshan University, Kunshan, China; Department of Food Science and Human Nutrition (Prof F Wu PhD), Michigan State University, East Lansing, MI, USA; Clinical Psychology Department (Y Wu MD), City Health Center, Suzhou, China; Division of Gastroenterology (Prof Z Wu PhD), Huazhong University of Science and Technology, Wuhan, China; School of Public Health (H Xiao PhD), Zhejiang University, Zhejiang, China; Department of Public Health Science (H Xiao PhD), Fred Hutchinson Cancer Research Center, Seattle, WA, USA; Department of Endocrinology (Prof S Xu PhD), University of Science and Technology of China, Hefei, China; Department of Public Health (Prof K Yamagishi MD, Prof N Yonemoto PhD), Faculty of Medicine (Y Yano MD), Juntendo University, Tokyo, Japan; Department of Public Health and Health Systems (Prof H Yatsuya PhD, Prof H Yatsuya PhD), Nagoya University, Nagoya, Japan; Biostatics, Epidemiology, and Science Computing Department (S Yezli PhD), King Faisal Specialist Hospital & Research Center, Riyadh, Saudi Arabia; KHANA Center for Population Health Research, Phnom Penh, Cambodia (Prof S Yi PhD); Department of Respiratory Medicine (X Yi MD), Military Medical University, Chongqing, China; Department of Biostatistics (Prof N Yonemoto PhD), University of Toyama, Toyama, Japan; Department of Epidemiology (E A Yu PhD), Vitalant Research Institute, San Francisco, CA, USA; The First Affiliated Hospital (Prof K Yun PhD), China Medical University, Shenyang, China; Association for Socially Applicable Research (ASAR), Pune, India (S Zadey MS); Department of Emergency Medicine (S Zadey MS), Global Emergency Medicine Innovation and Implementation (GEMINI) Research Center, Durham, NC, USA; Department of Health Sciences (S Zaman MSc), James Madison University, Harrisonburg, VA, USA; Sant'Elia Hospital (A Zanghì MD), University of Catania, Caltanissetta, Italy; Research and Development Department (I Zare BSc), Sina Medical Biochemistry Technologies, Shiraz, Iran; Department of Administration (Prof M Zastrozhin PhD), PGxAI, San Francisco, CA, USA; Department of Anesthesiology (Y Zeng MD), Third Xiangya Hospital of Central South University, Changsha, China; Medical Oncology Department of Gastrointestinal Cancer (L Zhang MS), Cancer Hospital of Dalian University of Technology, Shenyang, China; School of Biomedical Engineering (L Zhang MS), Dalian University of Technology, Dalian, China; School of Public Health (Y Zhang PhD), Hubei Province Key Laboratory of Occupational Hazard Identification and Control (Y Zhang PhD), Wuhan University of Science and Technology, Wuhan, China; Tianjin Medical University General Hospital (Z Zhang MD), Tianjin Centers for Disease Control and Prevention, Tianjin, China; College of Traditional Chinese Medicine (H Zhao MD), Hebei University, Baoding, China; School of Humanities and Management (Prof S C Zhou PhD), Zhejiang Chinese Medical University, Hangzhou, China; School of Public Health and Emergency Management (B Zhu PhD), Southern University of Science and Technology, Shenzhen, China; The Institute of Cancer Research (L Zhu PhD), The Third Affiliated Hospital of Kunming Medical University, Kunming, China; Endocrinology and Metabolism Research Center (G Zoghi MD), Hormozgan University of Medical Sciences, Bandar Abbas, Iran;

Department of Clinical and Community Pharmacy (Prof S H Zyoud PhD), Clinical Research Centre (Prof S H Zyoud PhD), An-Najah National University, Nablus, Palestine.

Authors' Contributions

Providing data or critical feedback on data sources

Yohannes Habtegiorgis Abate, Samar Abd ElHafeez, Ahmed Abdelalim, Parsa Abdi, Rami Abd-Rabu, Auwal Abdullahi, Mesfin Abebe, E S Abhilash, Olugbenga Olusola Abiodun, Richard Gyan Aboagye, Mohamed Abouzid, Lucas Guimarães Abreu, Samir Abu Rumeileh, Ahmed Abualhasan, Niveen ME Abu-Rmeileh, Salahdein Aburuz, Ahmed Abu-Zaid, Juan Manuel Acuna, Denberu Eshetie Adane, Rufus Adesoji Adedoyin, Oyelola A Adegboye, Victor Adekanmbi, Qorinah Estiningtyas Sakilah Adnani, Leticia Akua Adzigbli, Aanuoluwapo Adeyimika Afolabi, Muhammad Sohail Afzal, Saira Afzal, Bright Opoku Ahinkorah, Muayyad M Ahmad, Sajjad Ahmad, Tauseef Ahmad, Ali Ahmed, Ayman Ahmed, Haroon Ahmed, Budi Aji, Salah Al Awaidy, Hanadi Al Hamad, Mohammad Al Qadire, Abid Ali, Syed Shujait Ali, Sheikh Mohammad Alif, Syed Mohamed Aljunid, Wael Almahmeed, Sabah Al-Marwani, Jaber S Alqahtani, Zaid Altaany, Awais Altaf, Alaa B Al-Tammemi, Nelson Alvis-Guzman, Mohammad Al-Wardat, Hany Aly, Alireza Amindarolzarbi, Hubert Amu, Deanna Anderlini, Dhanalakshmi Angappan, Saeid Anvari, Saleha Anwar, Sumadi Lukman Anwar, Ekenedilichukwu Emmanuel Anyabolo, Geminn Louis Carace Apostol, Jalal Arabloo, Olatunde Aremu, Johan Ärnlöv, Anton A Artamonov, Judie Arulappan, Akram M Asbeutah, Mulusew A Asemahagn, Tahira Ashraf, Haftu Asmerom Asmerom, Seyyed Shamsadin Athari, Prince Atorkey, Alok Atreya, Marcel Ausloos, Setognal Birara Aychiluhm, Amdehiwot A Aynalem, Hiva Azami, Ahmed Y Azzam, Ashish D Badiye, Atif Amin Baig, Abdulaziz T Bako, Ovidiu Constantin Baltatu, Maciej Banach, Palash Chandra Banik, Kannu Bansal, Miguel A Barboza, Mainak Bardhan, Till Winfried Bärnighausen, Hiba Jawdat Barqawi, Amadou Barrow, Mohammad-Mahdi Bastan, Sanjay Basu, Ahmet Begde, Babak Behnam, Melesse B Y Belayneh, Derrick A Bennett, Habtamu B B Beyene, Akshaya Srikanth Bhagavathula, Sonu Bhaskar, Ajay Nagesh Bhat, Vivek Bhat, Gurjit Kaur Bhatti, Jasvinder Singh Singh Bhatti, Mohiuddin Ahmed Bhuiyan, Boris Bikbov, Keralem Anteneh Bishaw, Bijit Biswas, Archith Boloor, Milad Bonakdar Hashemi, Berrak Bora Basara, Hamed Borhany, Souad Bouaoud, Abdelhakim Bouyahya, Dejana Braithwaite, Lemma N Bulto, Katrin Burkart, Florentino Luciano Caetano dos Santos, Luis Alberto Cámera, Ismael R Campos-Nonato, Andre F Carvalho, Joao Mauricio Castaldelli-Maia, Carlos A Castañeda-Orjuela, Francieli Cembranel, Joshua Chadwick, Rama Mohan Chandika, Gashaw Sisay Chanie, Vijay Kumar Chattu, Akhilanand Chaurasia, Abdulaal Chitheer, Dong-Woo Choi, Bryan Chong, Chean Lin Chong, Hitesh Chopra, Dinh-Toi Chu, Alyssa Columbus, Rosa A S Couto, Michael H Criqui, Natalia Cruz-Martins, Alanna Gomes da Silva, Xiaochen Dai, Giovanni Damiani, Lalit Dandona, Rakhi Dandona, Saswati Das, Sean DeAngelo, Aklilu Tamire Debele, Ivan Delgado-Enciso, Berecha Hundessa Demessa, Andreas K Demetriades, Hardik Dineshbhai Desai, Fikreab Desta, Vinoth Gnana Chellaiyan Devanbu, Meghnath Dhimal, Vishal R Dhulipala, Michael J Diaz, Milad Dodangeh, Klara Georgieva Dokova, Ojas Prakashbhai Doshi, Rajkumar Prakashbhai Doshi, Abdel Douiri, Robert Kokou Dowou, Haneil Larson Dsouza, Bruce B Duncan, Andre Rodrigues Duraes, Michael Ekholuenetale, Ibrahim Farahat El Bayoumy, Maysaa El Sayed Zaki, Chadi Eltaha, Adeniyi Francis Fagbamigbe, Omotayo Francis Fagbule,

Ildar Ravisovich Fakhradiyev, Luca Falzone, Jawad Fares, Andre Faro, Hossein Farrokhpour, Abidemi Omolara Fasanmi, Timur Fazylov, Alireza Feizkhah, Seyed-Mohammad Fereshtehnejad, Luisa S Flor, Roham Foroumadi, Matteo Foschi, Richard Charles Franklin, Sridevi G, Peter Andras Gaal, Yaseen Galali, Silvano Gallus, Balasankar Ganesan, Lemma Getacher, Mansour Ghafourifard, Ramy Mohamed Ghazy, Ehsan Gholami, Zainab Gholami, Sherief Ghozy, Jaleed Ahmed Gilani, Mahaveer Golechha, Pouya Goleij, Philimon N Gona, Alessandra C Goulart, Barbara Niegia Garcia Goulart, Giuseppe Grosso, Shi-Yang Guan, Avirup Guha, Snigdha Gulati, Rajat Das Gupta, Sapna Gupta, Najah R Hadi, Dariush Haghmorad, Arvin Haj-Mirzaian, Aram Halimi, Nadia M Hamdy, Zaim Anan Haq, Josep Maria Haro, Eka Mishbahatul Marah Has, Johannes Haubold, Rasmus J Havmoeller, Jeffrey J Hebert, Mehdi Hemmati, Demisu Zenbaba Heyi, Nguyen Quoc Hoan, Nobuyuki Horita, Md Mahbub Hossain, Mehdi Hosseinzadeh, Chengxi Hu, Salman Hussain, Le Duc Huy, Hong-Han Huynh, Segun Emmanuel Ibitoye, Nayu Ikeda, Olayinka Stephen Ilesanmi, Arnaud Iradukunda, Lalu Muhammad Irham, Nahlah Elkudssiah Ismail, Gaetano Isola, Vinothini J, Jalil Jaafari, Haitham Jahrami, Nityanand Jain, Ammar Abdulrahman Jairoun, Abhishek Jaiswal, Mihajlo Jakovljevic, Reza Jalilzadeh Yengejeh, Sathish Kumar Jayapal, Shubha Jayaram, Bijay Mukesh Jeswani, Zixiang Ji, Yingzhao Jin, Catherine O Johnson, Jost B Jonas, Tamas Joo, Charity Ehimwenma Joshua, Jacek Jerzy Jozwiak, Mikk Jürisson, Md Awal Kabir, Zubair Kabir, Vidya Kadashetti, Rizwan Kalani, Sanjay Kalra, Vineet Kumar Kamal, Mona Kanaan, Himal Kandel, Rami S Kantar, Neeti Kapoor, André Karch, Salah Eddin Karimi, Prabin Karki, Harkiran Kaur, Neda Kaydi, Gbenga A Kayode, Yousef Saleh Khader, Morteza Abdullatif Khafaie, Himanshu Khajuria, Nauman Khalid, M Nuruzzaman Khan, Maseer Khan, Moien AB Khan, Yusra H Khan, Khaled Khatab, Haitham Khatatbeh, Sorour Khateri, Mahalaqua Nazli Khatib, Manoj Khokhar, Atulya Aman Khosla, Majid Khosravi, Jagdish Khubchandani, Min Seo Kim, Yun Jin Kim, Adnan Kisa, Sezer Kisa, Shivakumar KM Marulasiddaiah Kondlahalli, Oleksii Korzh, Soewarta Kosen, Nikhil Kothari, Sindhura Lakshmi Koulmane Laxminarayana, Kewal Krishan, Vijay Krishnamoorthy, Barthelemy Kuate Defo, Nuworza Kugbey, Akshay Kumar, Dewesh Kumar, G Anil Kumar, Senthil Kumaran D, Om P Kurmi, Dian Kusuma, L V Simhachalam Kutikuppala, Ville Kytö, Chandrakant Lahariya, Dharmesh Kumar Lal, Savita Lasrado, Kaveh Latifinaibin, Nhi Huu Hanh Le, Thao Thi Thu Le, Trang Diep Thanh Le, Seung Won Lee, Wei-Chen Lee, Yichong Li, Lee-Ling Lim, Stephen S Lim, Gang Liu, Runben Liu, Xuefeng Liu, Erand Llanaj, José Francisco López-Gil, László Lorenzovici, Stefan Lorkowski, Paulo A Lotufo, Jay B Lusk, Zheng Feei Ma, Monika Machoy, Kashish Malhotra, Tauqeer Hussain Mallhi, Deborah Carvalho Malta, Abdullah A Mamun, Mohammad Hadi Mansouri, Pejman Mansouri, Mohammad Ali Mansournia, Lorenzo Giovanni Mantovani, Abdoljalal Marjani, Daniela Martini, Sharmeen Maryam, Roy Rillera Marzo, Awoke Masrie, Enkeleint A Mechili, Tesfahun Mekene Meto, Walter Mendoza, Ritesh G Menezes, Atte Meretoja, Tuomo J Meretoja, Irmina Maria Michalek, Le Huu Nhat Minh, Erkin M Mirrakhimov, Ameen Mosa Mohammad, Soheil Mohammadi, Abdollah Mohammadian-Hafshejani, Ibrahim Mohammadzadeh, Mustapha Mohammed, Salahuddin Mohammed, Shafiu Mohammed, Ali H Mokdad, Lorenzo Monasta, Mohammad Ali Moni, Mahmood Moosazadeh, Maryam Moradi, Yousef Moradi, Ahmed Msherghi, Sumaira Mubarik, Admir Mulita, Christopher J L Murray, Ahamarshan Jayaraman Nagarajan, Ganesh R Naik, Balakrishnan Sukumaran Nair, Sanjeev Nair, Sreenivas Narasimha Swamy, Shumaila Nargus, Bruno Ramos Nascimento, Zuhair S Natto, Nawsherwan Nawsherwan, Biswa Prakash Nayak, Rawlance Ndejjo, Ionut Negoi, Chakib Nejjari, Samata Nepal, Charles Richard James Newton, Dang H Nguyen, Hau Thi Hien Nguyen, Hien Quang Nguyen, Nhien Ngoc Y Nguyen,

Phat Tuan Nguyen, Van Thanh Nguyen, Dina Nur Anggraini Ningrum, Chukwudi A Nnaji, Lawrence Achilles Nnyanzi, Syed Toukir Ahmed Noor, Jean Jacques Noubiap, Fred Nugen, Chimezie Igwegbe Nzoputam, Ogochukwu Janet Nzoputam, Bogdan Oancea, Kehinde O Obamiro, Ismail A Odetokun, Martin James O'Donnell, James Odhiambo Oguta, In-Hwan Oh, Osaretin Christabel Okonji, Andrew T Olagunju, Tosin Abiola Olasehinde, Gláucia Maria Moraes Oliveira, Yinka Doris Doris Oluwafemi, Hany A Omar, Ahmed Omar Bali, Sok King Ong, Obinna E Onwujekwe, Alberto Ortiz, Uchechukwu Levi Osuagwu, Mayowa O Owolabi, Mahesh Padukudru P A, Kevin Pacheco-Barrios, Jagadish Rao Padubidri, Songhomitra Panda-Jonas, Anamika Pandey, Paraskevi Papadopoulou, Shahina Pardhan, Romil R Parikh, Shrikant Pawar, Prince Peprah, Mario F P Peres, Arokiasamy Perianayagam, Simone Perna, Hoang Nhat Pham, Hoang Tran Pham, Ramesh Poluru, Maarten J Postma, Naeimeh Pourtaheri, Jalandhar Pradhan, Elton Junio Sady Prates, Jagadeesh Puvvula, Pankaja Raghav Raghav, Pracheth Raghuveer, Fakher Rahim, Md Jillur Rahim, Mahban Rahimifard, Vafa Rahimi-Movaghar, Amir Masoud Rahmani, Mohammad Rahmanian, Vinoth Rajendran, Pushp Lata Rajpoot, Pradhum Ram, Shakthi Kumaran Ramasamy, Sheena Ramazanu, Juwel Rana, Kritika Rana, Rishabh Kumar Rana, Chhabi Lal Ranabhat, Nemanja Rancic, Mithun Rao, Sowmya J Rao, Sina Rashedi, Santosh Kumar Rauniyar, Ilari Rautalin, Salman Rawaf, Christian Razo, Inayat Ur Rehman, Taeho Gregory Rhee, Antonio Luiz P Ribeiro, Monica Rodrigues, Jefferson Antonio Buendia Rodriguez, Leonardo Roever, Debby Syahru Romadlon, Allen Guy Ross, Priyanka Roy, Simanta Roy, Godfrey M Rwegerera, Aly M A Saad, Korosh Saber, Cameron John Sabet, Siamak Sabour, Basema Ahmad Saddik, Umar Saeed, Sher Zaman Zaman Safi, Rajesh Sagar, Pragyan Monalisa Sahoo, Mirza Rizwan Sajid, Afeez Abolarinwa Salami, Giovanni A Salum, Sara Samadzadeh, Vijaya Paul Samuel, Abdallah M Samy, Juan Sanabria, Itamar S Santos, Milena M Santric-Milicevic, Maheswar Satpathy, Zafer Sattouf, Md Abu Sayeed, Markus P Schlaich, Maria Inês Schmidt, Art Schuermans, David C Schwebel, Siddharthan Selvaraj, Parijat Sen, Subramanian Senthilkumaran, Yashendra Sethi, Pritik A Shah, Masood Ali Shaikh, Muhammad Aaqib Shamim, Mehran Shams-Beyranvand, Anas Shamsi, Alfiya Shamsutdinova, Mohammed Shannawaz, Medha Sharath, Amin Sharifan, Javad Sharifi-Rad, Vishal Sharma, Rahim Ali Sheikhi, Adithi Shetty, Aminu Shittu, Luís Manuel Lopes Rodrigues Silva, Abhinav Singh, Baljinder Singh, Jasvinder A Singh, Narinder Pal Singh, Paramdeep Singh, Valentin Yurievich Skryabin, Anna Aleksandrovna Skryabina, Yerukneh Solomon, Reed J D Sorensen, Houman Sotoudeh, Ireneous N Soyiri, Michael Spartalis, Chandrashekhar T Sreeramareddy, Lauryn K Stafford, Benjamin A Stark, Muhammad Suleman, Johan Sundström, Chandan Kumar Swain, Lukasz Szarpak, Sree Sudha T Y, Rafael Tabarés-Seisdedos, Shima Tabatabai, Mohammad Tabish, Jabeen Taiba, Mircea Tampa, Ker-Kan Tan, Manoj Tanwar, Elvis Enowbeyang Tarkang, Pugazhenthan Thangaraju, Joe Thomas, Nikhil Kenny Kenny Thomas, Amanda G Thrift, Amir Tiyuri, Roman Topor-Madry, Marcos Roberto Tovani-Palone, Khaled Trabelsi, Ngoc Ha Tran, Nguyen Tran Minh Duc, Domenico Trico, Samuel Joseph Tromans, Aniefiok John Udoakang, Sana Ullah, Muhammad Umair, Bhaskaran Unnikrishnan, Jibrin Sammani Usman, Jef Van den Eynde, Shoban Babu Varthya, Tommi Juhani Vasankari, Balachandar Vellingiri, Narayanaswamy Venketasubramanian, Massimiliano Veroux, Georgios-Ioannis Verras, Maria Viskadourou, Simona Ruxandra Volovat, Victor Volovici, Theo Vos, Yasir Waheed, Waseem Wahood, Shu Wang, Song Wang, Yanzhong Wang, Haftom Legese Weldetinsaa, Dakshitha Praneeth Wickramasinghe, Tissa Wijeratne, Peter Willeit, Charles D A Wolfe, Yen Jun Wong, Utoomporn Wongsin, Felicia Wu, Zenghong Wu, Suowen Xu, Kazumasa Yamagishi, Yuichiro Yano, Habib Yaribeygi, Pengpeng Ye, Siyan Yi, Dong Keon Yon, Naohiro Yonemoto, Chuanhua

Yu, Burhan Abdullah Zaman, Iman Zare, Michael Zastrozhin, Beijian Zhang, Yunquan Zhang, Zhiqiang Zhang, Magdalena Zielińska, and Yossef Teshome Zikarg.

Developing methods or computational machinery

Qorinah Estiningtyas Sakilah Adnani, Saira Afzal, Ali Ahmed, Najim Z Alshahrani, Walid Adnan Al-Zyoud, Hubert Amu, Aleksandr Y Aravkin, Hiva Azami, Ahmed Y Azzam, Palash Chandra Banik, Yanping Bao, Mohammad-Mahdi Bastan, Akshaya Srikanth Bhagavathula, Sonu Bhaskar, Milad Bonakdar Hashemi, Hamed Borhany, Souad Bouaoud, Dana Bryazka, Fan Cao, Sinclair Carr, Gashaw Sisay Chanie, Haowei Chen, Dinh-Toi Chu, Rebecca M Cogen, Xiaochen Dai, Hardik Dineshbhai Desai, Robert Kokou Dowou, Michael Ekholuenetale, Maysaa El Sayed Zaki, Ayesha Fahim, Valery L Feigin, Ehsan Gholami, Sherief Ghozy, Ebisa Zerihun Gindaba, Shi-Yang Guan, Erin B Hamilton, Nasrin Hanifi, Simon I Hay, Mohammad Heidari, Mehdi Hosseinzadeh, Hong-Han Huynh, Sathish Kumar Jayapal, Catherine O Johnson, Charity Ehimwenma Joshua, Sanjay Kalra, M Nuruzzaman Khan, Sorour Khateri, Mahalaqua Nazli Khatib, Manoj Khokhar, Atulya Aman Khosla, Majid Khosravi, Adnan Kisa, Chandrakant Lahariya, Nhi Huu Hanh Le, Thao Thi Thu Le, Erand Llanaj, Elham Mahmoudi, Awoke Masrie, Le Huu Nhat Minh, Salahuddin Mohammed, Ali H Mokdad, Mohammad Ali Moni, Yousef Moradi, Admir Mulita, Christopher J L Murray, Shumaila Nargus, Bruno Ramos Nascimento, Ali Nasrollahizadeh, Amir Nasrollahizadeh, Hau Thi Hien Nguyen, Nhien Ngoc Y Nguyen, Phat Tuan Nguyen, Michal Ordak, Hoang Tran Pham, Jalandhar Pradhan, Pracheth Raghuveer, Amir Masoud Rahmani, Chhabi Lal Ranabhat, Monica Rodrigues, Gregory A Roth, Korosh Saber, Umar Saeed, Abdallah M Samy, Maheswar Satpathy, Ganesh Kumar Saya, Mehdi Sayyah, Austin E Schumacher, Yashendra Sethi, Mohammed Shannawaz, Yi Song, Reed J D Sorensen, Michael Spartalis, Lauryn K Stafford, Benjamin A Stark, Muhammad Suleman, Chandan Kumar Swain, Sree Sudha T Y, Muhammad Umair, Jorge Hugo Villafañe, Theo Vos, Haftom Legese Weldetinsaa, Dakshitha Praneeth Wickramasinghe, Amir Yarahmadi, Naohiro Yonemoto, Michael Zastrozhin, and Beijian Zhang.

Providing critical feedback on methods or results

Melsew Dagne Abate, Yohannes Habtegiorgis Abate, Samar Abd ElHafeez, Foad Abd-Allah, Ahmed Abdelalim, Atef Abdelkader, Michael Abdelmasseh, Sherief Abd-Elsalam, Parsa Abdi, Arash Abdollahi, Meriem Abdoun, Rami Abd-Rabu, Deldar Morad Abdulah, Auwal Abdullahi, Mesfin Abebe, Roberto Ariel Abeldaño Zuñiga, E S Abhilash, Olugbenga Olusola Abiodun, Rahim Abo Kasem, Richard Gyan Aboagye, Mohamed Abouzid, Lucas Guimarães Abreu, Woldu Aberhe Abrha, Dariush Abtahi, Samir Abu Rumeileh, Ahmed Abualhasan, Hasan Abualruz, Eman Abu-Gharbieh, Hana J Abukhadijah, Salahdein Aburuz, Ahmed Abu-Zaid, Juan Manuel Acuna, Denberu Eshetie Adane, Mesafint Molla Adane, Isaac Yeboah Addo, Rufus Adesoji Adedoyin, Oyelola A Adegboye, Victor Adekanmbi, Kishor Adhikari, Qorinah Estiningtyas Sakilah Adnani, Saryia Adra, Leticia Akua Adzigbli, Abdelrahman Yousry Afify, Aanuoluwapo Adeyimika Afolabi, Fatemeh Afrashteh, Muhammad Sohail Afzal, Saira Afzal, Shahin Aghamiri, Williams Agyemang-Duah, Bright Opoku Ahinkorah, Aqeel Ahmad, Muayyad M Ahmad, Sajjad Ahmad, Shahzaib Ahmad, Tauseef Ahmad, Amir Mahmoud Ahmadzade, Ali Ahmed, Ayman Ahmed, Haroon Ahmed, Syed Anees Ahmed, Budi Aji, Essona Matatom Akara, Rufus Olusola Akinyemi, Mohammed Ahmed Akkaif, Ashley E Akrami, Salah Al Awaidy, Hanadi Al Hamad, Syed Mahfuz Al Hasan, Mohammad Al Qadire, Omar Al Ta'ani, Yazan Al-Ajlouni,

Samer O Alalalmeh, Ziyad Al-Aly, Rasmieh Mustafa Al-amer, Mohammed S Aldossary, Seyedeh Yasaman Alemohammad, Bassam Al-Fatly, Adel Ali Saeed Al-Gheethi, Maryam Alharrasi, Abid Ali, Mohammed Usman Ali, Rafat Ali, Syed Shujait Ali, Waad Ali, Akram Al-Ibraheem, Sheikh Mohammad Alif, Syed Mohamed Aljunid, Wael Almahmeed, Sabah Al-Marwani, Mahmoud A Alomari, Jaber S Alqahtani, Rajaa M Mohammad Al-Raddadi, Mohammed A Alsabri, Najim Z Alshahrani, Zaid Altaany, Awais Altaf, Alaa B Al-Tammemi, Diala Altwalbeh, Nelson Alvis-Guzman, Hassan Alwafi, Mohammad Al-Wardat, Yaser Mohammed Al-Worafi, Hany Aly, Safwat Aly, Mohammad Sharif Ibrahim Alyahya, Karem H Alzoubi, Walid Adnan Al-Zyoud, Reza Amani, Prince M Amegbor, Tewodros Getnet Amera, Tarek Tawfik Amin, Alireza Amindarolzarbi, Sohrab Amiri, Hubert Amu, Dickson A Amugsi, Ganiyu Adeniyi Amusa, Robert Ancuceanu, Deanna Anderlini, Mohammed Tahir Tahir Ansari, Alireza Ansari-Moghaddam, Rockson Ansong, Saeid Anvari, Saleha Anwar, Sumadi Lukman Anwar, Ekenedilichukwu Emmanuel Anyabolo, Anayochukwu Edward Anyasodor, Geminn Louis Carace Apostol, Muhammad Aqeel, Jalal Arabloo, Mosab Arafat, Demelash Areda, Getnet Mesfin Aregu, Olatunde Aremu, Hidayat Arifin, Anton A Artamonov, Judie Arulappan, Umesh Raj Aryal, Zahra Aryan, Akram M Asbeutah, Mulusew A Asemahagn, Mulu Tiruneh Asemu, Mohammad Asghari-Jafarabadi, Mubarek Yesse Ashemo, Tahira Ashraf, Armin Aslani, Haftu Asmerom Asmerom, Thomas Astell-Burt, Seyyed Shamsadin Athari, Prince Atorkey, Maha Moh'd Wahbi Atout, Alok Atreya, Avinash Aujayeb, Marcel Ausloos, Abolfazl Avan, Hamzeh Awad, Adedapo Wasiu Awotidebe, Lemessa Assefa A Ayana, Setognal Birara Aychiluhm, Amdehiwot A Aynalem, Zewdu Bishaw Aynalem, Sina Azadnajafabad, Hiva Azami, Shahkaar Aziz, Ahmed Y Azzam, Abraham Samuel Babu, Giridhara Rathnaiah Babu, Muhammad Badar, Ashish D Badiye, Pegah Bahrami Taghanaki, Saeed Bahramian, Ruhai Bai, Atif Amin Baig, Shankar M Bakkannavar, Abdulaziz T Bako, Ovidiu Constantin Baltatu, Maciej Banach, Morteza Banakar, Soham Bandyopadhyay, Palash Chandra Banik, Kannu Bansal, Yanping Bao, Mainak Bardhan, Noel C Barengo, Till Winfried Bärnighausen, Hiba Jawdat Barqawi, Amadou Barrow, Lingkan Barua, Hameed Akande Bashiru, Mohammad-Mahdi Bastan, Sanjay Basu, Saurav Basu, Kavita Batra, Ahmet Begde, Babak Behnam, Amir Hossein Behnoush, Melesse B Y Belayneh, Umar Muhammad Bello, Derrick A Bennett, Isabela M Bensenor, Fentaw Tadese Berhe, Amiel Nazer C Bermudez, Habtamu B B Beyene, Devidas S Bhagat, Akshaya Srikanth Bhagavathula, Neeraj Bhala, Ashish Bhalla, Nikha Bhardwaj, Pankaj Bhardwaj, Sonu Bhaskar, Ajay Nagesh Bhat, Vivek Bhat, Gurjit Kaur Bhatti, Jasvinder Singh Singh Bhatti, Mohiuddin Ahmed Bhuiyan, Boris Bikbov, Cem Bilgin, Keralem Anteneh Bishaw, Bijit Biswas, Trupti Bodhare, Eyob Ketema Bogale, Archith Boloor, Milad Bonakdar Hashemi, Aime Bonny, Hamed Borhany, Samuel Adolf Bosoka, Souad Bouaoud, Abdelhakim Bouyahya, Edward J Boyko, Marija M Bozic, Dejana Braithwaite, Hermann Brenner, Gabrielle Britton, Andre R Brunoni, Dana Bryazka, Lemma N Bulto, Katrin Burkart, Yasser Bustanji, Zahid A Butt, Florentino Luciano Caetano dos Santos, Luis Alberto Cámera, Ismael R Campos-Nonato, Fan Cao, Angelo Capodici, Rosario Cárdenas, Sinclair Carr, Andre F Carvalho, Joao Mauricio Castaldelli-Maia, Carlos A Castañeda-Orjuela, Giulio Castelpietra, Alberico L Catapano, Luca Cegolon, Francieli Cembranel, Ester Cerin, Joshua Chadwick, Chiranjib Chakraborty, Rama Mohan Chandika, Eeshwar K Chandrasekar, Gashaw Sisay Chanie, Vijay Kumar Chattu, Akhilanand Chaurasia, Haowei Chen, Mingling Chen, Gerald Chi, Fatemeh Chichagi, Ritesh Chimoriya, So Mi Jemma Cho, Dong-Woo Choi, Bryan Chong, Chean Lin Chong, Hitesh Chopra, Sonali Gajanan Choudhari, Rahul Choudhary, Dinh-Toi Chu, Isaac Sunday Chukwu, Sheng-Chia Chung, Zinhle Cindi, Iolanda Cioffi, Alyssa Columbus, Simona Costanzo, Rosa A S Couto, Michael H Criqui, Natalia Cruz-Martins, Silvia

Magali Cuadra-Hernández, Alanna Gomes da Silva, Omid Dadras, Xiaochen Dai, Koustuv Dalal, Lachlan L Dalli, Giovanni Damiani, Emanuele D'Amico, Lalit Dandona, Rakhi Dandona, Amira Hamed Darwish, Saswati Das, Mohsen Dashti, Mohadese Dashtkoohi, Mohammad Dashtkoohi, Maedeh Dastmardi, Kairat Davletov, Vanessa De la Cruz-Góngora, Aklilu Tamire Debele, Shayom Debopadhaya, Ivan Delgado-Enciso, Berecha Hundessa Demessa, Andreas K Demetriades, Emina Dervišević, Hardik Dineshbhai Desai, Aragaw Tesfaw Desale, Fikreab Desta, Vinoth Gnana Chellaiyan Devanbu, Devananda Devegowda, Amol S Dhane, Meghnath Dhimal, Vishal R Dhulipala, Michael J Diaz, Mengistie Diress, Milad Dodangeh, Klara Georgieva Dokova, Deepa Dongarwar, Mario D'Oria, Ojas Prakashbhai Doshi, Rajkumar Prakashbhai Doshi, Abdel Douiri, Robert Kokou Dowou, Haneil Larson Dsouza, Viola Savy Dsouza, Arkadiusz Marian Dziedzic, Michael Ekholuenetale, Ibrahim Farahat El Bayoumy, Maysaa El Sayed Zaki, Faris El-Dahiyat, Islam Y Elgendy, Muhammed Elhadi, Waseem El-Huneidi, Mohamed A Elmonem, Adel B Elmoselhi, Chadi Eltaha, Theophilus I Emeto, Zahra Esmaeili, Francesco Esposito, Mohammad Etoom, Natalia Fabin, Ibtihal Fadhil, Adeniyi Francis Fagbamigbe, Omotayo Francis Fagbule, Ayesha Fahim, Ildar Ravisovich Fakhradiyev, Luca Falzone, Mohammad Fareed, Jawad Fares, Carla Sofia e Sá Farinha, Pawan Sirwan Faris, Andre Faro, Hossein Farrokhpour, Abidemi Omolara Fasanmi, Patrick Fazeli, Valery L Feigin, Alireza Feizkhah, Ginenus Fekadu, Xiaoqi Feng, Seyed-Mohammad Fereshtehnejad, Pietro Ferrara, Getahun Fetensa, Bikila Regassa Feyisa, Florian Fischer, Luisa S Flor, Kristen Marie Foley, Ana Catarina Fonseca, Roham Foroumadi, Matteo Foschi, Richard Charles Franklin, Ni Kadek Yuni Fridayani, Sridevi G, Peter Andras Gaal, Abhay Motiramji Gaidhane, Abduzhappar Gaipov, Yaseen Galali, Balasankar Ganesan, Danijela Gasevic, Prem Gautam, Rupesh K Gautam, Miglas Welay Gebregergis, Mesfin Gebrehiwot, Lemma Getacher, Genanew K Getahun, Molla Getie, Delaram J Ghadimi, Amir Ghaffari Jolfayi, Mansour Ghafourifard, Afsaneh Ghasemzadeh, Ramy Mohamed Ghazy, Ehsan Gholami, Zainab Gholami, Sherief Ghozy, Stefano Giannoni Luza, Jaleed Ahmed Gilani, Tiffany K Gill, Ebisa Zerihun Gindaba, Elena V Gnedovskaya, Mahaveer Golechha, Davide Golinelli, Philimon N Gona, Alessandra C Goulart, Barbara Niegia Garcia Goulart, Michal Grivna, Ashna Grover, Shi-Yang Guan, Avirup Guha, Stefano Guicciardi, Snigdha Gulati, Damitha Asanga Gunawardane, Cui Guo, Zhifeng Guo, Anish Kumar Gupta, Bhawna Gupta, Mohak Gupta, Rahul Gupta, Rajat Das Gupta, Rajeev Gupta, Sapna Gupta, Farrokh Habibzadeh, Najah R Hadi, Hamed Haghi-Aminjan, Dariush Haghmorad, Arvin Haj-Mirzaian, Aram Halimi, Nadia M Hamdy, Samer Hamidi, Asif Hanif, Nasrin Hanifi, Graeme J Hankey, Zaim Anan Haq, Arief Hargono, Netanja I Harlianto, Eka Mishbahatul Marah Has, Ahmed I Hasaballah, Ikramul Hasan, Md Saquib Hasnain, Ikrama Hassan, Mahgol Sadat Hassan Zadeh Tabatabaei, Johannes Haubold, Rasmus J Havmoeller, Youssef Hbid, Jeffrey J Hebert, Mohammad Heidari, Mehdi Hemmati, Demisu Zenbaba Heyi, Kamal Hezam, Yuta Hiraike, Nguyen Quoc Hoan, Ramesh Holla, Nobuyuki Horita, Md Mahbub Hossain, Hassan Hosseinzadeh, Mehdi Hosseinzadeh, Mihaela Hostiuc, Chengxi Hu, Ayesha Humayun, Salman Hussain, Le Duc Huy, Hong-Han Huynh, Bing-Fang Hwang, Segun Emmanuel Ibitoye, Olayinka Stephen Ilesanmi, Irena M Ilic, Milena D Ilic, Mohammad Tarique Imam, Mustapha Immurana, Leeberk Raja Inbaraj, Muhammad Iqhrammullah, Lalu Muhammad Irham, Md Rabiul Islam, Faisal Ismail, Nahlah Elkudssiah Ismail, Gaetano Isola, Ramaiah Itumalla, Masao Iwagami, Chidozie Declan CD Iwu, Vinothini J, Jalil Jaafari, Louis Jacob, Haitham Jahrami, Akhil Jain, Nityanand Jain, Ammar Abdulrahman Jairoun, Abhishek Jaiswal, Mihajlo Jakovljevic, Balamurugan Janakiraman, Abubakar Ibrahim Jatau, Sathish Kumar Jayapal, Shubha Jayaram, Sun Ha Jee, Jayakumar Jeganathan, Mihretu Jegnie, Alelign Tasew Jema, Bijay Mukesh

Jeswani, Angeline Jeyakumar, Anil K Jha, Ravi Prakash Jha, Zixiang Ji, Heng Jiang, Shuai Jin, Yingzhao Jin, Catherine O Johnson, Mohammad Jokar, Jost B Jonas, Tamas Joo, Jobinse Jose, Nitin Joseph, Charity Ehimwenma Joshua, Farahnaz Joukar, Jacek Jerzy Jozwiak, Mikk Jürisson, Ali Kabir, Md Awal Kabir, Zubair Kabir, Vidya Kadashetti, Rizwan Kalani, Sanjay Kalra, Vineet Kumar Kamal, Haidong Kan, Mona Kanaan, Himal Kandel, Kehinde Kazeem Kanmodi, Rami S Kantar, Paschalis Karakasis, Ibraheem M Karaye, André Karch, Hanie Karimi, Yeganeh Karimi, Arman Karimi Behnagh, Prabin Karki, Joonas H Kauppila, Harkiran Kaur, Neda Kaydi, Gbenga A Kayode, Foad Kazemi, Sina Kazemian, Yousef Saleh Khader, Morteza Abdullatif Khafaie, Inn Kynn Khaing, Himanshu Khajuria, Amirmohammad Khalaji, Nauman Khalid, Anees Ahmed Khalil, Asaduzzaman Khan, Fayaz Khan, M Nuruzzaman Khan, Maseer Khan, Mohammad Jobair Khan, Moien AB Khan, Yusra H Khan, Shaghayegh Khanmohammadi, Khaled Khatab, Haitham Khatatbeh, Moawiah Mohammad Khatatbeh, Sorour Khateri, Mahalaqua Nazli Khatib, Maryam Khayamzadeh, Feriha Fatima Khidri, Manoj Khokhar, Atulya Aman Khosla, Majid Khosravi, Jagdish Khubchandani, Saeid Kian, Kwanghyun Kim, Min Seo Kim, Yun Jin Kim, Ruth W Kimokoti, Adnan Kisa, Sezer Kisa, Ali-Asghar Kolahi, Kamrun Nahar Koly, Farzad Kompani, Shivakumar KM Marulasiddaiah Kondlahalli, Miikka Korja, Vladimir Andreevich Korshunov, Oleksii Korzh, Karel Kostev, Nikhil Kothari, Sindhura Lakshmi Koulmane Laxminarayana, Kewal Krishan, Varun Krishna, Vijay Krishnamoorthy, Bindu Krishnan, Barthelemy Kuate Defo, Burcu Kucuk Bicer, Md Abdul Kuddus, Mohammed Kuddus, Nuworza Kugbey, Mukhtar Kulimbet, Vishnutheertha Kulkarni, Akshay Kumar, Ashish Kumar, Dewesh Kumar, G Anil Kumar, Nithin Kumar, Senthil Kumaran D, Amartya Kundu, Satyajit Kundu, Setor K Kunutsor, Om P Kurmi, Dian Kusuma, L V Simhachalam Kutikuppala, Ambily Kuttikkattu, Ville Kytö, Carlo La Vecchia, Chandrakant Lahariya, Dharmesh Kumar Lal, Tea Lallukka, Judit Lám, Iván Landires, Anders O Larsson, Savita Lasrado, Kaveh Latifinaibin, Paolo Lauriola, Pablo M Lavados, Basira Kankia Lawal, Long Khanh Dao Le, Nhi Huu Hanh Le, Thao Thi Thu Le, Trang Diep Thanh Le, Seung Won Lee, Wei-Chen Lee, Yo Han Lee, Ming-Chieh Li, Wei Li, Xiaopan Li, Lee-Ling Lim, Stephen S Lim, John C Lin, Daniel Lindholm, Shai Linn, Gang Liu, Shuke Liu, Xiaofeng Liu, Xuefeng Liu, Erand Llanaj, Chun-Han Lo, Warren David Lo, José Francisco López-Gil, László Lorenzovici, Stefan Lorkowski, Giancarlo Lucchetti, Lisha Luo, Jay B Lusk, Zheng Feei Ma, Monika Machoy, Farzan Madadizadeh, Ralph Maddison, Elham Mahmoudi, Golnaz Mahmoudvand, Omar M Makram, Elaheh Malakan Rad, Kashish Malhotra, Ahmad Azam Malik, Iram Malik, Tauqeer Hussain Mallhi, Deborah Carvalho Malta, Abdullah A Mamun, Yosef Manla, Vahid Mansouri, Mohammad Ali Mansournia, Emmanuel Manu, Hamid Reza Marateb, Abdoljalal Marjani, Daniela Martini, Santi Martini, Miquel Martorell, Sharmeen Maryam, Roy Rillera Marzo, Awoke Masrie, Yasith Mathangasinghe, Pallab K Maulik, Mahsa Mayeli, Mohsen Mazidi, Martin McKee, Steven M McPhail, Enkeleint A Mechili, Asim Mehmood, Kamran Mehrabani-Zeinabad, Tesfahun Mekene Meto, Hadush Negash Meles, Walter Mendoza, Ritesh G Menezes, George A Mensah, Sultan Ayoub Meo, Atte Meretoja, Tomislav Mestrovic, Chamila Dinushi Kukulege Mettananda, Tomasz Miazgowski, Irmina Maria Michalek, Ana Carolina Micheletti Gomide Nogueira de Sá, Le Huu Nhat Minh, GK Mini, Andreea Mirica, Erkin M Mirrakhimov, Mohammad Mirza-Aghazadeh-Attari, Manish Mishra, Sanjeev Misra, Prasanna Mithra, Ahmed Ismail Mohamed, Jama Mohamed, Nouh Saad Mohamed, Ameen Mosa Mohammad, Esmaeil Mohammadi, Saeed Mohammadi, Soheil Mohammadi, Abdollah Mohammadian-Hafshejani, Ibrahim Mohammadzadeh, Mustapha Mohammed, Salahuddin Mohammed, Shafiu Mohammed, Ali H Mokdad, Hossein Molavi Vardanjani, Mariam Molokhia, Shaher Momani, Mohammad Ali Moni, Fateme Montazeri,

AmirAli Moodi Ghalibaf, Mahmood Moosazadeh, Maryam Moradi, Yousef Moradi, Paula Moraga, Lidia Morawska, Rafael Silveira Moreira, Shane Douglas Morrison, Elias Mossialos, Ahmed Msherghi, Sumaira Mubarik, Admir Mulita, Malaisamy Muniyandi, Kavita Munjal, Efren Murillo-Zamora, Christopher J L Murray, Sathish Muthu, Woojae Myung, Ayoub Nafei, Ahamarshan Jayaraman Nagarajan, Pirouz Naghavi, Ganesh R Naik, Gurudatta Naik, Mukhammad David Naimzada, Sanjeev Nair, Tapas Sadasivan Nair, Soroush Najdaghi, Hastyar Hama Rashid Najmuldeen, Noureddin Nakhostin Ansari, Vinay Nangia, Sreenivas Narasimha Swamy, Shumaila Nargus, Delaram Narimani Davani, Bruno Ramos Nascimento, Gustavo G Nascimento, Ali Nasrollahizadeh, Amir Nasrollahizadeh, Zuhair S Natto, Javaid Nauman, Samidi Nirasha Kumari Navaratna, Nawsherwan Nawsherwan, Biswa Prakash Nayak, Vinod C Nayak, Athare Nazri-Panjaki, Rawlance Ndejjo, Ionut Negoi, Ruxandra Irina Negoi, Seyed Aria Nejadghaderi, Chakib Nejjari, Samata Nepal, Charles Richard James Newton, Dang H Nguyen, Duc Hoang Nguyen, Hau Thi Hien Nguyen, Hien Quang Nguyen, Nhien Ngoc Y Nguyen, Phat Tuan Nguyen, Van Thanh Nguyen, Yeshambel T Nigatu, Nasrin Nikravangolsefid, Dina Nur Anggraini Ningrum, Chukwudi A Nnaji, Lawrence Achilles Nnyanzi, Shuhei Nomura, Syed Toukir Ahmed Noor, Jean Jacques Noubiap, Chisom Adaobi Nri-Ezedi, George Ntaios, Fred Nugen, Mario Cesare Nurchis, Dieta Nurrika, Chimezie Igwegbe Nzoputam, Ogochukwu Janet Nzoputam, Bogdan Oancea, Kehinde O Obamiro, Ismail A Odetokun, Martin James O'Donnell, James Odhiambo Oguta, Tolulope R Ojo-Akosile, Hassan Okati-Aliabad, Akinkunmi Paul Okekunle, Lawrence Okidi, Osaretin Christabel Okonji, Morteza Oladnabi, Andrew T Olagunju, Muideen Tunbosun Olaiya, Oladotun Victor Olalusi, Tosin Abiola Olasehinde, Omotola O Olasupo, Matthew Idowu Olatubi, Arão Belitardo Oliveira, Abdulhakeem Abayomi Olorukooba, Isaac Iyinoluwa Olufadewa, Yinka Doris Doris Oluwafemi, Gideon Olamilekan Oluwatunase, Hany A Omar, Ahmed Omar Bali, Adrienne E O'Neil, Obinna E Onwujekwe, Abdulahi Opejin Opejin, Michal Ordak, Wael M S Osman, Uchechukwu Levi Osuagwu, Stanislav S Otstavnov, Mayowa O Owolabi, Ahmad Ozair, Mahesh Padukudru P A, Kevin Pacheco-Barrios, Jagadish Rao Padubidri, Tamás Palicz, Feng Pan, Songhomitra Panda-Jonas, Deepshikha Pande Katare, Anamika Pandey, Seithikurippu R Pandi-Perumal, Leonidas D Panos, Ioannis Pantazopoulos, Paraskevi Papadopoulou, Shahina Pardhan, Pragyan Paramita Parija, Romil R Parikh, Nicholas Parsons, Roberto Passera, Dimitrios Patoulias, Shrikant Pawar, Amy E Peden, Paolo Pedersini, Prince Peprah, Maria Odete Pereira, Arokiasamy Perianayagam, Simone Perna, Richard G Pestell, Ionela-Roxana Petcu, Fanny Emily Petermann-Rocha, Hoang Nhat Pham, Hoang Tran Pham, Michael R Phillips, Michael A Piradov, Saeed Pirouzpanah, Evgenii Plotnikov, Dimitri Poddighe, Ramesh Poluru, Maarten J Postma, Akram Pourshams, Jalandhar Pradhan, Pranil Man Singh Pradhan, V Prakash, Manya Prasad, Elton Junio Sady Prates, Dimas Ria Angga Pribadi, Jagadeesh Puvvula, Ibrahim Qattea, Gangzhen Qian, Alberto Raggi, Pankaja Raghav Raghav, Pracheth Raghuveer, Fakher Rahim, Md Jillur Rahim, Mahban Rahimifard, Vafa Rahimi-Movaghar, Md Mosfequr Rahman, Mohammad Hifz Ur Rahman, Mosiur Rahman, Muhammad Aziz Rahman, Amir Masoud Rahmani, Mohammad Rahmanian, Nazanin Rahmanian, Vahid Rahmanian, Rahem Rahmati, Setyaningrum Rahmawaty, Gerard Marshall Raj, Sathish Rajaa, Vinoth Rajendran, Pushp Lata Rajpoot, Prashant Rajput, Pradhum Ram, Mahmoud Mohammed Ramadan, Majed Ramadan, Venkitachalam Ramanarayanan, Shakthi Kumaran Ramasamy, Juwel Rana, Kritika Rana, Rishabh Kumar Rana, Chhabi Lal Ranabhat, Nemanja Rancic, Amey Rane, Annemarei Ranta, Mithun Rao, Sowmya J Rao, Sina Rashedi, Mohammad-Mahdi Rashidi, Ashkan Rasouli-Saravani, Devarajan Rathish, Santosh Kumar Rauniyar, Ilari Rautalin, Salman Rawaf, Christian

Razo, Murali Mohan Rama Krishna Reddy, Elrashdy Moustafa Mohamed Redwan, Inayat Ur Rehman, Nazila Rezaei, Mohsen Rezaeian, Taeho Gregory Rhee, Antonio Luiz P Ribeiro, Monica Rodrigues, Thales Philipe R Rodrigues da Silva, Jefferson Antonio Buendia Rodriguez, Leonardo Roever, Debby Syahru Romadlon, Allen Guy Ross, Gregory A Roth, Himanshu Sekhar Rout, Priyanka Roy, Simanta Roy, Michele Russo, Godfrey M Rwegerera, Chandan S N, Aly M A Saad, Korosh Saber, Maha Mohamed Saber-Ayad, Cameron John Sabet, Siamak Sabour, Simona Sacco, Basema Ahmad Saddik, Erfan Sadeghi, Mohammad Reza Saeb, Umar Saeed, Sher Zaman Zaman Safi, Rajesh Sagar, Fatemeh Saheb Sharif-Askari, Pragyan Monalisa Sahoo, Soumya Swaroop Sahoo, Mirza Rizwan Sajid, Afeez Abolarinwa Salami, Mohamed A Saleh, Sara Samadzadeh, Saad Samargandy, Yoseph Leonardo Samodra, Vijaya Paul Samuel, Abdallah M Samy, Juan Sanabria, Milena M Santric-Milicevic, Made Ary Sarasmita, Yaser Sarikhani, Gargi Sachin Sarode, Sachin C Sarode, Maheswar Satpathy, Zafer Sattouf, Ganesh Kumar Saya, Md Abu Sayeed, Mehdi Sayyah, Nikolaos Scarmeas, Markus P Schlaich, Ione Jayce Ceola Schneider, Art Schuermans, Aletta Elisabeth Schutte, David C Schwebel, Parijat Sen, Sabyasachi Senapati, Subramanian Senthilkumaran, Mihretu Tagesse Sergindo, Yashendra Sethi, Mahan Shafie, Pritik A Shah, Saeed Shahabi, Ataollah Shahbandi, Samiah Shahid, Hamid R Shahsavari, Masood Ali Shaikh, Ali S Shalash, Muhammad Aaqib Shamim, Mehran Shams-Beyranvand, Anas Shamsi, Mohd Shanawaz, Mohammed Shannawaz, Medha Sharath, Amin Sharifan, Azam Sharifi, Javad Sharifi-Rad, Anupam Sharma, Sourabh Sharma, Ujjawal Sharma, Vishal Sharma, Desalegn Shiferaw, Mika Shigematsu, Tariku Shimels, Min-Jeong Shin, Rahman Shiri, Aminu Shittu, Ivy Shiue, Seyed Afshin Shorofi, Sunil Shrestha, Kerem Shuval, Yafei Si, Emmanuel Edwar Siddig, Mithun Sikdar, Luís Manuel Lopes Rodrigues Silva, Abhinav Singh, Baljinder Singh, Garima Singh, Harmanjit Singh, Jasvinder A Singh, Narinder Pal Singh, Paramdeep Singh, Puneetpal Singh, Shravan Sivakumar, Valentin Yurievich Skryabin, Anna Aleksandrovna Skryabina, David A Sleet, Abdullah Al Mamun Sohag, Yerukneh Solomon, Yi Song, Soroush Soraneh, Reed J D Sorensen, Houman Sotoudeh, Ireneous N Soyiri, Michael Spartalis, Chandrashekhar T Sreeramareddy, Suresh Kumar Srinivasamurthy, Lauryn K Stafford, Benjamin A Stark, Antonina V Starodubova, Narayan Subedi, Vetriselvan Subramaniyan, Muhammad Suleman, Abida Sultana, Johan Sundström, Vinay Suresh, Chandan Kumar Swain, Lukasz Szarpak, Sree Sudha T Y, Payam Tabaee Damavandi, Rafael Tabarés-Seisdedos, Seyyed Mohammad Tabatabaei, Shima Tabatabai, Celine Tabche, Mohammad Tabish, Jyothi Tadakamadla, Santosh Kumar Tadakamadla, Jabeen Taiba, Iman M Talaat, Ashis Talukder, Mircea Tampa, Jacques Lukenze Tamuzi, Ker-Kan Tan, Haosu Tang, Manoj Tanwar, Ingan Ukur Tarigan, Elvis Enowbeyang Tarkang, Seyed Mohammad Tavangar, Mojtaba Teimoori, Mohamad-Hani Temsah, Reem Mohamad Hani Temsah, Masayuki Teramoto, Wegen Beyene Tesfamariam, Edosa Geta Tesfaye Gta, Ramna Thakur, Pugazhenthan Thangaraju, Rajshree Thapa, Rekha Thapar, Rasiah Thayakaran, Sathish Thirunavukkarasu, Joe Thomas, Nikhil Kenny Kenny Thomas, Amanda G Thrift, Jing Tian, Ales Tichopad, Jansje Henny Vera Ticoalu, Chalachew Tiruneh, Krishna Tiwari, Amir Tiyuri, Marcello Tonelli, Roman Topor-Madry, Marcos Roberto Tovani-Palone, Khaled Trabelsi, Ngoc Ha Tran, Thang Huu Tran, Nguyen Tran Minh Duc, Domenico Trico, Samuel Joseph Tromans, Daniel Hsiang-Te Tsai, Ermias A A Turuse, Stefanos Tyrovolas, Arit Udoh, Atta Ullah, Sana Ullah, Muhammad Umair, Muhammad Umar, Bhaskaran Unnikrishnan, Jibrin Sammani Usman, Seyed Mohammad Vahabi, Sanaz Vahdati, Omid Vakili, Rohollah Valizadeh, Jef Van den Eynde, Orsolya Varga, Shoban Babu Varthya, Balachandar Vellingiri, Narayanaswamy Venketasubramanian, Madhur Verma, Massimiliano Veroux, Georgios-Ioannis Verras,

Dominique Vervoort, Jorge Hugo Villafañe, Simona Villani, Manish Vinayak, Maria Viskadourou, Simona Ruxandra Volovat, Victor Volovici, Theo Vos, Hatem A Wafa, Yasir Waheed, Waseem Wahood, Cong Wang, Fang Wang, Shu Wang, Song Wang, Yanzhong Wang, Yuan-Pang Wang, Mary Njeri Wanjau, Muhammad Waqas, Emebet Gashaw Wassie, Gizachew Tadesse Wassie, Zihan Wei, Haftom Legese Weldetinsaa, Dakshitha Praneeth Wickramasinghe, Nuwan Darshana Wickramasinghe, Tissa Wijeratne, Peter Willeit, Charles D A Wolfe, Yen Jun Wong, Utoomporn Wongsin, Chenkai Wu, Felicia Wu, Zenghong Wu, Hong Xiao, Suowen Xu, Xiaoyue Xu, Danting Yang, Amir Yarahmadi, Habib Yaribeygi, Yuichi Yasufuku, Hiroshi Yatsuya, Fereshteh Yazdanpanah, Mohammad Hosein Yazdanpanah, Pengpeng Ye, Renjulal Yesodharan, Saber Yezli, Siyan Yi, Xinglin Yi, Dehui Yin, Dong Keon Yon, Naohiro Yonemoto, Chuanhua Yu, Elaine A Yu, Ke Yun, Hadiza Yusuf, Siddhesh Zadey, Nima Zafari, Burhan Abdullah Zaman, Sojib Bin Zaman, Aurora Zanghì, Fatemeh Zarimeidani, Armin Zarrintan, Michael Zastrozhin, Dawit Zemedikun, Youjie Zeng, Beijian Zhang, Haijun Zhang, Liqun Zhang, Zhiqiang Zhang, Hanqing Zhao, Claire Chenwen Zhong, Shang Cheng Zhou, Bin Zhu, Lei Zhu, Abzal Zhumagaliuly, Magdalena Zielińska, Yossef Teshome Zikarg, Ghazal Zoghi, Sa'ed H Zyoud, and Samer H Zyoud.

Drafting the work or revising it critically for important intellectual content

Melsew Dagne Abate, Yohannes Habtegiorgis Abate, Samar Abd ElHafeez, Foad Abd-Allah, Ahmed Abdelalim, Atef Abdelkader, Michael Abdelmasseh, Sherief Abd-Elsalam, Parsa Abdi, Arash Abdollahi, Rami Abd-Rabu, Auwal Abdullahi, Mesfin Abebe, Roberto Ariel Abeldaño Zuñiga, Olugbenga Olusola Abiodun, Olumide Abiodun, Rahim Abo Kasem, Mohamed Abouzid, Lucas Guimarães Abreu, Samir Abu Rumeileh, Ahmed Abualhasan, Hasan Abualruz, Eman Abu-Gharbieh, Hana J Abukhadijah, Niveen ME Abu-Rmeileh, Salahdein Aburuz, Ahmed Abu-Zaid, Juan Manuel Acuna, Denberu Eshetie Adane, Mesafint Molla Adane, Isaac Yeboah Addo, Oyelola A Adegboye, Victor Adekanmbi, Kishor Adhikari, Qorinah Estiningtyas Sakilah Adnani, Saryia Adra, Abdelrahman Yousry Afify, Aanuoluwapo Adeyimika Afolabi, Muhammad Sohail Afzal, Saira Afzal, Bright Opoku Ahinkorah, Ayman Ahmed, Haroon Ahmed, Syed Anees Ahmed, Marjan Ajami, Essona Matatom Akara, Rufus Olusola Akinyemi, Mohammed Ahmed Akkaif, Ashley E Akrami, Salah Al Awaidy, Omar Al Ta'ani, Yazan Al-Ajlouni, Samer O Alalalmeh, Tariq A Alalwan, Rasmieh Mustafa Al-amer, Wafa A Aldhaleei, Mohammed S Aldossary, Bassam Al-Fatly, Fadwa Naji Alhalaiqa, Abid Ali, Mohammed Usman Ali, Rafat Ali, Syed Shujait Ali, Waad Ali, Akram Al-Ibraheem, Wael Almahmeed, Mahmoud A Alomari, Jordi Alonso, Jaber S Alqahtani, Ahmad Alrawashdeh, Najim Z Alshahrani, Awais Altaf, Alaa B Al-Tammemi, Nelson Alvis-Guzman, Hassan Alwafi, Mohammad Al-Wardat, Yaser Mohammed Al-Worafi, Hany Aly, Safwat Aly, Mohammad Sharif Ibrahim Alyahya, Karem H Alzoubi, Walid Adnan Al-Zyoud, Reza Amani, Tarek Tawfik Amin, Alireza Amindarolzarbi, Sohrab Amiri, Hubert Amu, Dickson A Amugsi, Ganiyu Adeniyi Amusa, Robert Ancuceanu, Deanna Anderlini, Dhanalakshmi Angappan, Abhishek Anil, Saeid Anvari, Saleha Anwar, Sumadi Lukman Anwar, Anayochukwu Edward Anyasodor, Geminn Louis Carace Apostol, Francis Appiah, Jalal Arabloo, Razman Arabzadeh Bahri, Mosab Arafat, Ali Ardekani, Brhane Berhe Aregawi, Olatunde Aremu, Hidayat Arifin, Johan Ärnlöv, Judie Arulappan, Umesh Raj Aryal, Zahra Aryan, Akram M Asbeutah, Mulusew A Asemahagn, Mulu Tiruneh Asemu, Haftu Asmerom Asmerom, Seyyed Shamsadin Athari, Prince Atorkey, Maha Moh'd Wahbi Atout,

Alok Atreya, Avinash Aujayeb, Marcel Ausloos, Abolfazl Avan, Adedapo Wasiu Awotidebe, Zewdu Bishaw Aynalem, Sina Azadnajafabad, Hiva Azami, Ahmed Y Azzam, Abraham Samuel Babu, Giridhara Rathnaiah Babu, Muhammad Badar, Ashish D Badiye, Ruhai Bai, Atif Amin Baig, Abdulaziz T Bako, Ovidiu Constantin Baltatu, Kiran Bam, Maciej Banach, Morteza Banakar, Soham Bandyopadhyay, Kannu Bansal, Yanping Bao, Miguel A Barboza, Mainak Bardhan, Noel C Barengo, Suzanne Lyn Barker-Collo, Till Winfried Bärnighausen, Hiba Jawdat Barqawi, Amadou Barrow, Azadeh Bashiri, Afisu Basiru, Mohammad-Mahdi Bastan, Sanjay Basu, Ahmet Begde, Amir Hossein Behnoush, Melesse B Y Belayneh, Michael Belingheri, Umar Muhammad Bello, Isabela M Bensenor, Fentaw Tadese Berhe, Habtamu B B Beyene, Kebede A Beyene, Akshaya Srikanth Bhagavathula, Neeraj Bhala, Ashish Bhalla, Sonu Bhaskar, Ajay Nagesh Bhat, Vivek Bhat, Anjali Bhati, Gurjit Kaur Bhatti, Jasvinder Singh Singh Bhatti, Mohiuddin Ahmed Bhuiyan, Subarna Bhusal, Boris Bikbov, Cem Bilgin, Antonio Biondi, Catherine Bisignano, Atanu Biswas, Bijit Biswas, Milad Bonakdar Hashemi, Hamed Borhany, Souad Bouaoud, Abdelhakim Bouyahya, Edward J Boyko, Dejana Braithwaite, Susanne Breitner, Hermann Brenner, Gabrielle Britton, Andre R Brunoni, Raffaele Bugiardini, Yasser Bustanji, Florentino Luciano Caetano dos Santos, Luciana Aparecida Campos, Angelo Capodici, Sinclair Carr, Giulia Carreras, Andre F Carvalho, Felix Carvalho, Joao Mauricio Castaldelli-Maia, Carlos A Castañeda-Orjuela, Giulio Castelpietra, Alberico L Catapano, Maria Sofia Cattaruzza, Luca Cegolon, Francieli Cembranel, Edina Cenko, Ester Cerin, Joshua Chadwick, Chiranjib Chakraborty, Sandip Chakraborty, Jeffrey Shi Kai Chan, Rama Mohan Chandika, Eeshwar K Chandrasekar, Vijay Kumar Chattu, Anis Ahmad Chaudhary, Akhilanand Chaurasia, Haowei Chen, Simiao Chen, Fatemeh Chichagi, Ritesh Chimoriya, Patrick R Ching, Dong-Woo Choi, Bryan Chong, Rahul Choudhary, Dinh-Toi Chu, Iolanda Cioffi, Alyssa Columbus, Simona Costanzo, Rosa A S Couto, Michael H Criqui, Natalia Cruz-Martins, Silvia Magali Cuadra-Hernández, Alanna Gomes da Silva, Sriharsha Dadana, Koustuv Dalal, Giovanni Damiani, Emanuele D'Amico, Amira Hamed Darwish, Mohsen Dashti, Mohadese Dashtkoohi, Maedeh Dastmardi, Sean DeAngelo, Aklilu Tamire Debele, Ivan Delgado-Enciso, Berecha Hundessa Demessa, Andreas K Demetriades, Edgar Denova-Gutiérrez, Hardik Dineshbhai Desai, Devananda Devegowda, Syed Masudur Rahman Dewan, Amol S Dhane, Meghnath Dhimal, Vishal R Dhulipala, Michael J Diaz, Milad Dodangeh, Phidelia Theresa Doegah, Sushil Dohare, Mohamed Fahmy Doheim, Deepa Dongarwar, Mario D'Oria, Ojas Prakashbhai Doshi, Rajkumar Prakashbhai Doshi, Abdel Douiri, Robert Kokou Dowou, Ashel Chelsea Dsouza, Haneil Larson Dsouza, Bruce B Duncan, Arkadiusz Marian Dziedzic, Michael Ekholuenetale, Ibrahim Farahat El Bayoumy, Maysaa El Sayed Zaki, Iffat Elbarazi, Faris El-Dahiyat, Islam Y Elgendy, Muhammed Elhadi, Mohamed A Elmonem, Adel B Elmoselhi, Chadi Eltaha, Theophilus I Emeto, Christopher Imokhuede Esezobor, Negin Esfandiari, Zahra Esmaeili, Francesco Esposito, Mohammad Etoom, Natalia Fabin, Adeniyi Francis Fagbamigbe, Omotayo Francis Fagbule, Shahriar Faghani, Ayesha Fahim, Luca Falzone, Mohammad Fareed, Jawad Fares, Carla Sofia e Sá Farinha, MoezAlIslam Ezzat Mahmoud Faris, Mohsen Farjoud Kouhanjani, Andre Faro, Nelsensius Klau Fauk, Valery L Feigin, Seyed-Mohammad Fereshtehnejad, Pietro Ferrara, Nuno Ferreira, Getahun Fetensa, Florian Fischer, Kristen Marie Foley, Ana Catarina Fonseca, Behzad Foroutan, Daniela Fortuna, Matteo Foschi, Ni Kadek Yuni Fridayani, Peter Andras Gaal, Abduzhappar Gaipov, Yaseen Galali, Silvano Gallus, Aravind P Gandhi, Balasankar Ganesan, Danijela Gasevic, Prem Gautam, Rupesh K Gautam, Miglas Welay Gebregergis, Kebre Gebrekirstos Gebrekidan, Lemma Getacher, Molla Getie, Delaram J Ghadimi, Fataneh Ghadirian, Mohammad-Reza Ghasemi, Afsaneh Ghasemzadeh,

Ehsan Gholami, Zainab Gholami, Sherief Ghozy, Stefano Giannoni Luza, Jaleed Ahmed Gilani, Tiffany K Gill, Richard F Gillum, Ebisa Zerihun Gindaba, Elena V Gnedovskaya, Amit Goel, Mohamad Goldust, Davide Golinelli, Philimon N Gona, Giuseppe Gorini, Barbara Niegia Garcia Goulart, Mahdi Gouravani, Michal Grivna, Ashna Grover, Shi-Yang Guan, Giovanni Guarducci, Avirup Guha, Stefano Guicciardi, Snigdha Gulati, Damitha Asanga Gunawardane, Cui Guo, Bhawna Gupta, Mohak Gupta, Rahul Gupta, Rajat Das Gupta, Rajeev Gupta, Sapna Gupta, Farrokh Habibzadeh, Najah R Hadi, Mohammad Haghani Dogahe, Hamed Haghi-Aminjan, Arvin Haj-Mirzaian, Aram Halimi, Nadia M Hamdy, Nasrin Hanifi, Graeme J Hankey, Md Abdul Hannan, Zaim Anan Haq, Netanja I Harlianto, Josep Maria Haro, Eka Mishbahatul Marah Has, Ahmed I Hasaballah, Md Saquib Hasnain, Ikrama Hassan, Mahgol Sadat Hassan Zadeh Tabatabaei, Johannes Haubold, Rasmus J Havmoeller, Jeffrey J Hebert, Omar E Hegazi, Mehdi Hemmati, Demisu Zenbaba Heyi, Kamal Hezam, Yuta Hiraike, Nguyen Quoc Hoan, Ramesh Holla, Nobuyuki Horita, Md Mahbub Hossain, Ahmad Hosseinzadeh Adli, Sorin Hostiuc, Ben Hu, Junjie Huang, Salman Hussain, Hong-Han Huynh, Segun Emmanuel Ibitoye, Adalia Ikiroma, Olayinka Stephen Ilesanmi, Irena M Ilic, Milena D Ilic, Mustapha Immurana, Muhammad Iqhrammullah, Arnaud Iradukunda, Lalu Muhammad Irham, Md Rabiul Islam, Faisal Ismail, Nahlah Elkudssiah Ismail, Hiroyasu Iso, Gaetano Isola, Ramaiah Itumalla, Chidozie Declan CD Iwu, Louis Jacob, Abdollah Jafarzadeh, Haitham Jahrami, Akhil Jain, Abhishek Jaiswal, Mihajlo Jakovljevic, Reza Jalilzadeh Yengejeh, Balamurugan Janakiraman, Abubakar Ibrahim Jatau, Sathish Kumar Jayapal, Shubha Jayaram, Alelign Tasew Jema, Bijay Mukesh Jeswani, Ravi Prakash Jha, Zixiang Ji, Shuai Jin, Yingzhao Jin, Catherine O Johnson, Jost B Jonas, Tamas Joo, Jobinse Jose, Nitin Joseph, Charity Ehimwenma Joshua, Jacek Jerzy Jozwiak, Mikk Jürisson, Ali Kabir, Md Awal Kabir, Vidya Kadashetti, Rizwan Kalani, Sanjay Kalra, Arun Kamireddy, Mona Kanaan, Himal Kandel, Kehinde Kazeem Kanmodi, Rami S Kantar, Neeti Kapoor, Paschalis Karakasis, André Karch, Hanie Karimi, Yeganeh Karimi, Prabin Karki, Hengameh Kasraei, Joonas H Kauppila, Gbenga A Kayode, Foad Kazemi, Emmanuelle Kesse-Guyot, Yousef Saleh Khader, Himanshu Khajuria, Amirmohammad Khalaji, Nauman Khalid, Anees Ahmed Khalil, Fayaz Khan, M Nuruzzaman Khan, Maseer Khan, Mohammad Jobair Khan, Moien AB Khan, Yusra H Khan, Shaghayegh Khanmohammadi, Khaled Khatab, Haitham Khatatbeh, Moawiah Mohammad Khatatbeh, Sorour Khateri, Mahalaqua Nazli Khatib, Hamid Reza Khayat Kashani, Feriha Fatima Khidri, Atulya Aman Khosla, Majid Khosravi, Jagdish Khubchandani, Kwanghyun Kim, Min Seo Kim, Yun Jin Kim, Adnan Kisa, Sezer Kisa, Farzad Kompani, Shivakumar KM Marulasiddaiah Kondlahalli, Oleksii Korzh, Karel Kostev, Nikhil Kothari, Ashwin Laxmikant Kotnis, Sindhura Lakshmi Koulmane Laxminarayana, Kewal Krishan, Varun Krishna, Jera Kruja, Barthelemy Kuate Defo, Burcu Kucuk Bicer, Md Abdul Kuddus, Mohammed Kuddus, Nuworza Kugbey, Mukhtar Kulimbet, Vishnutheertha Kulkarni, Ashish Kumar, Dewesh Kumar, Rakesh Kumar, Senthil Kumaran D, Amartya Kundu, Satyajit Kundu, Setor K Kunutsor, Om P Kurmi, Dian Kusuma, L V Simhachalam Kutikuppala, Ville Kytö, Carlo La Vecchia, Ben Lacey, Chandrakant Lahariya, Tea Lallukka, Judit Lám, Iván Landires, Anders O Larsson, Savita Lasrado, Kaveh Latifinaibin, Pablo M Lavados, Basira Kankia Lawal, Nhi Huu Hanh Le, Thao Thi Thu Le, Paul H Lee, Wei Li, Lee-Ling Lim, John C Lin, Daniel Lindholm, Runben Liu, Xuefeng Liu, Erand Llanaj, Chun-Han Lo, Warren David Lo, Valerie Lohner, José Francisco López-Gil, László Lorenzovici, Stefan Lorkowski, Paulo A Lotufo, Giancarlo Lucchetti, Jay B Lusk, Zheng Feei Ma, Monika Machoy, Farzan Madadizadeh, Elham Mahmoudi, Golnaz Mahmoudvand, Omar M Makram, Elaheh Malakan Rad, Kashish Malhotra, Ahmad Azam Malik, Tauqeer Hussain Mallhi, Deborah Carvalho Malta, Abdullah A Mamun, Vahid Mansouri, Lorenzo Giovanni

Mantovani, Emmanuel Manu, Hamid Reza Marateb, Daniela Martini, Miquel Martorell, Sharmeen Maryam, Roy Rillera Marzo, Awoke Masrie, Yasith Mathangasinghe, Mahsa Mayeli, Mohsen Mazidi, Steven M McPhail, Asim Mehmood, Kamran Mehrabani-Zeinabad, Tesfahun Mekene Meto, Hadush Negash Meles, Walter Mendoza, Ritesh G Menezes, George A Mensah, Sultan Ayoub Meo, Atte Meretoja, Tuomo J Meretoja, Tomislav Mestrovic, Tomasz Miazgowski, Irmina Maria Michalek, Ana Carolina Micheletti Gomide Nogueira de Sá, Giuseppe Minervini, Le Huu Nhat Minh, Mojgan Mirghafourvand, Mohammad Mirza-Aghazadeh-Attari, Manish Mishra, Prasanna Mithra, Nouh Saad Mohamed, Ameen Mosa Mohammad, Esmaeil Mohammadi, Abdollah Mohammadian-Hafshejani, Hussen Mohammed, Mustapha Mohammed, Salahuddin Mohammed, Shafiu Mohammed, Ali H Mokdad, Hossein Molavi Vardanjani, Mariam Molokhia, Shaher Momani, Lorenzo Monasta, Mohammad Ali Moni, Fateme Montazeri, AmirAli Moodi Ghalibaf, Mahmood Moosazadeh, Maryam Moradi, Yousef Moradi, Paula Moraga, Rafael Silveira Moreira, Shane Douglas Morrison, Reza Mosaddeghi Heris, Parsa Mousavi, Ahmed Msherghi, Lorenzo Muccioli, Admir Mulita, Malaisamy Muniyandi, Efren Murillo-Zamora, Christopher J L Murray, Amin Nabavi, Ashraf Fawzy Nabhan, Ayoub Nafei, Ahamarshan Jayaraman Nagarajan, Mukhammad David Naimzada, Balakrishnan Sukumaran Nair, Sanjeev Nair, Tapas Sadasivan Nair, Soroush Najdaghi, Noureddin Nakhostin Ansari, Sreenivas Narasimha Swamy, Shumaila Nargus, Delaram Narimani Davani, Bruno Ramos Nascimento, Gustavo G Nascimento, Ali Nasrollahizadeh, Amir Nasrollahizadeh, Zuhair S Natto, Javaid Nauman, Samidi Nirasha Kumari Navaratna, Nawsherwan Nawsherwan, Rawlance Ndejjo, Ionut Negoi, Ruxandra Irina Negoi, Mohammad Hadi Nematollahi, Samata Nepal, Charles Richard James Newton, Dang H Nguyen, Duc Hoang Nguyen, Hau Thi Hien Nguyen, Hien Quang Nguyen, Nhien Ngoc Y Nguyen, Phat Tuan Nguyen, Van Thanh Nguyen, Robina Khan Niazi, Yeshambel T Nigatu, Nasrin Nikravangolsefid, Chukwudi A Nnaji, Shuhei Nomura, Syed Toukir Ahmed Noor, Bo Norrving, Jean Jacques Noubiap, Chisom Adaobi Nri-Ezedi, George Ntaios, Mpiko Ntsekhe, Fred Nugen, Mario Cesare Nurchis, Dieta Nurrika, Chimezie Igwegbe Nzoputam, Ogochukwu Janet Nzoputam, Bogdan Oancea, Kehinde O Obamiro, Ismail A Odetokun, Martin James O'Donnell, Sylvester Reuben Okeke, Akinkunmi Paul Okekunle, Osaretin Christabel Okonji, Andrew T Olagunju, Oladotun Victor Olalusi, Tosin Abiola Olasehinde, Matthew Idowu Olatubi, Arão Belitardo Oliveira, Abdulhakeem Abayomi Olorukooba, Hany A Omar, Adrienne E O'Neil, Obinna E Onwujekwe, Michal Ordak, Raffaele Ornello, Doris V Ortega-Altamirano, Alberto Ortiz, Esteban Ortiz-Prado, Wael M S Osman, Uchechukwu Levi Osuagwu, Stanislav S Otstavnov, Mayowa O Owolabi, Ifeoluwa Temitayo Oyeyemi, Mahesh Padukudru P A, Kevin Pacheco-Barrios, Alicia Padron-Monedero, Jagadish Rao Padubidri, Tamás Palicz, Raul Felipe Palma-Alvarez, Feng Pan, Songhomitra Panda-Jonas, Deepshikha Pande Katare, Ashok Pandey, Ioannis Pantazopoulos, Paraskevi Papadopoulou, Shahina Pardhan, Romil R Parikh, Roberto Passera, Dimitrios Patoulias, Uttam Paudel, Shrikant Pawar, Amy E Peden, Paolo Pedersini, Maria Odete Pereira, Arokiasamy Perianayagam, Norberto Perico, Richard G Pestell, Ionela-Roxana Petcu, Fanny Emily Petermann-Rocha, Hoang Nhat Pham, Hoang Tran Pham, Michael R Phillips, Thomas Pilgrim, Michael A Piradov, Saeed Pirouzpanah, Ramesh Poluru, Djordje S Popovic, Maarten J Postma, Naeimeh Pourtaheri, Jalandhar Pradhan, Pranil Man Singh Pradhan, V Prakash, Manya Prasad, Elton Junio Sady Prates, Ibrahim Qattea, Yanan Qiao, Alberto Raggi, Pankaja Raghav Raghav, Pracheth Raghuveer, Fakher Rahim, Mahban Rahimifard, Vafa Rahimi-Movaghar, Md Mosfequr Rahman, Mohammad Hifz Ur Rahman, Mohammad Rahmanian, Rahem Rahmati, Sathish Rajaa, Pushp Lata Rajpoot, Prashant Rajput,

Pradhum Ram, Mahmoud Mohammed Ramadan, Venkitachalam Ramanarayanan, Shakthi Kumaran Ramasamy, Kritika Rana, Rishabh Kumar Rana, Chhabi Lal Ranabhat, Nemanja Rancic, Annemarei Ranta, Sowmya J Rao, Ashkan Rasouli-Saravani, Devarajan Rathish, Ilari Rautalin, Salman Rawaf, Elrashdy Moustafa Mohamed Redwan, Giuseppe Remuzzi, Nazila Rezaei, Hossein Rezazadeh, Taeho Gregory Rhee, Mavra A Riaz, Antonio Luiz P Ribeiro, Monica Rodrigues, Thales Philipe R Rodrigues da Silva, Jefferson Antonio Buendia Rodriguez, Leonardo Roever, Debby Syahru Romadlon, Allen Guy Ross, Himanshu Sekhar Rout, Bedanta Roy, Guilherme de Andrade Ruela, Michele Russo, Godfrey M Rwegerera, Aly M A Saad, Korosh Saber, Maha Mohamed Saber-Ayad, Cameron John Sabet, Siamak Sabour, Simona Sacco, Basema Ahmad Saddik, Umar Saeed, Rajesh Sagar, Alireza Saghafi, Dominic Sagoe, Fatemeh Saheb Sharif-Askari, Amirhossein Sahebkar, Soumya Swaroop Sahoo, Mirza Rizwan Sajid, Afeez Abolarinwa Salami, Luciane B Salaroli, Mohammed Z Y Salem, Giovanni A Salum, Sara Samadzadeh, Saad Samargandy, Vijaya Paul Samuel, Abdallah M Samy, Juan Sanabria, Itamar S Santos, Milena M Santric-Milicevic, Aswini Saravanan, Yaser Sarikhani, Gargi Sachin Sarode, Sachin C Sarode, Maheswar Satpathy, Zafer Sattouf, Ganesh Kumar Saya, Md Abu Sayeed, Nikolaos Scarmeas, Benedikt Michael Schaarschmidt, Markus P Schlaich, Maria Inês Schmidt, Ione Jayce Ceola Schneider, Art Schuermans, Aletta Elisabeth Schutte, David C Schwebel, Siddharthan Selvaraj, Sabyasachi Senapati, Yashendra Sethi, Allen Seylani, Mahan Shafie, Pritik A Shah, Saeed Shahabi, Samiah Shahid, Moyad Jamal Shahwan, Ali S Shalash, Muhammad Aaqib Shamim, Mehran Shams-Beyranvand, Anas Shamsi, Mohd Shanawaz, Mohammed Shannawaz, Medha Sharath, Amin Sharifan, Anupam Sharma, Manoj Sharma, Sourabh Sharma, Ujjawal Sharma, Vishal Sharma, Mahabalesh Shetty, Premalatha K Shetty, Mika Shigematsu, Aminu Shittu, Abdul-karim Olayinka Shitu, Seyed Afshin Shorofi, Sunil Shrestha, Emmanuel Edwar Siddig, João Pedro Silva, Luís Manuel Lopes Rodrigues Silva, Abhinav Singh, Baljinder Singh, Harmanjit Singh, Jasvinder A Singh, Kuldeep Singh, Narinder Pal Singh, Paramdeep Singh, Puneetpal Singh, Jussi O T Sipilä, Shravan Sivakumar, Valentin Yurievich Skryabin, Anna Aleksandrovna Skryabina, Farrukh Sobia, Bogdan Socea, Abdullah Al Mamun Sohag, Ranjan Solanki, Shipra Solanki, Yi Song, Soroush Soraneh, Ireneous N Soyiri, Michael Spartalis, Chandrashekhar T Sreeramareddy, Suresh Kumar Srinivasamurthy, Panagiotis Stachteas, Narayan Subedi, Vetriselvan Subramaniyan, Muhammad Suleman, Abida Sultana, Zhong Sun, Chandan Kumar Swain, Lukasz Szarpak, Payam Tabaee Damavandi, Shima Tabatabai, Celine Tabche, Jyothi Tadakamadla, Santosh Kumar Tadakamadla, Amirmasoud Taheri, Iman M Talaat, Mircea Tampa, Jacques Lukenze Tamuzi, Ker-Kan Tan, Manoj Tanwar, Nathan Y Tat, Seyed Mohammad Tavangar, Arash Tehrani-Banihashemi, Mojtaba Teimoori, Mohamad-Hani Temsah, Reem Mohamad Hani Temsah, Masayuki Teramoto, Wegen Beyene Tesfamariam, Ramna Thakur, Pugazhenthan Thangaraju, Sathish Thirunavukkarasu, Joe Thomas, Jing Tian, Marcello Tonelli, Roman Topor-Madry, Marcos Roberto Tovani-Palone, Khaled Trabelsi, Thang Huu Tran, Domenico Trico, Samuel Joseph Tromans, Thien Tan Tri Tai Truyen, Daniel Hsiang-Te Tsai, Aristidis Tsatsakis, Evangelia Eirini Tsermpini, Stefanos Tyrovolas, Aniefiok John Udoakang, Arit Udoh, Muhammad Umair, Brigid Unim, Bhaskaran Unnikrishnan, Daniele Urso, Jibrin Sammani Usman, Marco Vacante, Sanaz Vahdati, Asokan Govindaraj Vaithinathan, Omid Vakili, Jef Van den Eynde, Orsolya Varga, Shoban Babu Varthya, Tommi Juhani Vasankari, Balachandar Vellingiri, Narayanaswamy Venketasubramanian, Madhur Verma, Massimiliano Veroux, Georgios-Ioannis Verras, Dominique Vervoort, Simona Villani, Manish Vinayak, Simona Ruxandra Volovat, Victor Volovici, Hatem A Wafa, Waseem Wahood, Cong Wang, Fang Wang, Shu Wang, Song Wang,

Yuan-Pang Wang, Mary Njeri Wanjau, Emebet Gashaw Wassie, Gizachew Tadesse Wassie, Robert G Weintraub, Dakshitha Praneeth Wickramasinghe, Nuwan Darshana Wickramasinghe, Tissa Wijeratne, Peter Willeit, Charles D A Wolfe, Yen Jun Wong, YaJuan Wu, Kazumasa Yamagishi, Amir Yarahmadi, Yuichi Yasufuku, Mohammad Hosein Yazdanpanah, Pengpeng Ye, Saber Yezli, Dong Keon Yon, Naohiro Yonemoto, Elaine A Yu, Ke Yun, Hadiza Yusuf, Nima Zafari, Burhan Abdullah Zaman, Sojib Bin Zaman, Aurora Zanghì, Iman Zare, Fatemeh Zarimeidani, Armin Zarrintan, Michael Zastrozhin, Dawit Zemedikun, Youjie Zeng, Beijian Zhang, Haijun Zhang, Zhiqiang Zhang, Claire Chenwen Zhong, Abzal Zhumagaliuly, Makan Ziafati, Magdalena Zielińska, Yossef Teshome Zikarg, Ghazal Zoghi, Sa'ed H Zyoud, and Samer H Zyoud.

Managing the estimation or publications process

Erin B Hamilton, Simon I Hay, Ali H Mokdad, Christopher J L Murray, and Gregory A Roth.

Section 1. Summary of GBD methods for stroke burden estimates

This section provides further methodological detail for "Global, regional and national burden of stroke and its risk factors, 1990-2021: A systematic analysis for the Global Burden of Disease Study 2021." This study complies with the Guidelines for Accurate and Transparent Health Estimates Reporting (GATHER) recommendations.[8] It includes detailed tables and information on data to maximise transparency in our estimation processes and provides a comprehensive description of analytical steps. We intend this Appendix material to be a living document, to be updated with each iteration of the Global Burden of Disease Study (GBD).

We produced estimates for 204 countries and territories that were grouped into 21 regions and seven super-regions (Appendix Figure 1). The seven super-regions are central Europe, eastern Europe, and central Asia; high income; Latin America and the Caribbean; north Africa and the Middle East; south Asia; southeast Asia, east Asia, and Oceania; and sub-Saharan Africa. For GBD 2021, nine countries and territories (Cook Islands, Monaco, San Marino, Nauru, Niue, Palau, Saint Kitts and Nevis, Tokelau, and Tuvalu) were added, such that the GBD location hierarchy now includes all WHO member states. In this round, the GBD included subnational analyses for several new countries and continues to analyse at subnational levels countries that were added in previous cycles. Subnational estimation in GBD 2021 includes five new countries (Italy, Nigeria, Pakistan, the Philippines, and Poland) and 16 countries previously estimated at subnational levels (Brazil, China, Ethiopia, India, Indonesia, Iran, Japan, Kenya, Mexico, New Zealand, Norway, Russia, South Africa, Sweden, the UK, and the USA).

Results from GBD 2021 are available through an interactive data downloading tool on the Global Health Data Exchange (GHDx). The GHDx is the world's most comprehensive catalogue of surveys, censuses, vital statistics, and other health-related data. Results are measured in terabytes. The latest version of the data download tool, available here: http://ghdx.healthdata.org/GBD-resultstool, contains core summary results for GBD 2021.

These results include deaths, years of life lost (YLLs), YLDs, disability-adjusted life-years (DALYs), prevalence, incidence, and rate of change. The GHDx includes data for causes, risks, cause-risk attribution, aetiologies, and impairments. Data above a certain size cannot be viewed online but can be downloaded. Depending on the size of the download, users may need to enter an email address; a download location will be sent to them when the files are prepared. All GBD 2021 online data visualisations are available at http://vizhub.healthdata.org/GBD-compare, which provides results for all GBD health metrics.

1.1 Input data

For the GBD 2021 study, in order to better represent population-level disease incidence for IS, adjustments for alternative study methods and case definitions were applied to data prior to analysis in DisMod-MR. These adjustments were performed using the MR-BRT modelling tool. We adjusted for several study-specific factors such as whether the data were from a hospital and whether the data included both first-ever and recurrent ischaemic strokes. We updated our methods for redistributing deaths due to unspecified stroke (ICD-10 codes I62 and I64) to the three modelled stroke subtypes included in GBD.

Verbal autopsy and vital registration data were used to model cerebrovascular disease (stroke). We reassigned deaths from verbal autopsy reports for cerebrovascular disease to the parent cardiovascular disease for both sexes for those under 20 years of age. We outliered non-representative subnational verbal autopsy datapoints. We also outliered ICD8, ICD9BTL, and tabulated ICD10 datapoints which were inconsistent with the rest of the data and created implausible time trends. Datapoints from sources which were implausibly low in all age groups and data points that were causing the regional estimates to be improbably high were outliered.

Tables 1a, 1b, and 1c display source count information for non-fatal ischaemic stroke, intracerebral haemorrhage, and subarachnoid haemorrhage respectively.

Table 1a: Source counts for ischaemic stroke models.

Measure	Total sources	Countries with data
All measures	523	76
Prevalence	117	24
Incidence	332	62
Excess mortality rate	141	47
Case fatality rate	50	22

Table 1b: Source counts for intracerebral haemorrhage models.

Measure	Total sources	Countries with data
All measures	502	74
Prevalence	117	24
Incidence	322	61
Excess mortality rate	125	41
Case fatality rate	40	18

Table 1c: Source counts for subarachnoid haemorrhage models.

Measure	Total sources	Countries with data
All measures	435	63
Prevalence	117	24
Incidence	260	47
Excess mortality rate	88	28

1.2 Search terms for systematic review

A systematic review was performed for GBD 2021. Search terms, dates of search, and databases queried are as follow:

1) Ischaemic stroke
    a. Google scholar: ("ischemic stroke" OR “cerebral infarction” OR “ischaemic stroke”) AND (incidence OR prevalence OR mortality OR epidemiology). Reviewed first 1000 hits, sorted by relevance.
    b. Global Index Medicus search: (tw:("ischemic stroke") OR tw:(“cerebral infarction” OR tw:(“ischaemic stroke”)) AND (tw:(incidence) OR tw:(prevalence) OR tw:(mortality) OR tw:(epidemiology)) AND NOT (tw:(rats) OR tw:(mice) OR tw:(dogs) OR tw:(apes) OR tw:(monkeys)). Dates of search: 01Jan2010 – 31Aug2017
2) Intracerebral haemorrhage
    a. Google scholar: ("hemorrhagic stroke" OR “intracerebral hemorrhage” OR “haemorrhagic stroke” OR “intracerebral haemorrhage”) AND (incidence OR prevalence OR mortality OR epidemiology). Reviewed first 1000 hits, sorted by relevance.
    b. GIM search: (tw:("intracerebral hemorrhage") OR tw:(“intracerebral haemorrhage”) OR tw:(“hemorrhagic stroke”) OR tw:(“haemorrhagic stroke”)) AND (tw:(incidence) OR tw:(prevalence) OR tw:(mortality) OR tw:(epidemiology)) AND NOT (tw:(rats) OR tw:(mice) OR tw:(dogs) OR tw:(apes) OR tw:(monkeys)). Dates of search: 01Jan2010 – 31Aug2017
3) Subarachnoid haemorrhage
    a. Google scholar search: ("subarachnoid hemorrhage" OR “subarachnoid haemorrhage”) AND (incidence OR prevalence OR mortality OR epidemiology). Reviewed first 1000 hits, sorted by relevance.
    b. GIM search: (tw:("subarachnoid hemorrhage") OR tw:(“subarachnoid haemorrhage”)) AND (tw:(incidence) OR tw:(prevalence) OR tw:(mortality) OR tw:(epidemiology)) AND NOT (tw:(rats) OR tw:(mice) OR tw:(dogs) OR tw:(apes) OR tw:(monkeys)). Dates of search: 01Jan2010 – 31Aug2017

We included inpatient hospital data, adjusted for readmission and to any diagnosis using correction factors estimated from US claims data. We excluded data for locations where the data points were implausibly low (Vietnam, Philippines, India). In addition, we included unpublished stroke registry data for acute ischaemic stroke, acute intracerebral haemorrhage, and acute subarachnoid haemorrhage. We also included survey data for chronic stroke. These surveys were identified based on expert opinion and review of major survey series focused on world health that included questions regarding self- reported history of stroke. For GBD 2021, we split unspecified strokes (ICD-10 I64) into ischaemic stroke, intracerebral haemorrhage, and subarachnoid haemorrhage according to the proportions of subtype-specific coded strokes in the original data. We also split ICD-10 I62 into intracerebral haemorrhage, and subarachnoid haemorrhage using the same approach.

As with many models in GBD, the diversity of data sources available means that we needed to adjust available data to our reference case definition. We thus crosswalked incidence and excess mortality

data that did not meet our reference case definitions using MR- BRT, a Bayesian meta-regression tool developed for the GBD. More information on MR-BRT can be found elsewhere in the Appendix.

We adjusted data points for first and recurrent strokes combined, using data for first strokes only as reference. For ischaemic stroke and intracerebral haemorrhage, we also adjusted data points that reported all stroke subtypes combined, using reference studies with subtype-specific information. We also adjusted data which included only persons who survived to hospital admission, using reference data on both fatal and nonfatal strokes. In addition, we adjusted subtype-specific, inpatient clinical informatics data using subtype-specific literature estimates as a reference. These adjustments can be examined more closely in Table 2. The coefficients in Tables 2a, 2b, and 2c below can be used to calculate adjustment factors for alternative definitions. The formula for computing adjustment factors is given in equation 1 below. We also included a standardised age variable (age scaled) and a sex variable to the crosswalking procedure to adjust for the possibly of bias.

Equation 1: Calculation of adjustment factors:
Estimated Reference Def = invogit(logit(Alternative Def) – $Beta_{Alternative\ Def}$ – $Beta_{Sex}$ * Sex – $Beta_{Age\ scaled}$ * Age Scaled)

Table 2a: MR-BRT Crosswalk Adjustment Factors for Ischaemic stroke

	Data input	Measure	Reference or alternative case definition	Gamma	Beta Coefficient, Logit (95% CI)
Ischaemic stroke	First-ever, subtype-specific, fatal and nonfatal events	Incidence	Ref	---	---
Ischaemic stroke	Hospital data	Incidence	Alt	0.97	-0.26 (-2.22 to 1.70)
Ischaemic stroke	Any stroke	Incidence	Alt		0.02 (-1.94 to 1.98)
Ischaemic stroke	Acute first-ever stroke	Incidence	Alt		0.22 (-1.67 to 2.12)
Ischaemic stroke	Inpatient clinical informatics	Incidence	Alt		0.70 (-1.26 to 2.66)
Ischaemic stroke	Sex (male)	Incidence	Alt		0.07 (-1.82 to 1.96)
Ischaemic stroke	Age scaled	Incidence	Alt		0.28 (-1.61 to 2.17)

Table 2b: MR-BRT Crosswalk Adjustment Factors for Intracerebral Haemorrhage

	Data input	Measure	Reference or alternative case definition	Gamma	Beta Coefficient, Logit (95% CI)
Intracerebral Haemorrhage	First-ever, subtype-specific, fatal and nonfatal events	Incidence	Ref	---	---
Intracerebral Haemorrhage	Hospital data	Incidence	Alt		0.04 (-0.93 to 1.02)

Intracerebral Haemorrhage	Any stroke	Incidence	Alt		1.78 (0.80 to 2.76)
Intracerebral Haemorrhage	Acute first-ever stroke	Incidence	Alt	0.50	0.15 (-0.83 to 1.13)
Intracerebral Haemorrhage	Inpatient clinical informatics	Incidence	Alt		1.40 (0.41 to 2.38)
Intracerebral Haemorrhage	Age scaled	Incidence	Alt		0.09 (-0.88 to 1.07)
Intracerebral Haemorrhage	Sex (male)	Incidence	Alt		0.10 (-0.88 to 1.06)

Table 2c: MR-BRT Crosswalk Adjustment Factors for Subarachnoid Haemorrhage

	Data input	Measure	Reference or alternative case definition	Gamma	Beta Coefficient, Logit (95% CI)
Subarachnoid Haemorrhage	First-ever, subtype-specific, fatal and nonfatal events	Incidence	Ref	---	---
Subarachnoid Haemorrhage	Aneurysmal subarachnoid haemorrhage only	Incidence	Alt	0.76	-0.79 (-2.28 to 0.70)
Subarachnoid Haemorrhage	Age scaled	Incidence	Alt		-0.11 (-1.59 to 1.38)
Subarachnoid Haemorrhage	Sex (male)	Incidence	Alt		-0.07 (-1.56 to 1.42)

1.3 Severity split inputs

The table below illustrates the severity level, lay description, and disability weights for GBD 2021. In previous iterations of the GBD, severity splits for stroke were based on the standard approach described elsewhere (3). For GBD 2016, we undertook a review to identify epidemiologic literature which reported the degree of disability at 28 days (for acute stroke) or one year (for chronic stroke) using the modified Rankin scale (mRS) and the Mini-Mental State Examination (MMSE) or the Montreal Cognitive Assessment (MoCA). The mRS assesses functional capabilities, while the MMSE and MoCA tests provide evaluations of cognitive functioning. We then mapped these measures to the existing GBD categories as indicated below. This approach allowed us to include location-specific information and can be updated as more data on functional or cognitive status become available.

1.3.1 Acute stroke severity splits

Table 3a. Severity distribution, details on the severity levels for Acute Stroke in GBD 2021 and the associated disability weight (DW) with that severity.

Severity level	Lay description	Modified Rankin score	Cognitive status	DW (95% CI)
Stroke, mild	Has some difficulty in moving around and some weakness in one hand, but is able to walk without help.	1	N/A	0.019 (0.01–0.032)
Stroke, moderate	Has some difficulty in moving around, and in using the hands for lifting and holding things, dressing, and grooming.	2, 3	MoCA>=24 or MMSE>=26	0.07 (0.046–0.099)
Stroke, moderate plus cognition problems	Has some difficulty in moving around, in using the hands for lifting and holding things, dressing and grooming, and in speaking. The person is often forgetful and confused.	2, 3	MoCA<24 or MMSE<26	0.316 (0.206–0.437)
Stroke, severe	Is confined to bed or a wheelchair, has difficulty speaking, and depends on others for feeding, toileting, and dressing.	4, 5	MoCA>=24 or MMSE>=26	0.552 (0.377–0.707)
Stroke, severe plus cognition problems	Is confined to bed or a wheelchair, depends on others for feeding, toileting, and dressing, and has difficulty speaking, thinking clearly, and remembering things.		MoCA<24 or MMSE<26	0.588 (0.411–0.744)

1.3.2 Chronic stroke severity splits

Table 3b. Severity distribution, details on the severity levels for Chronic Stroke in GBD 2021 and the associated disability weight (DW) with that severity.

Severity level	Lay description	Modified Rankin score	Cognitive status	DW (95% CI)
Stroke, asymptomatic		0	N/A	N/A
Stroke, long-term consequences, mild	Has some difficulty in moving around and some weakness in one hand, but is able to walk without help.	1	N/A	0.019 (0.01–0.032)
Stroke, long-term consequences, moderate	Has some difficulty in moving around, and in using the hands for lifting and holding things, dressing, and grooming.	2, 3	MoCA>=24 or MMSE>=26	0.07 (0.046–0.099)

Stroke, long-term consequences, moderate plus cognition problems	Has some difficulty in moving around, in using the hands for lifting and holding things, dressing and grooming, and in speaking. The person is often forgetful and confused.	2, 3	MoCA<24 or MMSE<26	0.316 (0.206–0.437)
Stroke, long-term consequences, severe	Is confined to bed or a wheelchair, has difficulty speaking, and depends on others for feeding, toileting, and dressing.	4, 5	MoCA>=24 or MMSE>=26	0.552 (0.377–0.707)
Stroke, long-term consequences, severe plus cognition problems	Is confined to bed or a wheelchair, depends on others for feeding, toileting, and dressing, and has difficulty speaking, thinking clearly, and remembering things.	4, 5	MoCA<24 or MMSE<26	0.588 (0.411–0.744)

Table 4: Data input counts for the estimation process for the custom severity splits.

	Acute proportion	Chronic proportion
Site-years (total)	9	16
Number of countries with data	6	13
Number of GBD regions with data (out of 21 regions)	6	7
Number of GBD super-regions with data (out of 7 super-regions)	4	5

We used DisMod-MR, a Bayesian meta-regression tool, to model the six severity levels, with an independent proportion model for each. Reports which grouped mRS scores differently than our mapping (e.g., 0-2) were adjusted in DisMod by estimating the association between these alternate groupings and our preferred mappings. These statistical associations were used to adjust data points to the referent category as necessary. The six models were scaled such that the sum of the proportions for all levels equalled 1.

1.4 Modelling strategy

The general approach employed for all of the components of the stroke modelling process is detailed in the table below.

- Data points were adjusted from alternative to reference case definitions using estimates from statistical models generated by MR-BRT (discussed elsewhere in the Appendix) for the acute models. Coefficients for these crosswalks can be found in Tables 2a, 2b, and 2c.

- The GBD summary exposure values (SEV), which are the relative risk-weighted prevalence of exposure, were included as covariates for the ischaemic stroke or intracerebral haemorrhage models as appropriate, and a covariate for country income was used as a country-level covariate for both models (4). Subarachnoid haemorrhage did not include an SEV covariate but did include

a covariate for country income for excess mortality. Coefficients for these covariates can be found in Tables 5a, 5b, 5c for fixed effects located below.

- We used the ratio of acute:chronic cause-specific mortality estimated by the final GBD 2017 Dismod model estimates to divide GBD 2021 stroke deaths into acute and chronic stroke deaths, using the global average for the proportion of acute:chronic stroke mortality. The acute and chronic models were then run using the same incidence, prevalence, and case fatality data as well as the custom cause-specific mortality rates as input data.

- We ran the first-ever acute subtype-specific models with CSMR as derived from FauxCorrect and epidemiological data as described above using Dismod-MR.

- We then calculated the rate of surviving until 28 days after an acute event for all three subtypes using the modelled estimates of excess mortality and incidence from the acute stroke models.

- Twenty-eight-day survivorship data was uploaded into the chronic subtype-specific with CSMR models. These chronic models also use CSMR as derived from FauxCorrect and epidemiological data as described above. Models were evaluated based on expert opinion, comparison with previous iterations, and model fit.

Tables 5a, 5b, 5c below indicate the covariates used by cause in the estimation process, as well as the beta and exponentiated beta values.

Table 5a: Coefficients for covariates used in the acute and chronic ischemic stroke DisMod-MR models

Model	Variable name	Measure	beta	Exponentiated beta
First-ever acute ischaemic stroke with CSMR	Log-transformed age-standardised SEV scalar: Ischaemic stroke	Incidence	0.90 (0.85 to 0.95)	2.46 (2.34 to 2.58)
First-ever acute ischaemic stroke with CSMR	Healthcare access and quality index	Excess mortality rate	-0.035 (-0.035 to -0.035)	0.97 (0.97 to 0.97)
Chronic ischaemic stroke with CSMR	Log-transformed SEV scalar: Ischaemic stroke	Prevalence	0.85 (0.78 to 0.92)	2.34 (2.18 to 2.51)
Chronic ischaemic stroke with CSMR	LDI (I$ per capita)	Excess mortality rate	-0.41 (-0.46 to -0.36)	0.67 (0.63 to 0.70)

Table 5b: Coefficients for covariates used in the acute and chronic intracerebral haemorrhage DisMod-MR models

Model	Variable name	Measure	beta	Exponentiated beta
First-ever acute intracerebral haemorrhage with CSMR	Log-transformed SEV scalar: Intracerebral Haemorrhage	Incidence	0.76 (0.75 to 0.77)	2.13 (2.12 to 2.15)
First-ever acute intracerebral haemorrhage with CSMR	Healthcare access and quality index	Excess mortality rate	-0.07 (-0.07 to -0.069)	0.93 (0.93 to 0.93)

Chronic intracerebral haemorrhage with CSMR	Log-transformed SEV scalar: Intracerebral haemorrhage	Prevalence	0.75 (0.75 to 0.76)	2.12 (2.12 to 2.14)
Chronic intracerebral haemorrhage with CSMR	LDI (I$ per capita)	Excess mortality rate	-0.5 (-0.5 to -0.5)	0.61 (0.61 to 0.61)

Table 5c: Coefficients for covariates used in the acute and chronic subarachnoid DisMod-MR models

Model	Variable name	Measure	beta	Exponentiated beta
First-ever acute subarachnoid haemorrhage with CSMR	LDI (I$ per capita)	Excess mortality rate	-0.3 (-0.49 to -0.11)	0.74 (0.61 to 0.90)

1.5 Data sources and types

GBD 2021 synthesises a large and growing number of data input sources including surveys, censuses, vital statistics, and other health-related data sources. The data from these sources are used to estimate morbidity; illness and injury; and attributable risk for 204 countries and territories from 1990 to 2021. Mortality deaths are estimated from 1980 to 2021. The input sources are accessible through an interactive citation tool available in the GHDx. Citations for specific GBD components, causes and risks, and locations can be found through the Data Input Sources Tool in GHDx: http://ghdx.healthdata.org/gbd-2021/data-input-sources. This tool allows users to view and access GHDx records for input sources and export a comma-separated value (CSV) file that includes metadata, citations, and information about where the data were used in GBD. As required by GATHER, additional metadata for input sources are available through the citation tool as well.

The (Causes of Death) CoD database contains seven types of data sources (table S4): vital registration (VR), verbal autopsy (VA), cancer registry, police records, sibling history, surveillance, survey/census, and minimally invasive tissue sample (MITS) diagnoses. In countries with complete VR systems, there is no need to use any other data source. Less than half the world’s population has deaths captured in a VR system, therefore for countries with incomplete VR systems, vital statistics for causes of death may be Appendixed with other data types.

All-cause mortality rates are estimated from vital registration data in countries with complete coverage. For other countries, the probabilities of death before age 5 and between ages 15 and 60 are estimated from censuses and surveys asking mothers to provide a history of children ever born and those still alive, and surveys asking adults about siblings who are alive or have passed away. Using model life tables, these probabilities of death are transformed into age-specific death rates by location, year, and sex. The GBD has collated a large database of cause of death data from vital registrations and verbal autopsy surveys in which relatives are asked a standard set of questions to ascertain the likely cause of death, Appendixed with police and mortuary data for injury deaths in countries with no other data. For countries with vital registration data, the completeness is assessed with demographic methods based on comparing recorded deaths with population counts between two successive censuses. The cause of death information is provided in a large number of different classification systems based on versions of the International

Classification of Diseases or bespoke classifications in some countries. All data are mapped into the disease and injury categories of GBD. All classification systems contain codes that are less informative because they lack a specific diagnosis (e.g., unspecified cancer) or refer to codes that cannot be underlying cause of death (e.g., low back pain or senility) or are intermediate causes (e.g., heart failure or sepsis). Such deaths are redistributed to more precise underlying causes of death.[4] After these redistributions and corrections for under-registration, the data are analysed in CODEm (Cause Of Death Ensemble model), a highly systematised tool that runs many different models on the same data and chooses an ensemble of models that best reflects all the available input data. Models are chosen with variations in the statistical approach ("mixed effects" of spatiotemporal Gaussian Process Regression), in the unit of analysis (rates or cause fractions), and the choice of predictive covariates. The statistical performance of all models is tested by holding out 30% of the data and checking how well a model covers the data that were held out. To enforce consistency from CODEm, the sum of all cause-specific mortality rates is scaled to that of the all-cause mortality rates in each age, sex, location, and year category.

Non-fatal estimates are based on systematic reviews of published papers and unpublished documents, survey microdata, administrative records of health encounters, registries, and disease surveillance systems. Our Global Health Data Exchange (GHDx, http://ghdx.healthdata.org/) is the largest repository of health data globally. We first set a reference case definition and/or study method that best quantifies each disease or injury or consequence thereof. If there is evidence of a systematic bias in data that used different case definitions or methods compared to reference data, we adjust those data points to reflect what its value would have been if measured as the reference. This is a necessary step if one wants to use all data pertaining to a particular quantity of interest rather than choosing a small subset of data of the highest quality only. DisMod-MR 2.1, a Bayesian meta-regression tool, is our main method of analysing non-fatal data. It is designed as a geographical cascade where a first model is run on all the world's data, which produces an initial global fit and estimates coefficients for predictor variables and the adjustments for alternative study characteristics. The global fit adjusted by the values of random effects for each of seven GBD super-regions, the coefficients on sex and country predictors, are passed down as data to a model for each super-region together with the input data for that geography. The same steps are repeated going from super-region to 21 region fits and then to 195 fits by country and where applicable a further level down to subnational units. Below the global fit, all models are run separately by sex and for six time periods: 1990, 1995, 2000, 2005, 2010, and 2016. During each fit all data on prevalence, incidence, remission (i.e., cure rate) and mortality are forced to be internally consistent. For most diseases, the bulk of data on prevalence or incidence is at the disease level with fewer studies providing data on the proportions of cases of disease in each of the sequelae defined for the disease. The proportions in each sequela are pooled using DisMod-MR 2.1 or meta-analysis or derived from analyses of patient-level datasets. The multiplication of prevalent cases for each disease sequela and the appropriate disability weight produces YLD estimates that do not yet take into account comorbidity. To correct for comorbidity, these data are used in a simulation to create hypothetical individuals in each age, sex, location, and year combination who experience no, one, or multiple sequelae simultaneously. We assume that disability weights are multiplicative rather than additive as this avoids assigning a combined disability weight value in any individual to exceed 1, i.e., be worse than a "year lost due to death". This comorbidity adjustment leads to an average scaling down of disease- specific YLDs ranging from about 2% in young children up to 17% in oldest ages.

All our estimates of causes of death are categorical: each death is assigned to a single underlying cause. This has the attractive property that all estimates add to 100%. For risks, we use a different, "counterfactual" approach, i.e., answering the question: "what would the burden have been if

the population had been exposed to a theoretical minimum level of exposure to a risk". Thus, we need to define what level of exposure to a risk factor leads to the lowest amount of disease. We then analyse data on the prevalence of exposure to a risk and derive relative risks for any risk-outcome pair for which we find sufficient evidence of a causal relationship. Prevalence of exposure is estimated in DisMod-MR 2.1, using spatiotemporal Gaussian Process Regression, or from satellite imagery in the case of ambient air pollution. Relative risk data are pooled using meta-analysis of cohort, case-control and/or intervention studies. For each risk and outcome pair, we evaluate the evidence and judge if the evidence falls into the categories of "convincing" or "probable" as defined by the World Cancer Research Fund.[5] From the prevalence and relative risk results, population attributable fractions are estimated relative to the theoretical minimum risk exposure level (TMREL). When we aggregate estimates for clusters of risks, e.g., metabolic or behavioural risks, we use a multiplicative function rather than simple addition and take into account how much of each risk is mediated through another risk. For instance, some of the risk of high Body Mass Index is influence stroke outcome but much of its impact is mediated through high blood pressure, high cholesterol, or high fasting plasma glucose, and we would not want to double count the mediated effects when we estimate aggregates across risk factors.

We included inpatient hospital data, adjusted for readmission and any diagnosis using correction factors estimated from US claims data. We excluded data for locations where the data points were implausibly low (Vietnam, Philippines, India). In addition, we included unpublished stroke registry data for acute ischaemic and acute haemorrhagic strokes. We also included survey data for chronic cerebrovascular disease. These surveys were identified based on expert opinion and review of major survey series focused on world health that included questions regarding self-reported history of stroke.

As with many models in GBD, the diversity of data sources available means that we needed to adjust available data to our preferred or reference case definition (2). For the first ever acute stroke models we used DisMod to estimate the statistical association between measurements taken using different case definitions and then used these estimates to adjust the non-referent datapoints. We included study-level covariates to adjust data points for first and recurrent strokes combined, using data for first strokes only as reference. We also included study-level covariates to adjust ischaemic and haemorrhagic strokes combined (all stroke), using as reference studies with subtype-specific information.

Uncertainty is propagated throughout all these calculations by creating 1,000 values for each prevalence, death, YLL, YLD, or DALY estimate and performing aggregations across causes and locations at the level of each of the 1,000 values for all intermediate steps in the calculation. The lower and upper bounds of the 95% uncertainty interval are the 25th and 975th values of the ordered 1,000 values. For all age-standardised rates, GBD uses a standard population calculated as the non-weighted average across all countries of the percentage of the population in each five-year age group for the years 2010 to 2035 from the United Nations Population Division's World Population Prospects (2012 revision).

1.6 Stroke definition

Stroke was defined according to WHO criteria – rapidly developing clinical signs of focal (at times global) disturbance of cerebral function lasting more than 24 hours or leading to death with no apparent cause other than that of vascular origin.[9] Data on transient ischaemic attack (TIA) were not included.

Acute stroke: Stroke cases are considered acute from the day of incidence of a first-ever stroke through day 28 following the event.

Chronic stroke: Stroke cases are considered chronic beginning 28 days following the occurrence of an event. Chronic stroke includes the sequelae of an acute stroke AND all recurrent stroke events. GBD 2021 adopts this broader definition of chronic stroke than was used in prior iterations in order to model acute strokes using only first-ever incident events.

Ischaemic stroke: Incident ischaemic stroke is defined as the occurrence of first-ever ischaemic stroke, based on clinical diagnosis by a physician using diagnostic imaging. Ischaemic strokes are considered to include all vascular events leading to limited blood flow to brain tissue, with resulting infarction, including atherosclerotic and thromboembolic strokes but excluding strokes in which the underlying cause is intracranial haemorrhage.

Haemorrhagic or other strokes: This cause includes all non-ischaemic strokes of a vascular cause including subarachnoid and stroke due to intracranial haemorrhage. Intracerebral haemorrhage (ICH) was defined as stroke with a focal collection of blood in the brain not due to trauma. Subarachnoid haemorrhage (SAH) was defined as non-traumatic stroke due to bleeding into the subarachnoid space of the brain. ICD codes used for inclusion of hospital and claims data can be found in Appendix Table 2.

1.7 Modelling strategy

In GBD 2021, stroke incidence is reported as the instantaneous hazard of first-ever stroke while deaths are per capita and per year, with the employment of two separate models, an "acute" model of 30-day mortality and a "chronic" model of survivors beyond 30 days, based on the preponderance of available data. Thus, the incidence rate represents new events in a given year while the death rate represents those that occurred in that year regardless of when the stroke occurred. Therefore, while uncommon, in some cases death rates can be equal or even greater than incidence rates. Imprecision in this particular ratio of acute to long-term survival (which is estimated separately by year, age, sex, and location) will impact the relationship between incidence and deaths in a given year. There is limited literature on the proportion of deaths occurring within 30-days vs 1-year or longer that would be a perfect comparison with GBD, but some studies we have seen suggest that our estimated proportions may be too low, especially for older ages.[10-12] We intend to reassess this for GBD 2021.

For GBD 2021, we used a standard CODEm approach to model deaths from stroke. The covariates included in the ensemble modelling process are listed in the table below. For GBD 2021, adjusted dietary covariates for consumption of fruits, omega-3 fatty acids, vegetables, nuts and seeds, and polyunsaturated fatty acids (PUFA) were replaced with the summary exposure value scalars for diet low in each of these factors. The direction for each dietary covariate was changed from -1 to 1 to as our *a priori* assumption is that low levels of intake of these dietary factors are associated with increasing mortality risk from stroke. We dropped the dietary covariate for whole grains (kcal/capita, adjusted) and the socio-demographic index covariate as exploratory analyses indicated that these variables were not predictive of stroke mortality. In addition, we changed the alcohol consumption covariate from 0 to 1 to reflect the expected direction of the association for this risk factor with stroke mortality. Apart from these covariate changes, there are no substantive changes from the approach used in GBD 2017.

Table: Selected covariates for CODEm models, stroke

Covariate	Transformation	Level	Direction

Summary exposure variable, stroke	None	1	1
Cholesterol (total, mean per capita)	None	1	1
Smoking prevalence	None	1	1
Systolic blood pressure (mmHg)	None	1	1
Mean BMI	None	2	1
Elevation over 1,500m (proportion)	None	2	-1
Fasting plasma glucose	None	2	1
Outdoor pollution ($PM_{2.5}$)	None	2	1
Indoor air pollution	None	2	1
Healthcare Access and Quality Index	None	2	-1
Lag distributed income per capita (I$)	Log	3	-1
Summary exposure value, omega-3	None	3	1
Summary exposure value, fruits	None	3	1
Summary exposure value, vegetables	None	3	1
Summary exposure value, nuts and seeds	None	3	1
Pulses/legumes (kcal/capita, unadjusted)	None	3	-1
Summary exposure value, PUFA adjusted (percent)	None	3	1
Alcohol (litres per capita)	None	3	1
Trans fatty acid	None	3	1

1.7.1 Ischaemic stroke

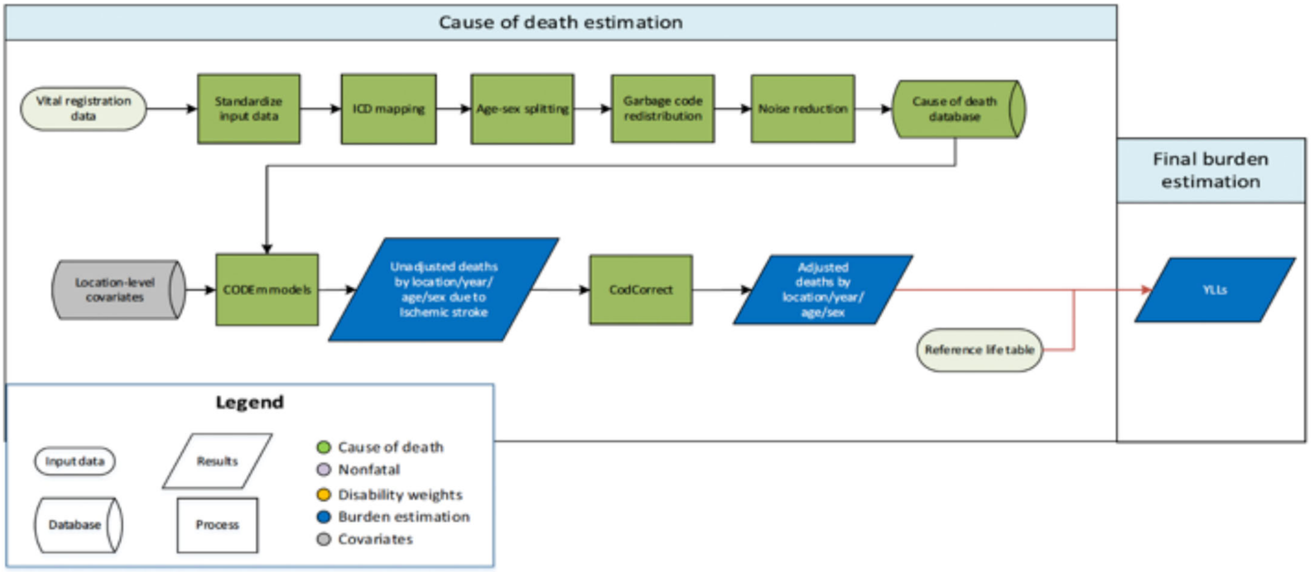

1.7.1.1 Input data

Vital registration data were used to model deaths from ischaemic stroke. We outliered ICD8 data points which were inconsistent with the rest of the data and created implausible time trends. We also outliered ICD10 data points in The Republic of Tajikistan due to unstable and implausible estimates in similar age groups.

1.7.1.2 Modelling strategy

We used a standard CODEm approach to model deaths from ischemic stroke. For GBD 2021, adjusted dietary covariates for consumption of fruits, omega-3 fatty acids, vegetables, nuts and seeds, and polyunsaturated fatty acids were replaced with the summary exposure value scalars for diet low in each of these factors. The direction for each dietary covariate was changed from -1 to 1 to as our *a priori* assumption is that low levels of intake of these dietary factors are associated with increasing mortality risk from ischaemic stroke. In addition, the dietary covariate for whole grains (kcal/capita, adjusted) and the socio-demographic index covariate were dropped as exploratory analyses indicated that the covariates were not predictive of the outcome. In addition, we changed alcohol variable from 0 to 1 to reflect our *a priori* hypothesis about the expected direction of the association between this risk factor and mortality risk of ischaemic stroke. We also changed the level of the trans fatty acid covariate from 1 to 3. Besides these covariate changes, there are no other substantive changes from the approach used in GBD 2017.

Table: Selected covariates for CODEm models, ischaemic stroke

Covariate	Transformation	Level	Direction
Summary exposure value, ischaemic stroke	None	1	1
Cholesterol (total, mean per capita)	None	1	1
Smoking prevalence	None	1	1
Systolic blood pressure (mmHg)	None	1	1
Mean BMI	None	2	1
Elevation over 1500m (proportion)	None	2	-1
Fasting plasma glucose	None	2	1
Outdoor pollution ($PM_{2.5}$)	None	2	1
Indoor air pollution	None	2	1
Healthcare access and quality index	None	2	-1
Lag distributed income per capita (I$)	Log	3	-1
Summary exposure value, omega-3	None	3	1
Summary exposure value, fruits	None	3	1
Summary exposure value, vegetables	None	3	1
Summary exposure value, nuts and seeds	None	3	1
Pulses/legumes (kcal/capita, unadjusted)	None	3	-1
Summary exposure value PUFA adjusted	None	3	1
Alcohol (litres per capita)	None	3	1
Trans fatty acid	None	3	1

1.7.2 Intracerebral haemorrhage

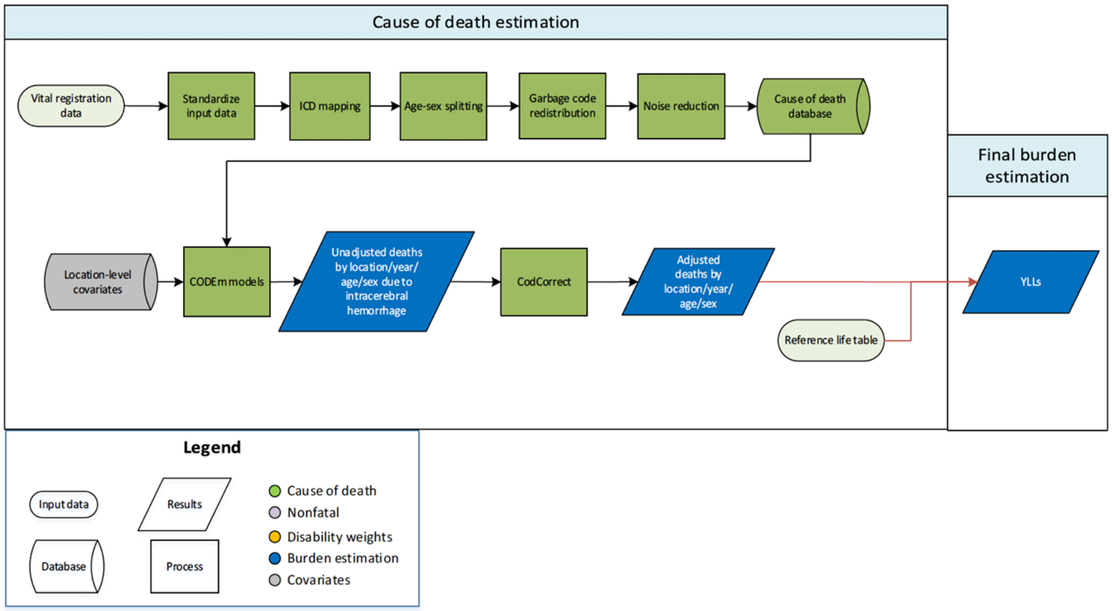

1.7.2.1 Input data

Vital registration data were used to model intracerebral haemorrhage. We outliered ICD8 data points which were inconsistent with the rest of the data and created implausible time trends. In addition, we outliered vital registration data points in certain countries in Latin American countries due to implausibly high values at the oldest age groups resulting in inconsistencies in time trends.

1.7.2.2 Modelling strategy

We used a standard CODEm approach to model deaths from intracerebral haemorrhage. For GBD 2021, adjusted dietary covariates for consumption of fruits, omega-3 fatty acids, vegetables, nuts and seeds, and polyunsaturated fatty acids were replaced with the summary exposure value scalars for diet low in each of these factors. The direction for each dietary covariate was changed from -1 to 1 to as our *a priori* assumption is that low levels of intake of these dietary factors are associated with increasing mortality risk from intracerebral haemorrhage. In addition, the dietary covariate for whole grains (kcal/capita, adjusted) and the social demographic index covariate were dropped as exploratory analyses indicated that these covariates were not predictive of the mortality risk from intracerebral haemorrhage. We changed the direction of the covariate for alcohol from 0 to 1 due to our *a priori* hypothesis about the direction of the association for this covariate. We also changed the level of the cholesterol covariate from 1 to 3 and the direction from 0 to -1 to reflect the mixed and inconclusive evidence regarding cholesterol levels and risk of intracerebral haemorrhage. In addition, we changed the level of the trans fatty acid from covariate from 1 to 3 in accordance with the expected importance of this risk factor on mortality from intracerebral haemorrhage. Besides these covariate changes, there are no other substantive changes from the approach used in GBD 2017.

1.7.3 Subarachnoid haemorrhage

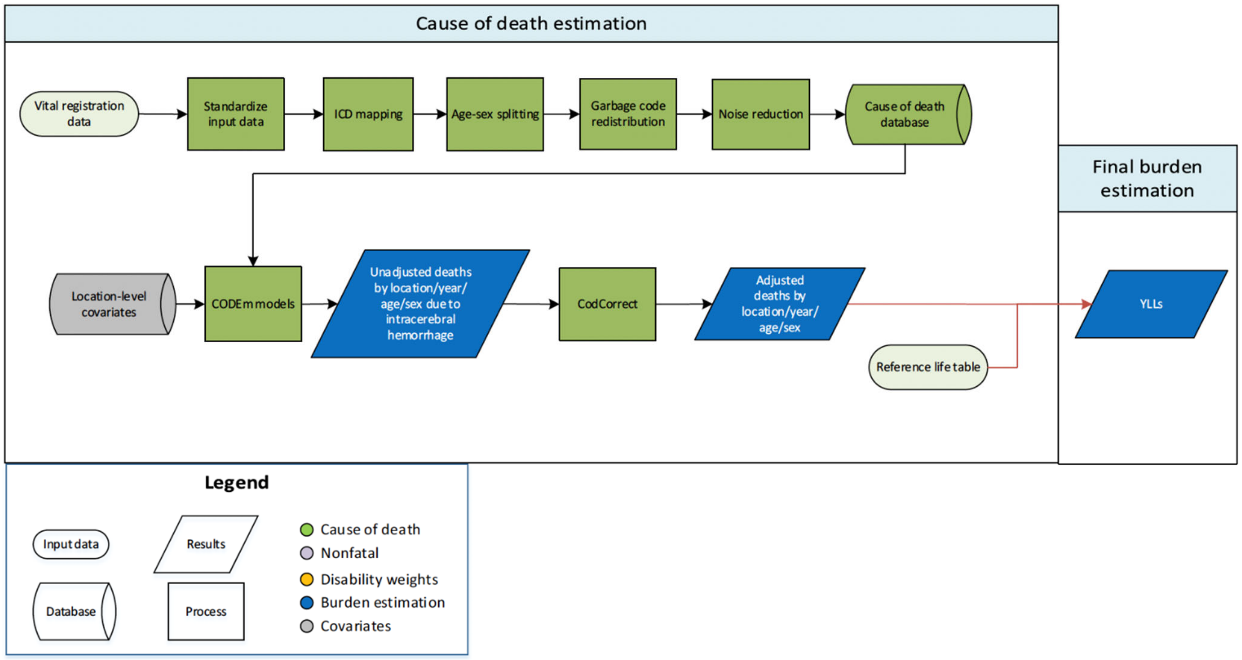

1.7.3.1 Input data

Vital registration data were used to model subarachnoid haemorrhage. We outliered ICD8 datapoints which were inconsistent with the rest of the data and created implausible time trends. In addition, we outliered vital registration data in Tibet that was implausibly high for all years and age groups.

1.7.3.2 Modelling strategy

We used a standard CODEm approach to model deaths from subarachnoid haemorrhage. The covariates chosen for inclusion in the ensemble modelling process are listed in the table below. For GBD 2021, we dropped the Socio-demographic Index covariate as exploratory analyses indicated that it was not predictive of the outcome. We also changed the alcohol covariate from 0 to 1 to reflect the expected direction of the association of this risk factor with mortality risk. Apart from these changes to the covariates, there are no substantive changes from the approach used in GBD 2017.

Table: Selected covariates for CODEm models, subarachnoid haemorrhage

Level	Covariate	Transformation	Direction
1	Smoking prevalence	None	1
1	Systolic blood pressure (mmHg)	None	1
2	Healthcare access and quality index	None	-1
3	Lag distributed income per capita (I$)	Log	-1
3	Alcohol (litres per capita)	None	1

1.8 Methods tables

1.8.1 Table 1. ICD Codes used in fatal and non-fatal analysis.

Fatal analysis	ICD10	ICD9
Cerebrovascular disease	G45-G46.8, I60-I63.9, I65-I66.9, I67.0-I67.3, I67.5-I67.6, I68.1-I68.2, I69.0-I69.3	430-435.9, 437.0-437.2, 437.5-437.8
Ischaemic stroke	G45-G46.8, I63-I63.9, I65-I66.9, I67.2-I67.3, I67.5-I67.6, I69.3	433-435.9, 437.0-437.1, 437.5-437.8
Haemorrhagic stroke	I60-I62.9, I67.0-I67.1, I68.1- I68.2, I69.0-I69.2	430-432.9, 437.2

Nonfatal analysis	ICD10	ICD9
Cerebrovascular disease	I60-I63.9, I65-I66.9, I67.0-I67.3, I67.5-I67.6, I68.1-I68.2, I69.0- I69.3	430-434.9, 437.0-437.2, 437.5-437.8
Ischaemic stroke	I63-I63.9, I65-I66.9, I67.2-I67.3, I67.5-I67.6, I69.3	433-434.9, 437.0-437.1, 437.5-437.8
Haemorrhagic stroke	I60-I62.9, I67.0-I67.1, I68.1- I68.2, I69.0-I69.2	430-432.9, 437.2

1.8.2 Table 2. Selected covariates for CODEm models, overall stroke and subtypes

CODEm is an analytical tool which explores a large variety of possible models to estimate trends in causes of death. Possible models are identified using a covariate selection algorithm that yields many plausible combinations of covariates, which are then run through four model classes. The model classes include mixed effects linear models and spatial-temporal Gaussian Process Regression models for cause fractions and death rates. All models for each cause of death are then assessed using out-of-sample predictive validity and combined into an ensemble with optimal out-of-sample predictive performance. Ensemble models for cause of death estimation outperform any single component model in tests of root mean square error, frequency of predicting correct temporal trends, and achieving 95% coverage of the prediction interval.[13]

Covariate	Level	Direction, Stroke	Direction, Ischaemic stroke	Direction, Haemorrhagic stroke
Summary exposure variable	1	+	+	+
Cholesterol (total, mean per capita)	1	+	+	0
Smoking prevalence	1	+	+	+
Systolic blood pressure (mmHg)	1	+	+	+

Trans fatty acid	1	+	+	+
Mean BMI	2	+	+	+
Elevation over 1500m (proportion)	2	-	-	-
Fasting plasma glucose	2	+	+	+
Outdoor pollution ($PM_{2.5}$)	2	+	+	+
Indoor air pollution	2	+	+	+
Healthcare access and quality index	2	-	-	-
Lag distributed income per capita (I$)*	3	-	-	-
Socio-demographic Index	3	0	0	0
Omega-3 (kcal/capita, adjusted) *	3	-	-	-
Fruits (kcal/capita, adjusted)	3	-	-	-
Vegetables (kcal/capita, adjusted)	3	-	-	-
Nuts and seeds (kcal/capita, adjusted)	3	-	-	-
Whole grains (kcal/capita, adjusted)	3	-	-	-
Pulses/legumes (kcal/capita, adjusted)	3	-	-	-
PUFA adjusted (percent)	3	-	-	-
Alcohol (litres per capita)	3	0	0	0

*Variables were log-transformed

1.8.3 Table 3. Counts of data used by measure and stroke model for GBD 2021

First-ever acute ischaemic stroke:

Region Name	Incidence	Prevalence	Remission	Mortality	Hospital Claims
East Asia	5	0	0	0	1
Southeast Asia	0	0	0	0	1
Oceania	0	0	0	0	0
Central Asia	1	0	0	0	1
Central Europe	3	0	0	2	14
Eastern Europe	5	0	0	1	5
High-income Asia Pacific	4	0	0	0	0
Australasia	8	0	0	0	4
Western Europe	27	0	0	6	31
Southern Latin America	2	0	0	1	0
High-income North America	1	0	0	0	5
Caribbean	0	0	0	0	0
Andean Latin America	0	0	0	0	0
Central Latin America	0	0	0	0	0
Tropical Latin America	2	0	0	0	0

North Africa and Middle East	9	0	0	7	2
South Asia	4	0	0	4	0
Central Sub-Saharan Africa	0	0	0	0	0
Eastern Sub-Saharan Africa	1	0	0	0	0
Southern Sub-Saharan Africa	1	0	0	0	0
Western Sub-Saharan Africa	0	0	0	0	0
Total	73	0	0	21	64

First-ever acute haemorrhagic stroke:

Region Name	Incidence	Prevalence	Remission	Mortality	Hospital Claims
East Asia	5	0	0	0	1
Southeast Asia	0	0	0	0	1
Oceania	0	0	0	0	0
Central Asia	1	0	0	1	1
Central Europe	3	0	0	1	14
Eastern Europe	5	0	0	2	5
High-income Asia Pacific	3	0	0	0	0
Australasia	9	0	0	3	4
Western Europe	29	0	0	17	31
Southern Latin America	2	0	0	2	0
High-income North America	2	0	0	0	5
Caribbean	0	0	0	0	0
Andean Latin America	0	0	0	0	0
Central Latin America	0	0	0	0	0
Tropical Latin America	0	0	0	0	0
North Africa and Middle East	8	0	0	13	2
South Asia	4	0	0	6	0
Central Sub-Saharan Africa	0	0	0	0	0
Eastern Sub-Saharan Africa	1	0	0	1	0
Southern Sub-Saharan Africa	1	0	0	0	0
Western Sub-Saharan Africa	0	0	0	0	0
Total	73	0	0	46	64

Chronic ischaemic stroke:

Region Name	Incidence	Prevalence	Remission	Mortality	Hospital Claims
East Asia	0	4	0	0	0
Southeast Asia	0	3	0	0	0
Oceania	0	0	0	0	0
Central Asia	0	0	0	0	0

Central Europe	0	0	0	1	0
Eastern Europe	0	0	0	0	0
High-income Asia Pacific	0	2	0	0	0
Australasia	0	1	0	2	0
Western Europe	0	42	0	7	0
Southern Latin America	0	1	0	0	0
High-income North America	0	39	0	0	0
Caribbean	0	2	0	0	0
Andean Latin America	0	1	0	0	0
Central Latin America	0	4	0	0	0
Tropical Latin America	0	1	0	0	0
North Africa and Middle East	0	2	0	0	0
South Asia	0	11	0	0	0
Central Sub-Saharan Africa	0	0	0	0	0
Eastern Sub-Saharan Africa	0	2	0	0	0
Southern Sub-Saharan Africa	0	0	0	0	0
Western Sub-Saharan Africa	0	4	0	0	0
Total	0	119	0	10	0

Chronic haemorrhagic stroke:

Region Name	Incidence	Prevalence	Remission	Mortality	Hospital Claims
East Asia	0	4	0	0	0
Southeast Asia	0	3	0	0	0
Oceania	0	0	0	0	0
Central Asia	0	0	0	0	0
Central Europe	0	0	0	1	0
Eastern Europe	0	0	0	0	0
High-income Asia Pacific	0	2	0	0	0
Australasia	0	1	0	2	0
Western Europe	0	42	0	7	0
Southern Latin America	0	1	0	0	0
High-income North America	0	39	0	0	0
Caribbean	0	2	0	0	0
Andean Latin America	0	1	0	0	0
Central Latin America	0	4	0	0	0
Tropical Latin America	0	1	0	0	0
North Africa and Middle East	0	2	0	0	0
South Asia	0	10	0	0	0
Central Sub-Saharan Africa	0	0	0	0	0
Eastern Sub-Saharan Africa	0	2	0	0	0
Southern Sub-Saharan Africa	0	0	0	0	0
Western Sub-Saharan Africa	0	4	0	0	0
Total	0	118	0	10	0

1.8.4 Table 4. DisMod covariates

Step 1:

Cause	Variable name	Measure	beta	Exponentiated beta
Chronic ischaemic stroke	Log-transformed SEV scalar: Isch Stroke	Prevalence	0.83 (0.75 — 1.03)	2.29 (2.12 — 2.80)
Chronic ischaemic stroke	LDI (I$ per capita)	Excess mortality rate	-0.16 (-0.29 — -0.1)	0.85 (0.75 — 0.90)
Chronic haemorrhagic stroke	Log-transformed SEV scalar: Hem Stroke	Prevalence	0.79 (0.75 — 0.92)	2.21 (2.12 — 2.50)
Chronic haemorrhagic stroke	LDI (I$ per capita)	Excess mortality rate	-0.12 (-0.16 — -0.1)	0.89 (0.85 — 0.90)
First ever acute haemorrhagic stroke	Hospital data	Incidence	0.54 (0.54 – 0.54)	1.71 (1.71 – 1.72)
First ever acute haemorrhagic stroke	Any stroke	Incidence	1.27 (1.27 – 1.28)	3.57 (3.56 – 3.59)
First ever acute haemorrhagic stroke	First-ever acute stroke, ischaemic or hemorrhagic	Incidence	0.52 (0.52 – 0.53)	1.69 (1.68 – 1.71)
First ever acute haemorrhagic stroke	Log-transformed age-standardised SEV scalar: hemorrhagic stroke	Incidence	0.77 (0.75 – 0.82)	2.17 (2.12 – 2.27)
First ever acute haemorrhagic stroke	Any stroke	Excess mortality rate	-0.48 (-0.66 – -0.32)	0.62 (0.52 – 0.73)
First ever acute haemorrhagic stroke	First-ever acute stroke, ischaemic or hemorrhagic	Excess mortality rate	-0.081 (-0.3 – 0.16)	0.62 (0.52 – 0.73)
First ever acute ischaemic stroke	Hospital data	Incidence	0.38 (0.37 – 0.38)	1.46 (1.45 – 1.46)
First ever acute ischaemic stroke	Any stroke	Incidence	0.31 (0.29 – 0.33)	1.37 (1.34 – 1.39)
First ever acute ischaemic stroke	First-ever acute stroke, ischaemic or hemorrhagic	Incidence	0.37 (0.36 – 0.38)	1.44 (1.43 – 1.46)
First ever acute ischaemic stroke	Log-transformed age-standardised SEV scalar: ischaemic stroke	Incidence	1.16 (1.09 – 1.22)	3.21 (2.99 – 3.39)

Step 2:

Cause	Variable name	Measure	beta	Exponentiated beta
Chronic ischaemic stroke with CSMR	Log-transformed SEV scalar: Ischaemic stroke	Prevalence	0.89 (0.75 – 1.19)	2.44 (2.13 – 3.27)
Chronic ischaemic stroke with CSMR	LDI (I$ per capita)	Excess mortality rate	-0.49 (-0.5 – - 0.46)	0.61 (0.61 – 0.63)
Chronic haemorrhagic stroke with CSMR	Log-transformed SEV scalar: haemorrhagic stroke	Prevalence	0.88 (0.75 – 1.15)	2.40 (2.13 – 3.17)
Chronic haemorrhagic stroke with CSMR	LDI (I$ per capita)	Excess mortality rate	-0.48 (-0.5 – - 0.44)	0.62 (0.61 -0.64)
First-ever acute haemorrhagic stroke with CSMR	Any stroke	Incidence	1.27 (1.27 – 1.29)	3.58 (3.56 – 3.62)
First-ever acute haemorrhagic stroke with CSMR	First-ever acute stroke, ischaemic or haemorrhagic	Incidence	0.52 (0.52 – 0.54)	1.69 (1.68 – 1.71)
First-ever acute haemorrhagic stroke with CSMR	Log-transformed SEV scalar: Hem stroke	Incidence	1.11 (1.01 – 1.20)	3.03 (2.74 – 3.33)
First-ever acute haemorrhagic stroke with CSMR	Any stroke	Excess mortality rate	-0.37 (-0.49 – -0.27)	0.69 (0.62 – 0.77)
First-ever acute haemorrhagic stroke with CSMR	First-ever acute stroke, ischaemic or haemorrhagic	Excess mortality rate	0.023 (-0.2 –0.23)	1.02 (0.82 – 1.25)
First-ever acute ischaemic stroke with CSMR	Any stroke	Incidence	0.32 (0.30 – 0.33)	1.38 (1.35 – 1.39)
First-ever acute ischaemic stroke with CSMR	First-ever acute stroke, ischaemic or haemorrhagic	Incidence	0.37 (0.36 – 0.38)	1.44 (1.43 – 1.46)
First-ever acute ischaemic stroke with CSMR	Log-transformed age-standardized SEV scalar: Ischaemic stroke	Incidence	1.11 (1.05 – 1.18)	3.04 (2.86 – 3.26)
First-ever acute ischaemic stroke with CSMR	Any stroke	Excess mortality rate	-0.34 (-0.45 – -0.24)	0.71 (0.64 – 0.79)
First-ever acute ischaemic stroke with CSMR	First-ever acute stroke, ischaemic or haemorrhagic	Excess mortality rate	-0.69 (-0.82 – -0.56)	0.51 (0.44 –0.57)

1.8.5 Table 5. Sequelae and disability weights for ischaemic stroke, intracerebral haemorrhage and subarachnoid haemorrhage

Sequela	Health state lay description	Disability weight
Asymptomatic chronic stroke		N/A
Acute and chronic stroke, severity level 1	Has some difficulty in moving around and some weakness in one hand, but is able to walk without help	0.019 (0.01 – 0.032)
Acute and chronic stroke, severity level 2	Has some difficulty in moving around and in using the hands for lifting and holding things, dressing and grooming	0.07 (0.046 – 0.099)
Acute and chronic stroke, severity level 3	Has some difficulty in moving around, in using the hands for lifting and holding things, dressing and grooming, and in speaking. The person is often forgetful and confused.	0.316 (0.205 – 0.438)
Acute and chronic stroke, severity level 4	Is confined to bed or a wheelchair, has difficulty speaking and depends on others for feeding, toileting and dressing.	0.552 (0.376 – 0.707)
Acute and chronic stroke, severity level 5	Is confined to bed or a wheelchair, depends on others for feeding, toileting and dressing, and has difficulty speaking, thinking clearly and remembering things.	0.588 (0.411 – 0.745)

Section 2. Summary of GBD methods for estimates of burden of risk factors

A complete set of risk-specific exposures, relative risks (RRs), theoretical minimum-risk exposure levels (TMRELs), and population attributable fractions (PAFs) were computed for the years 1990–2021. The current version of the data download tool is available in the GHDx and contains core summary results for the GBD 2021: http://ghdx.healthdata.org/gbd-results-tool. The core summary results include deaths, years of life lost (YLLs), years lived with disability (YLDs), disability-adjusted life-years (DALYs), and summary exposure values (SEVs). The GHDx includes data for causes, risks, cause-risk attribution, aetiologies, and impairments.

The GBD 2021 incorporated a large number and wide variety of input sources to estimate mortality, causes of death and illness, and risk factors for 204 countries and territories from 1990-2021. These input sources are accessible through an interactive citation tool available in the GHDx.

Users can retrieve citations for a specific GBD component, cause or risk, and location by choosing from the available selection boxes. They can then view and access GHDx records for input sources and export a comma-separated value (CSV) file that includes the GHDx metadata, citations, and information about where the data were used in GBD. Additional metadata for each input source are available through the citation tool as required by the GATHER statement. The citation tool is accessible through the GHDx at http://ghdx.healthdata.org/gbd-2021/data-input- sources.

We also assessed the following three clusters of risks: behavioural (smoking [including second-hand smoking], dietary [diet high in sodium, diet high in red meat, diet low in fruits, diet low in vegetables, diet low in whole grains, and alcohol use], low physical activity, environmental and occupational (ambient particulate matter [$PM_{2.5}$] pollution, household air pollution, suboptimal temperature, and lead exposure), and metabolic (high BMI, high fasting plasma glucose, high SBP, high LDL cholesterol, kidney dysfunction). Tobacco smoke (smoking and second-hand smoking) was considered as a separate cluster but was also included in the behavioural cluster.

2.1 Definition of risk factors and their estimation

As explained in detail elsewhere,[2, 7] ambient $PM_{2.5}$ pollution was defined as an annual average daily exposure to outdoor air concentrations of $PM_{2.5}$ more than 8.8 μg/m^3. Household air pollution was defined as any exposure to indoor concentration of $PM_{2.5}$. Lead exposure was defined as blood lead concentration of more than 20 μg/L. Diet high in sodium was defined as consumption of sodium more than 5 g/day. Diet low in fruits was defined as consumption of less than 200 g/day. Diet low in vegetables was defined as consumption of less than 350 g/day. Diet low in whole grains was defined as consumption of less than 100 g/day. Alcohol use was defined as any amount. Low physical activity was defined as average weekly work, home, transport-related, and recreational physical activity of less than 8000 metabolic equivalent of task-min. Smoking was defined as any previous or current tobacco smoking. Second-hand smoking was defined as daily indoor exposure to second-hand smoking. High body-mass index (BMI) was defined as body-mass index greater than 23.0 kg/m^2. High fasting plasma glucose was defined as serum fasting plasma glucose greater than 5.4 mmol/L. High systolic blood pressure (SBP) was defined as systolic blood pressure greater than 110-115 mm Hg. High (low density lipoprotein) LDL cholesterol was estimated in units of mmol/L (we used a TMREL with a uniform distribution between 0·7 and 1·3 mmol/L). Low glomerular filtration rate was defined as glomerular filtration rate less than 60 mL/min per 1.73m^2 (excluding end-stage renal disease). Theoretical minimum risk exposure levels were described elsewhere.[7, 14] The GBD risk factor list continues to evolve to reflect the policy relevance, public health, and medical care importance of major risk factors. Three risks were added to the list for GBD 2021: non-optimal temperature, high temperature, and low temperature.

The GBD 19 analytical method for estimating PAF had six analytical steps. (1) We included 19 risk–outcome pairs that met criteria for convincing or probable evidence on the basis of research studies. (2) Relative risks were estimated as a function of exposure based on published systematic reviews, GBD review and meta-regression. (3) Levels of exposure in each age-sex-location-year included in the study were estimated based on all available data sources using spatiotemporal Gaussian process regression, DisMod-MR 2.1, a Bayesian meta-regression method, or alternative methods. (4) We determined, from published trials or cohort studies, the level of exposure associated with minimum risk, called the theoretical minimum risk exposure level. (5) Attributable DALYs were computed by multiplying PAFs by the relevant outcome quantity for each age sex-location-year. (6) PAFs and attributable burden for combinations of risk factors were estimated taking into account mediation of different risk factors through other risk factors.

Estimates of attributable burden for each risk–outcome pair were established by multiplying the relevant cause measure by the PAF. All estimates for risk groupings or all risk factors combined were generated via an aggregation process that accounts for the fact that the effect of one risk factor might be partly or completely mediated through the effect of another. This mediation analysis is informed by individual-level data from prospective cohort studies on the joint effects of combinations of risk factors.

The comparative risk assessment (CRA) conceptual framework was developed by Murray and Lopez,[15] who established a causal web of hierarchically organised risks or causes that contribute to health outcomes, which allows for quantification of risks or causes at any level in the framework. In GBD 2021, as in previous iterations of the GBD study, we evaluated a set of behavioural, environmental and occupational, and metabolic risks, in which risk-outcome pairs were included based on evidence rules. These risks were organised in four hierarchical Levels, where Level 1 represents the overarching categories (behavioural, environmental and occupational, and metabolic) nested within Level 1 risks; Level 2 contains both single risks and risk clusters (such as child and maternal malnutrition); Level 3 contains the disaggregated single risks from within Level 2 risk clusters (such as low birthweight and short gestation); and Level 4 details risks with the most granular disaggregation, such as for specific occupational carcinogens, the subcomponents of child growth failure (stunting, wasting, underweight), and suboptimal breastfeeding (discontinued and non-exclusive breastfeeding). At each level of risk, we evaluated whether risk combinations were additive, multiplicative, or shared common pathways for intervention. This approach allows the quantification of the proportion of risk-attributable burden shared with another risk or combination of risks and the measurement of potential overlaps between behavioural, environmental and occupational, and metabolic risks. We do provide some insights into the potential magnitude of distal social, cultural, and economic factors through an analysis of the relationship between risk exposures and development measured by using the Socio-demographic Index (SDI).

Two types of risk assessments are possible within the CRA framework: attributable burden and avoidable burden. Attributable burden is the reduction in current disease burden that would have been possible if past population exposure had shifted to an alternative or counterfactual distribution of risk exposure. Avoidable burden is the potential reduction in future disease burden that could be achieved by changing the current distribution of exposure to a counterfactual distribution of exposure. Murray and Lopez identified four types of counterfactual exposure distributions: (1) theoretical minimum risk; (2) plausible minimum risk; (3) feasible minimum risk; and (4) cost-effective minimum risk.[16] The TMREL is the level of risk exposure that minimises risk at the population level or the level of risk that captures the maximum attributable burden. Other possible forms of risk quantification include plausible minimum risk – which reflects the distribution of risk that is conceivably possible and would minimise population-level risk if achieved – whereas feasible minimum risk describes the lowest risk distribution that has been attained within a population and cost-effective minimum risk is the lowest risk distribution for a population that can be attained in a cost-effective manner. Because no robust set of forecasts for all components of GBD is available, in this study we focus on quantifying attributable burden by using the theoretical minimum risk counterfactual distribution. According to the definition of avoidable burden, risk reversibility would be incorporated into this type of assessment because it would involve reducing risk to the counterfactual for the index year, given a history of past risk exposure.

Given the focus in this study on attributable burden, risk reversibility is not a criterion used in estimation here.

In general, this analysis follows the CRA methods used since GBD 2015.[17] The methods described here provide a high-level overview of the analytical logic and focus on areas of notable change from the methods employed in GBD 2015. Here we aim to provide sufficient detail on the methods and overall structure of the estimation process. This study complies with the GATHER recommendations proposed by the World Health Organization (WHO) and others, which include recommendations on documentation of data sources, estimation methods, and statistical analysis.[18]

2.2 Effect size estimation

2.2.1 Criteria for inclusion of risk-outcome pairs

Since GBD 2010 we have included risk-outcome pairs that we have assessed as meeting the World Cancer Research Fund (WCRF) grades of convincing or probable evidence.[19] In this framework, convincing evidence consists of biologically plausible associations between exposure and disease established from multiple epidemiological studies in different populations. Evidentiary studies must be substantial, include prospective observational studies, and, where relevant, randomised controlled trials (RCTs) of sufficient size, duration, and quality that show consistent effects. Probable evidence is similarly based on epidemiological studies with consistent associations between exposure and disease but for which shortcomings in the evidence exist, such as insufficient available trials (or prospective observational studies).

2.2.2 The World Cancer Research Fund grading system

2.2.2.1 Convincing evidence

Convincing evidence is evidence based on epidemiological studies showing consistent associations between exposure and disease and includes little or no evidence to the contrary. The available evidence is based on a substantial number of studies including prospective observational studies and, where relevant, RCTs of sufficient size, duration, and quality that show consistent effects. The association should be biologically plausible.

2.2.2.2 Probable evidence

Probable evidence is evidence based on epidemiological studies showing fairly consistent associations between exposure and disease, but for which perceived shortcomings in the available evidence exist or some evidence to the contrary precludes a more definite judgment. Shortcomings in the evidence may be any of the following: insufficient duration of trials (or studies); insufficient trials (or studies) available; inadequate sample sizes; or incomplete follow-up. Laboratory evidence is usually supportive. The association should be biologically plausible.

2.2.2.3 Possible evidence

Possible evidence is evidence based mainly on findings from case-control and cross-sectional studies. Insufficient RCTs, observational studies, or non-randomised controlled trials are available. Evidence based on non-epidemiological studies, such as clinical and laboratory investigations, is supportive. More trials are needed to support the tentative associations, which should be biologically plausible.

2.2.2.4 Insufficient evidence

Insufficient evidence is evidence based on findings of a few studies which are suggestive but

insufficient to establish an association between exposure and disease. Little or no evidence is available from RCTs. More well-designed research is needed to support the tentative association.

2.3 Determine relative risks

The relative risk (RR) by level of exposure or by cause for mortality or morbidity can be found in published and unpublished primary studies or in secondary studies that summarise RRs. In Step 1a of the analytical process (figure S1), we collated information from RCTs, cohort, pooled cohort, and case-control studies, and in Step 1b, used these data to determine the RR for the risk-outcome pairs included in GBD 2021 (table S4). For most risks, data from pooled cohorts or meta-analyses of cohorts were used; in the case of the risk of cataracts from household air pollution (HAP), cohort data were not available, and instead we used case-control data. We estimated RRs of mortality and morbidity for 67 risk factors for which we determined attributable burden by using RR and exposure. We incorporated RRs from studies that controlled for confounding but not for factors along the causal pathway between exposure and outcome. For risk-outcome pairs with evidence available for only one element of mortality or morbidity, we generally assumed that the estimated RRs applied equally to both. Given evidence of statistically different RRs for mortality and morbidity, we incorporated different RRs for each. We did not find that RRs were consistently higher or lower for mortality compared with morbidity. Details and citation information for the data sources used for RRs are provided in searchable form through a web- tool (http://ghdx.healthdata.org/). Available data sources for determining RRs varied across risks.

For the following risks estimated from a continuous exposure distribution in which the effect size was reported by categories in pooled or meta-analysis studies, we converted those categories to RR per unit increase in exposure and assumed a linear increase in the log of the RR and exposure: ambient ozone pollution, radon, lead, high fasting plasma glucose, high LDL cholesterol, high systolic blood pressure, high body-mass index, low bone mineral density. Many meta-analyses convert RRs to per unit increase for convenience, particularly when studies choose different categories that could not otherwise be compared. If samples in the primary studies at high levels of exposure were sufficient to inform the shape of the tail of the distribution, we applied a cap to the maximum RR by using the midpoint of the last category for which an RR was reported.

In GBD 2021, for a selected set of continuous risk factors, we modelled RRs using meta-regression—Bayesian, regularised, trimmed (MR-BRT), relaxing the log-linear assumption to allow for monotonically increasing or decreasing but non-linear functions using cubic splines. The MR-BRT programme is a set of wrappers customised for global health problems that use the open source mixed effects package (https://github.com/zhengp0/limetr).[20] Risk factors for which we undertook this re-analysis include: all dietary risk factors, low physical activity, kidney dysfunction, and air pollution. Because knot placement can affect the shape of the risk function when modelling with a cubic spline, we generated a wide range of knot placements and created an ensemble across these different knot placements. We also included in the final estimation 10% trimming of the data to avoid the results being sensitive to outliers.

For GBD 2021, we conducted systematic literature reviews for 18 risks. For other risk factors, only a small fraction of the existing data appears in the published literature, and other sources predominate, such as survey data and satellite data. Data were systematically screened from household surveys archived in the GHDx (http://ghdx.healthdata.org), including Demographic and Health Surveys, Multiple Indicator Cluster Surveys, Living Standards Measurement Surveys, and Reproductive Health Surveys. Other national health surveys were identified based on survey series that had yielded usable data for past rounds of GBD, sources suggested to us by in-country collaborators, and surveys identified in major multinational survey data catalogues, such as the International Household Survey Network and the WHO Central Data Catalogue, as well as through country Ministry of Health and

Central Statistical Office websites. Citations for all data sources used for risk factor estimation in GBD 2021 are provided in searchable form through a web-tool (http://ghdx.healthdata.org.

Information on systematic reviews were managed by using Research Electronic Data Capture (REDCap) electronic data capture tools hosted at the University of Washington.[21] REDCap is a secure, web-based application designed to support data capture for research studies that provides 1) an intuitive interface for validated data entry; 2) audit trails for tracking data manipulation and export procedures; 3) automated export procedures for seamless data downloads to common statistical packages; and 4) procedures for importing data from external sources.

2.4 Search terms and data preparation

Search terms for updates of systematic reviews for GBD 2021 are shown by risk factor in Appendix section 4 to: "GBD 2021 Risk Factors Collaborators. Global burden of 87 risk factors in 204 countries and territories, 1990-2021: a systematic analysis for the Global Burden of Disease Study 2021".[2]

Survey data constitutes a substantial part of the underlying data used in the estimation process. During extraction, we concentrated on demographic variables (such as location, gender, age), survey design variables (such as sampling strategy and sampling weights), and the variables used to define the population estimate (such a prevalence or a proportion) and a measure of uncertainty (standard error, confidence interval or sample size and number of cases).

Several adjustments were applied to extracted exposure sources to make the data more consistent and suitable for modelling. In GBD 2021, we implemented adjustments of risk exposure data to deal with alternative case definitions or study methods prior to entering data into our main analytical tools of DisMod-MR 2.1 and ST-GPR. This decision also included the adjustment of data presented for both sexes to a male and female equivalent. The starting point was to explicitly state the reference case definition and study method and identify alternative definitions and study characteristics that fall within our inclusion criteria. We compiled data from both within-study comparisons (i.e., data that used alternative and reference definitions in the same population) and between-study comparisons (i.e., data that used an alternative definition in one population and a reference definition in another population that overlap in location, time, age and sex) of different case definitions. For between-study comparisons, we allowed a maximum calendar year difference between studies of five years. Where validation studies (i.e., those carried out at the introduction of a new set of diagnostic criteria comparing to previous criteria) were available, we extracted data on the comparison of alternative to reference. For quantities of interest with multiple alternative definitions/methods we also looked for pairs comparing two alternatives. In a network analysis, if A is the reference and B and C are two alternatives, a comparison of A vs B and B vs C provides an indirect comparison of the alternative C against the reference A.

We pooled either the logit difference between alternative and reference or the natural log of the ratio of alternative to reference. From simulations we found that the two methods provide almost identical results for quantities that after adjustment do not exceed a value of 0.5 (e.g., prevalence or proportion). The logit difference method much better dealt with higher values and avoided prevalence or proportions to exceed one. If the values of either the reference or alternative were zero, we aggregated values across age groups until both values had non-zero observations. We used the delta method to compute the standard error of the reference and alternative measures in logit space. The standard error of the logit difference was computed as the square root of the sum of the variances of each data point in a pair.

2.5 Data analysis

We used a network random effects meta-regression in MR-BRT (see section 2.2.2). In a network

analysis, if A is the reference and B and C are two alternatives, a comparison of A vs B and B vs C provides an indirect comparison of the alternative C against the reference A. To implement the network, we included dummy variables with a particular structure. This was implemented as follows, where A is the reference definition/method:

- Create *k* dummy variables where *k* are all definitions/methods other than A (e.g., *k* = B, C)
- Code dummy *k* as
  - 1 if the first term of the logit difference is *k*;
  - -1 if k is second term of the logit difference;
  - 0 otherwise

For example:

Study	Comparison	DummyB	DummyC
1	logit(B)-logit(A)	1	0
2	logit(B)-logit(A)	1	0
3	logit(C)-logit(A)	0	1
4	logit(C)-logit(A)	0	1
5	logit(C)-logit(B)	-1	1
6	logit(C)-logit(B)	-1	1

The coding structure outlined above in step 1 assumes that all case definitions are mutually exclusive. In some cases, however, individual case definitions are a function of different components or dimensions. For example, case definitions may vary by the type of symptoms that a respondent experience as well as the recall period over which those symptoms are experienced. In the presence of sparse data, it may be difficult to find both direct and indirect comparisons of all individual case definitions. In this case, an alternative approach is to assume different dimensions of case definitions have a multiplicative effect. In other words, the effect of recall period has the same relative effect across different categories of symptoms reported by respondents. To implement this coding scheme:

- Create *k* dummy variable columns for each case definition dimension.
- For each dummy variable k:
  - Add 1 if *k* is a component of the first term in the logit difference.
  - Subtract 1 if *k* is a component of the second term in the logit difference.

In MR-BRT, we ran random effects meta-regression of the logit difference (or log ratio) with all the *k* dummy variables as covariates, omitting the intercept in the meta-regression. We used a study_id variable for be the unique combination of the NIDs of the reference and alternative studies (or alternative1 to alternative2). The coefficients on the *k* dummy variables represent the pooled logit difference of the *k* alternative definition to the reference taking into account evidence from both direct and indirect comparisons. In the example above, the coefficient on DummyA is the pooled logit difference of B minus A; $ference_k)) = \sqrt{var_k + \gamma^2}$

the coefficient on DummyB is the pooled logit difference of C minus A. The standard error of the pooled logit difference incorporating the between study variance was calculated as:

Where: $se(\text{logit}(difference_k)$= standard error of the pooled logit difference of alternative k to the reference

var_k=variance of the coefficient on dummy variable k

γ^2 = between-study variance

If both between and within study pairs were available, we examined whether there was a systematic difference between these. If there was a significant difference, we made a judgement call as to whether within-study or between study data comparisons were most appropriate. In general, this was the within-study data, however there were important measurement or conceptual reasons for choosing between-study data. For example, for crosswalks between self-reported height and weight compared to measured height and weight, between-study comparisons may be preferable if respondents knew they would be measured and, therefore, were less likely to misreport their height and weight.

We also examined whether there were systematic differences in the adjustments by key demographics (age, sex, geographic location, year) and other potential factors that may lead to variation in crosswalks. This could only be done at present in a direct comparison model and not in a network. We did this when there was a strong rationale, e.g., biological plausibility, for variation by such characteristics. After obtaining the pooled logit difference or log ratio estimates, we predicted adjustments based on the statistical model, including uncertainty in the adjustment and sampling error of each data point. For non-significant logit differences or log ratios we still applied the adjustments if there was a conceptual reason to believe that the alternative definition is biased. This expands the variance of these alternative definition data points.

2.6 Estimates exposure

We used systematic literature reviews to identify risk factor exposure studies published or identified since GBD 2017 and combined these with existing data from household and health examination surveys and census, morbidity, or satellite imagery and ground sensor data (used for estimation of particulate matter <2.5 [illegible]m in diameter [$PM_{2.5}$]). Certain risks, such as poor diet and excessive alcohol consumption, also incorporated administrative record systems. Data sources used in estimating risk factor exposure can be accessed through the data source tool at http://ghdx.healthdata.org/.

Once data were collected and compiled, the analytical flowchart describes the adjustments applied, where necessary, to correct for bias. Examples of these adjustments include use of urban studies for lead; crosswalks between different measurements, methods, and definitions, such as for self-reporting of obesity and glycated haemoglobin (HbA_{1C}) for diabetes; and age-sex splitting of data, such as for fasting plasma glucose (FPG) level, cholesterol level, and systolic blood pressure that may be reported from broad age-groups.

For the GBD, we developed two modelling approaches, a Bayesian meta-regression model (DisMod-MR 2.1) and a spatiotemporal Gaussian process regression model (ST-GPR), to pool data from different sources, control and adjust for bias in data, and incorporate other types of information such as country-level covariates. DisMod-MR 2.1 and ST-GPR are mixed effect models that borrow information across age, time, and locations to synthesise multiple data sources into unified estimates of levels and trends. A detailed description of the likelihood used for estimation and a full description of improvements made for DisMod-MR 2.1 were detailed by Vos and colleagues,[22] who provided additional detail in the Appendix to that paper.[8] The ST-GPR model has three main hyper-parameters that control for smoothing across time, age, and location. Values for these hyper-parameters were selected on the basis of cross- validation. Cross-validation tests were conducted for different combinations of the hyper-parameters for three types of models: one data-sparse model, one data-

moderate model, and one data-dense model. In each test, 20% of the data were held out, and the performance of each combination of hyper-parameters was evaluated on the held-out data. For each hyper-parameter combination, 10 cross-validation tests were conducted. The performance of each model in predicting the withheld 20% of the data was evaluated by using a combined measure based on root mean square error (RMSE) and uncertainty interval (UI) coverage. A detailed description of the ST-GPR process regression can be found below.

The main difference between these methods is their power to include unstructured types of data by sex and age group and their degree of flexibility. DisMod-MR 2.1 is the preferred tool in these cases because of its ability to integrate over age and adjust for different exposure definitions in the data; however, the use of Bayesian Markov Chain Monte Carlo (MCMC) simulations with large volumes of data renders the analysis computationally intensive and reduces the number of iterations that are possible. If standard age-group data are available – as is generally the case for metabolic risks – using ST-GPR becomes the preferred approach.

In some cases, we adapted our methods of modelling exposure to risks where necessary to account for complexities in the risk-outcome relationship or the need for particular handling of data, for example, dietary risks and ambient air pollution.

2.6.1 Theoretical minimum-risk exposure level (TMREL)

In this and all previous GBD studies, the counterfactual level of risk exposure used is the risk exposure that is both theoretically possible and minimises risk in the exposed population that consequently captures the maximum population-attributable burden.[16] For each risk evaluated in GBD 2021, Step 4 of the analytical flowchart describes the use of the best available epidemiological evidence from published and unpublished RRs by level of exposure and the lowest observed level of exposure from cohorts, used to select a single level of risk exposure that minimises risk from all causes of deaths combined to establish the TMREL. In principle, the TMREL for a given risk may vary by age, sex, and location if supported by clear evidence. Based on the available evidence, the TMREL itself can be uncertain, which is reflected in the 95% UIs in table S6. In GBD 2021, we updated the process of estimating TMREL for dietary risks. We set the TMREL to zero for all harmful dietary risk factors with monotonically increasing risk functions (e.g., processed meat intake); this excludes sodium. For protective risks with monotonically declining risk functions with exposure (e.g., fruit intake), we first determined the 85th percentile of exposure in the cohorts or trials used in the meta-regression of each outcome that was associated with the risk. Then, we determined the TMREL by weighting each risk-outcome pair by the relative global magnitude of each outcome.

2.6.2 Estimate population-attributable fractions

Risks are categorised on the basis of how exposure was measured: dichotomous, polytomous, and continuous. High low-density lipoprotein (LDL) cholesterol level is an example of a risk measured on a continuous scale. The PAF, which represents the proportion of risk that would be reduced in a given year if the exposure to a risk factor in the past were reduced to an ideal exposure scenario, is defined for a continuous risk factor as:[21]

$$\text{[illegible]}t = \frac{\int_{x=l}^{u} RR_{joasg}(x)P_{jasgt}(x)dx - RR_{joasg}(TMREL_{jas})}{\int_{x=l}^{u} RR_{joasg}(x)P_{jasgt}(x)dx}$$

Where PAF_{joasgt} is the PAF for cause o due to risk factor j for age group a, sex s, location g, and year t.

$RR_{joasg}(x)$ is the RR as a function of exposure level x for risk factor j for cause o, age group a, sex s, and location g with the lowest level of observed exposure as l and the highest as u; $P_{jasgt}(x)$ is the distribution of exposure at x for age group a, sex s, location g, and year t; and $TMREL_{jas}$ is the TMREL for risk factor j, age group a, and

sex s.

The PAF_{joasgt} for dichotomous and polytomous risk factors for every country is defined as:

$$\frac{\sum_{x=1}^{u} RR_{joast}(x)P_{jasgt}(x) - RR_{joasg}(TMRE_{jas})}{\sum_{x=1}^{u} RR_{joas}(x)P_{jasgt}(x)}$$

where PAF_{joasgt} is the PAF for cause o due to risk factor j for age group a, sex s, location g, and year t.

$RR_{joasg}(x)$ is the RR as a function of exposure level x for risk factor j for cause o, age group a, sex s, and location g on a plausible range of exposure levels from l to u; $P_{jasgt}(x)$ is the proportion of the population in risk group (prevalence) for age group a, sex s, location g, and year t; and $TMREL_{jas}$ is the TMREL for risk factor j, age group a, and sex s.

2.6.3 Estimate summary exposure values

We first calculate risk, r, and cause, c, for specific SEVs by using the following equation, for each most-detailed age, sex, location, year, and outcome. PAF is the YLL (expect for occupational noise, bullying victimization, and occupational ergonomic factors, which are YLD only and thus use the YLD) PAF. RR_{max} for categorical risks is the RR at the highest category of exposure. For continuous risks, this is

$$= \frac{\frac{PAF_{rc}}{1 - PAF_{rc}}}{RR_{max} - 1}$$

$$= RR^{\frac{TMREL - 1^{st} exposure}{RR_{scalar}}}$$ if protective, or

$$= RR^{\frac{99^{th} exposure - TMREL}{RR_{scalar}}}$$

otherwise, and for custom modelled risks like ambient particulate matter pollution, HAP from solid fuels, alcohol, smoking, bullying, and activity, the modeller provides draws of RR_{max}. Generally, RRs do not vary across time and space. However, exceptions exist, such as risks from second-hand smoke (SHS) or HAP for which the RR is based on the integrated exposure response (IER) curve. In these cases, the RR is averaged across location and year to ensure no time or space variation. If the PAF is negative, which signifies a protective effect for that outcome, the PAF is set to 0 and the SEV is then also 0 because the SEV is univariate and constrained to be a value between 0 and 1. Once we obtained a set of risk-cause specific SEVs at the most-detailed risk, cause, age, sex, and location for all years, we averaged across causes to produce the final risk specific SEV_r, where

$$\frac{1}{N(c)} \sum_{c} SEV_{rc}$$

$N(c)$is the total number of outcomes for a risk.

2.6.4 Mediation

The portion of the burden of disease that is attributable to various combinations of risk factors or to all risk factors combined has been a topic of broad interest.[30] In GBD 2010, we only aggregated the burden of risk factors for some clusters of risks, including access to improved water and sanitation, child and maternal malnutrition, tobacco smoking, alcohol use, dietary risk factors, occupational risk factors, and sexual abuse and violence. We did not aggregate air pollution and metabolic risk factors. For GBD 2013 onwards, we aggregated all risk factors into three large categories—behavioural, environmental and occupational, and metabolic risks—and aggregated all GBD risk factors into a single attributable fraction for each disease and eventually for all causes of burden.

Aggregating risk factors at different levels shares three essential challenges:

1. Risk factor coexistence or aggregation: for example, metabolic risk factors often occur together, or high-risk behaviours such as drug abuse and unsafe sex are related.
2. Mediation: a risk factor may affect another risk factor that lies in the physiological pathway to a disease outcome. It can be inside a cluster of risk factors, such as the effect of obesity through an increase in FPG level and later cardiovascular disease (CVD) outcomes, or between clusters of risk factors, such as the effect of fibre on cholesterol.
3. The formula used to calculate the aggregated PAF.

The aggregation method is conceptually applicable to other aggregations such as socioeconomic factors, education, homelessness, and refugee status that are being considered for inclusion in future GBD iterations. In the next section, we explain our approach to dealing with these challenges.

There are three patterns of associations between risk factors to consider (Figure C). The first concerns confounding; risk B affects risk A and outcome C (Pattern 1 in *Patterns of associations between risk factors*). In these cases, the RR for A should be adjusted for B; for example, the fruit RR is adjusted for smoking. If part of the effect of A is through B, a mediator, we do not adjust the effect of A for B. For example, we do not adjust the RR of body-mass index (BMI) for cholesterol because cholesterol lies in the biological pathway between BMI and cardiovascular outcomes (Pattern 2 in in *Patterns of associations between risk factors*). The third pattern occurs when risks A and B are proxies of a third variable Z and aggregation aims to estimate the total effect of a latent variable Z on C. An example is child growth failure, which is measured by stunting, wasting, and underweight as proxies.

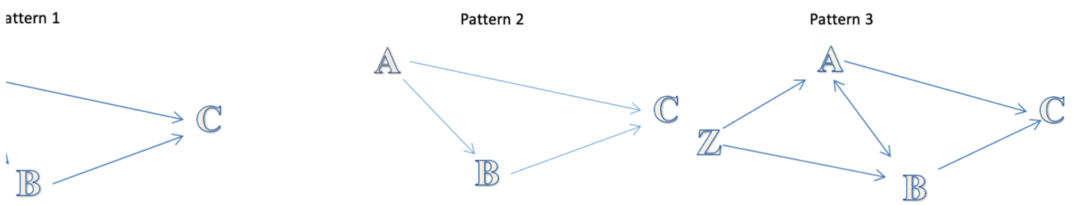

2.6.5 Calculating the burden of multiple risk factors.

Validation studies have reported congruency between the true risk associated with multiple risk factors affecting the same outcome and a multiplicative aggregation of the PAFs of the individual risk factors (formula below).[22]

$$PAF_{1..i} = 1 - \prod_{i=1}^{n}(1 - PAF_i)$$

where *PAF* is the population attributable fraction and *i* is each individual risk factor. The same validation studies also found that the overestimation from ignoring the covariance between risk factors is small. This small overestimation was important to note because few data sources exist from which we can draw information on covariance. We endeavoured to evaluate RRs that were controlled for confounders. However, because we had to rely on the literature for many RRs, we did not always have full control over the choice of confounders controlled for in each study.

2.6.6 Adjusting for mediation

When aggregating the effects of multiple risk factors, we included an MF if a part of the effect of one risk factor was included in the effect estimated for in the mediator. First, we prepared a list of possible mediations, and especially between behavioural risks and metabolic risk factors with cardiometabolic outcomes. We did not assume any mediation effect between risk factors for cancers. Danaei and colleagues assumed that part of the effect of BMI on ischaemic heart disease (IHD) is through high systolic blood pressure (SBP), cholesterol level, and FPG.[23] The proportion of the BMI effect that can be explained by other metabolic risk factors is the amount of mediation. The difference between the crude RR of BMI on IHD with the RR adjusted for SBP, FPG, and cholesterol level reflects the amount

of BMI effect on IHD that is mediated and already included in SBP, FPG, and cholesterol level:

$$\frac{RR_{crude} - RR_{adjusted}}{RR_{crude} - 1}$$

So, to aggregate the PAF of multiple risk factors, we first calculated the part of the excess risk (RR-1) of every risk factor that is not mediated, re-compute the PAF so that it only includes the non-mediated risk then aggregated PAFs by assuming they are independent. Therefore, if MF is the mediation factor of R2 through R1, the adjusted RR for R2 including only the non- mediated component of risk is The PAF accounting for mediation is then computed using the adjusted RR and the joint PAF computed. For every paired risk factor and outcome, the matrix of possible mediations was calculated and used.

$$= MF_{2/1}(RR_2 - 1) + 1$$

2.6.7 Calculating mediation factor

The best example is the mediation of BMI through SBP, FPG, and cholesterol level reported by Danaei et al.[23] In their meta-analysis, they report the adjusted and unadjusted RR of BMI on IHD and stroke based on combined data from individual cohorts. They calculated the MF by using the following equation, and we used it directly as the MF in risk factor aggregation. Using individual-level data from cohort studies, we estimated the MF for other metabolic risk factors and some dietary risks. For many other risk factors, no data are available to enable the use of the first method. Instead, we searched studies to estimate the effect of the risk factor on the mediator and, finally, the expected increase in IHD risk. We pooled available studies to calculate the unit increase in the mediator per unit increase in the risk factor to calculate the size of the IHD RR.

$$\frac{RR_{crude} - RR_{adjusted}}{RR_{crude} - 1}$$

2.6.8 Uncertainty of aggregated and mediated PAFs

We generated 1000 draws of the posterior distribution of the MF calculated by different methods to use beside draws of other inputs to the PAF aggregation.

2.6.9 Important assumptions in aggregating risk factors and including mediation

1 – The MFs or PAF adjustments are similar across countries, age, sex, and years. Although the size of mediation is probably different in different populations, little data is available to inform the covariance between different risk factors or the MF amount by age and country. For example, in some countries, the size of the mediated BMI-IHD PAF exerted through cholesterol level, as calculated by the MF, was even bigger than the total burden of cholesterol level. This finding indicated that less of the effect of BMI is mediated through cholesterol level and MFs are not similar across countries.

2 – For many risk-mediator-outcome pairs, no data are available, so we assumed the mediation is zero.

3 – Because the covariance between undernutrition indicators differs by location (and across time, but results were not reported), and an interaction exists between these indicators, the total burden might be underestimated.

4 – We assumed no significant covariance between PAFs, which might not be true between some risk factors, such as metabolic risk factors. Although this overestimation can be controlled by using adjusted RRs, using crude RRs for BMI and other metabolic risk factors may cause significant overestimation of the aggregated metabolic risks burden.

2.6.10 Estimate attributable burden

Four key components are included in the estimation of the burden attributable to a given risk factor: the metric of burden being assessed (the number of deaths, YLLs, YLDs, or DALYs [the sum of YLLs and YLDs]); the exposure levels for a risk factor; the RR of a given outcome due to exposure; and the counterfactual level of risk factor exposure. Estimates of attributable burden as DALYs for risk-outcome pairs were generated by using the following model:

$$[illegible]_{gt} = \sum_{o=1}^{w} DALY_{joasgt} PAF_{joasgt}$$

where AB_{jasgt} is the attributable burden for risk factor j for age group a, sex s, location g, and year t;

$DALY_{joasgt}$ is total DALYs for cause o (of w relevant outcomes for risk factor j) for age group a, sex s, location g, and year t; and PAF_{joasgt} is the PAF for cause o due to risk factor j for age group a, sex s, location g, and year t. The proportions of deaths, YLLs, or YLDs attributable to a given risk factor or risk factor cluster were analogously computed by sequentially substituting each metric in place of DALYs in the equation provided.

2.6.11 Decomposition analysis of deaths and DALYs

We conducted a decomposition analysis of changes in DALYs from 2010 to 2021, decomposing changes in all-age cause-specific DALYs attributable to all risk factors and individual risk factors due to changes in population growth, population age structure, exposure to the given risk for a disease, and risk-deleted death and DALY rates. In this case, risk-deleted rates are the rates obtained after removing the effect of a risk factor or combination of risk factors — in other words, observed DALY rates multiplied by one minus the PAF for the risk or set of risks. Our decomposition analyses draw from methods developed by Das Gupta[24] to provide a computationally tractable solution for isolating drivers of burden changes whereby all combinations of possible pathways are averaged across factors. Attributable burden was determined, following the methods of Das Gupta, as a product of three factors such that: where T_{asgt} represents the attributable burden at year t; $A_{sgt}$is the age-specific population size for a given age group a, sex s, and location g at year t; B_{asgt} is the underlying rate of the outcome unrelated to the risk factor or observed rate, multiplied by 1 − PAF for a given age group a, sex s, and location g at year t; and C_{asgt} is the ratio of the attributable burden to the underlying rate, which reflects the risk exposure effect for a given age group a, sex s, and location g at year t defined as $PAF/(1 - PAF)$when decomposing attributable burden to a risk. Risk exposure effects for individual risk factors are scaled such that they sum to the all-risk exposure effect by location, age, sex, and cause accounting for mediation. This process allows for aggregation of risks; the exposure for all risks for a disease can be split into exposure to metabolic, behavioural, and environmental risks. The contribution of each factor to total change in attributable burden was determined by changing the level of one factor from time t_0 to t_1 – here 2010 to 2021 – with all other factors held constant. Thus, the effect of any of the three factors, for example A_{asgt}on the change of the attributable burden between 2010 (A_{10}) and 2021 (A_{17}) is calculated as:

$$T_{asgt} = (A_{asgt} B_{asgt} C_{asgt})$$

$$[illegible] - A_{10})\left(\frac{B_{10}C_{10} + B_{19}C_{19}}{3} + \frac{B_{10}C_{19} + B_{19}C_{10}}{6}\right)$$

where E_A is the proportion of change due to factor A, and the subscripts for each factor in the equation denote the year for each estimate. Because the effect depends on the order of entry of the factor, we calculated the average of all combinations of the three factors.[35] The proportion of change due to factorA_{sgt}, the age-specific population size for a given age group a, sex s, and location g at year t, is then further split, setting change in population growth equal to the percentage change in the all-age population from time t_0 to t_1 and change in population age structure to the residual, giving four factors.

This three-factor decomposition method does not work for risks for which the PAF, by definition, is 100% (such as high FPG level and type 2 diabetes) or for which the PAF is directly estimated (such as for unsafe sex and HIV). We used a two-factor decomposition method that examines the contribution of population, ageing, and risk exposure in the cases of child underweight and protein-energy malnutrition, child wasting and protein-energy malnutrition, short gestation for birthweight and neonatal preterm birth complications, low birthweight for gestation and neonatal preterm birth complications, iron deficiency (ID) and iron-deficiency anaemia (IDA), alcohol use and liver cancer due to alcohol use, alcohol use and cirrhosis and other chronic liver diseases due to alcohol use, alcohol use and alcohol use disorders, alcohol use and alcoholic cardiomyopathy, drug use and drug use disorders, occupational particulate matter, gases, and fumes and other pneumoconiosis, occupational particulate matter, gases, and fumes and coal workers pneumoconiosis, occupational exposure to asbestos and asbestosis, and occupational exposure to silica and silicosis. Effectively, we assumed trends in these cases are driven by exposure, not change in the risk-deleted rates. Conversely, for unsafe sex and sexually transmitted diseases excluding HIV, we used a two-factor decomposition method that examines the contribution of population, ageing, and risk-deleted death and DALY rates and assumed that trends in these cases are driven by risk-deleted rates, not change in exposure. For high FPG level and type 1 and 2 diabetes, high FPG level and CKD due to type 1 and 2 diabetes, high SBP and hypertensive heart disease, high SBP and CKD due to hypertension, and impaired kidney function and CKD, we used GBD estimates of SEVs for the given risk and the case-fatality rate to decompose trends into an estimate of the contribution of the three factors. Similarly, for unsafe sex and cervical cancer, we used GBD estimates of the incidence of cervical cancer and the case-fatality rate to decompose trends into an estimate of the contribution of the three factors. For unsafe sex and HIV, we used spectrum counterfactual and CD4 risk-weighted prevalence.

Section 3. Changes in the modelling of stroke for GBD 2021

Several changes were made to the modelling strategy for stroke for the GBD 2021 study. In GBD 2019 and prior studies, chronic stroke was modelled for both subtypes (ischaemic and haemorrhagic or other) together to estimate the total prevalence of chronic stroke. For the GBD 2021 study, each stroke subtype was modelled independently, resulting in separate acute and chronic stroke models for ischaemic stroke (IS), intracerebral haemorrhage (ICH) and subarachnoid haemorrhage (SAH) type separately. This change was made in order to simplify the stroke modelling process and to ensure that all major pathological types were estimated correctly. In the GBD 2017 and prior studies, severity splits were based on estimates derived from standard GBD analysis of the U.S. Medical Expenditure Panel Survey. For the GBD 2021 study, a review of studies reporting modified Rankin scores following stroke was performed and disability weights were applied using a model of modified Rankin level by age and sex as described above. In GBD 2021, we updated the regressions for stroke and diabetes. We dropped the proportion of garbage from the regression formula and ran regression on high-quality, low proportion garbage data (4/5 stars, < 50% GC). We also included all covariates included in the CODEm models for both stroke and diabetes.

For the GBD 2021 study, in order to better represent population-level disease incidence for IS, adjustments for alternative study methods and case definitions were applied to data prior to analysis in DisMod-MR. These adjustments were performed using the MR-BRT modelling tool. We adjusted for several study-specific factors such as whether the data were from a hospital and whether the data included both first-ever and recurrent ischaemic strokes. We updated our methods for redistributing deaths due to unspecified stroke (ICD-10 codes I62 and I64) to the three modelled stroke subtypes included in GBD. All available data on incidence, prevalence and mortality were used to estimate stroke burden. Mortality was estimated using vital registration, verbal autopsy reports and trained informants, while incidence data were estimated using the DisMod-MR meta-regression modelling

tool.[3]

The tables below indicate the covariates used by cause in the estimation process, as well as the beta and exponentiated beta values.

Step 1:

Cause	Variable name	Measure	beta	Exponentiated beta
Chronic ischaemic stroke	Log-transformed SEV scalar: Ischaemic Stroke	Prevalence	0.83 (0.75 — 1.03)	2.29 (2.12 — 2.80)
Chronic ischaemic stroke	LDI (I$ per capita)	Excess mortality rate	-0.16 (-0.29 — -0.1)	0.85 (0.75 — 0.90)
Chronic haemorrhagic stroke	Log-transformed SEV scalar: Hem Stroke	Prevalence	0.79 (0.75 — 0.92)	2.21 (2.12 — 2.50)
Chronic haemorrhagic stroke	LDI (I$ per capita)	Excess mortality rate	-0.12 (-0.16 — -0.1)	0.89 (0.85 — 0.90)
First ever acute haemorrhagic stroke	Hospital data	Incidence	0.54 (0.54 – 0.54)	1.71 (1.71 – 1.72)
First ever acute haemorrhagic stroke	Any stroke	Incidence	1.27 (1.27 – 1.28)	3.57 (3.56 – 3.59)
First ever acute haemorrhagic stroke	First-ever acute stroke, ischemic or haemorrhagic	Incidence	0.52 (0.52 – 0.53)	1.69 (1.68 – 1.71)
First ever acute haemorrhagic stroke	Log-transformed age-standardised SEV scalar: haemorrhagic stroke	Incidence	0.77 (0.75 – 0.82)	2.17 (2.12 – 2.27)
First ever acute haemorrhagic stroke	Any stroke	Excess mortality rate	-0.48 (-0.66 – -0.32)	0.62 (0.52 – 0.73)
First ever acute haemorrhagic stroke	First-ever acute stroke, ischemic or haemorrhagic	Excess mortality rate	-0.081 (-0.3 – 0.16)	0.62 (0.52 – 0.73)
First ever acute ischaemic stroke	Hospital data	Incidence	0.38 (0.37 – 0.38)	1.46 (1.45 – 1.46)
First ever acute ischaemic stroke	Any stroke	Incidence	0.31 (0.29 – 0.33)	1.37 (1.34 – 1.39)
First ever acute ischaemic stroke	First-ever acute stroke, ischemic or haemorrhagic	Incidence	0.37 (0.36 – 0.38)	1.44 (1.43 – 1.46)
First ever acute ischaemic stroke	Log-transformed age-standardised SEV scalar: ischemic stroke	Incidence	1.16 (1.09 – 1.22)	3.21 (2.99 – 3.39)

Step 2:

Cause	Variable name	Measure	beta	Exponentiated beta
Chronic ischemic stroke with CSMR	Log-transformed SEV scalar: Ischaemic stroke	Prevalence	0.89 (0.75 – 1.19)	2.44 (2.13 – 3.27)
Chronic ischemic stroke with CSMR	LDI (I$ per capita)	Excess mortality rate	-0.49 (-0.5 – -0.46)	0.61 (0.61 – 0.63)

Chronic haemorrhagic stroke with CSMR	Log-transformed SEV scalar: Haemorrhagic stroke	Prevalence	0.88 (0.75 – 1.15)	2.40 (2.13 – 3.17)
Chronic haemorrhagic stroke with CSMR	LDI (I$ per capita)	Excess mortality rate	-0.48 (-0.5 – -0.44)	0.62 (0.61 – 0.64)
First-ever acute haemorrhagic stroke with CSMR	Any stroke	Incidence	1.27 (1.27 – 1.29)	3.58 (3.56 – 3.62)
First-ever acute haemorrhagic stroke with CSMR	First-ever acute stroke, ischemic or haemorrhagic	Incidence	0.52 (0.52 – 0.54)	1.69 (1.68 – 1.71)
First-ever acute haemorrhagic stroke with CSMR	Log-transformed SEV scalar: Hem stroke	Incidence	1.11 (1.01 – 1.20)	3.03 (2.74 – 3.33)
First-ever acute haemorrhagic stroke with CSMR	Any stroke	Excess mortality rate	-0.37 (-0.49 – -0.27)	0.69 (0.62 – 0.77)
First-ever acute haemorrhagic stroke with CSMR	First-ever acute stroke, ischemic or haemorrhagic	Excess mortality rate	0.023 (-0.2 – 0.23)	1.02 (0.82 – 1.25)
First-ever acute ischaemic stroke with CSMR	Any stroke	Incidence	0.32 (0.30 – 0.33)	1.38 (1.35 – 1.39)
First-ever acute ischaemic stroke with CSMR	First-ever acute stroke, ischemic or haemorrhagic	Incidence	0.37 (0.36 – 0.38)	1.44 (1.43 – 1.46)
First-ever acute ischaemic stroke with CSMR	Log-transformed age-standardised SEV scalar: Ischemic stroke	Incidence	1.11 (1.05 – 1.18)	3.04 (2.86 – 3.26)
First-ever acute ischaemic stroke with CSMR	Any stroke	Excess mortality rate	-0.34 (-0.45 – -0.24)	0.71 (0.64 – 0.79)
First-ever acute ischaemic stroke with CSMR	First-ever acute stroke, ischemic or haemorrhagic	Excess mortality rate	-0.69 (-0.82 – -0.56)	0.50 (0.44 – 0.57)

In GBD 2021, no substantial changes were made to Dismod-MR 2.1 but we made more substantial changes to how we used the tool. First, we added the year 2021 as an additional year of estimation. Second, we also included the option again to have random effects on cause-specific mortality rates (CSMR) and excess mortality rates (EMR). This functionality had been dropped a couple of GBD rounds earlier. Third, as we did all our adjustments for alternative case definition and study methods as well as adjustments to both sex data points prior to entering data into DisMod-MR 2.1, we no longer used the functionality in DisMod-MR 2.1 to estimate coefficients for study covariates. Fourth, based on simulation testing we found that coverage improved, and errors reduced when passing down priors with a wider setting of minimum coefficient of variation (which determines the uncertainty around priors and hence how 'informative' the priors are) than had generally been used in past GBD iterations. We settled on a default value of 0.8 where in the past values of 0.4 or less had been more commonly used. We made some exceptions for high prevalent conditions where a lower minimum CV setting

achieved the task of making priors less informative but not completely uninformative.

Fifth, we changed our approach to estimating excess mortality rates, the key link in the model between cause-specific mortality rates (CSMR) and incidence and prevalence. In the past two GBD rounds we calculated priors on excess mortality and entered these as data points by matching sex-specific prevalence data with an age width of 20 or less with the corresponding CSMR for the same location and year. For the sake of stability, we excluded calculation of EMR for prevalence data points of less than 1 in a million. EMR is simply calculated as CSMR divided by prevalence. As with previous GBD years, for diseases with an average duration of less than a year (as indicated by a setting of remission greater than one), we ran an initial global model to get an equivalent prevalence and used the following formula to calculate EMR:

EMR = CSMR * (remission + (ACMR - CSMR) + EMR_pred) / incidence

where, ACMR = all-cause mortality rate and EMRpred = EMR fit from an initial global DISMOD model.

Section 5. Appendix tables

Appendix Table 1. Absolute numbers and age-standardised rates per 100,000 people per year, with 95% uncertainty intervals (UI), of incident and prevalent strokes, deaths from stroke and disability-adjusted life years (DALYs) due to stroke by pathological type of stroke and World Bank country income level, and their changes (in % with 95% UI) from 1990 to 2021, for both sexes

Pathological types of stroke		Low-income countries		Low middle-income countries		Upper-middle income countries		High-income countries	
		Metric in 2021	Percentage change, 1990-2021	Metric in 2021	Percentage change, 1990-2021	Metric in 2021	Percentage change, 1990-2021	Metric in 2021	Percentage change, 1990-2021
**ISCHAEMIC STROKE**	Absolute number	321,450 (281,401 to 363,013)	102.6% (93.2 to 111.6)	2,093,798 (1,813,510 to 2,396,553)	107.9% (98.9 to 116.5)	3,889,250 (3,241,879 to 4,572,469)	149.3% (136.0 to 163.3)	1,492,981 (1,318,520 to 1,676,925)	5.1% (0.3 to 10.2)
incidence	Age-standardised rate per 100,000	105 (92 to 118)	-8.3% (-12.1 to -4.1)	84 (73 to 96)	-14.7% (-18.2 to -11.0)	118 (100 to 138)	0.5% (-4.1 to 5.0)	63 (56 to 71)	-42.1% (-44.4 to -39.4)
	Absolute number	3,181,845 (3,032,936 to 3,327,580)	105.4% (102.0 to 109.3)	19,280,414 (17,671,054 to 20,829,399)	118.4% (114.3 to 122.6)	30,292,707 (27,603,277 to 32,969,926)	143.0% (134.4 to 150.8)	17,132,935 (16,179,194 to 18,129,482)	45.4% (41.3 to 49.6)
prevalence	Age-standardised rate per 100,000	936 (889 to 981)	-7.7% (-9.4 to -6.0)	694 (636 to 750)	-6.0% (-7.6 to -4.3)	912 (833 to 990)	11.0% (8.2 to 13.6)	769 (728 to 812)	-15.7% (-17.8 to -13.4)
	Absolute number	127,301 (107,432 to 155,278)	93.0% (71.5 to 117.2)	1,076,284 (973,754 to 1,190,138)	105.9% (88.1 to 127.7)	1,782,747 (1,556,573 to 1,996,388)	87.9% (63.3 to 115.1)	601,118 (501,990 to 653,658)	-22.5% (-29.1 to -19.1)
death	Age-standardised rate per 100,000	55 (47 to 67)	-16.7% (-25.3 to -7.0)	56 (51 to 62)	-22.0% (-28.2 to -13.6)	56 (49 to 63)	-35.6% (-43.2 to -27.1)	20 (17 to 21)	-66.9% (-68.9 to -65.7)
	Absolute number	3,031,820 (2,590,322 to 3,619,623)	85.0% (64.5 to 106.0)	22,628,556 (20,495,843 to 25,270,952)	96.8% (79.6 to 115.9)	34,608,561 (30,758,910 to 38,381,406)	73.1% (51.6 to 96.6)	10,077,853 (8,938,425 to 10,994,541)	-22.4% (-26.4 to -18.9)
DALYs	Age-standardised rate per 100,000	1,054 (902 to 1,254)	-16.3% (-24.2 to -7.1)	1,018 (924 to 1,131)	-21.2% (-27.6 to -13.5)	1,031 (918 to 1,141)	-33.0% (-40.9 to -24.5)	385 (344 to 423)	-61.1% (-63.0 to -59.3)
**INTRACEREBRAL HAEMORRHAGE**	Absolute number	206,847 (189,385 to 224,140)	47.3% (42.4 to 52.5)	1,357,849 (1,190,849 to 1,530,558)	76.4% (70.7 to 83.4)	1,522,969 (1,324,208 to 1,713,890)	39.8% (32.6 to 48.4)	354,137 (316,688 to 387,754)	-0.6% (-4.5 to 4.0)
incidence	Age-standardised rate per 100,000	66 (60 to 71)	-32.9% (-34.9 to -30.4)	52 (46 to 58)	-26.5% (-28.9 to -23.4)	48 (42 to 54)	-40.4% (-43.1 to -37.4)	16 (14 to 17)	-44.6% (-46.3 to -42.7)
	Absolute number	981,840 (925,095 to 1,045,196)	55.4% (52.3 to 58.5)	6,592,374 (6,006,447 to 7,282,796)	85.1% (80.1 to 90.2)	6,217,517 (5,600,342 to 6,876,172)	32.8% (26.8 to 38.7)	2,801,034 (2,577,861 to 3,027,879)	22.4% (18.5 to 26.0)
prevalence	Age-standardised rate per 100,000	223 (208 to 239)	-32.8% (-34.2 to -31.4)	218 (197 to 241)	-16.1% (-18.2 to -14.2)	192 (175 to 211)	-29.7% (-31.8 to -27.6)	145 (135 to 156)	-23.3% (-25.0 to -21.7)
	Absolute number	208,864 (175,618 to 244,161)	40.8% (21.0 to 60.2)	1,217,947 (1,087,860 to 1,337,867)	70.1% (53.2 to 90.3)	1,619,051 (1,392,340 to 1,868,967)	36.6% (14.5 to 61.8)	259,925 (227,902 to 276,305)	-10.1% (-16.9 to -6.0)
death	Age-standardised rate per 100,000	69 (58 to 81)	-35.0% (-42.4 to -26.4)	51 (46 to 56)	-29.8% (-36.8 to -21.2)	49 (42 to 56)	-45.7% (-54.3 to -36.2)	10 (9 to 11)	-54.8% (-57.2 to -53.1)
	Absolute number	6,071,127	30.2% (7.8 to 51.3)	33,315,424	55.5% (40.7 to 74.7)	34,910,112 (30,338,131 to 40,111,545)	15.2% (-2.7 to 36.8)	5,100,101 (4,718,497 to 5,353,057)	-24.2% (-27.8 to -21.5)

DALYs		(5,071,775 to 7,095,486)		(30,059,372 to 36,563,324)					
	Age-standardised rate per 100,000	1,649 (1,383 to 1,925)	-37.3% (-45.7 to -28.9)	1,253 (1,125 to 1,373)	-31.5% (-38.0 to -23.6)	1,014 (883 to 1,163)	-49.1% (-56.9 to -39.9)	235 (220 to 245)	-56.8% (-58.3 to -55.2)
**SUBARACHNOID HAEMORRHAGE** incidence	Absolute number	32,548 (28,385 to 36,969)	85.5% (78.7 to 91.4)	250,056 (216,398 to 287,037)	74.2% (69.1 to 80.0)	267,598 (235,570 to 308,646)	15.2% (8.2 to 21.6)	146,669 (131,090 to 166,018)	27.7% (23.2 to 33.1)
	Age-standardised rate per 100,000	8 (7 to 9)	-18.5% (-21.1 to -15.7)	8 (7 to 10)	-21.1% (-23.8 to -18.1)	9 (8 to 10)	-41.9% (-45.5 to -38.5)	8 (7 to 9)	-19.1% (-21.3 to -16.5)
prevalence	Absolute number	378,329 (349,991 to 409,139)	97.7% (94.2 to 101.1)	2,616,363 (2,358,816 to 2,886,985)	92.4% (88.2 to 96.5)	2,726,092 (2,470,925 to 3,006,144)	38.8% (34.3 to 42.9)	2,124,949 (1,961,237 to 2,282,214)	53.9% (50.7 to 57.0)
	Age-standardised rate per 100,000	90 (84 to 97)	-13.7% (-15.3 to -12.2)	85 (77 to 94)	-12.3% (-14.0 to -10.7)	85 (78 to 94)	-25.5% (-27.3 to -23.8)	109 (101 to 117)	-3.4% (-5.1 to -1.7)
death	Absolute number	12,440 (5,994 to 28,705)	46.8% (13.6 to 87.7)	115,385 (90,852 to 146,911)	52.5% (26.3 to 88.1)	155,409 (129,167 to 181,619)	-32.6% (-51.5 to 7.7)	69,265 (61,155 to 73,496)	15.6% (7.7 to 21.5)
	Age-standardised rate per 100,000	4 (2 to 8)	-34.6% (-50.8 to -17.8)	5 (4 to 6)	-34.6% (-47.3 to -17.4)	5 (4 to 5)	-71.8% (-80.0 to -53.6)	3 (3 to 3)	-39.2% (-42.3 to -36.9)
DALYs	Absolute number	492,714 (264,287 to 1,057,249)	44.3% (15.9 to 86.7)	4,074,006 (3,289,337 to 5,071,888)	39.4% (21.1 to 64.2)	4,261,235 (3,633,575 to 4,875,629)	-37.7% (-53.2 to -7.3)	1,802,415 (1,678,826 to 1,916,006)	-6.3% (-10.3 to -2.8)
	Age-standardised rate per 100,000	112 (59 to 244)	-33.0% (-46.7 to -16.5)	142 (115 to 177)	-34.4% (-45.0 to -20.7)	127 (109 to 145)	-69.9% (-77.6 to -53.5)	93 (88 to 99)	-43.1% (-45.1 to -41.1)
**TOTAL STROKE** incidence	Absolute number	560,845 (518,145 to 604,503)	77.1% (71.9 to 82.4)	3,701,704 (3,374,075 to 4,043,086)	92.7% (87.8 to 97.9)	5,679,818 (5,053,180 to 6,432,158)	97.1% (88.3 to 106.3)	1,993,787 (1,822,111 to 2,172,681)	5.4% (1.6 to 9.6)
	Age-standardised rate per 100,000	178 (164 to 193)	-19.6% (-21.8 to -16.9)	144 (132 to 157)	-19.7% (-21.8 to -17.4)	175 (156 to 196)	-17.9% (-21.2 to -14.4)	87 (79 to 94)	-41.0% (-43.0 to -39.0)
prevalence	Absolute number	4,519,760 (4,365,302 to 4,672,635)	91.7% (89.1 to 94.1)	28,335,905 (26,853,274 to 30,089,037)	107.3% (104.3 to 110.2)	38,997,308 (36,331,464 to 41,833,182)	105.2% (99.4 to 111.4)	21,888,684 (21,018,495 to 22,892,978)	42.8% (39.8 to 45.9)
	Age-standardised rate per 100,000	1,242 (1,195 to 1,289)	-13.8% (-15.1 to -12.6)	991 (935 to 1,054)	-8.9% (-10.0 to -7.7)	1,182 (1,105 to 1,263)	-1.6% (-3.6 to 0.5)	1,016 (974 to 1,060)	-15.6% (-17.3 to -13.8)
death	Absolute number	348,605 (302,466 to 396,642)	56.5% (36.7 to 76.5)	2,409,616 (2,224,527 to 2,592,494)	83.3% (68.7 to 102.3)	3,557,207 (3,113,008 to 4,004,513)	50.4% (30.4 to 73.8)	930,308 (791,426 to 1,001,941)	-17.3% (-24.0 to -14.0)
	Age-standardised rate per 100,000	128 (112 to 146)	-28.2% (-35.8 to -19.9)	112 (103 to 121)	-26.3% (-32.1 to -18.6)	110 (96 to 123)	-43.4% (-50.5 to -35.1)	33 (29 to 35)	-62.2% (-64.2 to -61.1)
DALYs	Absolute number	9,595,662 (8,261,289 to 10,975,608)	44.4% (22.1 to 64.4)	60,017,986 (55,442,405 to 64,220,469)	67.4% (53.4 to 83.5)	73,779,907 (65,304,655 to 82,892,321)	29.1% (12.7 to 48.4)	16,980,369 (15,364,270 to 18,217,655)	-21.5% (-25.3 to -18.5)
	Age-standardised rate per 100,000	2,815 (2,436 to 3,213)	-30.6% (-38.5 to -22.2)	2,413 (2,230 to 2,585)	-27.7% (-33.3 to -20.5)	2,173 (1,926 to 2,440)	-45.1% (-51.7 to -37.4)	713 (650 to 765)	-58.0% (-59.6 to -56.5)

Appendix Table 2. Incident cases, deaths, prevalent cases, and DALYs for ischaemic stroke in 2021 and percentage changes of age-standardised rates for 1990-2021, by location

Country, region	Incident cases (95% uncertainty interval)		Deaths (95% uncertainty interval)		Prevalent cases (95% uncertainty interval)		DALYs (95% uncertainty interval)	
	2021 counts	% change in age-standardised rates, 1990-2021	2021 counts	% change in age-standardised rates, 1990-2021	2021 counts	% change in age-standardised rates, 1990-2021	2021 counts	% change in age-standardised rates, 1990-2021
**Countries categorised by the World Bank Income level**								
**Global**	7,804,449 (6,719,760 to 8,943,692)	-15.8% (-18.8 to -13.3)	3,591,499 (3,213,281 to 3,888,327)	-39.6% (-43.8 to -35.3)	69,944,885 (64,788,695 to 75,009,603)	-3.5% (-5.1 to -2.1)	70,357,912 (64,329,576 to 76,007,063)	-34.9% (-39.5 to -30.0)
**High income**	1,492,981 (1,318,520 to 1,676,925)	-42.1% (-44.4 to -39.4)	601,118 (501,990 to 653,658)	-66.9% (-68.9 to -65.7)	17,132,935 (16,179,194 to 18,129,482)	-15.7% (-17.8 to -13.4)	10,077,853 (8,938,425 to 10,994,541)	-61.1% (-63.0 to -59.3)
**Upper Middle income**	3,889,250 (3,241,879 to 4,572,469)	0.5% (-4.1 to 5.0)	1,782,747 (1,556,573 to 1,996,388)	-35.6% (-43.2 to -27.1)	30,292,707 (27,603,277 to 32,969,926)	11.0% (8.2 to 13.6)	34,608,561 (30,758,910 to 38,381,406)	-33.0% (-40.9 to -24.5)
**Lower Middle income**	2,093,798 (1,813,510 to 2,396,553)	-14.7% (-18.2 to -11.0)	1,076,284 (973,754 to 1,190,138)	-22.0% (-28.2 to -13.6)	19,280,414 (17,671,054 to 20,829,399)	-6.0% (-7.6 to -4.3)	22,628,556 (20,495,843 to 25,270,952)	-21.2% (-27.6 to -13.5)
**Low income**	321,450 (281,401 to 363,013)	-8.3% (-12.1 to -4.1)	127,301 (107,432 to 155,278)	-16.7% (-25.3 to -7.0)	3,181,845 (3,032,936 to 3,327,580)	-7.7% (-9.4 to -6.0)	3,031,820 (2,590,322 to 3,619,623)	-16.3% (-24.2 to -7.1)
**Countries categorised by the sociodemographic index (SDI) level**								
**High SDI**	1,351,912 (1,179,761 to 1,535,710)	-37.4% (-39.7 to -34.9)	507,950 (426,777 to 553,062)	-63.9% (-66.2 to -62.4)	15,864,865 (14,925,798 to 16,872,855)	-12.8% (-15.1 to -10.5)	8,975,176 (7,913,835 to 9,812,024)	-56.8% (-59.2 to -54.7)
**High-middle SDI**	2,243,340 (1,899,825 to 2,614,463)	-17.9% (-21.0 to -15.0)	1,151,655 (1,025,972 to 1,263,429)	-46.7% (-51.0 to -41.8)	17,110,640 (15,738,888 to 18,478,803)	-1.4% (-4.3 to 1.2)	21,054,343 (19,044,778 to 23,026,603)	-42.9% (-47.6 to -37.6)
**Middle SDI**	2,648,525 (2,240,569 to 3,082,587)	6.2% (1.5 to 10.8)	1,171,548 (1,037,070 to 1,296,280)	-22.4% (-32.7 to -11.5)	22,230,671 (20,239,639 to 24,119,856)	9.5% (7.0 to 11.9)	23,896,857 (21,522,553 to 26,137,545)	-21.4% (-31.3 to -10.6)
**Low-middle SDI**	1,120,427 (971,630 to 1,273,722)	-7.6% (-11.4 to -3.5)	581,649 (517,711 to 657,998)	-13.7% (-22.1 to -3.0)	10,234,283 (9,418,964 to 11,004,022)	-1.7% (-3.6 to 0.2)	12,300,862 (11,013,179 to 13,998,937)	-13.9% (-22.4 to -4.1)
**Low SDI**	433,298 (374,565 to 494,218)	-12.9% (-16.3 to -9.3)	174,655 (149,333 to 216,891)	-13.5% (-23.0 to -1.8)	4,447,699 (4,174,215 to 4,699,709)	-9.8% (-11.5 to -8.1)	4,059,455 (3,492,447 to 4,963,612)	-14.9% (-23.9 to -4.5)
**Countries categorised by the GBD super-regions**								
**Central Europe, Eastern Europe, and Central Asia**	831,675 (719,788 to 950,858)	-27.5% (-30.4 to -24.7)	534,361 (488,931 to 567,417)	-46.9% (-49.3 to -44.3)	5,547,179 (5,147,377 to 5,945,980)	-11.9% (-14.2 to -9.4)	9,292,425 (8,664,271 to 9,856,616)	-44.6% (-47.3 to -42.0)
**High-income**	1,266,828 (1,118,369 to 1,425,018)	-43.1% (-45.6 to -40.1)	483,852 (393,659 to 531,249)	-68.0% (-70.4 to -66.8)	15,378,645 (14,523,811 to 16,303,767)	-16.1% (-18.3 to -13.7)	8,056,812 (7,030,179 to 8,868,133)	-61.8% (-64.0 to -59.9)
**Latin America and Caribbean**	357,281 (308,111 to 408,879)	-37.5% (-39.9 to -35.2)	144,351 (128,996 to 155,384)	-55.4% (-58.0 to -52.6)	3,570,761 (3,324,276 to 3,833,679)	-23.9% (-25.6 to -22.0)	2,634,525 (2,417,154 to 2,823,446)	-54.1% (-56.4 to -51.5)

**North Africa and Middle East**	461,146<br>(409,815 to 516,837)	-11.9%<br>(-16.3 to -7.4)	253,284<br>(220,812 to 283,441)	-30.9%<br>(-39.5 to -21.3)	4,366,507<br>(4,157,818 to 4,571,158)	-4.7%<br>(-6.8 to -2.5)	5,405,417<br>(4,711,954 to 6,041,794)	-31.5%<br>(-39.8 to -22.0)
**South Asia**	853,370<br>(727,638 to 990,209)	-15.8%<br>(-19.8 to -11.7)	441,296<br>(382,997 to 539,467)	-12.3%<br>(-24.0 to 1.6)	7,828,397<br>(6,997,065 to 8,621,267)	-5.4%<br>(-7.9 to -2.7)	9,193,297<br>(8,004,944 to 11,543,775)	-14.7%<br>(-25.8 to -1.9)
**Southeast Asia, East Asia, and Oceania**	3,522,664<br>(2,935,605 to 4,169,755)	25.8%<br>(19.9 to 31.5)	1,545,602<br>(1,326,302 to 1,757,131)	-13.2%<br>(-28.5 to 2.5)	27,668,295<br>(25,009,572 to 30,271,983)	24.2%<br>(20.9 to 27.6)	31,388,175<br>(27,400,608 to 35,226,001)	-13.4%<br>(-27.9 to 0.9)
**Sub-Saharan Africa**	511,485<br>(444,646 to 580,614)	-6.9%<br>(-10.4 to -3.1)	188,753<br>(163,454 to 221,022)	-7.2%<br>(-18.7 to 8.0)	5,585,101<br>(5,260,715 to 5,895,442)	-7.5%<br>(-9.1 to -5.8)	4,387,261<br>(3,828,654 to 5,082,038)	-8.8%<br>(-19.7 to 5.2)
**Countries categorised by the GBD regions**								
**Andean Latin America**	27,415<br>(24,042 to 31,116)	-27.1%<br>(-30.9 to -23.1)	9,794<br>(8,202 to 11,614)	-44.1%<br>(-52.9 to -33.7)	312,485<br>(297,506 to 327,200)	-14.8%<br>(-16.7 to -13.0)	183,296<br>(154,621 to 214,987)	-44.2%<br>(-52.7 to -34.7)
**Australasia**	28,160<br>(25,188 to 31,109)	-42.1%<br>(-45.9 to -38.5)	9,400<br>(7,588 to 10,375)	-69.2%<br>(-71.7 to -67.0)	296,951<br>(286,363 to 308,252)	-22.6%<br>(-24.6 to -20.4)	149,125<br>(129,769 to 166,039)	-65.1%<br>(-67.4 to -62.8)
**Caribbean**	36,042<br>(32,142 to 39,961)	-11.6%<br>(-15.1 to -8.0)	19,948<br>(17,601 to 22,403)	-31.2%<br>(-38.4 to -22.9)	327,165<br>(312,541 to 341,548)	-5.0%<br>(-7.1 to -3.0)	353,682<br>(313,629 to 399,101)	-27.4%<br>(-35.1 to -18.5)
**Central Asia**	102,573<br>(91,018 to 114,463)	-6.3%<br>(-11.7 to -1.3)	44,870<br>(40,632 to 48,966)	-13.2%<br>(-21.4 to -3.4)	866,712<br>(829,519 to 902,763)	-8.4%<br>(-10.3 to -6.3)	985,309<br>(892,229 to 1,076,484)	-16.6%<br>(-24.1 to -7.8)
**Central Europe**	238,905<br>(209,922 to 266,604)	-32.3%<br>(-34.5 to -30.0)	160,200<br>(144,922 to 171,306)	-53.5%<br>(-56.5 to -50.5)	1,631,106<br>(1,537,835 to 1,733,827)	-19.1%<br>(-21.4 to -17.2)	2,593,398<br>(2,385,309 to 2,776,480)	-52.1%<br>(-55.2 to -49.2)
**Central Latin America**	127,075<br>(110,155 to 144,196)	-35.4%<br>(-37.6 to -33.2)	43,027<br>(37,956 to 47,740)	-50.2%<br>(-54.6 to -44.9)	1,387,552<br>(1,296,756 to 1,484,316)	-23.8%<br>(-25.5 to -22.1)	804,658<br>(727,628 to 889,436)	-47.6%<br>(-52.1 to -42.7)
**Central Sub-Saharan Africa**	59,173<br>(51,496 to 68,041)	-11.1%<br>(-16.4 to -4.9)	18,859<br>(13,940 to 25,745)	-7.9%<br>(-27.8 to 16.2)	596,223<br>(568,898 to 624,229)	-10.6%<br>(-13.3 to -8.0)	447,161<br>(347,102 to 579,987)	-11.8%<br>(-28.7 to 8.1)
**East Asia**	2,850,090<br>(2,363,158 to 3,405,882)	33.0%<br>(25.7 to 40.3)	1,202,218<br>(1,010,916 to 1,397,915)	-15.3%<br>(-32.4 to 4.2)	21,503,823<br>(19,292,836 to 23,714,701)	31.5%<br>(27.4 to 35.7)	24,021,156<br>(20,420,316 to 27,562,229)	-15.7%<br>(-31.3 to 2.5)
**Eastern Europe**	490,197<br>(415,356 to 571,451)	-28.0%<br>(-31.4 to -24.6)	329,291<br>(299,911 to 356,035)	-45.9%<br>(-49.2 to -42.3)	3,049,360<br>(2,763,322 to 3,340,261)	-10.5%<br>(-13.6 to -6.9)	5,713,718<br>(5,294,961 to 6,142,848)	-43.3%<br>(-46.9 to -39.6)
**Eastern Sub-Saharan Africa**	179,318<br>(154,966 to 204,334)	-6.6%<br>(-10.8 to -2.1)	51,923<br>(43,067 to 61,814)	-10.6%<br>(-25.2 to 7.2)	1,869,299<br>(1,761,941 to 1,973,402)	-7.0%<br>(-8.7 to -5.0)	1,231,675<br>(1,050,099 to 1,440,686)	-11.0%<br>(-25.6 to 6.0)
**High-income Asia Pacific**	284,445<br>(249,283 to 322,678)	-46.6%<br>(-50.6 to -42.2)	112,785<br>(87,155 to 127,258)	-74.9%<br>(-77.2 to -73.3)	3,295,962<br>(3,048,493 to 3,555,086)	-24.7%<br>(-27.5 to -21.1)	1,862,337<br>(1,582,664 to 2,083,583)	-68.0%<br>(-70.4 to -65.8)
**High-income North America**	352,963<br>(299,100 to 413,294)	-36.8%<br>(-40.2 to -33.0)	126,353<br>(103,455 to 138,349)	-43.3%<br>(-46.3 to -41.3)	5,699,098<br>(5,260,863 to 6,152,298)	-4.2%<br>(-8.4 to 0.1)	2,384,648<br>(2,094,174 to 2,640,685)	-36.4%<br>(-38.9 to -34.2)
**North Africa and Middle East**	461,146<br>(409,815 to 516,837)	-11.9%<br>(-16.3 to -7.4)	253,284<br>(220,812 to 283,441)	-30.9%<br>(-39.5 to -21.3)	4,366,507<br>(4,157,818 to 4,571,158)	-4.7%<br>(-6.8 to -2.5)	5,405,417<br>(4,711,954 to 6,041,794)	-31.5%<br>(-39.8 to -22.0)
**Oceania**	5,311<br>(4,625 to 6,003)	-10.2%<br>(-15.2 to -4.5)	1,843<br>(1,488 to 2,389)	-16.4%<br>(-30.3 to 0.0)	59,506<br>(57,089 to 62,156)	-8.4%<br>(-10.6 to -6.1)	48,691<br>(40,675 to 59,299)	-15.8%<br>(-28.2 to -0.5)
**South Asia**	853,370<br>(727,638 to 990,209)	-15.8%<br>(-19.8 to -11.7)	441,296<br>(382,997 to 539,467)	-12.3%<br>(-24.0 to 1.6)	7,828,397<br>(6,997,065 to 8,621,267)	-5.4%<br>(-7.9 to -2.7)	9,193,297<br>(8,004,944 to 11,543,775)	-14.7%<br>(-25.8 to -1.9)
**Southeast Asia**	667,263<br>(582,281 to 763,035)	0.0%<br>(-3.8 to 4.0)	341,541<br>(293,259 to 391,953)	-6.2%<br>(-20.8 to 9.0)	6,104,966<br>(5,651,319 to 6,562,857)	-0.2%<br>(-2.1 to 1.8)	7,318,328<br>(6,238,954 to 8,317,300)	-6.6%<br>(-19.8 to 6.8)

**Southern Latin America**	52,070 (46,169 to 58,185)	-38.3% (-42.1 to -34.3)	19,658 (17,471 to 21,165)	-63.2% (-65.7 to -60.8)	546,569 (524,189 to 570,536)	-25.5% (-27.8 to -23.1)	352,925 (323,074 to 382,286)	-59.8% (-62.4 to -57.1)
**Southern Sub-Saharan Africa**	64,729 (54,456 to 74,991)	-0.3% (-4.3 to 4.6)	24,959 (22,677 to 27,158)	27.5% (14.6 to 52.4)	640,170 (583,548 to 694,855)	-12.0% (-15.3 to -8.8)	523,153 (480,110 to 569,334)	17.1% (6.5 to 34.7)
**Tropical Latin America**	166,749 (140,998 to 194,698)	-44.3% (-47.4 to -41.2)	71,582 (63,128 to 76,435)	-63.1% (-64.9 to -61.7)	1,543,560 (1,392,395 to 1,692,608)	-28.7% (-31.5 to -26.0)	1,292,888 (1,180,666 to 1,372,019)	-62.0% (-63.6 to -60.6)
**Western Europe**	549,191 (496,802 to 601,606)	-45.1% (-48.1 to -42.1)	215,656 (175,955 to 236,146)	-73.4% (-75.4 to -72.2)	5,540,066 (5,333,482 to 5,752,362)	-22.4% (-24.3 to -20.4)	3,307,777 (2,903,214 to 3,626,266)	-69.1% (-70.9 to -67.5)
**Western Sub-Saharan Africa**	208,265 (181,391 to 236,454)	-9.0% (-12.6 to -4.9)	93,012 (77,668 to 111,241)	-10.7% (-25.4 to 10.8)	2,479,408 (2,331,911 to 2,621,733)	-5.7% (-7.3 to -3.9)	2,185,272 (1,840,340 to 2,608,225)	-11.5% (-25.8 to 8.2)
**Countries in alphabetical order**								
**Afghanistan**	13,465 (11,936 to 15,296)	-6.9% (-13.6 to 0.7)	7,556 (5,695 to 10,192)	-8.0% (-29.1 to 16.7)	117,239 (111,184 to 123,129)	-3.9% (-8.5 to 0.4)	187,324 (140,596 to 255,648)	-9.2% (-30.5 to 16.3)
**Albania**	2,716 (2,339 to 3,155)	-11.1% (-16.5 to -5.1)	1,814 (1,353 to 2,401)	-17.2% (-41.5 to 14.2)	23,506 (22,182 to 24,759)	-10.7% (-13.8 to -7.4)	29,324 (22,395 to 37,641)	-24.1% (-43.8 to 1.5)
**Algeria**	40,525 (34,841 to 46,589)	-13.3% (-22.5 to -3.6)	19,111 (14,778 to 23,724)	-29.4% (-43.8 to -10.8)	369,746 (354,425 to 385,887)	-4.3% (-7.8 to -0.7)	364,823 (284,814 to 458,548)	-31.0% (-44.4 to -12.2)
**American Samoa**	35 (31 to 41)	-17.0% (-23.8 to -10.2)	12 (10 to 15)	-24.5% (-38.9 to -6.4)	398 (382 to 416)	-14.8% (-17.5 to -11.8)	289 (247 to 333)	-22.9% (-34.1 to -7.2)
**Andorra**	69 (59 to 83)	-31.1% (-36.9 to -25.6)	26 (18 to 33)	-52.1% (-66.4 to -33.9)	697 (661 to 733)	-21.9% (-24.8 to -18.9)	397 (310 to 497)	-49.6% (-62.7 to -34.7)
**Angola**	14,134 (12,326 to 16,327)	-11.2% (-19.3 to -2.0)	4,261 (3,365 to 5,331)	-2.7% (-26.6 to 28.3)	148,899 (142,446 to 156,124)	-7.2% (-11.1 to -3.4)	105,410 (84,803 to 128,113)	-7.9% (-29.0 to 18.6)
**Antigua and Barbuda**	65 (57 to 73)	-19.7% (-26.3 to -12.7)	32 (30 to 34)	-35.0% (-40.3 to -29.6)	614 (583 to 643)	-13.6% (-16.3 to -10.6)	552 (515 to 593)	-36.6% (-41.4 to -31.7)
**Argentina**	33,273 (29,391 to 37,720)	-37.0% (-42.0 to -31.7)	11,397 (10,147 to 12,308)	-65.1% (-67.7 to -62.3)	354,764 (339,411 to 370,887)	-23.9% (-26.8 to -20.8)	214,952 (197,251 to 233,692)	-60.2% (-63.2 to -57.0)
**Armenia**	3,606 (3,213 to 4,011)	-36.1% (-41.3 to -29.8)	2,199 (1,927 to 2,456)	-31.6% (-38.8 to -22.8)	34,443 (32,918 to 35,970)	-14.3% (-17.6 to -10.5)	41,205 (37,054 to 45,943)	-31.7% (-38.5 to -23.3)
**Australia**	23,830 (21,336 to 26,510)	-42.2% (-46.7 to -37.8)	7,568 (6,099 to 8,378)	-71.0% (-73.5 to -68.8)	252,326 (244,095 to 260,214)	-22.8% (-24.8 to -20.8)	121,464 (105,324 to 135,376)	-66.3% (-68.7 to -64.0)
**Austria**	13,175 (11,499 to 15,430)	-24.3% (-32.3 to -14.0)	3,185 (2,617 to 3,504)	-80.4% (-82.0 to -79.0)	153,609 (148,086 to 159,505)	15.6% (12.0 to 19.1)	60,500 (52,694 to 68,087)	-71.6% (-74.5 to -69.0)
**Azerbaijan**	9,015 (7,861 to 10,288)	8.3% (-1.6 to 18.9)	2,873 (2,214 to 3,658)	-13.7% (-39.4 to 22.2)	81,841 (77,847 to 85,845)	0.3% (-4.0 to 4.7)	67,046 (53,651 to 82,737)	-16.6% (-38.3 to 10.8)
**Bahamas**	240 (210 to 269)	-17.1% (-23.5 to -10.3)	93 (79 to 111)	-29.5% (-41.6 to -14.5)	2,513 (2,398 to 2,625)	-10.1% (-13.3 to -6.9)	1,756 (1,496 to 2,071)	-31.6% (-43.2 to -17.0)
**Bahrain**	492 (422 to 574)	-28.9% (-34.6 to -23.3)	211 (175 to 249)	-41.2% (-50.7 to -29.2)	6,883 (6,515 to 7,326)	-17.7% (-20.8 to -14.8)	5,170 (4,380 to 6,046)	-44.0% (-52.5 to -33.0)
**Bangladesh**	107,066 (92,201 to 123,095)	-0.8% (-8.9 to 8.3)	78,848 (61,369 to 101,644)	-6.2% (-26.0 to 23.2)	865,771 (820,024 to 914,545)	-3.0% (-6.7 to 0.9)	1,402,963 (1,086,243 to 1,841,188)	-14.5% (-32.1 to 10.6)

**Barbados**	345<br>(300 to 389)	-22.5%<br>(-28.6 to -15.8)	231<br>(190 to 274)	-39.2%<br>(-49.6 to -26.9)	3,004<br>(2,848 to 3,158)	-11.3%<br>(-15.3 to -6.8)	3,545<br>(2,900 to 4,235)	-38.1%<br>(-49.0 to -25.1)
**Belarus**	22,187<br>(19,277 to 25,305)	-23.4%<br>(-30.0 to -16.6)	12,332<br>(10,422 to 14,334)	-28.5%<br>(-39.0 to -16.5)	151,094<br>(142,724 to 160,615)	-8.8%<br>(-16.3 to 0.0)	226,406<br>(192,635 to 263,057)	-30.2%<br>(-40.1 to -18.6)
**Belgium**	12,982<br>(11,652 to 14,236)	-45.5%<br>(-51.4 to -38.5)	4,775<br>(3,803 to 5,321)	-73.9%<br>(-76.0 to -72.3)	123,050<br>(119,044 to 126,841)	-16.0%<br>(-20.9 to -10.7)	74,750<br>(64,855 to 82,816)	-68.5%<br>(-70.5 to -66.5)
**Belize**	157<br>(138 to 178)	-9.1%<br>(-16.9 to -0.4)	63<br>(56 to 71)	-11.3%<br>(-20.9 to 0.3)	1,631<br>(1,549 to 1,716)	-4.4%<br>(-8.0 to -1.0)	1,156<br>(1,025 to 1,285)	-13.9%<br>(-23.3 to -2.4)
**Benin**	5,459<br>(4,771 to 6,227)	-16.2%<br>(-22.6 to -8.0)	2,829<br>(2,305 to 3,544)	-11.7%<br>(-26.0 to 8.2)	63,633<br>(60,511 to 66,668)	-12.6%<br>(-15.5 to -9.2)	61,150<br>(50,505 to 75,402)	-13.9%<br>(-27.9 to 6.4)
**Bermuda**	67<br>(58 to 77)	-37.1%<br>(-41.8 to -32.1)	32<br>(27 to 39)	-60.1%<br>(-66.0 to -51.0)	672<br>(639 to 705)	-21.4%<br>(-24.0 to -18.4)	499<br>(431 to 592)	-59.0%<br>(-64.9 to -50.0)
**Bhutan**	390<br>(338 to 446)	-8.6%<br>(-16.0 to 0.7)	200<br>(156 to 245)	-15.3%<br>(-36.5 to 20.6)	3,425<br>(3,232 to 3,617)	-2.9%<br>(-6.5 to 1.3)	3,804<br>(3,003 to 4,604)	-19.2%<br>(-37.8 to 10.1)
**Bolivia (Plurinational State of)**	4,368<br>(3,717 to 5,067)	-23.0%<br>(-27.5 to -17.0)	2,142<br>(1,513 to 2,965)	-38.7%<br>(-52.5 to -18.9)	45,241<br>(42,796 to 47,846)	-19.5%<br>(-22.5 to -16.8)	40,844<br>(29,094 to 55,794)	-42.0%<br>(-55.0 to -22.9)
**Bosnia and Herzegovina**	8,674<br>(7,495 to 9,801)	-14.1%<br>(-21.4 to -5.9)	5,857<br>(4,690 to 6,913)	-27.1%<br>(-41.4 to -9.7)	63,409<br>(60,465 to 66,821)	-2.6%<br>(-6.6 to 2.1)	100,394<br>(82,555 to 116,938)	-29.0%<br>(-42.2 to -12.2)
**Botswana**	2,135<br>(1,833 to 2,452)	5.6%<br>(-3.7 to 16.2)	543<br>(443 to 676)	-32.4%<br>(-47.8 to -8.5)	19,280<br>(18,419 to 20,183)	1.1%<br>(-3.0 to 5.4)	12,403<br>(10,368 to 15,136)	-29.0%<br>(-44.1 to -6.7)
**Brazil**	162,711<br>(137,407 to 190,295)	-44.7%<br>(-47.9 to -41.6)	69,595<br>(61,559 to 74,395)	-63.7%<br>(-65.4 to -62.3)	1,507,567<br>(1,357,108 to 1,655,969)	-29.0%<br>(-31.8 to -26.2)	1,258,850<br>(1,149,139 to 1,335,368)	-62.5%<br>(-64.0 to -61.1)
**Brunei Darussalam**	318<br>(271 to 368)	-44.5%<br>(-49.3 to -39.3)	69<br>(59 to 81)	-49.6%<br>(-57.7 to -39.2)	3,372<br>(3,222 to 3,526)	-40.8%<br>(-42.8 to -38.7)	1,753<br>(1,528 to 2,002)	-52.3%<br>(-59.2 to -43.8)
**Bulgaria**	23,741<br>(20,664 to 26,748)	-3.1%<br>(-10.5 to 5.2)	21,792<br>(19,148 to 24,451)	-18.7%<br>(-27.6 to -9.2)	137,219<br>(124,625 to 150,670)	-2.7%<br>(-9.9 to 4.2)	352,137<br>(310,468 to 397,947)	-15.9%<br>(-25.2 to -5.1)
**Burkina Faso**	7,184<br>(6,206 to 8,258)	-6.6%<br>(-13.7 to 1.0)	3,120<br>(2,416 to 4,062)	0.9%<br>(-19.3 to 26.1)	86,465<br>(82,444 to 90,432)	-8.3%<br>(-11.5 to -5.6)	71,547<br>(56,989 to 91,531)	-1.8%<br>(-21.1 to 22.4)
**Burundi**	5,160<br>(4,468 to 5,998)	-28.2%<br>(-34.3 to -21.2)	1,576<br>(1,157 to 2,135)	-37.5%<br>(-53.3 to -15.3)	53,274<br>(50,745 to 55,846)	-25.6%<br>(-28.3 to -22.8)	37,840<br>(29,089 to 48,885)	-39.8%<br>(-54.2 to -20.3)
**Cv¥te d'Ivoire**	12,507<br>(10,917 to 14,192)	-14.2%<br>(-21.1 to -6.7)	5,713<br>(4,348 to 7,458)	-7.1%<br>(-25.4 to 19.0)	152,892<br>(146,002 to 159,948)	-15.0%<br>(-17.6 to -12.0)	144,264<br>(110,164 to 188,375)	-9.0%<br>(-27.5 to 17.4)
**Cabo Verde**	386<br>(337 to 445)	8.3%<br>(0.4 to 18.2)	268<br>(219 to 325)	32.4%<br>(3.9 to 74.9)	4,780<br>(4,589 to 4,996)	-0.7%<br>(-3.8 to 2.5)	5,030<br>(4,171 to 5,937)	21.7%<br>(-4.3 to 55.4)
**Cambodia**	11,185<br>(9,612 to 12,804)	2.1%<br>(-6.3 to 11.9)	6,007<br>(4,680 to 7,254)	-1.9%<br>(-24.1 to 23.1)	92,092<br>(87,597 to 96,723)	-0.4%<br>(-4.6 to 4.3)	122,669<br>(99,132 to 146,213)	-8.8%<br>(-29.1 to 12.4)
**Cameroon**	12,998<br>(11,470 to 14,823)	3.9%<br>(-3.7 to 13.8)	5,974<br>(4,480 to 8,270)	10.2%<br>(-14.7 to 44.8)	154,862<br>(148,047 to 161,887)	-1.0%<br>(-4.3 to 2.4)	142,484<br>(108,965 to 189,763)	10.3%<br>(-13.7 to 44.8)
**Canada**	41,315<br>(37,976 to 44,826)	-39.7%<br>(-45.3 to -32.6)	10,781<br>(8,823 to 11,889)	-63.9%<br>(-66.7 to -61.3)	562,109<br>(548,506 to 574,789)	-10.5%<br>(-13.4 to -7.5)	206,238<br>(180,736 to 231,056)	-53.7%<br>(-57.0 to -50.6)
**Central African Republic**	2,378<br>(2,033 to 2,760)	-6.5%<br>(-14.4 to 2.3)	797<br>(562 to 1,142)	-8.9%<br>(-29.1 to 13.2)	23,617<br>(22,430 to 24,842)	-8.3%<br>(-12.1 to -4.7)	20,563<br>(14,922 to 28,243)	-11.5%<br>(-29.7 to 9.7)

**Chad**	6,548 (5,692 to 7,465)	-4.5% (-11.7 to 3.0)	3,200 (2,399 to 4,271)	14.9% (-10.1 to 50.5)	75,881 (72,357 to 78,950)	-5.1% (-8.2 to -2.0)	78,401 (61,185 to 101,443)	13.2% (-10.3 to 45.6)
**Chile**	14,875 (12,895 to 17,007)	-37.1% (-42.6 to -31.7)	5,943 (5,229 to 6,456)	-61.2% (-64.2 to -58.4)	155,202 (148,079 to 162,378)	-21.5% (-24.3 to -18.3)	102,550 (92,831 to 112,233)	-59.9% (-62.5 to -57.5)
**China**	2,772,053 (2,295,713 to 3,319,150)	35.7% (27.9 to 43.7)	1,176,952 (986,876 to 1,372,707)	-14.3% (-31.9 to 6.1)	20,803,932 (18,615,867 to 22,995,491)	34.2% (29.9 to 38.8)	23,430,411 (19,918,946 to 26,933,909)	-14.9% (-30.9 to 4.2)
**Colombia**	25,862 (22,399 to 29,348)	-45.3% (-49.9 to -40.3)	8,179 (6,817 to 9,465)	-62.1% (-67.9 to -55.8)	273,597 (261,970 to 286,798)	-33.8% (-35.9 to -31.2)	150,357 (129,215 to 173,444)	-60.7% (-66.0 to -54.8)
**Comoros**	531 (460 to 605)	-17.3% (-25.0 to -9.7)	173 (131 to 225)	-27.0% (-44.4 to -2.9)	5,561 (5,307 to 5,827)	-16.1% (-19.1 to -12.9)	3,796 (3,015 to 4,786)	-28.3% (-44.8 to -7.0)
**Congo**	3,397 (2,934 to 3,894)	-14.7% (-22.1 to -6.9)	1,097 (847 to 1,345)	-16.8% (-33.4 to 3.0)	34,965 (33,300 to 36,550)	-14.0% (-17.3 to -10.4)	26,289 (20,866 to 31,739)	-18.9% (-34.6 to -1.8)
**Cook Islands**	19 (17 to 22)	-7.0% (-14.1 to 0.9)	6 (5 to 7)	-45.9% (-58.2 to -31.3)	223 (214 to 232)	2.4% (-1.0 to 5.9)	131 (107 to 155)	-38.3% (-49.7 to -25.6)
**Costa Rica**	2,724 (2,343 to 3,138)	-28.3% (-33.6 to -22.6)	904 (758 to 1,017)	-40.0% (-46.8 to -33.2)	29,273 (27,852 to 30,793)	-16.5% (-19.4 to -13.4)	15,108 (13,261 to 16,627)	-39.9% (-46.2 to -34.4)
**Croatia**	9,245 (8,638 to 9,821)	-38.6% (-42.1 to -35.1)	5,005 (4,355 to 5,622)	-65.6% (-69.2 to -61.9)	64,357 (62,236 to 66,261)	-15.1% (-21.1 to -8.9)	77,608 (69,180 to 86,416)	-64.8% (-68.0 to -60.9)
**Cuba**	11,976 (10,625 to 13,408)	-13.0% (-19.2 to -5.3)	7,357 (6,482 to 8,238)	-21.3% (-31.4 to -10.7)	104,406 (99,314 to 109,629)	-8.3% (-11.9 to -4.4)	119,030 (105,556 to 132,769)	-19.3% (-29.5 to -8.5)
**Cyprus**	781 (689 to 879)	-51.8% (-57.1 to -46.2)	512 (420 to 599)	-73.3% (-78.8 to -67.1)	6,094 (5,641 to 6,734)	-44.3% (-47.5 to -40.4)	7,164 (6,024 to 8,303)	-73.7% (-78.6 to -68.2)
**Czechia**	19,392 (16,684 to 22,281)	-52.4% (-56.9 to -47.4)	7,306 (6,383 to 8,175)	-80.7% (-82.7 to -78.5)	161,271 (154,895 to 168,609)	-25.2% (-29.4 to -20.7)	129,117 (114,221 to 143,143)	-77.9% (-80.2 to -75.3)
**Democratic People's Republic of Korea**	43,909 (38,065 to 50,459)	3.9% (-5.2 to 14.4)	18,869 (14,851 to 24,754)	-7.0% (-25.8 to 18.4)	346,744 (330,062 to 365,847)	-0.7% (-4.8 to 3.7)	429,296 (341,946 to 548,679)	-3.8% (-22.7 to 20.9)
**Democratic Republic of the Congo**	37,504 (32,504 to 43,159)	-11.4% (-18.0 to -3.8)	12,127 (8,087 to 17,786)	-8.1% (-32.3 to 21.3)	369,929 (351,659 to 388,421)	-11.9% (-15.7 to -8.5)	281,741 (206,383 to 389,246)	-12.1% (-33.4 to 13.9)
**Denmark**	6,056 (5,212 to 6,934)	-51.5% (-55.7 to -46.4)	2,691 (2,258 to 2,930)	-61.5% (-64.5 to -58.8)	61,099 (58,488 to 63,609)	-38.0% (-40.3 to -35.6)	41,702 (36,920 to 45,444)	-61.6% (-64.0 to -59.1)
**Djibouti**	778 (675 to 898)	-2.0% (-10.9 to 7.5)	189 (140 to 261)	-3.5% (-28.4 to 32.9)	8,253 (7,881 to 8,620)	-4.3% (-7.8 to -0.7)	4,947 (3,805 to 6,550)	-4.4% (-27.8 to 27.2)
**Dominica**	50 (43 to 57)	-8.1% (-15.9 to -0.2)	40 (35 to 45)	-18.5% (-30.0 to -5.0)	439 (418 to 462)	-9.7% (-13.1 to -6.5)	646 (565 to 743)	-18.2% (-29.4 to -4.0)
**Dominican Republic**	7,958 (6,951 to 9,042)	22.2% (14.9 to 31.5)	3,264 (2,579 to 4,128)	-16.7% (-36.4 to 10.2)	75,674 (72,191 to 79,237)	12.6% (8.6 to 16.6)	62,109 (49,573 to 77,698)	-8.6% (-29.0 to 20.2)
**Ecuador**	8,683 (7,648 to 9,804)	-22.1% (-27.1 to -16.3)	2,789 (2,322 to 3,318)	-46.6% (-55.1 to -36.8)	97,528 (92,742 to 101,861)	-17.2% (-19.9 to -14.5)	50,780 (42,637 to 59,967)	-50.2% (-58.1 to -41.2)
**Egypt**	83,090 (73,098 to 95,678)	15.7% (7.7 to 28.2)	54,667 (42,714 to 68,604)	-23.5% (-39.9 to -2.5)	737,060 (702,531 to 773,598)	21.6% (14.6 to 27.7)	1,266,972 (984,970 to 1,597,188)	-20.7% (-38.4 to 2.5)
**El Salvador**	3,006 (2,631 to 3,409)	-24.5% (-29.9 to -18.5)	1,085 (870 to 1,316)	-36.6% (-49.9 to -22.6)	29,410 (27,945 to 30,946)	-17.2% (-20.0 to -14.0)	18,912 (15,666 to 22,329)	-38.7% (-50.7 to -26.1)

**Equatorial Guinea**	550<br>(470 to 637)	-22.5%<br>(-29.5 to -15.2)	182<br>(123 to 255)	-19.9%<br>(-47.6 to 18.5)	6,168<br>(5,880 to 6,497)	-11.6%<br>(-14.8 to -8.4)	4,281<br>(3,089 to 5,789)	-23.4%<br>(-47.9 to 9.3)
**Eritrea**	3,074<br>(2,661 to 3,597)	-14.8%<br>(-21.5 to -7.0)	823<br>(600 to 1,094)	-10.5%<br>(-30.5 to 19.4)	32,509<br>(30,979 to 34,009)	-7.1%<br>(-10.7 to -3.1)	20,981<br>(16,072 to 27,166)	-14.9%<br>(-32.0 to 9.3)
**Estonia**	1,888<br>(1,640 to 2,159)	-57.9%<br>(-62.2 to -53.1)	886<br>(754 to 994)	-80.7%<br>(-83.1 to -78.5)	15,108<br>(14,188 to 16,052)	-28.0%<br>(-33.5 to -22.5)	14,986<br>(13,200 to 16,732)	-78.3%<br>(-80.8 to -76.1)
**Eswatini**	662<br>(566 to 761)	11.2%<br>(0.8 to 21.6)	256<br>(181 to 351)	1.5%<br>(-27.3 to 38.3)	6,050<br>(5,763 to 6,354)	1.3%<br>(-2.5 to 5.8)	5,825<br>(4,257 to 7,901)	4.1%<br>(-22.7 to 41.1)
**Ethiopia**	31,926<br>(26,737 to 37,066)	-22.9%<br>(-27.1 to -18.3)	8,533<br>(6,624 to 11,236)	-21.5%<br>(-48.0 to 5.7)	338,614<br>(304,513 to 374,421)	-19.8%<br>(-23.6 to -15.9)	195,828<br>(158,179 to 247,585)	-25.5%<br>(-49.1 to -3.4)
**Fiji**	696<br>(608 to 799)	-13.5%<br>(-20.5 to -5.6)	200<br>(159 to 250)	-6.1%<br>(-26.6 to 22.4)	7,860<br>(7,535 to 8,189)	-10.2%<br>(-13.0 to -6.9)	5,262<br>(4,286 to 6,298)	-11.6%<br>(-27.8 to 10.8)
**Finland**	9,251<br>(8,181 to 10,433)	-44.4%<br>(-48.9 to -39.7)	3,232<br>(2,600 to 3,593)	-67.7%<br>(-70.9 to -65.1)	96,833<br>(93,437 to 100,116)	-24.7%<br>(-26.8 to -22.8)	52,985<br>(46,211 to 58,598)	-65.2%<br>(-67.9 to -62.7)
**France**	70,227<br>(64,649 to 76,089)	-21.9%<br>(-26.9 to -16.8)	28,514<br>(23,424 to 31,457)	-70.2%<br>(-72.7 to -68.2)	740,251<br>(720,510 to 762,133)	-1.2%<br>(-5.4 to 2.9)	425,563<br>(370,417 to 471,781)	-62.5%<br>(-65.0 to -59.7)
**Gabon**	1,209<br>(1,037 to 1,381)	-9.7%<br>(-18.2 to -0.6)	395<br>(297 to 502)	-8.2%<br>(-28.7 to 20.1)	12,646<br>(12,128 to 13,214)	-12.1%<br>(-15.2 to -8.3)	8,876<br>(6,884 to 11,022)	-10.9%<br>(-28.3 to 11.2)
**Gambia**	1,156<br>(1,015 to 1,315)	-4.9%<br>(-11.5 to 4.2)	674<br>(497 to 881)	13.2%<br>(-16.5 to 49.1)	13,514<br>(12,927 to 14,066)	-8.1%<br>(-11.3 to -5.0)	14,913<br>(11,386 to 19,175)	7.4%<br>(-20.1 to 41.5)
**Georgia**	7,503<br>(6,565 to 8,391)	5.1%<br>(-4.8 to 15.6)	6,374<br>(5,661 to 7,047)	23.1%<br>(4.5 to 50.0)	50,798<br>(47,962 to 53,894)	8.3%<br>(4.2 to 12.7)	103,988<br>(93,106 to 114,540)	21.7%<br>(3.3 to 48.8)
**Germany**	151,528<br>(134,230 to 167,831)	-39.4%<br>(-44.6 to -34.1)	43,942<br>(36,060 to 48,549)	-74.4%<br>(-76.5 to -72.4)	1,660,910<br>(1,606,057 to 1,709,638)	-19.0%<br>(-21.1 to -16.6)	797,270<br>(701,814 to 883,712)	-66.8%<br>(-69.3 to -64.2)
**Ghana**	26,184<br>(23,176 to 29,266)	2.3%<br>(-7.1 to 11.0)	11,748<br>(9,003 to 14,986)	5.1%<br>(-20.4 to 38.4)	313,625<br>(301,341 to 327,553)	2.3%<br>(-1.3 to 5.7)	285,112<br>(226,667 to 358,224)	2.3%<br>(-20.7 to 31.7)
**Greece**	19,139<br>(16,787 to 21,384)	-44.2%<br>(-49.5 to -38.5)	10,915<br>(9,136 to 11,881)	-71.4%<br>(-73.4 to -69.9)	140,778<br>(134,169 to 148,220)	-23.7%<br>(-27.3 to -20.0)	141,981<br>(123,221 to 154,800)	-69.0%<br>(-70.7 to -67.4)
**Greenland**	56<br>(49 to 66)	-53.2%<br>(-56.6 to -49.1)	15<br>(12 to 18)	-64.9%<br>(-71.3 to -56.1)	645<br>(615 to 673)	-41.4%<br>(-44.5 to -38.9)	319<br>(269 to 384)	-63.8%<br>(-69.7 to -56.6)
**Grenada**	87<br>(78 to 98)	-18.6%<br>(-25.5 to -12.0)	45<br>(40 to 49)	-42.8%<br>(-50.1 to -34.7)	784<br>(720 to 851)	-17.0%<br>(-22.0 to -11.3)	817<br>(729 to 897)	-48.4%<br>(-55.4 to -40.7)
**Guam**	157<br>(138 to 178)	-18.0%<br>(-24.8 to -11.0)	26<br>(21 to 30)	-72.7%<br>(-77.3 to -67.4)	1,950<br>(1,869 to 2,024)	-0.3%<br>(-3.7 to 3.2)	858<br>(729 to 996)	-50.3%<br>(-57.0 to -43.0)
**Guatemala**	5,070<br>(4,441 to 5,755)	-20.3%<br>(-26.7 to -13.1)	1,654<br>(1,441 to 1,878)	-40.4%<br>(-48.4 to -30.4)	52,159<br>(49,607 to 54,918)	-16.1%<br>(-19.0 to -12.5)	30,378<br>(26,766 to 34,395)	-41.5%<br>(-49.6 to -31.7)
**Guinea**	6,410<br>(5,655 to 7,214)	0.2%<br>(-7.6 to 9.2)	3,477<br>(2,591 to 4,566)	12.7%<br>(-13.9 to 53.9)	72,860<br>(69,721 to 76,039)	-0.3%<br>(-3.8 to 3.2)	78,602<br>(59,721 to 101,610)	11.3%<br>(-14.5 to 49.0)
**Guinea-Bissau**	935<br>(811 to 1,071)	-6.1%<br>(-14.9 to 2.4)	472<br>(366 to 612)	-1.1%<br>(-23.8 to 26.4)	11,030<br>(10,545 to 11,515)	-7.9%<br>(-11.0 to -4.8)	12,278<br>(9,557 to 15,699)	-6.4%<br>(-27.4 to 20.7)
**Guyana**	588<br>(517 to 666)	-27.7%<br>(-32.4 to -22.4)	344<br>(281 to 421)	-37.2%<br>(-49.6 to -23.7)	5,073<br>(4,836 to 5,346)	-21.5%<br>(-25.1 to -17.9)	6,731<br>(5,447 to 8,268)	-42.3%<br>(-54.2 to -29.1)

**Haiti**	6,038<br>(5,299 to 6,899)	-10.7%<br>(-17.0 to -3.8)	3,979<br>(2,871 to 5,532)	-23.6%<br>(-41.2 to -2.2)	52,968<br>(50,226 to 55,759)	-8.9%<br>(-12.7 to -5.2)	84,370<br>(60,496 to 118,305)	-25.4%<br>(-43.1 to -3.1)
**Honduras**	3,689<br>(3,174 to 4,231)	5.3%<br>(-2.9 to 12.9)	2,605<br>(2,002 to 3,350)	32.4%<br>(5.9 to 69.8)	35,480<br>(33,693 to 37,426)	-8.1%<br>(-11.6 to -4.8)	48,553<br>(37,815 to 62,100)	20.6%<br>(-2.2 to 52.1)
**Hungary**	18,242<br>(16,024 to 20,714)	-49.4%<br>(-53.3 to -44.6)	9,325<br>(8,066 to 10,520)	-68.3%<br>(-72.3 to -64.3)	141,662<br>(134,388 to 149,052)	-38.8%<br>(-41.3 to -35.9)	165,518<br>(145,127 to 185,316)	-65.9%<br>(-69.8 to -61.9)
**Iceland**	305<br>(263 to 354)	-50.7%<br>(-55.9 to -45.2)	99<br>(77 to 113)	-66.1%<br>(-69.6 to -62.2)	3,292<br>(3,150 to 3,423)	-31.6%<br>(-34.0 to -29.1)	1,581<br>(1,355 to 1,780)	-64.1%<br>(-67.1 to -60.7)
**India**	626,033<br>(529,532 to 730,397)	-18.9%<br>(-23.1 to -14.9)	313,668<br>(267,425 to 393,593)	-12.7%<br>(-26.7 to 1.7)	5,797,618<br>(5,134,722 to 6,439,023)	-5.7%<br>(-8.5 to -2.6)	6,680,576<br>(5,770,847 to 8,536,838)	-15.4%<br>(-27.9 to -1.7)
**Indonesia**	306,628<br>(258,913 to 363,145)	17.3%<br>(12.0 to 23.5)	148,107<br>(111,694 to 183,952)	36.8%<br>(4.0 to 72.7)	2,721,699<br>(2,432,158 to 3,036,175)	2.1%<br>(-0.9 to 5.4)	3,339,844<br>(2,536,606 to 4,124,779)	24.9%<br>(-1.4 to 53.2)
**Iran (Islamic Republic of)**	62,281<br>(53,414 to 72,121)	-30.0%<br>(-33.8 to -26.8)	34,027<br>(30,355 to 37,132)	-46.9%<br>(-51.9 to -40.9)	653,016<br>(591,272 to 718,461)	-12.7%<br>(-15.9 to -9.0)	683,977<br>(625,155 to 745,106)	-47.5%<br>(-52.3 to -42.0)
**Iraq**	32,006<br>(28,248 to 36,542)	2.3%<br>(-6.6 to 11.2)	20,402<br>(16,163 to 24,219)	-1.5%<br>(-22.4 to 21.3)	284,908<br>(271,715 to 297,853)	-0.3%<br>(-4.2 to 4.3)	443,210<br>(350,564 to 534,121)	-12.5%<br>(-32.5 to 10.4)
**Ireland**	3,086<br>(2,688 to 3,582)	-62.6%<br>(-65.9 to -58.9)	1,268<br>(1,013 to 1,416)	-76.9%<br>(-79.8 to -74.9)	29,124<br>(27,487 to 30,762)	-46.7%<br>(-49.1 to -43.9)	18,924<br>(16,186 to 20,965)	-76.4%<br>(-78.4 to -74.5)
**Israel**	5,773<br>(4,961 to 6,693)	-52.5%<br>(-56.9 to -48.1)	1,592<br>(1,303 to 1,766)	-69.8%<br>(-72.9 to -67.1)	74,015<br>(71,007 to 77,111)	-30.9%<br>(-33.1 to -28.6)	29,592<br>(25,346 to 33,191)	-64.5%<br>(-66.9 to -61.6)
**Italy**	67,741<br>(59,294 to 77,123)	-56.4%<br>(-60.8 to -50.9)	41,773<br>(32,901 to 46,706)	-70.9%<br>(-73.4 to -69.4)	569,282<br>(512,598 to 629,688)	-29.5%<br>(-32.5 to -25.7)	539,857<br>(447,429 to 596,628)	-70.2%<br>(-72.1 to -68.6)
**Jamaica**	2,230<br>(1,952 to 2,466)	-15.7%<br>(-22.5 to -7.9)	1,624<br>(1,293 to 1,988)	-29.1%<br>(-43.3 to -12.4)	16,218<br>(15,403 to 17,150)	-11.0%<br>(-14.7 to -7.7)	24,038<br>(19,469 to 29,433)	-30.5%<br>(-44.6 to -12.9)
**Japan**	212,866<br>(184,985 to 243,882)	-41.1%<br>(-45.9 to -34.4)	88,917<br>(67,686 to 100,981)	-74.9%<br>(-76.9 to -73.5)	2,455,645<br>(2,232,199 to 2,694,573)	-17.4%<br>(-21.4 to -12.1)	1,409,376<br>(1,190,419 to 1,583,340)	-66.1%<br>(-68.7 to -63.6)
**Jordan**	9,791<br>(8,604 to 10,979)	-17.8%<br>(-24.9 to -9.4)	2,630<br>(2,105 to 3,182)	-50.6%<br>(-61.5 to -36.5)	97,941<br>(93,499 to 102,010)	0.7%<br>(-2.9 to 5.4)	64,418<br>(53,929 to 76,318)	-50.5%<br>(-61.1 to -37.0)
**Kazakhstan**	25,493<br>(22,256 to 28,888)	-19.7%<br>(-27.1 to -12.2)	13,175<br>(11,609 to 14,822)	-15.2%<br>(-25.9 to -0.6)	223,075<br>(213,059 to 233,879)	-19.8%<br>(-23.1 to -16.3)	270,137<br>(235,800 to 304,369)	-23.6%<br>(-33.2 to -11.3)
**Kenya**	22,370<br>(19,009 to 26,101)	-0.5%<br>(-5.2 to 4.6)	5,802<br>(4,316 to 7,372)	13.3%<br>(-9.0 to 41.4)	242,424<br>(218,340 to 267,619)	-6.1%<br>(-8.4 to -3.7)	137,041<br>(110,726 to 165,865)	8.2%<br>(-9.9 to 29.5)
**Kiribati**	85<br>(75 to 97)	-6.6%<br>(-13.3 to 0.8)	24<br>(19 to 29)	6.3%<br>(-13.6 to 40.2)	925<br>(886 to 966)	-9.1%<br>(-12.4 to -6.1)	690<br>(568 to 831)	-0.7%<br>(-16.6 to 24.7)
**Kuwait**	2,494<br>(2,188 to 2,826)	-9.6%<br>(-16.5 to -3.0)	539<br>(445 to 646)	-28.2%<br>(-39.4 to -15.5)	32,360<br>(30,771 to 33,928)	-7.7%<br>(-10.9 to -4.1)	14,657<br>(12,519 to 17,060)	-30.2%<br>(-39.3 to -19.6)
**Kyrgyzstan**	5,085<br>(4,471 to 5,827)	-33.1%<br>(-39.6 to -25.6)	2,323<br>(1,976 to 2,684)	-48.1%<br>(-56.5 to -38.9)	41,747<br>(39,666 to 43,806)	-28.9%<br>(-31.9 to -25.8)	58,321<br>(49,602 to 66,795)	-40.2%<br>(-48.9 to -29.7)
**Lao People's Democratic Republic**	4,719<br>(4,108 to 5,383)	-7.8%<br>(-16.1 to 2.6)	2,496<br>(1,917 to 3,134)	-27.1%<br>(-45.8 to -3.8)	42,189<br>(40,150 to 44,221)	-4.3%<br>(-8.2 to -0.4)	55,537<br>(43,016 to 69,493)	-30.7%<br>(-48.3 to -8.1)
**Latvia**	5,679<br>(5,047 to 6,349)	-33.2%<br>(-38.7 to -27.3)	4,304<br>(3,770 to 4,748)	-40.8%<br>(-46.5 to -34.5)	34,613<br>(32,364 to 36,543)	-6.4%<br>(-13.1 to 2.6)	65,466<br>(58,444 to 72,264)	-41.8%<br>(-47.8 to -35.9)

**Lebanon**	5,676<br>(4,934 to 6,390)	-14.7%<br>(-21.0 to -7.3)	1,910<br>(1,572 to 2,245)	-62.6%<br>(-72.5 to -51.6)	52,502<br>(49,631 to 55,544)	7.6%<br>(4.0 to 11.8)	34,130<br>(29,288 to 38,742)	-60.1%<br>(-70.1 to -48.9)
**Lesotho**	1,311<br>(1,105 to 1,530)	40.6%<br>(28.8 to 56.5)	654<br>(484 to 855)	55.8%<br>(10.6 to 120.0)	10,559<br>(9,987 to 11,121)	20.4%<br>(15.0 to 26.2)	13,901<br>(10,484 to 18,450)	58.9%<br>(15.3 to 119.2)
**Liberia**	2,131<br>(1,848 to 2,435)	-19.2%<br>(-25.3 to -11.6)	1,067<br>(798 to 1,404)	-4.1%<br>(-24.6 to 24.1)	25,788<br>(24,579 to 26,941)	-14.6%<br>(-17.4 to -11.7)	25,079<br>(19,026 to 32,952)	-7.1%<br>(-26.9 to 19.2)
**Libya**	4,785<br>(4,256 to 5,402)	14.9%<br>(6.1 to 25.0)	2,355<br>(1,727 to 3,163)	10.0%<br>(-16.5 to 45.6)	52,232<br>(49,804 to 54,509)	10.0%<br>(6.2 to 13.9)	56,023<br>(42,323 to 73,821)	12.2%<br>(-13.2 to 45.1)
**Lithuania**	8,869<br>(7,639 to 10,148)	-28.0%<br>(-34.8 to -21.9)	3,783<br>(3,338 to 4,184)	-29.3%<br>(-37.5 to -22.1)	43,581<br>(37,885 to 49,795)	-5.3%<br>(-17.2 to 7.4)	60,850<br>(54,067 to 67,264)	-33.9%<br>(-41.1 to -27.4)
**Luxembourg**	441<br>(399 to 483)	-58.8%<br>(-62.1 to -55.0)	203<br>(173 to 225)	-83.2%<br>(-84.8 to -81.5)	4,081<br>(3,906 to 4,240)	-42.0%<br>(-47.0 to -36.2)	2,958<br>(2,603 to 3,257)	-81.4%<br>(-82.9 to -79.8)
**Madagascar**	16,080<br>(14,010 to 18,498)	-1.0%<br>(-9.1 to 7.0)	4,490<br>(3,209 to 5,882)	-6.8%<br>(-30.0 to 19.6)	171,927<br>(163,837 to 179,173)	-3.3%<br>(-6.6 to -0.1)	117,563<br>(89,508 to 149,247)	-8.2%<br>(-29.4 to 16.3)
**Malawi**	8,415<br>(7,318 to 9,673)	-5.4%<br>(-13.4 to 3.3)	3,219<br>(2,532 to 4,042)	11.6%<br>(-10.1 to 39.3)	87,603<br>(83,267 to 91,842)	-7.1%<br>(-10.7 to -3.4)	74,119<br>(59,846 to 91,545)	8.3%<br>(-11.0 to 34.3)
**Malaysia**	26,669<br>(23,128 to 30,696)	-19.7%<br>(-25.9 to -12.4)	9,487<br>(8,311 to 10,673)	-18.7%<br>(-30.9 to -4.6)	280,509<br>(268,072 to 293,768)	-1.9%<br>(-5.3 to 1.7)	222,302<br>(197,108 to 248,126)	-21.9%<br>(-31.1 to -11.9)
**Maldives**	263<br>(228 to 301)	-39.6%<br>(-44.6 to -34.3)	101<br>(83 to 118)	-49.6%<br>(-59.4 to -38.3)	2,664<br>(2,534 to 2,802)	-37.1%<br>(-39.6 to -34.5)	2,052<br>(1,728 to 2,363)	-54.7%<br>(-62.6 to -46.3)
**Mali**	7,566<br>(6,539 to 8,777)	-16.2%<br>(-21.9 to -9.2)	3,249<br>(2,413 to 4,520)	-5.9%<br>(-25.0 to 20.0)	90,828<br>(86,421 to 94,968)	-12.6%<br>(-15.4 to -9.9)	79,037<br>(60,052 to 106,759)	-7.4%<br>(-26.2 to 17.9)
**Malta**	377<br>(327 to 433)	-59.6%<br>(-62.9 to -56.0)	179<br>(146 to 202)	-75.2%<br>(-77.8 to -72.4)	3,324<br>(3,150 to 3,501)	-40.1%<br>(-43.4 to -36.9)	2,677<br>(2,300 to 2,981)	-74.6%<br>(-76.8 to -72.3)
**Marshall Islands**	34<br>(29 to 39)	-3.4%<br>(-10.5 to 3.3)	10<br>(7 to 13)	-13.6%<br>(-31.9 to 8.6)	366<br>(349 to 383)	-3.8%<br>(-7.3 to -0.2)	283<br>(219 to 369)	-12.4%<br>(-29.6 to 6.8)
**Mauritania**	2,176<br>(1,905 to 2,478)	-27.5%<br>(-33.3 to -21.6)	1,199<br>(873 to 1,680)	-18.6%<br>(-37.0 to 5.4)	26,222<br>(25,044 to 27,418)	-23.0%<br>(-25.4 to -20.5)	25,194<br>(19,100 to 34,546)	-23.5%<br>(-39.5 to -2.2)
**Mauritius**	1,384<br>(1,183 to 1,588)	-48.3%<br>(-52.8 to -43.4)	586<br>(534 to 622)	-64.6%<br>(-67.0 to -62.0)	12,789<br>(12,208 to 13,374)	-37.7%<br>(-40.1 to -35.2)	12,492<br>(11,539 to 13,341)	-63.9%<br>(-66.4 to -61.7)
**Mexico**	65,181<br>(55,026 to 75,805)	-37.0%<br>(-39.8 to -33.7)	19,544<br>(17,332 to 21,720)	-57.4%<br>(-61.4 to -53.4)	757,765<br>(687,567 to 832,271)	-22.9%<br>(-25.5 to -20.1)	385,010<br>(347,021 to 430,103)	-52.2%<br>(-56.4 to -47.6)
**Micronesia (Federated States of)**	72<br>(62 to 83)	-6.4%<br>(-13.9 to 1.8)	24<br>(19 to 33)	-16.6%<br>(-34.1 to 9.5)	759<br>(724 to 793)	-6.9%<br>(-10.1 to -3.5)	649<br>(516 to 830)	-16.5%<br>(-32.5 to 4.6)
**Monaco**	62<br>(54 to 72)	-44.8%<br>(-49.4 to -39.6)	42<br>(32 to 52)	-59.6%<br>(-69.0 to -44.1)	573<br>(544 to 605)	-29.2%<br>(-31.7 to -26.4)	576<br>(472 to 691)	-58.0%<br>(-66.6 to -44.0)
**Mongolia**	1,800<br>(1,570 to 2,082)	14.2%<br>(5.2 to 23.6)	372<br>(268 to 486)	1.3%<br>(-33.0 to 46.5)	21,810<br>(20,690 to 22,850)	10.2%<br>(6.1 to 15.0)	11,492<br>(9,309 to 13,966)	1.9%<br>(-23.6 to 32.9)
**Montenegro**	716<br>(620 to 825)	2.2%<br>(-6.3 to 9.9)	520<br>(411 to 638)	65.0%<br>(23.7 to 117.3)	5,643<br>(5,335 to 5,957)	-1.6%<br>(-5.6 to 2.0)	8,382<br>(6,819 to 10,309)	40.4%<br>(7.5 to 81.6)
**Morocco**	40,538<br>(35,616 to 46,053)	6.9%<br>(-1.2 to 16.0)	25,825<br>(20,230 to 32,471)	-3.3%<br>(-24.1 to 19.3)	333,629<br>(317,092 to 351,288)	4.7%<br>(0.7 to 8.7)	524,384<br>(408,240 to 659,417)	-7.7%<br>(-27.7 to 13.6)

**Mozambique**	16,509<br>(14,381 to 18,823)	13.0%<br>(4.2 to 23.5)	6,743<br>(4,988 to 8,656)	21.7%<br>(-10.8 to 56.9)	158,534<br>(151,210 to 166,120)	12.2%<br>(7.5 to 17.3)	162,429<br>(120,565 to 204,982)	23.8%<br>(-6.7 to 57.2)
**Myanmar**	45,302<br>(39,623 to 51,753)	-12.4%<br>(-20.1 to -3.7)	27,392<br>(21,971 to 34,317)	-26.1%<br>(-44.1 to -0.6)	397,617<br>(379,246 to 417,558)	-8.4%<br>(-11.7 to -4.7)	569,525<br>(454,554 to 706,404)	-30.2%<br>(-46.1 to -8.2)
**Namibia**	1,726<br>(1,481 to 1,990)	-8.9%<br>(-16.6 to 0.4)	735<br>(567 to 906)	-9.4%<br>(-28.4 to 15.0)	14,988<br>(14,256 to 15,710)	-13.4%<br>(-16.9 to -9.9)	15,140<br>(12,140 to 18,420)	-11.6%<br>(-28.8 to 9.2)
**Nauru**	7<br>(6 to 8)	-21.4%<br>(-28.1 to -14.2)	3<br>(2 to 4)	-10.7%<br>(-30.1 to 17.1)	91<br>(88 to 95)	-5.0%<br>(-8.4 to -1.8)	85<br>(67 to 106)	-10.1%<br>(-27.5 to 14.6)
**Nepal**	13,138<br>(11,289 to 15,164)	-8.9%<br>(-16.0 to -1.6)	7,429<br>(5,610 to 10,154)	-23.0%<br>(-42.2 to 2.3)	112,478<br>(105,958 to 119,184)	-9.6%<br>(-12.9 to -5.8)	149,263<br>(114,540 to 204,972)	-27.2%<br>(-45.1 to -3.3)
**Netherlands**	19,396<br>(16,696 to 22,566)	-49.3%<br>(-53.6 to -44.5)	8,584<br>(7,092 to 9,551)	-56.2%<br>(-59.3 to -53.1)	199,639<br>(189,617 to 208,840)	-39.8%<br>(-42.0 to -37.8)	129,839<br>(113,705 to 142,232)	-56.9%<br>(-59.5 to -54.6)
**New Zealand**	4,330<br>(3,709 to 4,973)	-41.1%<br>(-46.2 to -35.9)	1,832<br>(1,501 to 2,026)	-58.4%<br>(-61.9 to -55.2)	44,625<br>(40,088 to 49,773)	-21.4%<br>(-27.4 to -15.2)	27,662<br>(23,868 to 30,738)	-58.3%<br>(-61.3 to -55.3)
**Nicaragua**	2,774<br>(2,409 to 3,150)	-30.3%<br>(-36.2 to -24.6)	683<br>(564 to 840)	-42.5%<br>(-52.7 to -30.1)	27,969<br>(26,570 to 29,526)	-20.6%<br>(-23.4 to -17.5)	13,898<br>(11,879 to 16,498)	-41.5%<br>(-50.7 to -30.3)
**Niger**	8,182<br>(6,968 to 9,516)	-16.8%<br>(-23.1 to -10.4)	3,030<br>(2,138 to 4,357)	0.4%<br>(-20.5 to 30.4)	93,307<br>(88,200 to 97,757)	-15.2%<br>(-18.0 to -12.7)	72,340<br>(52,678 to 100,252)	-5.9%<br>(-24.4 to 19.8)
**Nigeria**	91,564<br>(77,814 to 107,150)	-10.8%<br>(-14.9 to -6.2)	38,019<br>(30,052 to 48,057)	-22.8%<br>(-41.5 to 7.7)	1,089,161<br>(987,484 to 1,197,106)	-5.0%<br>(-7.0 to -2.7)	883,707<br>(706,488 to 1,104,749)	-22.5%<br>(-42.2 to 6.7)
**Niue**	2<br>(2 to 2)	-14.3%<br>(-21.6 to -5.9)	1<br>(1 to 1)	-18.2%<br>(-33.5 to 2.9)	22<br>(21 to 23)	-8.9%<br>(-11.9 to -5.8)	19<br>(16 to 23)	-15.4%<br>(-29.4 to 1.5)
**North Macedonia**	5,666<br>(4,900 to 6,428)	-4.1%<br>(-12.0 to 4.7)	4,879<br>(4,031 to 5,734)	11.6%<br>(-6.6 to 30.8)	32,571<br>(29,511 to 35,984)	-16.5%<br>(-22.4 to -9.9)	80,990<br>(66,704 to 95,496)	-6.6%<br>(-22.5 to 10.5)
**Northern Mariana Islands**	35<br>(30 to 41)	-8.2%<br>(-16.4 to 2.5)	10<br>(9 to 12)	-33.1%<br>(-46.0 to -14.6)	397<br>(381 to 415)	-10.0%<br>(-13.3 to -6.9)	265<br>(229 to 299)	-30.2%<br>(-42.5 to -15.9)
**Norway**	7,597<br>(6,370 to 8,923)	-45.2%<br>(-49.7 to -40.3)	1,847<br>(1,522 to 2,027)	-74.2%<br>(-76.0 to -72.8)	74,867<br>(67,524 to 82,606)	-30.8%<br>(-34.3 to -26.8)	32,033<br>(27,904 to 35,777)	-69.4%<br>(-71.7 to -67.5)
**Oman**	2,338<br>(2,058 to 2,635)	-3.1%<br>(-12.5 to 7.9)	674<br>(546 to 804)	-28.5%<br>(-46.2 to -1.5)	29,347<br>(28,018 to 30,707)	2.5%<br>(-1.8 to 7.0)	18,108<br>(15,133 to 21,167)	-34.4%<br>(-50.5 to -11.2)
**Pakistan**	106,744<br>(91,891 to 124,115)	-3.1%<br>(-8.8 to 3.2)	41,149<br>(32,090 to 52,004)	2.5%<br>(-16.9 to 30.7)	1,049,106<br>(930,005 to 1,169,892)	-0.5%<br>(-4.4 to 3.5)	956,691<br>(779,500 to 1,193,626)	2.5%<br>(-15.3 to 28.0)
**Palau**	22<br>(19 to 26)	-3.2%<br>(-10.0 to 5.1)	8<br>(6 to 9)	-13.6%<br>(-32.9 to 12.5)	257<br>(246 to 268)	1.0%<br>(-2.2 to 4.8)	200<br>(167 to 238)	-14.2%<br>(-31.3 to 6.6)
**Palestine**	2,771<br>(2,483 to 3,126)	-2.1%<br>(-9.9 to 5.9)	1,436<br>(1,232 to 1,634)	-37.8%<br>(-49.8 to -23.5)	22,022<br>(20,849 to 23,088)	-8.3%<br>(-12.1 to -4.4)	29,158<br>(25,372 to 33,111)	-39.1%<br>(-51.2 to -24.1)
**Panama**	2,535<br>(2,175 to 2,897)	-28.4%<br>(-34.4 to -22.6)	1,074<br>(825 to 1,274)	-37.9%<br>(-50.0 to -26.5)	23,977<br>(22,891 to 25,269)	-16.6%<br>(-19.3 to -13.5)	17,107<br>(13,802 to 20,251)	-39.7%<br>(-50.7 to -28.9)
**Papua New Guinea**	3,047<br>(2,614 to 3,502)	-5.4%<br>(-12.2 to 2.9)	1,193<br>(876 to 1,653)	-10.6%<br>(-34.0 to 19.8)	34,606<br>(33,022 to 36,305)	-4.8%<br>(-8.1 to -1.1)	31,503<br>(25,005 to 40,327)	-12.1%<br>(-32.2 to 14.3)
**Paraguay**	4,038<br>(3,526 to 4,523)	-20.2%<br>(-27.7 to -11.9)	1,988<br>(1,559 to 2,431)	-32.9%<br>(-47.2 to -16.9)	35,993<br>(34,227 to 37,844)	-14.6%<br>(-18.0 to -10.6)	34,038<br>(27,270 to 41,660)	-31.7%<br>(-46.1 to -16.2)

**Peru**	14,365<br>(12,519 to 16,441)	-30.6%<br>(-35.7 to -24.9)	4,863<br>(3,742 to 6,147)	-43.4%<br>(-57.1 to -27.3)	169,716<br>(161,505 to 177,970)	-12.8%<br>(-15.7 to -9.9)	91,672<br>(73,669 to 112,555)	-40.6%<br>(-54.2 to -25.5)
**Philippines**	68,843<br>(58,884 to 80,269)	26.8%<br>(19.8 to 35.2)	28,253<br>(24,470 to 32,341)	-18.2%<br>(-29.3 to -6.4)	691,807<br>(621,854 to 768,552)	15.7%<br>(12.1 to 19.1)	671,394<br>(585,886 to 758,824)	-5.4%<br>(-17.4 to 8.1)
**Poland**	58,610<br>(48,783 to 69,317)	-28.2%<br>(-32.0 to -24.4)	34,630<br>(30,084 to 37,881)	-65.3%<br>(-68.1 to -62.7)	409,061<br>(364,814 to 458,234)	-8.8%<br>(-13.3 to -4.2)	563,558<br>(506,055 to 617,924)	-62.9%<br>(-65.6 to -59.9)
**Portugal**	12,970<br>(11,437 to 14,480)	-70.3%<br>(-72.4 to -68.1)	9,540<br>(7,857 to 10,497)	-83.0%<br>(-84.5 to -81.9)	91,609<br>(86,448 to 96,824)	-58.3%<br>(-61.4 to -54.8)	123,071<br>(106,002 to 133,863)	-82.7%<br>(-84.0 to -81.7)
**Puerto Rico**	2,800<br>(2,434 to 3,204)	-30.7%<br>(-36.0 to -25.4)	911<br>(725 to 1,063)	-70.2%<br>(-74.9 to -65.6)	31,354<br>(29,892 to 32,781)	-7.4%<br>(-10.8 to -3.7)	14,711<br>(12,499 to 16,895)	-62.0%<br>(-66.7 to -57.2)
**Qatar**	929<br>(753 to 1,130)	-35.2%<br>(-41.2 to -29.6)	113<br>(88 to 142)	-59.7%<br>(-68.8 to -49.1)	14,421<br>(13,556 to 15,333)	-38.8%<br>(-40.7 to -36.6)	4,321<br>(3,482 to 5,099)	-59.4%<br>(-68.0 to -49.2)
**Republic of Korea**	66,827<br>(56,987 to 76,617)	-63.9%<br>(-67.1 to -60.8)	23,232<br>(18,526 to 27,079)	-78.1%<br>(-81.6 to -74.3)	780,867<br>(748,703 to 815,512)	-49.6%<br>(-51.4 to -47.7)	434,175<br>(367,708 to 495,670)	-76.5%<br>(-79.6 to -73.2)
**Republic of Moldova**	6,405<br>(5,552 to 7,300)	-14.9%<br>(-22.9 to -5.4)	3,133<br>(2,830 to 3,459)	-40.9%<br>(-47.5 to -32.8)	43,663<br>(41,335 to 46,122)	2.5%<br>(-2.7 to 8.2)	63,140<br>(57,401 to 69,442)	-26.3%<br>(-34.1 to -15.8)
**Romania**	49,449<br>(43,256 to 55,128)	-29.6%<br>(-35.8 to -23.1)	38,210<br>(33,943 to 42,349)	-44.1%<br>(-50.0 to -37.5)	321,912<br>(304,643 to 339,513)	-14.5%<br>(-19.6 to -8.6)	593,583<br>(534,628 to 651,730)	-41.6%<br>(-48.0 to -34.4)
**Russian Federation**	337,709<br>(285,475 to 395,210)	-26.7%<br>(-30.5 to -22.7)	240,445<br>(218,161 to 258,644)	-46.6%<br>(-50.0 to -43.4)	2,048,668<br>(1,836,873 to 2,268,685)	-8.0%<br>(-11.7 to -3.9)	4,128,008<br>(3,829,112 to 4,445,427)	-44.4%<br>(-47.7 to -41.0)
**Rwanda**	6,105<br>(5,230 to 7,092)	-30.1%<br>(-35.4 to -23.7)	1,874<br>(1,325 to 2,504)	-43.8%<br>(-59.3 to -24.4)	62,428<br>(59,258 to 65,459)	-27.3%<br>(-30.3 to -24.4)	42,433<br>(32,072 to 54,790)	-46.6%<br>(-60.4 to -29.8)
**Saint Kitts and Nevis**	58<br>(51 to 67)	-34.6%<br>(-39.3 to -29.6)	34<br>(29 to 38)	-46.1%<br>(-52.9 to -39.3)	511<br>(484 to 541)	-33.9%<br>(-37.6 to -29.6)	628<br>(535 to 711)	-46.9%<br>(-54.7 to -38.8)
**Saint Lucia**	168<br>(148 to 188)	-38.4%<br>(-43.4 to -33.2)	119<br>(99 to 137)	-56.1%<br>(-62.5 to -49.1)	1,496<br>(1,429 to 1,568)	-24.4%<br>(-28.7 to -19.6)	1,799<br>(1,537 to 2,071)	-55.7%<br>(-62.1 to -48.2)
**Saint Vincent and the Grenadines**	96<br>(85 to 109)	-28.3%<br>(-34.0 to -22.2)	60<br>(54 to 67)	-36.7%<br>(-43.1 to -29.8)	852<br>(809 to 897)	-21.9%<br>(-25.5 to -18.2)	1,000<br>(897 to 1,104)	-37.9%<br>(-44.5 to -30.3)
**Samoa**	137<br>(120 to 155)	-8.5%<br>(-17.1 to 1.4)	47<br>(39 to 57)	-17.4%<br>(-34.3 to 3.2)	1,551<br>(1,487 to 1,615)	-1.7%<br>(-5.2 to 2.3)	1,115<br>(941 to 1,299)	-14.6%<br>(-29.1 to 2.9)
**San Marino**	41<br>(35 to 48)	-40.1%<br>(-45.0 to -35.4)	17<br>(12 to 23)	-70.1%<br>(-78.4 to -59.5)	393<br>(369 to 414)	-26.2%<br>(-28.7 to -23.7)	251<br>(190 to 325)	-64.0%<br>(-72.3 to -53.8)
**Sao Tome and Principe**	152<br>(134 to 171)	3.5%<br>(-4.1 to 11.7)	61<br>(52 to 74)	13.7%<br>(-4.2 to 34.9)	1,837<br>(1,763 to 1,915)	-0.4%<br>(-3.8 to 2.9)	1,424<br>(1,191 to 1,719)	11.8%<br>(-4.8 to 30.3)
**Saudi Arabia**	19,021<br>(16,691 to 21,461)	-13.3%<br>(-21.4 to -5.5)	7,410<br>(5,956 to 9,258)	-31.4%<br>(-47.4 to -7.5)	202,682<br>(193,392 to 212,307)	3.0%<br>(-1.0 to 7.1)	216,063<br>(174,938 to 268,571)	-30.3%<br>(-46.1 to -7.3)
**Senegal**	8,115<br>(7,085 to 9,236)	-16.2%<br>(-22.7 to -9.7)	4,577<br>(3,476 to 6,123)	-5.7%<br>(-26.9 to 17.4)	99,786<br>(95,383 to 104,071)	-14.0%<br>(-16.5 to -11.2)	99,245<br>(78,418 to 128,640)	-11.7%<br>(-30.1 to 7.9)
**Serbia**	25,510<br>(22,730 to 28,366)	-20.2%<br>(-27.0 to -13.3)	22,405<br>(18,994 to 26,072)	-46.4%<br>(-55.4 to -36.2)	131,246<br>(119,625 to 143,085)	-22.3%<br>(-28.0 to -16.5)	344,281<br>(293,380 to 398,179)	-45.2%<br>(-53.8 to -34.9)
**Seychelles**	94<br>(81 to 108)	-19.4%<br>(-25.3 to -13.1)	41<br>(33 to 47)	-30.8%<br>(-38.7 to -22.4)	965<br>(922 to 1,010)	-12.8%<br>(-16.1 to -9.3)	875<br>(718 to 1,007)	-32.6%<br>(-39.6 to -25.3)

**Sierra Leone**	4,392 (3,873 to 5,003)	-11.1% (-17.4 to -4.0)	2,325 (1,787 to 2,978)	-7.2% (-26.0 to 20.5)	52,972 (50,763 to 55,199)	-9.2% (-12.4 to -6.5)	55,693 (42,580 to 72,029)	-9.6% (-28.1 to 15.9)
**Singapore**	4,434 (3,706 to 5,272)	-65.0% (-68.9 to -61.1)	567 (469 to 631)	-87.6% (-89.1 to -86.4)	56,077 (53,412 to 58,842)	-51.6% (-53.7 to -49.4)	17,033 (14,427 to 19,670)	-81.9% (-83.9 to -80.2)
**Slovakia**	10,730 (9,259 to 12,349)	-34.0% (-39.8 to -27.3)	4,682 (3,879 to 5,563)	-50.5% (-59.4 to -38.9)	95,162 (90,419 to 99,499)	-26.5% (-29.0 to -23.7)	89,377 (76,107 to 104,499)	-49.2% (-57.1 to -39.1)
**Slovenia**	2,737 (2,438 to 3,043)	-57.0% (-60.8 to -53.4)	1,443 (1,205 to 1,628)	-69.8% (-73.5 to -66.8)	20,347 (19,472 to 21,263)	-29.7% (-34.7 to -24.3)	21,383 (18,596 to 23,932)	-70.6% (-73.5 to -67.8)
**Solomon Islands**	446 (390 to 508)	2.4% (-5.4 to 12.3)	112 (81 to 156)	-6.0% (-25.8 to 21.0)	4,327 (4,113 to 4,515)	-0.7% (-4.6 to 3.1)	2,966 (2,291 to 3,884)	-6.1% (-24.6 to 17.3)
**Somalia**	6,892 (5,920 to 8,050)	-10.2% (-17.8 to -2.8)	1,361 (848 to 2,070)	-19.1% (-39.6 to 9.3)	71,934 (68,304 to 75,821)	-10.0% (-13.4 to -6.4)	39,115 (27,179 to 56,839)	-18.7% (-36.3 to 5.3)
**South Africa**	50,938 (42,146 to 60,032)	-4.7% (-9.6 to 1.0)	19,779 (17,764 to 21,751)	30.8% (16.4 to 59.0)	510,187 (458,127 to 560,994)	-17.1% (-20.9 to -13.6)	404,688 (365,293 to 443,552)	15.8% (5.2 to 34.1)
**South Sudan**	3,722 (3,182 to 4,284)	-10.4% (-18.1 to -2.3)	1,067 (765 to 1,438)	-15.9% (-34.9 to 10.3)	39,578 (37,543 to 41,674)	-11.7% (-14.7 to -8.6)	26,423 (20,042 to 35,182)	-15.9% (-33.5 to 7.4)
**Spain**	53,333 (49,630 to 56,840)	-51.0% (-55.7 to -45.2)	19,845 (15,792 to 22,143)	-80.4% (-81.9 to -79.0)	566,959 (552,727 to 580,911)	-15.1% (-21.4 to -7.8)	306,534 (264,923 to 339,166)	-74.5% (-76.5 to -72.7)
**Sri Lanka**	21,902 (18,948 to 25,221)	-17.4% (-22.9 to -10.9)	15,166 (10,889 to 19,542)	-32.0% (-51.8 to -9.7)	204,025 (194,396 to 213,416)	-11.4% (-14.3 to -8.3)	267,585 (199,624 to 337,945)	-31.7% (-50.4 to -10.7)
**Sudan**	24,581 (21,644 to 27,806)	1.3% (-6.2 to 9.8)	12,090 (8,790 to 16,044)	-21.9% (-40.1 to 4.2)	225,083 (214,045 to 236,472)	8.0% (3.5 to 12.5)	283,445 (202,996 to 377,398)	-22.8% (-41.4 to 2.5)
**Suriname**	470 (411 to 529)	-8.3% (-15.9 to -0.4)	239 (183 to 298)	-26.5% (-44.2 to -5.4)	4,158 (3,958 to 4,371)	-8.7% (-12.1 to -5.1)	4,397 (3,499 to 5,392)	-26.6% (-42.5 to -7.1)
**Sweden**	14,224 (11,664 to 17,150)	-37.5% (-42.0 to -32.2)	4,608 (3,767 to 5,210)	-66.6% (-69.7 to -63.5)	140,796 (125,529 to 156,341)	-18.6% (-25.6 to -11.3)	73,163 (62,149 to 82,863)	-62.0% (-65.3 to -58.7)
**Switzerland**	7,835 (6,796 to 8,925)	-42.2% (-47.4 to -34.9)	2,936 (2,297 to 3,285)	-73.2% (-75.9 to -71.1)	79,395 (75,994 to 82,783)	-19.6% (-23.4 to -16.6)	44,159 (37,922 to 49,457)	-68.5% (-70.8 to -66.1)
**Syrian Arab Republic**	10,526 (9,257 to 11,913)	-17.9% (-24.4 to -11.8)	6,152 (4,810 to 7,714)	-18.8% (-38.7 to 9.4)	106,037 (100,689 to 111,409)	-18.7% (-21.4 to -16.0)	133,988 (104,878 to 167,219)	-25.2% (-43.7 to 1.2)
**Turkey**	69,523 (60,002 to 79,849)	-32.5% (-38.6 to -25.4)	37,640 (30,897 to 44,916)	-43.2% (-55.1 to -29.0)	701,854 (671,660 to 733,949)	-27.2% (-29.7 to -24.5)	670,470 (566,003 to 797,213)	-47.4% (-57.1 to -35.6)
**Taiwan (Province of China)**	34,128 (29,303 to 39,374)	-38.3% (-44.6 to -32.1)	6,398 (5,400 to 7,029)	-75.1% (-77.5 to -73.1)	353,147 (338,417 to 367,800)	-25.1% (-27.7 to -22.7)	161,449 (139,143 to 182,768)	-66.5% (-69.7 to -63.8)
**Tajikistan**	6,863 (5,964 to 7,812)	20.2% (10.1 to 31.2)	2,762 (2,207 to 3,369)	-13.8% (-34.1 to 13.8)	51,653 (48,943 to 54,270)	-2.5% (-6.1 to 1.5)	63,406 (51,375 to 76,772)	-18.9% (-37.5 to 5.0)
**Thailand**	68,522 (59,605 to 78,340)	-27.0% (-32.3 to -21.2)	28,619 (21,671 to 35,981)	-40.5% (-55.9 to -18.8)	754,917 (723,050 to 787,758)	-14.6% (-17.9 to -11.4)	615,507 (499,422 to 746,499)	-35.9% (-49.5 to -17.9)
**Timor-Leste**	811 (695 to 941)	13.7% (4.1 to 26.0)	454 (329 to 599)	10.4% (-20.2 to 56.8)	6,744 (6,392 to 7,076)	6.0% (1.8 to 9.9)	9,556 (7,036 to 12,343)	7.8% (-20.4 to 45.0)
**Togo**	4,217 (3,691 to 4,815)	-12.0% (-17.8 to -4.6)	2,009 (1,465 to 2,611)	-0.4% (-22.3 to 26.9)	49,934 (47,555 to 52,356)	-12.1% (-14.9 to -8.8)	49,746 (36,902 to 62,920)	-3.0% (-24.3 to 21.7)

**Tokelau**	1<br>(1 to 1)	-15.8%<br>(-24.3 to -7.7)	1<br>(1 to 1)	-31.1%<br>(-46.8 to -11.6)	13<br>(12 to 13)	-2.3%<br>(-6.0 to 1.2)	13<br>(10 to 15)	-26.8%<br>(-41.3 to -8.5)
**Tonga**	59<br>(52 to 67)	-2.5%<br>(-10.4 to 6.4)	22<br>(18 to 27)	-5.2%<br>(-26.3 to 26.2)	648<br>(619 to 679)	-4.2%<br>(-7.2 to -1.0)	467<br>(385 to 550)	-7.8%<br>(-25.1 to 14.5)
**Trinidad and Tobago**	1,329<br>(1,146 to 1,509)	-36.1%<br>(-40.7 to -31.2)	773<br>(615 to 949)	-50.3%<br>(-59.5 to -39.0)	12,843<br>(12,229 to 13,448)	-25.9%<br>(-28.7 to -23.0)	13,346<br>(10,799 to 16,321)	-48.8%<br>(-58.0 to -37.0)
**Tunisia**	11,480<br>(9,981 to 13,302)	0.2%<br>(-7.2 to 8.2)	6,787<br>(4,808 to 9,147)	-25.6%<br>(-45.9 to -0.7)	97,244<br>(92,888 to 102,140)	8.0%<br>(4.3 to 12.2)	125,685<br>(92,452 to 167,656)	-24.6%<br>(-43.5 to -0.8)
**Turkmenistan**	5,980<br>(5,235 to 6,765)	13.3%<br>(3.2 to 23.9)	2,920<br>(2,247 to 3,687)	17.9%<br>(-5.0 to 44.8)	53,872<br>(51,405 to 56,407)	22.4%<br>(17.9 to 27.9)	72,322<br>(56,423 to 90,146)	18.4%<br>(-3.9 to 44.9)
**Tuvalu**	9<br>(8 to 11)	-3.2%<br>(-10.5 to 5.4)	4<br>(4 to 5)	-21.4%<br>(-36.4 to -2.6)	98<br>(94 to 102)	-1.9%<br>(-5.6 to 1.5)	98<br>(82 to 116)	-21.8%<br>(-35.0 to -6.3)
**Uganda**	16,918<br>(14,455 to 19,391)	-6.7%<br>(-15.3 to 2.4)	3,792<br>(2,911 to 4,957)	-24.1%<br>(-43.1 to 5.0)	179,088<br>(170,916 to 188,243)	-9.5%<br>(-12.5 to -5.9)	93,294<br>(74,822 to 118,587)	-22.5%<br>(-39.6 to 1.2)
**Ukraine**	107,459<br>(89,951 to 126,619)	-31.5%<br>(-36.4 to -26.3)	64,409<br>(50,727 to 79,552)	-48.1%<br>(-59.0 to -35.5)	712,633<br>(634,548 to 793,791)	-15.3%<br>(-21.6 to -9.0)	1,154,862<br>(915,982 to 1,417,130)	-43.6%<br>(-54.8 to -31.1)
**United Arab Emirates**	7,842<br>(6,528 to 9,343)	-24.1%<br>(-30.4 to -17.9)	645<br>(494 to 790)	-29.9%<br>(-43.6 to -11.1)	88,363<br>(84,297 to 92,705)	-12.8%<br>(-16.7 to -9.3)	27,700<br>(22,906 to 33,046)	-36.6%<br>(-48.4 to -19.4)
**United Kingdom**	72,319<br>(62,977 to 82,443)	-46.9%<br>(-50.0 to -43.4)	25,142<br>(21,088 to 27,152)	-75.1%<br>(-76.8 to -74.2)	714,518<br>(661,108 to 769,755)	-27.1%<br>(-29.4 to -24.7)	397,336<br>(354,482 to 433,353)	-72.0%<br>(-73.6 to -70.5)
**United Republic of Tanzania**	31,679<br>(27,731 to 36,056)	16.8%<br>(8.2 to 25.9)	9,495<br>(7,010 to 12,617)	26.3%<br>(-8.8 to 79.4)	328,680<br>(314,012 to 343,891)	16.1%<br>(11.5 to 20.6)	211,680<br>(161,632 to 275,192)	21.9%<br>(-9.5 to 65.6)
**United States of America**	311,587<br>(259,142 to 370,269)	-36.6%<br>(-40.0 to -32.6)	115,555<br>(94,590 to 126,652)	-40.6%<br>(-43.6 to -38.7)	5,136,254<br>(4,700,312 to 5,584,577)	-3.4%<br>(-8.0 to 1.2)	2,178,054<br>(1,909,480 to 2,407,925)	-34.3%<br>(-36.9 to -32.1)
**United States Virgin Islands**	100<br>(87 to 116)	-2.0%<br>(-9.8 to 6.8)	34<br>(27 to 41)	-55.3%<br>(-65.6 to -43.3)	882<br>(837 to 930)	-1.7%<br>(-5.4 to 2.5)	582<br>(477 to 700)	-53.2%<br>(-63.2 to -41.4)
**Uruguay**	3,919<br>(3,451 to 4,436)	-46.7%<br>(-51.1 to -42.1)	2,317<br>(2,020 to 2,502)	-55.8%<br>(-58.8 to -52.9)	36,573<br>(34,931 to 38,305)	-39.5%<br>(-41.8 to -36.9)	35,404<br>(31,882 to 37,966)	-55.2%<br>(-57.7 to -51.8)
**Uzbekistan**	37,228<br>(32,988 to 42,472)	11.9%<br>(1.6 to 22.8)	11,872<br>(10,308 to 13,733)	1.7%<br>(-13.1 to 21.1)	307,474<br>(292,279 to 322,147)	-0.7%<br>(-5.3 to 4.7)	297,392<br>(259,388 to 339,123)	-7.5%<br>(-19.9 to 8.1)
**Vanuatu**	209<br>(183 to 237)	0.5%<br>(-6.9 to 8.7)	57<br>(43 to 71)	-18.5%<br>(-36.0 to -0.6)	2,344<br>(2,251 to 2,453)	0.4%<br>(-3.1 to 3.9)	1,612<br>(1,308 to 1,947)	-16.4%<br>(-32.2 to 1.2)
**Venezuela (Bolivarian Republic of)**	16,235<br>(14,039 to 18,535)	-23.3%<br>(-29.5 to -16.7)	7,299<br>(5,579 to 9,246)	-16.9%<br>(-34.9 to 9.2)	157,922<br>(150,265 to 165,808)	-18.4%<br>(-21.3 to -15.3)	125,337<br>(99,415 to 156,775)	-20.6%<br>(-36.9 to 4.1)
**Viet Nam**	110,010<br>(100,113 to 120,837)	6.6%<br>(-4.2 to 16.3)	74,355<br>(59,508 to 88,026)	6.5%<br>(-18.5 to 33.9)	888,433<br>(852,548 to 926,826)	11.6%<br>(7.1 to 17.3)	1,418,783<br>(1,129,765 to 1,695,470)	5.7%<br>(-19.7 to 31.5)
**Yemen**	16,560<br>(14,635 to 18,601)	-0.2%<br>(-8.2 to 9.4)	10,868<br>(7,885 to 14,995)	-6.1%<br>(-29.6 to 26.6)	137,863<br>(130,780 to 145,322)	0.7%<br>(-3.2 to 4.9)	246,350<br>(178,758 to 335,845)	-9.2%<br>(-31.6 to 21.5)
**Zambia**	9,002<br>(7,767 to 10,263)	13.4%<br>(3.5 to 25.4)	2,741<br>(2,016 to 3,662)	17.2%<br>(-11.7 to 55.9)	87,264<br>(83,128 to 91,212)	3.8%<br>(0.0 to 7.7)	63,114<br>(48,813 to 81,719)	14.7%<br>(-11.7 to 47.5)
**Zimbabwe**	7,957<br>(6,834 to 9,149)	17.7%<br>(9.3 to 27.1)	2,992<br>(2,425 to 3,687)	34.2%<br>(6.2 to 76.0)	79,105<br>(75,100 to 82,800)	11.0%<br>(7.0 to 15.0)	71,195<br>(58,621 to 86,965)	39.7%<br>(12.7 to 79.1)

Appendix Table 3. Incident cases, deaths, prevalent cases, and DALYs for intracerebral haemorrhage in 2021 and percentage changes of age-standardised rates for 1990-2021, by location

Country, region	Incident cases (95% uncertainty interval)		Deaths (95% uncertainty interval)		Prevalent cases (95% uncertainty interval)		DALYs (95% uncertainty interval)	
	2021 counts	% change in age-standardised rates, 1990-2021	2021 counts	% change in age-standardised rates, 1990-2021	2021 counts	% change in age-standardised rates, 1990-2021	2021 counts	% change in age-standardised rates, 1990-2021
**Countries categorised by the World Bank Income level**								
**Global**	3,444,338 (3,053,009 to 3,812,043)	-15.8% (-18.8 to -13.3)	3,308,367 (3,021,075 to 3,594,725)	-39.6% (-43.8 to -35.3)	16,603,836 (15,159,477 to 18,183,399)	-3.5% (-5.1 to -2.1)	79,457,427 (72,748,913 to 85,480,165)	-34.9% (-39.5 to -30.0)
**High income**	354,137 (316,688 to 387,754)	-44.6% (-46.3 to -42.7)	259,925 (227,902 to 276,305)	-54.8% (-57.2 to -53.1)	2,801,034 (2,577,861 to 3,027,879)	-23.3% (-25.0 to -21.7)	5,100,101 (4,718,497 to 5,353,057)	-56.8% (-58.3 to -55.2)
**Upper Middle income**	1,522,969 (1,324,208 to 1,713,890)	-40.4% (-43.1 to -37.4)	1,619,051 (1,392,340 to 1,868,967)	-45.7% (-54.3 to -36.2)	6,217,517 (5,600,342 to 6,876,172)	-29.7% (-31.8 to -27.6)	34,910,112 (30,338,131 to 40,111,545)	-49.1% (-56.9 to -39.9)
**Lower Middle income**	1,357,849 (1,190,849 to 1,530,558)	-26.5% (-28.9 to -23.4)	1,217,947 (1,087,860 to 1,337,867)	-29.8% (-36.8 to -21.2)	6,592,374 (6,006,447 to 7,282,796)	-16.1% (-18.2 to -14.2)	33,315,424 (30,059,372 to 36,563,324)	-31.5% (-38.0 to -23.6)
**Low income**	206,847 (189,385 to 224,140)	-32.9% (-34.9 to -30.4)	208,864 (175,618 to 244,161)	-35.0% (-42.4 to -26.4)	981,840 (925,095 to 1,045,196)	-32.8% (-34.2 to -31.4)	6,071,127 (5,071,775 to 7,095,486)	-37.3% (-45.7 to -28.9)
**Countries categorised by the sociodemographic index (SDI) level**								
**High SDI**	318,162 (280,065 to 351,618)	-37.4% (-39.7 to -34.9)	229,919 (203,098 to 245,599)	-63.9% (-66.2 to -62.4)	2,610,494 (2,394,350 to 2,825,910)	-12.8% (-15.1 to -10.5)	4,654,165 (4,290,257 to 4,945,586)	-56.8% (-59.2 to -54.7)
**High-middle SDI**	723,025 (633,802 to 810,231)	-17.9% (-21.0 to -15.0)	723,418 (631,717 to 818,101)	-46.7% (-51.0 to -41.8)	3,101,700 (2,798,346 to 3,414,063)	-1.4% (-4.3 to 1.2)	15,541,585 (13,649,187 to 17,524,255)	-42.9% (-47.6 to -37.6)
**Middle SDI**	1,326,676 (1,162,442 to 1,482,718)	6.2% (1.5 to 10.8)	1,376,759 (1,208,750 to 1,544,296)	-22.4% (-32.7 to -11.5)	5,670,069 (5,112,851 to 6,287,683)	9.5% (7.0 to 11.9)	32,108,446 (28,710,686 to 35,682,222)	-21.4% (-31.3 to -10.6)
**Low-middle SDI**	762,317 (673,983 to 855,941)	-7.6% (-11.4 to -3.5)	695,529 (617,641 to 780,047)	-13.7% (-22.1 to -3.0)	3,638,970 (3,332,064 to 3,994,135)	-1.7% (-3.6 to 0.2)	18,877,162 (16,896,813 to 21,050,928)	-13.9% (-22.4 to -4.1)
**Low SDI**	311,636 (280,528 to 344,290)	-12.9% (-16.3 to -9.3)	280,175 (236,563 to 322,806)	-13.5% (-23.0 to -1.8)	1,571,605 (1,456,994 to 1,697,189)	-9.8% (-11.5 to -8.1)	8,211,608 (6,933,861 to 9,441,941)	-14.9% (-23.9 to -4.5)
**Countries categorised by the GBD super-regions**								
**Central Europe, Eastern Europe, and Central Asia**	198,074 (179,365 to 214,735)	-27.5% (-30.4 to -24.7)	161,217 (151,038 to 171,405)	-46.9% (-49.3 to -44.3)	686,083 (626,039 to 750,388)	-11.9% (-14.2 to -9.4)	3,772,528 (3,534,492 to 4,017,821)	-44.6% (-47.3 to -42.0)
**High-income**	308,443 (274,242 to 338,995)	-43.1% (-45.6 to -40.1)	223,508 (192,736 to 239,030)	-68.0% (-70.4 to -66.8)	2,605,894 (2,392,497 to 2,822,566)	-16.1% (-18.3 to -13.7)	4,251,974 (3,867,345 to 4,486,977)	-61.8% (-64.0 to -59.9)
**Latin America and Caribbean**	129,415 (115,407 to 143,362)	-37.5% (-39.9 to -35.2)	102,886 (94,534 to 111,046)	-55.4% (-58.0 to -52.6)	796,106 (731,059 to 859,428)	-23.9% (-25.6 to -22.0)	2,703,773 (2,509,898 to 2,916,248)	-54.1% (-56.4 to -51.5)

**North Africa and Middle East**	121,272<br>(109,767 to 132,332)	-11.9%<br>(-16.3 to -7.4)	106,099<br>(91,137 to 121,433)	-30.9%<br>(-39.5 to -21.3)	803,051<br>(752,918 to 856,261)	-4.7%<br>(-6.8 to -2.5)	3,051,893<br>(2,633,083 to 3,493,646)	-31.5%<br>(-39.8 to -22.0)
**South Asia**	703,200<br>(607,008 to 804,764)	-15.8%<br>(-19.8 to -11.7)	558,346<br>(456,819 to 640,417)	-12.3%<br>(-24.0 to 1.6)	3,473,328<br>(3,115,617 to 3,881,651)	-5.4%<br>(-7.9 to -2.7)	15,045,582<br>(12,776,920 to 17,158,102)	-14.7%<br>(-25.8 to -1.9)
**Southeast Asia, East Asia, and Oceania**	1,674,028<br>(1,465,467 to 1,880,406)	25.8%<br>(19.9 to 31.5)	1,874,646<br>(1,618,226 to 2,141,661)	-13.2%<br>(-28.5 to 2.5)	6,597,049<br>(5,911,345 to 7,317,533)	24.2%<br>(20.9 to 27.6)	42,356,952<br>(37,093,195 to 48,079,107)	-13.4%<br>(-27.9 to 0.9)
**Sub-Saharan Africa**	309,906<br>(281,320 to 339,832)	-6.9%<br>(-10.4 to -3.1)	281,666<br>(240,280 to 322,385)	-7.2%<br>(-18.7 to 8.0)	1,642,325<br>(1,531,604 to 1,762,971)	-7.5%<br>(-9.1 to -5.8)	8,274,723<br>(7,016,360 to 9,572,664)	-8.8%<br>(-19.7 to 5.2)
**Countries categorised by the GBD regions**								
**Andean Latin America**	10,818<br>(9,858 to 11,861)	-27.1%<br>(-30.9 to -23.1)	8,766<br>(7,271 to 10,653)	-44.1%<br>(-52.9 to -33.7)	77,724<br>(72,458 to 83,004)	-14.8%<br>(-16.7 to -13.0)	234,343<br>(193,965 to 284,558)	-44.2%<br>(-52.7 to -34.7)
**Australasia**	5,698<br>(5,118 to 6,243)	-42.1%<br>(-45.9 to -38.5)	3,912<br>(3,371 to 4,301)	-69.2%<br>(-71.7 to -67.0)	37,993<br>(34,898 to 41,104)	-22.6%<br>(-24.6 to -20.4)	67,763<br>(61,317 to 73,378)	-65.1%<br>(-67.4 to -62.8)
**Caribbean**	17,584<br>(16,192 to 18,935)	-11.6%<br>(-15.1 to -8.0)	16,729<br>(14,312 to 19,374)	-31.2%<br>(-38.4 to -22.9)	87,175<br>(82,051 to 92,794)	-5.0%<br>(-7.1 to -3.0)	443,350<br>(375,380 to 519,204)	-27.4%<br>(-35.1 to -18.5)
**Central Asia**	55,548<br>(51,306 to 59,650)	-6.3%<br>(-11.7 to -1.3)	34,963<br>(31,599 to 38,840)	-13.2%<br>(-21.4 to -3.4)	180,253<br>(168,875 to 191,994)	-8.4%<br>(-10.3 to -6.3)	884,451<br>(792,892 to 985,113)	-16.6%<br>(-24.1 to -7.8)
**Central Europe**	50,101<br>(45,976 to 53,823)	-32.3%<br>(-34.5 to -30.0)	47,995<br>(44,202 to 51,503)	-53.5%<br>(-56.5 to -50.5)	151,846<br>(140,163 to 164,066)	-19.1%<br>(-21.4 to -17.2)	957,740<br>(882,029 to 1,029,987)	-52.1%<br>(-55.2 to -49.2)
**Central Latin America**	48,325<br>(43,161 to 53,249)	-35.4%<br>(-37.6 to -33.2)	33,528<br>(29,703 to 37,865)	-50.2%<br>(-54.6 to -44.9)	339,240<br>(312,470 to 366,494)	-23.8%<br>(-25.5 to -22.1)	853,699<br>(762,557 to 971,209)	-47.6%<br>(-52.1 to -42.7)
**Central Sub-Saharan Africa**	37,323<br>(33,809 to 41,188)	-11.1%<br>(-16.4 to -4.9)	36,781<br>(27,714 to 47,047)	-7.9%<br>(-27.8 to 16.2)	174,316<br>(163,264 to 186,749)	-10.6%<br>(-13.3 to -8.0)	1,064,701<br>(797,681 to 1,357,572)	-11.8%<br>(-28.7 to 8.1)
**East Asia**	1,218,007<br>(1,046,031 to 1,377,277)	33.0%<br>(25.7 to 40.3)	1,366,572<br>(1,147,252 to 1,610,948)	-15.3%<br>(-32.4 to 4.2)	4,554,930<br>(4,050,940 to 5,081,761)	31.5%<br>(27.4 to 35.7)	28,529,140<br>(23,808,304 to 33,766,175)	-15.7%<br>(-31.3 to 2.5)
**Eastern Europe**	92,425<br>(80,057 to 103,800)	-28.0%<br>(-31.4 to -24.6)	78,259<br>(72,124 to 84,285)	-45.9%<br>(-49.2 to -42.3)	353,984<br>(313,522 to 398,007)	-10.5%<br>(-13.6 to -6.9)	1,930,338<br>(1,777,209 to 2,078,211)	-43.3%<br>(-46.9 to -39.6)
**Eastern Sub-Saharan Africa**	112,870<br>(102,527 to 124,053)	-6.6%<br>(-10.8 to -2.1)	111,874<br>(95,012 to 130,095)	-10.6%<br>(-25.2 to 7.2)	535,714<br>(497,161 to 575,131)	-7.0%<br>(-8.7 to -5.0)	3,231,776<br>(2,741,932 to 3,779,885)	-11.0%<br>(-25.6 to 6.0)
**High-income Asia Pacific**	77,253<br>(67,778 to 85,468)	-46.6%<br>(-50.6 to -42.2)	54,899<br>(45,364 to 60,784)	-74.9%<br>(-77.2 to -73.3)	813,438<br>(735,552 to 887,603)	-24.7%<br>(-27.5 to -21.1)	1,038,269<br>(916,660 to 1,124,028)	-68.0%<br>(-70.4 to -65.8)
**High-income North America**	79,595<br>(68,334 to 90,253)	-36.8%<br>(-40.2 to -33.0)	62,750<br>(56,014 to 66,579)	-43.3%<br>(-46.3 to -41.3)	843,200<br>(761,615 to 932,205)	-4.2%<br>(-8.4 to 0.1)	1,343,437<br>(1,250,162 to 1,412,207)	-36.4%<br>(-38.9 to -34.2)
**North Africa and Middle East**	121,272<br>(109,767 to 132,332)	-11.9%<br>(-16.3 to -7.4)	106,099<br>(91,137 to 121,433)	-30.9%<br>(-39.5 to -21.3)	803,051<br>(752,918 to 856,261)	-4.7%<br>(-6.8 to -2.5)	3,051,893<br>(2,633,083 to 3,493,646)	-31.5%<br>(-39.8 to -22.0)
**Oceania**	5,443<br>(4,926 to 5,964)	-10.2%<br>(-15.2 to -4.5)	7,262<br>(5,783 to 8,882)	-16.4%<br>(-30.3 to 0.0)	24,845<br>(23,415 to 26,480)	-8.4%<br>(-10.6 to -6.1)	211,111<br>(167,044 to 258,871)	-15.8%<br>(-28.2 to -0.5)
**South Asia**	703,200<br>(607,008 to 804,764)	-15.8%<br>(-19.8 to -11.7)	558,346<br>(456,819 to 640,417)	-12.3%<br>(-24.0 to 1.6)	3,473,328<br>(3,115,617 to 3,881,651)	-5.4%<br>(-7.9 to -2.7)	15,045,582<br>(12,776,920 to 17,158,102)	-14.7%<br>(-25.8 to -1.9)
**Southeast Asia**	450,578<br>(400,978 to 497,072)	0.0%<br>(-3.8 to 4.0)	500,812<br>(438,999 to 571,700)	-6.2%<br>(-20.8 to 9.0)	2,017,274<br>(1,834,696 to 2,208,645)	-0.2%<br>(-2.1 to 1.8)	13,616,701<br>(12,011,136 to 15,407,482)	-6.6%<br>(-19.8 to 6.8)

**Southern Latin America**	21,618<br>(19,466 to 23,530)	-38.3%<br>(-42.1 to -34.3)	13,214<br>(12,212 to 14,024)	-63.2%<br>(-65.7 to -60.8)	159,305<br>(150,466 to 168,995)	-25.5%<br>(-27.8 to -23.1)	314,123<br>(295,955 to 332,443)	-59.8%<br>(-62.4 to -57.1)
**Southern Sub-Saharan Africa**	20,705<br>(18,235 to 23,292)	-0.3%<br>(-4.3 to 4.6)	27,476<br>(25,442 to 29,751)	27.5%<br>(14.6 to 52.4)	103,534<br>(93,715 to 114,465)	-12.0%<br>(-15.3 to -8.8)	754,889<br>(693,688 to 820,770)	17.1%<br>(6.5 to 34.7)
**Tropical Latin America**	52,688<br>(45,343 to 60,232)	-44.3%<br>(-47.4 to -41.2)	43,863<br>(41,028 to 45,959)	-63.1%<br>(-64.9 to -61.7)	291,967<br>(261,326 to 323,598)	-28.7%<br>(-31.5 to -26.0)	1,172,380<br>(1,118,584 to 1,218,666)	-62.0%<br>(-63.6 to -60.6)
**Western Europe**	124,279<br>(111,584 to 135,124)	-45.1%<br>(-48.1 to -42.1)	88,732<br>(76,162 to 95,377)	-73.4%<br>(-75.4 to -72.2)	751,958<br>(699,013 to 806,480)	-22.4%<br>(-24.3 to -20.4)	1,488,382<br>(1,347,101 to 1,577,865)	-69.1%<br>(-70.9 to -67.5)
**Western Sub-Saharan Africa**	139,009<br>(125,319 to 152,586)	-9.0%<br>(-12.6 to -4.9)	105,535<br>(83,668 to 126,793)	-10.7%<br>(-25.4 to 10.8)	828,760<br>(775,626 to 889,950)	-5.7%<br>(-7.3 to -3.9)	3,223,357<br>(2,546,461 to 3,936,778)	-11.5%<br>(-25.8 to 8.2)
**Countries in alphabetical order**								
**Afghanistan**	6,824<br>(6,177 to 7,494)	-43.1%<br>(-45.7 to -40.1)	6,844<br>(4,753 to 9,390)	-46.2%<br>(-60.3 to -29.1)	38,568<br>(36,435 to 40,948)	-41.6%<br>(-43.5 to -39.5)	241,355<br>(173,077 to 326,234)	-47.3%<br>(-60.8 to -29.2)
**Albania**	3,294<br>(3,034 to 3,554)	-16.8%<br>(-21.3 to -12.5)	4,243<br>(3,437 to 5,072)	-27.7%<br>(-42.3 to -11.5)	5,626<br>(5,249 to 6,058)	-30.2%<br>(-33.0 to -26.8)	65,219<br>(53,094 to 78,550)	-38.1%<br>(-51.5 to -23.6)
**Algeria**	8,459<br>(7,545 to 9,362)	-52.4%<br>(-54.9 to -49.6)	7,091<br>(4,831 to 10,280)	-51.0%<br>(-64.8 to -37.1)	57,578<br>(53,886 to 61,638)	-41.1%<br>(-43.3 to -38.7)	180,749<br>(126,506 to 253,357)	-55.3%<br>(-66.8 to -42.5)
**American Samoa**	29<br>(26 to 32)	-27.9%<br>(-32.0 to -23.4)	30<br>(26 to 36)	-26.3%<br>(-38.2 to -9.3)	150<br>(141 to 159)	-24.4%<br>(-27.6 to -21.0)	818<br>(687 to 972)	-25.8%<br>(-38.7 to -8.2)
**Andorra**	16<br>(14 to 18)	-22.1%<br>(-27.2 to -16.1)	8<br>(6 to 11)	-42.7%<br>(-61.7 to -18.3)	123<br>(115 to 133)	-13.4%<br>(-16.9 to -10.1)	164<br>(122 to 210)	-47.0%<br>(-64.9 to -25.9)
**Angola**	7,307<br>(6,567 to 8,140)	-41.3%<br>(-44.9 to -37.6)	7,358<br>(5,576 to 9,220)	-37.9%<br>(-54.4 to -17.7)	39,373<br>(36,689 to 42,099)	-33.8%<br>(-36.5 to -31.1)	219,652<br>(168,036 to 276,356)	-42.0%<br>(-57.9 to -22.8)
**Antigua and Barbuda**	31<br>(28 to 34)	-39.9%<br>(-43.1 to -36.1)	31<br>(28 to 34)	-47.5%<br>(-52.5 to -41.8)	161<br>(151 to 172)	-30.7%<br>(-34.6 to -27.4)	721<br>(662 to 790)	-51.2%<br>(-56.1 to -45.9)
**Argentina**	14,710<br>(13,241 to 16,125)	-52.8%<br>(-55.7 to -49.7)	9,371<br>(8,670 to 9,988)	-63.4%<br>(-66.6 to -60.2)	101,763<br>(95,927 to 108,049)	-45.4%<br>(-47.7 to -42.9)	225,483<br>(211,364 to 238,981)	-65.3%<br>(-67.8 to -62.5)
**Armenia**	782<br>(702 to 864)	-57.1%<br>(-59.8 to -54.3)	513<br>(454 to 575)	-69.8%<br>(-74.2 to -65.0)	3,593<br>(3,303 to 3,888)	-33.1%<br>(-36.4 to -29.9)	11,833<br>(10,559 to 13,317)	-67.6%<br>(-72.0 to -62.5)
**Australia**	4,867<br>(4,375 to 5,355)	-41.8%<br>(-46.0 to -38.1)	3,289<br>(2,827 to 3,621)	-53.0%<br>(-57.4 to -48.6)	32,460<br>(29,831 to 35,029)	-25.0%<br>(-28.0 to -22.0)	56,597<br>(51,170 to 61,414)	-56.7%<br>(-59.9 to -53.1)
**Austria**	2,498<br>(2,200 to 2,804)	-61.6%<br>(-64.4 to -58.7)	1,251<br>(1,078 to 1,369)	-65.5%<br>(-68.7 to -62.6)	21,430<br>(20,168 to 22,644)	-44.3%<br>(-46.7 to -40.8)	22,562<br>(20,295 to 24,453)	-68.9%<br>(-71.2 to -66.6)
**Azerbaijan**	7,623<br>(6,948 to 8,239)	6.1%<br>(0.1 to 13.0)	5,025<br>(3,923 to 6,197)	-24.0%<br>(-42.5 to -2.0)	21,124<br>(19,575 to 22,739)	-25.9%<br>(-29.1 to -22.0)	115,226<br>(89,453 to 142,874)	-32.8%<br>(-50.1 to -12.7)
**Bahamas**	124<br>(113 to 135)	-30.7%<br>(-34.1 to -26.7)	96<br>(78 to 118)	-47.0%<br>(-58.3 to -33.5)	728<br>(684 to 779)	-23.9%<br>(-27.0 to -20.8)	2,649<br>(2,139 to 3,268)	-47.4%<br>(-58.3 to -33.0)
**Bahrain**	172<br>(151 to 196)	-50.9%<br>(-53.7 to -47.7)	134<br>(112 to 158)	-61.4%<br>(-68.5 to -53.2)	2,197<br>(2,048 to 2,354)	-33.5%<br>(-35.8 to -30.9)	4,258<br>(3,612 to 4,981)	-63.7%<br>(-69.8 to -55.7)
**Bangladesh**	94,996<br>(85,170 to 103,929)	-28.5%<br>(-32.7 to -23.2)	87,155<br>(67,137 to 110,040)	-39.1%<br>(-52.3 to -22.3)	403,371<br>(375,283 to 433,403)	-24.2%<br>(-28.2 to -20.5)	2,170,499<br>(1,682,691 to 2,773,704)	-44.7%<br>(-57.3 to -27.9)

**Barbados**	116<br>(105 to 129)	-34.5%<br>(-38.0 to -30.5)	117<br>(94 to 144)	-39.6%<br>(-53.8 to -24.4)	597<br>(555 to 638)	-22.9%<br>(-25.8 to -19.6)	2,548<br>(2,021 to 3,171)	-41.8%<br>(-56.1 to -27.3)
**Belarus**	3,899<br>(3,525 to 4,278)	-30.7%<br>(-34.6 to -26.2)	3,051<br>(2,491 to 3,654)	-22.6%<br>(-40.2 to -2.8)	16,922<br>(15,540 to 18,370)	-20.7%<br>(-23.9 to -17.1)	78,410<br>(63,989 to 94,812)	-26.6%<br>(-43.0 to -8.6)
**Belgium**	3,135<br>(2,829 to 3,428)	-46.6%<br>(-50.4 to -42.6)	2,347<br>(1,987 to 2,589)	-56.9%<br>(-61.0 to -53.0)	16,494<br>(15,314 to 17,807)	-33.8%<br>(-37.0 to -30.4)	39,513<br>(34,941 to 43,057)	-61.8%<br>(-64.7 to -58.8)
**Belize**	88<br>(80 to 97)	-25.3%<br>(-29.2 to -21.0)	73<br>(64 to 82)	-34.6%<br>(-43.7 to -25.5)	519<br>(484 to 558)	-21.1%<br>(-24.4 to -17.9)	1,936<br>(1,698 to 2,176)	-36.7%<br>(-45.6 to -27.6)
**Benin**	4,110<br>(3,750 to 4,529)	-24.6%<br>(-29.1 to -19.1)	3,334<br>(2,561 to 4,094)	-31.7%<br>(-46.0 to -12.7)	23,764<br>(22,525 to 25,213)	-20.5%<br>(-23.7 to -17.3)	93,795<br>(71,075 to 115,992)	-33.6%<br>(-47.8 to -13.7)
**Bermuda**	17<br>(15 to 19)	-49.2%<br>(-53.0 to -45.6)	12<br>(10 to 14)	-61.2%<br>(-69.1 to -51.4)	122<br>(114 to 131)	-28.3%<br>(-30.9 to -25.5)	240<br>(202 to 286)	-61.9%<br>(-69.3 to -53.0)
**Bhutan**	224<br>(197 to 250)	-37.2%<br>(-41.8 to -32.3)	171<br>(130 to 219)	-45.1%<br>(-60.2 to -25.7)	1,139<br>(1,047 to 1,226)	-26.8%<br>(-30.4 to -23.0)	4,269<br>(3,180 to 5,470)	-48.4%<br>(-63.4 to -29.0)
**Bolivia (Plurinational State of)**	2,234<br>(2,001 to 2,465)	-46.2%<br>(-49.1 to -43.0)	2,363<br>(1,668 to 3,270)	-54.3%<br>(-64.9 to -36.6)	12,496<br>(11,477 to 13,469)	-43.2%<br>(-45.5 to -40.6)	62,243<br>(44,498 to 84,686)	-58.5%<br>(-69.2 to -42.3)
**Bosnia and Herzegovina**	1,055<br>(950 to 1,163)	-32.4%<br>(-36.8 to -27.1)	1,051<br>(811 to 1,309)	-48.8%<br>(-61.4 to -33.5)	3,770<br>(3,467 to 4,084)	-23.0%<br>(-25.7 to -20.1)	20,977<br>(16,131 to 26,391)	-52.4%<br>(-64.3 to -38.0)
**Botswana**	880<br>(787 to 969)	-25.4%<br>(-29.8 to -21.3)	618<br>(468 to 817)	-52.2%<br>(-65.2 to -34.4)	3,991<br>(3,711 to 4,263)	-25.4%<br>(-28.4 to -22.1)	16,866<br>(12,737 to 22,513)	-53.4%<br>(-66.7 to -35.5)
**Brazil**	51,212<br>(43,934 to 58,713)	-60.2%<br>(-62.4 to -57.6)	42,419<br>(39,721 to 44,402)	-65.1%<br>(-66.8 to -63.4)	283,407<br>(253,059 to 314,724)	-52.9%<br>(-54.8 to -50.8)	1,135,060<br>(1,081,881 to 1,181,019)	-66.4%<br>(-67.9 to -64.9)
**Brunei Darussalam**	124<br>(110 to 139)	-51.6%<br>(-55.1 to -47.8)	75<br>(64 to 89)	-53.7%<br>(-62.9 to -39.4)	1,123<br>(1,064 to 1,191)	-45.6%<br>(-48.1 to -43.0)	2,287<br>(1,947 to 2,699)	-55.4%<br>(-64.5 to -42.8)
**Bulgaria**	6,520<br>(6,090 to 6,940)	-52.8%<br>(-54.9 to -50.8)	5,562<br>(4,774 to 6,399)	-67.0%<br>(-71.5 to -62.1)	15,142<br>(13,945 to 16,555)	-46.6%<br>(-49.3 to -43.4)	114,715<br>(98,095 to 133,746)	-64.7%<br>(-69.9 to -58.5)
**Burkina Faso**	6,357<br>(5,774 to 6,968)	-9.2%<br>(-15.0 to -2.3)	5,043<br>(3,749 to 6,349)	-17.0%<br>(-35.1 to 6.8)	34,467<br>(32,334 to 36,737)	-13.6%<br>(-17.1 to -9.1)	148,575<br>(109,435 to 187,639)	-20.0%<br>(-37.9 to 4.4)
**Burundi**	3,610<br>(3,278 to 3,947)	-48.1%<br>(-50.9 to -45.1)	3,754<br>(2,884 to 4,657)	-50.3%<br>(-63.6 to -32.2)	16,506<br>(15,390 to 17,613)	-47.2%<br>(-49.5 to -44.6)	111,540<br>(86,083 to 138,710)	-53.4%<br>(-66.5 to -35.0)
**Cv¥te d'Ivoire**	9,037<br>(8,220 to 9,804)	-22.7%<br>(-27.1 to -17.4)	6,644<br>(4,768 to 8,746)	-20.7%<br>(-41.3 to 8.1)	57,876<br>(54,849 to 61,024)	-22.2%<br>(-25.0 to -19.1)	217,568<br>(155,228 to 286,404)	-22.7%<br>(-42.5 to 5.1)
**Cabo Verde**	247<br>(220 to 268)	-17.0%<br>(-22.0 to -11.5)	162<br>(127 to 202)	-18.9%<br>(-38.4 to 7.8)	1,564<br>(1,478 to 1,657)	-22.7%<br>(-25.5 to -19.7)	4,198<br>(3,277 to 5,268)	-27.8%<br>(-45.1 to -4.8)
**Cambodia**	10,875<br>(10,011 to 11,789)	-21.4%<br>(-25.5 to -17.7)	11,226<br>(8,742 to 13,978)	-31.1%<br>(-47.5 to -12.1)	38,227<br>(35,641 to 40,763)	-19.2%<br>(-22.6 to -15.6)	289,683<br>(221,551 to 363,150)	-36.8%<br>(-52.0 to -18.4)
**Cameroon**	11,010<br>(10,105 to 12,072)	-16.3%<br>(-21.0 to -10.7)	8,897<br>(6,143 to 12,323)	-19.0%<br>(-38.8 to 12.2)	63,589<br>(60,032 to 67,195)	-13.0%<br>(-16.5 to -9.4)	276,017<br>(189,171 to 380,907)	-19.4%<br>(-40.1 to 11.7)
**Canada**	8,894<br>(7,654 to 9,868)	-27.1%<br>(-33.5 to -19.6)	4,563<br>(3,960 to 4,946)	-39.5%<br>(-44.4 to -34.1)	89,586<br>(84,487 to 94,780)	-7.7%<br>(-11.1 to -4.2)	90,457<br>(82,077 to 96,911)	-41.3%<br>(-45.6 to -36.8)
**Central African Republic**	2,057<br>(1,853 to 2,261)	-22.9%<br>(-26.9 to -18.2)	2,276<br>(1,582 to 3,194)	-18.3%<br>(-34.9 to 2.3)	8,649<br>(8,122 to 9,263)	-24.3%<br>(-27.6 to -20.8)	72,279<br>(49,271 to 103,092)	-21.0%<br>(-39.3 to 0.4)

**Chad**	5,784<br>(5,295 to 6,293)	-6.1%<br>(-11.3 to 0.0)	4,626<br>(3,403 to 6,195)	-4.3%<br>(-28.4 to 24.2)	31,854<br>(30,225 to 33,695)	-8.5%<br>(-11.9 to -4.7)	151,884<br>(111,464 to 201,773)	-4.5%<br>(-28.7 to 24.3)
**Chile**	5,737<br>(5,111 to 6,328)	-48.7%<br>(-52.2 to -45.2)	3,021<br>(2,762 to 3,257)	-65.6%<br>(-68.3 to -63.0)	49,555<br>(46,531 to 52,499)	-27.3%<br>(-30.6 to -23.8)	71,369<br>(66,204 to 76,532)	-67.0%<br>(-69.4 to -64.8)
**China**	1,173,288<br>(1,003,993 to 1,330,455)	-43.9%<br>(-46.7 to -40.5)	1,322,893<br>(1,108,046 to 1,567,711)	-50.7%<br>(-60.3 to -39.9)	4,385,240<br>(3,892,101 to 4,906,565)	-28.0%<br>(-30.6 to -25.3)	27,463,746<br>(22,839,243 to 32,676,709)	-52.2%<br>(-61.2 to -41.1)
**Colombia**	8,568<br>(7,662 to 9,538)	-57.6%<br>(-60.4 to -54.2)	5,046<br>(4,215 to 5,931)	-69.8%<br>(-74.8 to -64.6)	60,284<br>(56,115 to 64,452)	-44.1%<br>(-46.7 to -41.3)	118,100<br>(98,664 to 139,123)	-70.7%<br>(-75.7 to -65.8)
**Comoros**	257<br>(232 to 285)	-42.2%<br>(-45.7 to -38.7)	279<br>(214 to 351)	-45.0%<br>(-58.9 to -24.9)	1,247<br>(1,162 to 1,342)	-38.7%<br>(-40.9 to -36.2)	7,392<br>(5,666 to 9,299)	-48.0%<br>(-62.0 to -27.9)
**Congo**	1,710<br>(1,534 to 1,895)	-42.1%<br>(-45.1 to -38.5)	1,785<br>(1,315 to 2,327)	-41.2%<br>(-54.1 to -23.9)	8,344<br>(7,810 to 8,941)	-39.6%<br>(-41.9 to -37.3)	52,248<br>(38,140 to 69,644)	-43.5%<br>(-57.1 to -26.4)
**Cook Islands**	11<br>(10 to 12)	-37.5%<br>(-41.7 to -33.4)	9<br>(8 to 11)	-54.1%<br>(-63.8 to -43.0)	61<br>(57 to 65)	-18.8%<br>(-22.3 to -15.0)	220<br>(178 to 266)	-52.9%<br>(-63.5 to -40.7)
**Costa Rica**	855<br>(749 to 960)	-40.2%<br>(-43.9 to -36.6)	424<br>(370 to 474)	-53.0%<br>(-59.2 to -47.1)	7,084<br>(6,658 to 7,586)	-25.0%<br>(-27.7 to -22.0)	10,216<br>(8,998 to 11,349)	-51.8%<br>(-57.7 to -45.8)
**Croatia**	1,391<br>(1,281 to 1,507)	-62.2%<br>(-64.4 to -59.8)	1,371<br>(1,196 to 1,555)	-70.4%<br>(-74.1 to -66.1)	4,536<br>(4,169 to 4,920)	-51.3%<br>(-53.7 to -49.0)	25,824<br>(22,646 to 29,404)	-73.4%<br>(-76.9 to -69.3)
**Cuba**	3,753<br>(3,397 to 4,152)	-42.1%<br>(-45.6 to -38.4)	3,485<br>(3,017 to 3,947)	-42.7%<br>(-51.4 to -33.7)	20,360<br>(19,023 to 21,744)	-33.4%<br>(-36.4 to -30.3)	77,947<br>(67,022 to 88,956)	-44.8%<br>(-53.0 to -36.4)
**Cyprus**	357<br>(315 to 393)	-43.9%<br>(-48.8 to -38.7)	211<br>(172 to 247)	-73.1%<br>(-78.7 to -65.8)	2,277<br>(2,128 to 2,442)	-34.0%<br>(-37.1 to -31.4)	3,907<br>(3,293 to 4,514)	-71.7%<br>(-77.1 to -64.8)
**Czechia**	2,832<br>(2,494 to 3,196)	-54.1%<br>(-58.1 to -49.7)	1,775<br>(1,558 to 1,994)	-75.3%<br>(-78.6 to -71.9)	12,860<br>(11,773 to 13,945)	-37.0%<br>(-39.6 to -34.2)	35,133<br>(30,940 to 39,726)	-76.8%<br>(-79.8 to -73.6)
**Democratic People's Republic of Korea**	30,962<br>(28,575 to 33,440)	-19.7%<br>(-23.8 to -14.7)	37,079<br>(29,284 to 44,919)	-13.5%<br>(-32.4 to 14.6)	96,479<br>(90,954 to 102,494)	-22.3%<br>(-25.4 to -19.1)	907,833<br>(708,135 to 1,120,909)	-9.6%<br>(-31.2 to 20.7)
**Democratic Republic of the Congo**	25,441<br>(23,000 to 28,131)	-24.1%<br>(-28.2 to -19.0)	24,616<br>(17,489 to 33,334)	-13.1%<br>(-35.5 to 16.3)	113,491<br>(105,398 to 122,150)	-26.4%<br>(-29.7 to -23.0)	699,729<br>(495,548 to 941,354)	-18.8%<br>(-39.4 to 8.9)
**Denmark**	1,500<br>(1,313 to 1,679)	-46.8%<br>(-50.5 to -42.6)	1,207<br>(1,070 to 1,311)	-54.0%<br>(-57.8 to -50.3)	9,333<br>(8,690 to 10,068)	-28.9%<br>(-32.0 to -26.1)	20,960<br>(19,226 to 22,480)	-59.1%<br>(-61.9 to -55.9)
**Djibouti**	367<br>(323 to 408)	-34.4%<br>(-38.5 to -30.3)	360<br>(256 to 478)	-36.7%<br>(-53.8 to -12.5)	2,059<br>(1,920 to 2,200)	-30.1%<br>(-33.1 to -27.0)	10,687<br>(7,565 to 14,433)	-37.9%<br>(-55.8 to -12.2)
**Dominica**	21<br>(19 to 23)	-29.0%<br>(-32.9 to -25.0)	28<br>(24 to 34)	-35.3%<br>(-46.3 to -22.5)	103<br>(96 to 111)	-24.9%<br>(-27.5 to -21.9)	659<br>(552 to 799)	-34.3%<br>(-45.9 to -20.6)
**Dominican Republic**	4,416<br>(4,033 to 4,793)	3.1%<br>(-2.0 to 8.0)	3,432<br>(2,663 to 4,402)	-25.3%<br>(-42.9 to 1.8)	21,391<br>(19,978 to 22,890)	-4.7%<br>(-8.9 to -0.6)	92,540<br>(72,337 to 119,199)	-22.0%<br>(-40.8 to 5.1)
**Ecuador**	3,087<br>(2,791 to 3,384)	-41.7%<br>(-44.9 to -38.5)	2,177<br>(1,742 to 2,683)	-56.5%<br>(-65.6 to -45.4)	21,586<br>(20,035 to 23,291)	-33.1%<br>(-35.8 to -30.3)	54,638<br>(43,759 to 67,963)	-61.8%<br>(-70.1 to -52.1)
**Egypt**	16,886<br>(15,018 to 18,785)	-28.9%<br>(-33.8 to -23.2)	17,014<br>(10,723 to 23,174)	-61.9%<br>(-70.4 to -50.1)	104,297<br>(96,402 to 113,114)	-25.1%<br>(-28.0 to -22.2)	516,111<br>(339,533 to 692,517)	-65.0%<br>(-73.4 to -56.2)
**El Salvador**	1,246<br>(1,117 to 1,387)	-51.8%<br>(-54.8 to -48.6)	859<br>(694 to 1,049)	-56.1%<br>(-65.7 to -43.6)	7,392<br>(6,866 to 7,959)	-47.4%<br>(-49.8 to -45.2)	21,001<br>(16,889 to 25,704)	-58.3%<br>(-67.5 to -46.7)

**Equatorial Guinea**	258<br>(228 to 292)	-53.6%<br>(-56.8 to -49.7)	218<br>(138 to 321)	-64.1%<br>(-75.7 to -46.7)	1,583<br>(1,473 to 1,699)	-42.4%<br>(-44.9 to -40.0)	6,504<br>(4,025 to 9,705)	-66.2%<br>(-77.5 to -49.4)
**Eritrea**	2,199<br>(1,979 to 2,429)	-43.9%<br>(-47.2 to -40.8)	2,391<br>(1,782 to 3,197)	-41.4%<br>(-53.2 to -26.0)	10,516<br>(9,862 to 11,189)	-33.7%<br>(-36.2 to -30.8)	72,883<br>(52,892 to 97,728)	-45.3%<br>(-57.3 to -29.3)
**Estonia**	276<br>(242 to 308)	-68.7%<br>(-71.8 to -65.3)	197<br>(172 to 223)	-76.6%<br>(-79.7 to -73.4)	1,225<br>(1,122 to 1,336)	-49.6%<br>(-52.4 to -47.0)	3,988<br>(3,518 to 4,494)	-76.9%<br>(-80.1 to -73.7)
**Eswatini**	353<br>(319 to 383)	-9.6%<br>(-13.9 to -4.8)	434<br>(291 to 622)	-18.1%<br>(-41.5 to 14.0)	1,511<br>(1,403 to 1,638)	-8.5%<br>(-12.4 to -4.4)	12,607<br>(8,300 to 18,352)	-14.2%<br>(-40.1 to 21.9)
**Ethiopia**	23,136<br>(20,179 to 26,013)	-57.3%<br>(-59.9 to -54.2)	20,820<br>(16,700 to 25,525)	-59.7%<br>(-71.1 to -48.1)	111,674<br>(99,979 to 125,164)	-53.2%<br>(-55.4 to -50.8)	568,468<br>(458,477 to 688,994)	-62.7%<br>(-74.4 to -51.5)
**Fiji**	383<br>(348 to 424)	-36.7%<br>(-40.5 to -32.6)	514<br>(400 to 646)	-26.8%<br>(-42.8 to -3.6)	2,203<br>(2,073 to 2,339)	-29.6%<br>(-32.4 to -26.5)	14,050<br>(10,868 to 17,756)	-30.6%<br>(-46.1 to -8.2)
**Finland**	2,051<br>(1,812 to 2,305)	-26.9%<br>(-32.0 to -21.3)	1,125<br>(957 to 1,234)	-49.9%<br>(-55.9 to -44.2)	15,838<br>(14,953 to 16,772)	-10.1%<br>(-13.4 to -6.7)	20,286<br>(18,147 to 21,938)	-55.7%<br>(-60.0 to -51.1)
**France**	17,253<br>(15,444 to 18,888)	-35.2%<br>(-40.2 to -30.8)	11,601<br>(9,953 to 12,685)	-60.2%<br>(-63.6 to -57.1)	102,960<br>(95,646 to 110,311)	-15.3%<br>(-18.9 to -11.5)	187,821<br>(168,461 to 204,118)	-63.4%<br>(-66.2 to -60.5)
**Gabon**	549<br>(487 to 617)	-38.9%<br>(-42.4 to -35.2)	528<br>(380 to 713)	-39.2%<br>(-53.6 to -20.2)	2,877<br>(2,685 to 3,091)	-34.2%<br>(-36.9 to -31.1)	14,289<br>(9,847 to 19,672)	-40.8%<br>(-55.4 to -20.4)
**Gambia**	762<br>(688 to 837)	-12.0%<br>(-17.0 to -6.0)	725<br>(548 to 930)	-6.2%<br>(-30.0 to 27.3)	4,255<br>(4,012 to 4,526)	-19.0%<br>(-22.7 to -15.2)	21,238<br>(16,361 to 26,960)	-8.9%<br>(-32.0 to 25.4)
**Georgia**	4,232<br>(3,857 to 4,600)	-41.7%<br>(-44.7 to -38.3)	3,388<br>(2,969 to 3,783)	-50.0%<br>(-58.2 to -41.0)	11,230<br>(10,441 to 12,086)	-41.2%<br>(-43.7 to -38.6)	67,427<br>(58,933 to 76,196)	-52.4%<br>(-60.0 to -43.7)
**Germany**	24,874<br>(22,250 to 27,565)	-37.9%<br>(-44.1 to -31.9)	14,801<br>(12,749 to 16,158)	-58.3%<br>(-63.7 to -53.2)	179,961<br>(168,862 to 191,748)	-19.7%<br>(-23.5 to -16.2)	266,748<br>(240,637 to 287,154)	-62.0%<br>(-66.3 to -58.0)
**Ghana**	14,326<br>(13,067 to 15,797)	-18.8%<br>(-23.6 to -13.2)	12,668<br>(9,411 to 16,306)	-23.0%<br>(-42.3 to 4.8)	87,942<br>(83,252 to 92,915)	-18.0%<br>(-21.0 to -14.6)	382,247<br>(285,721 to 486,121)	-26.9%<br>(-45.2 to 0.3)
**Greece**	6,577<br>(5,911 to 7,195)	-45.8%<br>(-49.3 to -42.3)	5,589<br>(4,894 to 6,038)	-60.5%<br>(-63.0 to -57.9)	26,946<br>(25,254 to 28,704)	-34.6%<br>(-37.7 to -31.7)	89,144<br>(80,288 to 95,133)	-59.2%<br>(-61.6 to -56.7)
**Greenland**	20<br>(18 to 22)	-40.5%<br>(-43.9 to -36.6)	16<br>(13 to 18)	-54.3%<br>(-62.3 to -43.8)	124<br>(116 to 132)	-38.0%<br>(-40.6 to -35.6)	396<br>(332 to 463)	-56.0%<br>(-63.9 to -47.7)
**Grenada**	36<br>(33 to 39)	-40.2%<br>(-43.4 to -36.6)	35<br>(31 to 39)	-49.1%<br>(-57.7 to -40.2)	180<br>(168 to 194)	-32.4%<br>(-35.0 to -29.2)	871<br>(750 to 979)	-54.2%<br>(-62.1 to -45.7)
**Guam**	82<br>(74 to 90)	-26.6%<br>(-31.4 to -21.7)	48<br>(42 to 54)	-50.8%<br>(-57.4 to -42.1)	471<br>(441 to 504)	-6.5%<br>(-10.3 to -2.0)	1,410<br>(1,255 to 1,589)	-34.7%<br>(-43.6 to -23.6)
**Guatemala**	3,272<br>(3,020 to 3,568)	-29.8%<br>(-34.3 to -25.3)	2,218<br>(1,898 to 2,548)	-36.7%<br>(-47.0 to -26.0)	20,698<br>(19,491 to 22,015)	-37.2%<br>(-39.6 to -34.8)	58,589<br>(50,234 to 67,434)	-42.9%<br>(-52.4 to -33.5)
**Guinea**	5,424<br>(4,991 to 5,903)	-8.7%<br>(-13.9 to -2.3)	4,199<br>(3,048 to 5,404)	-14.7%<br>(-36.3 to 16.6)	28,478<br>(26,956 to 30,191)	-10.6%<br>(-14.3 to -6.7)	127,742<br>(92,467 to 165,084)	-17.2%<br>(-38.7 to 13.1)
**Guinea-Bissau**	776<br>(710 to 846)	-24.1%<br>(-28.4 to -19.8)	789<br>(590 to 1,009)	-22.8%<br>(-42.0 to -0.6)	4,219<br>(4,011 to 4,483)	-27.8%<br>(-30.6 to -24.8)	25,872<br>(19,420 to 33,090)	-27.6%<br>(-46.3 to -5.0)
**Guyana**	390<br>(359 to 420)	-50.5%<br>(-53.1 to -48.3)	374<br>(287 to 475)	-59.3%<br>(-69.4 to -48.3)	1,628<br>(1,535 to 1,732)	-45.6%<br>(-48.7 to -42.7)	10,348<br>(7,879 to 13,297)	-60.2%<br>(-70.4 to -48.7)

**Haiti**	5,047<br>(4,600 to 5,478)	-34.4%<br>(-37.5 to -31.4)	5,954<br>(4,325 to 8,006)	-36.4%<br>(-52.7 to -16.2)	22,257<br>(20,892 to 23,755)	-33.2%<br>(-35.8 to -30.5)	179,645<br>(129,578 to 242,449)	-38.9%<br>(-55.0 to -18.2)
**Honduras**	1,807<br>(1,606 to 1,997)	-8.8%<br>(-13.6 to -4.0)	2,926<br>(2,338 to 3,636)	13.4%<br>(-9.7 to 44.5)	9,992<br>(9,264 to 10,830)	-27.5%<br>(-30.3 to -24.7)	73,226<br>(59,082 to 91,180)	-3.6%<br>(-23.9 to 24.7)
**Hungary**	3,050<br>(2,731 to 3,369)	-65.1%<br>(-67.5 to -62.7)	2,139<br>(1,833 to 2,447)	-79.0%<br>(-81.9 to -75.8)	11,912<br>(10,988 to 12,944)	-52.6%<br>(-54.6 to -50.2)	45,991<br>(39,477 to 52,795)	-77.7%<br>(-80.8 to -74.5)
**Iceland**	64<br>(57 to 72)	-51.9%<br>(-55.4 to -47.8)	38<br>(31 to 42)	-58.1%<br>(-62.8 to -53.2)	524<br>(491 to 559)	-34.2%<br>(-36.9 to -31.5)	670<br>(588 to 742)	-62.5%<br>(-66.2 to -58.5)
**India**	523,413<br>(446,706 to 604,615)	-29.3%<br>(-32.9 to -24.6)	411,023<br>(332,439 to 479,199)	-29.0%<br>(-40.8 to -15.5)	2,609,906<br>(2,326,121 to 2,944,799)	-11.2%<br>(-14.7 to -7.7)	11,098,171<br>(9,239,866 to 12,841,993)	-31.0%<br>(-41.6 to -18.8)
**Indonesia**	207,346<br>(176,874 to 238,266)	-17.2%<br>(-21.1 to -12.1)	241,504<br>(194,265 to 298,009)	-10.4%<br>(-28.1 to 8.0)	937,898<br>(828,663 to 1,062,485)	-25.7%<br>(-28.8 to -22.9)	6,741,746<br>(5,461,358 to 8,249,714)	-17.2%<br>(-32.1 to -0.8)
**Iran (Islamic Republic of)**	10,019<br>(8,702 to 11,432)	-41.2%<br>(-44.2 to -37.9)	6,875<br>(6,193 to 7,540)	-55.1%<br>(-60.3 to -48.3)	81,266<br>(72,921 to 90,120)	-29.0%<br>(-32.1 to -25.8)	186,754<br>(171,075 to 203,510)	-57.3%<br>(-62.6 to -51.1)
**Iraq**	11,445<br>(10,431 to 12,444)	-27.0%<br>(-31.1 to -22.2)	9,865<br>(7,601 to 12,358)	-35.8%<br>(-52.2 to -14.5)	73,419<br>(69,394 to 77,415)	-25.8%<br>(-28.2 to -23.0)	297,781<br>(228,418 to 374,238)	-44.0%<br>(-58.3 to -25.1)
**Ireland**	877<br>(770 to 985)	-50.8%<br>(-55.0 to -47.1)	426<br>(365 to 471)	-66.3%<br>(-69.8 to -62.9)	7,062<br>(6,600 to 7,577)	-33.7%<br>(-36.2 to -30.6)	8,101<br>(7,266 to 8,811)	-68.7%<br>(-71.3 to -66.1)
**Israel**	1,565<br>(1,381 to 1,733)	-53.1%<br>(-56.2 to -49.2)	1,077<br>(941 to 1,181)	-66.2%<br>(-69.7 to -63.2)	9,435<br>(8,633 to 10,197)	-34.2%<br>(-37.6 to -30.4)	20,101<br>(18,349 to 21,850)	-67.8%<br>(-70.7 to -65.1)
**Italy**	19,386<br>(17,001 to 21,929)	-42.1%<br>(-47.0 to -35.4)	17,121<br>(14,326 to 18,658)	-49.1%<br>(-52.7 to -46.4)	96,753<br>(85,927 to 107,522)	-31.2%<br>(-34.1 to -28.4)	269,662<br>(237,623 to 289,330)	-56.9%<br>(-59.3 to -54.6)
**Jamaica**	1,120<br>(1,022 to 1,221)	-22.7%<br>(-26.9 to -18.1)	1,100<br>(860 to 1,388)	-26.2%<br>(-44.1 to -3.4)	5,252<br>(4,899 to 5,607)	-19.1%<br>(-22.6 to -15.3)	25,032<br>(19,310 to 31,761)	-31.8%<br>(-48.6 to -11.4)
**Japan**	55,281<br>(48,068 to 62,043)	-43.4%<br>(-47.8 to -37.9)	43,254<br>(35,296 to 47,796)	-54.3%<br>(-56.9 to -52.2)	578,707<br>(508,783 to 643,656)	-22.6%<br>(-26.3 to -19.0)	776,369<br>(691,099 to 840,237)	-50.9%<br>(-52.6 to -49.2)
**Jordan**	1,511<br>(1,354 to 1,705)	-49.9%<br>(-53.2 to -46.5)	935<br>(742 to 1,136)	-62.5%<br>(-71.5 to -51.2)	12,379<br>(11,512 to 13,313)	-36.1%<br>(-38.7 to -33.6)	29,676<br>(24,459 to 35,227)	-66.5%<br>(-73.7 to -57.1)
**Kazakhstan**	9,921<br>(9,132 to 10,790)	-17.9%<br>(-22.1 to -12.2)	8,497<br>(7,335 to 9,767)	-0.9%<br>(-17.6 to 17.7)	36,472<br>(34,045 to 39,032)	-28.5%<br>(-31.5 to -25.4)	206,921<br>(176,004 to 239,608)	-13.6%<br>(-27.8 to 2.5)
**Kenya**	12,822<br>(11,113 to 14,655)	-21.6%<br>(-24.9 to -17.1)	11,369<br>(8,685 to 14,322)	-8.5%<br>(-25.5 to 13.4)	64,230<br>(57,605 to 72,140)	-21.0%<br>(-23.5 to -18.5)	309,639<br>(239,190 to 391,183)	-9.9%<br>(-26.7 to 11.7)
**Kiribati**	115<br>(106 to 123)	-25.8%<br>(-29.3 to -22.5)	83<br>(66 to 103)	-14.9%<br>(-31.4 to 7.7)	588<br>(559 to 619)	-26.4%<br>(-29.3 to -23.3)	2,705<br>(2,134 to 3,421)	-16.4%<br>(-34.0 to 7.5)
**Kuwait**	587<br>(525 to 654)	-6.1%<br>(-12.5 to 0.6)	213<br>(176 to 259)	-39.8%<br>(-50.8 to -28.1)	6,478<br>(6,056 to 6,900)	-9.2%<br>(-12.8 to -5.7)	7,483<br>(6,280 to 8,910)	-45.3%<br>(-54.7 to -35.0)
**Kyrgyzstan**	2,156<br>(1,978 to 2,334)	-50.6%<br>(-53.6 to -47.3)	1,318<br>(1,085 to 1,549)	-64.5%<br>(-70.7 to -57.3)	8,826<br>(8,201 to 9,457)	-50.5%<br>(-52.9 to -48.3)	39,214<br>(32,283 to 46,177)	-62.9%<br>(-69.5 to -55.2)
**Lao People's Democratic Republic**	4,141<br>(3,786 to 4,470)	-30.2%<br>(-34.1 to -25.9)	4,316<br>(3,309 to 5,384)	-43.6%<br>(-58.2 to -25.0)	17,739<br>(16,757 to 18,850)	-25.1%<br>(-28.3 to -21.8)	126,434<br>(98,017 to 159,613)	-47.8%<br>(-61.9 to -30.0)
**Latvia**	726<br>(661 to 791)	-61.1%<br>(-63.5 to -58.6)	625<br>(550 to 701)	-67.7%<br>(-71.5 to -63.1)	2,323<br>(2,109 to 2,537)	-43.1%<br>(-45.8 to -39.9)	12,811<br>(11,287 to 14,340)	-66.3%<br>(-70.4 to -61.7)

**Lebanon**	1,117<br>(991 to 1,245)	-49.8%<br>(-52.6 to -47.0)	916<br>(750 to 1,110)	-75.6%<br>(-81.7 to -67.6)	7,765<br>(7,289 to 8,319)	-30.9%<br>(-33.2 to -28.2)	19,417<br>(16,415 to 23,159)	-76.8%<br>(-82.5 to -68.9)
**Lesotho**	880<br>(806 to 950)	32.5%<br>(25.7 to 40.2)	1,205<br>(892 to 1,608)	35.7%<br>(-2.4 to 96.8)	2,840<br>(2,631 to 3,065)	22.4%<br>(16.0 to 29.4)	33,030<br>(24,087 to 45,246)	46.3%<br>(3.3 to 116.5)
**Liberia**	1,548<br>(1,398 to 1,701)	-27.9%<br>(-31.4 to -23.6)	1,427<br>(1,042 to 1,911)	-18.0%<br>(-39.2 to 11.9)	9,601<br>(9,042 to 10,171)	-30.6%<br>(-33.3 to -27.3)	45,497<br>(32,283 to 61,766)	-22.0%<br>(-42.6 to 6.4)
**Libya**	1,031<br>(925 to 1,143)	-30.5%<br>(-34.2 to -26.7)	988<br>(666 to 1,393)	-28.8%<br>(-48.4 to -3.3)	9,191<br>(8,657 to 9,797)	-30.3%<br>(-32.7 to -27.9)	33,845<br>(24,218 to 47,236)	-28.6%<br>(-46.7 to -5.0)
**Lithuania**	919<br>(824 to 1,003)	-31.7%<br>(-37.0 to -26.7)	675<br>(588 to 749)	-37.8%<br>(-45.5 to -28.5)	3,632<br>(3,331 to 3,939)	-19.4%<br>(-22.9 to -15.9)	14,124<br>(12,417 to 15,724)	-44.9%<br>(-51.7 to -37.4)
**Luxembourg**	121<br>(110 to 133)	-59.5%<br>(-61.9 to -56.8)	103<br>(90 to 114)	-69.0%<br>(-72.6 to -65.5)	775<br>(715 to 836)	-43.8%<br>(-46.9 to -40.4)	1,773<br>(1,591 to 1,966)	-73.4%<br>(-76.2 to -70.6)
**Madagascar**	11,932<br>(10,869 to 13,043)	-27.6%<br>(-30.8 to -24.1)	13,241<br>(9,935 to 17,160)	-24.4%<br>(-42.9 to -1.6)	60,236<br>(56,610 to 63,780)	-31.3%<br>(-34.1 to -28.1)	421,853<br>(315,226 to 545,905)	-26.6%<br>(-45.0 to -3.7)
**Malawi**	4,425<br>(4,033 to 4,809)	-31.2%<br>(-34.8 to -26.7)	5,836<br>(4,711 to 7,044)	-16.3%<br>(-33.7 to 4.0)	21,182<br>(19,772 to 22,631)	-28.9%<br>(-31.8 to -25.4)	170,118<br>(137,142 to 206,259)	-17.6%<br>(-34.6 to 3.7)
**Malaysia**	15,319<br>(13,747 to 16,799)	-41.4%<br>(-45.1 to -37.4)	12,826<br>(11,480 to 14,251)	-41.0%<br>(-48.9 to -32.0)	87,577<br>(82,472 to 92,851)	-26.2%<br>(-29.4 to -23.1)	338,609<br>(308,232 to 372,901)	-43.4%<br>(-50.2 to -35.8)
**Maldives**	162<br>(146 to 179)	-57.8%<br>(-60.6 to -54.7)	115<br>(93 to 137)	-73.1%<br>(-79.1 to -66.4)	1,114<br>(1,054 to 1,178)	-52.8%<br>(-55.0 to -50.6)	3,162<br>(2,581 to 3,754)	-76.8%<br>(-82.1 to -70.7)
**Mali**	6,101<br>(5,541 to 6,717)	-23.0%<br>(-27.3 to -17.6)	5,048<br>(3,674 to 6,526)	-31.5%<br>(-46.0 to -12.4)	36,216<br>(34,237 to 38,448)	-21.9%<br>(-24.7 to -19.1)	161,748<br>(119,932 to 207,185)	-33.3%<br>(-47.6 to -13.7)
**Malta**	142<br>(123 to 163)	-48.5%<br>(-52.7 to -43.4)	77<br>(66 to 86)	-69.2%<br>(-72.5 to -65.5)	955<br>(892 to 1,012)	-27.2%<br>(-29.9 to -24.4)	1,449<br>(1,291 to 1,599)	-69.2%<br>(-72.2 to -65.7)
**Marshall Islands**	43<br>(39 to 47)	-15.9%<br>(-19.9 to -11.4)	43<br>(33 to 56)	-21.0%<br>(-36.7 to -1.6)	207<br>(195 to 220)	-12.3%<br>(-15.7 to -8.9)	1,391<br>(1,055 to 1,824)	-19.0%<br>(-36.2 to 3.3)
**Mauritania**	1,268<br>(1,134 to 1,399)	-36.4%<br>(-40.5 to -31.7)	1,148<br>(846 to 1,560)	-44.5%<br>(-58.8 to -22.6)	7,804<br>(7,324 to 8,317)	-33.4%<br>(-35.9 to -30.8)	31,346<br>(23,314 to 42,689)	-46.8%<br>(-60.3 to -26.9)
**Mauritius**	575<br>(515 to 644)	-53.7%<br>(-56.3 to -50.4)	472<br>(437 to 497)	-63.1%<br>(-65.9 to -60.7)	3,203<br>(3,000 to 3,420)	-39.2%<br>(-41.8 to -36.7)	13,045<br>(12,064 to 13,735)	-61.8%<br>(-65.1 to -59.2)
**Mexico**	21,743<br>(18,800 to 24,735)	-39.1%<br>(-41.9 to -35.5)	13,568<br>(11,953 to 15,127)	-47.5%<br>(-53.2 to -41.6)	170,534<br>(152,715 to 189,258)	-29.3%<br>(-32.1 to -26.7)	356,817<br>(316,088 to 400,701)	-44.9%<br>(-51.0 to -38.5)
**Micronesia (Federated States of)**	88<br>(79 to 95)	-20.6%<br>(-24.6 to -16.6)	87<br>(68 to 110)	-28.6%<br>(-43.7 to -6.8)	408<br>(383 to 433)	-20.1%<br>(-23.0 to -17.1)	2,671<br>(2,089 to 3,433)	-28.1%<br>(-44.5 to -3.1)
**Monaco**	12<br>(10 to 13)	-40.7%<br>(-44.8 to -36.3)	10<br>(8 to 12)	-57.7%<br>(-68.8 to -39.8)	79<br>(74 to 85)	-20.8%<br>(-24.0 to -17.6)	177<br>(141 to 220)	-56.9%<br>(-68.7 to -40.8)
**Mongolia**	3,384<br>(3,156 to 3,621)	-5.7%<br>(-11.3 to -0.2)	1,926<br>(1,590 to 2,306)	-45.2%<br>(-56.7 to -30.1)	11,622<br>(10,921 to 12,330)	-18.2%<br>(-21.7 to -14.8)	52,403<br>(43,264 to 62,909)	-45.4%<br>(-57.3 to -30.2)
**Montenegro**	1,058<br>(974 to 1,138)	-11.5%<br>(-15.7 to -6.5)	1,369<br>(1,172 to 1,595)	11.5%<br>(-6.2 to 32.5)	1,733<br>(1,577 to 1,928)	-30.0%<br>(-33.4 to -25.4)	22,949<br>(19,574 to 27,078)	-7.7%<br>(-24.0 to 10.0)
**Morocco**	9,578<br>(8,581 to 10,593)	-35.1%<br>(-38.4 to -31.1)	9,573<br>(6,557 to 12,620)	-40.3%<br>(-55.1 to -24.7)	51,539<br>(47,861 to 55,330)	-32.6%<br>(-34.7 to -30.1)	251,011<br>(172,482 to 336,757)	-47.0%<br>(-60.4 to -31.9)

**Mozambique**	11,381<br>(10,389 to 12,383)	-8.6%<br>(-13.6 to -2.7)	13,702<br>(10,453 to 17,639)	0.1%<br>(-24.4 to 27.8)	47,771<br>(44,976 to 50,931)	-2.1%<br>(-6.1 to 2.3)	408,783<br>(306,981 to 536,142)	3.3%<br>(-22.2 to 32.8)
**Myanmar**	38,112<br>(35,041 to 41,461)	-36.5%<br>(-39.9 to -32.4)	46,968<br>(37,644 to 58,912)	-43.0%<br>(-57.3 to -22.3)	149,593<br>(140,405 to 158,786)	-30.5%<br>(-33.4 to -27.4)	1,288,648<br>(1,035,049 to 1,629,588)	-46.8%<br>(-60.6 to -27.4)
**Namibia**	747<br>(682 to 820)	-31.4%<br>(-35.1 to -27.1)	892<br>(664 to 1,140)	-27.3%<br>(-45.6 to -5.2)	3,133<br>(2,922 to 3,372)	-30.7%<br>(-33.7 to -27.9)	23,665<br>(17,263 to 30,898)	-28.9%<br>(-48.2 to -6.9)
**Nauru**	7<br>(6 to 7)	-33.3%<br>(-37.0 to -29.7)	8<br>(7 to 11)	-17.3%<br>(-34.9 to 8.9)	40<br>(38 to 42)	-24.5%<br>(-27.7 to -21.2)	283<br>(216 to 371)	-15.7%<br>(-34.3 to 11.8)
**Nepal**	10,731<br>(9,411 to 12,148)	-27.2%<br>(-32.3 to -21.5)	8,755<br>(6,577 to 11,471)	-38.6%<br>(-54.8 to -15.9)	46,846<br>(42,949 to 50,673)	-24.4%<br>(-28.0 to -20.6)	225,958<br>(172,301 to 296,343)	-42.5%<br>(-57.4 to -22.3)
**Netherlands**	4,449<br>(3,919 to 4,966)	-39.2%<br>(-43.6 to -34.8)	2,985<br>(2,573 to 3,255)	-49.4%<br>(-53.6 to -45.4)	28,412<br>(26,438 to 30,410)	-24.2%<br>(-27.2 to -21.3)	50,324<br>(44,960 to 54,068)	-56.8%<br>(-60.0 to -53.7)
**New Zealand**	831<br>(720 to 950)	-44.0%<br>(-48.3 to -38.6)	623<br>(534 to 685)	-48.8%<br>(-53.7 to -44.1)	5,533<br>(4,899 to 6,168)	-25.4%<br>(-30.0 to -20.3)	11,167<br>(10,099 to 12,080)	-55.5%<br>(-59.1 to -51.8)
**Nicaragua**	1,164<br>(1,042 to 1,294)	-44.5%<br>(-47.7 to -40.7)	635<br>(540 to 773)	-51.6%<br>(-59.7 to -40.5)	7,511<br>(7,023 to 8,070)	-32.4%<br>(-34.9 to -29.9)	16,900<br>(14,386 to 20,430)	-54.0%<br>(-61.5 to -43.3)
**Niger**	6,895<br>(6,319 to 7,565)	-21.8%<br>(-26.5 to -17.6)	5,783<br>(3,929 to 7,888)	-17.8%<br>(-35.6 to 5.8)	38,254<br>(36,244 to 40,357)	-25.3%<br>(-28.3 to -22.2)	174,553<br>(117,976 to 240,970)	-22.7%<br>(-41.3 to 1.2)
**Nigeria**	54,201<br>(46,891 to 61,164)	-36.5%<br>(-39.5 to -32.5)	34,626<br>(26,515 to 44,352)	-54.2%<br>(-64.2 to -39.1)	330,731<br>(295,042 to 371,441)	-25.8%<br>(-28.1 to -23.4)	1,049,149<br>(791,436 to 1,372,018)	-53.8%<br>(-63.9 to -37.7)
**Niue**	1<br>(1 to 2)	-31.2%<br>(-34.9 to -26.8)	2<br>(1 to 2)	-28.6%<br>(-42.4 to -11.2)	7<br>(6 to 7)	-23.9%<br>(-26.5 to -20.1)	40<br>(33 to 48)	-27.1%<br>(-42.6 to -8.9)
**North Macedonia**	1,476<br>(1,351 to 1,592)	-25.1%<br>(-28.7 to -21.3)	1,502<br>(1,214 to 1,875)	-32.8%<br>(-47.7 to -13.9)	4,140<br>(3,849 to 4,441)	-36.1%<br>(-38.3 to -33.6)	32,191<br>(25,402 to 40,294)	-42.7%<br>(-55.3 to -25.9)
**Northern Mariana Islands**	29<br>(25 to 32)	-26.6%<br>(-31.7 to -21.2)	26<br>(23 to 29)	-35.5%<br>(-49.8 to -21.2)	159<br>(148 to 170)	-26.5%<br>(-29.9 to -22.8)	720<br>(623 to 793)	-37.8%<br>(-51.2 to -23.3)
**Norway**	1,223<br>(1,042 to 1,413)	-36.7%<br>(-40.9 to -30.9)	772<br>(664 to 835)	-48.6%<br>(-52.3 to -45.6)	8,633<br>(7,586 to 9,606)	-20.6%<br>(-23.8 to -17.5)	12,814<br>(11,578 to 13,739)	-56.2%<br>(-58.8 to -53.8)
**Oman**	622<br>(557 to 688)	-41.1%<br>(-44.8 to -37.0)	303<br>(237 to 374)	-62.2%<br>(-72.8 to -44.9)	6,146<br>(5,750 to 6,578)	-32.0%<br>(-34.6 to -28.9)	10,353<br>(8,120 to 12,561)	-65.9%<br>(-75.0 to -51.3)
**Pakistan**	73,838<br>(62,787 to 84,518)	-28.6%<br>(-32.3 to -24.0)	51,242<br>(41,527 to 64,735)	-16.8%<br>(-33.5 to 6.5)	412,066<br>(367,896 to 465,591)	-17.3%<br>(-20.8 to -13.2)	1,546,685<br>(1,234,501 to 1,920,048)	-15.6%<br>(-32.7 to 7.6)
**Palau**	18<br>(16 to 20)	-25.3%<br>(-29.4 to -20.7)	16<br>(13 to 19)	-29.5%<br>(-45.2 to -8.6)	98<br>(93 to 105)	-15.0%<br>(-18.6 to -11.1)	470<br>(383 to 577)	-28.1%<br>(-45.2 to -5.3)
**Palestine**	683<br>(621 to 749)	-41.0%<br>(-44.6 to -37.0)	524<br>(444 to 607)	-54.1%<br>(-64.3 to -40.8)	4,228<br>(3,903 to 4,559)	-33.6%<br>(-36.1 to -31.1)	16,081<br>(13,822 to 18,321)	-57.6%<br>(-66.6 to -45.0)
**Panama**	1,003<br>(898 to 1,103)	-42.0%<br>(-45.6 to -38.2)	696<br>(540 to 838)	-46.3%<br>(-58.1 to -35.2)	6,593<br>(6,178 to 7,027)	-28.3%<br>(-31.3 to -25.3)	16,320<br>(12,737 to 19,642)	-46.7%<br>(-58.0 to -35.8)
**Papua New Guinea**	3,310<br>(2,954 to 3,647)	-18.5%<br>(-24.0 to -12.5)	5,228<br>(3,843 to 6,698)	-22.4%<br>(-43.1 to 7.0)	14,670<br>(13,659 to 15,820)	-14.9%<br>(-18.5 to -10.5)	152,418<br>(112,896 to 198,066)	-24.1%<br>(-45.0 to 6.4)
**Paraguay**	1,475<br>(1,342 to 1,616)	-47.9%<br>(-51.2 to -44.2)	1,444<br>(1,100 to 1,820)	-44.9%<br>(-58.7 to -28.1)	8,560<br>(7,951 to 9,232)	-38.7%<br>(-41.0 to -36.1)	37,320<br>(28,431 to 47,156)	-45.4%<br>(-59.6 to -28.6)

**Peru**	5,497<br>(4,956 to 6,081)	-53.5%<br>(-56.2 to -50.5)	4,226<br>(3,257 to 5,548)	-57.5%<br>(-69.3 to -42.4)	43,642<br>(40,725 to 46,445)	-38.8%<br>(-41.4 to -36.6)	117,462<br>(90,446 to 150,853)	-57.2%<br>(-69.1 to -42.3)
**Philippines**	54,563<br>(47,210 to 62,616)	29.8%<br>(22.9 to 39.1)	51,846<br>(43,593 to 60,218)	-12.2%<br>(-25.8 to 3.0)	260,764<br>(233,404 to 292,701)	32.5%<br>(26.4 to 38.7)	1,542,378<br>(1,302,134 to 1,800,681)	-4.7%<br>(-19.5 to 12.5)
**Poland**	10,413<br>(8,928 to 11,848)	-56.2%<br>(-58.8 to -53.6)	8,757<br>(7,867 to 9,547)	-68.1%<br>(-70.8 to -65.3)	40,245<br>(36,015 to 45,300)	-46.1%<br>(-48.5 to -43.3)	185,170<br>(168,955 to 201,869)	-68.3%<br>(-71.0 to -65.2)
**Portugal**	3,922<br>(3,570 to 4,264)	-69.6%<br>(-71.2 to -67.5)	3,708<br>(3,260 to 4,023)	-76.3%<br>(-78.2 to -74.5)	17,969<br>(16,440 to 19,467)	-55.3%<br>(-58.1 to -52.3)	64,204<br>(58,521 to 68,889)	-77.2%<br>(-78.7 to -75.6)
**Puerto Rico**	894<br>(792 to 990)	-42.4%<br>(-45.4 to -38.5)	551<br>(451 to 643)	-52.6%<br>(-60.9 to -44.3)	6,306<br>(5,880 to 6,742)	-25.4%<br>(-28.1 to -22.1)	11,227<br>(9,324 to 13,092)	-51.5%<br>(-59.8 to -43.3)
**Qatar**	340<br>(291 to 391)	-45.3%<br>(-48.7 to -41.9)	107<br>(81 to 140)	-73.3%<br>(-80.1 to -64.9)	4,774<br>(4,469 to 5,087)	-41.2%<br>(-43.4 to -38.9)	4,312<br>(3,347 to 5,507)	-73.6%<br>(-80.2 to -65.0)
**Republic of Korea**	20,452<br>(17,866 to 22,876)	-77.1%<br>(-78.9 to -75.2)	11,075<br>(9,144 to 12,771)	-88.2%<br>(-89.8 to -86.0)	216,557<br>(204,152 to 228,038)	-63.9%<br>(-65.5 to -62.3)	245,744<br>(214,675 to 283,292)	-87.4%<br>(-88.8 to -84.9)
**Republic of Moldova**	1,906<br>(1,743 to 2,086)	-59.4%<br>(-61.8 to -56.8)	1,891<br>(1,696 to 2,105)	-64.0%<br>(-68.3 to -59.0)	6,268<br>(5,798 to 6,765)	-47.2%<br>(-50.0 to -44.2)	45,113<br>(40,383 to 50,633)	-62.0%<br>(-66.8 to -57.0)
**Romania**	11,302<br>(10,511 to 12,134)	-49.3%<br>(-51.8 to -46.8)	12,915<br>(11,433 to 14,558)	-52.0%<br>(-59.0 to -44.3)	29,521<br>(27,280 to 32,075)	-40.2%<br>(-42.7 to -37.8)	264,153<br>(232,990 to 297,279)	-53.0%<br>(-60.0 to -45.9)
**Russian Federation**	63,552<br>(54,799 to 71,646)	-34.8%<br>(-38.2 to -30.9)	57,522<br>(53,053 to 61,872)	-39.3%<br>(-43.7 to -34.9)	239,832<br>(210,561 to 272,091)	-22.1%<br>(-25.3 to -18.6)	1,393,904<br>(1,291,389 to 1,501,590)	-35.0%<br>(-39.9 to -29.9)
**Rwanda**	4,097<br>(3,676 to 4,547)	-57.1%<br>(-59.6 to -54.4)	4,288<br>(3,112 to 5,593)	-61.9%<br>(-73.4 to -48.4)	17,904<br>(16,731 to 19,312)	-57.0%<br>(-58.9 to -55.0)	116,450<br>(84,177 to 152,026)	-66.1%<br>(-76.9 to -53.1)
**Saint Kitts and Nevis**	29<br>(26 to 32)	-53.2%<br>(-55.8 to -50.4)	27<br>(23 to 32)	-56.7%<br>(-64.8 to -47.6)	135<br>(124 to 147)	-49.1%<br>(-52.0 to -46.3)	708<br>(579 to 848)	-59.2%<br>(-66.9 to -50.4)
**Saint Lucia**	73<br>(67 to 79)	-48.0%<br>(-50.9 to -44.7)	70<br>(58 to 83)	-56.9%<br>(-64.8 to -48.5)	366<br>(343 to 393)	-35.2%<br>(-37.6 to -32.5)	1,641<br>(1,350 to 1,967)	-57.4%<br>(-65.5 to -48.6)
**Saint Vincent and the Grenadines**	49<br>(45 to 53)	-13.3%<br>(-18.3 to -7.4)	48<br>(43 to 55)	-44.6%<br>(-52.7 to -35.7)	222<br>(208 to 237)	-21.4%<br>(-24.4 to -18.1)	1,186<br>(1,036 to 1,350)	-42.7%<br>(-51.0 to -33.1)
**Samoa**	121<br>(110 to 132)	-25.4%<br>(-30.3 to -20.3)	131<br>(109 to 158)	-24.4%<br>(-37.7 to -5.9)	627<br>(591 to 667)	-13.5%<br>(-17.0 to -9.1)	3,523<br>(2,903 to 4,323)	-22.3%<br>(-37.8 to -0.2)
**San Marino**	9<br>(8 to 11)	-34.0%<br>(-38.3 to -29.4)	5<br>(3 to 7)	-65.0%<br>(-76.5 to -49.8)	62<br>(57 to 66)	-18.2%<br>(-21.5 to -15.4)	90<br>(63 to 124)	-62.7%<br>(-74.9 to -46.8)
**Sao Tome and Principe**	87<br>(79 to 96)	-16.2%<br>(-20.8 to -11.0)	58<br>(48 to 73)	-17.8%<br>(-34.0 to 4.9)	575<br>(544 to 608)	-15.6%<br>(-18.6 to -12.5)	1,755<br>(1,408 to 2,227)	-17.9%<br>(-35.1 to 6.1)
**Saudi Arabia**	7,381<br>(6,725 to 8,117)	-39.5%<br>(-43.3 to -35.9)	5,692<br>(4,472 to 7,076)	-50.1%<br>(-62.7 to -30.7)	54,408<br>(50,851 to 58,236)	-18.6%<br>(-21.4 to -15.4)	206,491<br>(160,589 to 263,007)	-50.5%<br>(-63.6 to -31.6)
**Senegal**	5,077<br>(4,610 to 5,593)	-20.2%<br>(-24.9 to -15.1)	4,873<br>(3,660 to 6,244)	-20.7%<br>(-38.7 to 1.5)	31,481<br>(29,687 to 33,369)	-20.3%<br>(-23.2 to -17.0)	138,317<br>(104,373 to 175,927)	-26.4%<br>(-43.8 to -5.2)
**Serbia**	4,917<br>(4,522 to 5,296)	-49.4%<br>(-52.5 to -46.3)	4,832<br>(3,972 to 5,801)	-60.5%<br>(-69.1 to -50.0)	11,699<br>(10,746 to 12,686)	-47.8%<br>(-50.1 to -45.6)	94,196<br>(77,373 to 112,748)	-61.6%<br>(-69.8 to -51.4)
**Seychelles**	46<br>(41 to 50)	-36.0%<br>(-39.9 to -31.6)	38<br>(33 to 44)	-46.2%<br>(-53.8 to -36.9)	267<br>(250 to 284)	-27.5%<br>(-30.5 to -24.3)	1,045<br>(902 to 1,207)	-48.6%<br>(-55.6 to -40.6)

**Sierra Leone**	3,049<br>(2,762 to 3,326)	-14.5%<br>(-18.8 to -8.5)	2,675<br>(1,975 to 3,562)	-14.8%<br>(-33.8 to 11.0)	19,082<br>(18,068 to 20,209)	-15.5%<br>(-18.5 to -11.9)	85,990<br>(62,276 to 116,334)	-18.3%<br>(-36.9 to 8.1)
**Singapore**	1,396<br>(1,216 to 1,572)	-64.9%<br>(-67.8 to -62.0)	496<br>(446 to 537)	-80.2%<br>(-82.1 to -78.3)	17,051<br>(16,166 to 17,987)	-50.9%<br>(-53.1 to -48.4)	13,868<br>(12,728 to 14,989)	-79.3%<br>(-80.8 to -77.8)
**Slovakia**	1,536<br>(1,394 to 1,684)	-52.7%<br>(-55.2 to -49.7)	1,440<br>(1,204 to 1,743)	-63.9%<br>(-70.4 to -54.8)	6,395<br>(5,883 to 6,911)	-36.4%<br>(-39.3 to -33.7)	31,471<br>(26,505 to 37,983)	-65.6%<br>(-71.7 to -56.6)
**Slovenia**	528<br>(475 to 582)	-58.7%<br>(-61.2 to -56.0)	342<br>(289 to 387)	-70.1%<br>(-74.1 to -65.9)	2,058<br>(1,891 to 2,250)	-45.3%<br>(-47.9 to -42.5)	5,812<br>(5,000 to 6,549)	-76.8%<br>(-79.8 to -73.6)
**Solomon Islands**	714<br>(664 to 763)	-9.7%<br>(-14.2 to -4.4)	479<br>(376 to 606)	-14.0%<br>(-32.3 to 13.4)	2,764<br>(2,625 to 2,932)	-11.5%<br>(-15.3 to -7.2)	13,946<br>(10,862 to 17,853)	-13.3%<br>(-34.8 to 22.7)
**Somalia**	5,562<br>(4,999 to 6,080)	-32.2%<br>(-35.6 to -28.3)	5,868<br>(4,086 to 8,130)	-32.7%<br>(-48.2 to -11.5)	24,781<br>(23,195 to 26,475)	-32.7%<br>(-35.5 to -29.8)	185,015<br>(124,189 to 261,150)	-34.4%<br>(-51.2 to -11.2)
**South Africa**	15,401<br>(13,262 to 17,627)	-35.0%<br>(-38.9 to -31.1)	19,000<br>(17,069 to 20,756)	1.9%<br>(-9.1 to 19.1)	79,659<br>(71,380 to 89,614)	-42.0%<br>(-44.5 to -39.2)	513,344<br>(460,157 to 559,193)	-11.3%<br>(-19.1 to -1.2)
**South Sudan**	2,284<br>(2,047 to 2,548)	-32.6%<br>(-36.3 to -28.6)	2,897<br>(2,024 to 4,046)	-29.3%<br>(-47.3 to -5.0)	11,058<br>(10,357 to 11,883)	-34.7%<br>(-37.3 to -32.0)	88,306<br>(60,388 to 124,037)	-30.2%<br>(-49.1 to -3.6)
**Spain**	13,024<br>(11,597 to 14,241)	-55.6%<br>(-58.7 to -52.5)	9,634<br>(8,086 to 10,698)	-66.6%<br>(-69.3 to -64.0)	70,053<br>(65,283 to 75,231)	-48.2%<br>(-50.7 to -45.3)	158,737<br>(140,787 to 172,071)	-69.6%<br>(-71.7 to -67.5)
**Sri Lanka**	6,495<br>(5,720 to 7,211)	-41.3%<br>(-45.2 to -37.4)	9,520<br>(6,512 to 12,873)	-45.0%<br>(-63.6 to -22.9)	38,549<br>(35,754 to 41,501)	-30.3%<br>(-32.9 to -27.5)	212,752<br>(142,521 to 290,905)	-46.9%<br>(-65.3 to -24.8)
**Sudan**	7,239<br>(6,482 to 7,973)	-49.2%<br>(-51.8 to -46.5)	6,850<br>(4,598 to 9,477)	-60.5%<br>(-70.9 to -44.8)	47,126<br>(44,116 to 50,529)	-40.5%<br>(-42.7 to -38.4)	229,271<br>(158,311 to 319,231)	-64.0%<br>(-75.3 to -48.4)
**Suriname**	302<br>(277 to 327)	-20.0%<br>(-23.7 to -15.5)	287<br>(222 to 355)	-31.8%<br>(-48.1 to -13.3)	1,304<br>(1,230 to 1,392)	-25.4%<br>(-28.7 to -21.9)	7,620<br>(5,979 to 9,331)	-31.0%<br>(-47.3 to -12.7)
**Sweden**	2,873<br>(2,392 to 3,351)	-30.7%<br>(-35.3 to -25.7)	1,791<br>(1,530 to 1,998)	-48.6%<br>(-54.2 to -43.0)	25,398<br>(22,242 to 28,496)	-6.9%<br>(-12.2 to -1.6)	30,434<br>(26,712 to 33,779)	-52.2%<br>(-56.8 to -47.2)
**Switzerland**	1,788<br>(1,564 to 2,010)	-45.8%<br>(-50.5 to -41.0)	941<br>(773 to 1,049)	-67.0%<br>(-71.0 to -62.7)	13,431<br>(12,577 to 14,374)	-25.9%<br>(-28.8 to -23.1)	15,772<br>(13,805 to 17,362)	-69.6%<br>(-72.8 to -66.0)
**Syrian Arab Republic**	4,653<br>(4,240 to 5,064)	-39.0%<br>(-42.0 to -36.1)	4,109<br>(3,106 to 5,277)	-42.1%<br>(-58.4 to -14.5)	28,511<br>(26,892 to 30,200)	-42.9%<br>(-44.8 to -40.8)	115,702<br>(88,144 to 150,989)	-52.2%<br>(-65.8 to -30.5)
**Turkey**	22,173<br>(19,642 to 24,612)	-50.0%<br>(-53.3 to -46.6)	18,325<br>(15,013 to 22,123)	-59.4%<br>(-67.7 to -47.5)	146,139<br>(136,158 to 155,838)	-42.7%<br>(-45.0 to -40.6)	414,909<br>(344,731 to 499,256)	-65.4%<br>(-72.7 to -56.3)
**Taiwan (Province of China)**	13,756<br>(12,119 to 15,397)	-58.5%<br>(-61.4 to -55.2)	6,601<br>(5,914 to 7,132)	-79.2%<br>(-80.9 to -77.7)	73,211<br>(67,738 to 78,831)	-37.6%<br>(-39.7 to -35.5)	157,562<br>(144,186 to 169,334)	-76.4%<br>(-78.2 to -74.7)
**Tajikistan**	4,012<br>(3,648 to 4,383)	0.1%<br>(-5.6 to 6.8)	2,600<br>(2,024 to 3,240)	-25.1%<br>(-45.2 to 0.5)	12,341<br>(11,407 to 13,257)	-31.0%<br>(-34.2 to -27.8)	69,630<br>(54,485 to 87,307)	-32.6%<br>(-50.5 to -9.6)
**Thailand**	36,765<br>(32,600 to 40,278)	-46.2%<br>(-49.7 to -41.9)	32,814<br>(25,457 to 40,801)	-49.2%<br>(-61.5 to -32.6)	197,962<br>(183,713 to 210,724)	-29.0%<br>(-31.7 to -26.1)	827,878<br>(651,226 to 1,029,518)	-42.9%<br>(-56.7 to -24.7)
**Timor-Leste**	626<br>(570 to 688)	-6.9%<br>(-12.5 to -1.0)	795<br>(587 to 1,022)	-16.3%<br>(-38.5 to 11.3)	2,354<br>(2,197 to 2,520)	-9.0%<br>(-12.7 to -4.9)	20,932<br>(15,553 to 27,236)	-19.0%<br>(-40.9 to 7.2)
**Togo**	2,947<br>(2,663 to 3,247)	-18.1%<br>(-22.2 to -13.5)	2,807<br>(2,029 to 3,690)	-10.3%<br>(-32.2 to 17.7)	16,999<br>(16,055 to 18,109)	-21.0%<br>(-24.0 to -17.9)	85,828<br>(60,621 to 113,149)	-14.4%<br>(-36.7 to 13.3)

**Tokelau**	1<br>(1 to 1)	-42.0%<br>(-45.8 to -38.0)	1<br>(1 to 1)	-42.3%<br>(-54.7 to -27.8)	4<br>(4 to 4)	-26.5%<br>(-29.4 to -23.3)	27<br>(21 to 34)	-39.4%<br>(-52.5 to -23.3)
**Tonga**	38<br>(34 to 42)	-21.9%<br>(-26.8 to -16.4)	34<br>(27 to 42)	-20.1%<br>(-40.0 to 5.4)	191<br>(179 to 203)	-15.8%<br>(-19.3 to -12.5)	831<br>(648 to 1,032)	-21.4%<br>(-41.6 to 4.2)
**Trinidad and Tobago**	443<br>(399 to 490)	-47.2%<br>(-50.4 to -43.5)	417<br>(324 to 533)	-52.6%<br>(-64.0 to -39.0)	2,424<br>(2,265 to 2,606)	-32.4%<br>(-35.1 to -29.7)	10,306<br>(7,905 to 13,216)	-51.4%<br>(-63.3 to -37.0)
**Tunisia**	2,696<br>(2,377 to 3,014)	-38.0%<br>(-41.4 to -34.0)	2,320<br>(1,417 to 3,619)	-50.0%<br>(-66.5 to -29.8)	15,939<br>(14,831 to 17,092)	-23.0%<br>(-26.0 to -20.4)	55,139<br>(35,004 to 83,689)	-51.8%<br>(-67.7 to -32.2)
**Turkmenistan**	2,775<br>(2,551 to 2,995)	-9.8%<br>(-14.3 to -4.9)	2,488<br>(1,874 to 3,260)	24.1%<br>(-5.6 to 57.1)	11,575<br>(10,879 to 12,334)	1.6%<br>(-2.9 to 6.5)	73,336<br>(55,721 to 94,681)	22.3%<br>(-7.1 to 55.4)
**Tuvalu**	9<br>(8 to 10)	-32.3%<br>(-35.8 to -28.6)	10<br>(9 to 13)	-40.5%<br>(-51.0 to -27.3)	41<br>(39 to 44)	-22.2%<br>(-25.4 to -18.9)	290<br>(240 to 352)	-40.2%<br>(-51.1 to -25.7)
**Uganda**	9,466<br>(8,560 to 10,393)	-28.4%<br>(-32.1 to -24.3)	8,189<br>(5,891 to 10,928)	-41.8%<br>(-57.3 to -21.6)	45,270<br>(42,182 to 48,592)	-26.0%<br>(-28.9 to -22.9)	237,353<br>(175,517 to 316,183)	-42.4%<br>(-58.4 to -22.4)
**Ukraine**	21,148<br>(17,998 to 24,397)	-25.4%<br>(-30.7 to -20.0)	14,298<br>(10,820 to 18,222)	-31.6%<br>(-48.9 to -11.0)	83,782<br>(73,587 to 94,693)	-8.8%<br>(-14.2 to -2.9)	381,988<br>(282,138 to 487,859)	-25.6%<br>(-45.0 to -3.0)
**United Arab Emirates**	1,826<br>(1,559 to 2,116)	-42.7%<br>(-46.2 to -38.7)	491<br>(382 to 609)	-44.5%<br>(-57.1 to -29.1)	17,496<br>(16,420 to 18,676)	-32.6%<br>(-35.0 to -30.2)	18,961<br>(14,931 to 23,110)	-54.7%<br>(-65.6 to -42.3)
**United Kingdom**	16,453<br>(14,114 to 18,774)	-31.2%<br>(-34.8 to -26.6)	11,827<br>(10,455 to 12,573)	-40.1%<br>(-43.8 to -37.8)	96,392<br>(87,317 to 106,282)	-25.4%<br>(-27.2 to -23.3)	201,657<br>(186,833 to 212,286)	-48.2%<br>(-50.1 to -46.5)
**United Republic of Tanzania**	15,215<br>(13,875 to 16,728)	-29.4%<br>(-33.3 to -25.1)	12,632<br>(9,239 to 16,781)	-39.4%<br>(-53.8 to -17.6)	74,983<br>(70,639 to 80,009)	-23.9%<br>(-26.8 to -20.6)	344,582<br>(251,824 to 452,496)	-42.3%<br>(-56.7 to -21.0)
**United States of America**	70,680<br>(60,482 to 80,921)	-26.7%<br>(-30.8 to -21.8)	58,171<br>(52,026 to 61,737)	-12.6%<br>(-16.9 to -9.2)	753,477<br>(675,191 to 838,583)	6.3%<br>(1.8 to 10.8)	1,252,563<br>(1,164,768 to 1,317,343)	-17.2%<br>(-20.7 to -14.1)
**United States Virgin Islands**	40<br>(36 to 44)	-19.8%<br>(-24.7 to -14.6)	23<br>(18 to 29)	-59.3%<br>(-68.8 to -46.3)	167<br>(153 to 182)	-17.5%<br>(-20.7 to -14.2)	522<br>(415 to 653)	-57.5%<br>(-68.0 to -43.2)
**Uruguay**	1,170<br>(1,028 to 1,292)	-43.8%<br>(-47.5 to -40.0)	822<br>(746 to 881)	-57.3%<br>(-61.5 to -53.2)	7,979<br>(7,413 to 8,532)	-37.1%<br>(-39.8 to -34.6)	17,254<br>(15,965 to 18,479)	-61.3%<br>(-64.5 to -57.9)
**Uzbekistan**	20,663<br>(18,954 to 22,275)	7.2%<br>(0.9 to 14.3)	9,209<br>(7,893 to 10,710)	-43.9%<br>(-52.8 to -34.0)	63,469<br>(59,236 to 67,920)	-15.8%<br>(-19.6 to -11.4)	248,461<br>(211,433 to 290,713)	-47.9%<br>(-56.2 to -38.0)
**Vanuatu**	201<br>(185 to 218)	-11.8%<br>(-16.5 to -6.4)	187<br>(150 to 228)	-20.7%<br>(-35.5 to -2.6)	1,040<br>(988 to 1,100)	-6.8%<br>(-11.2 to -2.6)	5,819<br>(4,662 to 7,136)	-19.2%<br>(-37.1 to 3.0)
**Venezuela (Bolivarian Republic of)**	8,667<br>(7,808 to 9,554)	-33.1%<br>(-36.6 to -29.0)	7,156<br>(5,347 to 9,202)	-35.0%<br>(-52.2 to -16.4)	49,152<br>(46,162 to 52,698)	-28.0%<br>(-31.1 to -25.0)	182,530<br>(135,007 to 237,172)	-34.4%<br>(-51.6 to -15.8)
**Viet Nam**	74,925<br>(68,600 to 80,667)	-24.9%<br>(-28.7 to -20.4)	87,674<br>(70,631 to 104,639)	-26.9%<br>(-45.7 to -4.8)	279,214<br>(262,128 to 296,737)	-18.1%<br>(-21.6 to -14.4)	2,191,395<br>(1,747,215 to 2,643,822)	-27.6%<br>(-46.3 to -4.2)
**Yemen**	5,915<br>(5,357 to 6,478)	-43.5%<br>(-46.4 to -40.2)	6,830<br>(4,715 to 9,574)	-47.2%<br>(-61.6 to -25.4)	32,859<br>(30,598 to 35,251)	-37.8%<br>(-40.3 to -35.2)	209,389<br>(149,622 to 286,739)	-50.6%<br>(-64.5 to -30.1)
**Zambia**	6,019<br>(5,534 to 6,545)	-17.8%<br>(-22.6 to -12.5)	6,151<br>(4,557 to 8,062)	-23.4%<br>(-42.8 to 0.9)	25,833<br>(24,130 to 27,523)	-23.6%<br>(-26.7 to -20.3)	175,895<br>(128,002 to 236,042)	-24.5%<br>(-45.5 to 0.7)
**Zimbabwe**	2,443<br>(2,198 to 2,702)	2.4%<br>(-3.0 to 8.9)	5,327<br>(4,156 to 6,695)	42.7%<br>(9.2 to 91.6)	12,400<br>(11,493 to 13,526)	0.2%<br>(-4.6 to 4.9)	155,377<br>(119,000 to 199,626)	56.2%<br>(18.0 to 112.9)

Appendix Table 4. Incident cases, deaths, prevalent cases, and DALYs for subarachnoid haemorrhage in 2021 and percentage changes of age-standardised rates for 1990-2021, by location

Country, region	Incident cases (95% uncertainty interval)		Deaths (95% uncertainty interval)		Prevalent cases (95% uncertainty interval)		DALYs (95% uncertainty interval)	
	2021 counts	% change in age-standardised rates, 1990-2021	2021 counts	% change in age-standardised rates, 1990-2021	2021 counts	% change in age-standardised rates, 1990-2021	2021 counts	% change in age-standardised rates, 1990-2021
**Countries categorised by the World Bank Income level**								
**Global**	697,486 (614,334 to 795,785)	-28.8% (-31.6 to -25.7)	352,810 (309,015 to 401,474)	-56.1% (-64.3 to -40.7)	7,852,792 (7,164,804 to 8,578,767)	-16.1% (-17.7 to -14.8)	10,641,882 (9,398,963 to 12,121,263)	-54.6% (-61.9 to -42.8)
**High income**	146,669 (131,090 to 166,018)	-19.1% (-21.3 to -16.5)	69,265 (61,155 to 73,496)	-39.2% (-42.3 to -36.9)	2,124,949 (1,961,237 to 2,282,214)	-3.4% (-5.1 to -1.7)	1,802,415 (1,678,826 to 1,916,006)	-43.1% (-45.1 to -41.1)
**Upper Middle income**	267,598 (235,570 to 308,646)	-41.9% (-45.5 to -38.5)	155,409 (129,167 to 181,619)	-71.8% (-80.0 to -53.6)	2,726,092 (2,470,925 to 3,006,144)	-25.5% (-27.3 to -23.8)	4,261,235 (3,633,575 to 4,875,629)	-69.9% (-77.6 to -53.5)
**Lower Middle income**	250,056 (216,398 to 287,037)	-21.1% (-23.8 to -18.1)	115,385 (90,852 to 146,911)	-34.6% (-47.3 to -17.4)	2,616,363 (2,358,816 to 2,886,985)	-12.3% (-14.0 to -10.7)	4,074,006 (3,289,337 to 5,071,888)	-34.4% (-45.0 to -20.7)
**Low income**	32,548 (28,385 to 36,969)	-18.5% (-21.1 to -15.7)	12,440 (5,994 to 28,705)	-34.6% (-50.8 to -17.8)	378,329 (349,991 to 409,139)	-13.7% (-15.3 to -12.2)	492,714 (264,287 to 1,057,249)	-33.0% (-46.7 to -16.5)
**Countries categorised by the sociodemographic index (SDI) level**								
**High SDI**	130,354 (116,018 to 148,269)	-22.4% (-24.4 to -20.0)	59,891 (53,246 to 63,667)	-45.0% (-48.4 to -42.4)	1,936,626 (1,780,962 to 2,087,720)	-5.2% (-7.1 to -3.5)	1,591,961 (1,476,738 to 1,698,022)	-46.9% (-49.4 to -44.9)
**High-middle SDI**	127,288 (113,021 to 145,761)	-36.0% (-38.5 to -33.3)	67,001 (58,711 to 79,143)	-62.1% (-71.0 to -46.4)	1,313,530 (1,193,924 to 1,448,151)	-22.5% (-24.1 to -21.0)	1,809,134 (1,634,520 to 2,096,841)	-62.2% (-69.8 to -50.3)
**Middle SDI**	239,549 (209,800 to 276,045)	-38.3% (-42.7 to -34.6)	132,720 (109,151 to 153,401)	-70.8% (-78.6 to -50.1)	2,493,467 (2,255,823 to 2,751,504)	-22.9% (-24.8 to -21.2)	3,869,942 (3,304,695 to 4,361,939)	-67.9% (-75.5 to -50.8)
**Low-middle SDI**	145,850 (127,059 to 166,815)	-19.4% (-22.2 to -16.0)	71,892 (55,284 to 91,826)	-31.8% (-45.8 to -11.9)	1,502,242 (1,368,010 to 1,645,542)	-11.8% (-13.7 to -10.1)	2,526,899 (2,013,969 to 3,186,821)	-33.2% (-43.9 to -18.3)
**Low SDI**	53,833 (46,819 to 61,526)	-18.0% (-20.6 to -15.0)	20,996 (11,605 to 40,675)	-27.4% (-42.9 to -11.2)	599,907 (550,045 to 653,032)	-12.2% (-13.9 to -10.5)	833,678 (503,947 to 1,503,254)	-27.5% (-40.9 to -14.7)
**Countries categorised by the GBD super-regions**								
**Central Europe, Eastern Europe, and Central Asia**	48,263 (43,147 to 54,469)	-9.4% (-12.3 to -6.6)	29,217 (27,317 to 31,101)	-13.1% (-19.5 to -6.6)	436,526 (396,177 to 484,315)	-9.4% (-10.9 to -8.0)	809,933 (757,348 to 861,105)	-18.3% (-23.6 to -13.1)
**High-income**	135,260 (121,010 to 153,598)	-18.8% (-21.0 to -16.1)	64,203 (56,362 to 68,321)	-40.1% (-43.2 to -37.7)	2,002,293 (1,849,081 to 2,152,988)	-2.6% (-4.5 to -0.9)	1,663,406 (1,541,579 to 1,767,869)	-43.4% (-45.4 to -41.6)
**Latin America and Caribbean**	67,637 (59,743 to 76,924)	-17.5% (-20.3 to -14.7)	32,250 (29,576 to 34,801)	-17.0% (-23.8 to -10.4)	842,590 (780,377 to 911,019)	-15.9% (-17.3 to -14.5)	1,075,758 (998,225 to 1,160,262)	-26.2% (-32.5 to -20.8)

**North Africa and Middle East**	32,386<br>(28,114 to 36,307)	-31.7%<br>(-34.6 to -28.4)	12,261<br>(9,703 to 15,966)	-54.4%<br>(-72.7 to -35.2)	422,056<br>(389,157 to 456,659)	-22.1%<br>(-23.5 to -20.7)	433,904<br>(351,654 to 547,035)	-57.9%<br>(-72.3 to -45.4)
**South Asia**	140,534<br>(120,483 to 162,708)	-22.6%<br>(-25.9 to -19.2)	67,618<br>(48,401 to 93,444)	-33.9%<br>(-49.6 to -7.7)	1,355,698<br>(1,203,895 to 1,512,996)	-11.8%<br>(-14.2 to -9.7)	2,362,779<br>(1,772,137 to 3,126,993)	-33.8%<br>(-47.0 to -14.0)
**Southeast Asia, East Asia, and Oceania**	227,898<br>(198,688 to 264,184)	-47.4%<br>(-51.1 to -43.9)	133,463<br>(107,489 to 162,727)	-76.9%<br>(-84.1 to -58.8)	2,212,273<br>(1,991,892 to 2,454,350)	-28.2%<br>(-30.3 to -26.4)	3,707,425<br>(3,055,740 to 4,421,410)	-73.9%<br>(-80.9 to -57.7)
**Sub-Saharan Africa**	45,508<br>(39,100 to 51,997)	-15.5%<br>(-17.4 to -13.3)	13,799<br>(6,748 to 33,225)	-27.0%<br>(-44.2 to -11.2)	581,356<br>(534,996 to 632,860)	-12.0%<br>(-13.5 to -10.3)	588,678<br>(324,402 to 1,272,728)	-25.8%<br>(-41.0 to -12.0)
**Countries categorised by the GBD regions**								
**Andean Latin America**	7,842<br>(7,001 to 8,776)	-24.6%<br>(-27.6 to -21.1)	3,642<br>(2,951 to 4,430)	-25.7%<br>(-44.9 to -4.7)	108,449<br>(103,325 to 113,846)	-17.5%<br>(-19.5 to -15.4)	126,533<br>(105,431 to 151,575)	-32.4%<br>(-48.0 to -15.9)
**Australasia**	2,630<br>(2,332 to 2,987)	-20.7%<br>(-25.4 to -14.7)	1,321<br>(1,160 to 1,435)	-45.3%<br>(-49.7 to -41.0)	35,127<br>(32,840 to 37,590)	-9.3%<br>(-12.4 to -6.3)	33,262<br>(30,723 to 35,750)	-48.4%<br>(-51.8 to -44.8)
**Caribbean**	5,327<br>(4,777 to 6,036)	-9.3%<br>(-12.6 to -5.4)	2,627<br>(2,037 to 3,279)	-22.3%<br>(-35.5 to -7.3)	71,256<br>(67,509 to 74,883)	-9.8%<br>(-11.3 to -8.0)	96,276<br>(73,559 to 118,612)	-21.8%<br>(-35.1 to -5.7)
**Central Asia**	7,814<br>(6,960 to 8,734)	-3.6%<br>(-7.2 to 0.0)	4,006<br>(3,646 to 4,454)	13.4%<br>(-3.3 to 30.0)	78,142<br>(73,007 to 83,491)	-7.3%<br>(-9.2 to -5.1)	125,918<br>(112,904 to 139,772)	-0.6%<br>(-14.2 to 13.1)
**Central Europe**	12,712<br>(11,518 to 13,977)	-23.3%<br>(-25.7 to -21.3)	7,012<br>(6,434 to 7,558)	-37.6%<br>(-43.0 to -32.0)	113,645<br>(104,928 to 123,406)	-20.0%<br>(-21.1 to -18.8)	189,358<br>(174,636 to 203,796)	-43.9%<br>(-48.4 to -39.4)
**Central Latin America**	28,466<br>(25,351 to 32,268)	-6.6%<br>(-9.3 to -3.6)	11,946<br>(10,601 to 13,370)	8.9%<br>(-3.0 to 22.5)	384,034<br>(357,569 to 411,854)	-8.8%<br>(-10.3 to -7.2)	392,188<br>(350,309 to 440,360)	-2.7%<br>(-13.0 to 9.2)
**Central Sub-Saharan Africa**	6,071<br>(5,215 to 7,067)	-6.0%<br>(-10.1 to -2.2)	1,846<br>(766 to 5,027)	-12.3%<br>(-40.9 to 31.0)	70,727<br>(65,523 to 76,565)	-3.2%<br>(-6.0 to -0.2)	74,837<br>(37,073 to 182,350)	-15.0%<br>(-39.0 to 25.9)
**East Asia**	151,816<br>(131,565 to 176,502)	-55.5%<br>(-59.2 to -52.3)	95,180<br>(70,278 to 119,163)	-82.2%<br>(-88.3 to -67.2)	1,392,548<br>(1,241,283 to 1,558,662)	-35.2%<br>(-37.3 to -33.2)	2,396,955<br>(1,840,650 to 2,934,107)	-80.7%<br>(-86.7 to -67.3)
**Eastern Europe**	27,737<br>(24,528 to 31,835)	-2.4%<br>(-6.4 to 1.6)	18,199<br>(16,681 to 19,688)	-1.2%<br>(-9.4 to 8.4)	244,739<br>(216,483 to 276,836)	-3.5%<br>(-5.7 to -1.5)	494,657<br>(457,049 to 532,537)	-3.3%<br>(-10.6 to 5.0)
**Eastern Sub-Saharan Africa**	18,584<br>(15,962 to 21,445)	-19.3%<br>(-21.5 to -16.6)	5,580<br>(2,115 to 15,463)	-35.0%<br>(-49.7 to -20.4)	220,524<br>(202,101 to 239,702)	-15.0%<br>(-16.9 to -13.2)	234,778<br>(105,026 to 589,266)	-32.8%<br>(-45.9 to -19.2)
**High-income Asia Pacific**	46,618<br>(41,128 to 53,645)	-18.4%<br>(-22.7 to -13.3)	17,742<br>(14,894 to 19,535)	-56.4%<br>(-60.4 to -52.6)	747,779<br>(674,046 to 832,435)	1.1%<br>(-1.8 to 3.8)	485,219<br>(434,134 to 531,777)	-52.2%<br>(-56.0 to -48.7)
**High-income North America**	33,649<br>(29,722 to 38,722)	-10.9%<br>(-14.2 to -6.6)	19,705<br>(17,782 to 20,833)	-16.8%<br>(-21.1 to -13.4)	512,781<br>(466,659 to 564,388)	3.3%<br>(-1.1 to 7.7)	525,516<br>(495,261 to 554,720)	-26.1%<br>(-29.1 to -23.3)
**North Africa and Middle East**	32,386<br>(28,114 to 36,307)	-31.7%<br>(-34.6 to -28.4)	12,261<br>(9,703 to 15,966)	-54.4%<br>(-72.7 to -35.2)	422,056<br>(389,157 to 456,659)	-22.1%<br>(-23.5 to -20.7)	433,904<br>(351,654 to 547,035)	-57.9%<br>(-72.3 to -45.4)
**Oceania**	1,269<br>(1,123 to 1,416)	-15.8%<br>(-19.7 to -11.7)	701<br>(503 to 962)	-30.2%<br>(-45.9 to -9.7)	15,490<br>(14,651 to 16,406)	-9.4%<br>(-11.6 to -7.3)	31,040<br>(23,596 to 40,113)	-27.1%<br>(-42.6 to -6.8)
**South Asia**	140,534<br>(120,483 to 162,708)	-22.6%<br>(-25.9 to -19.2)	67,618<br>(48,401 to 93,444)	-33.9%<br>(-49.6 to -7.7)	1,355,698<br>(1,203,895 to 1,512,996)	-11.8%<br>(-14.2 to -9.7)	2,362,779<br>(1,772,137 to 3,126,993)	-33.8%<br>(-47.0 to -14.0)
**Southeast Asia**	74,812<br>(65,466 to 85,580)	-20.1%<br>(-22.4 to -17.4)	37,582<br>(31,535 to 51,345)	-35.2%<br>(-50.7 to -18.9)	804,235<br>(736,668 to 876,796)	-13.6%<br>(-15.1 to -11.9)	1,279,431<br>(1,094,266 to 1,636,991)	-34.5%<br>(-46.4 to -21.1)

**Southern Latin America**	8,418<br>(7,547 to 9,475)	-34.0%<br>(-37.7 to -30.4)	3,522<br>(3,255 to 3,746)	-58.2%<br>(-61.8 to -54.0)	98,117<br>(90,840 to 107,907)	-21.2%<br>(-24.6 to -14.9)	108,157<br>(101,029 to 114,847)	-59.7%<br>(-62.7 to -56.5)
**Southern Sub-Saharan Africa**	3,590<br>(3,113 to 4,140)	-9.5%<br>(-12.3 to -5.6)	1,220<br>(1,008 to 1,573)	12.7%<br>(-6.8 to 32.3)	48,804<br>(44,313 to 53,406)	-10.2%<br>(-12.2 to -8.2)	47,011<br>(39,292 to 58,930)	7.1%<br>(-6.6 to 23.1)
**Tropical Latin America**	26,001<br>(22,460 to 30,290)	-26.7%<br>(-30.4 to -22.6)	14,035<br>(13,232 to 14,631)	-27.0%<br>(-31.0 to -23.2)	278,851<br>(248,464 to 313,442)	-25.6%<br>(-27.8 to -23.6)	460,760<br>(440,239 to 479,158)	-37.4%<br>(-40.2 to -34.4)
**Western Europe**	43,945<br>(39,398 to 49,548)	-22.3%<br>(-24.3 to -20.0)	21,913<br>(19,165 to 23,396)	-40.1%<br>(-43.4 to -37.3)	608,490<br>(569,044 to 647,049)	-13.6%<br>(-14.9 to -12.2)	511,251<br>(473,458 to 544,421)	-46.3%<br>(-48.4 to -44.1)
**Western Sub-Saharan Africa**	17,263<br>(14,765 to 19,802)	-17.3%<br>(-19.0 to -15.3)	5,152<br>(2,595 to 11,593)	-30.2%<br>(-48.6 to -9.8)	241,301<br>(219,739 to 264,665)	-12.6%<br>(-14.3 to -11.0)	232,052<br>(137,194 to 464,758)	-27.6%<br>(-44.9 to -8.6)
**Countries in alphabetical order**								
**Afghanistan**	1,535<br>(1,346 to 1,740)	-14.2%<br>(-25.3 to 0.1)	726<br>(383 to 1,213)	-33.9%<br>(-61.1 to 8.5)	15,608<br>(14,270 to 17,128)	-17.5%<br>(-20.7 to -14.2)	30,687<br>(17,826 to 48,512)	-33.6%<br>(-58.4 to -1.9)
**Albania**	242<br>(214 to 271)	-7.0%<br>(-11.8 to -1.6)	101<br>(65 to 140)	-36.2%<br>(-59.5 to -0.3)	2,369<br>(2,190 to 2,571)	-9.6%<br>(-12.8 to -6.3)	2,576<br>(1,883 to 3,419)	-39.8%<br>(-58.5 to -14.1)
**Algeria**	2,541<br>(2,187 to 2,874)	-33.3%<br>(-36.9 to -28.8)	795<br>(512 to 1,219)	-43.8%<br>(-68.2 to -9.0)	32,670<br>(30,269 to 35,364)	-22.9%<br>(-25.4 to -20.0)	26,648<br>(18,742 to 38,756)	-48.3%<br>(-66.7 to -24.0)
**American Samoa**	5<br>(5 to 6)	-9.3%<br>(-14.8 to -3.5)	3<br>(2 to 3)	-13.4%<br>(-35.0 to 17.8)	78<br>(74 to 82)	-5.1%<br>(-8.3 to -1.9)	100<br>(80 to 125)	-10.4%<br>(-31.8 to 20.6)
**Andorra**	8<br>(7 to 9)	-12.8%<br>(-17.0 to -7.6)	3<br>(2 to 4)	-46.0%<br>(-65.7 to -14.9)	114<br>(107 to 122)	-9.1%<br>(-12.6 to -5.2)	81<br>(59 to 106)	-48.8%<br>(-65.9 to -24.6)
**Angola**	1,209<br>(1,027 to 1,392)	-18.5%<br>(-23.6 to -13.4)	360<br>(188 to 663)	-28.1%<br>(-51.2 to 9.4)	14,325<br>(13,146 to 15,604)	-10.8%<br>(-14.2 to -7.3)	15,348<br>(9,102 to 26,373)	-28.7%<br>(-48.6 to 2.5)
**Antigua and Barbuda**	11<br>(9 to 12)	-17.5%<br>(-23.0 to -11.2)	4<br>(4 to 4)	-39.8%<br>(-45.9 to -32.7)	148<br>(139 to 156)	-12.6%<br>(-15.8 to -9.4)	128<br>(119 to 138)	-43.3%<br>(-48.6 to -37.2)
**Argentina**	5,583<br>(4,972 to 6,304)	-38.4%<br>(-43.0 to -33.9)	2,219<br>(2,030 to 2,374)	-64.3%<br>(-67.9 to -60.1)	65,232<br>(60,048 to 72,359)	-24.7%<br>(-28.7 to -18.0)	70,009<br>(64,858 to 74,939)	-64.8%<br>(-67.9 to -61.3)
**Armenia**	226<br>(201 to 253)	-28.0%<br>(-33.0 to -23.0)	98<br>(87 to 110)	-47.5%<br>(-55.7 to -38.4)	2,678<br>(2,491 to 2,865)	-16.2%<br>(-19.5 to -13.1)	2,817<br>(2,509 to 3,188)	-43.8%<br>(-51.3 to -35.5)
**Australia**	2,226<br>(1,969 to 2,539)	-16.5%<br>(-22.8 to -8.4)	1,080<br>(947 to 1,179)	-44.7%<br>(-49.3 to -39.6)	30,089<br>(28,168 to 32,117)	-6.5%<br>(-10.0 to -2.6)	26,841<br>(24,678 to 28,932)	-47.7%<br>(-51.1 to -43.7)
**Austria**	1,240<br>(1,072 to 1,429)	-27.6%<br>(-33.0 to -18.6)	425<br>(371 to 468)	-37.0%<br>(-43.1 to -31.4)	23,045<br>(21,682 to 24,171)	-12.7%<br>(-16.6 to -8.3)	11,325<br>(10,132 to 12,466)	-42.9%<br>(-47.1 to -38.6)
**Azerbaijan**	622<br>(525 to 726)	-20.0%<br>(-29.4 to -9.1)	106<br>(70 to 152)	-31.8%<br>(-57.8 to 5.4)	7,885<br>(7,284 to 8,525)	-11.0%<br>(-14.4 to -7.4)	4,441<br>(3,319 to 5,866)	-34.4%<br>(-54.0 to -9.8)
**Bahamas**	37<br>(33 to 42)	-9.5%<br>(-14.8 to -3.8)	14<br>(11 to 17)	-22.5%<br>(-39.1 to -0.2)	522<br>(492 to 552)	-5.9%<br>(-9.3 to -2.5)	509<br>(417 to 625)	-23.1%<br>(-37.9 to -1.6)
**Bahrain**	62<br>(51 to 73)	-29.7%<br>(-34.2 to -25.3)	15<br>(11 to 19)	-59.4%<br>(-72.9 to -43.4)	955<br>(869 to 1,049)	-20.0%<br>(-22.8 to -17.1)	622<br>(492 to 777)	-59.6%<br>(-71.5 to -45.6)
**Bangladesh**	19,096<br>(16,867 to 21,924)	-10.7%<br>(-17.7 to -1.2)	10,874<br>(6,357 to 18,041)	-42.9%<br>(-61.7 to -8.5)	189,241<br>(176,671 to 203,827)	-9.5%<br>(-13.2 to -5.1)	368,375<br>(234,654 to 583,507)	-45.1%<br>(-62.5 to -17.0)

**Barbados**	41<br>(37 to 47)	-16.5%<br>(-21.8 to -10.3)	23<br>(18 to 28)	-21.6%<br>(-38.7 to -1.1)	604<br>(571 to 640)	-7.6%<br>(-10.7 to -4.0)	637<br>(505 to 786)	-25.0%<br>(-41.0 to -5.4)
**Belarus**	948<br>(849 to 1,063)	-12.3%<br>(-17.4 to -6.8)	594<br>(483 to 707)	-7.3%<br>(-33.2 to 26.4)	9,367<br>(8,724 to 10,141)	-5.2%<br>(-9.1 to -0.7)	17,141<br>(14,054 to 20,346)	-10.6%<br>(-33.9 to 17.8)
**Belgium**	1,027<br>(912 to 1,176)	-11.6%<br>(-16.7 to -6.2)	548<br>(476 to 596)	-16.7%<br>(-23.2 to -10.5)	12,417<br>(11,560 to 13,366)	-9.4%<br>(-12.6 to -5.9)	12,761<br>(11,780 to 13,743)	-29.1%<br>(-34.0 to -24.2)
**Belize**	31<br>(28 to 35)	-12.6%<br>(-17.7 to -7.3)	9<br>(8 to 10)	-29.2%<br>(-39.7 to -17.2)	420<br>(393 to 447)	-9.2%<br>(-12.5 to -5.8)	338<br>(298 to 381)	-33.4%<br>(-42.8 to -22.7)
**Benin**	490<br>(418 to 565)	-13.5%<br>(-17.8 to -8.2)	150<br>(70 to 349)	-25.8%<br>(-46.7 to 5.3)	6,584<br>(6,064 to 7,217)	-9.3%<br>(-12.3 to -6.5)	6,295<br>(3,450 to 13,227)	-23.8%<br>(-43.9 to 4.7)
**Bermuda**	8<br>(7 to 9)	-23.0%<br>(-27.2 to -18.1)	3<br>(2 to 4)	-48.1%<br>(-58.4 to -35.1)	142<br>(134 to 149)	-13.9%<br>(-16.5 to -11.2)	80<br>(69 to 94)	-48.2%<br>(-57.6 to -37.5)
**Bhutan**	53<br>(45 to 60)	-27.9%<br>(-33.1 to -22.0)	23<br>(15 to 35)	-43.0%<br>(-62.5 to -3.9)	550<br>(506 to 595)	-17.7%<br>(-21.3 to -13.5)	766<br>(522 to 1,149)	-43.8%<br>(-61.8 to -13.2)
**Bolivia (Plurinational State of)**	1,485<br>(1,316 to 1,664)	-27.3%<br>(-31.8 to -21.4)	909<br>(645 to 1,276)	-38.5%<br>(-58.4 to -13.5)	17,761<br>(16,759 to 18,776)	-23.6%<br>(-26.3 to -21.1)	31,155<br>(22,889 to 42,571)	-44.3%<br>(-61.4 to -21.6)
**Bosnia and Herzegovina**	387<br>(351 to 428)	-25.6%<br>(-31.7 to -19.8)	190<br>(138 to 246)	-57.4%<br>(-73.8 to -35.3)	3,537<br>(3,279 to 3,802)	-16.7%<br>(-20.3 to -13.0)	5,279<br>(3,929 to 6,757)	-57.8%<br>(-72.0 to -41.0)
**Botswana**	110<br>(93 to 128)	-12.2%<br>(-17.4 to -7.2)	20<br>(13 to 34)	-49.1%<br>(-63.6 to -22.2)	1,450<br>(1,327 to 1,574)	-9.1%<br>(-12.2 to -5.6)	849<br>(597 to 1,353)	-43.4%<br>(-59.0 to -17.4)
**Brazil**	25,290<br>(21,784 to 29,544)	-26.9%<br>(-30.7 to -22.7)	13,701<br>(12,895 to 14,272)	-27.0%<br>(-31.2 to -23.2)	270,875<br>(240,858 to 305,149)	-25.9%<br>(-28.2 to -24.0)	449,297<br>(429,647 to 466,339)	-37.5%<br>(-40.3 to -34.5)
**Brunei Darussalam**	67<br>(59 to 79)	-32.3%<br>(-40.0 to -18.0)	26<br>(21 to 32)	-48.7%<br>(-62.2 to -29.5)	698<br>(660 to 739)	-21.9%<br>(-26.1 to -17.8)	989<br>(830 to 1,187)	-47.9%<br>(-59.9 to -30.5)
**Bulgaria**	900<br>(823 to 985)	2.9%<br>(-2.4 to 9.0)	663<br>(573 to 761)	18.0%<br>(1.3 to 39.5)	7,404<br>(6,836 to 7,975)	-0.8%<br>(-4.2 to 2.6)	16,938<br>(14,548 to 19,524)	-0.6%<br>(-15.1 to 16.9)
**Burkina Faso**	797<br>(687 to 912)	-10.2%<br>(-14.9 to -4.4)	234<br>(89 to 586)	-14.9%<br>(-37.2 to 25.3)	10,882<br>(9,978 to 11,904)	-7.3%<br>(-10.5 to -4.0)	10,138<br>(4,992 to 21,931)	-14.3%<br>(-35.7 to 18.6)
**Burundi**	605<br>(519 to 700)	-16.8%<br>(-21.2 to -12.2)	205<br>(49 to 724)	-42.1%<br>(-68.9 to 3.5)	7,028<br>(6,462 to 7,598)	-13.2%<br>(-16.4 to -9.8)	8,501<br>(2,814 to 26,544)	-43.1%<br>(-65.4 to -1.0)
**CV¥te d'Ivoire**	1,008<br>(863 to 1,152)	-12.9%<br>(-17.7 to -7.6)	344<br>(167 to 737)	-17.8%<br>(-43.7 to 23.1)	14,078<br>(12,887 to 15,366)	-9.7%<br>(-12.6 to -6.4)	15,993<br>(9,038 to 30,973)	-16.8%<br>(-41.4 to 19.3)
**Cabo Verde**	27<br>(23 to 32)	-13.0%<br>(-17.6 to -8.4)	8<br>(4 to 19)	-23.9%<br>(-49.3 to 27.4)	468<br>(435 to 503)	-7.4%<br>(-10.1 to -3.8)	322<br>(183 to 709)	-28.0%<br>(-47.7 to 0.9)
**Cambodia**	1,495<br>(1,316 to 1,692)	-17.5%<br>(-22.7 to -11.4)	671<br>(475 to 1,044)	-40.0%<br>(-60.6 to -12.3)	15,314<br>(14,188 to 16,432)	-10.6%<br>(-13.9 to -7.1)	22,819<br>(16,682 to 32,966)	-42.2%<br>(-59.4 to -20.3)
**Cameroon**	1,152<br>(984 to 1,335)	-7.4%<br>(-12.1 to -2.8)	425<br>(191 to 909)	-13.8%<br>(-41.7 to 27.2)	16,176<br>(14,688 to 17,659)	-6.0%<br>(-10.0 to -2.7)	19,001<br>(9,625 to 37,450)	-10.6%<br>(-37.7 to 26.6)
**Canada**	3,883<br>(3,430 to 4,442)	-24.6%<br>(-28.9 to -19.3)	1,655<br>(1,460 to 1,800)	-40.5%<br>(-46.2 to -34.5)	58,392<br>(54,702 to 62,100)	-16.9%<br>(-20.0 to -13.6)	44,085<br>(40,485 to 47,579)	-43.9%<br>(-48.6 to -39.0)
**Central African Republic**	252<br>(215 to 297)	-3.3%<br>(-8.8 to 2.4)	111<br>(41 to 251)	-11.3%<br>(-40.7 to 29.6)	2,767<br>(2,519 to 3,016)	-1.9%<br>(-5.6 to 1.9)	4,611<br>(1,987 to 9,667)	-12.4%<br>(-40.2 to 27.3)

**Chad**	642<br>(548 to 735)	-6.4%<br>(-11.1 to -0.7)	250<br>(105 to 583)	1.9%<br>(-22.4 to 46.0)	8,199<br>(7,477 to 8,933)	-5.3%<br>(-8.9 to -1.9)	11,577<br>(5,843 to 24,325)	1.9%<br>(-21.7 to 38.3)
**Chile**	2,225<br>(1,961 to 2,515)	-16.4%<br>(-22.0 to -10.9)	949<br>(874 to 1,023)	-25.7%<br>(-32.5 to -19.1)	26,323<br>(24,366 to 28,746)	-3.5%<br>(-7.8 to 4.0)	28,539<br>(26,750 to 30,665)	-34.0%<br>(-38.9 to -28.8)
**China**	145,138<br>(125,425 to 169,016)	-56.5%<br>(-60.2 to -53.3)	91,802<br>(66,672 to 116,215)	-82.7%<br>(-88.7 to -67.9)	1,323,287<br>(1,176,081 to 1,484,052)	-36.2%<br>(-38.3 to -34.2)	2,296,534<br>(1,727,442 to 2,847,370)	-81.3%<br>(-87.2 to -68.4)
**Colombia**	7,570<br>(6,798 to 8,535)	-8.9%<br>(-15.9 to -1.7)	3,353<br>(2,803 to 3,961)	-10.2%<br>(-25.3 to 6.0)	97,518<br>(92,132 to 102,356)	-12.2%<br>(-15.3 to -8.9)	100,437<br>(84,643 to 118,486)	-20.2%<br>(-33.3 to -6.5)
**Comoros**	45<br>(38 to 52)	-17.4%<br>(-21.7 to -13.1)	13<br>(5 to 31)	-35.8%<br>(-57.3 to -3.3)	586<br>(547 to 629)	-12.9%<br>(-15.9 to -9.8)	522<br>(254 to 1,122)	-35.0%<br>(-54.4 to -5.2)
**Congo**	244<br>(207 to 283)	-19.2%<br>(-24.2 to -13.7)	80<br>(42 to 152)	-30.0%<br>(-51.8 to 4.4)	3,003<br>(2,760 to 3,253)	-11.9%<br>(-15.3 to -7.7)	3,249<br>(1,906 to 5,801)	-29.6%<br>(-49.5 to 2.7)
**Cook Islands**	2<br>(2 to 2)	-29.6%<br>(-33.6 to -25.6)	1<br>(0 to 1)	-65.1%<br>(-79.3 to -42.2)	34<br>(32 to 35)	-16.0%<br>(-18.4 to -13.2)	24<br>(18 to 31)	-59.9%<br>(-73.5 to -38.9)
**Costa Rica**	541<br>(470 to 616)	-16.6%<br>(-22.4 to -11.2)	200<br>(173 to 221)	-3.3%<br>(-15.5 to 10.9)	8,384<br>(7,927 to 8,837)	-10.3%<br>(-13.2 to -7.4)	6,377<br>(5,673 to 6,991)	-6.8%<br>(-18.0 to 4.7)
**Croatia**	392<br>(355 to 431)	-31.5%<br>(-36.9 to -26.9)	299<br>(261 to 343)	-47.4%<br>(-55.2 to -39.2)	3,767<br>(3,462 to 4,075)	-19.4%<br>(-22.7 to -15.7)	6,910<br>(6,054 to 7,851)	-53.3%<br>(-59.7 to -46.1)
**Cuba**	1,278<br>(1,126 to 1,484)	-22.3%<br>(-27.1 to -17.5)	522<br>(447 to 594)	-35.8%<br>(-45.8 to -25.1)	21,079<br>(20,088 to 22,129)	-15.4%<br>(-18.4 to -12.5)	15,521<br>(13,537 to 17,519)	-40.1%<br>(-47.9 to -30.9)
**Cyprus**	123<br>(108 to 142)	-33.5%<br>(-38.3 to -28.3)	48<br>(38 to 60)	-69.9%<br>(-79.1 to -58.1)	1,634<br>(1,522 to 1,736)	-23.8%<br>(-26.5 to -21.1)	1,258<br>(1,045 to 1,520)	-67.1%<br>(-76.6 to -56.7)
**Czechia**	957<br>(857 to 1,066)	-31.8%<br>(-35.5 to -28.2)	468<br>(410 to 535)	-59.6%<br>(-65.0 to -52.6)	10,906<br>(10,107 to 11,698)	-20.0%<br>(-23.0 to -17.2)	12,537<br>(11,056 to 14,366)	-60.6%<br>(-65.4 to -54.6)
**Democratic People's Republic of Korea**	3,649<br>(3,192 to 4,137)	-21.0%<br>(-25.0 to -16.2)	2,432<br>(1,457 to 4,474)	-36.8%<br>(-60.4 to 9.8)	32,425<br>(30,231 to 34,869)	-12.8%<br>(-15.9 to -9.6)	70,875<br>(44,787 to 120,723)	-32.5%<br>(-57.5 to 15.4)
**Democratic Republic of the Congo**	4,234<br>(3,630 to 4,964)	-0.6%<br>(-5.7 to 5.1)	1,264<br>(432 to 4,111)	-4.3%<br>(-42.6 to 50.3)	48,959<br>(45,236 to 52,954)	0.3%<br>(-3.2 to 4.1)	50,353<br>(21,958 to 144,646)	-8.1%<br>(-39.2 to 42.0)
**Denmark**	655<br>(577 to 748)	-30.9%<br>(-36.1 to -25.4)	326<br>(291 to 354)	-47.9%<br>(-53.1 to -43.0)	9,041<br>(8,487 to 9,585)	-20.9%<br>(-23.9 to -17.8)	7,589<br>(6,948 to 8,133)	-56.2%<br>(-60.0 to -52.2)
**Djibouti**	72<br>(61 to 85)	-11.8%<br>(-16.3 to -6.9)	20<br>(8 to 45)	-26.7%<br>(-50.9 to 23.2)	897<br>(836 to 967)	-8.0%<br>(-11.2 to -4.5)	856<br>(391 to 1,774)	-24.9%<br>(-47.0 to 21.3)
**Dominica**	7<br>(6 to 8)	-16.3%<br>(-21.5 to -11.1)	4<br>(3 to 4)	-26.8%<br>(-41.4 to -7.3)	99<br>(93 to 105)	-13.0%<br>(-15.9 to -10.1)	110<br>(91 to 135)	-23.7%<br>(-38.6 to -3.4)
**Dominican Republic**	1,175<br>(1,037 to 1,337)	9.9%<br>(2.1 to 18.0)	419<br>(310 to 548)	-24.7%<br>(-50.0 to 7.3)	15,004<br>(14,096 to 15,895)	3.5%<br>(-0.3 to 7.3)	15,792<br>(12,273 to 19,934)	-25.9%<br>(-48.0 to -0.4)
**Ecuador**	2,585<br>(2,336 to 2,906)	-10.9%<br>(-16.7 to -4.7)	1,257<br>(1,001 to 1,554)	31.9%<br>(4.3 to 69.0)	34,297<br>(32,564 to 35,907)	-10.6%<br>(-13.6 to -7.5)	40,190<br>(32,586 to 49,511)	4.3%<br>(-15.9 to 31.3)
**Egypt**	4,578<br>(3,930 to 5,241)	-26.7%<br>(-31.3 to -21.9)	1,674<br>(1,142 to 2,457)	-60.6%<br>(-77.5 to -30.1)	58,524<br>(53,457 to 64,094)	-18.5%<br>(-21.7 to -15.4)	64,821<br>(47,313 to 93,504)	-64.8%<br>(-77.7 to -46.6)
**El Salvador**	616<br>(548 to 687)	-30.8%<br>(-35.6 to -25.9)	212<br>(163 to 272)	-40.2%<br>(-61.8 to -17.6)	8,410<br>(7,931 to 8,929)	-23.3%<br>(-25.7 to -20.4)	7,379<br>(5,845 to 9,176)	-43.7%<br>(-61.9 to -25.8)

**Equatorial Guinea**	49<br>(41 to 56)	-33.9%<br>(-37.7 to -29.3)	9<br>(5 to 16)	-64.3%<br>(-82.4 to -19.9)	597<br>(541 to 657)	-20.2%<br>(-23.1 to -16.7)	440<br>(239 to 694)	-61.1%<br>(-78.8 to -25.4)
**Eritrea**	358<br>(304 to 419)	-12.5%<br>(-17.9 to -5.7)	123<br>(44 to 283)	-35.4%<br>(-52.3 to -6.1)	4,228<br>(3,919 to 4,550)	-6.4%<br>(-10.2 to -2.7)	5,250<br>(2,186 to 11,402)	-35.2%<br>(-51.5 to -7.3)
**Estonia**	121<br>(108 to 136)	-33.8%<br>(-38.0 to -29.6)	66<br>(57 to 74)	-36.8%<br>(-47.2 to -27.1)	1,216<br>(1,120 to 1,322)	-28.7%<br>(-31.3 to -25.8)	1,547<br>(1,349 to 1,740)	-47.9%<br>(-55.5 to -39.6)
**Eswatini**	42<br>(36 to 48)	-4.9%<br>(-10.8 to 1.7)	14<br>(9 to 22)	-12.3%<br>(-42.7 to 31.2)	541<br>(494 to 594)	-5.5%<br>(-8.8 to -1.8)	555<br>(350 to 895)	-6.2%<br>(-38.1 to 36.1)
**Ethiopia**	4,282<br>(3,638 to 4,945)	-37.4%<br>(-39.9 to -34.6)	1,025<br>(344 to 3,100)	-54.4%<br>(-69.1 to -34.1)	51,392<br>(45,753 to 57,503)	-31.4%<br>(-33.8 to -29.1)	42,931<br>(18,319 to 113,072)	-53.5%<br>(-66.5 to -37.5)
**Fiji**	132<br>(119 to 146)	-17.9%<br>(-22.9 to -11.5)	53<br>(40 to 71)	-22.2%<br>(-41.9 to 6.6)	1,623<br>(1,534 to 1,709)	-14.4%<br>(-17.5 to -11.3)	2,154<br>(1,688 to 2,734)	-24.6%<br>(-42.8 to 0.1)
**Finland**	875<br>(772 to 993)	-24.1%<br>(-31.9 to -16.9)	400<br>(349 to 436)	-49.0%<br>(-54.3 to -44.0)	15,605<br>(14,829 to 16,369)	-17.7%<br>(-20.7 to -14.9)	10,568<br>(9,752 to 11,445)	-52.2%<br>(-56.3 to -48.3)
**France**	6,761<br>(6,085 to 7,535)	-0.3%<br>(-5.9 to 5.2)	3,013<br>(2,632 to 3,283)	-27.3%<br>(-33.0 to -22.0)	90,775<br>(85,342 to 96,584)	0.9%<br>(-2.7 to 4.7)	72,173<br>(65,760 to 78,076)	-25.2%<br>(-30.3 to -20.1)
**Gabon**	83<br>(70 to 96)	-17.7%<br>(-22.8 to -12.4)	21<br>(12 to 37)	-27.1%<br>(-53.1 to 9.3)	1,077<br>(990 to 1,161)	-10.3%<br>(-13.7 to -6.5)	837<br>(525 to 1,382)	-25.4%<br>(-48.6 to 8.3)
**Gambia**	86<br>(73 to 99)	-8.5%<br>(-13.1 to -3.3)	35<br>(15 to 87)	0.8%<br>(-35.3 to 42.2)	1,229<br>(1,132 to 1,348)	-7.1%<br>(-10.3 to -3.6)	1,520<br>(756 to 3,557)	-1.0%<br>(-33.4 to 36.8)
**Georgia**	831<br>(761 to 912)	17.8%<br>(10.0 to 26.7)	523<br>(432 to 617)	49.1%<br>(11.7 to 105.6)	6,621<br>(6,177 to 7,050)	7.2%<br>(2.9 to 11.4)	12,934<br>(10,722 to 15,161)	32.0%<br>(0.8 to 73.4)
**Germany**	8,639<br>(7,600 to 10,001)	-26.8%<br>(-31.1 to -21.8)	4,091<br>(3,540 to 4,460)	-45.4%<br>(-51.5 to -38.3)	134,983<br>(126,165 to 142,396)	-16.0%<br>(-18.9 to -12.9)	103,378<br>(94,795 to 111,589)	-49.9%<br>(-54.4 to -44.6)
**Ghana**	1,570<br>(1,334 to 1,819)	-7.5%<br>(-12.4 to -1.6)	625<br>(323 to 1,243)	-19.0%<br>(-43.4 to 31.3)	23,453<br>(21,811 to 25,292)	-6.9%<br>(-10.0 to -3.8)	26,771<br>(14,854 to 50,609)	-21.1%<br>(-42.5 to 18.2)
**Greece**	1,234<br>(1,093 to 1,413)	-3.3%<br>(-9.4 to 3.2)	702<br>(618 to 760)	-17.7%<br>(-28.1 to 3.2)	18,452<br>(17,285 to 19,551)	6.5%<br>(2.3 to 10.6)	16,241<br>(15,016 to 17,523)	-15.2%<br>(-24.3 to 0.7)
**Greenland**	7<br>(7 to 8)	3.2%<br>(-38.7 to 25.8)	5<br>(4 to 6)	-61.0%<br>(-68.9 to -50.7)	79<br>(73 to 84)	-15.5%<br>(-36.5 to -6.0)	161<br>(125 to 195)	-62.3%<br>(-69.2 to -53.3)
**Grenada**	11<br>(10 to 13)	-20.4%<br>(-25.5 to -15.0)	5<br>(5 to 6)	-37.2%<br>(-49.4 to -18.6)	155<br>(147 to 163)	-13.2%<br>(-16.3 to -10.3)	172<br>(147 to 195)	-41.1%<br>(-52.7 to -24.6)
**Guam**	19<br>(16 to 21)	-18.0%<br>(-22.4 to -12.2)	6<br>(5 to 7)	-53.4%<br>(-61.8 to -39.4)	291<br>(276 to 306)	-3.2%<br>(-6.5 to 0.4)	251<br>(216 to 293)	-31.2%<br>(-40.8 to -19.0)
**Guatemala**	1,198<br>(1,066 to 1,345)	-0.4%<br>(-6.7 to 6.0)	301<br>(257 to 348)	26.4%<br>(2.4 to 52.4)	16,258<br>(15,268 to 17,232)	-2.1%<br>(-5.3 to 1.5)	12,478<br>(10,725 to 14,234)	18.8%<br>(-0.9 to 40.7)
**Guinea**	555<br>(480 to 633)	-5.0%<br>(-10.1 to 0.7)	202<br>(92 to 441)	-6.7%<br>(-34.0 to 37.4)	7,528<br>(6,959 to 8,151)	-3.9%<br>(-7.3 to 0.0)	9,023<br>(4,898 to 17,589)	-7.0%<br>(-31.5 to 31.7)
**Guinea-Bissau**	76<br>(65 to 88)	-12.4%<br>(-17.1 to -7.3)	41<br>(17 to 90)	-17.1%<br>(-43.7 to 14.3)	1,025<br>(938 to 1,124)	-10.4%<br>(-13.5 to -6.9)	1,822<br>(850 to 3,822)	-18.7%<br>(-44.5 to 12.8)
**Guyana**	81<br>(73 to 90)	-20.6%<br>(-25.3 to -15.3)	37<br>(29 to 48)	-26.7%<br>(-45.8 to -4.3)	933<br>(875 to 994)	-12.7%<br>(-15.8 to -9.4)	1,402<br>(1,081 to 1,787)	-26.3%<br>(-44.5 to -4.5)

**Haiti**	1,464<br>(1,291 to 1,660)	-3.9%<br>(-14.2 to 8.7)	1,078<br>(568 to 1,682)	-23.7%<br>(-48.5 to 12.0)	14,615<br>(13,672 to 15,557)	-5.1%<br>(-8.5 to -1.1)	45,554<br>(24,479 to 66,864)	-27.4%<br>(-49.6 to 4.1)
**Honduras**	920<br>(819 to 1,036)	1.9%<br>(-4.8 to 8.9)	775<br>(542 to 1,013)	30.8%<br>(-11.4 to 93.3)	10,805<br>(10,100 to 11,517)	-12.2%<br>(-15.1 to -8.8)	25,507<br>(17,324 to 34,150)	3.0%<br>(-28.5 to 46.4)
**Hungary**	848<br>(767 to 942)	-23.3%<br>(-27.4 to -19.0)	381<br>(324 to 437)	-46.5%<br>(-54.9 to -38.0)	9,024<br>(8,324 to 9,730)	-18.4%<br>(-21.5 to -14.8)	11,389<br>(9,775 to 13,017)	-51.4%<br>(-58.1 to -44.2)
**Iceland**	34<br>(30 to 40)	-26.0%<br>(-30.2 to -21.3)	12<br>(10 to 14)	-48.0%<br>(-53.8 to -41.3)	507<br>(475 to 536)	-17.8%<br>(-20.6 to -15.0)	316<br>(284 to 350)	-52.6%<br>(-57.1 to -47.4)
**India**	101,503<br>(86,895 to 118,266)	-25.7%<br>(-28.9 to -22.2)	48,285<br>(33,672 to 66,531)	-33.9%<br>(-49.8 to -6.0)	975,215<br>(855,888 to 1,105,598)	-13.3%<br>(-15.8 to -11.0)	1,657,354<br>(1,212,105 to 2,198,270)	-34.3%<br>(-48.5 to -13.1)
**Indonesia**	29,375<br>(25,056 to 34,150)	-19.1%<br>(-22.4 to -15.6)	14,949<br>(10,997 to 22,996)	-24.2%<br>(-43.5 to 0.6)	313,471<br>(277,712 to 352,552)	-18.3%<br>(-20.7 to -16.2)	542,472<br>(421,993 to 753,771)	-28.7%<br>(-43.5 to -8.5)
**Iran (Islamic Republic of)**	3,972<br>(3,395 to 4,545)	-30.9%<br>(-33.1 to -28.7)	1,006<br>(787 to 1,213)	-61.9%<br>(-78.8 to -47.5)	54,855<br>(49,044 to 61,209)	-17.0%<br>(-18.8 to -15.2)	33,899<br>(28,616 to 40,649)	-60.8%<br>(-75.2 to -49.1)
**Iraq**	1,676<br>(1,428 to 1,906)	-40.9%<br>(-44.6 to -37.0)	581<br>(418 to 787)	-45.4%<br>(-70.8 to -18.8)	22,568<br>(20,667 to 24,841)	-27.2%<br>(-30.3 to -24.2)	23,352<br>(17,843 to 30,731)	-50.5%<br>(-68.9 to -30.0)
**Ireland**	500<br>(443 to 576)	-39.1%<br>(-43.7 to -33.8)	213<br>(185 to 234)	-56.9%<br>(-61.2 to -52.6)	6,889<br>(6,440 to 7,304)	-30.7%<br>(-33.5 to -27.3)	5,338<br>(4,881 to 5,806)	-61.4%<br>(-64.4 to -58.5)
**Israel**	606<br>(523 to 698)	-17.5%<br>(-21.7 to -13.1)	168<br>(148 to 185)	-29.1%<br>(-36.4 to -21.1)	8,758<br>(8,167 to 9,346)	-12.2%<br>(-15.6 to -8.9)	4,647<br>(4,226 to 5,067)	-41.1%<br>(-45.9 to -35.8)
**Italy**	5,151<br>(4,586 to 5,770)	-28.0%<br>(-33.3 to -21.3)	2,965<br>(2,531 to 3,219)	-31.6%<br>(-36.5 to -27.8)	64,232<br>(56,973 to 71,457)	-17.5%<br>(-19.6 to -15.2)	61,278<br>(55,771 to 66,042)	-44.4%<br>(-47.2 to -41.6)
**Jamaica**	351<br>(315 to 394)	-11.4%<br>(-18.7 to -3.7)	149<br>(116 to 190)	-24.7%<br>(-44.5 to -1.1)	4,839<br>(4,573 to 5,110)	-9.7%<br>(-12.8 to -6.1)	4,600<br>(3,582 to 5,839)	-33.5%<br>(-50.1 to -14.0)
**Japan**	37,011<br>(32,480 to 42,504)	-5.6%<br>(-10.9 to 1.2)	14,560<br>(12,000 to 16,027)	-47.1%<br>(-50.1 to -44.5)	624,487<br>(552,969 to 709,169)	12.1%<br>(8.8 to 15.6)	388,913<br>(344,185 to 426,023)	-40.8%<br>(-43.6 to -38.2)
**Jordan**	491<br>(408 to 575)	-27.2%<br>(-33.0 to -22.3)	38<br>(28 to 49)	-51.0%<br>(-70.9 to -21.3)	7,517<br>(6,916 to 8,195)	-15.7%<br>(-18.9 to -12.8)	2,460<br>(1,963 to 2,999)	-47.8%<br>(-63.7 to -28.7)
**Kazakhstan**	1,853<br>(1,646 to 2,077)	2.6%<br>(-3.1 to 8.5)	1,057<br>(909 to 1,218)	6.8%<br>(-20.6 to 38.5)	17,357<br>(16,125 to 18,620)	-2.9%<br>(-6.5 to 0.8)	31,555<br>(26,966 to 36,229)	-7.6%<br>(-29.4 to 17.2)
**Kenya**	2,162<br>(1,844 to 2,499)	-12.2%<br>(-14.5 to -9.5)	502<br>(223 to 1,215)	-7.9%<br>(-30.4 to 24.9)	27,042<br>(24,018 to 30,047)	-7.7%<br>(-9.7 to -5.7)	20,913<br>(10,865 to 44,370)	-6.3%<br>(-24.9 to 17.7)
**Kiribati**	21<br>(19 to 24)	9.9%<br>(-6.4 to 20.9)	7<br>(5 to 11)	-6.8%<br>(-30.9 to 29.2)	235<br>(223 to 248)	8.9%<br>(4.0 to 13.9)	355<br>(257 to 491)	-8.9%<br>(-31.2 to 20.5)
**Kuwait**	250<br>(200 to 302)	-13.7%<br>(-17.6 to -9.9)	16<br>(13 to 19)	-72.4%<br>(-77.4 to -66.9)	3,886<br>(3,608 to 4,217)	-9.8%<br>(-12.7 to -6.9)	1,126<br>(936 to 1,331)	-64.2%<br>(-69.4 to -58.7)
**Kyrgyzstan**	564<br>(503 to 629)	-14.8%<br>(-20.1 to -7.9)	301<br>(251 to 358)	-10.0%<br>(-30.0 to 11.8)	5,536<br>(5,113 to 5,917)	-13.8%<br>(-17.4 to -10.0)	10,558<br>(8,726 to 12,537)	-15.4%<br>(-34.9 to 7.5)
**Lao People's Democratic Republic**	571<br>(498 to 650)	-25.7%<br>(-29.6 to -20.7)	275<br>(195 to 405)	-55.9%<br>(-70.5 to -32.3)	5,994<br>(5,590 to 6,442)	-15.2%<br>(-18.8 to -11.8)	10,871<br>(8,038 to 15,242)	-55.5%<br>(-69.5 to -32.7)
**Latvia**	198<br>(176 to 222)	-30.7%<br>(-34.9 to -26.9)	122<br>(108 to 138)	-30.1%<br>(-40.3 to -19.1)	1,915<br>(1,768 to 2,085)	-20.7%<br>(-24.0 to -17.2)	2,962<br>(2,615 to 3,352)	-37.3%<br>(-46.4 to -27.4)

**Lebanon**	327<br>(280 to 369)	-34.6%<br>(-38.8 to -30.7)	131<br>(103 to 164)	-74.6%<br>(-84.9 to -60.6)	4,613<br>(4,265 to 4,969)	-20.6%<br>(-23.3 to -17.9)	3,676<br>(3,056 to 4,408)	-73.0%<br>(-82.4 to -60.2)
**Lesotho**	82<br>(71 to 94)	12.9%<br>(4.2 to 21.4)	38<br>(22 to 64)	32.0%<br>(-16.1 to 112.7)	1,006<br>(916 to 1,102)	3.6%<br>(-0.4 to 8.1)	1,365<br>(827 to 2,258)	39.7%<br>(-9.7 to 110.2)
**Liberia**	207<br>(175 to 243)	-12.8%<br>(-16.8 to -8.0)	78<br>(26 to 236)	-7.4%<br>(-50.9 to 59.5)	2,893<br>(2,639 to 3,172)	-10.7%<br>(-13.9 to -7.5)	3,515<br>(1,452 to 9,517)	-9.7%<br>(-48.1 to 53.2)
**Libya**	333<br>(281 to 386)	-24.6%<br>(-30.2 to -19.8)	114<br>(66 to 178)	-17.3%<br>(-56.4 to 24.0)	4,797<br>(4,407 to 5,189)	-15.7%<br>(-18.4 to -12.6)	4,864<br>(2,939 to 7,360)	-22.6%<br>(-54.9 to 9.1)
**Lithuania**	268<br>(239 to 303)	-21.0%<br>(-25.7 to -16.6)	170<br>(148 to 190)	-9.0%<br>(-24.0 to 6.6)	2,749<br>(2,519 to 3,003)	-5.2%<br>(-8.8 to -0.5)	4,201<br>(3,690 to 4,696)	-22.6%<br>(-34.6 to -8.7)
**Luxembourg**	47<br>(41 to 55)	-25.4%<br>(-29.9 to -21.0)	18<br>(16 to 19)	-54.2%<br>(-59.5 to -48.5)	615<br>(569 to 661)	-16.3%<br>(-19.6 to -13.1)	443<br>(403 to 492)	-59.4%<br>(-63.7 to -55.0)
**Madagascar**	1,770<br>(1,504 to 2,078)	-5.0%<br>(-10.1 to 0.8)	736<br>(253 to 2,031)	-20.4%<br>(-45.7 to 9.3)	20,986<br>(19,571 to 22,484)	-3.7%<br>(-7.2 to -0.2)	32,457<br>(13,117 to 81,903)	-18.5%<br>(-41.1 to 11.4)
**Malawi**	784<br>(667 to 912)	-9.4%<br>(-14.4 to -4.4)	298<br>(100 to 854)	-8.9%<br>(-35.9 to 16.0)	9,193<br>(8,420 to 10,041)	-6.7%<br>(-9.7 to -2.9)	12,329<br>(4,839 to 32,934)	-7.1%<br>(-31.5 to 19.9)
**Malaysia**	2,751<br>(2,408 to 3,101)	-33.0%<br>(-37.5 to -29.0)	932<br>(717 to 1,141)	-41.4%<br>(-62.7 to -19.7)	35,379<br>(33,144 to 37,644)	-17.1%<br>(-20.4 to -13.6)	32,529<br>(25,810 to 38,492)	-41.0%<br>(-57.9 to -23.8)
**Maldives**	40<br>(33 to 46)	-41.4%<br>(-45.3 to -37.4)	9<br>(7 to 11)	-69.1%<br>(-79.9 to -55.5)	493<br>(457 to 529)	-25.9%<br>(-28.7 to -23.0)	354<br>(280 to 456)	-69.4%<br>(-77.8 to -59.1)
**Mali**	826<br>(708 to 943)	-14.3%<br>(-18.7 to -9.6)	266<br>(126 to 574)	-25.1%<br>(-45.2 to 8.4)	11,098<br>(10,189 to 12,093)	-10.3%<br>(-13.0 to -7.2)	12,359<br>(7,098 to 23,856)	-23.5%<br>(-42.0 to 4.1)
**Malta**	40<br>(35 to 47)	-21.3%<br>(-25.2 to -16.9)	10<br>(9 to 12)	-42.9%<br>(-49.0 to -35.2)	655<br>(613 to 695)	-14.3%<br>(-17.5 to -10.8)	321<br>(285 to 362)	-39.6%<br>(-45.4 to -32.7)
**Marshall Islands**	9<br>(8 to 10)	-0.7%<br>(-8.3 to 8.9)	5<br>(3 to 7)	-25.5%<br>(-43.9 to -2.8)	101<br>(95 to 106)	0.7%<br>(-3.1 to 4.6)	208<br>(139 to 301)	-18.7%<br>(-37.7 to 6.4)
**Mauritania**	165<br>(140 to 193)	-19.2%<br>(-22.8 to -14.7)	53<br>(23 to 122)	-34.5%<br>(-58.5 to -1.4)	2,469<br>(2,271 to 2,689)	-13.5%<br>(-16.6 to -10.6)	2,171<br>(1,139 to 4,635)	-34.3%<br>(-55.4 to -5.8)
**Mauritius**	172<br>(152 to 195)	-15.5%<br>(-21.3 to -9.9)	97<br>(90 to 104)	-30.2%<br>(-36.4 to -24.6)	2,241<br>(2,091 to 2,390)	-7.7%<br>(-11.6 to -3.6)	3,139<br>(2,870 to 3,347)	-28.1%<br>(-34.2 to -21.8)
**Mexico**	12,883<br>(11,194 to 14,847)	-0.8%<br>(-4.3 to 3.4)	5,114<br>(4,541 to 5,694)	30.6%<br>(16.6 to 45.1)	176,786<br>(158,617 to 196,213)	-6.1%<br>(-8.2 to -3.8)	172,718<br>(154,807 to 192,396)	16.0%<br>(3.9 to 27.8)
**Micronesia (Federated States of)**	17<br>(15 to 19)	-4.6%<br>(-11.2 to 4.5)	9<br>(6 to 13)	-31.7%<br>(-50.7 to -5.2)	188<br>(177 to 199)	-3.4%<br>(-6.8 to 0.1)	367<br>(248 to 505)	-29.7%<br>(-48.5 to -1.5)
**Monaco**	4<br>(3 to 4)	-29.1%<br>(-33.0 to -24.6)	2<br>(1 to 2)	-59.7%<br>(-73.3 to -37.6)	54<br>(51 to 58)	-15.7%<br>(-18.6 to -12.9)	43<br>(32 to 55)	-55.7%<br>(-69.6 to -34.7)
**Mongolia**	330<br>(290 to 374)	12.1%<br>(3.9 to 21.6)	263<br>(192 to 341)	-2.4%<br>(-40.7 to 62.3)	2,831<br>(2,625 to 3,059)	-3.5%<br>(-7.4 to 0.9)	8,235<br>(6,171 to 10,980)	-3.7%<br>(-41.6 to 57.5)
**Montenegro**	40<br>(35 to 45)	-9.3%<br>(-14.2 to -4.7)	19<br>(14 to 26)	-8.4%<br>(-38.7 to 23.3)	468<br>(430 to 507)	-7.6%<br>(-11.0 to -4.0)	531<br>(407 to 684)	-22.8%<br>(-43.0 to -2.7)
**Morocco**	2,445<br>(2,129 to 2,778)	-23.7%<br>(-29.3 to -18.3)	1,111<br>(651 to 1,762)	-34.5%<br>(-59.5 to 15.6)	29,061<br>(26,717 to 31,461)	-19.3%<br>(-22.3 to -15.9)	35,214<br>(21,441 to 53,544)	-43.0%<br>(-60.8 to -12.8)

**Mozambique**	1,446<br>(1,243 to 1,674)	0.0%<br>(-7.4 to 7.9)	694<br>(223 to 1,992)	6.2%<br>(-27.4 to 55.9)	15,886<br>(14,602 to 17,285)	1.3%<br>(-2.8 to 5.7)	27,380<br>(10,081 to 74,493)	11.9%<br>(-21.8 to 54.6)
**Myanmar**	4,976<br>(4,419 to 5,618)	-31.0%<br>(-35.2 to -25.9)	2,854<br>(2,018 to 4,436)	-51.1%<br>(-67.8 to -26.4)	53,564<br>(49,834 to 57,330)	-19.7%<br>(-22.8 to -16.3)	103,280<br>(77,326 to 142,871)	-51.6%<br>(-66.7 to -31.3)
**Namibia**	101<br>(87 to 117)	-15.6%<br>(-19.9 to -10.8)	27<br>(17 to 44)	-20.3%<br>(-45.0 to 11.9)	1,342<br>(1,234 to 1,460)	-12.3%<br>(-15.5 to -9.1)	1,037<br>(709 to 1,628)	-19.3%<br>(-40.0 to 8.8)
**Nauru**	1<br>(1 to 2)	-6.0%<br>(-12.8 to 2.4)	1<br>(1 to 1)	-10.8%<br>(-42.1 to 34.3)	17<br>(16 to 18)	1.1%<br>(-2.8 to 5.0)	42<br>(31 to 56)	-9.4%<br>(-37.8 to 32.2)
**Nepal**	2,284<br>(1,969 to 2,636)	-18.2%<br>(-23.2 to -13.0)	1,068<br>(630 to 1,675)	-38.7%<br>(-60.6 to -6.9)	22,404<br>(20,694 to 24,275)	-12.6%<br>(-15.8 to -8.5)	36,134<br>(22,914 to 54,336)	-40.0%<br>(-58.8 to -12.4)
**Netherlands**	1,694<br>(1,496 to 1,949)	-29.8%<br>(-34.2 to -25.1)	792<br>(698 to 861)	-47.8%<br>(-51.8 to -43.6)	24,714<br>(23,148 to 26,277)	-20.1%<br>(-23.0 to -17.0)	19,117<br>(17,524 to 20,504)	-54.0%<br>(-57.2 to -51.0)
**New Zealand**	404<br>(356 to 460)	-37.6%<br>(-42.3 to -32.3)	240<br>(215 to 262)	-47.8%<br>(-52.2 to -43.0)	5,038<br>(4,455 to 5,619)	-23.3%<br>(-26.2 to -20.3)	6,421<br>(5,924 to 6,891)	-51.4%<br>(-55.0 to -47.8)
**Nicaragua**	573<br>(498 to 651)	-19.6%<br>(-24.5 to -13.4)	143<br>(112 to 174)	-31.8%<br>(-52.0 to -11.6)	7,786<br>(7,278 to 8,249)	-12.9%<br>(-16.1 to -10.0)	5,484<br>(4,476 to 6,642)	-34.3%<br>(-50.4 to -17.5)
**Niger**	917<br>(786 to 1,049)	-8.8%<br>(-13.7 to -3.4)	272<br>(106 to 666)	-17.7%<br>(-42.8 to 21.9)	11,722<br>(10,778 to 12,773)	-8.9%<br>(-11.8 to -5.8)	11,983<br>(6,066 to 25,236)	-20.8%<br>(-43.2 to 11.4)
**Nigeria**	7,452<br>(6,274 to 8,603)	-25.8%<br>(-27.6 to -24.1)	1,652<br>(811 to 3,669)	-48.6%<br>(-65.3 to -22.5)	104,736<br>(93,890 to 116,830)	-18.7%<br>(-20.8 to -16.7)	77,265<br>(47,024 to 148,445)	-43.6%<br>(-61.2 to -18.2)
**Niue**	0<br>(0 to 0)	-25.8%<br>(-30.2 to -21.1)	0<br>(0 to 0)	-31.1%<br>(-51.5 to -5.4)	3<br>(3 to 4)	-15.0%<br>(-17.9 to -12.1)	5<br>(4 to 7)	-14.6%<br>(-38.8 to 15.5)
**North Macedonia**	345<br>(313 to 379)	7.5%<br>(0.4 to 17.2)	225<br>(171 to 286)	-19.2%<br>(-43.5 to 9.9)	3,039<br>(2,828 to 3,261)	-10.1%<br>(-13.4 to -6.9)	6,415<br>(5,011 to 8,045)	-32.7%<br>(-50.0 to -12.1)
**Northern Mariana Islands**	7<br>(6 to 7)	-14.5%<br>(-19.8 to -8.7)	3<br>(2 to 4)	-30.8%<br>(-49.1 to -5.8)	95<br>(90 to 100)	-9.7%<br>(-12.6 to -6.5)	116<br>(93 to 145)	-30.1%<br>(-47.2 to -7.3)
**Norway**	548<br>(477 to 629)	-30.4%<br>(-33.9 to -26.2)	230<br>(201 to 248)	-60.1%<br>(-62.7 to -57.7)	8,416<br>(7,502 to 9,333)	-18.9%<br>(-21.0 to -16.6)	5,554<br>(5,076 to 6,019)	-63.3%<br>(-65.5 to -61.1)
**Oman**	322<br>(277 to 364)	5.5%<br>(-25.6 to 22.8)	31<br>(21 to 42)	-59.7%<br>(-79.0 to -33.4)	4,146<br>(3,849 to 4,459)	-10.9%<br>(-14.8 to -7.3)	1,662<br>(1,307 to 2,106)	-59.1%<br>(-74.2 to -40.2)
**Pakistan**	17,598<br>(15,071 to 20,383)	-16.7%<br>(-21.1 to -12.2)	7,368<br>(4,887 to 11,102)	-14.2%<br>(-35.6 to 14.2)	168,288<br>(148,166 to 189,881)	-8.8%<br>(-12.4 to -5.5)	300,150<br>(213,301 to 421,459)	-10.0%<br>(-31.0 to 14.6)
**Palau**	3<br>(3 to 4)	-20.3%<br>(-24.6 to -14.9)	1<br>(1 to 2)	-27.0%<br>(-49.5 to 1.2)	44<br>(41 to 46)	-8.7%<br>(-12.0 to -5.3)	53<br>(41 to 65)	-24.7%<br>(-46.3 to 0.3)
**Palestine**	208<br>(174 to 239)	-18.9%<br>(-23.1 to -14.8)	24<br>(18 to 29)	-47.8%<br>(-66.2 to -22.1)	2,915<br>(2,669 to 3,170)	-9.8%<br>(-13.0 to -6.4)	1,211<br>(1,015 to 1,438)	-46.0%<br>(-61.3 to -28.4)
**Panama**	522<br>(462 to 588)	-11.7%<br>(-17.9 to -5.1)	221<br>(174 to 266)	1.4%<br>(-22.6 to 24.6)	7,068<br>(6,694 to 7,440)	-8.3%<br>(-11.5 to -5.4)	6,784<br>(5,454 to 8,049)	-3.0%<br>(-23.0 to 17.0)
**Papua New Guinea**	806<br>(706 to 903)	-15.7%<br>(-20.5 to -9.8)	505<br>(340 to 732)	-31.6%<br>(-52.9 to -1.2)	10,052<br>(9,440 to 10,735)	-7.8%<br>(-11.1 to -4.8)	22,851<br>(16,572 to 30,614)	-29.4%<br>(-49.1 to 0.5)
**Paraguay**	712<br>(633 to 794)	-15.0%<br>(-22.1 to -8.9)	334<br>(250 to 436)	-23.9%<br>(-49.2 to 3.7)	7,976<br>(7,463 to 8,488)	-12.2%<br>(-15.5 to -8.8)	11,463<br>(8,839 to 14,749)	-28.7%<br>(-49.7 to -5.1)

**Peru**	3,772<br>(3,341 to 4,236)	-30.9%<br>(-35.5 to -26.6)	1,477<br>(1,061 to 1,978)	-39.7%<br>(-63.1 to -13.0)	56,391<br>(53,471 to 59,396)	-19.7%<br>(-22.5 to -17.0)	55,188<br>(40,897 to 70,950)	-40.5%<br>(-59.8 to -18.6)
**Philippines**	9,471<br>(8,155 to 10,901)	25.0%<br>(18.2 to 32.1)	3,721<br>(3,036 to 4,447)	7.6%<br>(-19.3 to 35.7)	105,545<br>(94,047 to 118,045)	23.3%<br>(19.1 to 26.9)	150,710<br>(120,818 to 177,279)	11.7%<br>(-9.4 to 33.9)
**Poland**	3,318<br>(2,911 to 3,856)	-37.5%<br>(-40.2 to -34.5)	1,678<br>(1,525 to 1,831)	-49.4%<br>(-54.1 to -44.7)	36,766<br>(32,368 to 41,420)	-28.9%<br>(-30.9 to -26.9)	51,621<br>(47,154 to 56,396)	-53.6%<br>(-57.7 to -49.5)
**Portugal**	1,092<br>(944 to 1,235)	-32.0%<br>(-43.0 to -26.8)	782<br>(681 to 852)	-43.4%<br>(-47.8 to -39.0)	12,043<br>(11,150 to 12,964)	-25.9%<br>(-28.9 to -22.8)	16,751<br>(15,399 to 18,035)	-50.3%<br>(-53.4 to -46.7)
**Puerto Rico**	369<br>(321 to 421)	-19.8%<br>(-23.7 to -15.8)	132<br>(109 to 154)	-36.6%<br>(-47.5 to -24.1)	6,510<br>(6,155 to 6,859)	-13.2%<br>(-16.2 to -10.2)	3,580<br>(3,025 to 4,155)	-37.5%<br>(-47.1 to -26.6)
**Qatar**	188<br>(152 to 227)	-11.8%<br>(-16.9 to -5.8)	29<br>(20 to 42)	-66.2%<br>(-81.7 to -43.0)	2,751<br>(2,565 to 2,944)	-10.9%<br>(-13.6 to -8.0)	1,506<br>(1,127 to 1,989)	-63.9%<br>(-78.6 to -41.1)
**Republic of Korea**	8,826<br>(7,696 to 10,360)	-46.2%<br>(-50.9 to -40.3)	3,044<br>(2,482 to 3,587)	-77.9%<br>(-84.0 to -70.9)	111,725<br>(105,688 to 117,850)	-24.7%<br>(-28.9 to -20.3)	90,559<br>(78,706 to 104,700)	-75.1%<br>(-81.2 to -68.2)
**Republic of Moldova**	273<br>(237 to 309)	-20.1%<br>(-23.9 to -15.9)	121<br>(109 to 136)	-27.2%<br>(-38.0 to -14.0)	2,832<br>(2,605 to 3,074)	-10.6%<br>(-14.2 to -6.2)	3,619<br>(3,236 to 4,074)	-24.4%<br>(-35.4 to -11.1)
**Romania**	3,153<br>(2,902 to 3,424)	-4.4%<br>(-9.5 to 0.7)	1,875<br>(1,627 to 2,109)	-5.4%<br>(-23.3 to 14.9)	18,663<br>(17,067 to 20,134)	-10.2%<br>(-13.4 to -6.6)	45,287<br>(38,768 to 51,071)	-13.2%<br>(-29.7 to 3.1)
**Russian Federation**	20,253<br>(17,895 to 23,246)	2.4%<br>(-2.5 to 7.4)	13,481<br>(12,417 to 14,528)	16.0%<br>(7.1 to 25.1)	175,732<br>(154,517 to 199,749)	-5.1%<br>(-7.5 to -2.9)	370,212<br>(343,245 to 400,093)	4.0%<br>(-3.9 to 11.7)
**Rwanda**	640<br>(544 to 738)	-31.3%<br>(-35.3 to -26.6)	184<br>(66 to 479)	-58.8%<br>(-74.0 to -38.0)	7,895<br>(7,321 to 8,567)	-25.7%<br>(-28.3 to -22.9)	7,397<br>(3,224 to 18,048)	-59.3%<br>(-72.8 to -40.2)
**Saint Kitts and Nevis**	7<br>(6 to 8)	-29.8%<br>(-34.4 to -25.1)	3<br>(2 to 3)	-45.4%<br>(-56.5 to -30.0)	92<br>(87 to 98)	-22.2%<br>(-24.8 to -19.3)	95<br>(79 to 114)	-49.3%<br>(-59.6 to -34.9)
**Saint Lucia**	21<br>(19 to 24)	-23.9%<br>(-28.8 to -18.8)	9<br>(7 to 10)	-47.6%<br>(-57.8 to -36.4)	300<br>(283 to 317)	-14.1%<br>(-17.4 to -10.8)	273<br>(226 to 328)	-46.8%<br>(-56.6 to -35.8)
**Saint Vincent and the Grenadines**	11<br>(10 to 12)	-22.3%<br>(-26.9 to -17.7)	4<br>(4 to 5)	-24.9%<br>(-36.1 to -13.1)	155<br>(146 to 165)	-15.4%<br>(-18.2 to -12.4)	148<br>(129 to 168)	-24.1%<br>(-35.8 to -13.1)
**Samoa**	26<br>(23 to 30)	-10.7%<br>(-16.5 to -4.8)	12<br>(8 to 17)	-28.9%<br>(-48.6 to -5.2)	321<br>(305 to 338)	-2.3%<br>(-5.7 to 1.0)	460<br>(332 to 625)	-24.3%<br>(-43.0 to -0.4)
**San Marino**	3<br>(2 to 3)	-18.0%<br>(-22.1 to -13.5)	1<br>(0 to 1)	-63.0%<br>(-76.9 to -44.0)	46<br>(42 to 49)	-9.2%<br>(-12.7 to -5.9)	17<br>(13 to 23)	-52.9%<br>(-66.6 to -37.2)
**Sao Tome and Principe**	10<br>(9 to 12)	-7.1%<br>(-11.6 to -2.6)	3<br>(1 to 8)	-3.7%<br>(-33.9 to 31.3)	154<br>(143 to 166)	-5.9%<br>(-9.3 to -2.2)	142<br>(68 to 344)	-4.1%<br>(-31.0 to 29.4)
**Saudi Arabia**	1,604<br>(1,317 to 1,891)	-22.0%<br>(-25.8 to -18.0)	336<br>(215 to 466)	-39.7%<br>(-70.6 to 2.3)	22,026<br>(20,099 to 24,047)	-11.1%<br>(-13.9 to -8.1)	16,540<br>(11,318 to 22,020)	-39.7%<br>(-67.3 to -6.2)
**Senegal**	596<br>(506 to 691)	-12.2%<br>(-16.4 to -7.9)	238<br>(109 to 545)	-15.4%<br>(-40.7 to 13.7)	8,844<br>(8,137 to 9,572)	-8.7%<br>(-12.1 to -5.5)	9,848<br>(5,070 to 21,005)	-18.6%<br>(-39.2 to 9.1)
**Serbia**	1,373<br>(1,258 to 1,495)	-18.2%<br>(-23.3 to -12.9)	758<br>(600 to 954)	-48.7%<br>(-63.0 to -30.6)	9,111<br>(8,404 to 9,834)	-25.4%<br>(-28.0 to -23.0)	19,696<br>(15,842 to 23,923)	-51.8%<br>(-64.8 to -35.6)
**Seychelles**	8<br>(7 to 10)	-28.0%<br>(-31.7 to -24.2)	2<br>(2 to 3)	-54.6%<br>(-69.1 to -42.1)	113<br>(105 to 122)	-15.8%<br>(-18.8 to -12.6)	89<br>(66 to 117)	-51.5%<br>(-62.0 to -40.8)

**Sierra Leone**	352<br>(299 to 400)	-6.5%<br>(-12.2 to -1.5)	137<br>(59 to 361)	-7.7%<br>(-34.5 to 29.6)	4,966<br>(4,578 to 5,401)	-5.2%<br>(-8.5 to -2.0)	6,316<br>(3,171 to 14,617)	-8.3%<br>(-33.6 to 27.9)
**Singapore**	714<br>(598 to 860)	-33.6%<br>(-38.3 to -28.6)	112<br>(100 to 121)	-71.3%<br>(-74.4 to -68.5)	10,869<br>(10,232 to 11,513)	-22.9%<br>(-26.0 to -20.3)	4,758<br>(4,161 to 5,325)	-65.2%<br>(-68.4 to -62.0)
**Slovakia**	409<br>(361 to 459)	-36.9%<br>(-40.7 to -33.3)	190<br>(148 to 235)	-48.6%<br>(-60.0 to -34.5)	4,992<br>(4,624 to 5,400)	-22.9%<br>(-25.3 to -19.9)	5,837<br>(4,791 to 7,064)	-49.6%<br>(-59.4 to -37.6)
**Slovenia**	163<br>(143 to 186)	-28.7%<br>(-32.6 to -24.6)	63<br>(53 to 72)	-58.8%<br>(-65.2 to -52.2)	1,943<br>(1,803 to 2,097)	-18.8%<br>(-22.0 to -15.7)	1,583<br>(1,353 to 1,791)	-61.7%<br>(-67.0 to -56.2)
**Solomon Islands**	113<br>(100 to 128)	20.9%<br>(-0.9 to 41.1)	41<br>(26 to 59)	-19.4%<br>(-43.6 to 25.2)	1,110<br>(1,049 to 1,165)	6.3%<br>(2.0 to 10.8)	1,647<br>(1,131 to 2,290)	-16.0%<br>(-41.5 to 33.7)
**Somalia**	1,002<br>(850 to 1,171)	-2.9%<br>(-7.9 to 2.7)	331<br>(59 to 1,095)	-28.0%<br>(-56.9 to 9.5)	10,958<br>(10,077 to 11,851)	-2.7%<br>(-6.2 to 1.3)	14,347<br>(3,649 to 43,038)	-26.2%<br>(-51.1 to 9.9)
**South Africa**	2,390<br>(2,039 to 2,798)	-16.8%<br>(-19.5 to -13.5)	677<br>(553 to 784)	8.2%<br>(-8.5 to 25.4)	33,949<br>(30,323 to 37,815)	-15.5%<br>(-17.7 to -13.2)	25,801<br>(21,832 to 29,661)	-7.7%<br>(-18.5 to 3.7)
**South Sudan**	400<br>(340 to 461)	-11.2%<br>(-15.8 to -5.7)	138<br>(56 to 317)	-19.2%<br>(-52.6 to 44.3)	4,807<br>(4,429 to 5,219)	-8.7%<br>(-11.7 to -5.3)	5,871<br>(2,870 to 12,151)	-18.2%<br>(-50.0 to 37.4)
**Spain**	4,624<br>(4,084 to 5,223)	-7.8%<br>(-13.4 to -1.9)	2,474<br>(2,123 to 2,720)	-8.0%<br>(-15.6 to -0.4)	61,132<br>(57,345 to 65,113)	-1.9%<br>(-6.0 to 2.6)	53,156<br>(48,538 to 57,519)	-26.3%<br>(-31.0 to -21.6)
**Sri Lanka**	2,616<br>(2,304 to 2,955)	-28.4%<br>(-32.8 to -23.6)	496<br>(331 to 685)	-45.2%<br>(-66.3 to -15.8)	29,340<br>(27,502 to 31,107)	-16.6%<br>(-19.3 to -13.9)	17,825<br>(13,270 to 22,812)	-41.2%<br>(-57.7 to -20.0)
**Sudan**	1,851<br>(1,608 to 2,099)	-31.4%<br>(-35.6 to -26.9)	772<br>(387 to 1,351)	-49.5%<br>(-68.9 to -14.5)	21,635<br>(19,688 to 23,837)	-23.3%<br>(-25.9 to -20.5)	32,379<br>(17,157 to 53,173)	-51.8%<br>(-67.9 to -26.3)
**Suriname**	64<br>(57 to 73)	-10.9%<br>(-16.0 to -5.1)	28<br>(21 to 37)	-24.0%<br>(-44.8 to 1.7)	830<br>(781 to 877)	-10.5%<br>(-13.6 to -7.2)	993<br>(783 to 1,248)	-24.7%<br>(-43.6 to -3.1)
**Sweden**	1,038<br>(895 to 1,202)	-29.8%<br>(-33.9 to -25.2)	412<br>(352 to 466)	-58.9%<br>(-63.4 to -54.0)	14,664<br>(13,015 to 16,327)	-19.9%<br>(-23.0 to -16.9)	9,604<br>(8,564 to 10,708)	-61.4%<br>(-65.4 to -57.5)
**Switzerland**	765<br>(667 to 891)	-25.9%<br>(-30.5 to -20.3)	332<br>(279 to 371)	-50.4%<br>(-55.5 to -45.8)	10,473<br>(9,750 to 11,168)	-9.2%<br>(-12.7 to -5.4)	7,362<br>(6,596 to 8,088)	-56.7%<br>(-60.3 to -52.6)
**Syrian Arab Republic**	880<br>(767 to 986)	-23.0%<br>(-36.6 to -15.8)	279<br>(185 to 400)	-44.3%<br>(-74.8 to -1.4)	14,352<br>(13,469 to 15,256)	-16.5%<br>(-19.4 to -13.5)	10,446<br>(7,545 to 14,292)	-51.4%<br>(-72.7 to -20.4)
**Turkey**	6,170<br>(5,447 to 6,955)	-39.2%<br>(-44.1 to -33.5)	3,415<br>(2,636 to 4,257)	-60.7%<br>(-77.3 to -40.7)	83,114<br>(77,362 to 89,138)	-28.8%<br>(-31.5 to -26.1)	99,852<br>(80,292 to 123,128)	-66.5%<br>(-79.1 to -52.2)
**Taiwan (Province of China)**	3,028<br>(2,679 to 3,453)	-13.7%<br>(-23.6 to -4.4)	945<br>(846 to 1,023)	-35.6%<br>(-41.7 to -30.2)	36,836<br>(34,164 to 39,654)	-3.5%<br>(-7.0 to -0.5)	29,545<br>(26,678 to 32,188)	-29.9%<br>(-35.4 to -24.7)
**Tajikistan**	761<br>(668 to 854)	13.9%<br>(7.4 to 21.2)	212<br>(150 to 288)	-18.7%<br>(-46.6 to 24.4)	7,155<br>(6,619 to 7,686)	-3.7%<br>(-7.9 to 0.0)	8,099<br>(5,901 to 10,920)	-22.6%<br>(-45.0 to 14.3)
**Thailand**	12,270<br>(10,909 to 14,011)	-33.7%<br>(-38.1 to -28.2)	8,543<br>(6,551 to 10,928)	-49.7%<br>(-65.9 to -30.5)	124,121<br>(116,044 to 131,462)	-18.5%<br>(-21.8 to -15.1)	233,063<br>(183,334 to 302,014)	-42.3%<br>(-58.5 to -23.2)
**Timor-Leste**	93<br>(81 to 105)	-11.4%<br>(-16.4 to -6.4)	54<br>(31 to 95)	-22.4%<br>(-49.2 to 11.4)	984<br>(903 to 1,062)	-4.7%<br>(-8.1 to -1.1)	1,924<br>(1,235 to 3,121)	-22.1%<br>(-46.0 to 8.3)
**Togo**	335<br>(288 to 387)	-9.6%<br>(-13.9 to -4.8)	141<br>(58 to 361)	-5.5%<br>(-35.6 to 37.2)	4,795<br>(4,417 to 5,207)	-7.8%<br>(-10.8 to -4.3)	5,988<br>(2,824 to 13,839)	-8.3%<br>(-35.4 to 30.5)

**Tokelau**	0<br>(0 to 0)	-25.4%<br>(-30.2 to -18.8)	0<br>(0 to 0)	-43.8%<br>(-65.5 to -17.8)	3<br>(2 to 3)	-14.3%<br>(-17.3 to -11.5)	4<br>(3 to 6)	-29.4%<br>(-52.8 to 1.5)
**Tonga**	9<br>(8 to 10)	-18.0%<br>(-22.3 to -13.2)	3<br>(2 to 5)	-25.6%<br>(-48.0 to 3.8)	120<br>(113 to 127)	-9.7%<br>(-12.8 to -6.7)	120<br>(87 to 169)	-23.2%<br>(-43.1 to 2.5)
**Trinidad and Tobago**	167<br>(149 to 189)	-19.0%<br>(-23.6 to -13.9)	91<br>(69 to 116)	-26.0%<br>(-43.5 to -2.8)	2,214<br>(2,079 to 2,336)	-15.5%<br>(-18.3 to -12.4)	2,966<br>(2,321 to 3,759)	-24.0%<br>(-40.7 to -1.8)
**Tunisia**	768<br>(664 to 875)	-26.7%<br>(-31.1 to -21.6)	263<br>(139 to 446)	-46.3%<br>(-72.2 to -1.2)	10,296<br>(9,529 to 11,100)	-15.3%<br>(-18.2 to -12.2)	7,970<br>(4,964 to 12,533)	-48.3%<br>(-69.3 to -16.1)
**Turkmenistan**	564<br>(503 to 631)	25.2%<br>(17.8 to 33.5)	350<br>(262 to 496)	49.8%<br>(13.2 to 99.3)	5,225<br>(4,886 to 5,588)	18.6%<br>(13.2 to 23.8)	12,388<br>(9,441 to 16,976)	41.7%<br>(9.0 to 85.4)
**Tuvalu**	2<br>(2 to 2)	-16.3%<br>(-21.8 to -9.1)	1<br>(1 to 2)	-44.8%<br>(-60.5 to -27.1)	21<br>(20 to 23)	-9.5%<br>(-12.6 to -5.9)	40<br>(28 to 57)	-42.8%<br>(-56.9 to -25.2)
**Uganda**	1,607<br>(1,384 to 1,858)	-13.7%<br>(-18.9 to -8.1)	399<br>(148 to 1,058)	-40.1%<br>(-56.4 to -1.3)	18,736<br>(17,236 to 20,551)	-9.5%<br>(-12.9 to -6.3)	17,762<br>(8,211 to 42,884)	-35.0%<br>(-52.0 to -4.5)
**Ukraine**	5,676<br>(5,004 to 6,504)	-8.6%<br>(-14.3 to -2.8)	3,646<br>(2,771 to 4,574)	-29.5%<br>(-48.9 to -8.3)	50,928<br>(44,858 to 57,601)	3.2%<br>(-0.7 to 7.8)	94,974<br>(71,883 to 118,614)	-17.9%<br>(-39.8 to 6.4)
**United Arab Emirates**	542<br>(427 to 668)	-25.2%<br>(-29.6 to -20.9)	108<br>(80 to 143)	-42.5%<br>(-73.0 to -9.5)	7,892<br>(7,244 to 8,569)	-17.6%<br>(-20.3 to -14.7)	5,088<br>(4,053 to 6,406)	-50.5%<br>(-72.9 to -24.2)
**United Kingdom**	7,198<br>(6,369 to 8,167)	-28.0%<br>(-30.6 to -24.8)	3,927<br>(3,516 to 4,141)	-47.2%<br>(-50.0 to -45.3)	88,688<br>(79,594 to 97,588)	-20.0%<br>(-21.6 to -18.4)	91,480<br>(86,291 to 96,131)	-54.3%<br>(-55.7 to -52.8)
**United Republic of Tanzania**	2,553<br>(2,193 to 2,945)	-14.9%<br>(-19.9 to -8.7)	625<br>(245 to 1,705)	-36.7%<br>(-58.1 to -2.4)	31,042<br>(28,726 to 33,529)	-10.3%<br>(-13.6 to -6.5)	26,522<br>(12,746 to 64,684)	-34.3%<br>(-53.0 to -7.5)
**United States of America**	29,758<br>(26,204 to 34,291)	-9.0%<br>(-12.7 to -3.9)	18,045<br>(16,292 to 19,093)	-13.9%<br>(-18.3 to -10.3)	454,302<br>(409,930 to 503,031)	6.0%<br>(1.1 to 11.2)	481,261<br>(452,793 to 508,845)	-23.9%<br>(-27.1 to -21.1)
**United States Virgin Islands**	13<br>(11 to 15)	-19.3%<br>(-23.9 to -14.0)	4<br>(3 to 5)	-57.8%<br>(-70.4 to -41.0)	185<br>(173 to 195)	-15.0%<br>(-17.9 to -11.8)	120<br>(93 to 153)	-54.0%<br>(-66.7 to -37.2)
**Uruguay**	609<br>(548 to 679)	-25.1%<br>(-30.8 to -19.1)	354<br>(325 to 381)	-39.2%<br>(-44.6 to -32.4)	6,557<br>(6,029 to 7,131)	-19.5%<br>(-23.0 to -14.2)	9,603<br>(8,915 to 10,264)	-45.6%<br>(-50.0 to -40.4)
**Uzbekistan**	2,063<br>(1,793 to 2,335)	3.0%<br>(-6.2 to 13.2)	1,095<br>(914 to 1,327)	111.1%<br>(60.7 to 170.2)	22,854<br>(21,189 to 24,490)	-0.7%<br>(-5.1 to 4.4)	34,891<br>(29,825 to 41,371)	56.8%<br>(25.9 to 95.9)
**Vanuatu**	40<br>(36 to 45)	2.2%<br>(-3.9 to 8.5)	19<br>(12 to 28)	-22.9%<br>(-43.9 to 3.9)	460<br>(436 to 485)	4.3%<br>(0.7 to 8.1)	847<br>(573 to 1,223)	-18.5%<br>(-40.8 to 9.6)
**Venezuela (Bolivarian Republic of)**	3,643<br>(3,245 to 4,223)	-11.2%<br>(-16.5 to -5.2)	1,628<br>(1,207 to 2,110)	5.9%<br>(-21.9 to 38.5)	51,018<br>(48,401 to 53,608)	-7.9%<br>(-11.2 to -4.5)	55,023<br>(41,132 to 70,455)	-2.4%<br>(-27.4 to 26.3)
**Viet Nam**	10,871<br>(9,515 to 12,536)	-19.3%<br>(-24.9 to -13.8)	4,926<br>(2,830 to 8,830)	-39.7%<br>(-64.0 to -7.9)	116,555<br>(109,076 to 124,411)	-13.2%<br>(-16.4 to -9.9)	158,572<br>(105,633 to 248,410)	-38.6%<br>(-59.6 to -10.5)
**Yemen**	1,615<br>(1,419 to 1,807)	-17.3%<br>(-22.6 to -11.4)	785<br>(374 to 1,445)	-29.7%<br>(-55.3 to 3.8)	17,482<br>(15,899 to 19,257)	-14.9%<br>(-17.6 to -11.8)	29,476<br>(15,799 to 48,742)	-35.2%<br>(-56.1 to -6.2)
**Zambia**	843<br>(719 to 972)	-6.9%<br>(-12.4 to -0.9)	282<br>(125 to 591)	-22.2%<br>(-50.2 to 21.6)	9,656<br>(8,845 to 10,453)	-6.7%<br>(-10.0 to -3.0)	11,535<br>(5,692 to 22,741)	-19.2%<br>(-46.0 to 15.3)
**Zimbabwe**	865<br>(754 to 986)	23.8%<br>(15.6 to 33.7)	444<br>(294 to 709)	51.6%<br>(11.8 to 102.4)	10,517<br>(9,793 to 11,249)	17.1%<br>(12.4 to 22.2)	17,404<br>(11,843 to 26,504)	64.6%<br>(20.2 to 119.1)

Appendix Table 5. Absolute number and age-standardised rates per 100,000 people per year, with 95% uncertainty intervals (UI), of incident and prevalent strokes, deaths from stroke and DALYs due to stroke in 2021 and percentage change in the metrics for 1990-2021, by sex and pathological type of stroke

Pathological type of stroke by sex		Incidence (95% UI)		Deaths (95% UI)		Prevalence (95% UI)		DALYs (95% UI)	
		Metric in 2021	Percentage change, 1990-2021	Metric in 2021	Percentage change, 1990-2021	Metric in 2021	Percentage change, 1990-2021	Metric in 2021	Percentage change, 1990-2021
**Ischaemic stroke** Males	Absolute number	4,022,421 (3,444,603 to 4,666,933)	100.0% (91.9 to 108.4)	1,778,735 (1,611,777 to 1,962,926	76.6% (56.1 to 99.5)	35,241,152 (32,609,809 to 37,813,501)	109.7% (105.0 to 114.1)	37,007,430 (33,581,055 to 40,659,723)	69.1% (49.7 to 89.9)
	Age-standardised rate	103 (89 to 119)	-12.2% (-15.4 to -9.1)	51 (46 to 56)	-33.1% (-40.3 to -25.0)	882 (819 to 945)	-2.6% (-4.3 to -0.8)	975 (886 to 1,070)	-29.5% (-37.1 to -21.3)
Females	Absolute number	3,782,028 (3,253,626 to 4,336,193)	76.7% (70.6 to 83.2)	1,812,764 (1,573,025 to 2,001,279)	38.4% (27.1 to 50.3)	34,703,732 (32,096,979 to 37,274,015)	94.3% (90.2 to 98.9)	33,350,482 (29,775,875 to 36,653,245)	37.3% (26.9 to 48.7)
	Age-standardised rate	83 (71 to 95)	-19.5% (-22.0 to -17.0)	39 (33 to 43)	-44.9% (-48.9 to -40.4)	769 (713 to 826)	-5.3% (-6.9 to -3.5)	720 (643 to 792)	-40.0% (-44.4 to -35.2)
**Intracerebral haemorrhage** Males	Absolute number	1,920,625 (1,693,928 to 2,134,864)	52.6% (47.1 to 59.3)	1,822,740 (1,630,620 to 2,047,646)	51.2% (31.0 to 75.7)	9,349,225 (8,494,933 to 10,241,209)	58.0% (52.6 to 62.7)	45,786,602 (41,290,470 to 51,086,283)	34.4% (16.8 to 54.1)
	Age-standardised rate	49 (43 to 54)	-29.1% (-31.6 to -26.3)	47 (42 to 53)	-32.6% (-41.5 to -21.7)	225 (205 to 246)	-18.0% (-19.9 to -16.2)	1,123 (1,014 to 1,252)	-35.2% (-43.5 to -25.5)
Females	Absolute number	1,523,713 (1,332,150 to 1,692,377)	38.5% (33.1 to 45.3)	1,485,627 (1,310,633 to 1,667,510)	30.8% (15.0 to 50.1)	7,254,612 (6,657,659 to 7,924,926)	38.0% (33.4 to 42.2)	33,670,825 (29,843,463 to 37,371,743)	15.6% (1.1 to 31.2)

	Age-standardised rate	34 (29 to 37)	-34.9% (-37.3 to -32.2)	32 (28 to 36)	-41.1% (-48.0 to -32.4)	166 (152 to 181)	-27.4% (-29.0 to -25.9)	743 (658 to 823)	-44.0% (-50.6 to -36.4)
**Subarachnoid haemorrhage** Males	Absolute number	340,845 (298,157 to 388,882)	39.9% (34.4 to 46.1)	173,754 (140,710 to 217,574)	-4.0% (-26.0 to 57.3)	3,543,050 (3,215,456 to 3,892,393)	61.7% (57.9 to 65.1)	5,483,458 (4,498,455 to 6,900,833)	-10.2% (-28.1 to 30.2)
	Age-standardised rate	9 (7 to 10)	-27.4% (-30.7 to -23.9)	4 (4 to 6)	-55.1% (-65.8 to -23.6)	86 (78 to 94)	-14.2% (-15.7 to -12.8)	134 (110 to 168)	-53.6% (-63.2 to -30.5)
Females	Absolute number	356,641 (315,063 to 408,979)	34.5% (30.0 to 39.1)	179,056 (156,258 to 208,098)	-7.7% (-26.0 to 26.5)	4,309,742 (3,947,844 to 4,690,950)	59.0% (55.6 to 62.1)	5,158,424 (4,617,347 to 5,889,984)	-13.0% (-26.9 to 14.4)
	Age-standardised rate	8 (7 to 9)	-30.4% (-32.9 to -27.6)	4 (3 to 5)	-57.2% (-65.5 to -41.1)	98 (90 to 107)	-17.6% (-19.2 to -16.2)	116 (104 to 133)	-55.8% (-63.0 to -42.2)
**Total stroke** Males	Absolute number	6,283,892 (5,642,547 to 7,014,118)	78.9% (73.7 to 84.2)	3,775,229 (3,426,108 to 4,147,370)	57.7% (40.3 to 76.5)	47,810,536 (45,336,514 to 50,574,534)	93.2% (90.0 to 96.7)	88,277,490 (80,629,517 to 97,164,305)	42.3% (26.9 to 59.1)
	Age-standardised rate	160 (144 to 178)	-19.0% (-21.0 to -16.7)	103 (93 to 113)	-34.3% (-41.2 to -27.0)	1,184 (1,124 to 1,252)	-6.7% (-8.1 to -5.4)	2,232 (2,037 to 2,451)	-34.4% (-41.3 to -26.9)
Females	Absolute number	5,662,382 (5,121,951 to 6,251,503)	61.5% (57.1 to 66.0)	3,477,446 (3,067,204 to 3,806,862)	31.7% (20.0 to 45.2)	46,005,878 (43,540,264 to 48,766,467)	79.2% (75.9 to 82.6)	72,179,731 (65,549,700 to 78,222,470)	21.6% (10.1 to 33.1)
	Age-standardised rate	125 (113 to 137)	-25.0% (-26.7 to -23.1)	75 (66 to 82)	-44.2% (-48.9 to -38.7)	1,028 (974 to 1,088)	-10.8% (-12.1 to -9.5)	1,578 (1,435 to 1,709)	-43.4% (-48.7 to -38.3)

Appendix Table 6. Percentage change (%) in stroke incidence, prevalence, death, and DALY rates per 100,000 between 1990 and 2021 by pathological type of stroke in people younger and older that 70 years (with 95% UIs)

**Stroke pathological type**		**<70 years old**	**>70 years old**
Ischaemic stroke	Incidence	17.1% (11.7 to 22.0)	-13.7% (-18.0 to -8.5)
	Prevalence	24.6% (22.0 to 27.5)	0.5% (-2.2 to 3.0)
	Death	-7.0% (-17.2 to 4.0)	-34.2% (-38.7 to -29.9)
	DALY	-3.0% (-11.6 to 6.0)	-34.0% (-38.6 to -29.7)
Intracerebral Haemorrhage	Incidence	-11.3% (-14.4 to -6.9)	-26.9% (-31.2 to -21.9)
	Prevalence	-3.8% (-6.8 to -0.9)	-10.8% (-13.3 to -8.5)
	Death	-17.2% (-25.3 to -8.2)	-31.5% (-38.8 to -24.2)
	DALY	-20.9% (-28.5 to -13.0)	-35.3% (-41.7 to -28.5)
Subarachnoid haemorrhage	Incidence	-11.6% (-14.5 to -8.1)	-28.6% (-33.8 to -21.5)
	Prevalence	3.4% (1.3 to 5.5)	-7.4% (-10.6 to -4.0)
	Death	-42.0% (-51.3 to -27.5)	-54.6% (-65.3 to -30.5)
	DALY	-41.9% (-49.7 to -30.1)	-55.9% (-66.1 to -34.8)
**All strokes combined**	Incidence	4.1% (0.9 to 7.6)	-18.2% (-21.3 to -14.6)
	Prevalence	14.8% (13.1 to 16.8)	-1.0% (-3.1 to 1.2)
	Death	-17.4% (-25.0 to -8.9)	-34.2% (-39.4 to -29.3)
	DALY	-19.0% (-26.0 to -11.6)	-35.6% (-40.2 to -30.8)

Appendix Table 7. Stroke related disability-adjusted life years (DALYs) (absolute numbers with 95% UI and age-standardized percentages with 95% UI) associated with risk factors and their clusters by Socio-demographic Index (SDI) quintiles in 2021, both sexes

	High SDI		High-middle SDI		Middle SDI		Low-middle SDI		Low SDI		Globally	
	Absolute number	Percentage	Absolute number	Percentage	Absolute number	Percentage	Absolute number	Percentage	Absolute number	Percentage	Absolute number	Percentage
**Air pollution and environmental risks**												
Ambient particulate matter pollution	1,618,292 (1,220,382 to 2,075,514)	10.9% (8.4 to 13.9)	7,173,173 (5,095,857 to 9,077,255)	18.5% (13.8 to 22.8)	12,300,098 (7,781,567 to 15,739,017)	20.5% (13.2 to 25.7)	4,628,331 (2,745,367 to 6,587,800)	13.8% (8.1 to 19.2)	1,042,429 (659,962 to 1,532,130)	8.5% (5.4 to 11.8)	26,779,431 (18,076,188 to 34,223,093)	16.6% (11.5 to 20.9)
High temperature	76,049 (-27,450 to 227,016)	0.6% (-0.1 to 1.7)	133,315 (-138,352 to 557,724)	0.4% (-0.3 to 1.5)	574,416 (117,524 to 1,314,623)	1.0% (0.2 to 2.2)	773,429 (232,163 to 1,522,314)	2.3% (0.7 to 4.5)	237,420 (98,413 to 431,554)	1.8% (0.7 to 3.3)	1,795,479 (328,542 to 4,115,043)	1.1% (0.2 to 2.5)
Household air pollution from solid fuels	7,420 (10 to 73,145)	0.0% (0.0 to 0.5)	626,885 (37,276 to 3,288,770)	1.6% (0.1 to 8.3)	4,520,000 (1,097,732 to 11,102,783)	7.5% (1.7 to 19.1)	8,206,284 (5,344,160 to 11,525,425)	24.6% (16.1 to 33.9)	4,798,952 (3,778,918 to 5,774,129)	38.7% (31.3 to 45.7)	18,175,209 (10,671,999 to 30,875,848)	11.2% (6.4 to 19.3)
Lead exposure	574,022 (-75,595 to 1,294,490)	3.6% (-0.5 to 8.3)	2,353,716 (-307,659 to 5,288,335)	6.0% (-0.8 to 13.3)	4,677,725 (-616,460 to 10,273,817)	7.8% (-1.0 to 17.5)	3,177,634 (-429,455 to 6,933,809)	9.7% (-1.3 to 21.5)	1,231,329 (-162,735 to 2,659,560)	10.3% (-1.4 to 22.7)	12,023,066 (-1,593,055 to 26,565,153)	7.4% (-1.0 to 16.5)
Low temperature	969,580 (838,100 to 1,119,868)	6.2% (5.4 to 7.1)	2,753,367 (2,334,423 to 3,230,787)	7.1% (6.2 to 8.1)	2,811,088 (2,349,827 to 3,399,022)	4.8% (4.2 to 5.4)	777,105 (482,011 to 1,138,994)	2.3% (1.5 to 3.4)	293,527 (216,413 to 386,072)	2.3% (1.7 to 3.0)	7,610,886 (6,484,027 to 9,012,218)	4.8% (4.1 to 5.5)
**Dietary risks**												
Alcohol use	1,207,772 (220,316 to 2,472,101)	7.8% (1.6 to 15.6)	2,641,310 (587,484 to 5,246,739)	6.8% (1.4 to 13.5)	3,209,061 (824,090 to 5,960,606)	5.1% (1.3 to 9.6)	960,469 (220,850 to 1,849,954)	2.7% (0.6 to 5.2)	410,029 (87,207 to 817,197)	3.2% (0.7 to 6.3)	8,437,272 (2,056,466 to 16,101,315)	5.2% (1.3 to 9.8)
Diet high in processed meat	157,788 (38,009 to 277,982)	1.0% (0.2 to 1.8)	172,956 (40,103 to 305,737)	0.4% (0.1 to 0.7)	56,943 (13,808 to 101,400)	0.1% (0.0 to 0.2)	36,633 (8,550 to 66,730)	0.1% (0.0 to 0.2)	11,643 (2,650 to 21,840)	0.1% (0.0 to 0.2)	436,528 (101,726 to 781,514)	0.3% (0.1 to 0.5)
Diet high in red meat	-504,373 (-2,197,734 to 756,836)	-4.3% (-19.1 to 6.4)	-1,533,828 (-6,621,509 to 2,197,905)	-4.2% (-17.9 to 5.9)	-2,463,134 (-10,549,357 to 3,393,182)	-3.8% (-15.8 to 5.5)	-464,043 (-1,776,295 to 675,237)	-1.2% (-4.6 to 1.7)	-191,050 (-722,698 to 300,900)	-1.2% (-4.5 to 1.9)	-5,160,253 (-21,845,162 to 7,265,547)	-3.1% (-13.2 to 4.6)
Diet high in sodium	1,195,030 (193,711 to 2,973,680)	7.9% (1.3 to 19.2)	5,186,845 (1,648,629 to 10,453,621)	13.2% (4.2 to 25.9)	7,992,428 (2,394,524 to 16,504,209)	12.8% (3.7 to 25.5)	2,407,904 (298,788 to 6,255,313)	7.1% (0.8 to 18.6)	605,122 (22,072 to 1,857,144)	5.1% (0.2 to 15.1)	17,402,145 (4,611,509 to 37,981,974)	10.6% (2.8 to 22.8)

Diet high in sugar-sweetened beverages	44,049 (21,332 to 69,942)	0.3% (0.1 to 0.5)	48,900 (24,176 to 76,852)	0.1% (0.1 to 0.2)	35,403 (17,400 to 56,087)	0.1% (0.0 to 0.1)	13,936 (6,441 to 22,399)	0.0% (0.0 to 0.1)	2,082 (927 to 3,488)	0.0% (0.0 to 0.0)	144,644 (70,033 to 228,901)	0.1% (0.0 to 0.1)
Diet low in fiber	264,290 (-52,925 to 572,398)	2.1% (-0.5 to 4.6)	535,730 (-106,849 to 1,154,498)	1.5% (-0.3 to 3.1)	1,663,103 (-378,735 to 3,490,815)	2.6% (-0.6 to 5.4)	1,302,892 (-310,827 to 2,682,815)	3.5% (-0.8 to 7.2)	303,831 (-71,890 to 621,516)	2.0% (-0.5 to 4.1)	4,073,480 (-924,081 to 8,455,822)	2.5% (-0.6 to 5.1)
Diet low in fruits	516,332 (47,111 to 934,592)	4.2% (0.4 to 7.3)	1,398,156 (132,755 to 2,602,604)	3.7% (0.3 to 6.7)	3,357,858 (187,612 to 6,172,691)	5.2% (0.3 to 9.4)	3,155,983 (229,264 to 5,384,288)	8.5% (0.7 to 14.8)	1,192,835 (63,368 to 2,073,298)	8.1% (0.5 to 14.2)	9,627,705 (659,562 to 16,956,862)	5.9% (0.4 to 10.4)
Diet low in polyunsaturated fatty acids	1,430 (373 to 2,767)	0.0% (0.0 to 0.0)	4,874 (1,376 to 9,315)	0.0% (0.0 to 0.0)	6,274 (1,850 to 11,841)	0.0% (0.0 to 0.0)	3,598 (1,085 to 6,692)	0.0% (0.0 to 0.0)	1,321 (409 to 2,502)	0.0% (0.0 to 0.0)	17,512 (5,110 to 33,142)	0.0% (0.0 to 0.0)
Diet low in vegetables	80,456 (16,588 to 143,442)	0.6% (0.2 to 1.0)	107,956 (41,774 to 181,354)	0.3% (0.1 to 0.5)	533,475 (174,883 to 885,970)	0.8% (0.3 to 1.4)	945,810 (220,408 to 1,607,529)	2.6% (0.6 to 4.5)	869,415 (133,198 to 1,497,932)	5.9% (1.1 to 10.3)	2,540,439 (644,870 to 4,266,752)	1.6% (0.4 to 2.6)
Diet low in whole grains	305,307 (-296,593 to 932,256)	2.3% (-2.4 to 6.5)	998,664 (-993,408 to 2,783,931)	2.6% (-2.6 to 7.3)	1,041,418 (-1,058,613 to 2,777,922)	1.6% (-1.7 to 4.5)	551,268 (-573,076 to 1,522,328)	1.5% (-1.6 to 4.3)	225,727 (-233,963 to 624,915)	1.6% (-1.6 to 4.8)	3,125,286 (-3,174,925 to 8,780,802)	1.9% (-1.9 to 5.2)
**Physical activity**												
Low physical activity	369,729 (-29,176 to 824,958)	2.5% (0.5 to 4.9)	938,283 (163,766 to 1,887,964)	2.4% (0.4 to 4.9)	1,238,873 (407,237 to 2,276,382)	2.0% (0.5 to 4.0)	649,397 (236,683 to 1,132,656)	1.9% (0.6 to 3.6)	159,112 (60,352 to 277,054)	1.3% (0.4 to 2.4)	3,358,676 (907,212 to 6,300,659)	2.1% (0.5 to 3.9)
**Tobacco smoking**												
Secondhand smoke	379,437 (255,116 to 511,694)	2.9% (2.0 to 3.8)	1,749,930 (1,188,073 to 2,334,656)	4.6% (3.1 to 6.1)	2,923,158 (1,985,424 to 3,882,163)	4.8% (3.2 to 6.3)	1,510,920 (1,033,501 to 2,033,989)	4.3% (3.0 to 5.7)	401,115 (269,515 to 545,049)	2.9% (2.0 to 3.9)	6,970,263 (4,734,823 to 9,224,428)	4.3% (2.9 to 5.7)
Smoking	1,844,712 (1,546,770 to 2,203,924)	14.0% (12.0 to 16.0)	6,329,602 (5,214,966 to 7,672,789)	16.4% (14.0 to 18.9)	9,431,182 (7,769,576 to 11,238,709)	14.8% (12.6 to 17.1)	3,974,032 (3,373,136 to 4,638,700)	11.1% (9.5 to 12.7)	925,091 (762,977 to 1,114,591)	6.6% (5.6 to 7.6)	22,522,286 (19,059,171 to 26,588,415)	13.7% (11.8 to 15.8)
**Physiological factors**												
High body-mass index	1,049,202 (78,748 to 2,119,661)	8.0% (0.5 to 15.9)	2,346,771 (192,252 to 4,872,138)	6.1% (0.5 to 12.5)	2,551,553 (197,781 to 5,325,535)	4.0% (0.3 to 8.3)	1,337,693 (115,553 to 2,732,602)	3.7% (0.3 to 7.4)	392,914 (30,460 to 848,985)	2.7% (0.2 to 5.6)	7,687,489 (623,220 to 15,857,422)	4.7% (0.4 to 9.8)
High fasting plasma glucose	2,149,123 (1,683,977 to 2,626,381)	12.9% (10.4 to 15.4)	4,516,418 (3,547,301 to 5,627,111)	11.5% (9.1 to 14.1)	5,701,967 (4,402,662 to 7,154,884)	9.8% (7.7 to 12.1)	3,174,197 (2,392,568 to 3,984,963)	10.2% (8.0 to 12.8)	911,486 (675,732 to 1,145,383)	8.4% (6.4 to 10.5)	16,470,575 (12,733,096 to 20,293,678)	10.3% (8.1 to 12.6)
High LDL cholesterol	2,663,216 (900,957 to 4,379,680)	17.1% (6.1 to 27.4)	6,609,150 (2,320,767 to 10,837,819)	17.0% (6.0 to 27.3)	7,148,661 (2,541,446 to 11,776,116)	12.0% (4.2 to 19.7)	3,460,220 (1,236,731 to 5,729,942)	10.6% (3.7 to 18.0)	1,074,118 (379,636 to 1,799,295)	9.0% (3.0 to 15.3)	20,977,424 (7,409,044 to 34,664,410)	13.1% (4.6 to 21.3)
High systolic blood pressure	8,136,019 (5,997,155 to 10,020,959)	52.1% (38.1 to 63.0)	22,821,648 (16,771,193 to 28,096,412)	58.5% (44.0 to 69.8)	34,699,045 (25,780,138 to 43,171,436)	57.4% (42.8 to 69.0)	19,285,154 (14,613,290 to 23,617,773)	58.0% (43.6 to 68.9)	6,827,094 (5,098,028 to 8,523,048)	55.2% (40.8 to 66.2)	91,854,061 (69,066,470 to 112,223,192)	56.8% (42.5 to 68.0)

Kidney dysfunction	1,259,755 (843,611 to 1,716,268)	7.8% (5.5 to 10.2)	3,250,919 (2,322,619 to 4,274,134)	8.3% (5.9 to 10.8)	5,733,695 (4,164,893 to 7,312,493)	9.4% (6.8 to 12.0)	3,529,135 (2,595,110 to 4,485,993)	10.6% (7.9 to 13.4)	1,223,014 (887,186 to 1,592,856)	9.9% (7.4 to 12.6)	15,009,653 (10,939,085 to 19,133,717)	9.3% (6.8 to 11.8)
**Cluster of risk factors**												
Air pollution*	1,625,947 (1,232,130 to 2,082,329)	11.0% (8.4 to 14.0)	7,801,888 (5,931,357 to 10,188,263)	20.1% (15.8 to 25.7)	16,823,974 (12,428,844 to 21,838,219)	28.0% (21.5 to 35.6)	12,835,905 (10,109,819 to 15,248,023)	38.4% (31.3 to 45.7)	5,841,672 (4,664,357 to 6,927,649)	47.2% (39.4 to 54.6)	44,962,167 (35,020,339 to 55,467,024)	27.7% (22.3 to 34.2)
Behavioral risks§	4,743,551 (3,372,752 to 6,391,027)	33.0% (24.5 to 42.6)	14,709,720 (11,131,069 to 19,278,853)	37.8% (28.7 to 47.8)	22,642,487 (17,144,906 to 29,775,285)	36.2% (27.3 to 46.2)	11,454,081 (7,900,439 to 14,764,054)	32.6% (22.6 to 42.6)	3,898,121 (2,289,341 to 5,278,960)	28.7% (16.8 to 40.0)	57,498,006 (44,175,632 to 73,990,120)	35.2% (26.9 to 44.7)
Dietary risks‡	1,771,389 (672,660 to 3,470,653)	11.9% (4.2 to 22.6)	6,350,546 (2,674,046 to 11,335,959)	16.1% (6.5 to 28.4)	10,488,502 (4,607,336 to 18,575,208)	16.7% (7.5 to 29.1)	6,230,996 (1,912,694 to 10,098,439)	17.6% (5.4 to 29.7)	2,513,888 (669,506 to 4,042,223)	18.3% (4.8 to 31.2)	27,379,104 (13,000,767 to 45,753,348)	16.7% (8.0 to 27.9)
Environmental/occupational risks¶	3,011,109 (2,270,371 to 3,792,847)	19.8% (15.2 to 24.3)	11,772,237 (8,937,819 to 14,976,768)	30.3% (23.9 to 37.2)	22,276,229 (16,665,682 to 28,375,703)	37.1% (28.6 to 45.9)	15,561,930 (12,221,100 to 18,538,020)	46.6% (37.2 to 55.0)	6,739,259 (5,471,566 to 7,905,821)	54.4% (45.3 to 62.4)	59,405,418 (45,886,431 to 72,098,856)	36.7% (29.0 to 44.2)
Metabolic risks¦	10,574,907 (8,793,030 to 12,289,710)	67.6% (56.5 to 76.4)	27,849,540 (22,707,467 to 32,430,269)	71.6% (60.1 to 80.0)	41,489,603 (34,014,479 to 48,707,818)	68.9% (57.6 to 77.6)	22,920,830 (19,072,462 to 26,493,109)	69.2% (58.5 to 77.7)	8,078,363 (6,538,457 to 9,611,319)	65.8% (54.7 to 74.9)	111,016,625 (91,925,356 to 127,719,012)	68.8% (57.6 to 77.5)
Tobacco smoke†	2,162,952 (1,783,646 to 2,618,514)	16.4% (13.7 to 18.9)	7,792,133 (6,367,762 to 9,468,308)	20.2% (16.8 to 23.6)	11,954,434 (9,556,942 to 14,486,472)	18.9% (15.5 to 22.2)	5,309,571 (4,305,287 to 6,378,888)	14.9% (12.3 to 17.6)	1,289,369 (1,019,469 to 1,579,744)	9.2% (7.4 to 11.0)	28,531,039 (23,461,345 to 34,072,552)	17.4% (14.4 to 20.3)
**Combined risk factors****												
**All risk factors**	12,194,107 (10,726,181 to 13,572,660)	79.1% (71.7 to 85.1)	32,710,791 (28,728,388 to 36,828,680)	84.3% (77.8 to 89.4)	50,568,879 (44,955,126 to 56,277,992)	83.9% (77.4 to 88.7)	28,712,503 (26,036,544 to 31,238,361)	85.9% (80.1 to 89.9)	10,660,334 (9,314,549 to 11,898,069)	85.6% (79.6 to 89.3)	134,968,222 (122,023,025 to 147,456,520)	83.6% (77.3 to 88.3)

Percentages and number of DALYs are not mutually exclusive. The sum of percentages and number of DALYs in the columns exceeds the totals for all risk factors combined because the effect of many of these risk factors are mediated partly or wholly through another risk factors. 0% represents very low numbers. *Air pollution cluster includes ambient PM2.5 pollution and household air pollution. †Tobacco smoke includes smoking and second-hand smoking. ‡Dietary risks cluster includes diet high in sodium, diet high in processed meat diet, high in red meat, diet high in sugar-sweetened beverages, diet low in polyunsaturated fatty acids, diet low in fruits, diet low in vegetables and diet low in whole grains. §Behavioural risks cluster includes smoking (including second-hand smoking), dietary risks (diet high in sodium, diet high in processed meat diet, high in red meat, diet high in sugar-sweetened beverages, diet low in polyunsaturated fatty acids, diet low in fruits, diet low in vegetables and diet low in whole grains), alcohol use and low physical activity. ¶Environmental risks cluster includes air pollution cluster, low ambient temperature, high ambient temperature and lead exposure. ¦Metabolic risks cluster includes high fasting plasma glucose, high LDL cholesterol, high systolic blood pressure, high body-mass index, and kidney dysfunction. **Age-standardised total percentage of DALYs due to all risk factors combined.

Appendix Table 8. Ischaemic stroke related DALYs (absolute numbers with 95% UI and age-standardized percentage with 95% UI) associated with risk factors and their clusters in 2021, by SDI, all ages, both sexes

	**High SDI**		**High-middle SDI**		**Middle SDI**		**Low-middle SDI**		**Low SDI**		**Globally**	
	Absolute number	Percentage (95% UI)	Absolute number	Percentage (95% UI)	Absolute number	Percentage (95% UI)	Absolute number	Percentage (95% UI)	Absolute number	Percentage (95% UI)	Absolute number	Percentage (95% UI)
**Air pollution and environmental risks**												
Ambient particulate matter pollution	957,915 (713,359 to 1,235,694)	11.1% (8.5 to 14.1)	3,745,151 (2,693,104 to 4,811,235)	17.7% (13.3 to 22.1)	5,027,046 (3,166,611 to 6,347,908)	20.9% (13.6 to 26.2)	1,898,290 (1,200,332 to 2,645,082)	15.3% (9.5 to 20.7)	360,991 (226,540 to 528,617)	9.2% (5.9 to 12.9)	11,998,572 (8,338,820 to 15,136,955)	16.9% (12.2 to 21.2)
High temperature	36,134 (-14,728 to 111,030)	0.5% (-0.1 to 1.4)	54,308 (-77,301 to 251,909)	0.3% (-0.4 to 1.2)	227,138 (35,621 to 532,391)	1.0% (0.1 to 2.2)	279,077 (75,517 to 552,406)	2.3% (0.6 to 4.5)	70,716 (28,785 to 131,080)	1.8% (0.7 to 3.3)	667,744 (75,229 to 1,553,718)	0.9% (0.1 to 2.2)
Household air pollution from solid fuels	4,980 (7 to 49,719)	0.1% (0.0 to 0.6)	318,745 (19,621 to 1,708,971)	1.5% (0.1 to 7.8)	1,714,060 (389,310 to 4,374,978)	7.2% (1.6 to 18.6)	2,760,043 (1,759,589 to 3,966,476)	22.7% (14.9 to 31.6)	1,491,483 (1,157,136 to 1,916,187)	38.2% (30.6 to 45.3)	6,293,712 (3,469,052 to 11,655,621)	8.8% (4.9 to 16.5)
Lead exposure	348,023 (-46,709 to 779,006)	3.8% (-0.5 to 8.7)	1,248,894 (-168,998 to 2,814,289)	5.8% (-0.8 to 13.0)	1,934,698 (-268,741 to 4,271,303)	8.0% (-1.1 to 18.1)	1,226,869 (-168,722 to 2,660,433)	10.1% (-1.4 to 22.3)	415,432 (-53,865 to 925,465)	10.8% (-1.5 to 23.7)	5,178,062 (-707,596 to 11,341,852)	7.3% (-1.0 to 16.2)
Low temperature	536,296 (452,630 to 628,640)	5.6% (4.9 to 6.5)	1,493,834 (1,275,373 to 1,741,885)	7.1% (6.1 to 8.0)	1,075,846 (902,137 to 1,302,146)	4.5% (4.0 to 5.2)	268,630 (173,911 to 391,435)	2.2% (1.4 to 3.1)	83,560 (59,273 to 115,381)	2.1% (1.6 to 2.9)	3,461,979 (2,977,105 to 4,031,398)	5.0% (4.3 to 5.7)
**Dietary risks**												
Alcohol use	769,236 (-113,561 to 1,836,778)	8.7% (-1.3 to 21.2)	1,430,923 (-206,794 to 3,579,962)	6.7% (-0.9 to 16.5)	1,217,916 (-210,417 to 3,045,300)	4.9% (-0.8 to 11.8)	319,307 (-49,580 to 850,861)	2.5% (-0.4 to 6.2)	128,259 (-22,158 to 337,906)	3.1% (-0.5 to 7.9)	3,870,519 (-603,265 to 9,620,467)	5.4% (-0.8 to 13.4)
Diet high in processed meat	157,788 (38,009 to 277,982)	1.9% (0.5 to 3.2)	172,956 (40,103 to 305,737)	0.8% (0.2 to 1.4)	56,943 (13,808 to 101,400)	0.2% (0.1 to 0.4)	36,633 (8,550 to 66,730)	0.3% (0.1 to 0.5)	11,643 (2,650 to 21,840)	0.3% (0.1 to 0.5)	436,528 (101,726 to 781,514)	0.6% (0.2 to 1.1)
Diet high in red meat	66,488 (-39,334 to 227,582)	1.1% (-0.7 to 3.4)	100,848 (-42,307 to 487,725)	0.5% (-0.2 to 2.4)	36,594 (-79,130 to 412,017)	0.1% (-0.3 to 1.5)	-59,250 (-172,791 to 33,715)	-0.4% (-1.2 to 0.2)	-27,361 (-79,234 to 13,987)	-0.5% (-1.4 to 0.3)	117,227 (-133,464 to 1,161,650)	0.2% (-0.2 to 1.6)
Diet high in sodium	691,534 (108,994 to 1,743,736)	7.9% (1.3 to 19.4)	2,568,298 (733,455 to 5,328,254)	11.9% (3.5 to 24.2)	2,993,402 (829,443 to 6,236,503)	11.9% (3.2 to 24.4)	794,064 (82,268 to 2,145,680)	6.4% (0.6 to 17.1)	191,925 (6,526 to 600,071)	4.9% (0.2 to 14.9)	7,247,272 (1,787,265 to 16,006,306)	10.1% (2.5 to 22.0)

Diet high in sugar-sweetened beverages	44,049 (21,332 to 69,942)	0.5% (0.3 to 0.8)	48,900 (24,176 to 76,852)	0.2% (0.1 to 0.4)	35,403 (17,400 to 56,087)	0.1% (0.1 to 0.2)	13,936 (6,441 to 22,399)	0.1% (0.1 to 0.2)	2,082 (927 to 3,488)	0.0% (0.0 to 0.1)	144,644 (70,033 to 228,901)	0.2% (0.1 to 0.3)
Diet low in fiber	86,821 (-5,043 to 203,465)	1.2% (0.0 to 2.7)	173,126 (-5,587 to 371,925)	0.9% (0.0 to 1.9)	368,479 (-15,874 to 757,829)	1.4% (-0.1 to 2.9)	257,867 (-11,930 to 533,055)	1.8% (-0.1 to 3.8)	60,364 (-2,835 to 126,319)	1.1% (0.0 to 2.4)	947,562 (-40,174 to 1,923,802)	1.3% (-0.1 to 2.7)
Diet low in fruits	125,009 (41,949 to 219,726)	1.8% (0.9 to 2.7)	341,278 (155,936 to 543,548)	1.6% (0.8 to 2.6)	645,892 (349,146 to 1,005,745)	2.4% (1.3 to 3.9)	713,890 (395,061 to 1,141,218)	5.0% (2.6 to 8.0)	237,270 (131,120 to 381,681)	4.6% (2.4 to 7.4)	2,064,516 (1,091,752 to 3,269,132)	2.8% (1.5 to 4.5)
Diet low in polyunsaturated fatty acids	1,430 (373 to 2,767)	0.0% (0.0 to 0.0)	4,874 (1,376 to 9,315)	0.0% (0.0 to 0.0)	6,274 (1,850 to 11,841)	0.0% (0.0 to 0.0)	3,598 (1,085 to 6,692)	0.0% (0.0 to 0.1)	1,321 (409 to 2,502)	0.0% (0.0 to 0.1)	17,512 (5,110 to 33,142)	0.0% (0.0 to 0.0)
Diet low in vegetables	72,252 (11,170 to 130,711)	1.0% (0.4 to 1.6)	102,211 (39,061 to 172,491)	0.5% (0.2 to 0.8)	320,334 (173,473 to 483,660)	1.2% (0.6 to 1.9)	412,018 (225,433 to 637,936)	2.9% (1.3 to 4.6)	284,074 (166,852 to 430,646)	5.6% (2.8 to 8.5)	1,192,174 (636,011 to 1,826,386)	1.6% (0.9 to 2.5)
Diet low in whole grains	305,307 (-296,593 to 932,256)	4.3% (-4.5 to 12.1)	998,664 (-993,408 to 2,783,931)	4.8% (-4.8 to 13.6)	1,041,418 (-1,058,613 to 2,777,922)	3.9% (-4.0 to 10.9)	551,268 (-573,076 to 1,522,328)	3.8% (-3.8 to 10.3)	225,727 (-233,963 to 624,915)	4.3% (-4.5 to 12.1)	3,125,286 (-3,174,925 to 8,780,802)	4.3% (-4.4 to 12.0)
**Physical activity**												
**Low physical activity**	369,729 (-29,176 to 824,958)	4.6% (0.9 to 8.9)	938,283 (163,766 to 1,887,964)	4.4% (0.7 to 8.9)	1,238,873 (407,237 to 2,276,382)	4.8% (1.1 to 9.4)	649,397 (236,683 to 1,132,656)	4.8% (1.4 to 8.7)	159,112 (60,352 to 277,054)	3.5% (1.0 to 6.4)	3,358,676 (907,212 to 6,300,659)	4.6% (1.1 to 8.9)
**Tobacco smoking**												
Secondhand smoke	194,492 (129,580 to 267,826)	2.5% (1.7 to 3.3)	856,804 (574,267 to 1,159,679)	4.1% (2.8 to 5.5)	1,076,398 (707,046 to 1,446,984)	4.4% (3.0 to 5.9)	498,076 (331,699 to 677,223)	3.9% (2.7 to 5.2)	113,660 (73,577 to 158,387)	2.6% (1.8 to 3.5)	2,741,835 (1,819,495 to 3,680,992)	3.9% (2.6 to 5.1)
Smoking	897,930 (729,645 to 1,108,880)	11.5% (9.7 to 13.4)	2,890,289 (2,370,698 to 3,517,453)	13.5% (11.4 to 15.8)	3,216,256 (2,560,206 to 3,910,313)	12.5% (10.4 to 15.0)	1,247,267 (995,320 to 1,566,962)	9.3% (7.7 to 11.2)	252,216 (197,524 to 325,059)	5.5% (4.5 to 6.7)	8,510,889 (7,039,201 to 10,283,725)	11.7% (9.9 to 13.8)
**Physiological factors**												
High body-mass index	661,436 (96,984 to 1,288,502)	8.5% (1.3 to 16.0)	1,536,338 (224,292 to 3,026,100)	7.3% (1.1 to 14.0)	1,345,497 (196,395 to 2,623,779)	5.2% (0.8 to 9.9)	714,612 (107,665 to 1,360,757)	5.1% (0.8 to 9.7)	175,786 (23,294 to 325,993)	3.6% (0.5 to 6.7)	4,439,186 (649,030 to 8,647,485)	6.2% (0.9 to 11.7)
High fasting plasma glucose	1,836,729 (1,418,883 to 2,258,219)	19.8% (15.9 to 24.0)	3,628,252 (2,827,169 to 4,540,170)	17.0% (13.5 to 21.0)	4,073,160 (3,138,040 to 5,120,243)	17.1% (13.5 to 21.3)	2,226,720 (1,661,985 to 2,814,897)	18.6% (14.7 to 22.9)	592,442 (436,513 to 779,610)	15.8% (12.6 to 19.6)	12,371,434 (9,587,506 to 15,382,662)	17.6% (13.9 to 21.6)

High LDL cholesterol	2,663,216 (900,957 to 4,379,680)	31.7% (11.2 to 50.7)	6,609,150 (2,320,767 to 10,837,819)	31.3% (10.9 to 50.5)	7,148,661 (2,541,446 to 11,776,116)	28.7% (9.7 to 47.0)	3,460,220 (1,236,731 to 5,729,942)	26.4% (8.9 to 43.8)	1,074,118 (379,636 to 1,799,295)	24.3% (8.0 to 41.3)	20,977,424 (7,409,044 to 34,664,410)	29.4% (10.1 to 47.8)
High systolic blood pressure	4,846,313 (3,528,041 to 6,025,938)	53.2% (39.1 to 64.3)	12,653,469 (9,472,289 to 15,556,384)	59.5% (44.9 to 70.8)	14,030,865 (10,449,419 to 17,558,598)	58.2% (43.9 to 69.8)	7,276,694 (5,559,566 to 8,974,222)	59.2% (44.6 to 70.2)	2,210,444 (1,629,250 to 2,846,478)	55.9% (41.9 to 66.7)	41,060,707 (31,145,960 to 50,200,697)	58.0% (43.9 to 69.1)
Kidney dysfunction	836,346 (541,350 to 1,164,318)	9.0% (6.1 to 12.0)	1,890,856 (1,312,268 to 2,525,713)	8.9% (6.2 to 11.7)	2,421,873 (1,698,633 to 3,171,060)	9.9% (7.0 to 12.9)	1,385,705 (993,011 to 1,817,496)	11.2% (8.0 to 14.5)	415,626 (292,863 to 575,965)	10.4% (7.4 to 13.6)	6,956,929 (4,935,530 to 9,161,374)	9.8% (6.9 to 12.8)
**Cluster of risk factors**												
Air pollution*	963,040 (722,057 to 1,240,918)	11.1% (8.6 to 14.2)	4,064,822 (3,025,087 to 5,315,163)	19.2% (15.0 to 24.6)	6,742,528 (4,974,277 to 8,716,821)	28.1% (21.7 to 35.8)	4,658,817 (3,697,821 to 5,706,834)	38.0% (31.0 to 45.3)	1,852,561 (1,460,592 to 2,348,454)	47.4% (39.6 to 55.0)	18,295,352 (14,324,971 to 22,541,397)	25.8% (20.6 to 32.0)
Behavioral risks§	2,988,671 (1,789,983 to 4,174,028)	36.0% (22.9 to 48.2)	8,158,822 (5,446,675 to 10,821,102)	38.3% (26.0 to 49.5)	9,372,560 (6,305,949 to 12,292,997)	37.1% (25.6 to 47.7)	4,215,931 (2,811,287 to 5,753,715)	31.8% (21.5 to 42.5)	1,282,504 (761,755 to 1,866,883)	28.4% (17.3 to 40.4)	26,044,034 (17,587,693 to 34,270,496)	36.2% (24.4 to 47.0)
Dietary risks‡	1,353,281 (283,219 to 2,431,364)	16.7% (2.9 to 29.4)	4,018,665 (1,165,427 to 6,833,028)	18.9% (5.3 to 32.4)	4,762,279 (1,559,088 to 7,979,041)	18.6% (5.7 to 31.4)	2,245,393 (650,464 to 3,795,548)	16.6% (4.8 to 28.7)	822,958 (255,667 to 1,398,851)	17.5% (5.5 to 30.0)	13,215,812 (3,969,750 to 22,309,700)	18.3% (5.6 to 31.2)
Environmental/occupational risks¶	1,749,194 (1,302,428 to 2,224,428)	19.5% (14.7 to 24.1)	6,217,631 (4,738,683 to 7,831,377)	29.3% (22.9 to 36.0)	8,936,331 (6,644,467 to 11,267,194)	37.3% (28.6 to 46.1)	5,693,202 (4,427,908 to 7,023,208)	46.5% (36.9 to 55.5)	2,142,316 (1,696,721 to 2,726,380)	54.8% (45.3 to 63.2)	24,758,794 (19,119,740 to 30,291,198)	34.9% (27.2 to 42.2)
Metabolic risks¦	6,897,434 (5,714,152 to 8,031,713)	76.8% (65.7 to 85.3)	16,716,268 (14,157,343 to 19,044,959)	78.9% (67.8 to 87.3)	18,734,000 (15,652,576 to 21,647,164)	77.6% (66.7 to 86.1)	9,631,680 (8,114,228 to 11,263,335)	78.0% (68.0 to 86.1)	2,966,947 (2,407,446 to 3,754,048)	74.5% (63.8 to 83.2)	55,003,220 (46,803,671 to 62,779,570)	77.8% (66.9 to 86.0)
Tobacco smoke†	1,066,638 (852,103 to 1,325,948)	13.6% (11.1 to 16.0)	3,626,881 (2,911,727 to 4,506,096)	17.0% (14.0 to 20.2)	4,164,719 (3,262,646 to 5,162,174)	16.4% (13.2 to 19.6)	1,694,893 (1,309,898 to 2,152,372)	12.9% (10.3 to 15.6)	356,516 (265,951 to 466,857)	8.0% (6.2 to 9.8)	10,918,708 (8,756,101 to 13,397,298)	15.1% (12.3 to 18.0)
**Combined risk factors****												
**All risk factors**	7,636,185 (6,612,329 to 8,640,185)	85.1% (77.1 to 90.9)	18,678,284 (16,611,711 to 20,847,596)	88.2% (81.4 to 93.0)	21,313,687 (18,900,345 to 23,830,054)	88.5% (82.5 to 93.0)	11,045,784 (9,782,699 to 12,687,153)	89.7% (84.2 to 93.5)	3,529,650 (3,003,828 to 4,360,220)	89.1% (83.4 to 92.9)	62,266,248 (55,752,316 to 68,687,310)	88.1% (81.8 to 92.6)

Percentages and number of DALYs are not mutually exclusive. The sum of percentages and number of DALYs in the columns exceeds the totals for all risk factors combined because of overlap between various risk factors. 0% represents very low numbers. *Air pollution cluster includes ambient PM2.5 pollution and household air pollution. †Tobacco smoke includes smoking and second-hand smoking. ‡Dietary risks cluster includes diet high in sodium, diet high in processed meat diet, high in red meat, diet high in sugar-sweetened beverages, diet low in polyunsaturated fatty acids, diet low in fruits, diet low in vegetables, diet low in whole grains, and alcohol use. §Behavioural risks cluster includes smoking (including second-hand smoking), dietary risks (diet high in sodium, diet high in processed meat diet, high in red meat, diet high in sugar-sweetened beverages, diet low in polyunsaturated fatty acids, diet low in fruits, diet low in vegetables, diet low in whole grains, and alcohol use), and low physical activity. ¶Environmental/occupational risks cluster includes air pollution cluster, suboptimal ambient temperature and lead exposure. ¦Metabolic risks cluster includes high fasting plasma glucose, high LDL cholesterol, high systolic blood pressure, high body-mass index, and kidney dysfunction. **Age-standardised total percentage of DALYs due to all risk factors combined.

Appendix Table 9. Intracerebral haemorrhage related DALYs (absolute numbers with 95% UI and age-standardized percentage with 95% UI) associated with risk factors and their clusters in 2021, by SDI, all ages, both sexes

	High SDI		High-middle SDI		Middle SDI		Low-middle SDI		Low SDI		Globally	
	Absolute number	Percentage (95% UI)	Absolute number	Percentage (95% UI)	Absolute number	Percentage (95% UI)	Absolute number	Percentage (95% UI)	Absolute number	Percentage (95% UI)	Absolute number	Percentage (95% UI)
**Air pollution and environmental risks**												
Ambient particulate matter pollution	534,883 (411,512 to 685,414)	12.0% (9.3 to 15.1)	3,141,399 (2,205,313 to 4,021,847)	19.9% (14.8 to 24.4)	6,524,013 (4,035,055 to 8,464,189)	20.3% (12.9 to 25.4)	2,424,332 (1,338,771 to 3,538,428)	12.9% (7.3 to 18.3)	617,961 (381,693 to 920,893)	8.0% (5.1 to 11.0)	13,249,506 (8,726,215 to 17,131,012)	16.6% (11.1 to 21.0)
High temperature	34,617 (-9,515 to 97,513)	0.9% (-0.1 to 2.4)	74,118 (-52,314 to 286,713)	0.5% (-0.3 to 1.8)	311,443 (69,027 to 714,431)	1.0% (0.2 to 2.2)	434,475 (138,427 to 846,702)	2.3% (0.7 to 4.4)	151,977 (63,006 to 277,273)	1.8% (0.8 to 3.4)	1,007,058 (229,134 to 2,241,702)	1.3% (0.3 to 2.8)
Household air pollution from solid fuels	2,031 (2 to 19,357)	0.0% (0.0 to 0.4)	283,899 (16,072 to 1,513,196)	1.8% (0.1 to 9.4)	2,564,981 (646,161 to 6,230,576)	8.0% (1.9 to 19.8)	4,861,676 (3,112,917 to 6,847,513)	26.1% (17.0 to 35.8)	3,039,240 (2,264,098 to 3,763,928)	39.3% (31.8 to 46.3)	10,761,864 (6,305,255 to 17,884,173)	13.4% (7.8 to 22.4)
Lead exposure	177,092 (-22,574 to 404,242)	3.6% (-0.5 to 8.4)	1,013,983 (-127,712 to 2,297,335)	6.3% (-0.8 to 14.1)	2,488,706 (-315,794 to 5,482,672)	7.7% (-1.0 to 17.4)	1,740,587 (-232,303 to 3,835,116)	9.5% (-1.3 to 20.9)	747,156 (-96,909 to 1,618,833)	10.0% (-1.4 to 22.1)	6,171,499 (-795,984 to 13,644,822)	7.7% (-1.0 to 17.1)
Low temperature	329,534 (287,413 to 377,422)	6.9% (6.1 to 7.8)	1,131,354 (947,913 to 1,340,321)	7.2% (6.3 to 8.2)	1,568,674 (1,300,372 to 1,905,991)	5.0% (4.4 to 5.7)	450,918 (276,551 to 676,165)	2.4% (1.5 to 3.5)	189,499 (139,133 to 255,496)	2.3% (1.7 to 3.1)	3,672,031 (3,075,164 to 4,442,831)	4.6% (4.0 to 5.4)
**Dietary risks**												
Alcohol use	438,536 (12,784 to 912,187)	9.5% (0.3 to 19.1)	1,210,387 (26,460 to 2,531,951)	7.7% (0.2 to 15.9)	1,991,145 (54,405 to 4,021,890)	6.0% (0.2 to 12.2)	641,162 (19,938 to 1,384,313)	3.3% (0.1 to 7.2)	281,770 (7,479 to 625,691)	3.6% (0.1 to 7.6)	4,566,754 (135,897 to 9,388,359)	5.7% (0.2 to 11.7)
Diet high in red meat	-405,285 (-1,635,599 to 566,750)	-10.1% (-41.5 to 14.2)	-1,441,661 (-6,042,605 to 1,982,636)	-9.5% (-39.2 to 13.2)	-2,190,405 (-9,195,900 to 3,038,357)	-6.5% (-26.4 to 9.3)	-336,177 (-1,336,990 to 545,639)	-1.6% (-6.8 to 2.6)	-147,766 (-586,761 to 261,306)	-1.6% (-6.3 to 2.8)	-4,524,366 (-18,840,833 to 6,288,533)	-5.6% (-22.8 to 8.1)
Diet high in sodium	393,791 (70,801 to 953,560)	8.4% (1.6 to 19.8)	2,410,132 (839,597 to 4,682,428)	15.1% (5.3 to 28.4)	4,536,417 (1,402,083 to 9,161,438)	13.6% (4.1 to 26.8)	1,452,681 (197,911 to 3,668,597)	7.8% (1.0 to 19.6)	379,830 (13,787 to 1,132,392)	5.2% (0.2 to 15.5)	9,178,888 (2,540,722 to 19,500,759)	11.4% (3.1 to 23.9)
Diet low in fiber	122,447 (-35,139 to 270,484)	2.9% (-0.9 to 6.3)	313,248 (-84,761 to 688,705)	2.1% (-0.6 to 4.6)	1,142,068 (-328,957 to 2,426,626)	3.4% (-1.0 to 7.1)	902,637 (-265,684 to 1,916,467)	4.5% (-1.4 to 9.6)	214,289 (-62,488 to 447,839)	2.4% (-0.7 to 5.1)	2,697,045 (-775,943 to 5,613,656)	3.4% (-1.0 to 7.0)

Diet low in fruits	278,549 (-18,862 to 529,576)	6.7% (-0.5 to 12.5)	932,726 (-64,129 to 1,859,099)	6.1% (-0.4 to 11.6)	2,393,953 (-165,407 to 4,598,227)	7.1% (-0.5 to 13.4)	2,123,256 (-171,115 to 3,895,848)	10.7% (-0.8 to 19.8)	860,446 (-71,866 to 1,574,449)	10.0% (-0.7 to 18.7)	6,593,536 (-492,868 to 12,308,073)	8.2% (-0.6 to 15.2)
Diet low in vegetables	5,849 (-543 to 12,164)	0.1% (0.0 to 0.3)	4,906 (-532 to 9,541)	0.0% (0.0 to 0.1)	185,027 (-20,003 to 368,486)	0.5% (-0.1 to 1.1)	461,336 (-49,493 to 881,831)	2.4% (-0.2 to 4.5)	534,149 (-57,088 to 1,013,436)	6.2% (-0.6 to 11.7)	1,193,018 (-128,584 to 2,253,219)	1.5% (-0.2 to 2.8)
**Tobacco smoking**												
Secondhand smoke	135,526 (92,065 to 178,235)	3.3% (2.3 to 4.4)	796,961 (536,878 to 1,060,840)	5.2% (3.6 to 6.9)	1,647,191 (1,125,602 to 2,188,722)	5.0% (3.5 to 6.7)	886,256 (594,808 to 1,193,890)	4.6% (3.2 to 6.1)	258,071 (173,306 to 355,477)	3.0% (2.1 to 4.0)	3,726,863 (2,545,045 to 4,925,845)	4.7% (3.2 to 6.1)
Smoking	701,148 (599,082 to 820,851)	17.0% (14.7 to 19.2)	3,087,892 (2,546,286 to 3,773,528)	19.9% (17.1 to 22.7)	5,635,090 (4,648,124 to 6,775,969)	16.7% (14.2 to 19.2)	2,405,728 (2,001,920 to 2,833,678)	12.2% (10.5 to 14.1)	604,885 (487,618 to 749,404)	7.0% (6.0 to 8.2)	12,444,021 (10,494,380 to 14,653,810)	15.4% (13.2 to 17.7)
**Physiological factors**												
High body-mass index	280,223 (-8,425 to 613,667)	7.1% (-0.2 to 15.5)	687,692 (-1,855 to 1,606,733)	4.5% (0.0 to 10.5)	1,018,992 (1,625 to 2,374,991)	3.0% (0.0 to 7.1)	531,367 (464 to 1,183,447)	2.6% (0.0 to 5.9)	197,154 (-1,941 to 476,079)	2.1% (0.0 to 5.1)	2,718,611 (6,442 to 6,343,090)	3.4% (0.0 to 7.9)
High fasting plasma glucose	312,394 (170,994 to 454,719)	6.4% (3.4 to 9.4)	888,166 (459,564 to 1,334,330)	5.6% (3.0 to 8.3)	1,628,808 (842,993 to 2,470,394)	5.1% (2.7 to 7.5)	947,477 (516,686 to 1,400,490)	5.2% (2.8 to 7.7)	319,043 (176,711 to 479,250)	4.3% (2.3 to 6.5)	4,099,140 (2,164,556 to 6,091,184)	5.1% (2.7 to 7.6)
High systolic blood pressure	2,472,728 (1,814,853 to 3,042,277)	51.5% (37.3 to 62.6)	9,132,227 (6,542,038 to 11,506,468)	57.7% (43.3 to 69.2)	18,598,692 (13,554,466 to 23,054,781)	57.4% (42.6 to 69.0)	10,728,195 (8,090,325 to 13,118,177)	57.7% (43.3 to 68.8)	4,245,615 (3,096,168 to 5,429,441)	55.2% (40.8 to 66.2)	45,214,435 (33,714,362 to 55,388,484)	56.4% (41.8 to 67.7)
Kidney dysfunction	423,409 (304,630 to 549,581)	8.7% (6.5 to 10.9)	1,360,064 (987,449 to 1,776,768)	8.6% (6.3 to 10.8)	3,311,822 (2,470,835 to 4,215,728)	10.1% (7.5 to 12.6)	2,143,429 (1,592,633 to 2,710,807)	11.5% (8.8 to 14.3)	807,388 (584,386 to 1,064,786)	10.5% (8.1 to 13.2)	8,052,724 (5,962,331 to 10,179,526)	10.0% (7.6 to 12.5)
**Cluster of risk factors**												
Air pollution*	536,988 (414,822 to 686,161)	12.0% (9.3 to 15.3)	3,426,118 (2,588,097 to 4,501,338)	21.7% (17.2 to 27.6)	9,091,147 (6,733,296 to 11,975,977)	28.3% (21.6 to 35.9)	7,286,755 (5,536,696 to 8,863,879)	39.0% (31.4 to 46.3)	3,657,378 (2,760,493 to 4,445,982)	47.3% (39.4 to 54.6)	24,015,342 (18,414,608 to 29,838,879)	30.0% (23.8 to 36.7)
Behavioral risks§	1,398,908 (712,148 to 2,055,941)	31.6% (16.7 to 45.7)	5,995,103 (3,562,359 to 8,305,164)	38.2% (24.2 to 51.2)	12,097,704 (8,341,704 to 16,137,003)	36.4% (25.3 to 47.4)	6,438,729 (4,306,781 to 8,429,361)	33.3% (22.4 to 43.8)	2,388,467 (1,351,233 to 3,387,045)	28.9% (16.6 to 40.5)	28,340,641 (20,878,908 to 36,942,020)	35.2% (25.9 to 45.1)
Dietary risks‡	332,808 (-474,733	6.8% (-11.9 to 23.7)	2,157,540 (-666,475 to 5,059,864)	13.4% (-4.6 to 30.8)	5,185,973 (937,869 to 9,776,052)	15.6% (2.7 to 29.1)	3,523,238 (1,161,867	18.3% (6.0 to 31.1)	1,536,227 (399,518	18.6% (4.8 to 32.0)	12,745,155 (4,643,556	15.8% (5.8 to 28.1)

	to 1,091,419)						to 5,762,480)		to 2,556,036)		to 22,891,812)	
Environmental/occupational risks¶	994,634 (766,469 to 1,230,789)	21.6% (16.8 to 26.2)	5,066,712 (3,784,268 to 6,483,903)	32.1% (25.5 to 39.3)	12,032,894 (8,884,427 to 15,278,541)	37.5% (28.8 to 46.3)	8,785,374 (6,689,607 to 10,742,096)	47.1% (37.7 to 55.5)	4,210,419 (3,308,507 to 5,145,073)	54.3% (45.4 to 62.4)	31,111,635 (24,063,013 to 37,913,167)	38.9% (30.8 to 46.7)
Metabolic risks¦	2,837,426 (2,266,752 to 3,338,982)	59.4% (47.4 to 68.7)	10,076,340 (7,775,936 to 12,315,104)	63.7% (51.5 to 73.6)	20,649,243 (16,326,495 to 24,785,072)	63.7% (51.7 to 73.4)	11,991,099 (9,720,051 to 14,153,927)	64.5% (52.5 to 73.6)	4,736,676 (3,719,675 to 5,862,737)	61.5% (49.3 to 71.2)	50,331,952 (40,288,092 to 59,299,298)	62.8% (50.8 to 72.4)
Tobacco smoke†	811,048 (676,295 to 961,295)	19.6% (16.6 to 22.6)	3,736,286 (3,007,190 to 4,582,985)	24.1% (20.2 to 27.9)	7,036,927 (5,621,087 to 8,576,795)	21.0% (17.4 to 24.5)	3,183,252 (2,559,029 to 3,822,007)	16.2% (13.4 to 19.0)	838,638 (647,817 to 1,056,490)	9.8% (7.9 to 11.7)	15,617,850 (12,811,183 to 18,749,349)	19.4% (16.1 to 22.6)
**Combined risk factors****												
**All risk factors**	3,508,931 (3,086,515 to 3,939,112)	74.7% (65.8 to 81.7)	12,683,354 (10,692,840 to 14,683,857)	80.6% (72.9 to 86.7)	26,417,828 (22,968,172 to 30,043,112)	81.6% (74.9 to 87.2)	15,772,621 (13,857,635 to 17,589,097)	84.2% (77.7 to 88.5)	6,547,152 (5,535,813 to 7,609,072)	84.0% (77.8 to 88.0)	64,981,778 (57,891,339 to 71,720,874)	81.2% (74.7 to 86.3)

Percentages and number of DALYs are not mutually exclusive. The sum of percentages and number of DALYs in the columns exceeds the totals for all risk factors combined because of overlap between various risk factors. 0% represents very low numbers. *Air pollution cluster includes ambient PM2.5 pollution and household air pollution. †Tobacco smoke includes smoking and second-hand smoking. ‡Dietary risks cluster includes diet high in sodium, diet high in red meat, diet low in fiber, diet low in fruits, diet low in vegetables, and alcohol use. §Behavioural risks cluster includes smoking (including second-hand smoking) and dietary risks (diet high in sodium, diet high in red meat, diet low in fiber, diet low in fruits, diet low in vegetables, and alcohol use). ¶Environmental risks cluster includes air pollution cluster, suboptimal ambient temperature and lead exposure. ¦Metabolic risks cluster includes high fasting plasma glucose, high systolic blood pressure, high body-mass index, and kidney dysfunction. **Age-standardised total percentage of DALYs due to all risk factors combined.

Appendix Table 10. Subarachnoid haemorrhage related DALYs (absolute numbers with 95% UI and age-standardized percentages with 95% UI) associated with risk factors and their clusters in 2021, by SDI, all ages, both sexes

	**High SDI**		**High-middle SDI**		**Middle SDI**		**Low-middle SDI**		**Low SDI**		**Globally**	
	Absolute number	Percentage (95% UI)	Absolute number	Percentage (95% UI)	Absolute number	Percentage (95% UI)	Absolute number	Percentage (95% UI)	Absolute number	Percentage (95% UI)	Absolute number	Percentage (95% UI)
**Air pollution and environmental risks**												
Ambient particulate matter pollution	125,494 (87,242 to 168,096)	7.8% (5.6 to 10.3)	286,624 (208,435 to 379,812)	15.3% (11.4 to 19.2)	749,039 (493,469 to 974,189)	19.1% (12.8 to 23.9)	305,709 (166,503 to 471,028)	12.3% (7.1 to 17.5)	63,477 (29,787 to 119,538)	8.6% (5.4 to 12.1)	1,531,353 (1,031,890 to 1,965,982)	14.2% (9.8 to 18.0)
High temperature	5,298 (-3,822 to 17,739)	0.4% (-0.2 to 1.2)	4,890 (-6,040 to 21,738)	0.3% (-0.3 to 1.2)	35,834 (10,612 to 78,022)	0.9% (0.3 to 2.0)	59,877 (15,829 to 121,097)	2.3% (0.7 to 4.5)	14,727 (3,642 to 34,418)	1.8% (0.5 to 3.4)	120,677 (22,618 to 265,456)	1.1% (0.2 to 2.5)
Household air pollution from solid fuels	410 (1 to 3,894)	0.0% (0.0 to 0.3)	24,241 (1,524 to 124,460)	1.3% (0.1 to 6.5)	240,958 (55,738 to 646,043)	6.1% (1.4 to 16.3)	584,564 (343,136 to 915,081)	23.6% (15.4 to 32.5)	268,229 (145,685 to 514,561)	35.8% (28.5 to 42.9)	1,119,633 (575,795 to 1,959,215)	10.3% (5.5 to 17.4)
Lead exposure	48,907 (-6,313 to 112,052)	2.8% (-0.3 to 6.4)	90,839 (-11,144 to 204,277)	4.7% (-0.6 to 10.6)	254,321 (-33,663 to 564,154)	6.5% (-0.8 to 14.6)	210,178 (-28,476 to 481,557)	8.7% (-1.2 to 19.4)	68,742 (-7,832 to 180,547)	9.7% (-1.4 to 21.5)	673,505 (-89,475 to 1,494,563)	6.2% (-0.8 to 13.7)
Low temperature	103,750 (91,776 to 118,461)	6.5% (5.8 to 7.4)	128,179 (107,298 to 153,547)	7.0% (6.0 to 7.9)	166,568 (124,182 to 206,831)	4.3% (3.6 to 5.1)	57,558 (29,889 to 96,410)	2.3% (1.3 to 3.5)	20,468 (10,131 to 37,103)	2.5% (1.7 to 3.4)	476,875 (392,726 to 576,639)	4.5% (3.8 to 5.3)
**Dietary risks**												
Diet high in red meat	-165,577 (-671,612 to 226,573)	-12.0% (-49.6 to 16.2)	-193,014 (-792,361 to 263,687)	-11.0% (-44.6 to 14.7)	-309,323 (-1,244,506 to 437,715)	-7.6% (-31.1 to 10.6)	-68,616 (-276,651 to 99,204)	-2.6% (-10.7 to 3.9)	-15,923 (-73,973 to 24,548)	-1.8% (-7.1 to 3.0)	-753,114 (-3,100,659 to 1,055,841)	-7.0% (-29.0 to 10.0)
Diet high in sodium	109,705 (12,517 to 286,780)	6.6% (0.8 to 17.2)	208,415 (59,552 to 433,739)	10.9% (3.2 to 22.4)	462,609 (126,245 to 991,938)	11.5% (3.3 to 23.6)	161,159 (15,410 to 478,437)	6.5% (0.7 to 17.5)	33,367 (925 to 131,849)	4.6% (0.2 to 14.1)	975,985 (223,809 to 2,243,550)	8.9% (2.0 to 19.8)
Diet low in fiber	55,023 (-15,791 to 116,960)	3.9% (-1.1 to 8.3)	49,357 (-13,909 to 106,398)	2.9% (-0.8 to 6.1)	152,556 (-45,070 to 320,831)	3.8% (-1.2 to 7.9)	142,388 (-43,987 to 310,911)	5.4% (-1.6 to 11.1)	29,178 (-8,161 to 69,746)	3.4% (-1.0 to 7.2)	428,874 (-127,174 to 912,456)	4.0% (-1.2 to 8.3)
Diet low in fruits	112,775 (-8,146 to 210,193)	7.8% (-0.6 to 14.3)	124,152 (-9,204 to 232,794)	7.0% (-0.5 to 12.8)	318,013 (-23,082 to 584,754)	7.9% (-0.6 to 14.4)	318,838 (-22,884 to 604,535)	12.2% (-1.0 to 21.9)	95,119 (-5,588 to 220,234)	11.5% (-0.9 to 21.0)	969,652 (-68,958 to 1,820,185)	9.0% (-0.7 to 16.4)
Diet low in vegetables	2,354 (-234 to 4,552)	0.2% (0.0 to 0.3)	838 (-102 to 1,590)	0.0% (0.0 to 0.1)	28,114 (-3,203 to 55,595)	0.7% (-0.1 to 1.4)	72,456 (-7,713 to 151,762)	2.7% (-0.3 to 5.4)	51,192 (-5,508 to 124,551)	6.0% (-0.6 to 11.5)	155,247 (-16,288 to 325,250)	1.4% (-0.2 to 3.0)

**Tobacco smoking**												
Secondhand smoke	49,419 (33,452 to 66,118)	3.5% (2.4 to 4.7)	96,165 (65,961 to 130,473)	5.3% (3.7 to 7.0)	199,569 (132,513 to 270,365)	5.0% (3.4 to 6.6)	126,588 (80,715 to 182,857)	4.9% (3.4 to 6.5)	29,384 (15,141 to 52,320)	3.7% (2.5 to 5.0)	501,565 (336,169 to 673,382)	4.7% (3.2 to 6.2)
Smoking	245,635 (212,588 to 287,201)	17.1% (14.8 to 19.4)	351,421 (291,812 to 441,904)	19.3% (16.7 to 21.8)	579,836 (426,309 to 719,403)	14.2% (11.7 to 16.6)	321,038 (216,335 to 467,182)	12.4% (9.7 to 15.1)	67,990 (33,613 to 144,408)	8.4% (6.4 to 10.6)	1,567,375 (1,277,562 to 1,897,374)	14.4% (12.4 to 16.5)
**Physiological factors**												
High body-mass index	107,543 (-2,790 to 233,382)	7.7% (-0.2 to 16.7)	122,741 (-2,951 to 271,564)	6.8% (-0.2 to 14.9)	187,064 (73 to 418,397)	4.6% (0.0 to 10.5)	91,713 (428 to 212,130)	3.5% (0.0 to 7.8)	19,974 (-332 to 56,983)	2.3% (0.0 to 5.7)	529,692 (418 to 1,191,346)	4.9% (0.0 to 11.0)
High systolic blood pressure	816,978 (600,667 to 1,010,527)	48.5% (35.0 to 59.3)	1,035,952 (765,472 to 1,286,443)	54.8% (39.9 to 66.0)	2,069,489 (1,484,090 to 2,623,753)	52.5% (38.1 to 63.8)	1,280,265 (868,477 to 1,805,017)	52.3% (38.6 to 63.2)	371,035 (191,430 to 762,760)	50.2% (36.3 to 61.4)	5,578,919 (4,049,218 to 7,062,420)	51.6% (37.9 to 62.6)
**Cluster of risk factors**												
Air pollution*	125,918 (88,077 to 168,177)	7.8% (5.6 to 10.4)	310,948 (230,501 to 418,467)	16.5% (12.7 to 21.5)	990,300 (704,510 to 1,312,211)	25.2% (19.2 to 31.9)	890,333 (632,110 to 1,232,807)	35.9% (28.9 to 43.0)	331,733 (182,586 to 617,303)	44.4% (37.3 to 51.6)	2,651,473 (1,994,098 to 3,505,549)	24.5% (19.4 to 30.5)
Behavioral risks§	355,971 (109,003 to 580,500)	23.6% (6.8 to 37.8)	555,795 (281,486 to 829,009)	30.0% (14.5 to 43.4)	1,172,223 (720,219 to 1,676,872)	29.1% (18.0 to 40.4)	799,421 (499,981 to 1,216,274)	31.2% (21.7 to 41.3)	227,150 (97,393 to 454,821)	28.3% (16.0 to 39.6)	3,113,331 (2,079,102 to 4,320,276)	28.7% (19.4 to 38.8)
Dietary risks‡	85,301 (-225,547 to 353,452)	4.6% (-17.1 to 22.2)	174,341 (-188,990 to 503,993)	9.0% (-10.3 to 26.4)	540,250 (17,866 to 1,108,851)	13.5% (0.4 to 26.7)	462,364 (166,451 to 832,640)	18.0% (6.4 to 30.0)	154,704 (34,411 to 336,478)	19.2% (4.8 to 32.0)	1,418,137 (258,578 to 2,798,625)	13.0% (2.4 to 24.7)
Environmental/occupational risks¶	267,281 (201,242 to 340,187)	16.5% (12.6 to 20.4)	487,894 (367,313 to 640,019)	26.0% (20.4 to 32.2)	1,307,004 (935,527 to 1,692,193)	33.4% (25.4 to 41.2)	1,083,355 (762,346 to 1,482,217)	43.7% (34.6 to 52.1)	386,525 (216,571 to 727,654)	51.5% (42.4 to 59.2)	3,534,989 (2,644,789 to 4,586,447)	32.7% (25.8 to 39.5)
Metabolic risks¦	840,047 (614,947 to 1,030,083)	50.2% (36.3 to 61.3)	1,056,932 (774,901 to 1,313,887)	56.0% (41.6 to 66.6)	2,106,360 (1,513,791 to 2,661,510)	53.4% (39.4 to 64.9)	1,298,051 (881,822 to 1,831,893)	53.0% (39.2 to 63.6)	374,739 (194,457 to 767,376)	50.6% (36.8 to 61.7)	5,681,453 (4,152,446 to 7,122,971)	52.5% (38.9 to 63.5)
Tobacco smoke†	285,267 (241,085 to 337,713)	19.9% (16.8 to 22.9)	428,967 (353,527 to 533,774)	23.6% (19.8 to 27.1)	752,788 (560,456 to 936,159)	18.6% (15.3 to 21.8)	431,425 (297,446 to 611,958)	16.7% (13.4 to 20.1)	94,215 (48,649 to 188,627)	11.6% (8.9 to 14.4)	1,994,481 (1,584,808 to 2,450,625)	18.4% (15.3 to 21.5)
**Combined risk factors****												

**All risk factors**	1,048,991 (865,377 to 1,210,060)	64.3% (52.3 to 74.0)	1,349,153 (1,146,338 to 1,596,630)	72.2% (62.7 to 79.9)	2,837,363 (2,344,089 to 3,322,794)	72.0% (63.4 to 79.3)	1,894,098 (1,448,235 to 2,466,214)	76.3% (68.3 to 81.8)	583,532 (330,280 to 1,103,910)	77.3% (70.2 to 82.7)	7,720,196 (6,518,904 to 9,114,453)	71.5% (63.2 to 78.2)

Percentages and number of DALYs are not mutually exclusive. The sum of percentages and number of DALYs in the columns exceeds the totals for all risk factors combined because of overlap between various risk factors. 0% represents very low numbers. *Air pollution cluster includes ambient PM2.5 pollution and household air pollution. †Tobacco smoke includes smoking and second-hand smoking. ‡Dietary risks cluster includes diet high in sodium, diet low in fruits, diet low in vegetables, diet high in red meat, and diet low in fiber. §Behavioural risks cluster includes smoking (including second-hand smoking) and dietary risks (diet high in sodium, diet low in fruits, diet low in vegetables, diet high in red meat, and diet low in fiber). ¶Environmental risks cluster includes air pollution cluster, suboptimal ambient temperature and lead exposure. ¦Metabolic risks cluster includes high systolic blood pressure and high body-mass index. **Age-standardised total percentage of DALYs due to all risk factors combined.

Appendix Table 11. Age-standardized percent of DALYs (with 95% uncertainty intervals) due to stroke associated with individual risk factors by 7 GBD super-region, 21 GBD region and country for both sexes combined in 2021.

Country/region	Alcohol use	Ambient particulate matter pollution	Diet high in processed meat	Diet high in red meat	Diet high in sodium	Diet high in sugar-sweetened beverages	Diet low in fiber	Diet low in fruits	Diet low in polyunsaturated fatty acids	Diet low in vegetables	Diet low in whole grains	High body-mass index	High fasting plasma glucose	High LDL cholesterol
**GBD super-regions in alphabetical order**														
**Central Europe, Eastern Europe, and Central Asia**	7.5% (1.2 to 15.8)	10.8% (7.8 to 14.5)	0.9% (0.2 to 1.6)	-3.6% (-15.3 to 5.2)	8.6% (1.5 to 20.3)	0.2% (0.1 to 0.3)	1.7% (-0.3 to 3.5)	4.1% (0.6 to 7.1)	0.0% (0.0 to 0.0)	0.6% (0.2 to 0.9)	3.6% (-3.8 to 10.2)	9.3% (0.8 to 18.7)	12.1% (9.7 to 14.7)	22.3% (7.9 to 35.1)
**High-income**	8.2% (1.6 to 16.1)	6.8% (4.6 to 9.3)	1.1% (0.3 to 2.0)	-4.7% (-20.8 to 6.9)	6.1% (0.5 to 16.7)	0.3% (0.1 to 0.5)	2.5% (-0.5 to 5.3)	4.0% (0.3 to 7.2)	0.0% (0.0 to 0.0)	0.6% (0.2 to 1.1)	2.1% (-2.1 to 6.0)	7.5% (0.5 to 15.1)	12.4% (10.0 to 14.8)	16.0% (5.6 to 25.9)
**Latin America and Caribbean**	4.5% (1.0 to 8.6)	9.5% (6.7 to 12.9)	0.1% (0.0 to 0.3)	-5.4% (-23.5 to 7.8)	6.4% (0.4 to 17.5)	0.2% (0.1 to 0.2)	2.3% (-0.6 to 4.9)	4.0% (0.1 to 7.2)	0.0% (0.0 to 0.0)	2.3% (0.7 to 3.8)	1.3% (-1.2 to 3.7)	8.5% (0.3 to 17.8)	10.6% (8.5 to 12.8)	13.3% (4.6 to 21.4)
**North Africa and Middle East**	0.4% (0.1 to 1.0)	22.9% (18.1 to 28.0)	0.1% (0.0 to 0.2)	-1.4% (-5.1 to 1.8)	1.5% (0.0 to 7.4)	0.1% (0.1 to 0.2)	1.0% (-0.2 to 2.1)	2.6% (0.4 to 4.5)	0.0% (0.0 to 0.0)	1.1% (0.4 to 1.7)	4.3% (-4.7 to 12.0)	10.8% (1.0 to 20.9)	16.2% (13.1 to 19.4)	20.3% (7.2 to 32.6)
**South Asia**	2.6% (0.6 to 5.2)	16.7% (9.9 to 22.8)	0.1% (0.0 to 0.2)	-0.4% (-1.5 to 0.6)	6.2% (0.3 to 17.9)	0.0% (0.0 to 0.1)	3.4% (-0.8 to 7.1)	11.6% (1.2 to 20.1)	0.0% (0.0 to 0.0)	2.8% (0.7 to 4.7)	1.4% (-1.3 to 4.1)	2.2% (0.2 to 4.6)	10.7% (8.2 to 13.4)	9.6% (3.4 to 16.5)
**Southeast Asia, East Asia, and Oceania**	5.7% (1.4 to 10.6)	20.7% (13.5 to 25.7)	0.1% (0.0 to 0.2)	-4.1% (-17.1 to 5.9)	15.3% (4.9 to 29.0)	0.1% (0.0 to 0.1)	2.7% (-0.6 to 5.5)	4.5% (0.2 to 8.2)	0.0% (0.0 to 0.0)	0.7% (0.2 to 1.1)	1.5% (-1.5 to 4.3)	3.2% (0.3 to 6.8)	9.5% (7.4 to 11.8)	11.8% (4.1 to 19.7)
**Sub-Saharan Africa**	4.8% (1.1 to 9.3)	9.5% (5.7 to 13.4)	0.1% (0.0 to 0.2)	-1.5% (-6.0 to 2.4)	4.6% (0.1 to 14.1)	0.0% (0.0 to 0.0)	0.9% (-0.2 to 2.0)	6.7% (0.4 to 11.9)	0.0% (0.0 to 0.0)	5.3% (1.0 to 9.2)	1.4% (-1.3 to 4.0)	4.1% (0.3 to 8.5)	8.2% (6.4 to 10.1)	9.7% (3.2 to 16.4)
**GBD regions in alphabetical order**														
**Andean Latin America**	4.4% (1.0 to 8.3)	16.1% (10.1 to 22.8)	0.1% (0.0 to 0.1)	-4.2% (-16.9 to 6.1)	5.9% (0.2 to 16.8)	0.1% (0.0 to 0.2)	3.1% (-0.8 to 6.3)	4.5% (0.0 to 8.0)	0.0% (0.0 to 0.0)	3.1% (0.7 to 5.4)	1.3% (-1.3 to 3.7)	8.2% (0.2 to 17.5)	8.1% (6.3 to 9.9)	10.5% (3.6 to 17.1)
**Australasia**	9.3% (1.6 to 18.8)	4.9% (2.9 to 7.3)	1.3% (0.3 to 2.3)	-4.3% (-22.0 to 7.0)	2.6% (0.0 to 10.4)	0.4% (0.2 to 0.7)	2.5% (-0.5 to 5.3)	3.8% (0.3 to 6.8)	0.0% (0.0 to 0.0)	0.6% (0.1 to 1.1)	1.7% (-1.8 to 5.2)	8.7% (0.6 to 17.5)	12.7% (10.1 to 15.3)	17.9% (6.0 to 29.2)
**Caribbean**	4.8% (1.1 to 9.5)	10.0% (5.2 to 16.3)	0.1% (0.0 to 0.2)	-2.3% (-9.0 to 3.3)	3.8% (0.0 to 13.0)	0.1% (0.0 to 0.1)	2.6% (-0.7 to 5.3)	4.2% (0.1 to 7.5)	0.0% (0.0 to 0.0)	4.7% (0.8 to 8.0)	1.7% (-1.7 to 4.7)	6.1% (0.3 to 12.8)	9.8% (7.7 to 12.0)	11.3% (4.0 to 18.2)
**Central Asia**	4.7% (1.0 to 9.9)	17.0% (11.8 to 22.2)	0.3% (0.1 to 0.5)	-4.2% (-18.0 to 6.2)	6.4% (0.4 to 18.0)	0.1% (0.0 to 0.1)	2.1% (-0.4 to 4.4)	4.5% (0.4 to 8.0)	0.0% (0.0 to 0.0)	0.2% (0.1 to 0.4)	4.1% (-4.7 to 11.2)	8.1% (0.6 to 17.0)	10.1% (7.9 to 12.2)	16.0% (5.6 to 25.5)
**Central Europe**	9.3% (1.6 to 18.9)	12.8% (9.5 to 16.1)	0.8% (0.2 to 1.4)	-3.3% (-14.5 to 5.0)	15.2% (4.1 to 29.2)	0.4% (0.2 to 0.6)	1.3% (-0.2 to 2.9)	3.2% (0.4 to 5.7)	0.0% (0.0 to 0.0)	0.3% (0.1 to 0.6)	2.8% (-2.8 to 8.1)	8.6% (0.8 to 17.4)	15.8% (12.8 to 18.9)	21.8% (7.5 to 35.1)
**Central Latin America**	3.5% (0.8 to 6.9)	9.8% (6.8 to 12.9)	0.1% (0.0 to 0.2)	-4.7% (-19.3 to 6.6)	7.4% (0.7 to 18.9)	0.2% (0.1 to 0.2)	1.8% (-0.4 to 3.7)	4.4% (0.1 to 7.9)	0.0% (0.0 to 0.0)	1.7% (0.5 to 2.8)	1.0% (-1.0 to 3.0)	9.5% (0.3 to 19.6)	10.6% (8.4 to 12.8)	12.5% (4.3 to 20.3)
**Central Sub-Saharan Africa**	4.2% (0.8 to 8.3)	7.1% (4.4 to 10.3)	0.1% (0.0 to 0.2)	-1.1% (-4.3 to 1.8)	2.0% (0.0 to 9.5)	0.0% (0.0 to 0.0)	2.1% (-0.5 to 4.5)	6.0% (0.2 to 10.9)	0.0% (0.0 to 0.0)	8.3% (1.4 to 14.7)	1.1% (-1.1 to 3.5)	3.5% (0.2 to 7.3)	10.0% (7.6 to 12.5)	8.4% (2.7 to 14.6)
**East Asia**	6.5% (1.6 to 12.1)	23.8% (16.1 to 29.3)	0.1% (0.0 to 0.2)	-4.9% (-21.2 to 7.3)	17.0% (6.0 to 31.0)	0.1% (0.0 to 0.1)	1.4% (-0.3 to 3.1)	4.0% (0.2 to 7.4)	0.0% (0.0 to 0.0)	0.1% (0.0 to 0.1)	1.9% (-1.9 to 5.2)	3.5% (0.3 to 7.5)	10.2% (7.9 to 12.8)	12.6% (4.3 to 20.8)

**Eastern Europe**	7.2% (1.1 to 16.1)	8.4% (5.4 to 12.6)	1.1% (0.3 to 2.0)	-3.6% (-14.4 to 4.9)	6.0% (0.4 to 16.9)	0.1% (0.1 to 0.2)	1.7% (-0.3 to 3.6)	4.3% (0.7 to 7.5)	0.0% (0.0 to 0.0)	0.8% (0.4 to 1.3)	3.8% (-4.0 to 10.7)	9.9% (0.9 to 19.6)	10.8% (8.6 to 13.3)	24.0% (8.7 to 37.5)
**Eastern Sub-Saharan Africa**	4.3% (0.9 to 8.2)	4.8% (3.0 to 7.1)	0.1% (0.0 to 0.1)	-1.3% (-5.0 to 2.1)	7.1% (0.4 to 19.0)	0.0% (0.0 to 0.0)	1.1% (-0.3 to 2.3)	7.4% (0.3 to 13.4)	0.0% (0.0 to 0.0)	7.1% (0.9 to 12.8)	1.0% (-1.0 to 3.1)	2.4% (0.2 to 5.2)	5.8% (4.4 to 7.2)	7.4% (2.4 to 12.7)
**High-income Asia Pacific**	7.6% (1.4 to 14.5)	11.1% (6.9 to 16.3)	0.7% (0.2 to 1.3)	-4.0% (-16.2 to 5.8)	10.4% (1.5 to 23.3)	0.2% (0.1 to 0.3)	3.4% (-0.8 to 7.1)	5.4% (0.3 to 9.6)	0.0% (0.0 to 0.0)	0.2% (0.1 to 0.4)	1.4% (-1.4 to 4.1)	2.6% (0.3 to 5.4)	11.5% (9.3 to 13.9)	14.3% (5.1 to 23.0)
**High-income North America**	7.1% (1.4 to 14.5)	3.1% (1.5 to 5.0)	1.7% (0.4 to 2.9)	-5.1% (-23.1 to 7.5)	4.9% (0.1 to 14.8)	0.4% (0.2 to 0.7)	2.1% (-0.4 to 4.6)	4.1% (0.3 to 7.3)	0.0% (0.0 to 0.0)	0.9% (0.4 to 1.5)	2.1% (-2.2 to 6.0)	11.0% (0.6 to 21.6)	14.6% (11.9 to 17.4)	15.3% (5.5 to 24.8)
**North Africa and Middle East**	0.4% (0.1 to 1.0)	22.9% (18.1 to 28.0)	0.1% (0.0 to 0.2)	-1.4% (-5.1 to 1.8)	1.5% (0.0 to 7.4)	0.1% (0.1 to 0.2)	1.0% (-0.2 to 2.1)	2.6% (0.4 to 4.5)	0.0% (0.0 to 0.0)	1.1% (0.4 to 1.7)	4.3% (-4.7 to 12.0)	10.8% (1.0 to 20.9)	16.2% (13.1 to 19.4)	20.3% (7.2 to 32.6)
**Oceania**	1.4% (0.2 to 3.0)	5.1% (1.8 to 11.1)	0.0% (0.0 to 0.1)	-3.4% (-13.2 to 5.4)	8.4% (0.9 to 20.4)	0.0% (0.0 to 0.0)	0.2% (0.0 to 0.4)	6.9% (-0.2 to 12.6)	0.0% (0.0 to 0.0)	4.1% (0.3 to 7.5)	1.1% (-1.2 to 3.4)	5.1% (0.2 to 11.1)	8.6% (6.3 to 11.0)	6.0% (2.1 to 10.0)
**South Asia**	2.6% (0.6 to 5.2)	16.7% (9.9 to 22.8)	0.1% (0.0 to 0.2)	-0.4% (-1.5 to 0.6)	6.2% (0.3 to 17.9)	0.0% (0.0 to 0.1)	3.4% (-0.8 to 7.1)	11.6% (1.2 to 20.1)	0.0% (0.0 to 0.0)	2.8% (0.7 to 4.7)	1.4% (-1.3 to 4.1)	2.2% (0.2 to 4.6)	10.7% (8.2 to 13.4)	9.6% (3.4 to 16.5)
**Southeast Asia**	3.7% (0.9 to 6.9)	12.9% (7.5 to 17.5)	0.0% (0.0 to 0.1)	-2.1% (-8.3 to 3.0)	11.9% (2.2 to 25.1)	0.0% (0.0 to 0.0)	5.7% (-1.4 to 11.9)	5.6% (0.2 to 10.1)	0.0% (0.0 to 0.0)	2.0% (0.6 to 3.5)	0.8% (-0.7 to 2.2)	2.3% (0.2 to 4.9)	8.2% (6.3 to 10.2)	10.2% (3.5 to 17.0)
**Southern Latin America**	7.9% (1.8 to 14.8)	11.2% (6.4 to 17.0)	0.5% (0.1 to 0.9)	-7.1% (-31.9 to 10.3)	6.7% (0.2 to 18.5)	0.3% (0.2 to 0.5)	3.5% (-0.9 to 7.3)	3.7% (0.1 to 6.9)	0.0% (0.0 to 0.0)	0.7% (0.2 to 1.1)	2.2% (-2.3 to 6.5)	9.5% (0.4 to 19.9)	11.0% (8.6 to 13.2)	13.5% (4.7 to 22.1)
**Southern Sub-Saharan Africa**	5.6% (1.3 to 10.8)	12.9% (8.9 to 16.9)	0.1% (0.0 to 0.1)	-3.9% (-15.2 to 5.5)	2.6% (0.0 to 10.7)	0.1% (0.0 to 0.1)	1.1% (-0.3 to 2.3)	8.3% (0.9 to 14.5)	0.0% (0.0 to 0.0)	4.4% (1.0 to 7.7)	0.9% (-0.8 to 2.5)	8.8% (0.4 to 17.9)	9.8% (7.6 to 11.9)	11.4% (3.7 to 19.2)
**Tropical Latin America**	5.1% (1.1 to 9.7)	7.9% (4.5 to 12.3)	0.2% (0.0 to 0.4)	-7.1% (-32.0 to 10.3)	6.7% (0.3 to 18.5)	0.2% (0.1 to 0.3)	2.5% (-0.6 to 5.5)	3.6% (0.1 to 6.6)	0.0% (0.0 to 0.0)	2.0% (0.6 to 3.3)	1.4% (-1.3 to 3.9)	8.5% (0.4 to 18.0)	11.3% (8.9 to 13.8)	15.0% (5.2 to 23.9)
**Western Europe**	9.8% (1.9 to 19.2)	6.6% (4.7 to 9.1)	1.0% (0.2 to 1.7)	-4.1% (-18.6 to 6.4)	4.3% (0.1 to 13.7)	0.2% (0.1 to 0.3)	2.1% (-0.4 to 4.5)	3.2% (0.3 to 5.9)	0.0% (0.0 to 0.0)	0.6% (0.2 to 1.1)	2.4% (-2.5 to 7.2)	6.8% (0.5 to 14.3)	11.3% (9.0 to 13.6)	18.3% (6.2 to 29.7)
**Western Sub-Saharan Africa**	5.3% (1.2 to 10.3)	13.2% (7.3 to 19.5)	0.2% (0.0 to 0.4)	-1.3% (-5.1 to 2.0)	3.8% (0.0 to 13.2)	0.0% (0.0 to 0.0)	0.4% (-0.1 to 0.9)	5.8% (0.5 to 10.3)	0.0% (0.0 to 0.0)	3.2% (0.9 to 5.4)	1.9% (-1.8 to 5.5)	4.6% (0.3 to 9.6)	9.3% (7.2 to 11.5)	11.5% (3.9 to 19.2)
**Countries in alphabetical order**														
**Afghanistan**	0.1% (0.0 to 0.2)	6.9% (4.2 to 10.3)	0.1% (0.0 to 0.2)	-2.2% (-8.3 to 3.1)	1.5% (0.0 to 7.7)	0.0% (0.0 to 0.0)	4.5% (-1.0 to 9.3)	6.0% (0.7 to 10.4)	0.0% (0.0 to 0.0)	10.3% (3.3 to 16.6)	4.4% (-4.7 to 12.5)	6.5% (0.4 to 13.1)	15.1% (11.9 to 18.4)	16.0% (5.8 to 25.9)
**Albania**	5.2% (1.0 to 10.3)	11.0% (6.7 to 14.5)	1.2% (0.3 to 2.1)	-4.6% (-19.4 to 6.9)	15.7% (4.5 to 29.6)	0.2% (0.1 to 0.3)	1.1% (-0.3 to 2.6)	2.1% (0.0 to 4.2)	0.0% (0.0 to 0.0)	0.0% (0.0 to 0.1)	1.6% (-1.8 to 4.8)	5.6% (0.2 to 12.6)	9.4% (6.9 to 12.2)	9.1% (3.0 to 15.2)
**Algeria**	0.4% (0.1 to 1.0)	17.6% (12.4 to 23.7)	0.2% (0.0 to 0.3)	-1.2% (-4.3 to 1.4)	1.3% (0.0 to 7.1)	0.1% (0.1 to 0.3)	0.6% (-0.1 to 1.2)	2.5% (0.3 to 4.4)	0.0% (0.0 to 0.0)	0.7% (0.2 to 1.2)	4.1% (-4.4 to 12.1)	8.5% (0.8 to 16.9)	18.7% (15.1 to 22.5)	19.0% (6.3 to 31.7)
**American Samoa**	0.5% (0.0 to 1.6)	3.2% (0.4 to 6.7)	0.1% (0.0 to 0.2)	-5.5% (-22.2 to 7.5)	3.9% (0.1 to 13.0)	0.1% (0.1 to 0.2)	0.1% (0.0 to 0.3)	5.9% (-0.1 to 10.6)	0.0% (0.0 to 0.0)	4.1% (0.5 to 7.5)	1.3% (-1.4 to 3.8)	14.6% (-0.1 to 27.8)	15.4% (11.8 to 19.2)	7.9% (2.8 to 13.1)
**Andorra**	9.2% (1.4 to 18.5)	4.9% (2.7 to 7.5)	1.2% (0.3 to 2.2)	-4.1% (-19.0 to 5.8)	2.9% (0.0 to 11.7)	0.4% (0.2 to 0.6)	1.4% (-0.3 to 3.1)	2.8% (0.2 to 5.1)	0.0% (0.0 to 0.0)	0.4% (0.1 to 0.8)	2.4% (-2.5 to 7.1)	6.1% (0.5 to 12.9)	11.9% (9.6 to 14.6)	19.3% (6.5 to 31.1)
**Angola**	5.4% (1.2 to 10.6)	14.3% (6.8 to 21.6)	0.1% (0.0 to 0.1)	-1.9% (-7.6 to 3.1)	3.1% (0.0 to 12.1)	0.0% (0.0 to 0.0)	0.7% (-0.2 to 1.5)	5.0% (0.1 to 9.2)	0.0% (0.0 to 0.0)	2.2% (0.6 to 3.8)	1.4% (-1.4 to 4.2)	2.8% (0.2 to 6.2)	8.4% (6.5 to 10.5)	9.4% (3.1 to 16.6)
**Antigua and Barbuda**	5.2% (1.2 to 10.1)	13.6% (5.0 to 24.4)	0.1% (0.0 to 0.1)	-2.2% (-8.0 to 3.4)	4.3% (0.0 to 14.6)	0.0% (0.0 to 0.0)	5.4% (-1.2 to 11.5)	3.5% (0.1 to 6.5)	0.0% (0.0 to 0.0)	1.7% (0.5 to 2.9)	2.3% (-2.4 to 6.8)	7.3% (0.3 to 15.3)	11.5% (9.1 to 14.1)	11.6% (3.7 to 19.5)

**Argentina**	7.7% (1.8 to 14.4)	9.8% (5.0 to 16.2)	0.4% (0.1 to 0.7)	-7.7% (-35.1 to 11.2)	6.5% (0.2 to 18.2)	0.4% (0.2 to 0.6)	4.0% (-1.0 to 8.4)	3.5% (0.0 to 6.5)	0.0% (0.0 to 0.0)	0.6% (0.2 to 1.0)	2.1% (-2.1 to 6.1)	9.5% (0.3 to 19.9)	10.8% (8.5 to 13.0)	12.7% (4.4 to 20.5)
**Armenia**	4.3% (0.5 to 10.3)	22.9% (14.9 to 31.4)	0.3% (0.1 to 0.5)	-3.0% (-11.4 to 3.7)	6.7% (0.3 to 18.7)	0.0% (0.0 to 0.1)	1.5% (-0.3 to 3.2)	2.2% (0.4 to 3.8)	0.0% (0.0 to 0.0)	0.0% (0.0 to 0.0)	5.5% (-6.2 to 15.4)	8.8% (0.9 to 17.5)	12.1% (9.5 to 14.8)	19.2% (6.4 to 32.0)
**Australia**	9.3% (1.6 to 18.9)	5.3% (3.2 to 7.8)	1.3% (0.3 to 2.3)	-4.3% (-22.1 to 7.0)	2.4% (0.0 to 9.8)	0.5% (0.2 to 0.7)	2.6% (-0.6 to 5.4)	3.9% (0.3 to 7.0)	0.0% (0.0 to 0.0)	0.6% (0.1 to 1.1)	1.7% (-1.7 to 5.1)	8.8% (0.6 to 17.7)	12.5% (10.0 to 15.1)	17.9% (6.0 to 29.2)
**Austria**	9.5% (1.5 to 19.7)	7.3% (5.2 to 9.8)	1.0% (0.2 to 1.7)	-3.7% (-17.8 to 6.0)	6.8% (0.3 to 18.9)	0.3% (0.1 to 0.5)	1.2% (-0.2 to 2.6)	2.5% (0.2 to 4.6)	0.0% (0.0 to 0.0)	0.6% (0.2 to 1.1)	1.8% (-1.7 to 5.4)	6.2% (0.5 to 13.3)	8.8% (6.8 to 10.9)	20.4% (7.1 to 32.4)
**Azerbaijan**	4.2% (0.8 to 8.8)	15.9% (8.3 to 25.4)	0.3% (0.1 to 0.5)	-3.2% (-13.0 to 4.7)	6.2% (0.3 to 17.7)	0.1% (0.0 to 0.1)	1.1% (-0.2 to 2.4)	3.6% (0.1 to 6.9)	0.0% (0.0 to 0.0)	0.0% (0.0 to 0.1)	2.8% (-3.1 to 7.8)	7.7% (0.4 to 16.6)	9.3% (7.2 to 11.7)	11.4% (4.0 to 19.6)
**Bahamas**	4.6% (0.9 to 9.0)	13.1% (4.5 to 25.7)	0.1% (0.0 to 0.1)	-6.3% (-25.4 to 8.6)	4.0% (0.0 to 13.7)	0.0% (0.0 to 0.1)	5.2% (-1.4 to 11.0)	4.8% (0.0 to 8.8)	0.0% (0.0 to 0.0)	0.6% (0.2 to 0.9)	2.3% (-2.6 to 6.7)	10.1% (0.3 to 20.8)	11.3% (8.7 to 14.0)	11.7% (4.0 to 19.3)
**Bahrain**	0.5% (0.1 to 1.1)	35.5% (27.6 to 43.6)	0.2% (0.1 to 0.4)	-1.9% (-7.3 to 2.5)	1.5% (0.0 to 7.9)	0.3% (0.1 to 0.5)	0.3% (0.0 to 0.6)	1.7% (0.2 to 3.0)	0.0% (0.0 to 0.0)	0.2% (0.0 to 0.5)	3.3% (-3.5 to 9.8)	10.4% (0.9 to 20.3)	21.8% (17.8 to 25.7)	20.0% (6.8 to 32.7)
**Bangladesh**	0.3% (0.0 to 0.8)	8.3% (4.3 to 13.4)	0.3% (0.1 to 0.5)	-0.2% (-0.6 to 0.2)	5.9% (0.1 to 17.7)	0.0% (0.0 to 0.0)	7.6% (-2.1 to 15.7)	8.7% (0.6 to 15.5)	0.0% (0.0 to 0.0)	6.4% (1.0 to 11.1)	0.5% (-0.5 to 1.7)	1.5% (0.1 to 3.3)	10.6% (8.0 to 13.1)	7.9% (2.6 to 14.4)
**Barbados**	5.5% (1.3 to 11.0)	16.8% (6.7 to 29.6)	0.2% (0.0 to 0.3)	-3.3% (-12.5 to 4.5)	2.8% (0.0 to 11.2)	0.1% (0.1 to 0.2)	3.2% (-0.7 to 7.0)	5.0% (0.4 to 9.1)	0.0% (0.0 to 0.0)	1.3% (0.4 to 2.3)	2.0% (-2.0 to 6.0)	9.2% (0.4 to 19.0)	15.6% (12.7 to 18.6)	14.4% (4.8 to 24.2)
**Belarus**	9.0% (1.3 to 19.4)	11.1% (8.0 to 15.0)	0.7% (0.2 to 1.3)	-3.5% (-16.7 to 5.9)	3.3% (0.0 to 12.2)	0.1% (0.0 to 0.1)	0.3% (-0.1 to 0.7)	4.0% (0.6 to 6.9)	0.0% (0.0 to 0.0)	0.3% (0.0 to 0.6)	4.8% (-5.2 to 13.2)	10.1% (0.9 to 20.3)	8.3% (6.4 to 10.2)	21.2% (7.3 to 34.5)
**Belgium**	10.0% (1.9 to 19.4)	7.1% (4.9 to 9.5)	1.2% (0.3 to 2.2)	-4.7% (-21.0 to 7.3)	6.0% (0.1 to 16.9)	0.3% (0.2 to 0.6)	2.1% (-0.4 to 4.5)	3.6% (0.2 to 6.6)	0.0% (0.0 to 0.0)	0.3% (0.0 to 0.6)	2.2% (-2.2 to 6.5)	6.2% (0.4 to 13.0)	10.8% (8.5 to 13.2)	16.6% (5.6 to 27.3)
**Belize**	4.6% (1.0 to 8.9)	17.3% (6.7 to 31.8)	0.1% (0.0 to 0.1)	-1.6% (-6.2 to 2.5)	4.1% (0.0 to 14.1)	0.0% (0.0 to 0.1)	1.5% (-0.4 to 3.2)	1.8% (0.0 to 3.4)	0.0% (0.0 to 0.0)	3.3% (0.7 to 5.9)	1.5% (-1.4 to 4.3)	10.3% (0.3 to 20.5)	9.3% (7.2 to 11.5)	10.7% (3.5 to 18.0)
**Benin**	3.3% (0.7 to 6.8)	8.7% (4.8 to 13.8)	0.1% (0.0 to 0.2)	-0.5% (-1.9 to 0.7)	4.6% (0.0 to 15.7)	0.0% (0.0 to 0.0)	0.1% (0.0 to 0.1)	6.2% (0.5 to 11.1)	0.0% (0.0 to 0.0)	1.5% (0.6 to 2.6)	1.3% (-1.3 to 3.8)	4.7% (0.2 to 9.7)	8.2% (6.5 to 10.1)	10.4% (3.5 to 17.8)
**Bermuda**	7.5% (1.5 to 15.2)	3.2% (0.6 to 6.1)	0.2% (0.0 to 0.3)	-4.9% (-20.4 to 6.9)	4.4% (0.0 to 14.6)	0.1% (0.0 to 0.2)	3.6% (-0.7 to 7.6)	4.0% (0.3 to 7.2)	0.0% (0.0 to 0.0)	0.7% (0.2 to 1.2)	2.7% (-2.8 to 7.9)	10.5% (0.7 to 20.8)	14.0% (11.2 to 16.9)	16.3% (5.5 to 27.1)
**Bhutan**	0.7% (0.1 to 1.7)	19.5% (11.6 to 26.1)	0.2% (0.1 to 0.4)	-0.6% (-2.1 to 0.8)	6.0% (0.1 to 18.0)	0.0% (0.0 to 0.0)	3.5% (-0.8 to 7.7)	5.6% (0.3 to 10.3)	0.0% (0.0 to 0.0)	2.2% (0.7 to 3.8)	1.1% (-1.0 to 3.3)	4.5% (0.3 to 10.0)	10.2% (8.1 to 12.6)	14.2% (4.8 to 23.8)
**Bolivia (Plurinational State of)**	4.4% (1.0 to 8.3)	14.7% (7.6 to 23.3)	0.0% (0.0 to 0.1)	-6.2% (-24.2 to 8.7)	5.8% (0.1 to 16.9)	0.1% (0.0 to 0.1)	3.1% (-0.7 to 6.4)	4.9% (0.0 to 9.0)	0.0% (0.0 to 0.0)	4.3% (0.8 to 7.8)	1.2% (-1.2 to 3.7)	6.9% (0.2 to 14.9)	8.5% (6.5 to 10.5)	9.9% (3.2 to 16.4)
**Bosnia and Herzegovina**	6.6% (0.7 to 14.3)	18.2% (10.2 to 23.4)	0.6% (0.2 to 1.1)	-1.3% (-4.5 to 1.6)	16.0% (4.2 to 30.3)	0.2% (0.1 to 0.4)	0.1% (0.0 to 0.2)	2.4% (0.5 to 4.3)	0.0% (0.0 to 0.0)	0.4% (0.1 to 0.7)	2.4% (-2.2 to 7.1)	8.1% (0.9 to 16.0)	18.2% (14.3 to 22.2)	25.4% (8.8 to 41.3)
**Botswana**	3.4% (0.8 to 6.8)	13.6% (7.7 to 19.6)	0.1% (0.0 to 0.1)	-3.4% (-13.3 to 4.9)	2.5% (0.0 to 10.5)	0.0% (0.0 to 0.0)	1.6% (-0.3 to 3.5)	8.6% (1.1 to 15.1)	0.0% (0.0 to 0.0)	4.6% (1.1 to 7.8)	1.4% (-1.3 to 4.2)	6.7% (0.4 to 13.6)	9.7% (7.6 to 12.1)	10.1% (3.1 to 17.9)
**Brazil**	5.0% (1.1 to 9.6)	8.0% (4.5 to 12.3)	0.2% (0.0 to 0.4)	-7.1% (-32.1 to 10.3)	6.7% (0.3 to 18.5)	0.2% (0.1 to 0.3)	2.6% (-0.6 to 5.6)	3.6% (0.1 to 6.6)	0.0% (0.0 to 0.0)	1.9% (0.6 to 3.3)	1.4% (-1.3 to 3.9)	8.6% (0.4 to 18.0)	11.3% (8.9 to 13.8)	15.0% (5.2 to 23.9)
**Brunei Darussalam**	0.3% (0.0 to 0.8)	3.9% (0.8 to 7.8)	0.3% (0.1 to 0.5)	-2.8% (-10.9 to 3.9)	11.4% (1.7 to 24.6)	0.1% (0.0 to 0.1)	4.4% (-1.1 to 9.5)	5.9% (0.3 to 10.5)	0.0% (0.0 to 0.0)	1.6% (0.5 to 2.8)	1.4% (-1.3 to 4.0)	5.5% (0.2 to 10.9)	13.8% (11.0 to 16.9)	14.1% (4.7 to 23.1)
**Bulgaria**	10.8% (1.7 to 21.9)	12.6% (9.4 to 16.1)	0.9% (0.2 to 1.6)	-3.0% (-14.1 to 4.8)	16.1% (4.5 to 30.1)	0.3% (0.2 to 0.5)	2.6% (-0.4 to 5.5)	3.6% (0.5 to 6.2)	0.0% (0.0 to 0.0)	0.2% (0.0 to 0.4)	3.4% (-3.4 to 9.8)	8.7% (0.8 to 17.8)	15.8% (12.8 to 19.3)	22.6% (7.8 to 36.3)

**Burkina Faso**	7.2% (1.7 to 13.8)	8.5% (4.8 to 13.5)	0.1% (0.0 to 0.2)	-2.3% (-8.8 to 3.3)	3.7% (0.0 to 13.4)	0.0% (0.0 to 0.0)	0.2% (0.0 to 0.4)	13.2% (1.6 to 22.6)	0.0% (0.0 to 0.0)	8.2% (1.3 to 14.4)	1.6% (-1.7 to 4.7)	1.2% (0.1 to 3.0)	7.0% (5.4 to 8.9)	6.3% (1.9 to 11.3)
**Burundi**	5.6% (1.0 to 11.0)	3.6% (1.9 to 5.9)	0.0% (0.0 to 0.1)	-0.1% (-0.5 to 0.2)	7.0% (0.3 to 18.9)	0.0% (0.0 to 0.0)	0.3% (-0.1 to 0.7)	3.8% (0.0 to 7.0)	0.0% (0.0 to 0.0)	7.9% (0.9 to 14.1)	1.6% (-1.6 to 4.7)	0.8% (0.0 to 2.2)	5.4% (4.1 to 6.9)	6.6% (2.1 to 11.7)
**Cv¥te d'Ivoire**	8.5% (1.9 to 16.1)	12.7% (6.2 to 20.2)	0.2% (0.0 to 0.3)	-1.9% (-7.1 to 2.7)	4.1% (0.0 to 14.4)	0.0% (0.0 to 0.0)	0.0% (0.0 to 0.1)	4.6% (0.2 to 8.3)	0.0% (0.0 to 0.0)	3.8% (1.1 to 6.3)	2.2% (-2.2 to 6.3)	5.1% (0.3 to 10.7)	9.2% (7.0 to 11.3)	11.7% (4.0 to 19.7)
**Cabo Verde**	7.3% (1.4 to 13.8)	23.0% (13.7 to 31.7)	0.2% (0.1 to 0.4)	-1.5% (-5.6 to 2.1)	3.8% (0.0 to 13.8)	0.0% (0.0 to 0.0)	1.1% (-0.2 to 2.4)	5.0% (0.6 to 8.8)	0.0% (0.0 to 0.0)	0.7% (0.2 to 1.3)	1.6% (-1.6 to 4.6)	5.4% (0.4 to 10.9)	13.1% (10.4 to 15.9)	14.2% (4.9 to 24.0)
**Cambodia**	5.4% (1.2 to 10.2)	7.0% (3.4 to 12.2)	0.0% (0.0 to 0.0)	-1.7% (-6.8 to 2.9)	11.1% (1.7 to 24.6)	0.0% (0.0 to 0.0)	7.7% (-2.1 to 16.1)	8.1% (0.4 to 14.6)	0.0% (0.0 to 0.0)	7.0% (0.9 to 12.5)	0.4% (-0.3 to 1.2)	1.0% (0.0 to 2.5)	6.7% (5.2 to 8.3)	8.9% (2.9 to 15.0)
**Cameroon**	8.6% (1.8 to 16.3)	10.7% (5.5 to 17.8)	0.2% (0.0 to 0.4)	-1.4% (-5.7 to 2.3)	3.7% (0.0 to 13.3)	0.0% (0.0 to 0.0)	0.1% (0.0 to 0.2)	4.2% (0.1 to 7.6)	0.0% (0.0 to 0.0)	0.7% (0.3 to 1.3)	1.2% (-1.2 to 3.7)	7.8% (0.3 to 16.7)	9.9% (7.7 to 12.4)	6.7% (2.0 to 12.0)
**Canada**	7.2% (1.3 to 15.4)	2.2% (0.8 to 4.1)	0.5% (0.1 to 0.9)	-4.7% (-20.0 to 6.7)	5.1% (0.1 to 15.2)	0.2% (0.1 to 0.3)	1.4% (-0.3 to 3.1)	3.6% (0.3 to 6.5)	0.0% (0.0 to 0.0)	0.6% (0.3 to 1.1)	2.0% (-2.0 to 5.8)	8.6% (0.6 to 17.5)	10.0% (8.0 to 12.1)	17.5% (6.3 to 28.4)
**Central African Republic**	3.0% (0.4 to 6.6)	4.5% (2.7 to 7.0)	0.1% (0.0 to 0.2)	-6.1% (-23.5 to 8.8)	2.9% (0.0 to 11.8)	0.0% (0.0 to 0.0)	1.6% (-0.4 to 3.4)	6.7% (0.0 to 12.2)	0.0% (0.0 to 0.0)	10.7% (1.2 to 19.1)	1.2% (-1.1 to 3.7)	2.4% (0.2 to 5.4)	9.3% (6.8 to 11.8)	7.0% (2.2 to 12.4)
**Chad**	4.5% (0.8 to 10.6)	9.1% (5.1 to 14.3)	0.2% (0.0 to 0.3)	-2.2% (-8.2 to 3.2)	3.7% (0.0 to 13.5)	0.0% (0.0 to 0.0)	0.6% (-0.1 to 1.3)	11.3% (1.4 to 19.3)	0.0% (0.0 to 0.0)	10.6% (2.2 to 18.0)	3.0% (-3.2 to 8.9)	2.5% (0.2 to 5.1)	9.0% (6.8 to 11.3)	9.6% (3.2 to 16.3)
**Chile**	8.6% (1.7 to 16.6)	16.2% (10.2 to 23.1)	0.8% (0.2 to 1.5)	-6.0% (-25.5 to 8.6)	7.0% (0.3 to 19.4)	0.3% (0.1 to 0.4)	2.6% (-0.6 to 5.6)	4.3% (0.2 to 7.9)	0.0% (0.0 to 0.0)	0.4% (0.1 to 0.8)	2.3% (-2.4 to 6.8)	10.0% (0.5 to 20.4)	11.7% (9.2 to 14.1)	14.8% (5.1 to 24.2)
**China**	6.5% (1.6 to 12.1)	24.4% (16.4 to 29.9)	0.1% (0.0 to 0.2)	-5.0% (-21.5 to 7.4)	17.0% (6.0 to 31.1)	0.1% (0.0 to 0.1)	1.3% (-0.3 to 2.9)	3.9% (0.2 to 7.3)	0.0% (0.0 to 0.0)	0.0% (0.0 to 0.1)	1.9% (-1.9 to 5.2)	3.6% (0.3 to 7.6)	10.2% (7.9 to 12.9)	12.7% (4.3 to 20.9)
**Colombia**	2.5% (0.6 to 5.2)	9.9% (6.3 to 13.9)	0.1% (0.0 to 0.2)	-5.2% (-20.5 to 7.1)	11.8% (2.4 to 25.1)	0.1% (0.1 to 0.2)	3.0% (-0.7 to 6.3)	4.1% (0.1 to 7.5)	0.0% (0.0 to 0.0)	1.6% (0.6 to 2.7)	1.4% (-1.3 to 4.0)	8.0% (0.3 to 16.9)	8.6% (6.8 to 10.5)	12.7% (4.5 to 20.8)
**Comoros**	0.6% (0.1 to 1.3)	2.6% (1.3 to 4.3)	0.0% (0.0 to 0.1)	-0.6% (-2.2 to 0.8)	7.2% (0.3 to 19.5)	0.0% (0.0 to 0.0)	1.8% (-0.4 to 3.8)	4.5% (0.0 to 8.4)	0.0% (0.0 to 0.0)	10.1% (1.8 to 17.5)	1.4% (-1.3 to 4.0)	3.9% (0.2 to 8.1)	6.0% (4.7 to 7.5)	9.8% (3.4 to 16.5)
**Congo**	7.0% (1.4 to 13.3)	13.6% (5.8 to 22.3)	0.1% (0.0 to 0.1)	-1.9% (-7.4 to 3.1)	3.0% (0.0 to 11.9)	0.0% (0.0 to 0.0)	3.6% (-0.8 to 7.6)	5.3% (0.2 to 9.9)	0.0% (0.0 to 0.0)	9.3% (1.7 to 16.0)	1.3% (-1.2 to 3.9)	5.1% (0.3 to 10.5)	9.0% (6.9 to 11.2)	10.0% (3.4 to 16.9)
**Cook Islands**	8.5% (1.7 to 16.5)	2.6% (0.0 to 6.3)	0.1% (0.0 to 0.2)	-5.6% (-22.5 to 7.2)	8.6% (0.9 to 20.4)	0.2% (0.1 to 0.4)	0.0% (0.0 to 0.1)	4.8% (0.0 to 8.9)	0.0% (0.0 to 0.0)	2.7% (0.7 to 4.8)	1.7% (-1.8 to 4.7)	14.0% (0.2 to 26.8)	17.1% (13.3 to 20.9)	10.5% (3.8 to 17.4)
**Costa Rica**	3.9% (0.9 to 8.0)	9.0% (5.9 to 12.5)	0.1% (0.0 to 0.2)	-3.9% (-15.3 to 5.3)	8.2% (0.6 to 20.9)	0.2% (0.1 to 0.3)	4.1% (-1.0 to 8.6)	3.6% (0.1 to 6.5)	0.0% (0.0 to 0.0)	2.9% (0.7 to 4.9)	1.9% (-2.0 to 5.6)	8.6% (0.4 to 18.4)	11.4% (9.0 to 13.6)	14.6% (5.0 to 24.0)
**Croatia**	9.2% (1.4 to 18.8)	11.3% (8.4 to 14.7)	0.2% (0.1 to 0.4)	-3.0% (-11.5 to 4.1)	16.3% (4.8 to 30.3)	0.4% (0.2 to 0.6)	2.7% (-0.5 to 5.8)	2.7% (0.3 to 4.8)	0.0% (0.0 to 0.0)	0.7% (0.2 to 1.3)	2.4% (-2.4 to 7.2)	8.5% (0.7 to 17.2)	16.3% (13.2 to 19.5)	20.4% (6.8 to 33.6)
**Cuba**	5.2% (1.1 to 10.6)	14.5% (6.7 to 24.3)	0.2% (0.0 to 0.3)	-3.8% (-14.9 to 5.2)	4.1% (0.0 to 14.0)	0.2% (0.1 to 0.4)	0.2% (0.0 to 0.5)	3.2% (0.2 to 5.6)	0.0% (0.0 to 0.0)	0.5% (0.2 to 0.8)	1.9% (-1.9 to 5.7)	7.6% (0.4 to 15.4)	13.6% (10.9 to 16.4)	15.4% (5.1 to 25.3)
**Cyprus**	7.2% (1.4 to 14.2)	10.5% (7.5 to 13.8)	0.4% (0.1 to 0.8)	-3.4% (-13.6 to 4.7)	3.9% (0.0 to 13.6)	0.1% (0.0 to 0.2)	3.0% (-0.7 to 6.5)	2.5% (0.1 to 4.7)	0.0% (0.0 to 0.0)	0.6% (-0.2 to 1.3)	2.1% (-2.2 to 7.2)	5.3% (0.4 to 11.4)	12.6% (9.9 to 15.1)	17.9% (5.7 to 30.5)
**Czechia**	11.4% (1.5 to 23.3)	9.8% (7.2 to 12.8)	0.7% (0.2 to 1.3)	-3.3% (-14.0 to 4.7)	15.8% (4.4 to 30.2)	0.3% (0.2 to 0.6)	2.4% (-0.4 to 5.0)	3.4% (0.5 to 6.0)	0.0% (0.0 to 0.0)	1.1% (0.4 to 1.8)	1.8% (-1.7 to 5.4)	9.2% (0.9 to 18.7)	17.8% (14.3 to 21.4)	23.6% (8.2 to 37.8)
**Democratic People's Republic of Korea**	4.9% (1.1 to 9.0)	5.9% (3.7 to 8.5)	0.1% (0.0 to 0.1)	-1.3% (-4.9 to 1.9)	15.1% (4.3 to 29.6)	0.0% (0.0 to 0.0)	3.7% (-0.8 to 7.6)	5.8% (0.0 to 10.6)	0.0% (0.0 to 0.0)	0.4% (0.2 to 0.7)	1.2% (-1.1 to 3.3)	1.1% (0.1 to 3.0)	8.4% (6.1 to 10.7)	10.6% (4.0 to 17.0)

**Democratic Republic of the Congo**	3.5% (0.6 to 7.2)	4.0% (2.5 to 5.9)	0.1% (0.0 to 0.2)	-0.2% (-0.6 to 0.3)	1.5% (0.0 to 8.2)	0.0% (0.0 to 0.0)	2.5% (-0.6 to 5.5)	6.4% (0.2 to 11.6)	0.0% (0.0 to 0.0)	10.2% (1.6 to 18.0)	1.0% (-1.0 to 3.2)	3.6% (0.2 to 7.6)	10.6% (7.9 to 13.3)	8.0% (2.6 to 14.0)
**Denmark**	10.5% (2.0 to 20.7)	5.5% (3.3 to 8.2)	1.1% (0.3 to 1.9)	-4.4% (-19.6 to 6.7)	4.5% (0.0 to 14.9)	0.2% (0.1 to 0.4)	1.6% (-0.3 to 3.7)	2.8% (0.2 to 5.1)	0.0% (0.0 to 0.0)	0.4% (0.0 to 0.9)	2.2% (-2.3 to 6.9)	5.6% (0.4 to 11.8)	9.2% (7.2 to 11.3)	18.1% (6.0 to 29.1)
**Djibouti**	0.2% (-0.1 to 0.5)	19.9% (9.9 to 34.0)	0.1% (0.0 to 0.1)	-2.9% (-11.7 to 4.1)	7.0% (0.2 to 19.6)	0.0% (0.0 to 0.0)	4.1% (-1.0 to 8.7)	10.4% (1.1 to 18.2)	0.0% (0.0 to 0.0)	2.4% (0.7 to 4.2)	2.1% (-2.2 to 5.9)	1.0% (0.1 to 2.6)	6.7% (5.2 to 8.5)	8.6% (2.8 to 14.8)
**Dominica**	5.3% (1.3 to 10.3)	14.1% (5.5 to 25.6)	0.1% (0.0 to 0.2)	-2.9% (-11.5 to 4.3)	4.1% (0.0 to 14.5)	0.1% (0.1 to 0.2)	0.3% (-0.1 to 0.6)	0.7% (0.0 to 1.4)	0.0% (0.0 to 0.0)	0.8% (0.3 to 1.3)	2.8% (-3.1 to 8.3)	10.2% (0.5 to 20.9)	14.2% (11.3 to 17.4)	11.9% (3.9 to 20.5)
**Dominican Republic**	5.2% (1.1 to 10.0)	13.6% (4.8 to 24.7)	0.1% (0.0 to 0.2)	-3.1% (-12.6 to 4.5)	4.0% (0.0 to 13.5)	0.1% (0.0 to 0.1)	3.4% (-0.8 to 7.2)	1.9% (0.0 to 3.4)	0.0% (0.0 to 0.0)	1.3% (0.5 to 2.0)	1.5% (-1.4 to 4.0)	7.4% (0.2 to 15.6)	8.7% (6.6 to 11.0)	11.7% (4.3 to 19.1)
**Ecuador**	2.7% (0.6 to 5.3)	12.2% (7.6 to 18.6)	0.1% (0.0 to 0.2)	-6.0% (-24.9 to 8.5)	5.9% (0.2 to 16.9)	0.2% (0.1 to 0.3)	5.9% (-1.6 to 12.1)	2.8% (0.0 to 5.2)	0.0% (0.0 to 0.0)	4.2% (0.9 to 7.5)	1.4% (-1.4 to 4.3)	9.8% (0.2 to 20.1)	10.2% (8.0 to 12.4)	10.8% (3.8 to 17.5)
**Egypt**	0.2% (0.0 to 0.4)	37.3% (28.2 to 45.8)	0.2% (0.0 to 0.3)	-1.5% (-5.1 to 1.7)	1.5% (0.0 to 7.8)	0.1% (0.1 to 0.2)	0.1% (0.0 to 0.1)	1.6% (0.3 to 2.8)	0.0% (0.0 to 0.0)	0.0% (0.0 to 0.1)	3.1% (-3.2 to 8.7)	13.5% (1.6 to 25.6)	16.3% (12.9 to 19.9)	22.5% (8.0 to 37.7)
**El Salvador**	2.9% (0.7 to 5.8)	12.0% (6.4 to 18.7)	0.1% (0.0 to 0.2)	-1.1% (-4.3 to 1.8)	7.6% (0.6 to 19.7)	0.1% (0.0 to 0.1)	0.6% (-0.1 to 1.3)	5.4% (0.1 to 9.8)	0.0% (0.0 to 0.0)	1.5% (0.6 to 2.7)	1.3% (-1.2 to 3.6)	10.3% (0.3 to 21.2)	9.4% (7.4 to 11.6)	12.6% (4.4 to 20.0)
**Equatorial Guinea**	6.7% (1.6 to 13.2)	22.0% (12.2 to 33.1)	0.3% (0.1 to 0.5)	-3.0% (-12.0 to 4.3)	3.1% (0.0 to 12.1)	0.2% (0.1 to 0.3)	0.3% (-0.1 to 0.8)	3.5% (0.2 to 6.7)	0.0% (0.0 to 0.0)	5.7% (1.3 to 9.8)	1.2% (-1.1 to 3.4)	6.2% (0.3 to 12.9)	14.9% (11.7 to 18.3)	11.7% (3.9 to 20.3)
**Eritrea**	1.4% (0.2 to 3.1)	7.2% (4.0 to 12.0)	0.0% (0.0 to 0.1)	-1.1% (-4.7 to 1.9)	6.9% (0.2 to 18.9)	0.0% (0.0 to 0.0)	1.0% (-0.3 to 2.2)	6.8% (0.0 to 12.6)	0.0% (0.0 to 0.0)	8.5% (0.8 to 15.1)	1.0% (-0.9 to 2.9)	1.1% (0.1 to 2.6)	4.9% (3.7 to 6.2)	6.5% (2.1 to 11.6)
**Estonia**	9.3% (1.1 to 21.2)	2.9% (1.0 to 5.2)	2.6% (0.6 to 4.9)	-3.7% (-14.4 to 5.1)	2.2% (0.0 to 9.6)	0.2% (0.1 to 0.3)	0.6% (-0.1 to 1.4)	3.9% (0.6 to 6.7)	0.0% (0.0 to 0.0)	0.7% (0.3 to 1.2)	3.1% (-3.3 to 8.9)	9.8% (0.9 to 19.6)	11.6% (9.2 to 14.1)	25.7% (9.6 to 39.6)
**Eswatini**	4.3% (0.9 to 8.3)	13.1% (5.7 to 20.7)	0.1% (0.0 to 0.1)	-4.2% (-15.9 to 6.1)	2.6% (0.0 to 10.8)	0.0% (0.0 to 0.0)	1.6% (-0.4 to 3.6)	5.8% (0.1 to 10.8)	0.0% (0.0 to 0.0)	8.0% (1.3 to 14.2)	0.7% (-0.6 to 2.1)	10.3% (0.4 to 21.0)	9.8% (7.6 to 12.2)	8.1% (2.5 to 14.0)
**Ethiopia**	4.6% (0.9 to 9.2)	5.6% (3.4 to 8.0)	0.1% (0.0 to 0.1)	-1.0% (-4.0 to 1.6)	6.9% (0.3 to 18.7)	0.0% (0.0 to 0.0)	0.3% (-0.1 to 0.7)	12.4% (0.7 to 21.9)	0.0% (0.0 to 0.0)	7.6% (0.8 to 13.7)	1.4% (-1.3 to 4.3)	1.2% (0.0 to 3.0)	5.5% (4.1 to 7.0)	5.6% (1.7 to 10.2)
**Fiji**	2.4% (0.4 to 4.9)	8.4% (2.8 to 18.5)	0.1% (0.0 to 0.2)	-6.0% (-24.2 to 8.7)	8.7% (0.9 to 20.9)	0.1% (0.0 to 0.2)	0.7% (-0.2 to 1.6)	8.4% (0.3 to 15.0)	0.0% (0.0 to 0.0)	2.8% (0.5 to 5.3)	1.5% (-1.5 to 4.1)	11.0% (0.1 to 21.7)	15.3% (12.0 to 19.3)	9.2% (3.2 to 14.9)
**Finland**	8.0% (1.4 to 16.0)	1.5% (0.2 to 3.3)	0.8% (0.2 to 1.4)	-4.7% (-20.2 to 6.9)	4.6% (0.1 to 14.4)	0.2% (0.1 to 0.3)	1.7% (-0.3 to 3.7)	4.0% (0.3 to 7.3)	0.0% (0.0 to 0.0)	0.8% (0.1 to 1.4)	2.1% (-2.1 to 6.3)	7.0% (0.5 to 14.7)	12.6% (10.1 to 15.2)	18.0% (6.1 to 29.3)
**France**	10.5% (2.0 to 20.4)	5.7% (3.8 to 8.1)	1.3% (0.3 to 2.3)	-4.6% (-21.8 to 7.2)	3.7% (0.0 to 13.4)	0.2% (0.1 to 0.4)	2.8% (-0.6 to 6.1)	4.1% (0.3 to 7.4)	0.0% (0.0 to 0.0)	0.8% (0.2 to 1.5)	3.0% (-3.2 to 8.9)	6.3% (0.4 to 13.3)	9.3% (7.3 to 11.3)	18.6% (6.5 to 29.8)
**Gabon**	7.9% (1.7 to 15.6)	19.6% (11.2 to 29.5)	0.2% (0.0 to 0.4)	-5.7% (-23.5 to 7.8)	3.1% (0.0 to 12.6)	0.1% (0.0 to 0.2)	0.9% (-0.2 to 1.9)	2.6% (0.1 to 5.0)	0.0% (0.0 to 0.0)	5.1% (1.2 to 9.1)	1.0% (-0.9 to 2.8)	7.4% (0.4 to 15.4)	13.4% (10.8 to 16.4)	10.6% (3.3 to 18.2)
**Gambia**	4.6% (0.9 to 9.5)	9.5% (5.3 to 14.7)	0.2% (0.0 to 0.3)	-0.5% (-1.9 to 0.7)	3.8% (0.0 to 13.8)	0.0% (0.0 to 0.0)	1.4% (-0.3 to 2.9)	13.4% (2.3 to 22.5)	0.0% (0.0 to 0.0)	7.7% (1.8 to 13.0)	1.6% (-1.5 to 4.5)	4.8% (0.3 to 10.1)	9.2% (6.9 to 11.5)	11.8% (4.1 to 19.8)
**Georgia**	6.3% (1.3 to 13.4)	12.4% (6.8 to 18.3)	0.2% (0.1 to 0.4)	-2.4% (-9.0 to 3.3)	7.0% (0.3 to 18.7)	0.0% (0.0 to 0.1)	1.6% (-0.3 to 3.5)	4.4% (0.4 to 8.0)	0.0% (0.0 to 0.0)	0.6% (0.2 to 1.0)	3.7% (-4.1 to 10.4)	7.3% (0.5 to 15.1)	9.5% (7.4 to 11.7)	14.5% (4.8 to 24.3)
**Germany**	11.7% (1.8 to 23.4)	6.5% (4.4 to 9.0)	1.0% (0.2 to 1.8)	-3.5% (-16.6 to 5.8)	4.8% (0.1 to 15.0)	0.2% (0.1 to 0.3)	2.0% (-0.4 to 4.3)	3.4% (0.4 to 6.1)	0.0% (0.0 to 0.0)	0.9% (0.3 to 1.4)	2.2% (-2.2 to 6.6)	7.5% (0.6 to 15.5)	12.3% (9.7 to 15.0)	22.0% (7.8 to 35.0)
**Ghana**	6.3% (1.4 to 12.1)	16.9% (8.1 to 27.1)	0.4% (0.1 to 0.7)	-0.9% (-3.3 to 1.2)	5.6% (0.1 to 17.0)	0.1% (0.0 to 0.1)	0.0% (0.0 to 0.0)	2.7% (0.1 to 4.8)	0.0% (0.0 to 0.0)	2.7% (1.0 to 4.6)	2.2% (-2.3 to 6.3)	5.2% (0.3 to 10.6)	11.8% (9.4 to 14.5)	14.7% (5.2 to 23.8)

**Greece**	8.1% (1.7 to 15.2)	10.4% (7.6 to 13.5)	0.6% (0.1 to 1.0)	-5.5% (-23.8 to 8.2)	4.2% (0.0 to 14.2)	0.2% (0.1 to 0.4)	1.3% (-0.3 to 2.9)	1.9% (0.1 to 3.7)	0.0% (0.0 to 0.0)	0.1% (0.0 to 0.2)	2.3% (-2.5 to 7.5)	6.9% (0.3 to 15.2)	10.9% (8.6 to 13.2)	14.1% (4.6 to 23.6)
**Greenland**	7.2% (1.6 to 14.0)	2.6% (0.1 to 6.7)	0.4% (0.1 to 0.8)	-6.9% (-29.2 to 9.5)	4.9% (0.1 to 15.6)	0.1% (0.1 to 0.2)	1.7% (-0.4 to 3.8)	4.0% (0.0 to 7.4)	0.0% (0.0 to 0.0)	0.3% (0.0 to 0.5)	1.4% (-1.4 to 4.1)	8.0% (0.3 to 16.9)	6.9% (5.4 to 8.6)	12.6% (4.3 to 20.6)
**Grenada**	5.1% (1.1 to 10.1)	17.3% (6.8 to 32.3)	0.1% (0.0 to 0.1)	-1.2% (-4.5 to 1.9)	4.1% (0.0 to 13.9)	0.0% (0.0 to 0.1)	2.2% (-0.5 to 4.7)	3.3% (0.1 to 6.1)	0.0% (0.0 to 0.0)	4.9% (1.1 to 8.7)	2.6% (-2.7 to 7.7)	7.0% (0.3 to 14.7)	12.6% (9.9 to 15.4)	13.3% (4.4 to 22.0)
**Guam**	4.1% (0.5 to 10.1)	5.6% (2.7 to 9.0)	0.1% (0.0 to 0.2)	-7.5% (-30.5 to 10.2)	7.5% (0.9 to 18.6)	0.3% (0.1 to 0.4)	0.0% (0.0 to 0.0)	5.1% (0.1 to 9.3)	0.0% (0.0 to 0.0)	1.9% (0.7 to 3.2)	2.1% (-2.2 to 5.3)	11.9% (0.2 to 24.2)	12.2% (9.5 to 15.2)	11.2% (4.3 to 17.2)
**Guatemala**	2.4% (0.5 to 4.9)	11.4% (4.8 to 17.7)	0.1% (0.0 to 0.1)	-1.0% (-3.8 to 1.7)	7.5% (0.5 to 19.8)	0.0% (0.0 to 0.1)	0.7% (-0.1 to 1.5)	5.4% (0.0 to 9.8)	0.0% (0.0 to 0.0)	1.5% (0.4 to 2.5)	1.0% (-1.0 to 2.9)	8.5% (0.1 to 17.8)	9.0% (7.0 to 11.0)	9.0% (3.0 to 15.2)
**Guinea**	2.3% (0.4 to 5.0)	7.9% (4.5 to 12.3)	0.2% (0.0 to 0.3)	-0.8% (-3.0 to 1.2)	3.7% (0.0 to 13.5)	0.0% (0.0 to 0.0)	1.3% (-0.3 to 2.8)	4.3% (0.1 to 7.9)	0.0% (0.0 to 0.0)	4.2% (1.1 to 7.3)	1.3% (-1.2 to 3.7)	3.3% (0.2 to 7.0)	8.2% (6.3 to 10.3)	10.7% (3.6 to 18.0)
**Guinea-Bissau**	4.8% (1.1 to 9.2)	8.2% (4.6 to 12.8)	0.1% (0.0 to 0.2)	-2.3% (-9.1 to 3.6)	3.7% (0.0 to 13.6)	0.0% (0.0 to 0.0)	2.9% (-0.7 to 6.3)	6.1% (0.2 to 10.9)	0.0% (0.0 to 0.0)	10.5% (2.0 to 17.9)	1.1% (-1.0 to 3.2)	3.8% (0.3 to 7.8)	8.2% (6.2 to 10.2)	9.8% (3.5 to 16.6)
**Guyana**	5.2% (1.2 to 9.9)	17.6% (7.3 to 32.3)	0.2% (0.0 to 0.3)	-0.5% (-1.8 to 0.7)	4.0% (0.0 to 13.9)	0.1% (0.1 to 0.2)	3.1% (-0.7 to 6.6)	5.6% (0.2 to 10.0)	0.0% (0.0 to 0.0)	1.6% (0.6 to 2.7)	1.4% (-1.4 to 4.0)	7.0% (0.3 to 15.1)	15.9% (12.6 to 19.4)	11.9% (4.0 to 20.2)
**Haiti**	4.6% (1.0 to 9.3)	4.0% (1.7 to 7.7)	0.0% (0.0 to 0.1)	-1.0% (-3.8 to 1.6)	3.8% (0.0 to 13.3)	0.0% (0.0 to 0.0)	2.8% (-0.7 to 6.0)	5.3% (0.0 to 9.5)	0.0% (0.0 to 0.0)	9.3% (1.4 to 16.3)	1.5% (-1.4 to 4.4)	2.7% (0.2 to 6.0)	8.8% (6.7 to 11.1)	9.4% (3.1 to 15.5)
**Honduras**	2.4% (0.6 to 4.9)	8.1% (3.7 to 13.3)	0.1% (0.0 to 0.1)	-1.4% (-5.3 to 2.2)	7.9% (0.5 to 20.8)	0.1% (0.0 to 0.1)	1.1% (-0.2 to 2.4)	4.8% (0.0 to 8.8)	0.0% (0.0 to 0.0)	2.0% (0.5 to 3.4)	1.0% (-0.9 to 3.0)	7.0% (0.3 to 15.5)	9.3% (7.1 to 11.8)	10.9% (3.6 to 18.1)
**Hungary**	9.9% (1.3 to 20.7)	10.2% (7.5 to 13.3)	0.6% (0.1 to 1.1)	-3.6% (-14.3 to 4.8)	18.4% (6.6 to 32.5)	0.2% (0.1 to 0.4)	2.5% (-0.4 to 5.3)	3.6% (0.6 to 6.2)	0.0% (0.0 to 0.0)	0.5% (0.2 to 0.8)	3.7% (-3.9 to 10.5)	10.8% (1.1 to 21.2)	15.1% (12.0 to 18.5)	25.8% (9.5 to 39.9)
**Iceland**	8.3% (1.5 to 16.7)	1.5% (0.3 to 3.4)	1.2% (0.3 to 2.1)	-4.1% (-19.7 to 6.5)	4.8% (0.1 to 14.7)	0.3% (0.1 to 0.4)	3.5% (-0.7 to 7.4)	3.6% (0.3 to 6.5)	0.0% (0.0 to 0.0)	1.0% (0.1 to 1.8)	2.3% (-2.4 to 7.1)	7.2% (0.5 to 15.2)	11.8% (9.3 to 14.4)	18.9% (6.4 to 30.8)
**India**	3.3% (0.8 to 6.7)	18.6% (11.2 to 24.6)	0.0% (0.0 to 0.1)	-0.2% (-0.6 to 0.2)	6.3% (0.4 to 17.9)	0.1% (0.0 to 0.1)	2.4% (-0.6 to 5.0)	13.2% (1.5 to 22.6)	0.0% (0.0 to 0.0)	1.7% (0.6 to 2.7)	1.3% (-1.2 to 4.1)	2.1% (0.2 to 4.4)	10.6% (8.1 to 13.3)	9.7% (3.5 to 16.8)
**Indonesia**	0.4% (0.1 to 0.9)	12.2% (7.3 to 16.8)	0.0% (0.0 to 0.1)	-0.5% (-1.9 to 0.7)	11.8% (2.2 to 25.2)	0.0% (0.0 to 0.0)	4.6% (-1.1 to 9.7)	5.5% (0.2 to 10.1)	0.0% (0.0 to 0.0)	2.5% (0.6 to 4.2)	0.7% (-0.7 to 2.0)	2.2% (0.2 to 4.8)	8.4% (6.4 to 10.6)	8.9% (2.9 to 15.4)
**Iran (Islamic Republic of)**	0.5% (0.1 to 1.3)	23.2% (18.5 to 27.8)	0.1% (0.0 to 0.3)	-1.1% (-3.9 to 1.3)	1.6% (0.0 to 7.6)	0.1% (0.1 to 0.2)	0.6% (-0.1 to 1.3)	1.4% (0.3 to 2.4)	0.0% (0.0 to 0.0)	0.3% (0.0 to 0.5)	5.9% (-6.9 to 16.2)	10.0% (1.2 to 19.3)	16.0% (12.9 to 19.5)	25.2% (8.9 to 40.2)
**Iraq**	0.2% (0.0 to 0.4)	25.8% (18.7 to 33.5)	0.1% (0.0 to 0.2)	-0.4% (-1.4 to 0.5)	1.7% (0.0 to 8.0)	0.1% (0.0 to 0.1)	1.8% (-0.3 to 3.8)	3.6% (0.5 to 6.3)	0.0% (0.0 to 0.0)	0.3% (0.1 to 0.6)	4.3% (-4.7 to 11.8)	10.6% (0.9 to 21.5)	20.5% (16.8 to 24.3)	20.1% (7.0 to 32.5)
**Ireland**	8.0% (1.5 to 16.4)	3.9% (2.0 to 6.1)	0.9% (0.2 to 1.6)	-4.4% (-21.7 to 7.0)	2.9% (0.0 to 11.3)	0.4% (0.2 to 0.6)	1.2% (-0.3 to 2.7)	3.4% (0.2 to 6.3)	0.0% (0.0 to 0.0)	0.4% (0.0 to 0.8)	1.1% (-1.0 to 3.5)	6.8% (0.4 to 14.1)	10.9% (8.8 to 13.2)	15.9% (5.2 to 26.6)
**Israel**	3.7% (0.6 to 7.7)	14.2% (10.9 to 18.0)	0.4% (0.1 to 0.7)	-4.2% (-17.1 to 5.8)	4.5% (0.0 to 14.7)	0.3% (0.1 to 0.4)	0.5% (-0.1 to 1.1)	1.9% (0.1 to 3.6)	0.0% (0.0 to 0.0)	0.0% (0.0 to 0.1)	2.9% (-3.1 to 8.6)	6.5% (0.4 to 13.8)	12.7% (10.2 to 15.5)	15.5% (5.4 to 25.4)
**Italy**	9.6% (1.9 to 18.4)	9.8% (7.4 to 12.7)	1.1% (0.3 to 1.9)	-4.3% (-18.8 to 6.3)	5.6% (0.3 to 16.1)	0.2% (0.1 to 0.4)	1.8% (-0.4 to 3.8)	2.2% (0.1 to 4.1)	0.0% (0.0 to 0.0)	0.4% (0.0 to 0.7)	2.8% (-3.1 to 8.8)	5.4% (0.4 to 11.8)	12.1% (9.5 to 14.8)	16.4% (5.4 to 27.3)
**Jamaica**	3.8% (0.9 to 7.4)	11.9% (6.2 to 19.2)	0.0% (0.0 to 0.1)	-1.4% (-5.3 to 2.1)	4.1% (0.0 to 13.6)	0.0% (0.0 to 0.1)	1.4% (-0.3 to 2.9)	4.1% (0.1 to 7.6)	0.0% (0.0 to 0.0)	0.9% (0.3 to 1.6)	2.0% (-2.0 to 5.9)	8.3% (0.3 to 17.1)	8.2% (6.4 to 10.1)	11.5% (3.7 to 19.5)
**Japan**	7.6% (1.5 to 14.4)	8.4% (4.7 to 13.2)	0.8% (0.2 to 1.5)	-3.5% (-13.6 to 5.0)	9.4% (1.0 to 22.3)	0.2% (0.1 to 0.3)	2.9% (-0.7 to 6.0)	6.3% (0.4 to 11.1)	0.0% (0.0 to 0.0)	0.2% (0.1 to 0.4)	1.6% (-1.5 to 4.4)	2.5% (0.2 to 5.4)	10.9% (8.7 to 13.1)	13.6% (4.9 to 21.7)

**Jordan**	0.2% (0.0 to 0.6)	21.7% (16.6 to 27.4)	0.2% (0.0 to 0.3)	-1.2% (-4.3 to 1.5)	1.5% (0.0 to 7.5)	0.2% (0.1 to 0.3)	1.7% (-0.3 to 3.6)	2.9% (0.6 to 5.0)	0.0% (0.0 to 0.0)	0.8% (0.3 to 1.3)	5.2% (-5.9 to 14.5)	13.2% (1.6 to 24.8)	22.0% (17.9 to 26.2)	24.5% (8.7 to 38.8)
**Kazakhstan**	5.6% (1.1 to 12.4)	15.3% (10.3 to 21.3)	0.3% (0.1 to 0.5)	-4.2% (-19.6 to 6.7)	6.6% (0.3 to 18.1)	0.1% (0.1 to 0.2)	1.7% (-0.3 to 3.7)	4.2% (0.4 to 7.5)	0.0% (0.0 to 0.0)	0.1% (0.0 to 0.3)	4.0% (-4.4 to 11.3)	8.2% (0.6 to 16.8)	11.0% (8.7 to 13.4)	16.4% (5.6 to 26.8)
**Kenya**	5.6% (1.1 to 10.5)	5.5% (2.9 to 8.7)	0.1% (0.0 to 0.2)	-2.1% (-8.4 to 3.3)	4.6% (0.3 to 13.0)	0.0% (0.0 to 0.0)	0.6% (-0.1 to 1.3)	5.7% (0.2 to 10.4)	0.0% (0.0 to 0.0)	2.2% (0.5 to 3.8)	1.0% (-1.0 to 3.0)	3.4% (0.2 to 7.3)	6.5% (4.9 to 8.2)	7.3% (2.3 to 13.1)
**Kiribati**	1.0% (0.0 to 3.0)	2.6% (1.0 to 6.1)	0.1% (0.0 to 0.1)	-2.3% (-9.5 to 3.7)	7.8% (0.9 to 19.0)	0.0% (0.0 to 0.1)	1.3% (-0.3 to 2.8)	6.8% (-0.1 to 12.4)	0.0% (0.0 to 0.0)	5.4% (0.5 to 10.0)	1.4% (-1.6 to 3.7)	11.7% (0.0 to 23.3)	12.9% (9.8 to 16.3)	7.0% (2.5 to 11.3)
**Kuwait**	0.1% (0.0 to 0.3)	31.6% (25.1 to 38.0)	0.2% (0.1 to 0.4)	-3.2% (-12.2 to 3.8)	3.6% (0.0 to 12.3)	0.3% (0.1 to 0.5)	1.3% (-0.2 to 2.8)	2.9% (0.6 to 5.0)	0.0% (0.0 to 0.0)	0.2% (0.0 to 0.4)	4.0% (-4.3 to 11.1)	14.5% (1.6 to 26.9)	19.9% (16.2 to 24.0)	23.6% (8.7 to 37.0)
**Kyrgyzstan**	4.5% (0.9 to 9.9)	12.2% (5.9 to 19.5)	0.4% (0.1 to 0.7)	-5.1% (-19.9 to 6.8)	6.1% (0.3 to 17.9)	0.1% (0.0 to 0.2)	1.6% (-0.3 to 3.7)	5.6% (0.8 to 9.7)	0.0% (0.0 to 0.0)	0.3% (0.1 to 0.5)	5.5% (-6.3 to 14.2)	10.0% (0.8 to 20.2)	8.5% (6.5 to 10.5)	18.8% (7.0 to 29.7)
**Lao People's Democratic Republic**	7.6% (2.1 to 14.3)	10.4% (4.2 to 17.0)	0.0% (0.0 to 0.0)	-2.9% (-11.1 to 4.2)	11.6% (1.9 to 25.7)	0.0% (0.0 to 0.0)	6.9% (-1.8 to 14.3)	5.1% (0.0 to 9.4)	0.0% (0.0 to 0.0)	1.3% (0.4 to 2.2)	0.5% (-0.5 to 1.5)	2.1% (0.2 to 4.9)	8.1% (6.2 to 10.1)	9.8% (3.3 to 16.1)
**Latvia**	9.7% (1.1 to 22.5)	8.4% (5.6 to 11.5)	3.2% (0.6 to 6.0)	-2.9% (-11.4 to 3.6)	4.5% (0.1 to 14.2)	0.1% (0.1 to 0.2)	1.5% (-0.2 to 3.4)	3.8% (0.9 to 6.6)	0.0% (0.0 to 0.0)	0.7% (0.2 to 1.2)	3.7% (-3.8 to 11.1)	9.4% (1.0 to 18.8)	12.6% (9.9 to 15.6)	25.7% (8.9 to 41.5)
**Lebanon**	1.6% (0.3 to 3.6)	14.6% (9.4 to 20.9)	0.1% (0.0 to 0.2)	-3.2% (-12.1 to 4.1)	1.6% (0.0 to 7.5)	0.1% (0.1 to 0.2)	0.5% (-0.1 to 1.0)	2.0% (0.2 to 3.7)	0.0% (0.0 to 0.0)	0.0% (0.0 to 0.1)	4.5% (-5.2 to 12.3)	11.0% (0.9 to 21.6)	16.5% (13.4 to 19.8)	20.3% (7.6 to 31.7)
**Lesotho**	4.7% (0.9 to 9.4)	6.0% (3.1 to 10.0)	0.0% (0.0 to 0.1)	-3.0% (-12.0 to 4.5)	2.6% (0.0 to 10.8)	0.0% (0.0 to 0.1)	0.0% (0.0 to 0.1)	10.3% (0.7 to 18.2)	0.0% (0.0 to 0.0)	7.5% (1.0 to 13.5)	0.5% (-0.4 to 1.4)	6.9% (0.3 to 14.6)	7.3% (5.7 to 9.2)	6.3% (1.9 to 11.2)
**Liberia**	5.0% (1.1 to 9.7)	5.9% (3.8 to 8.4)	0.2% (0.0 to 0.3)	-0.5% (-1.7 to 0.7)	3.8% (0.0 to 13.6)	0.0% (0.0 to 0.0)	4.8% (-1.2 to 10.0)	6.2% (0.3 to 11.1)	0.0% (0.0 to 0.0)	8.2% (1.6 to 14.1)	1.6% (-1.5 to 4.7)	7.3% (0.3 to 15.0)	9.9% (7.5 to 12.4)	10.4% (3.6 to 17.4)
**Libya**	0.2% (0.0 to 0.6)	22.7% (16.0 to 30.3)	0.1% (0.0 to 0.2)	-1.6% (-5.7 to 2.0)	1.4% (0.0 to 7.5)	0.1% (0.0 to 0.1)	1.5% (-0.3 to 3.0)	3.0% (0.4 to 5.1)	0.0% (0.0 to 0.0)	0.7% (0.4 to 1.2)	5.0% (-5.8 to 13.4)	13.1% (1.2 to 24.4)	13.2% (10.4 to 16.0)	19.7% (7.0 to 31.0)
**Lithuania**	9.5% (1.1 to 22.1)	6.2% (3.9 to 9.0)	3.4% (0.7 to 6.3)	-3.2% (-13.3 to 4.6)	4.4% (0.0 to 15.2)	0.2% (0.1 to 0.3)	0.6% (-0.1 to 1.5)	3.6% (0.6 to 6.2)	0.0% (0.0 to 0.0)	0.7% (0.3 to 1.2)	3.5% (-3.5 to 9.8)	9.8% (1.0 to 19.6)	10.8% (8.6 to 13.3)	26.5% (9.5 to 41.4)
**Luxembourg**	11.1% (2.3 to 21.0)	4.4% (2.5 to 6.6)	1.0% (0.2 to 1.8)	-4.2% (-19.7 to 6.7)	4.3% (0.0 to 14.3)	0.2% (0.1 to 0.3)	1.9% (-0.4 to 4.2)	3.3% (0.2 to 6.1)	0.0% (0.0 to 0.0)	0.5% (0.0 to 1.0)	1.6% (-1.6 to 5.1)	6.4% (0.4 to 13.7)	12.8% (10.2 to 15.5)	16.9% (5.7 to 27.8)
**Madagascar**	2.3% (0.4 to 4.8)	2.3% (1.4 to 3.5)	0.0% (0.0 to 0.1)	-1.7% (-6.8 to 2.9)	6.8% (0.2 to 18.7)	0.0% (0.0 to 0.0)	3.2% (-0.8 to 6.8)	7.0% (0.0 to 12.7)	0.0% (0.0 to 0.0)	9.9% (1.1 to 17.7)	0.6% (-0.6 to 1.9)	2.0% (0.1 to 4.4)	5.1% (3.8 to 6.6)	6.4% (2.1 to 11.0)
**Malawi**	3.5% (0.8 to 6.9)	3.4% (2.1 to 5.5)	0.1% (0.0 to 0.1)	-0.6% (-2.3 to 1.0)	7.1% (0.3 to 19.1)	0.0% (0.0 to 0.0)	0.0% (0.0 to 0.1)	5.6% (0.1 to 10.2)	0.0% (0.0 to 0.0)	7.4% (1.1 to 12.9)	0.6% (-0.6 to 1.9)	2.3% (0.2 to 5.2)	5.6% (4.2 to 7.1)	8.9% (3.1 to 15.1)
**Malaysia**	1.4% (0.3 to 2.9)	12.8% (8.5 to 18.1)	0.2% (0.0 to 0.3)	-1.5% (-5.8 to 2.2)	12.1% (2.0 to 25.9)	0.1% (0.0 to 0.2)	4.8% (-1.1 to 10.2)	4.7% (0.1 to 8.5)	0.0% (0.0 to 0.0)	1.4% (0.6 to 2.4)	1.6% (-1.6 to 4.5)	5.1% (0.3 to 10.4)	13.1% (10.2 to 16.3)	14.8% (5.5 to 23.0)
**Maldives**	1.0% (0.1 to 2.4)	8.1% (4.7 to 12.4)	0.0% (0.0 to 0.1)	-0.3% (-1.2 to 0.5)	11.7% (1.9 to 24.9)	0.0% (0.0 to 0.0)	4.4% (-1.0 to 9.2)	4.9% (0.2 to 9.0)	0.0% (0.0 to 0.0)	0.7% (0.2 to 1.2)	2.0% (-2.1 to 5.9)	3.8% (0.2 to 8.3)	10.0% (7.8 to 12.3)	13.7% (4.7 to 21.9)
**Mali**	1.5% (0.3 to 3.2)	9.6% (5.3 to 15.2)	0.3% (0.1 to 0.6)	-4.1% (-16.4 to 6.2)	3.5% (0.0 to 13.1)	0.0% (0.0 to 0.1)	0.3% (-0.1 to 0.6)	6.4% (0.2 to 11.4)	0.0% (0.0 to 0.0)	6.3% (1.2 to 10.9)	1.4% (-1.3 to 4.5)	2.5% (0.2 to 5.3)	10.3% (7.8 to 12.8)	9.2% (3.1 to 15.6)
**Malta**	7.0% (1.4 to 14.4)	8.2% (5.8 to 11.1)	1.0% (0.2 to 1.8)	-4.7% (-19.8 to 6.5)	5.6% (0.3 to 16.4)	0.3% (0.1 to 0.4)	1.3% (-0.3 to 2.9)	3.6% (0.2 to 6.6)	0.0% (0.0 to 0.0)	0.2% (-0.1 to 0.4)	2.9% (-3.3 to 8.8)	6.3% (0.4 to 13.2)	11.8% (9.5 to 14.4)	17.6% (6.0 to 28.6)
**Marshall Islands**	2.4% (0.3 to 5.4)	4.8% (1.7 to 10.2)	0.0% (0.0 to 0.1)	-4.6% (-19.1 to 6.9)	7.9% (0.9 to 19.1)	0.0% (0.0 to 0.0)	0.5% (-0.1 to 1.2)	7.5% (-0.2 to 13.8)	0.0% (0.0 to 0.0)	7.8% (0.3 to 14.2)	1.2% (-1.2 to 3.3)	11.9% (-0.1 to 24.1)	14.3% (10.6 to 18.0)	6.6% (2.5 to 11.0)

**Mauritania**	0.0% (0.0 to 0.0)	21.2% (11.5 to 32.0)	0.3% (0.1 to 0.5)	-3.6% (-13.6 to 5.1)	3.7% (0.0 to 13.4)	0.0% (0.0 to 0.1)	0.8% (-0.2 to 1.9)	10.9% (1.6 to 18.5)	0.0% (0.0 to 0.0)	7.3% (1.8 to 12.4)	2.8% (-2.8 to 8.4)	7.5% (0.4 to 15.8)	8.6% (6.5 to 10.8)	12.4% (4.3 to 20.9)
**Mauritius**	4.9% (1.1 to 9.0)	5.2% (1.8 to 9.4)	0.1% (0.0 to 0.1)	-2.3% (-8.6 to 3.5)	11.4% (1.8 to 25.0)	0.1% (0.1 to 0.2)	3.8% (-0.9 to 8.1)	7.7% (0.6 to 13.5)	0.0% (0.0 to 0.0)	1.0% (0.5 to 1.6)	2.1% (-2.2 to 5.7)	6.5% (0.3 to 13.6)	13.4% (10.6 to 16.2)	14.9% (5.5 to 23.1)
**Mexico**	4.1% (0.9 to 8.3)	9.8% (6.6 to 13.7)	0.2% (0.0 to 0.3)	-5.5% (-23.2 to 7.8)	5.2% (0.1 to 15.6)	0.2% (0.1 to 0.4)	0.5% (-0.1 to 1.0)	3.9% (0.1 to 6.9)	0.0% (0.0 to 0.0)	1.3% (0.5 to 2.2)	1.0% (-0.9 to 2.8)	10.3% (0.4 to 21.1)	12.5% (10.0 to 15.0)	14.2% (5.1 to 22.6)
**Micronesia (Federated States of)**	2.6% (0.3 to 5.7)	5.2% (1.7 to 12.0)	0.0% (0.0 to 0.1)	-4.5% (-18.0 to 6.3)	7.9% (0.8 to 19.2)	0.0% (0.0 to 0.0)	0.5% (-0.1 to 1.3)	7.6% (-0.1 to 13.8)	0.0% (0.0 to 0.0)	8.1% (0.5 to 14.6)	1.3% (-1.4 to 3.6)	12.8% (-0.1 to 25.4)	9.9% (7.2 to 12.5)	7.0% (2.5 to 11.2)
**Monaco**	7.0% (0.0 to 18.6)	5.5% (3.0 to 8.7)	2.2% (0.5 to 3.9)	-3.6% (-17.2 to 5.7)	4.1% (0.0 to 14.0)	1.1% (0.5 to 1.8)	0.3% (-0.1 to 0.7)	1.4% (0.1 to 2.6)	0.0% (0.0 to 0.0)	0.1% (0.0 to 0.2)	2.5% (-2.6 to 7.7)	7.8% (0.6 to 16.1)	13.3% (10.8 to 16.4)	21.0% (7.3 to 34.2)
**Mongolia**	6.9% (0.8 to 13.6)	20.1% (9.9 to 28.0)	0.2% (0.0 to 0.3)	-10.9% (-46.9 to 15.2)	6.7% (0.4 to 18.7)	0.0% (0.0 to 0.1)	6.9% (-2.0 to 14.7)	13.0% (0.3 to 23.0)	0.0% (0.0 to 0.0)	2.1% (0.3 to 3.8)	1.6% (-1.9 to 4.2)	5.7% (0.1 to 12.9)	5.1% (3.4 to 6.9)	6.2% (2.1 to 9.9)
**Montenegro**	7.6% (1.6 to 14.7)	13.2% (8.5 to 17.3)	0.4% (0.1 to 0.7)	-5.5% (-22.4 to 8.2)	15.6% (4.3 to 29.9)	0.1% (0.0 to 0.1)	0.9% (-0.2 to 2.0)	2.1% (0.0 to 4.0)	0.0% (0.0 to 0.0)	0.0% (0.0 to 0.1)	1.1% (-1.1 to 3.5)	7.5% (0.1 to 16.1)	11.4% (8.4 to 14.6)	8.3% (2.6 to 14.1)
**Morocco**	0.1% (0.0 to 0.2)	14.9% (10.4 to 19.9)	0.1% (0.0 to 0.3)	-1.7% (-6.1 to 2.2)	1.5% (0.0 to 7.8)	0.1% (0.0 to 0.2)	0.0% (0.0 to 0.1)	1.9% (0.3 to 3.4)	0.0% (0.0 to 0.0)	0.4% (0.2 to 0.8)	5.3% (-5.8 to 14.9)	8.5% (0.8 to 16.7)	19.8% (15.7 to 23.9)	21.6% (7.4 to 34.1)
**Mozambique**	3.2% (0.6 to 6.5)	3.4% (2.1 to 5.2)	0.0% (0.0 to 0.1)	-0.6% (-2.2 to 0.9)	7.1% (0.2 to 19.4)	0.0% (0.0 to 0.0)	0.9% (-0.2 to 2.1)	9.0% (0.5 to 16.0)	0.0% (0.0 to 0.0)	8.8% (1.2 to 15.5)	1.5% (-1.5 to 4.1)	2.7% (0.2 to 5.8)	5.4% (4.0 to 6.8)	8.9% (2.9 to 14.8)
**Myanmar**	3.9% (0.8 to 7.4)	13.3% (6.1 to 20.8)	0.0% (0.0 to 0.0)	-2.1% (-8.2 to 3.2)	11.9% (1.9 to 25.4)	0.0% (0.0 to 0.0)	6.4% (-1.7 to 13.4)	7.2% (0.2 to 12.9)	0.0% (0.0 to 0.0)	0.9% (0.4 to 1.5)	0.7% (-0.7 to 2.1)	1.6% (0.1 to 3.8)	9.1% (6.9 to 11.2)	9.2% (3.2 to 15.1)
**Namibia**	8.3% (1.8 to 15.7)	13.1% (6.2 to 19.5)	0.1% (0.0 to 0.1)	-3.9% (-15.4 to 5.9)	2.5% (0.0 to 10.7)	0.0% (0.0 to 0.0)	2.7% (-0.7 to 5.8)	7.7% (0.8 to 13.9)	0.0% (0.0 to 0.0)	4.6% (1.0 to 8.1)	1.3% (-1.2 to 3.9)	6.5% (0.3 to 13.7)	9.9% (7.7 to 12.3)	9.8% (3.0 to 16.8)
**Nauru**	4.7% (0.8 to 10.0)	2.5% (0.4 to 5.5)	0.1% (0.0 to 0.1)	-5.6% (-22.6 to 8.3)	7.8% (0.8 to 19.5)	0.1% (0.0 to 0.1)	0.1% (0.0 to 0.4)	6.5% (0.0 to 11.8)	0.0% (0.0 to 0.0)	5.6% (0.5 to 10.2)	1.5% (-1.6 to 4.0)	15.8% (-0.1 to 30.2)	11.2% (8.3 to 14.0)	8.6% (3.1 to 13.6)
**Nepal**	3.0% (0.6 to 6.7)	12.6% (7.5 to 18.2)	0.3% (0.1 to 0.5)	-1.8% (-6.9 to 2.9)	5.9% (0.1 to 17.6)	0.0% (0.0 to 0.0)	1.7% (-0.4 to 3.8)	5.7% (0.1 to 10.4)	0.0% (0.0 to 0.0)	1.6% (0.6 to 2.9)	1.0% (-0.8 to 3.0)	1.7% (0.1 to 3.8)	10.8% (8.2 to 13.6)	9.7% (3.3 to 16.6)
**Netherlands**	9.9% (1.6 to 19.9)	7.2% (5.0 to 9.8)	0.8% (0.2 to 1.4)	-3.4% (-16.1 to 5.6)	3.8% (0.0 to 13.4)	0.3% (0.2 to 0.5)	1.9% (-0.4 to 4.3)	2.9% (0.3 to 5.4)	0.0% (0.0 to 0.0)	0.9% (0.2 to 1.6)	2.2% (-2.3 to 6.8)	5.8% (0.5 to 12.1)	10.8% (8.4 to 13.4)	20.1% (6.8 to 32.7)
**New Zealand**	9.1% (1.4 to 18.6)	3.4% (1.4 to 5.7)	1.3% (0.3 to 2.2)	-4.3% (-21.1 to 6.9)	3.7% (0.0 to 12.8)	0.3% (0.2 to 0.5)	2.2% (-0.4 to 4.7)	3.3% (0.2 to 6.0)	0.0% (0.0 to 0.0)	0.6% (0.1 to 1.1)	1.8% (-1.9 to 5.8)	8.2% (0.5 to 16.8)	13.4% (10.7 to 16.3)	17.9% (6.0 to 29.3)
**Nicaragua**	3.4% (0.8 to 6.8)	7.3% (3.1 to 12.7)	0.1% (0.0 to 0.1)	-0.8% (-3.1 to 1.2)	8.0% (0.5 to 20.6)	0.1% (0.0 to 0.1)	1.8% (-0.5 to 3.8)	8.2% (0.7 to 14.5)	0.0% (0.0 to 0.0)	9.2% (1.8 to 15.9)	1.1% (-1.1 to 3.2)	9.6% (0.4 to 19.7)	9.5% (7.5 to 11.6)	12.7% (4.6 to 20.7)
**Niger**	0.4% (0.0 to 1.2)	8.5% (4.7 to 13.5)	0.2% (0.0 to 0.3)	-3.4% (-13.7 to 5.4)	3.7% (0.0 to 13.1)	0.0% (0.0 to 0.0)	0.5% (-0.1 to 1.1)	9.1% (0.7 to 16.2)	0.0% (0.0 to 0.0)	1.1% (0.4 to 2.0)	1.4% (-1.3 to 4.5)	2.2% (0.1 to 4.7)	8.2% (5.8 to 10.6)	8.0% (2.6 to 14.7)
**Nigeria**	6.1% (1.2 to 12.2)	16.8% (8.3 to 25.7)	0.2% (0.0 to 0.3)	-0.9% (-3.1 to 1.2)	3.2% (0.0 to 12.3)	0.0% (0.0 to 0.0)	0.1% (0.0 to 0.3)	4.6% (0.4 to 8.2)	0.0% (0.0 to 0.0)	1.3% (0.5 to 2.3)	2.0% (-1.9 to 5.8)	4.8% (0.4 to 10.0)	8.7% (6.7 to 10.7)	13.1% (4.5 to 22.3)
**Niue**	4.5% (0.7 to 9.5)	2.9% (0.3 to 6.2)	0.1% (0.0 to 0.2)	-4.6% (-18.2 to 6.2)	8.4% (1.0 to 19.8)	0.1% (0.1 to 0.2)	0.1% (0.0 to 0.2)	5.4% (0.0 to 9.8)	0.0% (0.0 to 0.0)	4.5% (0.6 to 7.9)	1.4% (-1.5 to 4.0)	11.4% (0.0 to 22.6)	14.9% (11.5 to 18.3)	7.9% (2.7 to 13.4)
**North Macedonia**	7.1% (1.1 to 14.4)	18.5% (12.5 to 23.2)	0.6% (0.1 to 1.0)	-1.6% (-6.1 to 2.0)	15.6% (4.1 to 30.0)	0.2% (0.1 to 0.4)	0.8% (-0.1 to 1.9)	2.0% (0.2 to 3.7)	0.0% (0.0 to 0.0)	0.1% (0.0 to 0.2)	3.0% (-3.0 to 9.8)	7.6% (0.7 to 15.5)	17.8% (14.3 to 21.5)	21.2% (7.0 to 35.6)
**Northern Mariana Islands**	3.3% (0.1 to 8.1)	5.8% (2.8 to 9.2)	0.1% (0.0 to 0.1)	-5.7% (-23.5 to 8.2)	9.0% (1.0 to 21.4)	0.1% (0.1 to 0.2)	0.0% (0.0 to 0.1)	4.8% (0.0 to 9.0)	0.0% (0.0 to 0.0)	2.7% (0.5 to 4.9)	1.4% (-1.4 to 3.9)	12.7% (0.0 to 25.0)	12.5% (9.6 to 15.5)	8.7% (3.1 to 14.4)

**Norway**	7.8% (1.3 to 16.3)	2.3% (0.9 to 4.2)	3.3% (0.8 to 5.7)	-3.4% (-15.4 to 5.3)	3.6% (0.0 to 12.6)	0.4% (0.2 to 0.6)	1.7% (-0.3 to 3.7)	2.7% (0.2 to 5.0)	0.0% (0.0 to 0.0)	1.1% (0.2 to 2.1)	2.7% (-2.8 to 8.2)	5.2% (0.4 to 10.7)	13.0% (10.4 to 15.9)	19.6% (6.7 to 31.8)
**Oman**	0.3% (0.0 to 0.8)	29.2% (21.2 to 37.8)	0.2% (0.0 to 0.3)	-2.9% (-10.7 to 3.9)	1.7% (0.0 to 8.4)	0.1% (0.1 to 0.2)	0.6% (-0.1 to 1.3)	1.6% (0.3 to 2.8)	0.0% (0.0 to 0.0)	0.6% (0.3 to 1.0)	3.6% (-3.8 to 10.2)	11.7% (1.2 to 22.9)	18.1% (14.7 to 21.6)	23.6% (8.3 to 37.2)
**Pakistan**	0.7% (0.1 to 1.5)	17.1% (8.7 to 25.8)	0.3% (0.1 to 0.6)	-2.0% (-7.8 to 3.1)	6.0% (0.1 to 17.7)	0.0% (0.0 to 0.0)	4.4% (-1.1 to 9.2)	6.7% (0.4 to 11.8)	0.0% (0.0 to 0.0)	5.3% (1.1 to 9.4)	3.0% (-3.2 to 8.5)	3.9% (0.3 to 8.3)	12.4% (9.7 to 15.3)	11.2% (3.9 to 19.1)
**Palau**	4.5% (0.6 to 10.7)	3.6% (0.3 to 7.5)	0.1% (0.0 to 0.2)	-5.6% (-22.9 to 7.6)	8.2% (0.8 to 20.0)	0.1% (0.1 to 0.2)	0.1% (0.0 to 0.2)	5.8% (0.0 to 10.3)	0.0% (0.0 to 0.0)	4.7% (0.8 to 8.3)	1.7% (-1.8 to 4.8)	12.9% (0.1 to 25.4)	15.3% (12.1 to 18.5)	10.1% (3.6 to 16.3)
**Palestine**	0.6% (0.1 to 1.4)	20.0% (13.7 to 27.5)	0.1% (0.0 to 0.1)	-0.6% (-2.1 to 0.7)	1.4% (0.0 to 7.3)	0.0% (0.0 to 0.0)	3.2% (-0.6 to 6.5)	2.8% (0.5 to 5.0)	0.0% (0.0 to 0.0)	0.8% (0.1 to 1.5)	4.3% (-4.7 to 12.7)	11.2% (1.2 to 21.9)	16.4% (13.1 to 19.9)	21.7% (7.4 to 35.0)
**Panama**	4.5% (1.1 to 9.1)	7.7% (4.2 to 12.2)	0.1% (0.0 to 0.2)	-5.5% (-21.8 to 7.5)	8.4% (0.6 to 21.1)	0.1% (0.0 to 0.1)	5.2% (-1.3 to 10.8)	4.8% (0.1 to 8.8)	0.0% (0.0 to 0.0)	3.8% (0.8 to 6.6)	0.8% (-0.7 to 2.4)	9.4% (0.4 to 19.6)	10.5% (8.2 to 12.9)	12.3% (4.1 to 20.5)
**Papua New Guinea**	1.1% (0.2 to 2.6)	5.0% (1.5 to 11.7)	0.0% (0.0 to 0.0)	-3.0% (-11.7 to 4.7)	8.6% (0.9 to 20.9)	0.0% (0.0 to 0.0)	0.1% (0.0 to 0.3)	6.7% (-0.2 to 12.4)	0.0% (0.0 to 0.0)	3.2% (0.3 to 5.9)	1.1% (-1.1 to 3.3)	3.4% (0.1 to 8.0)	7.5% (5.4 to 9.7)	5.4% (1.8 to 9.2)
**Paraguay**	7.7% (1.8 to 14.6)	6.8% (2.5 to 12.9)	0.1% (0.0 to 0.2)	-7.0% (-30.2 to 9.8)	6.8% (0.2 to 18.9)	0.1% (0.0 to 0.1)	0.6% (-0.1 to 1.3)	3.8% (0.0 to 7.1)	0.0% (0.0 to 0.0)	2.9% (0.7 to 5.0)	1.2% (-1.1 to 3.6)	8.1% (0.3 to 17.4)	11.3% (9.0 to 13.8)	13.9% (4.7 to 23.0)
**Peru**	5.4% (1.1 to 10.3)	19.1% (11.6 to 26.4)	0.0% (0.0 to 0.1)	-2.0% (-7.7 to 3.2)	5.9% (0.2 to 16.8)	0.1% (0.0 to 0.1)	1.4% (-0.3 to 3.0)	5.1% (0.0 to 9.0)	0.0% (0.0 to 0.0)	1.8% (0.6 to 3.1)	1.2% (-1.2 to 3.4)	7.9% (0.2 to 16.8)	6.8% (5.3 to 8.5)	10.8% (3.8 to 17.6)
**Philippines**	8.0% (1.9 to 14.8)	12.3% (6.7 to 16.7)	0.1% (0.0 to 0.1)	-4.3% (-17.2 to 6.3)	11.5% (2.2 to 24.7)	0.0% (0.0 to 0.1)	7.3% (-1.9 to 14.9)	5.0% (0.0 to 9.1)	0.0% (0.0 to 0.0)	1.8% (0.5 to 3.2)	0.8% (-0.8 to 2.2)	3.6% (0.2 to 7.5)	6.7% (5.1 to 8.4)	10.6% (3.8 to 16.8)
**Poland**	9.4% (1.6 to 19.2)	13.7% (10.4 to 17.2)	0.7% (0.2 to 1.1)	-3.5% (-16.8 to 5.5)	11.5% (2.0 to 25.0)	0.4% (0.2 to 0.7)	0.8% (-0.1 to 1.8)	3.9% (0.6 to 6.8)	0.0% (0.0 to 0.0)	0.2% (0.0 to 0.5)	3.0% (-3.0 to 8.6)	8.2% (0.7 to 16.8)	16.6% (13.4 to 20.0)	21.1% (7.4 to 34.1)
**Portugal**	9.6% (2.0 to 17.9)	4.0% (2.1 to 6.1)	0.5% (0.1 to 0.8)	-5.2% (-23.8 to 7.9)	4.4% (0.1 to 14.0)	0.2% (0.1 to 0.3)	1.5% (-0.3 to 3.4)	3.2% (0.1 to 6.0)	0.0% (0.0 to 0.0)	0.2% (0.0 to 0.4)	1.9% (-1.9 to 5.9)	6.3% (0.4 to 13.5)	13.9% (11.2 to 16.7)	16.5% (5.4 to 27.3)
**Puerto Rico**	4.4% (1.1 to 8.6)	2.6% (0.7 to 4.7)	0.2% (0.0 to 0.3)	-3.4% (-13.4 to 4.7)	4.1% (0.0 to 13.8)	0.1% (0.1 to 0.2)	2.3% (-0.5 to 5.0)	4.0% (0.1 to 7.1)	0.0% (0.0 to 0.0)	5.4% (1.2 to 9.2)	2.1% (-2.2 to 6.0)	11.3% (0.4 to 22.4)	14.7% (11.9 to 17.8)	13.4% (4.8 to 21.6)
**Qatar**	0.4% (0.1 to 1.0)	40.6% (32.2 to 48.4)	0.3% (0.1 to 0.5)	-2.6% (-10.0 to 3.6)	1.6% (0.0 to 7.9)	0.6% (0.3 to 1.0)	0.0% (0.0 to 0.1)	1.0% (0.1 to 1.9)	0.0% (0.0 to 0.0)	0.0% (0.0 to 0.1)	3.1% (-3.3 to 9.2)	12.1% (1.0 to 23.4)	23.6% (19.7 to 27.6)	16.7% (5.6 to 27.4)
**Republic of Korea**	7.4% (1.3 to 15.0)	18.9% (12.9 to 25.9)	0.5% (0.1 to 0.8)	-4.7% (-19.7 to 6.4)	13.3% (2.9 to 27.1)	0.2% (0.1 to 0.3)	4.5% (-1.0 to 9.5)	3.6% (0.3 to 6.5)	0.0% (0.0 to 0.0)	0.3% (0.0 to 0.6)	1.1% (-1.0 to 3.4)	2.6% (0.3 to 5.6)	13.1% (10.3 to 15.7)	15.7% (5.3 to 26.1)
**Republic of Moldova**	10.2% (2.0 to 21.9)	9.0% (4.3 to 15.1)	0.8% (0.2 to 1.4)	-2.4% (-8.7 to 3.5)	3.3% (0.0 to 12.4)	0.0% (0.0 to 0.1)	1.2% (-0.2 to 2.5)	4.7% (0.4 to 8.3)	0.0% (0.0 to 0.0)	0.8% (0.4 to 1.3)	2.6% (-2.6 to 7.2)	10.8% (0.8 to 21.5)	10.8% (8.3 to 13.1)	16.5% (5.7 to 26.7)
**Romania**	10.0% (1.9 to 19.7)	10.6% (7.7 to 13.6)	1.2% (0.3 to 2.2)	-4.1% (-18.0 to 6.2)	16.2% (4.6 to 30.8)	0.6% (0.3 to 0.9)	0.6% (-0.1 to 1.3)	3.3% (0.3 to 5.9)	0.0% (0.0 to 0.0)	0.0% (0.0 to 0.1)	2.1% (-2.0 to 6.3)	8.4% (0.6 to 17.1)	14.5% (11.6 to 17.7)	20.1% (6.9 to 32.7)
**Russian Federation**	6.9% (1.1 to 15.3)	7.6% (4.6 to 11.5)	1.3% (0.3 to 2.3)	-3.5% (-14.5 to 4.9)	7.1% (0.5 to 18.9)	0.1% (0.1 to 0.2)	1.9% (-0.3 to 4.0)	4.1% (0.6 to 7.1)	0.0% (0.0 to 0.0)	0.9% (0.4 to 1.5)	3.6% (-3.7 to 10.3)	9.8% (0.9 to 19.4)	11.2% (8.9 to 13.9)	24.2% (8.7 to 37.8)
**Rwanda**	6.3% (1.3 to 12.4)	3.8% (1.9 to 6.9)	0.0% (0.0 to 0.1)	-0.6% (-2.4 to 1.0)	7.1% (0.2 to 19.6)	0.0% (0.0 to 0.0)	0.0% (0.0 to 0.0)	0.3% (0.0 to 0.6)	0.0% (0.0 to 0.0)	5.3% (0.7 to 9.4)	1.3% (-1.3 to 3.8)	1.4% (0.1 to 3.6)	4.7% (3.5 to 5.9)	5.4% (1.7 to 9.6)
**Saint Kitts and Nevis**	3.0% (0.0 to 8.8)	3.9% (1.4 to 6.7)	0.1% (0.0 to 0.2)	-2.5% (-9.4 to 3.5)	4.3% (0.0 to 14.6)	0.1% (0.0 to 0.1)	6.0% (-1.3 to 12.4)	5.6% (0.5 to 10.1)	0.0% (0.0 to 0.0)	2.0% (0.6 to 3.5)	2.3% (-2.4 to 7.0)	8.1% (0.4 to 16.7)	13.5% (10.7 to 16.5)	14.1% (4.6 to 23.9)
**Saint Lucia**	6.8% (1.7 to 13.1)	16.4% (6.6 to 29.5)	0.1% (0.0 to 0.2)	-4.0% (-16.1 to 5.6)	4.3% (0.0 to 14.5)	0.1% (0.0 to 0.1)	3.9% (-0.9 to 8.2)	3.9% (0.1 to 7.1)	0.0% (0.0 to 0.0)	4.4% (1.0 to 7.8)	2.5% (-2.6 to 7.4)	6.9% (0.3 to 14.6)	13.2% (10.7 to 15.9)	11.9% (3.8 to 20.7)

**Saint Vincent and the Grenadines**	7.7% (1.9 to 14.2)	16.5% (6.2 to 31.1)	0.1% (0.0 to 0.1)	-2.1% (-8.0 to 3.1)	4.2% (0.0 to 14.1)	0.1% (0.0 to 0.1)	2.8% (-0.7 to 6.0)	3.6% (0.1 to 6.6)	0.0% (0.0 to 0.0)	3.3% (0.7 to 5.7)	2.1% (-2.2 to 6.1)	5.7% (0.3 to 12.1)	12.0% (9.4 to 14.6)	11.1% (3.6 to 18.9)
**Samoa**	2.1% (0.3 to 4.5)	6.3% (1.8 to 14.5)	0.1% (0.0 to 0.1)	-5.3% (-20.9 to 7.5)	2.8% (0.0 to 10.4)	0.1% (0.0 to 0.1)	0.0% (0.0 to 0.1)	6.6% (-0.1 to 11.9)	0.0% (0.0 to 0.0)	10.2% (0.8 to 18.4)	1.6% (-1.7 to 4.4)	13.4% (-0.1 to 26.4)	12.5% (9.3 to 15.7)	6.5% (2.2 to 10.6)
**San Marino**	9.4% (-0.1 to 22.3)	6.3% (3.4 to 9.8)	1.4% (0.3 to 2.6)	-3.4% (-15.3 to 5.3)	4.2% (0.0 to 14.0)	0.4% (0.2 to 0.7)	1.5% (-0.3 to 3.4)	2.7% (0.3 to 4.9)	0.0% (0.0 to 0.0)	0.5% (0.1 to 1.0)	2.9% (-3.1 to 8.5)	7.4% (0.6 to 15.2)	13.2% (10.6 to 16.2)	20.9% (7.0 to 33.5)
**Sao Tome and Principe**	6.4% (1.4 to 12.4)	8.2% (4.4 to 13.8)	0.2% (0.1 to 0.4)	-0.2% (-0.7 to 0.3)	3.8% (0.0 to 14.0)	0.0% (0.0 to 0.0)	0.0% (0.0 to 0.1)	3.0% (0.2 to 5.4)	0.0% (0.0 to 0.0)	6.1% (1.6 to 10.2)	2.9% (-3.1 to 7.9)	6.6% (0.4 to 13.6)	9.5% (7.5 to 11.9)	12.7% (4.3 to 21.6)
**Saudi Arabia**	0.1% (0.0 to 0.4)	34.9% (27.4 to 42.6)	0.1% (0.0 to 0.3)	-1.5% (-5.3 to 1.8)	1.7% (0.0 to 8.0)	0.5% (0.2 to 0.8)	0.7% (-0.1 to 1.4)	3.8% (0.5 to 6.6)	0.0% (0.0 to 0.0)	0.9% (0.5 to 1.4)	3.8% (-4.1 to 10.3)	14.6% (1.2 to 27.7)	16.6% (13.5 to 20.0)	19.3% (7.0 to 30.9)
**Senegal**	0.8% (0.1 to 1.8)	8.3% (4.0 to 14.4)	0.2% (0.1 to 0.4)	-1.3% (-5.0 to 2.0)	3.8% (0.0 to 13.3)	0.0% (0.0 to 0.0)	0.2% (0.0 to 0.5)	7.7% (0.9 to 13.5)	0.0% (0.0 to 0.0)	2.6% (0.8 to 4.4)	1.7% (-1.6 to 5.1)	4.2% (0.3 to 8.9)	11.8% (9.2 to 14.6)	11.0% (3.6 to 18.1)
**Serbia**	7.2% (1.1 to 15.7)	16.4% (11.3 to 20.7)	0.4% (0.1 to 0.8)	-2.2% (-8.1 to 2.7)	16.0% (4.4 to 30.2)	0.2% (0.1 to 0.3)	1.3% (-0.2 to 2.9)	2.0% (0.3 to 3.6)	0.0% (0.0 to 0.0)	0.6% (0.1 to 1.1)	3.1% (-3.0 to 9.2)	8.8% (0.9 to 17.4)	17.0% (13.6 to 20.6)	25.1% (8.7 to 41.1)
**Seychelles**	4.6% (1.2 to 8.5)	5.4% (1.7 to 9.9)	0.1% (0.0 to 0.1)	-1.1% (-4.4 to 1.5)	9.9% (1.3 to 22.6)	0.0% (0.0 to 0.1)	2.7% (-0.6 to 5.8)	4.9% (0.3 to 8.8)	0.0% (0.0 to 0.0)	4.6% (1.1 to 7.6)	1.3% (-1.2 to 3.6)	8.6% (0.4 to 17.6)	14.6% (11.4 to 17.8)	16.0% (5.7 to 25.4)
**Sierra Leone**	3.9% (0.9 to 7.6)	6.2% (3.8 to 9.6)	0.1% (0.0 to 0.3)	-0.3% (-1.3 to 0.5)	3.7% (0.0 to 13.5)	0.0% (0.0 to 0.0)	5.3% (-1.2 to 11.1)	6.5% (0.6 to 11.7)	0.0% (0.0 to 0.0)	4.5% (1.2 to 7.6)	1.5% (-1.5 to 4.1)	3.5% (0.3 to 7.6)	8.3% (6.3 to 10.2)	11.0% (3.8 to 18.2)
**Singapore**	2.2% (0.3 to 4.6)	12.0% (6.4 to 18.7)	0.4% (0.1 to 0.6)	-5.9% (-24.4 to 8.2)	10.9% (1.7 to 24.3)	0.2% (0.1 to 0.3)	3.1% (-0.7 to 6.7)	3.4% (0.2 to 6.2)	0.0% (0.0 to 0.0)	0.3% (0.1 to 0.6)	1.4% (-1.4 to 3.9)	5.1% (0.3 to 10.7)	13.0% (10.5 to 15.8)	16.3% (6.1 to 26.0)
**Slovakia**	10.6% (1.6 to 21.4)	11.2% (8.4 to 14.4)	1.2% (0.3 to 2.1)	-3.5% (-14.3 to 4.8)	16.1% (4.8 to 30.3)	0.3% (0.2 to 0.5)	3.1% (-0.6 to 6.6)	3.6% (0.6 to 6.3)	0.0% (0.0 to 0.0)	0.7% (0.2 to 1.2)	3.2% (-3.3 to 9.3)	9.7% (0.9 to 19.7)	15.7% (12.4 to 18.9)	22.9% (7.9 to 36.0)
**Slovenia**	7.1% (0.6 to 16.8)	9.6% (6.9 to 12.7)	2.4% (0.6 to 4.2)	-2.7% (-11.8 to 4.1)	16.2% (4.8 to 30.3)	0.5% (0.2 to 0.8)	1.3% (-0.2 to 2.7)	2.2% (0.3 to 3.8)	0.0% (0.0 to 0.0)	0.8% (0.2 to 1.5)	2.1% (-2.1 to 6.5)	8.5% (0.8 to 17.1)	16.8% (13.7 to 20.2)	22.2% (7.4 to 36.5)
**Solomon Islands**	1.1% (0.1 to 2.5)	3.0% (1.0 to 6.8)	0.0% (0.0 to 0.0)	-1.2% (-4.7 to 2.0)	8.4% (0.9 to 20.2)	0.0% (0.0 to 0.0)	0.1% (0.0 to 0.2)	7.1% (-0.2 to 13.1)	0.0% (0.0 to 0.0)	11.3% (0.7 to 20.5)	0.9% (-0.9 to 2.7)	6.3% (0.1 to 14.0)	6.5% (4.6 to 8.2)	5.8% (2.0 to 9.9)
**Somalia**	0.0% (0.0 to 0.0)	3.2% (2.1 to 4.6)	0.0% (0.0 to 0.1)	-3.3% (-13.8 to 5.3)	7.0% (0.2 to 19.1)	0.0% (0.0 to 0.0)	6.8% (-1.9 to 14.2)	10.4% (0.2 to 18.8)	0.0% (0.0 to 0.0)	10.9% (0.8 to 19.9)	1.1% (-1.1 to 3.6)	1.7% (0.0 to 3.9)	4.8% (3.4 to 6.2)	4.9% (1.6 to 9.0)
**South Africa**	5.9% (1.4 to 11.2)	15.1% (10.6 to 20.0)	0.1% (0.0 to 0.2)	-4.5% (-18.2 to 6.3)	2.3% (0.0 to 10.0)	0.1% (0.0 to 0.1)	1.1% (-0.3 to 2.4)	7.4% (0.8 to 13.0)	0.0% (0.0 to 0.0)	2.6% (0.8 to 4.5)	0.9% (-0.8 to 2.5)	9.7% (0.5 to 19.5)	10.4% (8.2 to 12.7)	12.4% (4.1 to 20.9)
**South Sudan**	0.2% (-0.1 to 0.7)	6.6% (3.6 to 10.8)	0.1% (0.0 to 0.1)	-1.7% (-6.7 to 2.6)	6.9% (0.2 to 19.1)	0.0% (0.0 to 0.1)	0.2% (0.0 to 0.4)	6.2% (0.0 to 11.5)	0.0% (0.0 to 0.0)	5.5% (0.7 to 9.9)	0.9% (-0.9 to 2.6)	0.5% (-0.2 to 1.6)	5.6% (4.2 to 7.1)	6.2% (2.0 to 10.7)
**Spain**	8.5% (1.6 to 16.4)	5.3% (3.4 to 7.6)	0.8% (0.2 to 1.5)	-4.7% (-20.5 to 7.1)	2.7% (0.1 to 10.0)	0.2% (0.1 to 0.4)	2.4% (-0.5 to 5.0)	2.3% (0.2 to 4.4)	0.0% (0.0 to 0.0)	0.4% (0.0 to 0.7)	2.0% (-2.0 to 6.1)	7.9% (0.5 to 16.5)	12.3% (9.8 to 14.9)	16.5% (5.6 to 27.1)
**Sri Lanka**	3.0% (0.7 to 5.9)	13.3% (6.0 to 19.4)	0.0% (0.0 to 0.1)	-0.1% (-0.3 to 0.1)	11.4% (1.6 to 24.7)	0.0% (0.0 to 0.1)	1.3% (-0.3 to 2.8)	5.1% (0.6 to 9.2)	0.0% (0.0 to 0.0)	2.1% (0.7 to 3.8)	1.3% (-1.2 to 3.9)	3.2% (0.3 to 6.4)	12.4% (9.9 to 15.1)	17.3% (5.8 to 28.3)
**Sudan**	0.0% (0.0 to 0.0)	19.2% (10.3 to 30.0)	0.1% (0.0 to 0.2)	-1.4% (-5.0 to 1.7)	1.6% (0.0 to 7.8)	0.0% (0.0 to 0.1)	2.5% (-0.5 to 5.3)	3.9% (0.5 to 6.9)	0.0% (0.0 to 0.0)	1.3% (0.7 to 2.2)	4.7% (-5.1 to 13.2)	9.7% (0.7 to 19.6)	13.5% (10.6 to 16.6)	16.9% (5.9 to 27.6)
**Suriname**	4.9% (1.1 to 9.3)	20.5% (8.7 to 34.2)	0.1% (0.0 to 0.1)	-1.0% (-4.2 to 1.6)	3.9% (0.0 to 13.4)	0.1% (0.0 to 0.1)	7.0% (-1.8 to 14.5)	5.3% (0.0 to 9.6)	0.0% (0.0 to 0.0)	2.5% (0.6 to 4.4)	1.2% (-1.2 to 3.4)	5.8% (0.3 to 12.9)	11.3% (8.8 to 13.8)	11.4% (4.0 to 18.4)
**Sweden**	9.1% (1.7 to 18.3)	2.1% (0.6 to 4.0)	1.7% (0.4 to 3.3)	-3.5% (-15.8 to 5.4)	4.3% (0.0 to 14.3)	0.2% (0.1 to 0.4)	1.8% (-0.3 to 3.9)	3.1% (0.3 to 5.6)	0.0% (0.0 to 0.0)	0.9% (0.2 to 1.6)	2.6% (-2.9 to 8.1)	5.9% (0.4 to 12.3)	11.8% (9.3 to 14.4)	19.5% (6.6 to 31.4)

**Switzerland**	9.9% (1.6 to 20.0)	5.4% (3.5 to 7.6)	0.6% (0.2 to 1.1)	-3.4% (-16.4 to 5.7)	4.0% (0.0 to 13.6)	0.2% (0.1 to 0.3)	3.1% (-0.6 to 6.9)	3.0% (0.3 to 5.6)	0.0% (0.0 to 0.0)	0.7% (0.1 to 1.4)	2.8% (-3.0 to 8.4)	5.1% (0.4 to 10.7)	12.5% (9.9 to 15.2)	20.1% (6.8 to 32.3)
**Syrian Arab Republic**	0.5% (0.1 to 1.3)	18.6% (13.3 to 24.3)	0.1% (0.0 to 0.2)	-1.7% (-6.2 to 2.4)	1.6% (0.0 to 7.8)	0.1% (0.0 to 0.1)	1.9% (-0.4 to 4.0)	3.3% (0.3 to 5.8)	0.0% (0.0 to 0.0)	0.9% (0.4 to 1.4)	4.1% (-4.7 to 11.2)	13.0% (0.9 to 25.0)	14.1% (11.4 to 17.1)	17.6% (6.4 to 28.2)
**Turkey**	1.3% (0.3 to 3.0)	17.2% (13.2 to 21.3)	0.2% (0.0 to 0.3)	-1.5% (-5.8 to 2.1)	1.1% (0.0 to 6.4)	0.2% (0.1 to 0.4)	0.1% (0.0 to 0.3)	1.2% (0.1 to 2.3)	0.0% (0.0 to 0.0)	0.0% (0.0 to 0.0)	3.7% (-4.1 to 10.8)	10.1% (0.7 to 20.0)	14.7% (11.9 to 17.9)	16.8% (5.8 to 27.5)
**Taiwan (Province of China)**	5.2% (1.2 to 9.7)	12.2% (9.2 to 15.5)	0.2% (0.0 to 0.4)	-6.9% (-31.0 to 9.8)	7.9% (0.6 to 19.9)	0.1% (0.0 to 0.2)	3.5% (-0.8 to 7.4)	3.6% (0.1 to 6.5)	0.0% (0.0 to 0.0)	0.3% (0.1 to 0.5)	2.1% (-2.1 to 5.6)	5.2% (0.4 to 11.3)	11.0% (8.6 to 13.3)	15.5% (5.8 to 24.5)
**Tajikistan**	1.2% (0.2 to 2.4)	13.5% (7.4 to 20.8)	0.3% (0.1 to 0.6)	-1.3% (-5.2 to 2.0)	6.3% (0.3 to 17.9)	0.0% (0.0 to 0.0)	3.9% (-0.9 to 8.0)	5.3% (0.5 to 9.5)	0.0% (0.0 to 0.0)	0.2% (0.0 to 0.4)	3.8% (-4.3 to 10.7)	7.4% (0.5 to 15.4)	10.6% (8.3 to 13.1)	14.3% (4.8 to 23.3)
**Thailand**	5.3% (1.2 to 9.7)	21.2% (15.2 to 27.3)	0.0% (0.0 to 0.1)	-2.8% (-10.9 to 4.1)	10.7% (1.7 to 24.1)	0.1% (0.0 to 0.1)	7.3% (-2.0 to 14.6)	4.4% (0.0 to 7.8)	0.0% (0.0 to 0.0)	2.5% (0.8 to 4.3)	0.8% (-0.8 to 2.2)	4.5% (0.3 to 9.3)	7.9% (6.3 to 10.0)	12.5% (4.7 to 19.7)
**Timor-Leste**	3.4% (0.8 to 6.7)	8.8% (3.1 to 17.0)	0.0% (0.0 to 0.0)	-1.4% (-5.4 to 2.3)	11.9% (2.1 to 25.8)	0.0% (0.0 to 0.0)	3.1% (-0.7 to 6.9)	10.2% (0.6 to 18.0)	0.0% (0.0 to 0.0)	7.5% (0.9 to 13.4)	0.7% (-0.7 to 2.1)	0.5% (-0.1 to 1.4)	6.6% (5.0 to 8.4)	8.2% (2.6 to 14.2)
**Togo**	3.7% (0.9 to 7.5)	8.4% (5.1 to 13.2)	0.2% (0.0 to 0.3)	-0.5% (-1.8 to 0.7)	3.8% (0.0 to 13.6)	0.0% (0.0 to 0.0)	0.1% (0.0 to 0.2)	12.4% (1.9 to 21.2)	0.0% (0.0 to 0.0)	8.9% (1.9 to 15.1)	1.6% (-1.5 to 4.6)	4.2% (0.3 to 8.9)	7.5% (5.7 to 9.3)	11.4% (3.9 to 18.8)
**Tokelau**	3.0% (0.4 to 6.4)	2.4% (0.0 to 6.1)	0.1% (0.0 to 0.1)	-4.5% (-19.1 to 6.5)	7.9% (0.8 to 19.2)	0.1% (0.0 to 0.1)	0.2% (0.0 to 0.5)	6.2% (-0.1 to 11.4)	0.0% (0.0 to 0.0)	5.7% (0.6 to 10.4)	1.4% (-1.5 to 3.9)	11.8% (0.0 to 23.5)	14.6% (11.3 to 17.9)	8.1% (2.9 to 13.5)
**Tonga**	0.9% (0.1 to 2.2)	6.9% (2.3 to 15.2)	0.1% (0.0 to 0.1)	-3.7% (-14.8 to 5.5)	8.8% (0.8 to 21.2)	0.0% (0.0 to 0.1)	0.3% (-0.1 to 0.6)	5.8% (0.1 to 10.5)	0.0% (0.0 to 0.0)	6.3% (1.0 to 10.9)	1.7% (-1.7 to 4.8)	14.1% (0.2 to 26.5)	12.4% (9.7 to 15.5)	10.6% (3.8 to 17.4)
**Trinidad and Tobago**	5.5% (1.2 to 10.9)	17.5% (6.0 to 33.8)	0.1% (0.0 to 0.2)	-1.9% (-6.9 to 2.8)	4.3% (0.0 to 14.5)	0.1% (0.1 to 0.2)	2.6% (-0.6 to 5.6)	5.2% (0.3 to 9.2)	0.0% (0.0 to 0.0)	3.6% (1.0 to 6.0)	2.8% (-2.9 to 7.9)	8.9% (0.4 to 18.2)	13.0% (10.4 to 15.6)	15.4% (5.2 to 25.1)
**Tunisia**	1.0% (0.1 to 2.2)	17.3% (12.0 to 23.6)	0.1% (0.0 to 0.2)	-1.2% (-4.5 to 1.5)	1.5% (0.0 to 7.7)	0.1% (0.1 to 0.2)	0.3% (0.0 to 0.6)	2.4% (0.4 to 4.4)	0.0% (0.0 to 0.0)	0.2% (0.0 to 0.5)	4.5% (-4.9 to 12.6)	9.0% (0.9 to 17.9)	15.5% (12.3 to 18.7)	21.4% (7.2 to 34.3)
**Turkmenistan**	5.5% (1.1 to 11.5)	14.7% (8.3 to 22.3)	0.5% (0.1 to 0.9)	-5.5% (-26.3 to 8.7)	6.3% (0.3 to 17.6)	0.2% (0.1 to 0.3)	2.9% (-0.7 to 6.2)	4.3% (0.3 to 7.7)	0.0% (0.0 to 0.0)	0.1% (0.0 to 0.1)	4.5% (-5.0 to 11.6)	8.1% (0.6 to 17.0)	9.7% (7.5 to 12.1)	16.2% (5.9 to 26.6)
**Tuvalu**	2.3% (0.3 to 5.2)	1.7% (0.7 to 3.4)	0.0% (0.0 to 0.0)	-4.3% (-16.3 to 6.7)	8.1% (0.9 to 19.8)	0.0% (0.0 to 0.0)	0.5% (-0.1 to 1.2)	7.1% (-0.1 to 13.0)	0.0% (0.0 to 0.0)	7.6% (0.6 to 14.0)	1.4% (-1.5 to 3.9)	12.3% (-0.1 to 24.3)	7.4% (5.6 to 9.2)	7.6% (2.7 to 12.0)
**Uganda**	6.7% (1.3 to 12.9)	6.2% (3.6 to 9.7)	0.0% (0.0 to 0.1)	-1.3% (-5.5 to 2.2)	7.0% (0.2 to 18.9)	0.0% (0.0 to 0.0)	0.2% (0.0 to 0.5)	2.5% (0.0 to 4.8)	0.0% (0.0 to 0.0)	7.5% (1.1 to 13.3)	0.9% (-0.8 to 2.7)	2.0% (0.2 to 4.3)	6.2% (4.6 to 7.8)	6.6% (2.1 to 12.0)
**Ukraine**	7.3% (1.2 to 16.8)	10.9% (6.5 to 17.2)	0.4% (0.1 to 0.8)	-3.7% (-14.5 to 4.8)	3.2% (0.0 to 12.0)	0.1% (0.0 to 0.1)	1.4% (-0.3 to 3.0)	5.1% (1.0 to 8.6)	0.0% (0.0 to 0.0)	0.3% (0.1 to 0.6)	4.4% (-4.7 to 12.3)	10.0% (0.9 to 19.8)	10.0% (7.9 to 12.5)	24.0% (8.7 to 37.5)
**United Arab Emirates**	1.5% (0.2 to 3.7)	28.2% (21.0 to 35.9)	0.3% (0.1 to 0.6)	-1.9% (-7.2 to 2.5)	1.5% (0.0 to 8.2)	0.4% (0.2 to 0.7)	0.6% (-0.1 to 1.3)	2.0% (0.3 to 3.6)	0.0% (0.0 to 0.0)	0.6% (0.2 to 1.0)	3.5% (-3.6 to 10.3)	11.8% (1.2 to 22.8)	21.9% (17.8 to 25.9)	22.0% (7.4 to 35.6)
**United Kingdom**	8.9% (1.7 to 17.6)	5.5% (3.7 to 7.8)	0.7% (0.2 to 1.3)	-4.0% (-16.8 to 5.9)	3.3% (0.0 to 11.6)	0.1% (0.1 to 0.2)	2.4% (-0.6 to 5.2)	4.4% (0.4 to 8.0)	0.0% (0.0 to 0.0)	0.8% (0.2 to 1.4)	2.2% (-2.3 to 6.6)	8.0% (0.5 to 16.7)	9.2% (7.3 to 11.2)	16.5% (5.6 to 26.9)
**United Republic of Tanzania**	5.8% (1.4 to 11.1)	5.1% (2.8 to 8.2)	0.1% (0.0 to 0.2)	-0.9% (-3.2 to 1.3)	9.8% (0.8 to 23.3)	0.0% (0.0 to 0.0)	0.3% (-0.1 to 0.7)	4.5% (0.1 to 8.3)	0.0% (0.0 to 0.0)	4.2% (0.9 to 7.5)	0.8% (-0.7 to 2.3)	4.2% (0.3 to 8.7)	6.8% (5.2 to 8.6)	11.6% (3.7 to 19.3)
**United States of America**	7.0% (1.4 to 14.5)	3.2% (1.6 to 5.1)	1.8% (0.4 to 3.1)	-5.1% (-23.3 to 7.5)	4.8% (0.1 to 14.7)	0.4% (0.2 to 0.7)	2.2% (-0.4 to 4.8)	4.1% (0.3 to 7.3)	0.0% (0.0 to 0.0)	1.0% (0.4 to 1.6)	2.1% (-2.2 to 6.0)	11.2% (0.6 to 21.9)	15.0% (12.3 to 17.9)	15.1% (5.4 to 24.5)
**United States Virgin Islands**	6.3% (0.0 to 15.0)	3.8% (1.8 to 6.3)	0.1% (0.0 to 0.3)	-3.7% (-14.7 to 5.1)	4.1% (0.0 to 13.9)	0.2% (0.1 to 0.3)	1.9% (-0.5 to 4.3)	3.1% (0.1 to 5.6)	0.0% (0.0 to 0.0)	1.1% (0.4 to 1.9)	2.0% (-2.0 to 5.7)	10.6% (0.3 to 21.4)	14.0% (11.1 to 17.2)	12.8% (4.3 to 21.3)

**Uruguay**	6.9% (1.4 to 13.8)	6.2% (2.4 to 11.0)	0.5% (0.1 to 0.8)	-5.9% (-27.4 to 9.0)	7.0% (0.3 to 19.3)	0.3% (0.1 to 0.5)	2.3% (-0.5 to 4.8)	4.1% (0.2 to 7.3)	0.0% (0.0 to 0.0)	1.8% (0.6 to 3.0)	2.9% (-3.1 to 8.5)	8.5% (0.5 to 17.8)	10.1% (8.0 to 12.3)	16.5% (5.7 to 26.7)
**Uzbekistan**	3.9% (0.7 to 8.5)	22.2% (13.5 to 31.0)	0.3% (0.1 to 0.5)	-4.6% (-18.9 to 6.5)	6.4% (0.3 to 17.6)	0.1% (0.0 to 0.1)	1.7% (-0.3 to 3.7)	4.0% (0.4 to 7.1)	0.0% (0.0 to 0.0)	0.0% (0.0 to 0.0)	4.7% (-5.4 to 12.5)	8.5% (0.7 to 17.7)	10.2% (8.1 to 12.4)	18.2% (6.7 to 29.0)
**Vanuatu**	2.5% (0.3 to 5.5)	3.6% (1.2 to 8.1)	0.0% (0.0 to 0.1)	-7.3% (-29.7 to 10.6)	8.5% (0.9 to 20.1)	0.0% (0.0 to 0.0)	0.2% (0.0 to 0.4)	6.2% (-0.1 to 11.2)	0.0% (0.0 to 0.0)	6.2% (0.5 to 11.3)	1.4% (-1.4 to 3.9)	6.5% (0.2 to 14.7)	9.7% (7.1 to 12.3)	7.7% (2.8 to 12.6)
**Venezuela (Bolivarian Republic of)**	3.7% (0.9 to 7.4)	10.1% (5.8 to 15.4)	0.1% (0.0 to 0.2)	-5.0% (-20.8 to 7.4)	8.1% (0.6 to 20.5)	0.1% (0.0 to 0.1)	4.0% (-1.0 to 8.6)	4.8% (0.0 to 8.8)	0.0% (0.0 to 0.0)	1.6% (0.4 to 2.7)	0.8% (-0.7 to 2.4)	9.7% (0.2 to 20.3)	9.2% (7.2 to 11.2)	9.5% (3.1 to 16.2)
**Viet Nam**	9.6% (2.3 to 16.7)	11.9% (5.8 to 17.5)	0.0% (0.0 to 0.0)	-5.1% (-20.6 to 7.2)	12.6% (2.2 to 26.4)	0.0% (0.0 to 0.0)	6.7% (-1.6 to 14.0)	4.9% (0.1 to 9.0)	0.0% (0.0 to 0.0)	0.5% (0.1 to 0.8)	0.7% (-0.6 to 1.9)	0.8% (0.0 to 2.0)	7.8% (6.0 to 9.9)	11.3% (3.7 to 19.2)
**Yemen**	0.3% (0.0 to 0.6)	16.5% (9.1 to 25.1)	0.1% (0.0 to 0.1)	-0.9% (-3.1 to 1.3)	1.5% (0.0 to 7.8)	0.0% (0.0 to 0.0)	3.5% (-0.7 to 7.3)	5.5% (0.8 to 9.5)	0.0% (0.0 to 0.0)	4.8% (1.7 to 7.9)	4.6% (-5.0 to 12.9)	6.1% (0.5 to 11.9)	11.7% (9.2 to 14.2)	18.0% (6.2 to 28.8)
**Zambia**	5.7% (1.2 to 11.2)	7.6% (3.7 to 13.3)	0.1% (0.0 to 0.1)	-1.3% (-5.2 to 2.2)	7.0% (0.2 to 19.1)	0.0% (0.0 to 0.0)	0.5% (-0.1 to 1.1)	12.2% (1.1 to 21.8)	0.0% (0.0 to 0.0)	7.6% (1.0 to 13.5)	0.6% (-0.5 to 1.7)	3.9% (0.2 to 8.4)	8.2% (6.3 to 10.2)	6.4% (2.0 to 11.2)
**Zimbabwe**	4.3% (0.9 to 8.3)	4.3% (2.4 to 7.1)	0.0% (0.0 to 0.1)	-1.4% (-5.4 to 2.3)	3.9% (0.0 to 14.4)	0.0% (0.0 to 0.0)	0.7% (-0.2 to 1.7)	11.6% (1.2 to 20.3)	0.0% (0.0 to 0.0)	10.5% (1.6 to 18.3)	0.9% (-0.8 to 2.5)	5.9% (0.2 to 12.3)	7.7% (5.9 to 9.6)	8.7% (2.8 to 15.0)

Appendix Table 11 continues. Age-standardized percent of DALYs (with 95% uncertainty intervals) due to stroke associated with individual risk factors by 7 GBD super-region, 21 GBD region and country for both sexes combined in 2021.

Country/region	High systolic blood pressure	High temperature	Household air pollution from solid fuels	Kidney dysfunction	Lead exposure	Low physical activity	Low temperature	Secondhand smoke	Smoking
**GBD super-regions in alphabetical order**									
**Central Europe, Eastern Europe, and Central Asia**	62.6% (47.9 to 73.5)	0.1% (-0.5 to 0.7)	1.2% (0.3 to 4.5)	9.8% (7.1 to 12.5)	3.4% (-0.4 to 7.8)	2.3% (0.2 to 4.8)	8.6% (7.2 to 9.8)	3.6% (2.4 to 4.7)	14.3% (12.4 to 16.2)
**High-income**	51.8% (38.1 to 62.4)	0.2% (-0.2 to 0.8)	0.0% (0.0 to 0.2)	7.7% (5.3 to 10.1)	3.4% (-0.4 to 8.0)	2.3% (0.1 to 4.7)	6.2% (5.5 to 7.1)	2.5% (1.7 to 3.3)	13.1% (11.2 to 15.1)
**Latin America and Caribbean**	54.4% (40.2 to 65.6)	0.4% (0.2 to 0.7)	4.7% (2.6 to 9.1)	8.5% (6.4 to 10.6)	7.4% (-1.0 to 16.8)	2.1% (0.5 to 4.0)	2.5% (2.1 to 2.8)	2.8% (1.9 to 3.8)	9.1% (7.8 to 10.6)
**North Africa and Middle East**	56.7% (42.3 to 67.7)	3.1% (0.3 to 6.8)	3.6% (2.6 to 5.1)	10.1% (7.2 to 13.1)	9.2% (-1.3 to 20.6)	4.2% (1.5 to 7.6)	5.1% (4.2 to 6.2)	4.6% (3.1 to 6.1)	10.5% (9.0 to 12.2)
**South Asia**	55.6% (41.2 to 66.9)	2.8% (0.7 to 5.5)	24.9% (16.5 to 34.6)	10.4% (7.8 to 13.3)	10.6% (-1.4 to 23.5)	1.7% (0.4 to 3.3)	2.4% (0.9 to 4.1)	4.3% (3.0 to 5.7)	10.1% (8.5 to 11.9)
**Southeast Asia, East Asia, and Oceania**	57.2% (42.8 to 68.8)	0.7% (0.0 to 1.7)	8.2% (2.8 to 19.6)	8.8% (6.3 to 11.2)	7.7% (-1.0 to 17.2)	1.9% (0.3 to 3.9)	5.3% (4.7 to 6.0)	5.2% (3.5 to 6.9)	16.8% (14.3 to 19.5)
**Sub-Saharan Africa**	59.2% (44.7 to 70.0)	1.5% (0.9 to 2.2)	33.7% (26.3 to 41.2)	10.5% (7.9 to 13.2)	7.6% (-1.0 to 17.1)	1.4% (0.3 to 2.6)	1.8% (1.5 to 2.2)	2.3% (1.5 to 3.0)	5.2% (4.4 to 6.0)
**GBD regions in alphabetical order**									
**Andean Latin America**	45.3% (31.8 to 56.8)	0.1% (0.0 to 0.3)	3.7% (0.7 to 11.6)	5.8% (4.2 to 7.5)	6.1% (-0.8 to 13.9)	1.2% (0.2 to 2.3)	5.6% (4.9 to 6.6)	1.9% (1.2 to 2.5)	6.3% (5.3 to 7.6)
**Australasia**	51.8% (37.4 to 63.9)	0.1% (-0.1 to 0.4)	0.0% (0.0 to 0.0)	6.9% (4.4 to 9.4)	5.4% (-0.7 to 12.5)	2.9% (-0.1 to 6.3)	5.2% (4.4 to 6.2)	2.0% (1.4 to 2.7)	9.0% (7.6 to 10.8)
**Caribbean**	53.6% (39.7 to 64.8)	0.4% (0.3 to 0.6)	15.0% (10.9 to 19.8)	7.7% (5.8 to 9.7)	9.3% (-1.3 to 20.8)	1.9% (0.5 to 3.5)	0.3% (0.2 to 0.5)	2.4% (1.6 to 3.3)	8.9% (7.5 to 10.4)
**Central Asia**	62.7% (47.5 to 73.8)	0.5% (-0.4 to 1.7)	4.5% (1.8 to 10.2)	12.3% (9.2 to 15.4)	4.2% (-0.5 to 9.6)	1.7% (0.3 to 3.3)	8.2% (7.0 to 9.2)	4.4% (3.0 to 5.9)	11.8% (10.2 to 13.4)
**Central Europe**	62.6% (48.0 to 73.4)	0.1% (-0.3 to 0.9)	1.2% (0.0 to 7.7)	7.6% (5.2 to 10.0)	4.1% (-0.5 to 9.4)	2.5% (0.1 to 5.3)	8.6% (7.7 to 10.3)	3.8% (2.6 to 5.0)	13.4% (11.5 to 15.3)
**Central Latin America**	55.1% (40.9 to 66.2)	0.5% (0.2 to 0.8)	4.3% (1.7 to 9.6)	9.6% (7.2 to 12.1)	8.3% (-1.1 to 18.5)	1.5% (0.3 to 3.0)	3.1% (2.6 to 3.6)	2.7% (1.9 to 3.6)	6.8% (5.7 to 7.8)
**Central Sub-Saharan Africa**	57.8% (43.1 to 69.6)	0.3% (0.0 to 0.7)	36.1% (29.1 to 43.4)	12.5% (9.4 to 15.6)	7.3% (-1.0 to 16.5)	1.4% (0.2 to 2.7)	1.0% (0.9 to 1.3)	1.9% (1.3 to 2.6)	4.5% (3.6 to 5.5)
**East Asia**	55.9% (41.7 to 67.9)	0.5% (-0.3 to 1.9)	6.4% (1.7 to 18.6)	7.5% (5.3 to 9.8)	8.3% (-1.1 to 18.6)	1.9% (0.3 to 3.9)	7.1% (6.4 to 7.9)	5.2% (3.6 to 7.0)	17.9% (15.0 to 21.0)
**Eastern Europe**	62.6% (47.7 to 73.6)	-0.1% (-0.6 to 0.3)	0.4% (0.1 to 1.6)	10.2% (7.3 to 13.2)	2.9% (-0.4 to 6.7)	2.4% (0.2 to 4.9)	8.7% (7.1 to 10.2)	3.2% (2.2 to 4.4)	15.3% (13.4 to 17.3)

**Eastern Sub-Saharan Africa**	56.1% (41.8 to 67.1)	0.5% (0.1 to 1.2)	43.0% (35.8 to 49.9)	8.1% (5.8 to 10.6)	8.3% (-1.1 to 18.6)	0.7% (0.2 to 1.4)	2.7% (2.3 to 3.2)	2.1% (1.4 to 2.8)	5.9% (5.1 to 6.9)
**High-income Asia Pacific**	51.4% (37.8 to 62.2)	0.1% (-0.2 to 0.7)	0.0% (0.0 to 0.0)	8.0% (5.4 to 10.5)	3.1% (-0.4 to 7.2)	2.4% (0.7 to 4.5)	6.1% (5.3 to 6.8)	3.1% (2.1 to 4.2)	14.3% (12.2 to 16.3)
**High-income North America**	48.2% (34.5 to 60.3)	0.3% (-0.5 to 1.4)	0.0% (0.0 to 0.0)	8.6% (6.1 to 11.2)	2.9% (-0.4 to 6.8)	2.1% (0.1 to 4.5)	5.5% (4.7 to 6.2)	1.9% (1.3 to 2.6)	13.4% (11.3 to 15.6)
**North Africa and Middle East**	56.7% (42.3 to 67.7)	3.1% (0.3 to 6.8)	3.6% (2.6 to 5.1)	10.1% (7.2 to 13.1)	9.2% (-1.3 to 20.6)	4.2% (1.5 to 7.6)	5.1% (4.2 to 6.2)	4.6% (3.1 to 6.1)	10.5% (9.0 to 12.2)
**Oceania**	44.6% (31.2 to 56.3)	0.2% (0.1 to 0.3)	35.4% (26.7 to 43.5)	9.5% (7.2 to 11.8)	3.1% (-0.4 to 7.2)	1.0% (0.4 to 1.8)	2.5% (2.1 to 3.2)	6.0% (4.0 to 8.0)	11.8% (9.7 to 14.2)
**South Asia**	55.6% (41.2 to 66.9)	2.8% (0.7 to 5.5)	24.9% (16.5 to 34.6)	10.4% (7.8 to 13.3)	10.6% (-1.4 to 23.5)	1.7% (0.4 to 3.3)	2.4% (0.9 to 4.1)	4.3% (3.0 to 5.7)	10.1% (8.5 to 11.9)
**Southeast Asia**	61.7% (47.1 to 72.4)	1.1% (0.8 to 1.5)	12.8% (5.4 to 23.4)	12.1% (9.1 to 15.1)	6.0% (-0.8 to 13.6)	2.0% (0.5 to 3.8)	0.6% (0.4 to 0.9)	5.0% (3.4 to 6.6)	14.2% (12.2 to 16.3)
**Southern Latin America**	56.8% (42.8 to 68.0)	0.1% (-0.1 to 0.5)	0.3% (0.0 to 2.9)	6.2% (4.4 to 8.2)	3.7% (-0.5 to 8.3)	1.3% (0.1 to 2.6)	6.4% (5.9 to 7.0)	4.2% (2.9 to 5.6)	12.8% (10.9 to 14.7)
**Southern Sub-Saharan Africa**	63.3% (47.9 to 74.0)	0.2% (-0.2 to 0.8)	10.9% (7.0 to 17.8)	12.8% (9.8 to 15.8)	5.8% (-0.8 to 12.9)	2.6% (0.5 to 5.0)	5.6% (5.0 to 6.1)	4.0% (2.7 to 5.3)	8.2% (7.0 to 9.6)
**Tropical Latin America**	56.1% (41.4 to 67.6)	0.4% (0.2 to 0.8)	2.1% (0.4 to 5.9)	8.4% (6.4 to 10.5)	6.5% (-0.9 to 14.9)	2.6% (0.5 to 5.1)	2.1% (1.7 to 2.5)	3.2% (2.2 to 4.3)	11.4% (9.6 to 13.5)
**Western Europe**	55.2% (41.1 to 66.2)	0.1% (-0.1 to 0.3)	0.0% (0.0 to 0.0)	7.0% (4.8 to 9.3)	4.0% (-0.5 to 9.1)	2.5% (-0.3 to 5.5)	7.1% (6.3 to 8.5)	2.4% (1.6 to 3.1)	12.3% (10.5 to 14.3)
**Western Sub-Saharan Africa**	61.3% (46.6 to 71.9)	2.9% (2.1 to 4.0)	31.6% (22.5 to 40.6)	11.2% (8.5 to 14.0)	7.7% (-1.0 to 17.2)	1.6% (0.4 to 3.0)	0.4% (0.1 to 0.7)	2.0% (1.4 to 2.7)	4.0% (3.3 to 4.7)
**Countries in alphabetical order**									
Afghanistan	52.2% (36.9 to 65.5)	1.0% (-0.4 to 2.9)	38.5% (31.6 to 45.6)	10.2% (7.4 to 13.0)	14.0% (-2.0 to 30.1)	3.6% (1.4 to 6.1)	7.5% (6.4 to 8.5)	4.1% (2.7 to 5.5)	6.6% (5.2 to 8.3)
Albania	63.3% (48.4 to 75.0)	0.1% (-0.3 to 0.6)	4.3% (0.3 to 16.8)	7.6% (5.0 to 10.3)	5.3% (-0.7 to 11.8)	1.0% (-0.2 to 2.4)	8.5% (7.4 to 9.7)	4.5% (3.0 to 6.0)	16.0% (13.1 to 19.2)
Algeria	54.8% (40.2 to 67.2)	1.4% (0.0 to 3.3)	0.0% (0.0 to 0.2)	9.5% (6.5 to 12.6)	7.8% (-1.0 to 17.2)	3.9% (0.7 to 7.9)	6.4% (5.6 to 7.2)	5.4% (3.7 to 7.2)	9.5% (7.6 to 12.0)
American Samoa	59.5% (44.5 to 71.6)	0.1% (0.0 to 0.1)	1.0% (0.0 to 6.9)	10.8% (8.4 to 13.4)	1.5% (-0.2 to 3.3)	2.2% (0.9 to 3.9)	0.0% (-0.1 to 0.2)	5.7% (3.9 to 7.7)	14.2% (11.7 to 16.9)
Andorra	57.2% (42.3 to 68.9)	0.0% (0.0 to 0.0)	0.0% (0.0 to 0.0)	7.2% (4.9 to 9.6)	2.9% (-0.4 to 6.7)	2.5% (-0.4 to 5.6)	8.7% (7.5 to 9.8)	2.4% (1.6 to 3.3)	12.2% (9.7 to 15.0)
Angola	59.9% (45.2 to 71.9)	0.2% (-0.2 to 0.8)	14.7% (4.8 to 27.3)	12.2% (9.1 to 15.4)	7.0% (-1.0 to 15.9)	1.3% (0.2 to 2.7)	1.5% (1.2 to 1.9)	1.9% (1.2 to 2.5)	7.3% (5.8 to 8.8)
Antigua and Barbuda	55.4% (39.8 to 67.4)	0.4% (0.2 to 0.5)	0.1% (0.0 to 0.4)	8.5% (6.2 to 10.9)	5.3% (-0.7 to 12.0)	1.8% (0.0 to 3.9)	0.0% (-0.1 to 0.1)	2.1% (1.4 to 2.8)	6.8% (5.4 to 8.6)
Argentina	55.9% (42.0 to 67.5)	0.2% (-0.2 to 0.8)	0.2% (0.0 to 1.5)	6.1% (4.3 to 8.0)	4.0% (-0.5 to 8.9)	1.1% (0.1 to 2.4)	5.9% (5.4 to 6.4)	4.2% (2.8 to 5.5)	13.8% (11.7 to 16.1)

Armenia	61.9% (46.4 to 73.5)	0.1% (-0.6 to 1.0)	1.0% (0.1 to 3.9)	11.8% (8.6 to 15.1)	4.4% (-0.6 to 10.0)	2.5% (0.2 to 5.0)	7.7% (6.4 to 8.7)	5.1% (3.5 to 6.8)	17.2% (14.9 to 19.6)
Australia	51.9% (37.5 to 64.0)	0.1% (-0.1 to 0.5)	0.0% (0.0 to 0.0)	6.8% (4.4 to 9.4)	5.5% (-0.7 to 12.7)	3.0% (0.1 to 6.4)	4.8% (4.1 to 5.8)	2.0% (1.3 to 2.7)	8.7% (7.2 to 10.4)
Austria	57.7% (42.8 to 69.9)	0.0% (-0.2 to 0.3)	0.0% (0.0 to 0.0)	7.3% (4.9 to 9.6)	3.2% (-0.4 to 7.4)	2.5% (-0.2 to 5.4)	6.6% (5.6 to 7.5)	2.8% (1.9 to 3.7)	14.1% (11.8 to 16.3)
Azerbaijan	62.3% (46.7 to 73.6)	0.2% (-0.6 to 1.1)	0.7% (0.0 to 4.8)	12.9% (9.6 to 16.2)	4.1% (-0.5 to 8.9)	1.3% (0.2 to 2.8)	8.0% (6.6 to 8.9)	5.6% (3.8 to 7.5)	12.9% (10.3 to 15.6)
Bahamas	55.4% (40.9 to 68.3)	0.7% (-0.2 to 1.9)	0.0% (0.0 to 0.3)	8.1% (6.0 to 10.2)	3.4% (-0.4 to 8.1)	1.9% (0.3 to 3.9)	0.4% (0.1 to 0.7)	2.3% (1.5 to 3.1)	7.5% (6.0 to 9.4)
Bahrain	56.5% (42.8 to 68.5)	6.2% (2.1 to 11.2)	0.0% (0.0 to 0.0)	9.8% (6.7 to 13.2)	5.4% (-0.7 to 12.3)	3.5% (0.1 to 7.4)	2.3% (0.7 to 4.2)	3.9% (2.6 to 5.2)	9.3% (7.5 to 11.5)
Bangladesh	56.6% (42.5 to 68.4)	2.2% (0.2 to 4.9)	37.9% (29.3 to 46.0)	9.3% (6.7 to 12.0)	11.2% (-1.6 to 24.1)	1.2% (0.1 to 2.5)	1.8% (0.6 to 3.1)	5.2% (3.6 to 6.9)	13.3% (11.2 to 15.6)
Barbados	59.0% (43.9 to 70.7)	0.4% (0.2 to 0.6)	0.0% (0.0 to 0.1)	7.8% (5.6 to 10.0)	4.5% (-0.6 to 10.2)	3.1% (0.5 to 6.1)	0.0% (0.0 to 0.0)	1.3% (0.9 to 1.7)	5.1% (4.0 to 6.3)
Belarus	66.2% (50.9 to 77.6)	-0.2% (-0.6 to 0.0)	0.1% (0.0 to 0.6)	10.8% (7.9 to 13.9)	3.6% (-0.5 to 8.3)	2.6% (0.6 to 5.2)	9.2% (7.1 to 10.6)	3.6% (2.4 to 4.8)	17.5% (15.3 to 19.9)
Belgium	60.8% (45.6 to 72.4)	0.0% (-0.1 to 0.1)	0.0% (0.0 to 0.0)	7.3% (5.0 to 9.6)	6.7% (-0.9 to 15.3)	2.4% (-0.3 to 5.1)	7.2% (6.3 to 9.3)	2.1% (1.4 to 2.9)	13.3% (11.2 to 15.6)
Belize	53.6% (38.3 to 66.5)	1.0% (0.7 to 1.6)	4.4% (0.7 to 12.0)	8.5% (6.3 to 10.7)	5.3% (-0.7 to 12.0)	1.6% (0.1 to 3.3)	0.3% (0.1 to 0.4)	2.4% (1.6 to 3.3)	8.5% (6.8 to 10.4)
Benin	61.3% (45.5 to 72.9)	2.7% (1.6 to 3.6)	39.8% (32.1 to 47.6)	10.6% (7.9 to 13.5)	7.2% (-1.0 to 15.5)	0.8% (0.1 to 1.6)	0.1% (0.0 to 0.2)	1.5% (1.0 to 2.0)	3.1% (2.4 to 4.0)
Bermuda	53.7% (39.0 to 66.4)	0.4% (-0.3 to 1.4)	0.0% (0.0 to 0.0)	7.1% (5.1 to 9.3)	3.0% (-0.4 to 6.8)	2.3% (0.1 to 5.0)	1.9% (1.6 to 2.4)	2.4% (1.6 to 3.3)	9.5% (7.6 to 11.6)
Bhutan	54.6% (39.2 to 66.5)	0.1% (0.0 to 0.2)	6.3% (1.2 to 16.9)	10.3% (7.6 to 13.2)	12.5% (-1.7 to 27.3)	4.1% (1.2 to 7.1)	7.9% (7.2 to 9.1)	3.2% (2.1 to 4.3)	6.0% (4.4 to 7.7)
Bolivia (Plurinational State of)	42.9% (30.6 to 55.7)	0.3% (-0.1 to 0.8)	8.1% (2.5 to 16.8)	6.3% (4.5 to 8.0)	7.7% (-1.0 to 17.4)	1.2% (0.1 to 2.5)	6.7% (6.1 to 7.7)	2.2% (1.3 to 3.1)	5.9% (4.7 to 7.2)
Bosnia and Herzegovina	63.6% (47.8 to 75.9)	0.1% (-0.3 to 0.9)	6.8% (0.5 to 24.6)	7.7% (5.3 to 10.1)	6.2% (-0.8 to 13.7)	3.0% (0.3 to 6.2)	8.5% (7.5 to 10.3)	4.2% (2.8 to 5.8)	15.9% (13.4 to 18.4)
Botswana	62.3% (46.6 to 73.9)	0.6% (-0.4 to 2.3)	3.6% (0.1 to 21.7)	12.6% (9.5 to 15.7)	7.7% (-1.0 to 16.9)	2.8% (0.7 to 5.6)	3.2% (2.7 to 4.0)	3.8% (2.5 to 5.1)	10.2% (8.3 to 12.3)
Brazil	56.0% (41.4 to 67.6)	0.4% (0.2 to 0.7)	1.9% (0.4 to 5.4)	8.4% (6.4 to 10.5)	6.5% (-0.9 to 14.9)	2.7% (0.5 to 5.1)	2.1% (1.7 to 2.5)	3.2% (2.2 to 4.3)	11.4% (9.6 to 13.5)
Brunei Darussalam	54.4% (39.2 to 66.7)	0.6% (0.3 to 0.9)	0.0% (0.0 to 0.0)	9.7% (6.8 to 12.7)	4.2% (-0.6 to 9.7)	2.1% (0.2 to 4.5)	0.0% (0.0 to 0.1)	2.8% (1.9 to 3.7)	11.1% (9.1 to 13.7)
Bulgaria	62.4% (48.0 to 74.4)	0.2% (-0.4 to 1.2)	1.1% (0.0 to 8.8)	7.8% (5.5 to 10.3)	4.0% (-0.5 to 9.1)	2.3% (0.1 to 5.1)	8.7% (7.8 to 10.9)	3.7% (2.5 to 5.0)	14.0% (12.0 to 16.0)
Burkina Faso	55.4% (39.9 to 67.7)	6.1% (4.5 to 8.2)	39.9% (32.2 to 47.2)	10.2% (7.7 to 12.8)	11.7% (-1.5 to 25.7)	1.0% (0.2 to 1.9)	0.3% (0.1 to 0.6)	2.8% (1.8 to 3.9)	4.3% (3.5 to 5.4)

Burundi	57.9% (42.6 to 69.4)	0.0% (0.0 to 0.1)	46.0% (38.3 to 53.4)	8.2% (5.8 to 10.7)	9.0% (-1.2 to 19.8)	0.7% (0.1 to 1.4)	2.6% (2.0 to 3.8)	1.4% (0.9 to 2.0)	4.5% (3.5 to 5.7)
CV¥te d'Ivoire	62.4% (48.9 to 73.5)	0.8% (0.5 to 1.0)	32.3% (22.7 to 41.5)	10.6% (8.0 to 13.4)	7.9% (-1.1 to 17.7)	1.6% (0.5 to 3.0)	0.1% (0.0 to 0.2)	3.3% (2.2 to 4.3)	5.9% (4.7 to 7.2)
Cabo Verde	64.4% (49.2 to 75.8)	0.1% (-0.1 to 0.2)	8.1% (2.3 to 17.8)	10.4% (7.6 to 13.2)	6.3% (-0.9 to 14.4)	1.2% (0.1 to 2.5)	2.2% (1.8 to 3.2)	2.3% (1.5 to 3.1)	4.1% (3.4 to 5.1)
Cambodia	47.0% (33.0 to 59.6)	2.4% (2.0 to 2.6)	37.4% (28.7 to 45.6)	11.6% (8.6 to 14.6)	7.5% (-1.0 to 16.7)	0.6% (-0.1 to 1.3)	0.5% (0.1 to 1.1)	5.0% (3.4 to 6.7)	15.2% (12.5 to 18.1)
Cameroon	63.9% (49.3 to 74.7)	1.7% (1.2 to 2.5)	33.5% (25.2 to 41.7)	13.1% (10.3 to 16.0)	8.6% (-1.2 to 18.9)	1.4% (0.3 to 2.6)	0.9% (0.6 to 1.2)	1.8% (1.2 to 2.5)	4.7% (3.7 to 5.7)
Canada	47.0% (33.7 to 58.8)	-0.1% (-0.2 to 0.1)	0.0% (0.0 to 0.0)	6.9% (4.6 to 9.3)	2.7% (-0.3 to 6.2)	1.4% (-0.2 to 3.5)	6.4% (5.4 to 7.3)	2.6% (1.7 to 3.5)	12.4% (10.3 to 14.7)
Central African Republic	57.8% (42.1 to 71.0)	1.0% (0.2 to 1.7)	45.2% (38.0 to 52.3)	12.5% (9.6 to 15.6)	9.1% (-1.2 to 20.6)	0.9% (0.1 to 1.8)	0.2% (0.1 to 0.3)	2.0% (1.3 to 2.7)	5.1% (3.9 to 6.6)
Chad	54.7% (39.4 to 66.7)	5.4% (3.6 to 8.1)	39.2% (31.0 to 46.5)	10.2% (7.7 to 12.9)	12.1% (-1.7 to 26.4)	1.2% (0.3 to 2.3)	0.6% (0.1 to 1.3)	2.1% (1.4 to 2.8)	5.0% (3.7 to 6.5)
Chile	59.4% (44.9 to 70.7)	0.0% (0.0 to 0.0)	0.6% (0.0 to 6.7)	6.4% (4.3 to 8.5)	2.4% (-0.3 to 5.6)	1.5% (0.1 to 3.1)	7.8% (7.0 to 8.8)	4.6% (3.1 to 6.3)	10.4% (8.9 to 11.9)
China	56.0% (41.7 to 68.1)	0.5% (-0.3 to 1.9)	5.5% (0.8 to 18.0)	7.5% (5.3 to 9.8)	8.4% (-1.1 to 18.7)	1.9% (0.2 to 4.0)	7.1% (6.5 to 7.9)	5.2% (3.6 to 7.0)	17.9% (15.0 to 21.1)
Colombia	57.6% (42.8 to 69.2)	0.3% (0.3 to 0.4)	1.2% (0.0 to 6.8)	8.1% (5.9 to 10.2)	6.1% (-0.8 to 13.8)	2.3% (0.8 to 4.1)	3.9% (3.2 to 4.7)	2.6% (1.7 to 3.6)	6.2% (5.1 to 7.3)
Comoros	59.4% (44.1 to 71.7)	0.0% (0.0 to 0.1)	41.3% (33.8 to 48.6)	8.1% (5.7 to 10.7)	6.8% (-0.9 to 15.6)	0.9% (0.2 to 1.7)	0.4% (0.1 to 0.7)	3.6% (2.4 to 5.0)	5.8% (4.3 to 7.5)
Congo	61.5% (46.1 to 73.1)	0.2% (0.0 to 0.5)	22.6% (10.7 to 34.3)	12.6% (9.5 to 15.9)	5.0% (-0.6 to 11.4)	1.2% (0.3 to 2.3)	0.2% (0.1 to 0.4)	2.5% (1.7 to 3.4)	5.6% (4.3 to 7.1)
Cook Islands	56.1% (42.2 to 68.3)	0.2% (-0.1 to 0.5)	0.1% (0.0 to 1.3)	9.4% (7.1 to 11.9)	1.4% (-0.2 to 3.3)	1.7% (0.6 to 3.0)	0.0% (0.0 to 0.0)	5.0% (3.4 to 6.8)	12.8% (10.6 to 15.6)
Costa Rica	60.2% (45.0 to 71.8)	0.0% (0.0 to 0.1)	0.7% (0.0 to 4.6)	10.8% (7.8 to 13.7)	7.1% (-1.0 to 16.0)	1.5% (0.1 to 3.2)	2.2% (1.5 to 2.9)	2.7% (1.8 to 3.6)	8.4% (7.0 to 9.9)
Croatia	63.3% (48.7 to 75.0)	0.2% (-0.5 to 1.2)	0.2% (0.0 to 2.2)	7.4% (5.0 to 9.9)	4.1% (-0.5 to 9.3)	2.4% (-0.6 to 5.8)	7.8% (6.7 to 9.2)	4.8% (3.2 to 6.4)	15.1% (12.7 to 17.9)
Cuba	49.0% (34.5 to 60.9)	0.6% (0.2 to 1.1)	0.6% (0.1 to 2.0)	7.6% (5.5 to 9.7)	8.6% (-1.1 to 19.2)	2.3% (0.4 to 4.4)	0.4% (0.1 to 0.7)	3.6% (2.4 to 4.8)	14.0% (11.8 to 16.4)
Cyprus	56.6% (41.9 to 68.6)	1.5% (-0.4 to 4.7)	0.0% (0.0 to 0.0)	8.3% (5.4 to 11.4)	5.6% (-0.7 to 12.3)	2.1% (-1.8 to 6.0)	4.6% (3.5 to 5.8)	3.0% (2.0 to 3.9)	11.6% (9.5 to 13.9)
Czechia	60.2% (44.2 to 72.3)	0.0% (-0.2 to 0.1)	0.0% (0.0 to 0.2)	7.1% (4.8 to 9.3)	2.9% (-0.4 to 6.6)	3.0% (0.0 to 6.3)	8.1% (7.0 to 9.8)	3.4% (2.3 to 4.5)	14.2% (12.1 to 16.4)
Democratic People's Republic of Korea	52.6% (37.7 to 65.5)	0.3% (-0.4 to 1.5)	41.3% (34.0 to 48.5)	8.1% (6.1 to 10.1)	7.1% (-0.9 to 15.8)	1.5% (0.4 to 2.7)	8.1% (7.3 to 9.5)	5.6% (3.9 to 7.5)	15.2% (12.9 to 17.7)
Democratic Republic of the Congo	56.7% (41.8 to 68.9)	0.2% (-0.1 to 0.7)	44.6% (37.3 to 51.7)	12.6% (9.4 to 15.8)	7.5% (-1.0 to 16.8)	1.4% (0.1 to 2.9)	1.0% (0.9 to 1.2)	1.9% (1.1 to 2.6)	3.4% (2.6 to 4.4)

Denmark	59.9% (45.3 to 72.0)	0.0% (0.0 to 0.0)	0.0% (0.0 to 0.0)	7.3% (5.0 to 9.7)	3.6% (-0.5 to 8.0)	2.1% (-0.3 to 5.0)	8.3% (7.3 to 10.9)	1.9% (1.3 to 2.6)	15.6% (13.1 to 18.4)
Djibouti	60.1% (44.7 to 72.3)	7.0% (4.1 to 11.0)	12.1% (6.1 to 19.3)	8.0% (5.6 to 10.6)	7.6% (-1.0 to 16.7)	0.9% (0.2 to 1.8)	1.8% (0.6 to 3.7)	4.4% (2.8 to 6.1)	10.4% (8.2 to 13.0)
Dominica	55.7% (41.5 to 68.0)	0.3% (0.1 to 0.4)	1.8% (0.2 to 6.7)	8.7% (6.4 to 11.2)	5.0% (-0.7 to 11.2)	1.5% (-0.3 to 3.6)	0.0% (-0.1 to 0.2)	2.1% (1.4 to 3.0)	5.8% (4.6 to 7.3)
Dominican Republic	53.3% (37.8 to 66.1)	0.2% (0.1 to 0.4)	0.9% (0.0 to 6.0)	8.1% (6.1 to 10.1)	10.2% (-1.4 to 22.4)	1.9% (0.6 to 3.6)	0.6% (0.4 to 0.9)	2.5% (1.7 to 3.4)	12.0% (9.8 to 14.7)
Ecuador	43.8% (30.8 to 55.9)	0.0% (0.0 to 0.1)	1.2% (0.1 to 4.9)	5.9% (4.3 to 7.5)	4.7% (-0.6 to 10.5)	1.1% (0.2 to 2.0)	3.8% (3.3 to 4.6)	2.0% (1.2 to 2.7)	6.3% (5.3 to 7.5)
Egypt	57.9% (42.1 to 69.7)	3.5% (-0.8 to 9.4)	0.1% (0.0 to 0.3)	10.4% (7.4 to 13.5)	12.3% (-1.8 to 27.4)	4.1% (1.2 to 7.8)	2.9% (1.7 to 3.8)	4.4% (3.0 to 5.8)	12.1% (10.1 to 14.4)
El Salvador	54.4% (39.2 to 66.4)	0.6% (0.4 to 0.7)	5.3% (1.1 to 12.9)	9.4% (7.1 to 11.7)	11.3% (-1.6 to 24.9)	1.3% (0.3 to 2.6)	0.1% (0.0 to 0.1)	2.5% (1.7 to 3.3)	6.4% (5.1 to 7.7)
Equatorial Guinea	62.2% (46.5 to 73.8)	0.0% (-0.1 to 0.1)	0.7% (0.0 to 7.3)	12.5% (9.3 to 15.9)	5.7% (-0.7 to 13.1)	1.6% (0.3 to 3.2)	0.4% (0.3 to 0.5)	3.7% (2.3 to 5.2)	4.6% (3.4 to 6.1)
Eritrea	53.9% (38.6 to 66.7)	2.7% (1.5 to 4.4)	36.9% (30.0 to 44.3)	8.0% (5.7 to 10.4)	7.2% (-0.9 to 16.0)	0.4% (0.1 to 0.8)	2.0% (1.6 to 2.6)	2.4% (1.6 to 3.3)	4.0% (2.9 to 5.2)
Estonia	64.3% (48.1 to 75.5)	-0.1% (-0.2 to 0.0)	0.2% (0.0 to 2.2)	10.4% (7.5 to 13.3)	2.8% (-0.3 to 6.3)	2.4% (0.1 to 4.9)	8.0% (6.9 to 9.1)	2.8% (1.9 to 3.8)	15.4% (13.4 to 17.6)
Eswatini	63.2% (48.0 to 74.8)	0.2% (-0.2 to 0.7)	15.4% (2.8 to 33.6)	12.9% (9.9 to 16.1)	7.4% (-1.0 to 16.5)	1.6% (0.2 to 3.2)	5.2% (4.7 to 6.0)	2.4% (1.6 to 3.4)	4.8% (3.6 to 6.1)
Ethiopia	47.3% (33.8 to 58.9)	0.3% (0.1 to 0.5)	41.8% (34.7 to 48.5)	7.8% (5.6 to 10.2)	10.4% (-1.4 to 22.7)	0.8% (0.2 to 1.5)	4.6% (4.1 to 5.3)	1.6% (1.1 to 2.2)	2.9% (2.3 to 3.6)
Fiji	60.5% (45.6 to 71.9)	0.1% (0.0 to 0.2)	7.5% (0.9 to 20.3)	10.2% (7.7 to 12.8)	2.1% (-0.3 to 4.7)	1.4% (0.5 to 2.6)	0.5% (-0.2 to 0.9)	4.5% (2.9 to 6.1)	10.4% (8.7 to 12.5)
Finland	60.1% (45.2 to 72.0)	-0.1% (-0.3 to 0.0)	0.0% (0.0 to 0.0)	6.4% (4.3 to 8.5)	1.9% (-0.2 to 4.3)	2.3% (-0.3 to 5.1)	7.8% (6.3 to 8.9)	1.6% (1.1 to 2.2)	10.5% (8.8 to 12.4)
France	56.6% (42.1 to 68.8)	0.0% (-0.1 to 0.2)	0.0% (0.0 to 0.0)	6.2% (4.2 to 8.4)	4.2% (-0.5 to 9.5)	2.5% (-0.2 to 5.4)	6.8% (6.0 to 8.4)	2.5% (1.7 to 3.3)	13.0% (10.9 to 15.2)
Gabon	61.0% (46.8 to 73.4)	0.1% (0.0 to 0.1)	1.0% (0.0 to 7.3)	12.5% (9.3 to 15.7)	4.6% (-0.6 to 10.6)	0.8% (0.0 to 1.7)	0.3% (0.1 to 0.4)	2.6% (1.7 to 3.6)	5.1% (3.9 to 6.6)
Gambia	63.5% (47.6 to 74.5)	2.1% (1.3 to 3.2)	39.3% (31.4 to 46.9)	10.6% (7.9 to 13.3)	8.9% (-1.2 to 19.6)	1.2% (0.3 to 2.4)	0.2% (0.0 to 0.4)	3.7% (2.5 to 5.0)	5.8% (4.7 to 7.1)
Georgia	65.0% (49.0 to 76.5)	0.2% (-0.3 to 1.2)	4.9% (0.5 to 16.3)	11.7% (8.6 to 15.0)	6.4% (-0.9 to 14.4)	1.9% (-0.1 to 4.2)	8.5% (7.8 to 10.6)	5.2% (3.5 to 6.9)	17.0% (14.7 to 19.2)
Germany	60.1% (45.3 to 72.0)	0.0% (-0.1 to 0.1)	0.0% (0.0 to 0.0)	7.3% (5.0 to 9.8)	2.5% (-0.3 to 5.8)	2.4% (0.0 to 5.3)	6.9% (6.0 to 8.9)	2.2% (1.5 to 3.0)	13.2% (11.1 to 15.5)
Ghana	61.9% (47.4 to 73.1)	2.1% (1.8 to 2.5)	27.7% (15.3 to 38.9)	8.5% (6.1 to 11.1)	5.4% (-0.7 to 12.1)	1.8% (0.5 to 3.3)	0.1% (0.0 to 0.2)	1.5% (1.0 to 2.1)	4.8% (3.8 to 6.0)
Greece	50.8% (36.1 to 62.8)	0.3% (-0.2 to 1.1)	0.0% (0.0 to 0.2)	7.7% (5.2 to 10.2)	5.4% (-0.7 to 12.1)	1.6% (-0.8 to 4.1)	6.9% (6.3 to 7.5)	3.4% (2.3 to 4.5)	16.5% (13.9 to 19.2)

Greenland	47.9% (33.3 to 60.4)	-0.2% (-0.5 to 0.0)	0.0% (0.0 to 0.0)	7.8% (5.4 to 10.4)	2.6% (-0.3 to 6.0)	1.4% (-0.3 to 3.1)	7.7% (4.6 to 11.8)	3.9% (2.6 to 5.3)	19.1% (16.0 to 22.6)
Grenada	55.7% (40.3 to 68.4)	0.4% (0.3 to 0.6)	0.3% (0.0 to 1.8)	8.8% (6.4 to 11.3)	7.6% (-1.0 to 17.2)	1.9% (0.1 to 4.2)	0.0% (0.0 to 0.0)	2.1% (1.4 to 2.9)	5.8% (4.7 to 7.1)
Guam	55.2% (39.3 to 67.0)	0.7% (0.3 to 1.2)	0.1% (0.0 to 0.3)	9.4% (7.3 to 11.6)	1.3% (-0.2 to 2.9)	1.3% (0.5 to 2.2)	0.0% (0.0 to 0.0)	4.9% (3.4 to 6.7)	14.8% (12.3 to 17.9)
Guatemala	53.7% (39.4 to 64.9)	0.2% (0.1 to 0.3)	18.5% (5.2 to 33.6)	10.9% (8.2 to 13.8)	12.9% (-1.8 to 27.4)	0.7% (-0.2 to 1.6)	3.0% (1.7 to 4.0)	2.4% (1.5 to 3.4)	5.4% (4.3 to 6.6)
Guinea	56.4% (41.9 to 68.4)	1.1% (0.6 to 2.0)	40.4% (33.0 to 47.8)	10.5% (8.0 to 13.1)	9.9% (-1.4 to 21.7)	0.8% (0.1 to 1.6)	0.2% (0.1 to 0.4)	2.9% (2.0 to 4.0)	5.3% (4.2 to 6.6)
Guinea-Bissau	60.2% (44.3 to 72.5)	2.2% (1.6 to 2.7)	40.8% (33.4 to 48.3)	10.9% (8.2 to 13.6)	9.0% (-1.2 to 20.3)	1.4% (0.4 to 2.7)	0.1% (0.0 to 0.3)	2.2% (1.5 to 2.9)	4.0% (3.1 to 4.9)
Guyana	55.2% (40.0 to 67.4)	0.3% (0.0 to 0.6)	2.4% (0.5 to 6.8)	8.9% (6.7 to 11.3)	8.2% (-1.1 to 17.9)	1.9% (0.3 to 3.7)	0.1% (0.0 to 0.2)	2.6% (1.7 to 3.4)	7.5% (6.1 to 9.1)
Haiti	59.2% (45.0 to 70.6)	0.4% (0.3 to 0.5)	42.5% (35.0 to 50.2)	8.3% (6.1 to 10.6)	12.7% (-1.8 to 27.7)	1.7% (0.4 to 3.3)	0.2% (0.1 to 0.4)	1.6% (1.0 to 2.2)	3.9% (3.1 to 4.9)
Honduras	56.4% (40.7 to 68.6)	0.4% (0.1 to 0.8)	25.5% (15.7 to 34.3)	10.9% (8.0 to 13.7)	13.0% (-1.8 to 27.8)	1.2% (0.1 to 2.4)	1.1% (0.5 to 1.7)	3.7% (2.5 to 5.0)	8.1% (6.5 to 10.1)
Hungary	68.3% (53.0 to 79.2)	0.2% (-0.7 to 1.4)	0.5% (0.0 to 6.3)	7.4% (5.2 to 9.7)	4.0% (-0.5 to 9.1)	2.8% (0.4 to 5.7)	7.6% (6.5 to 8.9)	3.3% (2.2 to 4.4)	14.3% (12.3 to 16.7)
Iceland	54.7% (40.4 to 66.5)	0.0% (0.0 to 0.0)	0.0% (0.0 to 0.0)	5.7% (3.8 to 7.6)	3.4% (-0.4 to 7.7)	2.3% (-0.4 to 5.5)	9.1% (7.9 to 10.3)	1.8% (1.2 to 2.4)	10.9% (8.9 to 13.1)
India	55.3% (40.4 to 66.9)	2.6% (0.8 to 4.8)	22.2% (13.8 to 32.6)	10.7% (8.0 to 13.7)	10.4% (-1.4 to 23.3)	1.8% (0.4 to 3.4)	2.1% (0.8 to 3.6)	4.0% (2.8 to 5.4)	9.5% (7.9 to 11.3)
Indonesia	67.4% (52.4 to 77.5)	0.4% (0.3 to 0.5)	9.1% (3.0 to 19.4)	12.6% (9.4 to 15.8)	6.8% (-0.9 to 15.2)	3.1% (0.5 to 6.1)	0.4% (0.3 to 0.7)	5.3% (3.7 to 7.2)	13.5% (11.1 to 15.9)
Iran (Islamic Republic of)	53.6% (40.2 to 64.6)	1.9% (-0.3 to 4.8)	0.0% (0.0 to 0.2)	10.4% (7.4 to 13.4)	12.3% (-1.8 to 26.9)	4.5% (1.4 to 8.0)	6.4% (5.3 to 7.5)	2.8% (1.9 to 3.8)	9.0% (7.4 to 10.6)
Iraq	64.3% (49.2 to 74.9)	9.4% (0.8 to 19.2)	0.1% (0.0 to 0.9)	10.8% (7.6 to 14.1)	7.6% (-1.0 to 17.1)	4.6% (1.6 to 8.3)	5.8% (2.7 to 9.9)	5.6% (3.8 to 7.4)	14.3% (12.0 to 16.9)
Ireland	57.3% (42.4 to 68.5)	0.0% (0.0 to 0.0)	0.0% (0.0 to 0.0)	8.9% (6.2 to 11.6)	4.1% (-0.5 to 9.3)	2.5% (-0.9 to 6.2)	7.0% (5.7 to 9.6)	2.4% (1.6 to 3.2)	11.3% (9.3 to 13.3)
Israel	59.4% (44.1 to 70.9)	1.2% (-0.6 to 4.4)	0.0% (0.0 to 0.0)	8.0% (5.6 to 10.5)	3.2% (-0.4 to 7.3)	2.1% (-0.1 to 4.6)	2.6% (1.7 to 3.3)	2.5% (1.7 to 3.4)	11.2% (9.3 to 13.2)
Italy	49.2% (34.6 to 60.2)	0.1% (-0.3 to 0.6)	0.0% (0.0 to 0.1)	6.6% (4.4 to 8.9)	4.5% (-0.6 to 10.3)	2.3% (-0.9 to 5.6)	7.4% (6.6 to 8.4)	2.7% (1.8 to 3.6)	10.6% (8.9 to 12.6)
Jamaica	55.8% (41.3 to 68.0)	0.5% (0.1 to 0.9)	3.6% (0.6 to 10.1)	8.1% (6.0 to 10.4)	7.4% (-1.0 to 17.0)	2.5% (0.4 to 4.9)	0.1% (0.0 to 0.2)	2.4% (1.6 to 3.2)	8.4% (6.9 to 10.3)
Japan	54.6% (40.8 to 65.6)	0.1% (-0.2 to 0.4)	0.0% (0.0 to 0.0)	8.1% (5.6 to 10.7)	2.5% (-0.3 to 6.0)	2.4% (0.7 to 4.4)	6.1% (5.4 to 6.9)	3.0% (2.0 to 4.1)	15.0% (12.8 to 17.3)
Jordan	54.4% (39.1 to 66.2)	0.9% (-0.6 to 3.5)	0.0% (0.0 to 0.1)	9.9% (6.9 to 13.0)	6.5% (-0.9 to 14.8)	4.1% (1.1 to 8.0)	4.4% (3.5 to 5.4)	4.9% (3.4 to 6.6)	13.9% (11.8 to 16.4)

Kazakhstan	67.9% (52.5 to 78.6)	0.2% (-0.5 to 1.2)	0.3% (0.0 to 3.1)	12.3% (9.0 to 15.5)	3.0% (-0.4 to 6.7)	2.0% (0.3 to 4.2)	8.6% (7.4 to 9.9)	4.0% (2.7 to 5.4)	11.0% (9.3 to 12.7)
Kenya	58.6% (44.6 to 69.7)	0.2% (0.1 to 0.2)	39.0% (31.2 to 46.3)	8.2% (5.8 to 10.8)	6.3% (-0.8 to 14.3)	1.2% (0.3 to 2.3)	3.6% (3.0 to 4.6)	2.0% (1.3 to 2.6)	5.2% (4.2 to 6.4)
Kiribati	47.6% (32.4 to 59.1)	0.2% (0.0 to 0.4)	29.1% (23.1 to 35.8)	10.2% (8.0 to 12.5)	2.8% (-0.4 to 6.3)	1.7% (0.7 to 2.8)	0.0% (-0.1 to 0.1)	8.4% (5.8 to 11.1)	25.1% (21.7 to 28.5)
Kuwait	52.9% (38.9 to 64.0)	9.9% (2.2 to 17.8)	0.0% (0.0 to 0.0)	9.3% (6.4 to 12.2)	5.1% (-0.7 to 11.7)	5.9% (2.4 to 10.0)	4.1% (1.0 to 7.8)	4.5% (3.0 to 5.9)	13.0% (10.8 to 15.4)
Kyrgyzstan	55.7% (39.3 to 69.0)	0.0% (-0.5 to 0.6)	16.7% (9.9 to 24.4)	11.5% (9.0 to 14.3)	4.7% (-0.6 to 10.7)	2.1% (0.5 to 3.8)	8.8% (7.2 to 10.1)	5.4% (3.7 to 7.2)	20.1% (17.4 to 23.0)
Lao People's Democratic Republic	55.7% (40.2 to 67.8)	1.0% (0.2 to 2.1)	30.5% (13.4 to 45.7)	12.6% (9.6 to 15.7)	8.3% (-1.1 to 18.0)	0.8% (0.2 to 1.6)	1.6% (1.1 to 2.1)	5.7% (3.8 to 7.7)	15.0% (12.7 to 17.5)
Latvia	65.4% (50.0 to 77.4)	-0.1% (-0.3 to 0.0)	0.5% (0.0 to 5.1)	11.1% (7.9 to 14.2)	2.8% (-0.4 to 6.6)	2.8% (0.4 to 5.5)	9.6% (8.0 to 10.7)	3.5% (2.3 to 4.7)	12.1% (10.4 to 14.0)
Lebanon	55.4% (40.7 to 66.6)	0.2% (-0.3 to 1.0)	0.1% (0.0 to 0.4)	9.4% (6.6 to 12.2)	4.8% (-0.6 to 10.8)	3.6% (1.1 to 6.5)	5.1% (4.4 to 6.0)	5.2% (3.5 to 6.9)	19.5% (16.3 to 22.9)
Lesotho	57.8% (42.2 to 70.3)	0.0% (0.0 to 0.0)	32.5% (25.2 to 39.8)	12.8% (9.9 to 15.8)	9.0% (-1.2 to 20.2)	0.8% (-0.2 to 1.9)	8.6% (7.5 to 10.9)	4.9% (3.4 to 6.4)	12.0% (9.3 to 15.0)
Liberia	61.9% (46.7 to 73.4)	0.2% (0.1 to 0.4)	42.6% (35.7 to 49.6)	10.6% (7.9 to 13.3)	8.9% (-1.2 to 19.9)	1.9% (0.6 to 3.6)	0.1% (0.0 to 0.2)	1.9% (1.2 to 2.6)	3.9% (3.1 to 4.9)
Libya	56.8% (42.3 to 67.4)	2.8% (-0.3 to 7.9)	0.1% (0.0 to 0.4)	9.4% (6.7 to 12.3)	6.3% (-0.9 to 14.2)	4.5% (1.7 to 7.8)	3.6% (2.7 to 4.5)	6.1% (4.2 to 8.1)	8.7% (6.9 to 11.0)
Lithuania	66.4% (50.9 to 77.4)	-0.1% (-0.4 to 0.0)	0.1% (0.0 to 0.8)	10.8% (7.9 to 13.9)	2.8% (-0.4 to 6.5)	3.4% (0.7 to 6.8)	9.2% (7.4 to 10.4)	2.9% (1.9 to 4.0)	13.2% (11.3 to 15.5)
Luxembourg	59.1% (44.3 to 71.4)	0.0% (-0.1 to 0.1)	0.0% (0.0 to 0.0)	7.7% (5.2 to 10.3)	3.3% (-0.4 to 7.7)	2.0% (-0.6 to 4.9)	7.9% (6.9 to 10.5)	2.2% (1.5 to 2.9)	11.9% (9.6 to 14.6)
Madagascar	56.5% (41.6 to 67.8)	0.3% (-0.1 to 0.8)	46.7% (39.0 to 53.8)	7.9% (5.6 to 10.2)	6.8% (-0.9 to 14.9)	0.7% (0.1 to 1.3)	3.4% (3.0 to 4.1)	2.4% (1.4 to 3.3)	4.1% (3.3 to 4.9)
Malawi	62.6% (46.3 to 74.3)	0.7% (-0.4 to 2.2)	45.9% (38.5 to 53.1)	8.5% (6.0 to 11.1)	8.1% (-1.1 to 18.3)	0.8% (0.2 to 1.5)	2.5% (2.1 to 3.1)	2.7% (1.8 to 3.6)	8.9% (6.9 to 11.2)
Malaysia	67.7% (52.0 to 78.2)	0.7% (0.6 to 1.0)	0.0% (0.0 to 0.3)	13.2% (10.0 to 16.5)	5.1% (-0.6 to 11.6)	2.3% (0.8 to 4.2)	0.1% (0.0 to 0.1)	5.2% (3.5 to 6.9)	11.1% (9.4 to 13.1)
Maldives	59.7% (44.5 to 71.3)	0.9% (0.5 to 1.3)	0.9% (0.0 to 6.8)	11.7% (8.7 to 15.0)	6.0% (-0.8 to 13.4)	3.6% (0.9 to 6.7)	0.0% (-0.1 to 0.0)	5.7% (3.8 to 7.6)	14.7% (12.4 to 17.4)
Mali	54.7% (40.3 to 67.1)	7.2% (4.8 to 10.6)	38.9% (31.0 to 46.3)	10.3% (7.8 to 12.9)	11.3% (-1.6 to 24.9)	1.5% (0.4 to 2.9)	0.3% (0.1 to 0.8)	3.7% (2.5 to 5.1)	5.2% (4.1 to 6.5)
Malta	56.4% (41.8 to 68.9)	0.5% (-0.5 to 2.3)	0.0% (0.0 to 0.0)	7.4% (5.1 to 9.9)	8.6% (-1.2 to 18.9)	2.7% (-0.3 to 5.9)	3.9% (2.2 to 5.2)	2.2% (1.5 to 3.0)	11.1% (9.3 to 13.2)
Marshall Islands	47.2% (33.5 to 60.2)	0.9% (0.4 to 1.5)	18.2% (11.9 to 25.3)	10.1% (7.9 to 12.4)	2.6% (-0.3 to 6.0)	2.0% (0.8 to 3.4)	0.0% (0.0 to 0.0)	5.2% (3.5 to 6.9)	11.8% (9.6 to 14.3)
Mauritania	61.4% (45.6 to 72.9)	9.7% (4.8 to 16.0)	23.2% (14.3 to 32.4)	10.3% (7.7 to 13.1)	5.8% (-0.8 to 12.7)	2.9% (0.9 to 5.2)	0.8% (0.2 to 1.5)	4.3% (2.8 to 5.9)	4.2% (3.2 to 5.3)

Mauritius	59.8% (45.0 to 71.8)	0.1% (-0.1 to 0.4)	0.1% (0.0 to 0.6)	12.6% (9.7 to 15.5)	3.7% (-0.5 to 8.5)	1.7% (0.4 to 3.2)	1.1% (0.3 to 1.8)	5.7% (3.9 to 7.7)	14.9% (12.9 to 17.1)
Mexico	52.5% (38.3 to 63.8)	0.6% (0.0 to 1.4)	1.8% (0.1 to 7.7)	9.5% (7.1 to 12.0)	7.9% (-1.0 to 17.4)	1.5% (0.3 to 3.0)	4.3% (3.7 to 5.0)	2.4% (1.6 to 3.1)	6.4% (5.3 to 7.5)
Micronesia (Federated States of)	44.8% (30.4 to 57.9)	0.9% (0.4 to 1.4)	18.9% (12.2 to 26.2)	10.4% (8.1 to 12.8)	2.5% (-0.3 to 5.7)	2.2% (0.9 to 3.7)	0.0% (0.0 to 0.0)	7.0% (4.5 to 9.5)	18.0% (14.9 to 21.0)
Monaco	56.5% (42.3 to 68.4)	0.0% (-0.1 to 0.1)	0.0% (0.0 to 0.0)	7.5% (5.1 to 10.1)	3.0% (-0.4 to 6.9)	2.7% (-0.6 to 6.3)	7.3% (6.6 to 8.1)	2.2% (1.5 to 3.0)	12.6% (10.1 to 15.7)
Mongolia	64.3% (49.3 to 76.3)	-0.1% (-0.2 to 0.0)	7.9% (0.6 to 27.5)	11.7% (9.1 to 14.4)	5.5% (-0.7 to 12.5)	0.4% (0.1 to 0.8)	9.6% (7.1 to 12.3)	4.8% (3.3 to 6.3)	18.2% (15.3 to 21.4)
Montenegro	62.9% (47.9 to 74.5)	0.1% (-0.2 to 0.5)	3.2% (0.0 to 21.4)	8.1% (5.4 to 10.9)	3.2% (-0.4 to 7.3)	0.9% (-0.3 to 2.2)	9.3% (8.2 to 10.5)	4.7% (3.1 to 6.2)	14.0% (11.5 to 16.8)
Morocco	63.0% (47.4 to 74.5)	0.6% (-0.1 to 1.9)	1.1% (0.2 to 3.4)	9.3% (6.5 to 12.3)	6.6% (-0.9 to 14.6)	4.6% (1.5 to 8.1)	6.0% (5.0 to 7.2)	4.4% (3.0 to 5.9)	5.3% (4.2 to 6.7)
Mozambique	64.3% (48.9 to 75.8)	0.8% (-0.4 to 2.5)	45.8% (38.4 to 52.9)	8.7% (6.2 to 11.2)	10.5% (-1.5 to 22.9)	0.8% (0.2 to 1.5)	1.5% (1.1 to 1.9)	2.2% (1.4 to 3.1)	7.6% (6.1 to 9.2)
Myanmar	58.0% (43.5 to 69.9)	2.3% (1.4 to 3.6)	28.7% (14.7 to 41.2)	12.0% (9.0 to 15.0)	7.5% (-1.0 to 16.9)	0.6% (0.1 to 1.2)	0.9% (0.6 to 1.3)	4.3% (2.8 to 6.0)	11.9% (10.0 to 14.4)
Namibia	58.7% (42.8 to 71.9)	0.6% (-0.6 to 2.1)	10.6% (1.0 to 30.6)	12.2% (9.1 to 15.5)	6.4% (-0.9 to 14.6)	2.0% (0.3 to 4.1)	2.9% (2.4 to 3.5)	3.3% (2.1 to 4.7)	8.3% (6.7 to 10.0)
Nauru	60.7% (45.7 to 71.9)	1.0% (0.5 to 1.5)	0.7% (0.0 to 3.3)	10.7% (8.3 to 13.1)	1.7% (-0.2 to 4.0)	1.5% (0.6 to 2.5)	0.0% (0.0 to 0.0)	6.9% (4.5 to 9.2)	16.2% (13.4 to 19.1)
Nepal	43.6% (30.2 to 56.0)	0.8% (-0.5 to 2.7)	33.3% (26.1 to 40.9)	12.7% (9.3 to 15.9)	13.8% (-1.9 to 29.9)	1.2% (0.3 to 2.5)	4.3% (3.7 to 5.1)	4.0% (2.6 to 5.4)	13.5% (11.2 to 15.9)
Netherlands	56.9% (41.6 to 68.5)	0.0% (0.0 to 0.1)	0.0% (0.0 to 0.0)	7.7% (5.2 to 10.3)	3.5% (-0.4 to 8.0)	1.9% (-0.7 to 4.5)	6.9% (5.8 to 9.4)	1.8% (1.2 to 2.4)	12.3% (10.2 to 14.7)
New Zealand	52.0% (37.5 to 63.7)	0.0% (0.0 to 0.0)	0.0% (0.0 to 0.0)	7.1% (4.6 to 9.7)	5.2% (-0.7 to 11.7)	2.6% (-0.8 to 6.2)	6.7% (5.7 to 8.1)	2.2% (1.5 to 2.9)	10.5% (8.6 to 12.6)
Nicaragua	58.9% (43.3 to 70.4)	0.7% (0.6 to 0.8)	22.7% (12.4 to 32.0)	13.2% (10.0 to 16.2)	9.0% (-1.3 to 19.9)	1.4% (0.3 to 2.8)	0.3% (0.2 to 0.5)	4.1% (2.8 to 5.6)	7.7% (6.2 to 9.5)
Niger	58.4% (42.9 to 69.8)	7.6% (4.9 to 11.4)	41.1% (33.1 to 49.0)	9.8% (7.4 to 12.5)	13.3% (-1.9 to 28.8)	1.2% (0.2 to 2.4)	1.3% (0.4 to 2.8)	2.0% (1.3 to 2.7)	2.5% (2.0 to 3.3)
Nigeria	62.7% (47.2 to 73.9)	2.2% (1.7 to 3.0)	24.9% (14.3 to 35.6)	12.9% (9.8 to 16.1)	6.1% (-0.8 to 13.6)	1.9% (0.4 to 3.6)	0.4% (0.1 to 0.7)	1.3% (0.8 to 1.7)	2.5% (2.0 to 3.2)
Niue	56.2% (42.1 to 67.7)	0.2% (0.2 to 0.2)	0.7% (0.0 to 3.9)	9.9% (7.5 to 12.2)	1.5% (-0.2 to 3.5)	1.4% (0.5 to 2.6)	0.4% (0.2 to 0.6)	4.3% (2.9 to 5.7)	10.9% (8.6 to 13.4)
North Macedonia	62.1% (46.9 to 74.3)	0.2% (-0.3 to 1.3)	2.8% (0.1 to 15.0)	9.3% (6.3 to 12.7)	4.2% (-0.5 to 9.6)	2.3% (-0.9 to 5.8)	9.1% (8.2 to 11.8)	5.9% (4.0 to 8.0)	13.2% (11.0 to 15.6)
Northern Mariana Islands	57.3% (42.7 to 69.4)	0.7% (0.6 to 0.9)	0.2% (0.0 to 1.8)	10.4% (8.0 to 12.8)	1.3% (-0.2 to 3.0)	1.3% (0.4 to 2.4)	0.2% (0.1 to 0.3)	5.2% (3.4 to 6.9)	13.0% (10.7 to 15.7)
Norway	57.8% (42.6 to 69.2)	0.0% (0.0 to 0.0)	0.0% (0.0 to 0.0)	6.8% (4.5 to 9.2)	3.4% (-0.4 to 7.8)	2.4% (-0.3 to 5.5)	7.9% (6.9 to 8.9)	1.7% (1.2 to 2.3)	9.5% (7.9 to 11.3)

Oman	59.4%<br>(44.1 to 70.9)	8.5%<br>(3.9 to 13.9)	0.0%<br>(0.0 to 0.1)	10.1%<br>(7.2 to 13.3)	6.4%<br>(-0.8 to 14.2)	4.4%<br>(1.3 to 8.0)	2.9%<br>(0.7 to 5.9)	3.4%<br>(2.3 to 4.5)	6.3%<br>(5.1 to 7.6)
Pakistan	59.1%<br>(43.6 to 71.1)	5.6%<br>(0.8 to 11.7)	24.0%<br>(13.7 to 34.4)	10.0%<br>(7.4 to 12.7)	10.6%<br>(-1.5 to 23.3)	1.9%<br>(0.6 to 3.5)	5.0%<br>(2.2 to 8.3)	5.0%<br>(3.4 to 6.6)	9.7%<br>(7.7 to 12.2)
Palau	56.0%<br>(41.1 to 68.0)	0.6%<br>(0.3 to 1.0)	0.0%<br>(0.0 to 0.0)	10.3%<br>(7.9 to 12.6)	1.3%<br>(-0.2 to 3.0)	1.5%<br>(0.5 to 2.7)	0.0%<br>(0.0 to 0.0)	4.3%<br>(2.9 to 5.8)	11.8%<br>(9.6 to 14.5)
Palestine	50.1%<br>(35.3 to 62.9)	1.8%<br>(-0.6 to 5.9)	1.3%<br>(0.5 to 2.7)	10.0%<br>(6.9 to 13.2)	9.0%<br>(-1.2 to 19.5)	4.2%<br>(0.9 to 8.1)	3.7%<br>(2.9 to 4.7)	4.4%<br>(3.0 to 5.9)	11.4%<br>(9.4 to 14.0)
Panama	58.3%<br>(42.5 to 69.3)	0.2%<br>(0.1 to 0.4)	0.4%<br>(0.0 to 4.1)	10.0%<br>(7.4 to 12.7)	7.2%<br>(-0.9 to 15.8)	1.3%<br>(0.0 to 2.7)	0.3%<br>(0.2 to 0.4)	2.0%<br>(1.3 to 2.6)	6.4%<br>(5.1 to 7.8)
Papua New Guinea	40.8%<br>(27.3 to 52.9)	0.1%<br>(0.1 to 0.2)	40.1%<br>(30.0 to 48.6)	9.3%<br>(7.0 to 11.6)	3.2%<br>(-0.4 to 7.3)	0.9%<br>(0.3 to 1.6)	3.4%<br>(2.9 to 4.2)	6.2%<br>(4.1 to 8.4)	10.8%<br>(8.5 to 13.6)
Paraguay	57.7%<br>(42.1 to 69.7)	1.0%<br>(-0.7 to 3.4)	6.9%<br>(0.6 to 21.6)	8.8%<br>(6.5 to 11.1)	6.2%<br>(-0.8 to 13.7)	1.3%<br>(0.0 to 2.8)	2.5%<br>(1.8 to 3.3)	3.6%<br>(2.4 to 4.8)	13.2%<br>(10.7 to 16.2)
Peru	47.6%<br>(33.8 to 59.5)	0.0%<br>(0.0 to 0.1)	2.9%<br>(0.2 to 12.9)	5.6%<br>(4.0 to 7.2)	6.1%<br>(-0.8 to 13.9)	1.3%<br>(0.2 to 2.6)	6.1%<br>(5.1 to 7.2)	1.6%<br>(1.0 to 2.3)	6.5%<br>(5.2 to 8.0)
Philippines	50.0%<br>(36.0 to 61.4)	1.0%<br>(0.9 to 1.1)	17.8%<br>(9.1 to 28.8)	12.7%<br>(9.8 to 15.7)	4.6%<br>(-0.6 to 10.2)	1.0%<br>(0.2 to 1.9)	0.1%<br>(0.0 to 0.2)	5.0%<br>(3.4 to 6.7)	15.1%<br>(12.7 to 17.7)
Poland	55.8%<br>(41.2 to 67.5)	0.0%<br>(-0.2 to 0.1)	0.5%<br>(0.0 to 4.6)	7.7%<br>(5.4 to 10.1)	4.4%<br>(-0.6 to 10.1)	2.9%<br>(0.4 to 6.0)	8.9%<br>(8.0 to 11.0)	3.3%<br>(2.2 to 4.5)	14.2%<br>(12.2 to 16.5)
Portugal	53.5%<br>(39.4 to 65.4)	0.0%<br>(0.0 to 0.1)	0.0%<br>(0.0 to 0.1)	6.1%<br>(4.2 to 8.1)	7.0%<br>(-0.9 to 15.7)	2.2%<br>(-0.8 to 5.4)	8.2%<br>(7.7 to 8.8)	2.9%<br>(2.0 to 3.9)	9.4%<br>(7.9 to 10.9)
Puerto Rico	55.9%<br>(41.0 to 68.3)	0.4%<br>(0.3 to 0.6)	0.0%<br>(0.0 to 0.0)	7.5%<br>(5.4 to 9.6)	3.5%<br>(-0.4 to 8.0)	1.9%<br>(0.3 to 3.6)	0.0%<br>(-0.1 to 0.1)	1.6%<br>(1.1 to 2.2)	9.6%<br>(7.7 to 11.9)
Qatar	57.8%<br>(43.3 to 69.1)	9.1%<br>(3.2 to 15.7)	0.0%<br>(0.0 to 0.0)	9.5%<br>(6.6 to 12.6)	4.2%<br>(-0.5 to 9.3)	3.5%<br>(0.7 to 6.8)	3.3%<br>(0.8 to 6.6)	4.3%<br>(2.9 to 5.7)	7.8%<br>(6.1 to 9.5)
Republic of Korea	43.5%<br>(29.2 to 55.1)	0.3%<br>(-0.4 to 1.6)	0.0%<br>(0.0 to 0.0)	7.4%<br>(4.8 to 10.0)	4.8%<br>(-0.6 to 10.9)	2.5%<br>(0.2 to 5.3)	6.3%<br>(5.5 to 7.3)	3.4%<br>(2.3 to 4.5)	13.3%<br>(11.1 to 15.7)
Republic of Moldova	69.1%<br>(53.5 to 80.2)	0.1%<br>(-0.7 to 1.1)	5.7%<br>(2.0 to 10.9)	13.1%<br>(10.0 to 16.5)	3.9%<br>(-0.5 to 9.0)	2.2%<br>(0.6 to 4.0)	8.0%<br>(6.9 to 9.8)	3.1%<br>(2.2 to 4.2)	15.2%<br>(13.1 to 17.6)
Romania	65.7%<br>(50.7 to 76.7)	0.2%<br>(-0.4 to 1.2)	0.6%<br>(0.0 to 5.8)	7.8%<br>(5.2 to 10.2)	4.1%<br>(-0.5 to 9.1)	2.4%<br>(0.1 to 4.9)	8.8%<br>(7.8 to 10.4)	3.5%<br>(2.4 to 4.7)	11.5%<br>(9.8 to 13.4)
Russian Federation	61.8%<br>(46.9 to 72.8)	-0.1%<br>(-0.5 to 0.3)	0.1%<br>(0.0 to 1.0)	9.9%<br>(7.0 to 12.8)	3.0%<br>(-0.4 to 6.9)	2.5%<br>(0.1 to 5.3)	8.7%<br>(7.0 to 10.4)	3.1%<br>(2.1 to 4.3)	15.5%<br>(13.6 to 17.7)
Rwanda	54.8%<br>(39.8 to 68.0)	0.0%<br>(0.0 to 0.0)	46.1%<br>(38.7 to 53.8)	8.0%<br>(5.6 to 10.4)	7.6%<br>(-1.0 to 17.1)	0.7%<br>(0.1 to 1.6)	4.2%<br>(3.6 to 5.5)	2.3%<br>(1.5 to 3.1)	12.2%<br>(9.4 to 15.5)
Saint Kitts and Nevis	58.1%<br>(43.8 to 70.4)	0.4%<br>(0.2 to 0.5)	0.1%<br>(0.0 to 0.6)	8.7%<br>(6.3 to 11.1)	5.3%<br>(-0.7 to 12.1)	2.2%<br>(-0.1 to 4.6)	0.0%<br>(-0.1 to 0.1)	1.9%<br>(1.3 to 2.6)	4.7%<br>(3.7 to 6.0)
Saint Lucia	56.1%<br>(41.1 to 68.2)	0.4%<br>(0.3 to 0.7)	0.6%<br>(0.0 to 3.5)	8.5%<br>(6.1 to 10.9)	7.8%<br>(-1.1 to 17.4)	2.0%<br>(-0.1 to 4.4)	0.0%<br>(0.0 to 0.1)	1.7%<br>(1.1 to 2.4)	6.7%<br>(5.5 to 8.1)
Saint Vincent and the Grenadines	53.6%<br>(38.7 to 65.8)	0.4%<br>(0.3 to 0.6)	0.7%<br>(0.0 to 3.1)	8.6%<br>(6.3 to 10.9)	8.7%<br>(-1.2 to 19.4)	1.9%<br>(0.1 to 4.0)	0.0%<br>(0.0 to 0.0)	2.3%<br>(1.5 to 3.1)	7.1%<br>(5.8 to 8.6)

Samoa	54.2% (39.5 to 66.8)	0.0% (0.0 to 0.1)	26.6% (12.7 to 38.2)	10.2% (7.8 to 12.6)	2.1% (-0.3 to 5.0)	1.7% (0.6 to 2.9)	0.1% (-0.1 to 0.3)	5.8% (4.0 to 7.7)	15.6% (13.1 to 18.4)
San Marino	57.4% (43.4 to 69.5)	0.1% (-0.2 to 0.5)	0.0% (0.0 to 0.0)	7.5% (5.2 to 10.0)	2.8% (-0.4 to 6.4)	2.8% (-0.2 to 6.2)	6.9% (5.8 to 7.8)	2.0% (1.3 to 2.7)	11.4% (9.0 to 14.1)
Sao Tome and Principe	65.6% (50.7 to 77.2)	0.2% (0.0 to 0.4)	24.9% (17.6 to 32.3)	11.4% (8.7 to 14.3)	7.0% (-1.0 to 15.9)	1.2% (0.2 to 2.2)	0.0% (0.0 to 0.1)	1.1% (0.7 to 1.6)	3.3% (2.5 to 4.3)
Saudi Arabia	54.9% (39.4 to 67.0)	8.8% (2.5 to 16.0)	0.0% (0.0 to 0.0)	10.8% (7.9 to 13.8)	7.5% (-1.0 to 17.0)	4.6% (1.6 to 8.1)	3.3% (1.1 to 6.0)	4.3% (2.9 to 5.7)	8.4% (6.7 to 10.2)
Senegal	63.2% (48.4 to 74.3)	4.2% (3.0 to 6.0)	38.4% (30.3 to 46.4)	9.3% (6.8 to 11.9)	6.8% (-0.9 to 15.0)	1.9% (0.6 to 3.6)	0.3% (0.1 to 0.6)	3.7% (2.5 to 4.9)	4.8% (3.8 to 5.9)
Serbia	65.9% (50.6 to 76.8)	0.3% (-0.4 to 1.5)	2.6% (0.1 to 15.8)	6.7% (4.5 to 9.0)	3.9% (-0.5 to 8.9)	2.6% (-0.2 to 5.8)	8.7% (7.7 to 10.6)	4.5% (3.0 to 6.0)	12.6% (10.7 to 14.8)
Seychelles	60.9% (45.0 to 72.3)	0.4% (0.3 to 0.5)	0.0% (0.0 to 0.2)	13.0% (9.8 to 16.2)	3.0% (-0.4 to 7.0)	1.9% (0.0 to 3.9)	0.1% (-0.1 to 0.3)	4.5% (3.0 to 6.0)	14.5% (12.4 to 17.3)
Sierra Leone	65.1% (50.3 to 76.0)	0.7% (0.5 to 1.0)	41.7% (34.3 to 48.5)	10.4% (7.9 to 13.2)	8.6% (-1.2 to 18.6)	1.6% (0.5 to 2.9)	0.1% (0.0 to 0.3)	3.3% (2.1 to 4.6)	5.4% (4.3 to 6.7)
Singapore	40.6% (27.8 to 52.2)	0.4% (0.4 to 0.5)	0.0% (0.0 to 0.0)	9.6% (6.8 to 12.5)	5.7% (-0.7 to 12.9)	2.3% (0.7 to 4.2)	0.0% (-0.1 to 0.0)	2.5% (1.7 to 3.4)	8.4% (7.1 to 9.7)
Slovakia	63.3% (47.6 to 74.6)	0.0% (-0.3 to 0.4)	0.0% (0.0 to 0.2)	7.4% (5.1 to 9.8)	3.4% (-0.4 to 7.7)	2.3% (0.1 to 4.7)	8.4% (7.2 to 9.5)	4.0% (2.7 to 5.3)	12.6% (10.5 to 15.0)
Slovenia	62.4% (46.4 to 73.2)	0.1% (-0.2 to 0.5)	0.2% (0.0 to 1.5)	7.2% (4.8 to 9.8)	3.0% (-0.4 to 6.9)	2.3% (-0.7 to 5.4)	7.6% (6.8 to 9.4)	3.4% (2.3 to 4.6)	10.5% (8.7 to 12.4)
Solomon Islands	44.7% (30.5 to 57.3)	0.3% (0.1 to 0.6)	44.6% (36.8 to 52.1)	9.9% (7.7 to 12.3)	5.0% (-0.6 to 11.3)	1.2% (0.4 to 2.1)	0.0% (0.0 to 0.1)	6.6% (4.4 to 8.8)	17.1% (14.4 to 19.8)
Somalia	54.5% (38.9 to 66.8)	0.9% (0.0 to 1.5)	46.1% (38.6 to 53.3)	7.9% (5.6 to 10.3)	13.8% (-1.9 to 29.6)	0.6% (0.1 to 1.2)	0.4% (0.3 to 0.6)	2.8% (1.7 to 3.9)	6.1% (4.3 to 8.4)
South Africa	63.7% (47.7 to 75.0)	0.1% (-0.1 to 0.4)	4.0% (0.7 to 10.4)	12.9% (9.8 to 16.0)	5.1% (-0.6 to 11.3)	3.1% (0.6 to 6.0)	6.1% (5.4 to 6.7)	4.1% (2.8 to 5.5)	7.6% (6.4 to 8.9)
South Sudan	55.4% (39.6 to 67.6)	5.2% (3.8 to 7.2)	42.1% (33.9 to 49.5)	7.9% (5.6 to 10.3)	8.4% (-1.1 to 19.1)	0.6% (0.1 to 1.3)	0.4% (0.1 to 0.8)	2.5% (1.6 to 3.5)	6.4% (4.8 to 8.5)
Spain	55.2% (40.5 to 67.1)	0.1% (-0.1 to 0.4)	0.0% (0.0 to 0.1)	6.6% (4.4 to 8.9)	5.7% (-0.7 to 13.2)	2.4% (-0.1 to 5.1)	6.6% (5.9 to 7.3)	3.1% (2.1 to 4.1)	12.1% (10.2 to 14.4)
Sri Lanka	60.4% (44.6 to 71.8)	0.6% (0.4 to 0.7)	10.5% (0.8 to 33.8)	12.2% (8.8 to 15.6)	3.4% (-0.4 to 7.6)	1.1% (0.0 to 2.4)	0.4% (0.2 to 0.6)	3.2% (2.1 to 4.7)	6.0% (4.9 to 7.2)
Sudan	62.8% (48.0 to 74.3)	9.0% (4.8 to 14.5)	19.3% (10.9 to 28.0)	10.1% (7.2 to 13.0)	11.1% (-1.6 to 24.5)	7.1% (3.0 to 11.3)	1.3% (0.3 to 2.7)	3.7% (2.5 to 4.9)	7.4% (5.8 to 9.4)
Suriname	47.9% (33.2 to 60.6)	0.4% (0.2 to 0.8)	1.0% (0.0 to 6.3)	8.6% (6.6 to 10.8)	7.1% (-0.9 to 16.0)	2.0% (0.6 to 3.7)	0.0% (-0.1 to 0.1)	3.6% (2.4 to 4.7)	11.7% (9.5 to 14.1)
Sweden	54.9% (40.2 to 66.2)	0.0% (-0.1 to 0.0)	0.0% (0.0 to 0.0)	8.1% (5.4 to 10.7)	2.3% (-0.3 to 5.4)	2.2% (-0.3 to 4.8)	7.9% (6.9 to 9.0)	1.5% (1.0 to 2.1)	12.0% (9.8 to 14.3)
Switzerland	50.2% (35.4 to 62.4)	0.0% (-0.1 to 0.1)	0.0% (0.0 to 0.0)	8.0% (5.4 to 10.8)	4.1% (-0.5 to 9.5)	2.4% (-0.4 to 5.4)	7.6% (6.8 to 9.3)	2.0% (1.3 to 2.7)	12.2% (10.1 to 14.6)

Syrian Arab Republic	54.5% (40.7 to 66.1)	1.5% (-0.2 to 4.1)	0.1% (0.0 to 0.2)	9.8% (6.9 to 12.7)	8.9% (-1.2 to 19.8)	3.6% (1.1 to 6.6)	5.7% (4.7 to 6.5)	4.5% (3.1 to 6.1)	12.0% (9.9 to 14.1)
Turkey	54.7% (40.3 to 66.1)	0.3% (-0.3 to 1.0)	0.1% (0.0 to 0.8)	10.0% (6.9 to 13.3)	5.1% (-0.7 to 11.4)	3.0% (0.2 to 6.3)	7.5% (6.6 to 8.3)	5.1% (3.5 to 6.9)	11.8% (9.9 to 13.8)
Taiwan (Province of China)	48.6% (34.3 to 61.5)	1.2% (-0.8 to 4.2)	0.1% (0.0 to 0.5)	7.6% (5.7 to 9.6)	5.8% (-0.7 to 13.2)	2.5% (0.9 to 4.5)	2.1% (1.6 to 2.5)	4.7% (3.2 to 6.2)	15.3% (13.2 to 17.6)
Tajikistan	60.3% (44.9 to 72.3)	0.7% (-0.4 to 2.1)	21.3% (14.3 to 28.4)	11.8% (8.7 to 14.8)	6.3% (-0.8 to 14.4)	1.7% (0.2 to 3.6)	8.0% (6.8 to 8.9)	3.3% (2.3 to 4.4)	7.1% (5.5 to 9.1)
Thailand	48.5% (34.1 to 60.9)	2.0% (1.8 to 2.5)	1.4% (0.0 to 9.3)	11.6% (9.0 to 14.2)	3.5% (-0.4 to 8.1)	1.4% (0.3 to 2.8)	0.7% (0.2 to 1.3)	4.1% (2.9 to 5.5)	14.3% (12.4 to 16.5)
Timor-Leste	60.8% (46.4 to 72.6)	0.1% (0.0 to 0.2)	28.9% (11.8 to 44.1)	12.4% (9.3 to 15.4)	8.8% (-1.2 to 19.0)	0.7% (0.1 to 1.4)	0.6% (0.4 to 0.9)	5.5% (3.6 to 7.4)	12.8% (10.2 to 15.7)
Togo	63.3% (47.6 to 74.9)	2.4% (2.0 to 3.0)	39.8% (32.6 to 46.6)	10.4% (7.8 to 13.0)	7.6% (-1.0 to 17.0)	1.0% (0.2 to 1.9)	0.1% (0.0 to 0.2)	2.5% (1.7 to 3.3)	7.4% (5.8 to 9.3)
Tokelau	52.3% (36.7 to 63.3)	0.6% (0.2 to 1.1)	0.0% (0.0 to 0.1)	9.4% (7.1 to 11.7)	1.7% (-0.2 to 4.0)	1.4% (0.5 to 2.5)	0.0% (0.0 to 0.0)	5.2% (3.4 to 6.9)	11.5% (9.0 to 14.1)
Tonga	58.5% (43.3 to 70.9)	0.4% (0.2 to 0.6)	18.0% (8.0 to 27.9)	10.1% (7.7 to 12.7)	2.3% (-0.3 to 5.3)	1.8% (0.6 to 3.3)	0.0% (0.0 to 0.0)	5.8% (4.0 to 7.7)	14.0% (11.4 to 16.7)
Trinidad and Tobago	59.7% (45.2 to 71.1)	0.3% (0.1 to 0.5)	0.0% (0.0 to 0.0)	8.1% (6.0 to 10.4)	3.3% (-0.4 to 7.5)	3.0% (0.8 to 5.6)	0.0% (-0.1 to 0.1)	3.3% (2.2 to 4.4)	9.4% (7.7 to 11.4)
Tunisia	51.8% (37.8 to 64.0)	2.2% (-0.1 to 6.0)	0.1% (0.0 to 0.3)	9.7% (6.8 to 12.7)	7.7% (-1.1 to 17.3)	2.9% (0.2 to 6.1)	4.9% (3.9 to 5.9)	5.1% (3.4 to 6.6)	13.2% (10.2 to 16.3)
Turkmenistan	62.9% (48.7 to 74.8)	1.9% (0.1 to 4.4)	0.0% (0.0 to 0.1)	12.5% (9.7 to 15.4)	3.6% (-0.5 to 8.1)	1.9% (0.6 to 3.4)	7.5% (6.2 to 8.9)	4.6% (3.2 to 6.2)	11.3% (9.4 to 13.1)
Tuvalu	54.4% (39.2 to 67.0)	0.7% (0.3 to 1.1)	7.2% (4.1 to 11.5)	10.2% (7.9 to 12.6)	2.8% (-0.3 to 6.3)	1.3% (0.4 to 2.2)	0.0% (0.0 to 0.0)	6.5% (4.3 to 8.8)	14.7% (12.0 to 18.0)
Uganda	58.2% (43.7 to 69.8)	0.1% (0.0 to 0.2)	42.8% (35.3 to 49.9)	8.3% (5.9 to 10.8)	8.4% (-1.1 to 19.0)	0.8% (0.1 to 1.6)	1.3% (1.0 to 1.6)	1.6% (1.0 to 2.2)	5.0% (3.9 to 6.5)
Ukraine	64.1% (48.5 to 75.4)	-0.1% (-0.8 to 0.5)	1.0% (0.1 to 3.9)	10.9% (8.0 to 13.9)	2.4% (-0.3 to 5.6)	2.0% (0.4 to 4.1)	8.7% (7.1 to 10.0)	3.5% (2.4 to 4.7)	14.2% (10.9 to 17.6)
United Arab Emirates	56.0% (40.8 to 68.0)	11.7% (5.3 to 19.4)	0.0% (0.0 to 0.0)	10.2% (7.0 to 13.5)	4.5% (-0.6 to 10.1)	3.9% (0.9 to 7.3)	4.1% (1.0 to 8.6)	4.3% (2.9 to 5.7)	6.9% (5.3 to 8.5)
United Kingdom	50.9% (37.6 to 61.5)	0.0% (0.0 to 0.0)	0.0% (0.0 to 0.0)	7.4% (5.0 to 9.8)	3.1% (-0.4 to 7.0)	3.6% (-0.2 to 7.7)	7.1% (5.8 to 9.7)	1.8% (1.2 to 2.4)	12.1% (10.1 to 14.4)
United Republic of Tanzania	56.8% (41.9 to 69.1)	0.1% (0.0 to 0.4)	43.2% (35.3 to 50.6)	8.0% (5.6 to 10.7)	5.6% (-0.7 to 12.5)	0.4% (0.0 to 0.9)	1.6% (1.3 to 2.0)	2.1% (1.3 to 2.9)	7.7% (6.3 to 9.6)
United States of America	48.4% (34.4 to 60.5)	0.4% (-0.5 to 1.6)	0.0% (0.0 to 0.0)	8.8% (6.2 to 11.3)	3.0% (-0.4 to 6.9)	2.2% (0.1 to 4.6)	5.4% (4.6 to 6.1)	1.9% (1.3 to 2.6)	13.4% (11.4 to 15.7)
United States Virgin Islands	53.3% (38.6 to 65.2)	0.3% (0.2 to 0.5)	0.0% (0.0 to 0.0)	7.9% (5.8 to 10.0)	3.2% (-0.4 to 7.3)	1.6% (-0.2 to 3.4)	0.0% (-0.1 to 0.1)	2.2% (1.5 to 3.0)	7.6% (5.8 to 9.5)
Uruguay	55.5% (39.9 to 66.9)	0.1% (-0.1 to 0.3)	0.1% (0.0 to 1.2)	6.4% (4.4 to 8.4)	5.6% (-0.7 to 12.5)	1.9% (0.4 to 3.8)	6.2% (5.7 to 7.0)	3.2% (2.2 to 4.3)	13.6% (11.5 to 15.7)

Uzbekistan	59.1% (44.0 to 71.6)	0.7% (-0.5 to 2.2)	4.3% (0.8 to 11.7)	12.7% (9.7 to 15.8)	4.0% (-0.5 to 9.2)	1.2% (0.3 to 2.3)	7.7% (6.1 to 8.9)	4.0% (2.7 to 5.4)	9.3% (7.8 to 10.8)
Vanuatu	63.3% (48.0 to 74.4)	0.1% (0.1 to 0.2)	41.5% (33.4 to 49.4)	10.3% (7.9 to 12.7)	4.2% (-0.5 to 9.4)	0.5% (0.1 to 0.9)	0.3% (0.1 to 0.4)	3.5% (2.3 to 4.8)	9.3% (7.7 to 11.1)
Venezuela (Bolivarian Republic of)	57.0% (42.0 to 68.8)	0.5% (0.4 to 0.6)	0.2% (0.0 to 0.9)	10.2% (7.6 to 12.7)	8.3% (-1.1 to 18.9)	1.1% (0.0 to 2.4)	0.9% (0.7 to 1.2)	3.4% (2.2 to 4.6)	7.7% (6.4 to 9.3)
Viet Nam	63.4% (47.6 to 75.1)	1.8% (0.2 to 4.0)	16.2% (5.8 to 27.9)	10.3% (7.4 to 13.4)	5.8% (-0.8 to 13.0)	1.2% (0.3 to 2.5)	1.3% (0.8 to 1.9)	4.7% (3.1 to 6.2)	17.2% (14.6 to 19.7)
Yemen	52.9% (37.2 to 65.2)	1.6% (0.7 to 2.7)	17.1% (11.4 to 23.5)	9.7% (7.0 to 12.5)	13.8% (-2.0 to 29.7)	3.9% (1.2 to 6.9)	2.9% (2.4 to 3.6)	5.2% (3.5 to 6.9)	13.0% (10.7 to 15.6)
Zambia	53.0% (38.3 to 66.0)	0.6% (-0.4 to 2.1)	37.4% (27.1 to 46.1)	8.1% (5.8 to 10.7)	6.7% (-0.9 to 15.0)	0.9% (0.2 to 1.8)	2.6% (2.2 to 3.1)	2.4% (1.5 to 3.5)	6.5% (5.1 to 8.0)
Zimbabwe	64.9% (49.8 to 76.0)	0.5% (-0.4 to 1.8)	37.9% (30.9 to 45.1)	12.4% (9.5 to 15.3)	8.0% (-1.1 to 17.8)	1.3% (0.2 to 2.5)	3.4% (2.6 to 4.3)	3.6% (2.4 to 4.7)	10.2% (8.1 to 12.8)

Appendix Table 12. Age-standardized percent of DALYs (with 95% uncertainty intervals) due to ischemic stroke associated with individual risk factors by 7 GBD super-region, 21 GBD region and country for both sexes combined in 2021.

Country/region	Alcohol use	Ambient particulate matter pollution	Diet high in processed meat	Diet high in red meat	Diet high in sodium	Diet high in sugar-sweetened beverages	Diet low in fiber	Diet low in fruits	Diet low in polyunsaturated fatty acids	Diet low in vegetables	Diet low in whole grains	High body-mass index	High fasting plasma glucose	High LDL cholesterol
**GBD super-regions in alphabetical order**														
**Central Europe, Eastern Europe, and Central Asia**	7.4% (-0.8 to 19.0)	10.6% (7.6 to 14.4)	1.4% (0.3 to 2.5)	0.0% (-0.7 to 1.7)	8.4% (1.5 to 20.2)	0.3% (0.1 to 0.4)	1.0% (0.0 to 2.2)	2.0% (1.0 to 3.2)	0.0% (0.0 to 0.0)	0.8% (0.3 to 1.4)	5.6% (-5.9 to 15.7)	9.9% (1.5 to 18.7)	16.4% (13.0 to 20.4)	34.2% (12.1 to 54.2)
**High-income**	9.3% (-1.4 to 22.7)	6.8% (4.6 to 9.2)	2.2% (0.5 to 3.8)	1.2% (-0.8 to 3.5)	5.8% (0.4 to 16.4)	0.6% (0.3 to 0.9)	1.4% (-0.1 to 3.0)	1.6% (0.7 to 2.6)	0.0% (0.0 to 0.0)	1.1% (0.4 to 1.8)	4.0% (-4.1 to 11.6)	8.0% (1.2 to 14.9)	19.8% (16.0 to 24.0)	31.0% (10.7 to 50.0)
**Latin America and Caribbean**	4.8% (-0.7 to 12.1)	9.7% (6.8 to 13.0)	0.4% (0.1 to 0.6)	0.8% (-0.5 to 2.7)	6.8% (0.4 to 18.6)	0.4% (0.2 to 0.6)	1.1% (0.0 to 2.5)	1.3% (0.6 to 2.0)	0.0% (0.0 to 0.0)	2.9% (1.3 to 4.6)	3.1% (-2.9 to 8.8)	8.6% (1.3 to 16.4)	19.6% (15.5 to 23.9)	31.4% (10.9 to 50.5)
**North Africa and Middle East**	0.4% (0.0 to 1.2)	23.9% (19.0 to 29.0)	0.2% (0.1 to 0.4)	-0.8% (-2.2 to 0.4)	1.5% (0.0 to 7.5)	0.2% (0.1 to 0.3)	0.6% (0.0 to 1.3)	1.3% (0.7 to 2.0)	0.0% (0.0 to 0.0)	1.1% (0.6 to 1.7)	6.5% (-7.2 to 18.4)	11.5% (1.9 to 21.2)	21.8% (17.5 to 26.6)	30.9% (10.9 to 49.6)
**South Asia**	2.4% (-0.3 to 6.6)	17.0% (10.0 to 23.2)	0.3% (0.1 to 0.5)	-0.2% (-0.6 to 0.1)	5.7% (0.2 to 17.1)	0.1% (0.1 to 0.2)	1.9% (-0.1 to 4.0)	8.3% (4.4 to 12.9)	0.0% (0.0 to 0.1)	3.3% (1.5 to 5.2)	3.5% (-3.5 to 9.7)	2.8% (0.4 to 5.3)	19.7% (15.7 to 24.4)	24.9% (8.3 to 42.0)
**Southeast Asia, East Asia, and Oceania**	5.6% (-0.9 to 13.5)	21.9% (14.5 to 27.2)	0.2% (0.1 to 0.4)	0.4% (-0.2 to 2.1)	15.0% (4.5 to 28.6)	0.1% (0.1 to 0.2)	1.4% (-0.1 to 2.9)	1.7% (0.8 to 2.7)	0.0% (0.0 to 0.0)	0.8% (0.4 to 1.3)	3.7% (-3.6 to 10.4)	4.3% (0.6 to 8.2)	16.4% (12.8 to 20.2)	28.3% (9.6 to 46.6)
**Sub-Saharan Africa**	5.0% (-0.9 to 12.2)	10.7% (6.5 to 15.1)	0.3% (0.1 to 0.6)	-0.5% (-1.5 to 0.3)	4.6% (0.1 to 14.2)	0.1% (0.0 to 0.1)	0.4% (0.0 to 0.9)	3.4% (1.6 to 5.5)	0.0% (0.0 to 0.1)	4.7% (2.2 to 7.4)	3.4% (-3.4 to 9.8)	5.1% (0.8 to 9.6)	14.8% (11.6 to 18.2)	23.9% (7.8 to 40.9)
**GBD regions in alphabetical order**														
**Andean Latin America**	5.0% (-0.8 to 12.5)	16.9% (10.6 to 23.8)	0.2% (0.0 to 0.3)	-0.8% (-2.3 to 0.4)	6.4% (0.2 to 18.2)	0.3% (0.1 to 0.5)	1.6% (-0.1 to 3.4)	1.4% (0.6 to 2.2)	0.0% (0.0 to 0.0)	3.8% (1.4 to 6.1)	3.6% (-3.6 to 10.4)	8.3% (1.3 to 15.7)	17.1% (13.6 to 20.9)	29.5% (10.1 to 48.2)
**Australasia**	10.8% (-1.4 to 26.9)	4.9% (2.9 to 7.4)	2.3% (0.6 to 4.1)	3.3% (-2.2 to 8.4)	2.3% (0.0 to 9.9)	0.8% (0.4 to 1.2)	1.4% (-0.1 to 3.1)	1.4% (0.5 to 2.5)	0.0% (0.0 to 0.0)	1.1% (0.2 to 2.0)	3.1% (-3.2 to 9.4)	8.9% (1.4 to 17.6)	19.1% (15.1 to 23.2)	32.2% (10.9 to 52.4)
**Caribbean**	4.7% (-0.6 to 12.0)	11.3% (5.9 to 18.5)	0.2% (0.1 to 0.4)	-0.8% (-2.4 to 0.4)	4.2% (0.0 to 13.9)	0.2% (0.1 to 0.4)	1.2% (-0.1 to 2.5)	1.4% (0.7 to 2.2)	0.0% (0.0 to 0.0)	4.1% (2.2 to 6.2)	4.3% (-4.4 to 12.2)	6.9% (1.1 to 13.1)	19.0% (15.1 to 23.1)	29.1% (10.0 to 47.4)
**Central Asia**	4.4% (-0.5 to 12.3)	17.1% (11.8 to 22.2)	0.6% (0.1 to 1.0)	0.4% (-0.3 to 2.5)	6.6% (0.4 to 18.1)	0.2% (0.1 to 0.2)	1.2% (-0.1 to 2.5)	2.1% (1.1 to 3.2)	0.0% (0.0 to 0.0)	0.3% (0.1 to 0.6)	7.8% (-8.8 to 21.4)	9.3% (1.4 to 17.6)	15.6% (12.2 to 19.1)	30.2% (10.6 to 48.8)
**Central Europe**	9.5% (-1.2 to 22.7)	12.9% (9.7 to 16.3)	1.2% (0.3 to 2.1)	0.5% (-0.3 to 2.4)	15.4% (4.1 to 29.4)	0.6% (0.3 to 0.9)	0.9% (-0.1 to 1.9)	1.5% (0.6 to 2.5)	0.0% (0.0 to 0.0)	0.4% (0.1 to 0.8)	4.2% (-4.2 to 12.3)	9.1% (1.4 to 17.4)	21.2% (17.0 to 25.7)	32.9% (11.3 to 53.0)
**Central Latin America**	3.7% (-0.6 to 9.7)	10.0% (7.0 to 13.3)	0.3% (0.1 to 0.5)	-0.6% (-1.8 to 0.6)	7.6% (0.7 to 19.2)	0.4% (0.2 to 0.6)	0.8% (0.0 to 1.8)	1.4% (0.7 to 2.3)	0.0% (0.0 to 0.0)	2.6% (1.1 to 4.1)	2.5% (-2.4 to 7.3)	9.6% (1.5 to 18.3)	20.2% (16.2 to 24.8)	30.5% (10.6 to 49.3)
**Central Sub-Saharan Africa**	4.1% (-0.6 to 10.7)	7.4% (4.4 to 11.0)	0.3% (0.1 to 0.6)	-0.3% (-1.0 to 0.2)	2.2% (0.0 to 9.7)	0.1% (0.0 to 0.1)	1.0% (-0.1 to 2.3)	2.5% (1.1 to 4.0)	0.0% (0.0 to 0.1)	7.3% (3.1 to 11.6)	3.1% (-3.0 to 9.1)	4.2% (0.6 to 8.0)	18.1% (14.4 to 22.4)	23.0% (7.3 to 39.8)

**East Asia**	6.2% (-1.0 to 15.0)	24.5% (16.8 to 30.0)	0.3% (0.1 to 0.5)	0.8% (-0.4 to 2.9)	16.1% (5.3 to 29.9)	0.1% (0.1 to 0.2)	0.8% (0.0 to 1.6)	1.5% (0.7 to 2.5)	0.0% (0.0 to 0.0)	0.1% (0.0 to 0.3)	4.2% (-4.2 to 11.9)	4.7% (0.7 to 9.1)	16.6% (13.0 to 20.4)	28.6% (9.6 to 47.0)
**Eastern Europe**	6.9% (-0.7 to 18.7)	8.4% (5.5 to 12.6)	1.6% (0.4 to 2.9)	-0.3% (-1.5 to 1.2)	5.6% (0.3 to 16.2)	0.2% (0.1 to 0.3)	1.1% (0.0 to 2.3)	2.3% (1.1 to 3.5)	0.0% (0.0 to 0.0)	1.1% (0.5 to 1.8)	5.6% (-5.9 to 15.9)	10.3% (1.6 to 19.6)	14.4% (11.3 to 18.0)	35.3% (12.7 to 55.4)
**Eastern Sub-Saharan Africa**	4.0% (-0.7 to 9.9)	4.8% (3.0 to 7.1)	0.2% (0.0 to 0.3)	-0.5% (-1.4 to 0.3)	7.7% (0.5 to 20.3)	0.0% (0.0 to 0.1)	0.4% (0.0 to 1.0)	3.8% (1.8 to 6.2)	0.0% (0.0 to 0.1)	6.1% (2.7 to 9.4)	3.2% (-3.1 to 9.2)	3.3% (0.5 to 6.3)	11.9% (9.3 to 14.9)	22.5% (7.2 to 39.1)
**High-income Asia Pacific**	8.8% (-1.5 to 21.1)	11.3% (7.0 to 16.5)	1.6% (0.4 to 2.8)	-0.5% (-1.8 to 0.8)	10.7% (1.6 to 23.9)	0.4% (0.2 to 0.7)	2.0% (-0.1 to 4.1)	2.2% (1.2 to 3.4)	0.0% (0.0 to 0.0)	0.5% (0.1 to 0.9)	3.1% (-3.1 to 8.8)	3.4% (0.5 to 6.5)	19.9% (16.0 to 24.2)	30.9% (10.9 to 49.5)
**High-income North America**	7.6% (-1.0 to 19.4)	3.1% (1.5 to 5.0)	3.2% (0.8 to 5.5)	1.6% (-1.1 to 4.5)	4.5% (0.1 to 14.3)	0.8% (0.4 to 1.3)	1.3% (0.0 to 2.8)	1.7% (0.8 to 2.6)	0.0% (0.0 to 0.0)	1.4% (0.6 to 2.3)	4.0% (-4.1 to 11.4)	11.2% (1.8 to 20.5)	23.2% (18.7 to 28.0)	29.2% (10.3 to 47.3)
**North Africa and Middle East**	0.4% (0.0 to 1.2)	23.9% (19.0 to 29.0)	0.2% (0.1 to 0.4)	-0.8% (-2.2 to 0.4)	1.5% (0.0 to 7.5)	0.2% (0.1 to 0.3)	0.6% (0.0 to 1.3)	1.3% (0.7 to 2.0)	0.0% (0.0 to 0.0)	1.1% (0.6 to 1.7)	6.5% (-7.2 to 18.4)	11.5% (1.9 to 21.2)	21.8% (17.5 to 26.6)	30.9% (10.9 to 49.6)
**Oceania**	1.2% (-0.2 to 3.2)	5.2% (1.9 to 11.1)	0.1% (0.0 to 0.2)	-0.8% (-2.4 to 0.4)	9.1% (0.9 to 21.5)	0.1% (0.1 to 0.2)	0.1% (0.0 to 0.2)	2.6% (1.2 to 4.2)	0.0% (0.0 to 0.0)	4.4% (2.0 to 6.6)	5.2% (-5.6 to 14.7)	6.0% (1.0 to 11.1)	21.2% (16.7 to 26.0)	27.6% (9.4 to 45.5)
**South Asia**	2.4% (-0.3 to 6.6)	17.0% (10.0 to 23.2)	0.3% (0.1 to 0.5)	-0.2% (-0.6 to 0.1)	5.7% (0.2 to 17.1)	0.1% (0.1 to 0.2)	1.9% (-0.1 to 4.0)	8.3% (4.4 to 12.9)	0.0% (0.0 to 0.1)	3.3% (1.5 to 5.2)	3.5% (-3.5 to 9.7)	2.8% (0.4 to 5.3)	19.7% (15.7 to 24.4)	24.9% (8.3 to 42.0)
**Southeast Asia**	3.5% (-0.6 to 8.0)	13.1% (7.7 to 17.8)	0.1% (0.0 to 0.2)	-0.6% (-1.7 to 0.3)	11.8% (2.0 to 25.1)	0.1% (0.0 to 0.1)	3.3% (-0.2 to 6.7)	2.1% (1.1 to 3.3)	0.0% (0.0 to 0.1)	2.8% (1.3 to 4.4)	2.1% (-1.9 to 5.8)	2.9% (0.4 to 5.5)	15.9% (12.3 to 19.7)	27.5% (9.2 to 45.4)
**Southern Latin America**	8.8% (-1.3 to 21.1)	11.5% (6.6 to 17.4)	1.2% (0.3 to 2.0)	2.8% (-1.8 to 7.0)	6.8% (0.2 to 18.6)	0.7% (0.4 to 1.2)	1.7% (-0.1 to 3.8)	1.2% (0.5 to 1.9)	0.0% (0.0 to 0.0)	1.4% (0.4 to 2.3)	5.1% (-5.3 to 14.9)	9.7% (1.5 to 18.8)	19.6% (15.6 to 23.9)	31.1% (10.6 to 50.3)
**Southern Sub-Saharan Africa**	5.1% (-0.8 to 13.2)	13.3% (9.2 to 17.7)	0.2% (0.0 to 0.3)	-0.6% (-1.9 to 0.5)	2.3% (0.0 to 10.2)	0.1% (0.1 to 0.2)	0.5% (0.0 to 1.2)	4.7% (2.2 to 7.7)	0.0% (0.0 to 0.0)	4.1% (1.7 to 6.4)	1.9% (-1.8 to 5.6)	9.4% (1.5 to 18.1)	16.9% (13.3 to 20.8)	25.1% (8.1 to 42.8)
**Tropical Latin America**	5.5% (-0.8 to 13.4)	8.0% (4.5 to 12.4)	0.4% (0.1 to 0.8)	2.3% (-1.4 to 5.7)	7.0% (0.3 to 19.9)	0.4% (0.2 to 0.6)	1.2% (0.0 to 2.7)	1.1% (0.5 to 1.8)	0.0% (0.0 to 0.0)	2.7% (1.2 to 4.4)	3.0% (-2.8 to 8.6)	8.6% (1.3 to 16.5)	19.6% (15.4 to 24.1)	33.0% (11.5 to 52.6)
**Western Europe**	11.0% (-1.7 to 25.8)	6.7% (4.7 to 9.1)	1.7% (0.4 to 3.1)	1.3% (-0.8 to 3.7)	4.1% (0.1 to 13.4)	0.4% (0.2 to 0.6)	1.2% (-0.1 to 2.6)	1.3% (0.5 to 2.1)	0.0% (0.0 to 0.0)	1.1% (0.2 to 1.9)	4.2% (-4.4 to 12.8)	7.3% (1.1 to 14.3)	17.2% (13.5 to 21.2)	32.4% (11.0 to 52.5)
**Western Sub-Saharan Africa**	5.8% (-1.0 to 13.8)	14.0% (7.8 to 20.5)	0.4% (0.1 to 0.8)	-0.6% (-1.6 to 0.3)	3.9% (0.0 to 13.5)	0.1% (0.0 to 0.1)	0.2% (0.0 to 0.5)	3.0% (1.5 to 4.8)	0.0% (0.0 to 0.1)	3.5% (1.7 to 5.5)	4.0% (-4.0 to 11.3)	5.3% (0.8 to 9.9)	15.3% (12.0 to 18.8)	24.7% (8.2 to 41.7)
**Countries in alphabetical order**														
Afghanistan	0.1% (0.0 to 0.2)	7.1% (4.3 to 10.6)	0.2% (0.0 to 0.3)	-1.0% (-2.8 to 0.5)	1.5% (0.0 to 7.6)	0.0% (0.0 to 0.1)	3.1% (-0.2 to 6.1)	3.3% (1.8 to 5.1)	0.0% (0.0 to 0.1)	10.0% (6.0 to 14.4)	8.5% (-9.3 to 22.8)	7.2% (1.1 to 13.5)	23.5% (18.5 to 28.6)	31.1% (11.0 to 49.9)
Albania	5.5% (-0.6 to 13.7)	11.1% (6.7 to 14.5)	3.9% (0.9 to 6.8)	-0.2% (-1.1 to 1.0)	15.9% (4.6 to 29.6)	0.7% (0.3 to 1.1)	0.5% (0.0 to 1.3)	0.6% (0.1 to 1.2)	0.0% (0.0 to 0.0)	0.1% (-0.1 to 0.3)	5.5% (-5.9 to 16.8)	7.9% (1.2 to 16.1)	17.2% (13.4 to 21.2)	30.3% (10.1 to 50.5)
Algeria	0.4% (0.0 to 1.2)	17.8% (12.5 to 24.1)	0.2% (0.1 to 0.4)	-0.7% (-1.9 to 0.3)	1.3% (0.0 to 7.1)	0.2% (0.1 to 0.4)	0.3% (0.0 to 0.8)	1.2% (0.4 to 2.0)	0.0% (0.0 to 0.0)	1.0% (0.2 to 1.7)	5.9% (-6.5 to 17.4)	8.8% (1.4 to 17.1)	24.4% (19.5 to 29.7)	27.6% (9.1 to 45.8)
American Samoa	0.5% (0.0 to 1.7)	3.3% (0.4 to 6.8)	0.3% (0.1 to 0.6)	-0.9% (-3.0 to 0.6)	5.0% (0.1 to 15.4)	0.4% (0.2 to 0.7)	0.0% (0.0 to 0.1)	1.9% (0.9 to 2.9)	0.0% (0.0 to 0.0)	4.3% (2.1 to 6.7)	4.9% (-5.2 to 13.7)	13.3% (2.3 to 23.6)	30.3% (24.9 to 36.2)	28.9% (10.1 to 47.3)
Andorra	10.5% (-1.3 to 25.3)	5.0% (2.7 to 7.6)	2.1% (0.5 to 3.8)	1.6% (-0.9 to 4.4)	2.8% (0.0 to 11.3)	0.6% (0.3 to 1.0)	0.8% (-0.1 to 2.0)	1.0% (0.3 to 1.8)	0.0% (0.0 to 0.0)	0.7% (0.1 to 1.3)	4.0% (-4.3 to 12.3)	6.4% (0.9 to 12.7)	17.6% (13.8 to 21.6)	32.6% (11.2 to 52.8)
Angola	4.8% (-0.8 to 12.0)	14.4% (6.8 to 21.8)	0.2% (0.0 to 0.3)	-0.7% (-1.9 to 0.4)	3.4% (0.0 to 12.9)	0.0% (0.0 to 0.1)	0.3% (0.0 to 0.8)	1.8% (0.8 to 2.9)	0.0% (0.0 to 0.0)	3.0% (1.0 to 5.0)	3.5% (-3.3 to 10.3)	3.4% (0.5 to 6.4)	15.4% (11.9 to 19.0)	23.3% (7.5 to 40.3)

Antigua and Barbuda	4.5% (-0.6 to 11.7)	13.7% (5.0 to 24.6)	0.1% (0.0 to 0.3)	-0.7% (-1.9 to 0.4)	4.4% (0.0 to 15.1)	0.1% (0.0 to 0.1)	3.0% (-0.2 to 6.7)	1.1% (0.4 to 1.8)	0.0% (0.0 to 0.0)	2.4% (0.7 to 4.2)	5.4% (-5.6 to 15.9)	7.5% (1.2 to 14.8)	20.6% (16.2 to 25.2)	27.0% (8.6 to 45.5)
Argentina	8.5% (-1.3 to 20.7)	10.0% (5.1 to 16.4)	0.9% (0.2 to 1.7)	3.8% (-2.5 to 9.3)	6.6% (0.1 to 18.6)	0.9% (0.4 to 1.4)	2.0% (-0.1 to 4.4)	1.0% (0.4 to 1.7)	0.0% (0.0 to 0.0)	1.5% (0.5 to 2.5)	5.2% (-5.4 to 15.3)	9.7% (1.5 to 19.1)	20.0% (15.9 to 24.2)	31.4% (10.8 to 50.4)
Armenia	4.1% (-0.5 to 11.5)	22.9% (15.0 to 31.5)	0.4% (0.1 to 0.7)	-1.0% (-3.1 to 0.6)	6.8% (0.3 to 18.8)	0.1% (0.0 to 0.1)	1.1% (0.0 to 2.3)	1.2% (0.5 to 1.9)	0.0% (0.0 to 0.0)	0.0% (0.0 to 0.1)	7.6% (-8.4 to 21.0)	9.3% (1.4 to 17.8)	15.2% (11.9 to 18.7)	26.2% (8.8 to 43.7)
Australia	10.9% (-1.4 to 27.0)	5.3% (3.2 to 7.8)	2.4% (0.6 to 4.2)	3.4% (-2.3 to 8.6)	2.1% (0.0 to 9.3)	0.9% (0.4 to 1.3)	1.5% (-0.1 to 3.2)	1.5% (0.5 to 2.6)	0.0% (0.0 to 0.0)	1.1% (0.2 to 2.0)	3.1% (-3.1 to 9.2)	9.1% (1.4 to 18.0)	18.8% (14.8 to 23.0)	32.5% (11.1 to 52.8)
Austria	11.1% (-1.7 to 26.0)	7.3% (5.2 to 9.9)	1.6% (0.4 to 2.8)	1.7% (-1.1 to 4.9)	7.0% (0.3 to 19.4)	0.5% (0.2 to 0.8)	0.7% (-0.1 to 1.6)	1.0% (0.4 to 1.7)	0.0% (0.0 to 0.0)	1.0% (0.3 to 1.8)	3.0% (-2.9 to 9.1)	6.9% (1.0 to 14.0)	12.8% (10.1 to 16.1)	34.2% (12.1 to 55.0)
Azerbaijan	3.9% (-0.5 to 10.7)	15.9% (8.3 to 25.5)	0.7% (0.2 to 1.2)	-1.1% (-3.0 to 0.6)	6.4% (0.3 to 18.1)	0.2% (0.1 to 0.3)	0.6% (0.0 to 1.3)	1.4% (0.7 to 2.2)	0.0% (0.0 to 0.0)	0.1% (0.0 to 0.3)	7.4% (-8.3 to 20.2)	9.5% (1.5 to 18.3)	16.5% (12.9 to 20.4)	29.5% (10.2 to 47.8)
Bahamas	3.8% (-0.5 to 10.1)	13.2% (4.5 to 26.0)	0.2% (0.0 to 0.4)	-0.9% (-2.9 to 0.6)	4.2% (0.0 to 14.2)	0.1% (0.0 to 0.2)	2.7% (-0.1 to 5.7)	1.5% (0.7 to 2.4)	0.0% (0.0 to 0.0)	1.4% (0.5 to 2.3)	5.8% (-6.4 to 16.8)	9.7% (1.5 to 18.1)	20.8% (16.4 to 25.6)	29.2% (9.8 to 48.1)
Bahrain	0.4% (0.0 to 1.3)	36.2% (28.2 to 44.4)	0.4% (0.1 to 0.7)	-0.7% (-2.2 to 0.4)	1.4% (0.0 to 7.7)	0.5% (0.2 to 0.8)	0.1% (0.0 to 0.3)	0.7% (0.2 to 1.2)	0.0% (0.0 to 0.0)	0.3% (0.0 to 0.7)	5.2% (-5.5 to 15.6)	10.9% (1.7 to 20.6)	29.3% (23.6 to 34.6)	31.4% (10.6 to 51.4)
Bangladesh	0.2% (0.0 to 0.8)	8.4% (4.3 to 13.4)	0.6% (0.1 to 1.1)	-0.1% (-0.4 to 0.1)	6.1% (0.1 to 18.0)	0.0% (0.0 to 0.0)	4.2% (-0.3 to 9.3)	4.8% (1.8 to 8.3)	0.0% (0.0 to 0.0)	5.2% (1.6 to 8.9)	1.3% (-1.2 to 3.8)	1.8% (0.2 to 3.5)	19.0% (15.0 to 23.3)	20.1% (6.2 to 35.8)
Barbados	5.0% (-0.7 to 12.7)	16.9% (6.8 to 29.8)	0.3% (0.1 to 0.6)	-0.9% (-2.5 to 0.4)	2.8% (0.0 to 11.0)	0.3% (0.1 to 0.5)	1.7% (-0.1 to 3.9)	2.0% (0.7 to 3.4)	0.0% (0.0 to 0.0)	2.1% (0.5 to 3.5)	3.9% (-3.9 to 11.6)	8.9% (1.4 to 17.2)	25.0% (19.8 to 30.2)	28.2% (9.4 to 46.9)
Belarus	8.8% (-0.8 to 23.4)	11.1% (8.0 to 15.0)	1.0% (0.2 to 1.8)	2.2% (-1.1 to 6.0)	3.0% (0.0 to 11.9)	0.1% (0.0 to 0.1)	0.2% (0.0 to 0.4)	2.0% (1.0 to 3.1)	0.0% (0.0 to 0.0)	0.4% (0.1 to 0.8)	7.0% (-7.6 to 19.6)	10.4% (1.6 to 20.0)	11.0% (8.3 to 13.8)	31.2% (10.7 to 50.4)
Belgium	11.3% (-1.7 to 26.3)	7.1% (4.9 to 9.6)	2.3% (0.5 to 4.1)	1.8% (-1.2 to 4.7)	6.1% (0.1 to 17.3)	0.6% (0.3 to 1.0)	1.1% (-0.1 to 2.7)	1.3% (0.4 to 2.2)	0.0% (0.0 to 0.0)	0.6% (0.1 to 1.1)	4.0% (-4.1 to 12.3)	6.8% (1.0 to 13.2)	16.8% (13.1 to 20.5)	31.1% (10.7 to 51.0)
Belize	3.9% (-0.6 to 10.2)	17.4% (6.7 to 32.0)	0.2% (0.0 to 0.3)	-0.6% (-1.7 to 0.3)	4.4% (0.0 to 14.6)	0.1% (0.1 to 0.2)	0.7% (0.0 to 1.6)	0.5% (0.2 to 0.8)	0.0% (0.0 to 0.0)	3.6% (1.5 to 5.6)	3.8% (-3.8 to 11.2)	10.1% (1.6 to 18.9)	18.0% (14.1 to 22.2)	27.8% (9.2 to 46.3)
Benin	3.3% (-0.6 to 8.6)	8.9% (4.9 to 14.3)	0.3% (0.1 to 0.5)	-0.3% (-0.9 to 0.2)	4.5% (0.0 to 15.8)	0.0% (0.0 to 0.1)	0.0% (0.0 to 0.0)	3.1% (1.4 to 5.0)	0.0% (0.0 to 0.1)	2.6% (1.1 to 4.0)	2.9% (-2.9 to 8.4)	4.9% (0.7 to 9.5)	14.2% (11.0 to 17.5)	23.4% (7.5 to 40.6)
Bermuda	7.4% (-1.1 to 19.2)	3.2% (0.6 to 6.1)	0.3% (0.1 to 0.6)	0.4% (-0.5 to 2.4)	4.5% (0.0 to 15.1)	0.2% (0.1 to 0.3)	2.1% (-0.1 to 4.7)	1.5% (0.7 to 2.5)	0.0% (0.0 to 0.0)	1.2% (0.3 to 2.0)	4.7% (-4.9 to 14.1)	10.4% (1.7 to 19.9)	20.9% (16.6 to 25.5)	28.9% (9.8 to 47.5)
Bhutan	0.8% (-0.1 to 2.2)	19.7% (11.7 to 26.4)	0.5% (0.1 to 0.9)	-0.3% (-0.9 to 0.2)	6.1% (0.1 to 18.2)	0.0% (0.0 to 0.1)	2.0% (-0.1 to 4.5)	2.5% (1.1 to 4.2)	0.0% (0.0 to 0.0)	2.9% (1.3 to 4.8)	2.5% (-2.2 to 7.3)	5.0% (0.7 to 9.8)	17.5% (13.7 to 21.4)	31.1% (10.4 to 50.7)
Bolivia (Plurinational State of)	5.2% (-0.8 to 12.8)	15.2% (7.9 to 24.1)	0.1% (0.0 to 0.2)	-0.8% (-2.5 to 0.5)	6.4% (0.1 to 18.3)	0.2% (0.1 to 0.3)	1.4% (-0.1 to 3.2)	1.4% (0.6 to 2.4)	0.0% (0.0 to 0.0)	4.0% (1.5 to 6.5)	3.5% (-3.5 to 10.5)	6.9% (1.1 to 13.4)	17.9% (13.9 to 22.1)	28.4% (9.5 to 47.2)
Bosnia and Herzegovina	6.7% (-0.8 to 16.2)	18.2% (10.2 to 23.5)	0.8% (0.2 to 1.4)	-0.8% (-2.2 to 0.4)	16.2% (4.2 to 30.5)	0.3% (0.1 to 0.5)	0.0% (0.0 to 0.1)	1.4% (0.6 to 2.3)	0.0% (0.0 to 0.0)	0.5% (0.1 to 0.9)	3.1% (-2.9 to 9.0)	8.4% (1.3 to 16.5)	22.1% (17.4 to 27.4)	32.7% (11.4 to 52.5)
Botswana	3.0% (-0.4 to 7.9)	13.8% (7.8 to 19.8)	0.1% (0.0 to 0.2)	-0.9% (-2.8 to 0.5)	2.3% (0.0 to 10.2)	0.0% (0.0 to 0.1)	0.8% (0.0 to 1.8)	5.7% (2.4 to 9.2)	0.0% (0.0 to 0.0)	4.3% (1.6 to 7.0)	2.9% (-2.8 to 8.5)	7.4% (1.1 to 14.2)	16.0% (12.5 to 20.0)	20.8% (6.4 to 36.3)
Brazil	5.4% (-0.8 to 13.2)	8.1% (4.5 to 12.4)	0.5% (0.1 to 0.8)	2.3% (-1.4 to 5.8)	7.0% (0.3 to 19.9)	0.4% (0.2 to 0.7)	1.3% (0.0 to 2.8)	1.1% (0.5 to 1.8)	0.0% (0.0 to 0.0)	2.7% (1.2 to 4.3)	3.0% (-2.8 to 8.6)	8.6% (1.3 to 16.5)	19.6% (15.4 to 24.1)	33.1% (11.5 to 52.7)
Brunei Darussalam	0.3% (0.0 to 1.0)	3.9% (0.8 to 7.8)	0.6% (0.1 to 1.1)	-0.8% (-2.3 to 0.4)	11.3% (1.5 to 25.0)	0.1% (0.1 to 0.2)	2.3% (-0.1 to 5.0)	2.3% (1.0 to 3.8)	0.0% (0.0 to 0.0)	2.4% (0.8 to 4.1)	3.0% (-2.9 to 8.7)	4.6% (0.7 to 8.5)	24.5% (19.5 to 30.1)	31.1% (10.5 to 50.8)

Bulgaria	10.8% (-1.4 to 25.7)	12.7% (9.5 to 16.2)	1.2% (0.3 to 2.3)	1.3% (-0.8 to 4.1)	16.3% (4.5 to 30.5)	0.5% (0.2 to 0.8)	1.7% (-0.1 to 3.8)	1.8% (0.8 to 2.9)	0.0% (0.0 to 0.0)	0.2% (0.0 to 0.5)	4.9% (-4.9 to 14.2)	9.2% (1.4 to 17.8)	20.5% (16.2 to 25.1)	32.5% (11.3 to 52.2)
Burkina Faso	7.6% (-1.2 to 18.3)	8.7% (4.9 to 14.0)	0.3% (0.1 to 0.5)	-0.8% (-2.3 to 0.4)	3.8% (0.0 to 13.6)	0.0% (0.0 to 0.0)	0.1% (0.0 to 0.2)	10.1% (4.9 to 15.7)	0.0% (0.0 to 0.1)	7.2% (3.6 to 10.9)	4.3% (-4.3 to 12.3)	2.0% (0.3 to 3.8)	13.6% (10.5 to 16.9)	16.8% (5.1 to 30.8)
Burundi	4.6% (-0.8 to 11.9)	3.5% (1.9 to 5.8)	0.1% (0.0 to 0.3)	-0.1% (-0.3 to 0.1)	7.6% (0.3 to 20.1)	0.0% (0.0 to 0.1)	0.1% (0.0 to 0.3)	1.1% (0.5 to 1.8)	0.0% (0.0 to 0.1)	6.6% (2.9 to 10.3)	5.0% (-5.0 to 14.5)	1.7% (0.2 to 3.3)	11.5% (8.7 to 14.3)	21.1% (6.6 to 37.0)
C√¥te d'Ivoire	9.1% (-1.6 to 22.1)	13.1% (6.4 to 21.0)	0.4% (0.1 to 0.7)	-0.8% (-2.2 to 0.4)	4.2% (0.0 to 14.9)	0.1% (0.0 to 0.1)	0.0% (0.0 to 0.0)	1.8% (0.9 to 2.8)	0.0% (0.0 to 0.1)	4.2% (2.2 to 6.3)	4.6% (-4.7 to 12.8)	5.6% (0.8 to 10.6)	15.3% (11.8 to 19.0)	24.7% (8.1 to 41.5)
Cabo Verde	7.5% (-1.4 to 17.7)	23.2% (13.9 to 32.1)	0.4% (0.1 to 0.8)	-0.7% (-2.0 to 0.4)	3.8% (0.0 to 13.8)	0.0% (0.0 to 0.1)	0.6% (0.0 to 1.4)	2.5% (1.2 to 4.0)	0.0% (0.0 to 0.0)	1.3% (0.4 to 2.2)	3.0% (-2.9 to 8.1)	5.7% (0.9 to 10.9)	19.2% (15.3 to 23.6)	25.7% (8.8 to 42.8)
Cambodia	4.7% (-0.8 to 11.6)	7.0% (3.4 to 12.2)	0.1% (0.0 to 0.1)	-0.6% (-1.7 to 0.3)	10.8% (1.5 to 24.0)	0.0% (0.0 to 0.1)	4.2% (-0.2 to 9.2)	4.2% (1.8 to 7.0)	0.0% (0.0 to 0.1)	5.7% (2.1 to 9.3)	1.1% (-1.1 to 3.4)	1.6% (0.2 to 3.1)	14.2% (10.8 to 17.9)	26.4% (8.6 to 44.8)
Cameroon	8.8% (-1.4 to 21.0)	11.1% (5.7 to 18.2)	0.6% (0.1 to 1.0)	-0.6% (-1.8 to 0.3)	3.8% (0.0 to 13.6)	0.1% (0.0 to 0.1)	0.0% (0.0 to 0.1)	1.4% (0.7 to 2.2)	0.0% (0.0 to 0.1)	1.7% (0.7 to 2.8)	3.0% (-2.9 to 8.5)	8.4% (1.3 to 16.5)	17.3% (13.5 to 21.4)	16.5% (4.9 to 30.0)
Canada	8.2% (-1.2 to 20.7)	2.3% (0.8 to 4.1)	0.9% (0.2 to 1.6)	0.9% (-0.5 to 3.2)	4.9% (0.1 to 15.1)	0.3% (0.2 to 0.5)	0.8% (0.0 to 1.8)	1.5% (0.7 to 2.4)	0.0% (0.0 to 0.0)	1.1% (0.5 to 1.9)	3.6% (-3.6 to 10.3)	9.1% (1.4 to 17.7)	15.6% (12.4 to 19.2)	31.0% (10.9 to 50.1)
Central African Republic	3.0% (-0.4 to 8.4)	4.4% (2.6 to 6.8)	0.3% (0.1 to 0.6)	-0.9% (-2.7 to 0.5)	3.3% (0.0 to 12.8)	0.1% (0.0 to 0.1)	0.7% (0.0 to 1.6)	2.7% (1.2 to 4.5)	0.0% (0.0 to 0.1)	9.2% (4.3 to 14.0)	3.8% (-3.6 to 11.1)	3.0% (0.4 to 5.9)	18.2% (14.1 to 22.4)	22.8% (7.2 to 39.7)
Chad	4.6% (-0.7 to 13.2)	9.5% (5.3 to 14.8)	0.4% (0.1 to 0.7)	-0.8% (-2.4 to 0.4)	3.8% (0.0 to 14.2)	0.0% (0.0 to 0.0)	0.3% (0.0 to 0.7)	8.4% (4.3 to 12.8)	0.0% (0.0 to 0.1)	9.7% (4.9 to 14.4)	7.3% (-7.9 to 20.1)	3.0% (0.4 to 5.8)	16.1% (12.5 to 20.1)	23.5% (7.7 to 40.1)
Chile	9.7% (-1.5 to 23.6)	16.5% (10.4 to 23.5)	1.7% (0.4 to 3.0)	0.8% (-0.4 to 3.1)	7.1% (0.2 to 19.4)	0.5% (0.3 to 0.8)	1.3% (-0.1 to 3.0)	1.5% (0.6 to 2.4)	0.0% (0.0 to 0.0)	0.8% (0.2 to 1.5)	4.7% (-4.8 to 13.8)	9.9% (1.5 to 19.0)	19.7% (15.4 to 24.1)	30.1% (10.3 to 49.4)
China	6.3% (-1.0 to 15.2)	25.0% (17.1 to 30.5)	0.3% (0.1 to 0.5)	0.8% (-0.4 to 2.9)	16.1% (5.4 to 29.9)	0.1% (0.1 to 0.2)	0.7% (0.0 to 1.6)	1.5% (0.7 to 2.5)	0.0% (0.0 to 0.0)	0.1% (0.0 to 0.2)	4.2% (-4.2 to 11.9)	4.8% (0.7 to 9.2)	16.6% (13.0 to 20.4)	28.4% (9.6 to 46.8)
Colombia	2.9% (-0.4 to 7.6)	10.1% (6.5 to 14.3)	0.2% (0.1 to 0.4)	-1.1% (-3.1 to 0.6)	12.3% (2.5 to 25.5)	0.3% (0.1 to 0.4)	1.6% (-0.1 to 3.4)	1.3% (0.6 to 2.2)	0.0% (0.0 to 0.0)	2.7% (1.1 to 4.3)	3.4% (-3.3 to 9.7)	8.5% (1.3 to 16.6)	17.5% (13.8 to 21.6)	31.4% (11.0 to 50.6)
Comoros	0.5% (-0.1 to 1.5)	2.6% (1.3 to 4.3)	0.1% (0.0 to 0.2)	-0.3% (-0.9 to 0.2)	7.6% (0.3 to 20.2)	0.0% (0.0 to 0.1)	0.9% (0.0 to 1.9)	1.4% (0.6 to 2.3)	0.0% (0.0 to 0.1)	8.8% (4.0 to 13.5)	3.6% (-3.6 to 10.7)	4.6% (0.6 to 8.8)	11.8% (9.1 to 14.8)	26.4% (8.6 to 44.1)
Congo	7.1% (-1.1 to 17.8)	13.6% (5.8 to 22.4)	0.2% (0.0 to 0.3)	-0.7% (-2.0 to 0.4)	3.3% (0.0 to 12.8)	0.0% (0.0 to 0.1)	1.9% (-0.1 to 4.0)	2.0% (0.9 to 3.2)	0.0% (0.0 to 0.1)	8.1% (3.4 to 12.2)	3.1% (-3.1 to 9.1)	5.4% (0.8 to 10.4)	16.3% (12.7 to 20.2)	24.3% (7.9 to 41.4)
Cook Islands	7.0% (-1.1 to 18.2)	2.6% (0.0 to 6.4)	0.4% (0.1 to 0.7)	-0.8% (-2.8 to 0.8)	9.3% (0.9 to 21.6)	0.7% (0.3 to 1.1)	0.0% (0.0 to 0.0)	1.6% (0.8 to 2.5)	0.0% (0.0 to 0.0)	3.6% (1.7 to 5.6)	4.8% (-5.0 to 13.3)	13.3% (2.3 to 23.6)	31.6% (26.1 to 37.3)	30.6% (11.0 to 49.1)
Costa Rica	4.2% (-0.6 to 11.3)	9.1% (5.9 to 12.7)	0.3% (0.1 to 0.5)	-1.0% (-2.9 to 0.5)	7.9% (0.5 to 20.3)	0.3% (0.2 to 0.6)	2.2% (-0.1 to 4.7)	1.1% (0.5 to 1.8)	0.0% (0.0 to 0.0)	3.2% (0.9 to 5.3)	4.1% (-4.2 to 12.0)	8.4% (1.3 to 16.4)	20.1% (15.8 to 24.7)	30.9% (10.5 to 50.6)
Croatia	9.4% (-1.1 to 22.7)	11.4% (8.4 to 14.8)	0.3% (0.1 to 0.6)	-0.8% (-2.4 to 0.5)	16.2% (4.9 to 30.3)	0.6% (0.3 to 0.9)	1.7% (-0.1 to 3.7)	1.1% (0.3 to 1.9)	0.0% (0.0 to 0.0)	1.0% (0.2 to 1.9)	3.6% (-3.6 to 10.8)	8.8% (1.3 to 17.4)	21.9% (17.3 to 26.6)	30.7% (10.3 to 50.7)
Cuba	5.0% (-0.7 to 13.1)	14.6% (6.8 to 24.6)	0.3% (0.1 to 0.5)	-1.1% (-3.0 to 0.6)	4.4% (0.0 to 14.5)	0.4% (0.2 to 0.7)	0.1% (0.0 to 0.3)	1.1% (0.5 to 1.8)	0.0% (0.0 to 0.0)	0.9% (0.3 to 1.6)	3.6% (-3.6 to 10.6)	7.6% (1.1 to 14.4)	21.0% (16.6 to 25.5)	28.8% (9.7 to 47.1)
Cyprus	7.4% (-0.9 to 17.7)	10.6% (7.6 to 13.9)	0.7% (0.2 to 1.3)	-0.4% (-1.5 to 0.4)	3.6% (0.0 to 12.9)	0.2% (0.1 to 0.3)	1.5% (-0.3 to 4.0)	0.8% (-0.1 to 1.6)	0.0% (0.0 to 0.0)	1.0% (-0.4 to 2.1)	3.4% (-3.7 to 12.0)	5.5% (0.8 to 10.8)	18.3% (14.4 to 22.5)	29.6% (9.4 to 49.9)
Czechia	12.2% (-1.5 to 28.2)	9.9% (7.3 to 12.9)	1.1% (0.2 to 1.9)	0.1% (-0.8 to 1.6)	16.0% (4.4 to 30.4)	0.5% (0.2 to 0.8)	1.6% (-0.1 to 3.3)	1.7% (0.7 to 2.8)	0.0% (0.0 to 0.0)	1.5% (0.6 to 2.5)	2.6% (-2.5 to 7.7)	9.6% (1.5 to 18.8)	23.4% (18.7 to 28.5)	33.9% (11.8 to 54.3)

Democratic People's Republic of Korea	4.5% (-0.7 to 11.1)	5.8% (3.6 to 8.5)	0.2% (0.1 to 0.4)	-0.7% (-1.8 to 0.3)	15.3% (4.3 to 30.0)	0.0% (0.0 to 0.1)	2.2% (-0.1 to 4.4)	2.2% (1.2 to 3.4)	0.0% (0.0 to 0.0)	1.4% (0.7 to 2.1)	3.7% (-3.6 to 10.1)	3.2% (0.4 to 6.6)	16.2% (12.7 to 19.8)	33.8% (12.4 to 53.3)
Democratic Republic of the Congo	3.4% (-0.4 to 9.4)	3.9% (2.5 to 5.8)	0.4% (0.1 to 0.7)	-0.1% (-0.4 to 0.1)	1.5% (0.0 to 8.2)	0.1% (0.0 to 0.1)	1.2% (-0.1 to 2.9)	2.8% (1.2 to 4.5)	0.0% (0.0 to 0.1)	8.7% (3.8 to 13.4)	2.9% (-2.8 to 8.6)	4.3% (0.6 to 8.3)	19.1% (15.1 to 23.6)	22.7% (7.2 to 39.3)
Denmark	12.0% (-1.6 to 27.9)	5.6% (3.3 to 8.2)	1.9% (0.5 to 3.5)	1.3% (-0.7 to 3.6)	4.3% (0.0 to 14.6)	0.4% (0.2 to 0.7)	0.9% (-0.1 to 2.1)	1.0% (0.2 to 1.8)	0.0% (0.0 to 0.0)	0.8% (0.1 to 1.6)	4.1% (-4.1 to 12.4)	6.0% (0.9 to 12.0)	14.1% (11.0 to 17.5)	32.7% (11.1 to 53.4)
Djibouti	0.2% (0.0 to 0.8)	20.0% (9.8 to 33.9)	0.2% (0.0 to 0.3)	-0.9% (-2.5 to 0.5)	7.5% (0.3 to 20.3)	0.0% (0.0 to 0.1)	2.3% (-0.1 to 5.0)	7.1% (3.3 to 11.3)	0.0% (0.0 to 0.0)	3.2% (1.3 to 5.1)	5.2% (-5.4 to 14.8)	1.8% (0.2 to 3.5)	12.6% (9.8 to 15.9)	22.0% (7.0 to 38.2)
Dominica	4.5% (-0.7 to 11.7)	14.2% (5.6 to 25.8)	0.3% (0.1 to 0.5)	-0.8% (-2.4 to 0.4)	4.2% (0.0 to 14.4)	0.2% (0.1 to 0.4)	0.1% (0.0 to 0.3)	0.2% (0.1 to 0.4)	0.0% (0.0 to 0.0)	1.5% (0.5 to 2.7)	6.0% (-6.4 to 17.5)	10.4% (1.7 to 19.8)	23.5% (18.8 to 28.8)	25.0% (8.1 to 42.3)
Dominican Republic	4.7% (-0.7 to 12.0)	13.8% (4.9 to 25.1)	0.3% (0.1 to 0.5)	-1.0% (-2.9 to 0.5)	4.3% (0.0 to 14.2)	0.2% (0.1 to 0.4)	1.9% (-0.1 to 3.9)	0.6% (0.3 to 0.9)	0.0% (0.0 to 0.0)	2.6% (1.3 to 4.0)	3.9% (-3.8 to 10.6)	7.6% (1.2 to 14.3)	15.8% (12.4 to 19.6)	30.8% (11.0 to 49.6)
Ecuador	2.9% (-0.4 to 7.6)	12.6% (7.8 to 19.2)	0.3% (0.1 to 0.5)	-0.8% (-2.5 to 0.5)	6.3% (0.2 to 17.8)	0.5% (0.2 to 0.8)	3.2% (-0.2 to 6.7)	0.8% (0.3 to 1.3)	0.0% (0.0 to 0.0)	4.8% (1.1 to 8.2)	3.9% (-3.9 to 11.5)	9.5% (1.5 to 17.7)	21.1% (17.0 to 25.7)	29.3% (10.0 to 48.0)
Egypt	0.2% (0.0 to 0.5)	37.9% (28.8 to 46.6)	0.2% (0.1 to 0.4)	-0.9% (-2.4 to 0.5)	1.5% (0.0 to 7.8)	0.2% (0.1 to 0.3)	0.0% (0.0 to 0.1)	0.9% (0.4 to 1.4)	0.0% (0.0 to 0.0)	0.0% (0.0 to 0.1)	4.1% (-4.2 to 11.7)	14.1% (2.3 to 26.0)	20.1% (15.8 to 24.6)	30.0% (10.6 to 48.6)
El Salvador	2.9% (-0.4 to 8.0)	12.3% (6.6 to 19.1)	0.2% (0.1 to 0.4)	-0.5% (-1.4 to 0.3)	7.9% (0.5 to 20.2)	0.2% (0.1 to 0.4)	0.3% (0.0 to 0.6)	1.9% (0.9 to 2.9)	0.0% (0.0 to 0.0)	2.7% (1.1 to 4.3)	3.3% (-3.2 to 9.3)	10.4% (1.6 to 19.9)	18.4% (14.3 to 22.8)	32.5% (11.6 to 51.9)
Equatorial Guinea	6.6% (-1.0 to 16.7)	22.2% (12.3 to 33.4)	0.6% (0.2 to 1.0)	-0.8% (-2.4 to 0.4)	3.4% (0.0 to 12.6)	0.4% (0.2 to 0.6)	0.1% (0.0 to 0.4)	1.2% (0.6 to 2.0)	0.0% (0.0 to 0.1)	5.1% (2.2 to 8.0)	2.5% (-2.3 to 7.1)	6.7% (0.9 to 13.1)	23.1% (18.4 to 27.9)	24.6% (8.0 to 42.2)
Eritrea	1.2% (-0.1 to 3.4)	7.0% (3.9 to 11.7)	0.1% (0.0 to 0.2)	-0.5% (-1.3 to 0.2)	7.5% (0.3 to 20.3)	0.0% (0.0 to 0.1)	0.4% (0.0 to 0.9)	2.8% (1.3 to 4.5)	0.0% (0.0 to 0.1)	7.1% (3.2 to 11.0)	3.2% (-3.1 to 9.3)	1.7% (0.2 to 3.3)	10.8% (8.2 to 13.6)	21.9% (6.9 to 38.0)
Estonia	9.6% (-0.9 to 25.4)	2.9% (1.0 to 5.3)	3.8% (0.9 to 7.1)	-0.9% (-2.6 to 0.7)	2.1% (0.0 to 9.3)	0.3% (0.1 to 0.4)	0.4% (0.0 to 0.9)	2.0% (1.1 to 3.2)	0.0% (0.0 to 0.0)	1.1% (0.4 to 1.8)	4.6% (-4.8 to 13.1)	10.2% (1.5 to 19.3)	15.4% (12.0 to 19.0)	37.5% (13.9 to 57.9)
Eswatini	3.9% (-0.6 to 10.4)	13.2% (5.6 to 20.8)	0.1% (0.0 to 0.3)	-0.9% (-2.7 to 0.5)	2.3% (0.0 to 10.3)	0.1% (0.0 to 0.1)	0.7% (0.0 to 1.7)	2.2% (0.8 to 3.6)	0.0% (0.0 to 0.1)	6.6% (2.5 to 10.4)	1.7% (-1.6 to 5.3)	11.7% (1.8 to 22.0)	18.4% (14.3 to 22.6)	20.6% (6.4 to 36.6)
Ethiopia	4.1% (-0.7 to 10.8)	5.8% (3.6 to 8.2)	0.2% (0.0 to 0.4)	-0.4% (-1.2 to 0.2)	7.4% (0.3 to 19.8)	0.0% (0.0 to 0.1)	0.1% (0.0 to 0.3)	8.6% (3.9 to 13.7)	0.0% (0.0 to 0.1)	6.4% (2.6 to 10.2)	4.6% (-4.6 to 13.5)	2.1% (0.3 to 4.1)	11.8% (9.1 to 14.8)	19.1% (5.9 to 33.9)
Fiji	2.0% (-0.3 to 5.3)	8.5% (2.8 to 18.8)	0.3% (0.1 to 0.6)	-1.1% (-3.1 to 0.6)	9.5% (1.0 to 22.3)	0.4% (0.2 to 0.6)	0.3% (0.0 to 0.7)	4.6% (2.3 to 7.2)	0.0% (0.0 to 0.0)	3.6% (1.6 to 5.7)	5.0% (-5.3 to 13.6)	11.7% (1.9 to 21.2)	30.3% (24.7 to 35.8)	31.2% (11.0 to 49.6)
Finland	9.3% (-1.4 to 22.4)	1.5% (0.2 to 3.3)	1.4% (0.3 to 2.6)	0.7% (-0.4 to 2.5)	4.2% (0.0 to 13.8)	0.4% (0.2 to 0.6)	0.9% (-0.1 to 2.3)	1.6% (0.6 to 2.7)	0.0% (0.0 to 0.0)	1.4% (0.2 to 2.5)	3.7% (-3.7 to 11.1)	7.5% (1.1 to 14.7)	19.8% (15.7 to 24.1)	31.9% (10.9 to 52.1)
France	12.1% (-1.8 to 28.7)	5.8% (3.9 to 8.2)	2.3% (0.5 to 4.1)	2.6% (-1.7 to 7.0)	3.7% (0.0 to 13.3)	0.4% (0.2 to 0.7)	1.6% (-0.1 to 3.6)	1.6% (0.6 to 2.7)	0.0% (0.0 to 0.0)	1.5% (0.4 to 2.6)	5.5% (-5.9 to 16.2)	6.9% (1.0 to 13.1)	14.1% (11.0 to 17.6)	33.8% (11.6 to 54.2)
Gabon	7.4% (-1.1 to 18.5)	19.7% (11.2 to 29.8)	0.5% (0.1 to 0.8)	-0.2% (-1.3 to 1.1)	3.4% (0.0 to 13.2)	0.2% (0.1 to 0.4)	0.4% (0.0 to 1.0)	0.8% (0.4 to 1.4)	0.0% (0.0 to 0.1)	4.7% (1.8 to 7.5)	2.2% (-2.0 to 6.2)	8.0% (1.2 to 15.1)	21.9% (17.6 to 26.7)	24.1% (7.8 to 40.9)
Gambia	5.0% (-0.8 to 12.7)	9.8% (5.5 to 15.2)	0.4% (0.1 to 0.7)	-0.3% (-0.9 to 0.2)	3.8% (0.0 to 14.0)	0.0% (0.0 to 0.1)	0.8% (0.0 to 1.7)	10.9% (5.6 to 16.5)	0.0% (0.0 to 0.1)	7.0% (3.6 to 10.4)	3.4% (-3.4 to 9.8)	5.2% (0.7 to 9.9)	15.4% (12.0 to 19.2)	25.8% (8.5 to 43.1)
Georgia	5.9% (-0.7 to 16.5)	12.4% (6.8 to 18.3)	0.4% (0.1 to 0.7)	-0.9% (-2.5 to 0.5)	7.0% (0.3 to 19.0)	0.1% (0.0 to 0.1)	0.9% (0.0 to 2.1)	1.9% (0.9 to 3.1)	0.0% (0.0 to 0.0)	1.1% (0.4 to 1.9)	6.8% (-7.6 to 19.4)	7.9% (1.2 to 15.2)	14.8% (11.4 to 18.5)	26.8% (8.9 to 45.0)
Germany	13.1% (-2.2 to 30.3)	6.6% (4.5 to 9.0)	1.6% (0.4 to 2.8)	1.8% (-1.1 to 4.8)	4.5% (0.1 to 14.6)	0.3% (0.2 to 0.5)	1.3% (-0.1 to 2.8)	1.6% (0.7 to 2.5)	0.0% (0.0 to 0.0)	1.3% (0.5 to 2.2)	3.6% (-3.6 to 10.4)	8.1% (1.2 to 15.6)	17.7% (13.7 to 22.1)	34.9% (12.3 to 55.0)

Ghana	6.7% (-1.1 to 16.8)	17.3% (8.3 to 27.6)	0.8% (0.2 to 1.5)	-0.5% (-1.4 to 0.3)	5.7% (0.1 to 17.2)	0.1% (0.1 to 0.2)	0.0% (0.0 to 0.0)	1.0% (0.5 to 1.6)	0.0% (0.0 to 0.1)	3.6% (1.9 to 5.5)	4.7% (-4.9 to 12.9)	5.7% (0.8 to 11.0)	18.7% (14.8 to 22.7)	30.6% (10.9 to 49.2)
Greece	8.2% (-1.2 to 19.8)	10.5% (7.7 to 13.7)	1.2% (0.3 to 2.1)	1.4% (-0.8 to 3.8)	3.8% (0.0 to 13.5)	0.4% (0.2 to 0.7)	0.6% (-0.1 to 1.5)	0.5% (0.1 to 1.0)	0.0% (0.0 to 0.0)	0.1% (-0.1 to 0.4)	4.8% (-5.2 to 15.2)	7.1% (1.1 to 14.2)	17.6% (13.9 to 21.8)	28.9% (9.4 to 48.3)
Greenland	7.5% (-1.0 to 19.5)	2.6% (0.1 to 6.7)	0.9% (0.2 to 1.8)	1.6% (-1.0 to 4.5)	4.5% (0.0 to 15.4)	0.3% (0.1 to 0.4)	0.8% (0.0 to 1.8)	1.2% (0.4 to 2.0)	0.0% (0.0 to 0.0)	0.7% (0.1 to 1.3)	3.2% (-3.1 to 9.5)	8.1% (1.2 to 15.8)	13.0% (10.2 to 16.3)	29.1% (9.8 to 48.0)
Grenada	4.5% (-0.6 to 11.6)	17.4% (6.8 to 32.5)	0.2% (0.0 to 0.3)	-0.5% (-1.4 to 0.3)	4.2% (0.0 to 14.3)	0.1% (0.0 to 0.1)	1.1% (-0.1 to 2.5)	1.0% (0.4 to 1.7)	0.0% (0.0 to 0.0)	4.4% (1.5 to 7.2)	5.4% (-5.6 to 16.0)	6.9% (1.0 to 13.1)	20.9% (16.5 to 25.8)	27.5% (9.1 to 46.2)
Guam	3.7% (-0.6 to 11.0)	5.7% (2.7 to 9.1)	0.3% (0.1 to 0.6)	-0.7% (-2.9 to 1.5)	9.0% (1.0 to 21.4)	0.8% (0.4 to 1.3)	0.0% (0.0 to 0.0)	1.8% (1.1 to 2.7)	0.0% (0.0 to 0.0)	3.6% (2.2 to 5.1)	6.2% (-6.8 to 15.7)	12.3% (2.1 to 23.1)	23.7% (19.3 to 28.7)	34.0% (13.0 to 51.8)
Guatemala	2.3% (-0.3 to 6.2)	11.8% (4.9 to 18.2)	0.2% (0.0 to 0.3)	-0.4% (-1.2 to 0.2)	7.6% (0.4 to 20.1)	0.1% (0.1 to 0.2)	0.3% (0.0 to 0.6)	1.6% (0.7 to 2.7)	0.0% (0.0 to 0.0)	2.5% (0.8 to 4.2)	2.9% (-2.9 to 8.5)	8.3% (1.3 to 16.1)	18.8% (14.9 to 23.4)	26.6% (8.9 to 44.5)
Guinea	2.7% (-0.4 to 7.3)	8.3% (4.8 to 13.0)	0.4% (0.1 to 0.7)	-0.5% (-1.3 to 0.2)	3.8% (0.0 to 13.7)	0.0% (0.0 to 0.1)	0.7% (0.0 to 1.4)	1.6% (0.8 to 2.5)	0.0% (0.0 to 0.1)	4.5% (2.3 to 6.8)	3.0% (-2.9 to 8.4)	3.7% (0.5 to 7.1)	14.3% (11.1 to 17.5)	25.0% (8.2 to 42.2)
Guinea-Bissau	5.0% (-0.8 to 12.5)	8.5% (4.8 to 13.2)	0.3% (0.1 to 0.5)	-0.9% (-2.4 to 0.5)	3.8% (0.0 to 13.9)	0.0% (0.0 to 0.0)	1.6% (-0.1 to 3.5)	2.6% (1.4 to 3.9)	0.0% (0.0 to 0.1)	9.6% (5.2 to 13.9)	2.8% (-2.7 to 7.8)	4.3% (0.6 to 8.0)	15.2% (11.7 to 18.9)	24.8% (8.3 to 41.9)
Guyana	4.7% (-0.7 to 12.1)	17.8% (7.4 to 32.7)	0.4% (0.1 to 0.7)	-0.3% (-0.8 to 0.1)	4.3% (0.0 to 14.7)	0.3% (0.2 to 0.5)	1.6% (-0.1 to 3.5)	2.0% (1.0 to 3.3)	0.0% (0.0 to 0.0)	2.7% (1.1 to 4.3)	3.4% (-3.3 to 9.4)	7.3% (1.1 to 14.3)	27.5% (22.0 to 33.1)	28.6% (9.6 to 47.1)
Haiti	4.3% (-0.5 to 11.2)	4.1% (1.8 to 7.9)	0.1% (0.0 to 0.2)	-0.4% (-1.3 to 0.2)	4.2% (0.0 to 14.2)	0.0% (0.0 to 0.1)	1.4% (-0.1 to 3.0)	1.7% (0.8 to 2.8)	0.0% (0.0 to 0.0)	7.8% (3.8 to 12.1)	4.2% (-4.2 to 12.2)	3.1% (0.4 to 6.0)	18.3% (14.3 to 22.4)	26.5% (8.7 to 44.1)
Honduras	2.6% (-0.4 to 6.9)	8.3% (3.8 to 13.5)	0.2% (0.0 to 0.3)	-0.5% (-1.5 to 0.3)	8.1% (0.4 to 21.3)	0.1% (0.1 to 0.2)	0.5% (0.0 to 1.1)	1.5% (0.6 to 2.5)	0.0% (0.0 to 0.0)	2.8% (0.9 to 4.6)	2.6% (-2.4 to 7.6)	7.4% (1.1 to 14.6)	18.5% (14.3 to 23.1)	28.8% (9.3 to 47.7)
Hungary	10.1% (-1.3 to 24.3)	10.3% (7.6 to 13.4)	0.9% (0.2 to 1.5)	-0.6% (-2.2 to 0.9)	18.2% (6.4 to 32.3)	0.3% (0.2 to 0.6)	1.7% (-0.1 to 3.6)	1.9% (0.9 to 3.0)	0.0% (0.0 to 0.0)	0.7% (0.2 to 1.2)	5.3% (-5.5 to 14.9)	11.1% (1.7 to 20.6)	19.7% (15.4 to 24.3)	36.6% (13.3 to 56.6)
Iceland	9.6% (-1.2 to 23.4)	1.6% (0.3 to 3.5)	2.0% (0.5 to 3.6)	2.3% (-1.4 to 5.7)	4.7% (0.1 to 14.6)	0.5% (0.2 to 0.8)	2.0% (-0.1 to 4.6)	1.4% (0.4 to 2.4)	0.0% (0.0 to 0.0)	1.6% (0.1 to 2.9)	3.9% (-4.1 to 12.2)	7.6% (1.1 to 15.0)	17.7% (14.0 to 21.6)	33.0% (11.4 to 53.6)
India	3.1% (-0.4 to 8.5)	19.0% (11.4 to 25.1)	0.1% (0.0 to 0.2)	-0.1% (-0.4 to 0.1)	5.6% (0.2 to 16.8)	0.2% (0.1 to 0.3)	1.2% (0.0 to 2.6)	9.9% (5.3 to 15.2)	0.0% (0.0 to 0.1)	2.7% (1.4 to 4.1)	3.5% (-3.3 to 9.7)	2.8% (0.4 to 5.2)	19.6% (15.7 to 24.4)	25.8% (8.6 to 43.1)
Indonesia	0.3% (0.0 to 0.9)	12.5% (7.5 to 17.2)	0.1% (0.0 to 0.1)	-0.3% (-0.8 to 0.1)	11.6% (1.9 to 24.9)	0.0% (0.0 to 0.1)	2.5% (-0.1 to 5.4)	2.1% (0.9 to 3.3)	0.0% (0.0 to 0.1)	3.2% (1.3 to 5.0)	1.9% (-1.7 to 5.3)	2.6% (0.3 to 5.0)	15.8% (12.3 to 19.6)	23.4% (7.7 to 39.9)
Iran (Islamic Republic of)	0.5% (-0.1 to 1.5)	23.5% (18.7 to 28.2)	0.2% (0.0 to 0.3)	-0.8% (-2.1 to 0.4)	1.5% (0.0 to 7.6)	0.2% (0.1 to 0.3)	0.5% (0.0 to 1.0)	0.8% (0.4 to 1.2)	0.0% (0.0 to 0.0)	0.3% (0.1 to 0.7)	7.6% (-8.8 to 20.8)	10.4% (1.6 to 19.4)	19.3% (15.2 to 23.7)	32.3% (11.5 to 51.3)
Iraq	0.2% (0.0 to 0.5)	26.2% (19.1 to 34.0)	0.2% (0.0 to 0.3)	-0.3% (-0.9 to 0.2)	1.6% (0.0 to 8.1)	0.1% (0.0 to 0.1)	1.2% (-0.1 to 2.5)	1.8% (0.9 to 2.9)	0.0% (0.0 to 0.0)	0.5% (0.1 to 1.0)	6.6% (-7.2 to 18.1)	11.1% (1.7 to 21.4)	27.6% (22.0 to 33.2)	30.9% (10.7 to 49.5)
Ireland	9.5% (-1.3 to 23.0)	3.9% (2.0 to 6.2)	1.6% (0.4 to 2.9)	2.8% (-1.9 to 7.2)	2.8% (0.0 to 11.0)	0.7% (0.3 to 1.2)	0.6% (-0.1 to 1.5)	1.2% (0.2 to 2.2)	0.0% (0.0 to 0.0)	0.7% (0.0 to 1.5)	1.9% (-1.8 to 6.4)	7.0% (1.0 to 13.8)	17.1% (13.4 to 21.0)	28.4% (9.4 to 47.9)
Israel	3.9% (-0.5 to 10.0)	14.3% (11.0 to 18.1)	0.7% (0.2 to 1.4)	-0.7% (-2.3 to 0.5)	4.1% (0.0 to 14.2)	0.5% (0.2 to 0.8)	0.2% (0.0 to 0.6)	0.7% (0.2 to 1.2)	0.0% (0.0 to 0.0)	0.1% (0.0 to 0.2)	5.5% (-6.0 to 16.4)	7.4% (1.1 to 14.6)	20.4% (16.3 to 25.0)	30.0% (10.2 to 48.8)
Italy	10.2% (-1.4 to 24.0)	9.9% (7.5 to 12.9)	2.0% (0.5 to 3.6)	0.7% (-0.4 to 2.6)	4.9% (0.2 to 15.2)	0.4% (0.2 to 0.7)	0.9% (0.0 to 2.2)	0.7% (0.1 to 1.3)	0.0% (0.0 to 0.0)	0.6% (0.0 to 1.3)	5.1% (-5.7 to 16.2)	6.1% (0.9 to 12.1)	18.4% (14.6 to 22.7)	30.1% (9.9 to 50.1)
Jamaica	3.4% (-0.4 to 8.9)	11.9% (6.3 to 19.2)	0.1% (0.0 to 0.2)	-0.5% (-1.5 to 0.3)	4.3% (0.0 to 14.4)	0.1% (0.0 to 0.2)	0.6% (-0.1 to 1.5)	1.3% (0.5 to 2.1)	0.0% (0.0 to 0.0)	1.8% (0.4 to 3.1)	4.5% (-4.6 to 13.3)	8.1% (1.3 to 15.5)	14.9% (11.4 to 18.5)	26.3% (8.6 to 44.3)

Japan	9.2% (-1.6 to 21.9)	8.5% (4.7 to 13.3)	2.0% (0.5 to 3.4)	-0.9% (-2.6 to 0.5)	9.6% (1.0 to 22.6)	0.5% (0.2 to 0.8)	1.5% (-0.1 to 3.2)	2.8% (1.5 to 4.2)	0.0% (0.0 to 0.0)	0.5% (0.2 to 0.8)	3.6% (-3.6 to 10.1)	3.3% (0.5 to 6.3)	19.8% (15.8 to 24.1)	31.5% (11.2 to 50.3)
Jordan	0.2% (0.0 to 0.7)	22.4% (17.1 to 28.2)	0.3% (0.1 to 0.5)	-0.8% (-2.2 to 0.4)	1.5% (0.0 to 7.7)	0.2% (0.1 to 0.4)	1.3% (-0.1 to 2.7)	1.7% (0.9 to 2.7)	0.0% (0.0 to 0.0)	1.0% (0.4 to 1.7)	7.1% (-8.0 to 19.7)	13.9% (2.3 to 25.4)	27.5% (22.1 to 32.8)	33.4% (11.8 to 52.9)
Kazakhstan	5.2% (-0.6 to 14.8)	15.3% (10.3 to 21.4)	0.5% (0.1 to 1.0)	2.0% (-1.2 to 5.2)	6.6% (0.3 to 18.3)	0.2% (0.1 to 0.3)	1.0% (0.0 to 2.3)	1.9% (0.8 to 3.2)	0.0% (0.0 to 0.0)	0.2% (0.0 to 0.5)	7.0% (-7.8 to 19.9)	9.1% (1.4 to 17.6)	16.6% (13.1 to 20.5)	29.0% (9.9 to 47.7)
Kenya	4.7% (-0.8 to 11.9)	5.3% (2.8 to 8.3)	0.2% (0.1 to 0.4)	-0.6% (-1.8 to 0.3)	5.1% (0.3 to 14.4)	0.1% (0.0 to 0.1)	0.3% (0.0 to 0.6)	2.2% (1.0 to 3.8)	0.0% (0.0 to 0.1)	2.8% (0.9 to 4.7)	2.8% (-2.7 to 8.4)	4.0% (0.6 to 7.6)	12.1% (9.4 to 15.4)	20.1% (6.3 to 35.6)
Kiribati	0.8% (-0.1 to 3.1)	2.6% (1.0 to 6.0)	0.3% (0.1 to 0.5)	-0.8% (-2.4 to 0.4)	9.4% (1.2 to 21.8)	0.2% (0.1 to 0.3)	0.6% (0.0 to 1.1)	2.3% (1.2 to 3.4)	0.0% (0.0 to 0.1)	5.4% (2.9 to 7.8)	6.1% (-6.7 to 16.8)	11.0% (1.8 to 20.4)	28.2% (23.1 to 33.5)	29.2% (10.3 to 47.8)
Kuwait	0.1% (0.0 to 0.3)	32.8% (26.0 to 39.4)	0.3% (0.1 to 0.5)	-0.9% (-2.9 to 0.8)	3.6% (0.0 to 12.5)	0.5% (0.2 to 0.7)	1.0% (0.0 to 2.2)	1.7% (0.9 to 2.6)	0.0% (0.0 to 0.0)	0.3% (0.1 to 0.6)	5.7% (-6.2 to 15.9)	15.4% (2.6 to 27.1)	25.8% (20.9 to 30.9)	33.9% (12.7 to 52.8)
Kyrgyzstan	4.3% (-0.6 to 11.9)	12.2% (5.9 to 19.5)	0.7% (0.2 to 1.2)	-0.8% (-2.6 to 1.2)	6.2% (0.3 to 17.8)	0.2% (0.1 to 0.3)	1.1% (-0.1 to 2.2)	3.2% (1.8 to 4.7)	0.0% (0.0 to 0.1)	0.5% (0.2 to 0.9)	9.4% (-10.8 to 24.6)	11.0% (1.7 to 21.1)	12.4% (9.5 to 15.4)	32.2% (11.9 to 51.1)
Lao People's Democratic Republic	7.1% (-1.3 to 17.5)	10.6% (4.3 to 17.4)	0.1% (0.0 to 0.1)	-0.9% (-2.5 to 0.5)	11.8% (1.8 to 25.9)	0.0% (0.0 to 0.1)	4.0% (-0.2 to 8.4)	1.7% (0.7 to 2.7)	0.0% (0.0 to 0.1)	2.5% (1.1 to 4.1)	1.4% (-1.3 to 4.1)	2.6% (0.3 to 5.1)	16.6% (12.8 to 20.5)	28.1% (9.5 to 46.4)
Latvia	9.5% (-0.9 to 25.8)	8.4% (5.6 to 11.5)	4.2% (0.8 to 7.9)	-0.8% (-2.5 to 0.5)	4.3% (0.1 to 13.7)	0.2% (0.1 to 0.3)	1.0% (0.0 to 2.3)	2.3% (1.0 to 3.8)	0.0% (0.0 to 0.0)	0.9% (0.3 to 1.6)	4.9% (-4.9 to 14.5)	9.7% (1.5 to 19.0)	15.4% (11.9 to 19.2)	33.7% (11.6 to 54.3)
Lebanon	1.5% (-0.2 to 4.5)	14.9% (9.6 to 21.3)	0.2% (0.1 to 0.4)	-1.2% (-3.6 to 0.7)	1.5% (0.0 to 7.4)	0.2% (0.1 to 0.3)	0.3% (0.0 to 0.6)	0.9% (0.5 to 1.4)	0.0% (0.0 to 0.0)	0.1% (0.0 to 0.2)	7.7% (-9.0 to 21.2)	12.0% (1.9 to 22.6)	24.0% (19.4 to 29.2)	34.8% (12.6 to 54.2)
Lesotho	3.7% (-0.6 to 9.7)	5.8% (3.0 to 9.8)	0.1% (0.0 to 0.2)	-0.8% (-2.3 to 0.4)	2.3% (0.0 to 10.5)	0.1% (0.0 to 0.2)	0.0% (0.0 to 0.0)	6.3% (2.7 to 10.1)	0.0% (0.0 to 0.1)	6.1% (2.4 to 9.6)	1.4% (-1.3 to 4.0)	7.8% (1.2 to 14.9)	14.7% (11.3 to 18.5)	18.1% (5.4 to 32.9)
Liberia	5.1% (-0.8 to 12.4)	6.2% (4.0 to 8.9)	0.4% (0.1 to 0.7)	-0.3% (-0.9 to 0.2)	3.8% (0.0 to 13.8)	0.0% (0.0 to 0.1)	2.9% (-0.2 to 6.0)	2.9% (1.4 to 4.6)	0.0% (0.0 to 0.1)	7.4% (3.8 to 11.2)	3.6% (-3.6 to 10.2)	7.4% (1.1 to 14.0)	17.0% (13.2 to 21.0)	24.4% (8.1 to 40.6)
Libya	0.2% (0.0 to 0.8)	24.4% (17.3 to 32.0)	0.1% (0.0 to 0.2)	-1.0% (-2.7 to 0.5)	1.5% (0.0 to 7.9)	0.1% (0.1 to 0.2)	1.1% (0.0 to 2.3)	1.7% (0.9 to 2.6)	0.0% (0.0 to 0.0)	1.1% (0.6 to 1.8)	7.9% (-9.2 to 20.8)	14.3% (2.4 to 25.6)	18.7% (14.9 to 23.1)	31.1% (11.4 to 49.4)
Lithuania	9.7% (-0.9 to 25.5)	6.2% (3.9 to 9.0)	4.7% (1.0 to 8.7)	0.3% (-0.7 to 2.2)	4.4% (0.0 to 15.2)	0.3% (0.1 to 0.5)	0.4% (0.0 to 0.9)	2.0% (1.0 to 3.1)	0.0% (0.0 to 0.0)	0.9% (0.4 to 1.6)	4.8% (-4.8 to 13.5)	10.2% (1.6 to 19.6)	13.7% (10.7 to 17.1)	36.5% (13.0 to 57.0)
Luxembourg	12.0% (-1.7 to 28.2)	4.4% (2.6 to 6.7)	1.8% (0.4 to 3.4)	2.1% (-1.2 to 5.2)	4.0% (0.0 to 13.8)	0.3% (0.1 to 0.5)	1.1% (-0.1 to 2.5)	1.2% (0.3 to 2.1)	0.0% (0.0 to 0.0)	0.9% (0.0 to 1.7)	3.0% (-3.0 to 9.3)	7.0% (1.0 to 14.2)	19.9% (15.6 to 24.2)	31.1% (10.5 to 51.6)
Madagascar	2.1% (-0.3 to 5.4)	2.3% (1.4 to 3.5)	0.2% (0.0 to 0.3)	-0.6% (-1.7 to 0.3)	7.5% (0.3 to 20.2)	0.0% (0.0 to 0.1)	1.6% (-0.1 to 3.3)	2.8% (1.3 to 4.4)	0.0% (0.0 to 0.1)	8.5% (4.1 to 12.7)	2.2% (-2.1 to 6.5)	3.0% (0.4 to 5.6)	11.2% (8.7 to 14.3)	21.9% (6.9 to 38.3)
Malawi	3.1% (-0.6 to 7.8)	3.4% (2.0 to 5.4)	0.2% (0.0 to 0.3)	-0.3% (-0.9 to 0.2)	7.6% (0.3 to 20.2)	0.1% (0.0 to 0.1)	0.0% (0.0 to 0.0)	2.0% (0.9 to 3.2)	0.0% (0.0 to 0.1)	6.3% (2.8 to 9.7)	1.8% (-1.7 to 5.3)	3.1% (0.4 to 6.1)	10.8% (8.2 to 13.7)	24.6% (7.9 to 42.1)
Malaysia	1.2% (-0.2 to 3.5)	12.9% (8.5 to 18.3)	0.4% (0.1 to 0.7)	-0.7% (-1.9 to 0.3)	11.8% (1.9 to 25.3)	0.2% (0.1 to 0.4)	2.9% (-0.2 to 6.1)	1.7% (0.8 to 2.7)	0.0% (0.0 to 0.0)	2.6% (1.2 to 4.1)	3.9% (-3.9 to 11.0)	6.1% (0.9 to 11.3)	22.9% (18.4 to 28.0)	36.4% (13.4 to 56.7)
Maldives	0.9% (-0.1 to 2.6)	8.2% (4.8 to 12.5)	0.1% (0.0 to 0.1)	-0.2% (-0.6 to 0.1)	11.5% (1.7 to 24.6)	0.0% (0.0 to 0.1)	2.5% (-0.1 to 5.3)	1.8% (0.8 to 2.9)	0.0% (0.0 to 0.0)	1.6% (0.4 to 2.7)	4.6% (-4.8 to 13.5)	4.0% (0.6 to 7.4)	17.8% (14.0 to 21.9)	31.3% (10.8 to 50.7)
Mali	1.8% (-0.3 to 4.9)	10.0% (5.5 to 15.9)	0.8% (0.2 to 1.4)	-1.1% (-3.1 to 0.6)	3.6% (0.0 to 13.2)	0.1% (0.0 to 0.2)	0.1% (0.0 to 0.3)	2.9% (1.4 to 4.7)	0.0% (0.0 to 0.1)	5.7% (2.8 to 8.7)	3.6% (-3.5 to 10.5)	3.0% (0.4 to 5.7)	17.2% (13.7 to 21.3)	23.5% (7.7 to 40.1)

Malta	7.5% (-1.0 to 18.7)	8.4% (5.9 to 11.3)	1.9% (0.4 to 3.3)	-0.1% (-1.2 to 1.3)	4.9% (0.1 to 15.4)	0.5% (0.2 to 0.7)	0.7% (0.0 to 1.7)	1.2% (0.4 to 2.2)	0.0% (0.0 to 0.0)	0.3% (-0.1 to 0.8)	5.3% (-6.0 to 16.3)	6.6% (1.0 to 13.3)	18.5% (14.6 to 22.9)	32.3% (11.0 to 52.8)
Marshall Islands	1.8% (-0.2 to 5.3)	4.7% (1.7 to 10.1)	0.2% (0.0 to 0.3)	-1.0% (-3.0 to 0.5)	9.3% (1.0 to 21.8)	0.1% (0.1 to 0.2)	0.2% (0.0 to 0.5)	2.7% (1.4 to 4.2)	0.0% (0.0 to 0.1)	6.6% (3.1 to 10.1)	5.4% (-5.8 to 15.0)	11.4% (1.9 to 20.9)	35.4% (29.1 to 41.7)	30.9% (10.8 to 49.8)
Mauritania	0.0% (0.0 to 0.0)	21.8% (11.9 to 32.8)	0.6% (0.1 to 1.0)	-1.1% (-3.0 to 0.6)	3.7% (0.0 to 13.7)	0.1% (0.0 to 0.1)	0.4% (0.0 to 1.0)	8.1% (3.8 to 12.9)	0.0% (0.0 to 0.0)	6.6% (3.0 to 10.3)	5.8% (-6.1 to 16.2)	8.1% (1.2 to 15.6)	13.5% (10.5 to 16.7)	25.7% (8.7 to 43.3)
Mauritius	4.8% (-0.8 to 11.9)	5.3% (1.8 to 9.5)	0.2% (0.0 to 0.3)	-0.9% (-2.5 to 0.5)	11.6% (1.8 to 25.6)	0.3% (0.1 to 0.5)	2.1% (-0.1 to 4.3)	4.0% (2.2 to 6.1)	0.0% (0.0 to 0.0)	2.2% (1.1 to 3.3)	4.8% (-5.1 to 13.1)	6.9% (1.0 to 13.0)	23.4% (18.5 to 28.9)	33.8% (12.5 to 53.1)
Mexico	4.3% (-0.7 to 11.0)	10.1% (6.8 to 14.0)	0.4% (0.1 to 0.7)	-0.2% (-1.1 to 1.2)	5.4% (0.1 to 16.1)	0.6% (0.3 to 0.9)	0.2% (0.0 to 0.5)	1.3% (0.6 to 2.1)	0.0% (0.0 to 0.0)	2.3% (1.0 to 3.6)	2.2% (-2.1 to 6.3)	10.3% (1.6 to 19.2)	22.3% (18.0 to 27.3)	31.9% (11.3 to 51.0)
Micronesia (Federated States of)	2.0% (-0.3 to 5.7)	5.1% (1.7 to 11.8)	0.2% (0.0 to 0.3)	-1.1% (-3.0 to 0.5)	9.2% (0.9 to 21.6)	0.1% (0.1 to 0.2)	0.2% (0.0 to 0.5)	2.9% (1.5 to 4.5)	0.0% (0.0 to 0.1)	7.0% (3.7 to 10.5)	5.6% (-6.1 to 15.4)	12.0% (1.9 to 21.7)	22.2% (17.7 to 26.9)	30.7% (11.0 to 49.0)
Monaco	7.5% (-0.9 to 23.0)	5.6% (3.0 to 8.8)	3.4% (0.9 to 6.1)	2.6% (-1.7 to 7.2)	3.9% (0.0 to 13.7)	1.7% (0.8 to 2.8)	0.1% (0.0 to 0.4)	0.5% (0.1 to 0.9)	0.0% (0.0 to 0.0)	0.1% (0.0 to 0.3)	3.9% (-4.0 to 12.0)	7.8% (1.2 to 15.2)	18.1% (14.3 to 22.3)	32.5% (11.0 to 53.1)
Mongolia	6.4% (-0.9 to 17.0)	19.9% (9.7 to 27.8)	0.9% (0.2 to 1.6)	6.1% (-4.0 to 14.7)	6.7% (0.4 to 18.7)	0.2% (0.1 to 0.3)	4.6% (-0.2 to 9.1)	10.6% (6.0 to 15.4)	0.0% (0.0 to 0.0)	3.9% (2.2 to 5.7)	8.9% (-10.9 to 24.1)	7.9% (1.2 to 15.2)	11.1% (8.7 to 14.1)	34.4% (13.1 to 53.1)
Montenegro	7.9% (-0.8 to 18.9)	13.2% (8.6 to 17.3)	1.5% (0.3 to 2.6)	0.1% (-0.8 to 1.4)	15.8% (4.3 to 30.1)	0.3% (0.1 to 0.5)	0.4% (-0.1 to 0.9)	0.5% (0.1 to 1.1)	0.0% (0.0 to 0.0)	0.1% (-0.1 to 0.3)	4.1% (-4.2 to 13.1)	9.4% (1.4 to 18.2)	23.2% (18.4 to 27.9)	30.8% (10.3 to 51.0)
Morocco	0.1% (0.0 to 0.3)	15.1% (10.6 to 20.2)	0.2% (0.1 to 0.4)	-0.9% (-2.6 to 0.5)	1.5% (0.0 to 7.9)	0.1% (0.1 to 0.2)	0.0% (0.0 to 0.0)	1.0% (0.5 to 1.5)	0.0% (0.0 to 0.0)	0.6% (0.2 to 1.2)	7.9% (-8.9 to 21.6)	9.0% (1.4 to 17.0)	26.2% (20.9 to 31.5)	32.1% (11.3 to 51.3)
Mozambique	3.0% (-0.5 to 7.7)	3.3% (2.1 to 5.1)	0.1% (0.0 to 0.2)	-0.3% (-0.9 to 0.2)	7.7% (0.2 to 20.8)	0.0% (0.0 to 0.1)	0.4% (0.0 to 0.9)	5.2% (2.7 to 8.0)	0.0% (0.0 to 0.0)	7.8% (3.9 to 11.5)	4.3% (-4.2 to 12.2)	3.3% (0.4 to 6.2)	11.1% (8.5 to 14.0)	26.3% (8.8 to 44.0)
Myanmar	3.3% (-0.5 to 8.3)	13.4% (6.2 to 21.1)	0.1% (0.0 to 0.1)	-0.7% (-2.1 to 0.4)	11.9% (2.0 to 25.8)	0.1% (0.0 to 0.1)	3.6% (-0.2 to 7.6)	3.2% (1.6 to 5.0)	0.0% (0.0 to 0.1)	2.2% (0.9 to 3.5)	2.3% (-2.1 to 6.4)	2.3% (0.3 to 4.4)	18.6% (14.6 to 22.9)	27.9% (9.4 to 46.5)
Namibia	8.0% (-1.3 to 20.1)	13.1% (6.1 to 19.5)	0.2% (0.0 to 0.3)	-0.9% (-2.6 to 0.4)	2.2% (0.0 to 10.3)	0.0% (0.0 to 0.1)	1.4% (-0.1 to 3.1)	4.4% (1.6 to 7.4)	0.0% (0.0 to 0.1)	4.2% (1.1 to 7.1)	2.9% (-2.8 to 8.6)	7.1% (1.0 to 13.6)	16.6% (13.1 to 20.9)	21.9% (6.6 to 38.6)
Nauru	3.9% (-0.6 to 10.0)	2.6% (0.4 to 5.6)	0.2% (0.1 to 0.4)	-1.2% (-3.4 to 0.6)	9.3% (0.9 to 22.2)	0.2% (0.1 to 0.4)	0.1% (0.0 to 0.1)	2.3% (1.3 to 3.5)	0.0% (0.0 to 0.0)	5.6% (2.9 to 8.3)	5.8% (-6.2 to 15.6)	14.4% (2.6 to 25.2)	23.9% (19.1 to 29.2)	32.3% (11.9 to 50.6)
Nepal	2.8% (-0.4 to 8.3)	12.7% (7.6 to 18.7)	0.7% (0.1 to 1.2)	-0.7% (-1.9 to 0.4)	6.0% (0.1 to 17.8)	0.0% (0.0 to 0.1)	0.9% (-0.1 to 2.0)	2.3% (1.0 to 3.7)	0.0% (0.0 to 0.0)	2.7% (1.1 to 4.3)	2.4% (-2.2 to 6.8)	2.2% (0.3 to 4.0)	19.6% (15.5 to 24.2)	24.1% (7.8 to 41.0)
Netherlands	11.1% (-1.5 to 26.3)	7.3% (5.0 to 9.9)	1.3% (0.3 to 2.3)	1.5% (-1.0 to 4.2)	3.6% (0.0 to 13.3)	0.5% (0.2 to 0.8)	1.2% (-0.1 to 2.8)	1.2% (0.3 to 2.1)	0.0% (0.0 to 0.0)	1.4% (0.2 to 2.5)	3.6% (-3.7 to 11.1)	6.3% (0.9 to 12.2)	15.4% (12.1 to 19.0)	32.6% (10.9 to 53.3)
New Zealand	10.7% (-1.4 to 26.0)	3.4% (1.4 to 5.7)	2.2% (0.5 to 3.8)	2.7% (-1.7 to 7.0)	3.4% (0.0 to 12.4)	0.6% (0.3 to 0.9)	1.2% (-0.1 to 2.9)	1.1% (0.3 to 2.0)	0.0% (0.0 to 0.0)	1.0% (0.1 to 1.9)	3.2% (-3.2 to 9.9)	8.0% (1.2 to 15.8)	20.0% (15.7 to 24.7)	31.1% (10.4 to 50.6)
Nicaragua	3.3% (-0.5 to 9.0)	7.4% (3.2 to 12.9)	0.2% (0.0 to 0.3)	-0.4% (-1.2 to 0.2)	8.0% (0.5 to 20.5)	0.1% (0.1 to 0.2)	0.9% (0.0 to 2.0)	4.7% (2.4 to 7.5)	0.0% (0.0 to 0.0)	8.0% (3.8 to 12.4)	2.7% (-2.6 to 7.7)	9.9% (1.6 to 19.0)	17.7% (13.9 to 21.8)	30.1% (10.5 to 49.0)
Niger	0.6% (-0.1 to 1.9)	8.8% (4.8 to 13.9)	0.4% (0.1 to 0.8)	-0.9% (-2.7 to 0.5)	3.7% (0.0 to 13.5)	0.0% (0.0 to 0.1)	0.2% (0.0 to 0.5)	5.6% (2.4 to 9.1)	0.0% (0.0 to 0.1)	2.3% (0.9 to 3.7)	3.9% (-3.9 to 11.6)	2.6% (0.3 to 4.8)	15.2% (11.9 to 18.8)	22.3% (7.0 to 38.6)
Nigeria	6.6% (-1.1 to 15.7)	17.4% (8.8 to 26.5)	0.3% (0.1 to 0.6)	-0.5% (-1.3 to 0.3)	3.2% (0.0 to 12.3)	0.0% (0.0 to 0.1)	0.1% (0.0 to 0.2)	2.1% (1.0 to 3.3)	0.0% (0.0 to 0.1)	2.1% (0.8 to 3.4)	3.8% (-3.9 to 10.9)	5.7% (0.8 to 10.7)	13.7% (10.6 to 17.1)	25.2% (8.3 to 42.7)

Niue	3.5% (-0.5 to 9.7)	3.0% (0.4 to 6.4)	0.3% (0.1 to 0.6)	-0.9% (-2.7 to 0.5)	9.4% (1.0 to 22.0)	0.4% (0.2 to 0.6)	0.0% (0.0 to 0.1)	1.8% (0.9 to 2.9)	0.0% (0.0 to 0.0)	4.5% (2.1 to 7.0)	4.7% (-5.0 to 13.1)	11.0% (1.8 to 20.5)	29.0% (23.8 to 34.5)	26.2% (8.9 to 42.7)
North Macedonia	7.1% (-0.8 to 16.9)	18.6% (12.5 to 23.3)	0.8% (0.2 to 1.4)	-0.6% (-1.9 to 0.3)	15.7% (4.1 to 30.1)	0.3% (0.1 to 0.5)	0.4% (-0.1 to 1.2)	0.8% (0.1 to 1.6)	0.0% (0.0 to 0.0)	0.1% (-0.1 to 0.3)	4.2% (-4.2 to 13.7)	7.8% (1.1 to 15.4)	22.9% (18.0 to 28.1)	29.8% (9.7 to 49.9)
Northern Mariana Islands	2.8% (-0.3 to 8.5)	5.8% (2.8 to 9.2)	0.3% (0.1 to 0.5)	-0.8% (-2.5 to 0.6)	9.8% (1.1 to 22.7)	0.4% (0.2 to 0.7)	0.0% (0.0 to 0.0)	1.4% (0.7 to 2.3)	0.0% (0.0 to 0.0)	3.4% (1.4 to 5.5)	4.6% (-4.7 to 13.4)	12.1% (2.0 to 22.1)	26.0% (21.1 to 31.3)	29.7% (10.2 to 48.5)
Norway	8.9% (-1.2 to 21.7)	2.4% (0.9 to 4.3)	5.5% (1.3 to 9.6)	0.9% (-0.5 to 2.8)	3.5% (0.0 to 12.2)	0.6% (0.3 to 0.9)	1.0% (-0.1 to 2.6)	1.1% (0.3 to 2.0)	0.0% (0.0 to 0.0)	1.6% (0.2 to 3.0)	4.4% (-4.7 to 13.5)	5.7% (0.8 to 11.1)	18.7% (14.6 to 22.8)	32.7% (11.2 to 53.1)
Oman	0.3% (0.0 to 0.9)	29.6% (21.6 to 38.4)	0.2% (0.1 to 0.4)	-1.2% (-3.3 to 0.6)	1.6% (0.0 to 8.3)	0.2% (0.1 to 0.3)	0.4% (0.0 to 0.9)	0.8% (0.4 to 1.3)	0.0% (0.0 to 0.0)	0.9% (0.4 to 1.5)	5.1% (-5.3 to 14.4)	12.2% (2.0 to 22.9)	23.3% (18.7 to 28.1)	33.8% (12.1 to 53.6)
Pakistan	0.7% (-0.1 to 2.0)	17.5% (8.9 to 26.1)	0.8% (0.2 to 1.4)	-0.7% (-2.1 to 0.4)	6.2% (0.1 to 18.1)	0.0% (0.0 to 0.1)	2.6% (-0.1 to 5.4)	3.3% (1.8 to 5.3)	0.0% (0.0 to 0.1)	5.2% (2.5 to 8.0)	7.2% (-7.9 to 20.0)	4.7% (0.7 to 8.9)	21.7% (17.4 to 26.5)	27.1% (9.1 to 45.4)
Palau	3.8% (-0.5 to 10.8)	3.7% (0.3 to 7.6)	0.3% (0.1 to 0.5)	-1.0% (-3.2 to 0.6)	9.4% (1.0 to 22.5)	0.4% (0.2 to 0.6)	0.0% (0.0 to 0.1)	1.9% (1.0 to 2.9)	0.0% (0.0 to 0.0)	4.7% (2.4 to 7.1)	5.2% (-5.6 to 14.5)	12.4% (2.0 to 23.0)	29.7% (23.9 to 35.6)	30.5% (11.1 to 48.7)
Palestine	0.5% (-0.1 to 1.6)	20.5% (14.0 to 28.1)	0.1% (0.0 to 0.2)	-0.4% (-1.2 to 0.2)	1.4% (0.0 to 7.3)	0.0% (0.0 to 0.1)	2.3% (-0.1 to 5.1)	1.5% (0.6 to 2.5)	0.0% (0.0 to 0.0)	1.0% (0.0 to 2.0)	6.0% (-6.6 to 17.6)	11.8% (2.0 to 22.4)	21.0% (16.5 to 25.6)	30.4% (10.3 to 49.9)
Panama	4.3% (-0.6 to 11.5)	7.8% (4.3 to 12.4)	0.2% (0.0 to 0.4)	-0.8% (-2.6 to 0.5)	8.1% (0.5 to 20.7)	0.2% (0.1 to 0.3)	2.7% (-0.1 to 6.0)	1.5% (0.6 to 2.5)	0.0% (0.0 to 0.0)	3.6% (1.1 to 6.0)	1.9% (-1.7 to 5.5)	10.2% (1.6 to 19.6)	19.6% (15.6 to 24.0)	29.2% (9.9 to 48.4)
Papua New Guinea	0.9% (-0.1 to 2.6)	4.9% (1.4 to 11.5)	0.1% (0.0 to 0.2)	-0.8% (-2.3 to 0.4)	9.2% (0.9 to 21.6)	0.0% (0.0 to 0.1)	0.0% (0.0 to 0.1)	2.3% (1.0 to 3.9)	0.0% (0.0 to 0.1)	3.6% (1.6 to 5.6)	5.3% (-5.6 to 14.9)	3.9% (0.5 to 7.6)	19.0% (14.9 to 23.5)	26.4% (8.8 to 44.0)
Paraguay	8.1% (-1.1 to 19.8)	6.9% (2.6 to 13.1)	0.3% (0.1 to 0.5)	1.4% (-0.9 to 4.2)	7.0% (0.2 to 19.0)	0.1% (0.1 to 0.3)	0.2% (0.0 to 0.6)	1.1% (0.4 to 1.9)	0.0% (0.0 to 0.0)	3.1% (1.0 to 5.2)	2.7% (-2.5 to 8.1)	8.1% (1.2 to 16.0)	19.7% (15.6 to 24.0)	31.3% (10.4 to 51.1)
Peru	6.1% (-1.0 to 15.2)	20.0% (12.3 to 27.9)	0.1% (0.0 to 0.2)	-0.7% (-2.0 to 0.4)	6.5% (0.2 to 18.6)	0.2% (0.1 to 0.3)	0.6% (0.0 to 1.4)	1.6% (0.8 to 2.6)	0.0% (0.0 to 0.0)	2.9% (1.2 to 4.7)	3.3% (-3.3 to 9.4)	8.0% (1.2 to 15.5)	14.5% (11.3 to 18.1)	29.9% (10.3 to 48.6)
Philippines	7.0% (-1.3 to 16.6)	12.3% (6.7 to 16.8)	0.2% (0.0 to 0.3)	-1.0% (-2.8 to 0.5)	11.5% (2.0 to 25.1)	0.1% (0.1 to 0.2)	4.4% (-0.2 to 8.7)	1.7% (0.9 to 2.5)	0.0% (0.0 to 0.1)	3.0% (1.5 to 4.6)	2.5% (-2.4 to 6.8)	4.2% (0.6 to 7.8)	14.2% (11.1 to 17.9)	32.0% (11.5 to 51.0)
Poland	9.5% (-1.3 to 22.4)	13.8% (10.5 to 17.3)	1.0% (0.2 to 1.7)	1.5% (-1.0 to 4.3)	11.6% (2.0 to 25.3)	0.6% (0.3 to 1.0)	0.4% (0.0 to 1.0)	1.9% (0.8 to 3.1)	0.0% (0.0 to 0.0)	0.4% (0.1 to 0.8)	4.5% (-4.5 to 13.1)	8.8% (1.3 to 17.2)	22.3% (17.9 to 27.0)	31.7% (11.0 to 51.2)
Portugal	9.9% (-1.4 to 23.8)	4.0% (2.1 to 6.1)	0.9% (0.2 to 1.5)	2.1% (-1.4 to 5.3)	3.5% (0.1 to 12.3)	0.4% (0.2 to 0.7)	0.7% (-0.1 to 1.8)	1.0% (0.2 to 1.8)	0.0% (0.0 to 0.0)	0.3% (-0.1 to 0.8)	3.5% (-3.7 to 11.4)	6.5% (0.9 to 13.2)	22.0% (17.4 to 26.5)	31.3% (10.2 to 51.5)
Puerto Rico	4.1% (-0.6 to 10.9)	2.6% (0.7 to 4.8)	0.4% (0.1 to 0.7)	-1.0% (-2.8 to 0.5)	4.3% (0.0 to 14.3)	0.3% (0.1 to 0.5)	1.2% (0.0 to 2.7)	1.3% (0.7 to 2.1)	0.0% (0.0 to 0.0)	5.1% (2.1 to 8.3)	4.9% (-5.2 to 14.1)	11.1% (1.8 to 20.6)	26.3% (21.0 to 31.6)	31.5% (11.3 to 50.5)
Qatar	0.4% (0.0 to 1.1)	41.4% (32.7 to 49.2)	0.5% (0.1 to 0.9)	-0.8% (-2.5 to 0.4)	1.5% (0.0 to 7.6)	1.1% (0.5 to 1.7)	0.0% (0.0 to 0.1)	0.4% (0.2 to 0.7)	0.0% (0.0 to 0.0)	0.1% (0.0 to 0.2)	5.3% (-5.7 to 15.4)	12.9% (2.1 to 24.1)	33.6% (27.7 to 39.5)	28.3% (9.5 to 47.0)
Republic of Korea	7.8% (-1.3 to 19.0)	19.1% (13.0 to 26.2)	0.9% (0.2 to 1.4)	0.1% (-0.9 to 1.5)	13.7% (3.0 to 27.7)	0.3% (0.1 to 0.5)	2.8% (-0.2 to 6.2)	1.4% (0.5 to 2.3)	0.0% (0.0 to 0.0)	0.5% (-0.1 to 1.1)	2.0% (-1.9 to 6.0)	3.3% (0.4 to 6.4)	19.7% (15.7 to 24.2)	28.6% (9.7 to 46.9)
Republic of Moldova	10.0% (-0.8 to 26.3)	9.0% (4.3 to 15.1)	1.4% (0.3 to 2.5)	-1.0% (-2.8 to 0.5)	3.1% (0.0 to 12.1)	0.1% (0.0 to 0.1)	0.7% (0.0 to 1.4)	2.2% (1.2 to 3.4)	0.0% (0.0 to 0.0)	1.5% (0.8 to 2.3)	4.7% (-4.6 to 13.1)	11.8% (1.9 to 22.2)	15.6% (12.2 to 19.2)	29.8% (10.3 to 48.6)
Romania	9.9% (-1.2 to 23.9)	10.6% (7.7 to 13.7)	2.0% (0.5 to 3.5)	1.2% (-0.7 to 3.6)	16.3% (4.5 to 31.1)	0.9% (0.5 to 1.5)	0.3% (0.0 to 0.7)	1.3% (0.4 to 2.2)	0.0% (0.0 to 0.0)	0.0% (0.0 to 0.1)	3.4% (-3.3 to 10.4)	8.6% (1.3 to 16.4)	19.9% (15.7 to 24.4)	32.7% (11.0 to 53.1)
Russian Federation	6.6% (-0.7 to 17.8)	7.6% (4.6 to 11.7)	1.9% (0.4 to 3.4)	-0.2% (-1.3 to 1.3)	6.5% (0.4 to 17.9)	0.2% (0.1 to 0.3)	1.2% (0.0 to 2.6)	2.1% (1.0 to 3.3)	0.0% (0.0 to 0.0)	1.3% (0.6 to 2.1)	5.3% (-5.5 to 15.1)	10.2% (1.6 to 19.6)	14.9% (11.7 to 18.7)	35.6% (12.8 to 55.7)

Rwanda	5.2% (-0.8 to 13.3)	3.7% (1.8 to 6.8)	0.1% (0.0 to 0.2)	-0.3% (-0.9 to 0.2)	7.6% (0.2 to 20.3)	0.0% (0.0 to 0.1)	0.0% (0.0 to 0.0)	0.1% (0.0 to 0.1)	0.0% (0.0 to 0.1)	4.7% (1.2 to 7.8)	3.9% (-4.0 to 11.6)	2.4% (0.3 to 4.8)	10.1% (7.7 to 12.7)	16.7% (4.9 to 30.7)
Saint Kitts and Nevis	2.6% (-0.2 to 9.4)	3.9% (1.4 to 6.7)	0.2% (0.0 to 0.3)	-0.8% (-2.1 to 0.4)	4.4% (0.0 to 14.6)	0.1% (0.0 to 0.2)	3.5% (-0.2 to 7.8)	2.6% (1.0 to 4.4)	0.0% (0.0 to 0.0)	2.6% (0.8 to 4.4)	4.6% (-4.8 to 13.8)	8.1% (1.2 to 15.7)	21.4% (17.0 to 26.3)	27.7% (8.9 to 46.6)
Saint Lucia	5.7% (-0.9 to 14.9)	16.6% (6.6 to 29.8)	0.2% (0.0 to 0.3)	-0.9% (-2.7 to 0.5)	4.4% (0.0 to 14.9)	0.1% (0.1 to 0.2)	2.0% (-0.1 to 4.6)	1.2% (0.4 to 2.1)	0.0% (0.0 to 0.0)	3.9% (1.0 to 6.6)	5.0% (-5.2 to 15.0)	6.5% (1.0 to 12.8)	21.7% (16.9 to 26.6)	24.1% (7.7 to 41.3)
Saint Vincent and the Grenadines	6.5% (-0.8 to 16.4)	16.7% (6.3 to 31.3)	0.2% (0.0 to 0.3)	-0.7% (-2.0 to 0.3)	4.3% (0.0 to 14.2)	0.2% (0.1 to 0.3)	1.4% (-0.1 to 3.2)	1.1% (0.4 to 1.9)	0.0% (0.0 to 0.0)	3.4% (1.2 to 5.7)	4.7% (-4.8 to 13.6)	5.8% (0.9 to 11.5)	20.9% (16.7 to 25.6)	24.8% (8.0 to 42.6)
Samoa	1.6% (-0.2 to 4.5)	6.3% (1.8 to 14.6)	0.3% (0.1 to 0.5)	-1.0% (-3.0 to 0.5)	4.0% (0.0 to 13.3)	0.3% (0.1 to 0.5)	0.0% (0.0 to 0.0)	2.3% (1.2 to 3.5)	0.0% (0.0 to 0.1)	8.8% (4.4 to 13.0)	6.3% (-7.1 to 17.6)	12.6% (2.2 to 22.7)	26.5% (21.0 to 31.7)	26.0% (8.9 to 43.3)
San Marino	10.1% (-1.4 to 27.4)	6.4% (3.4 to 9.9)	2.3% (0.5 to 4.1)	1.5% (-0.9 to 4.5)	4.0% (0.0 to 13.7)	0.6% (0.3 to 1.0)	0.9% (0.0 to 2.1)	1.1% (0.4 to 2.0)	0.0% (0.0 to 0.0)	0.8% (0.2 to 1.5)	4.5% (-4.9 to 13.5)	7.8% (1.1 to 15.2)	18.1% (14.3 to 22.0)	32.9% (11.2 to 53.2)
Sao Tome and Principe	6.7% (-1.1 to 16.6)	8.4% (4.4 to 14.0)	0.5% (0.1 to 0.8)	-0.2% (-0.5 to 0.1)	3.8% (0.0 to 14.1)	0.1% (0.0 to 0.1)	0.0% (0.0 to 0.0)	1.1% (0.5 to 1.8)	0.0% (0.0 to 0.1)	5.8% (2.8 to 8.7)	5.8% (-6.4 to 15.6)	6.8% (1.0 to 13.3)	15.0% (11.8 to 18.6)	25.6% (8.7 to 42.8)
Saudi Arabia	0.1% (0.0 to 0.5)	35.2% (27.7 to 43.0)	0.2% (0.1 to 0.4)	-0.8% (-2.4 to 0.4)	1.6% (0.0 to 8.0)	0.8% (0.4 to 1.2)	0.4% (0.0 to 0.9)	1.8% (1.0 to 2.7)	0.0% (0.0 to 0.0)	1.4% (0.7 to 2.2)	6.2% (-6.6 to 17.0)	15.1% (2.5 to 27.1)	22.9% (18.1 to 28.0)	31.5% (11.6 to 50.6)
Senegal	0.9% (-0.1 to 2.6)	8.6% (4.2 to 14.9)	0.5% (0.1 to 0.8)	-0.6% (-1.7 to 0.3)	3.8% (0.0 to 13.7)	0.0% (0.0 to 0.0)	0.1% (0.0 to 0.3)	4.7% (2.3 to 7.3)	0.0% (0.0 to 0.0)	3.3% (1.5 to 5.2)	3.7% (-3.7 to 10.5)	4.6% (0.7 to 8.8)	19.7% (15.5 to 24.0)	23.9% (7.8 to 40.2)
Serbia	7.2% (-0.9 to 17.5)	16.4% (11.3 to 20.8)	0.6% (0.1 to 1.0)	-0.8% (-2.5 to 0.4)	16.2% (4.3 to 30.2)	0.2% (0.1 to 0.4)	0.8% (-0.1 to 2.0)	0.9% (0.3 to 1.6)	0.0% (0.0 to 0.0)	0.8% (0.2 to 1.5)	4.2% (-4.2 to 12.8)	9.1% (1.4 to 17.5)	21.5% (17.1 to 26.3)	34.4% (11.7 to 55.3)
Seychelles	3.9% (-0.6 to 9.8)	5.4% (1.7 to 9.9)	0.1% (0.0 to 0.2)	-0.5% (-1.5 to 0.3)	10.7% (1.4 to 24.1)	0.1% (0.0 to 0.1)	1.5% (-0.1 to 3.2)	1.9% (0.9 to 3.0)	0.0% (0.0 to 0.0)	4.6% (2.2 to 7.0)	2.7% (-2.6 to 7.7)	8.5% (1.3 to 15.9)	23.4% (18.5 to 28.4)	34.0% (12.5 to 54.2)
Sierra Leone	4.2% (-0.7 to 10.4)	6.6% (4.0 to 10.1)	0.3% (0.1 to 0.6)	-0.3% (-0.7 to 0.1)	3.8% (0.0 to 13.6)	0.0% (0.0 to 0.0)	3.4% (-0.2 to 6.9)	3.5% (1.8 to 5.4)	0.0% (0.0 to 0.1)	4.7% (2.3 to 7.2)	3.3% (-3.3 to 9.0)	4.0% (0.5 to 7.7)	14.2% (11.2 to 17.7)	24.5% (8.0 to 41.0)
Singapore	2.3% (-0.3 to 6.1)	12.1% (6.5 to 18.8)	0.8% (0.2 to 1.3)	0.1% (-1.1 to 2.0)	11.0% (1.6 to 24.2)	0.4% (0.2 to 0.7)	1.9% (-0.1 to 3.9)	1.3% (0.7 to 2.0)	0.0% (0.0 to 0.0)	0.7% (0.3 to 1.2)	2.9% (-2.9 to 8.0)	5.7% (0.8 to 10.7)	21.4% (17.2 to 25.8)	34.0% (12.6 to 53.4)
Slovakia	10.8% (-1.3 to 25.7)	11.3% (8.5 to 14.5)	1.7% (0.4 to 3.0)	-0.2% (-1.4 to 1.5)	16.3% (4.8 to 30.7)	0.5% (0.2 to 0.8)	2.1% (-0.1 to 4.6)	1.9% (0.8 to 3.0)	0.0% (0.0 to 0.0)	1.0% (0.3 to 1.7)	4.7% (-4.8 to 13.4)	10.2% (1.6 to 19.6)	20.3% (15.9 to 24.6)	33.2% (11.7 to 53.0)
Slovenia	7.5% (-0.7 to 20.1)	9.6% (7.0 to 12.7)	3.4% (0.8 to 6.0)	0.3% (-0.4 to 1.8)	16.2% (4.8 to 30.5)	0.7% (0.3 to 1.1)	0.8% (-0.1 to 1.9)	1.0% (0.3 to 1.8)	0.0% (0.0 to 0.0)	1.2% (0.2 to 2.1)	3.0% (-2.9 to 9.2)	9.0% (1.3 to 17.3)	21.4% (17.3 to 25.8)	31.4% (10.5 to 51.7)
Solomon Islands	0.8% (-0.1 to 2.3)	2.9% (1.0 to 6.6)	0.1% (0.0 to 0.1)	-0.5% (-1.4 to 0.3)	9.2% (1.0 to 22.1)	0.0% (0.0 to 0.1)	0.0% (0.0 to 0.1)	2.5% (1.2 to 3.9)	0.0% (0.0 to 0.1)	9.7% (4.9 to 14.5)	4.4% (-4.5 to 12.3)	6.5% (1.0 to 12.4)	16.3% (12.4 to 20.3)	27.8% (9.5 to 45.8)
Somalia	0.0% (0.0 to 0.0)	3.0% (2.1 to 4.4)	0.1% (0.0 to 0.2)	-0.9% (-2.6 to 0.5)	7.6% (0.2 to 20.5)	0.0% (0.0 to 0.0)	3.7% (-0.2 to 7.7)	6.3% (3.0 to 9.9)	0.0% (0.0 to 0.1)	9.5% (4.7 to 14.3)	4.8% (-4.9 to 13.9)	2.6% (0.3 to 5.0)	11.8% (9.1 to 15.0)	21.0% (6.7 to 36.6)
South Africa	5.3% (-0.9 to 13.6)	15.0% (10.5 to 19.9)	0.2% (0.0 to 0.3)	-0.6% (-1.8 to 0.6)	2.1% (0.0 to 9.6)	0.2% (0.1 to 0.3)	0.6% (0.0 to 1.2)	4.1% (1.8 to 6.8)	0.0% (0.0 to 0.0)	3.0% (1.2 to 4.9)	1.8% (-1.7 to 5.3)	10.0% (1.6 to 19.2)	17.2% (13.5 to 21.2)	25.8% (8.4 to 43.7)
South Sudan	0.2% (-0.1 to 0.9)	6.5% (3.6 to 10.7)	0.2% (0.1 to 0.4)	-0.6% (-1.7 to 0.3)	7.5% (0.2 to 20.3)	0.1% (0.0 to 0.2)	0.1% (0.0 to 0.2)	2.4% (1.1 to 3.9)	0.0% (0.0 to 0.1)	5.1% (2.2 to 7.8)	3.0% (-2.8 to 8.7)	1.4% (0.2 to 2.7)	12.1% (9.1 to 15.2)	21.1% (6.6 to 36.7)
Spain	9.3% (-1.4 to 22.4)	5.3% (3.4 to 7.7)	1.5% (0.3 to 2.8)	1.2% (-0.6 to 3.6)	2.2% (0.0 to 9.1)	0.4% (0.2 to 0.7)	1.3% (-0.1 to 2.9)	0.8% (0.3 to 1.4)	0.0% (0.0 to 0.0)	0.7% (0.1 to 1.3)	3.7% (-3.8 to 11.4)	8.6% (1.3 to 17.0)	19.6% (15.5 to 24.1)	30.9% (10.5 to 50.7)
Sri Lanka	2.6% (-0.4 to 6.8)	13.4% (6.0 to 19.6)	0.1% (0.0 to 0.1)	-0.1% (-0.3 to 0.1)	11.0% (1.5 to 24.3)	0.1% (0.0 to 0.1)	0.7% (0.0 to 1.6)	2.6% (0.9 to 4.3)	0.0% (0.0 to 0.1)	2.6% (0.6 to 4.6)	2.3% (-2.2 to 6.9)	3.5% (0.5 to 6.8)	18.6% (14.6 to 23.0)	30.7% (10.4 to 50.7)

Sudan	0.0% (0.0 to 0.0)	19.9% (10.8 to 31.2)	0.1% (0.0 to 0.2)	-0.8% (-2.2 to 0.4)	1.6% (0.0 to 8.1)	0.1% (0.0 to 0.1)	1.8% (-0.1 to 3.7)	2.0% (1.0 to 3.1)	0.0% (0.0 to 0.1)	2.1% (1.2 to 3.3)	7.7% (-8.7 to 21.2)	10.3% (1.6 to 19.8)	19.5% (15.0 to 24.2)	28.1% (9.9 to 45.8)
Suriname	4.2% (-0.6 to 10.8)	20.8% (8.8 to 34.7)	0.2% (0.1 to 0.4)	-0.5% (-1.3 to 0.2)	4.2% (0.0 to 14.2)	0.2% (0.1 to 0.3)	3.9% (-0.2 to 8.2)	1.7% (0.8 to 2.7)	0.0% (0.0 to 0.0)	3.4% (1.6 to 5.3)	3.4% (-3.4 to 9.8)	6.3% (0.9 to 12.1)	21.7% (17.1 to 26.4)	31.9% (11.2 to 51.3)
Sweden	10.0% (-1.4 to 24.0)	2.1% (0.6 to 4.0)	2.8% (0.6 to 5.4)	1.2% (-0.7 to 3.3)	4.2% (0.0 to 14.0)	0.4% (0.2 to 0.7)	1.1% (-0.1 to 2.5)	1.3% (0.4 to 2.3)	0.0% (0.0 to 0.0)	1.4% (0.3 to 2.5)	4.4% (-4.7 to 13.5)	6.4% (0.9 to 12.4)	17.0% (13.1 to 21.1)	32.5% (11.2 to 52.7)
Switzerland	11.4% (-1.5 to 27.0)	5.4% (3.5 to 7.7)	1.0% (0.3 to 1.8)	2.0% (-1.2 to 5.2)	3.9% (0.0 to 13.5)	0.3% (0.1 to 0.4)	1.9% (-0.1 to 4.4)	1.2% (0.3 to 2.1)	0.0% (0.0 to 0.0)	1.2% (0.2 to 2.2)	4.6% (-5.0 to 14.0)	5.7% (0.8 to 11.1)	18.5% (14.4 to 22.9)	33.1% (11.4 to 53.6)
Syrian Arab Republic	0.5% (0.0 to 1.5)	19.2% (13.7 to 25.2)	0.2% (0.0 to 0.4)	-0.9% (-2.4 to 0.5)	1.5% (0.0 to 7.8)	0.1% (0.1 to 0.2)	1.2% (-0.1 to 2.5)	1.4% (0.7 to 2.2)	0.0% (0.0 to 0.0)	1.6% (0.7 to 2.5)	7.3% (-8.3 to 20.0)	13.9% (2.3 to 25.4)	21.1% (17.0 to 25.9)	31.5% (11.4 to 50.5)
Turkey	1.2% (-0.1 to 3.6)	17.4% (13.4 to 21.7)	0.3% (0.1 to 0.5)	-0.7% (-2.0 to 0.4)	1.0% (0.0 to 6.3)	0.4% (0.2 to 0.7)	0.1% (0.0 to 0.2)	0.5% (0.2 to 0.8)	0.0% (0.0 to 0.0)	0.0% (0.0 to 0.1)	6.4% (-7.2 to 18.4)	10.7% (1.6 to 20.5)	21.4% (17.0 to 26.3)	28.8% (9.8 to 47.8)
Taiwan (Province of China)	4.4% (-0.6 to 10.7)	12.3% (9.3 to 15.6)	0.5% (0.1 to 0.8)	2.2% (-1.3 to 5.7)	9.1% (0.8 to 21.9)	0.2% (0.1 to 0.3)	2.0% (-0.1 to 4.3)	1.2% (0.6 to 1.9)	0.0% (0.0 to 0.0)	0.6% (0.2 to 1.2)	4.6% (-4.8 to 12.7)	6.1% (0.9 to 11.5)	18.9% (15.0 to 23.2)	35.0% (13.1 to 54.6)
Tajikistan	0.9% (-0.1 to 2.6)	13.6% (7.5 to 21.0)	0.7% (0.2 to 1.2)	-0.7% (-1.9 to 0.4)	6.6% (0.3 to 18.0)	0.1% (0.0 to 0.1)	2.5% (-0.1 to 5.2)	2.7% (1.3 to 4.3)	0.0% (0.0 to 0.0)	0.4% (0.1 to 0.8)	7.5% (-8.4 to 20.6)	8.4% (1.3 to 16.0)	16.2% (12.7 to 20.0)	28.0% (9.6 to 46.0)
Thailand	4.8% (-0.8 to 11.8)	21.6% (15.4 to 27.8)	0.1% (0.0 to 0.2)	-1.0% (-2.8 to 0.5)	11.3% (1.8 to 24.6)	0.2% (0.1 to 0.4)	4.5% (-0.3 to 8.7)	1.4% (0.8 to 2.1)	0.0% (0.0 to 0.0)	3.7% (2.1 to 5.5)	2.3% (-2.3 to 6.3)	5.5% (0.8 to 10.4)	16.7% (12.9 to 20.5)	35.5% (13.3 to 55.0)
Timor-Leste	3.3% (-0.5 to 8.7)	9.0% (3.1 to 17.2)	0.1% (0.0 to 0.1)	-0.6% (-1.6 to 0.3)	11.9% (1.9 to 25.7)	0.0% (0.0 to 0.0)	1.6% (-0.1 to 3.5)	6.4% (3.1 to 10.2)	0.0% (0.0 to 0.1)	6.5% (2.8 to 10.1)	2.2% (-2.0 to 6.3)	1.1% (0.1 to 2.1)	13.9% (10.9 to 17.3)	24.6% (8.3 to 41.8)
Togo	3.7% (-0.6 to 9.5)	8.7% (5.3 to 13.6)	0.4% (0.1 to 0.7)	-0.3% (-0.9 to 0.2)	3.8% (0.0 to 14.0)	0.0% (0.0 to 0.1)	0.0% (0.0 to 0.1)	9.7% (5.1 to 14.6)	0.0% (0.0 to 0.1)	8.1% (4.3 to 11.7)	3.6% (-3.6 to 10.3)	4.8% (0.7 to 9.1)	12.9% (10.0 to 16.2)	26.4% (8.9 to 44.0)
Tokelau	2.6% (-0.3 to 7.1)	2.5% (0.0 to 6.4)	0.3% (0.1 to 0.5)	-1.0% (-2.9 to 0.6)	9.1% (0.9 to 21.8)	0.2% (0.1 to 0.4)	0.1% (0.0 to 0.2)	2.3% (1.2 to 3.5)	0.0% (0.0 to 0.0)	5.3% (2.6 to 8.2)	5.0% (-5.3 to 14.1)	11.2% (1.8 to 20.5)	31.9% (25.8 to 38.1)	29.0% (10.1 to 47.1)
Tonga	0.8% (-0.1 to 2.5)	6.9% (2.3 to 15.4)	0.2% (0.0 to 0.3)	-0.9% (-2.7 to 0.4)	9.6% (0.9 to 22.8)	0.1% (0.1 to 0.2)	0.1% (0.0 to 0.3)	2.2% (1.1 to 3.5)	0.0% (0.0 to 0.0)	5.7% (2.6 to 8.7)	4.9% (-5.1 to 13.7)	13.5% (2.3 to 23.9)	23.4% (18.6 to 28.3)	30.9% (10.8 to 49.5)
Trinidad and Tobago	5.5% (-0.8 to 14.1)	17.8% (6.1 to 34.2)	0.2% (0.1 to 0.4)	-0.7% (-2.0 to 0.4)	4.4% (0.0 to 14.8)	0.3% (0.1 to 0.4)	1.4% (-0.1 to 3.1)	2.0% (1.0 to 3.2)	0.0% (0.0 to 0.0)	3.7% (1.6 to 6.0)	5.5% (-5.8 to 15.7)	8.6% (1.3 to 16.1)	21.4% (17.0 to 26.4)	30.7% (10.6 to 49.8)
Tunisia	0.9% (-0.1 to 2.6)	17.5% (12.1 to 23.8)	0.2% (0.0 to 0.3)	-0.7% (-2.0 to 0.4)	1.5% (0.0 to 7.6)	0.2% (0.1 to 0.4)	0.2% (0.0 to 0.3)	1.2% (0.6 to 1.9)	0.0% (0.0 to 0.0)	0.3% (0.0 to 0.7)	6.5% (-7.2 to 18.3)	9.4% (1.5 to 17.8)	20.5% (16.3 to 25.2)	31.4% (10.6 to 50.5)
Turkmenistan	5.0% (-0.6 to 14.0)	14.8% (8.3 to 22.4)	1.0% (0.3 to 1.8)	3.9% (-2.2 to 9.2)	6.2% (0.3 to 17.6)	0.4% (0.2 to 0.7)	1.9% (-0.1 to 3.9)	1.9% (1.1 to 2.8)	0.0% (0.0 to 0.1)	0.1% (0.0 to 0.3)	9.0% (-10.3 to 23.7)	9.4% (1.4 to 18.0)	15.1% (11.8 to 18.7)	32.3% (12.0 to 50.9)
Tuvalu	1.9% (-0.3 to 5.0)	1.7% (0.7 to 3.4)	0.1% (0.0 to 0.2)	-1.0% (-2.9 to 0.6)	9.4% (1.0 to 22.1)	0.1% (0.0 to 0.1)	0.2% (0.0 to 0.4)	2.7% (1.4 to 4.2)	0.0% (0.0 to 0.1)	6.6% (3.4 to 10.0)	5.4% (-5.7 to 15.2)	11.2% (1.9 to 20.4)	17.4% (13.6 to 21.6)	29.4% (10.0 to 47.7)
Uganda	5.2% (-0.9 to 13.7)	5.9% (3.5 to 9.4)	0.1% (0.0 to 0.3)	-0.5% (-1.6 to 0.3)	7.6% (0.2 to 20.0)	0.0% (0.0 to 0.1)	0.1% (0.0 to 0.2)	0.7% (0.3 to 1.2)	0.0% (0.0 to 0.1)	6.3% (2.6 to 10.1)	2.6% (-2.4 to 7.6)	2.8% (0.4 to 5.3)	12.6% (9.7 to 15.8)	19.6% (5.9 to 35.2)
Ukraine	7.0% (-0.7 to 19.6)	11.0% (6.5 to 17.2)	0.6% (0.2 to 1.1)	-1.2% (-3.4 to 0.6)	3.0% (0.0 to 11.6)	0.1% (0.0 to 0.1)	0.9% (0.0 to 1.8)	2.9% (1.4 to 4.5)	0.0% (0.0 to 0.0)	0.5% (0.1 to 0.9)	6.5% (-7.0 to 18.1)	10.4% (1.6 to 19.6)	13.3% (10.5 to 16.7)	35.3% (12.8 to 55.2)
United Arab Emirates	1.5% (-0.1 to 4.6)	28.6% (21.3 to 36.5)	0.5% (0.1 to 0.8)	-0.9% (-2.6 to 0.4)	1.5% (0.0 to 8.2)	0.6% (0.3 to 1.0)	0.4% (0.0 to 0.9)	1.0% (0.4 to 1.7)	0.0% (0.0 to 0.0)	0.9% (0.3 to 1.5)	5.1% (-5.4 to 15.0)	12.6% (2.0 to 23.5)	28.7% (23.2 to 34.1)	32.6% (11.0 to 53.1)

United Kingdom	10.4% (-1.5 to 24.8)	5.5% (3.6 to 7.8)	1.4% (0.3 to 2.5)	0.1% (-0.4 to 1.3)	3.4% (0.0 to 12.0)	0.2% (0.1 to 0.4)	1.3% (-0.1 to 3.0)	1.8% (0.6 to 3.2)	0.0% (0.0 to 0.0)	1.3% (0.2 to 2.4)	4.1% (-4.3 to 12.4)	8.4% (1.2 to 16.4)	15.1% (11.8 to 18.9)	31.4% (10.6 to 51.3)
United Republic of Tanzania	5.7% (-1.0 to 14.3)	5.1% (2.8 to 8.4)	0.2% (0.0 to 0.4)	-0.4% (-1.2 to 0.2)	10.2% (0.9 to 23.9)	0.1% (0.0 to 0.1)	0.1% (0.0 to 0.3)	1.6% (0.6 to 2.6)	0.0% (0.0 to 0.0)	4.2% (1.3 to 6.8)	1.8% (-1.7 to 5.3)	4.9% (0.7 to 9.6)	12.0% (9.3 to 14.9)	27.1% (9.0 to 45.3)
United States of America	7.6% (-1.0 to 19.3)	3.2% (1.6 to 5.1)	3.4% (0.9 to 5.8)	1.7% (-1.1 to 4.6)	4.5% (0.1 to 14.3)	0.8% (0.4 to 1.3)	1.3% (0.0 to 2.9)	1.7% (0.8 to 2.6)	0.0% (0.0 to 0.0)	1.5% (0.6 to 2.4)	4.0% (-4.1 to 11.5)	11.4% (1.8 to 20.8)	23.9% (19.2 to 28.8)	29.0% (10.2 to 47.0)
United States Virgin Islands	6.4% (-0.6 to 18.9)	3.9% (1.8 to 6.4)	0.3% (0.1 to 0.6)	-0.9% (-2.7 to 0.5)	4.4% (0.0 to 14.6)	0.4% (0.2 to 0.6)	0.9% (0.0 to 2.1)	0.9% (0.4 to 1.5)	0.0% (0.0 to 0.0)	2.0% (0.7 to 3.3)	4.5% (-4.8 to 13.0)	10.3% (1.6 to 19.4)	24.5% (19.7 to 30.2)	29.2% (10.1 to 48.1)
Uruguay	8.2% (-1.2 to 19.9)	6.3% (2.4 to 11.2)	0.9% (0.2 to 1.6)	2.7% (-1.5 to 7.2)	7.1% (0.2 to 19.3)	0.6% (0.3 to 0.9)	1.2% (-0.1 to 2.6)	1.4% (0.5 to 2.3)	0.0% (0.0 to 0.0)	2.5% (0.9 to 4.1)	5.7% (-6.2 to 16.7)	8.6% (1.3 to 16.6)	17.0% (13.2 to 21.1)	32.5% (11.2 to 52.4)
Uzbekistan	3.7% (-0.5 to 10.4)	22.3% (13.5 to 31.1)	0.5% (0.1 to 0.9)	-0.6% (-2.2 to 1.2)	6.5% (0.3 to 17.9)	0.1% (0.1 to 0.2)	1.1% (-0.1 to 2.4)	1.9% (1.0 to 2.9)	0.0% (0.0 to 0.1)	0.0% (0.0 to 0.1)	8.5% (-9.8 to 23.0)	9.9% (1.5 to 18.9)	15.4% (12.0 to 18.9)	33.1% (12.1 to 52.5)
Vanuatu	1.9% (-0.3 to 5.4)	3.6% (1.2 to 8.0)	0.2% (0.0 to 0.3)	-1.1% (-3.3 to 0.6)	9.8% (1.1 to 22.5)	0.1% (0.0 to 0.2)	0.1% (0.0 to 0.2)	1.9% (1.0 to 2.9)	0.0% (0.0 to 0.1)	5.6% (2.9 to 8.4)	5.4% (-5.9 to 14.9)	6.9% (1.0 to 13.2)	21.5% (17.0 to 26.2)	30.5% (10.7 to 49.4)
Venezuela (Bolivarian Republic of)	3.3% (-0.5 to 8.9)	10.4% (5.9 to 15.7)	0.2% (0.1 to 0.4)	-1.0% (-2.8 to 0.5)	8.1% (0.5 to 20.3)	0.2% (0.1 to 0.3)	1.9% (-0.1 to 4.2)	1.3% (0.5 to 2.2)	0.0% (0.0 to 0.0)	2.5% (0.8 to 4.1)	2.2% (-2.0 to 6.5)	9.3% (1.4 to 17.8)	18.5% (14.7 to 22.7)	25.7% (8.5 to 43.5)
Viet Nam	8.8% (-1.4 to 19.6)	11.8% (5.7 to 17.5)	0.0% (0.0 to 0.1)	-0.9% (-2.7 to 0.5)	12.3% (2.1 to 26.1)	0.0% (0.0 to 0.0)	4.0% (-0.2 to 8.4)	1.7% (0.8 to 2.8)	0.0% (0.0 to 0.1)	1.1% (0.3 to 1.9)	1.6% (-1.4 to 4.8)	1.3% (0.2 to 2.6)	14.3% (11.0 to 17.8)	27.2% (8.9 to 45.7)
Yemen	0.3% (0.0 to 0.7)	16.8% (9.3 to 25.7)	0.1% (0.0 to 0.2)	-0.5% (-1.5 to 0.3)	1.5% (0.0 to 8.0)	0.0% (0.0 to 0.1)	2.4% (-0.1 to 5.0)	3.3% (1.8 to 5.0)	0.0% (0.0 to 0.1)	4.9% (2.6 to 7.5)	7.9% (-8.8 to 21.7)	6.7% (1.0 to 12.6)	17.1% (13.4 to 21.3)	30.8% (10.6 to 49.2)
Zambia	4.9% (-0.8 to 12.6)	7.4% (3.6 to 13.0)	0.2% (0.1 to 0.4)	-0.5% (-1.5 to 0.3)	7.5% (0.2 to 20.3)	0.0% (0.0 to 0.1)	0.2% (0.0 to 0.4)	8.4% (3.5 to 13.5)	0.0% (0.0 to 0.1)	6.3% (2.4 to 9.8)	1.8% (-1.6 to 5.2)	4.4% (0.6 to 8.4)	16.5% (12.9 to 20.5)	19.5% (6.0 to 35.1)
Zimbabwe	4.3% (-0.5 to 10.9)	4.3% (2.4 to 7.1)	0.1% (0.0 to 0.2)	-0.6% (-1.5 to 0.3)	3.8% (0.0 to 14.2)	0.0% (0.0 to 0.1)	0.3% (0.0 to 0.8)	8.0% (3.6 to 12.7)	0.0% (0.0 to 0.0)	9.0% (4.4 to 13.6)	2.3% (-2.2 to 6.5)	6.3% (0.9 to 12.2)	15.1% (11.7 to 18.9)	23.5% (7.5 to 40.7)

Appendix Table 12 continues. Age-standardized percent of DALYs (with 95% uncertainty intervals) due to ischemic stroke associated with individual risk factors by 7 GBD super-region, 21 GBD region and country for both sexes combined in 2021.

Country/region	High systolic blood pressure	High temperature	Household air pollution from solid fuels	Kidney dysfunction	Lead exposure	Low physical activity	Low temperature	Secondhand smoke	Smoking
**GBD super-regions in alphabetical order**									
**Central Europe, Eastern Europe, and Central Asia**	62.8% (48.3 to 73.6)	0.0% (-0.5 to 0.6)	1.0% (0.3 to 4.2)	10.3% (7.3 to 13.4)	3.4% (-0.4 to 7.8)	3.6% (0.4 to 7.3)	8.4% (7.1 to 9.7)	3.1% (2.1 to 4.2)	11.2% (9.6 to 13.0)
**High-income**	52.9% (39.2 to 63.5)	0.1% (-0.2 to 0.6)	0.0% (0.0 to 0.2)	9.2% (6.1 to 12.3)	3.6% (-0.5 to 8.4)	4.4% (0.2 to 8.9)	5.5% (4.8 to 6.4)	2.0% (1.3 to 2.6)	10.3% (8.6 to 12.1)
**Latin America and Caribbean**	56.4% (41.9 to 67.5)	0.4% (0.2 to 0.7)	4.0% (2.0 to 8.4)	10.2% (7.3 to 13.1)	8.0% (-1.1 to 17.9)	4.9% (1.1 to 9.3)	2.3% (2.0 to 2.7)	2.4% (1.6 to 3.3)	7.5% (6.2 to 9.0)
**North Africa and Middle East**	58.1% (43.3 to 69.2)	3.0% (0.2 to 6.7)	3.0% (2.1 to 4.5)	10.6% (7.4 to 14.0)	9.7% (-1.4 to 21.6)	6.5% (2.2 to 11.4)	5.0% (4.1 to 6.1)	4.4% (3.0 to 5.8)	9.6% (8.1 to 11.3)
**South Asia**	56.6% (42.3 to 67.7)	2.6% (0.7 to 5.1)	24.4% (15.8 to 34.4)	11.4% (8.0 to 14.9)	10.9% (-1.5 to 24.0)	4.4% (1.0 to 8.3)	2.2% (0.9 to 3.7)	3.9% (2.7 to 5.3)	8.6% (6.8 to 10.6)
**Southeast Asia, East Asia, and Oceania**	58.0% (43.5 to 69.5)	0.6% (-0.1 to 1.6)	7.3% (2.2 to 18.7)	8.9% (6.3 to 11.6)	7.9% (-1.0 to 17.6)	4.6% (0.8 to 9.2)	5.2% (4.6 to 5.9)	4.8% (3.3 to 6.5)	14.7% (12.2 to 17.5)
**Sub-Saharan Africa**	60.3% (45.7 to 71.0)	1.4% (0.9 to 2.2)	32.0% (24.6 to 39.7)	10.8% (7.8 to 14.0)	7.6% (-1.0 to 16.9)	3.4% (0.8 to 6.5)	1.6% (1.4 to 1.9)	1.9% (1.3 to 2.6)	4.0% (3.2 to 4.9)
**GBD regions in alphabetical order**									
**Andean Latin America**	49.8% (36.1 to 61.6)	0.1% (0.0 to 0.2)	3.7% (0.7 to 12.0)	7.5% (5.2 to 10.0)	6.6% (-0.8 to 14.9)	3.4% (0.5 to 6.5)	5.2% (4.5 to 6.1)	1.5% (0.9 to 2.0)	5.0% (4.1 to 6.1)
**Australasia**	51.9% (37.4 to 63.7)	0.1% (-0.1 to 0.3)	0.0% (0.0 to 0.0)	8.4% (5.3 to 11.8)	5.7% (-0.7 to 12.9)	5.2% (-0.1 to 11.3)	4.5% (3.8 to 5.5)	1.6% (1.0 to 2.1)	6.7% (5.5 to 8.4)
**Caribbean**	54.4% (40.0 to 65.6)	0.4% (0.3 to 0.6)	11.3% (8.0 to 15.5)	8.6% (6.2 to 11.3)	9.6% (-1.3 to 21.5)	4.9% (1.3 to 8.8)	0.3% (0.1 to 0.5)	2.4% (1.6 to 3.2)	7.8% (6.5 to 9.3)
**Central Asia**	63.0% (47.7 to 73.9)	0.5% (-0.4 to 1.6)	4.4% (1.8 to 10.0)	12.9% (9.4 to 16.4)	4.2% (-0.6 to 9.7)	3.2% (0.5 to 6.1)	7.8% (6.6 to 8.8)	4.1% (2.8 to 5.6)	9.7% (8.3 to 11.1)
**Central Europe**	62.8% (48.0 to 73.5)	0.1% (-0.3 to 0.9)	1.2% (0.0 to 7.7)	8.1% (5.4 to 10.9)	4.2% (-0.5 to 9.5)	3.8% (0.2 to 8.0)	8.4% (7.5 to 10.1)	3.4% (2.3 to 4.5)	10.5% (8.9 to 12.3)
**Central Latin America**	57.5% (42.8 to 68.7)	0.5% (0.2 to 0.8)	4.0% (1.5 to 9.4)	11.7% (8.3 to 15.0)	9.0% (-1.2 to 19.8)	3.7% (0.8 to 7.1)	2.9% (2.5 to 3.4)	2.3% (1.6 to 3.1)	5.2% (4.3 to 6.1)
**Central Sub-Saharan Africa**	58.3% (43.7 to 70.2)	0.2% (0.0 to 0.7)	35.5% (28.2 to 42.6)	12.5% (8.9 to 16.3)	7.3% (-1.0 to 16.4)	3.7% (0.5 to 7.3)	1.0% (0.8 to 1.2)	1.6% (1.1 to 2.3)	3.1% (2.4 to 4.0)
**East Asia**	56.6% (42.1 to 68.6)	0.4% (-0.3 to 1.7)	5.7% (1.3 to 17.7)	7.9% (5.4 to 10.4)	8.4% (-1.1 to 18.7)	4.4% (0.6 to 8.9)	6.6% (5.9 to 7.4)	4.9% (3.3 to 6.7)	15.6% (13.0 to 18.6)
**Eastern Europe**	62.9% (48.1 to 73.9)	-0.1% (-0.5 to 0.3)	0.4% (0.0 to 1.6)	10.9% (7.7 to 14.3)	2.9% (-0.4 to 6.7)	3.5% (0.3 to 7.2)	8.6% (7.0 to 10.0)	2.8% (1.9 to 3.8)	11.9% (10.2 to 13.7)

**Eastern Sub-Saharan Africa**	56.1% (42.1 to 67.2)	0.4% (0.1 to 1.0)	43.5% (36.2 to 50.5)	8.2% (5.5 to 11.1)	8.4% (-1.1 to 18.6)	2.3% (0.6 to 4.2)	2.4% (2.1 to 2.9)	1.7% (1.1 to 2.3)	4.5% (3.6 to 5.7)
**High-income Asia Pacific**	51.9% (38.1 to 63.2)	0.1% (-0.2 to 0.6)	0.0% (0.0 to 0.0)	10.0% (6.4 to 13.5)	3.5% (-0.4 to 8.0)	5.3% (1.5 to 9.6)	5.2% (4.4 to 6.0)	2.5% (1.7 to 3.4)	11.2% (9.3 to 13.2)
**High-income North America**	50.0% (35.9 to 62.3)	0.3% (-0.4 to 1.2)	0.0% (0.0 to 0.0)	10.3% (7.1 to 13.7)	3.1% (-0.4 to 7.1)	4.0% (0.2 to 8.7)	4.6% (3.8 to 5.3)	1.5% (1.0 to 2.1)	10.7% (8.9 to 12.9)
**North Africa and Middle East**	58.1% (43.3 to 69.2)	3.0% (0.2 to 6.7)	3.0% (2.1 to 4.5)	10.6% (7.4 to 14.0)	9.7% (-1.4 to 21.6)	6.5% (2.2 to 11.4)	5.0% (4.1 to 6.1)	4.4% (3.0 to 5.8)	9.6% (8.1 to 11.3)
**Oceania**	44.7% (32.0 to 55.9)	0.2% (0.1 to 0.2)	33.5% (25.1 to 41.8)	9.9% (7.2 to 12.8)	3.3% (-0.4 to 7.7)	4.8% (1.6 to 8.4)	2.0% (1.7 to 2.6)	5.4% (3.6 to 7.3)	7.4% (6.0 to 9.0)
**South Asia**	56.6% (42.3 to 67.7)	2.6% (0.7 to 5.1)	24.4% (15.8 to 34.4)	11.4% (8.0 to 14.9)	10.9% (-1.5 to 24.0)	4.4% (1.0 to 8.3)	2.2% (0.9 to 3.7)	3.9% (2.7 to 5.3)	8.6% (6.8 to 10.6)
**Southeast Asia**	63.2% (48.7 to 73.6)	1.0% (0.7 to 1.5)	12.7% (5.1 to 23.5)	12.4% (9.0 to 15.9)	6.1% (-0.8 to 13.8)	5.5% (1.5 to 10.0)	0.6% (0.4 to 0.8)	4.5% (3.0 to 6.0)	11.5% (9.3 to 13.9)
**Southern Latin America**	59.4% (44.9 to 70.6)	0.1% (-0.1 to 0.5)	0.3% (0.0 to 3.1)	7.4% (4.8 to 10.0)	3.8% (-0.5 to 8.4)	2.9% (0.3 to 5.9)	5.9% (5.3 to 6.5)	3.3% (2.2 to 4.5)	8.2% (6.8 to 9.6)
**Southern Sub-Saharan Africa**	63.7% (48.4 to 74.5)	0.2% (-0.2 to 0.7)	9.4% (5.5 to 16.5)	12.9% (9.3 to 16.4)	5.7% (-0.7 to 12.6)	5.7% (1.0 to 10.9)	5.2% (4.7 to 5.8)	3.4% (2.3 to 4.5)	5.8% (4.7 to 6.9)
**Tropical Latin America**	57.3% (42.7 to 68.7)	0.4% (0.2 to 0.7)	2.1% (0.4 to 5.9)	10.0% (7.2 to 12.9)	7.1% (-0.9 to 16.1)	5.8% (1.2 to 11.2)	2.1% (1.7 to 2.6)	2.7% (1.8 to 3.7)	9.4% (7.6 to 11.5)
**Western Europe**	55.8% (41.6 to 66.5)	0.0% (-0.1 to 0.3)	0.0% (0.0 to 0.0)	8.3% (5.5 to 11.2)	4.1% (-0.5 to 9.4)	4.4% (-0.6 to 9.7)	6.4% (5.6 to 7.8)	1.9% (1.3 to 2.5)	9.7% (8.1 to 11.6)
**Western Sub-Saharan Africa**	62.3% (47.5 to 73.2)	2.6% (1.9 to 3.5)	30.9% (21.6 to 40.3)	11.5% (8.4 to 14.6)	7.7% (-1.0 to 17.2)	3.4% (0.9 to 6.5)	0.3% (0.1 to 0.6)	1.7% (1.1 to 2.3)	3.4% (2.7 to 4.3)
**Countries in alphabetical order**									
**Afghanistan**	53.7% (38.3 to 67.0)	1.0% (-0.3 to 2.9)	39.3% (32.4 to 46.5)	10.7% (7.5 to 14.0)	14.5% (-2.1 to 31.1)	7.0% (2.8 to 11.7)	7.4% (6.3 to 8.3)	3.8% (2.5 to 5.1)	5.7% (4.4 to 7.3)
**Albania**	63.4% (48.5 to 75.1)	0.1% (-0.3 to 0.5)	4.4% (0.3 to 16.9)	7.9% (5.2 to 10.8)	5.4% (-0.7 to 11.9)	3.2% (-0.8 to 7.7)	7.7% (6.8 to 8.9)	4.3% (2.8 to 5.9)	15.4% (12.3 to 18.8)
**Algeria**	56.2% (41.4 to 68.8)	1.3% (0.0 to 3.2)	0.0% (0.0 to 0.2)	9.9% (6.5 to 13.3)	8.1% (-1.1 to 17.8)	5.7% (1.0 to 11.3)	6.3% (5.5 to 7.0)	5.1% (3.4 to 6.8)	9.1% (7.1 to 11.8)
**American Samoa**	58.4% (42.9 to 70.9)	0.1% (0.0 to 0.1)	1.1% (0.0 to 7.2)	11.3% (8.2 to 14.4)	1.6% (-0.2 to 3.6)	8.1% (3.2 to 13.9)	0.0% (-0.1 to 0.2)	5.2% (3.5 to 7.0)	9.0% (7.3 to 10.9)
**Andorra**	57.7% (42.7 to 69.5)	0.0% (0.0 to 0.0)	0.0% (0.0 to 0.0)	8.5% (5.6 to 11.6)	3.0% (-0.4 to 7.0)	4.2% (-0.7 to 9.5)	8.1% (6.8 to 9.3)	1.9% (1.3 to 2.6)	9.6% (7.5 to 12.0)
**Angola**	60.3% (45.6 to 72.6)	0.2% (-0.2 to 0.7)	15.0% (4.9 to 27.7)	12.3% (8.7 to 15.9)	7.1% (-1.0 to 16.2)	3.3% (0.5 to 6.7)	1.4% (1.2 to 1.8)	1.5% (1.0 to 2.1)	5.2% (4.0 to 6.6)
**Antigua and Barbuda**	54.8% (39.1 to 67.1)	0.3% (0.2 to 0.5)	0.1% (0.0 to 0.5)	8.8% (6.0 to 11.7)	5.5% (-0.7 to 12.5)	4.2% (-0.1 to 9.2)	0.0% (-0.1 to 0.1)	1.8% (1.2 to 2.5)	4.8% (3.7 to 6.1)
**Argentina**	58.0% (43.1 to 69.4)	0.2% (-0.2 to 0.7)	0.2% (0.0 to 1.6)	7.2% (4.8 to 9.8)	4.1% (-0.5 to 9.0)	2.8% (0.2 to 6.0)	5.2% (4.6 to 5.7)	3.1% (2.1 to 4.1)	9.0% (7.4 to 10.7)

**Armenia**	61.8% (45.9 to 73.3)	0.1% (-0.6 to 0.9)	1.0% (0.1 to 4.0)	12.5% (8.9 to 16.1)	4.5% (-0.6 to 10.2)	3.4% (0.3 to 6.8)	7.5% (6.3 to 8.5)	5.0% (3.4 to 6.6)	15.2% (13.0 to 17.5)
**Australia**	51.8% (37.4 to 64.0)	0.1% (-0.1 to 0.4)	0.0% (0.0 to 0.0)	8.3% (5.2 to 11.8)	5.8% (-0.8 to 13.1)	5.4% (0.2 to 11.5)	4.2% (3.5 to 5.1)	1.6% (1.0 to 2.1)	6.4% (5.2 to 8.0)
**Austria**	58.0% (43.0 to 70.1)	0.0% (-0.2 to 0.2)	0.0% (0.0 to 0.0)	8.7% (5.8 to 11.7)	3.3% (-0.4 to 7.6)	4.1% (-0.4 to 9.0)	5.7% (4.8 to 6.7)	2.2% (1.5 to 3.0)	11.5% (9.5 to 13.6)
**Azerbaijan**	62.5% (46.8 to 74.2)	0.2% (-0.5 to 1.0)	0.7% (0.0 to 4.8)	13.0% (9.4 to 16.6)	4.1% (-0.5 to 9.1)	3.5% (0.6 to 7.4)	7.2% (5.9 to 8.2)	5.4% (3.6 to 7.2)	11.8% (9.2 to 14.9)
**Bahamas**	55.5% (40.6 to 69.0)	0.7% (-0.2 to 1.8)	0.0% (0.0 to 0.3)	8.5% (5.9 to 11.3)	3.6% (-0.5 to 8.6)	4.7% (0.6 to 9.5)	0.4% (0.1 to 0.7)	1.9% (1.2 to 2.5)	5.0% (3.8 to 6.5)
**Bahrain**	57.5% (43.4 to 69.7)	6.2% (2.1 to 11.0)	0.0% (0.0 to 0.0)	10.2% (6.8 to 13.9)	5.6% (-0.7 to 12.6)	5.5% (0.1 to 11.6)	2.3% (0.7 to 4.1)	3.7% (2.4 to 5.0)	8.4% (6.6 to 10.7)
**Bangladesh**	57.3% (43.4 to 69.3)	2.2% (0.2 to 4.7)	38.6% (30.1 to 46.8)	10.2% (6.9 to 13.6)	11.8% (-1.7 to 25.5)	3.1% (0.3 to 6.2)	1.7% (0.6 to 3.0)	4.8% (3.3 to 6.4)	10.4% (8.0 to 13.2)
**Barbados**	59.7% (44.4 to 71.5)	0.4% (0.2 to 0.6)	0.0% (0.0 to 0.1)	8.5% (5.8 to 11.3)	4.7% (-0.6 to 10.8)	6.1% (1.1 to 12.0)	0.0% (0.0 to 0.0)	1.0% (0.7 to 1.4)	3.7% (2.8 to 4.7)
**Belarus**	65.9% (50.5 to 77.6)	-0.2% (-0.6 to 0.0)	0.1% (0.0 to 0.6)	11.5% (8.2 to 14.9)	3.6% (-0.4 to 8.3)	3.8% (0.8 to 7.5)	8.9% (6.9 to 10.3)	3.1% (2.1 to 4.2)	14.3% (12.3 to 16.6)
**Belgium**	60.9% (45.5 to 72.7)	0.0% (0.0 to 0.1)	0.0% (0.0 to 0.0)	8.6% (5.8 to 11.5)	7.0% (-0.9 to 15.7)	4.4% (-0.6 to 9.6)	6.4% (5.5 to 8.4)	1.6% (1.1 to 2.2)	10.5% (8.5 to 12.6)
**Belize**	54.5% (39.5 to 67.4)	1.0% (0.6 to 1.5)	4.4% (0.7 to 12.2)	8.8% (6.2 to 11.5)	5.7% (-0.7 to 12.8)	4.3% (0.3 to 8.5)	0.3% (0.1 to 0.4)	2.0% (1.3 to 2.8)	6.2% (4.9 to 7.8)
**Benin**	61.1% (44.9 to 73.0)	2.6% (1.5 to 3.4)	40.1% (32.2 to 47.8)	10.7% (7.7 to 13.9)	7.7% (-1.1 to 16.6)	1.8% (0.2 to 3.8)	0.1% (0.0 to 0.2)	1.3% (0.8 to 1.7)	2.7% (2.1 to 3.6)
**Bermuda**	54.3% (39.4 to 66.9)	0.4% (-0.3 to 1.3)	0.0% (0.0 to 0.0)	8.0% (5.5 to 10.7)	3.2% (-0.4 to 7.2)	4.2% (0.3 to 8.9)	1.9% (1.5 to 2.3)	2.2% (1.4 to 3.0)	7.2% (5.7 to 9.1)
**Bhutan**	54.7% (39.1 to 66.8)	0.1% (0.0 to 0.2)	6.4% (1.2 to 17.0)	11.2% (7.8 to 14.6)	13.0% (-1.8 to 28.5)	8.9% (2.8 to 15.5)	7.5% (6.8 to 8.7)	2.8% (1.8 to 3.8)	5.3% (3.9 to 7.1)
**Bolivia (Plurinational State of)**	47.2% (33.5 to 60.0)	0.3% (-0.1 to 0.8)	8.4% (2.5 to 17.3)	7.8% (5.4 to 10.3)	8.3% (-1.1 to 18.7)	3.5% (0.2 to 7.2)	6.4% (5.8 to 7.5)	1.8% (1.0 to 2.5)	4.5% (3.6 to 5.8)
**Bosnia and Herzegovina**	63.7% (47.7 to 76.2)	0.1% (-0.3 to 0.8)	6.9% (0.5 to 24.7)	8.2% (5.5 to 10.8)	6.3% (-0.8 to 13.9)	3.8% (0.4 to 7.9)	8.3% (7.4 to 10.2)	4.1% (2.7 to 5.6)	14.4% (11.9 to 16.9)
**Botswana**	61.6% (45.8 to 73.8)	0.6% (-0.4 to 2.0)	3.7% (0.1 to 22.0)	12.6% (9.0 to 16.1)	7.9% (-1.1 to 17.2)	5.8% (1.4 to 11.2)	3.0% (2.4 to 3.6)	3.4% (2.2 to 4.6)	8.4% (6.5 to 10.3)
**Brazil**	57.3% (42.7 to 68.8)	0.3% (0.2 to 0.7)	1.9% (0.4 to 5.4)	10.0% (7.2 to 12.9)	7.1% (-0.9 to 16.1)	5.9% (1.2 to 11.3)	2.1% (1.7 to 2.6)	2.6% (1.8 to 3.6)	9.3% (7.6 to 11.4)
**Brunei Darussalam**	54.2% (39.0 to 66.7)	0.5% (0.3 to 0.8)	0.0% (0.0 to 0.0)	11.4% (7.4 to 15.4)	4.6% (-0.6 to 10.6)	4.7% (0.4 to 9.7)	0.0% (0.0 to 0.1)	2.1% (1.4 to 2.8)	8.3% (6.6 to 10.5)
**Bulgaria**	62.7% (48.4 to 75.0)	0.2% (-0.4 to 1.2)	1.1% (0.0 to 9.0)	8.3% (5.6 to 11.0)	4.0% (-0.5 to 9.3)	3.4% (0.1 to 7.2)	8.6% (7.7 to 10.8)	3.4% (2.2 to 4.6)	10.9% (9.2 to 12.7)
**Burkina Faso**	57.2% (41.2 to 70.1)	5.7% (4.2 to 7.7)	40.3% (32.7 to 47.9)	10.4% (7.6 to 13.3)	12.1% (-1.6 to 26.5)	2.7% (0.6 to 5.2)	0.2% (0.1 to 0.5)	2.4% (1.6 to 3.4)	3.1% (2.3 to 4.1)

**Burundi**	59.2%<br>(43.3 to 70.7)	0.0%<br>(0.0 to 0.0)	46.8%<br>(39.0 to 54.3)	8.3%<br>(5.5 to 11.1)	9.2%<br>(-1.3 to 20.3)	2.3%<br>(0.3 to 4.3)	2.4%<br>(1.8 to 3.5)	1.0%<br>(0.6 to 1.4)	2.7%<br>(2.0 to 3.7)
**CV¥te d'Ivoire**	62.9%<br>(49.2 to 74.4)	0.8%<br>(0.5 to 1.0)	32.6%<br>(22.9 to 41.9)	10.9%<br>(7.9 to 14.0)	8.3%<br>(-1.1 to 18.4)	3.4%<br>(1.0 to 6.2)	0.1%<br>(0.0 to 0.2)	2.9%<br>(1.9 to 4.0)	5.1%<br>(3.9 to 6.5)
**Cabo Verde**	64.2%<br>(48.8 to 76.0)	0.1%<br>(-0.1 to 0.2)	8.2%<br>(2.3 to 17.8)	10.6%<br>(7.7 to 13.7)	6.6%<br>(-0.9 to 14.7)	2.2%<br>(0.2 to 4.6)	2.2%<br>(1.8 to 3.1)	2.0%<br>(1.4 to 2.8)	3.4%<br>(2.7 to 4.3)
**Cambodia**	50.7%<br>(36.6 to 64.5)	2.2%<br>(1.9 to 2.5)	37.8%<br>(28.9 to 46.0)	11.7%<br>(8.3 to 15.2)	7.5%<br>(-1.0 to 16.5)	1.7%<br>(-0.3 to 4.1)	0.4%<br>(0.1 to 1.0)	4.5%<br>(3.0 to 6.2)	12.7%<br>(9.9 to 16.0)
**Cameroon**	64.6%<br>(49.6 to 75.4)	1.6%<br>(1.1 to 2.4)	33.8%<br>(25.2 to 42.3)	13.4%<br>(10.0 to 16.8)	9.0%<br>(-1.2 to 19.6)	3.4%<br>(0.8 to 6.6)	0.8%<br>(0.6 to 1.2)	1.6%<br>(1.0 to 2.2)	3.8%<br>(2.9 to 4.8)
**Canada**	48.8%<br>(35.2 to 60.1)	-0.1%<br>(-0.2 to 0.0)	0.0%<br>(0.0 to 0.0)	8.3%<br>(5.4 to 11.3)	2.8%<br>(-0.4 to 6.5)	2.5%<br>(-0.4 to 6.2)	5.3%<br>(4.3 to 6.2)	2.0%<br>(1.4 to 2.7)	10.4%<br>(8.5 to 12.6)
**Central African Republic**	58.8%<br>(42.4 to 72.2)	1.0%<br>(0.2 to 1.6)	46.1%<br>(38.7 to 53.4)	12.5%<br>(9.1 to 16.1)	9.1%<br>(-1.2 to 20.3)	2.8%<br>(0.5 to 5.6)	0.1%<br>(0.1 to 0.3)	1.6%<br>(1.1 to 2.3)	3.2%<br>(2.3 to 4.3)
**Chad**	55.7%<br>(40.6 to 68.2)	5.1%<br>(3.4 to 7.7)	39.7%<br>(31.4 to 47.3)	10.5%<br>(7.7 to 13.5)	12.7%<br>(-1.8 to 27.6)	3.0%<br>(0.9 to 5.4)	0.6%<br>(0.1 to 1.3)	1.7%<br>(1.2 to 2.4)	4.4%<br>(3.1 to 5.9)
**Chile**	63.3%<br>(48.7 to 74.6)	0.0%<br>(0.0 to 0.0)	0.6%<br>(0.0 to 6.8)	7.7%<br>(4.9 to 10.4)	2.5%<br>(-0.3 to 5.7)	3.0%<br>(0.1 to 6.1)	7.2%<br>(6.5 to 8.2)	4.2%<br>(2.7 to 5.8)	6.3%<br>(5.2 to 7.6)
**China**	56.7%<br>(42.2 to 68.7)	0.4%<br>(-0.3 to 1.7)	5.1%<br>(0.7 to 17.4)	7.8%<br>(5.4 to 10.4)	8.5%<br>(-1.1 to 18.7)	4.3%<br>(0.5 to 8.9)	6.6%<br>(5.9 to 7.4)	4.9%<br>(3.3 to 6.6)	15.7%<br>(13.0 to 18.7)
**Colombia**	61.8%<br>(47.2 to 72.9)	0.3%<br>(0.3 to 0.3)	1.3%<br>(0.0 to 6.9)	11.2%<br>(7.8 to 14.4)	7.0%<br>(-0.9 to 15.5)	5.7%<br>(2.1 to 10.1)	3.6%<br>(2.9 to 4.4)	2.3%<br>(1.5 to 3.2)	4.6%<br>(3.7 to 5.7)
**Comoros**	59.7%<br>(44.3 to 72.1)	0.0%<br>(0.0 to 0.1)	41.9%<br>(34.4 to 49.3)	8.2%<br>(5.5 to 11.1)	7.3%<br>(-1.0 to 17.0)	2.3%<br>(0.6 to 4.5)	0.4%<br>(0.1 to 0.7)	3.2%<br>(2.0 to 4.5)	4.6%<br>(3.3 to 6.2)
**Congo**	61.8%<br>(45.9 to 73.5)	0.2%<br>(0.0 to 0.4)	22.9%<br>(10.8 to 34.7)	12.6%<br>(9.1 to 16.3)	5.2%<br>(-0.7 to 11.8)	2.9%<br>(0.8 to 5.6)	0.2%<br>(0.1 to 0.3)	2.0%<br>(1.4 to 2.8)	4.4%<br>(3.3 to 5.9)
**Cook Islands**	56.2%<br>(42.3 to 68.4)	0.2%<br>(-0.1 to 0.4)	0.1%<br>(0.0 to 1.4)	10.0%<br>(7.2 to 12.9)	1.6%<br>(-0.2 to 3.5)	4.9%<br>(1.7 to 8.6)	0.0%<br>(0.0 to 0.0)	4.6%<br>(3.1 to 6.2)	8.9%<br>(7.2 to 11.0)
**Costa Rica**	61.1%<br>(45.4 to 72.9)	0.0%<br>(0.0 to 0.0)	0.7%<br>(0.0 to 4.7)	13.3%<br>(9.2 to 17.5)	7.6%<br>(-1.0 to 17.2)	3.2%<br>(0.1 to 6.7)	2.1%<br>(1.4 to 2.7)	2.2%<br>(1.5 to 3.0)	6.1%<br>(4.9 to 7.4)
**Croatia**	63.1%<br>(48.5 to 74.8)	0.2%<br>(-0.5 to 1.2)	0.2%<br>(0.0 to 2.2)	8.1%<br>(5.2 to 11.0)	4.2%<br>(-0.5 to 9.4)	3.6%<br>(-0.9 to 8.6)	7.5%<br>(6.5 to 8.9)	4.4%<br>(2.9 to 6.0)	12.4%<br>(10.1 to 15.2)
**Cuba**	50.0%<br>(35.1 to 62.1)	0.6%<br>(0.2 to 1.1)	0.6%<br>(0.1 to 2.1)	8.2%<br>(5.7 to 10.7)	9.0%<br>(-1.2 to 20.1)	4.3%<br>(0.8 to 8.3)	0.4%<br>(0.1 to 0.7)	3.3%<br>(2.2 to 4.5)	11.0%<br>(9.0 to 13.2)
**Cyprus**	56.1%<br>(40.6 to 69.3)	1.5%<br>(-0.4 to 4.7)	0.0%<br>(0.0 to 0.0)	9.5%<br>(5.8 to 13.3)	5.8%<br>(-0.8 to 12.7)	3.5%<br>(-2.9 to 9.8)	4.6%<br>(3.5 to 5.8)	2.4%<br>(1.6 to 3.2)	8.1%<br>(6.2 to 10.1)
**Czechia**	60.6%<br>(44.7 to 73.1)	0.0%<br>(-0.2 to 0.1)	0.0%<br>(0.0 to 0.2)	7.8%<br>(5.2 to 10.5)	3.0%<br>(-0.4 to 6.7)	4.3%<br>(0.0 to 9.1)	7.7%<br>(6.6 to 9.3)	3.1%<br>(2.1 to 4.2)	11.9%<br>(10.0 to 14.0)
**Democratic People's Republic of Korea**	54.3%<br>(39.2 to 67.0)	0.2%<br>(-0.4 to 1.4)	41.7%<br>(34.3 to 49.0)	8.6%<br>(6.3 to 10.9)	7.3%<br>(-0.9 to 16.2)	4.6%<br>(1.4 to 8.2)	7.3%<br>(6.5 to 8.7)	5.2%<br>(3.5 to 6.9)	12.3%<br>(9.9 to 14.8)
**Democratic Republic of the Congo**	57.0%<br>(42.1 to 69.7)	0.2%<br>(-0.1 to 0.6)	45.1%<br>(37.8 to 52.4)	12.6%<br>(9.0 to 16.4)	7.5%<br>(-1.0 to 16.8)	4.1%<br>(0.4 to 8.0)	1.0%<br>(0.8 to 1.2)	1.6%<br>(1.0 to 2.3)	2.1%<br>(1.5 to 3.0)

**Denmark**	60.2% (45.7 to 72.3)	0.0% (0.0 to 0.0)	0.0% (0.0 to 0.0)	8.6% (5.7 to 11.6)	3.7% (-0.5 to 8.2)	3.9% (-0.6 to 8.9)	7.6% (6.6 to 10.1)	1.5% (1.0 to 2.0)	13.3% (10.9 to 16.0)
**Djibouti**	60.3% (46.0 to 72.1)	6.3% (3.7 to 10.0)	12.3% (6.2 to 19.5)	8.1% (5.4 to 11.0)	8.0% (-1.1 to 17.3)	2.3% (0.5 to 4.6)	1.6% (0.5 to 3.4)	4.1% (2.6 to 5.7)	8.3% (6.3 to 10.7)
**Dominica**	55.1% (40.0 to 67.8)	0.2% (0.1 to 0.4)	1.9% (0.2 to 7.0)	9.0% (6.2 to 12.0)	5.3% (-0.7 to 11.8)	3.2% (-0.7 to 7.5)	0.0% (-0.1 to 0.2)	1.9% (1.2 to 2.6)	3.9% (3.0 to 5.1)
**Dominican Republic**	54.4% (38.6 to 67.2)	0.2% (0.1 to 0.4)	0.9% (0.0 to 6.1)	8.7% (6.2 to 11.3)	11.0% (-1.5 to 23.7)	4.9% (1.4 to 9.1)	0.6% (0.4 to 0.8)	2.2% (1.5 to 3.0)	10.7% (8.3 to 13.4)
**Ecuador**	46.3% (31.8 to 58.9)	0.0% (0.0 to 0.1)	1.3% (0.1 to 5.1)	7.9% (5.5 to 10.4)	5.3% (-0.7 to 11.8)	2.9% (0.5 to 5.4)	3.5% (3.0 to 4.3)	1.6% (1.0 to 2.3)	4.7% (3.8 to 5.8)
**Egypt**	58.5% (42.4 to 70.7)	3.4% (-0.8 to 9.4)	0.1% (0.0 to 0.3)	10.7% (7.5 to 14.1)	12.6% (-1.8 to 28.1)	5.5% (1.7 to 10.2)	2.9% (1.7 to 3.8)	4.2% (2.8 to 5.7)	11.3% (9.2 to 13.6)
**El Salvador**	56.9% (41.8 to 69.5)	0.5% (0.4 to 0.7)	5.4% (1.2 to 13.2)	10.8% (7.9 to 13.7)	12.4% (-1.7 to 27.1)	3.5% (0.8 to 6.7)	0.1% (0.0 to 0.1)	2.0% (1.3 to 2.6)	4.5% (3.5 to 5.6)
**Equatorial Guinea**	62.4% (46.1 to 74.2)	0.0% (-0.1 to 0.1)	0.7% (0.0 to 7.4)	12.6% (9.1 to 16.4)	5.9% (-0.8 to 13.4)	3.3% (0.6 to 6.4)	0.4% (0.2 to 0.5)	3.3% (2.1 to 4.6)	3.4% (2.4 to 4.6)
**Eritrea**	56.4% (41.0 to 69.0)	2.4% (1.3 to 3.9)	37.6% (30.6 to 45.0)	8.1% (5.5 to 10.8)	7.7% (-1.0 to 17.2)	1.3% (0.3 to 2.7)	1.8% (1.5 to 2.3)	1.8% (1.2 to 2.5)	2.1% (1.5 to 2.9)
**Estonia**	64.3% (48.2 to 75.7)	-0.1% (-0.2 to 0.0)	0.2% (0.0 to 2.2)	11.5% (8.1 to 14.9)	2.7% (-0.3 to 6.3)	3.4% (0.2 to 7.2)	7.6% (6.5 to 8.8)	2.5% (1.6 to 3.4)	12.7% (10.8 to 14.9)
**Eswatini**	62.9% (46.9 to 75.3)	0.2% (-0.2 to 0.7)	15.9% (3.1 to 34.4)	12.8% (9.3 to 16.4)	7.4% (-1.0 to 16.2)	4.2% (0.6 to 8.3)	4.9% (4.3 to 5.6)	1.9% (1.2 to 2.8)	3.9% (2.8 to 5.2)
**Ethiopia**	46.7% (33.4 to 58.7)	0.2% (0.1 to 0.4)	41.7% (34.6 to 48.3)	7.9% (5.3 to 10.7)	10.7% (-1.5 to 23.2)	2.6% (0.7 to 4.9)	4.2% (3.7 to 5.0)	1.2% (0.8 to 1.7)	1.9% (1.4 to 2.4)
**Fiji**	59.7% (44.3 to 71.7)	0.1% (0.0 to 0.2)	7.8% (0.9 to 20.8)	10.8% (7.9 to 13.9)	2.2% (-0.3 to 5.1)	4.8% (1.7 to 8.9)	0.4% (-0.2 to 0.7)	4.3% (2.8 to 5.9)	7.3% (6.0 to 8.8)
**Finland**	59.9% (45.0 to 71.7)	-0.1% (-0.3 to 0.0)	0.0% (0.0 to 0.0)	8.0% (5.2 to 10.8)	1.9% (-0.2 to 4.4)	4.1% (-0.5 to 9.1)	7.1% (5.6 to 8.2)	1.2% (0.8 to 1.6)	7.7% (6.3 to 9.4)
**France**	57.4% (42.9 to 69.4)	0.0% (-0.1 to 0.2)	0.0% (0.0 to 0.0)	7.6% (5.0 to 10.4)	4.4% (-0.6 to 10.1)	4.6% (-0.3 to 9.9)	6.1% (5.2 to 7.5)	1.8% (1.2 to 2.5)	9.9% (8.1 to 12.0)
**Gabon**	61.1% (46.1 to 73.5)	0.1% (0.0 to 0.1)	1.1% (0.0 to 7.4)	12.5% (8.8 to 16.1)	4.7% (-0.6 to 10.9)	1.8% (0.0 to 3.8)	0.2% (0.1 to 0.4)	2.2% (1.4 to 3.1)	3.4% (2.5 to 4.5)
**Gambia**	63.7% (48.3 to 75.0)	2.0% (1.2 to 3.0)	39.6% (31.7 to 47.2)	10.8% (7.9 to 13.8)	9.3% (-1.3 to 20.4)	2.6% (0.5 to 4.9)	0.2% (0.0 to 0.4)	3.3% (2.2 to 4.5)	5.2% (4.0 to 6.6)
**Georgia**	64.9% (49.0 to 76.7)	0.2% (-0.3 to 1.1)	5.0% (0.5 to 16.5)	12.6% (8.9 to 16.3)	6.3% (-0.8 to 14.0)	3.5% (-0.1 to 7.7)	8.3% (7.5 to 10.4)	4.9% (3.3 to 6.6)	13.1% (11.2 to 15.4)
**Germany**	61.3% (46.3 to 73.2)	0.0% (-0.1 to 0.1)	0.0% (0.0 to 0.0)	8.5% (5.6 to 11.5)	2.6% (-0.3 to 6.0)	3.8% (0.0 to 8.4)	6.2% (5.3 to 8.1)	1.8% (1.2 to 2.4)	11.1% (9.3 to 13.3)
**Ghana**	62.7% (48.5 to 73.7)	2.0% (1.7 to 2.4)	27.6% (15.4 to 38.9)	8.6% (5.9 to 11.4)	5.7% (-0.7 to 12.8)	3.6% (1.0 to 6.7)	0.1% (0.0 to 0.2)	1.2% (0.8 to 1.7)	4.5% (3.4 to 5.8)
**Greece**	51.0% (36.2 to 62.9)	0.3% (-0.2 to 1.0)	0.0% (0.0 to 0.2)	8.7% (5.6 to 12.1)	5.5% (-0.7 to 12.4)	3.2% (-1.6 to 8.5)	6.4% (5.8 to 7.1)	2.7% (1.8 to 3.7)	11.6% (9.3 to 14.1)

**Greenland**	50.2%<br>(35.3 to 63.4)	-0.2%<br>(-0.4 to 0.0)	0.0%<br>(0.0 to 0.0)	9.5%<br>(6.2 to 13.0)	2.8%<br>(-0.3 to 6.4)	3.2%<br>(-0.6 to 7.1)	6.9%<br>(4.1 to 10.7)	2.9%<br>(1.9 to 3.9)	13.0%<br>(10.4 to 16.2)
**Grenada**	55.4%<br>(40.3 to 68.5)	0.4%<br>(0.3 to 0.6)	0.3%<br>(0.0 to 1.9)	9.2%<br>(6.3 to 12.2)	7.9%<br>(-1.0 to 17.8)	4.0%<br>(0.1 to 8.7)	0.0%<br>(0.0 to 0.0)	1.8%<br>(1.2 to 2.5)	3.9%<br>(3.1 to 4.9)
**Guam**	55.9%<br>(39.6 to 67.7)	0.5%<br>(0.2 to 0.8)	0.1%<br>(0.0 to 0.3)	10.3%<br>(7.7 to 13.0)	1.4%<br>(-0.2 to 3.2)	3.9%<br>(1.7 to 6.7)	0.0%<br>(0.0 to 0.0)	4.4%<br>(3.0 to 6.0)	10.7%<br>(8.7 to 12.9)
**Guatemala**	51.9%<br>(37.5 to 63.4)	0.2%<br>(0.1 to 0.3)	19.1%<br>(5.4 to 34.7)	12.2%<br>(8.7 to 15.9)	14.1%<br>(-2.0 to 29.6)	2.0%<br>(-0.5 to 4.7)	2.8%<br>(1.6 to 3.7)	2.0%<br>(1.2 to 2.8)	4.2%<br>(3.3 to 5.4)
**Guinea**	56.4%<br>(42.3 to 68.4)	1.1%<br>(0.6 to 1.9)	40.9%<br>(33.2 to 48.5)	10.7%<br>(7.9 to 13.8)	10.6%<br>(-1.5 to 23.1)	1.9%<br>(0.3 to 3.9)	0.2%<br>(0.1 to 0.4)	2.5%<br>(1.7 to 3.4)	5.1%<br>(3.8 to 6.7)
**Guinea-Bissau**	61.0%<br>(45.3 to 73.0)	2.1%<br>(1.6 to 2.6)	41.1%<br>(33.5 to 48.7)	11.0%<br>(8.0 to 14.0)	9.5%<br>(-1.3 to 21.2)	3.6%<br>(1.0 to 6.8)	0.1%<br>(0.0 to 0.3)	1.8%<br>(1.2 to 2.5)	3.4%<br>(2.5 to 4.4)
**Guyana**	55.5%<br>(39.8 to 68.0)	0.3%<br>(0.0 to 0.6)	2.5%<br>(0.5 to 6.9)	9.2%<br>(6.6 to 11.9)	8.7%<br>(-1.2 to 18.9)	4.6%<br>(0.8 to 8.9)	0.1%<br>(0.0 to 0.2)	2.2%<br>(1.5 to 3.0)	5.2%<br>(4.1 to 6.6)
**Haiti**	58.8%<br>(43.6 to 71.1)	0.4%<br>(0.3 to 0.5)	44.0%<br>(36.2 to 51.6)	9.1%<br>(6.5 to 12.1)	13.6%<br>(-1.9 to 29.6)	4.6%<br>(1.0 to 9.0)	0.2%<br>(0.1 to 0.4)	1.4%<br>(0.9 to 1.9)	2.6%<br>(2.0 to 3.5)
**Honduras**	57.5%<br>(41.8 to 70.0)	0.4%<br>(0.1 to 0.8)	25.6%<br>(15.5 to 34.5)	12.4%<br>(8.9 to 16.0)	13.7%<br>(-2.0 to 29.3)	3.2%<br>(0.4 to 6.7)	1.1%<br>(0.5 to 1.6)	3.2%<br>(2.0 to 4.3)	6.7%<br>(5.1 to 8.6)
**Hungary**	69.0%<br>(53.8 to 79.7)	0.2%<br>(-0.7 to 1.4)	0.6%<br>(0.0 to 6.4)	8.0%<br>(5.5 to 10.5)	4.0%<br>(-0.5 to 9.1)	4.0%<br>(0.6 to 8.1)	7.3%<br>(6.3 to 8.7)	2.9%<br>(1.9 to 3.8)	11.8%<br>(10.0 to 13.8)
**Iceland**	55.6%<br>(41.3 to 67.8)	0.0%<br>(0.0 to 0.0)	0.0%<br>(0.0 to 0.0)	6.8%<br>(4.4 to 9.3)	3.7%<br>(-0.5 to 8.2)	4.1%<br>(-0.7 to 9.5)	8.2%<br>(6.9 to 9.4)	1.4%<br>(0.9 to 1.9)	8.9%<br>(7.1 to 11.1)
**India**	56.3%<br>(41.8 to 67.6)	2.4%<br>(0.8 to 4.4)	21.0%<br>(12.6 to 31.7)	11.6%<br>(8.2 to 15.3)	10.6%<br>(-1.4 to 23.4)	4.7%<br>(1.1 to 8.9)	1.9%<br>(0.7 to 3.3)	3.6%<br>(2.5 to 4.9)	8.1%<br>(6.4 to 10.2)
**Indonesia**	69.1%<br>(54.1 to 79.2)	0.4%<br>(0.3 to 0.5)	9.0%<br>(2.8 to 19.2)	12.8%<br>(9.2 to 16.5)	7.0%<br>(-0.9 to 15.6)	8.1%<br>(1.4 to 15.3)	0.4%<br>(0.3 to 0.6)	4.8%<br>(3.2 to 6.5)	11.3%<br>(8.6 to 14.1)
**Iran (Islamic Republic of)**	54.3%<br>(40.8 to 65.3)	1.9%<br>(-0.3 to 4.8)	0.0%<br>(0.0 to 0.2)	10.8%<br>(7.6 to 14.0)	12.6%<br>(-1.8 to 27.5)	5.7%<br>(1.8 to 10.2)	6.4%<br>(5.2 to 7.5)	2.7%<br>(1.8 to 3.7)	8.3%<br>(6.8 to 9.9)
**Iraq**	65.1%<br>(49.6 to 75.8)	9.3%<br>(0.8 to 19.0)	0.1%<br>(0.0 to 0.9)	11.1%<br>(7.5 to 14.6)	7.9%<br>(-1.0 to 17.7)	7.1%<br>(2.5 to 12.8)	5.8%<br>(2.6 to 9.8)	5.4%<br>(3.7 to 7.2)	13.1%<br>(10.7 to 15.7)
**Ireland**	58.2%<br>(43.3 to 69.6)	0.0%<br>(0.0 to 0.0)	0.0%<br>(0.0 to 0.0)	11.2%<br>(7.6 to 14.8)	4.3%<br>(-0.5 to 9.6)	4.5%<br>(-1.5 to 11.0)	6.6%<br>(5.4 to 9.2)	1.9%<br>(1.2 to 2.6)	9.1%<br>(7.2 to 11.1)
**Israel**	59.9%<br>(44.9 to 71.2)	1.0%<br>(-0.5 to 3.6)	0.0%<br>(0.0 to 0.0)	9.0%<br>(6.0 to 12.1)	3.3%<br>(-0.4 to 7.5)	4.1%<br>(-0.2 to 8.8)	2.1%<br>(1.4 to 2.7)	2.0%<br>(1.3 to 2.7)	8.7%<br>(7.1 to 10.5)
**Italy**	49.1%<br>(34.2 to 60.7)	0.1%<br>(-0.3 to 0.6)	0.0%<br>(0.0 to 0.1)	7.6%<br>(4.9 to 10.5)	4.8%<br>(-0.6 to 11.0)	4.3%<br>(-1.6 to 10.2)	7.0%<br>(6.1 to 8.0)	2.0%<br>(1.4 to 2.8)	7.8%<br>(6.3 to 9.5)
**Jamaica**	55.0%<br>(40.4 to 67.4)	0.5%<br>(0.1 to 0.9)	3.7%<br>(0.6 to 10.3)	8.5%<br>(5.8 to 11.3)	7.9%<br>(-1.0 to 18.0)	5.7%<br>(1.0 to 10.9)	0.1%<br>(0.0 to 0.2)	2.1%<br>(1.4 to 2.9)	5.9%<br>(4.7 to 7.5)
**Japan**	55.1%<br>(41.0 to 66.5)	0.1%<br>(-0.1 to 0.3)	0.0%<br>(0.0 to 0.0)	10.5%<br>(6.8 to 14.2)	2.8%<br>(-0.4 to 6.6)	5.6%<br>(1.7 to 10.0)	5.1%<br>(4.3 to 5.9)	2.3%<br>(1.5 to 3.1)	11.4%<br>(9.4 to 13.6)
**Jordan**	56.1%<br>(40.2 to 68.2)	0.9%<br>(-0.6 to 3.4)	0.0%<br>(0.0 to 0.1)	10.2%<br>(7.1 to 13.6)	6.8%<br>(-0.9 to 15.5)	5.6%<br>(1.5 to 10.7)	4.2%<br>(3.4 to 5.2)	4.8%<br>(3.3 to 6.5)	13.2%<br>(10.9 to 15.6)

**Kazakhstan**	68.3% (53.1 to 79.0)	0.2% (-0.5 to 1.2)	0.3% (0.0 to 3.1)	12.9% (9.1 to 16.6)	3.1% (-0.4 to 6.8)	3.6% (0.6 to 7.4)	8.2% (7.1 to 9.5)	3.7% (2.5 to 5.0)	8.5% (7.1 to 9.9)
**Kenya**	58.8% (44.5 to 69.9)	0.1% (0.1 to 0.2)	40.1% (32.3 to 47.4)	8.3% (5.5 to 11.1)	6.6% (-0.9 to 14.7)	3.3% (0.8 to 6.1)	3.2% (2.7 to 4.1)	1.7% (1.1 to 2.3)	3.6% (2.6 to 4.7)
**Kiribati**	49.0% (33.9 to 61.0)	0.2% (0.0 to 0.4)	29.6% (23.6 to 36.2)	10.7% (8.0 to 13.5)	3.1% (-0.4 to 7.1)	6.9% (2.8 to 11.5)	0.0% (-0.1 to 0.1)	7.8% (5.4 to 10.4)	18.9% (16.0 to 22.2)
**Kuwait**	55.4% (41.0 to 66.9)	9.5% (2.1 to 17.1)	0.0% (0.0 to 0.0)	9.9% (6.7 to 13.1)	5.4% (-0.7 to 12.5)	8.5% (3.4 to 14.3)	4.0% (1.0 to 7.4)	4.4% (3.0 to 5.9)	12.3% (10.1 to 14.9)
**Kyrgyzstan**	57.6% (41.0 to 70.7)	0.0% (-0.5 to 0.6)	16.8% (10.0 to 24.5)	12.6% (9.5 to 15.8)	4.8% (-0.6 to 11.1)	3.6% (0.9 to 6.6)	8.5% (6.9 to 9.9)	5.1% (3.5 to 6.8)	17.7% (15.2 to 20.3)
**Lao People's Democratic Republic**	57.0% (41.2 to 69.9)	0.9% (0.2 to 1.9)	31.0% (13.6 to 46.4)	12.8% (9.4 to 16.3)	8.6% (-1.2 to 18.8)	2.3% (0.5 to 4.5)	1.5% (1.0 to 2.0)	5.2% (3.5 to 7.0)	13.1% (10.5 to 15.9)
**Latvia**	65.3% (49.7 to 77.1)	-0.1% (-0.3 to 0.0)	0.5% (0.0 to 5.2)	11.6% (8.0 to 15.2)	2.8% (-0.4 to 6.5)	3.6% (0.6 to 7.2)	9.4% (7.9 to 10.6)	3.2% (2.1 to 4.4)	9.7% (8.2 to 11.4)
**Lebanon**	55.6% (40.7 to 67.1)	0.2% (-0.3 to 0.9)	0.1% (0.0 to 0.4)	10.1% (7.0 to 13.4)	5.1% (-0.7 to 11.4)	6.1% (1.8 to 11.1)	4.7% (4.0 to 5.6)	5.0% (3.3 to 6.6)	17.7% (14.6 to 21.0)
**Lesotho**	57.9% (42.0 to 70.9)	0.0% (0.0 to 0.0)	33.1% (25.7 to 40.4)	12.7% (9.1 to 16.1)	8.7% (-1.2 to 19.7)	2.2% (-0.6 to 5.6)	8.2% (7.0 to 10.4)	4.2% (2.8 to 5.6)	8.8% (6.4 to 12.1)
**Liberia**	62.6% (47.1 to 74.1)	0.2% (0.1 to 0.4)	43.1% (36.0 to 50.2)	10.8% (7.9 to 14.0)	9.4% (-1.3 to 20.9)	4.5% (1.5 to 8.2)	0.1% (0.0 to 0.2)	1.6% (1.0 to 2.2)	3.3% (2.5 to 4.2)
**Libya**	60.6% (45.3 to 71.8)	2.7% (-0.3 to 7.7)	0.1% (0.0 to 0.5)	10.5% (7.4 to 13.7)	7.1% (-1.0 to 15.9)	7.1% (2.9 to 12.2)	3.5% (2.6 to 4.4)	6.0% (4.1 to 7.9)	8.8% (6.8 to 11.2)
**Lithuania**	65.9% (50.3 to 77.0)	-0.1% (-0.4 to 0.0)	0.1% (0.0 to 0.8)	11.6% (8.3 to 14.9)	2.8% (-0.4 to 6.5)	4.7% (0.9 to 9.3)	8.9% (7.3 to 10.1)	2.6% (1.7 to 3.6)	10.9% (9.2 to 13.0)
**Luxembourg**	58.7% (43.4 to 71.5)	0.0% (-0.1 to 0.1)	0.0% (0.0 to 0.0)	8.8% (5.7 to 12.0)	3.5% (-0.4 to 7.9)	3.7% (-1.1 to 9.2)	7.3% (6.4 to 9.9)	1.7% (1.1 to 2.4)	9.4% (7.4 to 12.0)
**Madagascar**	57.6% (42.5 to 69.2)	0.3% (-0.1 to 0.8)	47.5% (39.8 to 55.0)	8.0% (5.4 to 10.7)	7.3% (-1.0 to 15.9)	2.3% (0.5 to 4.4)	3.1% (2.7 to 3.7)	1.9% (1.2 to 2.7)	2.8% (2.1 to 3.6)
**Malawi**	62.7% (46.5 to 74.9)	0.6% (-0.4 to 2.1)	46.5% (39.0 to 53.8)	8.5% (5.7 to 11.6)	8.5% (-1.2 to 19.2)	2.1% (0.5 to 4.1)	2.4% (1.9 to 2.9)	2.1% (1.4 to 2.8)	7.0% (5.0 to 9.5)
**Malaysia**	67.9% (52.1 to 78.6)	0.7% (0.5 to 0.9)	0.0% (0.0 to 0.3)	13.7% (10.1 to 17.4)	5.1% (-0.6 to 11.6)	5.7% (1.9 to 10.0)	0.1% (0.0 to 0.1)	4.7% (3.3 to 6.4)	8.8% (7.3 to 10.5)
**Maldives**	59.8% (45.1 to 71.8)	0.8% (0.5 to 1.2)	1.0% (0.0 to 6.9)	12.1% (8.6 to 15.7)	6.2% (-0.8 to 13.7)	8.2% (2.0 to 15.1)	0.0% (-0.1 to 0.0)	5.2% (3.5 to 6.9)	12.7% (10.4 to 15.5)
**Mali**	58.1% (42.8 to 69.9)	6.8% (4.5 to 10.0)	39.5% (31.4 to 47.1)	10.5% (7.6 to 13.4)	11.9% (-1.6 to 26.1)	3.9% (1.1 to 7.1)	0.3% (0.1 to 0.7)	3.2% (2.1 to 4.4)	5.0% (3.6 to 6.5)
**Malta**	57.0% (41.7 to 69.6)	0.5% (-0.5 to 2.3)	0.0% (0.0 to 0.0)	8.5% (5.5 to 11.6)	9.0% (-1.2 to 19.7)	5.0% (-0.6 to 11.0)	3.7% (2.1 to 5.0)	1.6% (1.1 to 2.2)	7.7% (6.1 to 9.3)
**Marshall Islands**	49.7% (35.3 to 62.7)	0.8% (0.4 to 1.3)	18.6% (12.4 to 25.9)	10.7% (7.8 to 13.7)	2.9% (-0.4 to 6.7)	9.4% (3.9 to 15.3)	0.0% (0.0 to 0.0)	4.7% (3.1 to 6.4)	7.2% (5.5 to 9.1)
**Mauritania**	62.0% (45.8 to 73.3)	9.3% (4.5 to 15.6)	23.0% (14.1 to 32.2)	10.4% (7.5 to 13.5)	6.1% (-0.8 to 13.4)	6.1% (2.0 to 10.8)	0.7% (0.2 to 1.5)	3.8% (2.5 to 5.2)	3.4% (2.6 to 4.5)

**Mauritius**	61.0% (46.0 to 73.3)	0.1% (-0.1 to 0.4)	0.1% (0.0 to 0.6)	13.8% (10.3 to 17.3)	3.8% (-0.5 to 8.8)	3.8% (1.0 to 7.1)	1.0% (0.3 to 1.7)	5.3% (3.6 to 7.1)	11.5% (9.7 to 13.6)
**Mexico**	54.6% (39.6 to 66.0)	0.6% (0.0 to 1.3)	1.9% (0.1 to 8.2)	11.6% (8.3 to 14.8)	8.7% (-1.2 to 19.1)	3.4% (0.7 to 6.8)	4.0% (3.4 to 4.6)	2.0% (1.4 to 2.7)	5.1% (4.1 to 6.1)
**Micronesia (Federated States of)**	45.6% (32.0 to 58.8)	0.8% (0.4 to 1.3)	19.4% (12.5 to 26.8)	10.9% (8.1 to 13.9)	2.6% (-0.3 to 5.9)	9.9% (4.2 to 16.4)	0.0% (0.0 to 0.0)	6.4% (4.2 to 8.8)	11.5% (9.2 to 13.8)
**Monaco**	57.1% (43.2 to 69.3)	0.0% (-0.1 to 0.1)	0.0% (0.0 to 0.0)	8.5% (5.6 to 11.7)	3.2% (-0.4 to 7.1)	4.2% (-0.9 to 9.7)	7.0% (6.4 to 7.8)	1.6% (1.1 to 2.2)	9.5% (7.3 to 12.1)
**Mongolia**	62.8% (47.9 to 74.5)	0.0% (-0.2 to 0.0)	7.9% (0.6 to 27.4)	12.7% (9.4 to 16.1)	5.7% (-0.7 to 12.8)	2.3% (0.7 to 4.6)	7.5% (5.3 to 9.9)	4.5% (3.1 to 6.0)	14.5% (11.8 to 17.4)
**Montenegro**	62.4% (47.5 to 73.8)	0.1% (-0.2 to 0.5)	3.1% (0.0 to 21.3)	8.3% (5.3 to 11.3)	3.2% (-0.4 to 7.4)	3.4% (-0.9 to 8.2)	8.6% (7.6 to 9.8)	4.5% (3.0 to 6.0)	11.2% (9.0 to 13.8)
**Morocco**	63.8% (48.3 to 75.3)	0.6% (-0.1 to 1.9)	1.1% (0.2 to 3.4)	9.7% (6.6 to 12.8)	6.9% (-0.9 to 15.2)	6.9% (2.1 to 12.4)	6.0% (4.9 to 7.1)	4.2% (2.9 to 5.7)	4.8% (3.7 to 6.2)
**Mozambique**	62.7% (47.6 to 74.8)	0.8% (-0.4 to 2.3)	46.2% (38.8 to 53.5)	8.7% (5.9 to 11.8)	10.9% (-1.6 to 23.5)	2.4% (0.6 to 4.7)	1.4% (1.0 to 1.8)	1.8% (1.2 to 2.6)	5.6% (4.2 to 7.3)
**Myanmar**	59.5% (45.1 to 71.6)	2.2% (1.3 to 3.3)	29.4% (15.1 to 42.0)	12.3% (9.0 to 15.9)	7.7% (-1.0 to 17.1)	1.7% (0.3 to 3.4)	0.8% (0.5 to 1.2)	3.9% (2.4 to 5.4)	10.0% (8.0 to 12.4)
**Namibia**	58.2% (41.7 to 71.5)	0.5% (-0.5 to 2.0)	10.8% (1.1 to 30.9)	12.2% (8.6 to 15.8)	6.4% (-0.9 to 14.4)	4.4% (0.7 to 9.6)	2.8% (2.3 to 3.3)	2.8% (1.8 to 4.1)	7.4% (5.6 to 9.2)
**Nauru**	59.9% (44.9 to 71.7)	0.9% (0.5 to 1.3)	0.7% (0.0 to 3.4)	11.2% (8.3 to 14.2)	1.9% (-0.2 to 4.3)	5.5% (2.2 to 9.2)	0.0% (0.0 to 0.0)	6.1% (4.0 to 8.3)	11.0% (8.9 to 13.3)
**Nepal**	42.6% (28.1 to 55.6)	0.8% (-0.5 to 2.6)	33.6% (26.2 to 41.3)	13.6% (9.6 to 17.8)	14.4% (-2.1 to 31.0)	3.0% (0.7 to 6.1)	4.2% (3.5 to 4.9)	3.6% (2.3 to 5.1)	12.2% (9.8 to 15.1)
**Netherlands**	57.3% (41.9 to 69.0)	0.0% (0.0 to 0.1)	0.0% (0.0 to 0.0)	8.9% (6.0 to 12.1)	3.6% (-0.5 to 8.3)	3.0% (-1.2 to 7.2)	6.5% (5.4 to 8.8)	1.4% (1.0 to 2.0)	10.5% (8.4 to 12.9)
**New Zealand**	52.7% (38.4 to 64.5)	0.0% (0.0 to 0.0)	0.0% (0.0 to 0.0)	8.8% (5.5 to 12.2)	5.2% (-0.7 to 11.7)	4.6% (-1.4 to 10.8)	6.2% (5.1 to 7.5)	1.5% (1.0 to 2.1)	8.0% (6.3 to 10.0)
**Nicaragua**	59.0% (43.7 to 70.9)	0.7% (0.6 to 0.7)	23.1% (12.6 to 32.6)	14.7% (10.7 to 18.5)	10.2% (-1.4 to 22.5)	3.3% (0.6 to 6.6)	0.3% (0.2 to 0.4)	3.6% (2.3 to 4.9)	6.0% (4.8 to 7.5)
**Niger**	59.0% (43.9 to 70.5)	7.1% (4.5 to 10.7)	41.5% (33.3 to 49.6)	10.0% (7.2 to 13.0)	13.7% (-2.0 to 29.0)	3.2% (0.6 to 6.3)	1.2% (0.3 to 2.6)	1.7% (1.1 to 2.3)	2.3% (1.6 to 3.2)
**Nigeria**	63.3% (47.8 to 74.7)	2.0% (1.5 to 2.7)	24.3% (13.7 to 35.3)	13.1% (9.6 to 16.7)	6.2% (-0.8 to 13.6)	3.6% (0.7 to 6.9)	0.3% (0.1 to 0.6)	1.1% (0.7 to 1.5)	2.3% (1.7 to 2.9)
**Niue**	58.1% (43.0 to 69.6)	0.2% (0.1 to 0.2)	0.7% (0.0 to 4.1)	10.5% (7.5 to 13.5)	1.6% (-0.2 to 3.7)	4.6% (1.5 to 8.5)	0.4% (0.2 to 0.5)	3.9% (2.6 to 5.3)	7.5% (5.8 to 9.3)
**North Macedonia**	62.0% (46.3 to 74.5)	0.2% (-0.3 to 1.2)	2.8% (0.1 to 15.1)	9.8% (6.3 to 13.6)	4.3% (-0.5 to 9.5)	3.2% (-1.3 to 8.1)	9.1% (8.2 to 11.7)	5.7% (3.8 to 7.8)	10.6% (8.5 to 12.9)
**Northern Mariana Islands**	56.9% (42.0 to 69.4)	0.6% (0.5 to 0.7)	0.2% (0.0 to 1.9)	10.9% (7.9 to 14.0)	1.4% (-0.2 to 3.1)	4.5% (1.4 to 8.2)	0.2% (0.1 to 0.3)	4.7% (3.1 to 6.4)	8.6% (6.9 to 10.6)
**Norway**	57.6% (42.5 to 68.9)	0.0% (0.0 to 0.0)	0.0% (0.0 to 0.0)	8.0% (5.2 to 10.8)	3.5% (-0.4 to 8.0)	4.0% (-0.6 to 9.0)	6.9% (5.9 to 7.9)	1.4% (0.9 to 1.9)	7.6% (6.1 to 9.3)

**Oman**	59.9%<br>(44.4 to 71.6)	8.3%<br>(3.9 to 13.7)	0.0%<br>(0.0 to 0.1)	10.5%<br>(7.3 to 13.9)	6.6%<br>(-0.9 to 14.8)	6.3%<br>(1.9 to 11.4)	2.8%<br>(0.7 to 5.8)	3.3%<br>(2.2 to 4.4)	5.7%<br>(4.6 to 7.0)
**Pakistan**	59.2%<br>(43.6 to 71.4)	5.4%<br>(0.7 to 11.2)	24.4%<br>(13.8 to 35.0)	10.9%<br>(7.7 to 14.2)	11.1%<br>(-1.6 to 24.5)	4.6%<br>(1.4 to 8.2)	4.7%<br>(2.1 to 7.9)	4.5%<br>(3.1 to 6.0)	8.5%<br>(6.5 to 10.7)
**Palau**	56.5%<br>(41.0 to 68.3)	0.5%<br>(0.2 to 0.9)	0.0%<br>(0.0 to 0.0)	10.8%<br>(8.0 to 13.9)	1.5%<br>(-0.2 to 3.3)	4.6%<br>(1.6 to 8.1)	0.0%<br>(0.0 to 0.0)	3.9%<br>(2.6 to 5.3)	8.0%<br>(6.5 to 9.9)
**Palestine**	51.3%<br>(36.3 to 64.4)	1.8%<br>(-0.6 to 5.8)	1.3%<br>(0.5 to 2.8)	10.2%<br>(7.0 to 13.7)	9.5%<br>(-1.3 to 20.6)	5.8%<br>(1.2 to 11.4)	3.6%<br>(2.8 to 4.6)	4.3%<br>(2.9 to 5.7)	10.5%<br>(8.5 to 13.0)
**Panama**	59.7%<br>(44.0 to 70.8)	0.2%<br>(0.1 to 0.4)	0.4%<br>(0.0 to 4.2)	11.8%<br>(8.2 to 15.3)	7.8%<br>(-1.0 to 16.8)	3.1%<br>(-0.1 to 6.5)	0.3%<br>(0.2 to 0.4)	1.6%<br>(1.1 to 2.2)	4.8%<br>(3.8 to 6.2)
**Papua New Guinea**	38.7%<br>(25.4 to 51.0)	0.1%<br>(0.1 to 0.2)	40.4%<br>(30.4 to 48.9)	9.6%<br>(6.9 to 12.4)	3.5%<br>(-0.4 to 8.1)	4.5%<br>(1.4 to 8.0)	3.0%<br>(2.6 to 3.8)	5.7%<br>(3.7 to 7.7)	6.4%<br>(4.8 to 8.4)
**Paraguay**	58.5%<br>(42.8 to 70.8)	0.9%<br>(-0.7 to 3.2)	7.0%<br>(0.6 to 21.9)	10.0%<br>(7.0 to 12.9)	6.5%<br>(-0.8 to 14.5)	3.0%<br>(0.1 to 6.3)	2.4%<br>(1.8 to 3.2)	3.1%<br>(2.0 to 4.2)	11.6%<br>(8.9 to 14.8)
**Peru**	53.3%<br>(39.0 to 65.1)	0.0%<br>(0.0 to 0.1)	3.1%<br>(0.2 to 13.6)	7.2%<br>(4.9 to 9.6)	6.6%<br>(-0.9 to 15.1)	3.6%<br>(0.5 to 7.2)	5.6%<br>(4.6 to 6.7)	1.3%<br>(0.8 to 1.8)	5.2%<br>(4.1 to 6.6)
**Philippines**	51.0%<br>(37.4 to 62.3)	0.9%<br>(0.8 to 1.0)	18.3%<br>(9.4 to 29.4)	13.0%<br>(9.6 to 16.4)	4.5%<br>(-0.6 to 10.1)	3.0%<br>(0.7 to 6.0)	0.1%<br>(0.0 to 0.2)	4.5%<br>(3.1 to 6.0)	11.1%<br>(9.3 to 13.3)
**Poland**	55.5%<br>(40.9 to 67.4)	0.0%<br>(-0.2 to 0.1)	0.5%<br>(0.0 to 4.7)	8.6%<br>(5.8 to 11.4)	4.5%<br>(-0.6 to 10.4)	4.4%<br>(0.6 to 9.0)	8.7%<br>(7.7 to 10.8)	2.8%<br>(1.9 to 3.8)	11.0%<br>(9.3 to 13.1)
**Portugal**	52.6%<br>(38.6 to 64.5)	0.0%<br>(0.0 to 0.1)	0.0%<br>(0.0 to 0.1)	7.1%<br>(4.7 to 9.7)	7.4%<br>(-1.0 to 16.5)	4.2%<br>(-1.5 to 10.2)	7.9%<br>(7.3 to 8.5)	2.2%<br>(1.5 to 3.0)	5.5%<br>(4.5 to 6.7)
**Puerto Rico**	55.9%<br>(40.6 to 68.6)	0.4%<br>(0.3 to 0.6)	0.0%<br>(0.0 to 0.0)	8.6%<br>(5.9 to 11.3)	3.8%<br>(-0.5 to 8.6)	4.4%<br>(0.7 to 8.5)	0.0%<br>(-0.1 to 0.1)	1.3%<br>(0.9 to 1.8)	7.1%<br>(5.6 to 9.1)
**Qatar**	58.9%<br>(44.5 to 70.4)	8.8%<br>(3.0 to 15.0)	0.0%<br>(0.0 to 0.0)	10.3%<br>(7.0 to 13.9)	4.3%<br>(-0.6 to 9.6)	5.9%<br>(1.1 to 11.6)	3.2%<br>(0.8 to 6.4)	4.2%<br>(2.8 to 5.6)	6.5%<br>(5.0 to 8.2)
**Republic of Korea**	44.5%<br>(30.2 to 56.4)	0.3%<br>(-0.4 to 1.5)	0.0%<br>(0.0 to 0.0)	8.7%<br>(5.4 to 12.1)	5.2%<br>(-0.7 to 11.7)	4.5%<br>(0.3 to 9.6)	5.8%<br>(5.0 to 6.8)	2.9%<br>(2.0 to 4.0)	11.3%<br>(9.2 to 13.8)
**Republic of Moldova**	69.1%<br>(52.9 to 80.7)	0.1%<br>(-0.7 to 1.0)	5.7%<br>(2.0 to 10.9)	13.6%<br>(10.0 to 17.4)	3.9%<br>(-0.5 to 8.9)	3.9%<br>(1.0 to 7.2)	7.7%<br>(6.6 to 9.4)	2.7%<br>(1.8 to 3.6)	12.4%<br>(10.5 to 14.7)
**Romania**	66.1%<br>(50.7 to 77.3)	0.2%<br>(-0.4 to 1.2)	0.6%<br>(0.0 to 5.9)	8.2%<br>(5.3 to 11.2)	4.2%<br>(-0.6 to 9.2)	3.8%<br>(0.2 to 7.9)	8.5%<br>(7.6 to 10.1)	3.0%<br>(2.0 to 4.0)	8.0%<br>(6.6 to 9.6)
**Russian Federation**	62.3%<br>(47.5 to 73.2)	-0.1%<br>(-0.5 to 0.3)	0.1%<br>(0.0 to 0.9)	10.6%<br>(7.3 to 13.9)	3.0%<br>(-0.4 to 6.8)	3.7%<br>(0.2 to 7.7)	8.6%<br>(6.9 to 10.2)	2.7%<br>(1.8 to 3.7)	12.0%<br>(10.4 to 13.9)
**Rwanda**	55.7%<br>(40.3 to 68.6)	0.0%<br>(0.0 to 0.0)	46.8%<br>(39.1 to 54.5)	8.0%<br>(5.3 to 10.8)	7.9%<br>(-1.1 to 17.7)	2.2%<br>(0.2 to 4.8)	3.8%<br>(3.3 to 5.0)	1.8%<br>(1.2 to 2.4)	10.6%<br>(7.7 to 13.9)
**Saint Kitts and Nevis**	56.4%<br>(41.9 to 70.0)	0.4%<br>(0.2 to 0.5)	0.1%<br>(0.0 to 0.6)	8.7%<br>(6.0 to 11.4)	5.6%<br>(-0.7 to 12.6)	4.3%<br>(-0.1 to 9.1)	0.0%<br>(-0.1 to 0.1)	1.7%<br>(1.1 to 2.3)	3.4%<br>(2.6 to 4.5)
**Saint Lucia**	55.6%<br>(40.1 to 68.2)	0.4%<br>(0.3 to 0.7)	0.7%<br>(0.0 to 3.6)	8.8%<br>(6.0 to 11.6)	8.3%<br>(-1.2 to 18.3)	4.0%<br>(-0.2 to 8.9)	0.0%<br>(0.0 to 0.1)	1.4%<br>(0.9 to 1.9)	4.3%<br>(3.4 to 5.5)
**Saint Vincent and the Grenadines**	52.8%<br>(38.1 to 65.1)	0.4%<br>(0.3 to 0.6)	0.7%<br>(0.1 to 3.2)	8.8%<br>(6.1 to 11.6)	9.5%<br>(-1.3 to 20.8)	4.2%<br>(0.2 to 8.9)	0.0%<br>(0.0 to 0.0)	2.0%<br>(1.3 to 2.7)	4.5%<br>(3.6 to 5.7)

**Samoa**	53.8%<br>(38.3 to 66.9)	0.0%<br>(0.0 to 0.1)	27.1%<br>(13.0 to 38.6)	10.6%<br>(7.7 to 13.4)	2.3%<br>(-0.3 to 5.5)	6.8%<br>(2.5 to 11.7)	0.1%<br>(-0.1 to 0.3)	5.2%<br>(3.6 to 7.0)	10.9%<br>(8.7 to 13.3)
**San Marino**	57.7%<br>(43.8 to 70.0)	0.1%<br>(-0.2 to 0.4)	0.0%<br>(0.0 to 0.0)	8.2%<br>(5.5 to 11.2)	3.0%<br>(-0.4 to 6.7)	4.4%<br>(-0.3 to 9.8)	6.5%<br>(5.4 to 7.4)	1.6%<br>(1.1 to 2.2)	9.4%<br>(7.2 to 11.9)
**Sao Tome and Principe**	65.2%<br>(50.2 to 77.0)	0.2%<br>(0.0 to 0.4)	25.0%<br>(17.7 to 32.4)	11.5%<br>(8.4 to 14.7)	7.4%<br>(-1.0 to 16.4)	2.3%<br>(0.5 to 4.5)	0.0%<br>(0.0 to 0.1)	0.9%<br>(0.6 to 1.3)	2.8%<br>(2.0 to 3.7)
**Saudi Arabia**	56.1%<br>(40.6 to 68.3)	8.6%<br>(2.4 to 15.7)	0.0%<br>(0.0 to 0.0)	10.9%<br>(7.8 to 14.2)	7.8%<br>(-1.0 to 17.7)	7.5%<br>(2.7 to 13.2)	3.2%<br>(1.1 to 5.9)	4.0%<br>(2.6 to 5.3)	7.0%<br>(5.5 to 8.6)
**Senegal**	64.6%<br>(49.9 to 76.0)	4.0%<br>(2.9 to 5.7)	38.7%<br>(30.4 to 46.6)	9.5%<br>(6.7 to 12.5)	7.3%<br>(-1.0 to 15.9)	4.1%<br>(1.3 to 7.8)	0.3%<br>(0.0 to 0.6)	3.3%<br>(2.2 to 4.5)	3.9%<br>(2.9 to 5.0)
**Serbia**	65.9%<br>(50.5 to 77.0)	0.3%<br>(-0.4 to 1.5)	2.7%<br>(0.1 to 15.9)	7.0%<br>(4.6 to 9.6)	3.9%<br>(-0.5 to 8.9)	3.6%<br>(-0.3 to 8.0)	8.6%<br>(7.6 to 10.5)	4.2%<br>(2.8 to 5.6)	10.1%<br>(8.4 to 12.2)
**Seychelles**	60.9%<br>(45.4 to 72.6)	0.4%<br>(0.2 to 0.5)	0.0%<br>(0.0 to 0.2)	13.2%<br>(9.7 to 16.9)	3.1%<br>(-0.4 to 7.2)	3.9%<br>(0.1 to 8.3)	0.1%<br>(-0.1 to 0.3)	4.1%<br>(2.7 to 5.4)	11.9%<br>(9.7 to 14.3)
**Sierra Leone**	66.7%<br>(52.4 to 78.0)	0.7%<br>(0.5 to 1.0)	42.5%<br>(35.1 to 49.6)	10.8%<br>(7.8 to 13.9)	9.0%<br>(-1.3 to 19.5)	3.5%<br>(1.0 to 6.4)	0.1%<br>(0.0 to 0.2)	3.0%<br>(1.9 to 4.2)	4.7%<br>(3.5 to 6.2)
**Singapore**	42.1%<br>(29.0 to 54.2)	0.3%<br>(0.3 to 0.4)	0.0%<br>(0.0 to 0.0)	11.2%<br>(7.7 to 14.8)	6.1%<br>(-0.8 to 13.8)	4.8%<br>(1.5 to 8.8)	0.0%<br>(-0.1 to 0.0)	2.1%<br>(1.4 to 2.9)	6.6%<br>(5.5 to 7.9)
**Slovakia**	63.6%<br>(47.7 to 74.9)	0.0%<br>(-0.3 to 0.4)	0.0%<br>(0.0 to 0.3)	7.9%<br>(5.3 to 10.6)	3.4%<br>(-0.4 to 7.8)	3.3%<br>(0.2 to 6.9)	8.0%<br>(6.8 to 9.1)	3.6%<br>(2.4 to 4.8)	10.4%<br>(8.6 to 12.5)
**Slovenia**	62.2%<br>(46.3 to 72.8)	0.1%<br>(-0.2 to 0.5)	0.2%<br>(0.0 to 1.5)	7.8%<br>(5.1 to 10.7)	3.0%<br>(-0.4 to 6.9)	3.2%<br>(-1.0 to 7.6)	7.4%<br>(6.6 to 9.1)	3.1%<br>(2.1 to 4.2)	8.5%<br>(6.9 to 10.4)
**Solomon Islands**	47.0%<br>(32.3 to 59.3)	0.3%<br>(0.1 to 0.5)	44.8%<br>(36.9 to 52.2)	10.2%<br>(7.5 to 13.0)	5.3%<br>(-0.6 to 11.9)	5.8%<br>(2.1 to 10.0)	0.0%<br>(0.0 to 0.1)	6.2%<br>(4.1 to 8.4)	10.5%<br>(8.5 to 12.8)
**Somalia**	55.8%<br>(39.8 to 68.2)	0.8%<br>(0.0 to 1.3)	46.8%<br>(39.4 to 53.9)	8.0%<br>(5.4 to 10.7)	13.7%<br>(-2.0 to 29.6)	2.4%<br>(0.6 to 4.6)	0.4%<br>(0.2 to 0.5)	2.4%<br>(1.4 to 3.4)	3.7%<br>(2.4 to 5.8)
**South Africa**	63.8%<br>(48.6 to 74.8)	0.1%<br>(-0.1 to 0.4)	4.3%<br>(0.8 to 11.1)	13.0%<br>(9.3 to 16.5)	5.1%<br>(-0.6 to 11.3)	6.4%<br>(1.2 to 12.3)	5.6%<br>(5.0 to 6.2)	3.5%<br>(2.3 to 4.7)	5.2%<br>(4.3 to 6.2)
**South Sudan**	56.5%<br>(40.2 to 68.5)	4.7%<br>(3.4 to 6.5)	43.1%<br>(34.9 to 50.5)	8.0%<br>(5.4 to 10.8)	8.8%<br>(-1.2 to 19.8)	2.2%<br>(0.5 to 4.5)	0.4%<br>(0.1 to 0.7)	2.2%<br>(1.4 to 3.1)	4.5%<br>(3.2 to 6.5)
**Spain**	55.2%<br>(40.7 to 67.2)	0.1%<br>(-0.1 to 0.4)	0.0%<br>(0.0 to 0.1)	7.9%<br>(5.0 to 10.7)	6.0%<br>(-0.8 to 13.7)	4.4%<br>(-0.3 to 9.6)	5.7%<br>(4.9 to 6.5)	2.7%<br>(1.8 to 3.6)	9.4%<br>(7.8 to 11.4)
**Sri Lanka**	60.1%<br>(44.4 to 71.5)	0.6%<br>(0.4 to 0.7)	10.7%<br>(0.9 to 34.3)	12.3%<br>(8.6 to 16.1)	3.4%<br>(-0.4 to 7.6)	2.0%<br>(0.0 to 4.3)	0.4%<br>(0.2 to 0.6)	3.0%<br>(1.9 to 4.4)	4.8%<br>(3.9 to 6.0)
**Sudan**	64.8%<br>(49.3 to 75.8)	8.8%<br>(4.7 to 14.2)	19.8%<br>(11.1 to 28.9)	10.8%<br>(7.6 to 14.1)	11.7%<br>(-1.7 to 26.0)	11.8%<br>(5.2 to 18.3)	1.3%<br>(0.3 to 2.7)	3.5%<br>(2.3 to 4.6)	7.1%<br>(5.3 to 9.3)
**Suriname**	48.8%<br>(33.9 to 62.2)	0.4%<br>(0.2 to 0.7)	1.0%<br>(0.0 to 6.5)	8.9%<br>(6.3 to 11.6)	7.8%<br>(-1.0 to 17.4)	5.6%<br>(1.7 to 10.5)	0.0%<br>(-0.1 to 0.1)	3.1%<br>(2.0 to 4.1)	7.9%<br>(6.2 to 9.9)
**Sweden**	55.7%<br>(40.7 to 67.1)	0.0%<br>(0.0 to 0.0)	0.0%<br>(0.0 to 0.0)	9.1%<br>(5.9 to 12.3)	2.3%<br>(-0.3 to 5.4)	3.6%<br>(-0.5 to 8.1)	7.2%<br>(6.1 to 8.2)	1.3%<br>(0.8 to 1.8)	10.2%<br>(8.1 to 12.5)
**Switzerland**	51.4%<br>(36.2 to 63.8)	0.0%<br>(-0.1 to 0.1)	0.0%<br>(0.0 to 0.0)	9.4%<br>(6.2 to 13.0)	4.3%<br>(-0.5 to 9.8)	3.9%<br>(-0.7 to 8.8)	7.0%<br>(6.1 to 8.6)	1.6%<br>(1.1 to 2.2)	10.5%<br>(8.5 to 12.8)

**Syrian Arab Republic**	56.5% (42.2 to 68.5)	1.5% (-0.2 to 4.0)	0.1% (0.0 to 0.2)	10.3% (7.1 to 13.7)	9.5% (-1.3 to 21.2)	6.4% (1.9 to 11.9)	5.6% (4.6 to 6.4)	4.2% (2.9 to 5.7)	10.5% (8.5 to 12.5)
**Turkey**	55.6% (41.0 to 66.9)	0.3% (-0.3 to 1.0)	0.1% (0.0 to 0.8)	10.8% (7.2 to 14.4)	5.4% (-0.7 to 11.9)	5.2% (0.4 to 10.9)	7.2% (6.4 to 8.0)	5.0% (3.3 to 6.7)	9.3% (7.7 to 11.0)
**Taiwan (Province of China)**	49.3% (34.3 to 62.4)	0.9% (-0.6 to 3.2)	0.1% (0.0 to 0.6)	8.4% (5.9 to 10.9)	6.0% (-0.8 to 13.6)	5.7% (2.0 to 10.0)	1.5% (1.2 to 1.9)	4.4% (2.9 to 5.8)	10.6% (8.7 to 12.5)
**Tajikistan**	60.9% (45.3 to 73.0)	0.6% (-0.3 to 2.0)	21.4% (14.3 to 28.5)	12.3% (8.8 to 15.8)	6.5% (-0.9 to 14.8)	3.4% (0.4 to 7.0)	7.7% (6.4 to 8.6)	3.1% (2.1 to 4.0)	6.2% (4.7 to 8.1)
**Thailand**	50.2% (35.4 to 62.7)	1.7% (1.5 to 2.2)	1.5% (0.0 to 9.7)	14.0% (10.5 to 17.5)	3.6% (-0.5 to 8.5)	4.0% (0.9 to 7.9)	0.6% (0.2 to 1.2)	3.8% (2.6 to 5.1)	10.4% (8.7 to 12.1)
**Timor-Leste**	60.3% (46.0 to 72.8)	0.1% (0.0 to 0.2)	29.2% (11.7 to 44.9)	12.6% (9.2 to 16.3)	9.1% (-1.2 to 20.0)	2.0% (0.4 to 4.0)	0.6% (0.4 to 0.8)	5.0% (3.2 to 6.9)	10.9% (7.8 to 14.1)
**Togo**	64.8% (48.7 to 76.2)	2.3% (1.9 to 2.8)	40.1% (32.8 to 46.8)	10.6% (7.7 to 13.6)	7.9% (-1.0 to 17.8)	2.3% (0.4 to 4.4)	0.1% (0.0 to 0.2)	2.0% (1.3 to 2.6)	6.7% (5.1 to 8.7)
**Tokelau**	54.5% (38.4 to 66.3)	0.6% (0.2 to 1.0)	0.0% (0.0 to 0.1)	10.2% (7.4 to 13.1)	2.0% (-0.3 to 4.5)	4.9% (1.8 to 8.7)	0.0% (0.0 to 0.0)	4.7% (3.1 to 6.5)	7.8% (6.1 to 9.7)
**Tonga**	57.3% (41.8 to 70.4)	0.3% (0.2 to 0.5)	18.3% (8.3 to 28.2)	10.6% (7.6 to 13.6)	2.5% (-0.3 to 5.8)	5.3% (1.8 to 9.5)	0.0% (0.0 to 0.0)	5.4% (3.7 to 7.2)	11.1% (8.8 to 13.8)
**Trinidad and Tobago**	59.7% (45.8 to 71.2)	0.2% (0.1 to 0.5)	0.0% (0.0 to 0.0)	9.0% (6.3 to 11.8)	3.5% (-0.5 to 8.0)	6.0% (1.5 to 11.1)	0.0% (-0.1 to 0.1)	2.9% (1.8 to 3.8)	6.9% (5.5 to 8.5)
**Tunisia**	52.2% (38.4 to 64.6)	2.1% (-0.1 to 6.0)	0.1% (0.0 to 0.3)	10.1% (6.9 to 13.4)	8.0% (-1.1 to 17.8)	4.3% (0.3 to 8.9)	4.9% (3.8 to 5.8)	4.7% (3.2 to 6.3)	12.6% (9.7 to 16.0)
**Turkmenistan**	63.7% (49.0 to 75.4)	1.8% (0.1 to 4.3)	0.0% (0.0 to 0.1)	13.4% (10.2 to 16.8)	3.7% (-0.5 to 8.4)	3.8% (1.1 to 6.8)	7.2% (5.9 to 8.6)	4.3% (3.0 to 5.8)	8.6% (6.8 to 10.5)
**Tuvalu**	55.1% (40.2 to 67.8)	0.6% (0.2 to 1.0)	7.4% (4.2 to 11.7)	10.7% (7.9 to 13.7)	3.1% (-0.4 to 7.0)	5.0% (1.7 to 8.6)	0.0% (0.0 to 0.0)	6.0% (4.0 to 8.2)	9.7% (7.8 to 12.1)
**Uganda**	59.2% (45.4 to 70.7)	0.0% (0.0 to 0.2)	43.7% (36.3 to 51.2)	8.5% (5.7 to 11.5)	8.4% (-1.2 to 18.6)	2.3% (0.2 to 4.8)	1.2% (0.9 to 1.4)	1.4% (0.9 to 2.0)	3.5% (2.6 to 4.6)
**Ukraine**	64.1% (48.1 to 75.1)	-0.1% (-0.7 to 0.5)	1.0% (0.1 to 4.0)	11.7% (8.3 to 15.1)	2.5% (-0.3 to 5.7)	2.9% (0.6 to 6.0)	8.5% (7.0 to 9.8)	3.1% (2.1 to 4.2)	10.9% (8.3 to 13.7)
**United Arab Emirates**	56.8% (41.2 to 68.7)	11.3% (5.1 to 18.9)	0.0% (0.0 to 0.0)	10.7% (7.3 to 14.3)	4.7% (-0.6 to 10.4)	5.8% (1.3 to 11.0)	4.0% (1.0 to 8.3)	4.2% (2.8 to 5.6)	6.5% (4.9 to 8.2)
**United Kingdom**	51.1% (37.8 to 61.6)	0.0% (0.0 to 0.0)	0.0% (0.0 to 0.0)	9.1% (5.9 to 12.4)	3.3% (-0.4 to 7.5)	6.9% (-0.3 to 14.5)	6.3% (5.1 to 8.7)	1.3% (0.9 to 1.7)	9.7% (7.8 to 12.0)
**United Republic of Tanzania**	55.4% (40.5 to 67.8)	0.1% (0.0 to 0.3)	43.8% (35.8 to 51.2)	8.1% (5.3 to 10.9)	5.8% (-0.8 to 13.0)	1.0% (0.1 to 2.0)	1.5% (1.2 to 1.8)	1.8% (1.1 to 2.5)	6.2% (4.7 to 8.1)
**United States of America**	50.1% (35.8 to 62.5)	0.3% (-0.4 to 1.3)	0.0% (0.0 to 0.0)	10.5% (7.2 to 13.9)	3.1% (-0.4 to 7.2)	4.1% (0.2 to 8.9)	4.5% (3.8 to 5.3)	1.5% (1.0 to 2.0)	10.7% (8.9 to 13.0)
**United States Virgin Islands**	53.5% (38.9 to 65.5)	0.3% (0.2 to 0.5)	0.0% (0.0 to 0.0)	8.9% (6.2 to 11.6)	3.5% (-0.5 to 8.2)	3.6% (-0.4 to 7.8)	0.0% (-0.1 to 0.1)	1.9% (1.2 to 2.5)	5.5% (3.9 to 7.2)
**Uruguay**	57.1% (40.5 to 68.7)	0.1% (-0.1 to 0.3)	0.1% (0.0 to 1.3)	8.0% (5.2 to 10.8)	5.7% (-0.7 to 12.7)	3.7% (0.8 to 7.4)	5.9% (5.4 to 6.7)	2.6% (1.7 to 3.5)	9.4% (7.7 to 11.3)

**Uzbekistan**	58.6% (43.5 to 71.1)	0.7% (-0.5 to 2.0)	4.3% (0.8 to 11.8)	13.4% (9.9 to 16.7)	4.0% (-0.5 to 9.4)	2.2% (0.6 to 4.2)	7.3% (5.8 to 8.4)	3.8% (2.6 to 5.2)	7.9% (6.6 to 9.4)
**Vanuatu**	64.0% (48.8 to 75.1)	0.1% (0.1 to 0.1)	42.1% (33.9 to 50.0)	10.8% (7.9 to 13.8)	4.5% (-0.6 to 10.2)	1.8% (0.3 to 3.5)	0.2% (0.1 to 0.4)	3.0% (2.0 to 4.1)	6.3% (5.1 to 7.8)
**Venezuela (Bolivarian Republic of)**	61.0% (45.6 to 72.8)	0.5% (0.4 to 0.5)	0.2% (0.0 to 0.9)	11.7% (8.1 to 15.2)	8.8% (-1.2 to 19.7)	3.1% (0.0 to 6.7)	0.9% (0.6 to 1.1)	2.9% (1.8 to 4.0)	5.3% (4.3 to 6.7)
**Viet Nam**	64.1% (49.1 to 75.7)	1.7% (0.2 to 3.8)	16.4% (5.9 to 28.1)	10.2% (7.0 to 13.5)	5.8% (-0.8 to 13.1)	3.0% (0.7 to 5.8)	1.3% (0.8 to 1.8)	4.4% (3.0 to 5.9)	13.5% (10.8 to 16.5)
**Yemen**	53.9% (38.4 to 66.2)	1.5% (0.7 to 2.7)	17.4% (11.5 to 23.7)	10.2% (7.2 to 13.5)	14.4% (-2.0 to 30.9)	6.7% (2.1 to 11.4)	2.9% (2.3 to 3.6)	4.9% (3.3 to 6.6)	11.7% (9.4 to 14.4)
**Zambia**	51.5% (36.0 to 64.5)	0.5% (-0.4 to 2.0)	38.1% (27.8 to 46.7)	8.1% (5.5 to 11.0)	7.1% (-0.9 to 15.9)	2.8% (0.6 to 5.4)	2.4% (2.0 to 2.9)	2.0% (1.2 to 2.9)	5.0% (3.6 to 6.5)
**Zimbabwe**	66.2% (51.0 to 77.5)	0.5% (-0.4 to 1.7)	38.4% (31.3 to 45.8)	12.7% (9.2 to 16.2)	8.1% (-1.1 to 17.9)	3.4% (0.6 to 6.6)	3.2% (2.4 to 4.1)	2.9% (1.9 to 3.9)	8.4% (6.4 to 10.8)

Appendix Table 13. Age-standardized percent of DALYs (with 95% uncertainty intervals) due to intracerebral haemorrhage associated with individual risk factors by 7 GBD super-region, 21 GBD region and country for both sexes combined in 2021.

Country/region	Alcohol use	Ambient particulate matter pollution	Diet high in processed meat	Diet high in red meat	Diet high in sodium	Diet high in sugar-sweetened beverages	Diet low in fiber	Diet low in fruits	Diet low in polyunsaturated fatty acids	Diet low in vegetables	Diet low in whole grains	High body-mass index	High fasting plasma glucose	High LDL cholesterol
**GBD super-regions in alphabetical order**														
**Central Europe, Eastern Europe, and Central Asia**	9.4% (0.3 to 19.0)	11.4% (8.3 to 14.9)	Not reported	-10.1% (-40.9 to 14.5)	9.0% (1.6 to 21.1)	Not reported	2.8% (-0.8 to 5.9)	7.7% (-0.6 to 14.5)	Not reported	0.1% (0.0 to 0.1)	Not reported	8.1% (-0.3 to 18.3)	4.8% (2.6 to 7.2)	Not reported
**High-income**	10.3% (0.3 to 20.7)	6.9% (4.7 to 9.5)	Not reported	-10.3% (-42.0 to 14.4)	6.5% (0.5 to 17.6)	Not reported	3.4% (-1.0 to 7.3)	6.2% (-0.4 to 11.8)	Not reported	0.1% (0.0 to 0.3)	Not reported	6.6% (-0.2 to 14.6)	6.4% (3.4 to 9.2)	Not reported
**Latin America and Caribbean**	6.0% (0.1 to 12.5)	9.5% (6.7 to 12.8)	Not reported	-9.3% (-38.9 to 13.1)	6.5% (0.4 to 17.6)	Not reported	3.1% (-0.9 to 6.5)	5.8% (-0.4 to 11.0)	Not reported	1.9% (-0.2 to 3.7)	Not reported	7.9% (-0.3 to 17.5)	5.6% (3.0 to 8.3)	Not reported
**North Africa and Middle East**	0.5% (0.0 to 1.3)	21.3% (16.7 to 25.9)	Not reported	-2.6% (-10.5 to 4.3)	1.5% (0.0 to 7.4)	Not reported	1.8% (-0.5 to 3.8)	5.2% (-0.4 to 9.6)	Not reported	1.1% (-0.1 to 2.0)	Not reported	9.3% (-0.4 to 19.8)	6.1% (3.2 to 9.0)	Not reported
**South Asia**	3.0% (0.1 to 6.9)	16.6% (9.9 to 22.8)	Not reported	-0.5% (-2.0 to 0.9)	6.5% (0.3 to 18.5)	Not reported	4.2% (-1.2 to 9.0)	13.6% (-1.0 to 25.0)	Not reported	2.3% (-0.2 to 4.6)	Not reported	1.7% (0.0 to 4.0)	5.9% (3.2 to 8.7)	Not reported
**Southeast Asia, East Asia, and Oceania**	6.2% (0.1 to 12.7)	19.9% (12.9 to 24.7)	Not reported	-7.2% (-29.5 to 10.2)	15.7% (5.3 to 29.5)	Not reported	3.6% (-1.0 to 7.4)	6.5% (-0.4 to 12.2)	Not reported	0.5% (-0.1 to 1.0)	Not reported	2.4% (0.0 to 5.9)	5.0% (2.6 to 7.4)	Not reported
**Sub-Saharan Africa**	5.0% (0.2 to 10.4)	8.7% (5.3 to 12.2)	Not reported	-2.2% (-9.0 to 3.8)	4.6% (0.1 to 14.4)	Not reported	1.3% (-0.3 to 2.7)	8.8% (-0.6 to 16.5)	Not reported	5.7% (-0.5 to 10.8)	Not reported	3.4% (0.0 to 7.7)	3.9% (2.1 to 6.0)	Not reported
**GBD regions in alphabetical order**														
**Andean Latin America**	6.3% (0.1 to 13.4)	16.3% (10.2 to 23.0)	Not reported	-5.6% (-23.4 to 8.6)	6.0% (0.2 to 17.1)	Not reported	3.6% (-1.1 to 7.6)	6.1% (-0.4 to 11.3)	Not reported	2.8% (-0.3 to 5.5)	Not reported	7.7% (-0.2 to 17.3)	4.7% (2.5 to 7.0)	Not reported
**Australasia**	11.6% (0.3 to 23.2)	5.0% (3.0 to 7.4)	Not reported	-12.0% (-49.5 to 16.3)	2.8% (0.0 to 11.0)	Not reported	3.4% (-1.0 to 7.4)	6.0% (-0.4 to 11.7)	Not reported	0.0% (0.0 to 0.0)	Not reported	7.4% (-0.3 to 16.4)	7.4% (4.0 to 10.8)	Not reported
**Caribbean**	6.0% (0.1 to 12.7)	9.5% (4.9 to 15.8)	Not reported	-3.2% (-13.1 to 5.1)	3.7% (0.0 to 13.0)	Not reported	3.4% (-1.1 to 7.0)	6.0% (-0.5 to 11.0)	Not reported	5.0% (-0.5 to 9.3)	Not reported	5.5% (-0.1 to 12.3)	4.9% (2.6 to 7.4)	Not reported
**Central Asia**	5.7% (0.1 to 12.4)	17.0% (11.8 to 22.2)	Not reported	-9.2% (-37.6 to 13.1)	6.3% (0.4 to 17.8)	Not reported	2.9% (-0.8 to 6.3)	7.1% (-0.5 to 13.4)	Not reported	0.1% (0.0 to 0.2)	Not reported	6.7% (-0.2 to 15.5)	4.4% (2.3 to 6.6)	Not reported
**Central Europe**	11.2% (0.4 to 22.1)	12.5% (9.3 to 15.7)	Not reported	-10.2% (-42.3 to 14.6)	15.5% (4.4 to 29.5)	Not reported	2.1% (-0.6 to 4.6)	6.3% (-0.4 to 11.8)	Not reported	0.0% (0.0 to 0.0)	Not reported	7.4% (-0.2 to 16.8)	6.5% (3.5 to 9.5)	Not reported
**Central Latin America**	5.0% (0.1 to 10.7)	9.8% (6.8 to 13.0)	Not reported	-6.9% (-28.8 to 10.4)	7.6% (0.8 to 19.5)	Not reported	2.3% (-0.6 to 4.8)	6.3% (-0.4 to 11.7)	Not reported	1.1% (-0.1 to 2.1)	Not reported	8.9% (-0.4 to 19.6)	5.7% (3.0 to 8.4)	Not reported
**Central Sub-Saharan Africa**	4.5% (0.1 to 10.0)	6.9% (4.3 to 10.0)	Not reported	-1.5% (-6.3 to 2.7)	2.0% (0.0 to 9.3)	Not reported	2.7% (-0.8 to 5.9)	8.0% (-0.6 to 15.1)	Not reported	8.9% (-0.9 to 16.9)	Not reported	3.1% (0.0 to 7.1)	5.7% (3.0 to 8.4)	Not reported
**East Asia**	7.2% (0.1 to 14.9)	23.3% (15.5 to 28.8)	Not reported	-9.4% (-38.4 to 13.2)	17.7% (6.5 to 32.1)	Not reported	1.9% (-0.5 to 4.1)	5.9% (-0.4 to 11.3)	Not reported	0.0% (0.0 to 0.0)	Not reported	2.5% (0.0 to 6.4)	5.5% (2.9 to 8.3)	Not reported

**Eastern Europe**	10.0% (0.2 to 20.6)	8.3% (5.3 to 12.5)	Not reported	-10.0% (-40.2 to 14.7)	7.1% (0.6 to 18.5)	Not reported	2.9% (-0.8 to 6.2)	8.5% (-0.6 to 15.9)	Not reported	0.0% (0.0 to 0.1)	Not reported	8.9% (-0.3 to 20.0)	4.2% (2.2 to 6.3)	Not reported
**Eastern Sub-Saharan Africa**	4.7% (0.1 to 9.8)	4.8% (2.9 to 7.0)	Not reported	-1.6% (-6.8 to 3.0)	6.8% (0.4 to 18.7)	Not reported	1.4% (-0.4 to 3.0)	9.1% (-0.7 to 17.2)	Not reported	7.5% (-0.8 to 14.4)	Not reported	1.9% (0.0 to 4.7)	3.0% (1.6 to 4.6)	Not reported
**High-income Asia Pacific**	10.1% (0.2 to 20.2)	11.2% (6.9 to 16.4)	Not reported	-6.8% (-27.4 to 10.1)	10.6% (1.6 to 23.9)	Not reported	4.4% (-1.3 to 9.4)	7.7% (-0.6 to 14.2)	Not reported	0.0% (0.0 to 0.0)	Not reported	1.8% (-0.1 to 4.8)	6.6% (3.5 to 9.6)	Not reported
**High-income North America**	9.3% (0.3 to 19.5)	3.2% (1.6 to 5.1)	Not reported	-11.7% (-48.3 to 16.2)	5.4% (0.2 to 15.9)	Not reported	2.9% (-0.8 to 6.3)	6.5% (-0.5 to 12.1)	Not reported	0.4% (0.0 to 0.7)	Not reported	10.2% (-0.4 to 21.4)	7.5% (4.1 to 10.8)	Not reported
**North Africa and Middle East**	0.5% (0.0 to 1.3)	21.3% (16.7 to 25.9)	Not reported	-2.6% (-10.5 to 4.3)	1.5% (0.0 to 7.4)	Not reported	1.8% (-0.5 to 3.8)	5.2% (-0.4 to 9.6)	Not reported	1.1% (-0.1 to 2.0)	Not reported	9.3% (-0.4 to 19.8)	6.1% (3.2 to 9.0)	Not reported
**Oceania**	1.6% (0.1 to 3.6)	5.2% (1.8 to 11.4)	Not reported	-4.0% (-16.2 to 6.7)	8.5% (1.0 to 20.8)	Not reported	0.2% (-0.1 to 0.5)	8.1% (-0.6 to 15.3)	Not reported	4.0% (-0.4 to 8.0)	Not reported	4.8% (0.0 to 10.9)	5.7% (3.1 to 8.5)	Not reported
**South Asia**	3.0% (0.1 to 6.9)	16.6% (9.9 to 22.8)	Not reported	-0.5% (-2.0 to 0.9)	6.5% (0.3 to 18.5)	Not reported	4.2% (-1.2 to 9.0)	13.6% (-1.0 to 25.0)	Not reported	2.3% (-0.2 to 4.6)	Not reported	1.7% (0.0 to 4.0)	5.9% (3.2 to 8.7)	Not reported
**Southeast Asia**	4.2% (0.1 to 8.7)	12.7% (7.4 to 17.2)	Not reported	-2.9% (-11.6 to 4.5)	12.1% (2.3 to 25.5)	Not reported	7.0% (-2.2 to 14.7)	7.6% (-0.5 to 14.1)	Not reported	1.6% (-0.2 to 3.1)	Not reported	1.9% (-0.1 to 4.8)	3.9% (2.0 to 6.0)	Not reported
**Southern Latin America**	9.8% (0.2 to 19.8)	11.0% (6.3 to 17.0)	Not reported	-14.1% (-57.9 to 18.9)	6.8% (0.2 to 19.0)	Not reported	4.6% (-1.4 to 9.9)	5.4% (-0.4 to 10.4)	Not reported	0.1% (0.0 to 0.2)	Not reported	9.0% (-0.3 to 20.1)	5.8% (3.0 to 8.6)	Not reported
**Southern Sub-Saharan Africa**	6.2% (0.2 to 13.3)	12.5% (8.7 to 16.5)	Not reported	-6.5% (-26.6 to 9.8)	2.7% (0.0 to 11.3)	Not reported	1.5% (-0.5 to 3.3)	11.1% (-0.8 to 20.5)	Not reported	4.6% (-0.4 to 9.1)	Not reported	8.3% (-0.3 to 17.7)	4.1% (2.1 to 6.1)	Not reported
**Tropical Latin America**	6.6% (0.2 to 13.5)	7.9% (4.5 to 12.3)	Not reported	-14.2% (-59.5 to 19.3)	6.7% (0.3 to 18.4)	Not reported	3.4% (-1.0 to 7.5)	5.5% (-0.4 to 10.4)	Not reported	1.3% (-0.2 to 2.6)	Not reported	8.2% (-0.2 to 18.3)	6.0% (3.3 to 8.9)	Not reported
**Western Europe**	11.7% (0.3 to 23.1)	6.8% (4.8 to 9.2)	Not reported	-10.2% (-41.2 to 14.3)	4.7% (0.2 to 14.5)	Not reported	2.9% (-0.8 to 6.3)	5.2% (-0.4 to 10.0)	Not reported	0.1% (0.0 to 0.1)	Not reported	5.5% (-0.1 to 13.1)	5.1% (2.7 to 7.5)	Not reported
**Western Sub-Saharan Africa**	5.2% (0.2 to 11.0)	12.5% (6.9 to 18.6)	Not reported	-2.0% (-8.2 to 3.5)	3.8% (0.0 to 13.2)	Not reported	0.6% (-0.2 to 1.3)	8.2% (-0.6 to 15.3)	Not reported	2.8% (-0.3 to 5.6)	Not reported	3.9% (0.0 to 9.0)	4.3% (2.3 to 6.5)	Not reported
**Countries in alphabetical order**														
**Afghanistan**	0.1% (0.0 to 0.2)	6.8% (4.0 to 10.1)	Not reprted	-3.4% (-14.2 to 5.7)	1.5% (0.0 to 7.6)	Not reprted	6.0% (-1.8 to 12.6)	8.8% (-0.6 to 16.3)	Not reprted	10.6% (-1.1 to 19.8)	Not reprted	5.8% (-0.1 to 12.9)	6.9% (3.8 to 10.1)	Not reprted
**Albania**	5.2% (0.2 to 11.6)	11.0% (6.7 to 14.4)	Not reprted	-6.2% (-25.5 to 9.5)	15.7% (4.4 to 29.6)	Not reprted	1.3% (-0.4 to 3.1)	2.7% (-0.2 to 5.4)	Not reprted	0.0% (0.0 to 0.0)	Not reprted	4.5% (-0.1 to 11.4)	6.4% (3.4 to 9.6)	Not reprted
**Algeria**	0.6% (0.0 to 1.4)	17.1% (12.1 to 23.1)	Not reprted	-2.4% (-9.6 to 4.1)	1.4% (0.0 to 7.3)	Not reprted	1.1% (-0.3 to 2.3)	5.4% (-0.4 to 10.1)	Not reprted	0.0% (0.0 to 0.0)	Not reprted	7.7% (-0.3 to 17.0)	7.1% (3.9 to 10.5)	Not reprted
**American Samoa**	0.6% (0.0 to 1.9)	3.2% (0.4 to 6.8)	Not reprted	-7.1% (-28.7 to 10.0)	3.6% (0.0 to 12.4)	Not reprted	0.1% (0.0 to 0.3)	7.3% (-0.5 to 13.5)	Not reprted	4.0% (-0.4 to 8.1)	Not reprted	14.7% (-0.9 to 28.8)	11.0% (5.9 to 15.9)	Not reprted
**Andorra**	11.0% (0.3 to 22.1)	4.9% (2.7 to 7.5)	Not reprted	-10.9% (-44.7 to 14.4)	3.2% (0.0 to 12.2)	Not reprted	2.1% (-0.6 to 4.7)	4.8% (-0.3 to 9.3)	Not reprted	0.0% (0.0 to 0.0)	Not reprted	5.0% (-0.1 to 12.2)	5.6% (3.0 to 8.5)	Not reprted
**Angola**	6.2% (0.1 to 13.5)	14.3% (6.8 to 21.6)	Not reprted	-2.7% (-11.2 to 4.9)	2.9% (0.0 to 11.6)	Not reprted	0.9% (-0.3 to 2.1)	7.1% (-0.5 to 13.4)	Not reprted	1.6% (-0.1 to 3.3)	Not reprted	2.4% (0.0 to 6.1)	3.9% (2.0 to 5.9)	Not reprted
**Antigua and Barbuda**	6.8% (0.1 to 15.0)	13.6% (5.0 to 24.5)	Not reprted	-3.2% (-12.1 to 5.4)	4.4% (0.0 to 14.8)	Not reprted	7.1% (-2.1 to 14.9)	5.2% (-0.4 to 9.9)	Not reprted	1.1% (-0.1 to 2.4)	Not reprted	6.8% (-0.2 to 15.2)	5.5% (3.0 to 8.2)	Not reprted

**Argentina**	9.6% (0.2 to 19.9)	9.8% (5.0 to 16.1)	Not reprted	-14.8% (-60.6 to 19.8)	6.7% (0.2 to 18.8)	Not reprted	5.0% (-1.5 to 10.8)	4.9% (-0.3 to 9.6)	Not reprted	0.0% (0.0 to 0.1)	Not reprted	9.0% (-0.3 to 20.1)	6.0% (3.2 to 9.0)	Not reprted
**Armenia**	6.1% (0.1 to 12.8)	22.9% (14.9 to 31.4)	Not reprted	-8.1% (-32.7 to 11.8)	6.7% (0.3 to 18.7)	Not reprted	2.5% (-0.7 to 5.8)	4.9% (-0.3 to 9.3)	Not reprted	0.0% (0.0 to 0.0)	Not reprted	7.2% (-0.2 to 16.5)	4.4% (2.3 to 6.6)	Not reprted
**Australia**	11.6% (0.3 to 23.3)	5.3% (3.2 to 7.8)	Not reprted	-12.0% (-49.7 to 16.3)	2.6% (0.0 to 10.4)	Not reprted	3.5% (-1.1 to 7.6)	6.2% (-0.4 to 11.9)	Not reprted	0.0% (0.0 to 0.0)	Not reprted	7.4% (-0.3 to 16.6)	7.3% (4.0 to 10.8)	Not reprted
**Austria**	11.5% (0.3 to 22.5)	7.2% (5.1 to 9.8)	Not reprted	-10.5% (-42.9 to 14.9)	6.8% (0.3 to 18.9)	Not reprted	1.8% (-0.5 to 4.1)	4.4% (-0.3 to 8.5)	Not reprted	0.0% (0.0 to 0.0)	Not reprted	4.6% (-0.1 to 11.1)	4.5% (2.3 to 6.8)	Not reprted
**Azerbaijan**	4.6% (0.1 to 10.4)	15.9% (8.3 to 25.4)	Not reprted	-4.5% (-18.8 to 7.4)	6.1% (0.3 to 17.6)	Not reprted	1.4% (-0.4 to 3.1)	5.0% (-0.3 to 9.9)	Not reprted	0.0% (0.0 to 0.0)	Not reprted	6.5% (-0.2 to 15.1)	5.0% (2.6 to 7.4)	Not reprted
**Bahamas**	6.1% (0.1 to 13.8)	13.1% (4.5 to 25.9)	Not reprted	-9.6% (-39.4 to 13.7)	4.0% (0.0 to 14.0)	Not reprted	6.7% (-2.1 to 14.1)	6.9% (-0.5 to 13.0)	Not reprted	0.0% (0.0 to 0.1)	Not reprted	10.0% (-0.4 to 21.9)	5.9% (3.1 to 8.9)	Not reprted
**Bahrain**	0.7% (0.0 to 1.6)	34.5% (26.7 to 42.1)	Not reprted	-3.8% (-16.5 to 6.2)	1.6% (0.0 to 8.1)	Not reprted	0.5% (-0.1 to 1.1)	3.4% (-0.2 to 6.4)	Not reprted	0.0% (0.0 to 0.0)	Not reprted	9.4% (-0.5 to 20.1)	9.6% (5.4 to 14.0)	Not reprted
**Bangladesh**	0.4% (0.0 to 1.1)	8.4% (4.3 to 13.3)	Not reprted	-0.2% (-0.8 to 0.4)	5.9% (0.1 to 17.7)	Not reprted	9.6% (-3.2 to 20.0)	10.9% (-0.8 to 20.5)	Not reprted	7.0% (-0.7 to 13.3)	Not reprted	1.2% (-0.1 to 3.2)	5.9% (3.2 to 9.0)	Not reprted
**Barbados**	7.6% (0.2 to 16.4)	16.8% (6.8 to 29.7)	Not reprted	-5.4% (-21.6 to 8.2)	3.0% (0.0 to 11.8)	Not reprted	4.5% (-1.3 to 10.0)	7.9% (-0.6 to 14.8)	Not reprted	0.5% (0.0 to 1.1)	Not reprted	8.9% (-0.3 to 19.7)	7.4% (3.9 to 10.9)	Not reprted
**Belarus**	11.7% (0.3 to 24.0)	11.1% (8.0 to 15.0)	Not reprted	-15.2% (-62.3 to 20.5)	3.8% (0.0 to 13.3)	Not reprted	0.6% (-0.2 to 1.4)	8.3% (-0.6 to 15.2)	Not reprted	0.0% (0.0 to 0.0)	Not reprted	9.4% (-0.3 to 21.0)	3.1% (1.7 to 4.8)	Not reprted
**Belgium**	12.0% (0.3 to 23.7)	7.0% (4.9 to 9.5)	Not reprted	-10.8% (-43.9 to 15.2)	6.2% (0.1 to 17.4)	Not reprted	2.8% (-0.8 to 6.3)	5.7% (-0.4 to 11.0)	Not reprted	0.0% (0.0 to 0.0)	Not reprted	4.9% (-0.1 to 11.6)	5.5% (3.1 to 8.2)	Not reprted
**Belize**	5.8% (0.1 to 12.5)	17.4% (6.7 to 31.9)	Not reprted	-2.1% (-8.6 to 3.7)	4.1% (0.0 to 14.3)	Not reprted	1.9% (-0.5 to 4.2)	2.5% (-0.2 to 4.9)	Not reprted	3.1% (-0.3 to 6.3)	Not reprted	10.0% (-0.4 to 21.2)	4.6% (2.4 to 6.8)	Not reprted
**Benin**	3.5% (0.1 to 8.1)	8.5% (4.6 to 13.5)	Not reprted	-0.6% (-2.7 to 1.1)	4.7% (0.0 to 15.8)	Not reprted	0.1% (0.0 to 0.2)	8.5% (-0.6 to 16.1)	Not reprted	0.7% (-0.1 to 1.6)	Not reprted	4.5% (0.0 to 9.8)	3.7% (1.9 to 5.5)	Not reprted
**Bermuda**	10.4% (0.2 to 21.5)	3.2% (0.6 to 6.1)	Not reprted	-11.0% (-44.6 to 15.3)	4.4% (0.0 to 14.7)	Not reprted	5.2% (-1.6 to 11.2)	6.8% (-0.5 to 13.0)	Not reprted	0.0% (0.0 to 0.0)	Not reprted	10.0% (-0.4 to 21.8)	7.0% (3.9 to 10.3)	Not reprted
**Bhutan**	0.8% (0.0 to 2.4)	19.5% (11.5 to 26.2)	Not reprted	-0.7% (-3.1 to 1.3)	6.0% (0.1 to 18.0)	Not reprted	4.6% (-1.4 to 10.5)	8.0% (-0.6 to 15.4)	Not reprted	1.6% (-0.1 to 3.3)	Not reprted	4.0% (0.0 to 9.8)	4.7% (2.5 to 7.0)	Not reprted
**Bolivia (Plurinational State of)**	5.8% (0.1 to 12.3)	14.8% (7.7 to 23.4)	Not reprted	-8.6% (-34.8 to 12.6)	5.9% (0.1 to 17.3)	Not reprted	3.7% (-1.0 to 8.2)	6.5% (-0.5 to 12.4)	Not reprted	4.3% (-0.4 to 8.7)	Not reprted	6.5% (-0.2 to 15.0)	4.9% (2.6 to 7.5)	Not reprted
**Bosnia and Herzegovina**	7.9% (0.2 to 16.6)	18.0% (10.4 to 23.2)	Not reprted	-2.9% (-11.9 to 5.4)	16.0% (4.4 to 30.3)	Not reprted	0.1% (0.0 to 0.3)	5.7% (-0.4 to 11.0)	Not reprted	0.0% (0.0 to 0.0)	Not reprted	6.3% (-0.2 to 14.8)	6.1% (3.3 to 9.3)	Not reprted
**Botswana**	4.0% (0.1 to 8.7)	13.6% (7.7 to 19.5)	Not reprted	-5.7% (-23.1 to 9.1)	2.7% (0.0 to 11.1)	Not reprted	2.2% (-0.6 to 5.2)	11.2% (-0.8 to 21.3)	Not reprted	4.8% (-0.4 to 9.3)	Not reprted	6.0% (-0.2 to 13.1)	4.0% (2.1 to 6.0)	Not reprted
**Brazil**	6.5% (0.2 to 13.4)	8.0% (4.5 to 12.3)	Not reprted	-14.3% (-59.6 to 19.3)	6.7% (0.3 to 18.3)	Not reprted	3.5% (-1.0 to 7.7)	5.5% (-0.4 to 10.4)	Not reprted	1.3% (-0.1 to 2.6)	Not reprted	8.2% (-0.2 to 18.3)	6.0% (3.3 to 8.9)	Not reprted
**Brunei Darussalam**	0.4% (0.0 to 1.1)	3.9% (0.8 to 7.7)	Not reprted	-4.1% (-16.6 to 6.6)	11.7% (1.8 to 25.2)	Not reprted	5.9% (-1.8 to 12.6)	8.4% (-0.6 to 15.8)	Not reprted	0.9% (-0.1 to 1.9)	Not reprted	5.7% (-0.1 to 12.2)	6.9% (3.8 to 10.3)	Not reprted
**Bulgaria**	12.7% (0.5 to 24.7)	12.5% (9.3 to 15.9)	Not reprted	-12.4% (-50.9 to 16.9)	15.9% (4.6 to 30.3)	Not reprted	4.4% (-1.3 to 9.6)	7.4% (-0.5 to 13.9)	Not reprted	0.0% (0.0 to 0.0)	Not reprted	7.6% (-0.3 to 17.3)	6.0% (3.3 to 8.9)	Not reprted

**Burkina Faso**	7.3% (0.2 to 15.8)	8.4% (4.7 to 13.5)	Not reprted	-3.1% (-12.2 to 5.4)	3.7% (0.0 to 13.5)	Not reprted	0.2% (-0.1 to 0.6)	14.9% (-1.2 to 27.3)	Not reprted	8.7% (-0.8 to 16.7)	Not reprted	0.7% (-0.3 to 2.7)	3.3% (1.7 to 5.0)	Not reprted
**Burundi**	6.5% (0.2 to 13.8)	3.7% (1.9 to 6.1)	Not reprted	-0.1% (-0.6 to 0.3)	6.8% (0.3 to 18.6)	Not reprted	0.4% (-0.1 to 0.9)	5.0% (-0.3 to 9.6)	Not reprted	8.4% (-0.8 to 16.2)	Not reprted	0.4% (-0.3 to 1.8)	2.8% (1.5 to 4.3)	Not reprted
**Cv¥te d'Ivoire**	8.5% (0.2 to 17.9)	12.4% (6.0 to 19.7)	Not reprted	-2.8% (-11.3 to 4.8)	4.1% (0.0 to 14.1)	Not reprted	0.0% (0.0 to 0.1)	7.0% (-0.5 to 13.3)	Not reprted	3.4% (-0.3 to 6.7)	Not reprted	4.6% (0.0 to 10.6)	4.0% (2.1 to 6.1)	Not reprted
**Cabo Verde**	7.5% (0.2 to 15.8)	22.7% (13.4 to 31.5)	Not reprted	-2.5% (-10.3 to 4.1)	3.8% (0.0 to 13.7)	Not reprted	1.7% (-0.5 to 3.7)	8.1% (-0.6 to 15.2)	Not reprted	0.0% (0.0 to 0.1)	Not reprted	4.9% (0.0 to 11.5)	5.8% (3.0 to 8.6)	Not reprted
**Cambodia**	6.2% (0.2 to 13.3)	7.0% (3.4 to 12.2)	Not reprted	-2.2% (-9.2 to 4.1)	11.4% (1.8 to 25.2)	Not reprted	9.3% (-3.0 to 19.5)	10.0% (-0.7 to 19.1)	Not reprted	7.6% (-0.8 to 14.6)	Not reprted	0.8% (-0.2 to 2.3)	3.1% (1.6 to 4.7)	Not reprted
**Cameroon**	8.9% (0.3 to 18.6)	10.5% (5.4 to 17.6)	Not reprted	-2.0% (-8.3 to 3.6)	3.7% (0.0 to 13.3)	Not reprted	0.1% (0.0 to 0.3)	6.0% (-0.4 to 11.4)	Not reprted	0.1% (0.0 to 0.2)	Not reprted	7.3% (-0.2 to 16.7)	5.1% (2.7 to 7.8)	Not reprted
**Canada**	9.5% (0.3 to 19.9)	2.3% (0.8 to 4.1)	Not reprted	-10.5% (-43.2 to 14.7)	5.5% (0.2 to 16.1)	Not reprted	2.0% (-0.6 to 4.5)	5.9% (-0.4 to 11.4)	Not reprted	0.0% (0.0 to 0.0)	Not reprted	7.2% (-0.3 to 16.0)	4.3% (2.3 to 6.5)	Not reprted
**Central African Republic**	3.2% (0.0 to 7.7)	4.6% (2.7 to 7.1)	Not reprted	-8.4% (-33.6 to 12.0)	2.8% (0.0 to 11.4)	Not reprted	1.9% (-0.6 to 4.3)	8.4% (-0.6 to 16.1)	Not reprted	11.4% (-1.1 to 21.5)	Not reprted	2.2% (0.0 to 5.4)	5.6% (3.1 to 8.5)	Not reprted
**Chad**	4.7% (0.1 to 12.1)	8.8% (4.9 to 14.0)	Not reprted	-3.1% (-12.5 to 5.2)	3.6% (0.0 to 13.4)	Not reprted	0.8% (-0.2 to 1.8)	13.3% (-1.0 to 24.7)	Not reprted	11.2% (-1.1 to 21.0)	Not reprted	2.1% (0.0 to 5.0)	4.3% (2.2 to 6.3)	Not reprted
**Chile**	10.6% (0.3 to 21.2)	16.2% (10.2 to 23.0)	Not reprted	-11.8% (-49.0 to 16.2)	7.2% (0.3 to 19.6)	Not reprted	3.6% (-1.0 to 7.7)	6.7% (-0.5 to 12.8)	Not reprted	0.0% (0.0 to 0.0)	Not reprted	9.5% (-0.4 to 20.8)	5.5% (2.9 to 8.1)	Not reprted
**China**	7.3% (0.1 to 15.0)	23.9% (15.9 to 29.5)	Not reprted	-9.6% (-39.1 to 13.4)	17.8% (6.6 to 32.2)	Not reprted	1.8% (-0.5 to 3.9)	5.9% (-0.4 to 11.2)	Not reprted	0.0% (0.0 to 0.0)	Not reprted	2.6% (0.0 to 6.5)	5.5% (2.9 to 8.3)	Not reprted
**Colombia**	4.0% (0.1 to 8.4)	9.9% (6.3 to 13.9)	Not reprted	-7.3% (-29.3 to 10.8)	12.5% (2.8 to 26.1)	Not reprted	3.7% (-1.1 to 8.0)	5.6% (-0.4 to 10.6)	Not reprted	0.8% (-0.1 to 1.8)	Not reprted	6.9% (-0.2 to 15.7)	4.7% (2.5 to 7.2)	Not reprted
**Comoros**	0.6% (0.0 to 1.6)	2.6% (1.4 to 4.4)	Not reprted	-0.7% (-2.9 to 1.3)	7.0% (0.3 to 19.3)	Not reprted	2.3% (-0.7 to 5.0)	6.3% (-0.4 to 12.0)	Not reprted	10.8% (-1.0 to 20.4)	Not reprted	3.4% (0.0 to 7.8)	2.7% (1.4 to 4.1)	Not reprted
**Congo**	7.3% (0.2 to 16.0)	13.5% (5.9 to 22.2)	Not reprted	-2.7% (-10.9 to 4.8)	2.8% (0.0 to 11.7)	Not reprted	4.7% (-1.3 to 10.1)	7.6% (-0.6 to 14.5)	Not reprted	10.1% (-1.0 to 19.4)	Not reprted	4.8% (0.0 to 10.5)	4.2% (2.1 to 6.4)	Not reprted
**Cook Islands**	10.4% (0.3 to 22.3)	2.6% (0.0 to 6.4)	Not reprted	-8.0% (-32.0 to 10.9)	8.6% (0.9 to 20.5)	Not reprted	0.0% (0.0 to 0.1)	6.4% (-0.4 to 12.2)	Not reprted	2.3% (-0.2 to 4.8)	Not reprted	14.0% (-0.8 to 28.1)	10.8% (5.9 to 16.0)	Not reprted
**Costa Rica**	5.9% (0.1 to 12.8)	9.0% (5.9 to 12.5)	Not reprted	-6.0% (-24.1 to 9.1)	8.9% (0.7 to 22.3)	Not reprted	5.4% (-1.6 to 11.5)	5.4% (-0.4 to 10.2)	Not reprted	2.6% (-0.3 to 5.3)	Not reprted	8.0% (-0.3 to 18.3)	5.7% (3.1 to 8.6)	Not reprted
**Croatia**	11.5% (0.3 to 23.6)	11.3% (8.3 to 14.6)	Not reprted	-6.8% (-27.5 to 10.6)	17.1% (5.2 to 31.5)	Not reprted	4.5% (-1.3 to 9.7)	5.5% (-0.4 to 10.7)	Not reprted	0.0% (0.0 to 0.0)	Not reprted	7.7% (-0.3 to 17.3)	6.7% (3.6 to 10.2)	Not reprted
**Cuba**	6.7% (0.1 to 14.4)	14.5% (6.7 to 24.3)	Not reprted	-6.7% (-27.5 to 10.0)	4.0% (0.0 to 14.0)	Not reprted	0.3% (-0.1 to 0.7)	5.3% (-0.4 to 10.2)	Not reprted	0.0% (0.0 to 0.0)	Not reprted	7.1% (-0.2 to 15.9)	6.2% (3.3 to 9.2)	Not reprted
**Cyprus**	9.2% (0.2 to 18.4)	10.5% (7.5 to 13.7)	Not reprted	-7.1% (-29.8 to 10.7)	4.4% (0.0 to 14.8)	Not reprted	4.8% (-1.5 to 10.4)	4.8% (-0.3 to 9.5)	Not reprted	0.1% (0.0 to 0.2)	Not reprted	4.7% (-0.1 to 11.2)	5.0% (2.6 to 7.5)	Not reprted
**Czechia**	13.9% (0.5 to 27.3)	9.7% (7.2 to 12.7)	Not reprted	-10.1% (-41.1 to 14.0)	16.3% (4.7 to 30.6)	Not reprted	3.8% (-1.2 to 8.3)	6.9% (-0.5 to 13.1)	Not reprted	0.1% (0.0 to 0.2)	Not reprted	7.8% (-0.3 to 17.8)	6.8% (3.8 to 10.2)	Not reprted
**Democratic People's Republic of Korea**	5.5% (0.1 to 11.4)	5.9% (3.7 to 8.6)	Not reprted	-1.6% (-6.4 to 2.6)	15.2% (4.4 to 29.7)	Not reprted	4.3% (-1.2 to 9.3)	7.4% (-0.5 to 14.1)	Not reprted	0.0% (0.0 to 0.0)	Not reprted	0.2% (-0.5 to 1.8)	5.1% (2.8 to 7.8)	Not reprted

**Democratic Republic of the Congo**	3.8%<br>(0.1 to 8.9)	4.1%<br>(2.6 to 6.0)	Not reprted	-0.2%<br>(-0.8 to 0.4)	1.5%<br>(0.0 to 8.1)	Not reprted	3.2%<br>(-0.9 to 7.0)	8.4%<br>(-0.6 to 15.9)	Not reprted	10.9%<br>(-1.0 to 20.7)	Not reprted	3.1%<br>(0.0 to 7.2)	6.3%<br>(3.3 to 9.4)	Not reprted
**Denmark**	12.3%<br>(0.2 to 24.3)	5.5%<br>(3.3 to 8.2)	Not reprted	-10.3%<br>(-42.5 to 14.7)	4.9%<br>(0.0 to 15.8)	Not reprted	2.4%<br>(-0.7 to 5.4)	4.6%<br>(-0.3 to 9.1)	Not reprted	0.0%<br>(0.0 to 0.0)	Not reprted	4.5%<br>(-0.1 to 10.8)	4.6%<br>(2.4 to 7.0)	Not reprted
**Djibouti**	0.2%<br>(-0.1 to 0.6)	19.9%<br>(9.9 to 34.0)	Not reprted	-4.1%<br>(-17.3 to 6.6)	6.8%<br>(0.2 to 19.0)	Not reprted	5.2%<br>(-1.6 to 11.1)	12.4%<br>(-0.9 to 23.2)	Not reprted	1.8%<br>(-0.2 to 3.6)	Not reprted	0.5%<br>(-0.4 to 2.1)	3.1%<br>(1.6 to 4.7)	Not reprted
**Dominica**	7.1%<br>(0.1 to 15.3)	14.1%<br>(5.5 to 25.7)	Not reprted	-4.7%<br>(-18.8 to 7.6)	4.2%<br>(0.0 to 14.9)	Not reprted	0.4%<br>(-0.1 to 1.0)	1.1%<br>(-0.1 to 2.3)	Not reprted	0.1%<br>(0.0 to 0.2)	Not reprted	9.9%<br>(-0.4 to 21.4)	6.8%<br>(3.5 to 9.9)	Not reprted
**Dominican Republic**	6.4%<br>(0.1 to 13.8)	13.6%<br>(4.8 to 24.8)	Not reprted	-4.3%<br>(-17.7 to 6.6)	3.9%<br>(0.0 to 13.6)	Not reprted	4.3%<br>(-1.3 to 8.9)	2.6%<br>(-0.2 to 4.9)	Not reprted	0.5%<br>(-0.1 to 1.0)	Not reprted	7.0%<br>(-0.2 to 15.9)	5.0%<br>(2.7 to 7.7)	Not reprted
**Ecuador**	4.4%<br>(0.1 to 9.8)	12.3%<br>(7.6 to 18.7)	Not reprted	-8.5%<br>(-36.0 to 12.4)	6.1%<br>(0.2 to 17.3)	Not reprted	7.2%<br>(-2.3 to 15.0)	3.8%<br>(-0.3 to 7.3)	Not reprted	4.1%<br>(-0.4 to 8.3)	Not reprted	9.4%<br>(-0.4 to 20.4)	6.6%<br>(3.5 to 9.9)	Not reprted
**Egypt**	0.2%<br>(0.0 to 0.6)	35.8%<br>(27.1 to 43.9)	Not reprted	-3.2%<br>(-13.2 to 5.3)	1.7%<br>(0.0 to 8.1)	Not reprted	0.1%<br>(0.0 to 0.3)	3.8%<br>(-0.2 to 7.3)	Not reprted	0.0%<br>(0.0 to 0.0)	Not reprted	11.7%<br>(-0.5 to 24.2)	5.1%<br>(2.7 to 7.8)	Not reprted
**El Salvador**	4.0%<br>(0.1 to 8.3)	12.0%<br>(6.4 to 18.7)	Not reprted	-1.4%<br>(-5.7 to 2.6)	7.9%<br>(0.6 to 20.5)	Not reprted	0.8%<br>(-0.2 to 1.7)	7.4%<br>(-0.5 to 13.8)	Not reprted	0.8%<br>(-0.1 to 1.7)	Not reprted	9.8%<br>(-0.4 to 21.4)	5.1%<br>(2.6 to 7.6)	Not reprted
**Equatorial Guinea**	7.2%<br>(0.2 to 16.0)	21.8%<br>(12.1 to 32.8)	Not reprted	-4.9%<br>(-20.1 to 7.8)	2.9%<br>(0.0 to 11.3)	Not reprted	0.5%<br>(-0.1 to 1.1)	5.6%<br>(-0.4 to 10.8)	Not reprted	6.1%<br>(-0.6 to 11.9)	Not reprted	5.8%<br>(-0.1 to 12.8)	7.8%<br>(4.2 to 11.4)	Not reprted
**Eritrea**	1.5%<br>(0.0 to 3.7)	7.3%<br>(4.0 to 12.2)	Not reprted	-1.4%<br>(-6.2 to 2.6)	6.7%<br>(0.2 to 18.4)	Not reprted	1.2%<br>(-0.4 to 2.7)	8.5%<br>(-0.6 to 16.0)	Not reprted	9.0%<br>(-0.9 to 17.3)	Not reprted	0.8%<br>(-0.1 to 2.4)	2.5%<br>(1.3 to 3.8)	Not reprted
**Estonia**	12.3%<br>(0.4 to 25.5)	2.9%<br>(1.0 to 5.3)	Not reprted	-9.6%<br>(-39.5 to 14.4)	2.5%<br>(0.0 to 10.3)	Not reprted	1.1%<br>(-0.3 to 2.5)	7.6%<br>(-0.6 to 14.2)	Not reprted	0.0%<br>(0.0 to 0.0)	Not reprted	8.6%<br>(-0.2 to 18.9)	4.5%<br>(2.4 to 6.9)	Not reprted
**Eswatini**	4.7%<br>(0.1 to 10.9)	13.1%<br>(5.8 to 20.6)	Not reprted	-6.2%<br>(-24.2 to 9.5)	2.8%<br>(0.0 to 11.3)	Not reprted	2.2%<br>(-0.6 to 4.8)	8.1%<br>(-0.6 to 15.3)	Not reprted	8.9%<br>(-0.9 to 16.9)	Not reprted	9.4%<br>(-0.4 to 20.1)	4.5%<br>(2.3 to 6.8)	Not reprted
**Ethiopia**	5.2%<br>(0.2 to 11.6)	5.6%<br>(3.4 to 7.9)	Not reprted	-1.2%<br>(-5.0 to 2.1)	6.8%<br>(0.3 to 18.4)	Not reprted	0.4%<br>(-0.1 to 0.9)	13.8%<br>(-1.0 to 25.7)	Not reprted	8.0%<br>(-0.8 to 15.2)	Not reprted	0.9%<br>(-0.2 to 2.6)	3.1%<br>(1.6 to 4.8)	Not reprted
**Fiji**	2.9%<br>(0.1 to 6.7)	8.5%<br>(2.8 to 18.6)	Not reprted	-7.9%<br>(-32.2 to 12.1)	8.8%<br>(1.0 to 20.9)	Not reprted	0.9%<br>(-0.2 to 1.9)	9.9%<br>(-0.7 to 18.3)	Not reprted	2.5%<br>(-0.3 to 5.2)	Not reprted	10.5%<br>(-0.5 to 22.0)	10.4%<br>(5.5 to 14.9)	Not reprted
**Finland**	10.4%<br>(0.2 to 20.7)	1.5%<br>(0.2 to 3.2)	Not reprted	-10.1%<br>(-41.0 to 14.3)	5.0%<br>(0.1 to 15.5)	Not reprted	2.4%<br>(-0.7 to 5.4)	6.5%<br>(-0.5 to 12.5)	Not reprted	0.1%<br>(0.0 to 0.1)	Not reprted	5.6%<br>(-0.1 to 13.2)	5.6%<br>(3.1 to 8.4)	Not reprted
**France**	12.7%<br>(0.4 to 24.9)	5.7%<br>(3.8 to 8.1)	Not reprted	-11.7%<br>(-48.5 to 16.1)	3.9%<br>(0.0 to 13.7)	Not reprted	3.8%<br>(-1.1 to 8.6)	6.4%<br>(-0.5 to 12.4)	Not reprted	0.1%<br>(0.0 to 0.2)	Not reprted	5.0%<br>(-0.1 to 11.7)	4.9%<br>(2.7 to 7.5)	Not reprted
**Gabon**	8.8%<br>(0.3 to 19.4)	19.5%<br>(11.2 to 29.5)	Not reprted	-9.8%<br>(-41.2 to 13.6)	2.9%<br>(0.0 to 12.2)	Not reprted	1.2%<br>(-0.3 to 2.8)	4.0%<br>(-0.3 to 7.7)	Not reprted	5.5%<br>(-0.6 to 10.9)	Not reprted	6.9%<br>(-0.2 to 15.3)	7.0%<br>(3.8 to 10.3)	Not reprted
**Gambia**	4.6%<br>(0.1 to 10.0)	9.2%<br>(5.2 to 14.3)	Not reprted	-0.6%<br>(-2.7 to 1.2)	3.8%<br>(0.0 to 13.7)	Not reprted	1.9%<br>(-0.5 to 4.1)	15.5%<br>(-1.2 to 28.3)	Not reprted	8.1%<br>(-0.8 to 15.7)	Not reprted	4.5%<br>(0.0 to 10.3)	4.1%<br>(2.1 to 6.2)	Not reprted
**Georgia**	8.3%<br>(0.2 to 18.1)	12.4%<br>(6.8 to 18.3)	Not reprted	-3.9%<br>(-15.5 to 6.3)	6.9%<br>(0.3 to 18.5)	Not reprted	2.3%<br>(-0.7 to 5.2)	7.1%<br>(-0.5 to 13.5)	Not reprted	0.0%<br>(0.0 to 0.0)	Not reprted	6.2%<br>(-0.1 to 14.5)	3.9%<br>(2.0 to 5.8)	Not reprted
**Germany**	13.9%<br>(0.5 to 27.5)	6.5%<br>(4.4 to 8.9)	Not reprted	-11.0%<br>(-45.4 to 15.7)	5.3%<br>(0.1 to 16.0)	Not reprted	3.0%<br>(-0.9 to 6.6)	6.1%<br>(-0.4 to 11.6)	Not reprted	0.0%<br>(0.0 to 0.1)	Not reprted	5.8%<br>(-0.1 to 13.6)	4.5%<br>(2.4 to 6.7)	Not reprted
**Ghana**	6.3%<br>(0.1 to 13.9)	16.7%<br>(8.0 to 26.8)	Not reprted	-1.2%<br>(-5.0 to 2.0)	5.6%<br>(0.1 to 17.1)	Not reprted	0.0%<br>(0.0 to 0.0)	4.2%<br>(-0.3 to 8.1)	Not reprted	1.9%<br>(-0.2 to 3.9)	Not reprted	4.7%<br>(0.0 to 10.6)	5.8%<br>(3.1 to 8.6)	Not reprted

**Greece**	9.8% (0.2 to 19.9)	10.4% (7.6 to 13.5)	Not reprted	-11.3% (-46.8 to 15.9)	4.6% (0.1 to 15.2)	Not reprted	1.9% (-0.5 to 4.2)	3.0% (-0.2 to 6.1)	Not reprted	0.0% (0.0 to 0.0)	Not reprted	6.4% (-0.2 to 14.9)	5.5% (2.8 to 8.2)	Not reprted
**Greenland**	9.8% (0.2 to 20.8)	2.6% (0.1 to 6.8)	Not reprted	-12.2% (-51.2 to 16.9)	5.5% (0.1 to 17.1)	Not reprted	2.2% (-0.6 to 5.0)	5.7% (-0.4 to 11.1)	Not reprted	0.0% (0.0 to 0.0)	Not reprted	7.4% (-0.2 to 16.7)	3.2% (1.6 to 4.8)	Not reprted
**Grenada**	6.8% (0.2 to 14.9)	17.3% (6.8 to 32.3)	Not reprted	-1.8% (-7.0 to 3.2)	4.1% (0.0 to 14.0)	Not reprted	3.0% (-0.9 to 6.5)	5.3% (-0.4 to 10.2)	Not reprted	5.4% (-0.5 to 10.6)	Not reprted	6.8% (-0.2 to 15.0)	5.7% (3.0 to 8.4)	Not reprted
**Guam**	5.2% (0.0 to 13.1)	5.7% (2.7 to 9.1)	Not reprted	-10.7% (-43.0 to 14.9)	7.2% (0.8 to 18.1)	Not reprted	0.0% (0.0 to 0.0)	6.6% (-0.4 to 12.4)	Not reprted	1.1% (-0.1 to 2.3)	Not reprted	11.5% (-0.5 to 24.1)	7.8% (4.0 to 11.8)	Not reprted
**Guatemala**	2.9% (0.1 to 6.6)	11.4% (4.8 to 17.6)	Not reprted	-1.2% (-5.0 to 2.3)	7.7% (0.5 to 20.1)	Not reprted	0.8% (-0.2 to 1.8)	7.1% (-0.5 to 13.3)	Not reprted	0.9% (-0.1 to 1.9)	Not reprted	8.3% (-0.3 to 18.1)	4.7% (2.5 to 7.0)	Not reprted
**Guinea**	2.1% (0.0 to 4.7)	7.6% (4.3 to 11.8)	Not reprted	-1.1% (-4.4 to 2.0)	3.7% (0.0 to 13.3)	Not reprted	1.7% (-0.5 to 3.8)	6.3% (-0.5 to 12.0)	Not reprted	4.0% (-0.4 to 8.0)	Not reprted	3.0% (0.0 to 7.2)	3.9% (2.0 to 5.9)	Not reprted
**Guinea-Bissau**	4.9% (0.1 to 10.0)	8.0% (4.5 to 12.5)	Not reprted	-3.2% (-13.4 to 5.6)	3.7% (0.0 to 13.6)	Not reprted	3.7% (-1.0 to 8.2)	8.3% (-0.6 to 15.5)	Not reprted	11.0% (-1.1 to 20.7)	Not reprted	3.4% (0.0 to 7.8)	3.8% (2.0 to 5.7)	Not reprted
**Guyana**	6.2% (0.1 to 13.1)	17.7% (7.3 to 32.4)	Not reprted	-0.6% (-2.4 to 1.0)	3.9% (0.0 to 13.7)	Not reprted	4.0% (-1.2 to 8.7)	8.0% (-0.6 to 14.9)	Not reprted	0.8% (-0.1 to 1.8)	Not reprted	6.7% (-0.1 to 15.1)	8.6% (4.4 to 12.7)	Not reprted
**Haiti**	5.6% (0.1 to 12.0)	4.1% (1.7 to 7.7)	Not reprted	-1.3% (-5.1 to 2.2)	3.8% (0.0 to 13.4)	Not reprted	3.5% (-1.0 to 7.5)	7.2% (-0.5 to 13.5)	Not reprted	10.1% (-1.0 to 19.0)	Not reprted	2.4% (0.0 to 5.9)	4.4% (2.3 to 6.6)	Not reprted
**Honduras**	3.1% (0.1 to 6.7)	8.1% (3.7 to 13.3)	Not reprted	-1.8% (-7.3 to 3.2)	8.1% (0.5 to 21.1)	Not reprted	1.4% (-0.4 to 3.2)	6.6% (-0.5 to 12.6)	Not reprted	1.5% (-0.1 to 2.9)	Not reprted	6.5% (-0.2 to 15.3)	4.7% (2.5 to 7.1)	Not reprted
**Hungary**	12.2% (0.4 to 24.4)	10.2% (7.5 to 13.2)	Not reprted	-10.0% (-40.8 to 14.6)	19.5% (7.4 to 33.7)	Not reprted	4.1% (-1.2 to 8.8)	7.4% (-0.5 to 14.1)	Not reprted	0.0% (0.0 to 0.0)	Not reprted	9.7% (-0.3 to 20.7)	5.5% (2.9 to 8.3)	Not reprted
**Iceland**	10.1% (0.1 to 20.3)	1.5% (0.3 to 3.4)	Not reprted	-11.3% (-46.3 to 15.4)	5.1% (0.1 to 15.4)	Not reprted	4.9% (-1.4 to 10.5)	6.0% (-0.4 to 11.6)	Not reprted	0.2% (0.0 to 0.5)	Not reprted	5.9% (-0.2 to 13.9)	5.9% (3.2 to 8.7)	Not reprted
**India**	3.9% (0.1 to 8.8)	18.3% (11.0 to 24.5)	Not reprted	-0.2% (-0.7 to 0.3)	6.7% (0.4 to 19.0)	Not reprted	3.0% (-0.8 to 6.4)	14.9% (-1.1 to 27.4)	Not reprted	1.0% (-0.1 to 2.0)	Not reprted	1.6% (0.0 to 3.8)	5.8% (3.1 to 8.7)	Not reprted
**Indonesia**	0.4% (0.0 to 1.1)	12.1% (7.2 to 16.6)	Not reprted	-0.6% (-2.5 to 1.1)	12.1% (2.4 to 25.6)	Not reprted	5.8% (-1.7 to 12.0)	7.6% (-0.5 to 14.1)	Not reprted	2.1% (-0.2 to 4.3)	Not reprted	1.8% (0.0 to 4.7)	4.1% (2.1 to 6.2)	Not reprted
**Iran (Islamic Republic of)**	0.7% (0.0 to 1.7)	22.2% (17.6 to 26.7)	Not reprted	-2.5% (-10.2 to 4.2)	1.7% (0.0 to 7.8)	Not reprted	1.2% (-0.3 to 2.6)	3.3% (-0.2 to 6.3)	Not reprted	0.0% (0.0 to 0.0)	Not reprted	8.3% (-0.3 to 18.3)	5.2% (2.8 to 7.7)	Not reprted
**Iraq**	0.2% (0.0 to 0.5)	25.1% (18.2 to 32.6)	Not reprted	-0.6% (-2.4 to 1.0)	1.8% (0.0 to 8.2)	Not reprted	2.9% (-0.8 to 6.4)	7.0% (-0.5 to 13.1)	Not reprted	0.0% (0.0 to 0.0)	Not reprted	9.5% (-0.4 to 20.9)	7.6% (4.0 to 11.1)	Not reprted
**Ireland**	10.6% (0.3 to 21.4)	3.8% (2.0 to 6.1)	Not reprted	-11.8% (-48.5 to 16.2)	3.2% (0.0 to 12.1)	Not reprted	1.8% (-0.5 to 3.9)	5.6% (-0.4 to 10.9)	Not reprted	0.0% (0.0 to 0.0)	Not reprted	5.7% (-0.1 to 13.2)	5.5% (2.9 to 8.3)	Not reprted
**Israel**	4.4% (0.0 to 9.9)	14.2% (10.9 to 18.0)	Not reprted	-7.5% (-30.3 to 11.4)	4.9% (0.1 to 15.7)	Not reprted	0.7% (-0.2 to 1.6)	3.1% (-0.2 to 6.2)	Not reprted	0.0% (0.0 to 0.0)	Not reprted	5.3% (-0.1 to 12.5)	5.6% (3.0 to 8.5)	Not reprted
**Italy**	11.3% (0.3 to 22.7)	9.7% (7.3 to 12.6)	Not reprted	-9.7% (-39.9 to 13.6)	6.4% (0.5 to 17.6)	Not reprted	2.6% (-0.7 to 5.7)	3.8% (-0.2 to 7.4)	Not reprted	0.0% (0.0 to 0.0)	Not reprted	4.4% (-0.1 to 10.8)	5.8% (3.2 to 8.6)	Not reprted
**Jamaica**	4.9% (0.1 to 10.7)	11.9% (6.3 to 19.2)	Not reprted	-1.9% (-7.8 to 3.3)	4.1% (0.0 to 13.8)	Not reprted	1.8% (-0.5 to 4.1)	6.2% (-0.4 to 11.9)	Not reprted	0.2% (0.0 to 0.5)	Not reprted	8.0% (-0.3 to 17.5)	3.6% (1.8 to 5.4)	Not reprted
**Japan**	10.4% (0.2 to 20.8)	8.4% (4.7 to 13.2)	Not reprted	-5.1% (-20.4 to 7.9)	9.7% (1.0 to 23.0)	Not reprted	3.7% (-1.0 to 7.9)	8.6% (-0.6 to 15.9)	Not reprted	0.0% (0.0 to 0.0)	Not reprted	1.7% (-0.1 to 4.7)	6.5% (3.5 to 9.5)	Not reprted

**Jordan**	0.3% (0.0 to 0.7)	20.1% (15.4 to 25.3)	Not reprted	-2.4% (-10.1 to 4.3)	1.5% (0.0 to 7.1)	Not reprted	2.8% (-0.8 to 6.2)	6.1% (-0.4 to 11.4)	Not reprted	0.0% (0.0 to 0.0)	Not reprted	10.9% (-0.5 to 22.8)	7.4% (3.9 to 10.9)	Not reprted
**Kazakhstan**	7.0% (0.2 to 15.1)	15.3% (10.3 to 21.3)	Not reprted	-12.1% (-49.5 to 16.7)	6.5% (0.3 to 18.1)	Not reprted	2.5% (-0.7 to 5.5)	7.1% (-0.5 to 13.4)	Not reprted	0.0% (0.0 to 0.0)	Not reprted	7.0% (-0.2 to 15.6)	4.3% (2.3 to 6.3)	Not reprted
**Kenya**	6.4% (0.2 to 13.8)	5.6% (3.0 to 8.9)	Not reprted	-3.0% (-12.3 to 4.9)	4.3% (0.3 to 12.5)	Not reprted	0.8% (-0.2 to 1.7)	7.6% (-0.5 to 14.4)	Not reprted	1.8% (-0.2 to 3.6)	Not reprted	3.0% (0.0 to 7.0)	3.4% (1.8 to 5.2)	Not reprted
**Kiribati**	1.1% (0.0 to 3.8)	2.7% (1.0 to 6.1)	Not reprted	-2.7% (-11.5 to 4.6)	7.7% (0.8 to 18.8)	Not reprted	1.5% (-0.4 to 3.3)	8.2% (-0.6 to 15.0)	Not reprted	5.4% (-0.6 to 10.8)	Not reprted	11.7% (-0.5 to 23.9)	8.9% (4.9 to 13.4)	Not reprted
**Kuwait**	0.1% (0.0 to 0.3)	29.1% (23.0 to 35.0)	Not reprted	-8.1% (-32.6 to 11.1)	3.6% (0.0 to 12.2)	Not reprted	2.1% (-0.6 to 4.6)	5.8% (-0.4 to 10.8)	Not reprted	0.0% (0.0 to 0.0)	Not reprted	12.3% (-0.7 to 24.7)	7.2% (4.0 to 10.7)	Not reprted
**Kyrgyzstan**	5.9% (0.1 to 12.6)	12.3% (6.0 to 19.7)	Not reprted	-10.7% (-43.3 to 15.5)	6.0% (0.3 to 17.8)	Not reprted	2.3% (-0.7 to 5.3)	8.7% (-0.7 to 16.4)	Not reprted	0.0% (0.0 to 0.0)	Not reprted	8.5% (-0.3 to 19.1)	3.8% (2.0 to 5.8)	Not reprted
**Lao People's Democratic Republic**	8.5% (0.2 to 17.7)	10.4% (4.2 to 16.9)	Not reprted	-4.0% (-15.6 to 6.2)	11.7% (2.0 to 25.7)	Not reprted	8.4% (-2.7 to 17.6)	7.0% (-0.5 to 13.1)	Not reprted	0.6% (-0.1 to 1.3)	Not reprted	1.8% (0.0 to 4.9)	3.8% (2.0 to 5.8)	Not reprted
**Latvia**	12.9% (0.4 to 26.1)	8.3% (5.6 to 11.5)	Not reprted	-9.2% (-37.2 to 13.4)	5.3% (0.1 to 15.9)	Not reprted	3.0% (-0.9 to 6.7)	8.5% (-0.6 to 15.9)	Not reprted	0.0% (0.0 to 0.0)	Not reprted	8.4% (-0.3 to 18.5)	4.6% (2.4 to 6.9)	Not reprted
**Lebanon**	2.0% (0.0 to 4.8)	14.2% (9.1 to 20.3)	Not reprted	-5.7% (-23.2 to 8.6)	1.7% (0.0 to 7.9)	Not reprted	0.7% (-0.2 to 1.6)	3.6% (-0.2 to 6.9)	Not reprted	0.0% (0.0 to 0.0)	Not reprted	9.5% (-0.4 to 20.3)	7.0% (3.6 to 10.6)	Not reprted
**Lesotho**	5.4% (0.1 to 11.8)	6.1% (3.1 to 10.2)	Not reprted	-4.2% (-17.3 to 6.8)	2.7% (0.0 to 11.1)	Not reprted	0.0% (0.0 to 0.1)	12.4% (-0.9 to 23.1)	Not reprted	8.3% (-0.8 to 16.0)	Not reprted	6.4% (-0.2 to 14.1)	3.5% (1.8 to 5.3)	Not reprted
**Liberia**	5.3% (0.2 to 11.2)	5.7% (3.7 to 8.0)	Not reprted	-0.6% (-2.3 to 1.0)	3.7% (0.0 to 13.5)	Not reprted	6.1% (-1.9 to 13.3)	8.5% (-0.6 to 16.0)	Not reprted	8.7% (-0.9 to 16.8)	Not reprted	7.2% (-0.2 to 15.9)	4.9% (2.5 to 7.3)	Not reprted
**Libya**	0.3% (0.0 to 0.6)	19.9% (13.9 to 27.0)	Not reprted	-2.7% (-11.1 to 4.5)	1.3% (0.0 to 6.7)	Not reprted	2.1% (-0.6 to 4.5)	5.4% (-0.4 to 9.9)	Not reprted	0.0% (0.0 to 0.0)	Not reprted	10.9% (-0.6 to 23.0)	4.1% (2.2 to 6.3)	Not reprted
**Lithuania**	12.3% (0.4 to 25.4)	6.2% (3.9 to 9.0)	Not reprted	-11.7% (-47.1 to 17.0)	4.5% (0.0 to 15.3)	Not reprted	1.2% (-0.3 to 2.8)	7.7% (-0.6 to 14.5)	Not reprted	0.0% (0.0 to 0.0)	Not reprted	8.3% (-0.3 to 18.6)	4.1% (2.1 to 6.2)	Not reprted
**Luxembourg**	12.8% (0.3 to 24.7)	4.4% (2.5 to 6.6)	Not reprted	-10.9% (-44.4 to 15.0)	4.7% (0.1 to 15.3)	Not reprted	2.8% (-0.7 to 6.1)	5.5% (-0.4 to 10.8)	Not reprted	0.0% (0.0 to 0.1)	Not reprted	5.3% (-0.1 to 12.7)	5.5% (2.8 to 8.2)	Not reprted
**Madagascar**	2.5% (0.0 to 6.0)	2.3% (1.4 to 3.6)	Not reprted	-2.2% (-8.7 to 3.8)	6.7% (0.2 to 18.5)	Not reprted	3.8% (-1.1 to 8.3)	8.6% (-0.6 to 16.1)	Not reprted	10.5% (-1.0 to 19.7)	Not reprted	1.6% (0.0 to 4.0)	2.7% (1.4 to 4.2)	Not reprted
**Malawi**	4.0% (0.1 to 8.4)	3.5% (2.1 to 5.5)	Not reprted	-0.7% (-3.1 to 1.5)	6.9% (0.3 to 18.6)	Not reprted	0.1% (0.0 to 0.1)	7.6% (-0.6 to 14.4)	Not reprted	8.0% (-0.8 to 15.4)	Not reprted	1.7% (-0.1 to 4.6)	2.7% (1.4 to 4.3)	Not reprted
**Malaysia**	1.7% (0.0 to 3.8)	12.8% (8.5 to 18.1)	Not reprted	-2.0% (-8.3 to 3.5)	12.4% (2.1 to 26.3)	Not reprted	6.1% (-1.8 to 13.0)	6.6% (-0.5 to 12.5)	Not reprted	0.6% (-0.1 to 1.3)	Not reprted	4.4% (0.0 to 9.8)	7.0% (3.7 to 10.5)	Not reprted
**Maldives**	1.1% (0.0 to 3.2)	8.1% (4.7 to 12.3)	Not reprted	-0.4% (-1.6 to 0.8)	11.9% (2.1 to 25.3)	Not reprted	5.8% (-1.8 to 12.3)	7.3% (-0.5 to 13.7)	Not reprted	0.1% (0.0 to 0.2)	Not reprted	3.7% (0.0 to 8.7)	4.3% (2.2 to 6.5)	Not reprted
**Mali**	1.5% (0.1 to 3.4)	9.3% (5.1 to 14.9)	Not reprted	-6.0% (-25.1 to 9.5)	3.5% (0.0 to 12.9)	Not reprted	0.4% (-0.1 to 0.8)	8.6% (-0.6 to 16.1)	Not reprted	6.6% (-0.7 to 12.9)	Not reprted	2.2% (0.0 to 5.4)	6.1% (3.3 to 9.2)	Not reprted
**Malta**	8.3% (0.2 to 16.9)	8.1% (5.7 to 10.9)	Not reprted	-9.4% (-39.0 to 13.1)	6.4% (0.4 to 17.7)	Not reprted	1.9% (-0.5 to 4.3)	6.1% (-0.4 to 11.5)	Not reprted	0.0% (0.0 to 0.0)	Not reprted	5.5% (-0.1 to 13.2)	4.9% (2.5 to 7.3)	Not reprted
**Marshall Islands**	2.8% (0.1 to 6.7)	4.8% (1.7 to 10.4)	Not reprted	-5.4% (-23.0 to 8.8)	7.9% (0.9 to 18.9)	Not reprted	0.6% (-0.1 to 1.3)	8.7% (-0.6 to 16.3)	Not reprted	8.0% (-0.9 to 15.5)	Not reprted	11.7% (-0.6 to 24.5)	9.6% (5.2 to 14.1)	Not reprted

**Mauritania**	0.0%<br>(0.0 to 0.0)	20.7%<br>(11.1 to 31.3)	Not reprted	-5.8%<br>(-24.1 to 9.1)	3.7%<br>(0.0 to 13.3)	Not reprted	1.2%<br>(-0.3 to 2.8)	13.3%<br>(-1.0 to 24.8)	Not reprted	7.9%<br>(-0.8 to 15.0)	Not reprted	7.0%<br>(-0.2 to 15.6)	4.3%<br>(2.3 to 6.4)	Not reprted
**Mauritius**	6.3%<br>(0.1 to 13.5)	5.2%<br>(1.7 to 9.4)	Not reprted	-3.3%<br>(-13.0 to 5.8)	11.6%<br>(1.9 to 25.5)	Not reprted	5.0%<br>(-1.6 to 10.9)	10.5%<br>(-0.8 to 19.1)	Not reprted	0.1%<br>(0.0 to 0.3)	Not reprted	6.0%<br>(0.0 to 13.7)	6.9%<br>(3.7 to 10.3)	Not reprted
**Mexico**	6.0%<br>(0.1 to 12.7)	9.8%<br>(6.6 to 13.6)	Not reprted	-9.3%<br>(-38.8 to 13.3)	5.3%<br>(0.1 to 15.7)	Not reprted	0.6%<br>(-0.2 to 1.3)	5.7%<br>(-0.4 to 10.6)	Not reprted	0.6%<br>(-0.1 to 1.2)	Not reprted	9.8%<br>(-0.5 to 21.3)	6.8%<br>(3.7 to 10.0)	Not reprted
**Micronesia (Federated States of)**	3.1%<br>(0.0 to 7.6)	5.2%<br>(1.7 to 12.2)	Not reprted	-5.3%<br>(-22.3 to 7.8)	7.8%<br>(0.9 to 19.2)	Not reprted	0.6%<br>(-0.2 to 1.5)	8.9%<br>(-0.7 to 16.6)	Not reprted	8.3%<br>(-0.9 to 16.2)	Not reprted	12.7%<br>(-0.6 to 26.1)	7.0%<br>(3.8 to 10.6)	Not reprted
**Monaco**	8.0%<br>(-0.5 to 23.1)	5.5%<br>(2.9 to 8.6)	Not reprted	-13.8%<br>(-57.1 to 18.0)	4.8%<br>(0.1 to 15.1)	Not reprted	0.5%<br>(-0.1 to 1.3)	2.9%<br>(-0.2 to 5.9)	Not reprted	0.0%<br>(0.0 to 0.0)	Not reprted	7.2%<br>(-0.2 to 16.6)	6.2%<br>(3.3 to 9.2)	Not reprted
**Mongolia**	8.1%<br>(0.2 to 17.1)	20.2%<br>(9.9 to 28.1)	Not reprted	-14.4%<br>(-59.4 to 19.1)	6.8%<br>(0.3 to 18.8)	Not reprted	7.4%<br>(-2.4 to 15.6)	13.4%<br>(-1.0 to 24.6)	Not reprted	1.7%<br>(-0.2 to 3.5)	Not reprted	5.1%<br>(-0.1 to 12.1)	4.3%<br>(2.3 to 6.5)	Not reprted
**Montenegro**	7.7%<br>(0.3 to 16.0)	13.2%<br>(8.5 to 17.3)	Not reprted	-7.4%<br>(-30.2 to 11.2)	15.6%<br>(4.3 to 29.9)	Not reprted	1.0%<br>(-0.3 to 2.4)	2.6%<br>(-0.2 to 5.2)	Not reprted	0.0%<br>(0.0 to 0.0)	Not reprted	6.7%<br>(-0.3 to 15.2)	7.3%<br>(3.9 to 10.6)	Not reprted
**Morocco**	0.2%<br>(0.0 to 0.4)	14.6%<br>(10.2 to 19.6)	Not reprted	-3.3%<br>(-13.2 to 5.5)	1.6%<br>(0.0 to 8.0)	Not reprted	0.1%<br>(0.0 to 0.1)	4.0%<br>(-0.3 to 7.5)	Not reprted	0.0%<br>(0.0 to 0.0)	Not reprted	7.4%<br>(-0.3 to 16.6)	7.5%<br>(4.0 to 11.1)	Not reprted
**Mozambique**	3.4%<br>(0.0 to 7.9)	3.4%<br>(2.1 to 5.2)	Not reprted	-0.7%<br>(-2.8 to 1.2)	6.8%<br>(0.2 to 19.1)	Not reprted	1.2%<br>(-0.3 to 2.7)	10.8%<br>(-0.8 to 20.2)	Not reprted	9.2%<br>(-1.0 to 17.7)	Not reprted	2.4%<br>(0.0 to 5.7)	2.6%<br>(1.3 to 3.9)	Not reprted
**Myanmar**	4.5%<br>(0.1 to 9.6)	13.3%<br>(6.2 to 20.7)	Not reprted	-2.7%<br>(-11.1 to 4.6)	12.0%<br>(1.9 to 25.8)	Not reprted	7.8%<br>(-2.5 to 16.5)	9.1%<br>(-0.7 to 17.0)	Not reprted	0.3%<br>(0.0 to 0.7)	Not reprted	1.3%<br>(-0.2 to 3.5)	4.6%<br>(2.4 to 7.0)	Not reprted
**Namibia**	8.8%<br>(0.3 to 18.7)	13.0%<br>(6.2 to 19.4)	Not reprted	-6.2%<br>(-25.2 to 10.2)	2.7%<br>(0.0 to 11.2)	Not reprted	3.8%<br>(-1.1 to 8.5)	10.4%<br>(-0.8 to 19.5)	Not reprted	4.9%<br>(-0.5 to 9.5)	Not reprted	5.9%<br>(-0.1 to 13.3)	4.6%<br>(2.4 to 6.8)	Not reprted
**Nauru**	5.6%<br>(0.1 to 12.7)	2.6%<br>(0.4 to 5.5)	Not reprted	-7.1%<br>(-29.1 to 10.8)	7.6%<br>(0.7 to 18.9)	Not reprted	0.2%<br>(0.0 to 0.5)	8.1%<br>(-0.6 to 14.8)	Not reprted	5.6%<br>(-0.6 to 10.9)	Not reprted	16.1%<br>(-1.0 to 31.6)	7.3%<br>(4.0 to 10.9)	Not reprted
**Nepal**	3.5%<br>(0.0 to 8.6)	12.5%<br>(7.5 to 18.2)	Not reprted	-2.5%<br>(-10.2 to 4.4)	5.9%<br>(0.1 to 17.8)	Not reprted	2.3%<br>(-0.7 to 5.1)	7.9%<br>(-0.6 to 14.8)	Not reprted	0.9%<br>(-0.1 to 1.9)	Not reprted	1.4%<br>(-0.1 to 3.5)	5.7%<br>(3.1 to 8.8)	Not reprted
**Netherlands**	11.5%<br>(0.3 to 22.9)	7.2%<br>(5.0 to 9.8)	Not reprted	-10.0%<br>(-41.2 to 14.3)	4.0%<br>(0.0 to 14.0)	Not reprted	2.9%<br>(-0.9 to 6.6)	5.2%<br>(-0.4 to 10.2)	Not reprted	0.1%<br>(0.0 to 0.2)	Not reprted	4.4%<br>(0.0 to 10.7)	4.9%<br>(2.6 to 7.5)	Not reprted
**New Zealand**	11.6%<br>(0.3 to 22.5)	3.4%<br>(1.4 to 5.7)	Not reprted	-11.8%<br>(-48.3 to 16.3)	4.2%<br>(0.0 to 14.0)	Not reprted	3.0%<br>(-0.8 to 6.8)	5.5%<br>(-0.4 to 10.5)	Not reprted	0.0%<br>(0.0 to 0.1)	Not reprted	7.0%<br>(-0.2 to 15.8)	7.6%<br>(4.1 to 11.1)	Not reprted
**Nicaragua**	4.5%<br>(0.1 to 9.6)	7.3%<br>(3.2 to 12.7)	Not reprted	-1.0%<br>(-4.2 to 1.8)	8.3%<br>(0.6 to 21.2)	Not reprted	2.3%<br>(-0.7 to 5.0)	10.4%<br>(-0.8 to 19.4)	Not reprted	9.9%<br>(-0.9 to 18.8)	Not reprted	8.9%<br>(-0.3 to 19.4)	4.4%<br>(2.3 to 6.7)	Not reprted
**Niger**	0.4%<br>(0.0 to 1.3)	8.3%<br>(4.7 to 13.3)	Not reprted	-4.8%<br>(-19.7 to 7.8)	3.7%<br>(0.0 to 13.3)	Not reprted	0.6%<br>(-0.2 to 1.5)	11.0%<br>(-0.8 to 20.6)	Not reprted	0.4%<br>(0.0 to 1.0)	Not reprted	2.0%<br>(0.0 to 4.8)	4.5%<br>(2.4 to 6.9)	Not reprted
**Nigeria**	6.0%<br>(0.3 to 12.6)	16.2%<br>(8.1 to 25.2)	Not reprted	-1.3%<br>(-5.2 to 2.2)	3.1%<br>(0.0 to 12.1)	Not reprted	0.2%<br>(-0.1 to 0.5)	7.3%<br>(-0.5 to 13.7)	Not reprted	0.4%<br>(0.0 to 1.0)	Not reprted	3.8%<br>(0.0 to 9.1)	3.5%<br>(1.8 to 5.4)	Not reprted
**Niue**	5.7%<br>(0.1 to 12.9)	2.9%<br>(0.3 to 6.4)	Not reprted	-6.2%<br>(-25.3 to 8.8)	8.5%<br>(1.1 to 20.3)	Not reprted	0.1%<br>(0.0 to 0.3)	7.0%<br>(-0.5 to 13.0)	Not reprted	4.6%<br>(-0.5 to 9.0)	Not reprted	11.6%<br>(-0.6 to 23.8)	10.1%<br>(5.6 to 14.8)	Not reprted
**North Macedonia**	8.4%<br>(0.3 to 17.4)	18.5%<br>(12.6 to 23.1)	Not reprted	-3.7%<br>(-15.7 to 5.9)	15.7%<br>(4.1 to 30.3)	Not reprted	1.5%<br>(-0.4 to 3.6)	4.7%<br>(-0.3 to 9.2)	Not reprted	0.0%<br>(0.0 to 0.0)	Not reprted	6.9%<br>(-0.2 to 15.7)	6.3%<br>(3.3 to 9.3)	Not reprted
**Northern Mariana Islands**	4.1%<br>(0.0 to 11.0)	5.8%<br>(2.8 to 9.2)	Not reprted	-7.5%<br>(-31.3 to 11.3)	9.1%<br>(1.0 to 21.7)	Not reprted	0.0%<br>(0.0 to 0.1)	6.1%<br>(-0.4 to 11.8)	Not reprted	2.4%<br>(-0.3 to 5.0)	Not reprted	12.5%<br>(-0.7 to 25.6)	8.0%<br>(4.3 to 11.8)	Not reprted

**Norway**	9.2% (0.3 to 18.9)	2.3% (0.9 to 4.3)	Not reprted	-8.4% (-35.5 to 12.3)	3.9% (0.0 to 13.2)	Not reprted	2.5% (-0.6 to 5.7)	4.6% (-0.3 to 9.3)	Not reprted	0.3% (0.0 to 0.8)	Not reprted	3.7% (0.0 to 9.0)	6.7% (3.6 to 10.0)	Not reprted
**Oman**	0.4% (0.0 to 1.0)	28.3% (20.5 to 36.7)	Not reprted	-7.0% (-28.0 to 11.3)	1.8% (0.0 to 8.7)	Not reprted	1.1% (-0.3 to 2.4)	3.5% (-0.2 to 6.6)	Not reprted	0.0% (0.0 to 0.0)	Not reprted	10.4% (-0.5 to 22.2)	6.9% (3.7 to 10.3)	Not reprted
**Pakistan**	0.8% (0.0 to 2.1)	17.0% (8.5 to 25.6)	Not reprted	-2.8% (-11.5 to 4.8)	6.0% (0.1 to 17.7)	Not reprted	5.6% (-1.7 to 11.7)	8.9% (-0.6 to 16.5)	Not reprted	5.3% (-0.5 to 10.5)	Not reprted	3.3% (0.0 to 7.9)	6.8% (3.7 to 10.0)	Not reprted
**Palau**	5.5% (0.0 to 14.9)	3.6% (0.3 to 7.5)	Not reprted	-7.7% (-32.5 to 11.3)	7.9% (0.8 to 19.4)	Not reprted	0.1% (0.0 to 0.3)	7.6% (-0.6 to 13.9)	Not reprted	4.7% (-0.5 to 9.3)	Not reprted	13.0% (-0.6 to 26.2)	9.2% (4.9 to 13.5)	Not reprted
**Palestine**	0.8% (0.0 to 1.9)	18.9% (12.9 to 25.9)	Not reprted	-1.0% (-4.3 to 1.8)	1.5% (0.0 to 7.3)	Not reprted	5.2% (-1.7 to 10.8)	6.1% (-0.4 to 11.4)	Not reprted	0.1% (0.0 to 0.3)	Not reprted	9.8% (-0.5 to 20.8)	5.3% (2.8 to 7.9)	Not reprted
**Panama**	6.7% (0.2 to 14.3)	7.7% (4.2 to 12.2)	Not reprted	-8.3% (-32.8 to 12.1)	9.0% (0.7 to 22.4)	Not reprted	6.7% (-2.1 to 14.2)	7.0% (-0.5 to 13.2)	Not reprted	3.8% (-0.4 to 7.5)	Not reprted	8.6% (-0.3 to 18.9)	5.5% (2.9 to 8.1)	Not reprted
**Papua New Guinea**	1.3% (0.0 to 3.2)	5.1% (1.5 to 12.0)	Not reprted	-3.5% (-14.0 to 5.8)	8.8% (0.9 to 21.4)	Not reprted	0.1% (0.0 to 0.3)	7.8% (-0.6 to 14.8)	Not reprted	3.1% (-0.3 to 6.1)	Not reprted	3.2% (0.0 to 8.1)	5.1% (2.7 to 7.5)	Not reprted
**Paraguay**	9.5% (0.2 to 19.8)	6.8% (2.5 to 13.0)	Not reprted	-13.1% (-53.2 to 18.0)	6.9% (0.2 to 19.5)	Not reprted	0.8% (-0.2 to 1.8)	5.8% (-0.4 to 11.2)	Not reprted	2.6% (-0.3 to 5.2)	Not reprted	7.8% (-0.2 to 17.8)	5.9% (3.1 to 8.8)	Not reprted
**Peru**	7.4% (0.1 to 16.2)	19.0% (11.6 to 26.3)	Not reprted	-2.6% (-10.5 to 4.5)	6.0% (0.2 to 16.9)	Not reprted	1.7% (-0.5 to 3.8)	6.9% (-0.5 to 12.7)	Not reprted	1.2% (-0.1 to 2.5)	Not reprted	7.5% (-0.2 to 16.9)	3.6% (1.9 to 5.4)	Not reprted
**Philippines**	9.3% (0.3 to 19.2)	12.3% (6.8 to 16.7)	Not reprted	-5.9% (-23.7 to 9.1)	11.7% (2.3 to 25.0)	Not reprted	8.7% (-2.8 to 18.3)	6.6% (-0.5 to 12.3)	Not reprted	1.3% (-0.1 to 2.6)	Not reprted	3.2% (0.0 to 7.4)	3.3% (1.7 to 5.0)	Not reprted
**Poland**	12.3% (0.5 to 23.7)	13.5% (10.3 to 17.0)	Not reprted	-12.6% (-53.1 to 17.2)	11.6% (2.1 to 24.6)	Not reprted	1.4% (-0.4 to 3.2)	7.6% (-0.5 to 14.2)	Not reprted	0.0% (0.0 to 0.0)	Not reprted	6.8% (-0.2 to 16.1)	6.8% (3.7 to 9.8)	Not reprted
**Portugal**	12.1% (0.4 to 23.5)	3.9% (2.1 to 6.0)	Not reprted	-12.4% (-50.9 to 17.2)	5.5% (0.2 to 16.0)	Not reprted	2.3% (-0.6 to 5.1)	5.3% (-0.4 to 10.3)	Not reprted	0.0% (0.0 to 0.0)	Not reprted	5.6% (-0.1 to 13.4)	6.4% (3.6 to 9.5)	Not reprted
**Puerto Rico**	6.3% (0.1 to 13.8)	2.6% (0.7 to 4.8)	Not reprted	-4.8% (-20.0 to 7.3)	4.2% (0.0 to 14.1)	Not reprted	2.9% (-0.8 to 6.6)	5.7% (-0.4 to 10.8)	Not reprted	5.3% (-0.5 to 10.5)	Not reprted	10.8% (-0.5 to 22.7)	8.5% (4.6 to 12.7)	Not reprted
**Qatar**	0.5% (0.0 to 1.3)	39.8% (31.5 to 47.5)	Not reprted	-4.9% (-20.3 to 8.1)	1.7% (0.0 to 8.2)	Not reprted	0.1% (0.0 to 0.2)	1.8% (-0.1 to 3.6)	Not reprted	0.0% (0.0 to 0.0)	Not reprted	10.6% (-0.5 to 22.1)	11.3% (6.4 to 16.2)	Not reprted
**Republic of Korea**	9.5% (0.2 to 19.3)	18.7% (12.7 to 25.7)	Not reprted	-9.8% (-39.5 to 14.0)	13.1% (2.9 to 27.0)	Not reprted	6.4% (-2.0 to 13.2)	6.0% (-0.4 to 11.4)	Not reprted	0.0% (0.0 to 0.0)	Not reprted	1.6% (-0.1 to 4.5)	6.7% (3.6 to 9.8)	Not reprted
**Republic of Moldova**	11.5% (0.3 to 24.3)	9.0% (4.3 to 15.1)	Not reprted	-4.0% (-15.9 to 7.1)	3.5% (0.0 to 12.9)	Not reprted	1.7% (-0.5 to 3.8)	7.7% (-0.6 to 14.5)	Not reprted	0.0% (0.0 to 0.0)	Not reprted	9.4% (-0.3 to 20.4)	5.1% (2.7 to 7.6)	Not reprted
**Romania**	12.0% (0.4 to 24.1)	10.5% (7.7 to 13.6)	Not reprted	-12.0% (-50.2 to 17.1)	16.4% (4.8 to 31.5)	Not reprted	0.9% (-0.2 to 2.0)	6.3% (-0.4 to 11.9)	Not reprted	0.0% (0.0 to 0.0)	Not reprted	7.8% (-0.3 to 17.0)	7.1% (3.7 to 10.5)	Not reprted
**Russian Federation**	9.8% (0.2 to 20.5)	7.5% (4.5 to 11.4)	Not reprted	-10.1% (-40.4 to 14.9)	8.4% (0.8 to 20.5)	Not reprted	3.2% (-0.9 to 6.9)	8.2% (-0.6 to 15.3)	Not reprted	0.1% (0.0 to 0.1)	Not reprted	8.8% (-0.3 to 19.5)	4.3% (2.3 to 6.4)	Not reprted
**Rwanda**	7.2% (0.2 to 15.1)	3.9% (1.9 to 7.1)	Not reprted	-0.7% (-3.1 to 1.3)	7.0% (0.2 to 19.6)	Not reprted	0.0% (0.0 to 0.0)	0.4% (0.0 to 0.8)	Not reprted	5.6% (-0.6 to 10.9)	Not reprted	0.9% (-0.2 to 3.1)	2.3% (1.2 to 3.5)	Not reprted
**Saint Kitts and Nevis**	3.9% (-0.1 to 12.1)	3.9% (1.4 to 6.7)	Not reprted	-4.1% (-16.6 to 6.7)	4.4% (0.0 to 14.8)	Not reprted	8.4% (-2.7 to 17.7)	8.6% (-0.6 to 16.2)	Not reprted	1.4% (-0.2 to 3.1)	Not reprted	7.8% (-0.3 to 17.3)	5.9% (3.1 to 8.8)	Not reprted
**Saint Lucia**	9.2% (0.2 to 19.6)	16.4% (6.6 to 29.5)	Not reprted	-6.8% (-28.3 to 10.4)	4.3% (0.0 to 14.7)	Not reprted	5.5% (-1.7 to 11.9)	6.4% (-0.5 to 11.9)	Not reprted	4.9% (-0.5 to 9.5)	Not reprted	6.8% (-0.2 to 15.4)	5.8% (3.1 to 8.5)	Not reprted

**Saint Vincent and the Grenadines**	9.8%<br>(0.1 to 20.3)	16.5%<br>(6.2 to 31.1)	Not reprted	-3.1%<br>(-12.5 to 5.1)	4.1%<br>(0.0 to 14.3)	Not reprted	3.8%<br>(-1.2 to 8.3)	5.5%<br>(-0.4 to 10.4)	Not reprted	3.1%<br>(-0.3 to 6.2)	Not reprted	5.3%<br>(0.0 to 12.6)	5.5%<br>(2.9 to 8.2)	Not reprted
**Samoa**	2.5%<br>(0.1 to 5.8)	6.4%<br>(1.9 to 14.6)	Not reprted	-6.6%<br>(-26.6 to 9.6)	2.6%<br>(0.0 to 9.9)	Not reprted	0.0%<br>(0.0 to 0.1)	7.9%<br>(-0.6 to 14.7)	Not reprted	10.5%<br>(-1.1 to 20.1)	Not reprted	13.4%<br>(-0.7 to 27.1)	8.8%<br>(4.8 to 13.1)	Not reprted
**San Marino**	10.1%<br>(-0.6 to 24.2)	6.3%<br>(3.4 to 9.7)	Not reprted	-11.1%<br>(-46.9 to 16.0)	4.4%<br>(0.1 to 14.5)	Not reprted	2.5%<br>(-0.7 to 5.5)	5.3%<br>(-0.4 to 10.2)	Not reprted	0.0%<br>(0.0 to 0.0)	Not reprted	6.4%<br>(-0.2 to 14.8)	5.9%<br>(3.3 to 8.9)	Not reprted
**Sao Tome and Principe**	6.4%<br>(0.2 to 13.6)	8.0%<br>(4.3 to 13.5)	Not reprted	-0.2%<br>(-1.0 to 0.4)	3.8%<br>(0.0 to 14.0)	Not reprted	0.1%<br>(0.0 to 0.1)	4.8%<br>(-0.3 to 9.2)	Not reprted	6.4%<br>(-0.6 to 12.2)	Not reprted	6.3%<br>(-0.1 to 14.1)	4.3%<br>(2.2 to 6.5)	Not reprted
**Saudi Arabia**	0.2%<br>(0.0 to 0.5)	34.5%<br>(27.1 to 42.1)	Not reprted	-2.4%<br>(-9.9 to 4.1)	1.8%<br>(0.0 to 8.2)	Not reprted	1.1%<br>(-0.3 to 2.3)	6.9%<br>(-0.5 to 12.8)	Not reprted	0.0%<br>(0.0 to 0.0)	Not reprted	13.6%<br>(-0.7 to 28.2)	7.0%<br>(3.7 to 10.6)	Not reprted
**Senegal**	0.7%<br>(0.0 to 1.9)	8.1%<br>(4.0 to 14.1)	Not reprted	-1.9%<br>(-7.7 to 3.3)	3.8%<br>(0.0 to 13.5)	Not reprted	0.3%<br>(-0.1 to 0.8)	10.2%<br>(-0.8 to 18.9)	Not reprted	1.9%<br>(-0.2 to 3.9)	Not reprted	3.9%<br>(0.0 to 8.8)	5.4%<br>(2.8 to 8.1)	Not reprted
**Serbia**	9.0%<br>(0.3 to 18.3)	16.4%<br>(11.3 to 20.6)	Not reprted	-5.3%<br>(-21.2 to 8.3)	16.2%<br>(4.6 to 30.9)	Not reprted	2.5%<br>(-0.7 to 5.5)	4.6%<br>(-0.3 to 9.0)	Not reprted	0.0%<br>(0.0 to 0.0)	Not reprted	7.7%<br>(-0.2 to 17.3)	5.8%<br>(3.1 to 8.8)	Not reprted
**Seychelles**	5.6%<br>(0.1 to 11.8)	5.4%<br>(1.7 to 9.9)	Not reprted	-1.6%<br>(-6.8 to 2.5)	9.4%<br>(1.2 to 21.7)	Not reprted	3.7%<br>(-1.1 to 8.0)	7.6%<br>(-0.6 to 14.0)	Not reprted	4.5%<br>(-0.5 to 8.7)	Not reprted	8.4%<br>(-0.3 to 18.6)	7.3%<br>(4.0 to 10.9)	Not reprted
**Sierra Leone**	4.0%<br>(0.2 to 8.5)	6.0%<br>(3.6 to 9.1)	Not reprted	-0.4%<br>(-1.7 to 0.7)	3.7%<br>(0.0 to 13.4)	Not reprted	6.8%<br>(-2.1 to 14.4)	8.9%<br>(-0.6 to 16.8)	Not reprted	4.2%<br>(-0.4 to 8.5)	Not reprted	3.1%<br>(0.0 to 7.2)	3.6%<br>(1.9 to 5.5)	Not reprted
**Singapore**	2.8%<br>(-0.1 to 6.2)	11.9%<br>(6.4 to 18.6)	Not reprted	-10.9%<br>(-44.0 to 15.7)	11.1%<br>(1.7 to 24.6)	Not reprted	4.1%<br>(-1.2 to 9.2)	5.3%<br>(-0.4 to 9.9)	Not reprted	0.0%<br>(0.0 to 0.0)	Not reprted	4.3%<br>(0.0 to 9.8)	7.1%<br>(3.8 to 10.5)	Not reprted
**Slovakia**	12.5%<br>(0.5 to 25.3)	11.2%<br>(8.4 to 14.3)	Not reprted	-10.1%<br>(-41.5 to 14.5)	16.3%<br>(5.0 to 31.0)	Not reprted	5.0%<br>(-1.5 to 10.7)	7.3%<br>(-0.5 to 13.8)	Not reprted	0.0%<br>(0.0 to 0.0)	Not reprted	8.5%<br>(-0.3 to 19.3)	6.7%<br>(3.5 to 10.1)	Not reprted
**Slovenia**	8.0%<br>(0.1 to 18.3)	9.5%<br>(6.9 to 12.6)	Not reprted	-9.0%<br>(-37.7 to 13.5)	16.8%<br>(5.0 to 31.1)	Not reprted	2.1%<br>(-0.6 to 4.7)	4.6%<br>(-0.3 to 8.9)	Not reprted	0.0%<br>(0.0 to 0.1)	Not reprted	6.8%<br>(-0.2 to 15.2)	7.4%<br>(3.9 to 11.1)	Not reprted
**Solomon Islands**	1.3%<br>(0.0 to 3.2)	3.1%<br>(1.1 to 6.9)	Not reprted	-1.3%<br>(-5.5 to 2.4)	8.4%<br>(0.9 to 20.3)	Not reprted	0.1%<br>(0.0 to 0.3)	8.2%<br>(-0.6 to 15.4)	Not reprted	11.6%<br>(-1.2 to 21.8)	Not reprted	6.1%<br>(-0.1 to 14.0)	4.3%<br>(2.2 to 6.4)	Not reprted
**Somalia**	0.0%<br>(0.0 to 0.0)	3.2%<br>(2.1 to 4.7)	Not reprted	-4.0%<br>(-16.8 to 6.8)	6.9%<br>(0.2 to 19.2)	Not reprted	7.7%<br>(-2.5 to 16.4)	11.6%<br>(-0.9 to 21.9)	Not reprted	11.3%<br>(-1.1 to 21.4)	Not reprted	1.4%<br>(-0.1 to 3.6)	2.9%<br>(1.5 to 4.3)	Not reprted
**South Africa**	6.7%<br>(0.2 to 14.2)	15.3%<br>(10.7 to 20.2)	Not reprted	-8.2%<br>(-33.6 to 12.0)	2.4%<br>(0.0 to 10.5)	Not reprted	1.7%<br>(-0.5 to 3.6)	10.4%<br>(-0.7 to 19.3)	Not reprted	2.2%<br>(-0.2 to 4.4)	Not reprted	9.4%<br>(-0.4 to 20.1)	4.2%<br>(2.2 to 6.2)	Not reprted
**South Sudan**	0.2%<br>(-0.1 to 0.8)	6.6%<br>(3.6 to 10.7)	Not reprted	-2.1%<br>(-8.7 to 3.6)	6.7%<br>(0.2 to 18.8)	Not reprted	0.2%<br>(-0.1 to 0.6)	7.7%<br>(-0.6 to 14.6)	Not reprted	5.6%<br>(-0.6 to 10.9)	Not reprted	0.1%<br>(-0.7 to 1.4)	3.1%<br>(1.7 to 4.7)	Not reprted
**Spain**	10.5%<br>(0.3 to 21.4)	5.3%<br>(3.4 to 7.6)	Not reprted	-10.4%<br>(-43.2 to 14.6)	3.2%<br>(0.1 to 11.0)	Not reprted	3.3%<br>(-0.9 to 7.0)	3.8%<br>(-0.2 to 7.2)	Not reprted	0.0%<br>(0.0 to 0.0)	Not reprted	6.6%<br>(-0.2 to 15.4)	5.4%<br>(2.9 to 8.1)	Not reprted
**Sri Lanka**	3.8%<br>(0.1 to 8.3)	13.3%<br>(6.1 to 19.3)	Not reprted	-0.1%<br>(-0.5 to 0.2)	11.9%<br>(1.8 to 25.4)	Not reprted	2.0%<br>(-0.6 to 4.4)	8.2%<br>(-0.6 to 15.4)	Not reprted	1.5%<br>(-0.2 to 3.1)	Not reprted	2.6%<br>(0.0 to 6.2)	4.7%<br>(2.4 to 7.0)	Not reprted
**Sudan**	0.0%<br>(0.0 to 0.0)	18.3%<br>(9.7 to 28.8)	Not reprted	-2.3%<br>(-9.7 to 3.6)	1.6%<br>(0.0 to 7.8)	Not reprted	3.7%<br>(-1.0 to 7.8)	6.9%<br>(-0.5 to 12.7)	Not reprted	0.2%<br>(0.0 to 0.5)	Not reprted	8.7%<br>(-0.4 to 19.3)	4.9%<br>(2.7 to 7.4)	Not reprted
**Suriname**	5.9%<br>(0.1 to 12.5)	20.6%<br>(8.7 to 34.3)	Not reprted	-1.3%<br>(-5.5 to 2.3)	3.9%<br>(0.0 to 13.3)	Not reprted	8.5%<br>(-2.8 to 17.7)	7.2%<br>(-0.5 to 13.4)	Not reprted	2.1%<br>(-0.2 to 4.2)	Not reprted	5.4%<br>(0.0 to 12.8)	6.3%<br>(3.3 to 9.6)	Not reprted
**Sweden**	10.7%<br>(0.2 to 22.1)	2.1%<br>(0.6 to 4.0)	Not reprted	-9.5%<br>(-39.5 to 13.6)	4.7%<br>(0.1 to 15.3)	Not reprted	2.6%<br>(-0.7 to 5.8)	5.4%<br>(-0.4 to 10.4)	Not reprted	0.1%<br>(0.0 to 0.3)	Not reprted	4.6%<br>(-0.1 to 11.2)	5.5%<br>(2.8 to 8.1)	Not reprted

**Switzerland**	11.6% (0.2 to 23.3)	5.3% (3.5 to 7.6)	Not reprted	-10.4% (-43.0 to 14.6)	4.3% (0.1 to 14.1)	Not reprted	4.5% (-1.4 to 9.9)	5.3% (-0.4 to 10.3)	Not reprted	0.0% (0.0 to 0.1)	Not reprted	3.7% (0.0 to 9.1)	5.0% (2.6 to 7.4)	Not reprted
**Syrian Arab Republic**	0.6% (0.0 to 1.5)	17.9% (12.8 to 23.4)	Not reprted	-2.8% (-11.2 to 4.8)	1.6% (0.0 to 7.8)	Not reprted	2.7% (-0.8 to 5.8)	5.6% (-0.4 to 10.6)	Not reprted	0.0% (0.0 to 0.1)	Not reprted	11.7% (-0.6 to 24.6)	5.8% (3.1 to 8.7)	Not reprted
**Turkey**	1.8% (0.1 to 4.2)	16.9% (13.0 to 21.0)	Not reprted	-2.6% (-10.9 to 4.4)	1.2% (0.0 to 6.6)	Not reprted	0.2% (-0.1 to 0.5)	2.2% (-0.1 to 4.3)	Not reprted	0.0% (0.0 to 0.0)	Not reprted	9.1% (-0.4 to 19.6)	6.5% (3.4 to 9.8)	Not reprted
**Taiwan (Province of China)**	7.1% (0.1 to 14.6)	12.2% (9.2 to 15.5)	Not reprted	-13.8% (-57.8 to 18.6)	7.0% (0.5 to 19.1)	Not reprted	4.6% (-1.5 to 9.8)	5.4% (-0.4 to 10.0)	Not reprted	0.0% (0.0 to 0.0)	Not reprted	4.4% (0.0 to 10.7)	5.6% (2.9 to 8.4)	Not reprted
**Tajikistan**	1.5% (0.0 to 3.6)	13.5% (7.4 to 20.7)	Not reprted	-2.0% (-8.3 to 3.5)	6.0% (0.3 to 17.6)	Not reprted	5.2% (-1.5 to 11.0)	7.8% (-0.6 to 14.9)	Not reprted	0.0% (0.0 to 0.0)	Not reprted	6.2% (-0.1 to 14.3)	5.3% (2.8 to 8.0)	Not reprted
**Thailand**	7.3% (0.2 to 14.7)	21.2% (15.2 to 27.3)	Not reprted	-3.7% (-15.1 to 6.0)	10.6% (1.8 to 23.9)	Not reprted	8.9% (-2.9 to 18.3)	6.0% (-0.4 to 10.9)	Not reprted	1.9% (-0.2 to 3.8)	Not reprted	3.9% (0.0 to 9.1)	4.1% (2.1 to 6.2)	Not reprted
**Timor-Leste**	3.8% (0.1 to 8.5)	8.8% (3.0 to 17.1)	Not reprted	-1.8% (-7.4 to 3.3)	12.1% (2.1 to 26.2)	Not reprted	3.8% (-1.1 to 8.4)	11.9% (-0.9 to 22.0)	Not reprted	8.0% (-0.8 to 15.4)	Not reprted	0.1% (-0.5 to 1.2)	3.1% (1.7 to 4.8)	Not reprted
**Togo**	3.9% (0.1 to 8.6)	8.2% (5.1 to 12.9)	Not reprted	-0.6% (-2.5 to 1.1)	3.8% (0.0 to 13.5)	Not reprted	0.1% (0.0 to 0.3)	14.4% (-1.1 to 26.5)	Not reprted	9.4% (-0.9 to 18.0)	Not reprted	3.7% (0.0 to 8.8)	3.5% (1.8 to 5.4)	Not reprted
**Tokelau**	3.7% (0.0 to 8.7)	2.4% (0.0 to 6.3)	Not reprted	-5.9% (-25.1 to 9.1)	8.0% (0.8 to 19.5)	Not reprted	0.2% (-0.1 to 0.6)	7.8% (-0.6 to 14.6)	Not reprted	5.9% (-0.7 to 11.7)	Not reprted	12.0% (-0.6 to 24.9)	9.2% (5.0 to 13.8)	Not reprted
**Tonga**	1.1% (0.0 to 2.9)	7.0% (2.4 to 15.3)	Not reprted	-5.1% (-20.7 to 8.0)	8.8% (0.8 to 21.2)	Not reprted	0.3% (-0.1 to 0.8)	7.6% (-0.6 to 14.3)	Not reprted	6.6% (-0.7 to 12.8)	Not reprted	14.1% (-0.8 to 27.2)	7.5% (4.0 to 11.3)	Not reprted
**Trinidad and Tobago**	7.2% (0.1 to 15.3)	17.6% (6.0 to 33.8)	Not reprted	-2.8% (-11.5 to 4.9)	4.4% (0.0 to 14.9)	Not reprted	3.7% (-1.0 to 7.8)	8.1% (-0.6 to 15.1)	Not reprted	3.4% (-0.4 to 6.7)	Not reprted	8.7% (-0.3 to 19.0)	5.8% (3.0 to 8.8)	Not reprted
**Tunisia**	1.3% (0.0 to 3.1)	16.9% (11.8 to 23.2)	Not reprted	-2.3% (-9.8 to 4.1)	1.6% (0.0 to 7.8)	Not reprted	0.5% (-0.1 to 1.1)	5.1% (-0.4 to 9.6)	Not reprted	0.0% (0.0 to 0.0)	Not reprted	7.9% (-0.3 to 17.6)	5.5% (2.8 to 8.1)	Not reprted
**Turkmenistan**	7.0% (0.1 to 15.3)	14.6% (8.3 to 22.3)	Not reprted	-14.6% (-59.4 to 19.8)	6.4% (0.3 to 17.7)	Not reprted	3.9% (-1.2 to 8.5)	6.7% (-0.5 to 12.6)	Not reprted	0.0% (0.0 to 0.0)	Not reprted	6.7% (-0.1 to 15.8)	4.9% (2.6 to 7.3)	Not reprted
**Tuvalu**	2.7% (0.1 to 6.6)	1.8% (0.7 to 3.5)	Not reprted	-5.3% (-20.4 to 8.5)	8.0% (0.9 to 19.7)	Not reprted	0.6% (-0.1 to 1.4)	8.6% (-0.6 to 16.0)	Not reprted	7.9% (-0.9 to 15.3)	Not reprted	12.4% (-0.6 to 25.1)	4.4% (2.3 to 6.6)	Not reprted
**Uganda**	7.8% (0.2 to 16.4)	6.3% (3.7 to 9.9)	Not reprted	-1.7% (-7.2 to 3.1)	6.7% (0.2 to 18.8)	Not reprted	0.3% (-0.1 to 0.6)	3.4% (-0.2 to 6.6)	Not reprted	8.1% (-0.8 to 15.7)	Not reprted	1.6% (-0.1 to 4.1)	3.0% (1.5 to 4.5)	Not reprted
**Ukraine**	9.8% (0.3 to 20.5)	10.9% (6.4 to 17.1)	Not reprted	-9.1% (-37.3 to 13.1)	3.7% (0.0 to 13.3)	Not reprted	2.6% (-0.7 to 5.7)	9.7% (-0.7 to 17.8)	Not reprted	0.0% (0.0 to 0.0)	Not reprted	9.3% (-0.3 to 20.5)	3.9% (2.1 to 6.0)	Not reprted
**United Arab Emirates**	1.7% (0.1 to 4.2)	27.4% (20.4 to 34.9)	Not reprted	-4.0% (-16.5 to 6.6)	1.6% (0.0 to 8.4)	Not reprted	1.0% (-0.3 to 2.3)	4.1% (-0.3 to 8.1)	Not reprted	0.0% (0.0 to 0.0)	Not reprted	9.9% (-0.5 to 21.0)	9.2% (4.9 to 13.9)	Not reprted
**United Kingdom**	11.0% (0.5 to 22.3)	5.6% (3.7 to 7.9)	Not reprted	-7.3% (-29.9 to 11.0)	3.4% (0.0 to 11.9)	Not reprted	3.3% (-1.0 to 7.1)	6.7% (-0.5 to 12.8)	Not reprted	0.2% (0.0 to 0.4)	Not reprted	6.8% (-0.2 to 15.6)	3.9% (2.1 to 5.9)	Not reprted
**United Republic of Tanzania**	6.3% (0.2 to 13.6)	5.0% (2.7 to 8.3)	Not reprted	-1.2% (-4.8 to 2.2)	9.7% (0.8 to 23.3)	Not reprted	0.4% (-0.1 to 1.0)	6.6% (-0.5 to 12.7)	Not reprted	4.3% (-0.4 to 8.6)	Not reprted	3.7% (0.0 to 8.4)	3.1% (1.6 to 4.8)	Not reprted
**United States of America**	9.2% (0.3 to 19.5)	3.2% (1.6 to 5.2)	Not reprted	-11.8% (-48.7 to 16.3)	5.4% (0.2 to 15.8)	Not reprted	2.9% (-0.8 to 6.4)	6.5% (-0.5 to 12.2)	Not reprted	0.4% (0.0 to 0.8)	Not reprted	10.4% (-0.5 to 21.8)	7.7% (4.2 to 11.1)	Not reprted
**United States Virgin Islands**	8.0% (-0.4 to 20.5)	3.8% (1.7 to 6.2)	Not reprted	-5.6% (-22.9 to 8.3)	4.1% (0.0 to 13.8)	Not reprted	2.6% (-0.8 to 5.8)	4.6% (-0.3 to 8.7)	Not reprted	0.3% (0.0 to 0.8)	Not reprted	10.3% (-0.4 to 22.4)	7.5% (4.1 to 11.4)	Not reprted

**Uruguay**	9.1%<br>(0.2 to 18.1)	6.1%<br>(2.4 to 11.0)	Not reprted	-13.6%<br>(-56.6 to 18.6)	7.3%<br>(0.3 to 20.0)	Not reprted	3.2%<br>(-0.9 to 6.8)	6.4%<br>(-0.5 to 12.1)	Not<br>reprted	1.0%<br>(-0.1 to 2.0)	Not reprted	7.8%<br>(-0.3 to 17.5)	4.8%<br>(2.5 to 7.2)	Not reprted
**Uzbekistan**	4.7%<br>(0.1 to 10.5)	22.1%<br>(13.5 to 30.9)	Not reprted	-9.4%<br>(-38.5 to 13.6)	6.2%<br>(0.3 to 17.6)	Not reprted	2.4%<br>(-0.7 to 5.2)	6.5%<br>(-0.5 to 12.5)	Not<br>reprted	0.0%<br>(0.0 to 0.0)	Not reprted	6.9%<br>(-0.2 to 16.2)	4.2%<br>(2.2 to 6.3)	Not reprted
**Vanuatu**	3.0%<br>(0.1 to 7.4)	3.7%<br>(1.2 to 8.2)	Not reprted	-9.2%<br>(-37.7 to 13.7)	8.4%<br>(1.0 to 20.2)	Not reprted	0.2%<br>(-0.1 to 0.5)	7.5%<br>(-0.6 to 13.9)	Not<br>reprted	6.3%<br>(-0.6 to 12.3)	Not reprted	6.3%<br>(-0.1 to 14.7)	6.5%<br>(3.4 to 9.6)	Not reprted
**Venezuela (Bolivarian Republic of)**	5.2%<br>(0.1 to 10.9)	10.2%<br>(5.8 to 15.4)	Not reprted	-7.0%<br>(-29.1 to 11.0)	8.6%<br>(0.7 to 21.7)	Not reprted	5.1%<br>(-1.5 to 11.1)	6.7%<br>(-0.5 to 12.5)	Not<br>reprted	1.0%<br>(-0.1 to 2.0)	Not reprted	9.5%<br>(-0.4 to 20.9)	5.0%<br>(2.7 to 7.7)	Not reprted
**Viet Nam**	10.8%<br>(0.3 to 21.3)	11.9%<br>(5.8 to 17.5)	Not reprted	-7.9%<br>(-32.1 to 11.6)	13.0%<br>(2.4 to 27.1)	Not reprted	8.5%<br>(-2.7 to 17.9)	7.1%<br>(-0.5 to 13.4)	Not<br>reprted	0.0%<br>(0.0 to 0.0)	Not reprted	0.4%<br>(-0.3 to 1.7)	3.3%<br>(1.7 to 5.0)	Not reprted
**Yemen**	0.3%<br>(0.0 to 0.8)	16.1%<br>(8.8 to 24.5)	Not reprted	-1.3%<br>(-5.2 to 2.5)	1.5%<br>(0.0 to 7.8)	Not reprted	5.0%<br>(-1.5 to 10.8)	8.6%<br>(-0.6 to 16.1)	Not<br>reprted	4.5%<br>(-0.5 to 9.0)	Not reprted	5.0%<br>(0.0 to 11.0)	4.6%<br>(2.4 to 6.9)	Not reprted
**Zambia**	6.4%<br>(0.2 to 13.7)	7.6%<br>(3.7 to 13.4)	Not reprted	-1.7%<br>(-7.1 to 3.1)	6.8%<br>(0.2 to 18.8)	Not reprted	0.6%<br>(-0.2 to 1.4)	14.0%<br>(-1.1 to 26.0)	Not<br>reprted	8.1%<br>(-0.8 to 15.8)	Not reprted	3.6%<br>(0.0 to 8.2)	4.4%<br>(2.3 to 6.7)	Not reprted
**Zimbabwe**	4.7%<br>(0.1 to 10.6)	4.3%<br>(2.4 to 7.0)	Not reprted	-1.8%<br>(-7.4 to 3.3)	4.1%<br>(0.0 to 14.5)	Not reprted	0.9%<br>(-0.3 to 2.2)	13.5%<br>(-1.1 to 25.0)	Not<br>reprted	11.2%<br>(-1.1 to 21.6)	Not reprted	5.6%<br>(-0.1 to 12.4)	3.7%<br>(1.9 to 5.5)	Not reprted

Appendix Table 13 continues. Age-standardized percent of DALYs (with 95% uncertainty intervals) due to intracerebral haemorrhage associated with individual risk factors by 7 GBD super-region, 21 GBD region and country for both sexes combined in 2021.

Country/region	High systolic blood pressure	High temperature	Household air pollution from solid fuels	Kidney dysfunction	Lead exposure	Low physical activity	Low temperature	Secondhand smoke	Smoking
**GBD super-regions in alphabetical order**									
**Central Europe, Eastern Europe, and Central Asia**	62.7% (47.6 to 73.7)	0.1% (-0.5 to 0.8)	1.6% (0.5 to 5.3)	10.8% (8.4 to 13.3)	3.5% (-0.4 to 8.0)	Not reported	9.1% (7.6 to 10.3)	4.3% (3.0 to 5.7)	19.6% (17.2 to 21.9)
**High-income**	51.7% (37.7 to 62.6)	0.2% (-0.3 to 1.0)	0.0% (0.0 to 0.2)	8.7% (6.5 to 11.0)	3.4% (-0.4 to 7.9)	Not reported	7.2% (6.5 to 8.1)	2.9% (2.0 to 3.8)	15.7% (13.6 to 17.7)
**Latin America and Caribbean**	54.7% (40.3 to 65.8)	0.4% (0.2 to 0.7)	5.6% (3.4 to 10.2)	10.2% (8.0 to 12.4)	7.5% (-1.0 to 16.8)	Not reported	2.5% (2.1 to 2.8)	3.1% (2.1 to 4.1)	10.5% (8.9 to 12.2)
**North Africa and Middle East**	54.5% (40.6 to 65.4)	3.5% (0.4 to 7.3)	4.8% (3.3 to 6.9)	10.3% (7.8 to 13.1)	8.4% (-1.2 to 18.7)	Not reported	5.3% (4.3 to 6.4)	5.0% (3.5 to 6.6)	12.3% (10.5 to 14.1)
**South Asia**	55.6% (41.0 to 67.2)	2.9% (0.7 to 5.7)	25.4% (16.9 to 35.2)	11.3% (8.7 to 14.3)	10.6% (-1.4 to 23.2)	Not reported	2.5% (1.0 to 4.3)	4.5% (3.1 to 6.0)	11.0% (9.1 to 13.1)
**Southeast Asia, East Asia, and Oceania**	57.0% (42.5 to 68.8)	0.7% (0.0 to 1.8)	8.9% (3.2 to 20.4)	9.5% (7.0 to 11.8)	7.6% (-1.0 to 17.1)	Not reported	5.4% (4.8 to 6.1)	5.4% (3.7 to 7.2)	18.5% (15.8 to 21.3)
**Sub-Saharan Africa**	58.7% (44.2 to 69.6)	1.5% (0.9 to 2.3)	35.0% (27.6 to 42.3)	10.8% (8.3 to 13.6)	7.7% (-1.0 to 17.4)	Not reported	2.0% (1.7 to 2.4)	2.5% (1.7 to 3.3)	5.9% (5.0 to 6.9)
**GBD regions in alphabetical order**									
**Andean Latin America**	44.7% (31.1 to 56.4)	0.1% (0.0 to 0.3)	3.8% (0.7 to 11.8)	7.4% (5.7 to 9.3)	6.2% (-0.8 to 14.3)	Not reported	6.0% (5.3 to 7.0)	2.0% (1.3 to 2.7)	7.4% (6.2 to 8.8)
**Australasia**	53.0% (38.4 to 65.4)	0.1% (-0.1 to 0.5)	0.0% (0.0 to 0.0)	7.9% (5.3 to 10.5)	5.4% (-0.7 to 12.6)	Not reported	6.1% (5.4 to 7.2)	2.3% (1.5 to 3.0)	10.2% (8.6 to 12.0)
**Caribbean**	55.0% (40.7 to 66.4)	0.4% (0.3 to 0.5)	17.6% (12.8 to 22.7)	8.8% (6.9 to 10.9)	9.6% (-1.3 to 21.1)	Not reported	0.3% (0.2 to 0.5)	2.5% (1.7 to 3.3)	9.9% (8.3 to 11.7)
**Central Asia**	62.8% (47.6 to 73.8)	0.5% (-0.4 to 1.8)	4.5% (1.8 to 10.4)	13.2% (10.3 to 16.2)	4.1% (-0.5 to 9.5)	Not reported	8.7% (7.3 to 9.9)	4.7% (3.2 to 6.2)	13.8% (12.0 to 15.7)
**Central Europe**	63.3% (48.9 to 74.2)	0.2% (-0.4 to 1.0)	1.2% (0.0 to 7.8)	8.1% (6.0 to 10.2)	4.1% (-0.5 to 9.5)	Not reported	9.2% (8.3 to 11.0)	4.4% (3.1 to 5.8)	18.5% (16.0 to 20.8)
**Central Latin America**	55.1% (40.9 to 66.3)	0.5% (0.2 to 0.9)	4.9% (2.0 to 10.1)	11.9% (9.3 to 14.6)	8.4% (-1.1 to 18.8)	Not reported	3.1% (2.6 to 3.6)	3.0% (2.0 to 3.9)	8.0% (6.8 to 9.2)
**Central Sub-Saharan Africa**	57.7% (42.8 to 69.6)	0.3% (0.0 to 0.8)	36.4% (29.4 to 43.7)	13.2% (10.3 to 16.2)	7.3% (-1.0 to 16.6)	Not reported	1.1% (0.9 to 1.3)	2.1% (1.4 to 2.8)	5.3% (4.3 to 6.5)
**East Asia**	55.6% (41.8 to 67.7)	0.6% (-0.3 to 2.1)	6.9% (2.0 to 19.3)	7.9% (5.7 to 10.1)	8.4% (-1.1 to 18.6)	Not reported	7.6% (6.9 to 8.4)	5.4% (3.7 to 7.2)	19.7% (16.5 to 23.1)
**Eastern Europe**	62.6% (47.3 to 73.4)	-0.1% (-0.6 to 0.4)	0.4% (0.1 to 1.6)	11.0% (8.4 to 13.7)	2.9% (-0.4 to 6.8)	Not reported	9.2% (7.4 to 10.8)	4.0% (2.7 to 5.4)	22.5% (19.8 to 25.1)

**Eastern Sub-Saharan Africa**	56.3% (41.8 to 67.4)	0.6% (0.2 to 1.2)	42.9% (35.6 to 49.7)	8.6% (6.2 to 11.0)	8.3% (-1.1 to 18.6)	Not reported	2.8% (2.4 to 3.4)	2.3% (1.5 to 3.1)	6.6% (5.5 to 7.7)
**High-income Asia Pacific**	51.6% (37.8 to 62.2)	0.2% (-0.2 to 0.8)	0.0% (0.0 to 0.0)	9.6% (6.9 to 12.3)	3.0% (-0.4 to 7.0)	Not reported	7.0% (6.3 to 7.7)	3.4% (2.3 to 4.6)	17.1% (14.7 to 19.2)
**High-income North America**	47.6% (33.3 to 59.6)	0.4% (-0.5 to 1.7)	0.0% (0.0 to 0.0)	9.7% (7.3 to 12.1)	2.9% (-0.4 to 6.8)	Not reported	6.6% (5.7 to 7.3)	2.2% (1.5 to 3.0)	15.8% (13.5 to 18.2)
**North Africa and Middle East**	54.5% (40.6 to 65.4)	3.5% (0.4 to 7.3)	4.8% (3.3 to 6.9)	10.3% (7.8 to 13.1)	8.4% (-1.2 to 18.7)	Not reported	5.3% (4.3 to 6.4)	5.0% (3.5 to 6.6)	12.3% (10.5 to 14.1)
**Oceania**	45.2% (31.7 to 57.3)	0.2% (0.1 to 0.3)	36.5% (27.3 to 44.8)	10.4% (8.0 to 12.9)	3.1% (-0.4 to 7.2)	Not reported	2.7% (2.3 to 3.4)	6.2% (4.2 to 8.2)	12.9% (10.6 to 15.5)
**South Asia**	55.6% (41.0 to 67.2)	2.9% (0.7 to 5.7)	25.4% (16.9 to 35.2)	11.3% (8.7 to 14.3)	10.6% (-1.4 to 23.2)	Not reported	2.5% (1.0 to 4.3)	4.5% (3.1 to 6.0)	11.0% (9.1 to 13.1)
**Southeast Asia**	61.4% (46.5 to 72.1)	1.1% (0.8 to 1.6)	13.1% (5.6 to 23.6)	13.0% (10.0 to 16.1)	6.1% (-0.8 to 13.8)	Not reported	0.6% (0.4 to 0.9)	5.3% (3.6 to 7.0)	16.0% (13.7 to 18.4)
**Southern Latin America**	56.0% (41.6 to 67.7)	0.2% (-0.2 to 0.6)	0.3% (0.0 to 2.6)	7.2% (5.3 to 9.2)	3.8% (-0.5 to 8.5)	Not reported	6.9% (6.4 to 7.6)	4.7% (3.2 to 6.3)	15.9% (13.7 to 18.2)
**Southern Sub-Saharan Africa**	63.2% (47.3 to 74.1)	0.2% (-0.2 to 0.8)	11.9% (8.0 to 18.6)	13.3% (10.6 to 16.3)	6.0% (-0.8 to 13.3)	Not reported	5.9% (5.3 to 6.5)	4.5% (3.1 to 5.9)	10.2% (8.7 to 11.8)
**Tropical Latin America**	56.3% (41.6 to 68.0)	0.4% (0.2 to 0.8)	2.1% (0.4 to 5.9)	9.9% (7.9 to 12.0)	6.3% (-0.8 to 14.4)	Not reported	2.1% (1.7 to 2.5)	3.7% (2.5 to 4.9)	13.3% (11.3 to 15.8)
**Western Europe**	55.4% (40.9 to 66.5)	0.1% (-0.1 to 0.4)	0.0% (0.0 to 0.1)	7.7% (5.6 to 9.8)	4.0% (-0.5 to 9.2)	Not reported	8.1% (7.3 to 9.6)	2.8% (1.9 to 3.7)	14.6% (12.5 to 16.7)
**Western Sub-Saharan Africa**	60.7% (46.0 to 71.4)	3.2% (2.3 to 4.4)	32.4% (23.6 to 41.1)	11.6% (9.1 to 14.4)	7.7% (-1.0 to 17.1)	Not reported	0.4% (0.1 to 0.8)	2.3% (1.6 to 3.1)	4.4% (3.6 to 5.3)
**Countries in alphabetical order**									
**Afghanistan**	50.9% (35.9 to 63.6)	1.0% (-0.4 to 3.0)	37.9% (30.9 to 44.9)	10.6% (8.1 to 13.3)	13.5% (-1.9 to 29.2)	Not reprted	7.7% (6.6 to 8.7)	4.4% (2.9 to 6.0)	7.4% (5.6 to 9.5)
**Albania**	63.3% (48.6 to 75.0)	0.1% (-0.3 to 0.6)	4.3% (0.3 to 16.9)	7.8% (5.1 to 10.5)	5.3% (-0.7 to 11.9)	Not reprted	8.9% (7.8 to 10.1)	4.5% (3.0 to 5.9)	16.1% (13.1 to 19.3)
**Algeria**	51.9% (37.9 to 64.1)	1.5% (0.0 to 3.5)	0.0% (0.0 to 0.2)	9.9% (7.2 to 12.8)	7.2% (-1.0 to 15.9)	Not reprted	6.8% (5.9 to 7.6)	6.1% (4.1 to 8.2)	10.3% (7.9 to 13.5)
**American Samoa**	60.5% (45.5 to 72.7)	0.1% (0.0 to 0.2)	1.0% (0.0 to 6.8)	12.0% (9.4 to 14.6)	1.4% (-0.2 to 3.3)	Not reprted	0.0% (-0.1 to 0.2)	5.9% (4.0 to 7.9)	16.2% (13.3 to 19.2)
**Andorra**	57.7% (42.8 to 69.8)	0.0% (0.0 to 0.0)	0.0% (0.0 to 0.0)	8.0% (5.8 to 10.2)	2.9% (-0.4 to 6.7)	Not reprted	9.9% (8.7 to 10.9)	2.8% (1.9 to 3.8)	14.6% (11.7 to 18.0)
**Angola**	59.8% (45.2 to 72.1)	0.2% (-0.2 to 0.8)	14.5% (4.7 to 27.0)	12.8% (9.9 to 15.9)	6.9% (-0.9 to 15.7)	Not reprted	1.6% (1.3 to 2.0)	2.1% (1.4 to 2.8)	8.6% (6.9 to 10.5)
**Antigua and Barbuda**	56.6% (41.3 to 69.0)	0.4% (0.2 to 0.5)	0.0% (0.0 to 0.4)	9.6% (7.4 to 12.1)	5.3% (-0.7 to 12.0)	Not reprted	0.0% (-0.1 to 0.1)	2.3% (1.5 to 3.0)	8.3% (6.6 to 10.5)
**Argentina**	55.6% (40.7 to 67.8)	0.2% (-0.2 to 0.9)	0.2% (0.0 to 1.5)	7.1% (5.3 to 9.0)	4.0% (-0.5 to 9.1)	Not reprted	6.5% (5.9 to 7.0)	4.7% (3.2 to 6.3)	16.7% (14.2 to 19.3)

**Armenia**	62.9%<br>(47.6 to 74.1)	0.1%<br>(-0.7 to 1.0)	1.0%<br>(0.1 to 3.8)	12.6%<br>(9.8 to 15.4)	4.3%<br>(-0.5 to 9.7)	Not reprted	8.3%<br>(7.0 to 9.4)	5.4%<br>(3.7 to 7.2)	22.5%<br>(19.9 to 25.2)
**Australia**	53.1%<br>(38.4 to 65.7)	0.2%<br>(-0.1 to 0.6)	0.0%<br>(0.0 to 0.0)	7.8%<br>(5.2 to 10.4)	5.5%<br>(-0.7 to 12.6)	Not reprted	5.8%<br>(5.1 to 6.8)	2.2%<br>(1.5 to 3.0)	9.8%<br>(8.2 to 11.7)
**Austria**	58.1%<br>(43.0 to 70.2)	0.0%<br>(-0.3 to 0.3)	0.0%<br>(0.0 to 0.0)	8.2%<br>(5.8 to 10.6)	3.2%<br>(-0.4 to 7.2)	Not reprted	8.3%<br>(7.4 to 9.4)	3.1%<br>(2.1 to 4.2)	16.1%<br>(13.8 to 18.5)
**Azerbaijan**	62.2%<br>(46.6 to 73.6)	0.2%<br>(-0.6 to 1.1)	0.7%<br>(0.0 to 4.9)	13.2%<br>(10.0 to 16.5)	4.0%<br>(-0.5 to 8.9)	Not reprted	8.6%<br>(7.1 to 9.5)	5.7%<br>(3.9 to 7.6)	13.4%<br>(10.6 to 16.5)
**Bahamas**	56.2%<br>(41.7 to 68.6)	0.7%<br>(-0.2 to 2.0)	0.0%<br>(0.0 to 0.3)	9.2%<br>(7.2 to 11.3)	3.3%<br>(-0.4 to 7.8)	Not reprted	0.4%<br>(0.1 to 0.7)	2.5%<br>(1.7 to 3.4)	9.3%<br>(7.4 to 11.5)
**Bahrain**	54.9%<br>(41.4 to 66.6)	6.4%<br>(2.2 to 11.5)	0.0%<br>(0.0 to 0.0)	10.2%<br>(7.3 to 13.2)	5.1%<br>(-0.7 to 11.6)	Not reprted	2.4%<br>(0.7 to 4.3)	4.2%<br>(2.8 to 5.6)	10.9%<br>(9.0 to 13.1)
**Bangladesh**	56.9%<br>(42.6 to 68.6)	2.3%<br>(0.2 to 5.0)	37.7%<br>(28.9 to 45.9)	10.0%<br>(7.6 to 12.6)	11.1%<br>(-1.5 to 23.7)	Not reprted	1.8%<br>(0.6 to 3.2)	5.4%<br>(3.7 to 7.1)	15.0%<br>(12.2 to 17.9)
**Barbados**	59.5%<br>(44.1 to 71.5)	0.4%<br>(0.2 to 0.6)	0.0%<br>(0.0 to 0.1)	9.0%<br>(6.9 to 11.4)	4.4%<br>(-0.6 to 10.0)	Not reprted	0.0%<br>(0.0 to 0.0)	1.5%<br>(1.0 to 2.0)	6.4%<br>(5.2 to 8.0)
**Belarus**	67.2%<br>(52.1 to 78.6)	-0.2%<br>(-0.6 to 0.0)	0.1%<br>(0.0 to 0.5)	11.5%<br>(8.9 to 14.2)	3.6%<br>(-0.5 to 8.2)	Not reprted	9.8%<br>(7.6 to 11.2)	4.5%<br>(3.1 to 6.1)	24.0%<br>(21.3 to 27.0)
**Belgium**	61.3%<br>(45.6 to 72.8)	0.0%<br>(-0.1 to 0.1)	0.0%<br>(0.0 to 0.0)	8.2%<br>(5.9 to 10.4)	6.7%<br>(-0.9 to 15.3)	Not reprted	8.3%<br>(7.3 to 10.6)	2.4%<br>(1.6 to 3.2)	15.2%<br>(12.9 to 17.7)
**Belize**	53.9%<br>(38.3 to 66.8)	1.1%<br>(0.7 to 1.6)	4.3%<br>(0.7 to 11.9)	9.5%<br>(7.4 to 11.8)	5.2%<br>(-0.7 to 11.8)	Not reprted	0.3%<br>(0.1 to 0.4)	2.5%<br>(1.7 to 3.5)	10.0%<br>(8.3 to 12.2)
**Benin**	61.8%<br>(45.8 to 73.3)	2.8%<br>(1.6 to 3.7)	39.8%<br>(32.0 to 47.5)	11.1%<br>(8.5 to 13.9)	6.8%<br>(-0.9 to 14.8)	Not reprted	0.1%<br>(0.0 to 0.2)	1.6%<br>(1.1 to 2.2)	3.4%<br>(2.6 to 4.4)
**Bermuda**	54.7%<br>(39.4 to 67.6)	0.4%<br>(-0.3 to 1.5)	0.0%<br>(0.0 to 0.0)	8.4%<br>(6.3 to 10.6)	2.9%<br>(-0.4 to 6.7)	Not reprted	2.1%<br>(1.7 to 2.5)	2.7%<br>(1.8 to 3.6)	12.2%<br>(10.0 to 14.7)
**Bhutan**	55.2%<br>(39.9 to 67.5)	0.1%<br>(0.0 to 0.2)	6.3%<br>(1.2 to 16.9)	11.2%<br>(8.5 to 14.0)	12.2%<br>(-1.6 to 26.8)	Not reprted	8.2%<br>(7.5 to 9.6)	3.4%<br>(2.3 to 4.6)	6.4%<br>(4.9 to 8.3)
**Bolivia (Plurinational State of)**	42.4%<br>(29.4 to 54.9)	0.3%<br>(-0.1 to 0.8)	8.1%<br>(2.4 to 16.8)	8.0%<br>(6.1 to 10.0)	7.8%<br>(-1.0 to 17.4)	Not reprted	6.9%<br>(6.3 to 8.0)	2.4%<br>(1.4 to 3.3)	6.9%<br>(5.4 to 8.5)
**Bosnia and Herzegovina**	63.9%<br>(48.3 to 75.9)	0.1%<br>(-0.3 to 0.9)	6.6%<br>(0.5 to 24.1)	8.1%<br>(5.9 to 10.3)	6.1%<br>(-0.8 to 13.5)	Not reprted	9.1%<br>(8.2 to 11.0)	4.6%<br>(3.1 to 6.2)	20.1%<br>(17.1 to 23.3)
**Botswana**	63.1%<br>(47.7 to 74.4)	0.7%<br>(-0.5 to 2.4)	3.5%<br>(0.1 to 21.4)	13.1%<br>(10.2 to 16.1)	7.6%<br>(-1.0 to 16.7)	Not reprted	3.5%<br>(2.9 to 4.3)	4.2%<br>(2.8 to 5.6)	11.8%<br>(9.7 to 14.2)
**Brazil**	56.3%<br>(41.6 to 67.9)	0.4%<br>(0.2 to 0.7)	1.9%<br>(0.4 to 5.4)	9.9%<br>(7.9 to 12.0)	6.3%<br>(-0.8 to 14.4)	Not reprted	2.1%<br>(1.7 to 2.5)	3.7%<br>(2.5 to 4.9)	13.3%<br>(11.2 to 15.7)
**Brunei Darussalam**	55.1%<br>(39.9 to 67.7)	0.6%<br>(0.4 to 0.9)	0.0%<br>(0.0 to 0.0)	11.4%<br>(8.4 to 14.4)	4.0%<br>(-0.5 to 9.3)	Not reprted	0.0%<br>(0.0 to 0.1)	3.1%<br>(2.1 to 4.2)	13.3%<br>(11.0 to 16.4)
**Bulgaria**	62.5%<br>(47.6 to 73.5)	0.2%<br>(-0.4 to 1.3)	1.0%<br>(0.0 to 8.5)	8.1%<br>(6.1 to 10.2)	3.9%<br>(-0.5 to 8.9)	Not reprted	9.0%<br>(8.1 to 11.3)	4.4%<br>(2.9 to 5.8)	20.6%<br>(18.0 to 23.1)
**Burkina Faso**	54.6%<br>(39.4 to 67.0)	6.4%<br>(4.7 to 8.7)	39.7%<br>(32.0 to 47.3)	10.6%<br>(8.2 to 13.2)	11.5%<br>(-1.5 to 25.2)	Not reprted	0.3%<br>(0.1 to 0.6)	3.0%<br>(2.0 to 4.2)	4.9%<br>(3.8 to 6.1)

**Burundi**	57.6%<br>(42.0 to 69.8)	0.0%<br>(0.0 to 0.1)	45.8%<br>(38.2 to 53.2)	8.6%<br>(6.3 to 11.1)	8.9%<br>(-1.2 to 19.7)	Not reprted	2.7%<br>(2.0 to 3.9)	1.6%<br>(1.0 to 2.3)	5.2%<br>(4.0 to 6.6)
**Cv¥te d'Ivoire**	62.2%<br>(48.4 to 73.7)	0.9%<br>(0.6 to 1.1)	32.2%<br>(22.9 to 41.4)	11.0%<br>(8.5 to 13.6)	7.6%<br>(-1.0 to 17.0)	Not reprted	0.1%<br>(0.0 to 0.2)	3.5%<br>(2.3 to 4.7)	6.5%<br>(5.1 to 8.1)
**Cabo Verde**	64.9%<br>(50.2 to 76.5)	0.1%<br>(-0.1 to 0.3)	8.2%<br>(2.3 to 17.8)	10.8%<br>(8.2 to 13.4)	6.1%<br>(-0.8 to 13.8)	Not reprted	2.4%<br>(1.9 to 3.3)	2.6%<br>(1.7 to 3.6)	5.0%<br>(3.9 to 6.1)
**Cambodia**	45.5%<br>(31.3 to 58.0)	2.4%<br>(2.1 to 2.7)	37.3%<br>(28.6 to 45.5)	12.4%<br>(9.4 to 15.5)	7.5%<br>(-1.0 to 16.8)	Not reprted	0.5%<br>(0.1 to 1.1)	5.2%<br>(3.5 to 6.9)	16.5%<br>(13.6 to 19.5)
**Cameroon**	63.7%<br>(49.4 to 74.6)	1.7%<br>(1.2 to 2.6)	33.4%<br>(25.2 to 41.5)	13.6%<br>(10.9 to 16.4)	8.4%<br>(-1.1 to 18.5)	Not reprted	0.9%<br>(0.7 to 1.3)	2.0%<br>(1.3 to 2.7)	5.3%<br>(4.0 to 6.6)
**Canada**	46.7%<br>(33.3 to 59.1)	-0.1%<br>(-0.3 to 0.1)	0.0%<br>(0.0 to 0.0)	7.9%<br>(5.6 to 10.3)	2.7%<br>(-0.3 to 6.2)	Not reprted	7.9%<br>(6.9 to 8.9)	2.9%<br>(2.0 to 3.9)	14.0%<br>(11.8 to 16.4)
**Central African Republic**	57.6%<br>(42.0 to 71.1)	1.1%<br>(0.2 to 1.7)	44.9%<br>(37.6 to 52.2)	13.2%<br>(10.4 to 16.2)	9.1%<br>(-1.2 to 20.8)	Not reprted	0.2%<br>(0.1 to 0.3)	2.2%<br>(1.4 to 2.9)	5.9%<br>(4.4 to 7.7)
**Chad**	54.2%<br>(39.0 to 66.6)	5.6%<br>(3.7 to 8.4)	38.9%<br>(30.7 to 46.3)	10.6%<br>(8.3 to 13.3)	11.7%<br>(-1.6 to 25.4)	Not reprted	0.6%<br>(0.2 to 1.4)	2.3%<br>(1.5 to 3.1)	5.3%<br>(3.9 to 7.1)
**Chile**	57.6%<br>(42.7 to 69.1)	0.0%<br>(0.0 to 0.0)	0.6%<br>(0.0 to 6.6)	7.3%<br>(5.4 to 9.5)	2.5%<br>(-0.3 to 5.7)	Not reprted	8.4%<br>(7.6 to 9.6)	5.0%<br>(3.3 to 6.8)	13.4%<br>(11.5 to 15.3)
**China**	55.8%<br>(41.9 to 67.9)	0.6%<br>(-0.3 to 2.0)	5.9%<br>(0.9 to 18.5)	7.9%<br>(5.6 to 10.1)	8.4%<br>(-1.1 to 18.7)	Not reprted	7.6%<br>(6.9 to 8.4)	5.4%<br>(3.7 to 7.2)	19.8%<br>(16.5 to 23.2)
**Colombia**	57.6%<br>(42.8 to 69.5)	0.3%<br>(0.3 to 0.4)	1.2%<br>(0.0 to 6.6)	11.1%<br>(8.5 to 13.8)	6.1%<br>(-0.8 to 13.5)	Not reprted	4.1%<br>(3.4 to 5.1)	2.7%<br>(1.8 to 3.8)	7.1%<br>(5.8 to 8.4)
**Comoros**	59.5%<br>(44.3 to 71.8)	0.0%<br>(0.0 to 0.1)	41.1%<br>(33.7 to 48.3)	8.5%<br>(6.2 to 11.0)	6.5%<br>(-0.9 to 14.9)	Not reprted	0.4%<br>(0.1 to 0.8)	3.9%<br>(2.6 to 5.3)	6.4%<br>(4.6 to 8.3)
**Congo**	61.5%<br>(45.7 to 73.2)	0.2%<br>(0.0 to 0.5)	22.5%<br>(10.6 to 34.0)	13.2%<br>(10.2 to 16.3)	4.9%<br>(-0.6 to 11.2)	Not reprted	0.2%<br>(0.1 to 0.4)	2.7%<br>(1.8 to 3.8)	6.3%<br>(4.7 to 8.0)
**Cook Islands**	57.0%<br>(42.4 to 68.9)	0.2%<br>(-0.1 to 0.6)	0.1%<br>(0.0 to 1.3)	10.3%<br>(8.0 to 12.8)	1.4%<br>(-0.2 to 3.3)	Not reprted	0.0%<br>(0.0 to 0.0)	5.3%<br>(3.6 to 7.2)	14.9%<br>(12.3 to 18.2)
**Costa Rica**	61.0%<br>(45.5 to 72.6)	0.0%<br>(0.0 to 0.1)	0.7%<br>(0.0 to 4.5)	13.8%<br>(10.8 to 16.8)	7.0%<br>(-1.0 to 15.8)	Not reprted	2.3%<br>(1.6 to 3.2)	2.9%<br>(2.0 to 3.9)	10.5%<br>(8.8 to 12.4)
**Croatia**	64.6%<br>(49.6 to 76.1)	0.2%<br>(-0.5 to 1.4)	0.2%<br>(0.0 to 2.0)	8.1%<br>(6.0 to 10.4)	4.1%<br>(-0.5 to 9.4)	Not reprted	8.6%<br>(7.4 to 10.0)	5.3%<br>(3.6 to 7.1)	19.6%<br>(16.9 to 22.7)
**Cuba**	48.9%<br>(34.3 to 61.4)	0.6%<br>(0.2 to 1.2)	0.6%<br>(0.1 to 2.0)	8.3%<br>(6.3 to 10.5)	8.4%<br>(-1.1 to 18.7)	Not reprted	0.4%<br>(0.1 to 0.7)	3.8%<br>(2.5 to 5.1)	17.7%<br>(14.9 to 20.4)
**Cyprus**	58.1%<br>(43.3 to 69.8)	1.5%<br>(-0.4 to 4.8)	0.0%<br>(0.0 to 0.0)	8.8%<br>(6.2 to 11.5)	5.6%<br>(-0.7 to 12.4)	Not reprted	4.7%<br>(3.5 to 6.0)	3.4%<br>(2.3 to 4.5)	15.9%<br>(13.1 to 18.8)
**Czechia**	60.6%<br>(44.5 to 72.0)	0.0%<br>(-0.2 to 0.1)	0.0%<br>(0.0 to 0.2)	7.6%<br>(5.6 to 9.7)	2.9%<br>(-0.4 to 6.5)	Not reprted	9.3%<br>(8.1 to 11.0)	3.9%<br>(2.6 to 5.1)	18.8%<br>(16.3 to 21.5)
**Democratic People's Republic of Korea**	52.1%<br>(37.3 to 65.1)	0.3%<br>(-0.5 to 1.6)	41.3%<br>(34.0 to 48.5)	8.5%<br>(6.6 to 10.5)	7.0%<br>(-0.9 to 15.6)	Not reprted	8.5%<br>(7.7 to 9.9)	5.8%<br>(4.0 to 7.6)	16.6%<br>(14.1 to 19.5)
**Democratic Republic of the Congo**	56.7%<br>(42.0 to 69.0)	0.3%<br>(-0.1 to 0.7)	44.4%<br>(37.1 to 51.4)	13.3%<br>(10.4 to 16.4)	7.5%<br>(-1.0 to 17.0)	Not reprted	1.1%<br>(0.9 to 1.3)	2.0%<br>(1.2 to 2.8)	4.1%<br>(3.1 to 5.3)

**Denmark**	60.6% (46.0 to 72.4)	0.0% (0.0 to 0.0)	0.0% (0.0 to 0.0)	8.2% (6.0 to 10.6)	3.6% (-0.4 to 8.0)	Not reprted	9.3% (8.4 to 12.3)	2.2% (1.5 to 3.0)	17.7% (15.0 to 20.4)
**Djibouti**	60.2% (44.6 to 72.2)	7.4% (4.5 to 11.7)	12.0% (6.0 to 19.2)	8.5% (6.2 to 11.0)	7.4% (-1.0 to 16.2)	Not reprted	1.9% (0.6 to 3.9)	4.6% (2.9 to 6.4)	11.7% (9.1 to 14.6)
**Dominica**	57.0% (42.4 to 69.8)	0.3% (0.1 to 0.4)	1.7% (0.1 to 6.6)	9.9% (7.7 to 12.4)	4.8% (-0.6 to 10.7)	Not reprted	0.0% (-0.1 to 0.2)	2.4% (1.6 to 3.3)	7.4% (5.9 to 9.2)
**Dominican Republic**	53.8% (37.7 to 66.5)	0.2% (0.1 to 0.4)	0.9% (0.0 to 6.0)	9.0% (7.0 to 11.1)	10.0% (-1.3 to 22.2)	Not reprted	0.7% (0.4 to 0.9)	2.6% (1.8 to 3.5)	13.1% (10.8 to 15.9)
**Ecuador**	43.9% (30.3 to 56.1)	0.0% (0.0 to 0.1)	1.2% (0.1 to 4.9)	8.0% (6.1 to 10.0)	4.7% (-0.6 to 10.5)	Not reprted	4.0% (3.5 to 4.8)	2.1% (1.3 to 2.9)	7.5% (6.3 to 8.9)
**Egypt**	56.4% (41.5 to 67.8)	3.5% (-0.8 to 9.7)	0.1% (0.0 to 0.3)	10.6% (7.9 to 13.4)	11.5% (-1.7 to 25.2)	Not reprted	3.0% (1.8 to 3.9)	4.9% (3.3 to 6.6)	14.7% (10.8 to 18.6)
**El Salvador**	54.5% (39.3 to 67.1)	0.6% (0.5 to 0.8)	5.2% (1.1 to 12.8)	11.4% (8.9 to 13.8)	11.1% (-1.5 to 24.4)	Not reprted	0.1% (0.0 to 0.1)	2.7% (1.8 to 3.6)	7.7% (6.1 to 9.3)
**Equatorial Guinea**	62.3% (46.9 to 73.8)	0.0% (-0.1 to 0.1)	0.6% (0.0 to 7.1)	13.1% (10.1 to 16.2)	5.6% (-0.7 to 12.9)	Not reprted	0.4% (0.3 to 0.5)	4.0% (2.5 to 5.6)	5.6% (4.0 to 7.7)
**Eritrea**	53.2% (37.9 to 66.5)	2.8% (1.5 to 4.6)	36.7% (29.8 to 44.1)	8.4% (6.1 to 10.9)	7.1% (-0.9 to 15.9)	Not reprted	2.1% (1.7 to 2.7)	2.6% (1.8 to 3.5)	4.6% (3.4 to 6.1)
**Estonia**	64.8% (49.1 to 76.3)	-0.1% (-0.3 to 0.0)	0.2% (0.0 to 2.0)	11.5% (8.7 to 14.4)	2.8% (-0.4 to 6.4)	Not reprted	9.1% (7.9 to 10.2)	3.4% (2.3 to 4.7)	20.9% (18.2 to 23.5)
**Eswatini**	63.5% (48.2 to 74.7)	0.2% (-0.2 to 0.7)	15.0% (2.8 to 33.2)	13.5% (10.7 to 16.5)	7.5% (-1.0 to 16.7)	Not reprted	5.5% (4.9 to 6.2)	2.7% (1.7 to 3.8)	5.4% (4.1 to 6.8)
**Ethiopia**	47.7% (33.9 to 59.4)	0.3% (0.2 to 0.6)	41.9% (34.8 to 48.7)	8.2% (5.9 to 10.6)	10.3% (-1.4 to 22.5)	Not reprted	4.8% (4.3 to 5.5)	1.8% (1.2 to 2.4)	3.3% (2.5 to 4.1)
**Fiji**	61.8% (46.4 to 73.3)	0.1% (0.0 to 0.3)	7.5% (0.9 to 20.3)	11.4% (8.8 to 14.0)	2.1% (-0.3 to 4.7)	Not reprted	0.5% (-0.2 to 0.9)	4.6% (2.9 to 6.2)	11.8% (9.9 to 14.1)
**Finland**	61.1% (45.8 to 72.9)	-0.1% (-0.3 to 0.0)	0.0% (0.0 to 0.0)	7.4% (5.3 to 9.5)	1.9% (-0.2 to 4.3)	Not reprted	9.0% (7.2 to 10.2)	1.9% (1.3 to 2.6)	12.4% (10.5 to 14.5)
**France**	57.2% (42.6 to 69.6)	0.0% (-0.1 to 0.2)	0.0% (0.0 to 0.0)	6.9% (5.0 to 9.0)	4.1% (-0.5 to 9.4)	Not reprted	8.0% (7.1 to 9.7)	2.7% (1.9 to 3.7)	14.9% (12.7 to 17.4)
**Gabon**	61.1% (46.2 to 73.5)	0.1% (0.0 to 0.2)	1.0% (0.0 to 7.2)	13.2% (10.2 to 16.3)	4.6% (-0.6 to 10.5)	Not reprted	0.3% (0.1 to 0.4)	2.9% (1.9 to 4.0)	6.3% (4.8 to 8.3)
**Gambia**	63.5% (47.5 to 74.6)	2.2% (1.4 to 3.3)	39.3% (31.5 to 46.9)	11.0% (8.5 to 13.7)	8.6% (-1.1 to 18.8)	Not reprted	0.2% (0.0 to 0.5)	4.0% (2.7 to 5.4)	6.3% (5.0 to 8.0)
**Georgia**	65.6% (50.2 to 76.9)	0.2% (-0.3 to 1.2)	4.8% (0.4 to 16.2)	12.9% (9.9 to 16.1)	6.6% (-0.9 to 14.9)	Not reprted	8.9% (8.1 to 11.1)	5.3% (3.6 to 7.1)	21.0% (18.4 to 23.6)
**Germany**	59.7% (44.6 to 71.4)	0.0% (-0.1 to 0.2)	0.0% (0.0 to 0.0)	7.9% (5.7 to 10.1)	2.6% (-0.3 to 5.9)	Not reprted	8.4% (7.4 to 10.5)	2.5% (1.7 to 3.4)	15.5% (13.1 to 18.0)
**Ghana**	61.4% (46.5 to 72.9)	2.2% (1.9 to 2.6)	27.8% (15.4 to 38.9)	8.9% (6.5 to 11.4)	5.2% (-0.7 to 11.6)	Not reprted	0.1% (0.0 to 0.2)	1.8% (1.2 to 2.4)	5.0% (3.9 to 6.3)
**Greece**	51.2% (36.7 to 63.3)	0.3% (-0.2 to 1.2)	0.0% (0.0 to 0.2)	8.1% (5.9 to 10.3)	5.4% (-0.7 to 12.1)	Not reprted	7.5% (6.9 to 8.2)	3.8% (2.6 to 5.1)	20.2% (17.2 to 23.2)

**Greenland**	47.9% (33.0 to 60.8)	-0.2% (-0.5 to 0.0)	0.0% (0.0 to 0.0)	9.1% (6.8 to 11.4)	2.6% (-0.3 to 6.0)	Not reprted	8.4% (5.1 to 12.8)	4.3% (2.9 to 5.8)	22.3% (18.8 to 26.1)
**Grenada**	56.7% (41.3 to 69.3)	0.5% (0.3 to 0.7)	0.3% (0.0 to 1.8)	10.0% (7.7 to 12.5)	7.4% (-0.9 to 17.1)	Not reprted	0.0% (0.0 to 0.0)	2.3% (1.6 to 3.2)	7.6% (6.2 to 9.2)
**Guam**	56.1% (40.1 to 68.3)	0.9% (0.4 to 1.4)	0.1% (0.0 to 0.3)	10.6% (8.4 to 13.0)	1.3% (-0.2 to 2.9)	Not reprted	0.0% (0.0 to 0.0)	5.2% (3.6 to 7.0)	16.9% (14.1 to 20.5)
**Guatemala**	55.4% (40.8 to 66.5)	0.2% (0.1 to 0.3)	18.3% (5.2 to 33.5)	12.2% (9.4 to 15.0)	12.6% (-1.8 to 26.9)	Not reprted	3.1% (1.8 to 4.1)	2.6% (1.6 to 3.6)	6.0% (4.8 to 7.3)
**Guinea**	56.7% (41.6 to 69.0)	1.2% (0.6 to 2.0)	40.2% (32.9 to 47.6)	10.9% (8.5 to 13.5)	9.5% (-1.3 to 21.0)	Not reprted	0.2% (0.1 to 0.4)	3.2% (2.2 to 4.3)	5.5% (4.2 to 6.8)
**Guinea-Bissau**	60.0% (43.7 to 72.4)	2.3% (1.7 to 2.8)	40.7% (33.4 to 48.2)	11.3% (8.9 to 14.0)	8.7% (-1.2 to 19.6)	Not reprted	0.1% (0.0 to 0.3)	2.4% (1.6 to 3.2)	4.3% (3.2 to 5.5)
**Guyana**	55.8% (40.6 to 68.2)	0.3% (0.0 to 0.6)	2.4% (0.5 to 6.8)	9.8% (7.7 to 12.0)	8.0% (-1.1 to 17.5)	Not reprted	0.1% (0.0 to 0.2)	2.8% (1.9 to 3.7)	9.0% (7.3 to 10.8)
**Haiti**	61.0% (46.7 to 72.5)	0.4% (0.3 to 0.5)	42.6% (35.0 to 50.3)	9.3% (7.2 to 11.8)	12.7% (-1.8 to 27.4)	Not reprted	0.2% (0.1 to 0.4)	1.7% (1.1 to 2.4)	4.6% (3.5 to 5.8)
**Honduras**	56.9% (41.6 to 69.3)	0.4% (0.1 to 0.9)	25.5% (15.8 to 34.4)	13.0% (10.0 to 15.9)	12.9% (-1.8 to 27.5)	Not reprted	1.2% (0.5 to 1.7)	3.9% (2.7 to 5.2)	9.0% (7.2 to 11.2)
**Hungary**	67.4% (52.5 to 78.7)	0.3% (-0.8 to 1.6)	0.5% (0.0 to 6.0)	7.7% (5.9 to 9.7)	4.0% (-0.5 to 9.3)	Not reprted	8.3% (7.1 to 9.7)	4.1% (2.8 to 5.4)	19.8% (17.1 to 22.6)
**Iceland**	55.2% (40.6 to 66.8)	0.0% (0.0 to 0.0)	0.0% (0.0 to 0.0)	6.4% (4.5 to 8.3)	3.4% (-0.4 to 7.6)	Not reprted	10.9% (9.5 to 12.0)	2.1% (1.4 to 2.8)	12.5% (10.3 to 14.9)
**India**	55.2% (40.2 to 67.1)	2.7% (0.8 to 5.1)	23.0% (14.6 to 33.4)	11.6% (8.9 to 14.7)	10.5% (-1.4 to 23.1)	Not reprted	2.2% (0.8 to 3.9)	4.2% (2.9 to 5.6)	10.3% (8.4 to 12.3)
**Indonesia**	66.9% (51.8 to 76.9)	0.4% (0.3 to 0.6)	9.2% (3.1 to 19.6)	13.5% (10.3 to 16.8)	6.7% (-0.9 to 15.0)	Not reprted	0.4% (0.3 to 0.7)	5.6% (3.9 to 7.6)	14.8% (11.9 to 18.1)
**Iran (Islamic Republic of)**	51.5% (38.6 to 62.4)	2.0% (-0.3 to 5.0)	0.0% (0.0 to 0.2)	10.5% (7.9 to 13.2)	11.5% (-1.6 to 24.9)	Not reprted	6.7% (5.6 to 7.8)	3.3% (2.3 to 4.5)	11.7% (9.9 to 13.6)
**Iraq**	63.1% (48.0 to 73.8)	9.6% (0.8 to 19.7)	0.1% (0.0 to 0.9)	11.2% (8.3 to 14.2)	7.2% (-1.0 to 16.1)	Not reprted	6.0% (2.7 to 10.1)	5.9% (4.0 to 7.9)	16.8% (14.2 to 19.5)
**Ireland**	57.5% (42.7 to 68.9)	0.0% (0.0 to 0.0)	0.0% (0.0 to 0.0)	10.5% (7.8 to 13.1)	4.0% (-0.5 to 9.2)	Not reprted	7.5% (6.1 to 10.4)	2.6% (1.8 to 3.6)	12.8% (10.8 to 15.0)
**Israel**	59.6% (44.3 to 71.4)	1.5% (-0.7 to 5.3)	0.0% (0.0 to 0.0)	8.7% (6.4 to 11.1)	3.2% (-0.4 to 7.2)	Not reprted	3.2% (2.2 to 4.0)	2.9% (2.0 to 3.9)	13.4% (11.4 to 15.7)
**Italy**	50.1% (35.1 to 61.5)	0.1% (-0.3 to 0.7)	0.0% (0.0 to 0.1)	6.9% (5.0 to 8.9)	4.3% (-0.6 to 9.8)	Not reprted	8.2% (7.3 to 9.1)	3.2% (2.2 to 4.3)	13.6% (11.6 to 16.0)
**Jamaica**	57.3% (42.2 to 69.7)	0.5% (0.1 to 1.0)	3.6% (0.6 to 10.0)	9.3% (7.2 to 11.6)	7.3% (-0.9 to 16.7)	Not reprted	0.1% (0.0 to 0.2)	2.6% (1.8 to 3.5)	10.3% (8.6 to 12.5)
**Japan**	55.3% (41.4 to 66.3)	0.1% (-0.2 to 0.5)	0.0% (0.0 to 0.0)	10.1% (7.4 to 13.0)	2.5% (-0.3 to 5.7)	Not reprted	7.2% (6.5 to 7.9)	3.3% (2.2 to 4.5)	17.9% (15.4 to 20.3)
**Jordan**	50.2% (36.2 to 60.9)	1.0% (-0.6 to 3.9)	0.0% (0.0 to 0.1)	9.6% (7.0 to 12.2)	5.7% (-0.8 to 13.0)	Not reprted	4.8% (4.0 to 5.9)	5.2% (3.6 to 7.0)	15.9% (13.6 to 18.4)

**Kazakhstan**	67.6%<br>(52.0 to 78.6)	0.2%<br>(-0.6 to 1.3)	0.3%<br>(0.0 to 3.0)	13.2%<br>(10.2 to 16.2)	2.9%<br>(-0.4 to 6.6)	Not reprted	9.1%<br>(7.9 to 10.5)	4.3%<br>(2.9 to 5.8)	14.0%<br>(12.0 to 16.1)
**Kenya**	58.7%<br>(44.2 to 69.7)	0.2%<br>(0.1 to 0.3)	38.6%<br>(30.6 to 45.8)	8.7%<br>(6.2 to 11.1)	6.3%<br>(-0.8 to 14.2)	Not reprted	3.8%<br>(3.3 to 4.9)	2.1%<br>(1.4 to 2.8)	6.1%<br>(4.9 to 7.5)
**Kiribati**	47.8%<br>(32.4 to 59.8)	0.2%<br>(0.0 to 0.5)	29.3%<br>(23.2 to 36.0)	11.1%<br>(8.8 to 13.6)	2.7%<br>(-0.3 to 6.2)	Not reprted	0.0%<br>(-0.1 to 0.1)	8.6%<br>(6.0 to 11.4)	27.2%<br>(23.6 to 30.9)
**Kuwait**	47.9%<br>(34.8 to 58.7)	11.1%<br>(2.5 to 20.2)	0.0%<br>(0.0 to 0.0)	8.9%<br>(6.5 to 11.4)	4.4%<br>(-0.6 to 9.9)	Not reprted	4.6%<br>(1.2 to 8.8)	4.5%<br>(3.1 to 6.0)	14.8%<br>(12.4 to 17.2)
**Kyrgyzstan**	53.8%<br>(38.2 to 67.7)	0.0%<br>(-0.5 to 0.7)	16.7%<br>(9.8 to 24.2)	12.7%<br>(10.3 to 15.3)	4.5%<br>(-0.6 to 10.3)	Not reprted	9.3%<br>(7.6 to 10.6)	5.7%<br>(4.0 to 7.6)	23.2%<br>(20.4 to 26.2)
**Lao People's Democratic Republic**	55.4%<br>(39.9 to 67.7)	1.0%<br>(0.2 to 2.2)	30.3%<br>(13.3 to 45.5)	13.4%<br>(10.4 to 16.7)	8.2%<br>(-1.1 to 17.8)	Not reprted	1.6%<br>(1.1 to 2.2)	6.0%<br>(4.0 to 8.1)	16.2%<br>(13.6 to 18.8)
**Latvia**	66.5%<br>(50.9 to 78.1)	-0.1%<br>(-0.3 to 0.0)	0.5%<br>(0.0 to 4.9)	11.7%<br>(9.0 to 14.7)	2.9%<br>(-0.4 to 6.7)	Not reprted	10.2%<br>(8.6 to 11.3)	4.3%<br>(2.8 to 5.7)	19.5%<br>(16.8 to 22.2)
**Lebanon**	55.5%<br>(40.8 to 66.2)	0.2%<br>(-0.4 to 1.1)	0.1%<br>(0.0 to 0.3)	9.9%<br>(7.3 to 12.7)	4.5%<br>(-0.6 to 10.1)	Not reprted	5.7%<br>(5.0 to 6.7)	5.5%<br>(3.7 to 7.3)	21.9%<br>(18.6 to 25.2)
**Lesotho**	57.9%<br>(42.6 to 70.3)	0.0%<br>(0.0 to 0.0)	32.3%<br>(25.1 to 39.4)	13.4%<br>(10.5 to 16.2)	9.1%<br>(-1.2 to 20.4)	Not reprted	8.9%<br>(7.7 to 11.3)	5.3%<br>(3.6 to 6.9)	13.6%<br>(10.7 to 16.8)
**Liberia**	61.6%<br>(46.3 to 73.4)	0.2%<br>(0.1 to 0.4)	42.4%<br>(35.6 to 49.3)	11.0%<br>(8.5 to 13.6)	8.6%<br>(-1.1 to 19.2)	Not reprted	0.1%<br>(0.0 to 0.3)	2.1%<br>(1.4 to 2.9)	4.4%<br>(3.4 to 5.5)
**Libya**	50.6%<br>(36.9 to 62.0)	3.0%<br>(-0.3 to 8.3)	0.1%<br>(0.0 to 0.4)	8.7%<br>(6.3 to 11.2)	5.0%<br>(-0.7 to 11.7)	Not reprted	3.8%<br>(2.8 to 4.7)	6.3%<br>(4.2 to 8.6)	8.8%<br>(6.2 to 12.1)
**Lithuania**	68.3%<br>(53.3 to 79.2)	-0.1%<br>(-0.4 to 0.0)	0.1%<br>(0.0 to 0.8)	11.7%<br>(9.0 to 14.7)	2.9%<br>(-0.4 to 6.5)	Not reprted	10.0%<br>(8.2 to 11.3)	3.5%<br>(2.4 to 4.9)	18.9%<br>(16.2 to 21.7)
**Luxembourg**	60.4%<br>(45.4 to 72.3)	0.0%<br>(-0.1 to 0.1)	0.0%<br>(0.0 to 0.0)	8.3%<br>(6.0 to 10.7)	3.3%<br>(-0.4 to 7.5)	Not reprted	8.8%<br>(7.8 to 11.6)	2.5%<br>(1.7 to 3.4)	14.2%<br>(11.6 to 17.2)
**Madagascar**	56.4%<br>(41.3 to 67.8)	0.3%<br>(-0.1 to 0.9)	46.5%<br>(38.8 to 53.6)	8.3%<br>(6.0 to 10.6)	6.6%<br>(-0.9 to 14.5)	Not reprted	3.6%<br>(3.1 to 4.3)	2.5%<br>(1.6 to 3.6)	4.5%<br>(3.6 to 5.5)
**Malawi**	62.7%<br>(46.4 to 74.3)	0.7%<br>(-0.4 to 2.3)	45.7%<br>(38.4 to 52.8)	9.0%<br>(6.6 to 11.5)	7.9%<br>(-1.0 to 17.9)	Not reprted	2.7%<br>(2.1 to 3.2)	3.0%<br>(2.0 to 4.1)	9.9%<br>(7.6 to 12.5)
**Malaysia**	67.9%<br>(52.4 to 78.3)	0.8%<br>(0.6 to 1.1)	0.0%<br>(0.0 to 0.3)	14.1%<br>(10.8 to 17.5)	5.1%<br>(-0.6 to 11.7)	Not reprted	0.1%<br>(0.0 to 0.2)	5.5%<br>(3.7 to 7.3)	12.9%<br>(10.9 to 15.1)
**Maldives**	60.0%<br>(44.6 to 71.8)	0.9%<br>(0.5 to 1.4)	0.9%<br>(0.0 to 6.7)	12.5%<br>(9.4 to 15.7)	6.0%<br>(-0.8 to 13.3)	Not reprted	0.0%<br>(-0.1 to 0.0)	6.1%<br>(4.1 to 8.1)	16.4%<br>(13.7 to 19.2)
**Mali**	52.9%<br>(38.7 to 65.5)	7.5%<br>(5.0 to 11.1)	38.7%<br>(30.8 to 46.1)	10.8%<br>(8.5 to 13.3)	11.0%<br>(-1.5 to 24.2)	Not reprted	0.4%<br>(0.1 to 0.8)	4.0%<br>(2.7 to 5.5)	5.3%<br>(4.1 to 6.9)
**Malta**	56.5%<br>(42.1 to 68.7)	0.6%<br>(-0.6 to 2.5)	0.0%<br>(0.0 to 0.0)	7.8%<br>(5.8 to 10.0)	8.5%<br>(-1.2 to 18.8)	Not reprted	4.2%<br>(2.4 to 5.7)	2.7%<br>(1.8 to 3.6)	14.3%<br>(12.1 to 16.7)
**Marshall Islands**	47.3%<br>(33.5 to 60.3)	1.0%<br>(0.4 to 1.5)	18.3%<br>(11.9 to 25.4)	11.2%<br>(8.9 to 13.7)	2.6%<br>(-0.3 to 6.0)	Not reprted	0.0%<br>(0.0 to 0.0)	5.3%<br>(3.5 to 7.1)	12.9%<br>(10.5 to 15.8)
**Mauritania**	61.2%<br>(45.1 to 72.9)	10.2%<br>(5.1 to 16.8)	23.5%<br>(14.7 to 32.7)	10.9%<br>(8.3 to 13.5)	5.6%<br>(-0.7 to 12.3)	Not reprted	0.8%<br>(0.2 to 1.6)	4.7%<br>(3.2 to 6.4)	4.8%<br>(3.5 to 6.2)

**Mauritius**	59.4%<br>(44.1 to 71.4)	0.1%<br>(-0.1 to 0.4)	0.1%<br>(0.0 to 0.6)	14.7%<br>(11.7 to 17.8)	3.7%<br>(-0.5 to 8.4)	Not reprted	1.2%<br>(0.3 to 2.0)	6.0%<br>(4.1 to 8.0)	18.1%<br>(15.7 to 20.9)
**Mexico**	52.4%<br>(38.0 to 64.1)	0.6%<br>(-0.1 to 1.5)	1.7%<br>(0.1 to 7.4)	11.7%<br>(9.1 to 14.4)	7.6%<br>(-1.0 to 16.8)	Not reprted	4.7%<br>(4.0 to 5.4)	2.6%<br>(1.8 to 3.4)	7.6%<br>(6.3 to 9.0)
**Micronesia (Federated States of)**	45.2%<br>(30.5 to 58.0)	0.9%<br>(0.4 to 1.5)	19.0%<br>(12.1 to 26.2)	11.5%<br>(9.1 to 14.0)	2.5%<br>(-0.3 to 5.7)	Not reprted	0.0%<br>(0.0 to 0.0)	7.1%<br>(4.6 to 9.7)	19.9%<br>(16.3 to 23.0)
**Monaco**	56.7%<br>(41.4 to 68.7)	0.0%<br>(-0.1 to 0.1)	0.0%<br>(0.0 to 0.0)	7.5%<br>(5.6 to 9.5)	2.9%<br>(-0.4 to 6.6)	Not reprted	7.9%<br>(7.3 to 8.7)	3.0%<br>(2.0 to 4.1)	17.5%<br>(14.3 to 21.3)
**Mongolia**	64.9%<br>(49.8 to 76.8)	-0.1%<br>(-0.2 to 0.0)	7.8%<br>(0.6 to 27.5)	13.2%<br>(10.3 to 16.1)	5.5%<br>(-0.7 to 12.5)	Not reprted	10.1%<br>(7.5 to 12.9)	4.7%<br>(3.3 to 6.3)	19.0%<br>(15.8 to 22.3)
**Montenegro**	63.2%<br>(48.0 to 74.7)	0.1%<br>(-0.2 to 0.5)	3.2%<br>(0.0 to 21.6)	8.3%<br>(5.6 to 11.0)	3.2%<br>(-0.4 to 7.2)	Not reprted	9.5%<br>(8.5 to 10.8)	4.7%<br>(3.2 to 6.2)	14.7%<br>(12.2 to 17.7)
**Morocco**	61.8%<br>(45.9 to 73.2)	0.7%<br>(-0.1 to 2.0)	1.1%<br>(0.2 to 3.4)	9.8%<br>(7.1 to 12.6)	6.1%<br>(-0.9 to 13.8)	Not reprted	6.3%<br>(5.2 to 7.5)	4.9%<br>(3.3 to 6.5)	6.3%<br>(4.6 to 8.4)
**Mozambique**	65.3%<br>(49.6 to 77.0)	0.8%<br>(-0.4 to 2.5)	45.7%<br>(38.2 to 52.9)	9.1%<br>(6.6 to 11.7)	10.4%<br>(-1.4 to 22.7)	Not reprted	1.5%<br>(1.1 to 2.0)	2.4%<br>(1.5 to 3.4)	8.5%<br>(6.8 to 10.3)
**Myanmar**	57.7%<br>(42.9 to 69.7)	2.4%<br>(1.5 to 3.7)	28.5%<br>(14.5 to 40.9)	12.8%<br>(9.8 to 15.7)	7.5%<br>(-1.0 to 16.9)	Not reprted	0.9%<br>(0.6 to 1.4)	4.5%<br>(2.9 to 6.2)	13.0%<br>(10.9 to 15.5)
**Namibia**	59.2%<br>(43.2 to 72.3)	0.6%<br>(-0.6 to 2.2)	10.4%<br>(1.0 to 30.4)	12.8%<br>(9.9 to 15.8)	6.4%<br>(-0.9 to 14.6)	Not reprted	3.1%<br>(2.6 to 3.7)	3.6%<br>(2.3 to 5.1)	9.0%<br>(7.5 to 10.8)
**Nauru**	62.0%<br>(46.6 to 73.4)	1.0%<br>(0.6 to 1.6)	0.7%<br>(0.0 to 3.3)	11.8%<br>(9.3 to 14.3)	1.7%<br>(-0.2 to 3.9)	Not reprted	0.0%<br>(0.0 to 0.0)	7.2%<br>(4.6 to 9.5)	18.2%<br>(15.1 to 21.4)
**Nepal**	44.6%<br>(30.9 to 56.8)	0.9%<br>(-0.5 to 2.8)	33.3%<br>(26.0 to 40.9)	13.9%<br>(10.7 to 17.0)	13.6%<br>(-1.9 to 29.4)	Not reprted	4.4%<br>(3.8 to 5.3)	4.2%<br>(2.7 to 5.6)	14.3%<br>(11.9 to 16.8)
**Netherlands**	57.6%<br>(42.7 to 69.1)	0.0%<br>(0.0 to 0.1)	0.0%<br>(0.0 to 0.0)	8.5%<br>(6.0 to 11.0)	3.4%<br>(-0.4 to 7.9)	Not reprted	7.8%<br>(6.7 to 10.7)	2.1%<br>(1.4 to 2.8)	14.0%<br>(11.9 to 16.5)
**New Zealand**	52.7%<br>(38.0 to 64.9)	0.0%<br>(0.0 to 0.0)	0.0%<br>(0.0 to 0.0)	8.3%<br>(5.8 to 10.8)	5.2%<br>(-0.7 to 12.1)	Not reprted	7.6%<br>(6.6 to 9.1)	2.4%<br>(1.6 to 3.3)	11.9%<br>(9.9 to 14.2)
**Nicaragua**	59.7%<br>(43.7 to 71.0)	0.8%<br>(0.7 to 0.8)	22.6%<br>(12.3 to 31.9)	15.6%<br>(12.4 to 18.7)	8.5%<br>(-1.2 to 18.9)	Not reprted	0.3%<br>(0.2 to 0.5)	4.4%<br>(3.0 to 5.9)	8.9%<br>(7.1 to 11.1)
**Niger**	58.2%<br>(42.6 to 69.7)	8.0%<br>(5.1 to 11.8)	41.0%<br>(33.2 to 48.8)	10.3%<br>(7.9 to 12.8)	13.1%<br>(-1.8 to 28.3)	Not reprted	1.4%<br>(0.4 to 2.9)	2.2%<br>(1.4 to 3.0)	2.6%<br>(2.0 to 3.6)
**Nigeria**	62.3%<br>(46.7 to 73.7)	2.4%<br>(1.8 to 3.4)	25.6%<br>(15.2 to 36.3)	13.5%<br>(10.7 to 16.5)	6.1%<br>(-0.8 to 13.6)	Not reprted	0.4%<br>(0.1 to 0.8)	1.4%<br>(1.0 to 1.9)	2.8%<br>(2.0 to 3.6)
**Niue**	57.9%<br>(43.5 to 69.6)	0.2%<br>(0.2 to 0.3)	0.7%<br>(0.0 to 3.9)	11.1%<br>(8.6 to 13.6)	1.5%<br>(-0.2 to 3.5)	Not reprted	0.4%<br>(0.2 to 0.6)	4.6%<br>(3.1 to 6.1)	12.8%<br>(10.2 to 15.7)
**North Macedonia**	63.2%<br>(48.6 to 74.9)	0.2%<br>(-0.3 to 1.3)	2.7%<br>(0.1 to 14.6)	9.9%<br>(7.2 to 12.6)	4.3%<br>(-0.5 to 9.8)	Not reprted	9.4%<br>(8.5 to 12.1)	6.2%<br>(4.3 to 8.3)	18.7%<br>(15.8 to 21.5)
**Northern Mariana Islands**	58.0%<br>(43.0 to 70.4)	0.8%<br>(0.7 to 0.9)	0.2%<br>(0.0 to 1.8)	11.7%<br>(9.3 to 14.4)	1.3%<br>(-0.2 to 2.9)	Not reprted	0.2%<br>(0.1 to 0.3)	5.3%<br>(3.5 to 7.1)	14.8%<br>(12.0 to 17.9)
**Norway**	58.9%<br>(43.4 to 70.2)	0.0%<br>(0.0 to 0.0)	0.0%<br>(0.0 to 0.0)	7.7%<br>(5.4 to 10.1)	3.4%<br>(-0.4 to 7.9)	Not reprted	9.8%<br>(8.9 to 10.7)	1.9%<br>(1.3 to 2.6)	10.5%<br>(8.8 to 12.4)

**Oman**	58.8% (43.6 to 69.7)	9.0% (4.2 to 14.8)	0.0% (0.0 to 0.0)	10.4% (7.8 to 13.4)	5.8% (-0.8 to 12.9)	Not reprted	3.1% (0.7 to 6.3)	3.7% (2.5 to 4.9)	7.8% (6.3 to 9.3)
**Pakistan**	59.7% (44.1 to 71.8)	5.9% (0.8 to 12.1)	24.0% (13.8 to 34.5)	10.8% (8.4 to 13.5)	10.4% (-1.4 to 22.8)	Not reprted	5.2% (2.3 to 8.6)	5.3% (3.6 to 7.0)	10.5% (8.0 to 13.2)
**Palau**	56.5% (41.2 to 69.0)	0.7% (0.3 to 1.0)	0.0% (0.0 to 0.0)	11.2% (8.9 to 13.6)	1.3% (-0.2 to 3.0)	Not reprted	0.0% (0.0 to 0.0)	4.5% (3.0 to 6.2)	13.8% (11.4 to 17.1)
**Palestine**	47.2% (33.2 to 59.1)	1.8% (-0.6 to 6.1)	1.2% (0.4 to 2.5)	9.9% (7.2 to 12.7)	7.8% (-1.0 to 16.9)	Not reprted	3.8% (3.0 to 4.8)	4.8% (3.3 to 6.4)	13.9% (11.7 to 16.5)
**Panama**	58.8% (43.0 to 69.8)	0.2% (0.1 to 0.5)	0.4% (0.0 to 4.0)	12.3% (9.6 to 15.1)	7.0% (-0.9 to 15.6)	Not reprted	0.3% (0.2 to 0.4)	2.1% (1.4 to 2.9)	7.7% (6.2 to 9.4)
**Papua New Guinea**	41.9% (28.2 to 54.4)	0.1% (0.1 to 0.2)	40.6% (30.3 to 49.2)	10.2% (7.8 to 12.6)	3.1% (-0.4 to 7.3)	Not reprted	3.5% (3.1 to 4.3)	6.4% (4.2 to 8.5)	11.8% (9.2 to 14.7)
**Paraguay**	57.8% (42.1 to 69.8)	1.0% (-0.7 to 3.5)	6.8% (0.6 to 21.5)	10.2% (8.0 to 12.4)	6.0% (-0.8 to 13.3)	Not reprted	2.6% (1.9 to 3.4)	3.9% (2.6 to 5.3)	15.0% (12.2 to 18.3)
**Peru**	46.5% (32.5 to 59.2)	0.0% (0.0 to 0.1)	2.8% (0.2 to 12.7)	6.8% (5.2 to 8.6)	6.1% (-0.8 to 14.0)	Not reprted	6.5% (5.4 to 7.7)	1.8% (1.1 to 2.5)	7.6% (6.1 to 9.4)
**Philippines**	49.9% (35.6 to 61.6)	1.1% (1.0 to 1.2)	17.8% (9.0 to 28.8)	13.7% (10.7 to 16.8)	4.7% (-0.6 to 10.5)	Not reprted	0.1% (0.0 to 0.3)	5.2% (3.6 to 6.9)	17.2% (14.5 to 20.1)
**Poland**	57.2% (42.6 to 68.6)	0.0% (-0.2 to 0.1)	0.4% (0.0 to 4.4)	7.9% (6.0 to 10.0)	4.4% (-0.6 to 10.1)	Not reprted	9.6% (8.6 to 11.9)	4.1% (2.8 to 5.5)	19.8% (17.1 to 22.5)
**Portugal**	55.3% (40.8 to 67.3)	0.0% (0.0 to 0.1)	0.0% (0.0 to 0.1)	6.7% (5.0 to 8.5)	6.8% (-0.9 to 15.3)	Not reprted	8.7% (8.2 to 9.2)	3.4% (2.3 to 4.6)	13.0% (11.1 to 15.0)
**Puerto Rico**	57.2% (42.3 to 69.9)	0.5% (0.3 to 0.7)	0.0% (0.0 to 0.0)	9.1% (7.0 to 11.5)	3.5% (-0.4 to 7.9)	Not reprted	0.0% (-0.1 to 0.1)	1.7% (1.1 to 2.3)	11.6% (9.4 to 14.2)
**Qatar**	56.8% (42.4 to 67.8)	9.8% (3.4 to 16.9)	0.0% (0.0 to 0.0)	10.4% (7.4 to 13.4)	4.0% (-0.5 to 9.1)	Not reprted	3.6% (0.9 to 7.2)	4.4% (2.9 to 5.8)	9.3% (7.3 to 11.2)
**Republic of Korea**	42.8% (28.9 to 54.5)	0.3% (-0.5 to 1.8)	0.0% (0.0 to 0.0)	8.1% (5.6 to 10.7)	4.6% (-0.6 to 10.3)	Not reprted	7.0% (6.2 to 8.1)	3.7% (2.5 to 4.9)	16.0% (13.6 to 18.4)
**Republic of Moldova**	69.3% (53.7 to 80.2)	0.1% (-0.7 to 1.2)	5.6% (1.9 to 10.8)	13.7% (10.7 to 16.8)	4.0% (-0.5 to 9.1)	Not reprted	8.6% (7.4 to 10.4)	3.6% (2.5 to 4.9)	18.3% (15.8 to 21.1)
**Romania**	66.0% (51.5 to 77.4)	0.2% (-0.4 to 1.3)	0.6% (0.0 to 5.6)	8.4% (6.3 to 10.5)	4.1% (-0.5 to 9.0)	Not reprted	9.3% (8.3 to 11.0)	4.3% (3.0 to 5.7)	16.6% (14.4 to 19.3)
**Russian Federation**	61.5% (46.3 to 72.7)	-0.1% (-0.5 to 0.3)	0.1% (0.0 to 1.0)	10.7% (8.1 to 13.5)	3.0% (-0.4 to 7.1)	Not reprted	9.1% (7.3 to 10.9)	3.9% (2.6 to 5.3)	22.8% (20.1 to 25.7)
**Rwanda**	54.6% (39.8 to 68.0)	0.0% (0.0 to 0.0)	46.0% (38.5 to 53.7)	8.4% (6.0 to 10.9)	7.5% (-1.0 to 17.0)	Not reprted	4.4% (3.9 to 5.7)	2.5% (1.7 to 3.4)	13.0% (10.0 to 16.2)
**Saint Kitts and Nevis**	60.5% (45.8 to 72.5)	0.4% (0.2 to 0.6)	0.0% (0.0 to 0.5)	9.8% (7.6 to 12.1)	5.2% (-0.7 to 11.7)	Not reprted	0.0% (-0.1 to 0.1)	2.2% (1.4 to 3.0)	6.1% (4.8 to 7.6)
**Saint Lucia**	57.4% (42.1 to 69.7)	0.5% (0.3 to 0.8)	0.6% (0.0 to 3.3)	9.6% (7.4 to 11.8)	7.5% (-1.0 to 16.9)	Not reprted	0.0% (0.0 to 0.1)	1.9% (1.3 to 2.6)	9.3% (7.6 to 11.1)
**Saint Vincent and the Grenadines**	55.1% (39.3 to 67.8)	0.5% (0.3 to 0.7)	0.7% (0.0 to 3.0)	9.4% (7.3 to 11.7)	8.4% (-1.1 to 18.9)	Not reprted	0.0% (0.0 to 0.0)	2.5% (1.7 to 3.5)	9.3% (7.6 to 11.2)

**Samoa**	54.9%<br>(40.0 to 68.0)	0.0%<br>(0.0 to 0.1)	26.6%<br>(12.7 to 38.3)	11.2%<br>(8.7 to 13.8)	2.1%<br>(-0.3 to 5.0)	Not reprted	0.1%<br>(-0.1 to 0.3)	5.9%<br>(4.0 to 7.9)	17.2%<br>(14.5 to 20.2)
**San Marino**	57.6%<br>(42.6 to 70.2)	0.1%<br>(-0.3 to 0.6)	0.0%<br>(0.0 to 0.0)	7.6%<br>(5.6 to 9.8)	2.7%<br>(-0.4 to 6.1)	Not reprted	8.1%<br>(7.1 to 8.9)	2.4%<br>(1.6 to 3.3)	13.9%<br>(11.0 to 17.1)
**Sao Tome and Principe**	66.3%<br>(51.0 to 77.8)	0.2%<br>(0.0 to 0.5)	24.9%<br>(17.7 to 32.5)	12.1%<br>(9.5 to 14.9)	6.8%<br>(-0.9 to 15.3)	Not reprted	0.0%<br>(0.0 to 0.1)	1.3%<br>(0.9 to 1.9)	3.7%<br>(2.8 to 4.9)
**Saudi Arabia**	53.4%<br>(38.0 to 65.3)	9.1%<br>(2.6 to 16.7)	0.0%<br>(0.0 to 0.0)	11.2%<br>(8.5 to 14.0)	7.0%<br>(-0.9 to 16.0)	Not reprted	3.4%<br>(1.1 to 6.2)	4.8%<br>(3.2 to 6.3)	10.6%<br>(8.6 to 12.8)
**Senegal**	62.3%<br>(47.1 to 73.9)	4.4%<br>(3.1 to 6.2)	38.3%<br>(30.2 to 46.1)	9.7%<br>(7.4 to 12.2)	6.4%<br>(-0.9 to 14.2)	Not reprted	0.3%<br>(0.1 to 0.6)	3.9%<br>(2.7 to 5.2)	5.4%<br>(4.1 to 6.9)
**Serbia**	66.6%<br>(51.3 to 77.4)	0.3%<br>(-0.5 to 1.6)	2.6%<br>(0.1 to 15.4)	7.3%<br>(5.4 to 9.5)	3.9%<br>(-0.5 to 8.9)	Not reprted	9.1%<br>(8.0 to 11.0)	5.0%<br>(3.5 to 6.7)	18.4%<br>(15.6 to 21.3)
**Seychelles**	61.3%<br>(45.1 to 73.0)	0.4%<br>(0.3 to 0.6)	0.0%<br>(0.0 to 0.2)	13.9%<br>(10.8 to 16.9)	3.0%<br>(-0.4 to 6.9)	Not reprted	0.1%<br>(-0.1 to 0.3)	4.8%<br>(3.3 to 6.5)	17.0%<br>(14.4 to 20.1)
**Sierra Leone**	64.1%<br>(48.4 to 75.1)	0.7%<br>(0.5 to 1.1)	41.3%<br>(34.0 to 48.2)	10.7%<br>(8.3 to 13.3)	8.3%<br>(-1.1 to 17.9)	Not reprted	0.1%<br>(0.0 to 0.3)	3.5%<br>(2.2 to 4.9)	5.9%<br>(4.6 to 7.4)
**Singapore**	39.9%<br>(26.9 to 51.9)	0.5%<br>(0.5 to 0.6)	0.0%<br>(0.0 to 0.0)	10.9%<br>(8.0 to 13.9)	5.4%<br>(-0.7 to 12.3)	Not reprted	-0.1%<br>(-0.1 to 0.0)	2.9%<br>(2.0 to 3.8)	10.1%<br>(8.5 to 11.7)
**Slovakia**	63.6%<br>(48.3 to 74.9)	0.0%<br>(-0.4 to 0.5)	0.0%<br>(0.0 to 0.2)	7.8%<br>(5.8 to 9.9)	3.4%<br>(-0.4 to 7.7)	Not reprted	9.5%<br>(8.2 to 10.8)	4.6%<br>(3.1 to 6.2)	17.1%<br>(14.3 to 20.2)
**Slovenia**	63.3%<br>(47.7 to 74.6)	0.1%<br>(-0.3 to 0.6)	0.2%<br>(0.0 to 1.5)	7.7%<br>(5.5 to 10.2)	3.0%<br>(-0.4 to 7.0)	Not reprted	8.5%<br>(7.8 to 10.4)	3.7%<br>(2.5 to 5.0)	13.6%<br>(11.5 to 15.9)
**Solomon Islands**	44.5%<br>(30.5 to 57.2)	0.3%<br>(0.1 to 0.6)	44.7%<br>(36.9 to 52.3)	10.8%<br>(8.4 to 13.2)	5.0%<br>(-0.6 to 11.3)	Not reprted	0.0%<br>(0.0 to 0.1)	6.7%<br>(4.4 to 8.9)	18.7%<br>(15.9 to 21.6)
**Somalia**	54.4%<br>(38.8 to 66.4)	1.0%<br>(0.0 to 1.5)	46.0%<br>(38.5 to 53.2)	8.4%<br>(6.1 to 10.8)	13.8%<br>(-1.9 to 29.6)	Not reprted	0.4%<br>(0.3 to 0.6)	2.9%<br>(1.8 to 4.1)	6.7%<br>(4.6 to 9.4)
**South Africa**	63.7%<br>(47.6 to 75.2)	0.1%<br>(-0.1 to 0.4)	3.7%<br>(0.7 to 9.8)	13.4%<br>(10.6 to 16.4)	5.1%<br>(-0.6 to 11.3)	Not reprted	6.6%<br>(5.9 to 7.3)	4.7%<br>(3.2 to 6.2)	9.8%<br>(8.4 to 11.5)
**South Sudan**	55.2%<br>(39.5 to 67.5)	5.5%<br>(4.0 to 7.5)	41.8%<br>(33.5 to 49.1)	8.3%<br>(6.0 to 10.7)	8.2%<br>(-1.1 to 18.7)	Not reprted	0.4%<br>(0.1 to 0.8)	2.7%<br>(1.7 to 3.7)	7.1%<br>(5.2 to 9.3)
**Spain**	56.2%<br>(40.9 to 68.1)	0.1%<br>(-0.2 to 0.5)	0.0%<br>(0.0 to 0.1)	7.3%<br>(5.2 to 9.4)	5.6%<br>(-0.7 to 13.0)	Not reprted	7.8%<br>(7.1 to 8.6)	3.4%<br>(2.2 to 4.5)	14.3%<br>(12.1 to 16.9)
**Sri Lanka**	61.0%<br>(45.2 to 72.2)	0.6%<br>(0.5 to 0.8)	10.3%<br>(0.8 to 33.4)	13.0%<br>(9.8 to 16.3)	3.4%<br>(-0.4 to 7.7)	Not reprted	0.4%<br>(0.3 to 0.6)	3.5%<br>(2.2 to 5.0)	7.5%<br>(6.1 to 8.9)
**Sudan**	60.4%<br>(45.5 to 72.2)	9.4%<br>(5.0 to 15.0)	18.7%<br>(10.6 to 26.9)	10.2%<br>(7.7 to 12.9)	10.3%<br>(-1.5 to 22.9)	Not reprted	1.4%<br>(0.3 to 2.8)	4.1%<br>(2.7 to 5.4)	7.7%<br>(5.7 to 10.3)
**Suriname**	48.3%<br>(33.5 to 60.5)	0.4%<br>(0.2 to 0.8)	1.0%<br>(0.0 to 6.2)	9.6%<br>(7.5 to 11.6)	6.9%<br>(-0.9 to 15.7)	Not reprted	0.0%<br>(-0.1 to 0.1)	3.8%<br>(2.5 to 5.0)	13.9%<br>(11.3 to 16.8)
**Sweden**	55.2%<br>(40.1 to 66.8)	0.0%<br>(-0.1 to 0.0)	0.0%<br>(0.0 to 0.0)	9.0%<br>(6.3 to 11.7)	2.3%<br>(-0.3 to 5.5)	Not reprted	9.3%<br>(8.3 to 10.2)	1.8%<br>(1.2 to 2.4)	13.8%<br>(11.5 to 16.4)
**Switzerland**	50.2%<br>(35.5 to 62.5)	0.0%<br>(-0.1 to 0.1)	0.0%<br>(0.0 to 0.0)	8.9%<br>(6.4 to 11.7)	4.1%<br>(-0.5 to 9.4)	Not reprted	8.8%<br>(7.9 to 10.6)	2.2%<br>(1.5 to 3.0)	13.8%<br>(11.5 to 16.3)

**Syrian Arab Republic**	52.3%<br>(38.7 to 63.2)	1.6%<br>(-0.2 to 4.3)	0.1%<br>(0.0 to 0.2)	9.9%<br>(7.4 to 12.5)	8.2%<br>(-1.1 to 18.1)	Not reprted	5.9%<br>(4.9 to 6.7)	4.9%<br>(3.3 to 6.6)	13.8%<br>(11.5 to 16.1)
**Turkey**	54.3%<br>(40.1 to 65.8)	0.3%<br>(-0.3 to 1.1)	0.1%<br>(0.0 to 0.8)	11.1%<br>(8.0 to 14.3)	4.9%<br>(-0.6 to 10.8)	Not reprted	7.9%<br>(7.0 to 8.8)	5.2%<br>(3.6 to 7.0)	14.7%<br>(12.5 to 17.2)
**Taiwan (Province of China)**	48.7%<br>(34.5 to 61.8)	1.5%<br>(-1.0 to 5.1)	0.1%<br>(0.0 to 0.5)	8.4%<br>(6.5 to 10.3)	5.7%<br>(-0.7 to 13.0)	Not reprted	2.5%<br>(2.0 to 3.1)	4.9%<br>(3.3 to 6.5)	19.7%<br>(17.1 to 22.4)
**Tajikistan**	60.1%<br>(44.4 to 72.0)	0.7%<br>(-0.4 to 2.2)	21.3%<br>(14.4 to 28.4)	12.4%<br>(9.5 to 15.3)	6.1%<br>(-0.8 to 14.0)	Not reprted	8.4%<br>(7.2 to 9.5)	3.6%<br>(2.5 to 4.8)	7.9%<br>(6.0 to 10.3)
**Thailand**	48.1%<br>(33.4 to 60.6)	2.2%<br>(1.9 to 2.6)	1.4%<br>(0.0 to 9.0)	13.3%<br>(10.6 to 16.2)	3.4%<br>(-0.4 to 8.0)	Not reprted	0.7%<br>(0.2 to 1.4)	4.3%<br>(2.9 to 5.7)	16.8%<br>(14.4 to 19.3)
**Timor-Leste**	61.3%<br>(46.6 to 72.9)	0.1%<br>(0.0 to 0.2)	28.9%<br>(11.9 to 43.9)	13.3%<br>(10.3 to 16.5)	8.7%<br>(-1.1 to 18.7)	Not reprted	0.6%<br>(0.4 to 0.9)	5.7%<br>(3.8 to 7.6)	13.7%<br>(11.0 to 16.7)
**Togo**	62.4%<br>(46.7 to 74.0)	2.5%<br>(2.1 to 3.1)	39.7%<br>(32.6 to 46.7)	10.8%<br>(8.3 to 13.3)	7.3%<br>(-0.9 to 16.6)	Not reprted	0.1%<br>(0.0 to 0.2)	2.8%<br>(1.9 to 3.8)	7.9%<br>(6.1 to 10.0)
**Tokelau**	53.7%<br>(37.7 to 65.5)	0.7%<br>(0.2 to 1.2)	0.0%<br>(0.0 to 0.1)	10.6%<br>(8.1 to 12.9)	1.7%<br>(-0.2 to 4.1)	Not reprted	0.0%<br>(0.0 to 0.0)	5.4%<br>(3.6 to 7.3)	13.2%<br>(10.5 to 16.3)
**Tonga**	59.8%<br>(44.3 to 72.3)	0.4%<br>(0.2 to 0.6)	18.0%<br>(8.0 to 28.1)	11.2%<br>(8.7 to 13.8)	2.3%<br>(-0.3 to 5.2)	Not reprted	0.0%<br>(0.0 to 0.0)	6.0%<br>(4.2 to 8.0)	15.7%<br>(12.7 to 18.7)
**Trinidad and Tobago**	61.5%<br>(46.2 to 72.7)	0.3%<br>(0.1 to 0.5)	0.0%<br>(0.0 to 0.0)	9.5%<br>(7.4 to 11.7)	3.2%<br>(-0.4 to 7.3)	Not reprted	0.0%<br>(-0.1 to 0.1)	3.6%<br>(2.4 to 4.8)	12.0%<br>(9.9 to 14.3)
**Tunisia**	51.3%<br>(37.2 to 64.0)	2.3%<br>(-0.1 to 6.3)	0.1%<br>(0.0 to 0.3)	10.1%<br>(7.4 to 12.9)	7.1%<br>(-1.0 to 16.2)	Not reprted	5.2%<br>(4.1 to 6.2)	5.7%<br>(3.8 to 7.4)	14.6%<br>(10.6 to 18.9)
**Turkmenistan**	62.6%<br>(47.9 to 74.7)	2.0%<br>(0.1 to 4.7)	0.0%<br>(0.0 to 0.1)	13.5%<br>(10.9 to 16.2)	3.5%<br>(-0.5 to 7.9)	Not reprted	7.9%<br>(6.5 to 9.4)	4.9%<br>(3.3 to 6.5)	13.8%<br>(11.3 to 16.3)
**Tuvalu**	55.0%<br>(39.7 to 67.5)	0.7%<br>(0.3 to 1.1)	7.3%<br>(4.1 to 11.5)	11.3%<br>(8.8 to 13.8)	2.8%<br>(-0.3 to 6.2)	Not reprted	0.0%<br>(0.0 to 0.0)	6.7%<br>(4.5 to 9.0)	16.4%<br>(13.5 to 20.1)
**Uganda**	57.9%<br>(43.2 to 69.3)	0.1%<br>(-0.1 to 0.2)	42.4%<br>(34.9 to 49.5)	8.7%<br>(6.2 to 11.3)	8.4%<br>(-1.1 to 19.0)	Not reprted	1.4%<br>(1.1 to 1.7)	1.7%<br>(1.1 to 2.3)	5.8%<br>(4.5 to 7.6)
**Ukraine**	64.7%<br>(48.7 to 76.0)	-0.1%<br>(-0.8 to 0.5)	0.9%<br>(0.1 to 3.7)	11.6%<br>(9.0 to 14.4)	2.2%<br>(-0.3 to 5.2)	Not reprted	9.2%<br>(7.7 to 10.7)	4.3%<br>(2.9 to 5.7)	21.3%<br>(16.6 to 25.9)
**United Arab Emirates**	54.6%<br>(39.9 to 66.6)	12.5%<br>(5.7 to 20.8)	0.0%<br>(0.0 to 0.0)	10.7%<br>(7.8 to 14.1)	4.2%<br>(-0.5 to 9.5)	Not reprted	4.4%<br>(1.1 to 9.2)	4.5%<br>(3.0 to 5.9)	7.8%<br>(6.1 to 9.6)
**United Kingdom**	51.7%<br>(38.3 to 62.5)	0.0%<br>(0.0 to 0.0)	0.0%<br>(0.0 to 0.0)	8.4%<br>(6.1 to 10.7)	3.0%<br>(-0.4 to 6.9)	Not reprted	8.3%<br>(6.8 to 11.3)	2.1%<br>(1.4 to 2.8)	13.6%<br>(11.4 to 15.9)
**United Republic of Tanzania**	58.0%<br>(42.2 to 70.2)	0.2%<br>(0.0 to 0.4)	42.9%<br>(34.9 to 50.3)	8.5%<br>(6.1 to 11.0)	5.4%<br>(-0.7 to 12.1)	Not reprted	1.8%<br>(1.4 to 2.1)	2.3%<br>(1.4 to 3.1)	8.8%<br>(7.1 to 10.8)
**United States of America**	47.7%<br>(33.2 to 59.6)	0.5%<br>(-0.6 to 1.8)	0.0%<br>(0.0 to 0.0)	9.8%<br>(7.4 to 12.2)	2.9%<br>(-0.4 to 6.8)	Not reprted	6.5%<br>(5.6 to 7.3)	2.2%<br>(1.5 to 3.0)	15.9%<br>(13.6 to 18.4)
**United States Virgin Islands**	54.5%<br>(39.6 to 67.2)	0.4%<br>(0.2 to 0.5)	0.0%<br>(0.0 to 0.0)	9.2%<br>(7.1 to 11.3)	3.0%<br>(-0.4 to 6.9)	Not reprted	0.0%<br>(-0.1 to 0.1)	2.4%<br>(1.6 to 3.3)	9.4%<br>(7.2 to 11.8)
**Uruguay**	55.3%<br>(40.3 to 66.7)	0.1%<br>(-0.1 to 0.4)	0.1%<br>(0.0 to 1.2)	7.6%<br>(5.7 to 9.8)	5.8%<br>(-0.7 to 12.9)	Not reprted	6.7%<br>(6.2 to 7.5)	3.6%<br>(2.4 to 4.8)	17.0%<br>(14.5 to 19.6)

**Uzbekistan**	59.8% (44.8 to 72.2)	0.8% (-0.6 to 2.3)	4.3% (0.8 to 11.7)	13.6% (10.6 to 16.6)	3.9% (-0.5 to 9.0)	Not reprted	8.3% (6.7 to 9.6)	4.2% (2.9 to 5.6)	10.7% (9.1 to 12.4)
**Vanuatu**	64.0% (48.4 to 75.2)	0.1% (0.1 to 0.2)	41.7% (33.5 to 49.6)	11.3% (8.8 to 13.8)	4.2% (-0.6 to 9.5)	Not reprted	0.3% (0.1 to 0.4)	3.7% (2.4 to 5.0)	10.4% (8.6 to 12.4)
**Venezuela (Bolivarian Republic of)**	56.8% (41.5 to 68.8)	0.5% (0.4 to 0.6)	0.2% (0.0 to 0.9)	12.1% (9.5 to 14.8)	8.4% (-1.1 to 18.9)	Not reprted	0.9% (0.7 to 1.2)	3.6% (2.3 to 5.0)	9.2% (7.6 to 11.1)
**Viet Nam**	63.3% (46.9 to 75.3)	1.9% (0.2 to 4.1)	16.0% (5.6 to 27.9)	11.1% (8.1 to 14.3)	5.8% (-0.8 to 13.0)	Not reprted	1.4% (0.9 to 2.0)	4.9% (3.3 to 6.5)	19.8% (16.9 to 22.8)
**Yemen**	51.8% (36.0 to 64.4)	1.6% (0.7 to 2.8)	16.9% (11.4 to 23.2)	10.1% (7.6 to 12.8)	13.2% (-1.9 to 28.5)	Not reprted	3.0% (2.5 to 3.7)	5.6% (3.7 to 7.5)	14.7% (11.8 to 17.9)
**Zambia**	53.9% (39.3 to 66.9)	0.6% (-0.4 to 2.2)	37.2% (26.8 to 45.9)	8.6% (6.2 to 11.1)	6.5% (-0.9 to 14.7)	Not reprted	2.7% (2.3 to 3.3)	2.6% (1.6 to 3.7)	7.1% (5.7 to 8.8)
**Zimbabwe**	64.7% (49.7 to 75.7)	0.5% (-0.4 to 1.9)	37.8% (30.9 to 45.0)	13.3% (10.6 to 16.3)	8.0% (-1.1 to 17.9)	Not reprted	3.6% (2.7 to 4.6)	3.9% (2.6 to 5.2)	11.2% (8.9 to 14.0)

Appendix Table 14. Age-standardized percent of DALYs (with 95% uncertainty intervals) due to subarachnoid haemorrhage associated with individual risk factors by 7 GBD super-region, 21 GBD region and country for both sexes combined in 2021.

Country/region	Alcohol use	Ambient particulate matter pollution	Diet high in processed meat	Diet high in red meat	Diet high in sodium	Diet high in sugar-sweetened beverages	Diet low in fiber	Diet low in fruits	Diet low in polyunsaturated fatty acids	Diet low in vegetables	Diet low in whole grains	High body-mass index	High fasting plasma glucose	High LDL cholesterol
**GBD super-regions in alphabetical order**														
**Central Europe, Eastern Europe, and Central Asia**	Not reported	10.4% (7.4 to 14.0)	Not reported	-12.0% (-48.4 to 16.6)	8.1% (1.3 to 19.8)	Not reported	3.3% (-1.0 to 7.0)	8.8% (-0.7 to 16.1)	Not reported	0.1% (0.0 to 0.1)	Not reported	9.0% (-0.3 to 20.0)	Not reported	Not reported
**High-income**	Not reported	6.6% (4.4 to 9.1)	Not reported	-12.6% (-52.0 to 16.9)	5.9% (0.4 to 16.4)	Not reported	4.2% (-1.2 to 8.9)	7.6% (-0.6 to 14.0)	Not reported	0.2% (0.0 to 0.3)	Not reported	7.9% (-0.2 to 17.0)	Not reported	Not reported
**Latin America and Caribbean**	Not reported	9.1% (6.3 to 12.3)	Not reported	-11.5% (-47.5 to 15.7)	5.5% (0.4 to 15.6)	Not reported	3.6% (-1.1 to 7.6)	6.5% (-0.5 to 11.8)	Not reported	1.8% (-0.2 to 3.4)	Not reported	9.4% (-0.3 to 20.3)	Not reported	Not reported
**North Africa and Middle East**	Not reported	19.3% (14.6 to 23.6)	Not reported	-3.0% (-12.0 to 4.9)	1.4% (0.0 to 6.8)	Not reported	1.6% (-0.5 to 3.5)	4.9% (-0.4 to 9.1)	Not reported	0.9% (-0.1 to 1.8)	Not reported	9.7% (-0.4 to 20.6)	Not reported	Not reported
**South Asia**	Not reported	16.0% (9.5 to 21.9)	Not reported	-0.7% (-2.7 to 1.1)	6.2% (0.4 to 17.4)	Not reported	5.0% (-1.5 to 10.5)	15.0% (-1.2 to 26.7)	Not reported	2.7% (-0.3 to 5.2)	Not reported	2.2% (0.0 to 5.4)	Not reported	Not reported
**Southeast Asia, East Asia, and Oceania**	Not reported	19.2% (12.6 to 24.0)	Not reported	-8.0% (-32.9 to 11.1)	14.3% (4.8 to 27.3)	Not reported	4.3% (-1.3 to 8.9)	7.0% (-0.5 to 12.8)	Not reported	0.7% (-0.1 to 1.3)	Not reported	3.0% (0.0 to 7.2)	Not reported	Not reported
**Sub-Saharan Africa**	Not reported	8.3% (4.9 to 11.7)	Not reported	-2.7% (-11.1 to 4.5)	3.8% (0.1 to 12.5)	Not reported	1.5% (-0.4 to 3.4)	9.9% (-0.7 to 18.1)	Not reported	6.3% (-0.6 to 12.6)	Not reported	3.9% (0.0 to 9.0)	Not reported	Not reported
**GBD regions in alphabetical order**														
**Andean Latin America**	Not reported	14.6% (9.2 to 20.8)	Not reported	-7.0% (-28.5 to 10.3)	4.8% (0.1 to 14.4)	Not reported	4.5% (-1.5 to 9.4)	6.4% (-0.5 to 11.5)	Not reported	2.8% (-0.3 to 5.4)	Not reported	9.1% (-0.3 to 19.8)	Not reported	Not reported
**Australasia**	Not reported	4.8% (2.8 to 7.2)	Not reported	-17.0% (-70.7 to 21.7)	3.1% (0.0 to 11.3)	Not reported	4.6% (-1.4 to 9.8)	7.8% (-0.6 to 14.5)	Not reported	0.0% (0.0 to 0.0)	Not reported	10.4% (-0.4 to 22.0)	Not reported	Not reported
**Caribbean**	Not reported	7.6% (3.9 to 12.9)	Not reported	-3.5% (-14.5 to 5.8)	2.7% (0.0 to 9.9)	Not reported	3.6% (-1.1 to 7.6)	6.3% (-0.5 to 11.6)	Not reported	5.4% (-0.6 to 10.4)	Not reported	6.3% (-0.1 to 14.1)	Not reported	Not reported
**Central Asia**	Not reported	16.6% (11.2 to 21.6)	Not reported	-11.5% (-46.5 to 15.5)	6.3% (0.4 to 17.4)	Not reported	3.6% (-1.1 to 7.5)	8.2% (-0.6 to 15.2)	Not reported	0.1% (0.0 to 0.3)	Not reported	7.4% (-0.2 to 16.9)	Not reported	Not reported
**Central Europe**	Not reported	12.2% (9.1 to 15.3)	Not reported	-13.5% (-55.8 to 18.1)	12.6% (3.2 to 25.3)	Not reported	2.9% (-0.8 to 5.9)	8.0% (-0.6 to 14.5)	Not reported	0.0% (0.0 to 0.0)	Not reported	9.0% (-0.3 to 19.6)	Not reported	Not reported
**Central Latin America**	Not reported	9.3% (6.4 to 12.3)	Not reported	-8.8% (-36.3 to 12.7)	6.7% (0.7 to 17.7)	Not reported	2.7% (-0.8 to 5.7)	6.8% (-0.5 to 12.4)	Not reported	1.1% (-0.1 to 2.1)	Not reported	10.2% (-0.4 to 21.9)	Not reported	Not reported
**Central Sub-Saharan Africa**	Not reported	6.7% (3.9 to 10.0)	Not reported	-1.9% (-8.3 to 3.3)	1.8% (0.0 to 8.5)	Not reported	3.2% (-1.0 to 7.1)	9.1% (-0.7 to 16.8)	Not reported	9.8% (-1.0 to 19.5)	Not reported	3.5% (0.0 to 8.1)	Not reported	Not reported
**East Asia**	Not reported	22.8% (14.9 to 28.2)	Not reported	-10.9% (-44.0 to 14.8)	17.2% (6.7 to 31.4)	Not reported	2.3% (-0.6 to 5.0)	6.5% (-0.5 to 12.0)	Not reported	0.0% (0.0 to 0.0)	Not reported	3.2% (0.0 to 7.8)	Not reported	Not reported

**Eastern Europe**	Not reported	8.1% (5.1 to 12.1)	Not reported	-11.5% (-45.3 to 16.1)	6.7% (0.6 to 17.9)	Not reported	3.4% (-1.0 to 7.3)	9.2% (-0.7 to 16.9)	Not reported	0.1% (0.0 to 0.1)	Not reported	9.4% (-0.3 to 20.8)	Not reported	Not reported
**Eastern Sub-Saharan Africa**	Not reported	4.5% (2.8 to 6.7)	Not reported	-2.1% (-8.6 to 3.6)	5.4% (0.3 to 15.2)	Not reported	1.7% (-0.5 to 3.7)	10.3% (-0.8 to 18.9)	Not reported	8.3% (-0.9 to 15.9)	Not reported	2.4% (-0.1 to 6.0)	Not reported	Not reported
**High-income Asia Pacific**	Not reported	10.6% (6.4 to 15.8)	Not reported	-7.7% (-31.2 to 11.3)	9.3% (1.1 to 22.1)	Not reported	5.1% (-1.5 to 10.8)	9.0% (-0.7 to 16.5)	Not reported	0.0% (0.0 to 0.0)	Not reported	2.1% (-0.1 to 5.6)	Not reported	Not reported
**High-income North America**	Not reported	3.0% (1.5 to 4.9)	Not reported	-14.3% (-59.0 to 18.9)	4.8% (0.2 to 14.6)	Not reported	3.4% (-1.0 to 7.4)	7.5% (-0.6 to 13.5)	Not reported	0.4% (0.0 to 0.7)	Not reported	12.0% (-0.5 to 24.9)	Not reported	Not reported
**North Africa and Middle East**	Not reported	19.3% (14.6 to 23.6)	Not reported	-3.0% (-12.0 to 4.9)	1.4% (0.0 to 6.8)	Not reported	1.6% (-0.5 to 3.5)	4.9% (-0.4 to 9.1)	Not reported	0.9% (-0.1 to 1.8)	Not reported	9.7% (-0.4 to 20.6)	Not reported	Not reported
**Oceania**	Not reported	4.4% (1.6 to 9.8)	Not reported	-4.8% (-19.6 to 7.6)	5.2% (0.4 to 13.9)	Not reported	0.3% (-0.1 to 0.7)	8.4% (-0.7 to 15.6)	Not reported	4.1% (-0.5 to 8.0)	Not reported	5.7% (0.0 to 12.9)	Not reported	Not reported
**South Asia**	Not reported	16.0% (9.5 to 21.9)	Not reported	-0.7% (-2.7 to 1.1)	6.2% (0.4 to 17.4)	Not reported	5.0% (-1.5 to 10.5)	15.0% (-1.2 to 26.7)	Not reported	2.7% (-0.3 to 5.2)	Not reported	2.2% (0.0 to 5.4)	Not reported	Not reported
**Southeast Asia**	Not reported	13.3% (8.3 to 17.7)	Not reported	-3.4% (-13.9 to 4.9)	10.0% (1.7 to 22.4)	Not reported	7.9% (-2.5 to 16.2)	7.9% (-0.6 to 14.4)	Not reported	1.7% (-0.2 to 3.3)	Not reported	2.7% (0.0 to 6.4)	Not reported	Not reported
**Southern Latin America**	Not reported	10.8% (6.2 to 16.3)	Not reported	-16.8% (-70.1 to 21.6)	5.8% (0.2 to 16.8)	Not reported	5.4% (-1.6 to 11.4)	6.5% (-0.5 to 11.9)	Not reported	0.1% (0.0 to 0.2)	Not reported	10.7% (-0.4 to 23.2)	Not reported	Not reported
**Southern Sub-Saharan Africa**	Not reported	10.9% (7.2 to 14.3)	Not reported	-6.9% (-28.6 to 10.2)	2.9% (0.0 to 11.0)	Not reported	1.8% (-0.5 to 3.8)	12.8% (-1.0 to 23.2)	Not reported	6.3% (-0.6 to 12.0)	Not reported	8.4% (-0.3 to 17.9)	Not reported	Not reported
**Tropical Latin America**	Not reported	7.7% (4.4 to 11.9)	Not reported	-16.7% (-69.7 to 21.5)	5.4% (0.2 to 15.6)	Not reported	4.1% (-1.2 to 8.7)	6.3% (-0.5 to 11.5)	Not reported	1.3% (-0.2 to 2.7)	Not reported	9.5% (-0.3 to 20.8)	Not reported	Not reported
**Western Europe**	Not reported	6.2% (4.3 to 8.5)	Not reported	-13.9% (-57.7 to 18.4)	4.3% (0.2 to 13.4)	Not reported	4.0% (-1.2 to 8.4)	7.0% (-0.5 to 12.9)	Not reported	0.1% (0.0 to 0.1)	Not reported	7.4% (-0.2 to 16.9)	Not reported	Not reported
**Western Sub-Saharan Africa**	Not reported	12.0% (6.7 to 18.0)	Not reported	-2.5% (-10.2 to 4.1)	3.3% (0.0 to 12.0)	Not reported	0.7% (-0.2 to 1.6)	9.1% (-0.7 to 17.0)	Not reported	3.1% (-0.3 to 6.2)	Not reported	4.4% (0.0 to 10.1)	Not reported	Not reported
**Countries in alphabetical order**														
**Afghanistan**	Not reported	6.6% (3.9 to 9.8)	Not reported	-3.6% (-14.6 to 5.7)	1.5% (0.0 to 7.6)	Not reported	6.2% (-1.9 to 13.0)	8.9% (-0.7 to 16.2)	Not reported	10.7% (-1.1 to 19.7)	Not reported	6.0% (-0.1 to 13.2)	Not reported	Not reported
**Albania**	Not reported	10.3% (6.2 to 13.7)	Not reported	-11.8% (-48.3 to 16.3)	13.2% (3.5 to 26.7)	Not reported	2.1% (-0.6 to 4.8)	4.2% (-0.3 to 8.1)	Not reported	0.0% (0.0 to 0.0)	Not reported	6.8% (-0.2 to 16.0)	Not reported	Not reported
**Algeria**	Not reported	16.4% (11.6 to 22.1)	Not reported	-2.8% (-11.2 to 4.7)	1.4% (0.0 to 7.2)	Not reported	1.2% (-0.3 to 2.7)	5.8% (-0.4 to 10.8)	Not reported	0.0% (0.0 to 0.0)	Not reported	8.6% (-0.3 to 18.6)	Not reported	Not reported
**American Samoa**	Not reported	3.0% (0.3 to 6.3)	Not reported	-8.7% (-36.2 to 11.5)	2.5% (0.0 to 9.8)	Not reported	0.2% (0.0 to 0.6)	8.2% (-0.7 to 14.7)	Not reported	4.4% (-0.5 to 8.6)	Not reported	17.8% (-1.0 to 34.2)	Not reported	Not reported
**Andorra**	Not reported	4.8% (2.6 to 7.3)	Not reported	-15.1% (-61.9 to 19.2)	3.0% (0.0 to 11.5)	Not reported	2.8% (-0.9 to 6.3)	6.3% (-0.5 to 11.7)	Not reported	0.0% (0.0 to 0.0)	Not reported	6.7% (-0.1 to 15.5)	Not reported	Not reported
**Angola**	Not reported	13.6% (6.4 to 20.6)	Not reported	-3.5% (-14.8 to 6.0)	2.6% (0.0 to 10.7)	Not reported	1.2% (-0.3 to 2.7)	8.0% (-0.6 to 14.7)	Not reported	1.7% (-0.2 to 3.4)	Not reported	3.0% (0.0 to 7.2)	Not reported	Not reported
**Antigua and Barbuda**	Not reported	12.8% (4.7 to 23.2)	Not reported	-4.3% (-17.0 to 7.1)	3.4% (0.0 to 12.1)	Not reported	8.4% (-2.6 to 17.1)	6.3% (-0.5 to 11.5)	Not reported	1.2% (-0.1 to 2.4)	Not reported	9.1% (-0.3 to 19.8)	Not reported	Not reported

**Argentina**	Not reported	9.4%<br>(4.8 to 15.5)	Not reported	-17.7%<br>(-74.2 to 22.5)	5.6%<br>(0.1 to 16.5)	Not reported	6.0%<br>(-1.9 to 12.7)	5.8%<br>(-0.4 to 10.8)	Not reported	0.0%<br>(0.0 to 0.1)	Not reported	10.5%<br>(-0.4 to 22.7)	Not reported	Not reported
**Armenia**	Not reported	22.2%<br>(14.5 to 30.5)	Not reported	-10.1%<br>(-40.7 to 14.6)	6.5%<br>(0.4 to 18.4)	Not reported	3.2%<br>(-0.9 to 6.9)	5.8%<br>(-0.4 to 10.7)	Not reported	0.0%<br>(0.0 to 0.0)	Not reported	7.9%<br>(-0.2 to 17.7)	Not reported	Not reported
**Australia**	Not reported	5.2%<br>(3.1 to 7.6)	Not reported	-17.0%<br>(-70.7 to 21.6)	2.9%<br>(0.0 to 10.8)	Not reported	4.7%<br>(-1.5 to 10.0)	8.0%<br>(-0.6 to 14.7)	Not reported	0.0%<br>(0.0 to 0.0)	Not reported	10.4%<br>(-0.4 to 22.0)	Not reported	Not reported
**Austria**	Not reported	7.2%<br>(5.1 to 9.7)	Not reported	-14.0%<br>(-57.7 to 18.5)	5.7%<br>(0.2 to 17.0)	Not reported	2.3%<br>(-0.6 to 5.0)	5.5%<br>(-0.4 to 10.4)	Not reported	0.0%<br>(0.0 to 0.0)	Not reported	5.9%<br>(-0.1 to 13.8)	Not reported	Not reported
**Azerbaijan**	Not reported	15.3%<br>(8.0 to 24.4)	Not reported	-7.1%<br>(-28.9 to 11.2)	5.8%<br>(0.2 to 16.9)	Not reported	2.0%<br>(-0.5 to 4.3)	6.6%<br>(-0.5 to 12.2)	Not reported	0.0%<br>(0.0 to 0.0)	Not reported	8.5%<br>(-0.2 to 19.1)	Not reported	Not reported
**Bahamas**	Not reported	12.5%<br>(4.3 to 24.5)	Not reported	-11.9%<br>(-48.2 to 16.2)	3.2%<br>(0.0 to 11.7)	Not reported	7.9%<br>(-2.7 to 16.2)	8.0%<br>(-0.6 to 14.4)	Not reported	0.0%<br>(0.0 to 0.1)	Not reported	12.2%<br>(-0.6 to 26.1)	Not reported	Not reported
**Bahrain**	Not reported	33.2%<br>(25.8 to 40.6)	Not reported	-4.6%<br>(-19.7 to 7.2)	1.6%<br>(0.0 to 7.7)	Not reported	0.6%<br>(-0.2 to 1.3)	3.9%<br>(-0.2 to 7.4)	Not reported	0.0%<br>(0.0 to 0.0)	Not reported	11.3%<br>(-0.6 to 23.5)	Not reported	Not reported
**Bangladesh**	Not reported	7.9%<br>(4.1 to 12.8)	Not reported	-0.3%<br>(-1.0 to 0.5)	5.2%<br>(0.1 to 15.7)	Not reported	11.2%<br>(-3.9 to 22.6)	12.8%<br>(-1.0 to 23.0)	Not reported	7.9%<br>(-0.8 to 15.0)	Not reported	1.7%<br>(-0.1 to 4.7)	Not reported	Not reported
**Barbados**	Not reported	16.1%<br>(6.5 to 28.5)	Not reported	-7.2%<br>(-29.3 to 10.9)	2.5%<br>(0.0 to 10.5)	Not reported	5.6%<br>(-1.7 to 12.3)	9.3%<br>(-0.7 to 16.8)	Not reported	0.5%<br>(-0.1 to 1.1)	Not reported	11.3%<br>(-0.5 to 24.2)	Not reported	Not reported
**Belarus**	Not reported	10.9%<br>(7.9 to 14.7)	Not reported	-17.1%<br>(-69.7 to 22.3)	3.7%<br>(0.0 to 12.9)	Not reported	0.8%<br>(-0.2 to 1.8)	9.0%<br>(-0.7 to 16.2)	Not reported	0.0%<br>(0.0 to 0.0)	Not reported	9.7%<br>(-0.3 to 22.0)	Not reported	Not reported
**Belgium**	Not reported	6.9%<br>(4.8 to 9.3)	Not reported	-15.3%<br>(-63.6 to 20.7)	5.1%<br>(0.1 to 15.5)	Not reported	3.8%<br>(-1.2 to 8.1)	7.3%<br>(-0.6 to 13.5)	Not reported	0.0%<br>(0.0 to 0.0)	Not reported	6.8%<br>(-0.1 to 15.9)	Not reported	Not reported
**Belize**	Not reported	16.4%<br>(6.4 to 30.2)	Not reported	-2.8%<br>(-11.0 to 5.0)	3.2%<br>(0.0 to 11.5)	Not reported	2.5%<br>(-0.7 to 5.5)	3.4%<br>(-0.2 to 6.3)	Not reported	3.5%<br>(-0.4 to 6.9)	Not reported	12.6%<br>(-0.6 to 26.4)	Not reported	Not reported
**Benin**	Not reported	8.2%<br>(4.4 to 13.4)	Not reported	-0.8%<br>(-3.5 to 1.5)	4.1%<br>(0.0 to 13.8)	Not reported	0.1%<br>(0.0 to 0.3)	9.7%<br>(-0.7 to 18.1)	Not reported	0.8%<br>(-0.1 to 1.7)	Not reported	5.3%<br>(0.0 to 11.9)	Not reported	Not reported
**Bermuda**	Not reported	3.1%<br>(0.6 to 5.8)	Not reported	-13.7%<br>(-54.7 to 18.2)	3.7%<br>(0.0 to 12.7)	Not reported	6.3%<br>(-1.9 to 13.2)	7.9%<br>(-0.6 to 14.3)	Not reported	0.0%<br>(0.0 to 0.0)	Not reported	11.9%<br>(-0.5 to 25.2)	Not reported	Not reported
**Bhutan**	Not reported	18.5%<br>(10.8 to 24.9)	Not reported	-0.9%<br>(-3.9 to 1.7)	5.4%<br>(0.1 to 16.6)	Not reported	5.5%<br>(-1.7 to 12.1)	9.2%<br>(-0.7 to 16.9)	Not reported	1.7%<br>(-0.2 to 3.5)	Not reported	5.1%<br>(0.0 to 12.4)	Not reported	Not reported
**Bolivia (Plurinational State of)**	Not reported	13.5%<br>(7.0 to 21.6)	Not reported	-10.2%<br>(-40.5 to 14.5)	4.7%<br>(0.1 to 13.9)	Not reported	4.3%<br>(-1.2 to 9.2)	7.2%<br>(-0.5 to 13.2)	Not reported	4.6%<br>(-0.5 to 9.2)	Not reported	7.7%<br>(-0.2 to 17.1)	Not reported	Not reported
**Bosnia and Herzegovina**	Not reported	17.2%<br>(9.8 to 22.2)	Not reported	-4.4%<br>(-17.9 to 7.7)	13.2%<br>(3.3 to 26.0)	Not reported	0.2%<br>(-0.1 to 0.5)	7.3%<br>(-0.6 to 13.3)	Not reported	0.0%<br>(0.0 to 0.0)	Not reported	8.2%<br>(-0.2 to 18.5)	Not reported	Not reported
**Botswana**	Not reported	12.9%<br>(7.3 to 18.6)	Not reported	-7.1%<br>(-29.0 to 11.2)	2.8%<br>(0.0 to 10.9)	Not reported	2.8%<br>(-0.8 to 6.1)	12.6%<br>(-1.0 to 22.9)	Not reported	5.4%<br>(-0.5 to 10.4)	Not reported	6.6%<br>(-0.2 to 14.2)	Not reported	Not reported
**Brazil**	Not reported	7.7%<br>(4.4 to 11.9)	Not reported	-16.7%<br>(-69.8 to 21.5)	5.4%<br>(0.2 to 15.6)	Not reported	4.2%<br>(-1.2 to 8.9)	6.3%<br>(-0.5 to 11.5)	Not reported	1.3%<br>(-0.2 to 2.6)	Not reported	9.5%<br>(-0.3 to 20.9)	Not reported	Not reported
**Brunei Darussalam**	Not reported	3.9%<br>(0.8 to 7.7)	Not reported	-5.3%<br>(-21.3 to 8.2)	10.9%<br>(1.6 to 24.3)	Not reported	7.1%<br>(-2.3 to 14.7)	9.9%<br>(-0.8 to 18.2)	Not reported	1.0%<br>(-0.1 to 2.1)	Not reported	7.6%<br>(-0.2 to 16.0)	Not reported	Not reported
**Bulgaria**	Not reported	11.9%<br>(8.9 to 15.3)	Not reported	-15.5%<br>(-63.7 to 19.8)	13.4%<br>(3.5 to 26.5)	Not reported	5.4%<br>(-1.7 to 11.5)	8.6%<br>(-0.7 to 15.6)	Not reported	0.0%<br>(0.0 to 0.0)	Not reported	8.7%<br>(-0.3 to 19.3)	Not reported	Not reported

**Burkina Faso**	Not reported	8.0% (4.6 to 13.1)	Not reported	-3.9% (-15.8 to 6.8)	3.2% (0.0 to 12.2)	Not reported	0.3% (-0.1 to 0.7)	16.4% (-1.3 to 29.5)	Not reported	9.6% (-0.9 to 18.6)	Not reported	0.9% (-0.4 to 3.5)	Not reported	Not reported
**Burundi**	Not reported	3.5% (1.8 to 5.9)	Not reported	-0.2% (-0.7 to 0.3)	5.4% (0.2 to 15.1)	Not reported	0.5% (-0.1 to 1.3)	5.8% (-0.4 to 10.6)	Not reported	9.2% (-0.9 to 17.6)	Not reported	0.5% (-0.4 to 2.1)	Not reported	Not reported
**CV¥te d'Ivoire**	Not reported	11.5% (5.6 to 18.6)	Not reported	-3.4% (-14.1 to 5.9)	3.5% (0.0 to 12.8)	Not reported	0.0% (0.0 to 0.1)	7.6% (-0.6 to 14.2)	Not reported	3.7% (-0.4 to 7.3)	Not reported	5.3% (0.0 to 11.6)	Not reported	Not reported
**Cabo Verde**	Not reported	21.3% (12.3 to 29.7)	Not reported	-3.1% (-12.8 to 5.1)	3.3% (0.0 to 12.4)	Not reported	2.1% (-0.6 to 4.7)	9.1% (-0.7 to 16.9)	Not reported	0.0% (0.0 to 0.1)	Not reported	5.9% (0.0 to 13.5)	Not reported	Not reported
**Cambodia**	Not reported	6.6% (3.2 to 11.8)	Not reported	-2.8% (-11.9 to 5.1)	9.6% (1.3 to 22.4)	Not reported	10.4% (-3.3 to 21.3)	11.2% (-0.9 to 20.7)	Not reported	8.4% (-0.9 to 16.2)	Not reported	1.0% (-0.2 to 3.1)	Not reported	Not reported
**Cameroon**	Not reported	10.0% (5.0 to 17.0)	Not reported	-2.5% (-10.4 to 4.4)	3.2% (0.0 to 11.9)	Not reported	0.2% (-0.1 to 0.5)	6.8% (-0.5 to 12.4)	Not reported	0.1% (0.0 to 0.2)	Not reported	8.3% (-0.2 to 18.6)	Not reported	Not reported
**Canada**	Not reported	2.2% (0.8 to 4.0)	Not reported	-14.0% (-57.2 to 18.5)	4.9% (0.2 to 14.8)	Not reported	2.6% (-0.8 to 5.7)	7.3% (-0.6 to 13.3)	Not reported	0.0% (0.0 to 0.0)	Not reported	9.2% (-0.3 to 20.4)	Not reported	Not reported
**Central African Republic**	Not reported	4.4% (2.5 to 6.9)	Not reported	-10.1% (-40.0 to 14.4)	2.5% (0.0 to 10.9)	Not reported	2.3% (-0.7 to 5.2)	9.3% (-0.7 to 17.3)	Not reported	12.4% (-1.2 to 23.5)	Not reported	2.6% (0.0 to 6.1)	Not reported	Not reported
**Chad**	Not reported	8.2% (4.5 to 13.2)	Not reported	-3.8% (-15.4 to 6.3)	3.1% (0.0 to 11.9)	Not reported	1.0% (-0.3 to 2.3)	14.4% (-1.1 to 25.8)	Not reported	12.0% (-1.2 to 22.6)	Not reported	2.1% (-0.1 to 5.2)	Not reported	Not reported
**Chile**	Not reported	15.6% (9.9 to 22.3)	Not reported	-14.6% (-60.2 to 19.4)	6.2% (0.2 to 17.4)	Not reported	4.4% (-1.3 to 9.2)	7.8% (-0.6 to 14.3)	Not reported	0.0% (0.0 to 0.0)	Not reported	11.6% (-0.5 to 24.8)	Not reported	Not reported
**China**	Not reported	23.5% (15.3 to 29.0)	Not reported	-11.1% (-44.6 to 15.0)	17.5% (6.8 to 31.6)	Not reported	2.2% (-0.6 to 4.7)	6.4% (-0.4 to 12.0)	Not reported	0.0% (0.0 to 0.0)	Not reported	3.3% (0.0 to 8.0)	Not reported	Not reported
**Colombia**	Not reported	9.5% (6.1 to 13.4)	Not reported	-8.8% (-35.5 to 12.5)	10.1% (1.9 to 22.5)	Not reported	4.3% (-1.3 to 9.2)	6.4% (-0.5 to 11.7)	Not reported	0.9% (-0.1 to 1.8)	Not reported	8.3% (-0.3 to 18.4)	Not reported	Not reported
**Comoros**	Not reported	2.5% (1.3 to 4.3)	Not reported	-0.9% (-3.8 to 1.6)	5.5% (0.2 to 15.7)	Not reported	2.8% (-0.8 to 6.2)	7.1% (-0.5 to 13.1)	Not reported	12.1% (-1.2 to 22.7)	Not reported	4.2% (0.0 to 9.4)	Not reported	Not reported
**Congo**	Not reported	13.0% (5.8 to 21.3)	Not reported	-3.4% (-13.8 to 5.9)	2.6% (0.0 to 10.7)	Not reported	5.6% (-1.6 to 11.8)	8.6% (-0.7 to 16.0)	Not reported	11.4% (-1.2 to 21.6)	Not reported	5.7% (0.0 to 12.6)	Not reported	Not reported
**Cook Islands**	Not reported	2.3% (0.0 to 5.7)	Not reported	-9.6% (-39.4 to 12.2)	5.1% (0.4 to 13.8)	Not reported	0.0% (0.0 to 0.1)	7.1% (-0.5 to 13.3)	Not reported	2.4% (-0.3 to 4.9)	Not reported	16.8% (-1.0 to 32.4)	Not reported	Not reported
**Costa Rica**	Not reported	8.7% (5.7 to 12.1)	Not reported	-7.6% (-31.3 to 11.0)	7.6% (0.5 to 19.6)	Not reported	6.6% (-2.0 to 13.7)	6.5% (-0.5 to 11.9)	Not reported	2.8% (-0.3 to 5.7)	Not reported	9.8% (-0.3 to 21.4)	Not reported	Not reported
**Croatia**	Not reported	11.0% (8.1 to 14.2)	Not reported	-9.1% (-35.3 to 13.5)	14.1% (3.8 to 28.2)	Not reported	5.6% (-1.7 to 11.7)	6.7% (-0.5 to 12.5)	Not reported	0.0% (0.0 to 0.0)	Not reported	9.3% (-0.3 to 20.7)	Not reported	Not reported
**Cuba**	Not reported	13.7% (6.3 to 22.9)	Not reported	-8.2% (-33.7 to 11.9)	3.1% (0.0 to 11.6)	Not reported	0.4% (-0.1 to 0.9)	6.1% (-0.4 to 11.3)	Not reported	0.0% (0.0 to 0.0)	Not reported	9.1% (-0.3 to 20.1)	Not reported	Not reported
**Cyprus**	Not reported	10.2% (7.3 to 13.4)	Not reported	-10.4% (-42.9 to 15.0)	4.4% (0.1 to 14.7)	Not reported	6.3% (-2.0 to 13.0)	6.4% (-0.5 to 11.8)	Not reported	0.1% (0.0 to 0.1)	Not reported	6.7% (-0.2 to 15.6)	Not reported	Not reported
**Czechia**	Not reported	9.4% (6.9 to 12.3)	Not reported	-13.7% (-55.4 to 17.9)	13.0% (3.3 to 26.5)	Not reported	5.1% (-1.5 to 10.5)	8.5% (-0.6 to 15.3)	Not reported	0.1% (0.0 to 0.1)	Not reported	9.0% (-0.3 to 20.1)	Not reported	Not reported
**Democratic People's Republic of Korea**	Not reported	5.6% (3.5 to 8.1)	Not reported	-1.8% (-7.4 to 3.0)	13.6% (3.6 to 27.2)	Not reported	4.8% (-1.4 to 10.1)	7.9% (-0.6 to 14.7)	Not reported	0.0% (0.0 to 0.0)	Not reported	0.0% (-0.8 to 1.7)	Not reported	Not reported

**Democratic Republic of the Congo**	Not reported	3.9%<br>(2.4 to 6.0)	Not reported	-0.2%<br>(-1.0 to 0.4)	1.4%<br>(0.0 to 7.6)	Not reported	3.8%<br>(-1.1 to 8.2)	9.5%<br>(-0.7 to 17.6)	Not reported	12.2%<br>(-1.2 to 22.9)	Not reported	3.5%<br>(0.0 to 8.2)	Not reported	Not reported
**Denmark**	Not reported	5.4%<br>(3.2 to 8.0)	Not reported	-14.0%<br>(-57.1 to 18.7)	4.5%<br>(0.1 to 14.4)	Not reported	3.1%<br>(-0.8 to 6.8)	5.9%<br>(-0.4 to 11.0)	Not reported	0.0%<br>(0.0 to 0.0)	Not reported	6.2%<br>(-0.1 to 14.1)	Not reported	Not reported
**Djibouti**	Not reported	19.0%<br>(9.6 to 32.2)	Not reported	-5.3%<br>(-21.8 to 8.1)	5.3%<br>(0.2 to 15.6)	Not reported	6.1%<br>(-1.9 to 12.7)	14.1%<br>(-1.1 to 25.5)	Not reported	2.0%<br>(-0.2 to 4.0)	Not reported	0.7%<br>(-0.4 to 2.7)	Not reported	Not reported
**Dominica**	Not reported	13.2%<br>(5.2 to 24.0)	Not reported	-6.1%<br>(-24.7 to 9.2)	3.5%<br>(0.0 to 12.6)	Not reported	0.6%<br>(-0.2 to 1.3)	1.7%<br>(-0.1 to 3.2)	Not reported	0.1%<br>(0.0 to 0.2)	Not reported	11.5%<br>(-0.5 to 24.1)	Not reported	Not reported
**Dominican Republic**	Not reported	12.3%<br>(4.4 to 22.5)	Not reported	-5.2%<br>(-22.1 to 8.3)	2.8%<br>(0.0 to 10.6)	Not reported	5.0%<br>(-1.5 to 10.3)	3.3%<br>(-0.2 to 6.1)	Not reported	0.5%<br>(0.0 to 1.0)	Not reported	8.6%<br>(-0.3 to 19.1)	Not reported	Not reported
**Ecuador**	Not reported	11.6%<br>(7.2 to 17.6)	Not reported	-10.0%<br>(-41.3 to 14.1)	5.1%<br>(0.1 to 15.3)	Not reported	8.0%<br>(-2.6 to 16.3)	4.4%<br>(-0.3 to 8.3)	Not reported	3.4%<br>(-0.3 to 6.6)	Not reported	10.7%<br>(-0.4 to 23.0)	Not reported	Not reported
**Egypt**	Not reported	33.3%<br>(25.0 to 41.0)	Not reported	-3.5%<br>(-14.3 to 5.6)	1.5%<br>(0.0 to 7.2)	Not reported	0.1%<br>(0.0 to 0.3)	4.0%<br>(-0.3 to 7.6)	Not reported	0.0%<br>(0.0 to 0.0)	Not reported	12.0%<br>(-0.6 to 24.8)	Not reported	Not reported
**El Salvador**	Not reported	11.3%<br>(5.9 to 17.6)	Not reported	-1.8%<br>(-7.0 to 3.2)	6.3%<br>(0.4 to 17.0)	Not reported	1.0%<br>(-0.3 to 2.2)	8.3%<br>(-0.6 to 15.1)	Not reported	0.8%<br>(-0.1 to 1.8)	Not reported	11.5%<br>(-0.5 to 24.4)	Not reported	Not reported
**Equatorial Guinea**	Not reported	20.6%<br>(11.4 to 31.1)	Not reported	-6.2%<br>(-25.6 to 9.5)	2.6%<br>(0.0 to 10.5)	Not reported	0.6%<br>(-0.2 to 1.4)	6.5%<br>(-0.4 to 12.1)	Not reported	6.9%<br>(-0.8 to 13.3)	Not reported	7.0%<br>(-0.1 to 15.3)	Not reported	Not reported
**Eritrea**	Not reported	7.0%<br>(3.9 to 11.9)	Not reported	-1.8%<br>(-7.7 to 3.3)	5.2%<br>(0.2 to 14.6)	Not reported	1.5%<br>(-0.5 to 3.4)	9.8%<br>(-0.8 to 18.0)	Not reported	10.2%<br>(-1.1 to 19.4)	Not reported	0.9%<br>(-0.1 to 2.9)	Not reported	Not reported
**Estonia**	Not reported	2.9%<br>(1.0 to 5.2)	Not reported	-11.0%<br>(-45.1 to 16.2)	2.3%<br>(0.0 to 9.7)	Not reported	1.3%<br>(-0.3 to 2.9)	8.3%<br>(-0.6 to 15.3)	Not reported	0.0%<br>(0.0 to 0.0)	Not reported	9.5%<br>(-0.3 to 21.0)	Not reported	Not reported
**Eswatini**	Not reported	12.6%<br>(5.5 to 19.9)	Not reported	-7.7%<br>(-30.1 to 11.3)	2.8%<br>(0.0 to 11.2)	Not reported	2.7%<br>(-0.7 to 5.8)	9.0%<br>(-0.7 to 16.7)	Not reported	9.8%<br>(-1.1 to 18.4)	Not reported	10.2%<br>(-0.4 to 21.4)	Not reported	Not reported
**Ethiopia**	Not reported	5.2%<br>(3.2 to 7.6)	Not reported	-1.6%<br>(-6.5 to 2.7)	5.3%<br>(0.2 to 15.0)	Not reported	0.5%<br>(-0.1 to 1.2)	15.5%<br>(-1.2 to 27.9)	Not reported	8.9%<br>(-1.0 to 17.0)	Not reported	1.0%<br>(-0.2 to 3.2)	Not reported	Not reported
**Fiji**	Not reported	7.4%<br>(2.5 to 16.4)	Not reported	-9.4%<br>(-39.3 to 13.3)	5.4%<br>(0.4 to 14.3)	Not reported	1.1%<br>(-0.3 to 2.4)	10.7%<br>(-0.9 to 19.3)	Not reported	2.6%<br>(-0.3 to 5.1)	Not reported	12.0%<br>(-0.5 to 24.2)	Not reported	Not reported
**Finland**	Not reported	1.4%<br>(0.2 to 3.2)	Not reported	-14.0%<br>(-58.1 to 18.9)	5.0%<br>(0.1 to 15.6)	Not reported	3.0%<br>(-0.9 to 6.7)	8.2%<br>(-0.6 to 15.1)	Not reported	0.0%<br>(0.0 to 0.1)	Not reported	7.6%<br>(-0.2 to 17.1)	Not reported	Not reported
**France**	Not reported	5.6%<br>(3.7 to 7.9)	Not reported	-16.8%<br>(-69.3 to 21.6)	3.5%<br>(0.0 to 12.2)	Not reported	5.2%<br>(-1.6 to 11.2)	8.4%<br>(-0.6 to 15.4)	Not reported	0.1%<br>(0.0 to 0.2)	Not reported	6.9%<br>(-0.1 to 15.9)	Not reported	Not reported
**Gabon**	Not reported	18.7%<br>(10.8 to 28.0)	Not reported	-12.2%<br>(-50.4 to 16.3)	2.6%<br>(0.0 to 10.9)	Not reported	1.6%<br>(-0.4 to 3.3)	4.9%<br>(-0.4 to 9.3)	Not reported	6.1%<br>(-0.7 to 12.1)	Not reported	8.5%<br>(-0.2 to 18.4)	Not reported	Not reported
**Gambia**	Not reported	8.8%<br>(4.8 to 14.1)	Not reported	-0.8%<br>(-3.4 to 1.6)	3.3%<br>(0.0 to 12.1)	Not reported	2.4%<br>(-0.7 to 5.2)	17.0%<br>(-1.4 to 30.6)	Not reported	9.0%<br>(-0.9 to 17.3)	Not reported	5.1%<br>(0.0 to 12.4)	Not reported	Not reported
**Georgia**	Not reported	12.2%<br>(6.7 to 18.0)	Not reported	-5.3%<br>(-21.1 to 8.2)	6.8%<br>(0.4 to 18.4)	Not reported	3.0%<br>(-0.9 to 6.7)	8.5%<br>(-0.7 to 15.6)	Not reported	0.0%<br>(0.0 to 0.0)	Not reported	7.9%<br>(-0.2 to 17.8)	Not reported	Not reported
**Germany**	Not reported	6.3%<br>(4.3 to 8.7)	Not reported	-15.2%<br>(-62.7 to 19.9)	5.1%<br>(0.2 to 15.2)	Not reported	3.9%<br>(-1.2 to 8.6)	7.7%<br>(-0.6 to 14.2)	Not reported	0.0%<br>(0.0 to 0.0)	Not reported	7.7%<br>(-0.2 to 17.4)	Not reported	Not reported
**Ghana**	Not reported	16.0%<br>(7.9 to 25.4)	Not reported	-1.5%<br>(-6.5 to 2.6)	5.0%<br>(0.0 to 15.4)	Not reported	0.0%<br>(0.0 to 0.0)	5.1%<br>(-0.4 to 9.5)	Not reported	2.2%<br>(-0.2 to 4.5)	Not reported	5.4%<br>(0.0 to 12.2)	Not reported	Not reported

**Greece**	Not reported	10.1% (7.4 to 13.2)	Not reported	-15.4% (-62.8 to 20.3)	4.6% (0.1 to 14.7)	Not reported	2.5% (-0.7 to 5.5)	4.1% (-0.3 to 7.8)	Not reported	0.0% (0.0 to 0.0)	Not reported	8.4% (-0.3 to 19.0)	Not reported	Not reported
**Greenland**	Not reported	2.5% (0.1 to 6.4)	Not reported	-16.0% (-66.5 to 20.8)	4.6% (0.1 to 14.5)	Not reported	2.9% (-0.8 to 6.3)	7.2% (-0.5 to 13.3)	Not reported	0.0% (0.0 to 0.0)	Not reported	9.5% (-0.4 to 20.8)	Not reported	Not reported
**Grenada**	Not reported	16.6% (6.5 to 31.1)	Not reported	-2.4% (-9.7 to 4.0)	3.4% (0.0 to 12.0)	Not reported	3.9% (-1.1 to 8.3)	6.5% (-0.5 to 12.0)	Not reported	6.1% (-0.7 to 11.6)	Not reported	9.3% (-0.4 to 19.6)	Not reported	Not reported
**Guam**	Not reported	5.1% (2.4 to 8.2)	Not reported	-11.8% (-47.8 to 15.7)	4.6% (0.4 to 13.0)	Not reported	0.0% (0.0 to 0.0)	7.0% (-0.5 to 12.9)	Not reported	1.1% (-0.1 to 2.5)	Not reported	12.6% (-0.5 to 26.4)	Not reported	Not reported
**Guatemala**	Not reported	10.3% (4.4 to 16.1)	Not reported	-1.5% (-6.3 to 2.9)	6.5% (0.5 to 17.4)	Not reported	1.1% (-0.3 to 2.4)	8.1% (-0.6 to 14.6)	Not reported	1.0% (-0.1 to 2.0)	Not reported	10.3% (-0.4 to 22.2)	Not reported	Not reported
**Guinea**	Not reported	7.1% (4.0 to 11.3)	Not reported	-1.3% (-5.4 to 2.4)	3.1% (0.0 to 11.6)	Not reported	2.1% (-0.6 to 4.6)	7.0% (-0.5 to 12.8)	Not reported	4.3% (-0.4 to 8.7)	Not reported	3.3% (0.0 to 7.9)	Not reported	Not reported
**Guinea-Bissau**	Not reported	7.6% (4.1 to 12.0)	Not reported	-4.1% (-17.2 to 6.5)	3.2% (0.0 to 11.8)	Not reported	4.4% (-1.3 to 9.6)	9.3% (-0.7 to 17.1)	Not reported	12.1% (-1.3 to 22.5)	Not reported	3.8% (0.0 to 8.9)	Not reported	Not reported
**Guyana**	Not reported	16.3% (6.8 to 30.0)	Not reported	-0.8% (-3.2 to 1.3)	3.2% (0.0 to 11.7)	Not reported	5.1% (-1.6 to 10.8)	9.2% (-0.7 to 16.5)	Not reported	0.9% (-0.1 to 2.0)	Not reported	8.2% (-0.2 to 18.2)	Not reported	Not reported
**Haiti**	Not reported	3.4% (1.5 to 6.5)	Not reported	-1.5% (-6.0 to 2.6)	2.9% (0.0 to 10.9)	Not reported	4.0% (-1.3 to 8.5)	7.4% (-0.6 to 13.6)	Not reported	10.2% (-1.0 to 19.0)	Not reported	3.1% (0.0 to 7.5)	Not reported	Not reported
**Honduras**	Not reported	7.6% (3.3 to 12.8)	Not reported	-2.3% (-9.4 to 4.3)	6.9% (0.4 to 18.7)	Not reported	1.8% (-0.5 to 4.0)	7.7% (-0.6 to 14.1)	Not reported	1.6% (-0.2 to 3.1)	Not reported	7.8% (-0.2 to 17.9)	Not reported	Not reported
**Hungary**	Not reported	9.9% (7.3 to 12.9)	Not reported	-13.0% (-53.5 to 18.1)	15.9% (5.1 to 29.6)	Not reported	5.2% (-1.5 to 11.3)	8.9% (-0.7 to 16.2)	Not reported	0.0% (0.0 to 0.0)	Not reported	11.5% (-0.4 to 24.9)	Not reported	Not reported
**Iceland**	Not reported	1.5% (0.3 to 3.3)	Not reported	-15.8% (-64.6 to 20.5)	4.5% (0.1 to 14.3)	Not reported	6.4% (-2.0 to 13.3)	7.7% (-0.6 to 14.1)	Not reported	0.2% (0.0 to 0.4)	Not reported	8.2% (-0.3 to 18.2)	Not reported	Not reported
**India**	Not reported	18.0% (10.8 to 23.9)	Not reported	-0.2% (-0.7 to 0.3)	6.7% (0.5 to 18.5)	Not reported	3.6% (-1.0 to 7.5)	16.6% (-1.3 to 29.6)	Not reported	1.1% (-0.1 to 2.1)	Not reported	2.0% (0.0 to 5.0)	Not reported	Not reported
**Indonesia**	Not reported	11.4% (6.7 to 15.8)	Not reported	-0.7% (-3.1 to 1.3)	10.0% (1.7 to 22.4)	Not reported	6.6% (-2.0 to 13.9)	8.4% (-0.6 to 15.3)	Not reported	2.3% (-0.3 to 4.6)	Not reported	2.5% (0.0 to 6.1)	Not reported	Not reported
**Iran (Islamic Republic of)**	Not reported	21.8% (17.3 to 26.5)	Not reported	-2.6% (-10.9 to 4.5)	1.5% (0.0 to 7.0)	Not reported	1.3% (-0.4 to 2.8)	3.6% (-0.2 to 6.6)	Not reported	0.0% (0.0 to 0.0)	Not reported	8.9% (-0.4 to 19.5)	Not reported	Not reported
**Iraq**	Not reported	23.7% (17.3 to 30.6)	Not reported	-0.7% (-2.8 to 1.2)	1.6% (0.0 to 7.3)	Not reported	3.3% (-0.9 to 7.3)	7.6% (-0.6 to 13.9)	Not reported	0.0% (0.0 to 0.0)	Not reported	10.6% (-0.4 to 22.7)	Not reported	Not reported
**Ireland**	Not reported	3.8% (1.9 to 6.0)	Not reported	-16.1% (-66.6 to 20.6)	3.1% (0.0 to 11.4)	Not reported	2.4% (-0.7 to 5.2)	7.1% (-0.5 to 13.1)	Not reported	0.0% (0.0 to 0.0)	Not reported	7.7% (-0.2 to 17.4)	Not reported	Not reported
**Israel**	Not reported	13.8% (10.6 to 17.5)	Not reported	-10.1% (-40.6 to 14.2)	4.7% (0.1 to 14.8)	Not reported	1.0% (-0.3 to 2.0)	4.1% (-0.3 to 7.7)	Not reported	0.0% (0.0 to 0.0)	Not reported	6.6% (-0.1 to 15.3)	Not reported	Not reported
**Italy**	Not reported	9.4% (7.1 to 12.2)	Not reported	-12.6% (-51.3 to 16.6)	6.3% (0.5 to 16.9)	Not reported	3.3% (-0.9 to 6.9)	4.7% (-0.3 to 9.0)	Not reported	0.0% (0.0 to 0.0)	Not reported	5.4% (0.0 to 12.6)	Not reported	Not reported
**Jamaica**	Not reported	11.3% (5.9 to 18.2)	Not reported	-2.6% (-10.5 to 4.7)	3.2% (0.0 to 11.6)	Not reported	2.5% (-0.8 to 5.2)	7.4% (-0.5 to 13.5)	Not reported	0.2% (0.0 to 0.4)	Not reported	10.8% (-0.4 to 23.2)	Not reported	Not reported
**Japan**	Not reported	8.4% (4.7 to 13.1)	Not reported	-6.1% (-24.7 to 9.5)	8.6% (0.8 to 21.2)	Not reported	4.5% (-1.3 to 9.5)	9.9% (-0.7 to 17.9)	Not reported	0.0% (0.0 to 0.0)	Not reported	2.0% (-0.1 to 5.4)	Not reported	Not reported

**Jordan**	Not reported	18.6% (14.1 to 23.3)	Not reported	-3.1% (-13.2 to 5.3)	1.4% (0.0 to 6.6)	Not reported	3.4% (-1.0 to 7.4)	6.9% (-0.5 to 12.7)	Not reported	0.0% (0.0 to 0.0)	Not reported	12.4% (-0.6 to 25.2)	Not reported	Not reported
**Kazakhstan**	Not reported	15.0% (10.1 to 21.0)	Not reported	-14.5% (-58.8 to 19.3)	6.4% (0.4 to 17.8)	Not reported	2.9% (-0.9 to 6.5)	8.0% (-0.6 to 14.9)	Not reported	0.0% (0.0 to 0.0)	Not reported	7.8% (-0.2 to 17.6)	Not reported	Not reported
**Kenya**	Not reported	5.5% (3.0 to 8.6)	Not reported	-3.9% (-16.0 to 6.3)	3.5% (0.2 to 10.8)	Not reported	1.0% (-0.3 to 2.2)	8.8% (-0.7 to 16.0)	Not reported	1.9% (-0.3 to 3.9)	Not reported	4.0% (0.0 to 9.4)	Not reported	Not reported
**Kiribati**	Not reported	2.4% (0.8 to 5.4)	Not reported	-3.2% (-13.5 to 5.3)	4.5% (0.4 to 12.4)	Not reported	1.8% (-0.5 to 4.2)	8.7% (-0.7 to 15.8)	Not reported	5.7% (-0.7 to 10.9)	Not reported	13.6% (-0.6 to 27.6)	Not reported	Not reported
**Kuwait**	Not reported	27.1% (21.4 to 32.5)	Not reported	-9.5% (-38.0 to 12.8)	3.0% (0.0 to 10.8)	Not reported	2.4% (-0.7 to 5.2)	6.4% (-0.5 to 11.6)	Not reported	0.0% (0.0 to 0.0)	Not reported	14.0% (-0.8 to 27.4)	Not reported	Not reported
**Kyrgyzstan**	Not reported	12.0% (5.9 to 19.3)	Not reported	-12.5% (-50.1 to 16.7)	5.9% (0.3 to 16.8)	Not reported	2.8% (-0.8 to 6.5)	9.7% (-0.8 to 17.8)	Not reported	0.0% (0.0 to 0.0)	Not reported	8.9% (-0.2 to 19.6)	Not reported	Not reported
**Lao People's Democratic Republic**	Not reported	9.5% (3.8 to 15.8)	Not reported	-4.8% (-19.4 to 7.5)	9.4% (1.3 to 22.3)	Not reported	9.3% (-3.0 to 19.0)	7.6% (-0.6 to 13.7)	Not reported	0.6% (-0.1 to 1.3)	Not reported	2.1% (0.0 to 5.5)	Not reported	Not reported
**Latvia**	Not reported	8.2% (5.5 to 11.3)	Not reported	-11.0% (-44.5 to 15.4)	4.8% (0.1 to 15.0)	Not reported	3.6% (-1.0 to 7.9)	9.5% (-0.7 to 17.6)	Not reported	0.0% (0.0 to 0.0)	Not reported	9.4% (-0.3 to 20.4)	Not reported	Not reported
**Lebanon**	Not reported	13.7% (8.8 to 19.6)	Not reported	-6.7% (-27.6 to 10.2)	1.6% (0.0 to 7.3)	Not reported	0.9% (-0.2 to 1.9)	4.1% (-0.3 to 7.8)	Not reported	0.0% (0.0 to 0.0)	Not reported	10.6% (-0.5 to 22.4)	Not reported	Not reported
**Lesotho**	Not reported	5.9% (3.0 to 10.0)	Not reported	-5.1% (-21.7 to 7.9)	2.8% (0.0 to 11.3)	Not reported	0.1% (0.0 to 0.1)	13.8% (-1.1 to 25.3)	Not reported	9.1% (-0.9 to 17.4)	Not reported	6.9% (-0.1 to 15.4)	Not reported	Not reported
**Liberia**	Not reported	5.4% (3.3 to 8.1)	Not reported	-0.7% (-3.0 to 1.2)	3.2% (0.0 to 12.0)	Not reported	7.1% (-2.3 to 14.6)	9.5% (-0.7 to 17.5)	Not reported	9.6% (-1.0 to 18.6)	Not reported	8.3% (-0.3 to 18.1)	Not reported	Not reported
**Libya**	Not reported	18.3% (13.0 to 24.8)	Not reported	-2.8% (-11.7 to 4.7)	1.2% (0.0 to 6.1)	Not reported	2.2% (-0.7 to 4.7)	5.4% (-0.4 to 9.7)	Not reported	0.0% (0.0 to 0.0)	Not reported	10.9% (-0.6 to 22.8)	Not reported	Not reported
**Lithuania**	Not reported	6.1% (3.8 to 8.8)	Not reported	-14.2% (-57.5 to 19.8)	4.2% (0.0 to 14.4)	Not reported	1.5% (-0.4 to 3.5)	8.8% (-0.7 to 16.1)	Not reported	0.0% (0.0 to 0.0)	Not reported	9.2% (-0.3 to 20.3)	Not reported	Not reported
**Luxembourg**	Not reported	4.3% (2.5 to 6.4)	Not reported	-14.9% (-61.9 to 19.0)	4.5% (0.1 to 14.7)	Not reported	3.6% (-1.0 to 7.6)	7.0% (-0.5 to 13.0)	Not reported	0.0% (0.0 to 0.0)	Not reported	7.0% (-0.2 to 16.2)	Not reported	Not reported
**Madagascar**	Not reported	2.2% (1.3 to 3.5)	Not reported	-2.8% (-11.6 to 4.7)	5.0% (0.1 to 14.8)	Not reported	4.6% (-1.4 to 9.6)	9.7% (-0.8 to 17.7)	Not reported	11.6% (-1.2 to 21.6)	Not reported	1.6% (-0.1 to 4.3)	Not reported	Not reported
**Malawi**	Not reported	3.3% (2.0 to 5.3)	Not reported	-1.0% (-3.9 to 1.7)	5.4% (0.2 to 15.5)	Not reported	0.1% (0.0 to 0.2)	8.6% (-0.6 to 15.7)	Not reported	8.9% (-0.9 to 16.9)	Not reported	2.2% (-0.1 to 5.8)	Not reported	Not reported
**Malaysia**	Not reported	12.4% (8.2 to 17.5)	Not reported	-2.4% (-10.0 to 4.0)	10.5% (1.5 to 23.2)	Not reported	6.8% (-2.1 to 14.5)	7.2% (-0.5 to 13.3)	Not reported	0.6% (-0.1 to 1.3)	Not reported	5.8% (-0.1 to 12.7)	Not reported	Not reported
**Maldives**	Not reported	7.7% (4.5 to 11.7)	Not reported	-0.5% (-1.9 to 0.9)	10.2% (1.6 to 22.7)	Not reported	6.5% (-2.0 to 13.6)	7.9% (-0.6 to 14.5)	Not reported	0.1% (0.0 to 0.2)	Not reported	4.5% (0.0 to 10.4)	Not reported	Not reported
**Mali**	Not reported	8.8% (4.8 to 14.2)	Not reported	-7.6% (-31.2 to 11.2)	2.9% (0.0 to 11.1)	Not reported	0.5% (-0.1 to 1.1)	9.6% (-0.7 to 17.6)	Not reported	7.4% (-0.7 to 13.9)	Not reported	2.5% (-0.1 to 6.6)	Not reported	Not reported
**Malta**	Not reported	7.7% (5.4 to 10.5)	Not reported	-12.9% (-53.3 to 16.9)	6.5% (0.4 to 17.5)	Not reported	2.6% (-0.8 to 5.3)	7.5% (-0.6 to 13.8)	Not reported	0.0% (0.0 to 0.0)	Not reported	7.4% (-0.2 to 16.7)	Not reported	Not reported
**Marshall Islands**	Not reported	4.3% (1.5 to 9.5)	Not reported	-6.7% (-28.6 to 10.4)	4.8% (0.4 to 13.6)	Not reported	0.8% (-0.2 to 1.9)	9.8% (-0.8 to 17.9)	Not reported	8.8% (-1.0 to 16.9)	Not reported	14.1% (-0.6 to 28.9)	Not reported	Not reported

**Mauritania**	Not reported	19.8% (10.3 to 29.8)	Not reported	-7.3% (-29.3 to 11.4)	3.3% (0.0 to 11.9)	Not reported	1.5% (-0.4 to 3.4)	14.9% (-1.2 to 26.9)	Not reported	8.7% (-0.9 to 16.6)	Not reported	8.0% (-0.2 to 17.5)	Not reported	Not reported
**Mauritius**	Not reported	5.0% (1.7 to 9.0)	Not reported	-3.6% (-15.0 to 6.3)	9.7% (1.3 to 22.3)	Not reported	5.5% (-1.8 to 11.9)	11.1% (-0.9 to 20.0)	Not reported	0.2% (0.0 to 0.4)	Not reported	6.9% (-0.1 to 15.4)	Not reported	Not reported
**Mexico**	Not reported	9.3% (6.3 to 12.9)	Not reported	-10.9% (-45.0 to 15.1)	4.6% (0.1 to 14.0)	Not reported	0.7% (-0.2 to 1.6)	6.3% (-0.5 to 11.7)	Not reported	0.6% (-0.1 to 1.2)	Not reported	11.3% (-0.5 to 24.0)	Not reported	Not reported
**Micronesia (Federated States of)**	Not reported	4.8% (1.6 to 11.1)	Not reported	-6.6% (-27.5 to 10.0)	4.9% (0.4 to 13.4)	Not reported	0.8% (-0.2 to 2.0)	10.0% (-0.8 to 17.9)	Not reported	9.2% (-1.0 to 17.5)	Not reported	15.0% (-0.7 to 30.5)	Not reported	Not reported
**Monaco**	Not reported	5.3% (2.8 to 8.2)	Not reported	-18.3% (-75.4 to 22.1)	4.1% (0.1 to 13.3)	Not reported	0.8% (-0.2 to 1.8)	4.0% (-0.3 to 7.7)	Not reported	0.0% (0.0 to 0.0)	Not reported	9.3% (-0.3 to 20.9)	Not reported	Not reported
**Mongolia**	Not reported	19.9% (9.9 to 27.7)	Not reported	-16.4% (-67.8 to 20.8)	6.5% (0.3 to 18.1)	Not reported	8.3% (-2.8 to 17.3)	14.7% (-1.2 to 27.1)	Not reported	1.9% (-0.2 to 3.7)	Not reported	5.9% (-0.1 to 13.6)	Not reported	Not reported
**Montenegro**	Not reported	12.6% (8.2 to 16.6)	Not reported	-13.3% (-53.5 to 17.1)	12.9% (3.3 to 26.4)	Not reported	1.6% (-0.5 to 3.6)	4.2% (-0.3 to 7.6)	Not reported	0.0% (0.0 to 0.0)	Not reported	10.3% (-0.4 to 22.1)	Not reported	Not reported
**Morocco**	Not reported	14.0% (9.8 to 18.8)	Not reported	-3.7% (-15.3 to 6.2)	1.5% (0.0 to 7.5)	Not reported	0.1% (0.0 to 0.2)	4.4% (-0.3 to 8.3)	Not reported	0.0% (0.0 to 0.0)	Not reported	8.0% (-0.3 to 18.1)	Not reported	Not reported
**Mozambique**	Not reported	3.3% (2.0 to 5.2)	Not reported	-0.9% (-3.4 to 1.6)	5.5% (0.1 to 16.0)	Not reported	1.5% (-0.4 to 3.5)	12.4% (-1.0 to 22.5)	Not reported	10.3% (-1.1 to 19.5)	Not reported	3.0% (0.0 to 6.9)	Not reported	Not reported
**Myanmar**	Not reported	12.1% (5.5 to 19.2)	Not reported	-3.3% (-13.0 to 5.3)	9.6% (1.2 to 21.7)	Not reported	8.6% (-2.7 to 17.6)	10.0% (-0.8 to 18.0)	Not reported	0.3% (0.0 to 0.7)	Not reported	1.6% (-0.2 to 4.4)	Not reported	Not reported
**Namibia**	Not reported	12.6% (5.9 to 18.7)	Not reported	-7.7% (-32.0 to 11.6)	2.8% (0.0 to 11.1)	Not reported	4.5% (-1.3 to 9.8)	11.8% (-0.9 to 21.5)	Not reported	5.4% (-0.6 to 10.3)	Not reported	6.6% (-0.1 to 14.8)	Not reported	Not reported
**Nauru**	Not reported	2.2% (0.4 to 4.9)	Not reported	-7.9% (-31.4 to 12.0)	4.6% (0.3 to 12.9)	Not reported	0.2% (-0.1 to 0.6)	8.3% (-0.7 to 15.1)	Not reported	5.7% (-0.7 to 10.9)	Not reported	17.9% (-1.0 to 34.2)	Not reported	Not reported
**Nepal**	Not reported	12.0% (7.3 to 17.5)	Not reported	-3.2% (-13.0 to 5.6)	5.3% (0.1 to 15.9)	Not reported	2.8% (-0.9 to 6.2)	9.0% (-0.7 to 16.5)	Not reported	1.0% (-0.1 to 2.1)	Not reported	2.0% (-0.1 to 5.4)	Not reported	Not reported
**Netherlands**	Not reported	7.0% (4.9 to 9.5)	Not reported	-14.5% (-59.2 to 19.2)	4.0% (0.1 to 13.9)	Not reported	3.9% (-1.2 to 8.3)	6.9% (-0.5 to 12.7)	Not reported	0.1% (0.0 to 0.2)	Not reported	6.5% (-0.1 to 14.9)	Not reported	Not reported
**New Zealand**	Not reported	3.3% (1.4 to 5.6)	Not reported	-17.2% (-71.1 to 21.9)	4.0% (0.0 to 13.4)	Not reported	4.2% (-1.2 to 9.1)	7.3% (-0.5 to 13.5)	Not reported	0.0% (0.0 to 0.1)	Not reported	10.5% (-0.4 to 22.8)	Not reported	Not reported
**Nicaragua**	Not reported	6.8% (2.9 to 12.0)	Not reported	-1.3% (-5.4 to 2.3)	7.0% (0.4 to 19.1)	Not reported	2.9% (-0.9 to 6.2)	12.1% (-1.0 to 21.7)	Not reported	11.2% (-1.2 to 20.8)	Not reported	10.8% (-0.4 to 23.5)	Not reported	Not reported
**Niger**	Not reported	8.0% (4.3 to 12.9)	Not reported	-6.0% (-23.7 to 9.4)	3.3% (0.0 to 12.1)	Not reported	0.8% (-0.2 to 1.8)	12.2% (-0.9 to 22.3)	Not reported	0.5% (0.0 to 1.0)	Not reported	2.1% (-0.1 to 5.7)	Not reported	Not reported
**Nigeria**	Not reported	15.5% (7.6 to 24.3)	Not reported	-1.6% (-7.0 to 2.8)	2.8% (0.0 to 11.2)	Not reported	0.3% (-0.1 to 0.7)	8.1% (-0.6 to 15.1)	Not reported	0.5% (-0.1 to 1.0)	Not reported	4.2% (0.0 to 10.1)	Not reported	Not reported
**Niue**	Not reported	2.1% (0.3 to 4.4)	Not reported	-6.1% (-25.0 to 8.2)	4.0% (0.3 to 11.1)	Not reported	0.1% (0.0 to 0.4)	6.2% (-0.4 to 11.2)	Not reported	3.9% (-0.5 to 7.5)	Not reported	11.3% (-0.6 to 22.3)	Not reported	Not reported
**North Macedonia**	Not reported	17.7% (11.8 to 22.2)	Not reported	-5.6% (-22.7 to 8.6)	12.8% (3.1 to 26.5)	Not reported	2.2% (-0.6 to 4.8)	6.2% (-0.4 to 11.3)	Not reported	0.0% (0.0 to 0.0)	Not reported	9.0% (-0.3 to 19.7)	Not reported	Not reported
**Northern Mariana Islands**	Not reported	5.5% (2.6 to 8.7)	Not reported	-9.4% (-39.7 to 12.8)	6.0% (0.5 to 16.1)	Not reported	0.0% (0.0 to 0.1)	7.1% (-0.5 to 13.2)	Not reported	2.6% (-0.3 to 5.4)	Not reported	15.4% (-0.8 to 30.6)	Not reported	Not reported

**Norway**	Not reported	2.3%<br>(0.9 to 4.2)	Not reported	-12.8%<br>(-52.6 to 17.2)	3.9%<br>(0.1 to 13.0)	Not reported	3.1%<br>(-0.8 to 6.6)	6.3%<br>(-0.4 to 11.9)	Not reported	0.3%<br>(0.0 to 0.7)	Not reported	5.7%<br>(-0.1 to 13.4)	Not reported	Not reported
**Oman**	Not reported	26.5%<br>(19.4 to 34.6)	Not reported	-7.7%<br>(-31.4 to 12.0)	1.6%<br>(0.0 to 7.7)	Not reported	1.2%<br>(-0.3 to 2.6)	3.8%<br>(-0.3 to 7.2)	Not reported	0.0%<br>(0.0 to 0.0)	Not reported	11.4%<br>(-0.6 to 23.9)	Not reported	Not reported
**Pakistan**	Not reported	15.8%<br>(8.0 to 24.1)	Not reported	-3.5%<br>(-14.4 to 5.8)	5.3%<br>(0.1 to 16.2)	Not reported	6.4%<br>(-2.0 to 13.1)	9.9%<br>(-0.8 to 18.1)	Not reported	5.8%<br>(-0.6 to 11.5)	Not reported	3.9%<br>(0.0 to 8.8)	Not reported	Not reported
**Palau**	Not reported	3.3%<br>(0.3 to 6.7)	Not reported	-9.0%<br>(-36.7 to 12.1)	4.7%<br>(0.4 to 13.2)	Not reported	0.1%<br>(0.0 to 0.5)	8.2%<br>(-0.6 to 14.5)	Not reported	4.9%<br>(-0.5 to 9.3)	Not reported	14.6%<br>(-0.7 to 29.8)	Not reported	Not reported
**Palestine**	Not reported	17.9%<br>(12.1 to 24.6)	Not reported	-1.3%<br>(-5.3 to 2.1)	1.3%<br>(0.0 to 6.5)	Not reported	5.9%<br>(-1.9 to 12.2)	6.8%<br>(-0.5 to 12.3)	Not reported	0.1%<br>(0.0 to 0.2)	Not reported	11.5%<br>(-0.5 to 23.6)	Not reported	Not reported
**Panama**	Not reported	7.3%<br>(4.0 to 11.7)	Not reported	-10.3%<br>(-41.8 to 14.3)	7.3%<br>(0.5 to 19.5)	Not reported	7.7%<br>(-2.5 to 16.1)	7.9%<br>(-0.6 to 14.4)	Not reported	4.1%<br>(-0.4 to 7.8)	Not reported	9.4%<br>(-0.3 to 20.7)	Not reported	Not reported
**Papua New Guinea**	Not reported	4.3%<br>(1.3 to 10.0)	Not reported	-4.1%<br>(-16.8 to 6.7)	5.4%<br>(0.4 to 14.3)	Not reported	0.2%<br>(0.0 to 0.5)	8.1%<br>(-0.6 to 15.2)	Not reported	3.1%<br>(-0.4 to 6.3)	Not reported	3.8%<br>(0.0 to 9.7)	Not reported	Not reported
**Paraguay**	Not reported	6.5%<br>(2.4 to 12.4)	Not reported	-16.2%<br>(-66.1 to 20.8)	5.6%<br>(0.1 to 16.7)	Not reported	1.1%<br>(-0.3 to 2.5)	6.9%<br>(-0.5 to 12.8)	Not reported	2.9%<br>(-0.3 to 5.6)	Not reported	9.4%<br>(-0.3 to 21.2)	Not reported	Not reported
**Peru**	Not reported	17.5%<br>(10.6 to 24.3)	Not reported	-3.0%<br>(-12.2 to 5.3)	4.7%<br>(0.1 to 13.8)	Not reported	2.0%<br>(-0.6 to 4.5)	7.4%<br>(-0.6 to 13.3)	Not reported	1.2%<br>(-0.1 to 2.4)	Not reported	8.6%<br>(-0.2 to 19.1)	Not reported	Not reported
**Philippines**	Not reported	12.0%<br>(6.9 to 16.3)	Not reported	-7.0%<br>(-28.0 to 10.6)	9.1%<br>(1.5 to 20.9)	Not reported	9.5%<br>(-3.1 to 19.5)	7.3%<br>(-0.6 to 13.2)	Not reported	1.3%<br>(-0.1 to 2.6)	Not reported	3.9%<br>(0.0 to 8.9)	Not reported	Not reported
**Poland**	Not reported	13.1%<br>(9.9 to 16.5)	Not reported	-16.5%<br>(-68.7 to 21.3)	9.9%<br>(1.6 to 22.7)	Not reported	1.9%<br>(-0.5 to 4.2)	9.3%<br>(-0.7 to 16.9)	Not reported	0.0%<br>(0.0 to 0.0)	Not reported	8.1%<br>(-0.2 to 18.6)	Not reported	Not reported
**Portugal**	Not reported	3.8%<br>(2.0 to 5.9)	Not reported	-16.1%<br>(-66.3 to 21.2)	5.3%<br>(0.2 to 15.3)	Not reported	3.0%<br>(-0.9 to 6.4)	6.6%<br>(-0.5 to 12.3)	Not reported	0.0%<br>(0.0 to 0.0)	Not reported	7.3%<br>(-0.2 to 16.8)	Not reported	Not reported
**Puerto Rico**	Not reported	2.5%<br>(0.7 to 4.6)	Not reported	-6.0%<br>(-25.3 to 8.8)	3.3%<br>(0.0 to 11.9)	Not reported	3.6%<br>(-1.0 to 7.7)	6.5%<br>(-0.5 to 11.9)	Not reported	6.1%<br>(-0.6 to 11.5)	Not reported	12.8%<br>(-0.6 to 26.2)	Not reported	Not reported
**Qatar**	Not reported	38.7%<br>(30.6 to 46.2)	Not reported	-5.9%<br>(-24.4 to 9.4)	1.8%<br>(0.0 to 8.1)	Not reported	0.1%<br>(0.0 to 0.3)	2.2%<br>(-0.1 to 4.3)	Not reported	0.0%<br>(0.0 to 0.0)	Not reported	12.4%<br>(-0.7 to 25.7)	Not reported	Not reported
**Republic of Korea**	Not reported	18.6%<br>(12.7 to 25.5)	Not reported	-12.6%<br>(-51.5 to 17.1)	11.9%<br>(2.4 to 25.5)	Not reported	7.5%<br>(-2.4 to 15.5)	6.9%<br>(-0.5 to 12.8)	Not reported	0.0%<br>(0.0 to 0.0)	Not reported	2.1%<br>(-0.1 to 5.8)	Not reported	Not reported
**Republic of Moldova**	Not reported	8.8%<br>(4.2 to 14.6)	Not reported	-5.0%<br>(-19.8 to 8.2)	3.6%<br>(0.0 to 12.7)	Not reported	2.2%<br>(-0.6 to 4.9)	8.7%<br>(-0.7 to 15.8)	Not reported	0.0%<br>(0.0 to 0.0)	Not reported	10.2%<br>(-0.3 to 21.7)	Not reported	Not reported
**Romania**	Not reported	10.0%<br>(7.3 to 12.9)	Not reported	-14.9%<br>(-61.3 to 19.4)	13.8%<br>(3.8 to 26.9)	Not reported	1.3%<br>(-0.4 to 2.8)	7.3%<br>(-0.6 to 13.4)	Not reported	0.0%<br>(0.0 to 0.0)	Not reported	9.0%<br>(-0.3 to 19.6)	Not reported	Not reported
**Russian Federation**	Not reported	7.3%<br>(4.4 to 11.1)	Not reported	-11.7%<br>(-46.0 to 16.4)	7.8%<br>(0.7 to 19.7)	Not reported	3.7%<br>(-1.1 to 7.9)	9.0%<br>(-0.7 to 16.5)	Not reported	0.1%<br>(0.0 to 0.1)	Not reported	9.5%<br>(-0.3 to 21.0)	Not reported	Not reported
**Rwanda**	Not reported	3.8%<br>(1.8 to 6.9)	Not reported	-1.0%<br>(-4.0 to 1.7)	5.5%<br>(0.2 to 15.9)	Not reported	0.0%<br>(0.0 to 0.0)	0.6%<br>(0.0 to 1.2)	Not reported	6.2%<br>(-0.7 to 11.9)	Not reported	1.1%<br>(-0.3 to 3.8)	Not reported	Not reported
**Saint Kitts and Nevis**	Not reported	3.6%<br>(1.3 to 6.3)	Not reported	-5.3%<br>(-22.1 to 8.2)	3.5%<br>(0.0 to 12.3)	Not reported	9.6%<br>(-3.2 to 19.6)	9.8%<br>(-0.8 to 17.7)	Not reported	1.5%<br>(-0.2 to 3.1)	Not reported	9.7%<br>(-0.4 to 21.1)	Not reported	Not reported
**Saint Lucia**	Not reported	15.5%<br>(6.2 to 27.9)	Not reported	-8.8%<br>(-36.3 to 12.6)	3.2%<br>(0.0 to 12.0)	Not reported	6.7%<br>(-2.1 to 13.9)	7.5%<br>(-0.6 to 13.6)	Not reported	5.4%<br>(-0.6 to 10.2)	Not reported	9.5%<br>(-0.4 to 20.4)	Not reported	Not reported

**Saint Vincent and the Grenadines**	Not reported	15.4% (5.8 to 28.9)	Not reported	-4.1% (-16.7 to 6.7)	3.1% (0.0 to 11.3)	Not reported	4.8% (-1.5 to 9.9)	6.6% (-0.5 to 11.9)	Not reported	3.4% (-0.4 to 6.8)	Not reported	7.2% (-0.1 to 16.2)	Not reported	Not reported
**Samoa**	Not reported	5.8% (1.6 to 13.4)	Not reported	-8.2% (-32.4 to 11.8)	1.7% (0.0 to 7.2)	Not reported	0.0% (0.0 to 0.1)	8.9% (-0.7 to 16.1)	Not reported	11.7% (-1.4 to 22.1)	Not reported	16.1% (-0.9 to 32.2)	Not reported	Not reported
**San Marino**	Not reported	6.1% (3.3 to 9.4)	Not reported	-15.3% (-61.8 to 20.3)	4.4% (0.1 to 14.4)	Not reported	3.3% (-1.0 to 7.1)	6.8% (-0.5 to 12.5)	Not reported	0.0% (0.0 to 0.0)	Not reported	8.4% (-0.3 to 18.7)	Not reported	Not reported
**Sao Tome and Principe**	Not reported	7.7% (3.9 to 12.9)	Not reported	-0.3% (-1.2 to 0.5)	3.3% (0.0 to 12.2)	Not reported	0.1% (0.0 to 0.2)	5.7% (-0.4 to 10.8)	Not reported	7.2% (-0.8 to 13.8)	Not reported	6.9% (0.0 to 16.0)	Not reported	Not reported
**Saudi Arabia**	Not reported	33.1% (26.0 to 40.5)	Not reported	-3.0% (-12.2 to 5.2)	1.5% (0.0 to 7.1)	Not reported	1.4% (-0.4 to 3.0)	7.8% (-0.6 to 14.2)	Not reported	0.0% (0.0 to 0.0)	Not reported	15.8% (-0.8 to 32.0)	Not reported	Not reported
**Senegal**	Not reported	7.7% (3.7 to 13.5)	Not reported	-2.4% (-9.9 to 3.9)	3.2% (0.0 to 11.8)	Not reported	0.4% (-0.1 to 1.0)	11.5% (-0.9 to 21.0)	Not reported	2.1% (-0.2 to 4.4)	Not reported	4.5% (0.0 to 10.3)	Not reported	Not reported
**Serbia**	Not reported	15.9% (10.9 to 20.2)	Not reported	-7.7% (-30.9 to 11.5)	13.3% (3.3 to 26.8)	Not reported	3.4% (-1.0 to 7.1)	6.1% (-0.4 to 11.4)	Not reported	0.0% (0.0 to 0.0)	Not reported	10.2% (-0.3 to 21.9)	Not reported	Not reported
**Seychelles**	Not reported	5.1% (1.7 to 9.4)	Not reported	-1.9% (-8.5 to 2.9)	6.8% (0.7 to 16.6)	Not reported	4.5% (-1.3 to 9.3)	8.5% (-0.7 to 15.4)	Not reported	5.0% (-0.6 to 9.6)	Not reported	10.5% (-0.4 to 22.2)	Not reported	Not reported
**Sierra Leone**	Not reported	5.6% (3.2 to 8.7)	Not reported	-0.5% (-2.1 to 0.9)	3.1% (0.0 to 12.1)	Not reported	7.5% (-2.4 to 15.8)	9.7% (-0.7 to 18.1)	Not reported	4.5% (-0.5 to 8.9)	Not reported	3.2% (0.0 to 8.0)	Not reported	Not reported
**Singapore**	Not reported	11.8% (6.3 to 18.4)	Not reported	-12.5% (-50.6 to 17.4)	10.2% (1.5 to 23.1)	Not reported	4.6% (-1.4 to 10.0)	5.9% (-0.4 to 10.8)	Not reported	0.0% (0.0 to 0.0)	Not reported	5.2% (0.0 to 11.8)	Not reported	Not reported
**Slovakia**	Not reported	10.7% (8.0 to 13.7)	Not reported	-13.6% (-53.9 to 18.3)	12.9% (3.4 to 26.4)	Not reported	6.5% (-2.0 to 13.2)	8.9% (-0.7 to 16.1)	Not reported	0.0% (0.0 to 0.0)	Not reported	9.8% (-0.3 to 21.6)	Not reported	Not reported
**Slovenia**	Not reported	9.3% (6.7 to 12.2)	Not reported	-13.3% (-53.5 to 18.2)	13.9% (3.6 to 27.4)	Not reported	3.0% (-0.9 to 6.5)	6.2% (-0.5 to 11.4)	Not reported	0.0% (0.0 to 0.1)	Not reported	9.2% (-0.3 to 20.1)	Not reported	Not reported
**Solomon Islands**	Not reported	2.9% (1.0 to 6.5)	Not reported	-1.7% (-7.1 to 3.0)	5.8% (0.5 to 15.5)	Not reported	0.2% (0.0 to 0.4)	9.4% (-0.7 to 17.1)	Not reported	13.1% (-1.3 to 24.3)	Not reported	7.7% (-0.1 to 17.2)	Not reported	Not reported
**Somalia**	Not reported	3.1% (2.0 to 4.8)	Not reported	-5.1% (-21.1 to 8.1)	5.6% (0.2 to 16.2)	Not reported	8.8% (-2.9 to 18.1)	13.1% (-1.0 to 23.9)	Not reported	12.6% (-1.2 to 23.4)	Not reported	2.0% (-0.2 to 5.4)	Not reported	Not reported
**South Africa**	Not reported	15.1% (10.7 to 19.9)	Not reported	-9.8% (-40.3 to 13.9)	2.3% (0.0 to 10.0)	Not reported	2.0% (-0.6 to 4.2)	11.6% (-0.9 to 21.0)	Not reported	2.3% (-0.3 to 4.6)	Not reported	10.6% (-0.5 to 22.4)	Not reported	Not reported
**South Sudan**	Not reported	6.2% (3.3 to 10.4)	Not reported	-2.6% (-10.6 to 4.5)	5.3% (0.2 to 15.5)	Not reported	0.3% (-0.1 to 0.7)	8.6% (-0.6 to 15.7)	Not reported	6.1% (-0.7 to 11.8)	Not reported	0.1% (-0.8 to 1.7)	Not reported	Not reported
**Spain**	Not reported	5.1% (3.3 to 7.4)	Not reported	-13.7% (-57.5 to 18.0)	3.7% (0.1 to 11.9)	Not reported	4.2% (-1.2 to 8.9)	4.8% (-0.3 to 9.1)	Not reported	0.0% (0.0 to 0.0)	Not reported	8.3% (-0.3 to 18.7)	Not reported	Not reported
**Sri Lanka**	Not reported	12.6% (5.6 to 18.5)	Not reported	-0.1% (-0.6 to 0.3)	10.2% (1.4 to 22.7)	Not reported	2.4% (-0.8 to 5.2)	9.5% (-0.7 to 17.3)	Not reported	1.6% (-0.2 to 3.3)	Not reported	3.4% (0.0 to 7.9)	Not reported	Not reported
**Sudan**	Not reported	16.9% (9.1 to 26.5)	Not reported	-2.4% (-10.0 to 3.6)	1.5% (0.0 to 7.6)	Not reported	3.8% (-1.1 to 8.0)	6.9% (-0.5 to 12.8)	Not reported	0.2% (0.0 to 0.5)	Not reported	8.9% (-0.4 to 19.6)	Not reported	Not reported
**Suriname**	Not reported	18.8% (8.0 to 31.4)	Not reported	-1.6% (-6.9 to 2.9)	2.9% (0.0 to 10.7)	Not reported	9.6% (-3.3 to 19.5)	8.0% (-0.6 to 14.7)	Not reported	2.2% (-0.3 to 4.5)	Not reported	6.7% (-0.1 to 15.5)	Not reported	Not reported
**Sweden**	Not reported	2.0% (0.6 to 3.9)	Not reported	-12.8% (-53.5 to 17.5)	4.2% (0.0 to 13.9)	Not reported	3.3% (-1.0 to 7.2)	6.7% (-0.5 to 12.4)	Not reported	0.1% (0.0 to 0.3)	Not reported	6.3% (-0.1 to 14.6)	Not reported	Not reported

**Switzerland**	Not reported	5.2% (3.4 to 7.4)	Not reported	-14.3% (-58.7 to 19.1)	4.2% (0.1 to 13.3)	Not reported	5.8% (-1.9 to 12.1)	6.7% (-0.5 to 12.5)	Not reported	0.0% (0.0 to 0.1)	Not reported	5.0% (0.0 to 11.9)	Not reported	Not reported
**Syrian Arab Republic**	Not reported	16.9% (12.1 to 22.3)	Not reported	-3.3% (-12.9 to 5.4)	1.5% (0.0 to 7.2)	Not reported	3.0% (-0.9 to 6.4)	6.1% (-0.5 to 11.2)	Not reported	0.0% (0.0 to 0.1)	Not reported	12.8% (-0.6 to 26.6)	Not reported	Not reported
**Turkey**	Not reported	16.2% (12.5 to 20.2)	Not reported	-3.3% (-13.8 to 5.6)	1.1% (0.0 to 6.4)	Not reported	0.3% (-0.1 to 0.6)	2.8% (-0.2 to 5.3)	Not reported	0.0% (0.0 to 0.0)	Not reported	10.8% (-0.4 to 22.7)	Not reported	Not reported
**Taiwan (Province of China)**	Not reported	11.8% (8.9 to 15.0)	Not reported	-15.4% (-64.1 to 20.4)	6.2% (0.3 to 17.1)	Not reported	5.1% (-1.6 to 10.4)	5.9% (-0.4 to 10.9)	Not reported	0.0% (0.0 to 0.0)	Not reported	5.4% (0.0 to 12.7)	Not reported	Not reported
**Tajikistan**	Not reported	12.9% (7.0 to 19.9)	Not reported	-2.7% (-11.4 to 4.7)	5.8% (0.3 to 17.2)	Not reported	6.3% (-1.9 to 13.3)	9.1% (-0.7 to 16.6)	Not reported	0.0% (0.0 to 0.0)	Not reported	7.1% (-0.2 to 16.9)	Not reported	Not reported
**Thailand**	Not reported	20.7% (14.8 to 26.6)	Not reported	-3.8% (-15.1 to 6.0)	9.9% (1.4 to 22.8)	Not reported	8.9% (-3.0 to 18.5)	6.0% (-0.4 to 11.1)	Not reported	1.9% (-0.2 to 3.9)	Not reported	4.2% (0.0 to 9.7)	Not reported	Not reported
**Timor-Leste**	Not reported	8.2% (2.8 to 15.6)	Not reported	-2.2% (-8.8 to 3.9)	10.1% (1.4 to 23.0)	Not reported	4.4% (-1.2 to 9.6)	13.1% (-1.0 to 23.4)	Not reported	8.6% (-0.9 to 16.3)	Not reported	0.3% (-0.5 to 1.6)	Not reported	Not reported
**Togo**	Not reported	7.9% (4.7 to 12.5)	Not reported	-0.8% (-3.3 to 1.4)	3.3% (0.0 to 11.9)	Not reported	0.2% (0.0 to 0.4)	15.9% (-1.3 to 28.6)	Not reported	10.4% (-1.1 to 19.8)	Not reported	4.2% (0.0 to 10.0)	Not reported	Not reported
**Tokelau**	Not reported	1.8% (0.0 to 4.7)	Not reported	-6.1% (-27.0 to 9.0)	4.0% (0.3 to 11.2)	Not reported	0.3% (-0.1 to 0.7)	7.3% (-0.6 to 13.3)	Not reported	5.4% (-0.7 to 10.7)	Not reported	12.2% (-0.6 to 24.6)	Not reported	Not reported
**Tonga**	Not reported	6.2% (2.1 to 13.6)	Not reported	-6.3% (-26.3 to 9.4)	5.6% (0.4 to 14.8)	Not reported	0.5% (-0.1 to 1.1)	8.4% (-0.7 to 15.2)	Not reported	7.2% (-0.8 to 13.3)	Not reported	16.7% (-0.9 to 32.2)	Not reported	Not reported
**Trinidad and Tobago**	Not reported	16.3% (5.6 to 31.6)	Not reported	-3.7% (-15.5 to 6.2)	3.5% (0.0 to 12.1)	Not reported	4.6% (-1.4 to 9.7)	9.3% (-0.7 to 16.7)	Not reported	3.8% (-0.5 to 7.3)	Not reported	10.9% (-0.4 to 23.2)	Not reported	Not reported
**Tunisia**	Not reported	16.3% (11.3 to 22.4)	Not reported	-2.7% (-11.0 to 4.9)	1.5% (0.0 to 7.3)	Not reported	0.6% (-0.2 to 1.3)	5.6% (-0.4 to 10.3)	Not reported	0.0% (0.0 to 0.0)	Not reported	8.9% (-0.3 to 19.4)	Not reported	Not reported
**Turkmenistan**	Not reported	14.2% (8.0 to 21.7)	Not reported	-17.0% (-68.7 to 21.9)	6.3% (0.3 to 17.4)	Not reported	4.6% (-1.4 to 9.8)	7.5% (-0.6 to 13.7)	Not reported	0.0% (0.0 to 0.0)	Not reported	7.1% (-0.1 to 16.4)	Not reported	Not reported
**Tuvalu**	Not reported	1.6% (0.7 to 3.1)	Not reported	-6.4% (-25.9 to 10.0)	5.0% (0.4 to 14.0)	Not reported	0.7% (-0.2 to 1.9)	9.5% (-0.7 to 17.0)	Not reported	8.5% (-1.0 to 16.2)	Not reported	14.7% (-0.7 to 29.6)	Not reported	Not reported
**Uganda**	Not reported	5.9% (3.3 to 9.2)	Not reported	-2.2% (-9.5 to 3.8)	5.3% (0.1 to 14.9)	Not reported	0.4% (-0.1 to 0.8)	4.1% (-0.3 to 7.8)	Not reported	8.9% (-0.9 to 17.0)	Not reported	2.1% (-0.1 to 5.4)	Not reported	Not reported
**Ukraine**	Not reported	10.7% (6.3 to 16.7)	Not reported	-9.7% (-40.4 to 13.6)	3.4% (0.0 to 12.5)	Not reported	2.9% (-0.9 to 6.3)	10.0% (-0.8 to 18.3)	Not reported	0.0% (0.0 to 0.0)	Not reported	9.0% (-0.3 to 20.1)	Not reported	Not reported
**United Arab Emirates**	Not reported	26.7% (19.8 to 34.0)	Not reported	-4.7% (-19.3 to 7.9)	1.6% (0.0 to 7.8)	Not reported	1.1% (-0.3 to 2.5)	4.6% (-0.3 to 8.9)	Not reported	0.0% (0.0 to 0.0)	Not reported	11.4% (-0.6 to 23.7)	Not reported	Not reported
**United Kingdom**	Not reported	5.6% (3.7 to 7.8)	Not reported	-10.5% (-43.2 to 14.8)	2.8% (0.0 to 10.2)	Not reported	4.3% (-1.3 to 9.2)	8.5% (-0.6 to 15.8)	Not reported	0.2% (0.0 to 0.5)	Not reported	9.0% (-0.3 to 19.9)	Not reported	Not reported
**United Republic of Tanzania**	Not reported	4.7% (2.5 to 8.0)	Not reported	-1.5% (-6.1 to 2.8)	7.8% (0.6 to 19.9)	Not reported	0.5% (-0.2 to 1.2)	7.5% (-0.6 to 13.8)	Not reported	4.7% (-0.5 to 9.4)	Not reported	4.3% (0.0 to 10.0)	Not reported	Not reported
**United States of America**	Not reported	3.1% (1.6 to 4.9)	Not reported	-14.3% (-59.1 to 19.0)	4.8% (0.1 to 14.6)	Not reported	3.4% (-1.0 to 7.6)	7.5% (-0.6 to 13.5)	Not reported	0.4% (0.0 to 0.8)	Not reported	12.3% (-0.5 to 25.5)	Not reported	Not reported
**United States Virgin Islands**	Not reported	3.6% (1.6 to 5.9)	Not reported	-7.0% (-29.8 to 10.1)	3.2% (0.0 to 11.8)	Not reported	3.2% (-1.0 to 7.0)	5.6% (-0.4 to 10.2)	Not reported	0.4% (0.0 to 0.9)	Not reported	12.8% (-0.6 to 27.3)	Not reported	Not reported

**Uruguay**	Not reported	6.0% (2.3 to 10.7)	Not reported	-17.0% (-70.6 to 22.0)	6.2% (0.2 to 17.6)	Not reported	4.0% (-1.2 to 8.4)	7.7% (-0.6 to 14.0)	Not reported	1.0% (-0.1 to 2.2)	Not reported	9.4% (-0.3 to 20.5)	Not reported	Not reported
**Uzbekistan**	Not reported	21.7% (13.4 to 30.2)	Not reported	-10.7% (-43.6 to 14.9)	6.3% (0.3 to 17.6)	Not reported	2.7% (-0.8 to 5.8)	7.0% (-0.5 to 13.0)	Not reported	0.0% (0.0 to 0.0)	Not reported	7.2% (-0.2 to 16.9)	Not reported	Not reported
**Vanuatu**	Not reported	3.2% (1.1 to 7.0)	Not reported	-11.2% (-45.4 to 15.2)	5.2% (0.4 to 14.3)	Not reported	0.3% (-0.1 to 0.7)	8.3% (-0.6 to 14.6)	Not reported	6.9% (-0.7 to 12.9)	Not reported	7.5% (-0.1 to 16.9)	Not reported	Not reported
**Venezuela (Bolivarian Republic of)**	Not reported	9.5% (5.4 to 14.4)	Not reported	-8.7% (-36.6 to 13.2)	6.7% (0.5 to 17.8)	Not reported	6.0% (-1.9 to 12.7)	7.6% (-0.6 to 13.8)	Not reported	1.1% (-0.1 to 2.1)	Not reported	11.2% (-0.5 to 23.9)	Not reported	Not reported
**Viet Nam**	Not reported	11.3% (5.5 to 16.9)	Not reported	-9.7% (-40.4 to 13.4)	10.8% (1.7 to 23.3)	Not reported	9.7% (-3.1 to 19.9)	7.9% (-0.6 to 14.6)	Not reported	0.0% (0.0 to 0.0)	Not reported	0.6% (-0.4 to 2.3)	Not reported	Not reported
**Yemen**	Not reported	15.3% (8.6 to 23.4)	Not reported	-1.4% (-5.8 to 2.5)	1.5% (0.0 to 7.4)	Not reported	5.2% (-1.5 to 11.0)	8.8% (-0.7 to 16.3)	Not reported	4.6% (-0.5 to 9.1)	Not reported	5.2% (0.0 to 11.5)	Not reported	Not reported
**Zambia**	Not reported	7.4% (3.5 to 13.2)	Not reported	-2.3% (-9.3 to 3.9)	5.4% (0.2 to 15.4)	Not reported	0.8% (-0.2 to 1.7)	15.9% (-1.3 to 28.4)	Not reported	9.2% (-0.9 to 17.5)	Not reported	4.3% (0.0 to 9.9)	Not reported	Not reported
**Zimbabwe**	Not reported	4.3% (2.3 to 6.9)	Not reported	-2.3% (-9.5 to 4.4)	3.9% (0.0 to 13.7)	Not reported	1.3% (-0.4 to 2.9)	15.0% (-1.3 to 27.0)	Not reported	12.4% (-1.2 to 22.9)	Not reported	5.5% (0.0 to 12.1)	Not reported	Not reported

Appendix Table 14 continues. Age-standardized percent of DALYs (with 95% uncertainty intervals) due to subarachnoid haemorrhage associated with individual risk factors by 7 GBD super-region, 21 GBD region and country for both sexes combined in 2021.

Country/region	High systolic blood pressure	High temperature	Household air pollution from solid fuels	Kidney dysfunction	Lead exposure	Low physical activity	Low temperature	Secondhand smoke	Smoking
**GBD super-regions in alphabetical order**									
**Central Europe, Eastern Europe, and Central Asia**	59.4% (44.7 to 70.3)	0.1% (-0.5 to 0.6)	1.2% (0.4 to 4.2)	Not reported	3.1% (-0.4 to 7.1)	Not reported	8.5% (7.1 to 9.7)	4.8% (3.3 to 6.4)	21.6% (19.0 to 24.1)
**High-income**	48.2% (34.9 to 59.0)	0.2% (-0.3 to 0.9)	0.0% (0.0 to 0.2)	Not reported	2.8% (-0.3 to 6.4)	Not reported	6.7% (6.0 to 7.5)	3.5% (2.4 to 4.7)	17.3% (15.1 to 19.7)
**Latin America and Caribbean**	48.6% (35.4 to 59.6)	0.4% (0.2 to 0.7)	4.0% (1.9 to 8.2)	Not reported	5.9% (-0.8 to 13.3)	Not reported	2.8% (2.4 to 3.2)	3.3% (2.2 to 4.4)	9.7% (8.3 to 11.2)
**North Africa and Middle East**	50.2% (37.1 to 60.3)	2.5% (0.3 to 5.4)	3.9% (2.3 to 6.1)	Not reported	7.3% (-1.0 to 16.0)	Not reported	5.1% (4.3 to 6.0)	5.1% (3.5 to 6.8)	12.3% (10.2 to 14.1)
**South Asia**	51.2% (37.4 to 62.3)	2.8% (0.7 to 5.6)	23.7% (15.7 to 32.9)	Not reported	9.2% (-1.2 to 20.5)	Not reported	2.4% (1.0 to 4.1)	4.8% (3.3 to 6.4)	11.3% (8.5 to 14.4)
**Southeast Asia, East Asia, and Oceania**	52.2% (37.7 to 63.5)	0.7% (0.1 to 1.7)	8.3% (2.9 to 19.5)	Not reported	6.5% (-0.8 to 14.7)	Not reported	4.7% (3.9 to 5.5)	5.8% (4.0 to 7.6)	16.8% (13.9 to 19.7)
**Sub-Saharan Africa**	54.0% (40.0 to 64.8)	1.3% (0.8 to 2.1)	32.8% (25.7 to 40.5)	Not reported	6.8% (-0.9 to 15.1)	Not reported	1.7% (1.4 to 2.0)	2.8% (1.9 to 3.8)	6.8% (4.9 to 9.2)
**GBD regions in alphabetical order**									
**Andean Latin America**	39.0% (26.7 to 50.1)	0.1% (0.0 to 0.3)	3.4% (0.7 to 10.5)	Not reported	5.1% (-0.6 to 11.7)	Not reported	5.5% (4.8 to 6.5)	2.2% (1.5 to 3.0)	6.5% (5.4 to 7.8)
**Australasia**	49.6% (35.3 to 62.4)	0.1% (-0.1 to 0.4)	0.0% (0.0 to 0.0)	Not reported	4.6% (-0.6 to 10.7)	Not reported	5.8% (5.0 to 6.8)	3.3% (2.2 to 4.4)	14.8% (12.6 to 17.2)
**Caribbean**	44.7% (32.0 to 55.0)	0.4% (0.3 to 0.5)	15.8% (10.3 to 22.2)	Not reported	7.0% (-1.0 to 15.2)	Not reported	0.3% (0.2 to 0.4)	2.5% (1.6 to 3.3)	8.3% (6.9 to 10.2)
**Central Asia**	60.2% (45.5 to 71.5)	0.5% (-0.4 to 1.7)	4.8% (1.9 to 10.8)	Not reported	3.9% (-0.5 to 8.9)	Not reported	8.1% (6.9 to 9.2)	5.1% (3.5 to 6.7)	16.2% (14.0 to 18.3)
**Central Europe**	58.3% (44.7 to 69.3)	0.1% (-0.3 to 0.8)	1.0% (0.0 to 6.8)	Not reported	3.5% (-0.4 to 8.0)	Not reported	8.5% (7.6 to 10.3)	5.3% (3.7 to 7.1)	21.9% (19.2 to 24.6)
**Central Latin America**	49.5% (36.1 to 60.5)	0.5% (0.2 to 0.8)	3.5% (1.4 to 8.4)	Not reported	6.6% (-0.9 to 14.9)	Not reported	3.3% (2.8 to 3.9)	3.1% (2.2 to 4.2)	7.6% (6.4 to 8.8)
**Central Sub-Saharan Africa**	53.7% (39.5 to 65.5)	0.3% (0.0 to 0.7)	34.6% (27.4 to 42.0)	Not reported	6.7% (-0.9 to 15.3)	Not reported	0.9% (0.7 to 1.2)	2.5% (1.6 to 3.4)	6.2% (4.0 to 8.7)
**East Asia**	52.4% (38.1 to 64.0)	0.5% (-0.3 to 1.8)	6.9% (1.9 to 19.2)	Not reported	7.5% (-1.0 to 16.9)	Not reported	7.0% (6.3 to 8.0)	5.9% (4.1 to 7.8)	18.2% (14.7 to 21.6)
**Eastern Europe**	59.6% (44.5 to 70.6)	-0.1% (-0.5 to 0.3)	0.3% (0.0 to 1.5)	Not reported	2.7% (-0.3 to 6.3)	Not reported	8.6% (6.9 to 10.3)	4.5% (3.0 to 6.1)	22.8% (20.0 to 25.6)

**Eastern Sub-Saharan Africa**	52.3% (38.2 to 63.7)	0.5% (0.1 to 1.1)	40.5% (33.8 to 47.2)	Not reported	7.2% (-1.0 to 15.8)	Not reported	2.4% (2.0 to 2.9)	2.7% (1.8 to 3.6)	7.3% (5.2 to 10.1)
**High-income Asia Pacific**	49.8% (35.9 to 60.6)	0.1% (-0.2 to 0.7)	0.0% (0.0 to 0.0)	Not reported	2.5% (-0.3 to 5.8)	Not reported	6.4% (5.7 to 7.1)	4.0% (2.7 to 5.3)	16.6% (14.2 to 18.9)
**High-income North America**	43.4% (30.1 to 54.6)	0.4% (-0.5 to 1.6)	0.0% (0.0 to 0.0)	Not reported	2.4% (-0.3 to 5.6)	Not reported	6.3% (5.4 to 7.0)	2.7% (1.9 to 3.7)	17.4% (15.0 to 20.1)
**North Africa and Middle East**	50.2% (37.1 to 60.3)	2.5% (0.3 to 5.4)	3.9% (2.3 to 6.1)	Not reported	7.3% (-1.0 to 16.0)	Not reported	5.1% (4.3 to 6.0)	5.1% (3.5 to 6.8)	12.3% (10.2 to 14.1)
**Oceania**	38.5% (26.6 to 49.5)	0.2% (0.1 to 0.2)	30.8% (22.8 to 38.3)	Not reported	2.2% (-0.3 to 5.1)	Not reported	2.5% (2.1 to 3.1)	5.8% (3.8 to 7.9)	13.5% (10.5 to 17.2)
**South Asia**	51.2% (37.4 to 62.3)	2.8% (0.7 to 5.6)	23.7% (15.7 to 32.9)	Not reported	9.2% (-1.2 to 20.5)	Not reported	2.4% (1.0 to 4.1)	4.8% (3.3 to 6.4)	11.3% (8.5 to 14.4)
**Southeast Asia**	54.3% (39.7 to 65.0)	1.1% (0.9 to 1.5)	10.7% (4.5 to 20.0)	Not reported	4.9% (-0.6 to 11.0)	Not reported	0.6% (0.3 to 0.8)	5.6% (3.9 to 7.4)	14.5% (12.0 to 17.5)
**Southern Latin America**	51.3% (37.6 to 63.0)	0.1% (-0.1 to 0.6)	0.3% (0.0 to 2.9)	Not reported	3.3% (-0.4 to 7.5)	Not reported	6.6% (6.1 to 7.2)	5.3% (3.6 to 7.0)	17.9% (15.4 to 20.4)
**Southern Sub-Saharan Africa**	57.8% (43.1 to 68.8)	0.2% (-0.2 to 0.9)	15.1% (10.3 to 21.3)	Not reported	5.7% (-0.7 to 12.6)	Not reported	4.7% (4.2 to 5.4)	4.9% (3.4 to 6.6)	11.3% (9.4 to 13.6)
**Tropical Latin America**	51.6% (37.9 to 63.2)	0.4% (0.2 to 0.7)	2.0% (0.4 to 5.7)	Not reported	5.3% (-0.7 to 12.2)	Not reported	2.1% (1.7 to 2.5)	3.8% (2.6 to 5.2)	12.6% (10.6 to 15.0)
**Western Europe**	52.1% (38.4 to 63.1)	0.0% (-0.1 to 0.3)	0.0% (0.0 to 0.0)	Not reported	3.3% (-0.4 to 7.5)	Not reported	7.5% (6.7 to 9.1)	3.7% (2.5 to 4.9)	18.3% (15.8 to 20.7)
**Western Sub-Saharan Africa**	54.9% (41.4 to 65.5)	2.7% (1.9 to 4.0)	29.6% (21.6 to 38.6)	Not reported	6.8% (-0.9 to 15.0)	Not reported	0.3% (0.1 to 0.7)	2.6% (1.7 to 3.4)	5.2% (3.3 to 8.0)
**Countries in alphabetical order**									
**Afghanistan**	47.3% (32.7 to 59.2)	1.0% (-0.3 to 2.8)	35.8% (29.3 to 42.4)	Not reported	12.6% (-1.8 to 27.3)	Not reported	7.3% (6.2 to 8.3)	4.4% (2.9 to 5.9)	8.0% (5.3 to 11.2)
**Albania**	60.3% (45.7 to 71.1)	0.1% (-0.2 to 0.5)	4.1% (0.3 to 15.7)	Not reported	4.6% (-0.6 to 10.4)	Not reported	7.5% (6.3 to 8.6)	6.0% (4.1 to 8.0)	19.5% (15.2 to 24.1)
**Algeria**	48.4% (34.2 to 60.5)	1.3% (0.0 to 3.0)	0.0% (0.0 to 0.2)	Not reported	6.5% (-0.9 to 14.7)	Not reported	5.9% (5.0 to 6.7)	6.4% (4.4 to 8.5)	10.2% (7.4 to 14.1)
**American Samoa**	55.0% (41.3 to 66.9)	0.1% (0.0 to 0.1)	1.0% (0.0 to 6.5)	Not reported	1.1% (-0.1 to 2.6)	Not reported	0.0% (-0.1 to 0.2)	6.1% (4.1 to 8.1)	16.6% (13.5 to 19.9)
**Andorra**	54.0% (39.0 to 66.7)	0.0% (0.0 to 0.0)	0.0% (0.0 to 0.0)	Not reported	2.6% (-0.3 to 5.9)	Not reported	9.0% (7.7 to 10.2)	4.0% (2.7 to 5.3)	18.5% (15.1 to 22.1)
**Angola**	55.4% (41.0 to 67.7)	0.2% (-0.2 to 0.7)	13.8% (4.5 to 25.3)	Not reported	6.2% (-0.8 to 14.0)	Not reported	1.4% (1.1 to 1.8)	2.6% (1.7 to 3.5)	9.9% (6.8 to 13.5)
**Antigua and Barbuda**	51.1% (35.7 to 63.0)	0.3% (0.2 to 0.5)	0.1% (0.0 to 0.4)	Not reported	4.3% (-0.5 to 9.8)	Not reported	0.0% (-0.1 to 0.1)	2.6% (1.8 to 3.5)	8.3% (6.5 to 10.1)
**Argentina**	51.3% (37.1 to 63.3)	0.2% (-0.2 to 0.8)	0.2% (0.0 to 1.5)	Not reported	3.5% (-0.5 to 7.9)	Not reported	6.0% (5.5 to 6.5)	5.4% (3.7 to 7.2)	18.3% (15.7 to 21.1)

**Armenia**	60.1%<br>(45.0 to 71.2)	0.1%<br>(-0.6 to 0.9)	0.9%<br>(0.1 to 3.7)	Not reported	3.8%<br>(-0.5 to 8.8)	Not reported	7.3%<br>(6.1 to 8.3)	6.0%<br>(4.1 to 8.0)	23.8%<br>(21.2 to 26.4)
**Australia**	49.9%<br>(35.5 to 62.7)	0.2%<br>(-0.1 to 0.5)	0.0%<br>(0.0 to 0.0)	Not reported	4.5%<br>(-0.6 to 10.6)	Not reported	5.4%<br>(4.7 to 6.3)	3.1%<br>(2.1 to 4.2)	14.3%<br>(12.1 to 16.8)
**Austria**	56.1%<br>(41.7 to 68.6)	0.0%<br>(-0.2 to 0.3)	0.0%<br>(0.0 to 0.0)	Not reported	2.9%<br>(-0.4 to 6.7)	Not reported	7.1%<br>(6.2 to 8.0)	4.3%<br>(3.0 to 5.7)	21.0%<br>(18.3 to 23.9)
**Azerbaijan**	57.8%<br>(42.4 to 69.8)	0.2%<br>(-0.4 to 0.8)	0.7%<br>(0.0 to 4.6)	Not reported	3.4%<br>(-0.4 to 7.8)	Not reported	6.1%<br>(4.7 to 7.2)	6.6%<br>(4.5 to 8.8)	17.3%<br>(12.4 to 22.7)
**Bahamas**	50.8%<br>(36.9 to 63.1)	0.7%<br>(-0.2 to 1.8)	0.0%<br>(0.0 to 0.3)	Not reported	2.7%<br>(-0.3 to 6.4)	Not reported	0.4%<br>(0.1 to 0.7)	2.8%<br>(1.9 to 3.8)	8.8%<br>(7.0 to 10.9)
**Bahrain**	52.3%<br>(39.2 to 63.6)	5.6%<br>(1.9 to 9.9)	0.0%<br>(0.0 to 0.0)	Not reported	4.6%<br>(-0.6 to 10.5)	Not reported	2.1%<br>(0.6 to 3.7)	4.5%<br>(3.0 to 6.1)	11.5%<br>(9.1 to 14.1)
**Bangladesh**	51.5%<br>(37.6 to 63.1)	2.2%<br>(0.2 to 4.7)	35.4%<br>(27.5 to 43.0)	Not reported	9.5%<br>(-1.3 to 20.6)	Not reported	1.7%<br>(0.6 to 3.0)	5.7%<br>(3.8 to 7.8)	16.5%<br>(11.8 to 21.6)
**Barbados**	53.9%<br>(38.5 to 66.0)	0.4%<br>(0.2 to 0.6)	0.0%<br>(0.0 to 0.1)	Not reported	3.6%<br>(-0.5 to 8.1)	Not reported	0.0%<br>(0.0 to 0.0)	1.8%<br>(1.2 to 2.5)	6.9%<br>(5.6 to 8.6)
**Belarus**	65.0%<br>(49.5 to 76.3)	-0.2%<br>(-0.6 to 0.0)	0.1%<br>(0.0 to 0.5)	Not reported	3.4%<br>(-0.4 to 7.8)	Not reported	9.2%<br>(7.1 to 10.5)	5.0%<br>(3.4 to 6.8)	25.3%<br>(22.3 to 28.6)
**Belgium**	58.9%<br>(43.3 to 70.3)	0.0%<br>(-0.1 to 0.1)	0.0%<br>(0.0 to 0.0)	Not reported	5.9%<br>(-0.8 to 13.5)	Not reported	7.7%<br>(6.7 to 9.8)	3.4%<br>(2.3 to 4.5)	20.0%<br>(17.1 to 22.9)
**Belize**	47.0%<br>(33.0 to 60.4)	1.0%<br>(0.6 to 1.5)	4.3%<br>(0.7 to 11.6)	Not reported	4.1%<br>(-0.5 to 9.5)	Not reported	0.3%<br>(0.1 to 0.4)	3.1%<br>(2.1 to 4.4)	9.3%<br>(7.5 to 11.4)
**Benin**	57.2%<br>(42.4 to 68.4)	2.4%<br>(1.4 to 3.4)	37.3%<br>(29.8 to 44.7)	Not reported	6.2%<br>(-0.8 to 14.0)	Not reported	0.1%<br>(0.0 to 0.2)	1.9%<br>(1.3 to 2.6)	4.0%<br>(2.5 to 6.5)
**Bermuda**	48.2%<br>(33.9 to 60.9)	0.3%<br>(-0.3 to 1.3)	0.0%<br>(0.0 to 0.0)	Not reported	2.4%<br>(-0.3 to 5.6)	Not reported	1.8%<br>(1.5 to 2.2)	3.0%<br>(2.0 to 4.1)	13.3%<br>(10.9 to 15.9)
**Bhutan**	50.6%<br>(35.7 to 61.9)	0.1%<br>(0.0 to 0.2)	6.1%<br>(1.2 to 16.0)	Not reported	10.6%<br>(-1.4 to 23.4)	Not reported	7.7%<br>(6.9 to 8.9)	3.9%<br>(2.6 to 5.3)	6.9%<br>(5.1 to 9.3)
**Bolivia (Plurinational State of)**	36.5%<br>(24.3 to 48.7)	0.3%<br>(-0.1 to 0.8)	7.7%<br>(2.4 to 15.8)	Not reported	6.5%<br>(-0.8 to 14.7)	Not reported	6.6%<br>(6.1 to 7.7)	2.7%<br>(1.6 to 3.8)	6.1%<br>(4.8 to 7.6)
**Bosnia and Herzegovina**	59.9%<br>(45.0 to 71.4)	0.1%<br>(-0.3 to 0.8)	6.5%<br>(0.5 to 23.2)	Not reported	5.1%<br>(-0.7 to 11.7)	Not reported	8.2%<br>(7.3 to 10.1)	5.5%<br>(3.7 to 7.4)	24.5%<br>(21.0 to 27.9)
**Botswana**	59.6%<br>(45.1 to 70.4)	0.5%<br>(-0.4 to 1.9)	3.3%<br>(0.1 to 20.1)	Not reported	6.8%<br>(-0.9 to 15.1)	Not reported	2.8%<br>(2.2 to 3.5)	4.7%<br>(3.2 to 6.4)	13.1%<br>(10.0 to 16.7)
**Brazil**	51.6%<br>(37.9 to 63.1)	0.4%<br>(0.2 to 0.7)	1.9%<br>(0.4 to 5.3)	Not reported	5.3%<br>(-0.7 to 12.2)	Not reported	2.1%<br>(1.7 to 2.5)	3.8%<br>(2.6 to 5.2)	12.6%<br>(10.6 to 15.0)
**Brunei Darussalam**	53.6%<br>(37.4 to 66.2)	0.6%<br>(0.4 to 0.9)	0.0%<br>(0.0 to 0.0)	Not reported	3.5%<br>(-0.5 to 8.1)	Not reported	0.0%<br>(0.0 to 0.1)	3.8%<br>(2.6 to 5.2)	13.9%<br>(11.1 to 17.3)
**Bulgaria**	58.0%<br>(43.6 to 68.9)	0.2%<br>(-0.4 to 1.2)	1.0%<br>(0.0 to 8.3)	Not reported	3.3%<br>(-0.4 to 7.7)	Not reported	8.5%<br>(7.6 to 10.6)	5.0%<br>(3.3 to 6.9)	23.6%<br>(20.7 to 26.3)
**Burkina Faso**	48.8%<br>(34.8 to 61.1)	5.4%<br>(3.6 to 7.6)	37.2%<br>(30.1 to 44.6)	Not reported	10.3%<br>(-1.3 to 22.7)	Not reported	0.2%<br>(0.0 to 0.5)	3.3%<br>(2.2 to 4.6)	6.1%<br>(3.8 to 9.2)

**Burundi**	53.2%<br>(38.0 to 64.9)	0.0%<br>(0.0 to 0.0)	43.3%<br>(36.5 to 50.3)	Not reported	7.7%<br>(-1.1 to 17.0)	Not reported	2.3%<br>(1.6 to 3.3)	1.9%<br>(1.2 to 2.9)	5.9%<br>(3.7 to 9.1)
**CV¥te d'Ivoire**	56.4%<br>(42.6 to 67.9)	0.8%<br>(0.5 to 1.0)	29.4%<br>(20.8 to 38.1)	Not reported	6.6%<br>(-0.9 to 15.1)	Not reported	0.1%<br>(0.0 to 0.2)	3.8%<br>(2.5 to 5.1)	7.5%<br>(5.1 to 10.7)
**Cabo Verde**	60.5%<br>(46.2 to 71.6)	0.0%<br>(0.0 to 0.2)	7.6%<br>(2.1 to 17.0)	Not reported	5.3%<br>(-0.7 to 12.2)	Not reported	1.9%<br>(1.4 to 2.7)	3.0%<br>(2.0 to 4.0)	5.6%<br>(3.7 to 8.2)
**Cambodia**	40.7%<br>(27.4 to 52.9)	2.2%<br>(1.9 to 2.4)	35.9%<br>(27.5 to 43.6)	Not reported	6.7%<br>(-0.8 to 15.2)	Not reported	0.4%<br>(0.1 to 1.0)	5.8%<br>(4.0 to 7.9)	15.5%<br>(11.9 to 19.8)
**Cameroon**	57.9%<br>(43.9 to 68.7)	1.6%<br>(1.0 to 2.4)	30.8%<br>(22.4 to 38.9)	Not reported	7.5%<br>(-1.0 to 16.9)	Not reported	0.8%<br>(0.6 to 1.1)	2.3%<br>(1.5 to 3.1)	6.5%<br>(4.1 to 9.7)
**Canada**	41.3%<br>(28.5 to 53.2)	-0.1%<br>(-0.3 to 0.1)	0.0%<br>(0.0 to 0.0)	Not reported	2.3%<br>(-0.3 to 5.3)	Not reported	7.6%<br>(6.5 to 8.5)	4.0%<br>(2.7 to 5.4)	16.7%<br>(14.2 to 19.7)
**Central African Republic**	53.2%<br>(39.2 to 65.7)	1.0%<br>(0.2 to 1.6)	42.4%<br>(35.6 to 49.3)	Not reported	8.2%<br>(-1.1 to 18.7)	Not reported	0.1%<br>(0.1 to 0.3)	2.5%<br>(1.6 to 3.4)	6.6%<br>(4.0 to 9.8)
**Chad**	48.4%<br>(33.8 to 60.4)	5.0%<br>(3.1 to 7.7)	35.9%<br>(28.3 to 43.7)	Not reported	10.4%<br>(-1.5 to 22.0)	Not reported	0.6%<br>(0.1 to 1.2)	2.6%<br>(1.8 to 3.5)	5.9%<br>(3.6 to 9.5)
**Chile**	51.0%<br>(36.5 to 63.0)	0.0%<br>(0.0 to 0.0)	0.6%<br>(0.0 to 6.6)	Not reported	2.2%<br>(-0.3 to 5.1)	Not reported	8.1%<br>(7.4 to 9.3)	5.2%<br>(3.5 to 7.2)	16.9%<br>(14.6 to 19.1)
**China**	52.7%<br>(38.2 to 64.3)	0.5%<br>(-0.3 to 1.8)	5.9%<br>(0.8 to 18.9)	Not reported	7.6%<br>(-1.0 to 17.0)	Not reported	7.1%<br>(6.3 to 8.0)	5.9%<br>(4.1 to 7.8)	18.3%<br>(14.6 to 21.8)
**Colombia**	51.4%<br>(36.6 to 62.9)	0.3%<br>(0.3 to 0.4)	1.2%<br>(0.0 to 6.7)	Not reported	5.0%<br>(-0.7 to 11.5)	Not reported	4.0%<br>(3.2 to 4.8)	2.9%<br>(1.9 to 4.1)	7.4%<br>(6.1 to 8.7)
**Comoros**	54.9%<br>(40.3 to 66.4)	0.0%<br>(0.0 to 0.1)	38.6%<br>(31.4 to 45.4)	Not reported	5.6%<br>(-0.8 to 12.5)	Not reported	0.3%<br>(0.1 to 0.7)	4.3%<br>(2.8 to 6.0)	7.3%<br>(4.7 to 11.3)
**Congo**	57.3%<br>(41.6 to 68.9)	0.2%<br>(0.0 to 0.4)	21.3%<br>(9.9 to 32.6)	Not reported	4.4%<br>(-0.5 to 9.9)	Not reported	0.2%<br>(0.1 to 0.3)	3.2%<br>(2.1 to 4.4)	7.4%<br>(4.7 to 10.8)
**Cook Islands**	48.9%<br>(36.2 to 60.4)	0.2%<br>(-0.1 to 0.5)	0.1%<br>(0.0 to 1.2)	Not reported	1.0%<br>(-0.1 to 2.3)	Not reported	0.0%<br>(0.0 to 0.0)	5.2%<br>(3.5 to 7.1)	14.4%<br>(11.5 to 17.7)
**Costa Rica**	56.6%<br>(42.0 to 68.6)	0.0%<br>(0.0 to 0.1)	0.7%<br>(0.0 to 4.5)	Not reported	6.1%<br>(-0.8 to 13.9)	Not reported	2.2%<br>(1.5 to 3.0)	3.4%<br>(2.2 to 4.6)	10.2%<br>(8.6 to 11.9)
**Croatia**	61.3%<br>(46.3 to 72.8)	0.2%<br>(-0.5 to 1.3)	0.2%<br>(0.0 to 2.1)	Not reported	3.7%<br>(-0.5 to 8.4)	Not reported	7.9%<br>(6.8 to 9.3)	6.2%<br>(4.2 to 8.1)	22.9%<br>(19.8 to 26.1)
**Cuba**	43.0%<br>(28.7 to 54.9)	0.6%<br>(0.2 to 1.1)	0.6%<br>(0.1 to 2.0)	Not reported	6.9%<br>(-0.9 to 15.6)	Not reported	0.4%<br>(0.1 to 0.6)	4.2%<br>(2.8 to 5.7)	16.6%<br>(13.9 to 19.4)
**Cyprus**	55.5%<br>(40.1 to 67.9)	1.4%<br>(-0.4 to 4.3)	0.0%<br>(0.0 to 0.0)	Not reported	4.7%<br>(-0.6 to 10.8)	Not reported	4.3%<br>(3.2 to 5.4)	5.0%<br>(3.5 to 6.7)	20.2%<br>(16.8 to 23.8)
**Czechia**	56.5%<br>(41.5 to 67.7)	0.0%<br>(-0.2 to 0.1)	0.0%<br>(0.0 to 0.2)	Not reported	2.4%<br>(-0.3 to 5.6)	Not reported	8.5%<br>(7.3 to 10.1)	4.8%<br>(3.2 to 6.5)	21.1%<br>(18.3 to 24.4)
**Democratic People's Republic of Korea**	47.8%<br>(33.8 to 60.5)	0.3%<br>(-0.4 to 1.5)	39.8%<br>(32.8 to 46.5)	Not reported	6.4%<br>(-0.8 to 14.2)	Not reported	7.9%<br>(6.9 to 9.3)	6.3%<br>(4.3 to 8.5)	15.6%<br>(11.6 to 20.6)
**Democratic Republic of the Congo**	52.8%<br>(38.4 to 64.8)	0.2%<br>(-0.1 to 0.6)	42.6%<br>(35.6 to 49.4)	Not reported	6.9%<br>(-1.0 to 16.0)	Not reported	0.9%<br>(0.7 to 1.1)	2.4%<br>(1.4 to 3.4)	4.9%<br>(2.8 to 7.8)

**Denmark**	57.3% (41.8 to 68.9)	0.0% (0.0 to 0.0)	0.0% (0.0 to 0.0)	Not reported	3.2% (-0.4 to 7.4)	Not reported	8.4% (7.4 to 11.0)	2.9% (2.0 to 3.9)	20.4% (17.5 to 23.4)
**Djibouti**	56.0% (41.2 to 67.8)	6.3% (3.6 to 10.3)	11.3% (5.5 to 18.4)	Not reported	6.4% (-0.9 to 14.3)	Not reported	1.6% (0.5 to 3.4)	4.9% (2.9 to 7.0)	13.2% (9.5 to 18.2)
**Dominica**	51.7% (37.9 to 63.5)	0.2% (0.1 to 0.4)	1.6% (0.1 to 6.2)	Not reported	3.9% (-0.5 to 8.8)	Not reported	0.0% (-0.1 to 0.2)	2.7% (1.8 to 3.7)	8.0% (6.3 to 10.0)
**Dominican Republic**	45.7% (31.4 to 58.2)	0.2% (0.1 to 0.4)	0.9% (0.0 to 5.7)	Not reported	7.7% (-1.0 to 17.1)	Not reported	0.6% (0.4 to 0.9)	2.7% (1.9 to 3.7)	11.1% (9.0 to 13.7)
**Ecuador**	40.2% (27.9 to 52.2)	0.0% (0.0 to 0.1)	1.2% (0.1 to 4.8)	Not reported	3.9% (-0.5 to 9.0)	Not reported	3.8% (3.3 to 4.6)	2.3% (1.5 to 3.2)	6.8% (5.7 to 8.1)
**Egypt**	51.6% (37.9 to 62.6)	3.2% (-0.8 to 8.8)	0.1% (0.0 to 0.2)	Not reported	10.3% (-1.5 to 22.3)	Not reported	2.7% (1.6 to 3.6)	5.0% (3.4 to 6.7)	14.0% (10.2 to 18.0)
**El Salvador**	48.2% (33.6 to 60.3)	0.6% (0.4 to 0.7)	5.1% (1.1 to 12.4)	Not reported	9.3% (-1.3 to 20.4)	Not reported	0.1% (0.0 to 0.1)	3.1% (2.0 to 4.2)	7.4% (5.7 to 9.1)
**Equatorial Guinea**	57.6% (43.7 to 69.3)	0.0% (-0.1 to 0.1)	0.6% (0.0 to 7.0)	Not reported	4.9% (-0.6 to 11.3)	Not reported	0.4% (0.2 to 0.5)	4.5% (2.8 to 6.3)	6.7% (4.2 to 10.2)
**Eritrea**	48.0% (33.1 to 60.7)	2.4% (1.3 to 4.0)	34.8% (27.8 to 41.6)	Not reported	6.1% (-0.7 to 13.7)	Not reported	1.9% (1.4 to 2.4)	3.1% (2.1 to 4.2)	6.2% (3.6 to 9.8)
**Estonia**	62.6% (46.9 to 74.2)	-0.1% (-0.2 to 0.0)	0.2% (0.0 to 2.1)	Not reported	2.7% (-0.3 to 6.2)	Not reported	8.1% (7.0 to 9.2)	3.8% (2.5 to 5.2)	22.2% (19.5 to 25.0)
**Eswatini**	59.0% (44.5 to 70.4)	0.1% (-0.2 to 0.6)	14.4% (2.7 to 31.7)	Not reported	6.8% (-0.9 to 15.1)	Not reported	4.8% (4.1 to 5.5)	3.1% (2.0 to 4.4)	5.5% (4.0 to 7.1)
**Ethiopia**	44.3% (30.7 to 55.7)	0.3% (0.1 to 0.5)	39.6% (33.0 to 46.3)	Not reported	8.7% (-1.1 to 19.0)	Not reported	3.9% (3.0 to 4.8)	2.2% (1.5 to 2.9)	3.7% (2.5 to 5.7)
**Fiji**	53.7% (40.3 to 64.5)	0.1% (0.0 to 0.2)	6.9% (0.8 to 18.6)	Not reported	1.5% (-0.2 to 3.4)	Not reported	0.5% (-0.2 to 0.8)	4.7% (3.0 to 6.4)	10.7% (8.6 to 13.1)
**Finland**	59.2% (44.0 to 70.8)	-0.1% (-0.3 to 0.0)	0.0% (0.0 to 0.0)	Not reported	1.7% (-0.2 to 3.9)	Not reported	8.3% (6.7 to 9.6)	2.7% (1.9 to 3.8)	16.7% (14.2 to 19.3)
**France**	52.8% (38.3 to 65.9)	0.0% (-0.1 to 0.2)	0.0% (0.0 to 0.0)	Not reported	3.4% (-0.4 to 7.7)	Not reported	7.3% (6.5 to 9.0)	4.2% (2.9 to 5.7)	20.4% (17.4 to 23.2)
**Gabon**	57.3% (42.7 to 69.9)	0.1% (0.0 to 0.1)	1.0% (0.0 to 7.0)	Not reported	4.0% (-0.5 to 9.0)	Not reported	0.2% (0.1 to 0.4)	3.5% (2.2 to 4.8)	7.4% (5.2 to 10.3)
**Gambia**	58.3% (43.6 to 69.4)	1.9% (1.2 to 2.9)	36.5% (29.2 to 43.6)	Not reported	7.6% (-1.0 to 17.4)	Not reported	0.2% (0.0 to 0.4)	4.3% (2.9 to 5.9)	7.5% (4.6 to 11.9)
**Georgia**	62.7% (47.0 to 74.4)	0.2% (-0.3 to 1.1)	4.7% (0.4 to 15.9)	Not reported	6.3% (-0.8 to 14.4)	Not reported	8.3% (7.5 to 10.4)	6.2% (4.2 to 8.1)	23.9% (20.9 to 26.9)
**Germany**	55.1% (40.3 to 66.9)	0.0% (-0.1 to 0.2)	0.0% (0.0 to 0.0)	Not reported	2.3% (-0.3 to 5.2)	Not reported	7.7% (6.8 to 9.7)	3.6% (2.4 to 4.9)	19.6% (16.7 to 22.5)
**Ghana**	55.2% (40.9 to 66.7)	1.9% (1.6 to 2.4)	26.1% (14.6 to 36.9)	Not reported	4.6% (-0.6 to 10.3)	Not reported	0.1% (0.0 to 0.2)	2.1% (1.4 to 3.0)	5.8% (3.8 to 8.4)
**Greece**	48.0% (33.4 to 60.7)	0.3% (-0.2 to 1.0)	0.0% (0.0 to 0.2)	Not reported	4.7% (-0.6 to 10.6)	Not reported	6.7% (6.0 to 7.3)	4.8% (3.3 to 6.4)	25.2% (21.9 to 28.8)

**Greenland**	41.8% (27.5 to 53.4)	-0.2% (-0.5 to 0.0)	0.0% (0.0 to 0.0)	Not reported	2.1% (-0.3 to 5.0)	Not reported	8.2% (5.0 to 12.4)	5.8% (3.9 to 7.8)	26.8% (22.6 to 30.9)
**Grenada**	52.2% (36.9 to 64.9)	0.4% (0.3 to 0.6)	0.3% (0.0 to 1.8)	Not reported	6.1% (-0.8 to 14.1)	Not reported	0.0% (0.0 to 0.0)	2.7% (1.8 to 3.8)	7.9% (6.5 to 9.5)
**Guam**	49.0% (35.1 to 60.4)	0.7% (0.3 to 1.2)	0.0% (0.0 to 0.2)	Not reported	1.0% (-0.1 to 2.2)	Not reported	0.0% (0.0 to 0.0)	5.0% (3.4 to 6.8)	16.1% (13.4 to 19.5)
**Guatemala**	51.0% (37.8 to 61.4)	0.2% (0.1 to 0.3)	16.9% (4.8 to 30.7)	Not reported	10.3% (-1.4 to 22.7)	Not reported	2.8% (1.6 to 3.8)	2.9% (1.8 to 4.1)	6.1% (4.8 to 7.4)
**Guinea**	51.6% (37.7 to 63.7)	1.1% (0.6 to 1.8)	36.7% (30.0 to 44.0)	Not reported	8.4% (-1.1 to 18.7)	Not reported	0.2% (0.1 to 0.4)	3.5% (2.4 to 4.8)	6.5% (4.0 to 10.2)
**Guinea-Bissau**	54.4% (40.1 to 66.1)	2.1% (1.5 to 2.6)	38.0% (30.7 to 45.0)	Not reported	7.8% (-1.0 to 17.6)	Not reported	0.1% (0.0 to 0.3)	2.7% (1.8 to 3.7)	5.2% (3.1 to 8.1)
**Guyana**	49.0% (35.8 to 61.1)	0.3% (0.0 to 0.6)	2.3% (0.4 to 6.3)	Not reported	6.4% (-0.9 to 14.2)	Not reported	0.1% (0.0 to 0.2)	3.2% (2.1 to 4.3)	10.3% (8.4 to 12.5)
**Haiti**	51.3% (37.9 to 62.0)	0.4% (0.3 to 0.5)	36.4% (28.8 to 43.4)	Not reported	9.9% (-1.4 to 21.5)	Not reported	0.2% (0.1 to 0.4)	1.8% (1.1 to 2.6)	4.5% (3.1 to 6.2)
**Honduras**	51.7% (36.4 to 64.8)	0.4% (0.1 to 0.8)	24.9% (15.9 to 33.4)	Not reported	11.3% (-1.6 to 24.3)	Not reported	1.1% (0.5 to 1.7)	4.5% (3.1 to 6.1)	8.8% (6.7 to 11.4)
**Hungary**	62.7% (48.8 to 74.7)	0.2% (-0.7 to 1.4)	0.5% (0.0 to 6.3)	Not reported	3.6% (-0.5 to 8.3)	Not reported	7.5% (6.4 to 8.9)	5.0% (3.4 to 6.7)	22.8% (19.6 to 26.3)
**Iceland**	50.4% (35.9 to 61.9)	0.0% (0.0 to 0.0)	0.0% (0.0 to 0.0)	Not reported	2.8% (-0.3 to 6.2)	Not reported	9.7% (8.4 to 10.8)	2.9% (2.0 to 4.0)	15.7% (13.2 to 18.4)
**India**	51.3% (37.3 to 62.8)	2.5% (0.8 to 4.8)	21.4% (13.5 to 31.2)	Not reported	9.3% (-1.2 to 20.8)	Not reported	2.1% (0.8 to 3.7)	4.5% (3.1 to 6.1)	10.4% (7.7 to 13.0)
**Indonesia**	60.5% (45.2 to 71.1)	0.4% (0.3 to 0.5)	8.8% (3.0 to 18.4)	Not reported	5.8% (-0.8 to 12.9)	Not reported	0.4% (0.3 to 0.7)	6.3% (4.2 to 8.4)	14.1% (10.0 to 18.9)
**Iran (Islamic Republic of)**	48.8% (36.3 to 59.5)	1.4% (-0.4 to 3.6)	0.0% (0.0 to 0.2)	Not reported	10.7% (-1.5 to 23.0)	Not reported	5.7% (4.7 to 6.6)	3.6% (2.4 to 4.8)	10.6% (8.7 to 12.8)
**Iraq**	58.5% (44.2 to 69.1)	8.6% (0.7 to 17.7)	0.1% (0.0 to 0.9)	Not reported	6.2% (-0.8 to 14.3)	Not reported	5.4% (2.4 to 9.1)	6.4% (4.4 to 8.5)	14.7% (12.0 to 17.8)
**Ireland**	54.1% (38.9 to 65.9)	0.0% (0.0 to 0.0)	0.0% (0.0 to 0.0)	Not reported	3.5% (-0.4 to 8.1)	Not reported	7.2% (5.9 to 9.8)	3.4% (2.3 to 4.6)	15.7% (13.4 to 18.0)
**Israel**	55.9% (41.1 to 67.3)	1.2% (-0.5 to 4.3)	0.0% (0.0 to 0.0)	Not reported	2.8% (-0.4 to 6.5)	Not reported	2.6% (1.7 to 3.3)	3.7% (2.5 to 4.9)	15.5% (13.2 to 17.8)
**Italy**	46.7% (33.1 to 57.9)	0.1% (-0.3 to 0.6)	0.0% (0.0 to 0.1)	Not reported	3.5% (-0.4 to 8.1)	Not reported	7.3% (6.5 to 8.3)	4.1% (2.8 to 5.5)	15.5% (13.3 to 18.1)
**Jamaica**	52.1% (37.9 to 64.2)	0.4% (0.1 to 0.9)	3.6% (0.6 to 9.8)	Not reported	5.8% (-0.7 to 13.5)	Not reported	0.1% (0.0 to 0.2)	3.1% (2.1 to 4.2)	10.2% (8.4 to 12.3)
**Japan**	52.6% (38.6 to 63.1)	0.1% (-0.2 to 0.4)	0.0% (0.0 to 0.0)	Not reported	2.1% (-0.3 to 5.0)	Not reported	6.5% (5.7 to 7.2)	3.9% (2.6 to 5.3)	17.5% (15.0 to 19.9)
**Jordan**	44.7% (31.4 to 55.2)	0.6% (-0.4 to 2.4)	0.0% (0.0 to 0.1)	Not reported	4.7% (-0.6 to 10.6)	Not reported	3.0% (2.3 to 3.9)	5.8% (4.0 to 7.6)	16.7% (14.4 to 19.1)

**Kazakhstan**	65.6%<br>(50.2 to 76.8)	0.2%<br>(-0.5 to 1.2)	0.3%<br>(0.0 to 2.9)	Not reported	2.7%<br>(-0.4 to 6.2)	Not reported	8.5%<br>(7.3 to 9.8)	4.8%<br>(3.3 to 6.4)	16.2%<br>(13.7 to 18.5)
**Kenya**	54.6%<br>(41.1 to 65.5)	0.2%<br>(0.1 to 0.2)	35.4%<br>(27.9 to 42.3)	Not reported	5.3%<br>(-0.7 to 11.9)	Not reported	3.2%<br>(2.5 to 4.0)	2.5%<br>(1.6 to 3.3)	7.1%<br>(5.0 to 9.9)
**Kiribati**	40.8%<br>(27.3 to 52.2)	0.2%<br>(0.0 to 0.4)	25.8%<br>(20.5 to 32.2)	Not reported	2.0%<br>(-0.3 to 4.7)	Not reported	0.0%<br>(-0.1 to 0.1)	8.4%<br>(5.7 to 11.2)	25.8%<br>(21.9 to 29.8)
**Kuwait**	42.4%<br>(30.5 to 52.7)	7.0%<br>(1.5 to 12.7)	0.0%<br>(0.0 to 0.0)	Not reported	3.5%<br>(-0.4 to 8.0)	Not reported	2.9%<br>(0.7 to 5.5)	5.2%<br>(3.5 to 6.8)	12.9%<br>(10.9 to 15.1)
**Kyrgyzstan**	49.9%<br>(35.0 to 63.4)	0.0%<br>(-0.5 to 0.6)	16.3%<br>(9.6 to 23.8)	Not reported	4.1%<br>(-0.5 to 9.5)	Not reported	8.8%<br>(7.2 to 10.1)	6.2%<br>(4.3 to 8.3)	24.4%<br>(21.2 to 27.5)
**Lao People's Democratic Republic**	49.4%<br>(35.1 to 61.4)	0.9%<br>(0.2 to 2.0)	28.4%<br>(12.5 to 42.0)	Not reported	7.0%<br>(-0.9 to 15.6)	Not reported	1.5%<br>(1.0 to 2.0)	6.4%<br>(4.3 to 8.6)	15.2%<br>(11.4 to 19.4)
**Latvia**	64.3%<br>(49.5 to 75.9)	-0.1%<br>(-0.3 to 0.0)	0.5%<br>(0.0 to 4.8)	Not reported	2.8%<br>(-0.4 to 6.4)	Not reported	9.2%<br>(7.7 to 10.4)	4.8%<br>(3.3 to 6.4)	21.3%<br>(18.3 to 24.2)
**Lebanon**	53.7%<br>(39.4 to 64.4)	0.2%<br>(-0.3 to 1.0)	0.1%<br>(0.0 to 0.3)	Not reported	4.0%<br>(-0.5 to 9.1)	Not reported	5.0%<br>(4.3 to 5.9)	5.9%<br>(4.1 to 7.9)	22.7%<br>(19.1 to 26.3)
**Lesotho**	54.9%<br>(40.2 to 66.3)	0.0%<br>(0.0 to 0.0)	31.1%<br>(23.8 to 38.1)	Not reported	8.6%<br>(-1.1 to 19.2)	Not reported	8.0%<br>(7.0 to 10.3)	5.8%<br>(4.0 to 7.7)	15.2%<br>(11.4 to 19.8)
**Liberia**	56.3%<br>(41.8 to 67.8)	0.2%<br>(0.1 to 0.4)	39.2%<br>(32.9 to 45.8)	Not reported	7.7%<br>(-1.1 to 17.1)	Not reported	0.1%<br>(0.0 to 0.2)	2.4%<br>(1.5 to 3.3)	5.3%<br>(3.0 to 9.0)
**Libya**	46.1%<br>(34.1 to 56.7)	2.7%<br>(-0.3 to 7.5)	0.1%<br>(0.0 to 0.4)	Not reported	4.3%<br>(-0.6 to 9.8)	Not reported	3.4%<br>(2.5 to 4.2)	6.3%<br>(4.2 to 8.5)	7.6%<br>(5.4 to 10.7)
**Lithuania**	66.7%<br>(51.0 to 77.6)	-0.1%<br>(-0.4 to 0.0)	0.1%<br>(0.0 to 0.8)	Not reported	2.7%<br>(-0.4 to 6.2)	Not reported	9.2%<br>(7.5 to 10.4)	4.2%<br>(2.8 to 5.7)	20.8%<br>(18.0 to 23.8)
**Luxembourg**	56.9%<br>(42.9 to 68.7)	0.0%<br>(-0.1 to 0.1)	0.0%<br>(0.0 to 0.0)	Not reported	2.8%<br>(-0.4 to 6.6)	Not reported	7.6%<br>(6.6 to 10.2)	3.5%<br>(2.4 to 4.7)	17.4%<br>(14.4 to 20.6)
**Madagascar**	51.1%<br>(36.7 to 63.1)	0.3%<br>(-0.1 to 0.8)	43.4%<br>(36.2 to 49.9)	Not reported	5.6%<br>(-0.7 to 12.6)	Not reported	3.2%<br>(2.6 to 3.8)	2.9%<br>(1.8 to 4.2)	5.6%<br>(3.7 to 8.4)
**Malawi**	57.7%<br>(43.0 to 69.0)	0.6%<br>(-0.4 to 2.0)	43.4%<br>(36.3 to 50.0)	Not reported	7.0%<br>(-0.9 to 15.7)	Not reported	2.3%<br>(1.8 to 3.0)	3.6%<br>(2.4 to 4.9)	10.5%<br>(6.4 to 16.1)
**Malaysia**	64.7%<br>(49.6 to 75.3)	0.7%<br>(0.5 to 0.9)	0.0%<br>(0.0 to 0.3)	Not reported	4.6%<br>(-0.6 to 10.5)	Not reported	0.1%<br>(0.0 to 0.1)	6.1%<br>(4.1 to 8.1)	10.9%<br>(8.6 to 13.5)
**Maldives**	55.9%<br>(40.8 to 67.4)	0.7%<br>(0.4 to 1.2)	0.9%<br>(0.0 to 6.6)	Not reported	5.3%<br>(-0.7 to 12.0)	Not reported	0.0%<br>(0.0 to 0.0)	6.7%<br>(4.7 to 9.0)	14.8%<br>(12.1 to 17.8)
**Mali**	45.6%<br>(32.3 to 57.1)	6.6%<br>(4.2 to 9.9)	35.9%<br>(28.4 to 43.3)	Not reported	9.7%<br>(-1.3 to 21.5)	Not reported	0.3%<br>(0.1 to 0.7)	4.4%<br>(2.9 to 6.0)	6.1%<br>(3.8 to 9.7)
**Malta**	52.6%<br>(38.4 to 65.2)	0.5%<br>(-0.5 to 2.1)	0.0%<br>(0.0 to 0.0)	Not reported	6.8%<br>(-0.9 to 15.1)	Not reported	3.5%<br>(2.0 to 4.7)	3.6%<br>(2.4 to 4.9)	18.5%<br>(15.7 to 21.5)
**Marshall Islands**	39.9%<br>(27.4 to 52.7)	0.9%<br>(0.4 to 1.4)	16.8%<br>(11.0 to 23.3)	Not reported	1.9%<br>(-0.2 to 4.4)	Not reported	0.0%<br>(0.0 to 0.0)	5.5%<br>(3.7 to 7.4)	13.7%<br>(10.5 to 17.6)
**Mauritania**	55.6%<br>(41.2 to 66.6)	8.7%<br>(4.3 to 14.5)	21.8%<br>(13.0 to 31.1)	Not reported	5.0%<br>(-0.7 to 11.2)	Not reported	0.7%<br>(0.2 to 1.4)	5.1%<br>(3.4 to 7.0)	7.2%<br>(4.7 to 11.7)

**Mauritius**	56.5%<br>(41.8 to 68.5)	0.1%<br>(-0.1 to 0.4)	0.1%<br>(0.0 to 0.6)	Not reported	3.3%<br>(-0.4 to 7.5)	Not reported	1.1%<br>(0.3 to 1.8)	6.6%<br>(4.5 to 8.9)	15.3%<br>(13.2 to 17.6)
**Mexico**	47.6%<br>(34.5 to 58.6)	0.6%<br>(0.0 to 1.4)	1.6%<br>(0.1 to 7.0)	Not reported	6.4%<br>(-0.8 to 14.5)	Not reported	4.5%<br>(3.7 to 5.2)	2.8%<br>(1.9 to 3.8)	7.2%<br>(6.0 to 8.4)
**Micronesia (Federated States of)**	39.6%<br>(26.4 to 52.1)	0.9%<br>(0.4 to 1.4)	17.7%<br>(11.4 to 24.6)	Not reported	1.9%<br>(-0.3 to 4.5)	Not reported	0.0%<br>(0.0 to 0.0)	7.4%<br>(4.8 to 10.2)	20.4%<br>(16.7 to 24.2)
**Monaco**	51.1%<br>(36.7 to 63.6)	0.0%<br>(-0.1 to 0.1)	0.0%<br>(0.0 to 0.0)	Not reported	2.4%<br>(-0.3 to 5.5)	Not reported	7.2%<br>(6.4 to 7.9)	3.9%<br>(2.7 to 5.4)	20.4%<br>(16.4 to 25.0)
**Mongolia**	63.1%<br>(48.4 to 75.1)	-0.1%<br>(-0.2 to 0.0)	7.8%<br>(0.6 to 27.3)	Not reported	5.1%<br>(-0.7 to 11.6)	Not reported	9.8%<br>(7.3 to 12.6)	5.4%<br>(3.6 to 7.1)	19.0%<br>(15.1 to 23.2)
**Montenegro**	59.2%<br>(44.7 to 71.1)	0.1%<br>(-0.2 to 0.4)	3.1%<br>(0.0 to 20.7)	Not reported	2.8%<br>(-0.4 to 6.3)	Not reported	8.0%<br>(7.0 to 9.2)	5.6%<br>(3.8 to 7.4)	24.9%<br>(21.4 to 28.9)
**Morocco**	58.4%<br>(43.0 to 69.9)	0.6%<br>(-0.1 to 1.8)	1.0%<br>(0.2 to 3.2)	Not reported	5.6%<br>(-0.8 to 12.6)	Not reported	5.7%<br>(4.7 to 6.9)	5.0%<br>(3.5 to 6.8)	6.4%<br>(4.5 to 9.3)
**Mozambique**	63.3%<br>(47.3 to 74.4)	0.8%<br>(-0.4 to 2.3)	44.1%<br>(36.9 to 51.0)	Not reported	9.3%<br>(-1.3 to 20.9)	Not reported	1.4%<br>(1.0 to 1.9)	2.8%<br>(1.7 to 4.0)	9.4%<br>(6.7 to 12.9)
**Myanmar**	51.1%<br>(37.7 to 62.5)	2.2%<br>(1.3 to 3.4)	26.5%<br>(13.6 to 37.7)	Not reported	6.4%<br>(-0.8 to 14.3)	Not reported	0.9%<br>(0.5 to 1.3)	4.9%<br>(3.2 to 6.8)	12.1%<br>(9.7 to 15.0)
**Namibia**	56.5%<br>(41.7 to 69.0)	0.5%<br>(-0.5 to 1.9)	10.0%<br>(1.0 to 29.0)	Not reported	5.9%<br>(-0.8 to 13.8)	Not reported	2.6%<br>(2.1 to 3.1)	4.1%<br>(2.6 to 6.0)	9.1%<br>(7.5 to 10.9)
**Nauru**	52.5%<br>(38.4 to 62.8)	1.0%<br>(0.5 to 1.5)	0.6%<br>(0.0 to 2.9)	Not reported	1.2%<br>(-0.2 to 2.9)	Not reported	0.0%<br>(0.0 to 0.0)	6.9%<br>(4.5 to 9.5)	17.3%<br>(14.2 to 20.7)
**Nepal**	42.0%<br>(27.9 to 54.8)	0.8%<br>(-0.5 to 2.7)	31.9%<br>(25.0 to 39.3)	Not reported	12.1%<br>(-1.7 to 26.2)	Not reported	4.2%<br>(3.5 to 5.0)	4.6%<br>(3.0 to 6.3)	14.3%<br>(11.8 to 17.3)
**Netherlands**	53.6%<br>(39.1 to 65.9)	0.0%<br>(0.0 to 0.1)	0.0%<br>(0.0 to 0.0)	Not reported	2.9%<br>(-0.4 to 6.8)	Not reported	7.0%<br>(6.0 to 9.7)	3.1%<br>(2.1 to 4.2)	17.8%<br>(15.2 to 20.4)
**New Zealand**	48.4%<br>(34.3 to 60.5)	0.0%<br>(0.0 to 0.0)	0.0%<br>(0.0 to 0.0)	Not reported	4.8%<br>(-0.6 to 11.1)	Not reported	7.4%<br>(6.3 to 8.7)	4.0%<br>(2.7 to 5.4)	16.8%<br>(14.3 to 19.6)
**Nicaragua**	55.7%<br>(39.9 to 67.4)	0.7%<br>(0.6 to 0.8)	21.9%<br>(12.0 to 30.7)	Not reported	6.8%<br>(-1.0 to 15.0)	Not reported	0.3%<br>(0.2 to 0.4)	5.0%<br>(3.4 to 6.8)	8.6%<br>(6.7 to 10.9)
**Niger**	52.7%<br>(38.4 to 64.8)	6.9%<br>(4.1 to 10.7)	38.1%<br>(30.8 to 45.4)	Not reported	12.0%<br>(-1.7 to 25.2)	Not reported	1.2%<br>(0.3 to 2.6)	2.5%<br>(1.6 to 3.4)	3.5%<br>(2.1 to 5.9)
**Nigeria**	56.8%<br>(42.6 to 68.3)	2.0%<br>(1.4 to 2.8)	23.0%<br>(13.3 to 33.2)	Not reported	5.4%<br>(-0.7 to 12.2)	Not reported	0.3%<br>(0.1 to 0.7)	1.6%<br>(1.1 to 2.3)	3.4%<br>(2.1 to 5.4)
**Niue**	39.1%<br>(28.1 to 48.8)	0.2%<br>(0.2 to 0.2)	0.5%<br>(0.0 to 2.8)	Not reported	0.9%<br>(-0.1 to 2.0)	Not reported	0.4%<br>(0.2 to 0.6)	3.6%<br>(2.4 to 4.9)	9.5%<br>(7.4 to 12.3)
**North Macedonia**	58.9%<br>(44.6 to 71.3)	0.2%<br>(-0.3 to 1.2)	2.7%<br>(0.1 to 14.4)	Not reported	3.7%<br>(-0.5 to 8.6)	Not reported	8.8%<br>(7.9 to 11.4)	7.1%<br>(4.8 to 9.4)	23.4%<br>(20.0 to 26.7)
**Northern Mariana Islands**	53.6%<br>(39.0 to 66.4)	0.7%<br>(0.6 to 0.8)	0.2%<br>(0.0 to 1.9)	Not reported	1.0%<br>(-0.1 to 2.5)	Not reported	0.2%<br>(0.1 to 0.3)	5.6%<br>(3.7 to 7.5)	14.9%<br>(12.0 to 18.1)
**Norway**	56.7%<br>(41.8 to 67.3)	0.0%<br>(0.0 to 0.0)	0.0%<br>(0.0 to 0.0)	Not reported	2.9%<br>(-0.4 to 7.0)	Not reported	8.7%<br>(7.8 to 9.6)	2.9%<br>(1.9 to 3.9)	15.9%<br>(13.6 to 18.4)

**Oman**	54.6% (40.7 to 64.8)	6.8% (3.1 to 11.5)	0.0% (0.0 to 0.0)	Not reported	5.0% (-0.6 to 11.3)	Not reported	2.3% (0.5 to 4.8)	3.8% (2.6 to 5.0)	7.2% (5.5 to 8.9)
**Pakistan**	54.2% (39.6 to 65.4)	5.6% (0.8 to 11.6)	22.1% (12.7 to 31.5)	Not reported	9.0% (-1.2 to 19.9)	Not reported	4.9% (2.2 to 8.2)	5.7% (3.8 to 7.6)	10.8% (7.7 to 15.2)
**Palau**	49.1% (36.0 to 60.5)	0.6% (0.3 to 0.9)	0.0% (0.0 to 0.0)	Not reported	1.0% (-0.1 to 2.2)	Not reported	0.0% (0.0 to 0.0)	4.4% (3.0 to 5.9)	11.8% (9.3 to 14.7)
**Palestine**	43.6% (30.0 to 55.6)	1.3% (-0.4 to 4.4)	1.1% (0.4 to 2.4)	Not reported	6.5% (-0.9 to 14.5)	Not reported	2.7% (2.0 to 3.6)	5.5% (3.8 to 7.2)	13.1% (10.7 to 15.8)
**Panama**	53.9% (38.6 to 65.6)	0.2% (0.1 to 0.4)	0.4% (0.0 to 4.1)	Not reported	6.0% (-0.8 to 13.4)	Not reported	0.3% (0.2 to 0.4)	2.4% (1.6 to 3.3)	6.9% (5.6 to 8.3)
**Papua New Guinea**	35.1% (22.6 to 47.1)	0.1% (0.1 to 0.2)	34.6% (26.2 to 42.6)	Not reported	2.3% (-0.3 to 5.3)	Not reported	3.3% (2.8 to 4.0)	5.9% (3.9 to 8.1)	12.9% (9.5 to 17.0)
**Paraguay**	54.5% (39.3 to 66.3)	1.0% (-0.7 to 3.3)	7.0% (0.6 to 21.1)	Not reported	5.6% (-0.7 to 12.5)	Not reported	2.5% (1.8 to 3.2)	4.7% (3.1 to 6.4)	13.1% (10.7 to 16.1)
**Peru**	39.9% (27.2 to 51.7)	0.0% (0.0 to 0.1)	2.7% (0.2 to 12.0)	Not reported	5.2% (-0.7 to 11.7)	Not reported	6.1% (5.0 to 7.2)	1.9% (1.2 to 2.7)	6.5% (5.0 to 8.2)
**Philippines**	44.1% (31.4 to 55.1)	1.0% (0.9 to 1.1)	15.5% (7.4 to 25.8)	Not reported	3.8% (-0.5 to 8.4)	Not reported	0.1% (0.0 to 0.2)	5.7% (3.9 to 7.7)	15.0% (12.1 to 17.8)
**Poland**	54.1% (39.8 to 65.4)	0.0% (-0.2 to 0.1)	0.5% (0.0 to 4.5)	Not reported	3.6% (-0.5 to 8.4)	Not reported	8.8% (7.9 to 11.0)	5.0% (3.5 to 6.8)	23.0% (19.9 to 26.0)
**Portugal**	51.5% (37.5 to 63.3)	0.0% (0.0 to 0.1)	0.0% (0.0 to 0.1)	Not reported	5.8% (-0.7 to 13.3)	Not reported	8.1% (7.6 to 8.7)	4.5% (3.0 to 6.0)	16.0% (13.6 to 18.2)
**Puerto Rico**	52.4% (38.0 to 65.2)	0.4% (0.3 to 0.6)	0.0% (0.0 to 0.0)	Not reported	3.0% (-0.4 to 6.7)	Not reported	0.0% (-0.1 to 0.1)	2.0% (1.3 to 2.7)	11.2% (9.0 to 13.8)
**Qatar**	54.0% (40.8 to 64.7)	8.8% (3.0 to 15.0)	0.0% (0.0 to 0.0)	Not reported	3.7% (-0.5 to 8.5)	Not reported	3.2% (0.8 to 6.3)	4.6% (3.1 to 6.1)	11.1% (8.8 to 14.0)
**Republic of Korea**	40.6% (26.5 to 52.2)	0.3% (-0.5 to 1.6)	0.0% (0.0 to 0.0)	Not reported	3.8% (-0.5 to 8.9)	Not reported	6.7% (5.8 to 7.7)	4.5% (3.1 to 6.0)	15.3% (12.7 to 18.1)
**Republic of Moldova**	65.5% (50.2 to 76.5)	0.1% (-0.6 to 1.0)	5.4% (1.8 to 10.4)	Not reported	3.8% (-0.5 to 8.6)	Not reported	7.5% (6.4 to 9.3)	4.3% (2.9 to 5.8)	21.5% (18.8 to 24.6)
**Romania**	59.6% (45.6 to 70.9)	0.2% (-0.4 to 1.2)	0.5% (0.0 to 5.5)	Not reported	3.5% (-0.5 to 7.8)	Not reported	8.7% (7.8 to 10.4)	5.1% (3.6 to 6.8)	19.0% (16.4 to 22.1)
**Russian Federation**	58.5% (43.9 to 69.7)	-0.1% (-0.5 to 0.3)	0.1% (0.0 to 0.9)	Not reported	2.9% (-0.4 to 6.6)	Not reported	8.6% (6.8 to 10.4)	4.5% (3.0 to 6.2)	23.2% (20.4 to 26.1)
**Rwanda**	50.4% (36.0 to 62.8)	0.0% (0.0 to 0.0)	43.7% (36.4 to 50.8)	Not reported	6.5% (-0.9 to 14.3)	Not reported	3.7% (2.9 to 5.0)	3.0% (2.1 to 4.2)	12.9% (9.8 to 17.1)
**Saint Kitts and Nevis**	54.8% (41.4 to 66.3)	0.3% (0.2 to 0.5)	0.0% (0.0 to 0.5)	Not reported	4.2% (-0.5 to 9.5)	Not reported	0.0% (-0.1 to 0.1)	2.4% (1.6 to 3.2)	5.8% (4.5 to 7.3)
**Saint Lucia**	51.8% (37.3 to 63.5)	0.4% (0.3 to 0.7)	0.6% (0.0 to 3.4)	Not reported	5.8% (-0.8 to 13.2)	Not reported	0.0% (0.0 to 0.1)	2.5% (1.6 to 3.5)	8.0% (6.6 to 9.6)
**Saint Vincent and the Grenadines**	48.4% (34.1 to 60.7)	0.4% (0.3 to 0.6)	0.7% (0.0 to 2.9)	Not reported	6.4% (-0.8 to 14.5)	Not reported	0.0% (0.0 to 0.0)	3.0% (2.0 to 4.1)	8.3% (6.8 to 10.2)

**Samoa**	49.3% (34.9 to 61.6)	0.0% (0.0 to 0.1)	24.9% (12.1 to 35.4)	Not reported	1.6% (-0.2 to 3.8)	Not reported	0.1% (-0.1 to 0.3)	6.2% (4.3 to 8.2)	17.1% (13.9 to 20.7)
**San Marino**	53.8% (38.9 to 66.3)	0.0% (-0.2 to 0.4)	0.0% (0.0 to 0.0)	Not reported	2.2% (-0.3 to 5.1)	Not reported	5.7% (4.4 to 6.9)	3.4% (2.2 to 4.6)	18.1% (14.9 to 21.9)
**Sao Tome and Principe**	62.2% (47.9 to 73.4)	0.2% (0.0 to 0.4)	23.3% (16.4 to 30.7)	Not reported	6.0% (-0.8 to 13.6)	Not reported	0.0% (0.0 to 0.1)	1.7% (1.1 to 2.4)	4.4% (2.6 to 7.1)
**Saudi Arabia**	47.3% (33.0 to 59.8)	7.7% (2.2 to 13.7)	0.0% (0.0 to 0.0)	Not reported	5.7% (-0.7 to 12.9)	Not reported	2.8% (1.0 to 5.1)	5.3% (3.6 to 7.2)	10.1% (7.0 to 13.1)
**Senegal**	55.9% (41.3 to 67.5)	3.9% (2.6 to 5.5)	35.7% (28.1 to 43.1)	Not reported	5.6% (-0.7 to 12.2)	Not reported	0.3% (0.0 to 0.5)	4.2% (2.9 to 5.7)	6.7% (3.9 to 10.8)
**Serbia**	63.3% (48.4 to 74.8)	0.3% (-0.4 to 1.5)	2.6% (0.1 to 15.4)	Not reported	3.4% (-0.4 to 7.7)	Not reported	8.5% (7.4 to 10.4)	5.9% (4.0 to 7.9)	24.2% (20.9 to 27.7)
**Seychelles**	56.0% (41.0 to 68.3)	0.3% (0.2 to 0.5)	0.0% (0.0 to 0.2)	Not reported	2.5% (-0.3 to 5.7)	Not reported	0.1% (-0.1 to 0.3)	5.6% (3.7 to 7.5)	14.8% (10.1 to 19.8)
**Sierra Leone**	57.2% (43.4 to 68.6)	0.6% (0.5 to 0.9)	37.5% (30.6 to 44.2)	Not reported	7.4% (-1.0 to 16.3)	Not reported	0.1% (0.0 to 0.2)	3.6% (2.3 to 5.0)	7.2% (4.5 to 11.6)
**Singapore**	37.2% (24.3 to 48.9)	0.4% (0.4 to 0.5)	0.0% (0.0 to 0.0)	Not reported	4.9% (-0.6 to 11.2)	Not reported	0.0% (-0.1 to 0.0)	3.2% (2.2 to 4.3)	9.5% (8.1 to 11.1)
**Slovakia**	58.3% (43.3 to 70.1)	0.0% (-0.3 to 0.4)	0.0% (0.0 to 0.2)	Not reported	2.8% (-0.4 to 6.4)	Not reported	8.3% (7.1 to 9.6)	5.7% (3.9 to 7.6)	19.0% (16.0 to 22.4)
**Slovenia**	60.6% (44.6 to 72.1)	0.1% (-0.2 to 0.5)	0.2% (0.0 to 1.5)	Not reported	2.6% (-0.3 to 6.1)	Not reported	6.8% (6.0 to 8.6)	5.0% (3.4 to 6.7)	19.7% (17.0 to 22.7)
**Solomon Islands**	40.4% (27.0 to 52.8)	0.3% (0.1 to 0.6)	42.7% (35.3 to 49.8)	Not reported	4.0% (-0.5 to 8.9)	Not reported	0.0% (0.0 to 0.1)	7.1% (4.7 to 9.5)	19.6% (15.9 to 23.8)
**Somalia**	50.4% (34.6 to 62.6)	0.8% (0.0 to 1.3)	44.0% (37.0 to 50.8)	Not reported	12.5% (-1.7 to 27.0)	Not reported	0.4% (0.2 to 0.5)	3.3% (1.9 to 4.6)	7.4% (3.7 to 12.9)
**South Africa**	60.1% (45.0 to 71.4)	0.1% (-0.1 to 0.3)	3.3% (0.6 to 9.0)	Not reported	4.6% (-0.6 to 10.4)	Not reported	5.6% (4.9 to 6.3)	5.3% (3.6 to 7.1)	10.5% (8.7 to 12.5)
**South Sudan**	50.3% (35.8 to 62.6)	4.8% (3.3 to 6.8)	39.2% (31.4 to 46.4)	Not reported	7.1% (-0.9 to 15.7)	Not reported	0.4% (0.1 to 0.7)	3.0% (1.9 to 4.1)	7.6% (5.0 to 11.0)
**Spain**	52.7% (37.5 to 64.8)	0.1% (-0.1 to 0.4)	0.0% (0.0 to 0.1)	Not reported	4.9% (-0.6 to 11.2)	Not reported	7.0% (6.4 to 7.7)	4.1% (2.7 to 5.5)	17.6% (15.1 to 20.4)
**Sri Lanka**	57.5% (42.6 to 68.8)	0.4% (0.3 to 0.6)	10.1% (0.8 to 32.4)	Not reported	3.1% (-0.4 to 7.0)	Not reported	0.3% (0.2 to 0.5)	4.0% (2.5 to 5.7)	7.4% (5.8 to 9.1)
**Sudan**	54.9% (41.4 to 65.3)	8.7% (4.5 to 14.0)	17.2% (9.9 to 24.6)	Not reported	9.2% (-1.3 to 20.7)	Not reported	1.3% (0.3 to 2.6)	4.0% (2.7 to 5.4)	7.8% (5.1 to 11.1)
**Suriname**	40.7% (27.8 to 51.9)	0.4% (0.2 to 0.7)	0.9% (0.0 to 5.9)	Not reported	5.3% (-0.7 to 12.1)	Not reported	0.0% (-0.1 to 0.1)	4.3% (2.9 to 5.7)	13.0% (10.5 to 15.8)
**Sweden**	49.4% (34.8 to 61.0)	0.0% (-0.1 to 0.0)	0.0% (0.0 to 0.0)	Not reported	2.0% (-0.3 to 4.8)	Not reported	8.4% (7.3 to 9.4)	2.3% (1.5 to 3.1)	16.4% (14.0 to 19.3)
**Switzerland**	45.1% (32.2 to 56.6)	0.0% (-0.1 to 0.1)	0.0% (0.0 to 0.0)	Not reported	3.6% (-0.4 to 8.2)	Not reported	8.1% (7.2 to 9.7)	3.0% (2.0 to 4.2)	17.1% (14.6 to 19.9)

**Syrian Arab Republic**	48.3%<br>(36.5 to 59.2)	1.3%<br>(-0.2 to 3.6)	0.1%<br>(0.0 to 0.2)	Not reported	7.1%<br>(-1.0 to 15.9)	Not reported	5.0%<br>(4.1 to 5.7)	5.4%<br>(3.6 to 7.2)	13.0%<br>(10.5 to 15.8)
**Turkey**	50.7%<br>(36.6 to 62.5)	0.3%<br>(-0.3 to 1.0)	0.1%<br>(0.0 to 0.8)	Not reported	4.2%<br>(-0.5 to 9.6)	Not reported	7.4%<br>(6.4 to 8.2)	5.6%<br>(3.8 to 7.5)	17.3%<br>(14.6 to 20.4)
**Taiwan (Province of China)**	44.7%<br>(31.3 to 57.5)	1.2%<br>(-0.9 to 4.2)	0.1%<br>(0.0 to 0.5)	Not reported	4.9%<br>(-0.6 to 11.4)	Not reported	2.1%<br>(1.6 to 2.6)	5.8%<br>(4.0 to 7.7)	15.6%<br>(13.6 to 17.9)
**Tajikistan**	55.3%<br>(39.8 to 67.2)	0.6%<br>(-0.3 to 1.9)	20.3%<br>(13.4 to 27.0)	Not reported	5.4%<br>(-0.7 to 12.2)	Not reported	7.4%<br>(6.2 to 8.4)	4.1%<br>(2.8 to 5.5)	9.7%<br>(6.9 to 13.5)
**Thailand**	46.1%<br>(31.7 to 58.1)	2.1%<br>(1.8 to 2.5)	1.4%<br>(0.0 to 9.0)	Not reported	3.2%<br>(-0.4 to 7.5)	Not reported	0.7%<br>(0.2 to 1.4)	4.5%<br>(3.0 to 6.0)	15.3%<br>(12.7 to 18.3)
**Timor-Leste**	56.1%<br>(42.2 to 67.7)	0.1%<br>(0.0 to 0.2)	27.0%<br>(11.0 to 41.1)	Not reported	7.6%<br>(-1.0 to 16.7)	Not reported	0.6%<br>(0.4 to 0.8)	6.1%<br>(3.9 to 8.3)	13.9%<br>(9.8 to 19.1)
**Togo**	55.7%<br>(41.3 to 67.2)	2.2%<br>(1.7 to 2.8)	37.1%<br>(30.1 to 43.9)	Not reported	6.6%<br>(-0.8 to 14.9)	Not reported	0.1%<br>(0.0 to 0.2)	3.5%<br>(2.4 to 4.6)	8.8%<br>(5.3 to 13.8)
**Tokelau**	38.4%<br>(26.1 to 48.8)	0.6%<br>(0.2 to 1.1)	0.0%<br>(0.0 to 0.0)	Not reported	1.1%<br>(-0.1 to 2.4)	Not reported	0.0%<br>(0.0 to 0.0)	4.6%<br>(3.0 to 6.3)	11.0%<br>(8.1 to 14.2)
**Tonga**	53.2%<br>(39.1 to 64.6)	0.3%<br>(0.2 to 0.5)	16.4%<br>(7.4 to 25.1)	Not reported	1.7%<br>(-0.2 to 3.8)	Not reported	0.0%<br>(0.0 to 0.0)	6.2%<br>(4.2 to 8.1)	14.0%<br>(11.0 to 17.5)
**Trinidad and Tobago**	54.3%<br>(39.6 to 65.5)	0.3%<br>(0.1 to 0.5)	0.0%<br>(0.0 to 0.0)	Not reported	2.6%<br>(-0.3 to 6.0)	Not reported	0.0%<br>(-0.1 to 0.1)	4.2%<br>(2.7 to 5.6)	11.9%<br>(9.8 to 14.4)
**Tunisia**	49.0%<br>(35.6 to 61.0)	1.9%<br>(-0.1 to 5.3)	0.1%<br>(0.0 to 0.3)	Not reported	6.3%<br>(-0.9 to 14.5)	Not reported	4.4%<br>(3.4 to 5.4)	6.2%<br>(4.2 to 8.4)	13.5%<br>(9.8 to 17.7)
**Turkmenistan**	59.1%<br>(44.7 to 71.8)	1.9%<br>(0.1 to 4.5)	0.0%<br>(0.0 to 0.1)	Not reported	3.1%<br>(-0.4 to 7.0)	Not reported	7.6%<br>(6.2 to 9.0)	5.2%<br>(3.5 to 6.9)	15.3%<br>(12.2 to 18.9)
**Tuvalu**	48.3%<br>(33.9 to 60.3)	0.6%<br>(0.3 to 1.1)	6.6%<br>(3.7 to 10.5)	Not reported	2.1%<br>(-0.3 to 4.7)	Not reported	0.0%<br>(0.0 to 0.0)	6.8%<br>(4.5 to 9.3)	16.5%<br>(13.1 to 20.5)
**Uganda**	53.0%<br>(38.7 to 64.8)	0.1%<br>(0.0 to 0.2)	39.8%<br>(32.5 to 46.3)	Not reported	7.3%<br>(-0.9 to 16.5)	Not reported	1.2%<br>(0.9 to 1.5)	1.8%<br>(1.2 to 2.6)	6.3%<br>(4.5 to 8.9)
**Ukraine**	62.3%<br>(46.5 to 73.9)	-0.1%<br>(-0.8 to 0.5)	1.0%<br>(0.1 to 3.7)	Not reported	2.1%<br>(-0.3 to 4.9)	Not reported	8.6%<br>(7.1 to 10.0)	4.4%<br>(3.0 to 5.9)	20.6%<br>(16.3 to 24.9)
**United Arab Emirates**	52.6%<br>(38.4 to 64.1)	11.7%<br>(5.2 to 19.3)	0.0%<br>(0.0 to 0.0)	Not reported	3.9%<br>(-0.5 to 8.7)	Not reported	4.1%<br>(1.0 to 8.5)	4.7%<br>(3.2 to 6.3)	8.0%<br>(6.1 to 10.2)
**United Kingdom**	48.8%<br>(35.8 to 59.5)	0.0%<br>(0.0 to 0.0)	0.0%<br>(0.0 to 0.0)	Not reported	2.5%<br>(-0.3 to 5.8)	Not reported	7.8%<br>(6.4 to 10.5)	3.0%<br>(2.1 to 4.0)	16.8%<br>(14.2 to 19.4)
**United Republic of Tanzania**	54.7%<br>(40.8 to 65.8)	0.1%<br>(0.0 to 0.3)	40.1%<br>(32.3 to 47.1)	Not reported	4.7%<br>(-0.6 to 10.8)	Not reported	1.4%<br>(1.1 to 1.8)	2.6%<br>(1.6 to 3.7)	10.0%<br>(7.3 to 13.8)
**United States of America**	43.6%<br>(30.0 to 55.0)	0.4%<br>(-0.5 to 1.8)	0.0%<br>(0.0 to 0.0)	Not reported	2.4%<br>(-0.3 to 5.6)	Not reported	6.2%<br>(5.3 to 6.9)	2.6%<br>(1.8 to 3.6)	17.4%<br>(15.0 to 20.2)
**United States Virgin Islands**	48.5%<br>(34.2 to 61.5)	0.3%<br>(0.2 to 0.5)	0.0%<br>(0.0 to 0.0)	Not reported	2.4%<br>(-0.3 to 5.3)	Not reported	0.0%<br>(-0.1 to 0.1)	2.7%<br>(1.8 to 3.6)	9.0%<br>(6.8 to 11.3)
**Uruguay**	51.5%<br>(37.5 to 63.8)	0.1%<br>(-0.1 to 0.3)	0.1%<br>(0.0 to 1.2)	Not reported	5.2%<br>(-0.6 to 11.7)	Not reported	6.5%<br>(6.0 to 7.3)	4.4%<br>(2.9 to 5.9)	19.5%<br>(16.8 to 22.2)

**Uzbekistan**	58.5%<br>(43.9 to 70.6)	0.7%<br>(-0.5 to 2.2)	4.1%<br>(0.7 to 11.3)	Not reported	3.7%<br>(-0.5 to 8.6)	Not reported	7.7%<br>(6.2 to 8.9)	4.4%<br>(3.0 to 5.9)	12.4%<br>(10.5 to 14.3)
**Vanuatu**	56.0%<br>(40.9 to 67.4)	0.1%<br>(0.1 to 0.2)	37.6%<br>(30.2 to 44.9)	Not reported	3.1%<br>(-0.4 to 7.3)	Not reported	0.3%<br>(0.1 to 0.4)	3.9%<br>(2.5 to 5.3)	9.9%<br>(7.1 to 12.8)
**Venezuela (Bolivarian Republic of)**	48.0%<br>(34.1 to 60.3)	0.5%<br>(0.4 to 0.6)	0.2%<br>(0.0 to 0.9)	Not reported	7.0%<br>(-0.9 to 16.4)	Not reported	0.9%<br>(0.6 to 1.1)	3.9%<br>(2.5 to 5.3)	8.7%<br>(7.0 to 10.5)
**Viet Nam**	58.5%<br>(43.3 to 70.8)	1.7%<br>(0.2 to 3.5)	15.8%<br>(5.7 to 27.1)	Not reported	5.1%<br>(-0.7 to 11.4)	Not reported	1.2%<br>(0.7 to 1.7)	5.7%<br>(3.8 to 7.6)	18.0%<br>(13.1 to 21.9)
**Yemen**	47.7%<br>(33.1 to 60.0)	1.5%<br>(0.7 to 2.6)	15.9%<br>(10.6 to 21.8)	Not reported	12.2%<br>(-1.8 to 26.0)	Not reported	2.8%<br>(2.3 to 3.5)	5.5%<br>(3.7 to 7.5)	15.3%<br>(10.8 to 19.7)
**Zambia**	51.3%<br>(37.5 to 64.4)	0.6%<br>(-0.4 to 2.0)	35.6%<br>(25.7 to 44.5)	Not reported	5.6%<br>(-0.8 to 12.9)	Not reported	2.4%<br>(1.9 to 3.0)	3.0%<br>(1.8 to 4.4)	7.6%<br>(5.6 to 9.9)
**Zimbabwe**	57.8%<br>(42.8 to 69.2)	0.5%<br>(-0.4 to 1.8)	35.7%<br>(29.2 to 42.3)	Not reported	7.6%<br>(-1.0 to 16.9)	Not reported	3.3%<br>(2.5 to 4.2)	4.4%<br>(3.0 to 5.9)	12.8%<br>(9.9 to 16.7)

Appendix Table 15. Age-standardised rates (per 100,000 people) and percentages of DALYs (with 95% uncertainty intervals) due to stroke attributable to metabolic, behavioural and environmental/occupational risk clusters and all risk factors combined globally by SDI and sex in 2021.

**Metrics**	**High SDI**		**High-middle SDI**		**Middle SDI**		**Low-middle SDI**		**Low SDI**		**Globally**	
	Males	Females	Males	Females	Males	Females	Males	Females	Males	Females	Males	Females
**Rates**												
**Metabolic risks**	590 (487 to 680)	405 (330 to 475)	1,744 (1,391 to 2,113)	1,147 (945 to 1,346)	1,916 (1,525 to 2,296)	1,294 (1,072 to 1,546)	1,761 (1,456 to 2,034)	1,483 (1,231 to 1,723)	1,728 (1,415 to 2,064)	1,514 (1,231 to 1,811)	1,525 (1,254 to 1,787)	1,093 (913 to 1,271)
**Behavioural risks**	339 (258 to 436)	153 (105 to 211)	1,163 (900 to 1,532)	407 (266 to 587)	1,262 (964 to 1,618)	453 (292 to 672)	1,019 (744 to 1,277)	524 (303 to 743)	869 (542 to 1,173)	552 (275 to 809)	967 (762 to 1,214)	395 (265 to 556)
**Environmental and occupational risks**	179 (137 to 225)	113 (86 to 141)	776 (568 to 1,014)	461 (352 to 591)	1,063 (780 to 1,362)	676 (501 to 867)	1,214 (940 to 1,476)	978 (751 to 1,169)	1,453 (1,152 to 1,718)	1,229 (980 to 1,471)	834 (635 to 1,031)	569 (449 to 694)
**All risks combined**	700 (630 to 772)	465 (402 to 525)	2,115 (1,824 to 2,454)	1,303 (1,136 to 1,488)	2,398 (2,087 to 2,746)	1,519 (1,323 to 1,733)	2,239 (2,032 to 2,452)	1,797 (1,602 to 1,997)	2,281 (2,004 to 2,568)	1,939 (1,691 to 2,204)	1,899 (1,703 to 2,111)	1,290 (1,153 to 1,434)
**Percentages**												
**Metabolic risks**	68.6% (57.5 to 77.6)	66.1% (55.0 to 74.9)	71.0% (60.0 to 80.0)	72.1% (60.1 to 80.6)	68.2% (57.0 to 77.4)	69.7% (58.0 to 78.6)	68.1% (57.4 to 77.0)	70.2% (59.4 to 78.5)	65.1% (53.8 to 74.2)	66.5% (55.2 to 75.1)	68.3% (57.2 to 77.3)	69.3% (58.0 to 77.6)
**Behavioural risks**	39.4% (29.9 to 49.6)	25.0% (17.2 to 34.3)	47.3% (37.7 to 57.3)	25.6% (16.5 to 36.3)	44.9% (35.7 to 54.7)	24.4% (15.8 to 34.9)	39.4% (29.4 to 48.8)	24.8% (14.7 to 35.4)	32.7% (20.9 to 43.5)	24.2% (12.1 to 35.6)	43.3% (34.4 to 52.6)	25.1% (17.0 to 34.6)
**Environmental and occupational risks**	20.9% (15.9 to 25.6)	18.5% (14.3 to 22.6)	31.5% (24.2 to 38.9)	29.0% (23.3 to 35.5)	37.8% (28.5 to 46.9)	36.4% (28.4 to 44.7)	47.0% (37.0 to 56.1)	46.3% (37.5 to 54.3)	54.8% (44.9 to 63.2)	54.0% (45.2 to 61.9)	37.4% (29.0 to 45.4)	36.0% (28.9 to 43.1)
**All risks combined**	81.3% (74.6 to 87.0)	76.0% (67.9 to 82.6)	86.1% (79.9 to 90.8)	81.9% (74.4 to 87.6)	85.4% (79.6 to 89.8)	81.8% (74.7 to 87.2)	86.6% (81.0 to 90.3)	85.1% (78.8 to 89.4)	86.0% (80.3 to 89.6)	85.1% (78.9 to 89.0)	85.1% (79.2 to 89.3)	81.7% (74.6 to 86.8)

Appendix Table 16. Age-standardised rates (per 100,000 people) and percentages of DALYs (with 95% uncertainty intervals) due to ischaemic stroke attributable to metabolic, behavioural and environmental/occupational risk clusters and all risk factors combined globally by SDI and sex in 2021.

Metrics	High SDI		High-middle SDI		Middle SDI		Low-middle SDI		Low SDI		Globally	
	Males	Females	Males	Females	Males	Females	Males	Females	Males	Females	Males	Females
**Rates**												
**Metabolic risks**	356 (297 to 410)	257 (209 to 300)	1,005 (824 to 1,182)	721 (601 to 837)	886 (723 to 1,050)	626 (518 to 745)	800 (660 to 955)	676 (560 to 814)	725 (585 to 900)	638 (489 to 825)	753 (630 to 866)	563 (470 to 652)
**Behavioural risks**	193 (127 to 260)	99 (57 to 143)	610 (437 to 808)	255 (142 to 369)	516 (371 to 674)	220 (117 to 322)	391 (274 to 526)	219 (118 to 325)	310 (187 to 460)	213 (109 to 342)	425 (304 to 555)	200 (113 to 284)
**Environmental and occupational risks**	95 (71 to 120)	62 (47 to 78)	396 (286 to 512)	254 (195 to 322)	439 (320 to 556)	291 (214 to 375)	488 (370 to 620)	394 (301 to 491)	542 (428 to 680)	463 (348 to 604)	351 (265 to 431)	244 (188 to 304)
**All risks combined**	399 (353 to 445)	282 (239 to 322)	1,148 (1,005 to 1,307)	789 (685 to 893)	1,029 (898 to 1,182)	699 (596 to 806)	935 (807 to 1,104)	764 (660 to 896)	875 (744 to 1,061)	757 (609 to 962)	868 (774 to 973)	626 (544 to 704)
**Percentages**												
**Metabolic risks**	77.1% (66.4 to 85.6)	76.1% (64.8 to 85.0)	78.4% (66.8 to 86.8)	79.1% (68.6 to 87.4)	76.8% (65.4 to 85.5)	78.3% (67.6 to 86.5)	76.9% (66.8 to 85.3)	79.1% (69.1 to 87.0)	73.9% (63.2 to 82.7)	75.0% (64.2 to 83.6)	77.2% (66.2 to 85.6)	78.3% (67.2 to 86.6)
**Behavioural risks**	41.8% (27.2 to 54.7)	29.4% (17.3 to 41.2)	47.5% (35.1 to 59.6)	28.0% (15.8 to 40.5)	44.7% (33.2 to 55.7)	27.5% (15.0 to 39.4)	37.5% (26.7 to 47.6)	25.6% (14.5 to 37.1)	31.5% (20.0 to 43.4)	25.0% (13.9 to 37.3)	43.6% (32.0 to 54.5)	27.7% (16.2 to 39.6)
**Environmental and occupational risks**	20.5% (15.3 to 25.5)	18.3% (14.0 to 22.6)	30.9% (23.7 to 38.1)	27.8% (22.2 to 34.1)	38.1% (28.7 to 47.4)	36.4% (28.5 to 44.8)	46.9% (36.5 to 56.3)	46.1% (37.1 to 54.4)	55.3% (45.0 to 63.9)	54.4% (45.5 to 62.5)	35.9% (27.7 to 44.0)	33.9% (27.0 to 40.6)
**All risks combined**	86.4% (78.8 to 91.7)	83.5% (74.7 to 89.7)	89.5% (83.1 to 93.8)	86.7% (78.9 to 92.2)	89.3% (83.5 to 93.5)	87.4% (80.8 to 92.4)	89.9% (84.7 to 93.6)	89.5% (83.9 to 93.3)	89.2% (83.9 to 93.0)	89.0% (83.3 to 92.8)	89.0% (83.1 to 93.3)	87.0% (79.9 to 92.0)

Appendix Table 17. Age-standardised rates (per 100,000 people) and percentages of DALYs (with 95% uncertainty intervals) due to intracerebral haemorrhage attributable to metabolic, behavioural and environmental/occupational risk clusters and all risk factors combined globally by SDI and sex in 2021.

Metrics	High SDI		High-middle SDI		Middle SDI		Low-middle SDI		Low SDI		Globally	
	Males	Females	Males	Females	Males	Females	Males	Females	Males	Females	Males	Females
**Rates**												
**Metabolic risks**	188 (151 to 220)	102 (82 to 122)	676 (495 to 850)	377 (292 to 466)	947 (716 to 1,168)	597 (468 to 737)	874 (701 to 1,050)	731 (580 to 887)	932 (710 to 1,158)	824 (626 to 1,044)	702 (548 to 839)	469 (386 to 576)
**Behavioural risks**	120 (69 to 165)	37 (9 to 62)	510 (348 to 689)	134 (46 to 226)	688 (497 to 907)	206 (106 to 321)	565 (402 to 723)	271 (147 to 403)	515 (309 to 710)	314 (141 to 484)	493 (370 to 634)	172 (111 to 251)
**Environmental and occupational risks**	70 (54 to 86)	36 (28 to 45)	350 (252 to 465)	185 (135 to 241)	570 (414 to 748)	342 (250 to 443)	651 (489 to 821)	523 (391 to 651)	838 (646 to 1,036)	714 (532 to 889)	438 (331 to 548)	288 (223 to 357)
**All risks combined**	241 (214 to 268)	125 (107 to 143)	885 (724 to 1,067)	452 (380 to 537)	1,254 (1,062 to 1,464)	728 (620 to 851)	1,174 (1,017 to 1,341)	927 (772 to 1,074)	1,296 (1,079 to 1,524)	1,104 (872 to 1,320)	933 (819 to 1,054)	584 (510 to 664)
**Percentages**												
**Metabolic risks**	60.6% (48.5 to 70.1)	57.1% (45.6 to 66.6)	63.5% (51.1 to 73.9)	63.9% (50.8 to 73.7)	63.1% (50.7 to 73.4)	64.6% (52.2 to 74.0)	63.5% (51.5 to 72.9)	65.5% (53.7 to 74.4)	60.9% (48.6 to 70.6)	62.2% (50.4 to 71.7)	62.5% (50.2 to 72.2)	63.2% (51.1 to 72.2)
**Behavioural risks**	38.5% (22.7 to 52.7)	20.5% (5.1 to 34.4)	47.9% (35.1 to 59.9)	22.7% (7.5 to 37.2)	45.8% (34.9 to 56.6)	22.4% (11.4 to 34.3)	41.0% (30.3 to 51.2)	24.3% (13.0 to 35.4)	33.6% (20.7 to 45.2)	23.7% (10.4 to 35.7)	43.9% (34.6 to 54.1)	23.2% (14.8 to 33.5)
**Environmental and occupational risks**	22.4% (17.3 to 27.3)	20.3% (16.0 to 24.5)	32.8% (25.3 to 40.4)	31.3% (25.2 to 38.2)	37.9% (28.8 to 47.0)	36.9% (28.8 to 45.3)	47.3% (37.5 to 56.5)	46.8% (37.8 to 55.0)	54.7% (45.0 to 63.0)	53.9% (45.1 to 61.8)	39.0% (30.4 to 47.2)	38.8% (31.2 to 46.2)
**All risks combined**	77.6% (68.6 to 84.2)	69.9% (60.2 to 77.8)	83.1% (76.1 to 88.6)	76.6% (67.2 to 83.8)	83.5% (77.5 to 88.5)	78.7% (70.8 to 85.1)	85.2% (79.1 to 89.1)	83.0% (75.9 to 87.7)	84.6% (78.5 to 88.5)	83.3% (76.6 to 87.5)	83.0% (77.0 to 87.8)	78.6% (71.3 to 84.3)

Appendix Table 18. Age-standardised rates (per 100,000 people) and percentages of DALYs (with 95% uncertainty intervals) due to subarachnoid haemorrhage attributable to metabolic, behavioural and environmental/occupational risk clusters and all risk factors combined globally by SDI and sex, 2021.

**Metrics**	**High SDI**		**High-middle SDI**		**Middle SDI**		**Low-middle SDI**		**Low SDI**		**Globally**	
	Males	Females	Males	Females	Males	Females	Males	Females	Males	Females	Males	Females
**Rates**												
**Metabolic risks**	46 (33 to 57)	46 (33 to 57)	63 (44 to 83)	49 (37 to 61)	83 (56 to 108)	71 (50 to 90)	87 (53 to 136)	76 (53 to 103)	71 (29 to 192)	52 (31 to 96)	71 (50 to 96)	61 (45 to 76)
**Behavioural risks**	26 (12 to 39)	17 (1 to 31)	43 (27 to 62)	18 (3 to 32)	58 (39 to 80)	27 (11 to 44)	63 (37 to 105)	34 (19 to 53)	45 (17 to 113)	25 (10 to 48)	49 (34 to 67)	24 (12 to 37)
**Environmental and occupational risks**	15 (12 to 19)	15 (11 to 19)	30 (22 to 41)	23 (17 to 29)	53 (34 to 72)	43 (31 to 58)	75 (44 to 119)	61 (44 to 83)	73 (31 to 182)	52 (36 to 92)	46 (31 to 65)	37 (28 to 47)
**All risks combined**	59 (50 to 68)	58 (46 to 68)	83 (69 to 105)	61 (51 to 73)	115 (87 to 141)	92 (74 to 113)	130 (84 to 199)	106 (84 to 139)	110 (48 to 281)	78 (55 to 144)	99 (78 to 127)	80 (67 to 95)
**Percentages**												
**Metabolic risks**	52.5% (38.0 to 63.6)	47.9% (34.6 to 59.3)	56.7% (42.2 to 68.0)	55.0% (40.6 to 65.9)	53.4% (39.0 to 64.9)	53.3% (38.9 to 64.6)	52.1% (38.1 to 63.1)	53.9% (40.3 to 64.2)	49.9% (35.7 to 61.7)	51.0% (37.6 to 62.0)	52.7% (38.6 to 64.0)	52.2% (38.6 to 63.0)
**Behavioural risks**	29.8% (13.2 to 44.1)	18.0% (0.8 to 32.7)	38.9% (24.5 to 51.2)	19.9% (3.7 to 34.7)	37.4% (27.4 to 48.4)	20.0% (8.1 to 32.3)	37.8% (28.0 to 47.0)	23.8% (14.2 to 33.7)	31.6% (19.6 to 42.1)	24.3% (10.8 to 36.0)	36.1% (27.7 to 45.8)	20.7% (10.6 to 31.4)
**Environmental and occupational risks**	17.2% (13.1 to 21.3)	15.8% (12.0 to 19.6)	26.9% (21.0 to 33.3)	25.2% (19.9 to 31.2)	34.2% (25.7 to 42.4)	32.5% (25.2 to 40.3)	44.5% (34.7 to 53.2)	42.7% (34.2 to 50.9)	51.5% (41.3 to 59.7)	51.3% (43.0 to 58.9)	33.9% (26.1 to 41.6)	31.5% (25.1 to 37.9)
**All risks combined**	67.9% (57.1 to 76.9)	60.9% (48.1 to 71.3)	75.3% (67.1 to 82.3)	68.5% (57.6 to 77.2)	74.5% (67.0 to 80.9)	69.2% (59.7 to 77.4)	77.5% (70.3 to 82.7)	74.9% (66.4 to 80.6)	77.4% (70.3 to 83.4)	76.7% (69.2 to 82.3)	73.9% (66.3 to 79.8)	68.9% (59.8 to 76.3)

Section 6. Appendix figures

Appendix Figure 1. 21 GBD regions

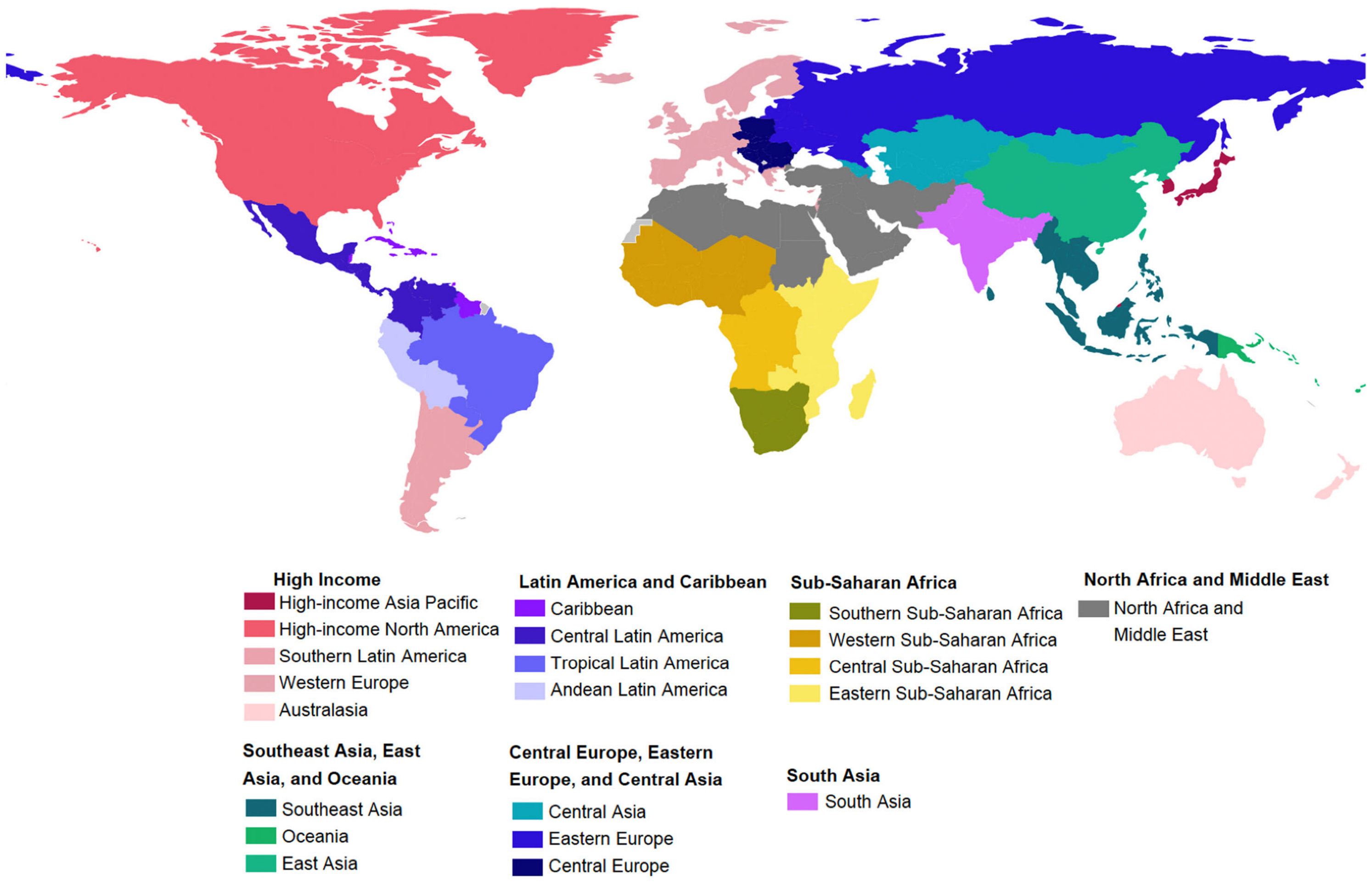

List of countries in 21 GBD regions

ASIA PACIFIC, HIGH INCOME: Brunei, Japan, Republic of Korea, Singapore; **ASIA CENTRAL:** Armenia, Azerbaijan, Georgia, Kazakhstan, Kyrgyzstan, Mongolia**,** Tajikistan, Turkmenistan, Uzbekistan; **ASIA, SOUTHEAST:** Cambodia, Indonesia**,** Lao People's Democratic Republic, Malaysia, Maldives**,** Mauritius, Mayotte**,** Myanmar**,** Philippines**,** Seychelles**,** Sri Lanka**,** Thailand, Timore Leste**,** Viet Nam; **ASIA EAST:** China, Democratic People's Republic of Korea, Hong Kong, Taiwan; **ASIA SOUTH:** Afghanistan, Bangladesh, Bhutan**,** India**,** Nepal**,** Pakistan; **AUSTRALASIA:** Australia, New Zealand; **CARIBBEAN:** Anguilla, Antigua and Barbuda, Aruba, Bahamas, Barbados, Belize, Bermuda, British Virgin Islands, Cayman Islands, Cuba, Dominica, Dominican Republic, French Guiana, Grenada, Guadeloupe, Guyana, Haiti, Jamaica, Martinique, Montserrat, Netherlands Antilles, Saint Kitts and Nevis, St. Lucia, St. Vincent, Suriname, Trinidad and Tobago, Turks and Caicos Islands; **EUROPE, CENTRAL:** Albania, Bosnia and Herzegovina, Bulgaria, Croatia, Czech Republic, Hungary, Poland, Romania, Serbia and Montenegro, Slovakia, Slovenia, The Former Yugoslav Republic of Macedonia; **EUROPE, EASTERN:** Belarus, Estonia, Latvia, Lithuania, Republic of Moldova, Russian Federation, Ukraine; **EUROPE, WESTERN:** Andorra, Austria, Belgium, Channel Islands, Cyprus, Denmark, Faeroe Islands, Finland, France, Germany, Gibraltar, Greece, Greenland, Holy See, Iceland, Ireland, Isle of Man, Israel, Italy, Liechtenstein, Luxembourg, Malta, Monaco, Netherlands, Norway, Portugal, Saint Pierre et Miquelon, San Marino, Spain, Sweden, Switzerland, United Kingdom; **LATIN AMERICA, ANDEAN:** Bolivia, Ecuador, Peru; **LATIN AMERICA, CENTRAL:** Colombia, Costa Rica, El Salvador, Guatemala, Honduras, Mexico, Nicaragua, Panama, Venezuela; **LATIN AMERICA, SOUTHERN:** Argentina, Chile, Falkland Islands (Malvinas), Uruguay; **LATIN AMERICA, TROPICAL:** Brazil, Paraguay; **NORTH AFRICA / MIDDLE EAST:** Algeria, Bahrain, Egypt, Iran (Islamic Republic of), Iraq, Jordan, Kuwait, Lebanon, Libyan Arab Jamahiriya, Morocco, Occupied Palestinian Territory, Oman, Qatar, Saudi Arabia, Syrian Arab Republic, Tunisia, Turkey, United Arab Emirates, Western Sahara, Yemen; **NORTH AMERICA, HIGH INCOME:** Canada, United States of America; **OCEANIA:** American Samoa, Cook Islands, Fiji, French Polynesia, Guam, Kiribati, Marshall Islands, Micronesia (Federated States of), Nauru, New Caledonia, Niue, Northern Mariana Islands, Palau, Papua New Guinea, Pitcairn, Samoa, Solomon Islands, Tokelau, Tonga, Tuvalu, Vanuatu, Wallis and Futuna Islands; **SUB-SAHARAN AFRICA, CENTRAL:** Angola, Central African Republic, Congo, Democratic Republic of the Congo, Equatorial Guinea, Gabon; **SUB-SAHARAN AFRICA, EAST:** Burundi, Comoros, Djibouti, Eritrea, Ethiopia, Kenya, Madagascar, Malawi, Mozambique, Rwanda, Somalia, Sudan, Uganda, United Republic of Tanzania, Zambia; **SUB-SAHARAN AFRICA, SOUTHERN:** Botswana, Lesotho, Namibia, South Africa, Swaziland, Zimbabwe; **SUB-SAHARAN AFRICA, WEST:** Benin, Burkina Faso, Cameroon, Cape Verde, Chad, Cote d'Ivoire, Gambia, Ghana, Guinea, Guinea-Bissau, Liberia, Mali, Mauritania, Niger, Nigeria, Saint Helena, Sao Tome and Principe, Senegal, Sierra Leone, Togo

Appendix Figure 2. Global number of deaths and DALYs by causes in 2021, both sexes, all ages

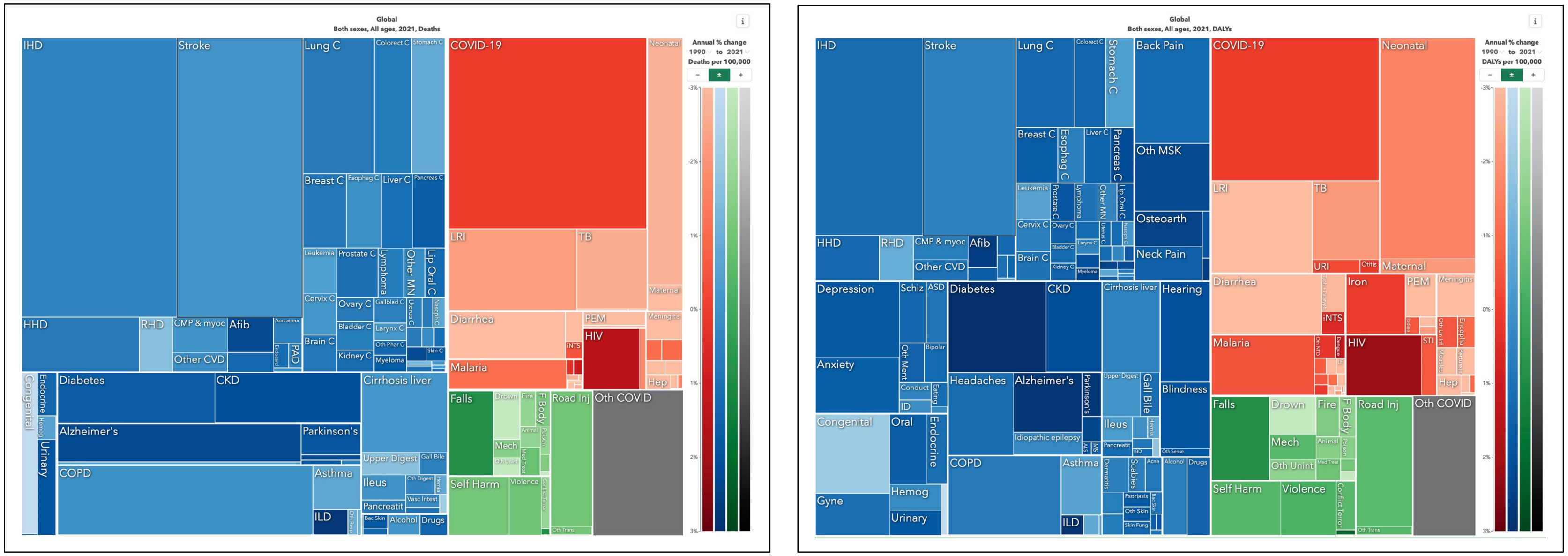

Appendix Figure 3. Global stroke incidence, prevalence, death and DALY rates per 100,000 per year from 1990 to 2021 (shadow colours stand for 95% UIs)

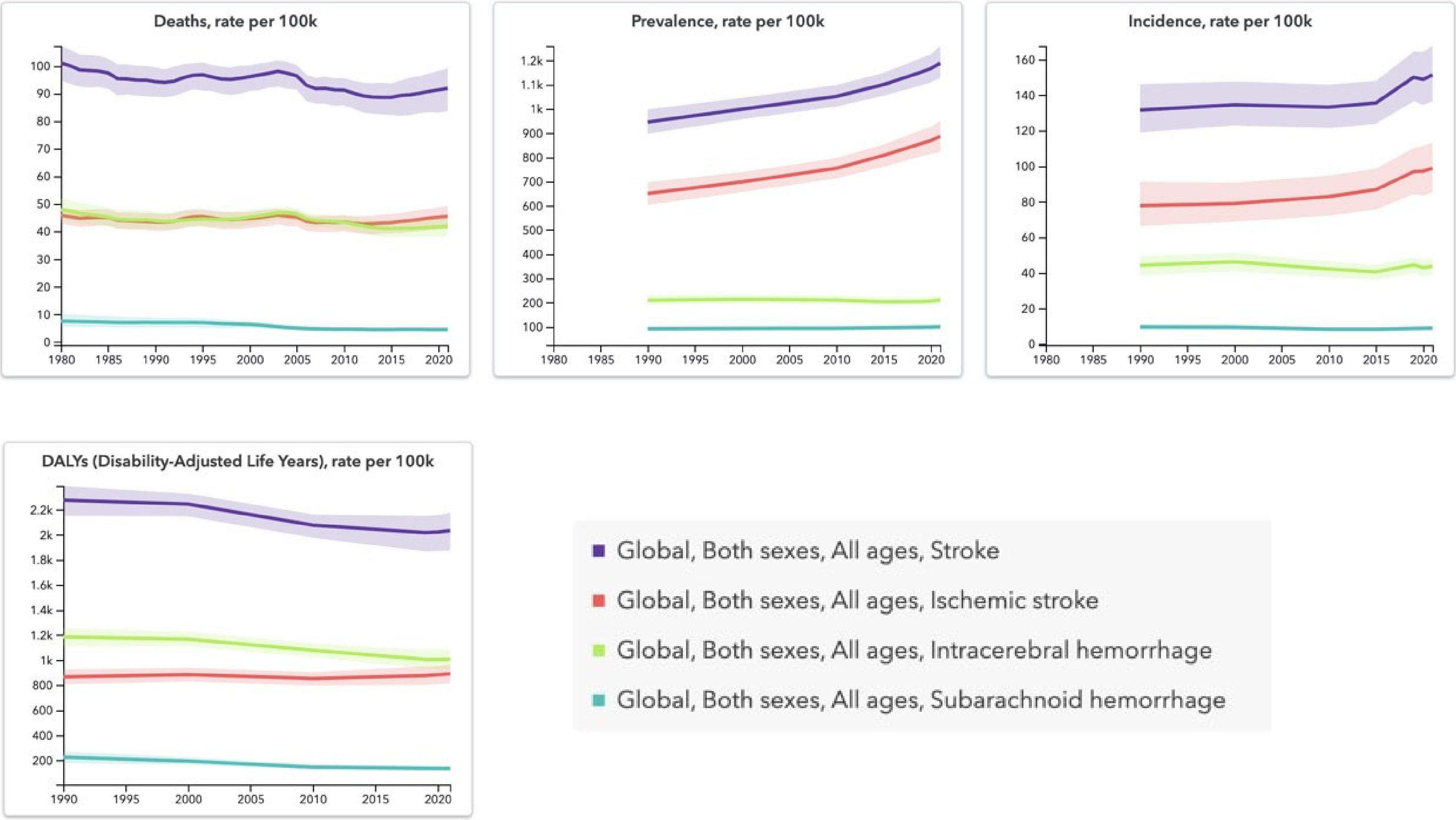

Appendix Figure 4. Global age-standardised stroke incidence, prevalence, death and DALY rates per 100,000 per year from 1990 to 2021 in 7 GBD super regions, both sexes (shadow colours stand for 95% UIs)

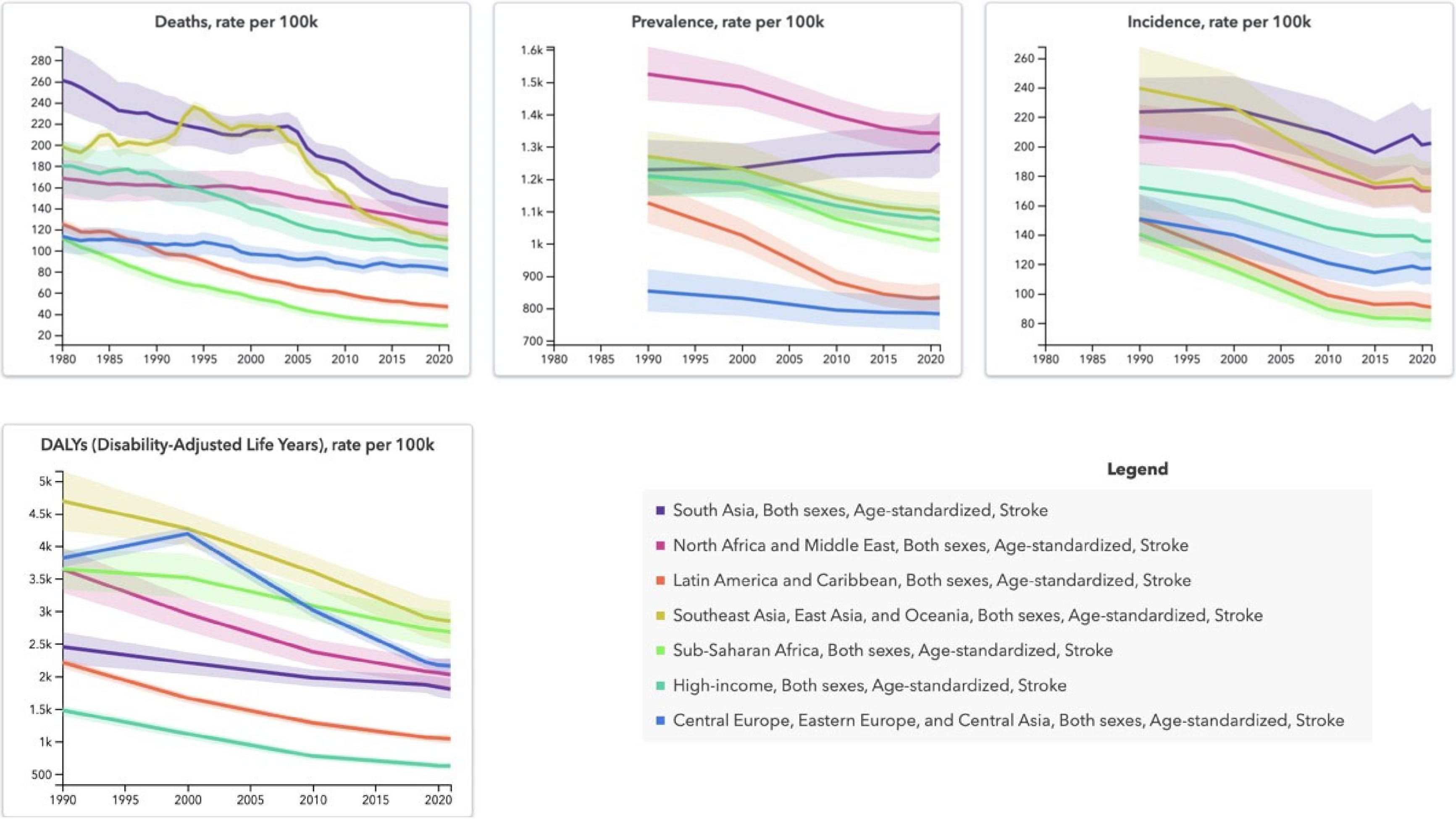

Appendix Figure 5. Global age-standardised stroke incidence, prevalence, death and DALY rates per 100,000 per year from 1990 to 2021 by pathological type of stroke, both sexes (shadow colours stand for 95% UIs)

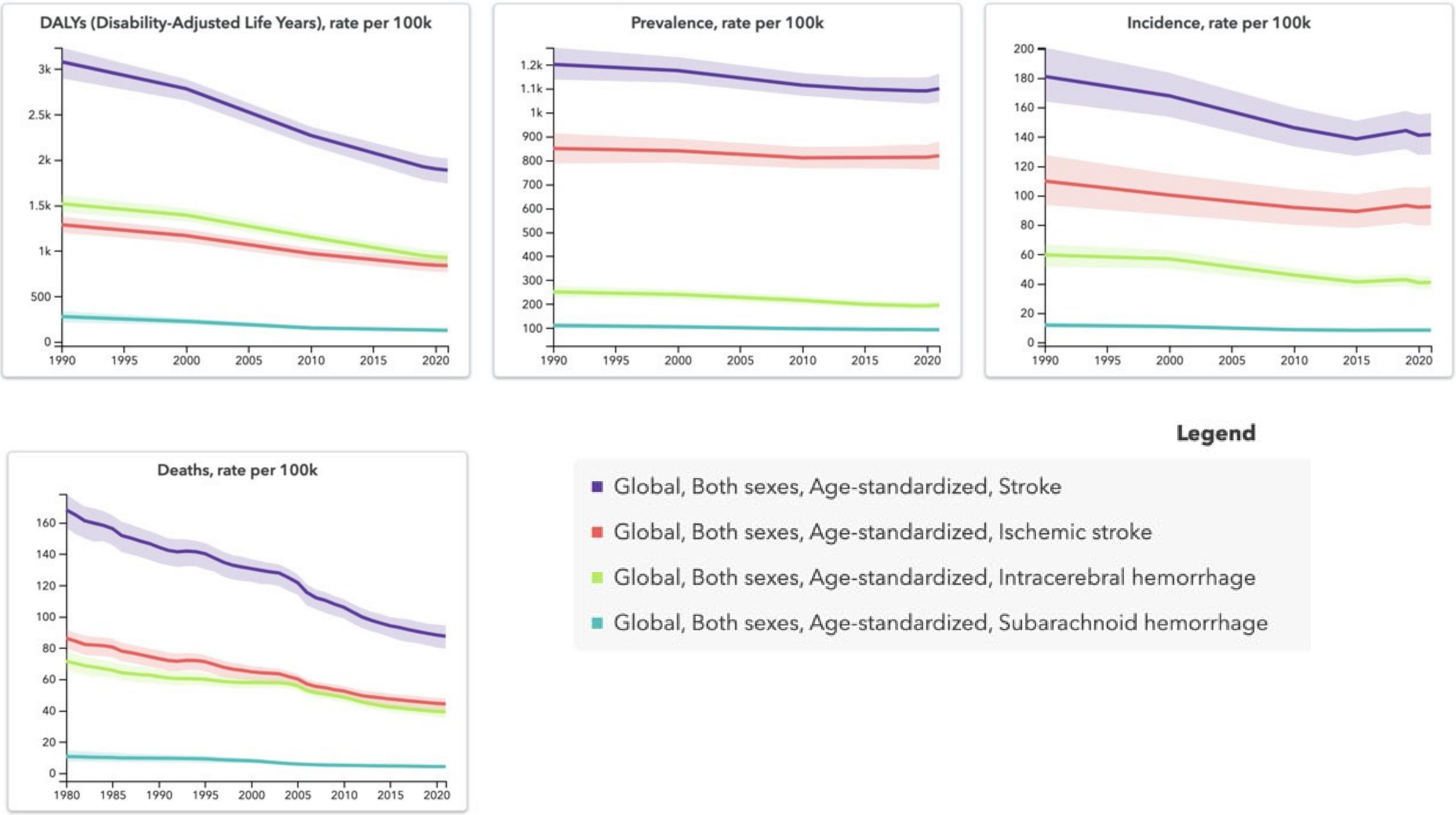

Appendix Figure 6. Global age-standardised stroke incidence, prevalence, death and DALY rates per 100,000 per year from 1990 to 2021 by SDI and pathological type of stroke, both sexes (shadow colours stand for 95% UIs)

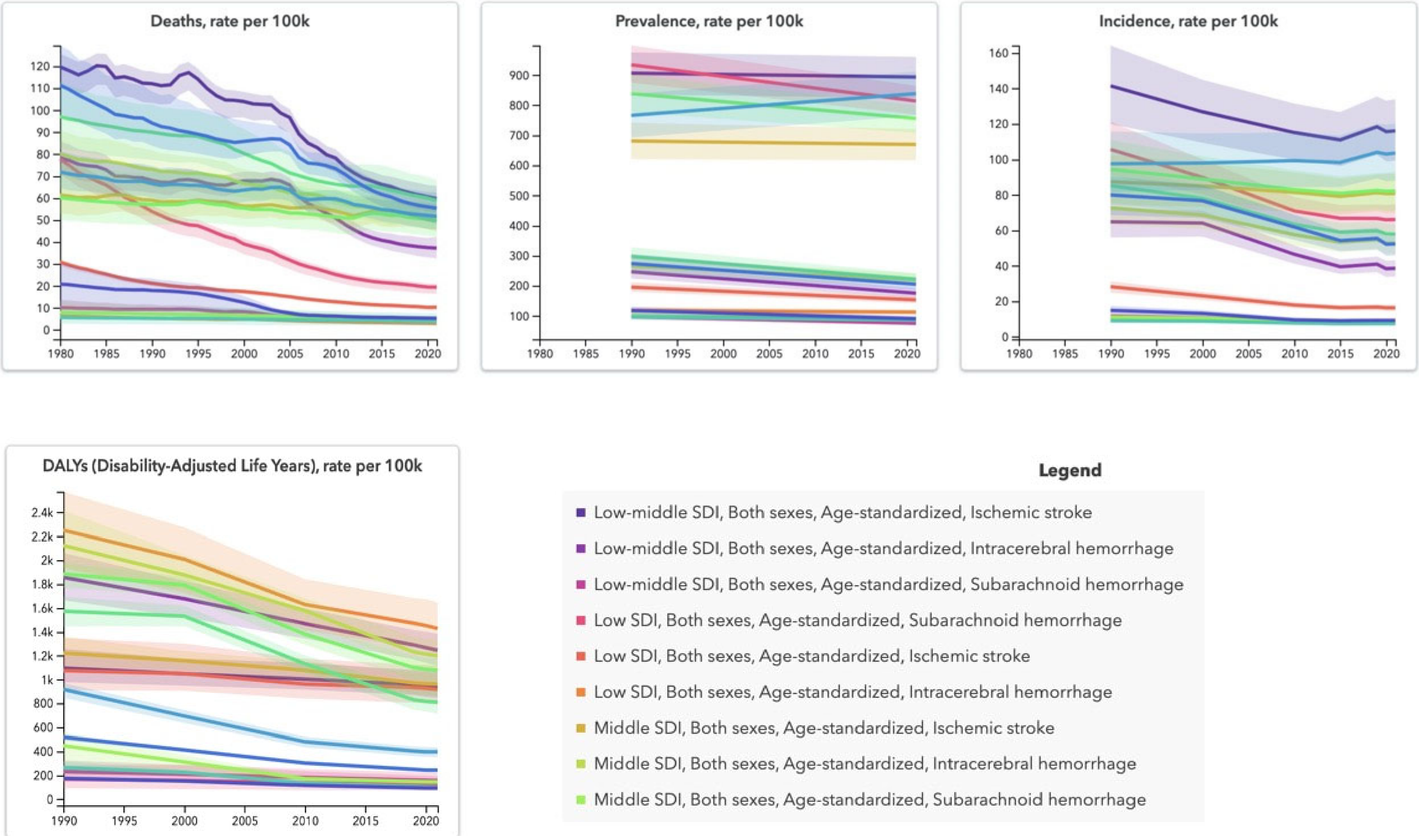

Appendix Figure 7. Global trends in age-standardised stroke incidence, prevalence, death, and DALY rates per 100,000 per year for 1990-2021 by SDI, for both sexes

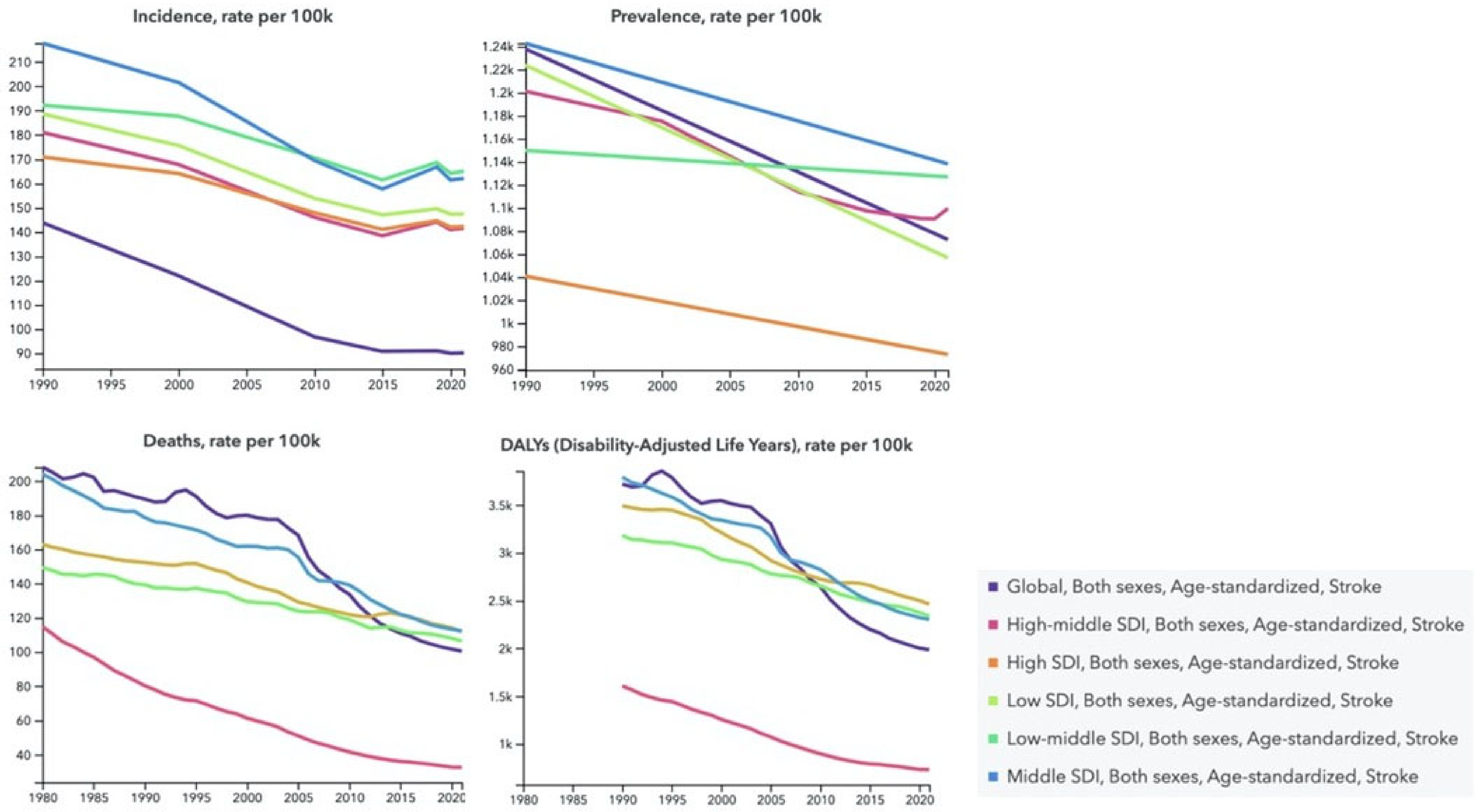

Appendix Figure 8. Annual stroke incidence, prevalence, death and DALY rates per 100,000 people younger than 70 years old (shadow areas are 95% uncertainty intervals)

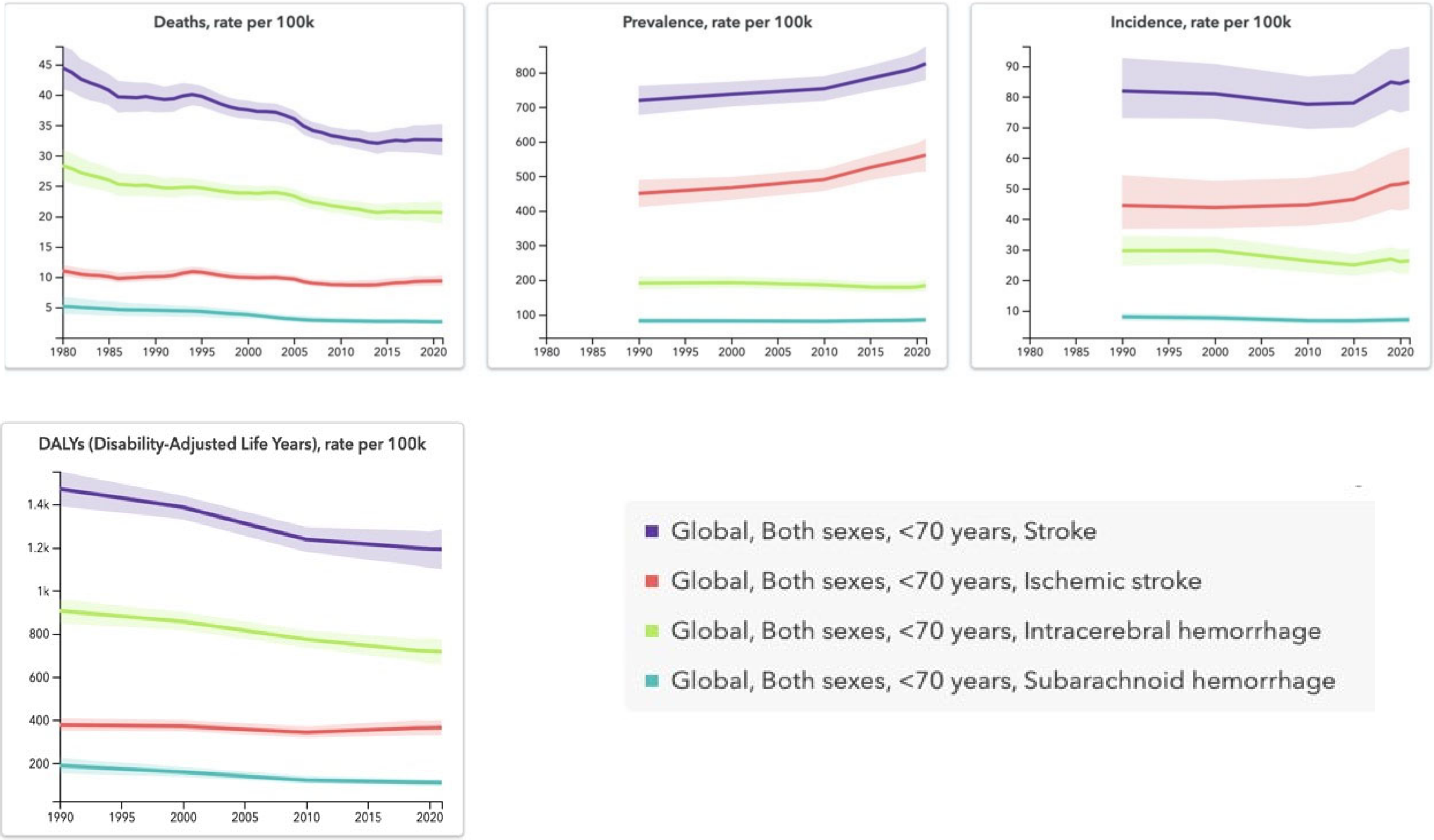

Appendix Figure 9. Annual stroke incidence, prevalence, death and DALY rates per 100,000 people of 70+ years old (shadow areas are 95% uncertainty intervals)

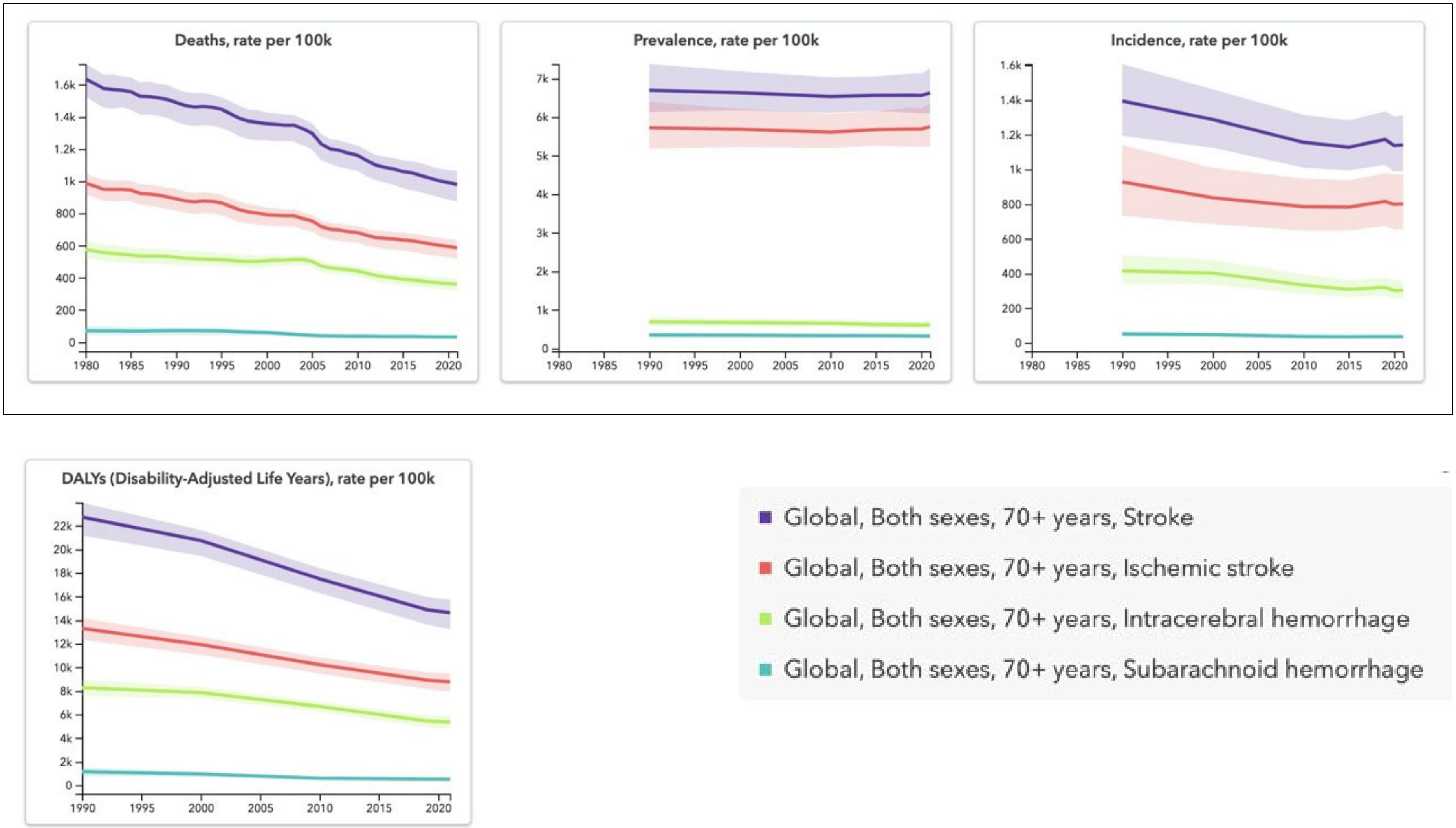

Appendix Figure 10. Global map showing age-standardised stroke DALYs attributable to all risk factors combined by pathological type of stroke, for both sexes, 2021

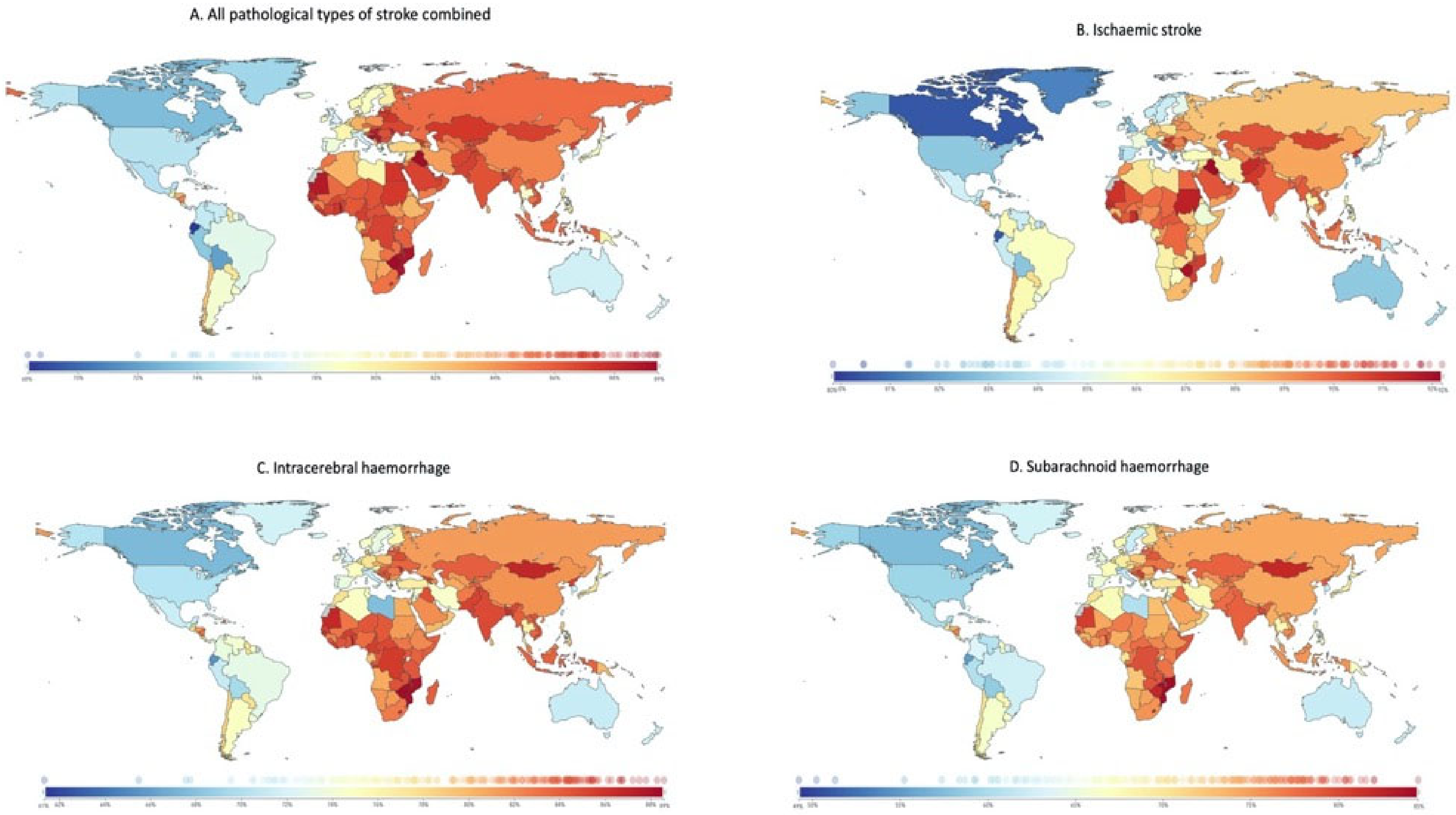

Appendix Figure 11. Trends in the percent of stroke-related DALYs attributable to risk factors in 1990 and 2021 at level 4 of the GBD risk factors hierarchy by pathological type of stroke, both sexes globally (rank based on age-standardised DALYs).

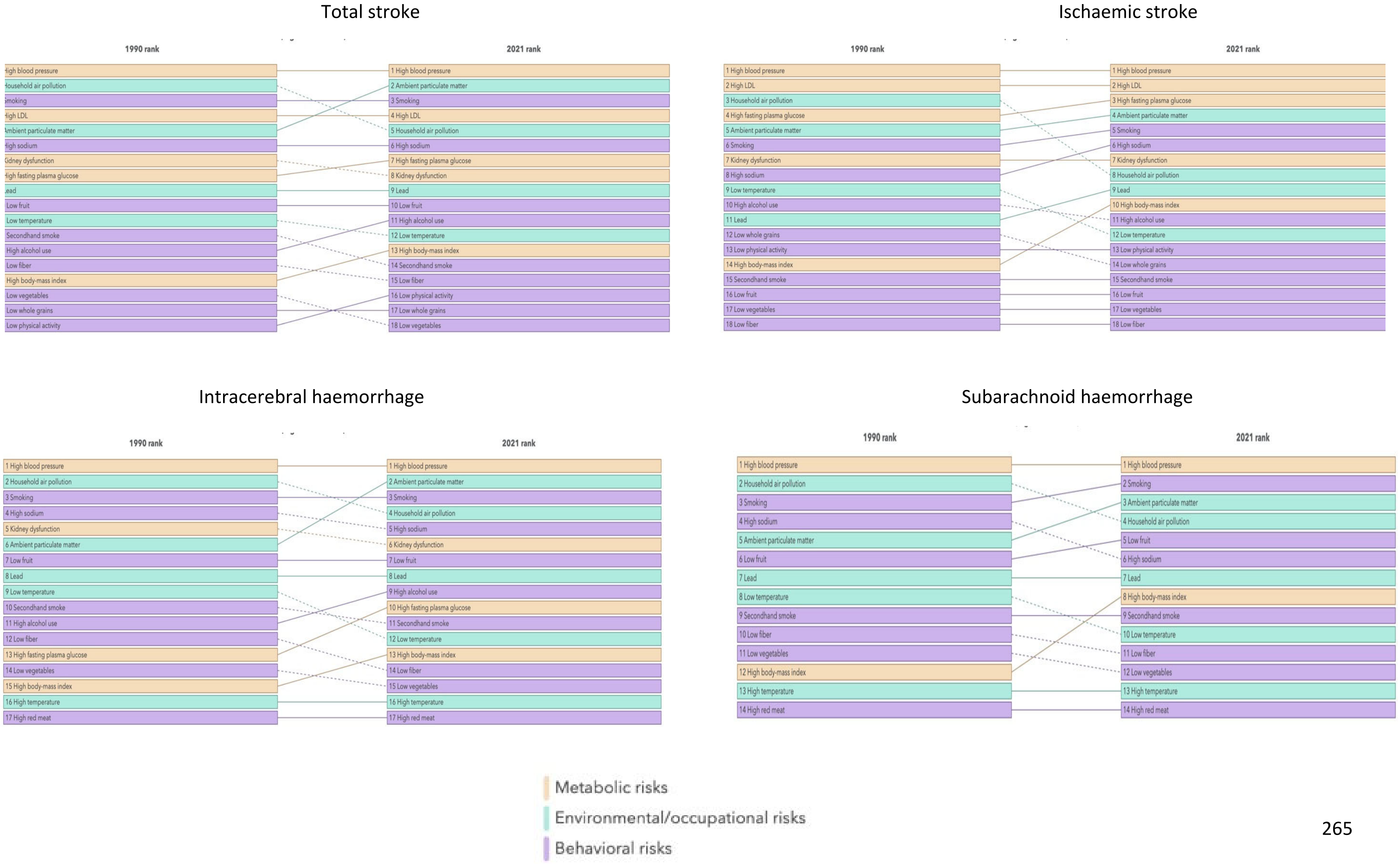

REFERENCES

1. Vos T, Lim SS, Abbafati C, Abbas KM, Abbasi M, Abbasifard M, et al. Global burden of 369 diseases and injuries in 204 countries and territories, 1990–2021: A systematic analysis for the global burden of disease study 2021. *The Lancet*. 2020;396:1204-1222
2. Murray CJL, Aravkin AY, Zheng P, Abbafati C, Abbas KM, Abbasi-Kangevari M, et al. Global burden of 87 risk factors in 204 countries and territories, 1990-2021: A systematic analysis for the global burden of disease study 2021. *The Lancet*. 2020;396:1223-1249
3. Johnson CO, Nguyen M, Roth GA, Nichols E, Alam T, Abate D, et al. Global, regional, and national burden of stroke, 1990-2016: A systematic analysis for the global burden of disease study 2016. *The Lancet Neurology*. 2021;18:439-458
4. Roth GA, Abate D, Abate KH, Abay SM, Abbafati C, Abbasi N, et al. Global, regional, and national age-sex-specific mortality for 282 causes of death in 195 countries and territories, 1980–2017: A systematic analysis for the global burden of disease study 2017. *The Lancet*. 2018;392:1736-1788
5. James SL, Abate D, Abate KH, Abay SM, Abbafati C, Abbasi N, et al. Global, regional, and national incidence, prevalence, and years lived with disability for 354 diseases and injuries for 195 countries and territories, 1990-2017: A systematic analysis for the global burden of disease study 2017. *The Lancet*. 2018;392:1789-1858
6. Kyu HH, Abate D, Abate KH, Abay SM, Abbafati C, Abbasi N, et al. Global, regional, and national disability-adjusted life-years (dalys) for 359 diseases and injuries and healthy life expectancy (hale) for 195 countries and territories, 1990-2017: A systematic analysis for the global burden of disease study 2017. *The Lancet*. 2018;392:1859-1922
7. Stanaway JD, Afshin A, Gakidou E, Lim SS, Abate D, Abate KH, et al. Global, regional, and national comparative risk assessment of 84 behavioural, environmental and occupational, and metabolic risks or clusters of risks for 195 countries and territories, 1990-2017: A systematic analysis for the global burden of disease study 2017. *The Lancet*. 2018;392:1923-1994
8. Lozano R, Freeman MK, James SL, Campbell B, Lopez AD, Flaxman AD, et al. Performance of interva for assigning causes of death to verbal autopsies: Multisite validation study using clinical diagnostic gold standards. *Popul Health Metr*. 2011;9:50
9. Aho K, Harmsen P, Hatano S, Marquardsen J, Smirnov VE, Strasser T. Cerebrovascular disease in the community: Results of a who collaborative study. *Bulletin of the World Health Organization.* 1980;58:113-130
10. Bamford J, Sandercock P, Dennis M, Burn J, Warlow C. A prospective study of acute cerebrovascular disease in the community: The oxfordshire community stroke project--1981-86. 2. Incidence, case fatality rates and overall outcome at one year of cerebral infarction, primary intracerebral and subarachnoid haemorrhage. *Journal of Neurology, Neurosurgery & Psychiatry*. 1990;53:16-22
11. Anderson CS, Jamrozik KD, Broadhurst RJ, Stewart-Wynne EG. Predicting survival for 1 year among different subtypes of stroke. Results from the perth community stroke study. *Stroke*. 1994;25:1935-1944
12. Nilsson OG, Lindgren A, Brandt L, Saveland H. Prediction of death in patients with primary intracerebral hemorrhage: A prospective study of a defined population. *J Neurosurg.* 2002;97:531-536
13. Foreman K, Lozano R, Lopez A, Murray C. Modeling causes of death: An integrated approach using codem. *Population health metrics*. 2012;10:1
14. Feigin VL, Roth GA, Naghavi M, Parmar P, Krishnamurthi R, Chugh S, et al. Global burden of stroke and risk factors in 188 countries, during 1990-2013: A systematic analysis for the global burden of disease study 2013. *The Lancet Neurology*. 2016;15:913-924

15. Murray CJ, Lopez AD. Global mortality, disability, and the contribution of risk factors: Global burden of disease study [see comments]. *Lancet*. 1997;349:1436-1442
16. Murray CJL, Lopez AD. On the comparable quantification of health risks: Lessons from the global burden of disease study. *Epidemiology*. 1999;10:594-605
17. Forouzanfar MH, Afshin A, Alexander LT, Anderson HR, Bhutta ZA, Biryukov S, et al. Global, regional, and national comparative risk assessment of 79 behavioural, environmental and occupational, and metabolic risks or clusters of risks, 1990-2015: A systematic analysis for the global burden of disease study 2015. *The Lancet*. 2016;388:1659-1724
18. Stevens GA, Alkema L, Black RE, Boerma JT, Collins GS, Ezzati M, et al. Guidelines for accurate and transparent health estimates reporting: The gather statement. *The Lancet*. 2016;388:e19-e23
19. Food, nutrition, physical activity and the prevention of cancer: A global perspective. Washington, d.C: World cancer research fund & american institute for cancer research, 2007. Https://discovery.Ucl.Ac.Uk/id/eprint/4841/1/4841.Pdf accesssed 6 november 2020.
20. Aravkin A, Davis D. Trimmed statistical estimation via variance reduction. *Mathematics of Operations Research*. 2021;45:292-322
21. Pavarin RM. Cocaine consumption and death risk: A follow-up study on 347 cocaine addicts in the metropolitan area of bologna. *Ann Ist Super Sanita*. 2008;44:91-98
22. Lim SS, Carnahan E, Nelson EC, Gillespie CW, Mokdad AH, Murray CJL, et al. Validation of a new predictive risk model: Measuring the impact of the major modifiable risks of death for patients and populations. *Population Health Metrics*. 2015;13:27
23. Danaei G, Singh GM, Paciorek CJ, Lin JK, Cowan MJ, Finucane MM, et al. The global cardiovascular risk transition: Associations of four metabolic risk factors with national income, urbanization, and western diet in 1980 and 2008. *Circulation*. 2013;127:1493-1502, 1502e1491-1498
24. Das gupta p. Standardization and decomposition of rates: A user's manual. Washington d.C.: U.S. Bureau of the census, 1993.
